# Enantioenriched Alkylboron Synthesis Via a Chiral Diborylalkane Platform

**DOI:** 10.1021/jacs.6c13223

**Published:** 2026-08-24

**Authors:** Hongyi Tao, Zhongxing Huang, Hairong Lyu

**Affiliations:** † Department of Chemistry, 26451 the Chinese University of Hong Kong, Shatin, N.T., Hong Kong SAR 999077, China; ‡ State Key Laboratory of Synthetic Chemistry, Shanghai Hong Kong Joint Laboratory in Chemical Synthesis, Department of Chemistry, the University of Hong Kong, Hong Kong SAR 999077, China; § Shanghai Hong Kong Joint Laboratory in Chemical Synthesis, the Chinese University of Hong Kong, Shatin, N.T., Hong Kong SAR 999077, China

## Abstract

Developing new reagents is one efficient strategy for expanding accessible chemical space. Recently, *gem*-diborylalkanes have emerged as a versatile class of boron-containing reagents with broad reactivity. However, most *gem*-diborylalkanes are achiral due to the pair of identical boryl groups, which restricts their utility in synthesis, especially for the production of enantioenriched products. To date, only a handful of chiral analogues have been reported, with constricted structural diversity and limited stereospecific transformations. Here, we report the design of a new class of chiral *gem*-diborylalkane (CDBA) reagents, featuring two boryl groups with chemically distinguishable reactivities, enabling highly selective and stepwise derivatizations. Their versatility is demonstrated through diverse downstream transformations, including the construction of boron stereogenic centers, the synthesis of boron-containing pharmaceuticals, and the preparation of key intermediates for small molecule drugs. These methods offer a powerful platform for the synthesis of enantioenriched alkylboron compounds and hold potential for enhancing synthetic capabilities in drug discovery and development.

## Graphical abstract

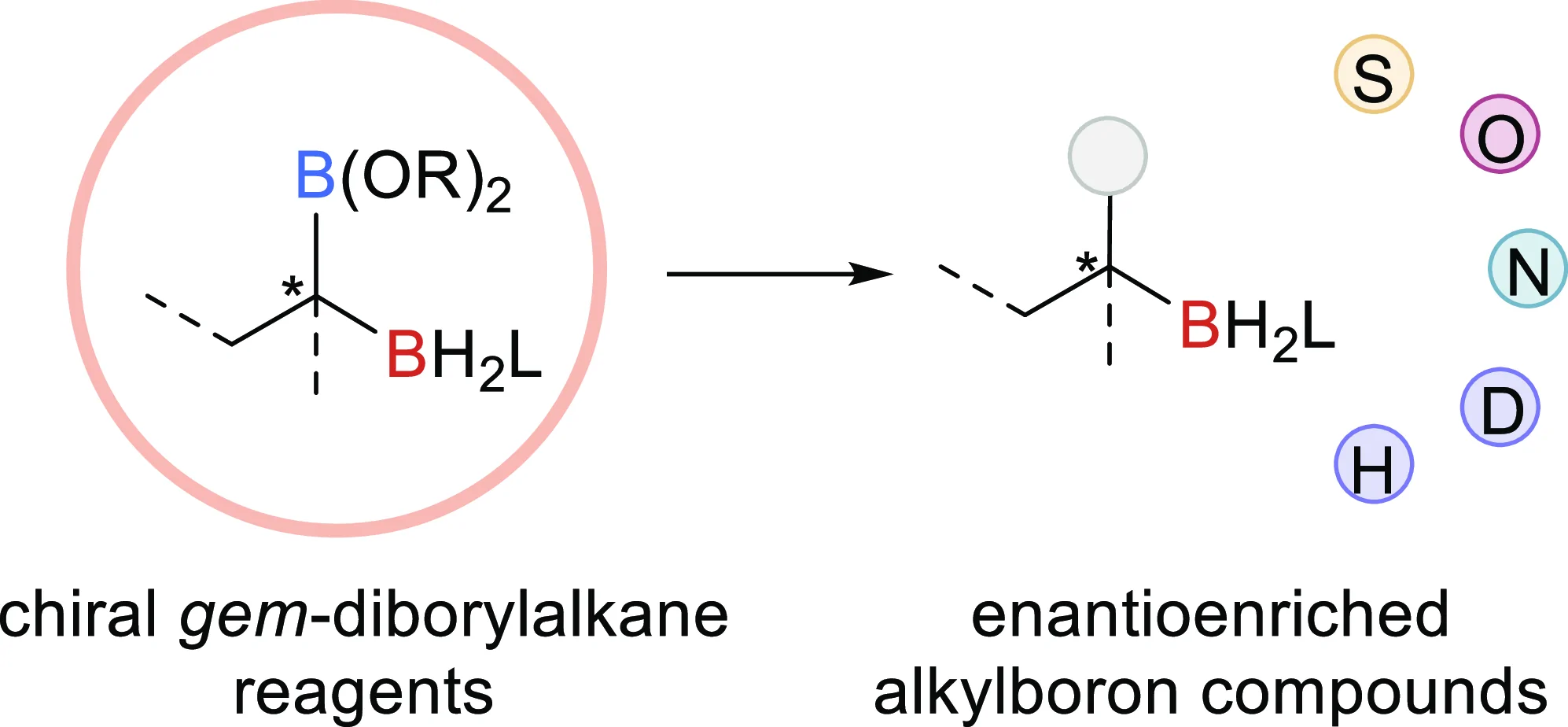

## Introduction

Organoboron compounds have found increasing applications in many areas.^1^ Among them, chiral organoboron compounds represent a particularly important class due to their unique properties and functionalities. Many applications of organoboron compounds, such as in small-molecule boron drugs,^1f,1g,1i,1j^ chiral catalysts and reagents,^2^ and chiroptical materials,^3^ rely on those enantioenriched derivatives. Despite their significance, current synthetic methods face limitations in efficiently producing these compounds with sufficient structural diversity. In addition to refining synthetic methodologies, the development of versatile boron reagents offers a complementary route to meeting this challenge.

Traditionally, boron reagents such as boranes, boron halides, and diboranes have been widely used in borylation methodologies.^1b^ ^,^ ^4^ More recently, *gem*-diborylalkanes have emerged as a new class of versatile boron-containing reagents due to their broad reactivity.^5^ These reagents participate in Lewis base-mediated coupling with electrophiles,^6^ boron-Wittig olefinations,^7^ transition-metal-catalyzed enantioselective couplings,^8^ and can even be converted into halo-*gem*-diborylalkanes,^9^ thereby expanding the organoboron chemical space (Figure 1a).^8a–8b,8c^ The use of *gem*-diborylalkanes as potential precursors for chiral boron compounds offers a promising solution for enriching this class of molecules. However, most *gem*-diborylalkanes are achiral due to their two identical boryl groups. In transformations involving achiral *gem*-diborylalkanes, such as the widely used bis­(pinacolato)­boryl alkanes, enantioenriched products can only be accessed through enantioselective coupling catalyzed by chiral transition metal catalysts.^8^

**Figure 1:**
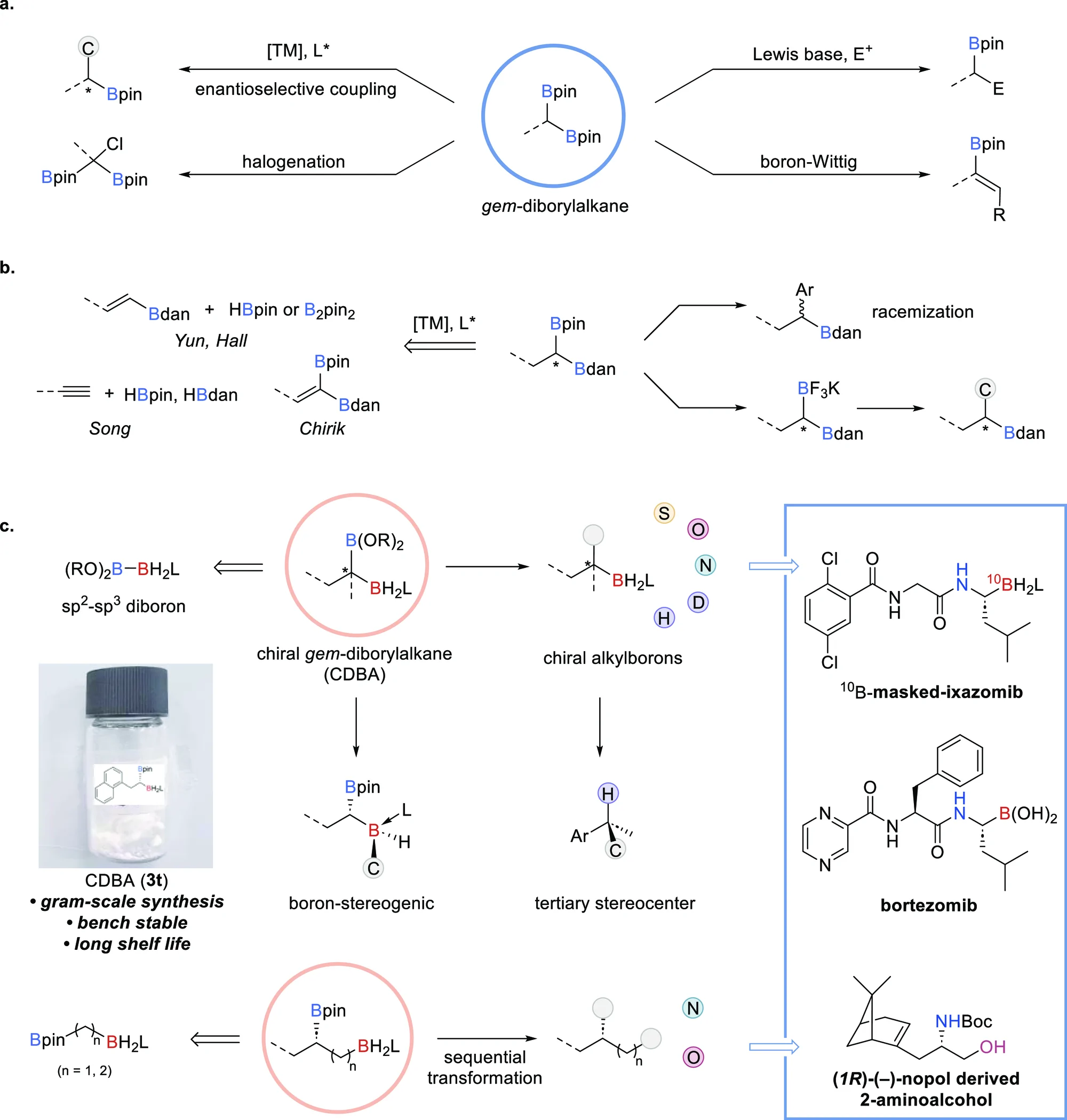
The *gem*-diborylalkane reagent. (**a**) Synthetic versatility of achiral *gem*-diborylalkane reagents. (**b**) State-of-art synthesis of chiral *gem*-diborylalkanes. (**c**) This work: preparation of new classes of chiral diborylalkane reagents and their synthetic applications.

While several pioneering studies have reported chiral *gem*-diborylalkanes containing −Bpin (pinacolato) and −Bdan (1,8-diaminonaphthalatoboryl) groups,^10^ their potential as reagents remains largely underexplored. Direct transformations often result in racemized products.^10a,10d^ For example, under basic conditions, nucleophilic deboration can generate an α-boryl-stabilized carbanion intermediate for subsequent reaction with electrophiles.^6b^ However, resonance between the α-boryl carbanion and a boron alkylidene species^11^ may result in a loss of stereochemical integrity. Currently, the only way to preserve stereochemical integrity involves preconversion of −Bpin to trifluoroborate salts followed by functionalization, but this is limited to C–C bond formation.^10a–10c^ This stands in stark contrast to the extensive C–B transformation toolbox available for racemic or monoborylated compounds (Figure 1b).^1a–1c,4a,12^

Therefore, we propose the synthesis of chiral diborylalkanes with robust structures and broader reactivities. By expanding their versatility and applications, these reagents could be utilized as potent chiral boron building blocks, unlocking new possibilities for the synthesis of enantioenriched alkylboron molecules. To address the reactivity limitations, we propose to keep the two boryl groups chemically easily distinguishable under typical transformation conditions so that reactions proceed at a single site without racemization of the stereogenic center. In this context, we envision that a chiral *gem*-diborylalkane (CDBA) reagent containing an sp^2^ boryl (−Bpin) and an sp^3^ boryl (−BH_2_L) might meet the criteria (Figure 1c).

A related structure was once reported by Zhu and co-workers via Cu-catalyzed asymmetric α-boryl carbene insertion into the B–H bond of borane–amines, though their structural diversity and further transformations remain to be further explored.^13^ On this basis, we envision that a stereospecific carbene insertion into the B–B bond^14^ of an unsymmetrical sp^2^-sp^3^ diboron reagent (LBH_2_–Bpin)^15^ could provide a more straightforward approach to access such compounds with more general structures for further applications as a class of boron reagents. This approach may eliminate the need for transition-metal catalysis or stepwise hydroboration processes. It is proposed that the reaction can go through a lithiation-borylation process, via nucleophilic addition of the lithium carbenoid to the sp^2^ boryl group in LBH_2_–Bpin, followed by a 1,2-migration of the sp^3^ boryl group. Although such lithiation-borylation strategy has been widely exploited for the stereocontrolled homologation of boronic esters,^16^ its use for synthesizing chiral diborylalkanes remains unexplored. The commonly used unsymmetrical sp^2^-sp^2^ diboron, Bpin–Bdan, has not been documented to undergo such transformation, due probably to the presence of acidic N–H protons that quench the sensitive lithium carbamate.

## Results and Discussion

We commenced our investigation with the lithiation of carbenoid precursor 3-phenylpropyl halides (**1a-1** and **1a-2**) in the presence of sp^2^-sp^3^ diboron **2a** (Figure 2a). However, instead of the desired *gem*-diborylalkane **3**, a 51% yield of alkyl borane **3′** was observed in both cases. It was deduced that instead of deprotonating the alkyl halides, ^*n*^BuLi attacked **2a**, triggering the B–B bond heterolytic cleavage to form a nucleophilic *N*-heterocyclic (NHC) boryl anion, which subsequently reacted with 3-phenylpropyl halides to afford **3′**.^17^ Screening of carbenoids revealed that the Hoppe-type carbenoid (LG = −OCb, −OCb = *N*,*N*-diisopropylcarbamate),^18^ first applied by Aggarwal and co-workers for homologation of boronic esters via 1,2-metalate rearrangement,^16b,16e^ afforded the racemic *gem*-diborylalkane product **3** with an optimal yield of 86%. Compared to halide leaving groups, the −OCb group facilitates the formation of a more table lithium carbenoid,^19^ enabling its efficient coordination to the boron center for subsequent 1,2-metalate rearrangement while effectively suppressing the competing NHC-boryl anion pathway.

**Figure 2:**
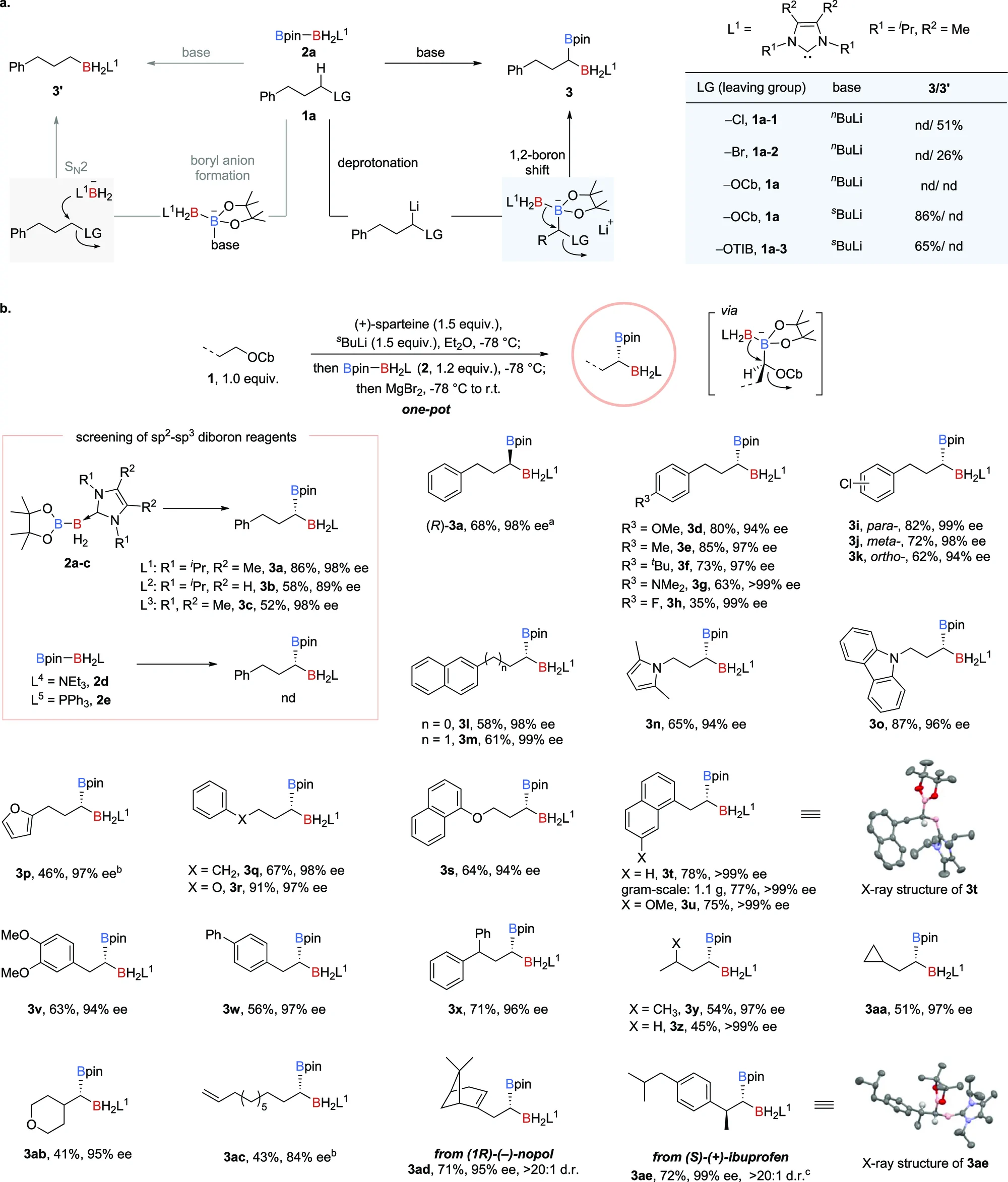
Synthesis of chiral *gem*-diborylalkanes. (**a**) Screening carbenoid precursors for carbene insertion into the B–B bond of sp^2^–sp^3^ diboron reagent **2a**; nd = not detected. (**b**) Synthesis of chiral *gem*-diborylalkanes. Reaction conditions: **1** (0.1 mmol, 1.0 equiv.), (+)-sparteine (0.15 mmol, 1.5 equiv.), ^*s*^BuLi (0.15 mmol, 1.5 equiv.), Et_2_O (0.5 mL), −78 °C, 30 min; then **2** (0.1 M in Et_2_O, 0.12 mmol, 1.2 equiv.), −78 °C, 10 min; then MgBr_2_ (0.5 M in Et_2_O, 0.2 mmol, 2.0 equiv.), −78 °C, 20 min, r.t., 30 min. All isolated yields were provided after flash column chromatography. Unless otherwise stated, all enantiomeric excesses (ee) were determined by HPLC analysis. ^a^(−)-Sparteine was used instead of (+)-sparteine. ^b^2.5 Equiv. of ^*s*^BuLi and (+)-sparteine were used. ^c^ *N,N,N′,N′*-Tetramethylethylenediamine (TMEDA) was used instead of (+)-sparteine. See Supporting Information for experimental details.

Furthermore, the use of (+)-sparteine auxiliary, which enabled the synthesis of enantioenriched lithium carbamates, facilitated the stereospecific synthesis of chiral *gem*-diborylalkane **3a**. A systematic investigation of reaction conditions, including solvents, temperature, and Lewis acids, resulted in **3a** with 86% yield with excellent enantioselectivity (98% ee, Table S2). Using (−)-sparteine instead of (+)-sparteine afforded (*R*)-**3a** with a yield of 68% and equally high but opposite enantioenrichment (Figure 2b). Subsequently, sp^2^–sp^3^ diboron reagents containing different Lewis base ligands (L) were evaluated (Figure 2b). Diboron **2b** afforded CDBA **3b** with a yield of 58% and 89% ee. The reduced yield and enantioselectivity might be attributed to competitive deprotonation of the backbone C­(sp^2^)–H on the NHC ligand (*R* ^2^ = H) under the strong basic conditions. Replacing the isopropyl groups in the NHC ligand of **2a** with methyl groups (**2c**) produced the desired product with excellent ee (98%), albeit with a moderate yield of 52%. This suggests that the steric hindrance of the NHC ligand is not the governing factor for stereocontrol. In contrast, attempts using triethylamine- and triphenylphosphine-coordinated diboron reagents **2d** and **2e** failed to generate the corresponding CDBAs.

The scope of carbenoid precursors was also examined (Figure 2b). Substrates bearing electron-donating groups on the phenyl ring, such as −OMe (**3d**, **3u**, **3v**), −Me (**3e**), −^*t*^Bu (**3f**), and −NMe_2_ (**3g**), demonstrated good reactivity under the standard conditions, affording the corresponding products in >60% yields with high enantioselectivity. Additionally, the reaction showed compatibility with halogens (–F and –Cl), yielding chiral *gem*-diborylalkanes **3h**–**3k** with moderate to high yields and up to 99% ee. Alkyl carbamates containing other functional groups, including naphthyl (**3l**, **3m**, **3t**, **3u**), *N*-heterocyclic (**3n**, **3o**), furan (**3p**), phenoxy (**3r**), naphthoxy (**3s**), cyclopropyl (**3aa**), tetrahydropyran (**3ab**), and terminal alkenyl (**3ac**) groups reacted well with diboron **2a** under the standard conditions. A long carbon chain can introduce steric effects that slightly compromise both the yield and enantioselectivity, as observed for **3ac**. Furthermore, CDBAs derived from natural products like (*1R*)-(−)-nopol (**3ad**) and (*S*)-(+)-ibuprofen (**3ae**), were successfully prepared in satisfactory yields and enantioselectivity. Finally, the synthesis of CDBA **3t** is readily scalable to a gram-scale, maintaining a high yield (77%) and excellent enantioselectivity (>99% ee) without requiring column chromatography. The (+)-sparteine auxiliary used in the reaction was efficiently recovered from the aqueous phase after extraction with a 65% yield (See Supporting Information Section 5.3 for experimental details). In addition, these CDBAs are bench-stable with a long shelf life. They also display robust stability during aqueous workup and basified silica gel chromatography.

Subsequently, the synthetic versatility of the newly prepared CDBA reagents was investigated (Figure 3a). The electron-deficient −Bpin (sp^2^ boryl) group is expected to exhibit higher reactivity than the −BH_2_L (sp^3^ boryl) group toward nucleophiles, undergoing corresponding transformations preferentially. Treatment with excess NaBO_3_ (5.0 equiv) selectively oxidized the −Bpin group, affording the chiral α-hydroxy borane **4** in 45% yield with 99% ee. The α-hydroxy boron compounds serve as valuable building blocks in organic synthesis and medicinal chemistry.^20^ Previous methods for preparing similar chiral α-hydroxy boronates protected by a BMIDA (MIDA = *N*-methyliminodiacetyl) group required multiple steps.^20d^ ^,^ ^21^ On the other hand, the corresponding sp^2^ boryl analogues, such as α-hydroxyboronate esters prepared via copper-catalyzed asymmetric borylation of aldehydes, are inherently unstable and require silyl protection for stabilization.^20b^

**Figure 3:**
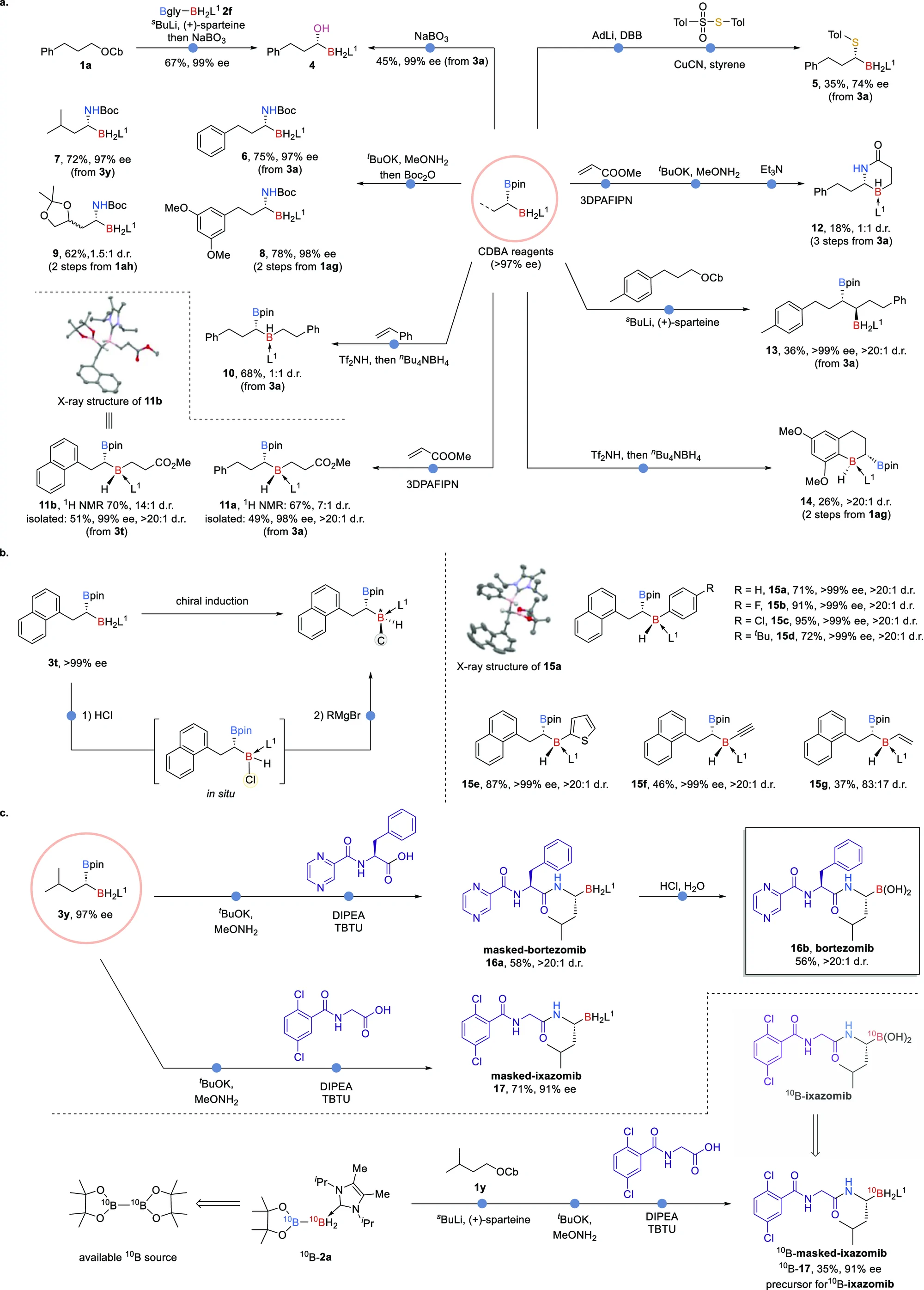
Transformations and synthetic applications of the CDBA reagent. (**a**) Transformations of chiral *gem*-diborylalkane reagents. (**b**) Synthesis of chiral organoboron compounds featuring both carbon and boron stereogenic centers using CDBA reagents. (**c**) Synthesis of boron-containing drug molecules. AdLi = 1-adamantyllithium; DBB = 4,4*′*-di-*tert*-butylbiphenyl; 3DPAFIPN = 2,4,6-tris­(diphenylamino)-5-fluoroisophthalonitrile; DIPEA = *N*,*N*-diisopropylethylamine; TBTU = 2-(1H-benzotriazole-1-yl)-1,1,3,3-tetramethylaminium tetrafluoroborate; ^1^H NMR yields were provided using 1,1,2,2-tetrachloroethane as internal standard.

Additionally, utilizing the method developed by Morken and co-workers,^22^ the −Bpin group in CDBA reagent **3a** selectively underwent thioetherification to give α-(*p*-tolylthio) borane **5** in 35% yield with 74% ee. Selective amination of CDBA also proceeded smoothly,^23^ delivering chiral α-amino boranes **6**–**8** in good yields with high enantioselectivity. Furthermore, stereoselective homologation of **3a** was successfully achieved, producing the chiral 1,2-diborylalkane **13** in 36% yield with >99% ee and >20:1 d.r..

In addition to transformations based on the −Bpin group in the CDBA reagent, functionalization of the editable B–H moiety on the −BH_2_L group offers further opportunities to expand the diversity of chiral boron compounds. Hydroboration of styrene using CDBA **3a** was readily achieved, providing difunctionalized boranes **10** in 68% yield with 1:1 d.r.. The reaction of **3a** with methyl acrylate delivered difunctionalized boranes **11a**, from which only the major diastereomer was isolated in 49% yield with 98% ee. Similarly, the reaction of methyl acrylate reacted with CDBA **3t** yielded **11b** in 51% isolated yield with 99% ee and >20:1 d.r.. The absolute configuration was further confirmed via X-ray analysis. Subsequent transformations of compound **11a**, involving selective amination followed by cyclization, produced the boron-containing heterocyclic lactam **12** in 18% yield and 1:1 d.r. over three steps. Moreover, upon treatment with Tf_2_NH, dehydridation of **3a** first generated a borenium species,^24^ which underwent intramolecular cyclization with the electron-rich aromatic ring,^25^ yielding boracycle **14** in 26% yield with >20:1 d.r..

During our investigation of the acylation of CDBA **3t** with benzoyl chloride, only a B–H chlorination product was observed. This intermediate was treated *in situ* with phenyl Grignard reagent, resulting in the displacement of chlorine by a phenyl group and affording **15a** in 20% yield with >99% ee and >20:1 d.r.. The observed diastereoselectivity suggests that the adjacent chiral carbon effectively induces chirality at the sp^3^-boron center,^26^ similar to the reaction pathway reported by Chuzel and co-workers.^2i^ By modifying the reaction conditions and using HCl for chlorination, the yield of **15a** was improved to 71% (Figure 3b). The absolute configuration of compound **15a** was confirmed via X-ray analysis. Using this method, we successfully synthesized enantioenriched organoboron compounds featuring both carbon and boron stereogenic centers,^27^ starting from the CDBA reagent. Aryl Grignard reagents with substituents such as −F (**15b**), −Cl (**15c**), and −^*t*^Bu (**15d**), as well as thienyl Grignard reagents (**15e**), reacted smoothly with high stereoselectivity. Ethynyl and vinyl Grignard reagents were also compatible, albeit with slightly lower yields or d.r. (**15f**, **15g**).

The newly prepared CDBA reagents have demonstrated remarkable utility in the synthesis of FDA-approved boron drugs and their derivatives (Figure 3c). Starting from CDBA **3y**, a one-step amination followed by *in situ* condensation with *N*-(pyrazinylcarbonyl)-*L*-phenylalanine and 2-(2,5-dichlorobenzamido)-acetic acid afforded the NHC-masked boron drugs **16a** (masked-bortezomib) and **17** (masked-ixazomib) in 58% yield, >20:1 d.r. and 71% yield, 91% ee, respectively. Subsequent treatment of **16a** with HCl enabled the successful synthesis of bortezomib (**16b**) in 56% yield and >20:1 d.r..

More importantly, a ^10^B isotope-labeled chiral diborylalkane reagent (^10^B-CDBA) was readily prepared from sp^2^-sp^3^ diboron (^10^B-**2a**, prepared from ^10^B_2_pin_2_ ^28^). This reagent was successfully utilized for the synthesis of ^10^B-enriched masked ixazomib (^10^B-**17**) with an overall yield of 35% over three steps and 91% ee, highlighting the potential of ^10^B-CDBA for the synthesis of various ^10^B-enriched molecules, which could be valuable for boron neutron-capture therapy (BNCT).^1i,29^

In addition to chiral *gem*-diborylalkanes **3**, the preparation of CDBA reagents with a fully substituted chiral carbon center was also explored, though it proved more challenging (Figure 4a). By replacing the −Bpin group in sp^2^-sp^3^ diboron **2a** with a less sterically hindered −—Bgly group (**2f**), the reaction with chiral secondary carbamates (**1ai**–**1al**) afforded corresponding CDBAs. *In situ* derivatization of these CDBAs afforded the corresponding enantioenriched organoborons (**18a**–**f**) in moderate to good yields and high enantioselectivity. Subsequent C–B­(sp^3^) transformation was also demonstrated. Deprotection of compound **18f** by removing the NHC ligand, followed by Suzuki coupling with 3-iodoanisole, afforded **18h** in 78% yield with 72% ee and 85% es. It is noteworthy that the use of the less bulky diboron **2f** also promoted the oxidation efficiency of the CDBA, leading to an enhanced yield of compound **4** (Figure 3a, top left).

**Figure 4:**
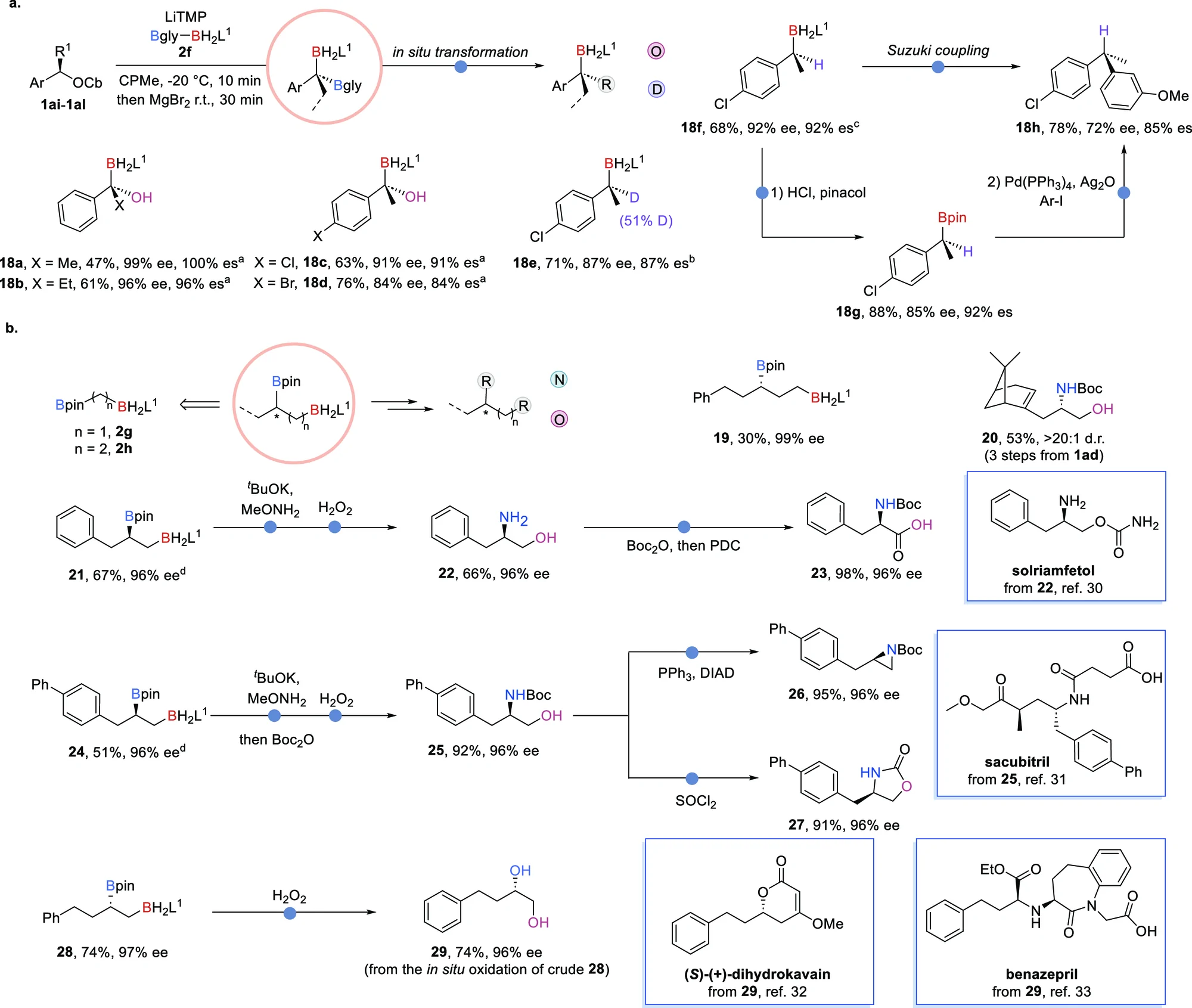
Synthetic elaborations. (**a**) Synthesis of chiral *gem*-diborylalkanes with a fully substituted chiral carbon center and their applications. (**b**) Synthesis and transformations of 1,2- and 1,3-diborylalkanes. ^a^For the *in situ* oxidation, NaBO_3_ was used. ^b^For the *in situ* deuterodeborylation, ^*t*^BuOK and MeOD were used. ^c^For the *in situ* protodeboration, ^*t*^BuOK and H_2_O was used. ^d^(−)-Sparteine was used instead of (+)-sparteine. See Supporting Information for experimental details.

In addition to unsymmetrical diboron reagents, diborylmethane (**2g**) and 1,2-diborylethane (**2h**) also reacted with alkyl carbamates under standard conditions, yielding chiral 1,2-diborylalkanes **21**, **24**, **28** and 1,3-diborylalkane **19** in moderate to good yields with high ee (Figure 4b). Further derivatization of these chiral 1,n-diborylalkanes was also explored. Using a sequential “amination-then-oxidation” strategy, the natural product (*1R*)-(−)-nopol was conveniently converted into the enantioenriched 2-aminoalcohol **20** in 53% three-step yield and >20:1 d.r.. The 2-aminoalcohols **22** and **25**, derived from chiral 1,2-diborylalkanes **21** and **24**, are valuable synthetic intermediates for the pharmaceuticals solriamfetol^30^ and sacubitril,^31^ respectively. Furthermore, these aminoalcohols could be transformed into Boc-D-Phe-OH **23** (98%, 96% ee), chiral aziridine **26** (95%, 96% ee), and oxazolidinone **27** (91%, 96% ee) in excellent yields without loss of enantiomeric excess. Additionally, direct oxidation of **28** with H_2_O_2_ provided the chiral diol **29** in 74% yield and 96% ee. This diol serves as a key intermediate in the synthesis of natural product (*S*)-(+)-dihydrokavain^32^ and the antihypertensive medication benazepril.^33^

## Conclusion

In conclusion, we have successfully synthesized a series of chiral *gem*-diborylalkane (CDBA) reagents and chiral 1,n-diborylalkanes featuring chemically distinguishable boryl groups. These reagents facilitate the synthesis of diverse chiral alkylboron compounds that are difficult to access via traditional methods or boron reagents. Furthermore, sequential derivatization of these chiral diborylalkane reagents underscores their potential for developing boron-based pharmaceuticals and other bioactive small molecules. We anticipate that these reagents will expand the available toolbox for boron chemistry and broaden the accessible chemical space for chiral organic molecules.

## Supplementary Material



## Notes

Experimental procedures, characterization data for new compounds, and NMR spectra (PDF)
