# Supplementary material for: Enantioenriched Alkylboron Synthesis Via a Chiral Diborylalkane Platform
Source: J Am Chem Soc. 2026 Aug 24;148(35):38422–31. doi: 10.1021/jacs.6c13223 (PMC13564432; doi:10.1021/jacs.6c13223)
Supplement: Supplementary file 1 [file ja6c13223_si_001.pdf]

Supporting Information

Enantioenriched alkylboron synthesis via a chiral diborylalkane platform

Hongyi Tao,[1] Zhongxing Huang,[2] Hairong Lyu[1,3]*

[1]Department of Chemistry, The Chinese University of Hong Kong, Shatin, N.T., Hong Kong SAR, China

[2]State Key Laboratory of Synthetic Chemistry, Shanghai Hong Kong Joint Laboratory in Chemical Synthesis, Department of Chemistry, The University of Hong Kong, Hong Kong SAR, China

[3]Shanghai Hong Kong Joint Laboratory in Chemical Synthesis, The Chinese University of Hong Kong, Shatin, N.T., Hong Kong SAR, China

**Corresponding author, e-mail:* hrlyu@cuhk.edu.hk

1. General information. 2
2. Synthesis of diboron reagents 2a-2g and ^{10}B-2a. 3
3. Synthesis of carbamates 1. 10
  3.1. Preparation of compounds 1a, 1l, 1q, 1r, 1t, 1w, 1x-1z, 1aa-1ac, 1af and 1ah. 10
  3.2. Preparation of compounds 1d-1k, 1m, 1p, 1u-1v, 1ad, 1ae and 1ag. 14
  3.3. Preparation of compound 1n. 19
  3.4. Preparation of compound 1o. 20
  3.5. Preparation of compound 1s. 21
  3.6. Preparation of compounds 1ai-1al. 22
4. Control experiments. 23
  4.1. Table S1. Screening of carbenoid precursors. 23
  4.2. Table S2. Condition optimization for the synthesis of 3a. 25
  4.3. Screening of diboron reagents 2b-2e. 26
5. Synthesis of chiral *gem*-diborylalkanes 3. 28
  5.1. Synthesis of compounds 3a, (*R*)-3a, 3d-3ad. 28
  5.2. Synthesis of compound 3ae. 51
  5.3. Gram-scale synthesis of compound 3t. 53
  5.4. Limitations of the substrate scope. 54
6. Transformations of chiral *gem*-diborylalkane products 3. 54
7. Synthesis of boron-stereogenic compounds using CDBA reagent. 72
8. Synthesis of boron-containing drug molecules. 80
9. Synthesis of fully substituted CDBAs and their applications. 85
10. Synthesis and transformations of 1,2- and 1,3-diborylalkanes. 93
  10.1. Synthesis of compound 19. 93
  10.2. Synthesis of compound 20. 94
  10.3. Synthesis of compounds 21 and 24. 96
  10.4. Transformations of compounds 21 and 24. 97
  10.5. Synthesis of compounds 28 and 29. 103
11. X-ray crystallographic data. 106
12. References. 111
13. NMR spectra 114

1. General information.

Unless noted otherwise, all solvents were dried and distilled from sodium benzophenone ketyl immediately prior to use. *N,N,N',N'*-tetramethylethylenediamine (TMEDA) was purchased from Aldrich and distilled over CaH_2 prior to use. 2,2,6,6-Tetramethylpiperidine was purchased from Aldrich and distilled over CaH_2 prior to use. (+)-Sparteine was purchased from Aladdin and used without further purification. (–)-Sparteine was prepared from the (–)-sparteine sulfate pentahydrate which was purchased from Aldrich.

Reaction temperatures were reported as the temperatures of the bather surrounding the flasks or vials. Sensitive reagents and solvents were transferred under nitrogen or handled in an argon-filled glovebox with standard techniques. All air and water sensitive reactions were carried out in oven dried glassware under nitrogen or argon atmosphere. Analytical thin-layer chromatography (TLC) was carried out using 0.2 mm commercial silica gel plates (TLC silica gel 60 F254 glass plates). Preparative thin-layer chromatography (PTLC) was carried out using 1.0 mm commercial silica gel plates (TLC silica gel 60 F254 glass plates). Flash column chromatography was done on silica gel (Merck Kieselgel 60). Deactivated silica gel was prepared according to literature reported procedures.[1] A slurry of silica gel in a 5 mol% NaOAc aqueous solution for 30 min. The deactivated silica gel was then collected by filtration and dried in an oven at 130 °C oven for 3 days. Vials (13 × 80 mm) pressure vessel, heavy wall (4 mL) and Schlenk tube, with vacuum valve and a Teflon screw cap (10 mL) were purchased from Synthware glass and flame-dried or put in an oven overnight.

Chiral HPLC separations were done on Agilent 1100 series normal phase high performance liquid chromatography, Waters 1525 System with 2998 PDA and Thermo HPLC UltiMate 3000 with *n*-hexane/isopropanol as the eluents. Daicel Chiralcel OJ-3, OX-H, OD-H columns (0.46 × 25 cm) and Daicel ChiralPAK IA-3, IB, IC, IG, AD-H (0.46 × 25 cm), IB-3 (0.46 × 15 cm) were used for normal phase separations. Preparative HPLC was performed using Shimadzu, DGU-20A5, Japan, equipped with a C18 column (HPLC Column, Agilent, ZORBAX 300SB-C18, 5 µm, 9.4 × 250 mm). 0.1% TFA in H_2O (v/v) and 0.1% TFA in CH_3CN (v/v) were used as the mobile phases, respectively.

High resolution mass spectra (HRMS) were obtained on an Thermo Q Exactive Focus Orbitrap Mass Spectrometer or a Waters Xevo G2-XS Tof mass spectrometer and are reported as m/z (relative intensity). Accurate masses are reported for the molecular ion M^+, $[M+H]^+$, $[M+Na]^+$. Infrared spectra were recorded on a Bruker Alpha spectrometer using a setup with a ZnSe crystal in the attenuated total reflection (ATR) mode or a Bruker TENSOR 27 spectrometer using neat thin film technique and reported in terms of frequency of absorption (cm^{-1}). Nuclear magnetic resonance spectra (^{1}H NMR, ^{13}C NMR, ^{11}B NMR, ^{19}F NMR) were recorded with a Bruker Avance III 400 NMR Spectrometer (400 MHz, ^{1}H at 400 MHz, ^{13}C at 101 MHz, ^{19}F at 377 MHz, ^{11}B at 128 MHz, ^{2}H at 61 MHz), a Bruker Avance III HD 500 NMR Spectrometer (500 MHz, ^{1}H at 500 MHz, ^{13}C at 126 MHz, ^{11}B at 160 MHz, ^{19}F at 471 MHz) and a JEOL superconducting magnet high-field NMR spectrometer (600 MHz, ^{1}H NMR at 600 MHz, ^{13}C NMR at 151 MHz). Chemical shifts are reported in parts per million (ppm,

δ), downfield from tetramethylsilane (TMS, δ = 0.00 ppm) and are referenced to residual solvent ($CDCl_3$, δ = 7.26 ppm (^{1}H) and 77.16 ppm (^{13}C); C_6D_6, δ = 7.16 ppm (^{1}H) and 128.06 ppm (^{13}C); toluene-d_8, δ = 7.09 ppm, 7.01 ppm, 6.97 ppm and 2.08 ppm (^{1}H); CD_3CN, δ = 1.96 ppm (^{1}H) and 118.26, 1.79 ppm (^{13}C)). All the ^{11}B chemical shifts were referenced to external $BF_3 \cdot OEt_2$ (0.00 ppm). All the ^{19}F chemical shifts were not referenced. Coupling constants were reported in Hertz (Hz). Data for ^{1}H NMR spectra were reported as follows: chemical shift (ppm, referenced to protium; s = singlet, d = doublet, t = triplet, q = quartet, dd = doublet of doublets, m = multiplet, br = broad, coupling constant (Hz), and integration).

2. Synthesis of diboron reagents 2a-2g and ^{10}B-2a.

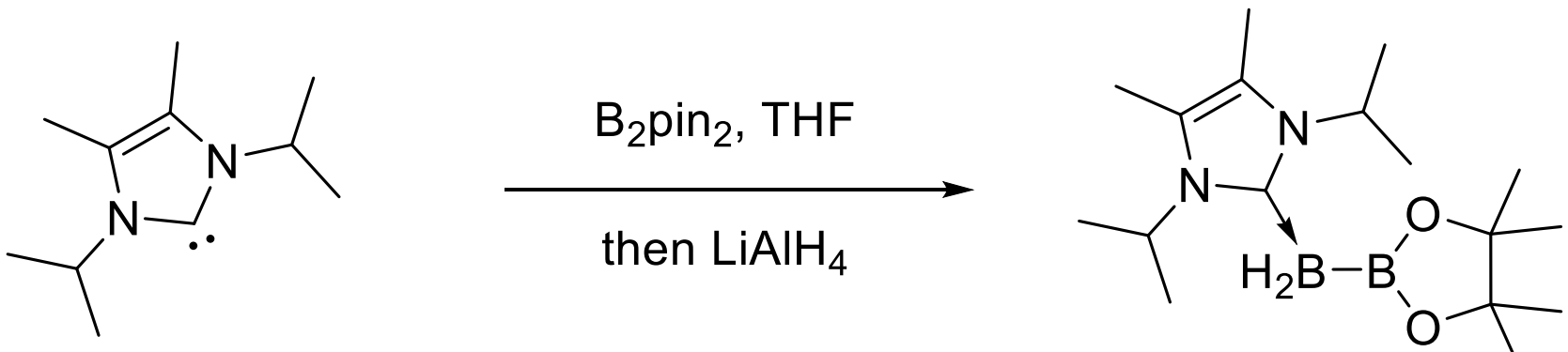

1,3-diisopropyl-4,5-dimethylimidazol-2-ylidene

2a

According to a reported literature,[2] to an oven dried 500 mL round-bottom flask charged with bis(pinacolato)diboron (7.62 g, 30.0 mmol, 1.0 equiv.) and THF (250 mL) was slowly added 1,3-diisopropyl-4,5-dimethylimidazol-2-ylidene (5.40 g, 30.0 mmol, 1.0 equiv.). The mixture was stirred at room temperature for 6 h and then cooled down to -36 °C. $LiAlH_4$ (911 mg, 24.0 mmol, 0.8 equiv.) was added in small portions into the cooled reaction mixture. The reaction mixture was then warmed to room temperature and stirred overnight under argon. The resulting mixture was filtered through a pad of Celite and washed with ethyl acetate. The filtrate was concentrated to dryness in vacuo. The resulting residue was further purified by flash column chromatography to afford the desired product **2a** (53%, 5.1 g).

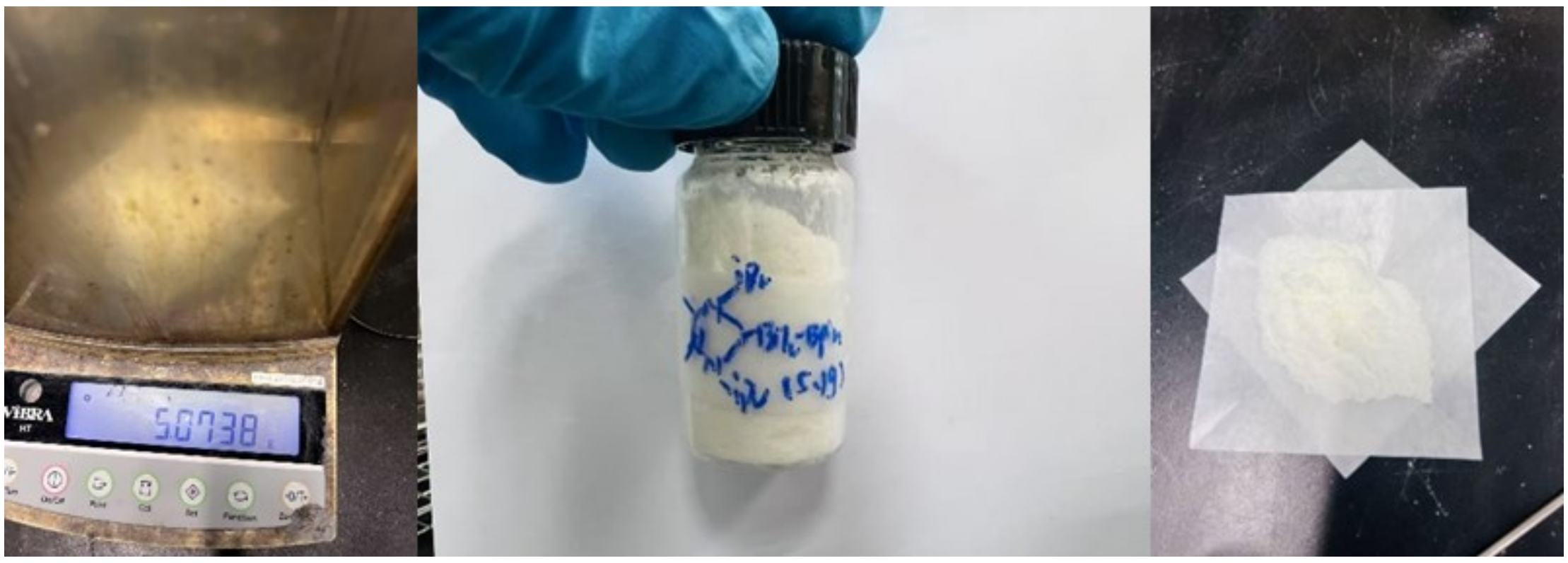

Fig. S1. Gram scale synthesis of **2a**.

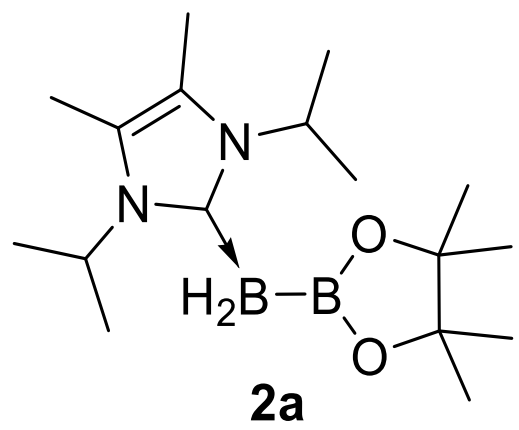

2a

2a: Yield: 5.1 g, 53%, white solid, R_f = 0.4 (*n*-hexane/ethyl acetate = 3/1).
^{1}H NMR (400 MHz, $CDCl_3$) δ 5.32 – 5.28 (m, 2H), 2.16 (s, 6H), 1.41 (d, *J* = 7.2 Hz, 12H), 1.13 (s, 12H).
^{13}C NMR (101 MHz, $CDCl_3$) δ 123.2, 80.9, 49.6, 25.3, 21.3, 10.5.
^{11}B NMR (128 MHz, $CDCl_3$) δ 41.8 (s, 1B), -38.6 (t, *J* = 75.4 Hz, 1B).
The NMR data matched with those reported in literature.[2]

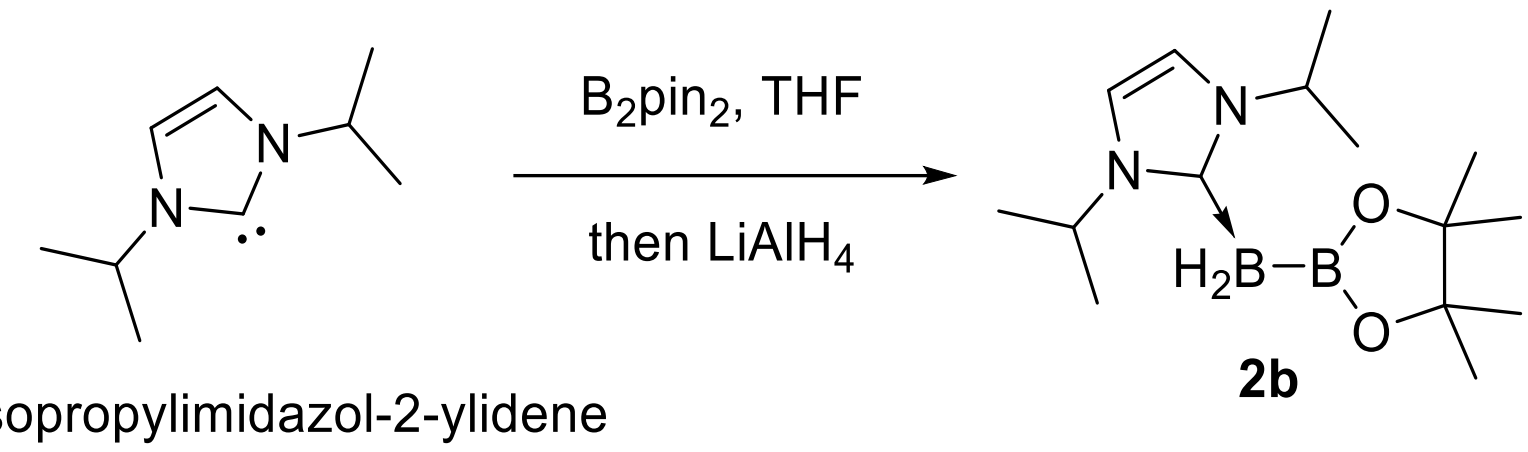

According to a reported literature,[2] to an oven dried 500 mL round-bottom flask charged with bis(pinacolato)diboron (2.52 g, 10.0 mmol, 1.0 equiv.) and THF (40 mL) was slowly added 1,3-diisopropylimidazol-2-ylidene (1.52 g, 10.0 mmol, 1.0 equiv.). The mixture was stirred at room temperature for 3 h and then cooled down to -36 °C. $LiAlH_4$ (228 mg, 6.0 mmol, 0.6 equiv.) was added in small portions into the cooled reaction mixture. The reaction mixture was then warmed to room temperature and stirred overnight under argon. The resulting mixture was filtered through a pad of Celite and washed with ethyl acetate. The filtrate was concentrated to dryness in vacuo. The resulting residue was further purified by flash column chromatography to afford the desired product **2b** (51%, 1.5 g).

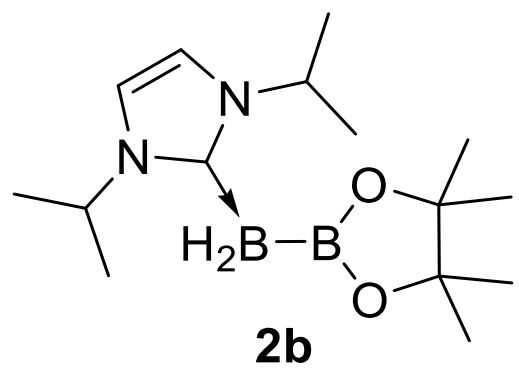

2b

2b: Yield: 1.5 g, 51%, white solid, R_f = 0.4 (*n*-hexane/ethyl acetate = 3/1).
^{1}H NMR (400 MHz, C_6D_6) δ 6.17 (s, 2H), 5.28 – 5.16 (m, 2H), 1.20 (s, 12H), 1.03 (d, *J* = 6.8 Hz, 12H).
The NMR data matched with those reported in literature.[2]

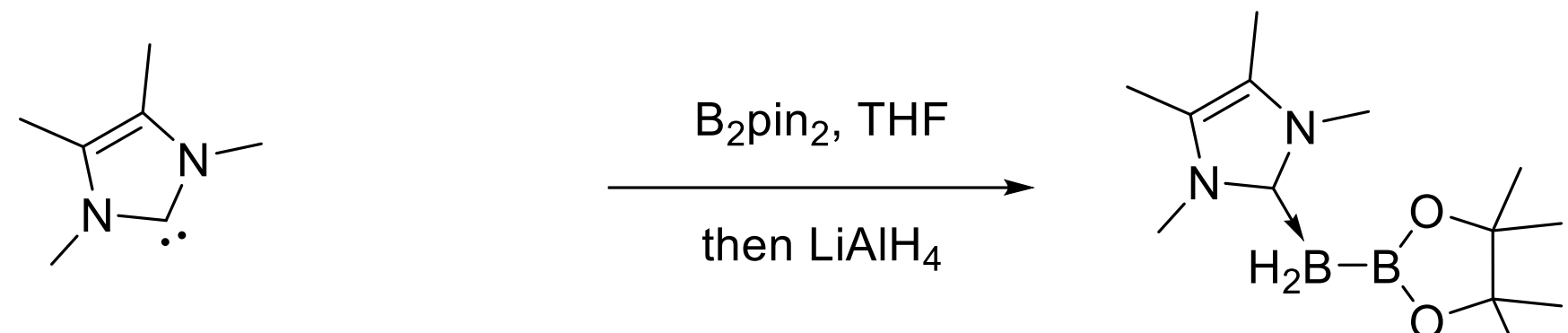

1,3,4,5-tetramethylimidazol-2-ylidene

2c

To an oven dried 100 mL round-bottom flask charged with bis(pinacolato)diboron (1.27 g, 5.0 mmol, 1.0 equiv.) and THF (50 mL) was slowly added the 1,3,4,5-tetramethylimidazol-2-ylidene (0.62 g, 5.0 mmol, 1.0 equiv.). The mixture was stirred at room temperature for 6 h and then cooled down to -36 °C. $LiAlH_4$ (152 mg, 4.0 mmol, 0.8 equiv.) was added in small portions into the cooled reaction mixture. The reaction mixture was then warmed to room temperature and stirred overnight under argon. The resulting mixture was filtered through a pad of Celite and washed with ethyl acetate. The filtrate was concentrated to dryness in vacuo. The resulting residue was further purified by flash column chromatography to afford the desired product **2c** (13%, 171.6 mg).

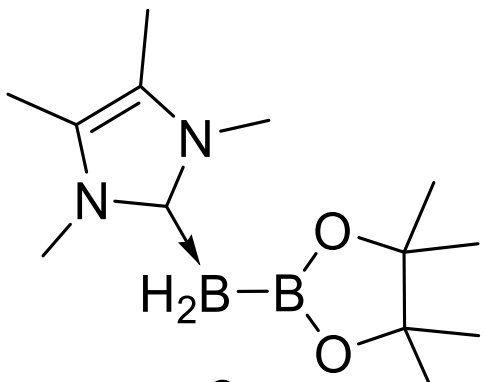

2c

2c: Yield: 171.6 mg, 13%, white solid, m.p. = 85 – 92 °C, R_f = 0.4 (*n*-hexane/ethyl acetate = 2/1).
^{1}H NMR (400 MHz, $CDCl_3$) δ 3.48 (s, 6H), 2.00 (s, 6H), 1.11 (s, 12H).
^{13}C NMR (101 MHz, $CDCl_3$) δ 122.6, 80.8, 32.4, 25.1, 8.8.
^{11}B NMR (128 MHz, $CDCl_3$) δ 41.3 (s, 1B), -39.4 (t, *J* = 64.9 Hz, 1B).
HRMS (ESI): Calcd for $C_{13}H_{26}B_2N_2O_2Na^+[M+Na]^+$: 287.2073, found: 287.2071.
IR (neat, cm^{-1}): *ν* 2976, 2926, 2856, 2319, 1660, 1470, 1458, 1442, 1394, 1369, 1263, 1233, 1141, 1090, 1007.

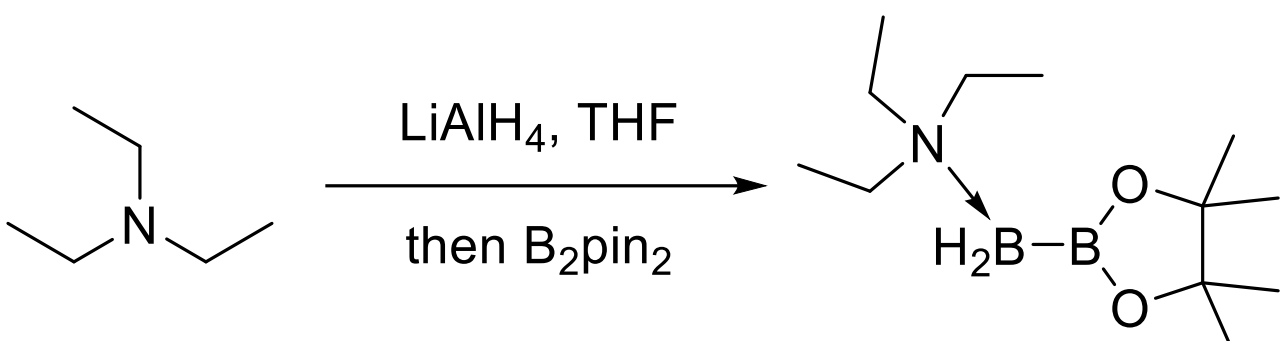

2d

To an oven dried 250 mL round-bottom flask charged with $LiAlH_4$ (380 mg, 10.0 mmol, 1.0 equiv.) and THF (100 mL) was slowly added the triethylamine (1.01 g, 10.0 mmol, 1.0 equiv.). The mixture was stirred at room temperature under argon for 12 h. Then bis(pinacolato)diboron (2.54 g, 10.0 mmol, 1.0 equiv.) was added in small portions into the reaction mixture and the mixture was stirred overnight under argon. The resulting mixture was filtered through a pad of Celite and washed with ethyl acetate. The filtrate was concentrated to dryness in vacuo. The resulting residue was further purified by flash column chromatography to afford the desired product **2d** (1.1 g, 46%).

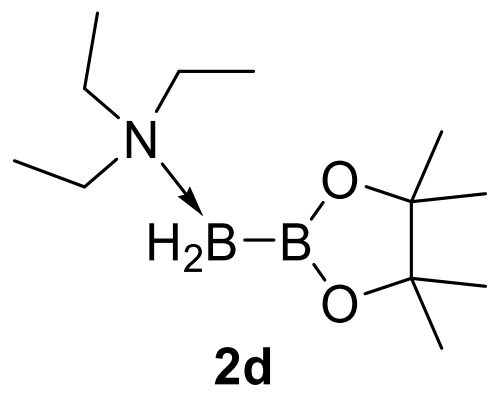

2d

2d: Yield: 1.1 g, 46%, colorless oil, R_f = 0.5 (*n*-hexane/ethyl acetate = 3:1).
^{1}H NMR (400 MHz, C_6D_6) δ 2.59 (q, *J* = 7.3 Hz, 6H), 1.19 (s, 12H), 0.81 (t, *J* = 7.3 Hz, 9H).
^{13}C NMR (101 MHz, C_6D_6) δ 80.8, 52.3, 25.5, 8.6.
^{11}B NMR (128 MHz, C_6D_6) δ 40.3 (s, 1B), -14.9 (br, 1B).
HRMS (ESI): Calcd for $C_{12}H_{29}B_2NO_2Na$ $[M+Na]^+$: 264.2281, found: 264.2276.
IR (neat, cm^{-1}): *ν* 2974, 2338, 1213, 1146, 1058.

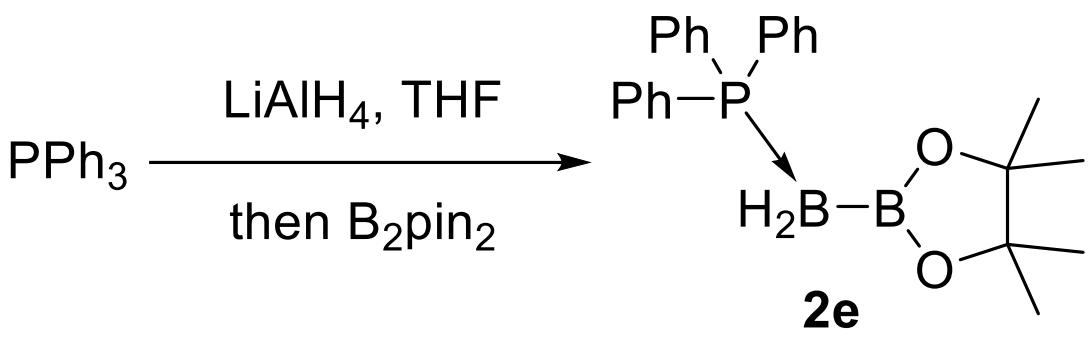

To an oven dried 100 mL round-bottom flask charged with $LiAlH_4$ (38.0 mg, 1.0 mmol, 1.0 equiv.) and THF (10 mL) was slowly added the triphenylphosphine (262 mg, 1.0 mmol, 1.0 equiv.). The mixture was stirred at room temperature for 12 h and then cooled down to -36 °C. Bis(pinacolato)diboron (254 mg, 1.0 mmol, 1.0 equiv.) was added in small portions into the cooled reaction mixture and the mixture was stirred overnight under argon. The resulting mixture was filtered through a pad of Celite and washed with ethyl acetate. The filtrate was concentrated to dryness in vacuo. The resulting residue was further purified by flash column chromatography to afford the desired product **2e** (85%, 342.6 mg).

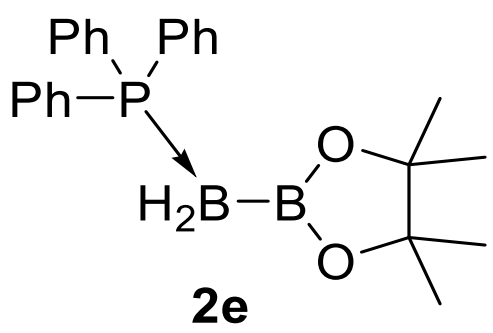

2e

2e: Yield: 342.6 mg, 85%, white solid, m.p. = 164 – 170 °C, R_f = 0.3 (*n*-hexane/ethyl acetate = 10/1).
^{1}H NMR (400 MHz, C_6D_6) δ 7.83 – 7.74 (m, 6H), 7.06 – 7.00 (m, 9H), 1.05 (s, 12H).
^{13}C NMR (101 MHz, C_6D_6) δ 134.0, 133.9, 131.6, 131.1, 130.9, 130.9, 128.7, 128.6, 81.4, 25.4.
^{11}B NMR (128 MHz, C_6D_6) δ 41.3 (s, 1B), -41.07 (t, *J* = 117.5 Hz, 1B)
HRMS (ESI): Calcd for $C_{24}H_{29}B_2O_2PNa^+$ $[M+Na]^+$: 425.1989, found: 425.1984.
IR (neat, cm^{-1}): *ν* 3054, 2987, 2306, 1422, 1264.

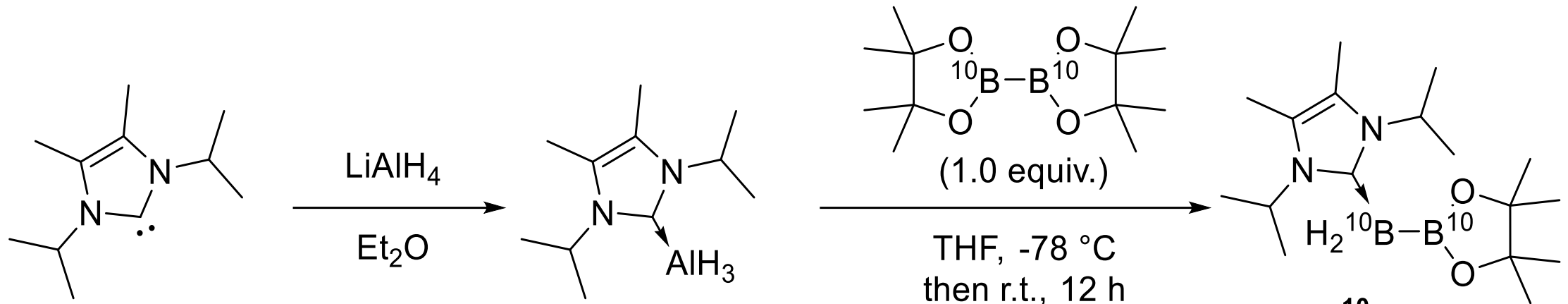

NHC-LAH **^{10}B-2a**

To an oven dried 100 mL round-bottom flask charged with $LiAlH_4$ (45.6 mg, 1.2 mmol, 1.2 equiv.) was added dry Et_2O (6 mL). The round-bottom flask was stored at -36 °C for 1 h before 1,3-diisopropyl-4,5-dimethylimidazol-2-ylidene (216 mg, 1.2 mmol, 1.2 equiv.) was added small portions to the solution. The reaction mixture was then warmed to room temperature and stirred overnight under argon. Upon completion, the resulting mixture was filtered thought a pad of Celite and washed with dry Et_2O. The filtrate was concentrated to dryness in vacuo to give crude **NHC-LAH** as white solid which was directly used for the next step without further purification.

To an oven dried 100 mL round-bottom flask charged with ^{10}B-bis(pinacolato)diboron (252 mg, 1.0 mmol, 1.0 equiv.) was added dry THF (5 mL). The **NHC-LAH** solution (1.2 mmol crude sample in 2 mL THF) was added dropwise to the solution at -78 °C. The reaction mixture was then warmed to room temperature and stirred overnight under argon. Upon completion, the resulting mixture was filtered through a pad of Celite and washed with ethyl acetate. The filtrate was concentrated to dryness in vacuo. The resulting residue was further purified by flash column chromatography to afford the desired product **^{10}B-2a** (30%, 95.4 mg).

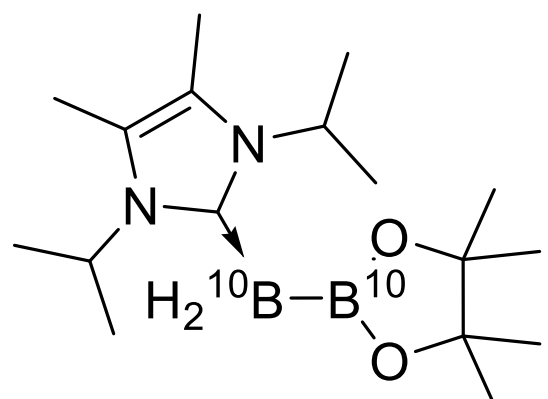

^{10}B-2a

^{10}B-2a: Yield: 95.4 mg, 30%, white solid, R_f = 0.4 (*n*-hexane/ethyl acetate = 3/1).
^{1}H NMR (400 MHz, $CDCl_3$) δ 5.32 – 5.28 (m, 2H), 2.16 (s, 6H), 1.41 (d, *J* = 7.2 Hz, 12H), 1.13 (s, 12H).
^{13}C NMR (101 MHz, $CDCl_3$) δ 123.2, 80.9, 49.6, 25.3, 21.3, 10.5.
HRMS (ESI): Calcd for $C_{17}H_{34}{}^{10}B_2N_2O_2Na^+$ $[M+Na]^+$: 341.2771, found: 341.2769.
IR (neat, cm^{-1}): *v* 3055, 2974, 2931, 2371, 1464, 1399, 1388, 1369, 1264, 1225, 1141.

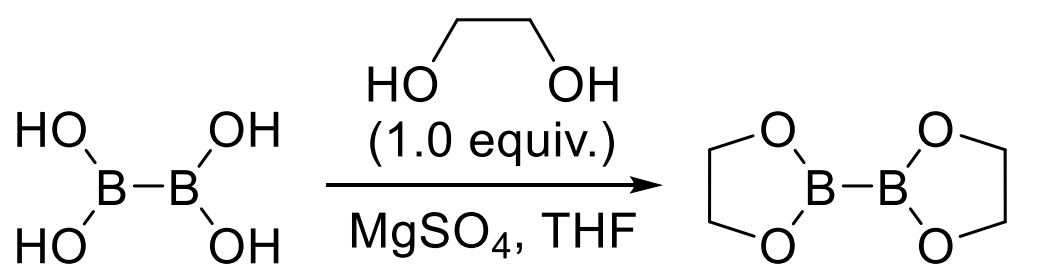

2f-S1

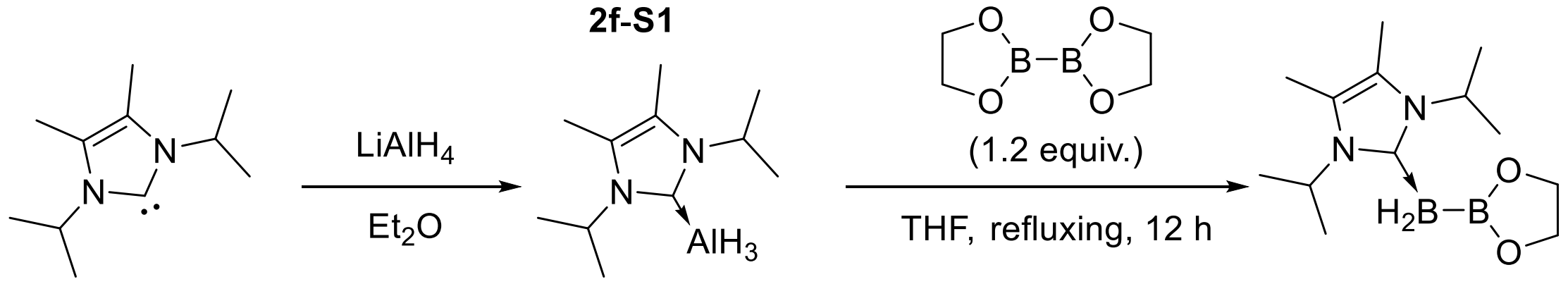

NHC-LAH **2f**

According to a reported literature,[3] to an oven dried 50 mL round-bottom flask charged

with hypodiboric acid (1.08 g, 12.0 mmol, 1.0 equiv.), $MgSO_4$ (12.0 g, 100 mmol, 8.0 equiv.) and THF (20 mL) was added ethane-1,2-diol (1.49 g, 24.0 mmol, 2.0 equiv.). The reaction mixture was stirred at room temperature for 12 h. Upon completion, the resulting mixture was filtered through a pad of Celite and washed with ethyl acetate. The filtrate was concentrated to dryness in vacuo. The residual solid was washed with *n*-hexane to obtain the **2f-S1** which was directly used for the next step without further purification.

To another oven dried 100 mL round-bottom flask charged with $LiAlH_4$ (380 mg, 10.0 mmol, 1.0 equiv.) was added dry Et_2O (60 mL). The round-bottom flask was stored at -36 °C for 1 h before 1,3-diisopropyl-4,5-dimethylimidazol-2-ylidene (1.80 g, 10.0 mmol, 1.0 equiv.) was added in small portions to the solution. The reaction mixture was then warmed to room temperature and stirred overnight under argon. Upon completion, the resulting mixture was filtered thought a pad of Celite and washed with dry Et_2O. The filtrate was concentrated to dryness in vacuo to give crude **NHC-LAH** as white solid which was directly used for the next step without further purification.

To an oven dried 100 mL round-bottom flask charged with crude **2f-S1** (*in-situ*, 12.0 mmol, 1.2 equiv.) was added dry THF (30 mL). The mixture was pre-heated to reflux for 5 min. The **NHC-LAH** solution (10 mmol crude sample in 10 mL THF) was then slowly added to the solution. The reaction mixture was then kept refluxing and stirred for another 12 h. Upon completion, the resulting mixture was filtered through a pad of Celite and washed with ethyl acetate. The filtrate was concentrated to dryness in vacuo. The resulting residue was further purified by flash column chromatography to afford the desired product **2f** (30%, 792.0 mg).

Note: To ensure optimal separation and product stability, it is recommended that flash column chromatography be completed within 30 minutes utilizing deactivated silica gel.

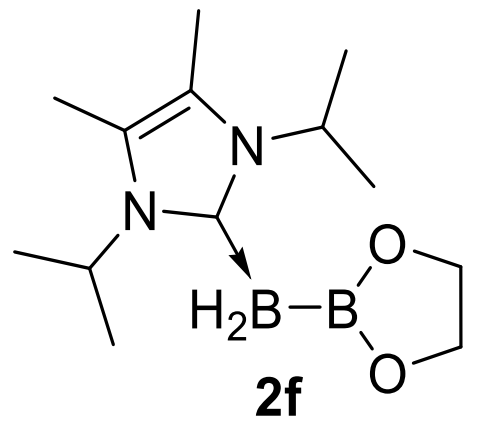

2f

2f: Yield: 792.0 mg, 30%, white solid, m.p. = 128 – 133 °C, R_f = 0.3 (*n*-hexane/ethyl acetate = 1/1).

^{1}H NMR (500 MHz, $CDCl_3$) δ 5.43 – 5.23 (m, 2H), 3.99 (s, 4H), 2.19 (s, 6H), 1.43 (d, J = 7.2 Hz, 12H).

^{13}C NMR (126 MHz, $CDCl_3$) δ 64.5, 49.8, 21.4, 10.6.

^{11}B NMR (160 MHz, $CDCl_3$) δ 41.5 (s, 1B), -39.5 (t, J = 75.6 Hz, 1B).

HRMS (ESI): Calcd for $C_{13}H_{26}B_2N_2O_2Na^+$ $[M+Na]^+$: 287.2073, found: 287.2070.

IR (neat, cm^{-1}): ν 2974, 2894, 2322, 1400, 1370, 1223, 1137, 1016.

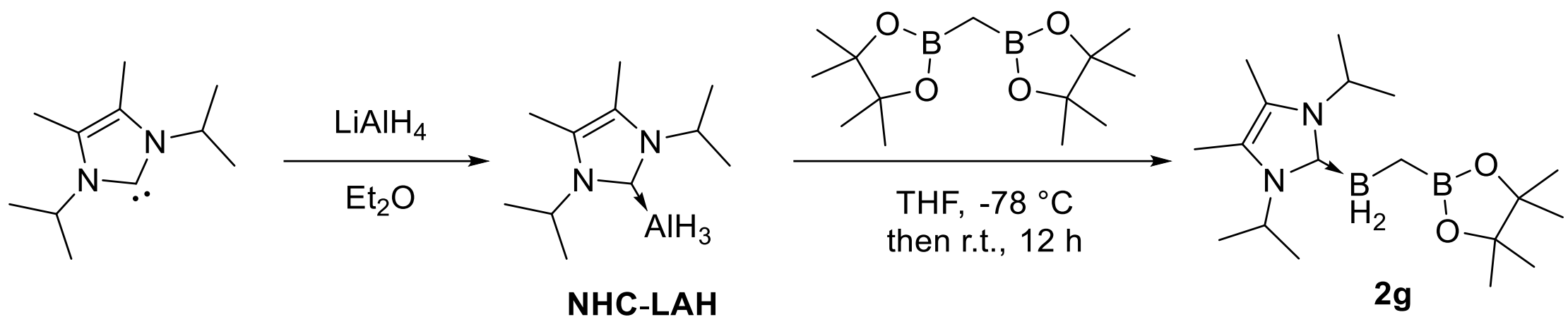

To an oven dried 100 mL round-bottom flask charged with $LiAlH_4$ (456 mg, 12.0 mmol, 1.2 equiv.) was added dry Et_2O (60 mL). The round-bottom flask was stored at -36 °C for 1 h before the addition of 1,3-diisopropyl-4,5-dimethylimidazol-2-ylidene (2.16 g, 12.0 mmol, 1.2 equiv.) in small portions to the solution. The reaction mixture was then warmed to room temperature and stirred overnight under argon. Upon completion, the resulting mixture was filtered thought a pad of Celite and washed with dry Et_2O. The filtrate was concentrated to dryness in vacuo to give crude **NHC-LAH** as white solid which was directly used for the next step without further purification.

To an oven dried 100 mL round-bottom flask charged with bis[(pinacolato)boryl]methane (2.68 g, 10.0 mmol, 1.2 equiv.) was added dry THF (60 mL). The **NHC-LAH** solution (12.0 mmol crude sample in 10 mL THF) was added dropwise to the solution at -78 °C. The reaction mixture was then warmed to room temperature and stirred overnight under argon. Upon completion, the resulting mixture was filtered through a pad of Celite and washed with ethyl acetate. The filtrate was concentrated to dryness in vacuo. The resulting residue was further purified by flash column chromatography to afford the desired product **2g** (30%, 1.0 g).

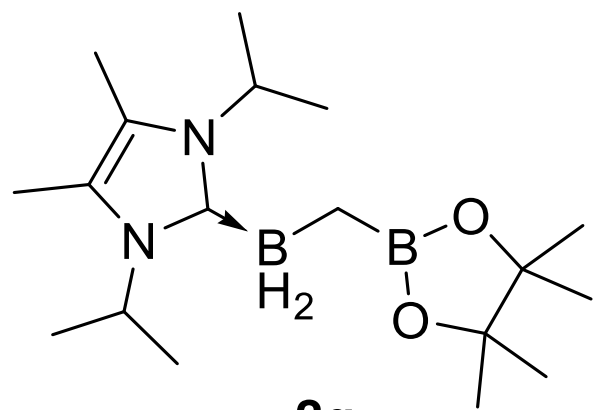

2g

2g: Yield: 1.0 g, 30%, white crystal, m.p. = 104 – 107 °C, R_f = 0.5 (*n*-hexane/ethyl acetate = 3:1).

^{1}H NMR (500 MHz, $CDCl_3$) δ 5.53 – 5.27 (m, 2H), 2.15 (s, 6H), 1.38 (d, *J* = 7.2 Hz, 12H), 1.20 (s, 12H), -0.23 – -0.78 (m, 2H).

^{13}C NMR (126 MHz, $CDCl_3$) δ 123.4, 81.4, 49.3, 24.8, 21.7, 10.4.

^{11}B NMR (160 MHz, $CDCl_3$) δ 36.2 (s, 1B), -28.6 (t, *J* = 85.4 Hz, 1B).

HRMS (ESI): Calcd for $C_{18}H_{36}B_2N_2O_2Na^+$ $[M+Na]^+$: 357.2856, found: 357.2849.

IR (neat, cm^{-1}): ν 2975, 2928, 2278, 1401, 1309, 1283, 1221, 1150.

3. Synthesis of carbamates 1.

Compounds **1a**, **1d-1z**, **1aa**, **1ab**, **1ac**, **1ad**, **1ae**, **1af**, **1ag**, **1ah**, **1ai**, **1aj**, **1ak** and **1al** were prepared according to the following procedures.

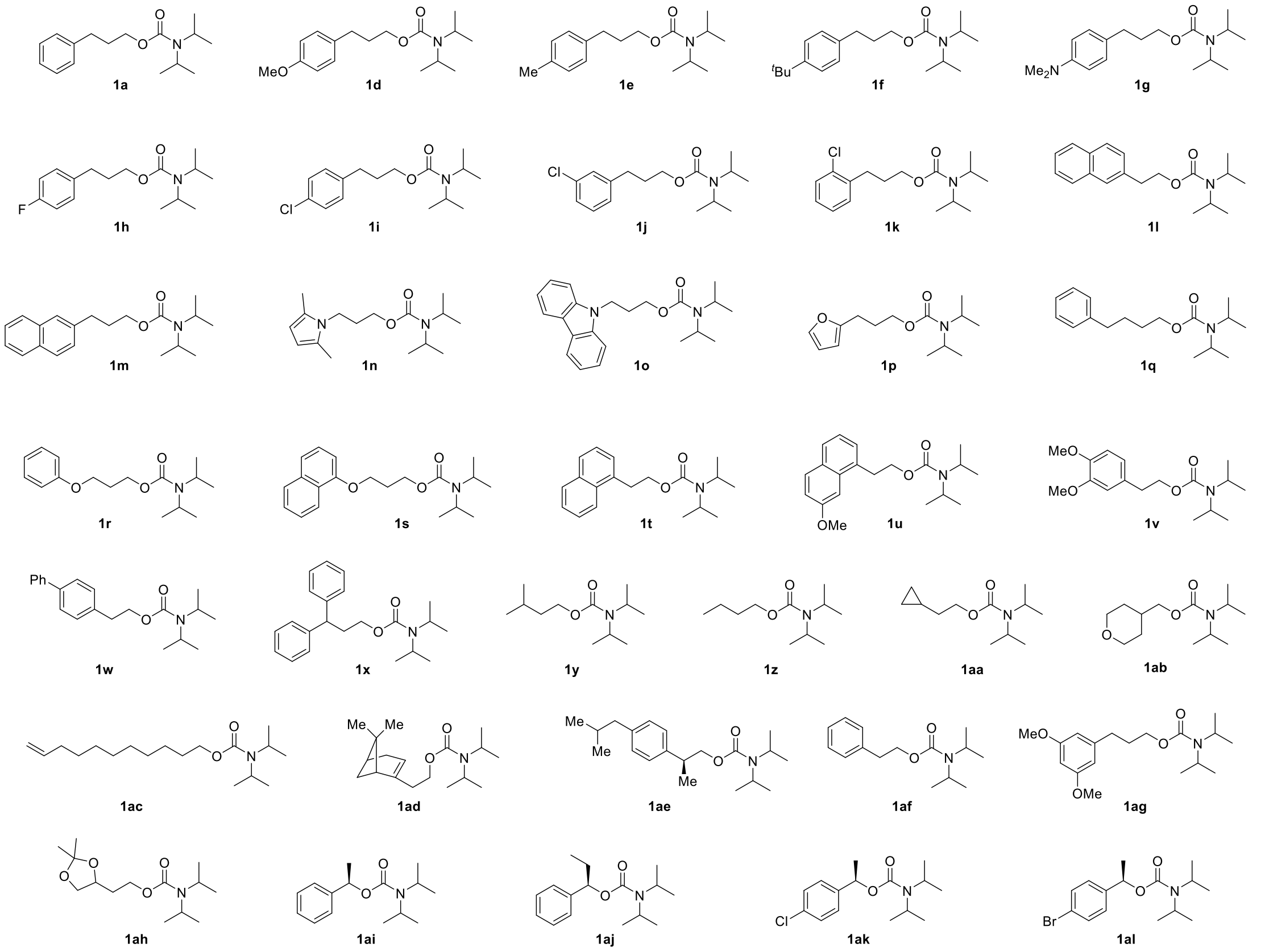

3.1. Preparation of compounds 1a, 1l, 1q, 1r, 1t, 1w, 1x-1z, 1aa-1ac, 1af and 1ah.

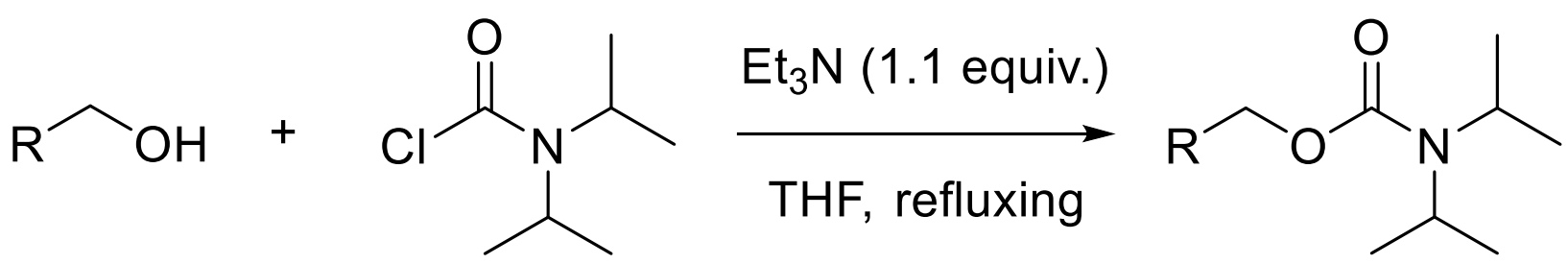

According to a reported literature,[4] to a Schlenk tube charged with *N,N*-diisopropylcarbamoyl chloride (897 mg, 5.5 mmol, 1.1 equiv.), Et_3N (556 mg, 5.5 mmol, 1.1 equiv.) and THF (10 mL) was added alcohol derivatives (5.0 mmol, 1.0 equiv.). The reaction mixture was then heated to reflux and stirred for 24 h. Upon completion, the resulting mixture was filtered through a pad of Celite and washed with ethyl acetate. The filtrate was concentrated to dryness in vacuo. The resulting residue was further purified by flash column chromatography to afford the desired products.

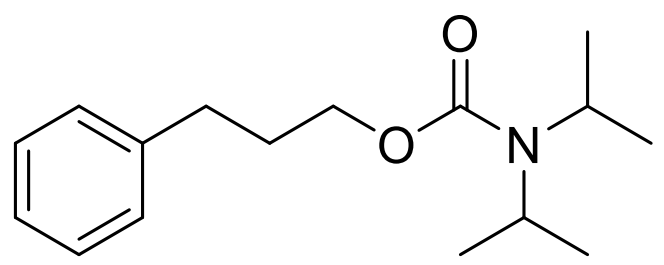

1a

1a: Yield: 1.3 g, 99%, colorless oil, R_f = 0.4 (*n*-hexane/ethyl acetate = 5/1).

^{1}H NMR (400 MHz, $CDCl_3$) δ 7.33 – 7.27 (m, 2H), 7.25 – 7.15 (m, 3H), 4.12 (t, *J* = 6.5 Hz, 2H), 4.03 – 3.71 (m, 2H), 2.76 – 2.68 (m, 2H), 2.27 – 1.83 (m, 2H), 1.26 – 1.00 (m, 12H).

The NMR data matched with those reported in literature.[4]

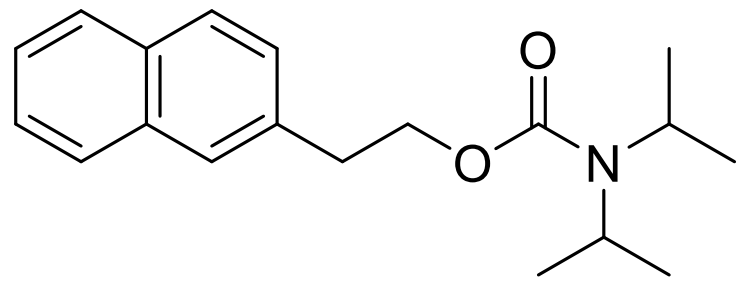

1l

1l: Yield: 1.5 g, 99%, white solid, m.p. = 47 – 51 °C, R_f = 0.4 (*n*-hexane/ethyl acetate = 5/1).

^{1}H NMR (600 MHz, $CDCl_3$) δ 7.88 – 7.74 (m, 3H), 7.74 – 7.68 (m, 1H), 7.49 – 7.35 (m, 3H), 4.61 – 4.31 (m, 2H), 4.09 – 3.68 (m, 2H), 3.36 – 3.01 (m, 2H), 1.46 – 0.84 (m, 12H).

^{13}C NMR (151 MHz, $CDCl_3$) δ 155.7, 136.0, 133.7, 132.3, 128.1, 127.7, 127.6, 127.5, 126.1, 125.5, 65.2, 45.9 (br), 35.9, 21.0 (br).

HRMS (ESI): Calcd for $C_{19}H_{25}NO_2Na^+$ $[M+Na]^+$: 322.1778, found: 322.1780.

IR (neat, cm^{-1}): *v* 3052, 2969, 1683, 1434, 1266, 1218, 1070, 1050.

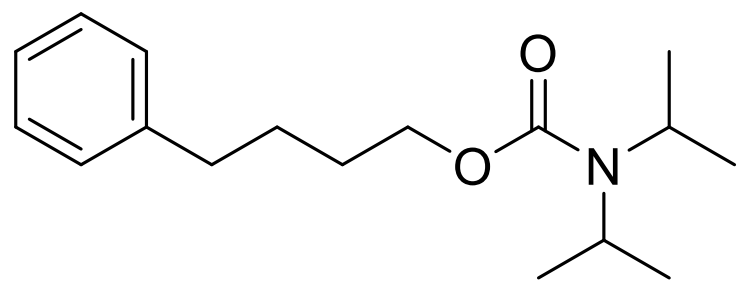

1q

1q: Yield: 1.3 g, 94%, colorless oil, R_f = 0.4 (*n*-hexane/ethyl acetate = 5/1).

^{1}H NMR (400 MHz, $CDCl_3$) δ 7.35 – 7.27 (m, 2H), 7.21 – 7.07 (m, 3H), 4.21 – 3.67 (m, 4H), 2.78 – 2.39 (m, 2H), 1.86 – 1.55 (m, 4H), 1.29 – 1.02 (m, 12H).

^{13}C NMR (101 MHz, $CDCl_3$) δ 155.9, 142.3, 128.5, 128.4, 125.8, 64.5, 45.8 (br), 35.6, 28.8, 28.1, 21.1 (br).

HRMS (ESI): Calcd for $C_{17}H_{27}NO_2Na^+$ $[M+Na]^+$: 300.1934, found: 300.1940.

IR (neat, cm^{-1}): *v* 2938, 1685, 1234, 1211.

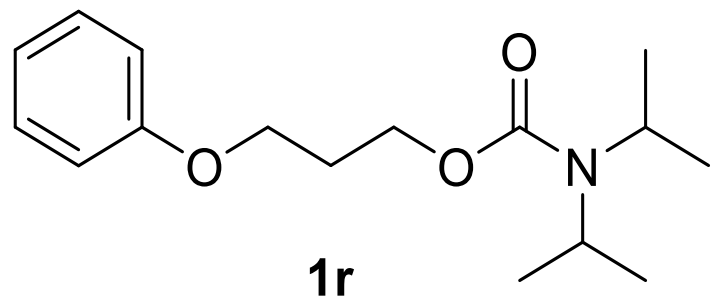

1r

1r: Yield: 1.1 g, 79%, colorless oil, R_f = 0.4 (*n*-hexane/ethyl acetate = 5/1).

^{1}H NMR (400 MHz, $CDCl_3$) δ 7.32 – 7.24 (m, 2H), 7.16 – 6.66 (m, 3H), 4.28 (t, *J* = 6.3 Hz, 2H), 4.07 (t, *J* = 6.3 Hz, 2H), 4.01 – 3.67 (m, 2H), 2.39 – 1.90 (m, 2H), 1.44 – 0.82 (m, 12H).

^{13}C NMR (101 MHz, $CDCl_3$) δ 159.0, 155.8, 129.6, 120.8, 114.6, 64.8, 61.7, 46.0 (br), 29.3, 21.2 (br).
HRMS (ESI): Calcd for $C_{16}H_{25}NO_3Na^+$ $[M+Na]^+$: 302.1727, found: 302.1735.
IR (neat, cm^{-1}): *v* 2969, 2934, 1687, 1244, 1217, 1066.

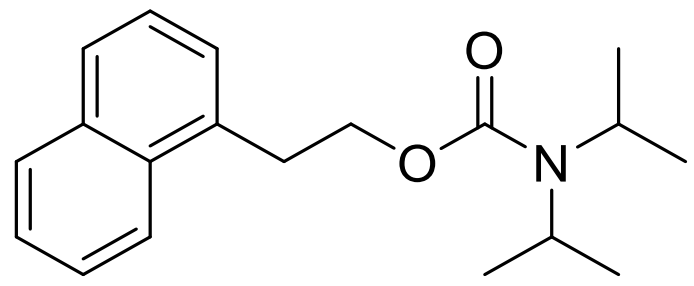

1t

1t: Yield: 1.4 g, 94%, yellow oil, R_f = 0.4 (*n*-hexane/ethyl acetate = 5/1).
^{1}H NMR (400 MHz, $CDCl_3$) δ 8.28 – 8.02 (m, 1H), 8.04 – 7.81 (m, 1H), 7.81 – 7.70 (m, 1H), 7.65 – 7.36 (m, 4H), 4.56 – 4.28 (m, 2H), 4.17 – 3.63 (m, 2H), 3.60 – 2.92 (m, 2H), 1.41 – 0.99 (m, 12H).
^{13}C NMR (101 MHz, $CDCl_3$) δ 155.7, 134.5, 134.0, 132.3, 128.9, 127.4, 127.2, 126.2, 125.7, 125.6, 124.0, 64.8, 45.9 (br), 32.9, 20.9 (br).
HRMS (ESI): Calcd for $C_{19}H_{25}NO_2Na^+$ $[M+Na]^+$: 322.1778, found: 322.1782.
IR (neat, cm^{-1}): *v* 3048, 2968, 2933, 1690, 1435, 1313, 1294, 1061.

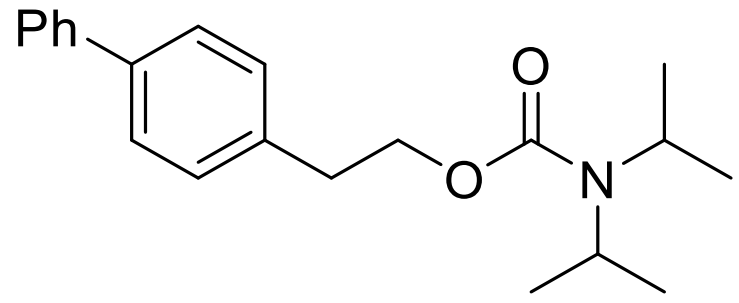

1w

1w: Yield: 1.5 g, 92%, white solid, m.p. = 50 – 55 °C, R_f = 0.4 (*n*-hexane/ethyl acetate = 5/1).
^{1}H NMR (400 MHz, $CDCl_3$) δ 7.72 – 7.51 (m, 4H), 7.50 – 7.41 (m, 2H), 7.39 – 7.30 (m, 3H), 4.44 – 3.63 (m, 4H), 3.02 (t, *J* = 7.0 Hz, 2H), 1.30 – 1.00 (m, 12H).
^{13}C NMR (101 MHz, $CDCl_3$) δ 155.7, 141.1, 139.4, 137.7, 129.5, 128.9, 127.2, 127.1, 65.2, 35.4, 21.1 (br).
HRMS (ESI): Calcd for $C_{21}H_{27}NO_2Na^+$ $[M+Na]^+$: 348.1934, found: 348.1939.
IR (neat, cm^{-1}): *v* 2968, 1685, 1485, 1310, 1291, 1071.

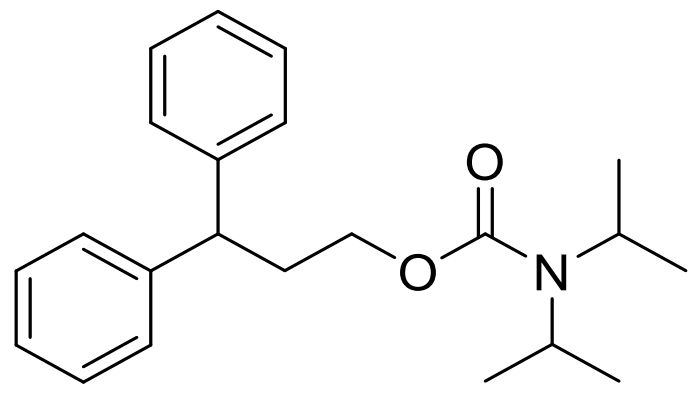

1x

1x: Yield: 1.4 g, 83%, colorless oil, R_f = 0.4 (*n*-hexane/ethyl acetate = 5/1).
^{1}H NMR (400 MHz, $CDCl_3$) δ 7.32 – 7.24 (m, 8H), 7.22 – 7.15 (m, 2H), 4.37 – 3.67 (m, 5H), 2.62 – 2.23 (m, 2H), 1.41 – 1.08 (m, 12H).
^{13}C NMR (101 MHz, $CDCl_3$) δ 155.8, 144.3, 128.7, 128.0, 126.5, 63.2, 48.1, 35.1, 21.8 (br).
HRMS (ESI): Calcd for $C_{22}H_{29}NO_2Na^+$ $[M+Na]^+$: 362.2091, found: 362.2094.
IR (neat, cm^{-1}): *v* 2967, 1686, 1435, 1308, 1289, 1156, 1048.

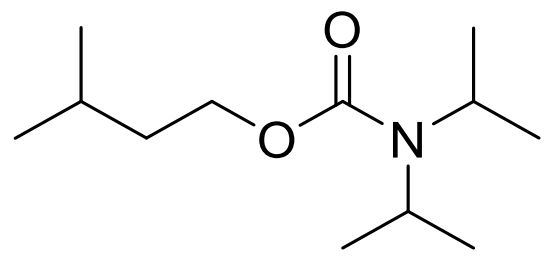

1y

1y: Yield: 870.8 mg, 81%, yellow oil, R*f* = 0.5 (*n*-hexane/ethyl acetate = 5/1).
¹H NMR (400 MHz, $CDCl_3$) δ 3.82 (t, *J* = 6.9 Hz, 2H), 3.77 – 3.41 (m, 2H), 1.56 – 1.36 (m, 1H), 1.35 – 1.15 (m, 2H), 1.04 – 0.74 (m, 12H), 0.75 – 0.55 (m, 6H).
The NMR data matched with those reported in literature.[4]

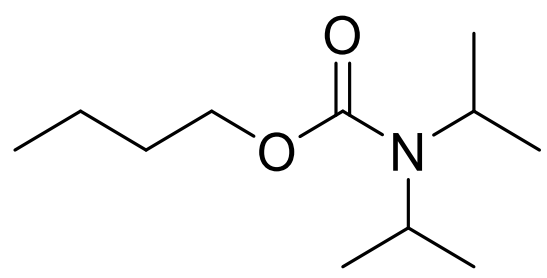

1z

1z: Yield: 995.0 mg, 99%, colorless oil, R*f* = 0.4 (*n*-hexane/ethyl acetate = 5/1).
¹H NMR (400 MHz, $CDCl_3$) δ 4.26 – 3.48 (m, 4H), 1.75 – 1.50 (m, 2H), 1.44 – 1.31 (m, 2H), 1.23 – 1.04 (m, 12H), 1.04 – 0.80 (m, 3H).
¹³C NMR (101 MHz, $CDCl_3$) δ 156.1, 64.5, 45.8 (br), 31.3, 21.1 (br), 19.5, 13.9.
HRMS (ESI): Calcd for $C_{11}H_{23}NO_2Na^+$ $[M+Na]^+$: 224.1621, found: 224.1618.
IR (neat, cm^{-1}): *ν* 2963, 1686, 1435, 1309, 1289, 1155, 1072, 1048.

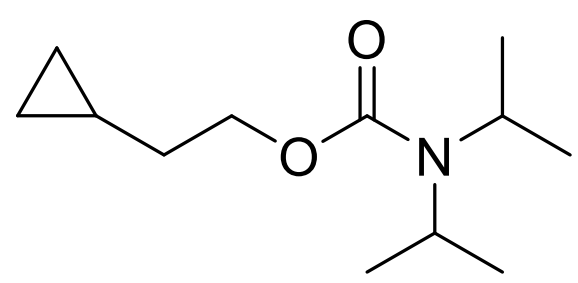

1aa

1aa: Yield: 1.0 g, 94%, colorless oil, R*f* = 0.4 (*n*-hexane/ethyl acetate = 5/1).
¹H NMR (400 MHz, $CDCl_3$) δ 4.23 – 3.49 (m, 4H), 1.53 (q, *J* = 6.8 Hz, 2H), 1.28 – 1.11 (m, 12H), 0.81 – 0.62 (m, 1H), 0.55 – 0.31 (m, 2H), 0.14 – 0.00 (m, 2H).
¹³C NMR (101 MHz, $CDCl_3$) δ 156.0, 64.9, 45.8 (br), 34.3, 21.1 (br), 8.1, 4.3.
HRMS (ESI): Calcd for $C_{12}H_{23}NO_2Na^+$ $[M+Na]^+$: 236.1621, found: 236.1618.
IR (neat, cm^{-1}): *ν* 2965, 1691, 1441, 1309, 1291, 1096.

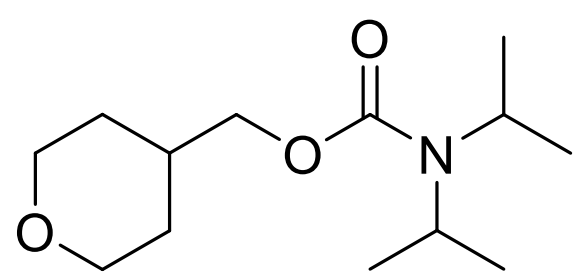

1ab

1ab: Yield: 1.1g, 92%, colorless oil, R*f* = 0.4 (*n*-hexane/ethyl acetate = 3/1).
¹H NMR (400 MHz, $CDCl_3$) δ 4.23 – 3.68 (m, 6H), 3.54 – 3.24 (m, 2H), 2.01 – 1.84 (m, 1H), 1.70 – 1.53 (m, 2H), 1.50 – 1.31 (m, 2H), 1.26 – 1.01 (m, 12H).
¹³C NMR (101 MHz, $CDCl_3$) δ 155.9, 69.3, 67.7, 46.4 (br), 35.0, 29.9, 20.9 (br).
HRMS (ESI): Calcd for $C_{13}H_{25}NO_3Na^+$ $[M+Na]^+$: 266.1727, found: 266.1730.
IR (neat, cm^{-1}): *ν* 2963, 2932, 2871, 1687, 1434, 1309, 1290, 1057.

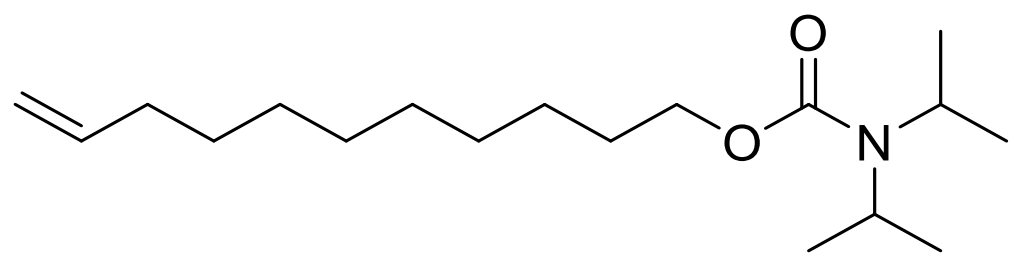

1ac

1ac: Yield: 1.4 g, 94%, colorless oil, R_f = 0.4 (*n*-hexane/ethyl acetate = 5/1).

^{1}H NMR (400 MHz, $CDCl_3$) δ 6.08 – 5.49 (m, 1H), 5.14 – 4.75 (m, 2H), 4.25 – 3.43 (m, 4H), 2.11 – 1.94 (m, 2H), 1.67 – 1.57 (m, 2H), 1.43 – 1.25 (m, 12H), 1.21 – 1.14 (m, 12H).

^{13}C NMR (101 MHz, $CDCl_3$) δ 156.1, 139.3, 114.2, 64.9, 45.8 (br), 33.9, 29.6, 29.5, 29.4, 29.2, 29.2, 29.1, 26.3, 21.2 (br).

HRMS (ESI): Calcd for $C_{18}H_{35}NO_2Na^+$ $[M+Na]^+$: 320.2560, found: 320.2564.

IR (neat, cm^{-1}): *v* 2926, 2855, 1689, 1435, 1309, 1289, 1156, 1069, 1049.

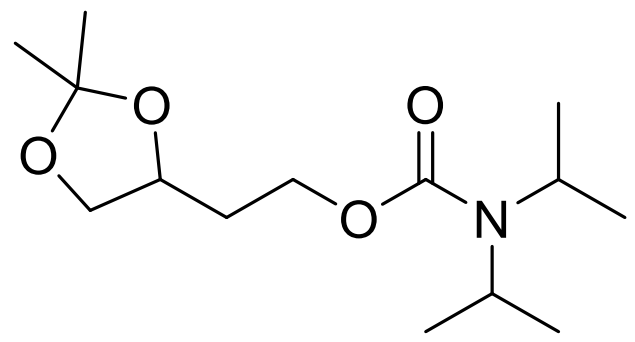

1ah

1ah: Yield: 969.2 mg, 71%, yellow oil, R_f = 0.4 (*n*-hexane/ethyl acetate = 5/1).

^{1}H NMR (500 MHz, $CDCl_3$) δ 4.29 – 3.71 (m, 6H), 3.62 – 3.44 (m, 1H), 2.06 – 1.83 (m, 2H), 1.40 (s, 3H), 1.34 (s, 3H), 1.27 – 1.06 (m, 12H).

^{13}C NMR (126 MHz, $CDCl_3$) δ 155.5, 108.9, 73.6, 69.6, 61.6, 46.3 (br), 33.4, 27.1, 25.8, 21.3 (br).

HRMS (ESI): Calcd for $C_{14}H_{27}NO_4Na^+$ $[M+Na]^+$: 296.1832, found: 296.1849.

IR (neat, cm^{-1}): *v* 2970, 2936, 2875, 1687, 1436, 1310.

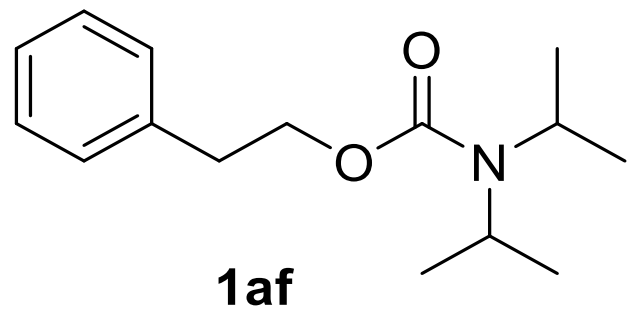

1af

1af: Yield: 1.0 g, 82%, colorless oil, R_f = 0.4 (*n*-hexane/ethyl acetate = 5/1).

^{1}H NMR (600 MHz, $CDCl_3$) δ 7.33 – 7.27 (m, 2H), 7.25 – 7.17 (m, 3H), 4.31 (t, *J* = 7.0 Hz, 2H), 4.12 – 3.57 (m, 2H), 2.97 (t, *J* = 7.0 Hz, 2H), 1.24 – 0.84 (m, 12H).

The NMR data matched with those reported in literature.[5]

3.2. Preparation of compounds 1d-1k, 1m, 1p, 1u-1v, 1ad, 1ae and 1ag.

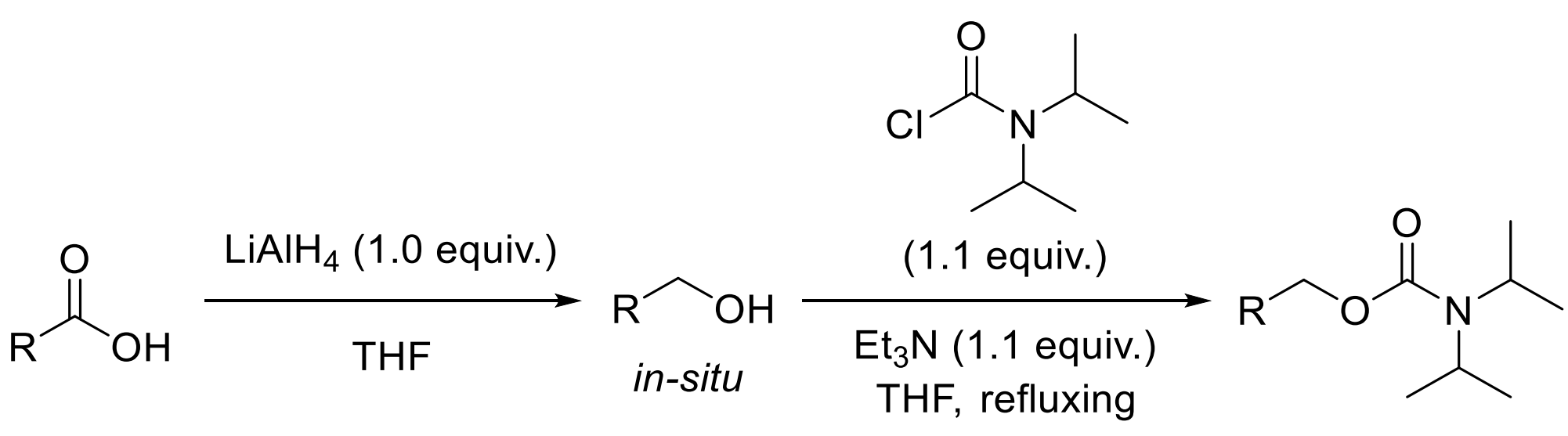

According to a reported literature,[6] an oven dried 50 mL round-bottom flask charged with $LiAlH_4$ (190 mg, 5.0 mmol, 1.0 equiv.) and THF (10 mL) was cooled to 0 °C.

Then, a solution of acid derivatives (5.0 mmol, 1.0 equiv.) in dry THF (5 mL) was added dropwise. The reaction mixture was warmed to room temperature and monitored by TLC. After the complete consumption of the starting material, the reaction mixture was cooled to 0 °C again and slowly poured into cold H_2O to quench the excess $LiAlH_4$. The resulting mixture was extracted with ethyl acetate (3 × 20 mL), dried over Na_2SO_4 and filtered through a pad of Celite. The filtrate was concentrated to dryness in vacuo to give the primary alcohol, which was used for next step without further purification. According to a reported literature,[4] to a Schlenk tube charged with *N,N*-diisopropylcarbamoyl chloride (897 mg, 5.5 mmol, 1.1 equiv.), Et_3N (556 mg, 5.5 mmol, 1.1 equiv.) and THF (10 mL) was added primary alcohol from the last step (5.0 mmol, 1.0 equiv.). The reaction mixture was then heated to reflux and stirred for 24 h. Upon completion, the resulting mixture was filtered through a pad of Celite and washed with ethyl acetate. The filtrate was concentrated to dryness in vacuo. The resulting residue was further purified by flash column chromatography to afford the desired products.

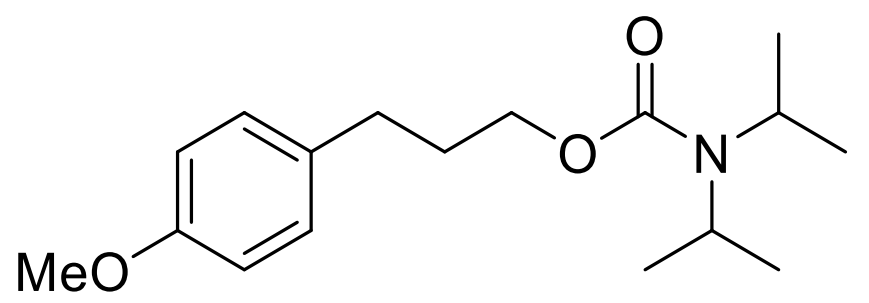

1d

1d: Yield: 1.4 g, 96%, colorless oil, R_f = 0.4 (*n*-hexane/ethyl acetate = 5/1).

^{1}H NMR (400 MHz, $CDCl_3$) δ 7.14 – 7.07 (m, 2H), 6.86 – 6.80 (m, 2H), 4.38 – 3.67 (m, 7H), 2.70 – 2.61 (m, 2H), 2.02 – 1.88 (m, 2H), 1.31 – 0.86 (m, 12H).

The NMR data matched with those reported in literature.[5]

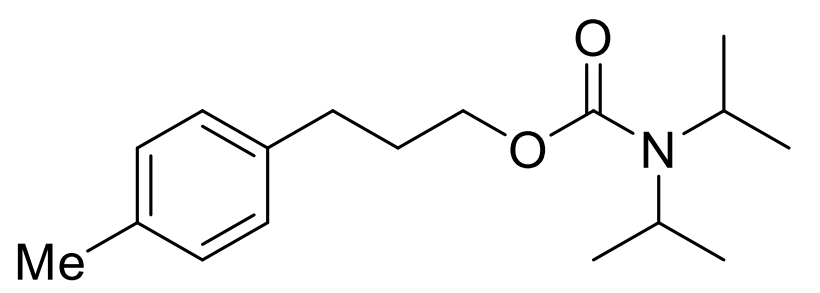

1e

1e: Yield: 1.2 g, 87%, colorless oil, R_f = 0.4 (*n*-hexane/ethyl acetate = 5/1).

^{1}H NMR (400 MHz, $CDCl_3$) δ 7.14 – 7.01 (m, 4H), 4.39 – 3.67 (m, 4H), 2.91 – 2.62 (m, 2H), 2.32 (s, 3H), 2.04 – 1.82 (m, 2H), 1.27 – 1.07 (m, 12H).

^{13}C NMR (101 MHz, $CDCl_3$) δ 155.9, 138.6, 135.4, 129.2, 128.4, 64.2, 45.9 (br), 32.2, 31.1, 21.9 (br).

HRMS (ESI): Calcd for $C_{17}H_{27}NO_2Na^+$ $[M+Na]^+$: 300.1934, found: 300.1942.

IR (neat, cm^{-1}): ν 3052, 2969, 1686, 1308, 1156.

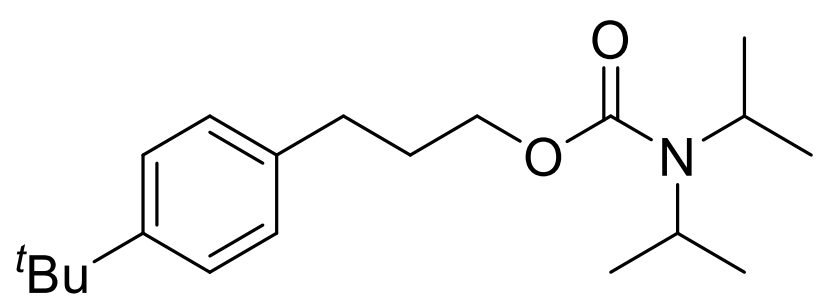

1f

1f: Yield: 1.5 g, 94%, colorless oil, R_f = 0.4 (*n*-hexane/ethyl acetate = 5/1).

^{1}H NMR (400 MHz, $CDCl_3$) δ 7.36 – 7.28 (m, 2H), 7.23 – 7.10 (m, 2H), 4.49 – 3.61 (m, 4H), 2.82 – 2.48 (m, 2H), 2.13 – 1.89 (m, 2H), 1.31 (s, 9H), 1.29 – 1.18 (m, 12H).

^{13}C NMR (101 MHz, $CDCl_3$) δ 156.0, 148.8, 138.6, 128.2, 125.4, 64.3, 45.8, 34.5, 32.1, 31.5, 30.9, 21.3 (br).
HRMS (ESI): Calcd for $C_{20}H_{33}NO_2Na^+$ $[M+Na]^+$: 342.2404, found: 342.2411.
IR (neat, cm^{-1}): *v* 2964, 2870, 1687, 1289, 1264, 1020.

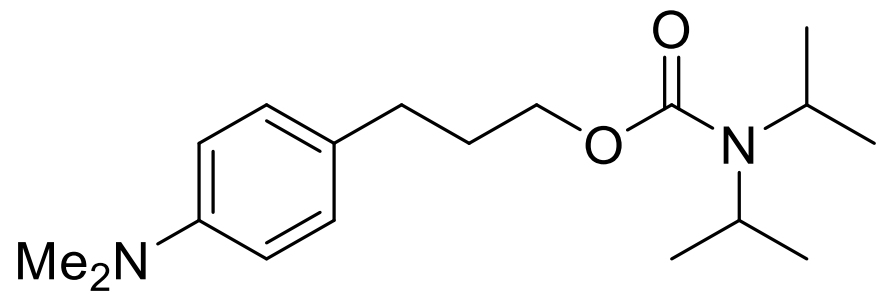

1g

1g: Yield: 1.3 g, 85%, yellow solid, m.p. = 53 – 57 °C, R_f = 0.4 (*n*-hexane/ethyl acetate = 2/1).
^{1}H NMR (600 MHz, $CDCl_3$) δ 7.10 – 7.01 (m, 2H), 6.98 – 6.55 (m, 2H), 4.23 – 3.66 (m, 4H), 2.91 (s, 6H), 2.79 – 2.44 (m, 2H), 2.12 – 1.80 (m, 2H), 1.32 – 0.91 (m, 12H).
^{13}C NMR (151 MHz, $CDCl_3$) δ 156.1, 149.3, 129.9, 129.1, 113.2, 64.3, 45.9 (br), 41.1, 31.6, 31.3, 21.0 (br).
HRMS (ESI): Calcd for $C_{18}H_{30}N_2O_2Na^+$ $[M+Na]^+$: 329.2200, found: 329.2201.
IR (neat, cm^{-1}): *v* 2965, 2931, 1689, 1522, 1438, 1310, 1290, 1134.

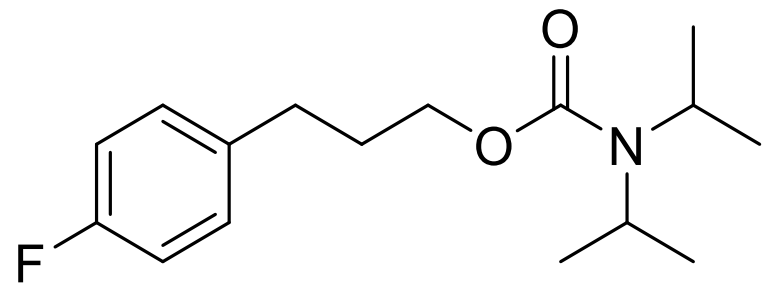

1h

1h: Yield: 1.1 g, 78%, colorless oil, R_f = 0.4 (*n*-hexane/ethyl acetate = 5/1).
^{1}H NMR (400 MHz, $CDCl_3$) δ 7.20 – 7.06 (m, 2H), 7.05 – 6.84 (m, 2H), 4.19 – 3.63 (m, 4H), 2.88 – 2.58 (m, 2H), 2.14 – 1.89 (m, 2H), 1.33 – 0.85 (m, 12H).
^{13}C NMR (101 MHz, $CDCl_3$) δ 161.4 (d, $J_{C\text{-}F}$ = 243.6 Hz), 155.9, 137.3 (d, $J_{C\text{-}F}$ = 3.3 Hz), 129.9, 129.8, 115.3 (d, $J_{C\text{-}F}$ = 21.1 Hz), 64.0, 45.8 (br), 31.9, 31.1, 21.2 (br).
^{19}F NMR (377 MHz, $CDCl_3$) δ -109.9 – -136.5 (m, 1F).
HRMS (ESI): Calcd for $C_{16}H_{24}FNO_2Na^+$ $[M+Na]^+$: 304.1684, found: 304.1685.
IR (neat, cm^{-1}): *v* 2969, 2934, 1688, 1542, 1478, 1191, 1016.

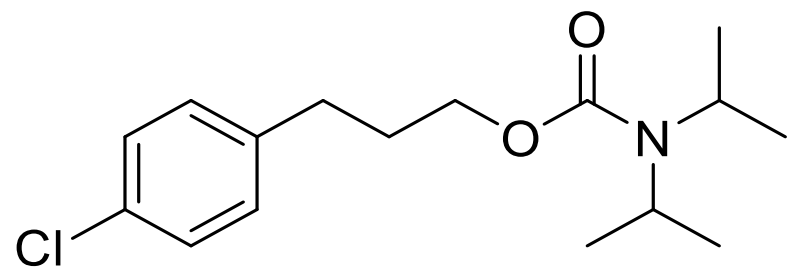

1i

1i: Yield: 1.4 g, 92%, colorless oil, R_f = 0.4 (*n*-hexane/ethyl acetate = 5/1).
^{1}H NMR (400 MHz, $CDCl_3$) δ 7.31 – 7.24 (m, 2H), 7.18 – 7.12 (m, 2H), 4.13 (t, *J* = 6.5 Hz, 2H), 3.81 – 3.76 (m, 2H), 2.75 – 2.67 (m, 2H), 2.04 – 1.92 (m, 2H), 1.45 – 1.02 (m, 12H).
^{13}C NMR (101 MHz, $CDCl_3$) δ 155.8, 140.1, 131.7, 129.8, 128.6, 63.9, 45.9 (br), 32.0, 30.9, 21.2 (br).
HRMS (ESI): Calcd for $C_{16}H_{24}ClNO_2Na^+$ $[M+Na]^+$: 320.1388, found: 320.1390.
IR (neat, cm^{-1}): *v* 2968, 2936, 1685, 1463, 1265, 1244, 1133, 1037.

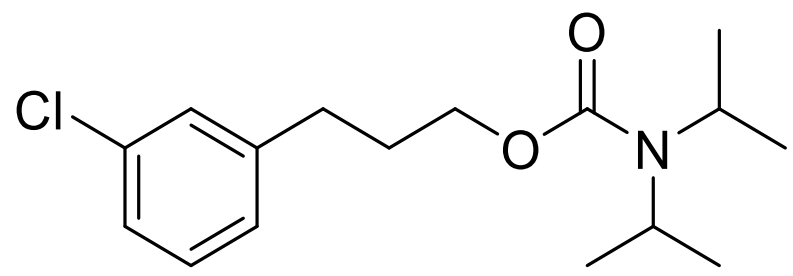

1j

1j: Yield: 1.3 g, 88%, colorless oil, R_f = 0.4 (*n*-hexane/ethyl acetate = 5/1).
^{1}H NMR (400 MHz, $CDCl_3$) δ 7.24 – 7.15 (m, 3H), 7.13 – 7.01 (m, 1H), 4.19 – 3.64 (m, 4H), 2.78 – 2.59 (m, 2H), 2.02 – 1.88 (m, 2H), 1.33 – 0.97 (m, 12H).
^{13}C NMR (101 MHz, $CDCl_3$) δ 155.9, 143.7, 134.3, 129.8, 128.7, 126.8, 126.3, 64.0, 45.8 (br), 32.4, 30.8, 21.2 (br).
HRMS (ESI): Calcd for $C_{16}H_{24}ClNO_2Na^+$ $[M+Na]^+$: 320.1388, found: 320.1391.
IR (neat, cm^{-1}): *v* 2968, 1685, 1433, 1308, 1289, 1133, 1048.

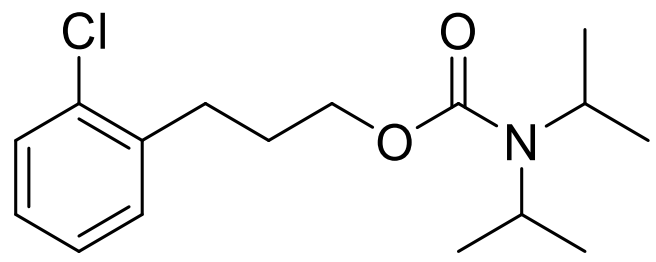

1k

1k: Yield: 1.2 g, 81%, colorless oil, R_f = 0.4 (*n*-hexane/ethyl acetate = 5/1).
^{1}H NMR (400 MHz, $CDCl_3$) δ 7.38 – 7.29 (m, 1H), 7.24 – 7.10 (m, 3H), 4.30 – 3.70 (m, 4H), 3.10 – 2.53 (m, 2H), 2.01 – 1.90 (m, 2H), 1.32 – 1.06 (m, 12H).
^{13}C NMR (101 MHz, $CDCl_3$) δ 155.9, 139.3, 134.0, 130.5, 129.6, 127.6, 126.9, 64.1, 45.9 (br), 30.6, 29.3, 21.2 (br).
HRMS (ESI): Calcd for $C_{16}H_{24}ClNO_2Na^+$ $[M+Na]^+$: 320.1388, found: 320.1393.
IR (neat, cm^{-1}): *v* 2968, 2934, 1685, 1474, 1434, 1307, 1288, 1133.

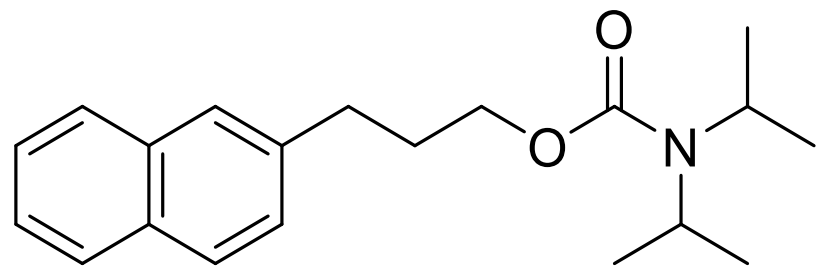

1m

1m: Yield: 1.4 g, 89%, white solid, m.p. = 71 – 73 °C, R_f = 0.4 (*n*-hexane/ethyl acetate = 5/1).
^{1}H NMR (600 MHz, $CDCl_3$) δ 7.88 – 7.75 (m, 3H), 7.71 – 7.59 (m, 1H), 7.54 – 7.41 (m, 2H), 7.39 – 7.32 (m, 1H), 4.51 – 3.60 (m, 4H), 3.17 – 2.76 (m, 2H), 2.28 – 1.80 (m, 2H), 1.46 – 0.88 (m, 12H).
^{13}C NMR (151 MHz, $CDCl_3$) δ 155.9, 139.2, 133.8, 132.2, 128.1, 127.7, 127.6, 127.4, 126.6, 126.1, 125.3, 64.2, 32.9, 30.9, 21.0 (br).
HRMS (ESI): Calcd for $C_{20}H_{27}NO_2Na^+$ $[M+Na]^+$: 336.1934, found: 336.1938.
IR (neat, cm^{-1}): *v* 3053, 2968, 1687, 1435, 1309, 1289, 1133, 1060.

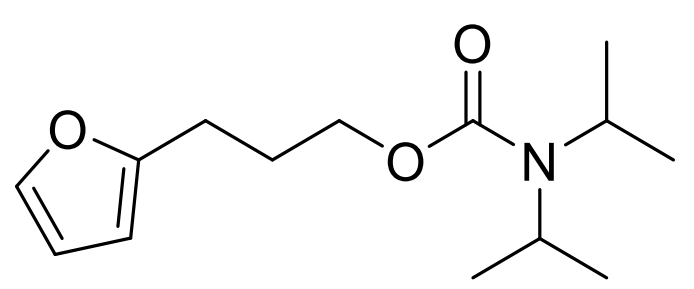

1p

1p: Yield: 1.1 g, 87%, yellow oil, R_f = 0.4 (*n*-hexane/ethyl acetate = 5/1).

^{1}H NMR (400 MHz, $CDCl_3$) δ 7.31 – 7.27 (m, 1H), 6.64 – 6.19 (m, 1H), 6.14 – 5.88 (m, 1H), 4.36 – 3.73 (m, 4H), 2.72 (t, J = 7.6 Hz, 2H), 2.36 – 1.92 (m, 2H), 1.42 – 0.90 (m, 12H).
^{13}C NMR (101 MHz, $CDCl_3$) δ 155.8, 155.3, 141.1, 110.2, 105.2, 63.9, 45.8 (br), 27.7, 25.0, 21.2 (br).
HRMS (ESI): Calcd for $C_{14}H_{23}NO_3Na^+$ $[M+Na]^+$: 276.1570, found: 276.1574.
IR (neat, cm^{-1}): v 2968, 1686, 1435, 1308, 1288, 1146, 1060.

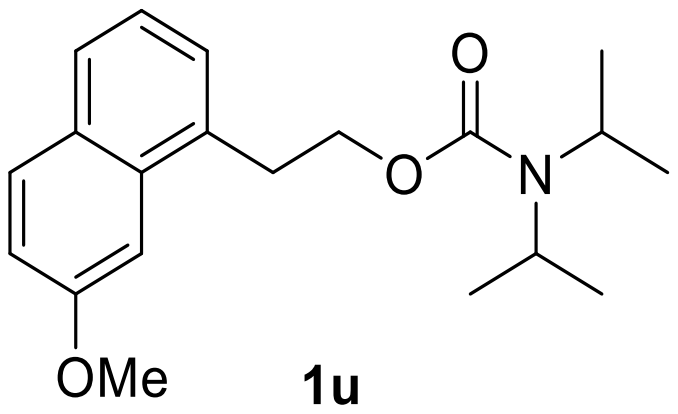

1u

1u: Yield: 740.3 mg, 45%, yellow oil, R_f = 0.4 (n-hexane/ethyl acetate = 3/1).
^{1}H NMR (400 MHz, $CDCl_3$) δ 7.85 – 7.72 (m, 1H), 7.72 – 7.64 (m, 1H), 7.52 – 7.41 (m, 1H), 7.41 – 7.32 (m, 1H), 7.28 – 7.22 (m, 1H), 7.19 – 7.13 (m, 1H), 4.44 (t, J = 7.2 Hz, 2H), 4.15 – 3.64 (m, 5H), 3.39 (t, J = 7.2 Hz, 2H), 1.23 – 1.05 (m, 12H).
^{13}C NMR (101 MHz, $CDCl_3$) δ 158.0, 155.7, 133.4, 133.0, 130.3, 129.4, 127.6, 127.0, 123.2, 118.1, 102.8, 64.4, 55.5, 45.6 (br), 33.1, 21.2 (br).
HRMS (ESI): Calcd for $C_{20}H_{27}NO_3Na^+$ $[M+Na]^+$: 352.1883, found: 352.1888.
IR (neat, cm^{-1}): v 3055, 2967, 1683, 1472, 1293, 1254, 1213, 1167.

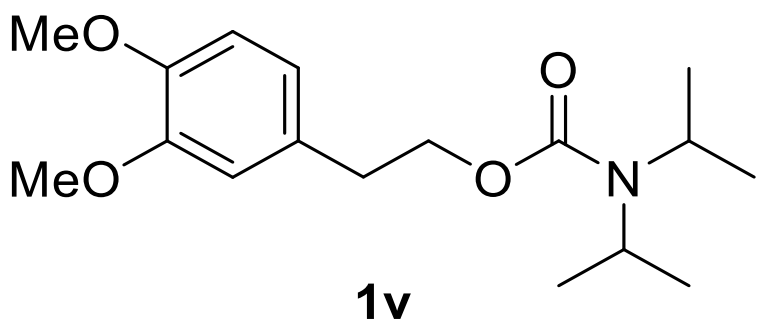

1v

1v: Yield: 571.7 mg, 37%, orange oil, R_f = 0.4 (n-hexane/ethyl acetate = 3/1).
^{1}H NMR (400 MHz, $CDCl_3$) δ 7.10 – 6.30 (m, 3H), 4.25 (t, J = 7.1 Hz, 2H), 4.07 – 3.53 (m, 8H), 2.87 (t, J = 7.1 Hz, 2H), 1.32 – 0.91 (m, 12H).
^{13}C NMR (101 MHz, $CDCl_3$) δ 155.6, 148.8, 147.5, 130.9, 120.9, 112.1, 111.2, 65.3, 55.9, 55.7, 35.2, 20.6 (br).
HRMS (ESI): Calcd for $C_{17}H_{27}NO_4Na^+$ $[M+Na]^+$: 332.1832, found: 332.1836.
IR (neat, cm^{-1}): v 2966, 2935, 1683, 1516, 1368, 1236, 1156, 1029.

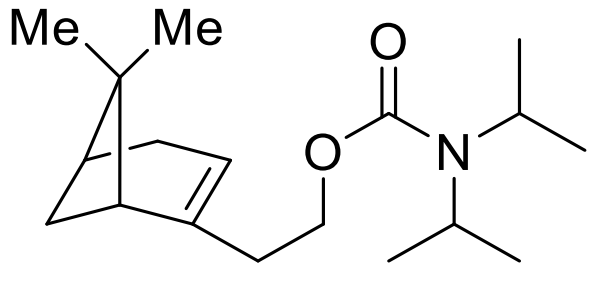

1ad

1ad: Yield: 1.4 g, 96%, colorless oil, R_f = 0.4 (n-hexane/ethyl acetate = 5/1).
^{1}H NMR (400 MHz, $CDCl_3$) δ 5.37 – 5.00 (m, 1H), 4.32 – 3.32 (m, 4H), 2.38 – 2.14 (m, 5H), 2.08 – 2.01 (m, 2H), 1.24 (s, 3H), 1.19 – 1.13 (m, 13H), 0.81 (s, 3H).
^{13}C NMR (101 MHz, $CDCl_3$) δ 155.7, 144.6, 118.5, 62.8, 45.7 (br), 40.8, 38.1, 36.7, 31.7, 31.5, 26.4, 21.2 (br).

HRMS (ESI): Calcd for $C_{18}H_{31}NO_2Na^+$ $[M+Na]^+$: 316.2247, found: 316.2250.
IR (neat, cm^{-1}): *v* 2967, 2879, 1687, 1488, 1347, 1190, 1140.

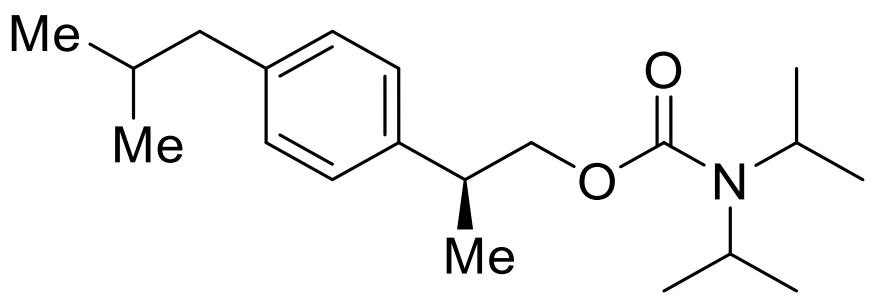

1ae

1ae: Yield: 1.4 g, 88%, colorless oil, R_f = 0.4 (*n*-hexane/ethyl acetate = 5/1).
^{1}H NMR (400 MHz, $CDCl_3$) δ 7.11 (d, *J* = 8.1 Hz, 2H), 7.04 (d, *J* = 8.1 Hz, 2H), 4.22 – 4.04 (m, 2H), 4.04 – 3.56 (m, 2H), 3.30 – 2.98 (m, 1H), 2.41 (d, *J* = 7.2 Hz, 2H), 1.89 – 1.72 (m, 1H), 1.27 (d, *J* = 7.0 Hz, 3H), 1.14 – 0.95 (m, 12H), 0.86 (d, *J* = 6.7 Hz, 6H).
^{13}C NMR (101 MHz, $CDCl_3$) δ 155.4, 140.8, 139.5, 128.9, 126.9, 69.8, 44.9, 38.8, 30.1, 22.2, 22.2, 20.9 (br), 18.2.
HRMS (ESI): Calcd for $C_{20}H_{33}NO_2Na^+$ $[M+Na]^+$: 342.2404, found: 342.2412.
IR (neat, cm^{-1}): *v* 2962, 1688, 1309, 1290, 1133, 1058.

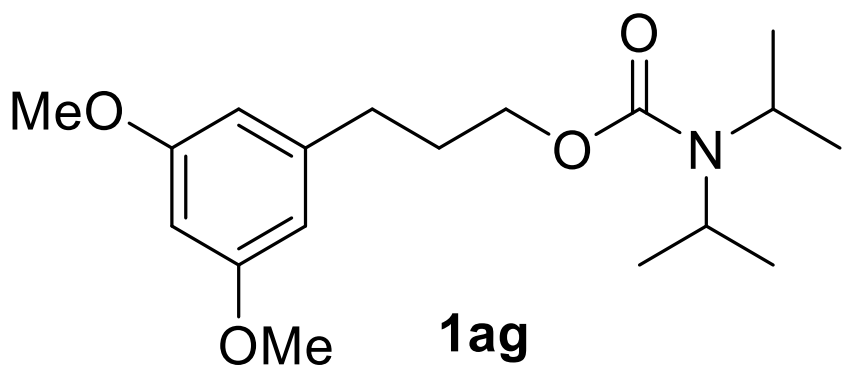

1ag

1ag: Yield: 1.5 g, 95%, colorless oil, R_f = 0.4 (*n*-hexane/ethyl acetate = 5/1).
^{1}H NMR (400 MHz, $CDCl_3$) δ 7.10 – 6.30 (m, 3H), 4.62 – 4.22 (m, 2H), 4.07 – 3.53 (m, 8H), 2.87 (t, *J* = 7.1 Hz, 2H), 1.21 – 0.88 (m, 12H), 0.85 – 0.75 (m, 1H).
^{13}C NMR (101 MHz, $CDCl_3$) δ 155.6, 148.8, 147.5, 130.9, 120.9, 112.1, 111.2, 65.3, 55.9, 55.7, 35.2, 20.8.
HRMS (ESI): Calcd for $C_{18}H_{29}NO_4Na^+$ $[M+Na]^+$: 346.1989, found: 346.1999.
IR (neat, cm^{-1}): *v* 2968, 1689, 1310, 1291.

3.3. Preparation of compound 1n.

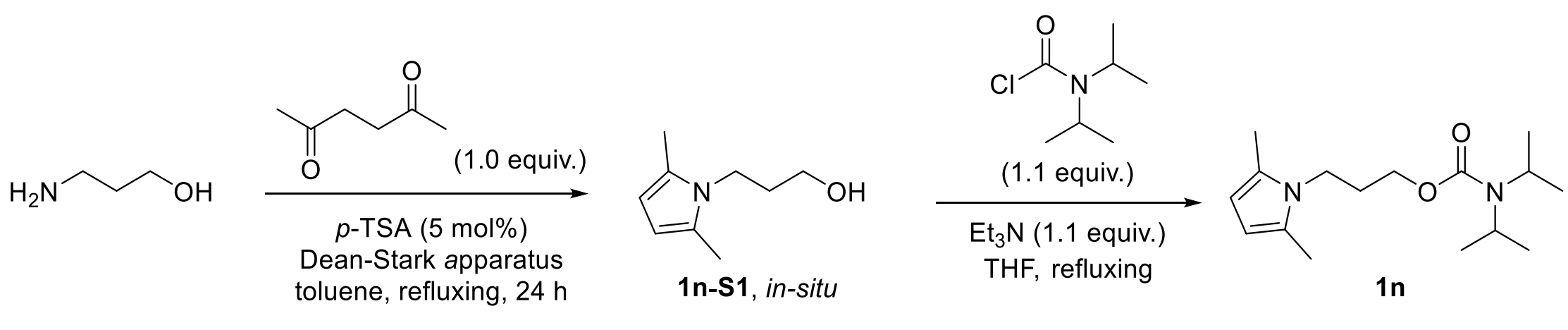

According to a reported literature,[7] to an oven dried 100 mL round-bottom flask charged with 3-aminopropanol (1.13 g, 15.0 mmol, 1.0 equiv.), 2,5-hexanedione (1.71 g, 15.0 mmol, 1.0 equiv.) and toluene (30 mL) was added *p*-toluenesulfonic acid monohydrate (143 mg, 0.75 mmol, 0.05 equiv.). The reaction mixture was then heated to reflux for 24 h using a Dean-Stark apparatus. The resulting mixture was then cooled to room temperature and washed with H_2O. The aqueous layer was extracted with DCM (3 × 20 mL) and the organic layer was combined, dried over Na_2SO_4 and filtered through a pad of Celite. The filtrate was concentrated to dryness in vacuo to give the **1n-S1**, which was used for next step without further purification.

According to a reported literature,[4] to a Schlenk tube charged with *N,N*-diisopropylcarbamoyl chloride (897 mg, 5.5 mmol, 1.1 equiv.), Et_3N (556 mg, 5.5 mmol, 1.1 equiv.) and THF (10 mL) was added **1n-S1** from the last step (765 mg, 5.0 mmol, 1.0 equiv.). The reaction mixture was then heated to reflux and stirred for 24 h. Upon completion, the resulting mixture was filtered through a pad of Celite and washed with ethyl acetate. The filtrate was concentrated to dryness in vacuo. The resulting residue was further purified by flash column chromatography to afford the desired product **1n** (86%, 1.2 g).

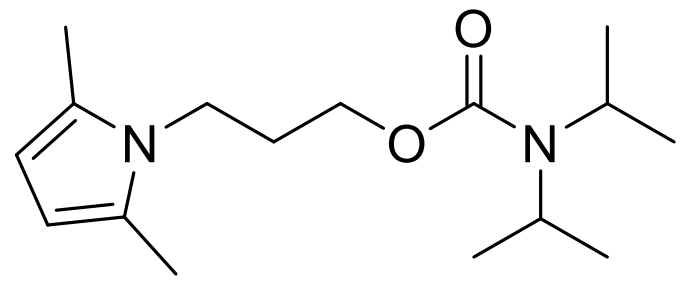

1n

1n: Yield: 1.2 g, 86%, orange oil, $R_f = 0.4$ (*n*-hexane/ethyl acetate = 5/1).
^{1}H NMR (400 MHz, $CDCl_3$) δ 5.77 (s, 2H), 4.20 – 3.72 (m, 6H), 2.22 (s, 6H), 2.01 – 1.90 (m, 2H), 1.35 – 1.06 (m, 12H).
^{13}C NMR (101 MHz, $CDCl_3$) δ 155.5, 127.3, 105.4, 62.0, 46.3 (br), 40.9, 30.7, 21.4 (br), 12.5.
HRMS (ESI): Calcd for $C_{16}H_{29}N_2O_2^+$ $[M+H]^+$: 281.2224, found: 281.2223.
IR (neat, cm^{-1}): *ν* 2968, 2933, 1683, 1435, 1368, 1289, 1155, 1064.

3.4. Preparation of compound 1o.

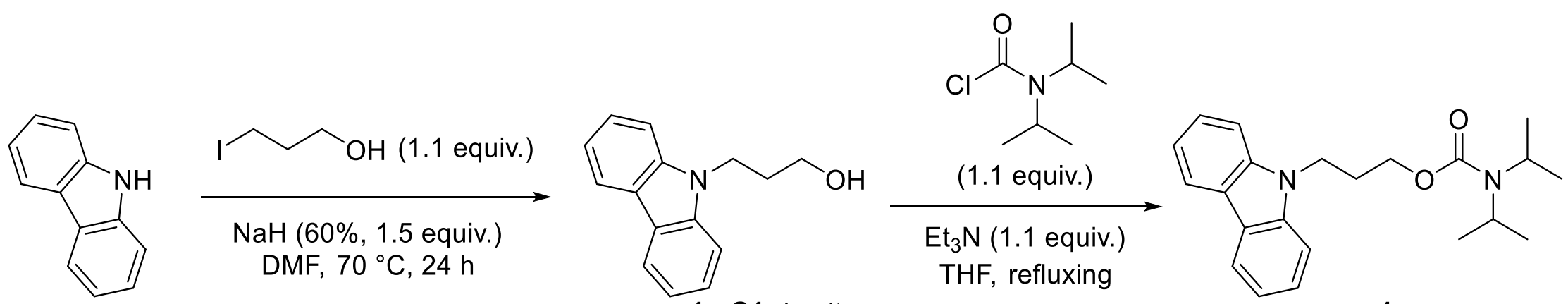

According to a reported literature,[8] to an oven dried 100 mL round-bottom flask charged with carbazole (836 mg, 5.0 mmol, 1.0 equiv.) and NaH (60% dispersed in mineral oil, 300 mg, 7.5 mmol, 1.5 equiv.) in DMF (30 mL) was added 3-iodopropan-1-ol (1.02 g, 5.5 mmol, 1.1 equiv.). The reaction mixture was then heated at 70 °C for 24 h. The resulting mixture was then cooled to room temperature and washed with H_2O. The aqueous layer was extracted with DCM (3 × 20 mL) and the organic layer was combined, dried over Na_2SO_4 and filtered through a pad of Celite. The filtrate was concentrated to dryness in vacuo to give the **1o-S1**, which was used for next step without further purification.
According to a reported literature,[4] to a Schlenk tube charged with *N,N*-diisopropylcarbamoyl chloride (897 mg, 5.5 mmol, 1.1 equiv.), Et_3N (5.5 mmol, 1.1 equiv.) and THF (10 mL) was added **1o-S1** from the last step (*in-situ*, 5.0 mmol, 1.0 equiv.). The reaction mixture was then heated to reflux and stirred for 24 h. Upon completion, the resulting mixture was filtered thought a pad of Celite and washed with ethyl acetate. The filtrate was concentrated to dryness in vacuo. The resulting residue

was further purified by flash column chromatography to afford the desired product **1o** (46%, 809.6 mg).

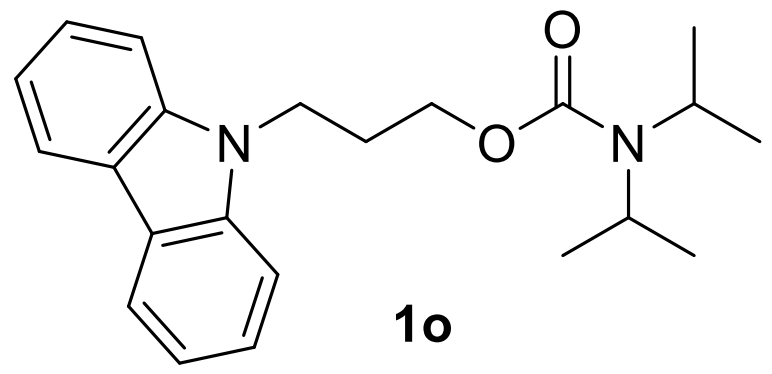

1o

1o: Yield: 809.6 mg, 46%, yellow oil, R_f = 0.4 (*n*-hexane/ethyl acetate = 3/1).
^{1}H NMR (400 MHz, $CDCl_3$) δ 8.18 – 8.01 (m, 2H), 7.53 – 7.33 (m, 4H), 7.25 – 7.17 (m, 2H), 4.44 (t, J = 7.2 Hz, 2H), 4.17 (t, J = 6.1 Hz, 2H), 4.11 – 3.87 (m, 2H), 2.33 – 2.19 (m, 2H), 1.44 – 1.16 (m, 12H).
^{13}C NMR (101 MHz, $CDCl_3$) δ 155.2, 140.2, 125.7, 122.9, 120.4, 118.9, 108.4, 62.1, 45.9 (br), 40.0, 28.6, 21.3 (br).
HRMS (ESI): Calcd for $C_{22}H_{28}N_2O_2Na^+$ $[M+Na]^+$: 375.2043, found: 375.2047.
IR (neat, cm^{-1}): v 2967, 1685, 1484, 1288, 1153, 1067.

3.5. Preparation of compound 1s.

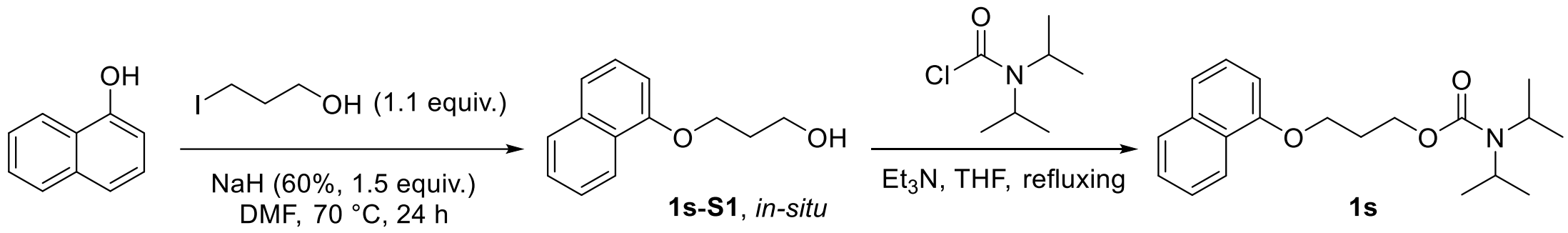

According to a reported literature,[9] to an oven dried 100 mL round-bottom flask charged with naphthalen-1-ol (721 mg, 5.0 mmol, 1.0 equiv.) and NaH (60% dispersed in mineral oil, 300 mg, 7.5 mmol, 1.5 equiv.) in DMF (30 mL) was added 3-iodopropan-1-ol (1.02 g, 5.5 mmol, 1.1 equiv.). The reaction mixture was then heated at 70 °C for 24 h. The resulting mixture was then cooled to room temperature and washed with H_2O. The aqueous layer was extracted with DCM (3 × 20 mL) and the organic layer was combined, dried over Na_2SO_4 and filtered through a pad of Celite. The filtrate was concentrated to dryness in vacuo to give the **1s-S1**, which was used for next step without further purification.
According to a reported literature,[4] to a Schlenk tube charged with *N,N*-diisopropylcarbamoyl chloride (897 mg, 5.5 mmol, 1.1 equiv.), Et_3N (556 mg, 5.5 mmol, 1.1 equiv.) and THF (10 mL) was added **1s-S1** (*in-situ*, 5.0 mmol, 1.0 equiv.). The reaction mixture was then heated to reflux and stirred for 24 h. Upon completion, the resulting mixture was filtered thought a pad of Celite and washed with ethyl acetate. The filtrate was concentrated to dryness in vacuo. The resulting residue was further purified by flash column chromatography to afford the desired product **1s** (73%, 1.2 g).

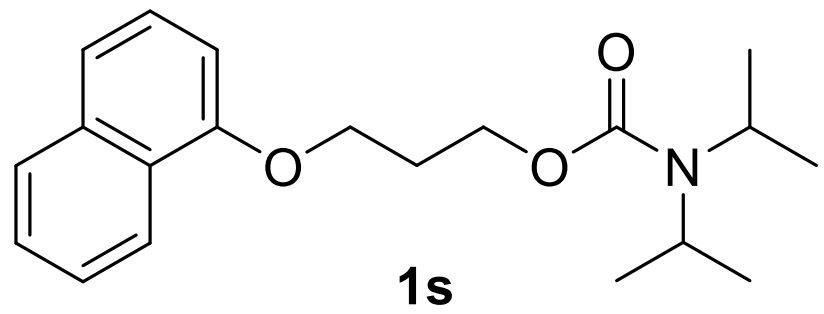

1s

1s: Yield: 1.2 g, 73%, white solid, m.p. = 69 – 73 °C, R_f = 0.4 (*n*-hexane/ethyl acetate = 5/1).

^{1}H NMR (400 MHz, $CDCl_3$) δ 8.42 – 8.19 (m, 1H), 8.00 – 7.75 (m, 1H), 7.59 – 7.34 (m, 4H), 7.05 – 6.73 (m, 1H), 4.42 (t, *J* = 6.3 Hz, 2H), 4.25 (t, *J* = 6.2 Hz, 2H), 4.11 – 3.69 (m, 2H), 2.60 – 2.24 (m, 2H), 1.48 – 0.81 (m, 12H).
^{13}C NMR (101 MHz, $CDCl_3$) δ 155.6, 154.6, 134.5, 127.4, 126.4, 125.8, 125.6, 125.2, 122.1, 120.2, 104.5, 64.9, 63.7, 61.7, 46.2 (br), 29.2, 21.0.
HRMS (ESI): Calcd for $C_{20}H_{27}NO_3Na^+$ $[M+Na]^+$: 352.1883, found: 352.1889.
IR (neat, cm^{-1}): *v* 2920, 2851, 1691, 1437, 1292, 1269, 1108, 1072.

3.6. Preparation of compounds 1ai-1al.

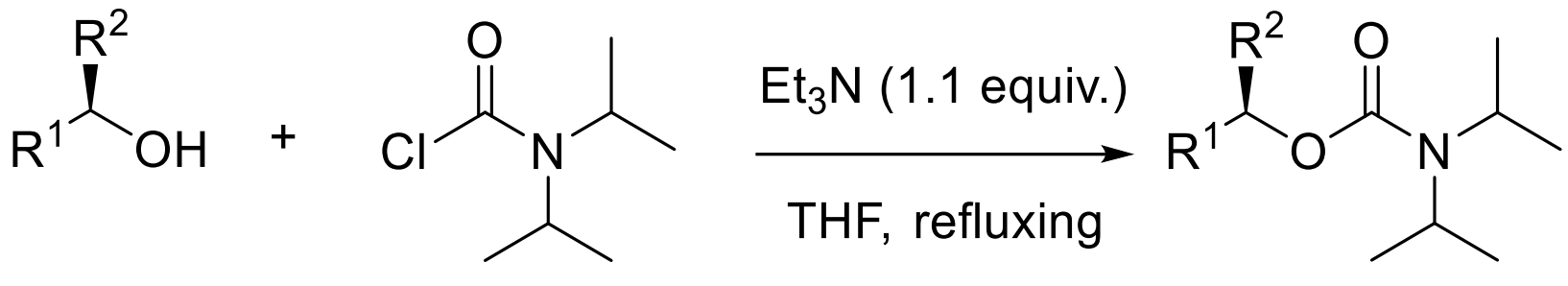

According to a reported literature,[10] to a Schlenk tube charged with *N,N*-diisopropylcarbamoyl chloride (897 mg, 5.5 mmol, 1.1 equiv.), Et_3N (556 mg, 5.5 mmol, 1.1 equiv.) and THF (10 mL) was added chiral secondary alcohol derivatives (5.0 mmol, 1.0 equiv.). The reaction mixture was then heated to reflux and stirred for 24 h. Upon completion, the resulting mixture was filtered thought a pad of Celite and washed with ethyl acetate. The filtrate was concentrated to dryness in vacuo. The resulting residue was further purified by flash column chromatography to afford the desired products.
Note: Chiral secondary alcohol substrates (>99% enantiopurity) were purchased from Bidepharm and used as received.

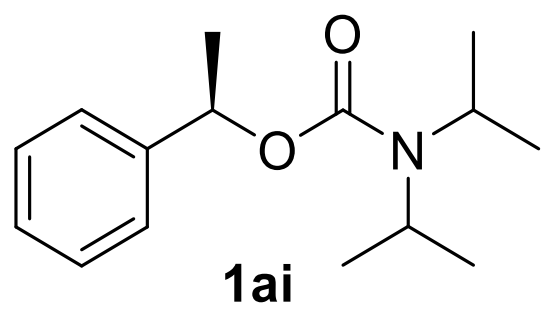

1ai

1ai: Yield: 1.1 g, 85%, colorless oil, R_f = 0.4 (*n*-hexane/ethyl acetate = 5/1).
^{1}H NMR (400 MHz, $CDCl_3$) δ 7.39 – 7.31 (m, 4H), 7.29 – 7.26 (m, 1H), 5.84 (q, *J* = 6.6 Hz, 1H), 4.21 – 3.72 (m, 2H), 1.54 (d, *J* = 6.6 Hz, 3H), 1.43 – 0.94 (m, 12H).
The NMR data matched with those reported in literature.[10]

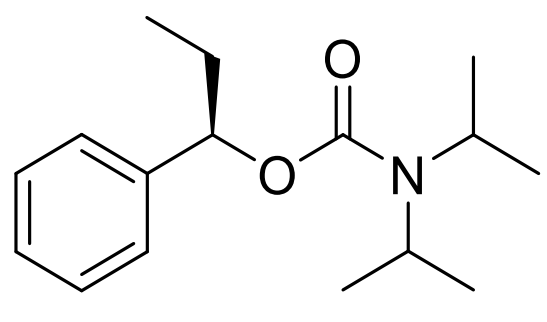

1aj

1aj: Yield: 1.0 g, 76%, colorless oil, R_f = 0.4 (*n*-hexane/ethyl acetate = 5/1).
^{1}H NMR (400 MHz, $CDCl_3$) δ 7.40 – 7.29 (m, 4H), 7.28 – 7.23 (m, 1H), 5.64 (t, *J* = 6.7 Hz, 1H), 4.25 – 3.61 (m, 2H), 2.10 – 1.73 (m, 2H), 1.35 – 1.13 (m, 12H), 0.89 (t, *J* = 7.4 Hz, 3H).
The NMR data matched with those reported in literature.[10]

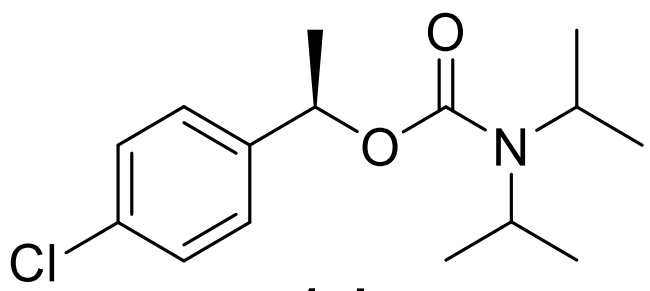

1ak

1ak: Yield: 1.3 g, 90%, colorless oil, $R_f = 0.4$ (*n*-hexane/ethyl acetate = 5/1).
^{1}H NMR (400 MHz, $CDCl_3$) δ 7.34 – 7.26 (m, 4H), 5.79 (q, $J = 6.6$ Hz, 1H), 4.15 – 3.63 (m, 2H), 1.52 (d, $J = 6.6$ Hz, 3H), 1.40 – 0.86 (m, 12H).
The NMR data matched with those reported in literature.[11]

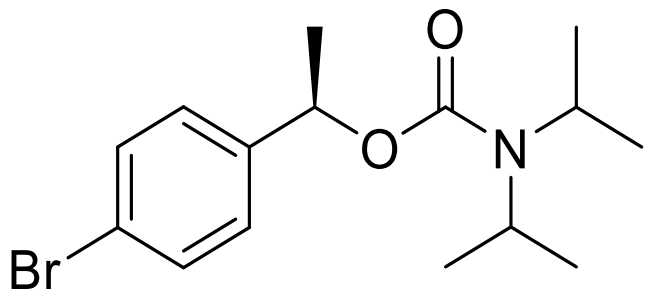

1al

1al: Yield: 1.5 g, 91%, colorless oil, $R_f = 0.4$ (*n*-hexane/ethyl acetate = 5/1).
^{1}H NMR (400 MHz, $CDCl_3$) δ 7.46 (d, $J = 8.4$ Hz, 2H), 7.25 – 7.14 (m, 2H), 5.78 (q, $J = 6.6$ Hz, 1H), 4.32 – 3.63 (m, 2H), 1.52 (d, $J = 6.6$ Hz, 3H), 1.37 – 1.06 (m, 12H).
The NMR data matched with those reported in literature.[12]

4. Control experiments.

4.1. Table S1. Screening of carbenoid precursors.

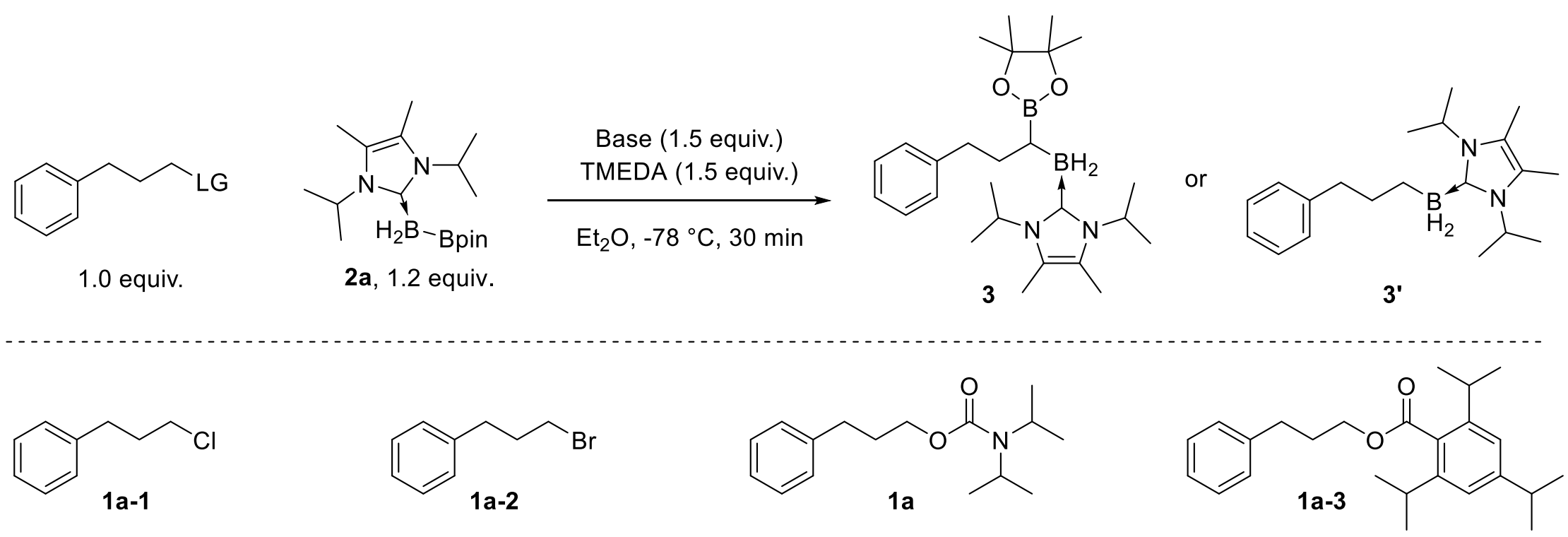

Entry	Carbenoid precursor (LG = leaving group)	Base	Yield of **3**/%	Yield of **3'**/%
1	**1a-1**, LG = Cl	^{n}BuLi	nd	51
2	**1a-2**, LG = Br	^{n}BuLi	nd	26
3	**1a**, LG = OCb	^{n}BuLi	nd	nd
4	**1a**, LG = OCb	^{s}BuLi	86	nd
5	**1a-3**, LG = OTIB	^{s}BuLi	65	nd

Reaction procedures for entries 1-3.

Under argon, to an oven dried 4 mL reaction tube equipped with a magnetic stir bar and a septum was added carbenoid precursor **1a-1** or **1a-2** or **1a** (0.1 mmol, 1.0 equiv.), TMEDA (17.4 mg, 0.15 mmol, 1.5 equiv.), **2a** (38.4 mg, 0.12 mmol, 1.2 equiv.) and Et_2O (1.5 mL). The reaction was cooled to -78 °C and stirred for 10 min. ^{n}BuLi (1.7 M in hexane, 0.09 mL, 0.15 mmol, 1.5 equiv.) was then slowly added and the mixture was stirred vigorously (without splashing) at -78 °C for 30 min. After that, the reaction mixture was warmed to room temperature and stirred for another 30 min. Upon completion, the reaction was quenched with aqueous NH_4Cl and extracted with ethyl acetate (3 × 3 mL). The combined organic phase was dried over $MgSO_4$, and the solvent was removed in vacuo. The resulting residue was purified by flash column chromatography to afford the desired product.

Reaction procedures for entries 4-5.

Under argon, to an oven dried 4 mL reaction tube equipped with a magnetic stir bar and a septum was added **1a** or **1a-3** (0.1 mmol, 1.0 equiv.), TMEDA (17.4 mg, 0.15 mmol, 1.5 equiv.) and Et_2O (500 µL). The reaction was cooled to -78 °C and stirred for 10 min. ^{s}BuLi (1.3 M in cyclohexane, 0.12 mL, 0.15 mmol, 1.5 equiv.) was then slowly added and the mixture was stirred vigorously (without splashing) at -78 °C for 30 min. After that, a solution of **2a** (38.4 mg, 0.12 mmol, 1.2 equiv.) in Et_2O (1.2 mL) was added dropwise. The reaction mixture was stirred at this temperature for an additional 10 min. After that, the reaction mixture was warmed to room temperature and stirred for another 30 min. Upon completion, the reaction was quenched with aqueous NH_4Cl and extracted with ethyl acetate (3 × 3 mL). The combined organic phase was dried over $MgSO_4$, and the solvent was removed in vacuo. The resulting residue was purified by flash column chromatography to afford the desired product.

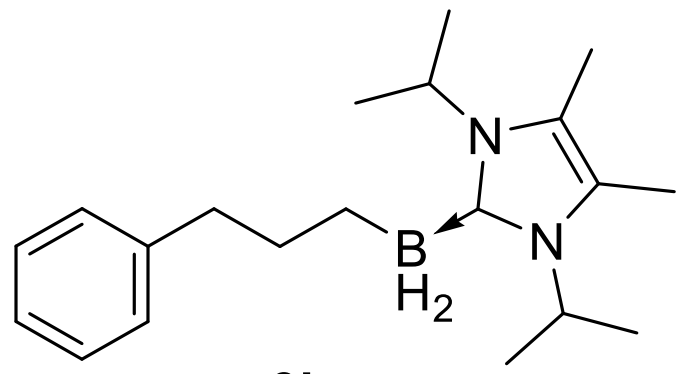

3'

3': Yield: 15.9 mg, 51%, colorless oil, R_f = 0.4 (*n*-hexane/ethyl acetate = 3/1).

^{1}H NMR (400 MHz, $CDCl_3$) δ 7.24 – 7.16 (m, 4H), 7.15 – 6.99 (m, 1H), 5.69 – 5.17 (m, 2H), 2.63 (t, *J* = 7.7 Hz, 2H), 2.19 (s, 6H), 1.62 – 1.51 (m, 1H), 1.48 – 1.19 (m, 12H), 0.91 – 0.73 (m, 1H), 0.59 – 0.24 (m, 2H).

^{13}C NMR (101 MHz, $CDCl_3$) δ 144.8, 128.7, 128.0, 125.0, 123.5, 49.5, 40.0, 34.4, 21.7, 10.5.

^{11}B NMR (128 MHz, $CDCl_3$) δ -26.3 (t, *J* = 82.9 Hz, 1B).

HRMS (ESI): Calcd for $C_{20}H_{33}BN_2Na^+$ $[M+Na]^+$: 335.2629, found: 335.2624.

IR (neat, cm^{-1}): *ν* 2972, 2917, 2847, 2270, 1495, 1453, 1397, 1370, 1321, 1220, 1135, 1111, 1068.

Discussion:

Our results suggest that steric hindrance of the NHC ligand is not the governing factor for stereocontrol. Both L^1 and L^3 afforded the desired products with 98% ee. Only NHC

ligand L^2 possesses acidic protons provided a reduced ee of 89%. The reduced yield and enantioselectivity might be attributed to competitive deprotonation of the backbone $C(sp^2)-H$ on the NHC ligand (R^2 = H) under the strong basic conditions, which leads to unwanted side reactions and compromises stereocontrol.

4.2. Table S2. Condition optimization for the synthesis of **3a**.

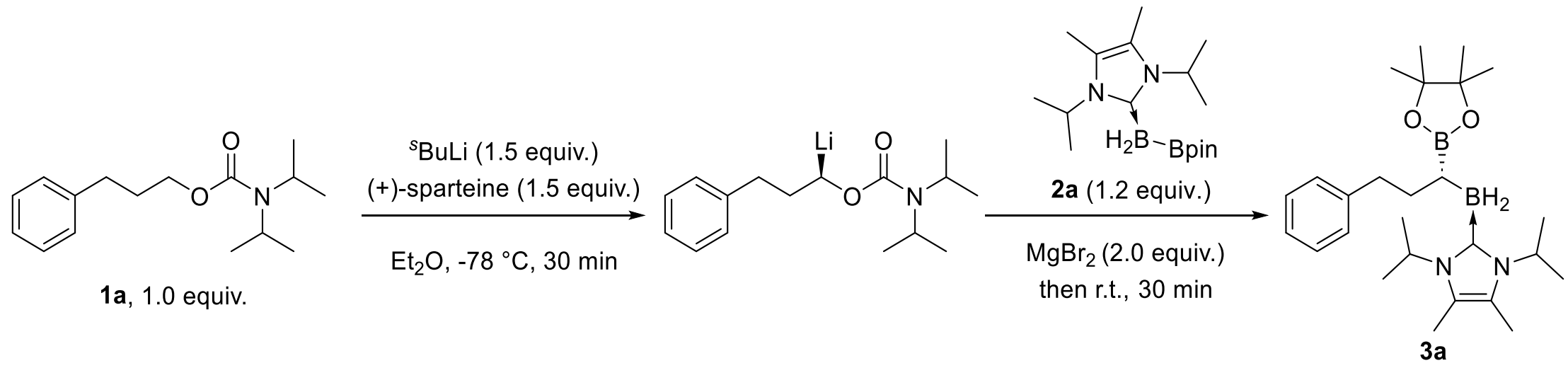

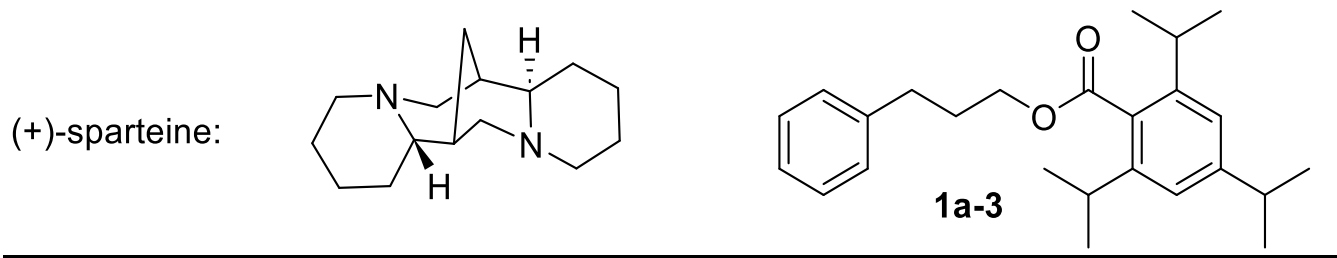

Entry	Variation from the standard reaction condition	**3a**/%	ee/%
1	none	95 (86)	98
2	without ^{s}BuLi	nd	-
3	without (+)-sparteine	trace	-
4	without $MgBr_2$	86	94
5	THF instead of Et_2O	70	13
6	0 min at r.t.[a]	80	98
7	^{n}BuLi instead of ^{s}BuLi	nd	-
8	(+)-sparteine (2.0 equiv.) instead of (+)-sparteine (1.5 equiv.)	55	96
9	^{s}BuLi (2.0 equiv.) instead of ^{s}BuLi (1.5 equiv.)	74	97
10	^{s}BuLi (1.0 equiv.) instead of ^{s}BuLi (1.5 equiv.)	30	95
11	**2a** (2.0 equiv.) instead of **2a** (1.2 equiv.)	99	97
12	-94 °C instead of -78 °C	50	96
13	**1a-3** instead of **1a**	65	93

Standard reaction conditions: **1a** (0.1 mmol, 1.0 equiv.), (+)-sparteine (0.15 mmol, 1.5 equiv.), ^{s}BuLi (0.15 mmol, 1.5 equiv.), Et_2O (0.5 mL), -78 °C, 30 min; then **2a** (38.4 mg in 1.2 mL Et_2O, 0.12 mmol, 1.2 equiv.), -78 °C, 10 min; then $MgBr_2$ (0.5 M in Et_2O, 0.2 mmol, 2.0 equiv.), -78 °C, 20 min, r.t., 30

min. ^{1}H NMR yields were provided using 1,1,2,2-tetrachloroethane as internal standard; isolated yields were provided in parentheses; unless otherwise stated, all enantiomeric excess (ee) were determined by HPLC analysis (Chiracel IB column). [a]H_2O was directly added at -78 °C.

4.3. Screening of diboron reagents 2b-2e.

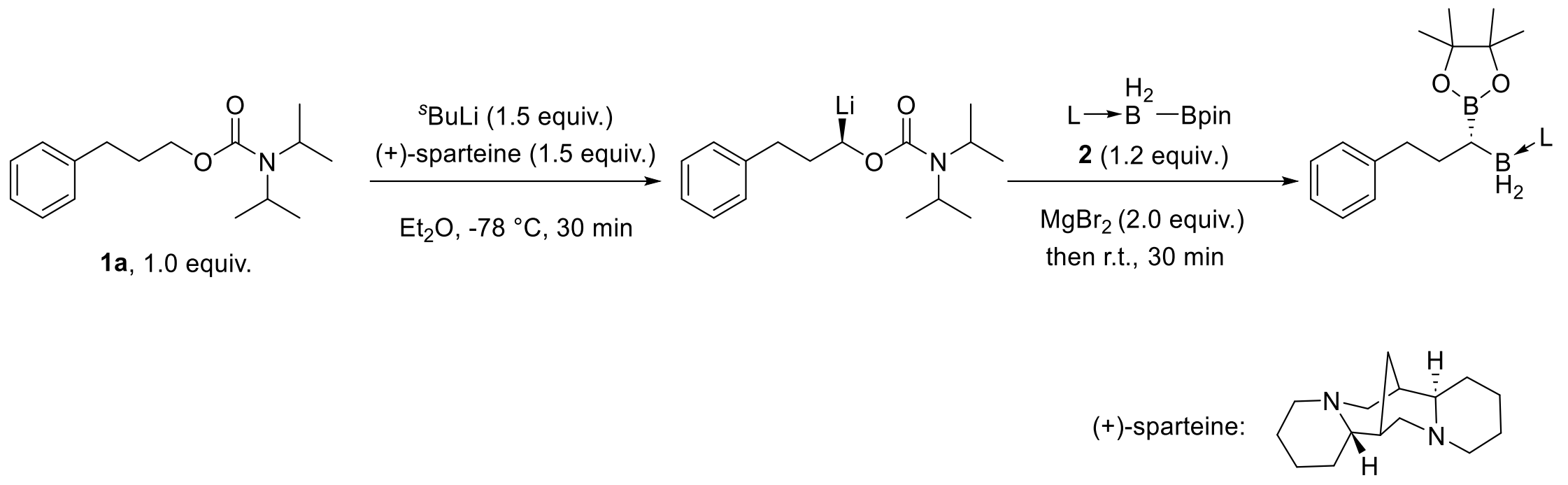

Entry	Diboron reagents **2**	Yield/%	ee/%
1	L[1]: R^1 = ^{i}Pr, R^2 = Me, **2a**	**3a**, 86	98
2	L[2]: R^1 = ^{i}Pr, R^2 = H, **2b**	**3b**, 58	89
3	L[3]: R^1, R^2 = Me, **2c**	**3c**, 52	98
4	L[4] = NEt_3, **2d**	nd	-
5	L[5] = PPh_3, **2e**	nd	-

Under argon, to an oven dried 4 mL reaction tube equipped with a magnetic stir bar and a septum was added carbamate **1a** (26.4 mg, 0.1 mmol, 1.0 equiv.), (+)-sparteine (35.2 mg, 0.15 mmol, 1.5 equiv.) and Et_2O (500 μL). The reaction was cooled to -78 °C and stirred for 10 min. ^{s}BuLi (1.3 M in cyclohexane, 0.12 mL, 0.15 mmol, 1.5 equiv.) was then slowly added and the mixture was stirred vigorously (without splashing) at -78 °C for 30 min. After that, a solution of **2** (0.12 mmol, 1.2 equiv.) in Et_2O (1.2 mL) was added dropwise. The reaction mixture was stirred at this temperature for an additional 10 min. $MgBr_2$ (freshly made from Mg and 1,2-dibromoethane, 0.5 M in Et_2O, 0.4 mL, 0.2 mmol, 2.0 equiv.) was then added dropwise and the reaction mixture was stirred at -78 °C for 20 min. After that, the reaction mixture was warmed to room temperature and stirred for another 30 min. Upon completion, the reaction was quenched with aqueous NH_4Cl and extracted with ethyl acetate (3 × 3 mL). The combined organic phase was dried over $MgSO_4$, and the solvent was removed in vacuo. The resulting residue was purified by flash column chromatography to afford the desired product. The racemic product was prepared using the same procedure, with *N,N,N′,N′*-tetramethylethylenediamine (TMEDA) substituted for (+)-sparteine.
Note: To ensure optimal separation and product stability, it is recommended that flash column chromatography be completed within 30 minutes.

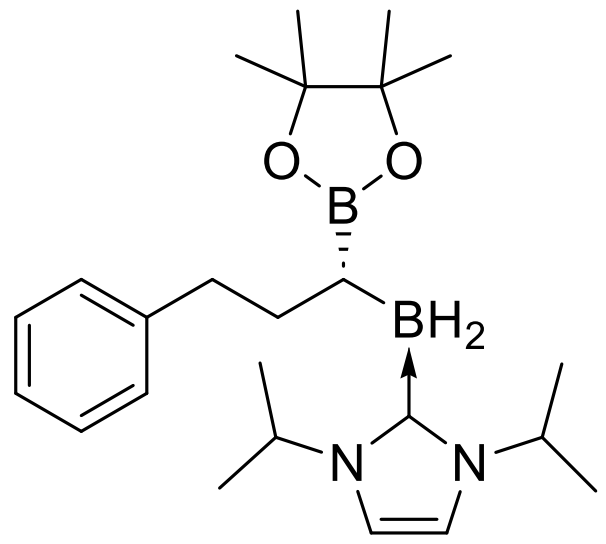

3b

3b: Yield: 23.8 mg, 58%, 89% ee, colorless oil, R_f = 0.4 (*n*-hexane/ethyl acetate = 3/1).
^{1}H NMR (400 MHz, $CDCl_3$) δ 7.21 – 7.05 (m, 5H), 6.91 – 6.84 (m, 2H), 5.50 – 4.86 (m, 2H), 2.77 – 2.63 (m, 1H), 2.45 – 2.30 (m, 1H), 1.97 – 1.80 (m, 1H), 1.33 – 1.30 (m, 6H), 1.29 – 1.23 (m, 18H), 1.14 – 1.05 (m, 1H), 0.29 – 0.17 (m, 1H).
^{13}C NMR (101 MHz, $CDCl_3$) δ 144.6, 128.6, 128.0, 125.0, 115.3, 81.6, 48.9, 40.2, 35.3, 25.3, 24.6, 23.4, 23.2.
^{11}B NMR (160 MHz, $CDCl_3$) δ 36.5 (s, 1B), -27.0 (t, *J* = 85.1 Hz, 1B).
HRMS (ESI): Calcd for $C_{24}H_{40}B_2N_2O_2Na^+$ $[M+Na]^+$: 433.3168, found: 433.3176.
IR (neat, cm^{-1}): *v* 2977, 2923, 2310, 1494, 1367, 1261, 1210, 1146, 1097, 1018.
Chiral HPLC (IB, ^{i}PrOH/*n*-hexane = 1/99, 1.0 mL/min) t_R 15.7 min (major), 21.5 min (minor).
$[\alpha]_D^{25}$ = -30.14 (*c* = 0.6, $CHCl_3$).

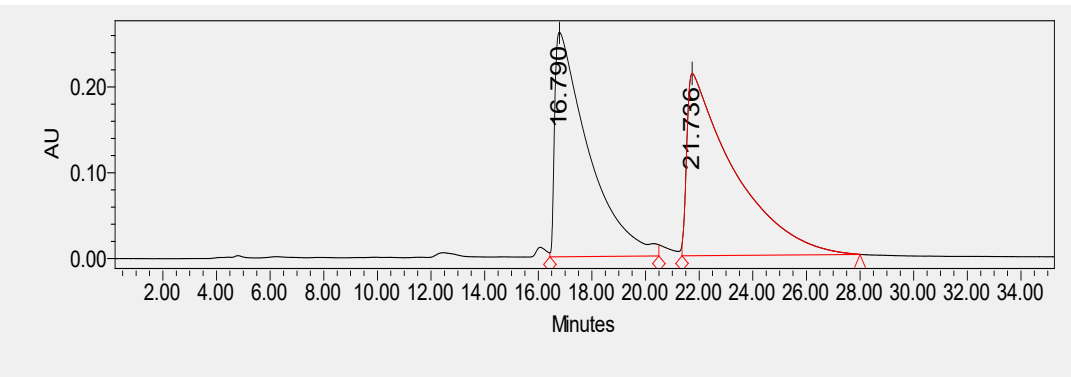

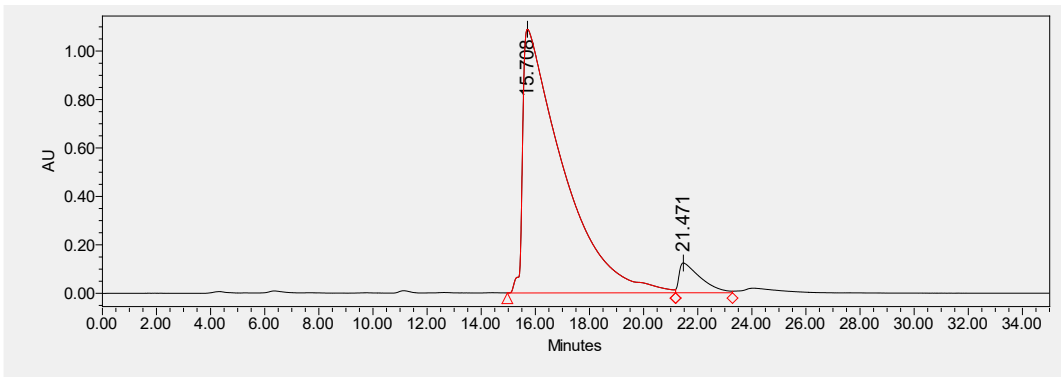

	Retention Time	Area	% Area
1	16.790	40485442	46.28
2	21.736	46993937	53.72

	Retention Time	Area	% Area
1	15.708	112054669	94.32
2	21.471	6747738	5.68

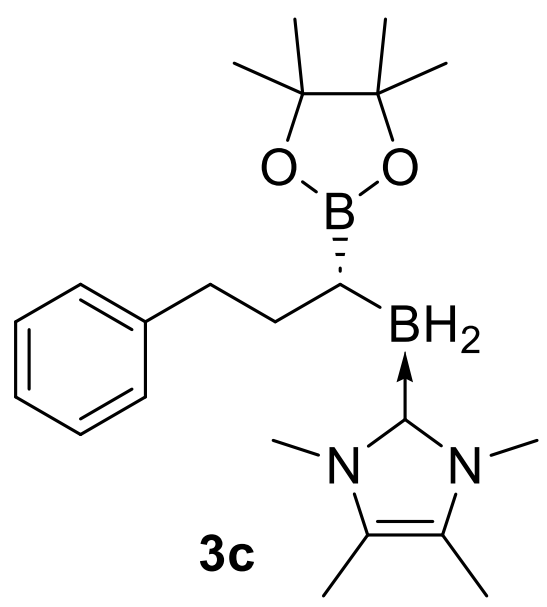

3c

3c: Yield: 19.9 mg, 52%, 98% ee, colorless oil, R_f = 0.4 (*n*-hexane/ethyl acetate = 1/1).
^{1}H NMR (400 MHz, $CDCl_3$) δ 7.22 – 7.17 (m, 2H), 7.16 – 7.06 (m, 3H), 3.59 (s, 6H), 2.82 – 2.55 (m, 1H), 2.45 – 2.31 (m, 1H), 2.07 (s, 6H), 1.93 – 1.80 (m, 1H), 1.26 – 1.14 (m, 13H), 0.27 – 0.18 (m, 1H).
^{13}C NMR (126 MHz, $CDCl_3$) δ 144.9, 128.7, 128.0, 125.0, 123.0, 81.5, 40.3, 35.7, 32.8, 25.3, 24.6, 8.9.
^{11}B NMR (128 MHz, $CDCl_3$) δ 33.5 (s, 1B), -26.5 (t, *J* = 86.0 Hz, 1B).
HRMS (ESI): Calcd for $C_{22}H_{36}B_2N_2O_2Na^+$ $[M+Na]^+$: 405.2855, found: 405.2851.

Chiral HPLC (IB, ^{i}PrOH/n-hexane = 10/90, 1.0 mL/min) t_R 11.3 min (major), 14.5 min (minor).
IR (neat, cm^{-1}): ν 2978, 2923, 2310, 1320, 1225, 1175.
$[\alpha]_D^{25}$= -14.10 (c = 0.1, $CHCl_3$).

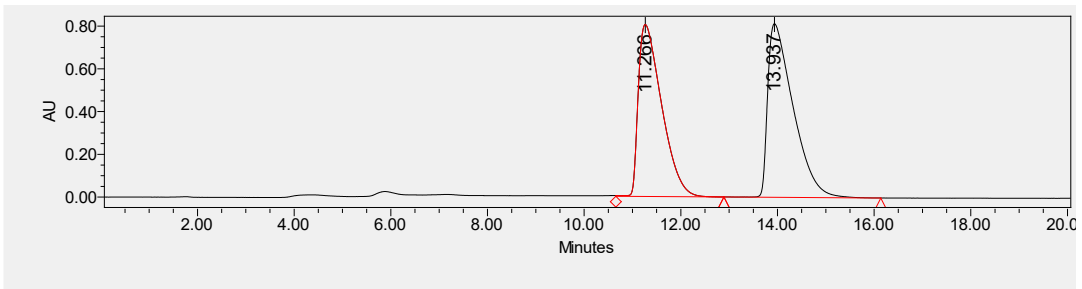

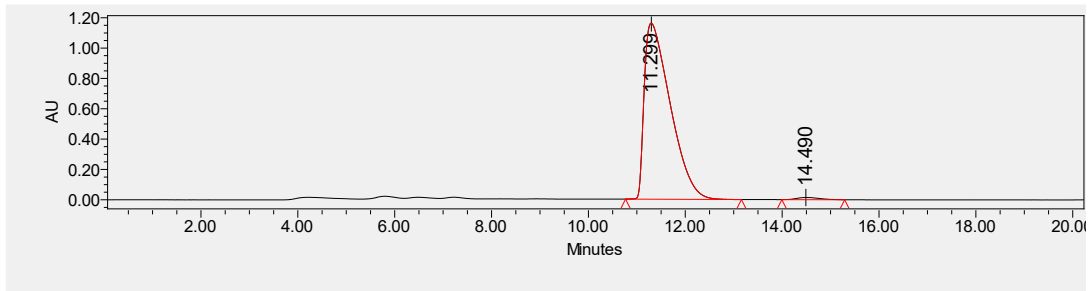

	Retention Time	Area	% Area
1	11.266	26930907	47.90
2	13.937	29286942	52.10

	Retention Time	Area	% Area
1	11.299	43189444	98.95
2	14.490	459100	1.05

5. Synthesis of chiral *gem*-diborylalkanes 3.

5.1. Synthesis of compounds 3a, (*R*)-3a, 3d-3ad.

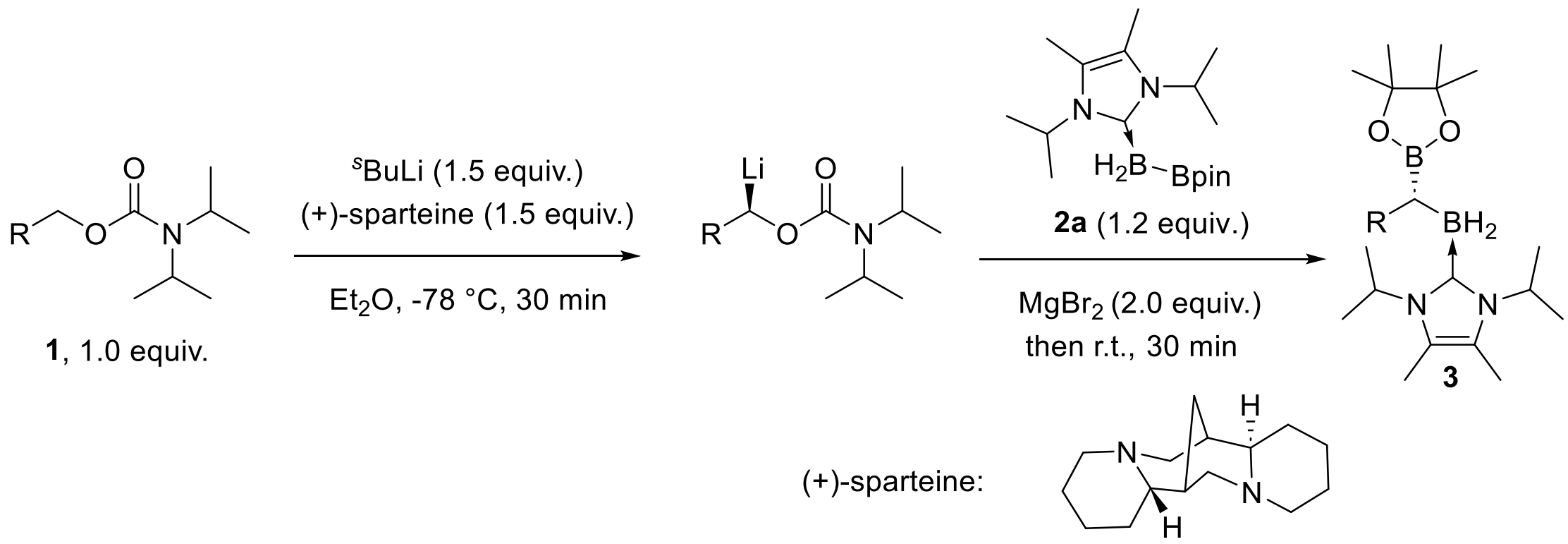

Synthesis of compounds 3 (a representative procedure for 3a, (*R*)-3a, 3d-3ad) Under argon, to an oven dried 4 mL reaction tube equipped with a magnetic stir bar and a septum was added carbamate **1** (0.1 mmol, 1.0 equiv.), (+)-sparteine (35.2 mg, 0.15 mmol, 1.5 equiv.) and Et_2O (500 μL). The reaction was cooled to -78 °C and stirred for 10 min. ^{s}BuLi (1.3 M in cyclohexane, 0.12 mL, 0.15 mmol, 1.5 equiv.) was then slowly added and the mixture was stirred vigorously (without splashing) at -78 °C for 30 min. After that, a solution of **2a** (38.4 mg, 0.12 mmol, 1.2 equiv.) in Et_2O (1.2 mL) was added dropwise. The reaction mixture was stirred at this temperature for an additional 10 min. $MgBr_2$ (freshly made from Mg and 1,2-dibromoethane, 0.5 M in Et_2O, 0.4 mL, 0.2 mmol, 2.0 equiv.) was then added dropwise and the reaction mixture was stirred at -78 °C for 20 min. After that, the reaction mixture was warmed to room temperature and stirred for another 30 min. Upon completion, the reaction was quenched with aqueous NH_4Cl and extracted with ethyl acetate (3 × 3 mL). The combined organic phase was dried over $MgSO_4$, and the solvent was removed in vacuo. The resulting residue was purified by flash column chromatography to afford the desired CDBA reagents **3**. The racemic product was prepared using the same procedure, with *N,N,N',N'*-tetramethylethylenediamine (TMEDA) substituted for (+)-sparteine. (*R*)-**3a**

was prepared using the same procedure, with (–)-sparteine substituted for (+)-sparteine. **3p** and **3ac** were prepared using 2.5 equiv. ^{s}BuLi and 2.5 equiv. (+)-sparteine.
Note: To ensure optimal separation and product stability, it is recommended that flash column chromatography be completed within 30 minutes.

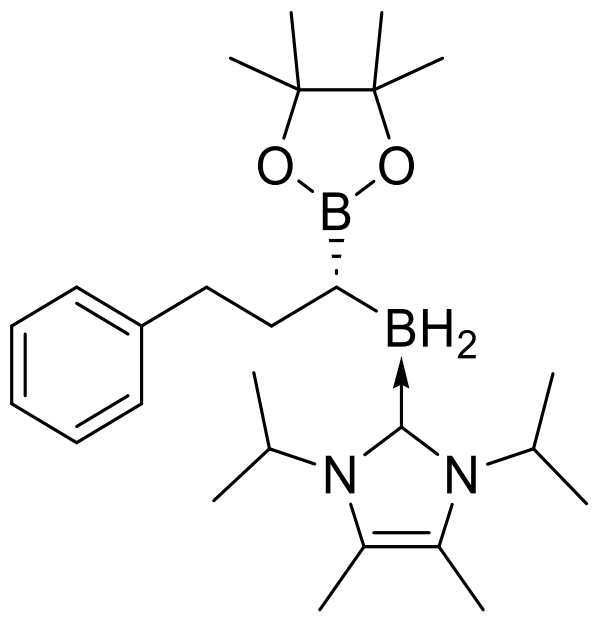

3a

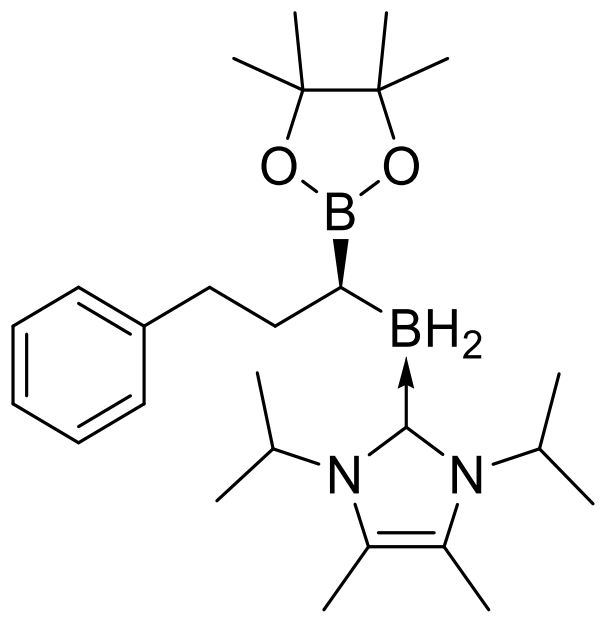

(*R*)-**3a**

3a: Yield: 37.7 mg, 86%, 98% ee, colorless oil, R_f = 0.4 (*n*-hexane/ethyl acetate = 3/1).
(*R*)-**3a**: Yield: 29.8 mg, 68%, 98% ee, colorless oil, R_f = 0.4 (*n*-hexane/ethyl acetate = 3/1).
(*R*)-**3a** was synthesized using (–)-sparteine instead of (+)-sparteine.
^{1}H NMR (400 MHz, $CDCl_3$) δ 7.23 – 7.01 (m, 5H), 5.75 – 5.34 (m, 2H), 2.76 – 2.66 (m, 1H), 2.41 – 2.31 (m, 1H), 2.18 (s, 6H), 1.91 – 1.79 (m, 1H), 1.40 – 1.30 (m, 12H), 1.30 – 1.19 (m, 12H), 1.18 – 1.02 (m, 1H), 0.29 – 0.11 (m, 1H).
^{13}C NMR (101 MHz, $CDCl_3$) δ 144.7, 128.7, 127.9, 125.0, 123.7, 81.5, 49.5, 40.4, 35.2, 25.3, 24.6, 21.7, 21.5, 10.6.
^{11}B NMR (128 MHz, $CDCl_3$) δ 37.7 (s, 1B), -26.0 (t, J = 85.2 Hz, 1B).
Note: After column chromatography, preparative thin-layer chromatography (PTLC) was employed to remove trace amounts of impurities to ensure the highest purity for the NMR spectra.
HRMS (ESI): Calcd for $C_{26}H_{44}B_2N_2O_2Na^+$ $[M+Na]^+$: 461.3481, found: 461.3490.
IR (neat, cm^{-1}): ν 2974, 2264, 1368, 1220, 1148.
For **3a**, **Chiral HPLC** (IB, ^{i}PrOH/*n*-hexane = 1/99, 1.0 mL/min) t_R 16.1 min (major), 26.2 min (minor).
$[\alpha]_D^{25}$= -49.89 (c = 0.7, $CHCl_3$).

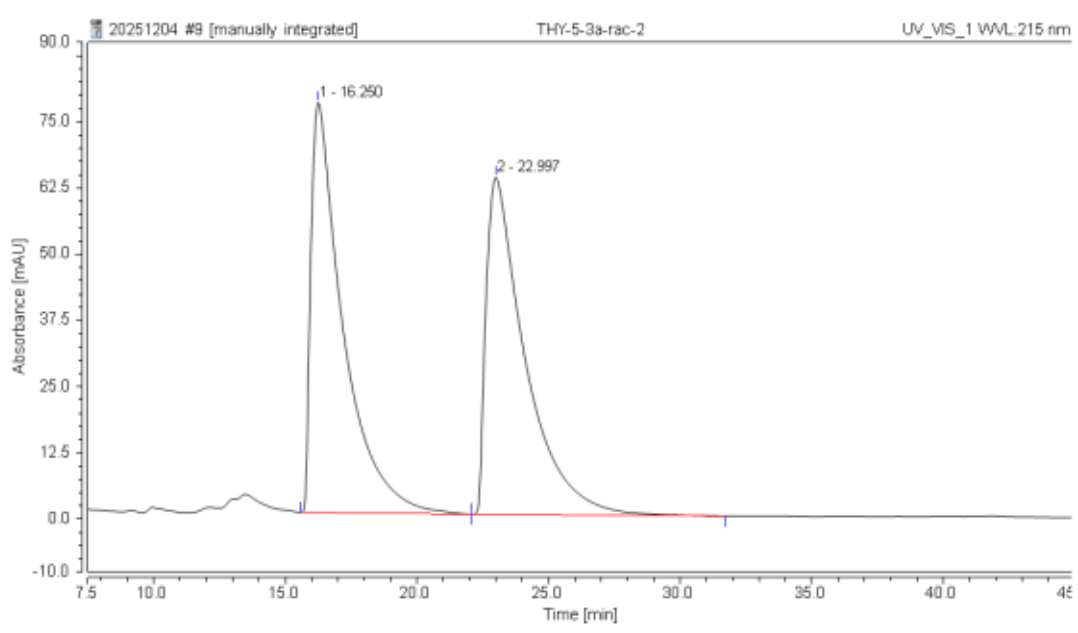

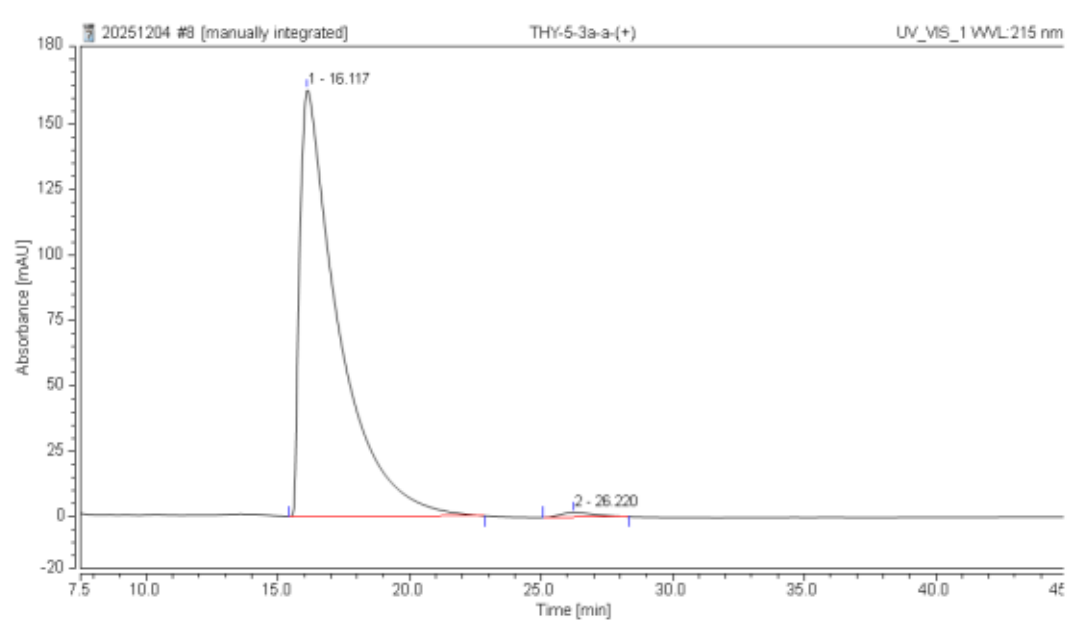

	Retention Time	Area	% Area
1	16.250	109.0133	49.89
2	22.997	109.5038	50.11

	Retention Time	Area	% Area
1	16.117	275.1637	99.04
2	26.220	2.6694	0.96

For (*R*)-**3a**, **Chiral HPLC** (IB, ^{i}PrOH/*n*-hexane = 1/99, 1.0 mL/min) t_R 17.4 min (minor), 22.7 min (major).

$[\alpha]_D^{25}$= 50.29 (*c* = 0.7, $CHCl_3$).

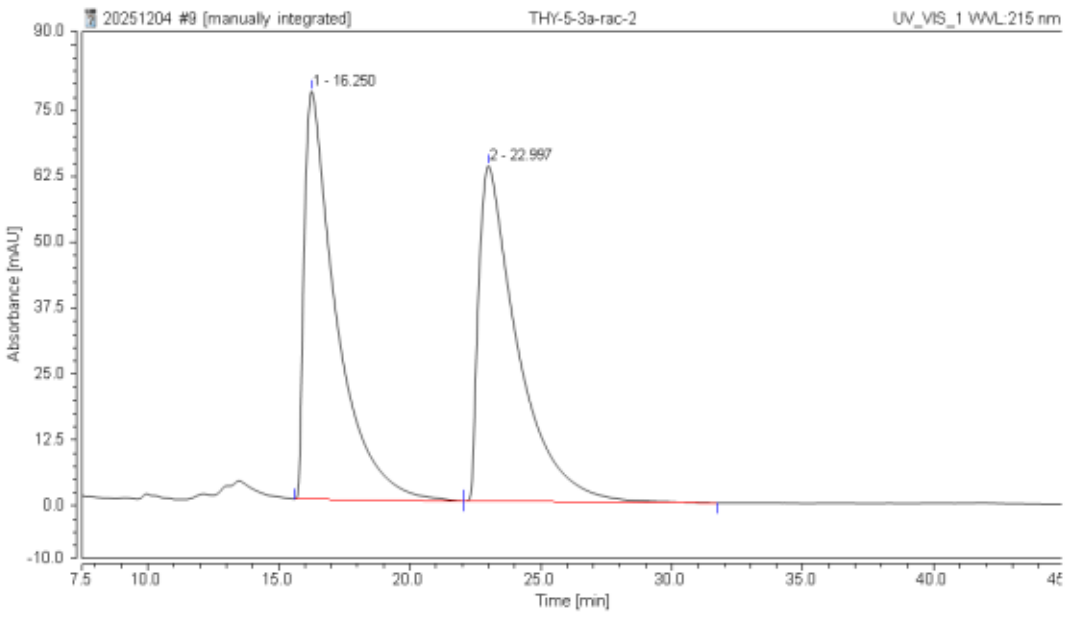

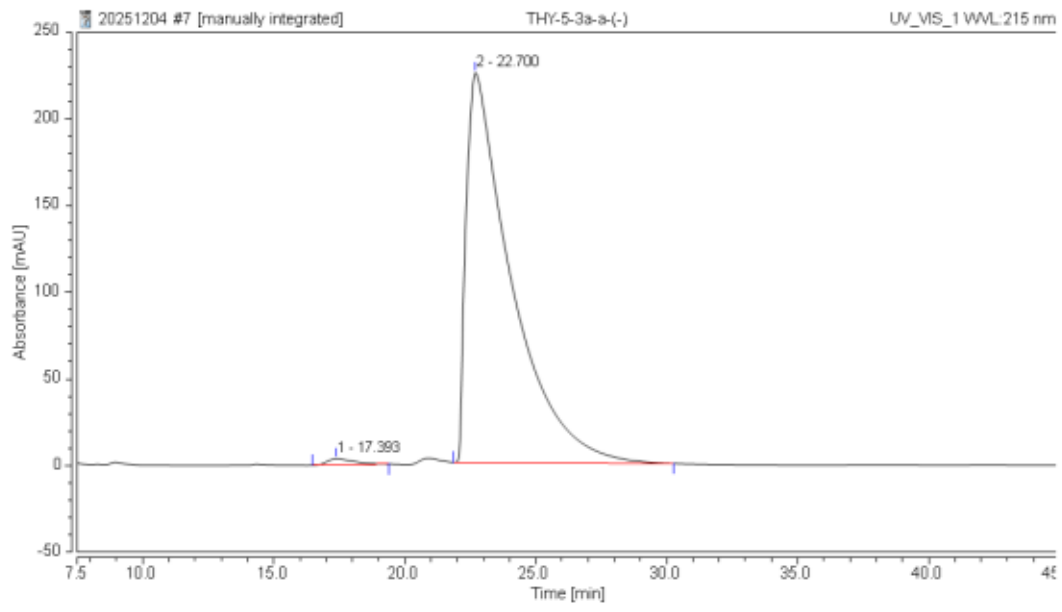

	Retention Time	Area	% Area
1	16.250	109.0133	49.89
2	22.997	109.5038	50.11

	Retention Time	Area	% Area
1	17.393	4.1011	0.91
2	22.700	446.7715	99.09

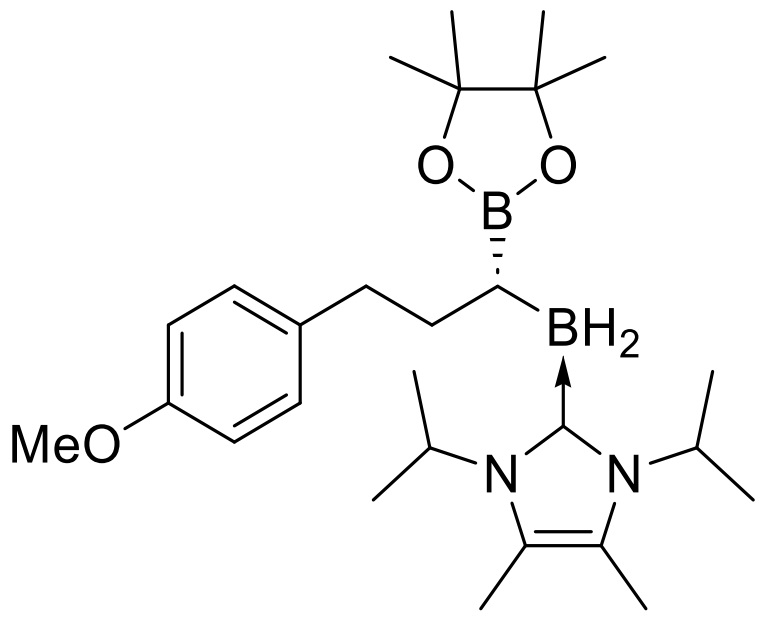

3d

3d: Yield: 37.6 mg, 80%, 94% ee, colorless oil, R_f = 0.4 (*n*-hexane/ethyl acetate = 3/1).

^{1}H NMR (400 MHz, $CDCl_3$) δ 7.03 (d, *J* = 8.5 Hz, 2H), 6.74 (d, *J* = 8.5 Hz, 2H), 5.60 – 5.37 (m, 2H), 3.74 (s, 3H), 2.75 – 2.56 (m, 1H), 2.36 – 2.24 (m, 1H), 2.18 (s, 6H), 1.90 – 1.76 (m, 1H), 1.40 – 1.30 (m, 12H), 1.29 – 1.24 (m, 12H), 1.11 – 1.02 (m, 1H), 0.20 – 0.17 (m, 1H).

^{13}C NMR (101 MHz, $CDCl_3$) δ 157.2, 136.9, 129.4, 123.7, 113.4, 81.5, 55.4, 49.5, 39.5, 35.4, 25.3, 24.6, 21.7, 21.5, 10.6.

^{11}B NMR (128 MHz, $CDCl_3$) δ 37.8 (s, 1B), -26.0 (t, *J* = 84.6 Hz, 1B).

HRMS (ESI): Calcd for $C_{27}H_{47}B_2N_2O_3^+$ $[M+H]^+$: 469.3767, found: 469.3777.

IR (neat, cm^{-1}): *v* 2958, 2269, 1381, 1305, 1097.

Chiral HPLC (OD-H, ^{i}PrOH/*n*-hexane = 1/99, 1.0 mL/min) t_R 14.1 min (major), 26.5 min (minor).

$[\alpha]_D^{25}$= -10.00 (*c* = 0.7, $CHCl_3$).

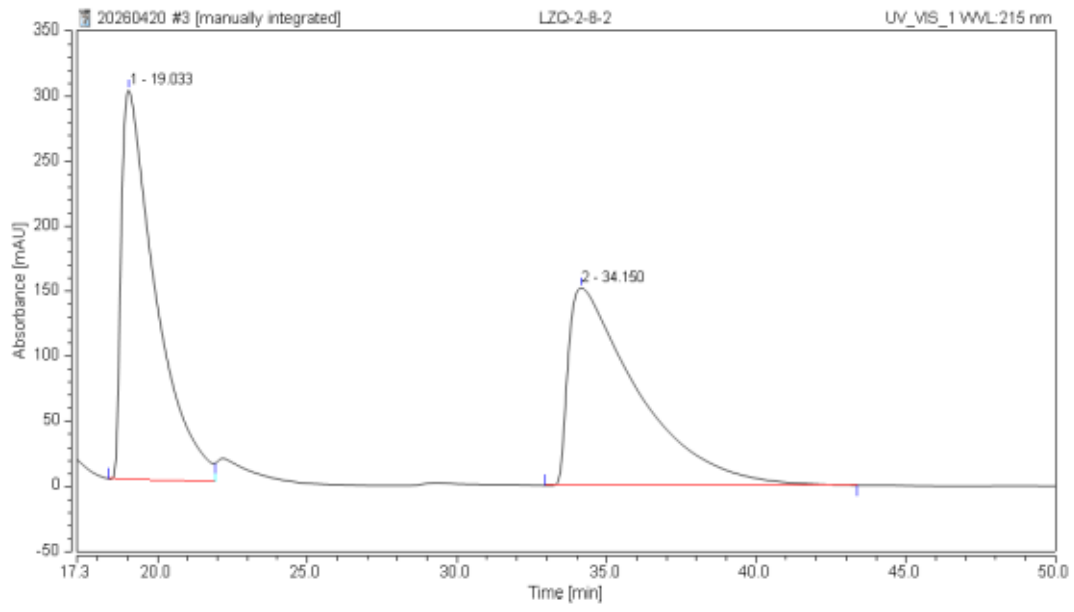

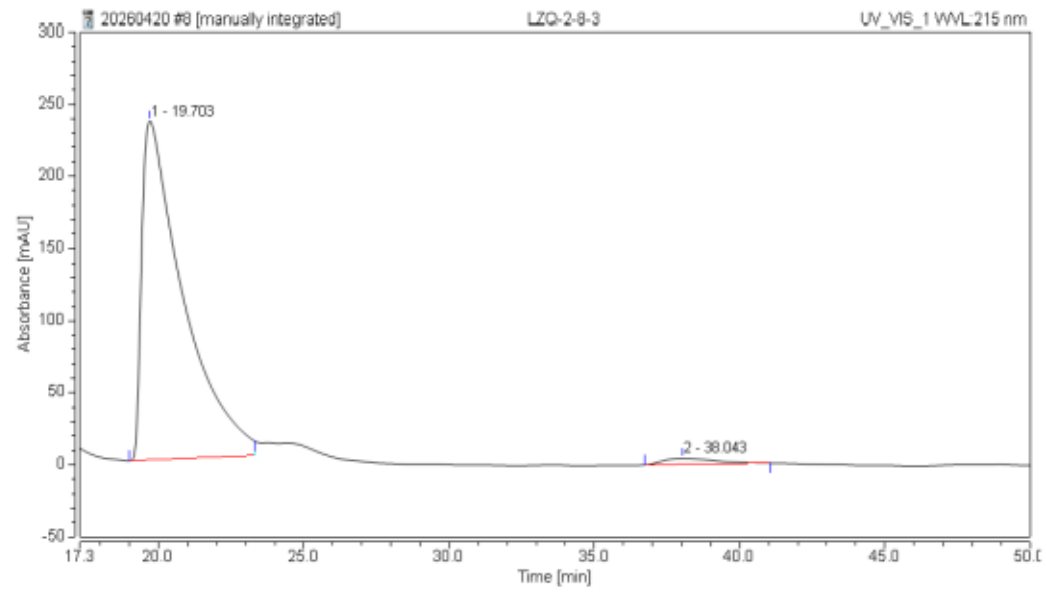

	Retention Time	Area	% Area
1	19.033	396.1367	49.33
2	34.15	406.9461	50.67

	Retention Time	Area	% Area
1	19.703	386.8741	97.83
2	38.043	8.5928	2.17

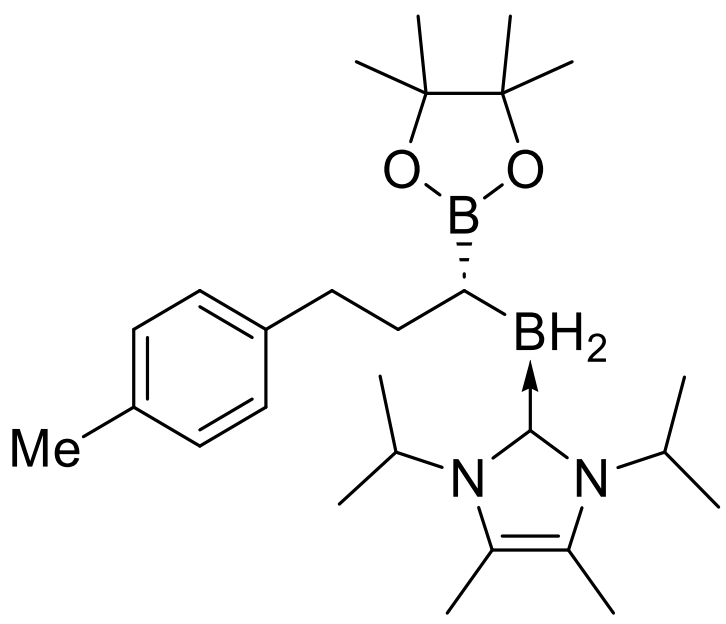

3e

3e: Yield: 38.4 mg, 85%, 97% ee, colorless oil, R_f = 0.4 (*n*-hexane/ethyl acetate = 3/1).
^{1}H NMR (500 MHz, $CDCl_3$) δ 7.04 – 6.87 (m, 4H), 5.67 – 5.38 (m, 2H), 2.74 – 2.59 (m, 1H), 2.38 – 2.28 (m, 1H), 2.26 (s, 3H), 2.18 (s, 6H), 1.93 – 1.73 (m, 1H), 1.48 – 1.29 (m, 12H), 1.29 – 1.21 (m, 12H), 1.14 – 1.03 (m, 1H), 0.27 – 0.13 (m, 1H).
^{13}C NMR (126 MHz, $CDCl_3$) δ 141.6, 134.2, 128.6, 128.5, 123.7, 81.5, 49.5, 40.0, 35.2, 25.3, 24.6, 21.7, 21.5, 21.1, 10.6.
^{11}B NMR (160 MHz, $CDCl_3$) δ 36.1 (s, 1B), -26.2 (t, J = 84.8 Hz, 1B).
Note: After column chromatography, preparative thin-layer chromatography (PTLC) was employed to remove trace amounts of impurities to ensure the highest purity for the NMR spectra.
HRMS (ESI): Calcd for $C_{27}H_{46}B_2N_2O_2Na^+$ $[M+Na]^+$: 475.3638, found: 475.3649.
IR (neat, cm^{-1}): ν 2973, 2923, 2287, 1443, 1398, 1261, 1147, 1022.
Chiral HPLC (IB-3, ^{i}PrOH/*n*-hexane = 1/99, 1.0 mL/min) t_R 7.6 min (major), 9.7 min (minor).
$[\alpha]_D^{25}$= -20.57 (c = 0.7, $CHCl_3$).

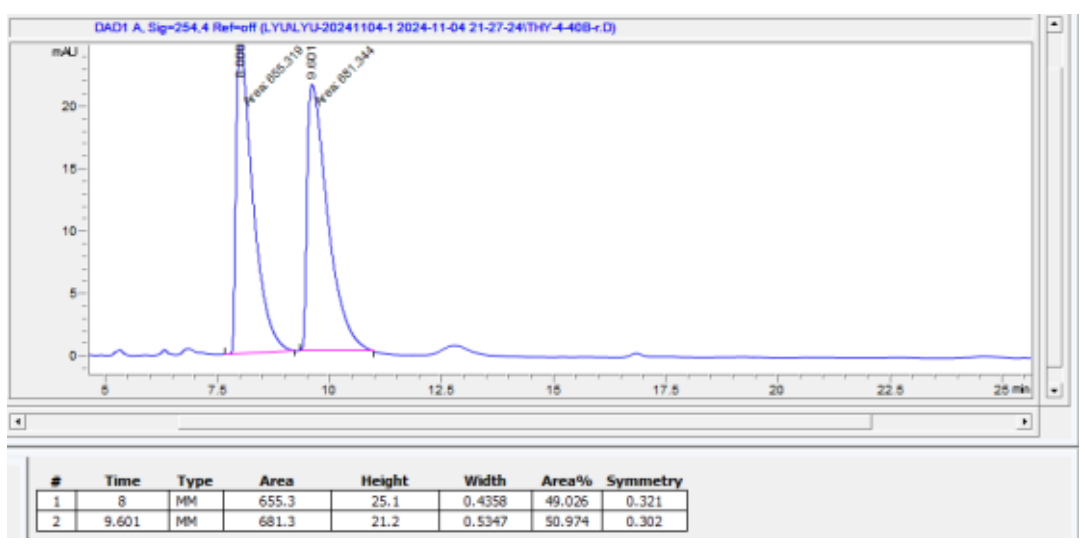

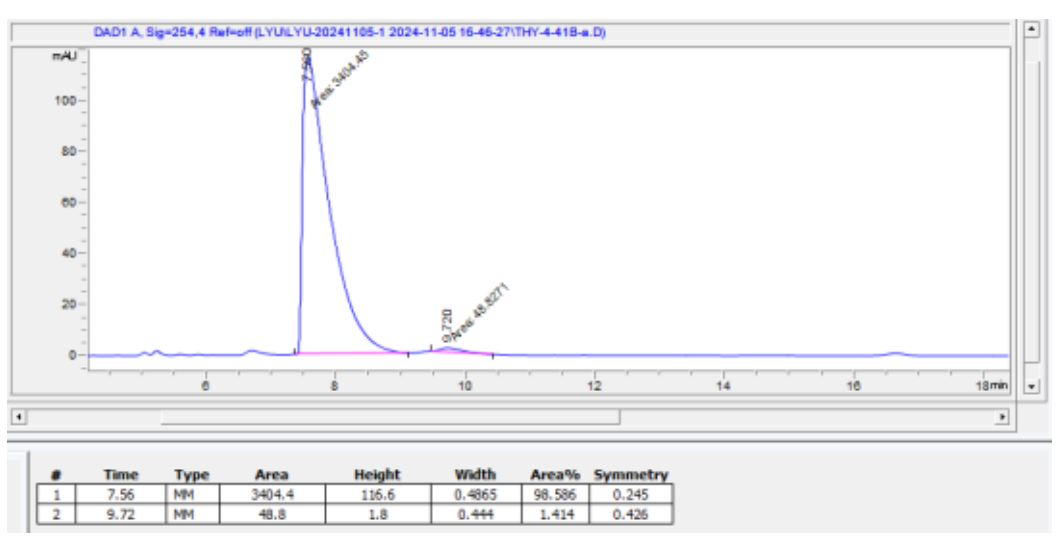

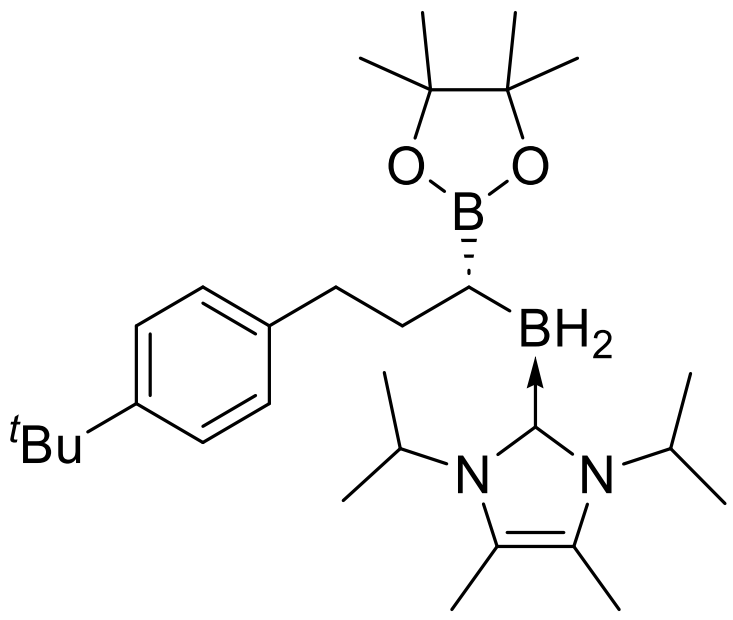

3f

3f: Yield: 36.1 mg, 73%, 97% ee, colorless oil, R_f = 0.4 (*n*-hexane/ethyl acetate = 3/1).
^{1}H NMR (400 MHz, $CDCl_3$) δ 7.21 (d, *J* = 8.2 Hz, 2H), 7.05 (d, *J* = 8.2 Hz, 2H), 5.68 – 5.41 (m, 2H), 2.76 – 2.61 (m, 1H), 2.41 – 2.27 (m, 1H), 2.17 (s, 6H), 1.99 – 1.79 (m, 1H), 1.43 – 1.34 (m, 6H), 1.34 – 1.11 (m, 27H), 1.14 – 1.02 (m, 1H), 0.25 – 0.11 (m, 1H).
^{13}C NMR (101 MHz, $CDCl_3$) δ 147.6, 141.5, 128.3, 124.8, 123.7, 81.5, 49.5, 39.6, 34.5, 34.3, 31.6, 25.2, 24.6, 21.6, 21.5, 10.6.
^{11}B NMR (128 MHz, $CDCl_3$) δ 36.0 (s, 1B), -26.0 (t, *J* = 84.8 Hz, 1B).
Note: After column chromatography, preparative thin-layer chromatography (PTLC) was employed to remove trace amounts of impurities to ensure the highest purity for the NMR spectra.
HRMS (ESI): Calcd for $C_{30}H_{52}B_2N_2O_2Na^+$ $[M+Na]^+$: 517.4107, found: 517.4122.
IR (neat, cm^{-1}): *v* 2347, 2923, 2341, 1565, 1330, 1191, 1023.
Chiral HPLC (IB-3, ^{i}PrOH/*n*-hexane = 1/99, 1.0 mL/min) t_R 6.5 min (major), 8.9 min (minor).
$[\alpha]_D^{25}$= -21.00 (*c* = 0.8, $CHCl_3$).

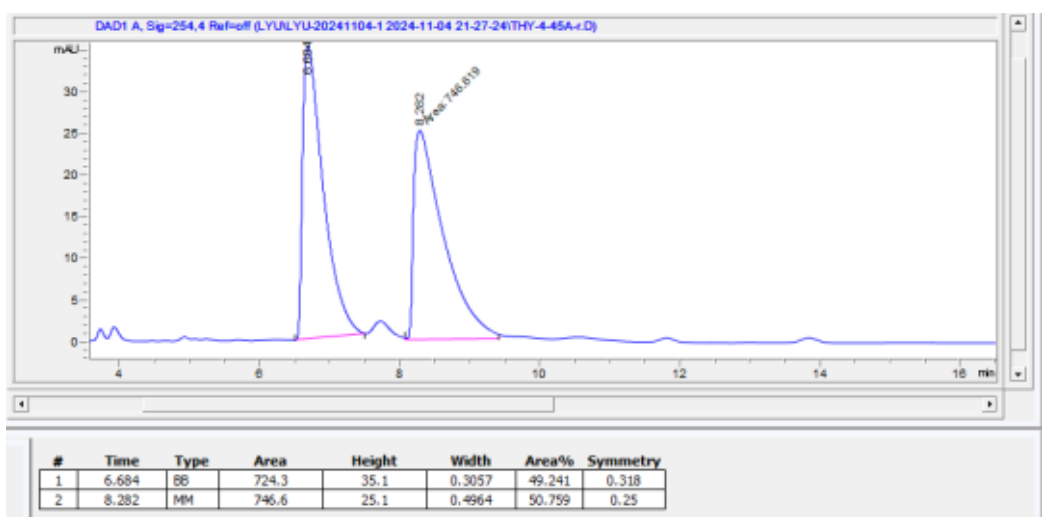

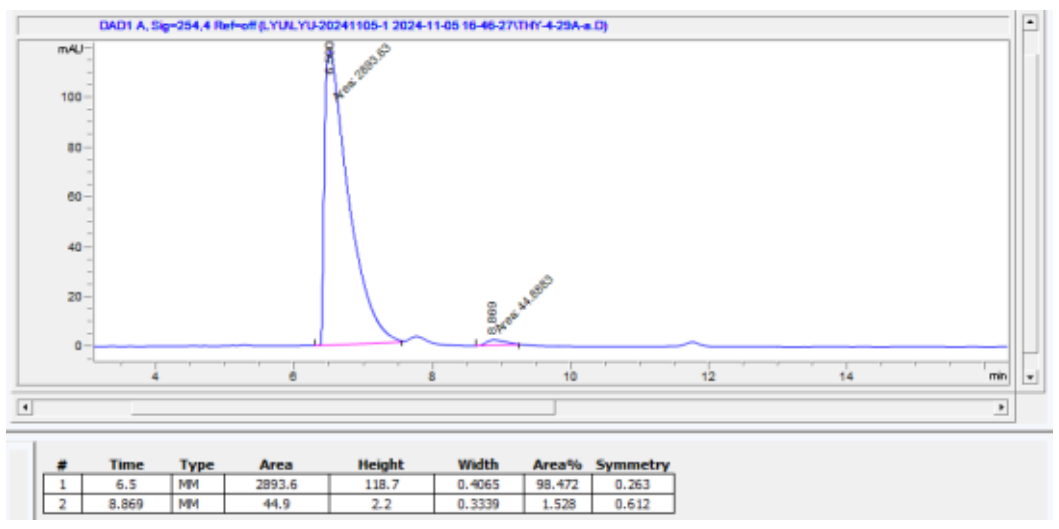

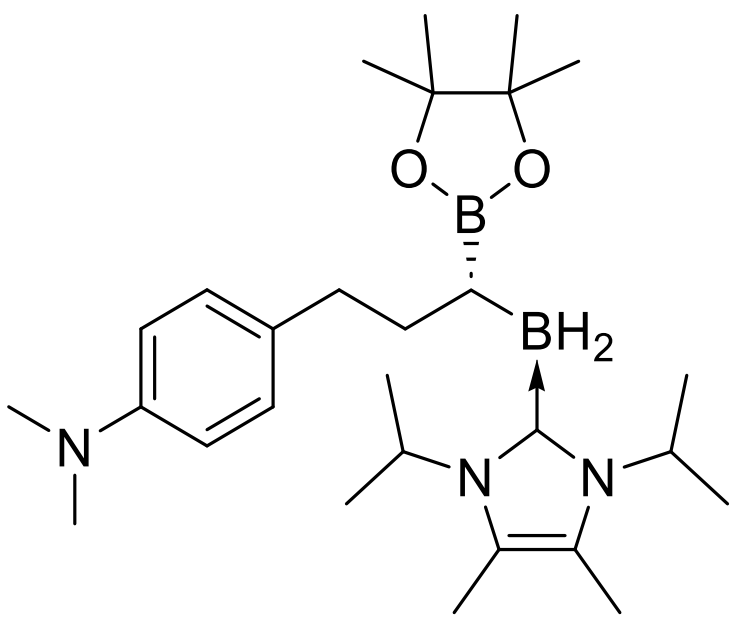

3g

3g: Yield: 30.3 mg, 63%, >99% ee, yellow oil, R_f = 0.4 (*n*-hexane/ethyl acetate = 2/1).

^{1}H NMR (400 MHz, $CDCl_3$) δ 7.01 (d, *J* = 8.6 Hz, 2H), 6.64 (d, *J* = 8.6 Hz, 2H), 5.72 – 5.25 (m, 2H), 2.85 (s, 6H), 2.70 – 2.47 (m, 1H), 2.34 – 2.21 (m, 1H), 2.17 (s, 6H), 1.94 – 1.74 (m, 1H), 1.42 – 1.31 (m, 12H), 1.31 – 1.22 (m, 12H), 1.13 – 0.97 (m, 1H), 0.30 – 0.13 (m, 1H).
^{13}C NMR (101 MHz, $CDCl_3$) δ 148.8, 133.6, 129.1, 123.7, 113.3, 81.4, 49.5, 41.4, 39.4, 35.2, 25.3, 24.6, 21.7, 21.5, 10.6.
^{11}B NMR (128 MHz, $CDCl_3$) δ 36.1 (s, 1B), -26.2 (t, *J* = 84.6 Hz, 1B).
HRMS (ESI): Calcd for $C_{28}H_{49}B_2N_3O_2Na^+$ $[M+Na]^+$: 504.3903, found: 504.3915.
IR (neat, cm^{-1}): *v* 2973, 2922, 2286, 1519, 1398, 1262, 1146, 1019.
Chiral HPLC (OD-H, ^{i}PrOH/*n*-hexane = 1/99, 1.0 mL/min) t_R 48.3 min (major), 61.3 min (minor).
$[\alpha]_D^{25}$= -11.86 (*c* = 0.6, $CHCl_3$).

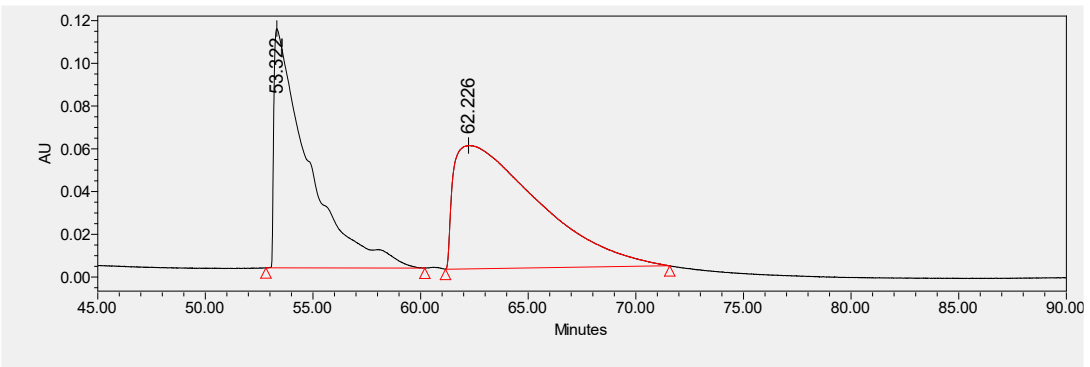

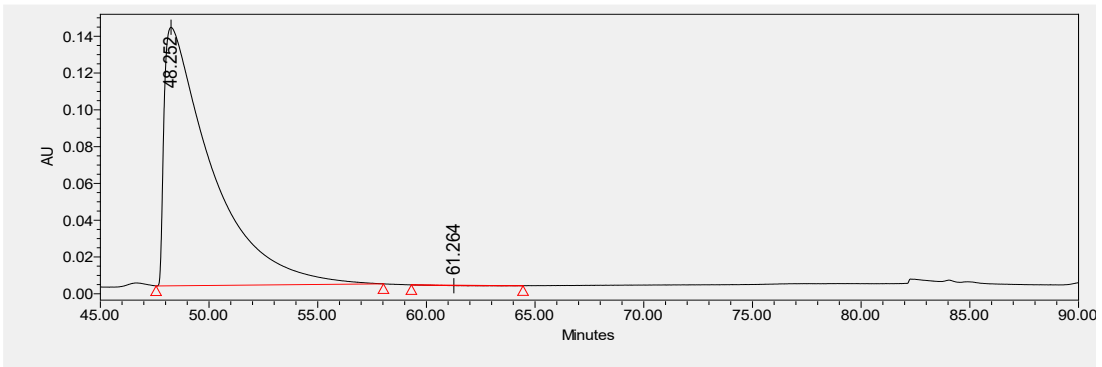

	Retention Time	Area	% Area
1	53.322	12334791	47.21
2	62.226	13790300	52.79

	Retention Time	Area	% Area
1	48.252	21283119	99.78
2	61.264	46847	0.22

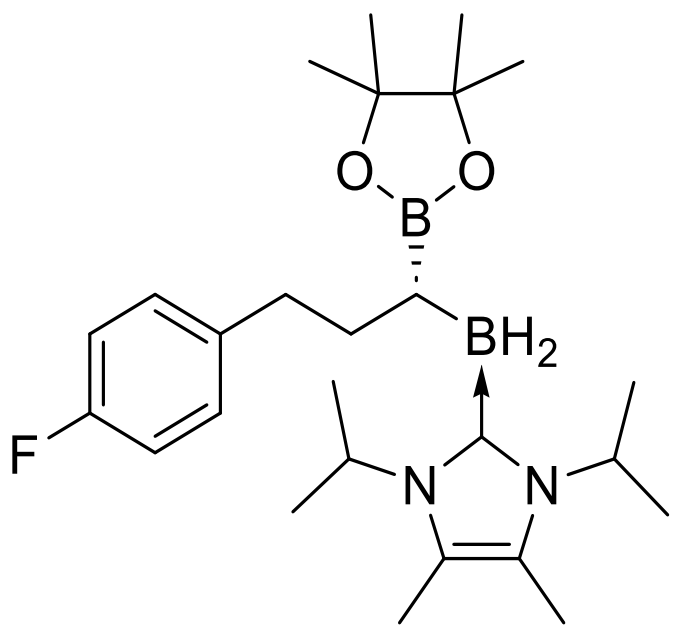

3h

3h: Yield: 16.1 mg, 35%, 99% ee, colorless oil, R_f = 0.4 (*n*-hexane/ethyl acetate = 3/1).
^{1}H NMR (400 MHz, $CDCl_3$) δ 7.17 – 7.01 (m, 2H), 6.95 – 6.80 (m, 2H), 5.64 – 5.38 (m, 2H), 2.79 – 2.61 (m, 1H), 2.47 – 2.31 (m, 1H), 2.21 (s, 6H), 1.94 – 1.77 (m, 1H), 1.44 – 1.32 (m, 12H), 1.32 – 1.20 (m, 12H), 1.14 – 1.04 (m, 1H), 0.26 – 0.11 (m, 1H).
^{13}C NMR (101 MHz, $CDCl_3$) δ 160.9 (d, $J_{C\text{-}F}$ = 241.5 Hz), 140.2 (d, $J_{C\text{-}F}$ = 3.3 Hz), 129.9 (d, $J_{C\text{-}F}$ = 7.5 Hz), 123.7, 114.5 (d, *J* = 20.8 Hz), 81.5, 49.5, 39.5, 35.6, 25.3, 24.6, 21.7, 21.5, 10.6.
^{11}B NMR (128 MHz, $CDCl_3$) δ 36.9 (s, 1B), -25.9 (t, *J* = 84.8 Hz, 1B).
^{19}F NMR (471 MHz, $CDCl_3$) δ -118.9 – -120.1 (m, 1F).
Note: After column chromatography, preparative thin-layer chromatography (PTLC) was employed to remove trace amounts of impurities to ensure the highest purity for the NMR spectra.
HRMS (ESI): Calcd for $C_{26}H_{43}B_2FN_2O_2Na^+$ $[M+Na]^+$: 479.3387, found: 479.3397.
IR (neat, cm^{-1}): *v* 2971, 2258, 1382, 1312, 1216, 1094.

Chiral HPLC (IB, ^{i}PrOH/n-hexane = 1/99, 1.0 mL/min) t_R 8.9 min (major), 10.6 min (minor).

$[\alpha]_D^{25}$= -13.67 (c = 0.3, $CHCl_3$).

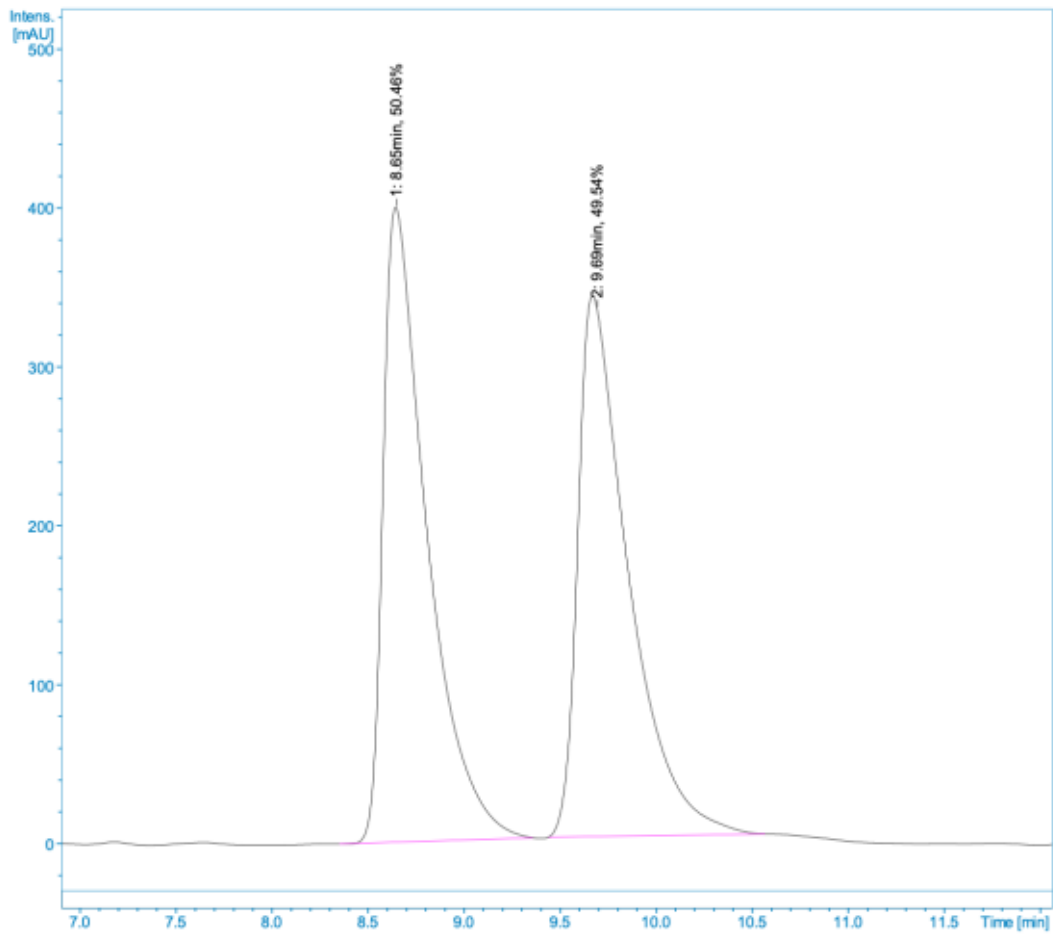

Peak	RT [min]	Peak Height [mAU]	Integral [%]	Peak Area [mAU*s]	FWHM [min]	Integral Region [min]	A
1	8.59	398.34	50.46	31648.9	0.2313	8.35 - 9.36	
2	9.64	331.21	49.54	31071.7	0.2703	9.44 - 10.57	

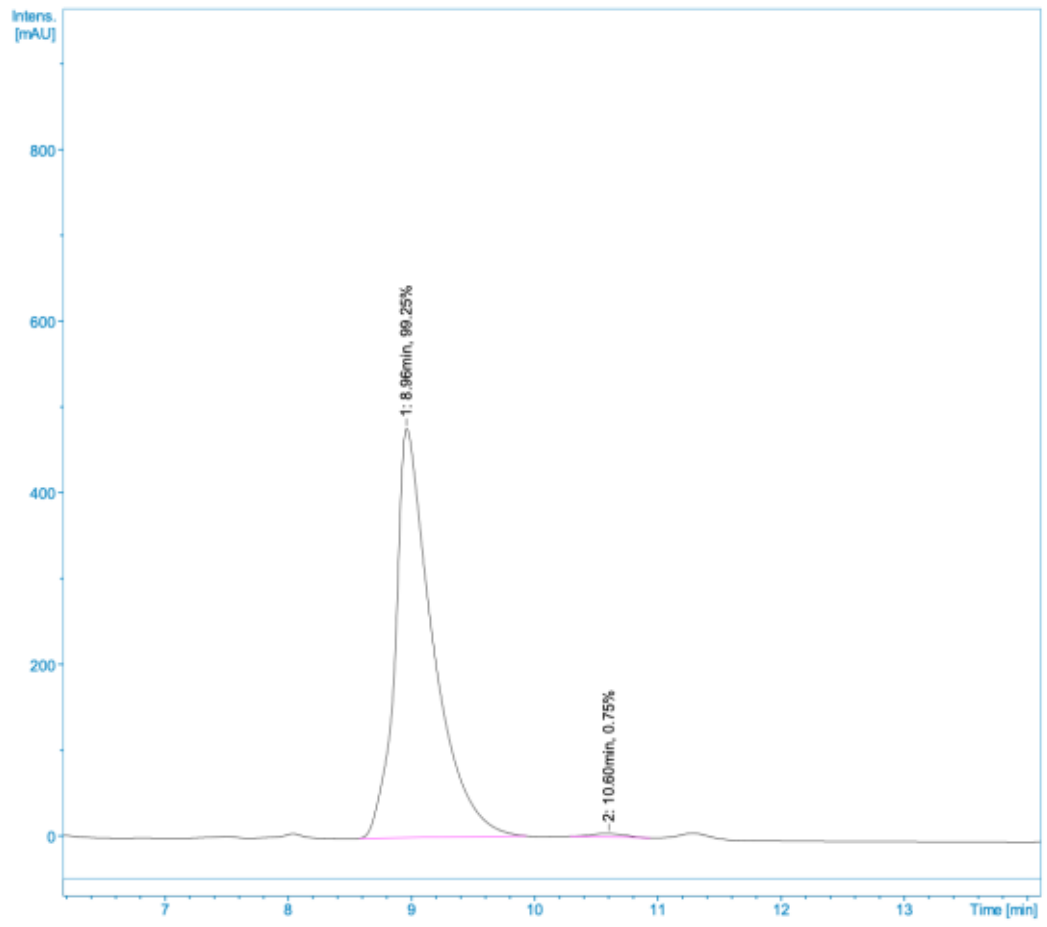

Peak	RT [min]	Peak Height [mAU]	Integral [%]	Peak Area [mAU*s]	FWHM [min]	Integral Region [min]	Ann
1	8.91	476.34	99.25	50600.2	0.2906	8.58 - 9.93	
2	10.55	4.01	0.75	384.8	0.3234	10.29 - 10.94	

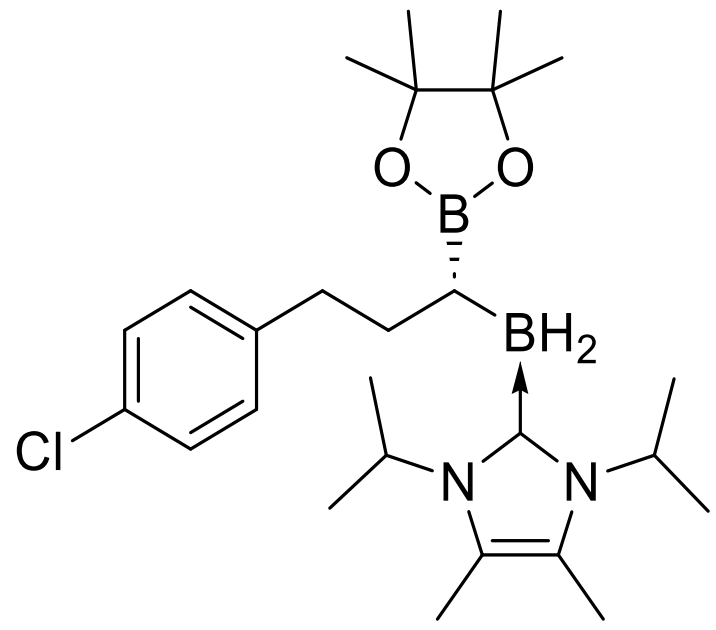

3i

3i: Yield: 38.7 mg, 82%, 99% ee, colorless oil, R_f = 0.4 (n-hexane/ethyl acetate = 3/1).

^{1}H NMR (400 MHz, $CDCl_3$) δ 7.13 (d, J = 8.0 Hz, 2H), 7.02 (d, J = 8.0 Hz, 2H), 5.55 – 5.35 (m, 2H), 2.82 – 2.57 (m, 1H), 2.41 – 2.29 (m, 1H), 2.18 (s, 6H), 1.88 – 1.75 (m, 1H), 1.39 – 1.29 (m, 12H), 1.30 – 1.20 (m, 12H), 1.09 – 1.01 (m, 1H), 0.19 – 0.11 (m, 1H).

^{13}C NMR (101 MHz, $CDCl_3$) δ 143.1, 130.5, 130.0, 127.9, 123.7, 81.5, 49.5, 39.7, 35.4, 25.2, 24.6, 21.6, 21.5, 10.6.

^{11}B NMR (128 MHz, $CDCl_3$) δ 34.7 (s, 1B), -26.0 (t, J = 87.4 Hz, 1B).

HRMS (ESI): Calcd for $C_{26}H_{43}B_2ClN_2O_2Na^+$ $[M+Na]^+$: 495.3091, found: 495.3104.

IR (neat, cm^{-1}): ν 2974, 2933, 2295, 1398, 1367, 1220, 1144, 1111.

Chiral HPLC (AD-H, ^{i}PrOH/n-hexane = 1/99, 1.0 mL/min) t_R 6.7 min (minor), 7.9 min (major).

$[\alpha]_D^{25}$= -43.33 (c = 0.6, $CHCl_3$).

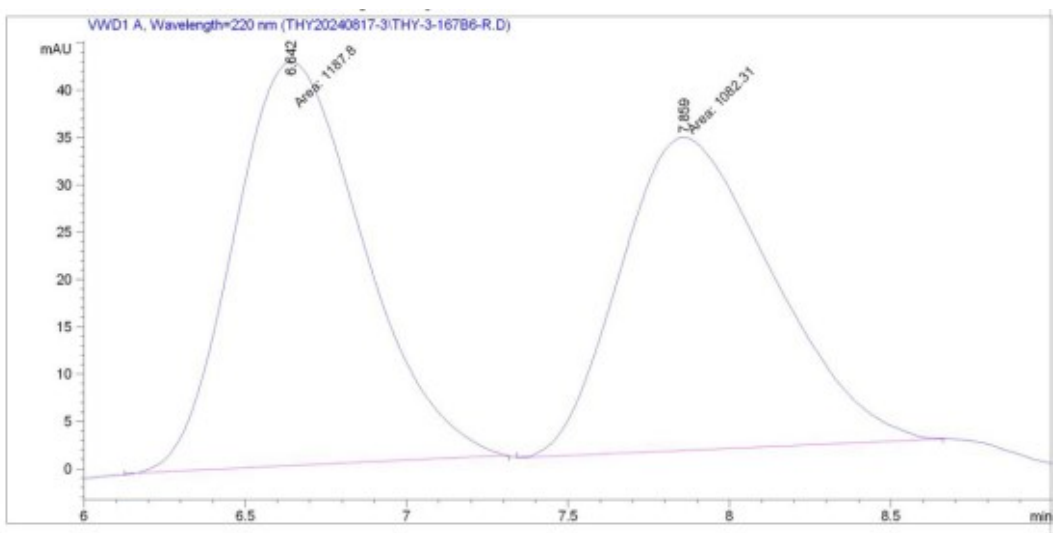

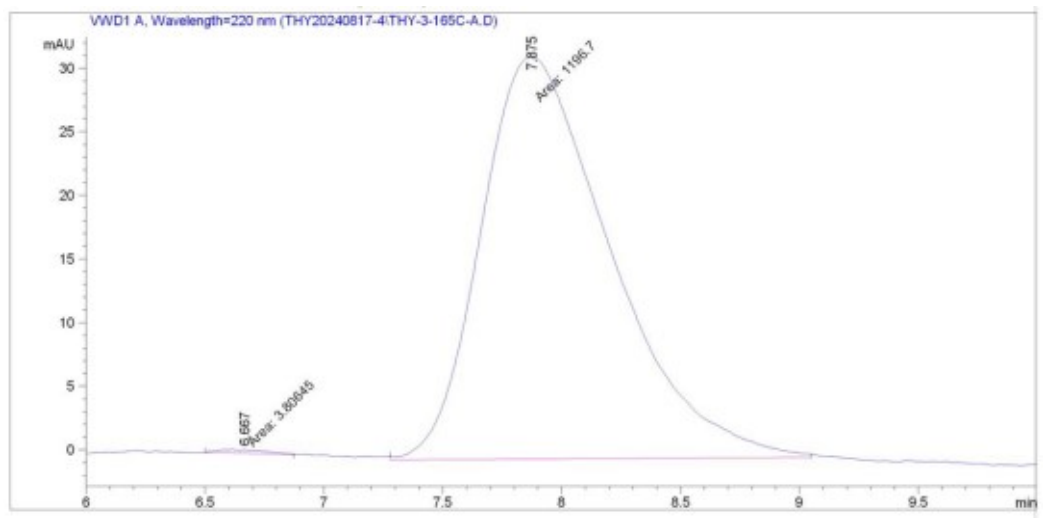

Peak #	RetTime [min]	Type	Width [min]	Area [mAU*s]	Height [mAU]	Area %
1	6.642	MM	0.4634	1187.80396	42.71758	52.3234
2	7.859	MM	0.5448	1082.31396	33.10775	47.6766

Peak #	RetTime [min]	Type	Width [min]	Area [mAU*s]	Height [mAU]	Area %
1	6.667	PM	0.2264	3.80645	2.80267e-1	0.3171
2	7.875	MM	0.6303	1196.69910	31.64190	99.6829

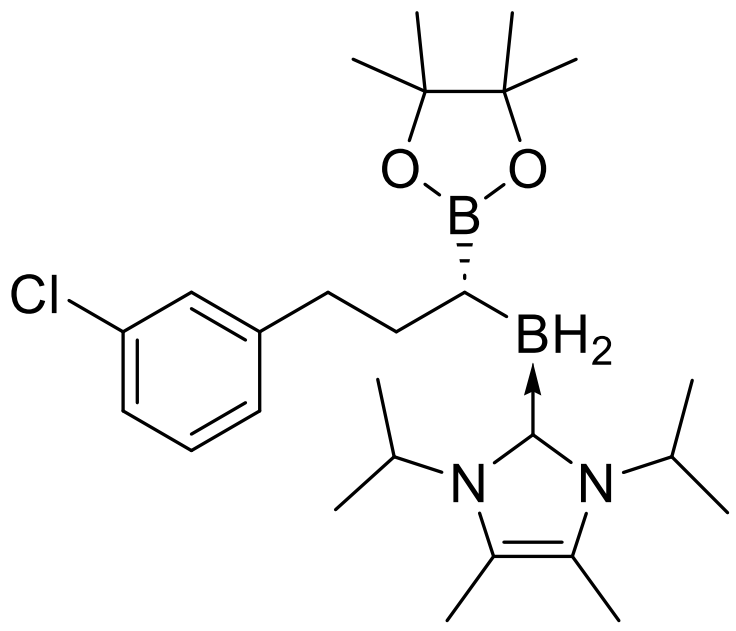

3j

3j: Yield: 34.0 mg, 72%, 98% ee, colorless oil, R_f = 0.4 (*n*-hexane/ethyl acetate = 3/1).
^{1}H NMR (500 MHz, $CDCl_3$) δ 7.15 – 6.89 (m, 4H), 5.56 – 5.37 (m, 2H), 2.76 – 2.58 (m, 1H), 2.41 – 2.30 (m, 1H), 2.19 (s, 6H), 1.89 – 1.76 (m, 1H), 1.42 – 1.29 (m, 12H), 1.29 – 1.22 (m, 12H), 1.18 – 1.02 (m, 1H), 0.27 – 0.05 (m, 1H).
^{13}C NMR (126 MHz, $CDCl_3$) δ 146.8, 133.6, 129.2, 128.6, 127.0, 125.1, 123.8, 81.5, 49.5, 39.9, 35.1, 25.2, 24.6, 21.6, 21.5, 10.6.
^{11}B NMR (160 MHz, $CDCl_3$) δ 36.3 (s, 1B), -26.2 (t, *J* = 85.5 Hz, 1B).
Note: After column chromatography, preparative thin-layer chromatography (PTLC) was employed to remove trace amounts of impurities to ensure the highest purity for the NMR spectra.
HRMS (ESI): Calcd for $C_{26}H_{43}B_2ClN_2O_2Na^+$ $[M+Na]^+$: 495.3091, found: 495.3104.
IR (neat, cm^{-1}): *ν* 2971, 2924, 2300, 1399, 1368, 1261, 1147, 1093, 1022.
Chiral HPLC (IB-3, ^{i}PrOH/*n*-hexane = 1/99, 1.0 mL/min) t_R 6.2 min (major), 7.5 min (minor).
$[\alpha]_D^{25}$ = -15.71 (*c* = 0.6, $CHCl_3$).

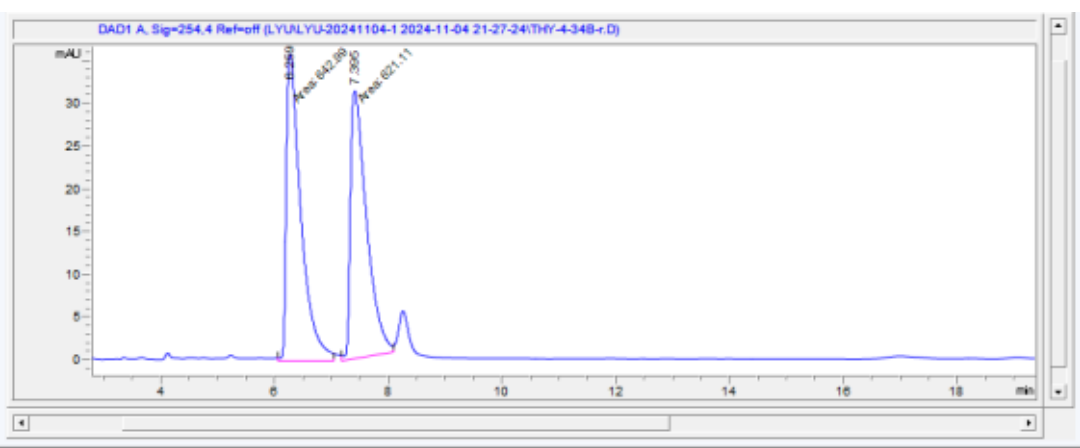

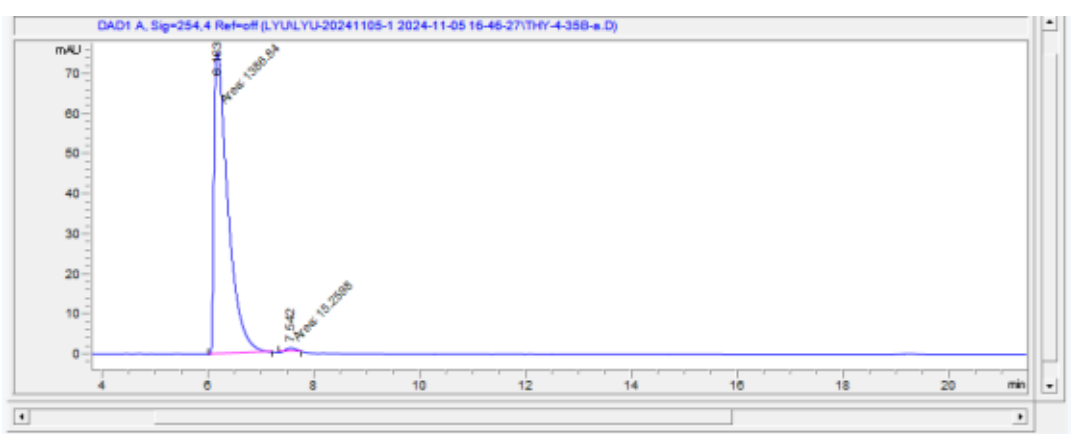

#	Time	Type	Area	Height	Width	Area%	Symmetry
1	6.259	MM	643	36	0.2978	50.865	0.331
2	7.395	MM	621.1	31.4	0.3295	49.135	0.354

#	Time	Type	Area	Height	Width	Area%	Symmetry
1	6.163	MM	1386.8	75.5	0.3062	98.912	0.292
2	7.542	MM	15.3	1.1	0.2347	1.088	0.83

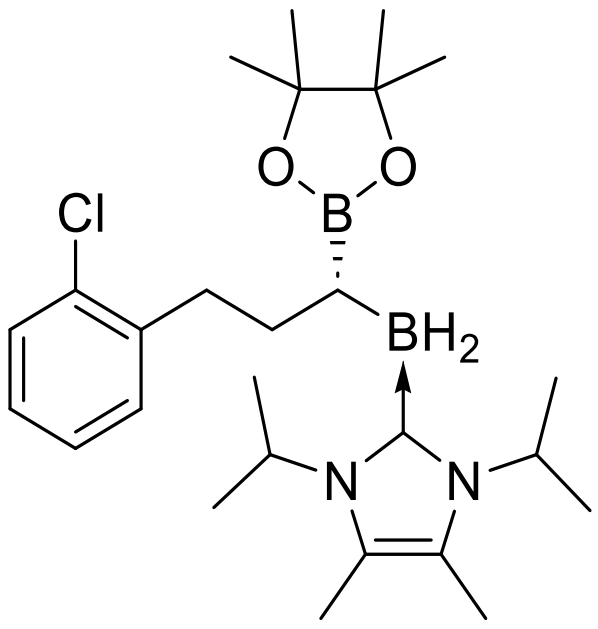

3k

3k: Yield: 29.3 mg, 62%, 94% ee, colorless oil, R_f = 0.4 (*n*-hexane/ethyl acetate = 3/1).
^{1}H NMR (500 MHz, $CDCl_3$) δ 7.23 – 6.96 (m, 4H), 5.60 – 5.40 (m, 2H), 2.82 – 2.69 (m, 1H), 2.63 – 2.41 (m, 1H), 2.18 (s, 6H), 1.83 – 1.72 (m, 1H), 1.49 – 1.30 (m, 12H), 1.30 – 1.20 (m, 12H), 1.20 – 1.14 (m, 1H), 0.24 – 0.11 (m, 1H).
^{13}C NMR (126 MHz, $CDCl_3$) δ 142.0, 133.8, 130.7, 129.0, 126.4, 126.4, 123.7, 81.5, 49.5, 37.7, 33.3, 25.3, 24.6, 22.7, 21.6, 21.5, 10.6.
^{11}B NMR (160 MHz, $CDCl_3$) δ 36.0 (s, 1B), -26.2 (t, J = 85.2 Hz, 1B).
Note: After column chromatography, preparative thin-layer chromatography (PTLC) was employed to remove trace amounts of impurities to ensure the highest purity for the NMR spectra.
HRMS (ESI): Calcd for $C_{26}H_{43}B_2ClN_2O_2Na^+$ $[M+Na]^+$: 495.3091, found: 495.3105.
IR (neat, cm^{-1}): ν 2972, 2927, 2297, 1691, 1399, 1368, 1290, 1220, 1146, 1049.
Chiral HPLC (IB-3, ^{i}PrOH/*n*-hexane = 1/99, 1.0 mL/min) t_R 7.4 min (major), 8.7 min (minor).
$[\alpha]_D^{25}$= -16.83 (c = 0.6, $CHCl_3$).

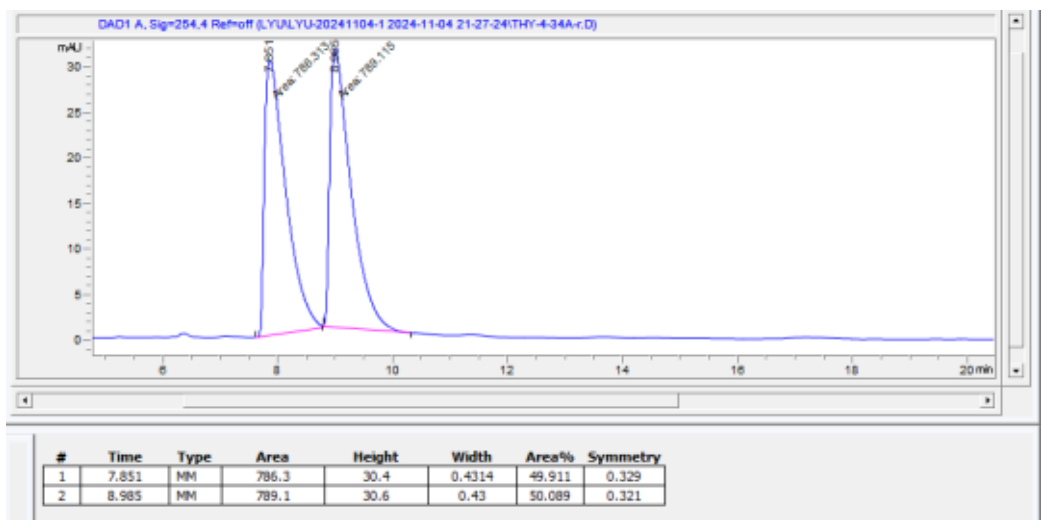

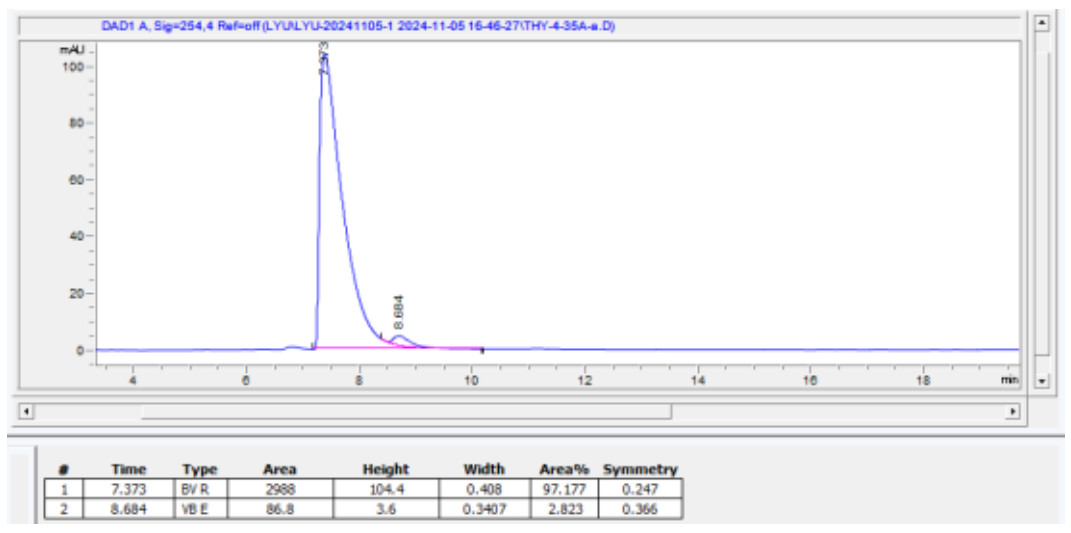

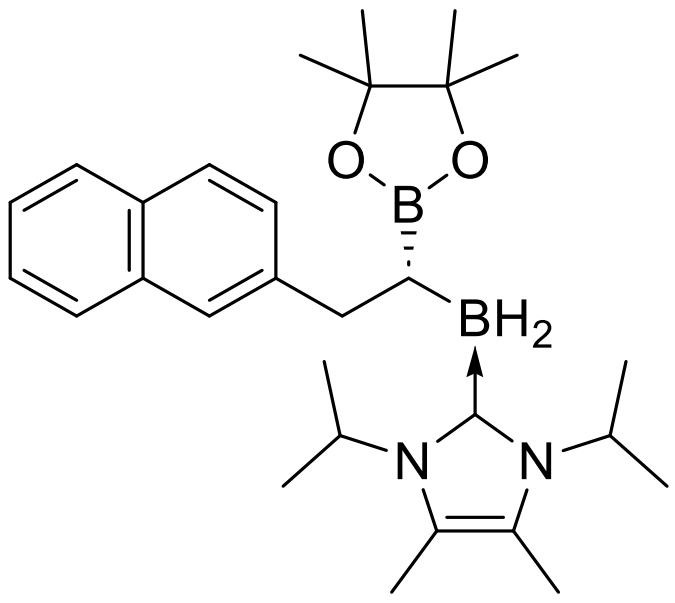

3l

3l: Yield: 27.5 mg, 58%, 98% ee, white solid, m.p. = 148 – 151 °C, R_f = 0.4 (*n*-hexane/ethyl acetate = 3/1).

^{1}H NMR (400 MHz, $CDCl_3$) δ 7.86 – 7.56 (m, 4H), 7.43 – 7.28 (m, 3H), 5.76 – 5.55 (m, 2H), 2.96 (t, *J* = 13.2 Hz, 1H), 2.50 – 2.32 (m, 1H), 2.24 (s, 6H), 1.52 – 1.42 (m, 12H), 1.18 – 1.06 (m, 12H), 0.63 – 0.52 (m, 1H).
^{13}C NMR (101 MHz, $CDCl_3$) δ 145.9, 133.8, 131.7, 128.3, 127.5, 127.4, 127.0, 125.6, 125.3, 124.3, 123.9, 81.6, 49.7, 39.4, 25.1, 24.6, 21.9, 21.6, 10.7.
^{11}B NMR (128 MHz, $CDCl_3$) δ 35.9 (s, 1B), -25.8 (t, *J* = 85.9 Hz, 1B).
Note: After column chromatography, preparative thin-layer chromatography (PTLC) was employed to remove trace amounts of impurities to ensure the highest purity for the NMR spectra.
HRMS (ESI): Calcd for $C_{29}H_{44}B_2N_2O_2Na^+$ [M+Na]$^+$: 497.3481, found: 497.3493.
IR (neat, cm^{-1}): *v* 2972, 2934, 2301, 1320, 1303, 1144, 1031.
Chiral HPLC (IC, ^{i}PrOH/*n*-hexane = 1/99, 1.0 mL/min) t_R 38.1 min (major), 53.7 min (minor).
$[\alpha]_D^{25}$= -52.00 (*c* = 0.5, $CHCl_3$).

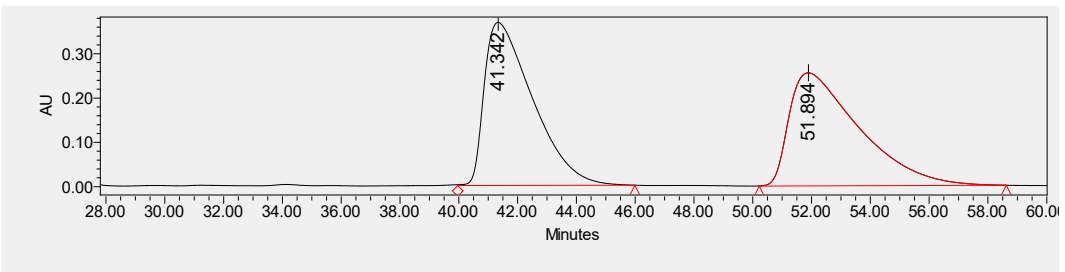

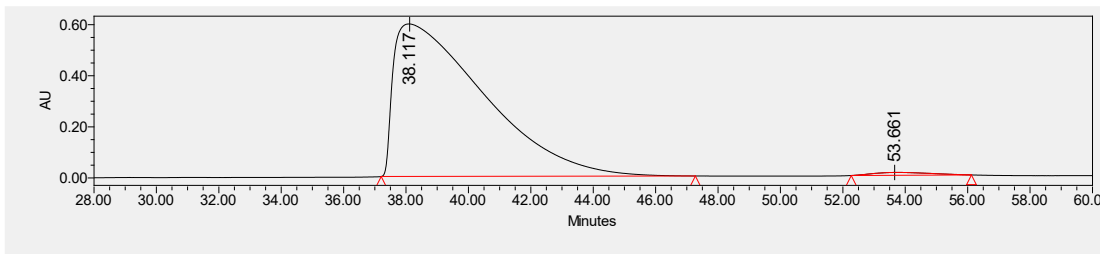

	Retention Time	Area	% Area
1	41.342	42150999	50.00
2	51.894	42158538	50.00

	Retention Time	Area	% Area
1	38.117	120764868	98.82
2	53.661	1435897	1.18

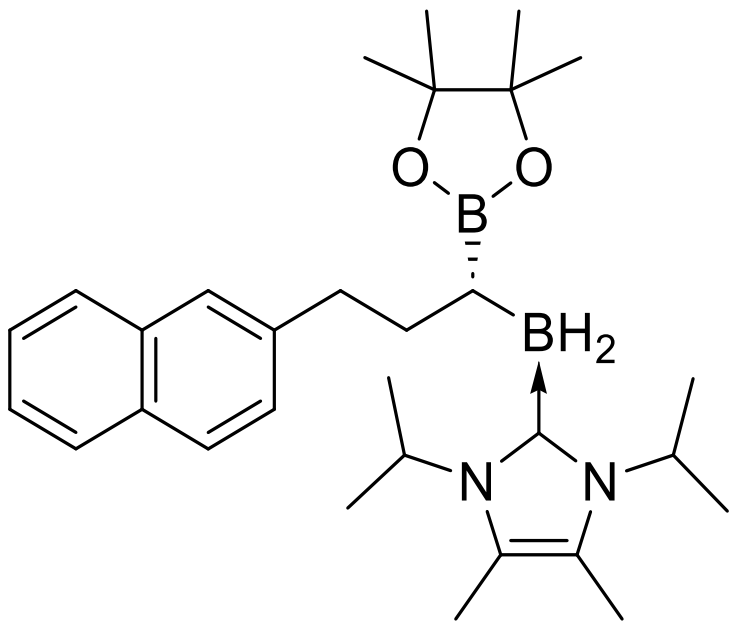

3m

3m: Yield: 29.8 mg, 61%, 99% ee, white solid, m.p. = 107 – 112 °C, R_f = 0.4 (*n*-hexane/ethyl acetate = 3/1).
^{1}H NMR (400 MHz, $CDCl_3$) δ 7.80 – 7.62 (m, 3H), 7.54 (s, 1H), 7.42 – 7.28 (m, 3H), 5.63 – 5.31 (m, 2H), 2.96 – 2.75 (m, 1H), 2.66 – 2.44 (m, 1H), 2.16 (s, 6H), 2.07 – 1.88 (m, 1H), 1.38 – 1.33 (m, 6H), 1.33 – 1.27 (m, 19H), 0.37 – 0.17 (m, 1H).
^{13}C NMR (101 MHz, $CDCl_3$) δ 142.3, 133.7, 131.8, 128.1, 127.6, 127.4, 127.2, 126.2, 125.5, 124.6, 123.7, 81.5, 49.5, 40.6, 35.2, 25.3, 24.6, 21.6, 21.5, 10.6.
^{11}B NMR (128 MHz, $CDCl_3$) δ 36.5 (s, 1B), -25.9 (t, *J* = 83.4 Hz, 1B).
Note: After column chromatography, preparative thin-layer chromatography (PTLC) was employed to remove trace amounts of impurities to ensure the highest purity for the NMR spectra.
HRMS (ESI): Calcd for $C_{30}H_{46}B_2N_2O_2Na^+$ [M+Na]$^+$: 511.3638, found: 511.3650.
IR (neat, cm^{-1}): *v* 2972, 2923, 2281, 1398, 1261, 1146, 1099, 1020.

Chiral HPLC (IB-3, ^{i}PrOH/n-hexane = 1/99, 1.0 mL/min) t_R 9.5 min (major), 13.4 min (minor).
$[\alpha]_D^{25}$= -30.00 (c = 0.6, $CHCl_3$).

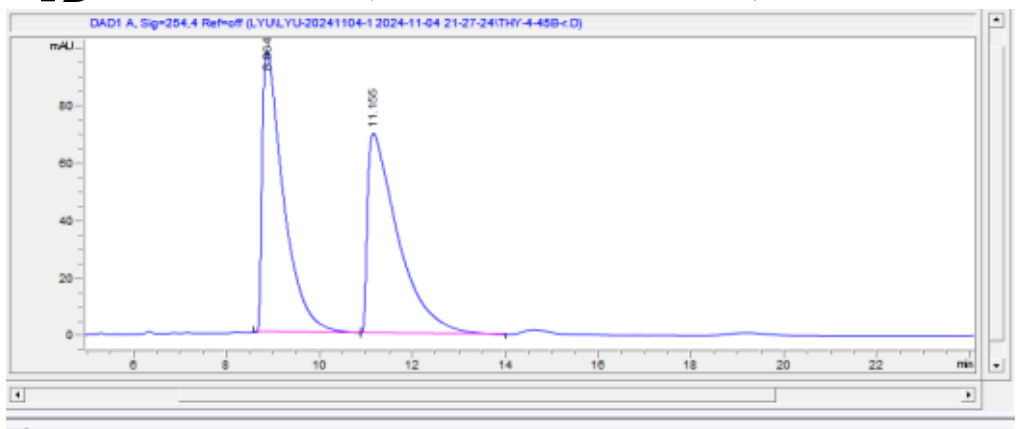

#	Time	Type	Area	Height	Width	Area%	Symmetry
1	8.864	BB	3108.7	97.7	0.4633	49.606	0.268
2	11.155	BB	3158.1	69.3	0.6174	50.394	0.215

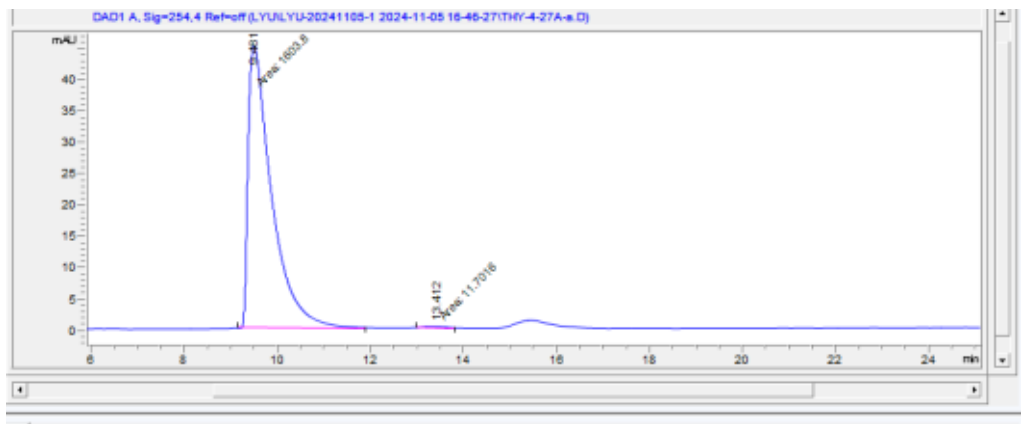

#	Time	Type	Area	Height	Width	Area%	Symmetry
1	9.481	MM	1603.8	45.3	0.5896	99.276	0.311
2	13.412	MM	11.7	3.1E-1	0.6362	0.724	0.637

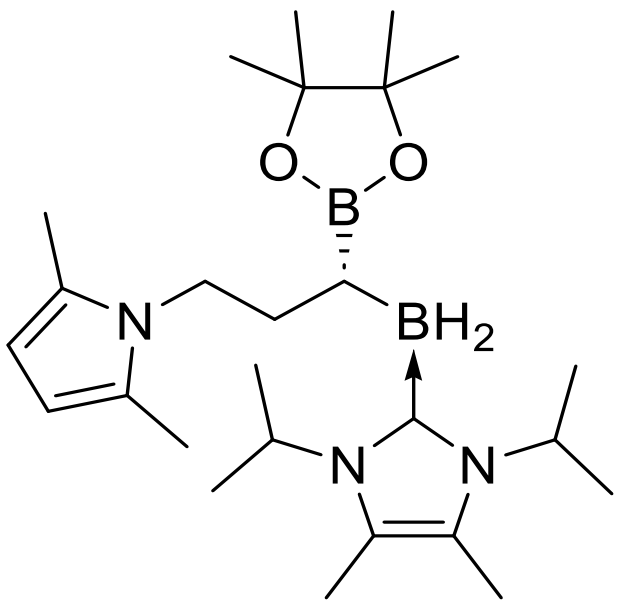

3n

3n: Yield: 29.6 mg, 65%, 94% ee, colorless oil, R_f = 0.4 (n-hexane/ethyl acetate = 3/1).
^{1}H NMR (500 MHz, $CDCl_3$) δ 5.66 (s, 2H), 5.58 – 5.40 (m, 2H), 3.88 – 3.62 (m, 1H), 3.62 – 3.22 (m, 1H), 2.19 (s, 6H), 2.16 (s, 6H), 1.81 – 1.69 (m, 1H), 1.45 – 1.30 (m, 12H), 1.30 – 1.19 (m, 12H), 1.15 – 1.07 (m, 1H), 0.10 – 0.06 (m, 1H).
^{13}C NMR (126 MHz, $CDCl_3$) δ 127.4, 123.8, 104.1, 81.7, 49.6, 47.5, 34.6, 25.3, 24.5, 21.7, 21.5, 12.6, 10.6.
^{11}B NMR (160 MHz, $CDCl_3$) δ 35.0 (s, 1B), -26.5 (t, J = 85.7 Hz, 1B).
Note: After column chromatography, preparative thin-layer chromatography (PTLC) was employed to remove trace amounts of impurities to ensure the highest purity for the NMR spectra.
HRMS (ESI): Calcd for $C_{26}H_{47}B_2N_3O_2Na^+$ $[M+Na]^+$: 478.3747, found: 478.3756.
IR (neat, cm^{-1}): ν 2958, 2924, 2262, 1660, 1556, 1452, 1380, 1060.
Chiral HPLC (IB-3, ^{i}PrOH/n-hexane = 1/99, 1.0 mL/min) t_R 6.8 min (major), 9.7 min (minor).
$[\alpha]_D^{25}$= -17.83 (c = 0.6, $CHCl_3$).

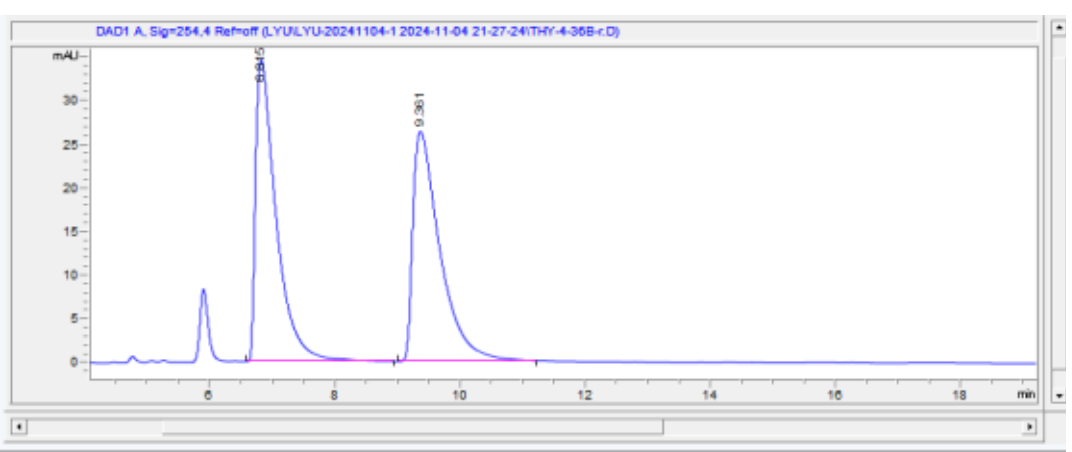

#	Time	Type	Area	Height	Width	Area%	Symmetry
1	6.815	BB	790.5	34.5	0.3373	50.074	0.336
2	9.361	BB	788.1	26.3	0.4363	49.926	0.358

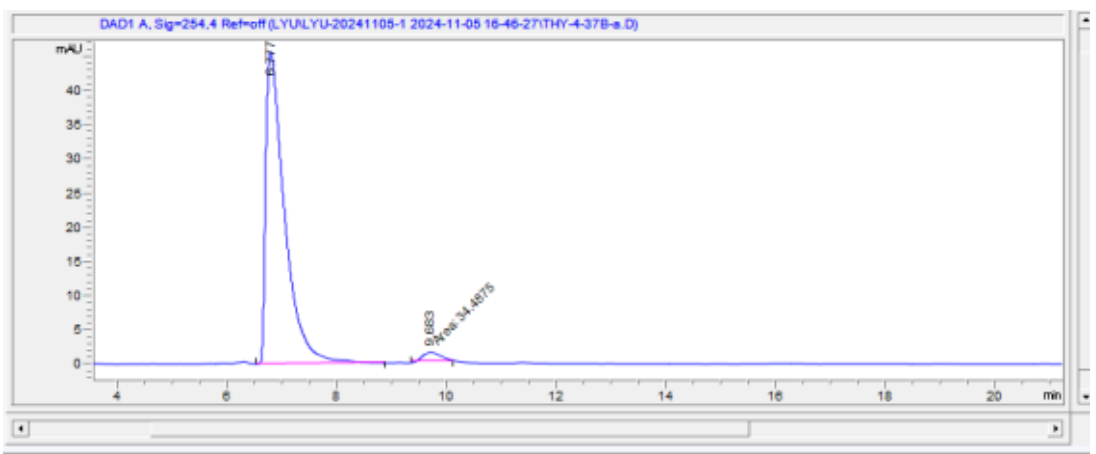

#	Time	Type	Area	Height	Width	Area%	Symmetry
1	6.777	BB	1086.3	45.5	0.3493	96.923	0.312
2	9.683	MM	34.5	1.4	0.4131	3.077	0.653

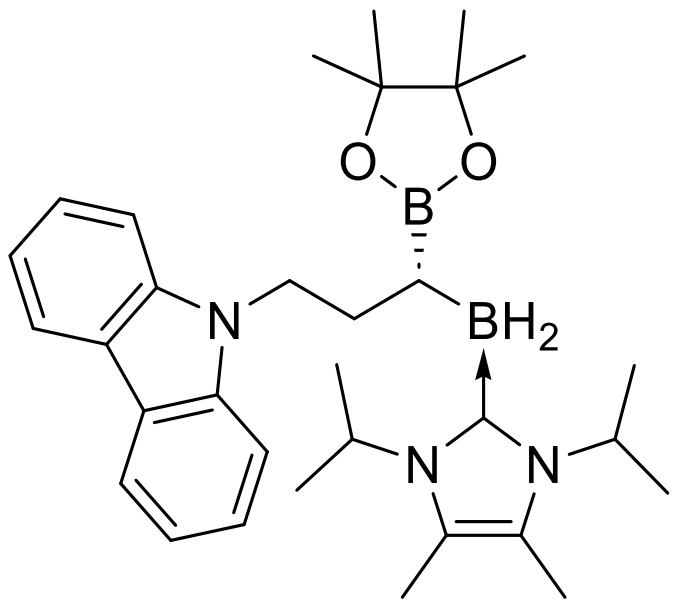

3o

3o: Yield: 45.8 mg, 87%, 96% ee, white solid, m.p. = 109 – 115 °C, R_f = 0.4 (*n*-hexane/ethyl acetate = 3/1).

^{1}H NMR (400 MHz, $CDCl_3$) δ 8.11 – 7.85 (m, 2H), 7.53 – 7.45 (m, 2H), 7.45 – 7.36 (m, 2H), 7.21 – 7.08 (m, 2H), 5.56 – 5.24 (m, 2H), 4.36 – 4.25 (m, 1H), 4.18 – 3.98 (m, 1H), 2.13 (s, 6H), 2.09 – 1.97 (m, 1H), 1.36 (s, 12H), 1.33 (d, *J* = 7.1 Hz, 6H), 1.30 – 1.18 (m, 7H), 0.34 – 0.16 (m, 1H).

^{13}C NMR (101 MHz, $CDCl_3$) δ 140.7, 125.3, 123.8, 122.6, 120.1, 118.2, 109.2, 81.8, 49.6, 46.8, 32.2, 25.4, 24.8, 21.6, 21.4, 10.5.

^{11}B NMR (128 MHz, $CDCl_3$) δ 36.5 (s, 1B), -26.2 (t, *J* = 85.5 Hz, 1B).

HRMS (ESI): Calcd for $C_{32}H_{47}B_2N_3O_2Na^+$ $[M+Na]^+$: 550.3747, found: 550.3761.

IR (neat, cm^{-1}): *v* 2958, 2800, 2273, 1381, 1122, 1094.

Chiral HPLC (OX-H, ^{i}PrOH/*n*-hexane = 1/99, 1.0 mL/min) t_R 43.6 min (major), 77.0 min (minor).

$[\alpha]_D^{25}$= -16.10 (*c* = 1.0, $CHCl_3$).

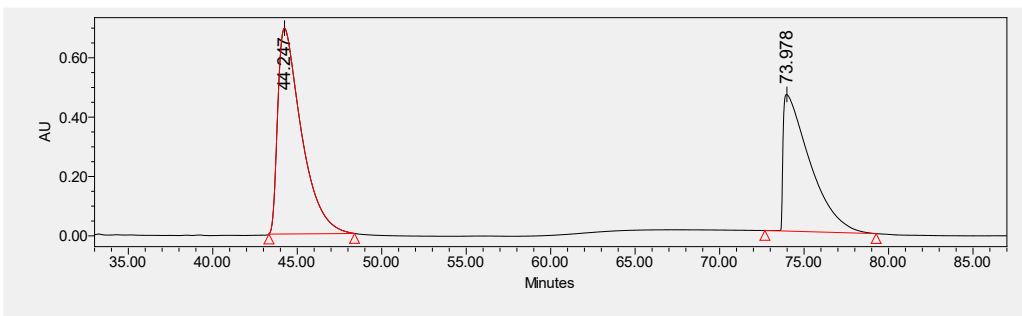

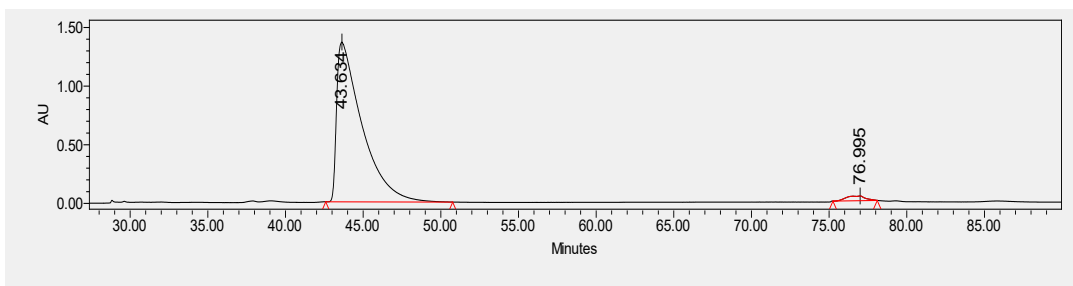

	Retention Time	Area	% Area
1	44.247	53922643	51.50
2	73.978	50786554	48.50

	Retention Time	Area	% Area
1	43.634	153645208	97.79
2	76.995	3470150	2.21

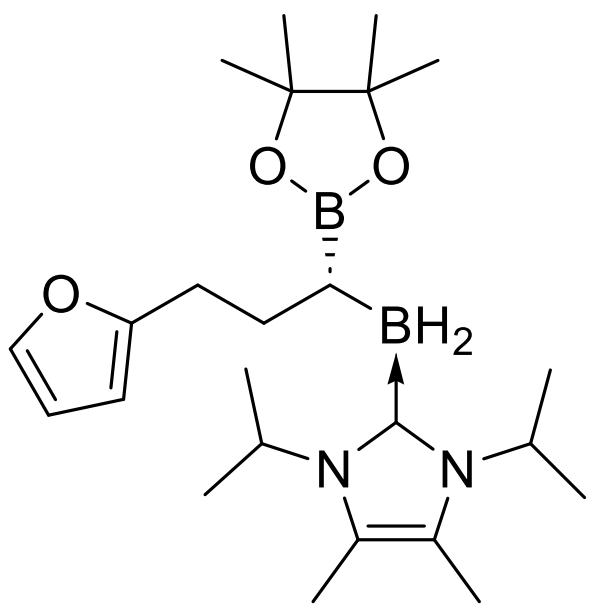

3p

3p: Yield: 19.7 mg, 46%, 97% ee, colorless oil, R_f = 0.4 (*n*-hexane/ethyl acetate = 3/1).

^{1}H NMR (400 MHz, $CDCl_3$) δ 7.21 – 7.10 (m, 1H), 6.39 – 6.14 (m, 1H), 5.99 – 5.80 (m, 1H), 5.64 – 5.43 (m, 2H), 2.74 – 2.56 (m, 1H), 2.49 – 2.30 (m, 1H), 2.20 (s, 6H),

1.92 – 1.75 (m, 1H), 1.51 – 1.32 (m, 12H), 1.32 – 1.24 (m, 12H), 1.21 – 1.12 (m, 1H), 0.32 – 0.14 (m, 1H).

^{13}C NMR (126 MHz, $CDCl_3$) δ 158.4, 140.0, 123.8, 110.0, 104.1, 81.5, 49.6, 31.4, 25.2, 24.6, 22.7, 21.7, 21.5, 10.6.

^{11}B NMR (160 MHz, $CDCl_3$) δ 36.2 (s, 1B), -26.2 (t, J = 85.7 Hz, 1B).

Note: After column chromatography, preparative thin-layer chromatography (PTLC) was employed to remove trace amounts of impurities to ensure the highest purity for the NMR spectra.

HRMS (ESI): Calcd for $C_{24}H_{42}B_2N_2O_3Na^+$ $[M+Na]^+$: 451.3274, found: 451.3281.

IR (neat, cm^{-1}): v 2931, 2042, 1345, 1305, 1230, 1085, 1064.

Chiral HPLC (IC, ^{i}PrOH/n-hexane = 1/99, 1.0 mL/min) t_R 36.3 min (minor), 49.2 min (major).

$[\alpha]_D^{25}$ = -15.50 (c = 0.2, $CHCl_3$).

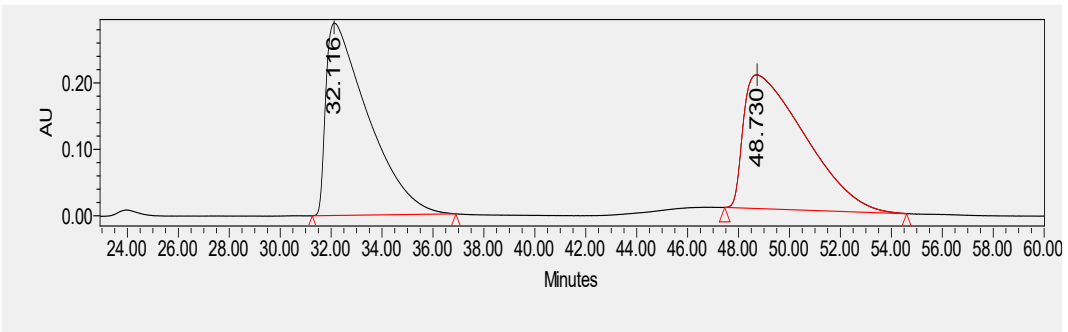

	Retention Time	Area	% Area
1	32.116	33377696	48.02
2	48.730	36123565	51.98

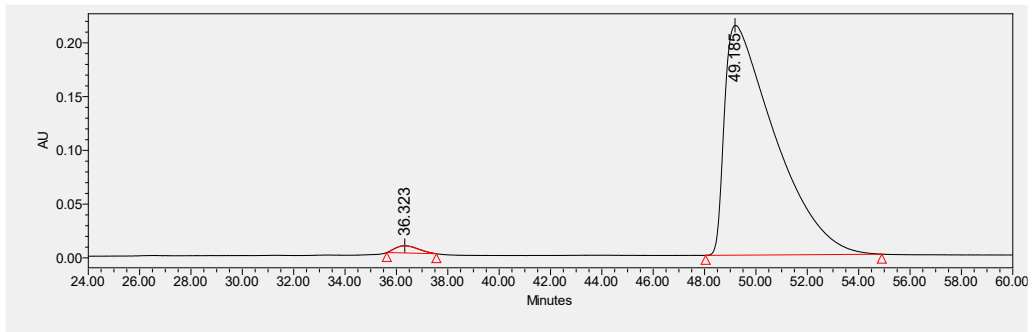

	Retention Time	Area	% Area
1	36.323	416563	1.41
2	49.185	29204738	98.59

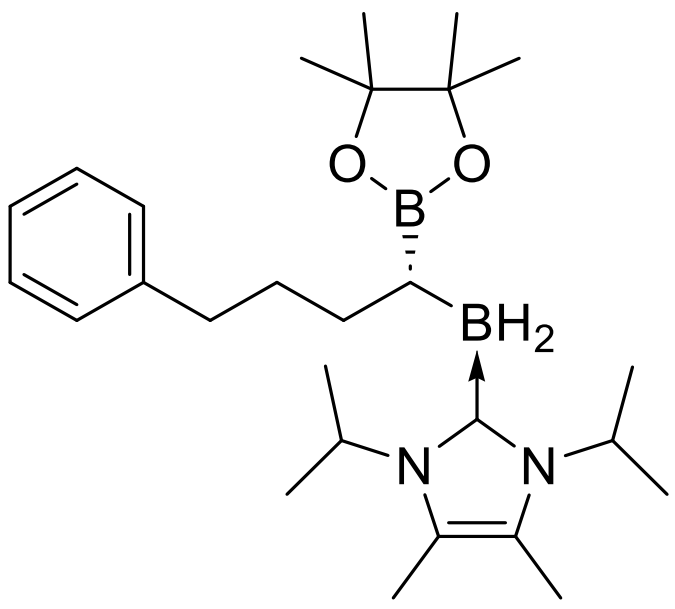

3q

3q: Yield: 30.3 mg, 67%, 98% ee, colorless oil, R_f = 0.4 (n-hexane/DCM = 1/1).

^{1}H NMR (400 MHz, $CDCl_3$) δ 7.22 – 7.04 (m, 5H), 5.65 – 5.28 (m, 2H), 2.52 (t, J = 7.8 Hz, 2H), 2.20 (s, 6H), 1.70 – 1.49 (m, 3H), 1.43 – 1.33 (m, 12H), 1.33 – 1.18 (m, 12H), 1.03 – 0.83 (m, 1H), 0.15 (m, 1H).

^{13}C NMR (126 MHz, $CDCl_3$) δ 144.0, 128.5, 128.1, 125.2, 123.7, 81.5, 49.5, 36.5, 35.9, 33.1, 25.3, 24.6, 21.8, 21.5, 10.6.

^{11}B NMR (160 MHz, $CDCl_3$) δ 36.3 (s, 1B), -26.1 (t, J = 84.6 Hz, 1B).

Note: After column chromatography, preparative thin-layer chromatography (PTLC) was employed to remove trace amounts of impurities to ensure the highest purity for the NMR spectra.

HRMS (ESI): Calcd for $C_{27}H_{46}B_2N_2O_2Na^+$ $[M+Na]^+$: 475.3638, found: 475.3647.

IR (neat, cm^{-1}): v 2973, 2923, 2293, 1398, 1263, 1147, 1012.

Chiral HPLC (IC, ^{i}PrOH/n-hexane = 1/99, 1.0 mL/min) t_R 31.3 min (major), 39.6 min (minor).

$[\alpha]_D^{25}$= -5.70 (c = 1.0, $CHCl_3$).

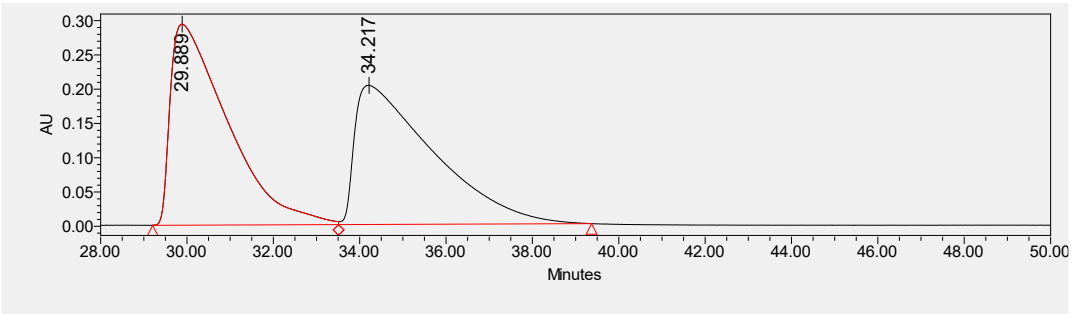

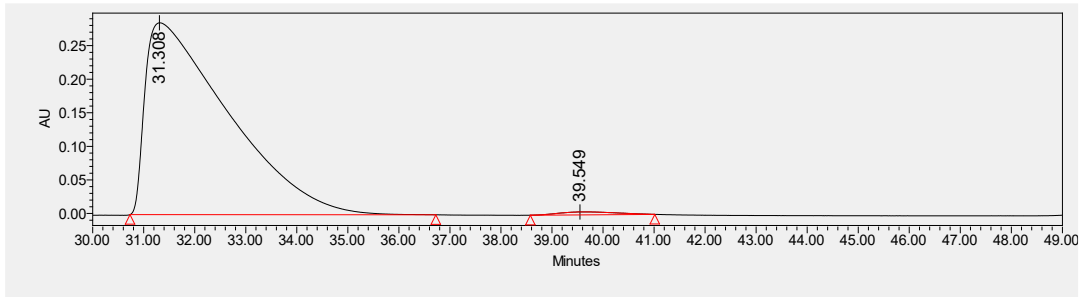

	Retention Time	Area	% Area
1	29.889	26995580	51.25
2	34.217	25682854	48.75

	Retention Time	Area	% Area
1	31.308	98.95	32352242
2	39.549	1.05	344119

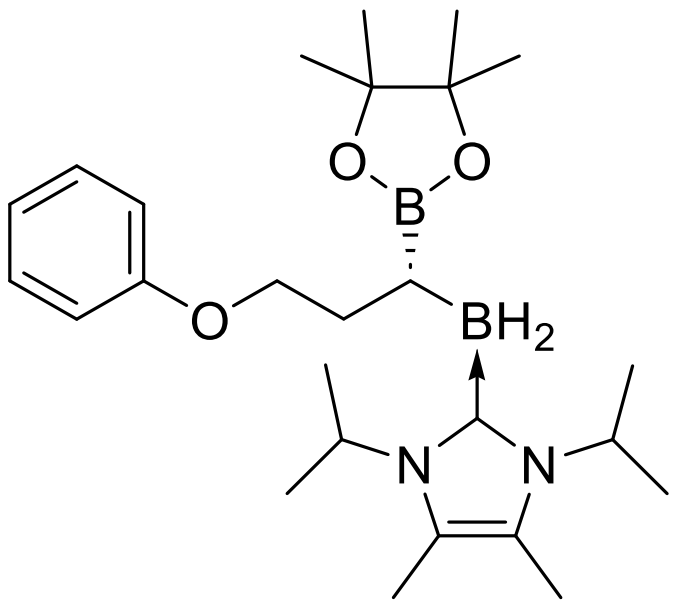

3r

3r: Yield: 41.3 mg, 91%, 97% ee, colorless oil, R_f = 0.4 (n-hexane/ethyl acetate = 3/1).
^{1}H NMR (500 MHz, $CDCl_3$) δ 7.24 – 7.18 (m, 2H), 6.94 – 6.80 (m, 3H), 5.68 – 5.37 (m, 2H), 4.06 – 3.89 (m, 1H), 3.83 – 3.63 (m, 1H), 2.20 (s, 6H), 1.97 – 1.90 (m, 1H), 1.49 – 1.36 (m, 12H), 1.35 – 1.13 (m, 13H), 0.27 – 0.16 (m, 1H).
^{13}C NMR (126 MHz, $CDCl_3$) δ 159.6, 129.3, 123.9, 119.9, 114.7, 81.7, 71.4, 49.6, 32.2, 25.2, 24.5, 21.8, 21.5, 10.6.
^{11}B NMR (160 MHz, $CDCl_3$) δ 36.2 (s, 1B), -26.1 (t, J = 85.1 Hz, 1B).
Note: After column chromatography, preparative thin-layer chromatography (PTLC) was employed to remove trace amounts of impurities to ensure the highest purity for the NMR spectra.
HRMS (ESI): Calcd for $C_{26}H_{44}B_2N_2O_3Na^+$ $[M+Na]^+$: 477.3430, found: 477.3443.
IR (neat, cm^{-1}): v 2972, 2925, 2302, 1599, 1399, 1261, 1146, 1078, 1011.
Chiral HPLC (OX-H, ^{i}PrOH/n-hexane = 2/98, 1.0 mL/min) t_R 17.2 min (minor), 20.8 min (major).
$[\alpha]_D^{25}$= -10.86 (c = 0.7, $CHCl_3$).

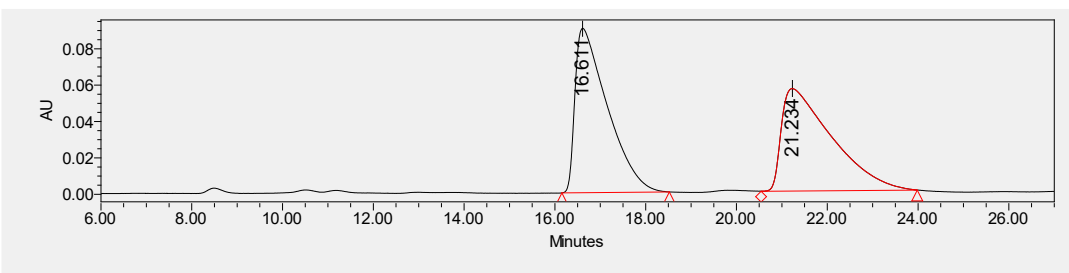

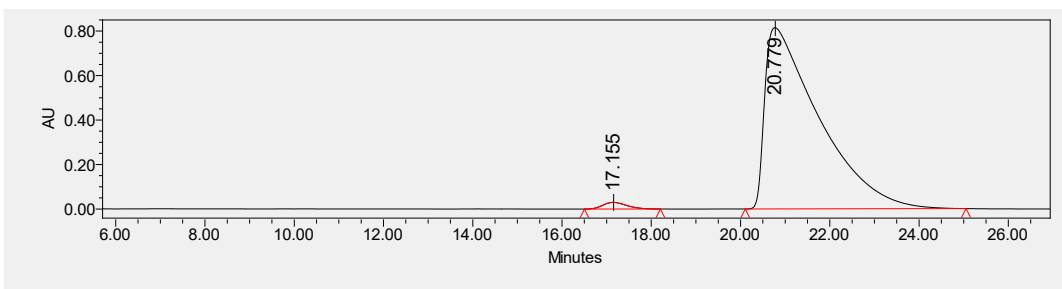

	Retention Time	Area	% Area
1	16.611	4471332	50.76
2	21.234	4337907	49.24

	Retention Time	Area	% Area
1	17.155	1110892	1.59
2	20.779	68784461	98.41

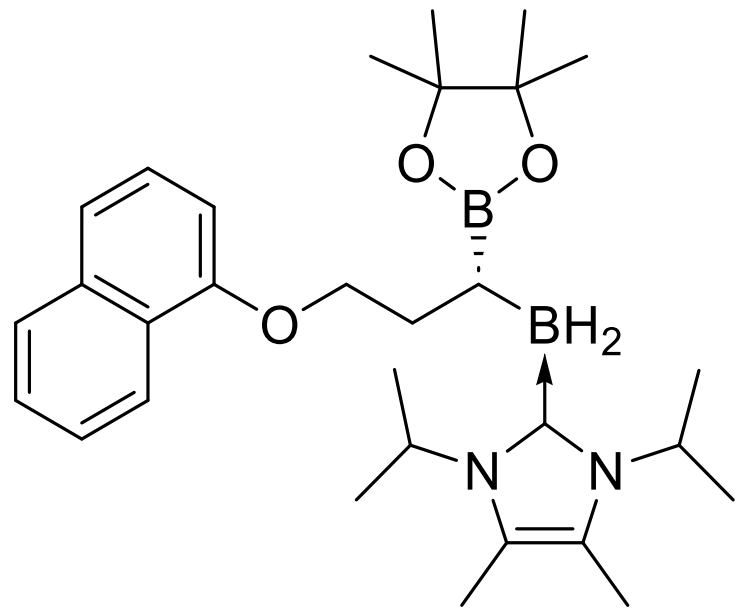

3s

3s: Yield: 32.2 mg, 64%, 94% ee, white solid, decomposed at 170 °C, R_f = 0.4 (*n*-hexane/ethyl acetate = 3/1).

^{1}H NMR (400 MHz, $CDCl_3$) δ 8.25 – 8.06 (m, 1H), 7.84 – 7.59 (m, 1H), 7.50 – 7.29 (m, 4H), 6.97 – 6.67 (m, 1H), 5.73 – 5.43 (m, 2H), 4.23 – 4.04 (m, 1H), 3.97 – 3.74 (m, 1H), 2.21 (s, 6H), 2.16 – 2.01 (m, 1H), 1.48 – 1.39 (m, 12H), 1.31 – 1.20 (m, 13H), 0.56 – 0.17 (m, 1H).

^{13}C NMR (101 MHz, $CDCl_3$) δ 155.4, 134.5, 127.4, 126.2, 126.1, 126.0, 124.7, 123.9, 122.4, 119.3, 104.9, 81.7, 71.8, 49.7, 32.3, 25.2, 24.6, 21.8, 21.5, 10.6.

^{11}B NMR (128 MHz, $CDCl_3$) δ 36.3 (s, 1B), -25.9 (t, *J* = 85.2 Hz, 1B).

HRMS (ESI): Calcd for $C_{30}H_{46}B_2N_2O_3Na^+$ $[M+Na]^+$: 527.3587, found: 527.3603.

IR (neat, cm^{-1}): *v* 2973, 2929, 2294, 1400, 1367, 1264, 1096, 1017.

Chiral HPLC (IB, ^{i}PrOH/*n*-hexane = 2/98, 1.0 mL/min) t_R 17.0 min (major), 21.2 min (minor).

$[\alpha]_D^{25}$ = -44.58 (*c* = 0.2, $CHCl_3$).

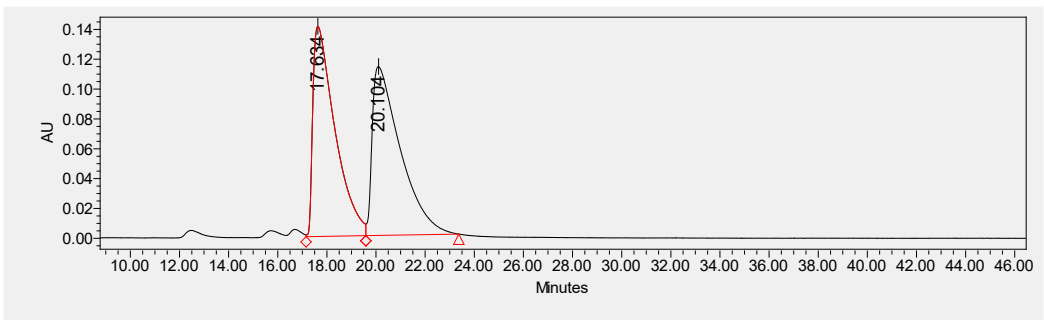

	Retention Time	Area	% Area
1	17.634	8784339	49.34
2	20.104	9019317	50.66

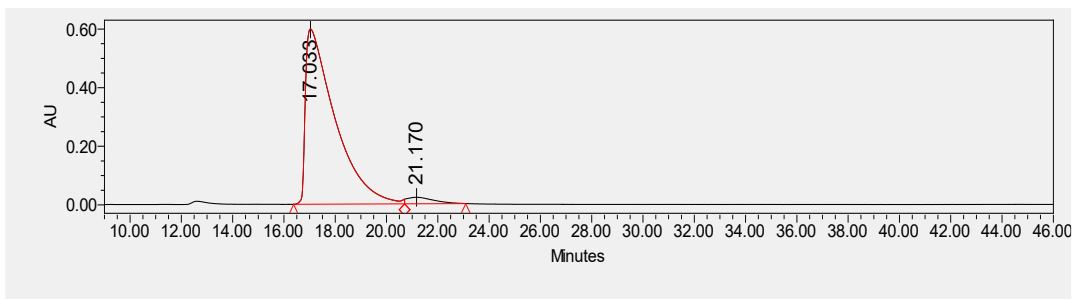

	Retention Time	Area	% Area
1	17.033	47831367	96.77
2	21.170	1598954	3.23

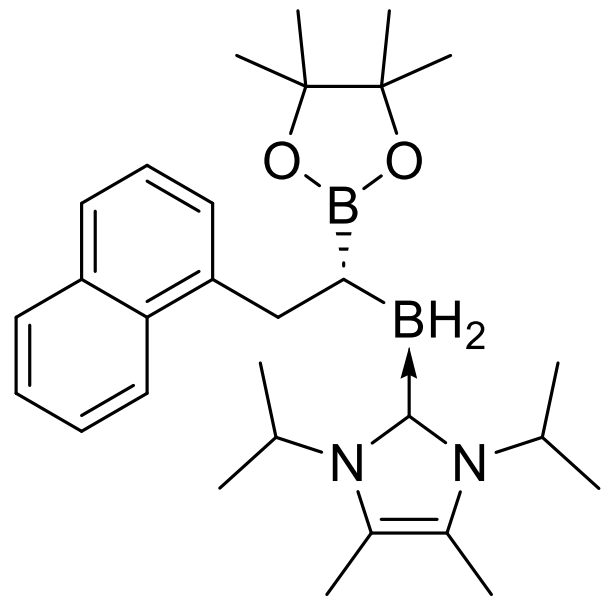

3t

3t: Yield: 37.0 mg, 78%, >99% ee, white solid, m.p. = 132 – 134 °C, R_f = 0.4 (*n*-hexane/ethyl acetate = 3/1).

^{1}H NMR (500 MHz, $CDCl_3$) δ 7.84 – 7.74 (m, 2H), 7.62 – 7.55 (m, 1H), 7.54 – 7.45 (m, 1H), 7.39 – 7.27 (m, 3H), 5.73 – 5.49 (m, 2H), 3.44 – 3.10 (m, 1H), 2.99 – 2.74 (m, 1H), 2.26 (s, 6H), 1.58 – 1.37 (m, 12H), 1.24 – 1.04 (m, 12H), 0.69 – 0.53 (m, 1H).

^{13}C NMR (126 MHz, $CDCl_3$) δ 144.0, 133.8, 132.2, 128.5, 125.6, 125.3, 125.2, 124.8, 124.7, 124.6, 124.0, 81.6, 49.8, 35.5, 25.0, 24.5, 21.8, 21.7, 10.7.

^{11}B NMR (160 MHz, $CDCl_3$) δ 35.9 (s, 1B), -26.0 (t, *J* = 85.2 Hz, 1B).

Note: After column chromatography, preparative thin-layer chromatography (PTLC) was employed to remove trace amounts of impurities to ensure the highest purity for the NMR spectra.

HRMS (ESI): Calcd for $C_{29}H_{44}B_2N_2O_2Na^+$ $[M+Na]^+$: 497.3481, found: 497.3489.

IR (neat, cm^{-1}): *ν* 2975, 2934, 2301, 1316, 1303, 1218, 1145, 1032.

Chiral HPLC (IB, ^{i}PrOH/*n*-hexane = 1/99, 1.0 mL/min) t_R 12.0 min (major), 14.1 min (minor).

$[\alpha]_D^{25}$= -35.91 (*c* = 0.2, $CHCl_3$).

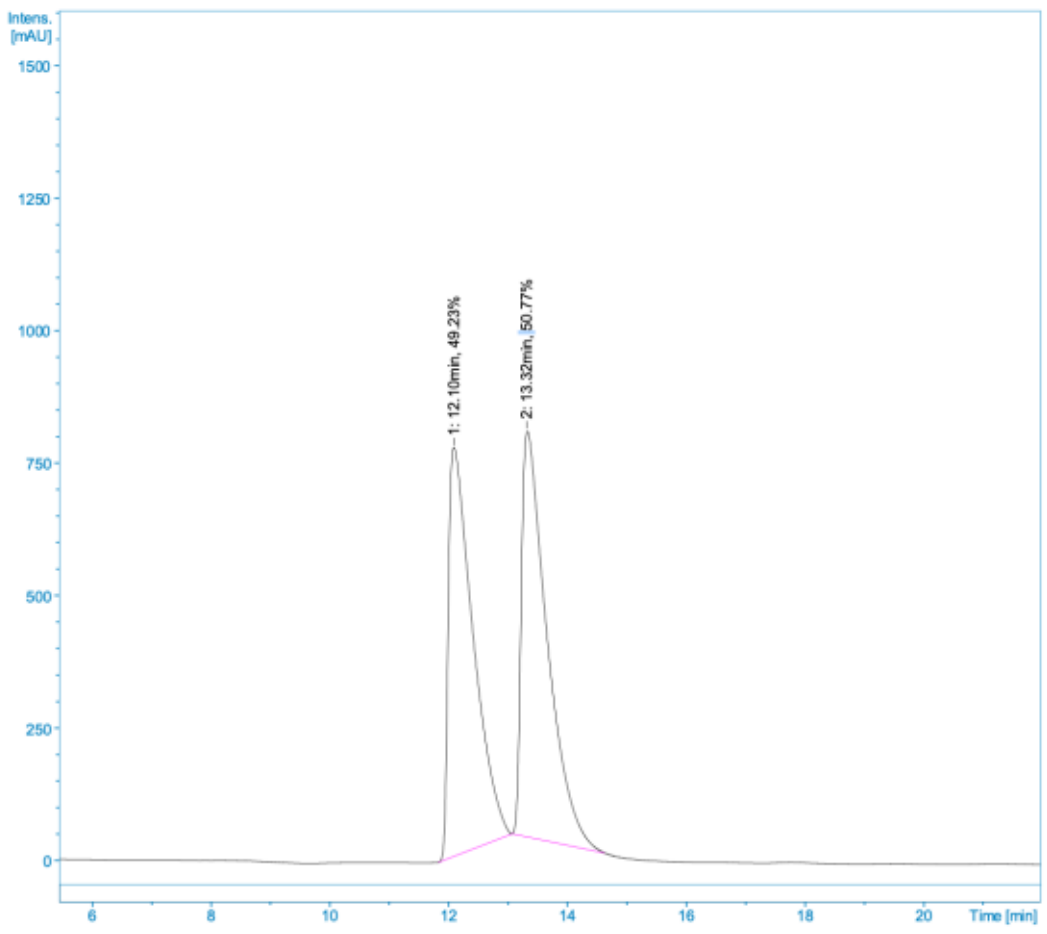

Peak	RT [min]	Peak Height [mAU]	Integral [%]	Peak Area [mAU*s]	FWHM [min]	Integral Region [min]	Ar
1	12.04	769.99	49.23	111367.8	0.4571	11.83 - 13.07	
2	13.26	765.05	50.77	114861.6	0.4408	13.07 - 14.63	

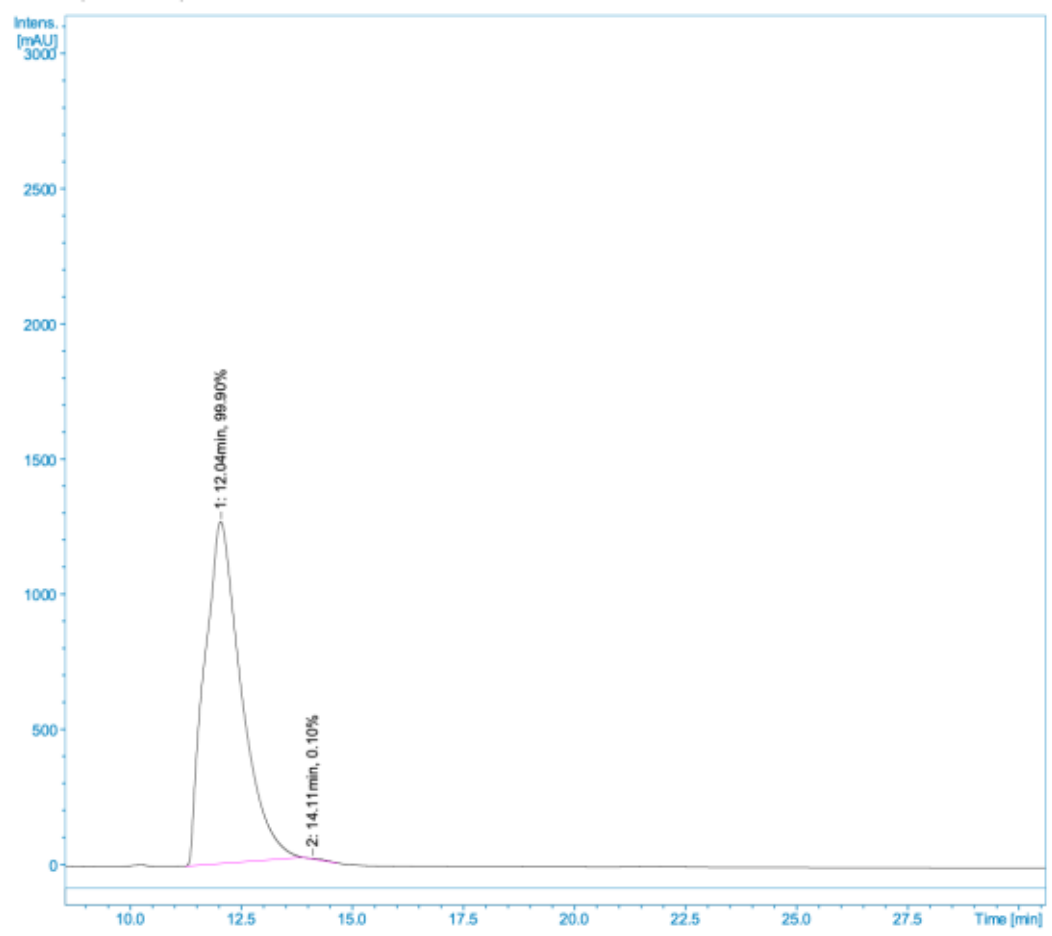

Peak	RT [min]	Peak Height [mAU]	Integral [%]	Peak Area [mAU*s]	FWHM [min]	Integral Region [min]	An
1	11.98	1264.03	99.90	361231.0	0.9094	11.24 - 13.87	
2	14.05	1.77	0.10	373.5	14.1176	13.87 - 14.67	

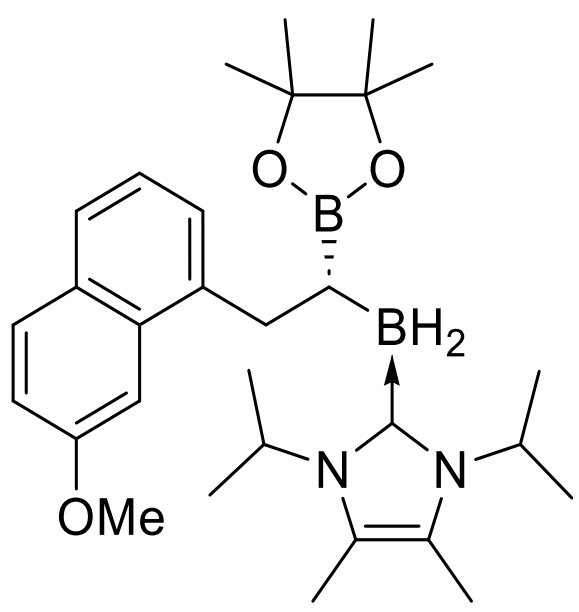

3u

3u: Yield: 37.8 mg, 75%, >99% ee, light yellow solid, m.p. = 148 – 157 °C, R_f = 0.4 (*n*-hexane/ethyl acetate = 3/1).

^{1}H NMR (400 MHz, $CDCl_3$) δ 7.80 – 7.60 (m, 1H), 7.57 – 7.40 (m, 2H), 7.25 – 7.16 (m, 2H), 7.12 – 7.00 (m, 1H), 5.69 – 5.60 (m, 2H), 3.77 (s, 3H), 3.32 – 3.16 (m, 1H), 2.89 – 2.62 (m, 1H), 2.22 (s, 6H), 1.57 – 1.29 (m, 12H), 1.32 – 0.95 (m, 12H), 0.63 – 0.56 (m, 1H).

^{13}C NMR (101 MHz, $CDCl_3$) δ 156.9, 142.4, 133.3, 130.0, 129.2, 125.3, 124.9, 123.9, 123.4, 117.1, 103.5, 81.6, 55.1, 49.8, 35.4, 25.0, 24.6, 21.8, 21.7, 10.6.

^{11}B NMR (128 MHz, $CDCl_3$) δ 36.6 (s, 1B), -25.9 (t, *J* = 86.2 Hz, 1B).

Note: After column chromatography, preparative thin-layer chromatography (PTLC) was employed to remove trace amounts of impurities to ensure the highest purity for the NMR spectra.
HRMS (ESI): Calcd for $C_{30}H_{46}B_2N_2O_3Na^+$ $[M+Na]^+$: 527.3587, found: 527.3594.
IR (neat, cm^{-1}): *v* 2931, 2854, 2303, 1485, 1387, 1250, 1187, 1100, 1023.
Chiral HPLC (OX-H, ^{i}PrOH/*n*-hexane = 2/98, 1.0 mL/min) t_R 47.3 min (major), 50.6 min (minor).
$[\alpha]_D^{25}$= -16.50 (*c* = 0.8, $CHCl_3$).

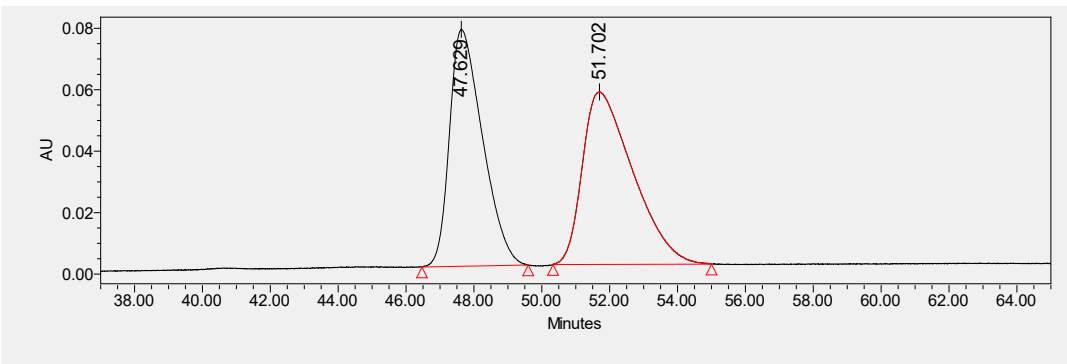

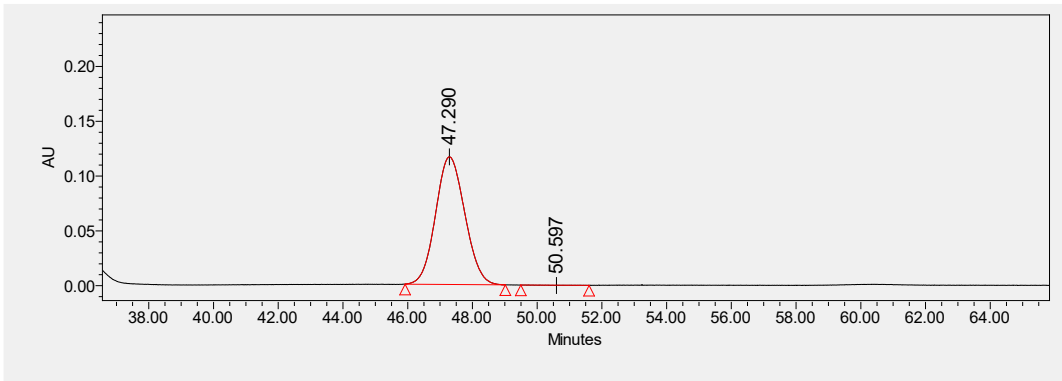

	Retention Time	Area	% Area
1	47.629	5208063	47.36
2	51.702	5789818	52.64

	Retention Time	% Area	Height
1	47.290	4964376	99.91
2	50.597	4296	0.09

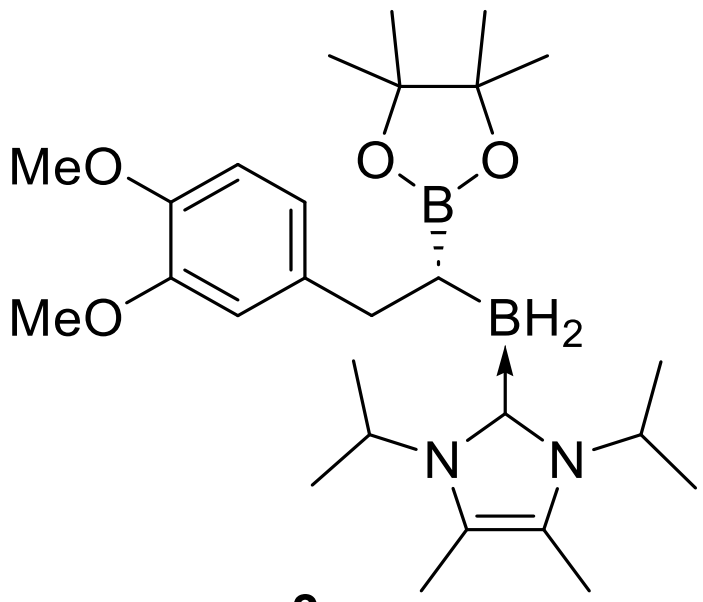

3v

3v: Yield: 30.5 mg, 63%, 94% ee, colorless oil, R_f = 0.4 (*n*-hexane/ethyl acetate = 3/1).
^{1}H NMR (500 MHz, $CDCl_3$) δ 6.91 – 6.72 (m, 1H), 6.73 – 6.12 (m, 2H), 5.77 – 5.24 (m, 2H), 4.20 – 3.55 (m, 6H), 2.81 – 2.62 (m, 1H), 2.37 – 1.98 (m, 7H), 1.67 – 1.29 (m, 12H), 1.27 – 1.00 (m, 12H), 0.59 – 0.42 (m, 1H).
^{13}C NMR (126 MHz, $CDCl_3$) δ 148.2, 146.2, 141.2, 123.9, 120.0, 112.0, 111.0, 81.5, 56.1, 55.7, 49.6, 38.7, 25.0, 24.7, 21.8, 21.6, 10.6.
^{11}B NMR (160 MHz, $CDCl_3$) δ 37.1(s, 1B), -26.0 (t, *J* = 85.6 Hz, 1B).
HRMS (ESI): Calcd for $C_{27}H_{46}B_2N_2O_4Na^+$ $[M+Na]^+$: 507.3536, found: 507.3545.
IR (neat, cm^{-1}): *v* 2952, 2270, 1425, 1381, 1258, 1191, 1013.
Chiral HPLC (OD-H, ^{i}PrOH/*n*-hexane = 20/80, 1.0 mL/min) t_R 5.7 min (minor), 7.6 min (major).
$[\alpha]_D^{25}$= 10.14 (*c* = 0.7, $CHCl_3$).

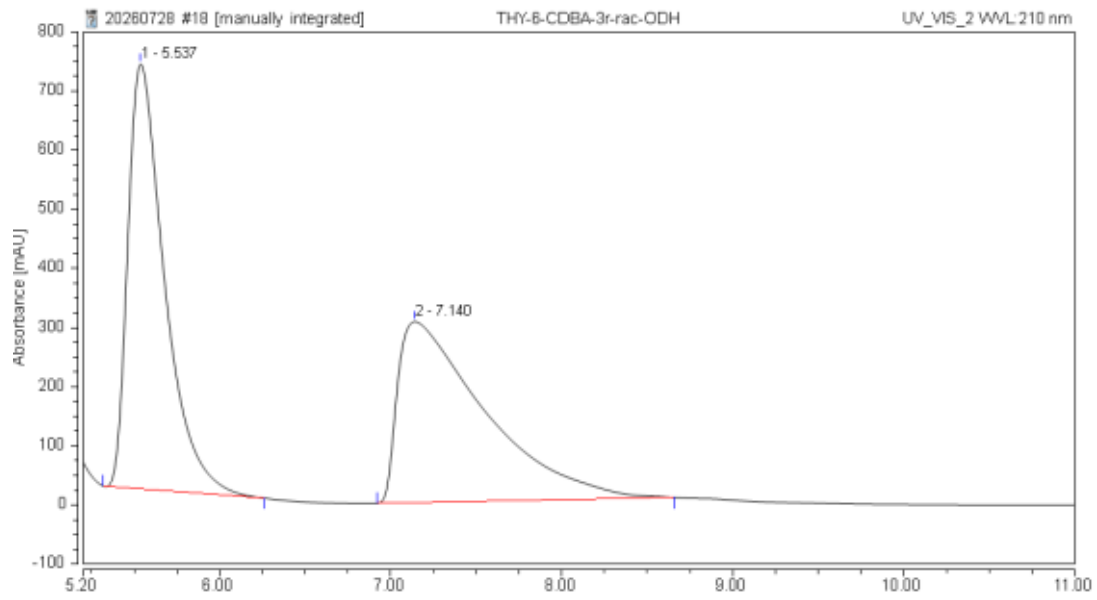

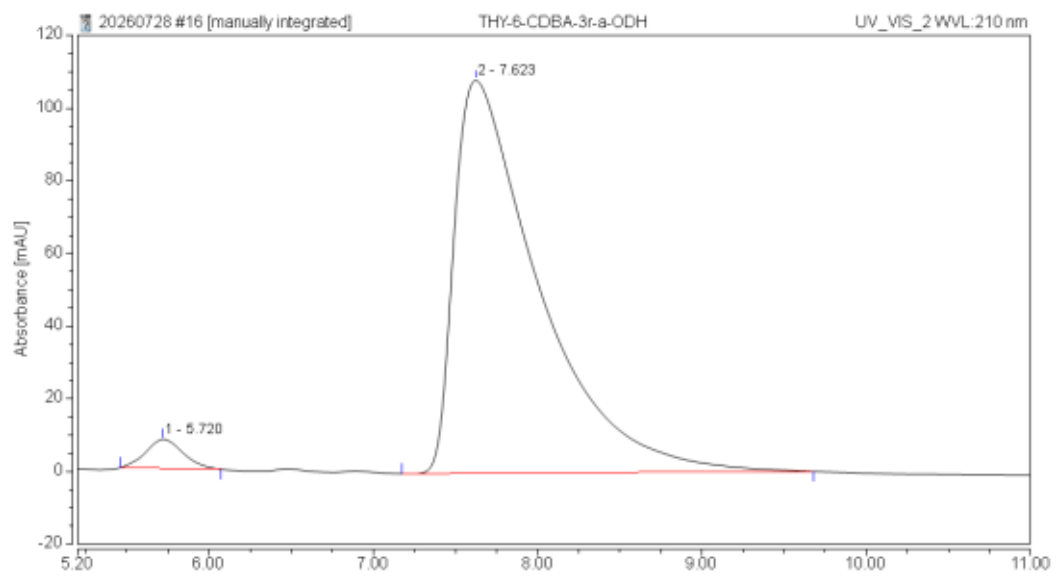

	Retention Time	Area	% Area
1	5.690	180.1835	49.56
2	7.393	183.3494	50.44

	Retention Time	Area	% Area
1	5.720	2.1115	3.17
2	7.623	64.601	96.83

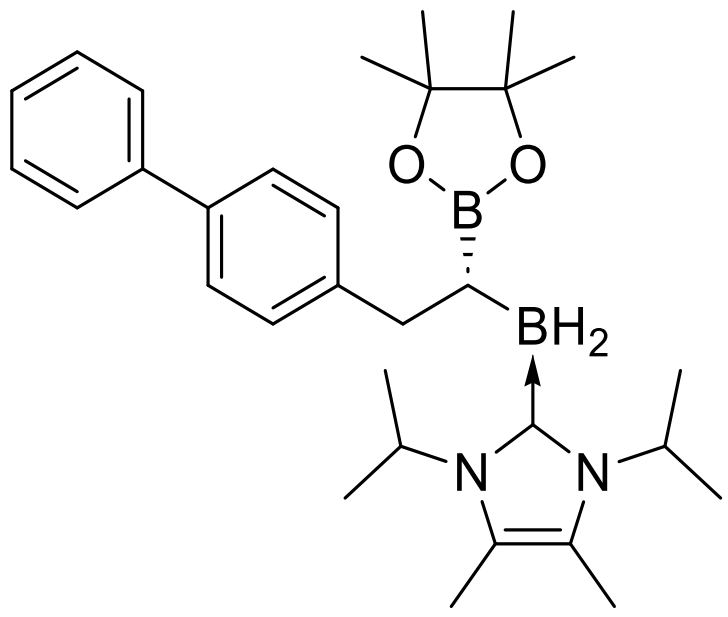

3w

3w: Yield: 28.0 mg, 56%, 97% ee, white solid, decomposed at 164 °C, R_f = 0.4 (*n*-hexane/ethyl acetate = 3/1).

^{1}H NMR (500 MHz, $CDCl_3$) δ 7.67 – 7.24 (m, 9H), 5.71 – 5.38 (m, 2H), 2.90 – 2.73 (m, 1H), 2.29 – 2.24 (m, 7H), 1.54 – 1.33 (m, 12H), 1.26 – 1.09 (m, 12H), 0.60 – 0.38 (m, 1H).

^{13}C NMR (126 MHz, $CDCl_3$) δ 147.6, 141.8, 137.3, 128.8, 128.7, 127.0, 126.6, 126.5, 123.9, 81.6, 49.7, 38.9, 25.1, 24.5, 22.5, 21.8, 21.6, 10.6.

^{11}B NMR (160 MHz, $CDCl_3$) δ 35.3 (s, 1B), -26.0 (t, *J* = 85.6 Hz, 1B).

Note: After column chromatography, preparative thin-layer chromatography (PTLC) was employed to remove trace amounts of impurities to ensure the highest purity for the NMR spectra.

HRMS (ESI): Calcd for $C_{31}H_{46}B_2N_2O_2Na^+$ $[M+Na]^+$: 523.3638, found: 523.3649.

IR (neat, cm^{-1}): *ν* 2972, 2312, 1440, 1398, 1259, 1222, 1141, 1089, 1021.

Chiral HPLC (IC, ^{i}PrOH/*n*-hexane = 2/98, 1.0 mL/min) t_R 20.7 min (minor), 25.4 min (major).

$[\alpha]_D^{25}$= 14.83 (*c* = 0.6, $CHCl_3$).

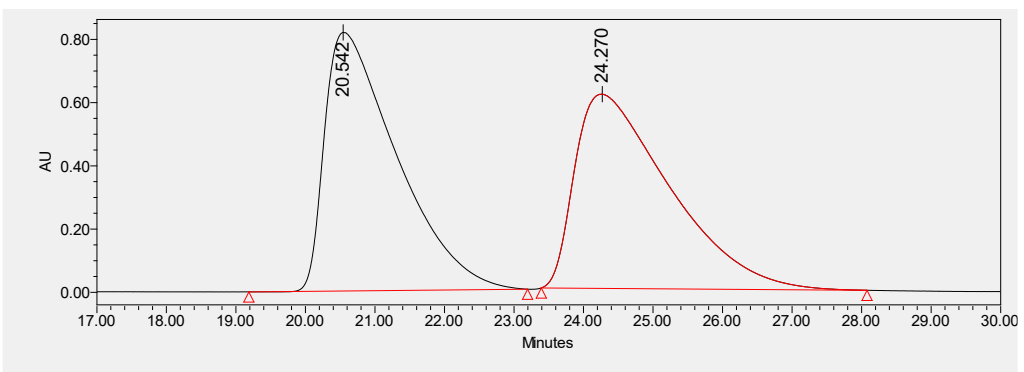

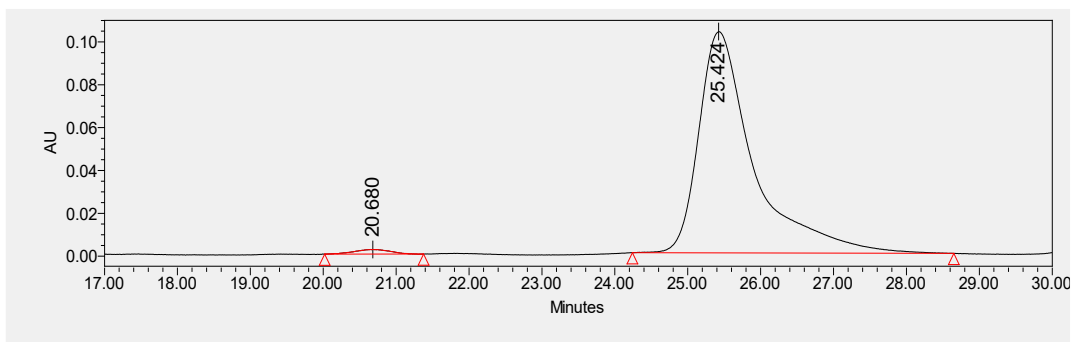

	Retention Time	Area	% Area
1	20.542	58865080	50.32
2	24.270	58120455	49.68

	Retention Time	Area	% Area
1	20.680	73693	1.35
2	25.424	5379537	98.65

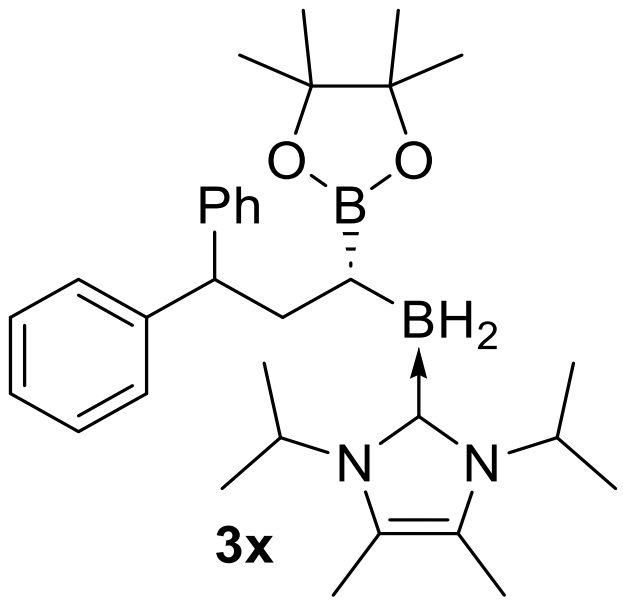

3x

3x: Yield: 36.5 mg, 71%, 96% ee, colorless oil, R_f = 0.4 (*n*-hexane/ethyl acetate = 3/1).
^{1}H NMR (500 MHz, $CDCl_3$) δ 7.24 – 6.96 (m, 10H), 5.52 – 5.27 (m, 2H), 3.90 – 3.77 (m, 1H), 2.26 – 2.18 (m, 7H), 1.63 – 1.52 (m, 1H), 1.36 (d, *J* = 7.1 Hz, 6H), 1.30 – 1.18 (m, 18H), 0.14 – 0.05 (m, 1H).
^{13}C NMR (126 MHz, $CDCl_3$) δ 147.7, 146.1, 128.7, 128.3, 128.0, 128.0, 125.4, 125.3, 123.7, 81.5, 54.8, 49.6, 39.7, 25.1, 24.7, 21.6, 21.5, 10.6.
^{11}B NMR (160 MHz, $CDCl_3$) δ 36.2 (s, 1B), -26.0 (t, *J* = 84.9 Hz, 1B).
Note: After column chromatography, preparative thin-layer chromatography (PTLC) was employed to remove trace amounts of impurities to ensure the highest purity for the NMR spectra.
HRMS (ESI): Calcd for $C_{32}H_{48}B_2N_2O_2Na^+$ $[M+Na]^+$: 537.3794, found: 537.3805.
IR (neat, cm^{-1}): *v* 2973, 2928, 2292, 1449, 1398, 1275, 1145, 1103, 1022.
Chiral HPLC (IC, ^{i}PrOH/*n*-hexane = 1/99, 1.0 mL/min) t_R 17.4 min (minor), 28.6 min (major).
$[\alpha]_D^{25}$ = -19.86 (*c* = 0.8, $CHCl_3$).

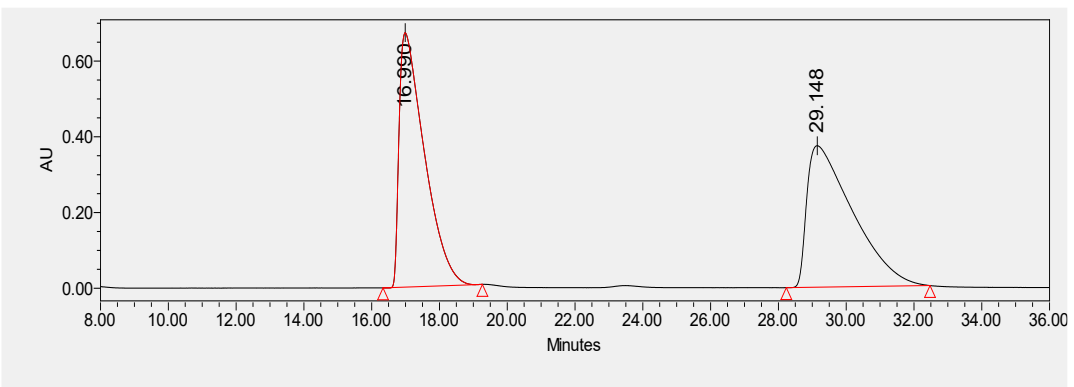

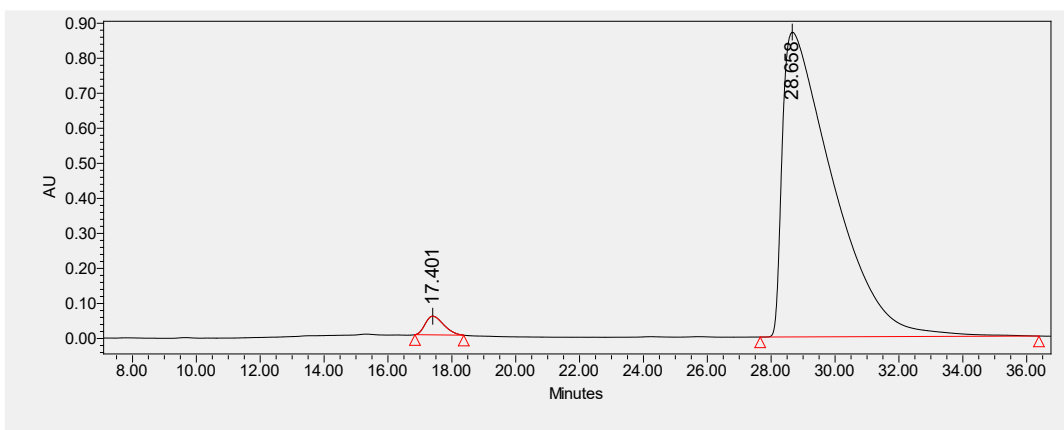

	Retention Time	Area	% Area
1	16.990	34733116	49.75
2	29.148	35087910	50.25

	Retention Time	Area	% Area
1	17.401	2143592	2.19
2	28.658	95916892	97.81

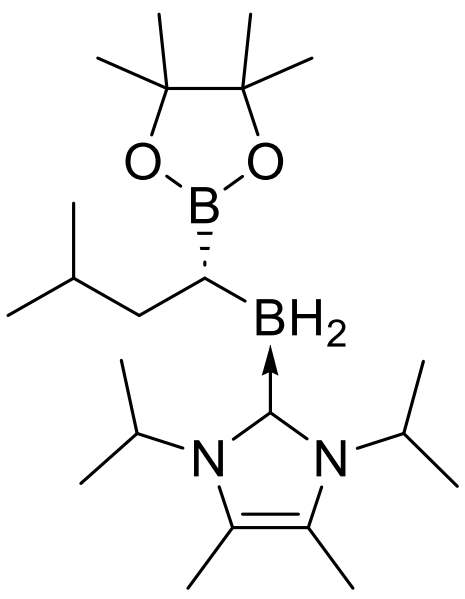

3y

3y: Yield: 21.1 mg, 54%, 97% ee, colorless oil, R_f = 0.4 (*n*-hexane/ethyl acetate = 3/1). Enantioselectivity was determined by the *in-situ* aminated product **7**.

¹H NMR (400 MHz, $CDCl_3$) δ 5.80 – 5.30 (m, 2H), 2.21 (s, 6H), 1.57 – 1.46 (m, 2H), 1.45 – 1.33 (m, 12H), 1.26 – 1.19 (m, 12H), 0.79 (d, *J* = 6.5 Hz, 3H), 0.73 (d, *J* = 6.5 Hz, 3H), 0.65 – 0.52 (m, 1H), 0.36 – 0.15 (m, 1H).
¹³C NMR (101 MHz, $CDCl_3$) δ 123.7, 81.3, 49.5, 43.0, 25.1, 24.6, 24.0, 22.2, 21.8, 21.5, 10.6.
¹¹B NMR (128 MHz, $CDCl_3$) δ 37.1 (s, 1B), -25.7 (t, *J* = 85.2 Hz, 1B).
Note: After column chromatography, preparative thin-layer chromatography (PTLC) was employed to remove trace amounts of impurities to ensure the highest purity for the NMR spectra.
HRMS (ESI): Calcd for $C_{22}H_{44}B_2N_2O_2Na^+$ $[M+Na]^+$: 413.3481, found: 413.3487.
IR (neat, cm^{-1}): *ν* 2971, 2929, 2294, 1399, 1368, 1274, 1147, 1111, 1047.
Chiral HPLC (OX-H, ^{i}PrOH/*n*-hexane = 2/98, 1.0 mL/min) t_R 22.2 min (minor), 26.0 min (major).
$[\alpha]_D^{25}$= -5.6 (*c* = 1.0, $CHCl_3$).

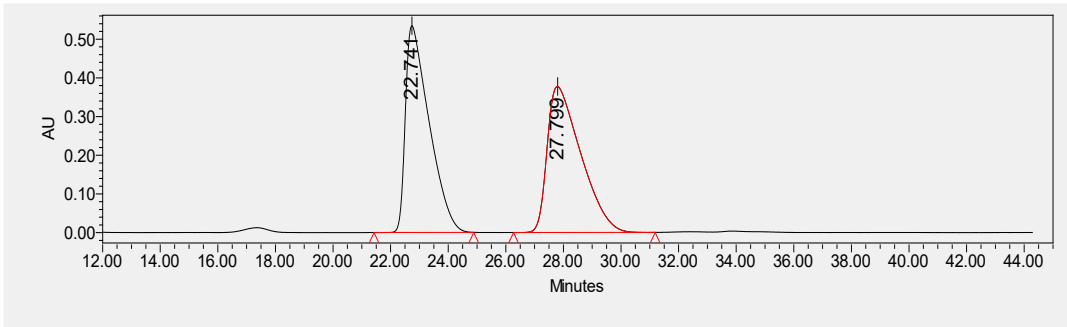

	Retention Time	Area	% Area
1	22.741	30551813	50.02
2	27.799	30529103	49.98

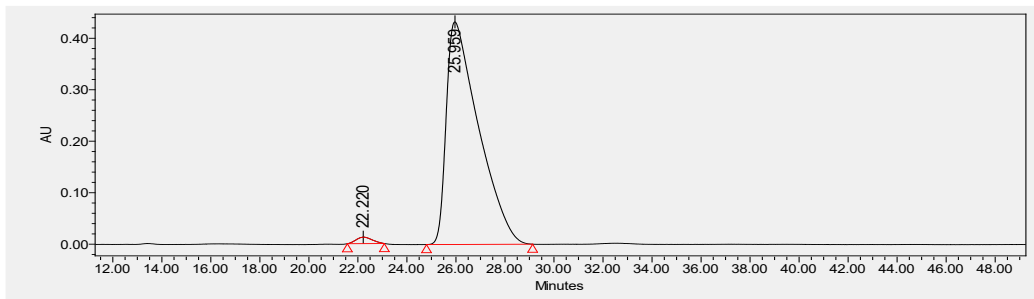

	Retention Time	Area	% Area
1	22.220	574754	1.44
2	25.959	39426913	98.56

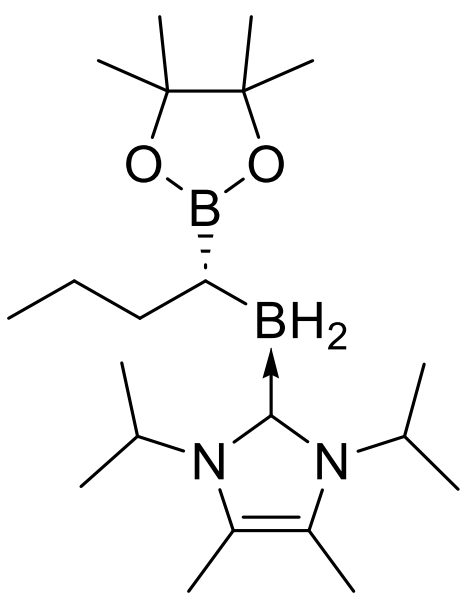

3z

3z: Yield: 16.9 mg, 45%, >99% ee, colorless oil, R_f = 0.4 (*n*-hexane/ethyl acetate = 3/1).
¹H NMR (500 MHz, $CDCl_3$) δ 5.66 – 5.45 (m, 2H), 2.21 (s, 6H), 1.73 – 1.61 (m, 1H), 1.52 – 1.39 (m, 14H), 1.28 – 1.16 (m, 13H), 1.14 – 1.00 (m, 1H), 0.78 (t, *J* = 7.3 Hz, 3H), 0.18 – 0.07 (m, 1H).
¹³C NMR (126 MHz, $CDCl_3$) δ 123.6, 81.4, 49.5, 46.1, 35.9, 25.2, 24.6, 21.8, 21.5, 14.7, 10.6.
¹¹B NMR (160 MHz, $CDCl_3$) δ 36.7 (s, 1B), -26.0 (t, *J* = 85.7 Hz, 1B).
Note: After column chromatography, preparative thin-layer chromatography (PTLC) was employed to remove trace amounts of impurities to ensure the highest purity for the NMR spectra.
HRMS (ESI): Calcd for $C_{21}H_{42}B_2N_2O_2Na^+$ $[M+Na]^+$: 399.3325, found: 399.3331.
IR (neat, cm^{-1}): *ν* 2973, 2931, 2302, 1398, 1367, 1240, 1147, 1079, 1015.

Chiral HPLC (IC, ^{i}PrOH/n-hexane = 1/99, 1.0 mL/min) t_R 28.4 min (major), 32.3 min (minor).

$[\alpha]_D^{25}$= -12.50 (c = 0.2, $CHCl_3$).

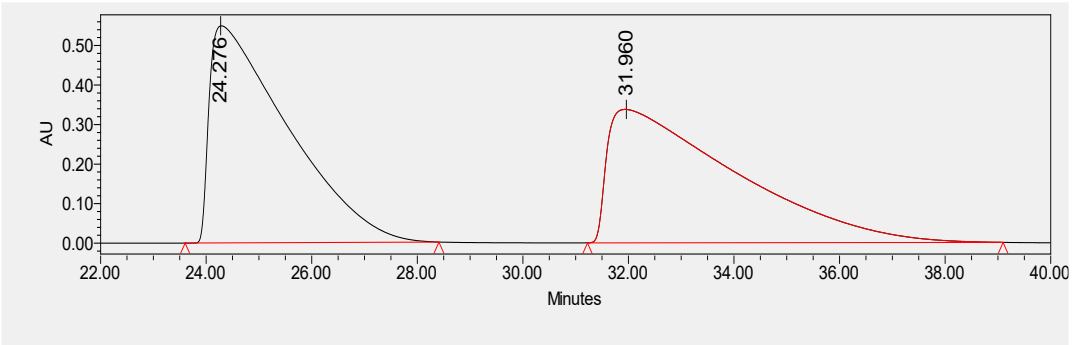

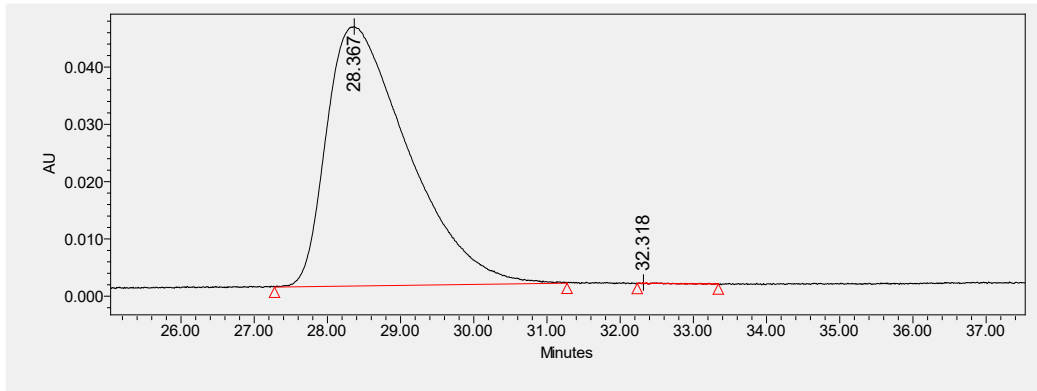

	Retention Time	Area	% Area
1	24.276	56692532	49.89
2	31.960	56951365	50.11

	Retention Time	Area	% Area
1	28.367	3506904	99.91
2	32.318	2987	0.09

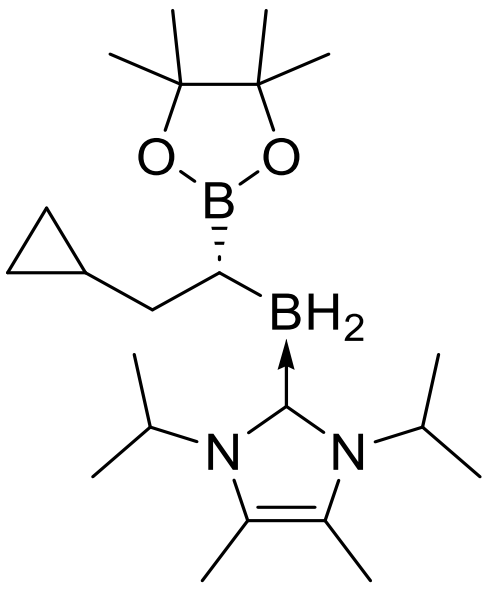

3aa

3aa: Yield: 19.8 mg, 51%, 97% ee, colorless oil, R_f = 0.4 (n-hexane/ethyl acetate = 3/1).

^{1}H NMR (500 MHz, $CDCl_3$) δ 5.63 – 5.08 (m, 2H), 2.20 (s, 6H), 1.45 – 1.32 (m, 13H), 1.32 – 1.18 (m, 12H), 0.80 – 0.67 (m, 1H), 0.67 – 0.50 (m, 1H), 0.36 – 0.19 (m, 3H), 0.10 – 0.00 (m, 1H), -0.13 – -0.28 (m, 1H).

^{13}C NMR (126 MHz, $CDCl_3$) δ 123.7, 81.4, 49.5, 38.5, 25.2, 24.6, 21.8, 21.5, 14.9, 10.6, 5.2, 4.6.

^{11}B NMR (160 MHz, $CDCl_3$) δ 37.3 (s, 1B), -26.1 (t, J = 85.0 Hz, 1B).

Note: After column chromatography, preparative thin-layer chromatography (PTLC) was employed to remove trace amounts of impurities to ensure the highest purity for the NMR spectra.

HRMS (ESI): Calcd for $C_{22}H_{42}B_2N_2O_2Na^+$ $[M+Na]^+$: 411.3325, found: 411.3330.

IR (neat, cm^{-1}): v 2972, 2923, 2853, 2275, 1440, 1398, 1302, 1147, 1093, 1010.

Chiral HPLC (OX-H, ^{i}PrOH/n-hexane = 1/99, 1.0 mL/min) t_R 23.0 min (minor), 24.3 min (major).

$[\alpha]_D^{25}$= -12.75 (c = 0.4, $CHCl_3$).

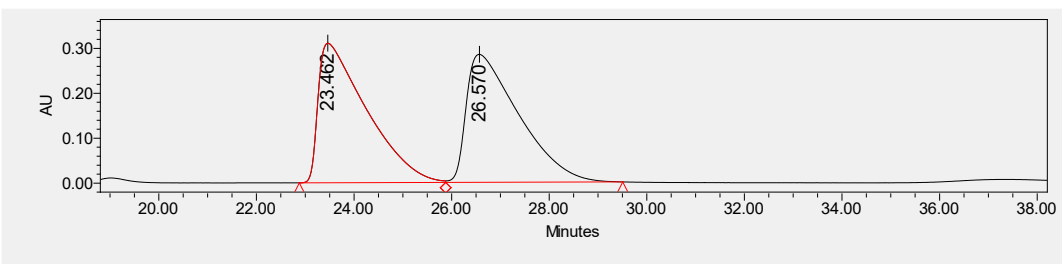

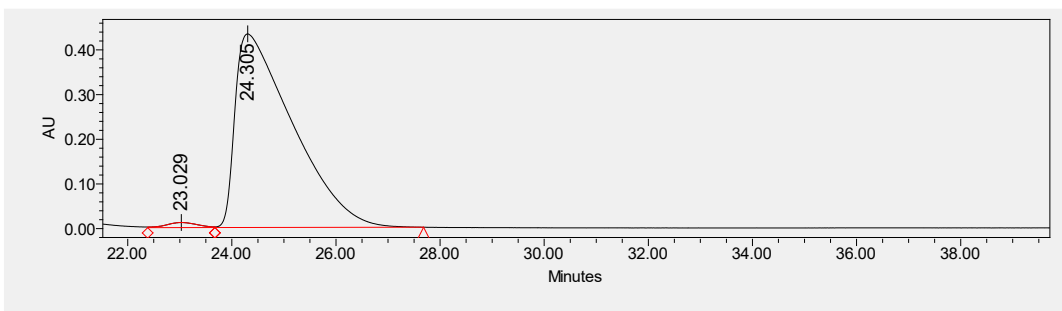

	Retention Time	Area	% Area
1	23.462	21303042	50.03
2	26.570	21273665	49.97

	Retention Time	Area	% Area
1	23.029	456505	1.35
2	24.305	33382250	98.65

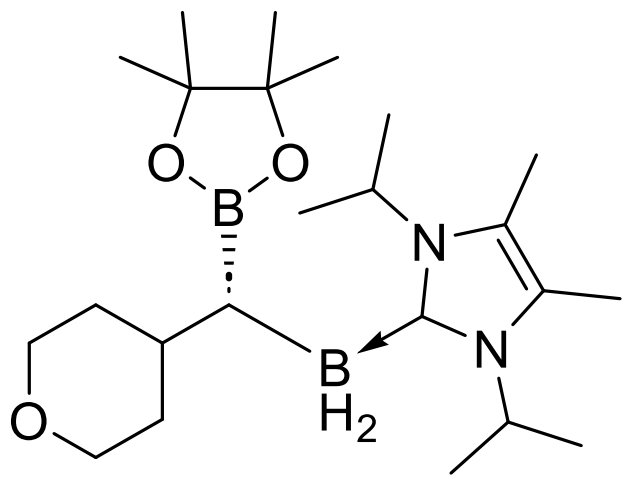

3ab

3ab: Yield: 17.1 mg, 41%, 95% ee, colorless oil, R_f = 0.4 (*n*-hexane/ethyl acetate = 3/1).
^{1}H NMR (400 MHz, $CDCl_3$) δ 5.61 – 5.41 (m, 2H), 4.13 – 3.69 (m, 2H), 3.46 – 3.10 (m, 2H), 2.71 (s, 1H), 2.20 (s, 6H), 1.81 – 1.65 (m, 1H), 1.55 – 1.47 (m, 1H), 1.63 – 1.36 (m, 12H), 1.34 – 1.28 (m, 1H), 1.23 (s, 6H), 1.17 (s, 6H), 1.08 – 0.95 (m, 1H), 0.06 – 0.01 (m, 1H).
^{13}C NMR (101 MHz, $CDCl_3$) δ 123.8, 81.4, 69.2, 69.1, 49.6, 38.7, 36.6, 36.1, 25.3, 24.5, 21.8, 21.4, 10.6.
^{11}B NMR (128 MHz, $CDCl_3$) δ 35.8 (s, 1B), -27.2 (t, J = 84.8 Hz, 1B).
Note: After column chromatography, preparative thin-layer chromatography (PTLC) was employed to remove trace amounts of impurities to ensure the highest purity for the NMR spectra.
HRMS (ESI): Calcd for $C_{23}H_{44}B_2N_2O_3Na^+$ $[M+Na]^+$: 441.3430, found: 441.3436.
IR (neat, cm^{-1}): v 2974, 2942, 2835, 2323, 2261, 1438, 1309, 1148, 1113.
Chiral HPLC (OX-H, ^{i}PrOH/*n*-hexane = 2/98, 1.0 mL/min) t_R 62.7 min (major), 78.3 min (minor).
$[\alpha]_D^{25}$= 3.5 (c = 0.6, $CHCl_3$).

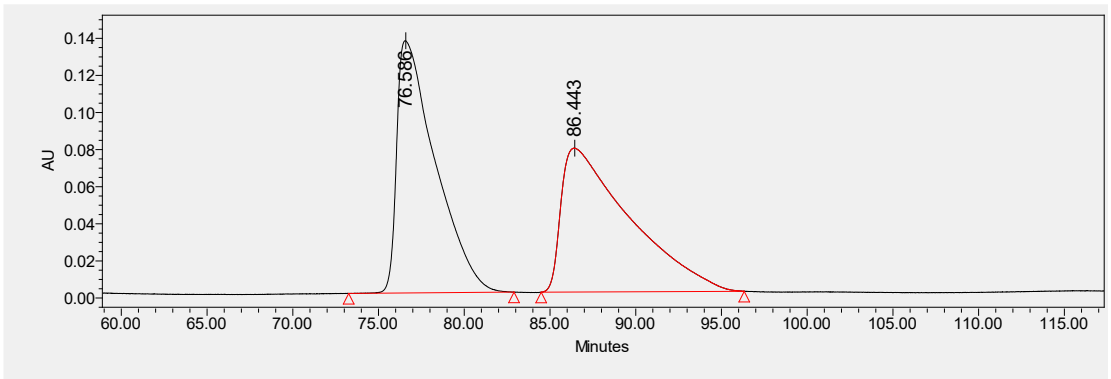

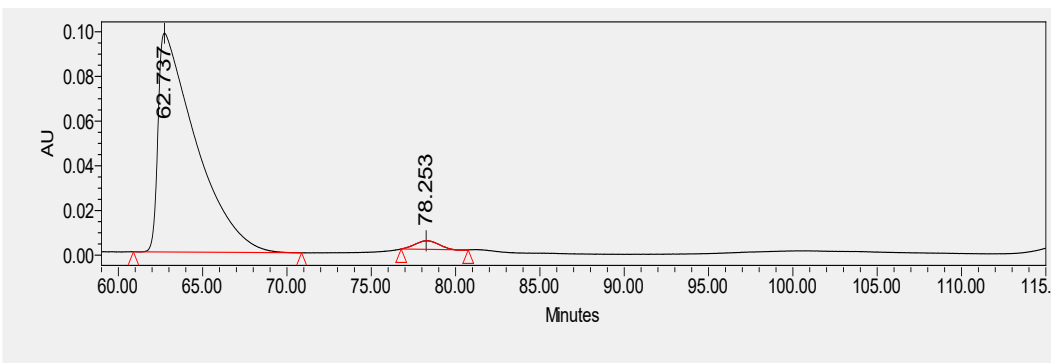

	Retention Time	Area	% Area
1	76.586	21275118	49.69
2	86.443	21542766	50.31

	Retention Time	Area	% Area
1	62.737	15282453	97.61
2	78.253	374076	2.39

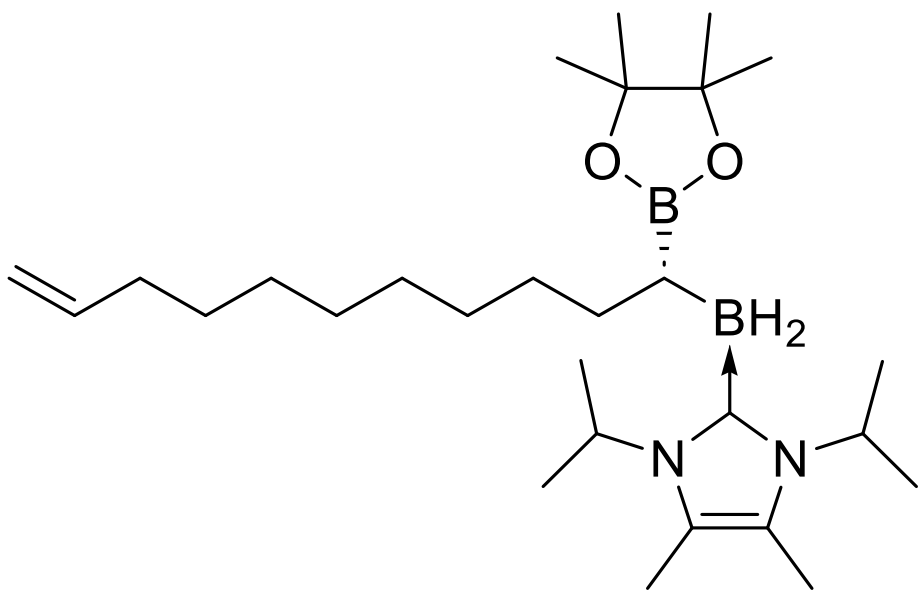

3ac

3ac: Yield: 20.3 mg, 43%, 84% ee, yellow oil, R_f = 0.4 (*n*-hexane/ethyl acetate = 3/1).

¹H NMR (400 MHz, $CDCl_3$) 5.86 – 5.73 (m, 1H), 5.70 – 5.46 (m, 2H), 5.17 – 4.86 (m, 2H), 2.21 (s, 6H), 2.05 – 1.94 (m, 2H), 1.50 – 1.34 (m, 12H), 1.27 – 1.15 (m, 24H), 1.13 – 1.01 (m, 1H), 0.89 – 0.77 (m, 1H), 0.20 – 0.06 (m, 1H).
¹³C NMR (126 MHz, $CDCl_3$) δ 139.5, 123.6, 114.1, 81.4, 49.5, 34.2, 34.2, 34.0, 33.5, 30.2, 29.9, 29.6, 29.3, 29.1, 25.2, 24.5, 21.8, 21.5, 10.6.
¹¹B NMR (128 MHz, $CDCl_3$) δ 37.4 (s, 1B), -25.9 (t, J = 84.6 Hz, 1B).
Note: After column chromatography, preparative thin-layer chromatography (PTLC) was employed to remove trace amounts of impurities to ensure the highest purity for the NMR spectra.
HRMS (ESI): Calcd for $C_{28}H_{54}B_2N_2O_2Na^+$ $[M+Na]^+$: 495.4264, found: 495.4276.
IR (neat, cm^{-1}): ν 2922, 2851, 2310, 1367, 1220, 1148, 1023.
Chiral HPLC (IB, ^{i}PrOH/n-hexane = 1/99, 1.0 mL/min) t_R 7.6 min (major), 9.1 min (minor).
$[\alpha]_D^{25}$= -6.25 (c = 0.4, $CHCl_3$).

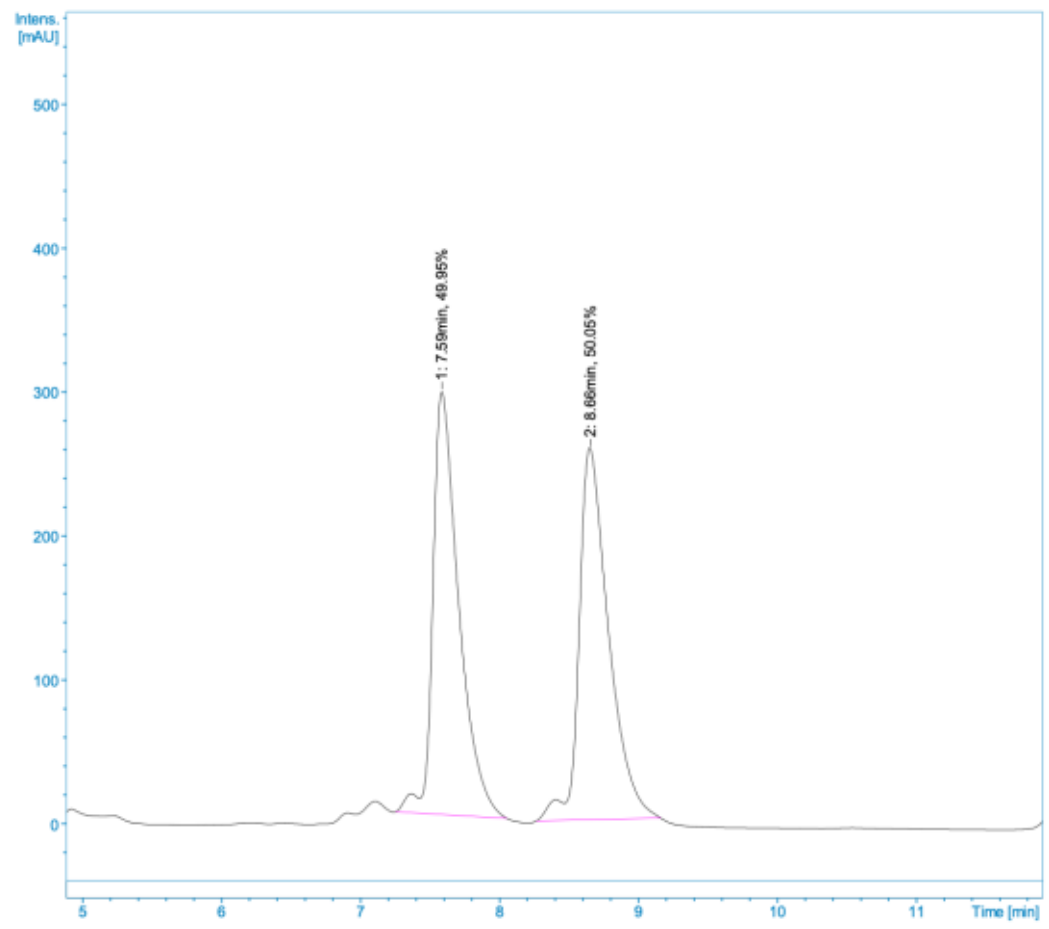

Peak	RT [min]	Peak Height [mAU]	Integral [%]	Peak Area [mAU*s]	FWHM [min]	Integral Region [min]	Ar
1	7.37	184.15	46.67	15491.2	0.2184	7.03 - 7.98	
2	8.82	181.30	53.33	17700.3	0.2541	8.37 - 9.45	

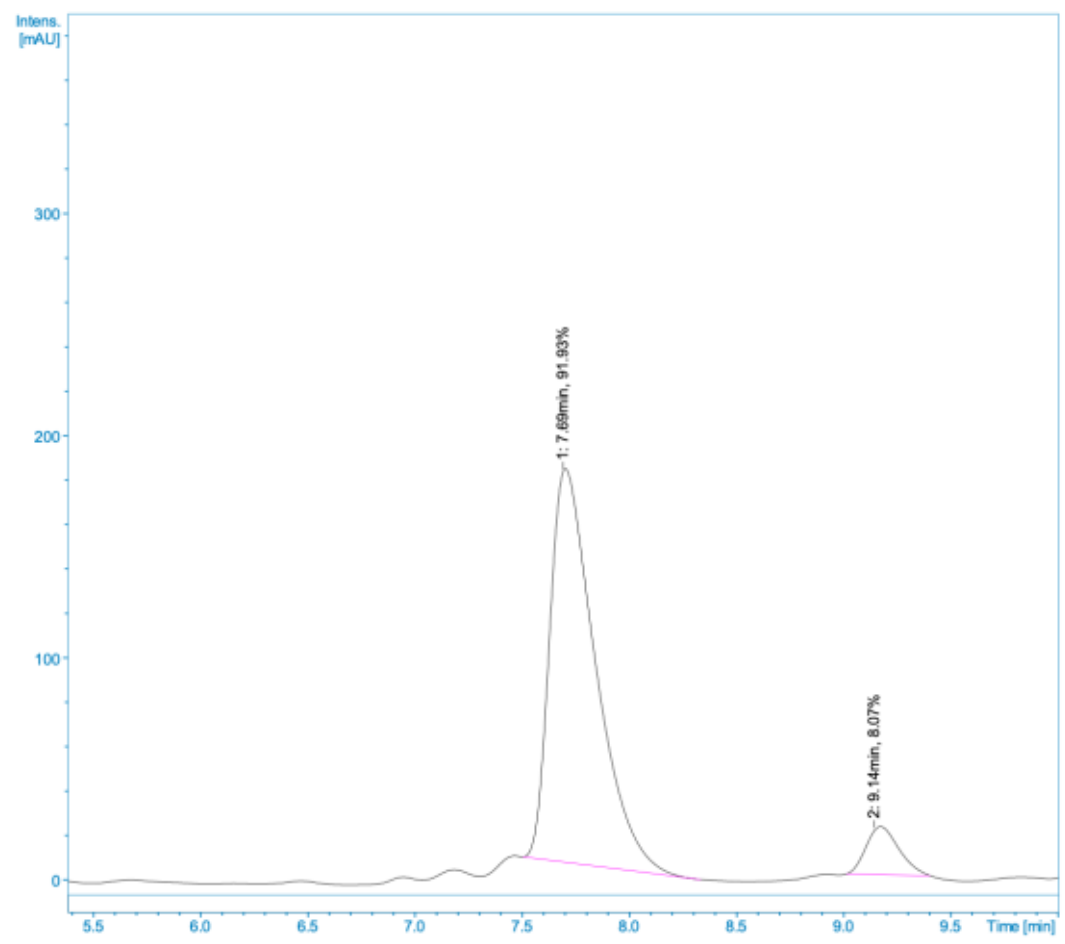

Peak	RT [min]	Peak Height [mAU]	Integral [%]	Peak Area [mAU*s]	FWHM [min]	Integral Region [min]	Ar
1	7.63	175.42	91.93	13127.4	0.2250	7.50 - 8.33	
2	9.09	19.74	8.07	1152.4	0.1777	9.01 - 9.40	

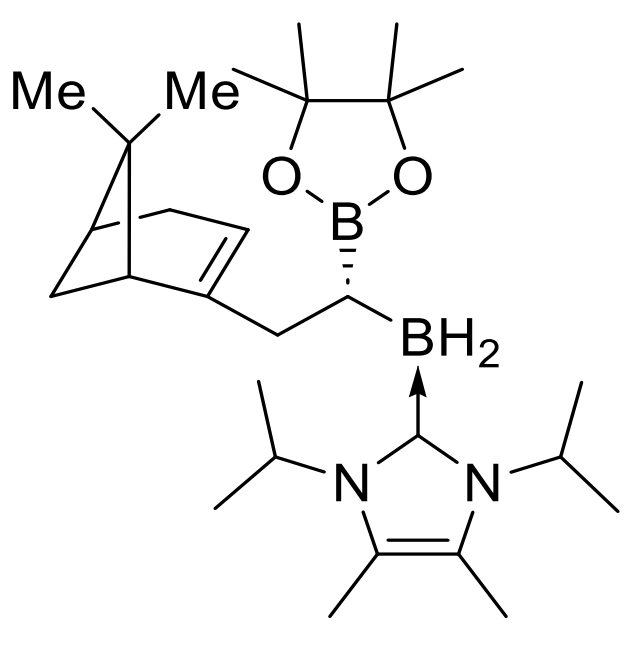

3ad

3ad: Yield: 33.2 mg, 71%, 95% ee, >20:1 d.r., colorless oil, R_f = 0.4 (n-hexane/ethyl acetate = 3/1).
¹H NMR (400 MHz, $CDCl_3$) δ 5.64 – 5.45 (m, 2H), 5.14 – 5.03 (m, 1H), 2.26 – 2.13 (m, 7H), 2.15 – 1.94 (m, 5H), 1.89 – 1.81 (m, 1H), 1.52 – 1.27 (m, 12H), 1.28 – 1.09 (m, 15H), 1.13 – 1.05 (m, 1H), 0.76 (s, 3H), 0.28 – 0.19 (m, 1H).

^{13}C NMR (101 MHz, $CDCl_3$) δ 153.2, 123.7, 112.5, 81.3, 49.5, 47.0, 41.1, 40.1, 37.9, 31.8, 31.3, 26.5, 25.3, 24.6, 21.8, 21.6, 21.5, 10.6.
^{11}B NMR (128 MHz, $CDCl_3$) δ 36.0 (s, 1B), -25.9 (t, J = 85.2 Hz, 1B).
Note: After column chromatography, preparative thin-layer chromatography (PTLC) was employed to remove trace amounts of impurities to ensure the highest purity for the NMR spectra.
HRMS (ESI): Calcd for $C_{28}H_{50}B_2N_2O_2Na^+$ $[M+Na]^+$: 491.3951, found: 491.3960.
IR (neat, cm^{-1}): v 2925, 2912, 2325, 1480, 1367, 1220, 1100.
Chiral HPLC (IB, ^{i}PrOH/n-hexane = 1/99, 1.0 mL/min) t_R 12.5 min (major), 16.9 min (minor).
$[\alpha]_D^{25}$= -16.33 (c = 0.6, $CHCl_3$).

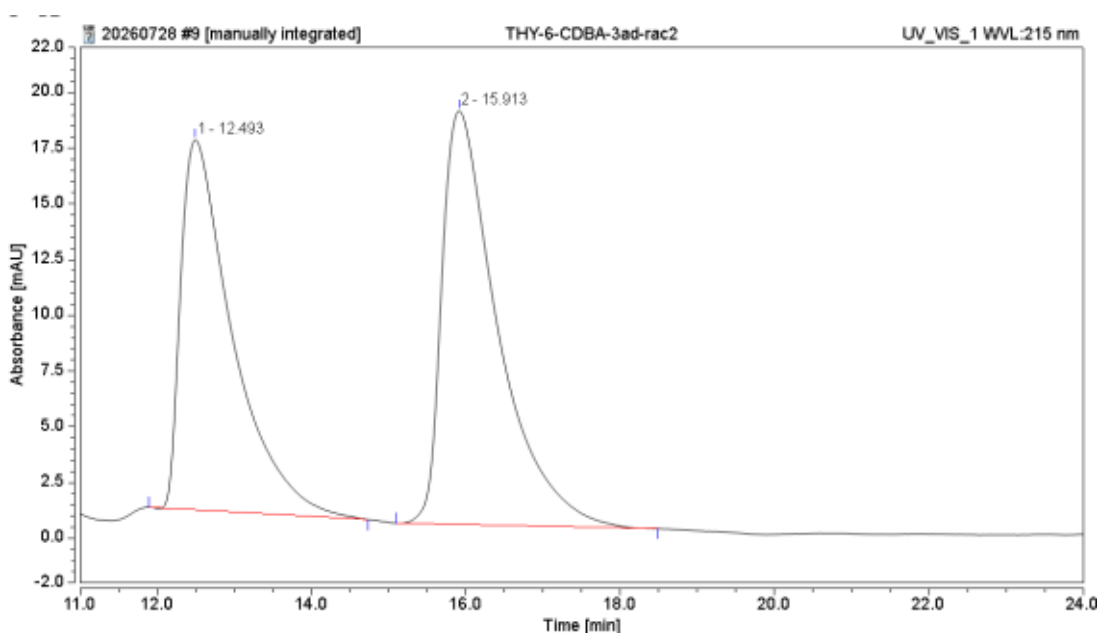

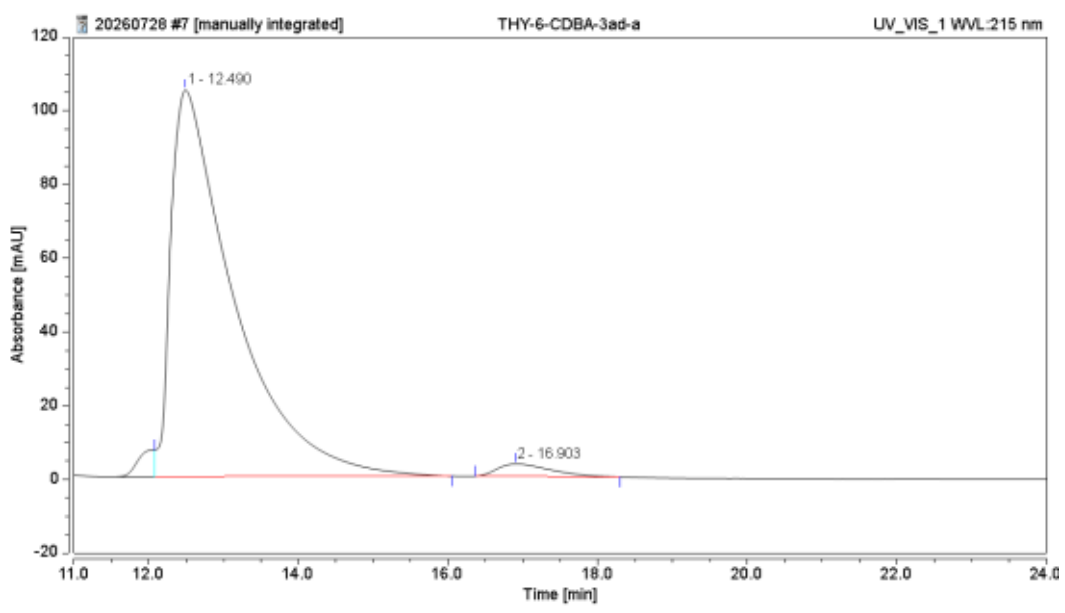

	Retention Time	Area	% Area
1	12.493	13.0769	47.23
2	15.913	14.6114	52.77

	Retention Time	Area	% Area
1	12.490	102.0326	97.33
2	16.903	2.7986	2.67

5.2. Synthesis of compound 3ae.

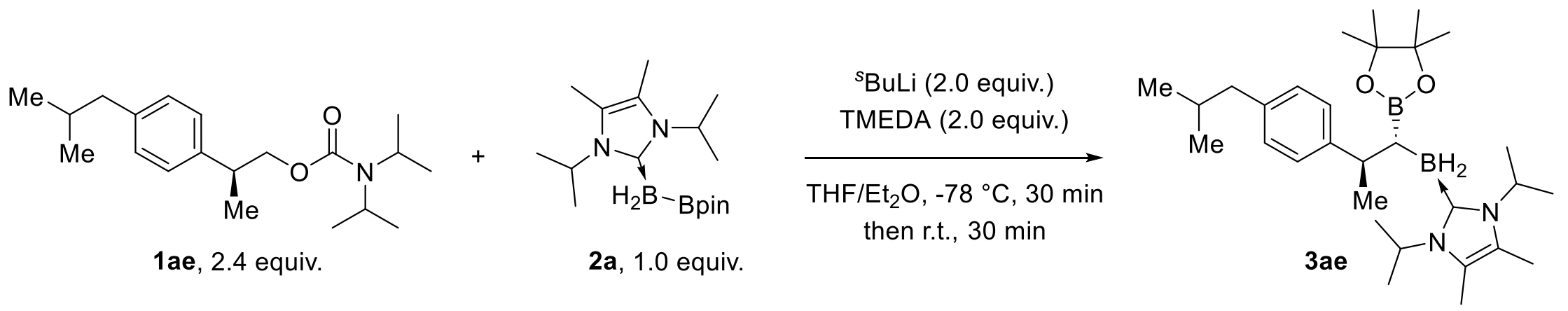

Under argon, to an oven-dried 4 mL reaction tube equipped with a magnetic stir bar and a septum was added diboron reagent **2a** (32.0 mg, 0.1 mmol, 1.0 equiv.), **1ae** (87.4 mg, 0.24 mmol, 2.4 equiv.), *N,N,N',N'*-tetramethylethylenediamine (23.2 mg, 0.2 mmol, 2.0 equiv.) and THF/Et_2O (1:1 ratio, 1 mL in total). The reaction was cooled to -78 °C and stirred for 10 min. ^{s}BuLi (1.3 M in cyclohexane, 0.15 mL, 0.2 mmol, 2.0 equiv.) was then slowly added and the mixture was stirred vigorously (without splashing) at -78 °C for 30 min. The reaction mixture was then warmed to room temperature and stirred for another 30 min. Upon completion, the reaction was quenched with aqueous NH_4Cl and extracted with ethyl acetate (3 × 3 mL). The combined organic phase was dried over $MgSO_4$, and the solvent was removed in vacuo. The resulting residue was purified by flash column chromatography to afford the desired product **3ae** (72%, 35.6 mg).

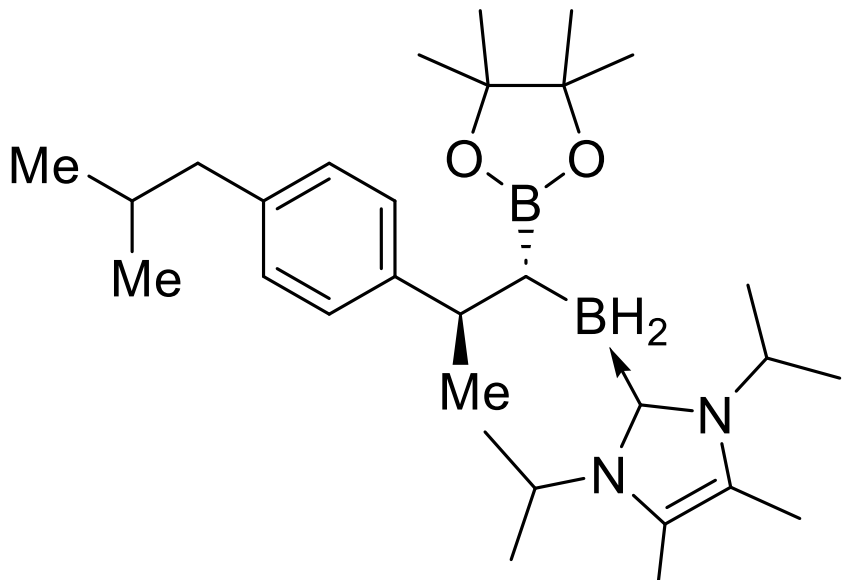

3ae

3ae: Yield: 35.6 mg, 72%, 99% ee, >20:1 d.r., white solid, m.p. = 153 – 158 °C, R_f = 0.4 (*n*-hexane/ethyl acetate = 3/1).

¹H NMR (500 MHz, $CDCl_3$) δ 7.16 (d, *J* = 7.6 Hz, 2H), 6.95 (d, *J* = 7.6 Hz, 2H), 5.99 – 5.21 (m, 2H), 2.91 – 2.70 (m, 1H), 2.37 (d, *J* = 7.0 Hz, 2H), 2.23 (s, 6H), 1.80 – 1.73 (m, 1H), 1.65 – 1.35 (m, 12H), 0.97 (s, 6H), 0.90 – 0.72 (m, 15H), 0.64 – 0.43 (m, 1H).

¹³C NMR (101 MHz, $CDCl_3$) δ 151.3, 137.7, 128.6, 127.0, 123.9, 81.1, 49.7, 45.3, 43.4, 30.5, 26.0, 24.7, 24.2, 22.5, 22.4, 21.8, 21.3, 10.6.

¹¹B NMR (160 MHz, $CDCl_3$) δ 35.2 (s, 1B), -26.4 (t, *J* = 84.2 Hz, 1B).

Note: After column chromatography, preparative thin-layer chromatography (PTLC) was employed to remove trace amounts of impurities to ensure the highest purity for the NMR spectra.

HRMS (ESI): Calcd for $C_{30}H_{52}B_2N_2O_2Na^+$ $[M+Na]^+$: 517.4107, found: 517.4117.

IR (neat, cm^{-1}): *v* 2959, 2925, 2854, 1556, 1462, 1377, 1289, 1109, 1094, 1020.

Chiral HPLC (IC, ^{i}PrOH/*n*-hexane = 3/97, 1.0 mL/min) t_R 6.3 min (minor), 6.8 (major).

$[\alpha]_D^{25}$ = 22.5 (*c* = 0.6, $CHCl_3$).

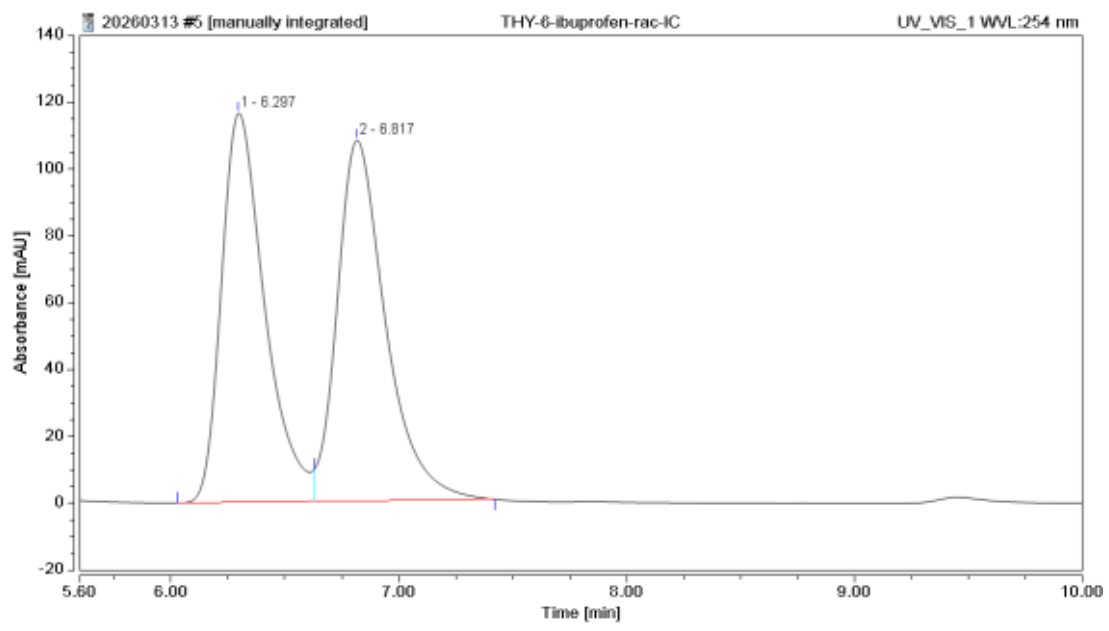

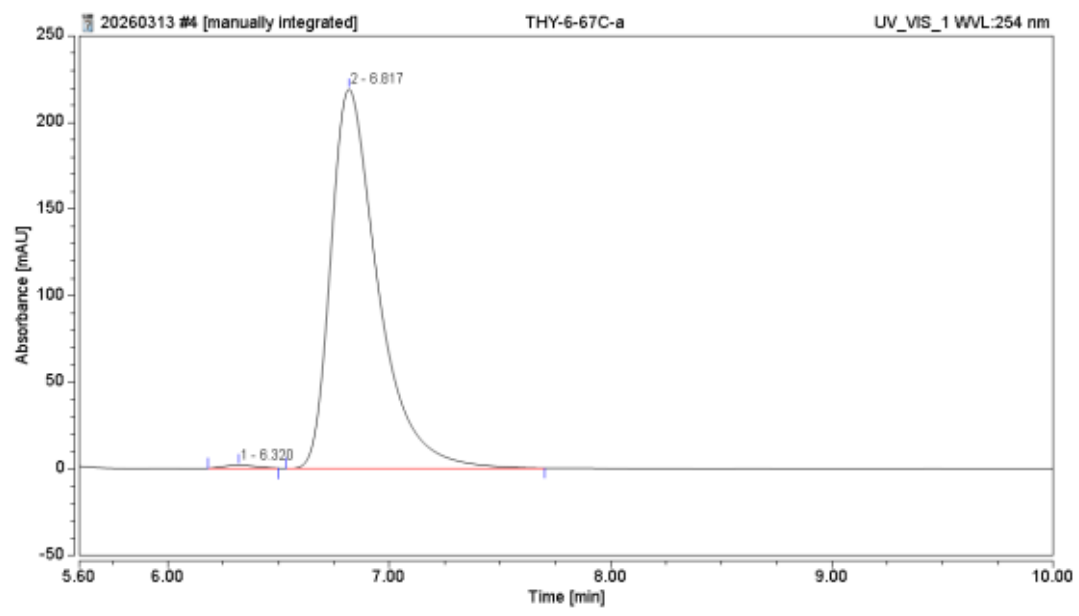

	Retention Time	Area	% Area
1	6.297	25.6139	49.14
2	6.817	26.5077	50.86

	Retention Time	Area	% Area
1	6.320	0.3014	0.55
2	6.817	54.0243	99.45

5.3. Gram-scale synthesis of compound 3t.

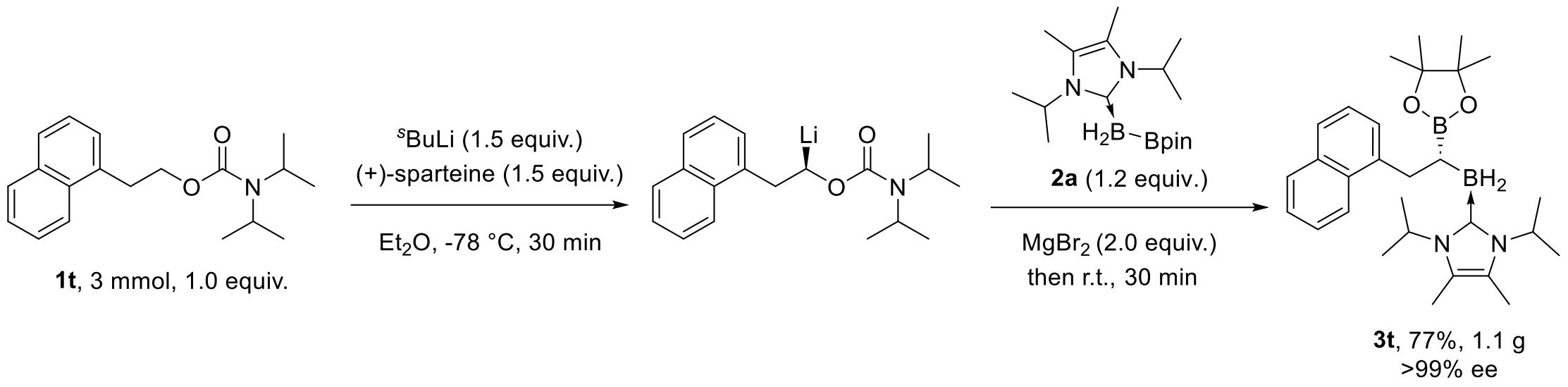

Under argon, to an oven-dried 250 mL round-bottom flask equipped with a magnetic stir bar and a septum was added carbamate **1t** (897 mg, 3.0 mmol, 1.0 equiv.), (+)-sparteine (1.06 g, 4.5 mmol, 1.5 equiv.) and Et_2O (15 mL). The reaction was cooled to -78 °C and stirred for 10 min. ^{s}BuLi (1.3 M in cyclohexane, 3.5 mL, 4.5 mmol, 1.5 equiv.) was then slowly added and the mixture was stirred vigorously (without splashing) at -78 °C for 30 min. After that, a solution of **2a** (1.15 g, 3.6 mmol, 1.2 equiv.) in Et_2O (36 mL) was added dropwise. The reaction mixture was stirred at this temperature for an additional 10 min. $MgBr_2$ (freshly made from Mg and 1,2-dibromoethane, 0.5 M in Et_2O, 12 mL, 6.0 mmol, 2.0 equiv.) was then added dropwise and the reaction mixture was stirred at -78 °C for 20 min. After that, the reaction mixture was warmed to room temperature and stirred for another 30 min. Upon completion, the reaction was quenched with aqueous NH_4Cl and extracted with ethyl acetate (3 × 15 mL). The combined organic phase was dried over $MgSO_4$, and the solvent was removed in vacuo. The resulting residue was purified through recrystallization from *n*-hexane/ethyl acetate to afford the desired product **3t** (77%, 1.1 g).

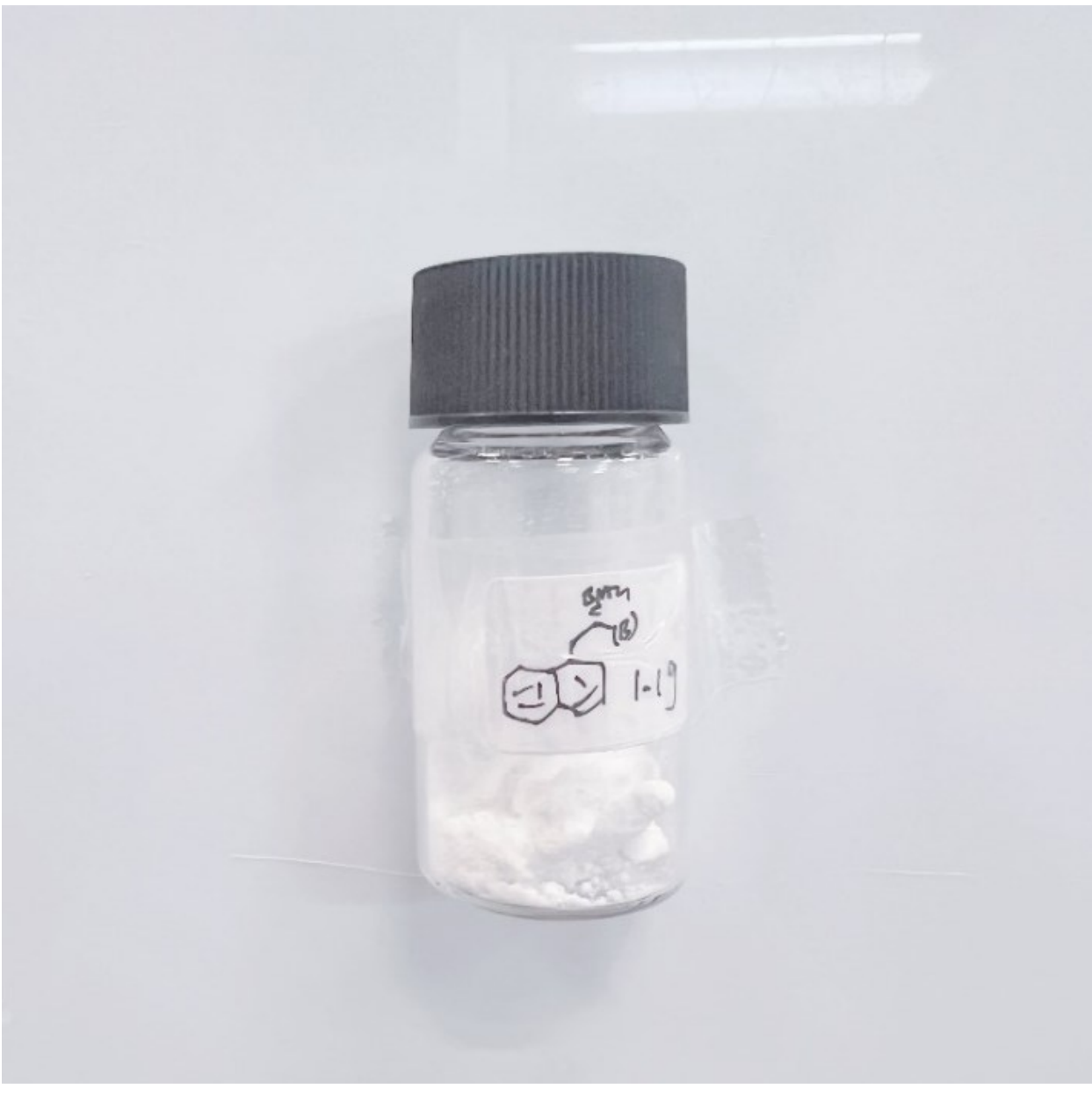

Fig. S2: Gram scale synthesis of **3t**.

(+)-Sparteine recovery.[13]

After the extraction, the combined aqueous layers were basified (about pH 11) with NaOH (20% aq.). The aqueous phase was extracted with Et_2O. The combined organic layers were dried over K_2CO_3, filtered and concentrated in vacuo to give crude (+)-sparteine. Distillation over CaH_2 of the residual oil gave pure (+)-sparteine (689 mg, 65%) as a colorless oil.

Distillation conditions: bulb to bulb; 10 cm Vigreux column; pressure: 2 mbar; oil bath: 150 °C; CaH_2 (100 mg/mL); (+)-sparteine b.p. = 137 – 138 °C (1.33 mbar).

5.4. Limitations of the substrate scope.

Several functional groups, including esters, nitriles, aryl bromides, amides, terminal alkynes, and pyridyl rings, were examined but either failed to yield the desired products or gave very low yields. These electrophilic and acidic functional groups were not compatible with the strongly basic conditions required for lithium carbenoid generation.[4, 14]

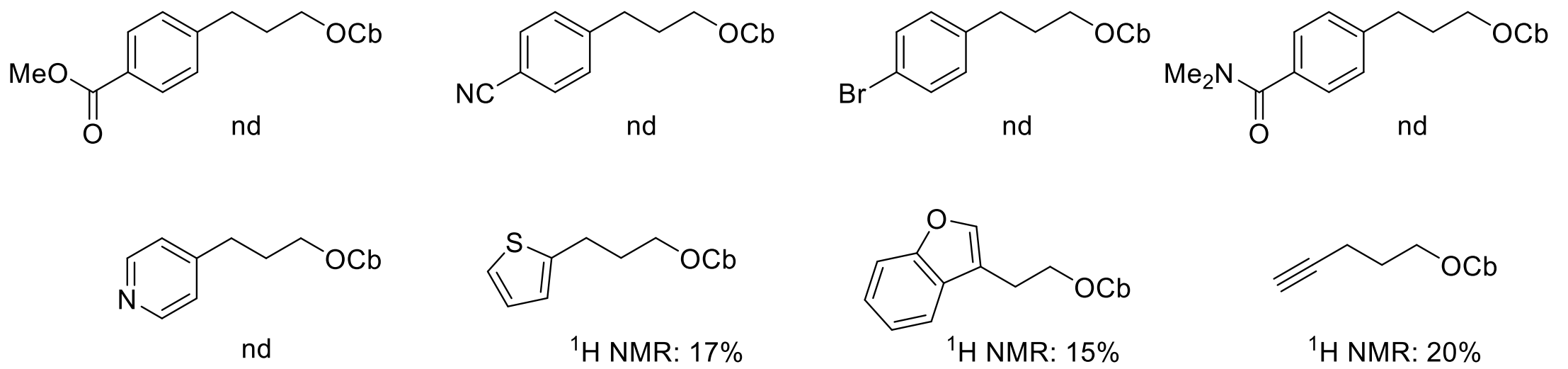

Fig. S3. Substrates with low compatibility. ^{1}H NMR yields of the corresponding products were provided using 1,1,2,2-tetrachloroethane as the internal standard.

6. Transformations of chiral *gem*-diborylalkane products 3.

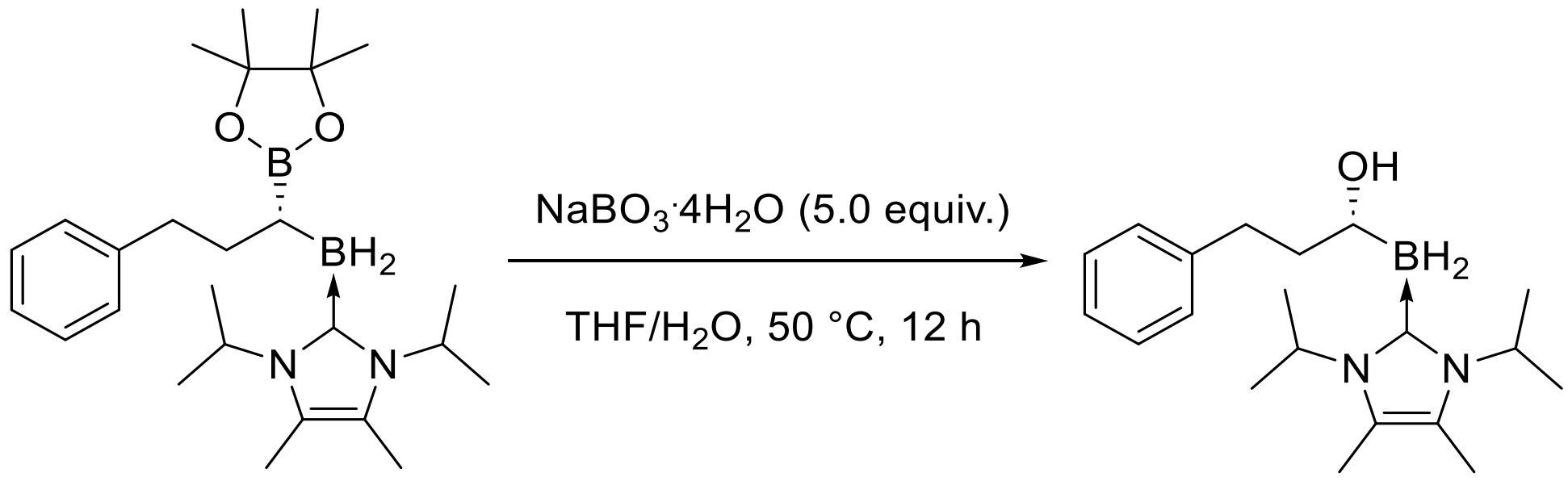

3a, 98% ee **4**

Under air, to an oven dried 4 mL reaction tube charged with a magnetic stir bar was added **3a** (43.8 mg, 0.1 mmol, 1.0 equiv.), sodium perborate tetrahydrate (77.0 mg, 0.5 mmol, 5.0 equiv.) and THF/H_2O (1:1, 2 mL in total). The reaction mixture was then heated at 50 °C and vigorously stirred for 12 h. Upon completion, the reaction was quenched with H_2O and extracted with Et_2O (3 × 3 mL). The combined organic phases were dried over $MgSO_4$, and the solvent was removed in vacuo. The resulting residue

was purified by flash column chromatography to afford the desired product **4** (45%, 14.7 mg).

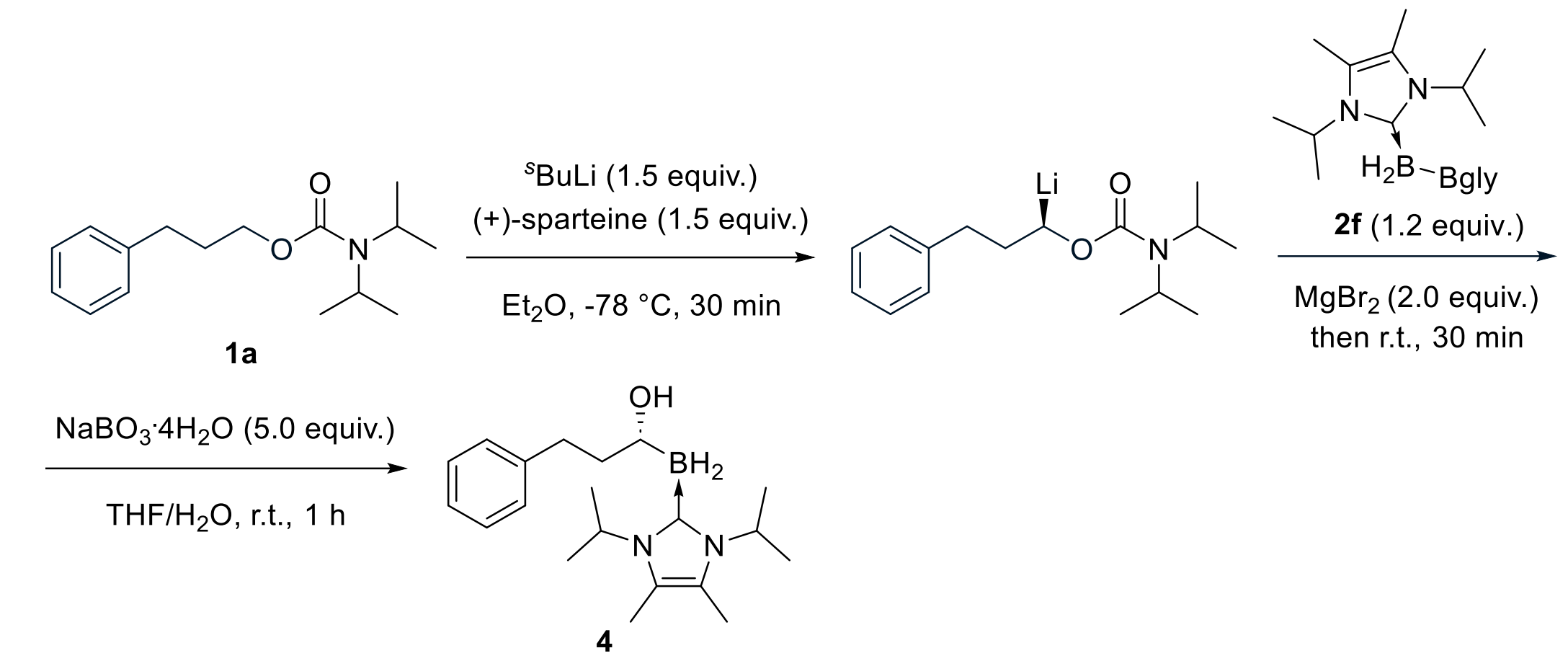

Under argon, to an oven dried 4 mL reaction tube equipped with a magnetic stir bar and a septum was charged with **1a** (26.3 mg, 0.1 mmol, 1.0 equiv.), (+)-sparteine (35.2 mg, 0.15 mmol, 1.5 equiv.) and Et_2O (500 μL). The reaction was cooled to -78 °C and stirred for 10 min. ^{s}BuLi (1.3 M in cyclohexane, 0.12 mL, 0.15 mmol, 1.5 equiv.) was then slowly added and the mixture was stirred vigorously (without splashing) at -78 °C for 30 min. After that, a solution of the **2f** (31.7 mg, 0.12 mmol, 1.2 equiv.) in Et_2O (2.0 mL) was added dropwise. The reaction mixture was stirred at this temperature for an additional 10 min. $MgBr_2$ (freshly made from Mg and 1,2-dibromoethane, 0.5 M in Et_2O, 0.4 mL, 0.2 mmol, 2.0 equiv.) was then added dropwise and the reaction mixture was stirred at -78 °C for 20 min. The reaction mixture was then warmed to room temperature and stirred for another 30 min. Upon completion, sodium perborate tetrahydrate (77.0 mg, 0.5 mmol, 5.0 equiv.) and THF/H_2O (1:1, 2 mL in total were added. The reaction mixture was stirred at room temperature for 1 h. Upon completion, the reaction was quenched with H_2O and extracted with Et_2O (3 × 3 mL). The combined organic phases were dried over $MgSO_4$, and the solvent was removed in vacuo. The resulting residue was purified by flash column chromatography to afford the desired product **4** (67%, 22.0 mg).

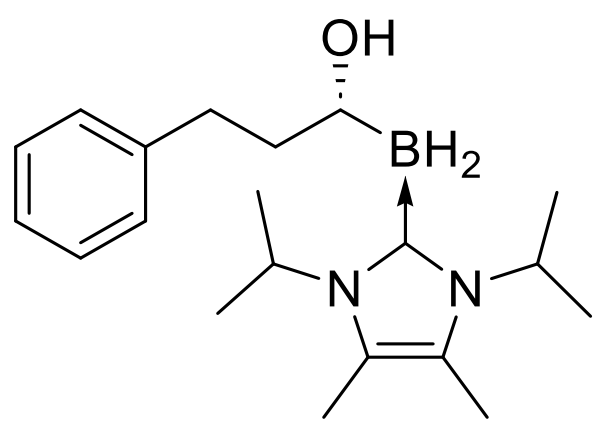

4

4: Yield: 14.7 mg, 45%, 99% ee, white solid, m.p. = 161 – 169 °C, R_f = 0.4 (*n*-hexane/ethyl acetate = 1/1).

^{1}H NMR (500 MHz, $CDCl_3$) δ 7.25 – 7.17 (m, 4H), 7.17 – 6.97 (m, 1H), 5.75 – 5.12 (m, 2H), 3.35 – 3.14 (m, 1H), 3.04 – 2.80 (m, 1H), 2.77 – 2.55 (m, 1H), 2.21 (s, 6H), 1.88 – 1.77 (m, 1H), 1.73 – 1.62 (m, 2H), 1.44 – 1.34 (m, 12H).

^{13}C NMR (126 MHz, $CDCl_3$) δ 144.3, 128.7, 128.1, 125.2, 124.1, 50.0, 42.6, 34.1, 21.6, 21.5, 10.6.
^{11}B NMR (128 MHz, $CDCl_3$) δ -24.5 (t, J = 83.8 Hz, 1B).
HRMS (ESI): Calcd for $C_{20}H_{33}BN_2ONa^+$ $[M+Na]^+$: 351.2578, found: 351.2582.
IR (neat, cm^{-1}): *v* 2923, 2852, 2298, 1399, 1321, 1108, 1019.
$[\alpha]_D^{25}$= 6.33 (*c* = 0.3, $CHCl_3$).
From **3a**, 99% ee, **chiral HPLC** (IB, ^{i}PrOH/*n*-hexane = 2/98, 1.0 mL/min) t_R 30.8 min (major), 35.4 min (minor).

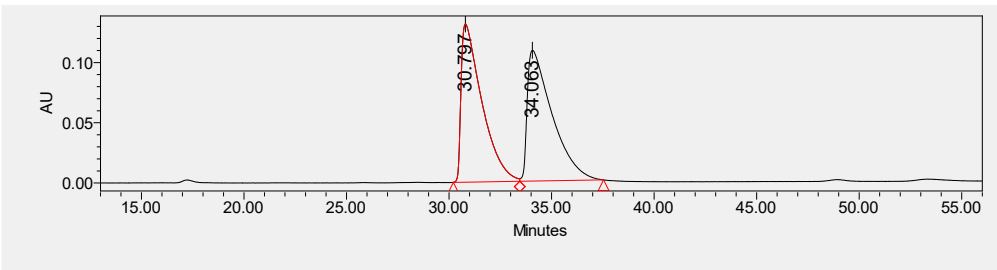

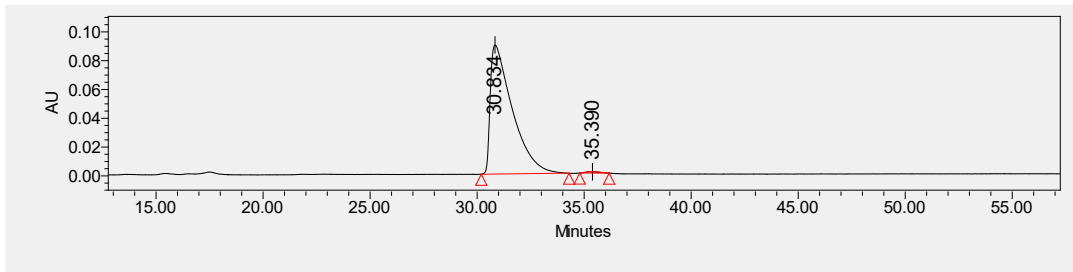

	Retention Time	Area	% Area
1	30.797	9220505	50.19
2	34.063	9151376	49.81

	Retention Time	Area	% Area
1	30.834	6397748	99.37
2	35.390	40603	0.63

From **1a** and **2f**, 99% ee, **chiral HPLC** (IB, ^{i}PrOH/*n*-hexane = 2/98, 1.0 mL/min) t_R 28.5 min (major), 35.4 min (minor).

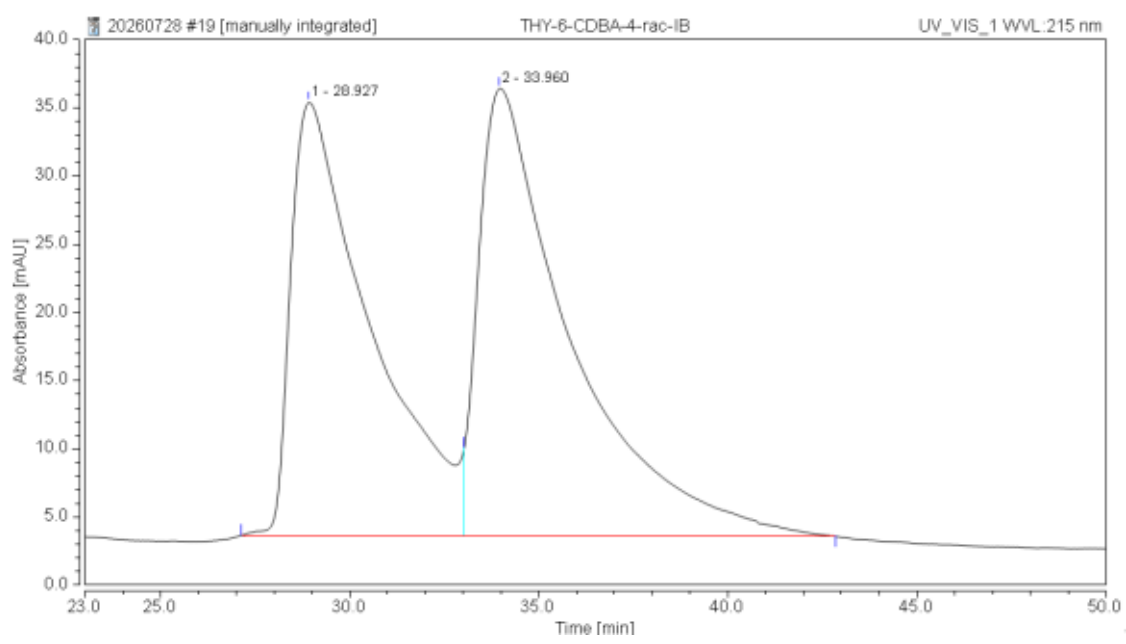

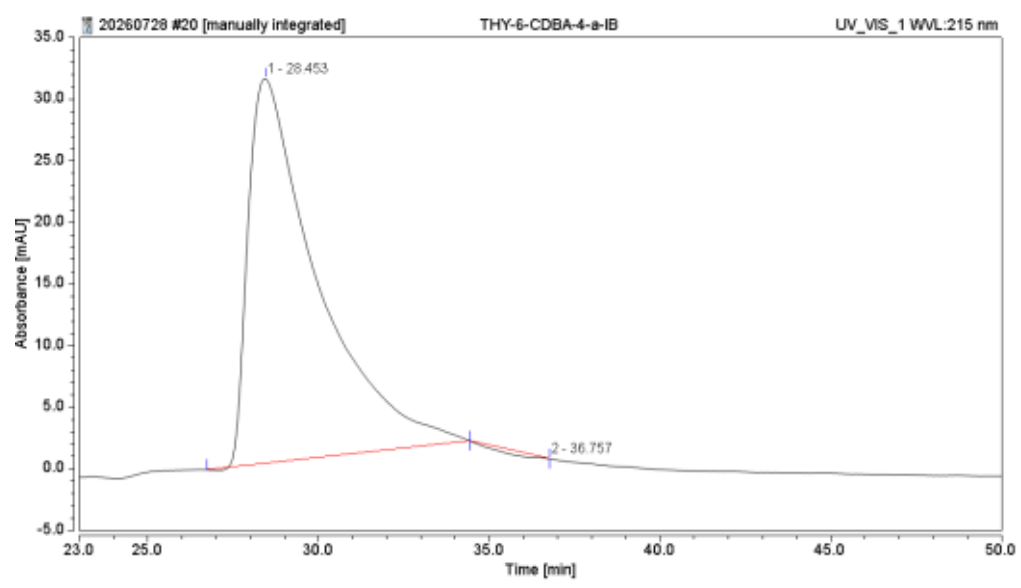

	Retention Time	Area	% Area
1	28.927	77.5200	45.76
2	33.960	91.9000	54.24

	Retention Time	Area	% Area
1	28.453	73.2436	99.27
2	36.757	0.5365	0.73

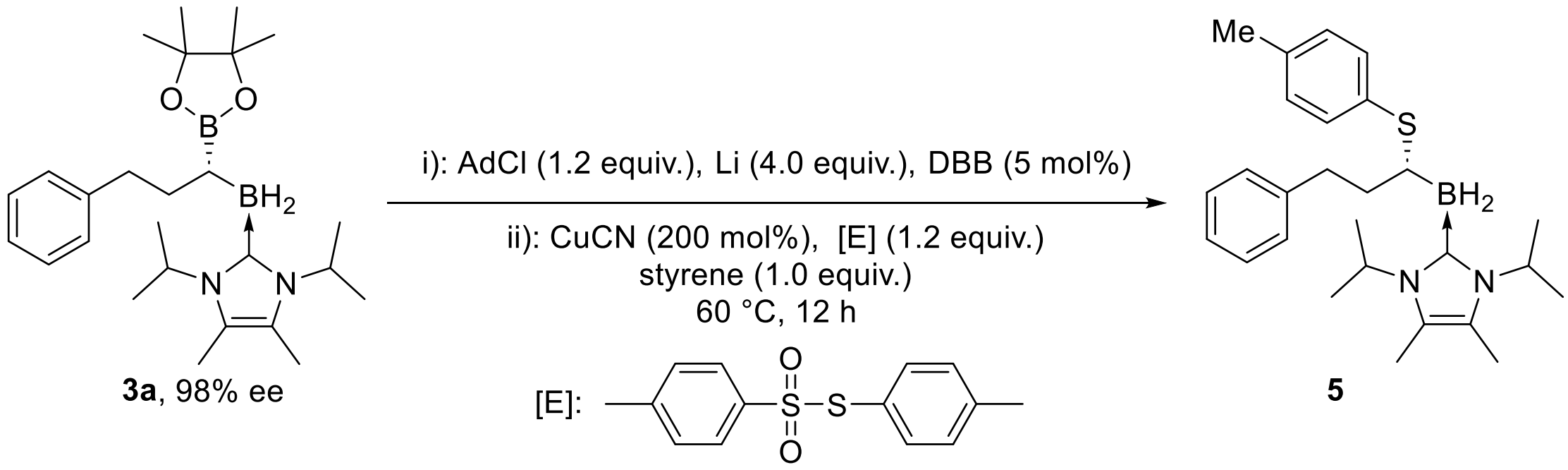

According to a reported literature,[15] in an argon-charged glovebox, to an oven-dried 4 mL vial charged with magnetic stir bar was added **3a** (43.8 mg, 0.1 mmol, 1.0 equiv.), 1-chloroadamantane (18.9 mg, 0.11 mmol, 1.1 equiv.), 1-tert-butyl-4-(4-tert-butylphenyl)benzene (DBB) (1.4 mg, 0.005 mmol, 0.05 equiv.), lithium (freshly cut, 3.5 mg, 5.0 equiv.) and anhydrous THF (150 µL). The vial was capped and stirred at

room temperature for 1 h until the reaction mixture turned dark red, indicating the formation of the borate complex. The solution was transferred to another reaction tube equipped with a magnetic stir bar, and the residual lithium was washed with anhydrous THF (350 μL). To the reaction tube was added copper cyanide (18.0 mg, 0.2 mmol, 2.0 equiv.), electrophile (33.4 mg, 0.12 mmol, 1.2 equiv.) and styrene (10.4 mg, 0.1 mmol, 1.0 equiv.). Then, the tube was capped, sealed with Teflon tape, brought out from glovebox, and heated at 60 °C for 12 h. Upon completion, the reaction mixture was passed through a short pad of silica, washed with ethyl acetate and concentrated in vacuo. The resulting residue was purified by flash column chromatography to afford the desired product **5** (35%, 15.2 mg).

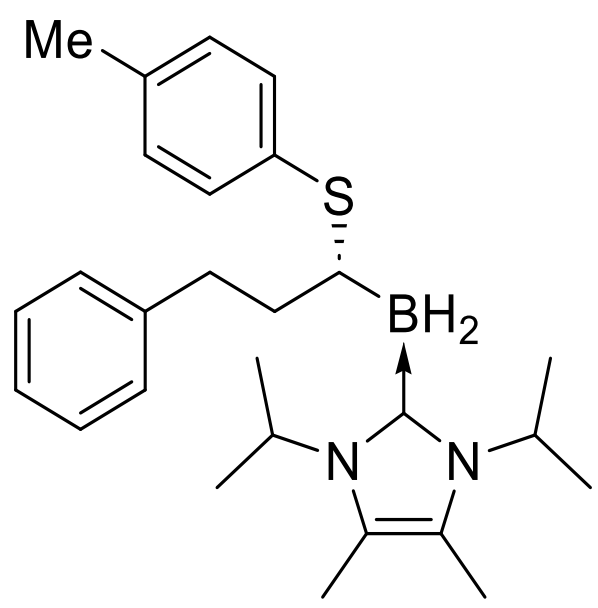

5

5: Yield: 15.2 mg, 35%, 74% ee, colorless oil, R_f = 0.4 (*n*-hexane/ethyl acetate = 4/1).
^{1}H NMR (500 MHz, $CDCl_3$) δ 7.25 – 7.10 (m, 5H), 7.06 – 6.85 (m, 4H), 5.56 – 5.38 (m, 2H), 2.97 – 2.87 (m, 1H), 2.86 – 2.70 (m, 1H), 2.50 – 2.38 (m, 1H), 2.26 (s, 3H), 2.21 (s, 6H), 2.14 – 1.99 (m, 1H), 1.93 – 1.80 (m, 1H), 1.53 – 1.49 (m, 6H), 1.40 – 1.32 (m, 6H).
^{13}C NMR (126 MHz, $CDCl_3$) δ 143.8, 137.0, 133.6, 129.1, 128.8, 128.2, 127.9, 125.1, 124.0, 49.9, 38.4, 34.2, 21.6, 21.2, 20.9, 10.5.
^{11}B NMR (160 MHz, $CDCl_3$) δ -26.3 (t, *J* = 86.0 Hz, 1B).
Note: After column chromatography, preparative thin-layer chromatography (PTLC) was employed to remove trace amounts of impurities to ensure the highest purity for the NMR spectra.
HRMS (ESI): Calcd for $C_{27}H_{39}BN_2SNa^+$ $[M+Na]^+$: 457.2819, found: 457.2817.
IR (neat, cm^{-1}): *v* 2961, 2922, 2853, 2300, 1454, 1261, 1088, 1019.
Chiral HPLC (OX-H, ^{i}PrOH/*n*-hexane = 2/98, 1.0 mL/min) t_R 7.9 min (minor), 10.6 min (major).
$[\boldsymbol{\alpha}]_D^{25}$= -46.67 (*c* = 0.1, $CHCl_3$)

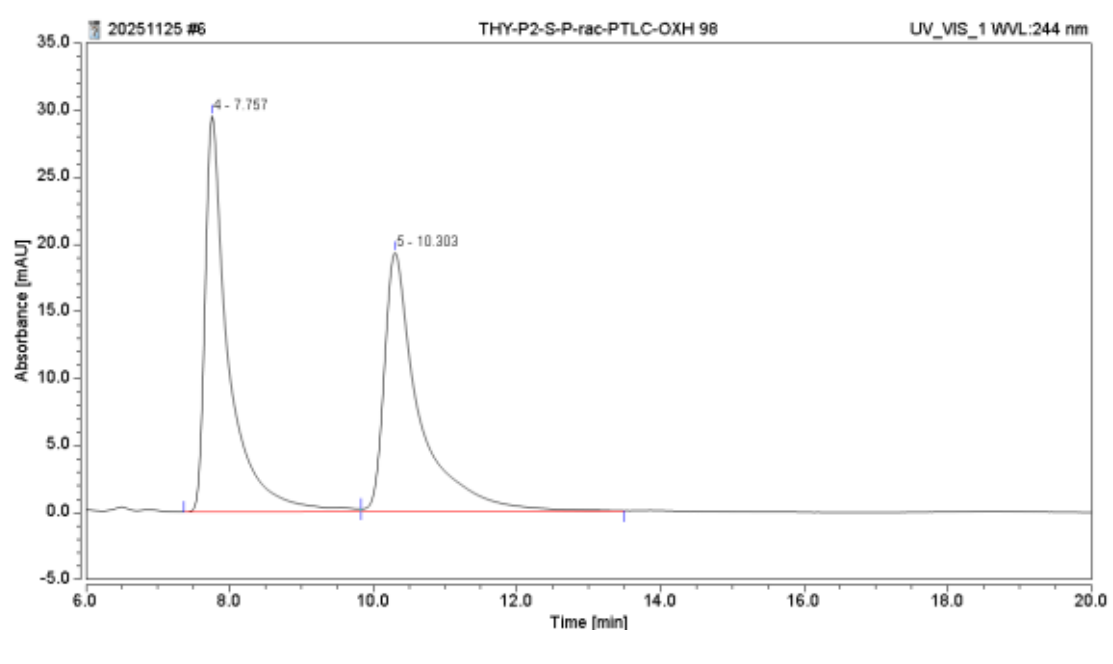

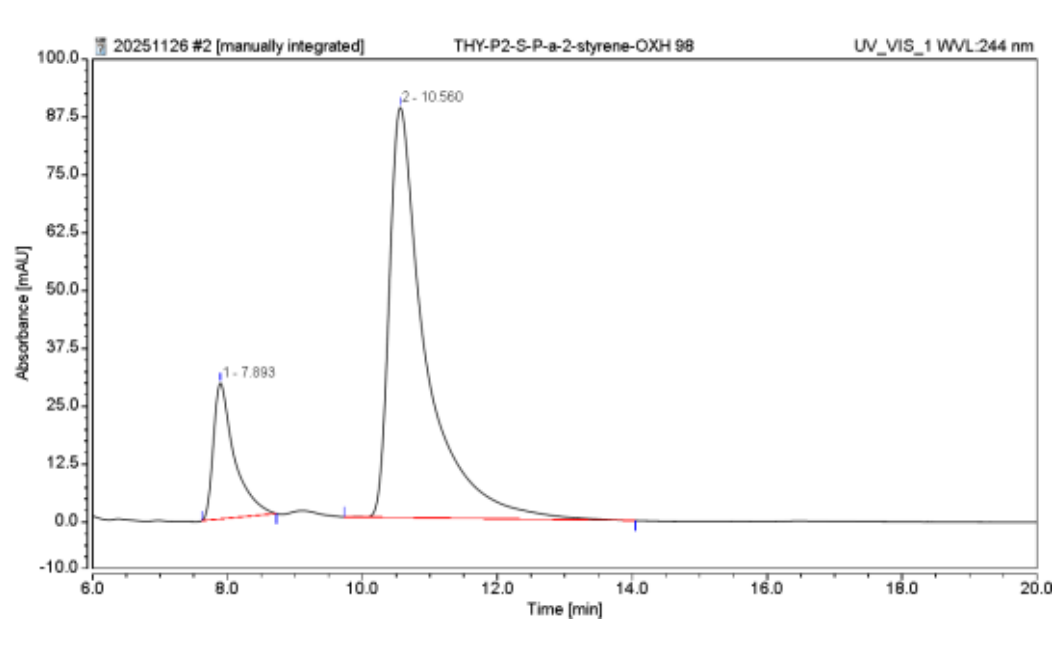

	Retention Time	Area	% Area
1	7.757	30.4774	50.86
2	10.303	29.4494	49.14

	Retention Time	Area	% Area
1	7.893	8.1709	12.91
2	10.56	55.0971	87.09

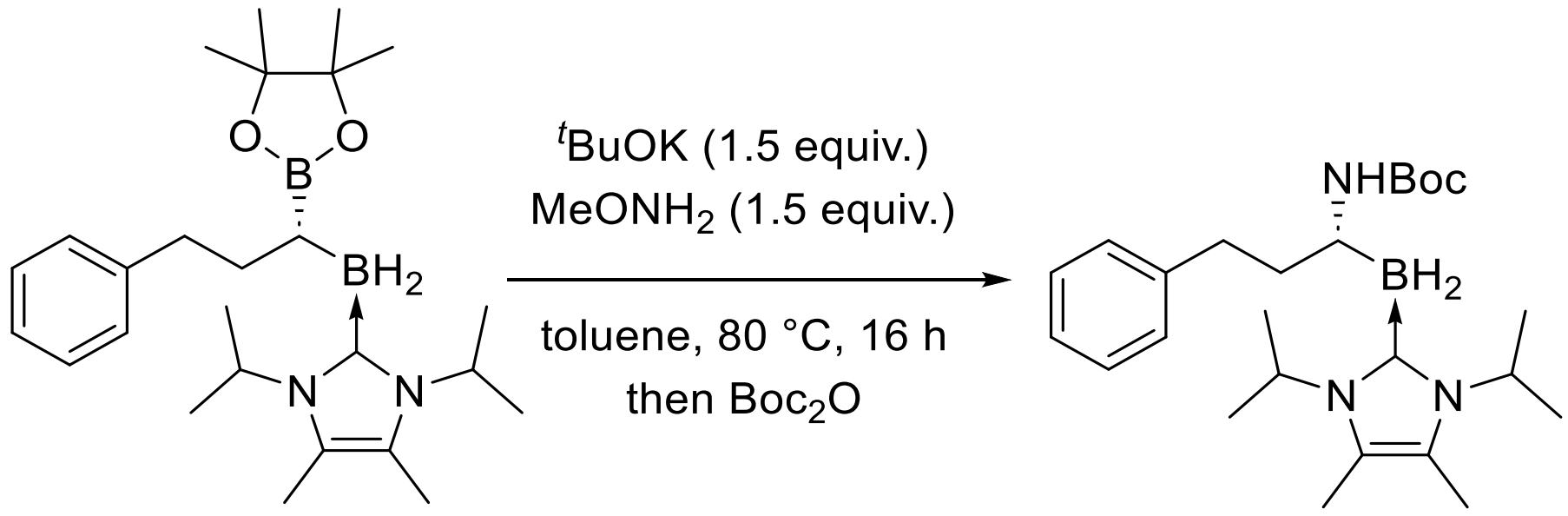

According to a reported literature,[16] under argon, to an oven dried 4 mL reaction tube charged with magnetic stir bar was added **3a** (43.8 mg, 0.1 mmol, 1.0 equiv.), $MeONH_2$ (1.5 M in THF, 100 μL, 0.15 mmol, 1.5 equiv.), *ᵗ*BuOK (16.8 mg, 0.15 mmol, 1.5 equiv.) and toluene (500 μL). The reaction mixture was then heated at 80 °C and stirred for 16 h. Upon completion, Boc_2O (1 M in THF, 100 μL, 1.5 mmol, 1.5 equiv.) was added and the reaction mixture was stirred at room temperature for another 1 h. Upon completion, the reaction was quenched with H_2O and extracted with ethyl acetate (3 × 3 mL). The combined organic phases were dried over $MgSO_4$, and the solvent was removed in vacuo. The resulting residue was purified by flash column chromatography to afford the desired product **6** (75%, 32.0 mg).

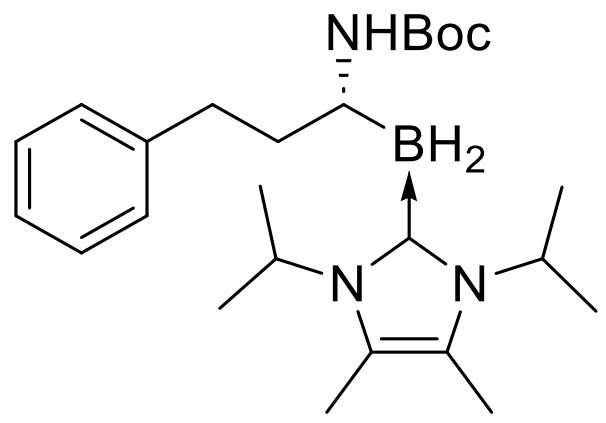

6

6: Yield: 32.0 mg, 75%, 97% ee, colorless oil, R_f = 0.4 (*n*-hexane/ethyl acetate = 3/1).
^{1}H NMR (400 MHz, $CDCl_3$) δ 7.24 – 7.18 (m, 4H), 7.14 – 7.06 (m, 1H), 5.60 – 5.27 (m, 2H), 4.45 – 4.25 (m, 1H), 3.05 – 2.88 (m, 1H), 2.86 – 2.58 (m, 2H), 2.20 (s, 6H), 1.81 – 1.67 (m, 2H), 1.46 – 1.40 (m, 12H), 1.36 (s, 9H).
^{13}C NMR (101 MHz, $CDCl_3$) δ 156.4, 144.6, 128.6, 128.1, 125.1, 124.0, 50.0, 41.0, 34.3, 28.7, 22.5, 21.5, 10.5.
^{11}B NMR (128 MHz, $CDCl_3$) δ -24.6 (t, J = 84.5 Hz, 1B).
HRMS (ESI): Calcd for $C_{25}H_{42}BN_3O_2Na^+$ $[M+Na]^+$: 450.3262, found: 450.3271.
IR (neat, cm^{-1}): ν 2971, 2924, 2852, 2301, 1703, 1484, 1390, 1171, 1136, 1024.
Chiral HPLC (AD-H, *ⁱ*PrOH/*n*-hexane = 1/99, 1.0 mL/min) t_R 12.5 min (major), 16.5 min (minor).
$[\alpha]_D^{25}$= -8.63 (c = 0.8, $CHCl_3$).

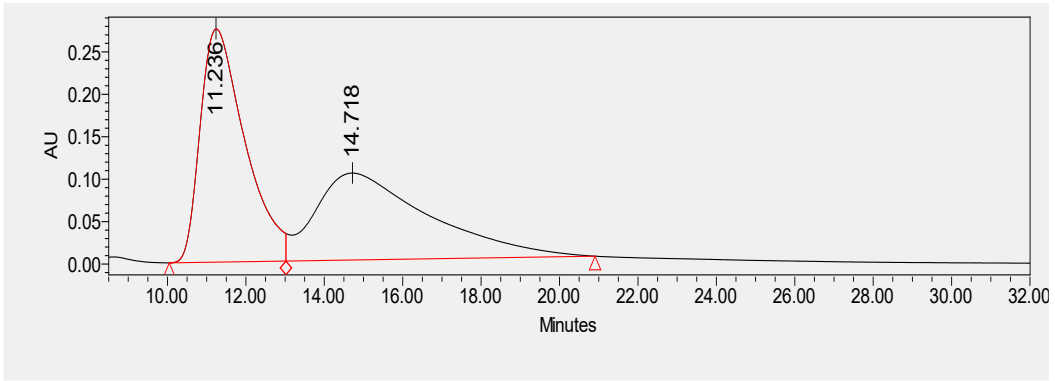

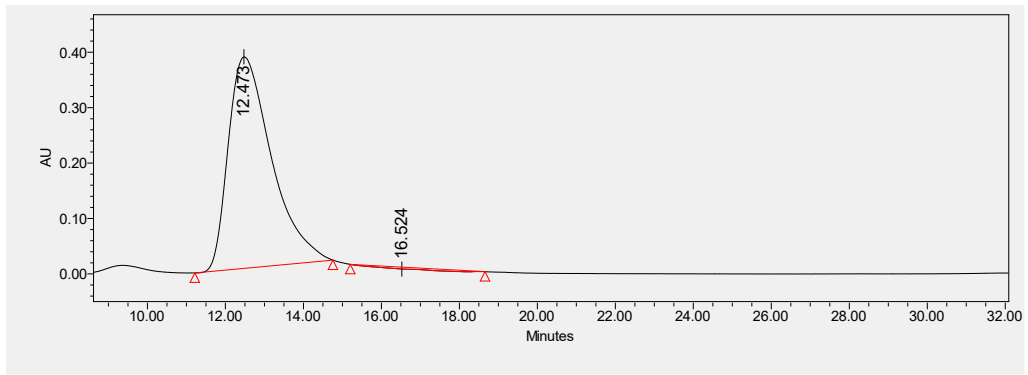

	Retention Time	Area	% Area
1	11.236	21724377	51.29
2	14.718	20630162	48.71

	Retention Time	Area	% Area
1	12.473	29406830	98.36
2	16.524	489919	1.64

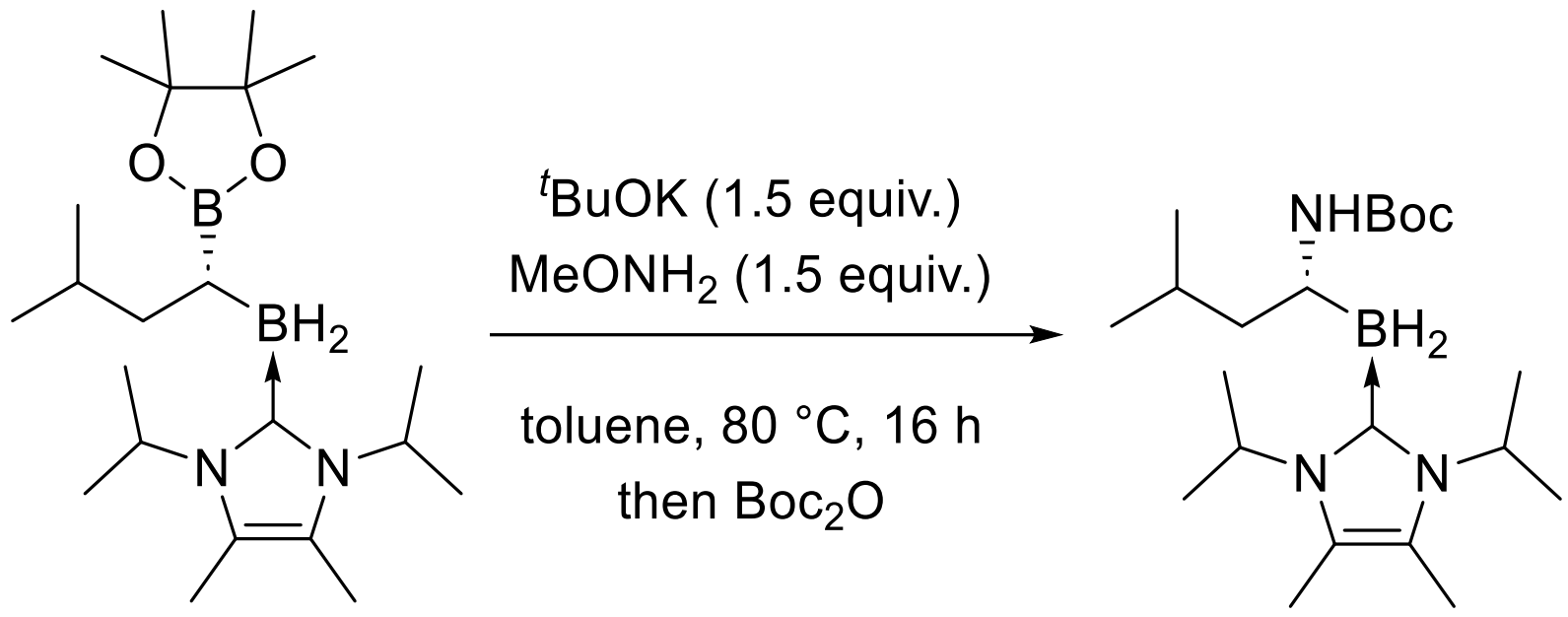

3y **7**

According to a reported literature,[16] under argon, to an oven dried 4 mL reaction tube equipped with magnetic stir bar was added **3y** (39.0 mg, 0.1 mmol, 1.0 equiv.), MeONH$_2$ (1.5 M in THF, 100 µL, 0.15 mmol, 1.5 equiv.), ^{t}BuOK (16.8 mg, 0.15 mmol, 1.5 equiv.) and toluene (500 µL). The reaction mixture was then heated at 80 °C and stirred for 16 h. Upon completion, Boc$_2$O (1 M in THF, 100 µL, 1.5 mmol, 1.5 equiv.) was added and the reaction mixture was stirred at room temperature for another 1 h. Upon completion, the reaction was quenched with H$_2$O and extracted with ethyl acetate (3 × 3 mL). The combined organic phases were dried over MgSO$_4$, and the solvent was removed in vacuo. The resulting residue was purified by flash column chromatography to afford the desired product **7** (72%, 27.3 mg).

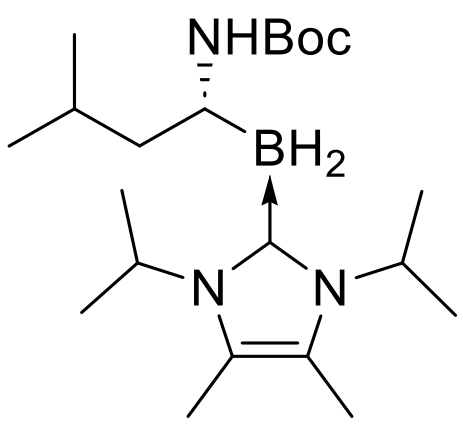

7

7: Yield: 27.3 mg, 72%, 97% ee, colorless oil, R_f = 0.4 (*n*-hexane/ethyl acetate = 3/1).
^{1}H NMR (500 MHz, CDCl$_3$) δ 5.78 – 5.16 (m, 2H), 4.27 – 3.97 (m, 1H), 3.01 – 2.90 (m, 1H), 2.56 – 1.96 (m, 6H), 1.70 – 1.64 (m, 1H), 1.56 – 1.38 (m, 12H), 1.32 (s, 9H), 1.16 – 1.04 (m, 1H), 1.04 – 0.81 (m, 7H).
^{13}C NMR (126 MHz, CDCl$_3$) δ 156.1, 123.9, 49.9, 49.4, 28.7, 28.4, 25.9, 23.7, 22.9, 22.4, 21.5, 21.5, 10.5.
^{11}B NMR (160 MHz, CDCl$_3$) δ -24.4 (t, *J* = 84.3 Hz, 1B).
HRMS (ESI): Calcd for $C_{21}H_{42}BN_3O_2Na^+$ [M+Na]$^+$: 402.3262, found: 402.3266.
IR (neat, cm^{-1}): *v* 2972, 2929, 2850, 2306, 1691, 1399, 1368, 1221, 1142.

Chiral HPLC (OX-H, ^{i}PrOH/n-hexane = 2/98, 1.0 mL/min) t_R 22.2 min (minor), 26.0 min (major).

$[\alpha]_D^{25}$= -10.17 (c = 0.6, $CHCl_3$).

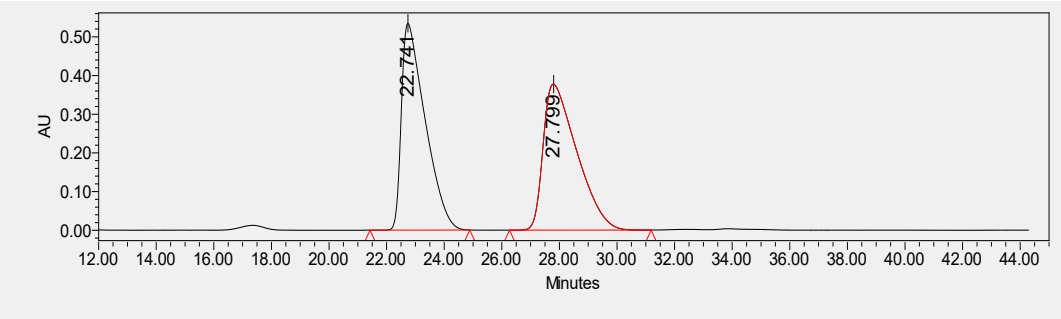

	Retention Time	Area	% Area
1	22.741	30551813	50.02
2	27.799	30529103	49.98

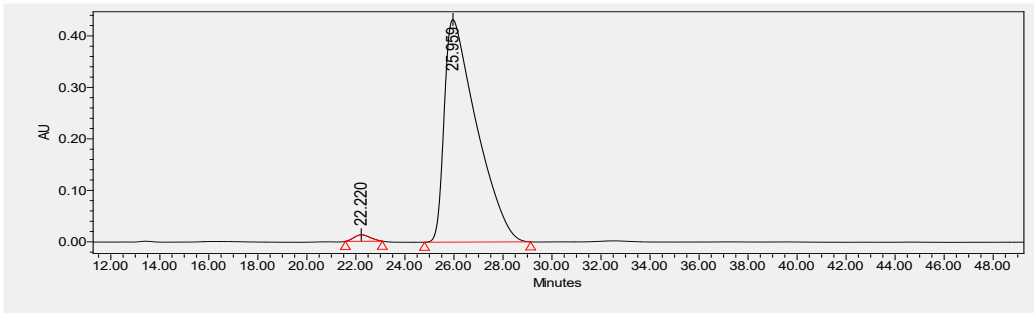

	Retention Time	Area	% Area
1	22.220	574754	1.44
2	25.959	39426913	98.56

Synthesis of compounds 8 and 9.

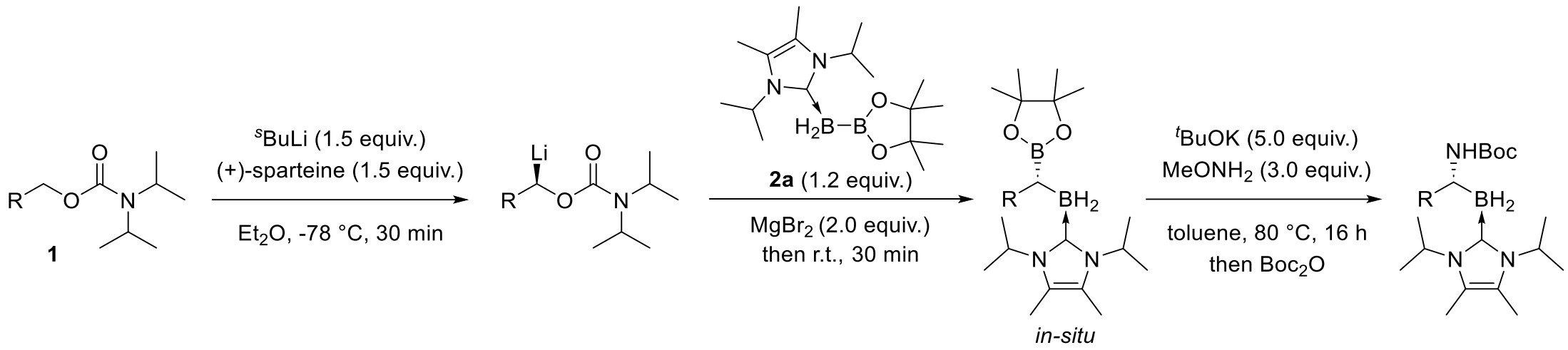

Under argon, to an oven dried 4 mL reaction tube equipped with a magnetic stir bar and a septum was added carbamate **1** (0.1 mmol, 1.0 equiv.), (+)-sparteine (35.2 mg, 0.15 mmol, 1.5 equiv.) and Et_2O (500 µL). The reaction was cooled to -78 °C and stirred for 10 min. ^{s}BuLi (1.3 M in cyclohexane, 0.12 mL, 0.15 mmol, 1.5 equiv.) was then slowly added and the mixture was stirred vigorously (without splashing) at -78 °C for 30 min. After that, a solution of **2a** (38.4 mg, 0.12 mmol, 1.2 equiv.) in Et_2O (1.2 mL) was added dropwise. The reaction mixture was stirred at this temperature for an additional 10 min. $MgBr_2$ (freshly made from Mg and 1,2-dibromoethane, 0.5 M in Et_2O, 0.4 mL, 0.2 mmol, 2.0 equiv.) was then added dropwise and the reaction mixture was stirred at -78 °C for 20 min. After that, the reaction mixture was warmed to room temperature and stirred for another 30 min. Upon completion, the reaction was quenched with aqueous NH_4Cl and extracted with ethyl acetate (3 × 3 mL). The combined organic phase was dried over $MgSO_4$, and the solvent was removed in vacuo. The resulting crude residue was used for the next step without purification.

According to a reported literature,[16] under argon, to an oven dried 4 mL reaction tube equipped with magnetic stir bar was added crude residual from the last step, $MeONH_2$ (1.5 M in THF, 200 µL, 0.3 mmol, 3.0 equiv.), ^{t}BuOK (56.1 mg, 0.5 mmol, 5.0 equiv.) and toluene (500 µL). The reaction mixture was then heated at 80 °C and stirred for 12 h. Upon completion, Boc_2O (1 M in THF, 3.0 mmol, 3.0 equiv.) was added and the reaction mixture was stirred at room temperature for another 1 h. Upon completion, the reaction was quenched with H_2O and extracted with ethyl acetate (3 × 3 mL). The combined organic phases were dried over $MgSO_4$, and the solvent was removed in vacuo. The resulting crude residue was purified by flash column chromatography to afford the desired product. The racemic product was prepared using the same procedure, with *N,N,N′,N′*-tetramethylethylenediamine (TMEDA) substituted for (+)-sparteine.

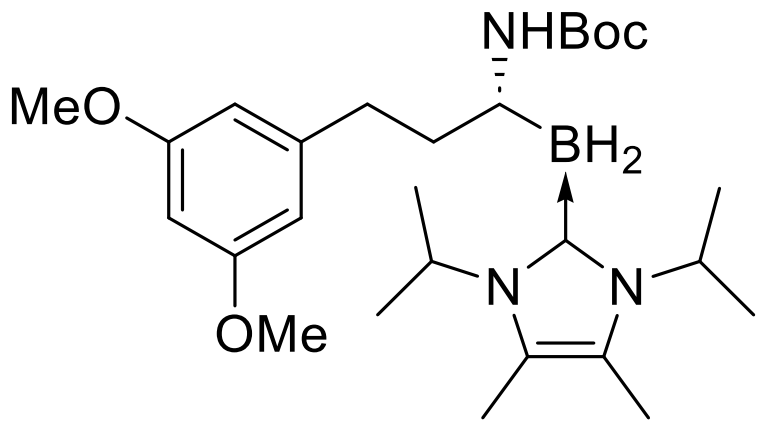

8

8: Yield: 38.0 mg, 78%, 98% ee, colorless oil, R_f = 0.4 (*n*-hexane/ethyl acetate = 3/1).
^{1}H NMR (500 MHz, $CDCl_3$) δ 6.75 – 6.33 (m, 2H), 6.33 – 6.18 (m, 1H), 5.53 – 5.32 (m, 2H), 4.48 – 4.26 (m, 1H), 3.76 (s, 6H), 3.03 – 2.86 (m, 1H), 2.83 – 2.51 (m, 2H), 2.21 (s, 6H), 1.76 – 1.58 (m, 2H), 1.50 – 1.39 (m, 12H), 1.36 (s, 9H).
^{13}C NMR (126 MHz, $CDCl_3$) δ 160.6, 156.4, 147.1, 124.1, 106.5, 97.7, 55.4, 50.0, 40.8, 34.7, 28.7, 21.6, 21.6, 10.6.
^{11}B NMR (160 MHz, $CDCl_3$) δ -24.9 (t, *J* = 110.4 Hz, 1B).
Note: After column chromatography, preparative thin-layer chromatography (PTLC) was employed to remove trace amounts of impurities to ensure the highest purity for the NMR spectra.
HRMS (ESI): Calcd for $C_{27}H_{46}BN_3O_4Na^+$ $[M+Na]^+$: 510.3474, found: 510.3484.
IR (neat, cm^{-1}): *ν* 2925, 2854, 2301, 1702, 1595, 1462, 1259, 1203, 1150, 1019.
Chiral HPLC (IC, ^{i}PrOH/*n*-hexane = 10/90, 1.0 mL/min) t_R 25.6 min (minor), 40.7 min (major).
$[\alpha]_D^{25}$ = 6.00 (*c* = 0.4, $CHCl_3$).

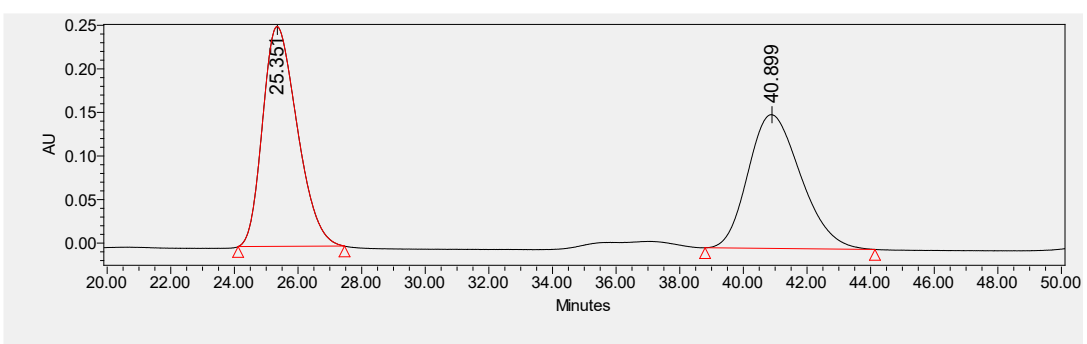

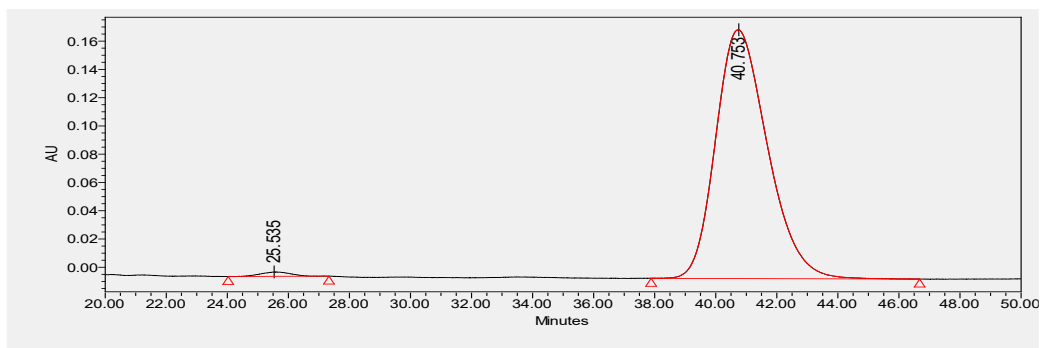

	Retention Time	Area	% Area
1	25.351	19015990	51.85
2	40.899	17656139	48.15

	Retention Time	Area	% Area
1	25.535	211876	1.01
2	40.753	20744252	98.99

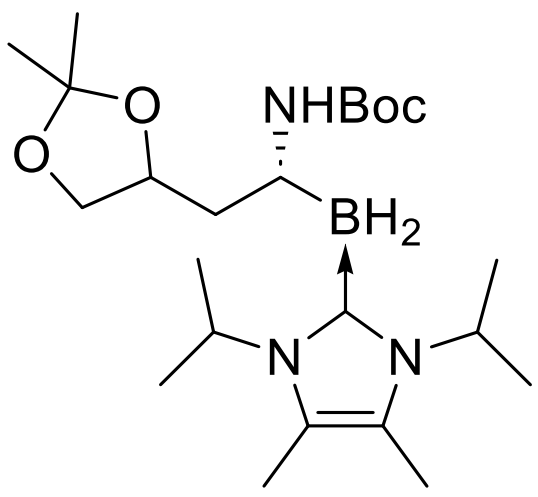

9

A mixture of geometrical isomers in ~ 1.5:1 ratio
9: Yield: 27.1 mg, 62%, 92% ee, 1.5:1 d.r., colorless oil, R_f = 0.4 (*n*-hexane/ethyl acetate = 3/1).
^{1}H NMR (500 MHz, $CDCl_3$) δ 5.51 – 5.30 (m, 2H), 4.36 – 4.17 (m, 2.4H), 4.15 – 4.01 (m, 0.6H), 3.59 – 3.48 (m, 1H), 2.93 (s, 0.4H), 2.82 (s, 0.6H), 2.34 (s, 1H), 2.21 (M, 6H), 1.89 – 1.74 (m, 1H), 1.52 – 1.41 (m, 12H), 1.39 – 1.31 (m, 15H).

^{13}C NMR (126 MHz, $CDCl_3$) δ 156.5, 156.2, 125.6, 124.2, 107.8, 107.3, 75.7, 70.5, 70.3, 51.4, 50.0, 43.3, 42.7, 28.7, 28.7, 27.3, 27.2, 26.0, 25.8, 22.5, 21.6, 21.6, 21.6, 21.5, 10.6, 10.5, 8.9.
^{11}B NMR (160 MHz, $CDCl_3$) δ -24.7 (t, J = 84.8 Hz, 1B).
HRMS (ESI): Calcd for $C_{23}H_{44}BN_3O_4Na^+$ $[M+Na]^+$: 460.3312, found: 460.3326.
IR (neat, cm^{-1}): v 2920, 2356, 1705, 1484, 1258, 1173, 1057.
Chiral HPLC (IC, ^{i}PrOH/n-hexane = 10/90, 1.0 mL/min) t_R 19.9 min (minor), 44.5 min (major).
$[\alpha]_D^{25}$= -15.33 (c = 0.3, $CHCl_3$).

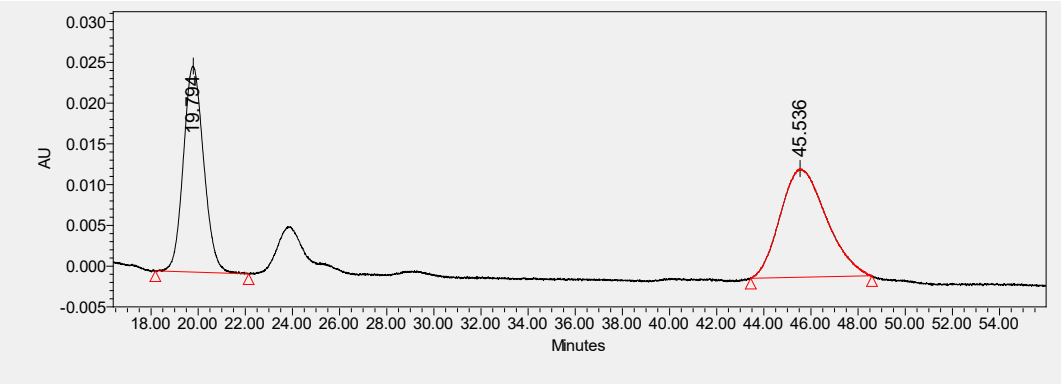

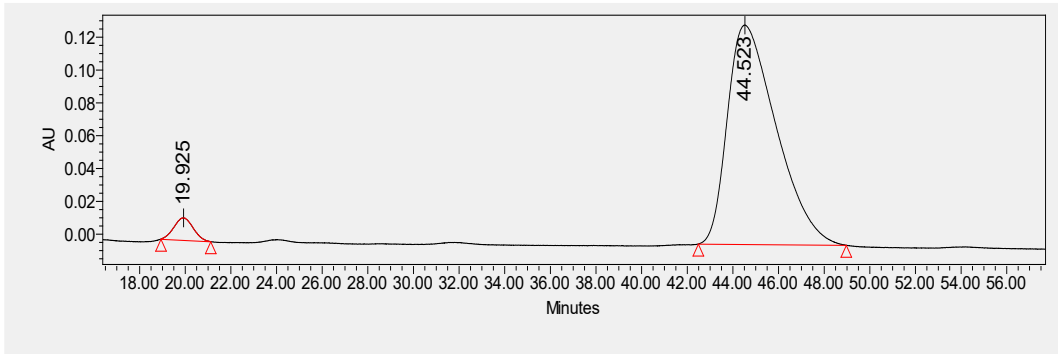

	Retention Time	Area	% Area
1	19.794	1419558	48.52
2	45.536	1506330	51.48

	Retention Time	Area	% Area
1	19.925	811049	3.92
2	44.523	19877054	96.08

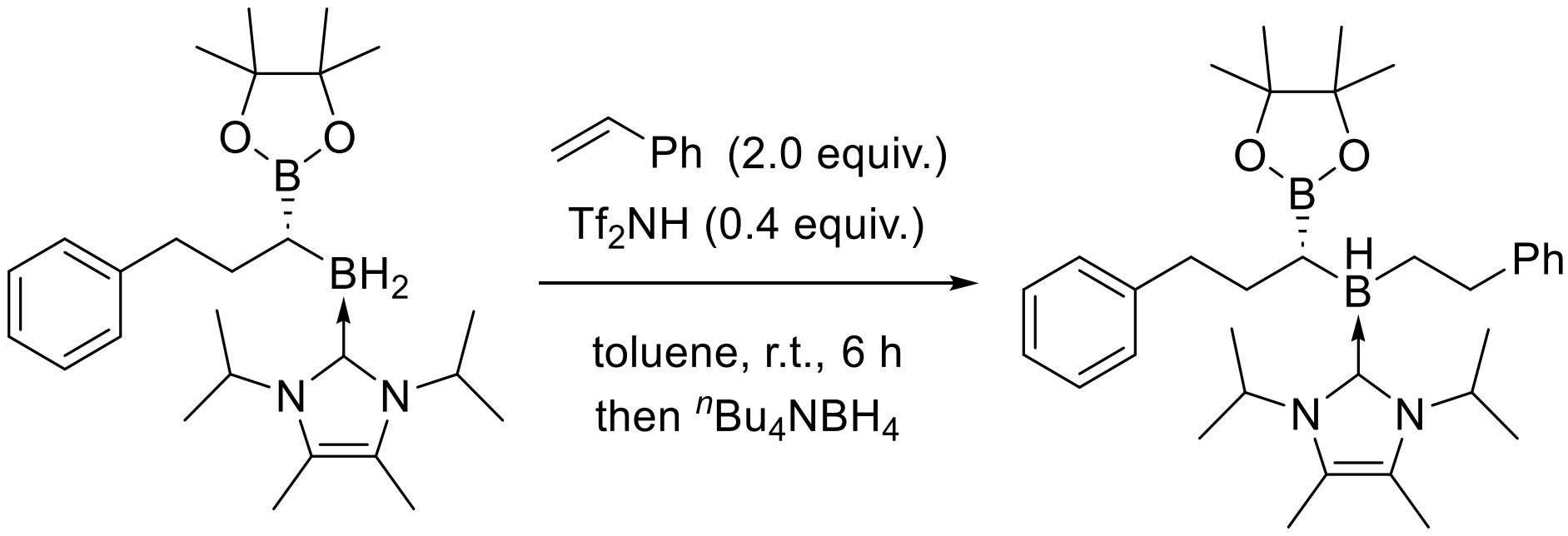

3a, 98% ee **10**

Under argon, to an oven dried 4 mL reaction tube charged with magnetic stir bar was added **3a** (43.8 mg, 0.1 mmol, 1.0 equiv.), Tf_2NH (11.2 mg, 0.04 mmol, 0.4 equiv.), styrene (20.8 mg, 0.2 mmol, 2.0 equiv.) and toluene (500 μL). The reaction mixture was then stirred at room temperature for 6 h. Upon completion, the reaction was quenched with $^n Bu_4NBH_4$ (10.3 mg, 0.04 mmol, 0.4 equiv.). The reaction mixture was then passed through a short pad of silica gel, washed with ethyl acetate and concentrated in vacuo. The resulting residue was purified by flash column chromatography to afford the desired product **10** (68%, 36.9 mg).

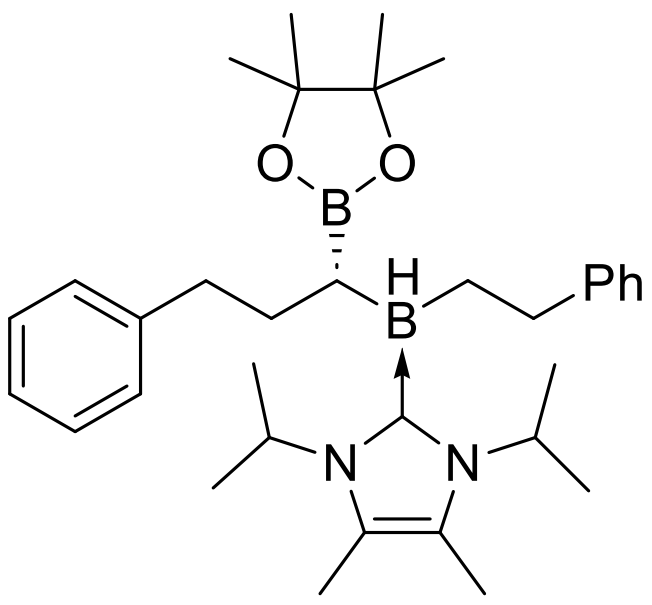

10

A mixture of diastereomers in ~ 1:1 ratio

10: Yield: 36.9 mg, 68%, 1:1 d.r., colorless oil, $R_f = 0.4$ (*n*-hexane/ethyl acetate = 4/1).
^{1}H NMR (400 MHz, $CDCl_3$) δ 7.23 – 6.94 (m, 10H), 6.25 – 5.78 (m, 1H), 5.53 – 4.97 (m, 1H), 2.85 – 2.64 (m, 1H), 2.63 – 2.34 (m, 2H), 2.32 – 2.01 (m, 6H), 2.09 – 1.73 (m, 3H), 1.52 – 1.34 (m, 12H), 1.30 (d, J = 8.3 Hz, 6H), 1.19 (s, 3H), 1.04 (s, 3H), 0.95 – 0.85 (m, 1H), 0.74 – 0.63 (m, 0.5H), 0.61 – 0.47 (m, 0.5H), 0.32 – 0.20 (m, 0.5H), 0.18 – 0.07 (m, 0.5H).
^{13}C NMR (101 MHz, $CDCl_3$) δ 148.8, 148.6, 145.1, 144.8, 128.7, 128.7, 128.0, 128.0, 128.0, 127.9, 125.0, 125.0, 124.5, 124.4, 81.7, 81.2, 49.3, 48.8, 48.7, 40.9, 40.3, 38.6, 38.6, 35.5, 34.8, 25.6, 25.4, 25.0, 24.3, 21.8, 21.7, 21.3, 10.9, 10.7, 10.5.
^{11}B NMR (128 MHz, $CDCl_3$) δ 37.1 (s, 1B), -16.5 (d, J = 86.4 Hz, 0.5B), -18.2 (d, J = 84.3 Hz, 0.5B).
HRMS (ESI): Calcd for $C_{34}H_{52}B_2N_2O_2Na^+$ $[M+Na]^+$: 565.4107, found: 565.4109.
IR (neat, cm^{-1}): *ν* 3024, 2974, 2923, 2851, 1452, 1368, 1287, 1145, 1057.
Chiral HPLC (ODH, ^{i}PrOH/*n*-hexane = 2/98, 1.0 mL/min) t_R 4.4 min (major), 4.8 min (minor), 5.8 min (major), 6.7 min (minor).
$[\alpha]_D^{25}$= -0.85 (c = 0.8, $CHCl_3$)

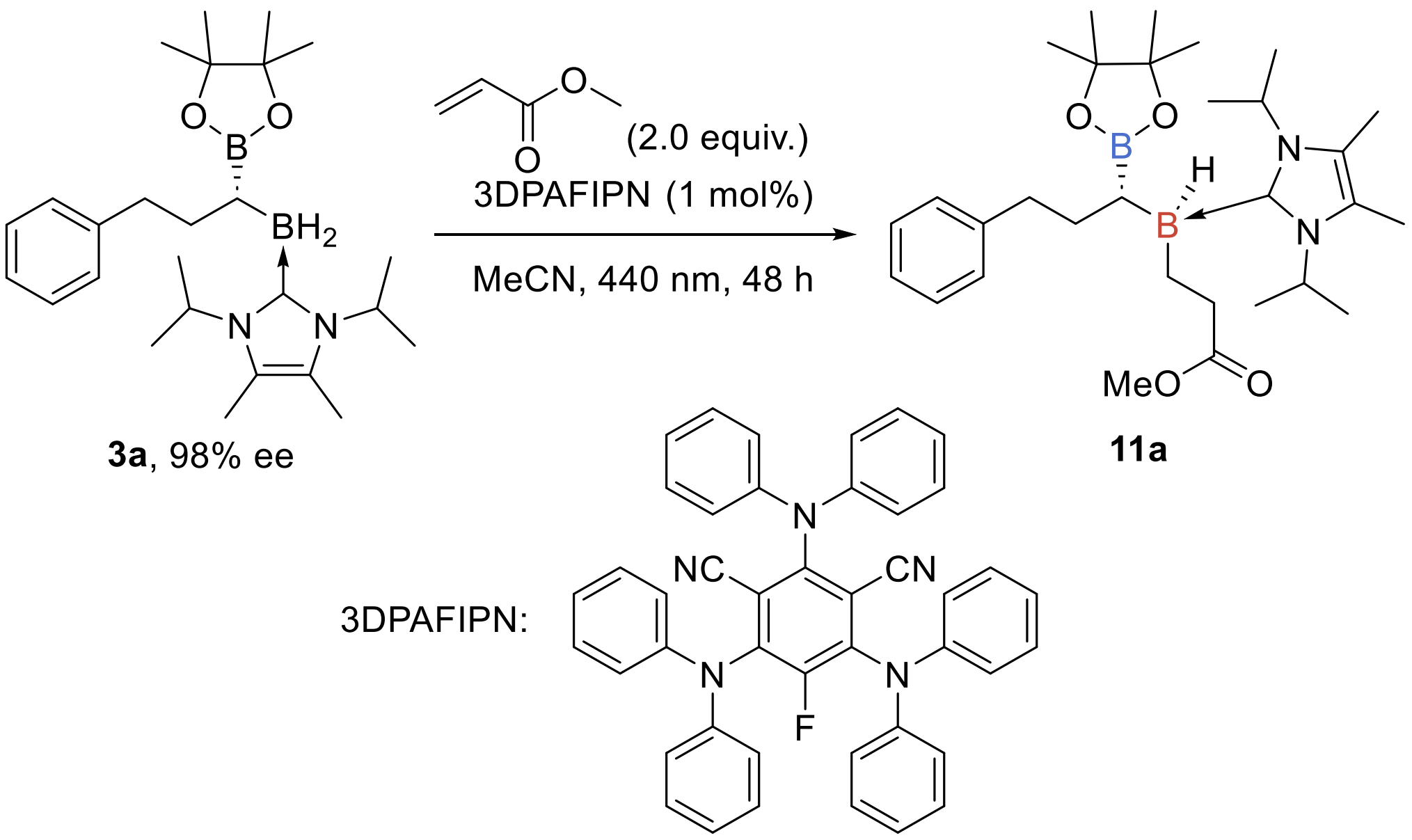

According to a reported literature,[17] under argon, to an oven dried 4 mL vial charged with magnetic stir bar was added **3a** (43.8 mg, 0.1 mmol, 1.0 equiv.), 3DPAFIPN (3.2 mg, 0.001 mmol, 1 mol%), methyl acrylate (17.2 mg, 0.2 mmol, 2.0 equiv.) and MeCN (2 mL). The reaction mixture was irradiated under 440 nm at room temperature for 48 h. Upon completion, the reaction mixture was then passed through a short pad of silica, washed with ethyl acetate and concentrated in vacuo. The resulting residue was purified by flash column chromatography to afford the desired product **11** (49%, 25.7 mg).

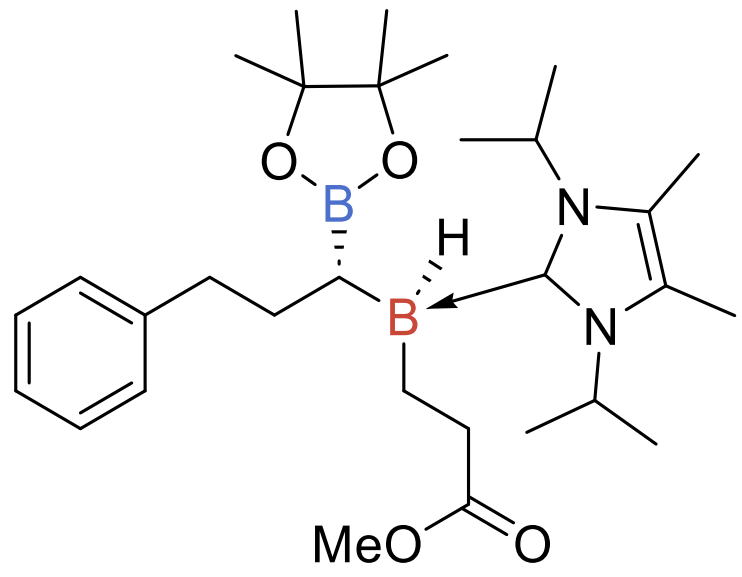

11a

11a: 7:1 d.r. from crude ^{1}H NMR, isolated yield: 25.7 mg, 49%, 98% ee, >20:1 d.r., colorless oil, R_f = 0.4 (*n*-hexane/ethyl acetate = 2/1).

^{1}H NMR (400 MHz, $CDCl_3$) δ 7.24 – 7.16 (m, 4H), 7.13 – 7.05 (m, 1H), 5.96 – 5.63 (m, 1H), 5.30 – 4.95 (m, 1H), 3.56 (s, 3H), 2.75 – 2.62 (m, 1H), 2.51 – 2.36 (m, 1H), 2.24 – 2.07 (m, 7H), 1.85 – 1.70 (m, 2H), 1.68 – 1.62 (m, 1H), 1.49 – 1.39 (m, 12H), 1.17 (s, 6H), 1.07 – 0.99 (m, 7H), 0.63 – 0.40 (m, 1H), 0.20 – 0.05 (m,1H).

^{13}C NMR (101 MHz, $CDCl_3$) δ 178.2, 145.0, 128.7, 128.0, 125.0, 124.1, 81.3, 51.1, 49.4, 40.8, 36.7, 34.7, 25.4, 24.3, 22.1, 10.7.

^{11}B NMR (128 MHz, $CDCl_3$) δ 36.3 (s, 1B), -18.5 (d, J = 83.7 Hz, 1B).

Note: After column chromatography, preparative thin-layer chromatography (PTLC) was employed to remove trace amounts of impurities to ensure the highest purity for the NMR spectra.

HRMS (ESI): Calcd for $C_{30}H_{50}B_2N_2O_4Na^+$ [M+Na]$^+$: 547.3849, found: 547.3859.

IR (neat, cm^{-1}): ν 2975, 2925, 2851, 2310, 1729, 1557, 1495, 1386, 1217, 1145.

Chiral HPLC (IC, ^{i}PrOH/*n*-hexane = 2/98, 1.0 mL/min) t_R 20.6 min (minor), 23.2 min (minor), 26.1 min (major), 38.0 min (minor).

$[\alpha]_D^{25}$= -24.00 (c = 0.3, $CHCl_3$).

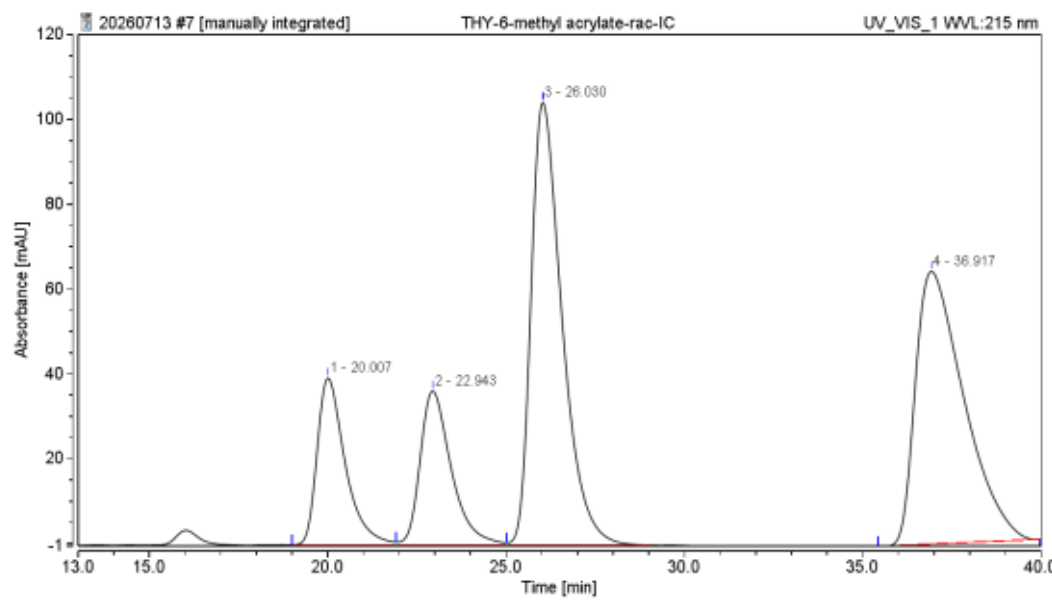

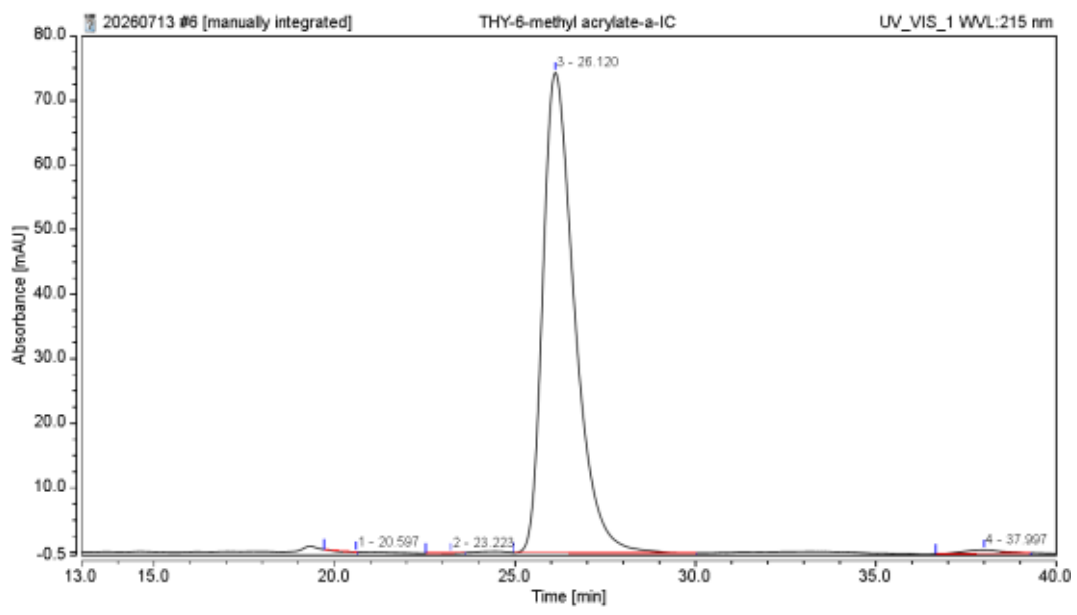

	Retention Time	% Area	Area
1	20.007	35.9273	12.88
2	22.943	36.7952	13.19
3	26.03	105.3738	37.77
4	36.917	100.872	36.16

	Retention Time	% Area	Area
1	20.597	0.0641	0.08
2	23.223	0.0218	0.03
3	26.120	75.545	99.01
4	37.997	0.6719	0.88

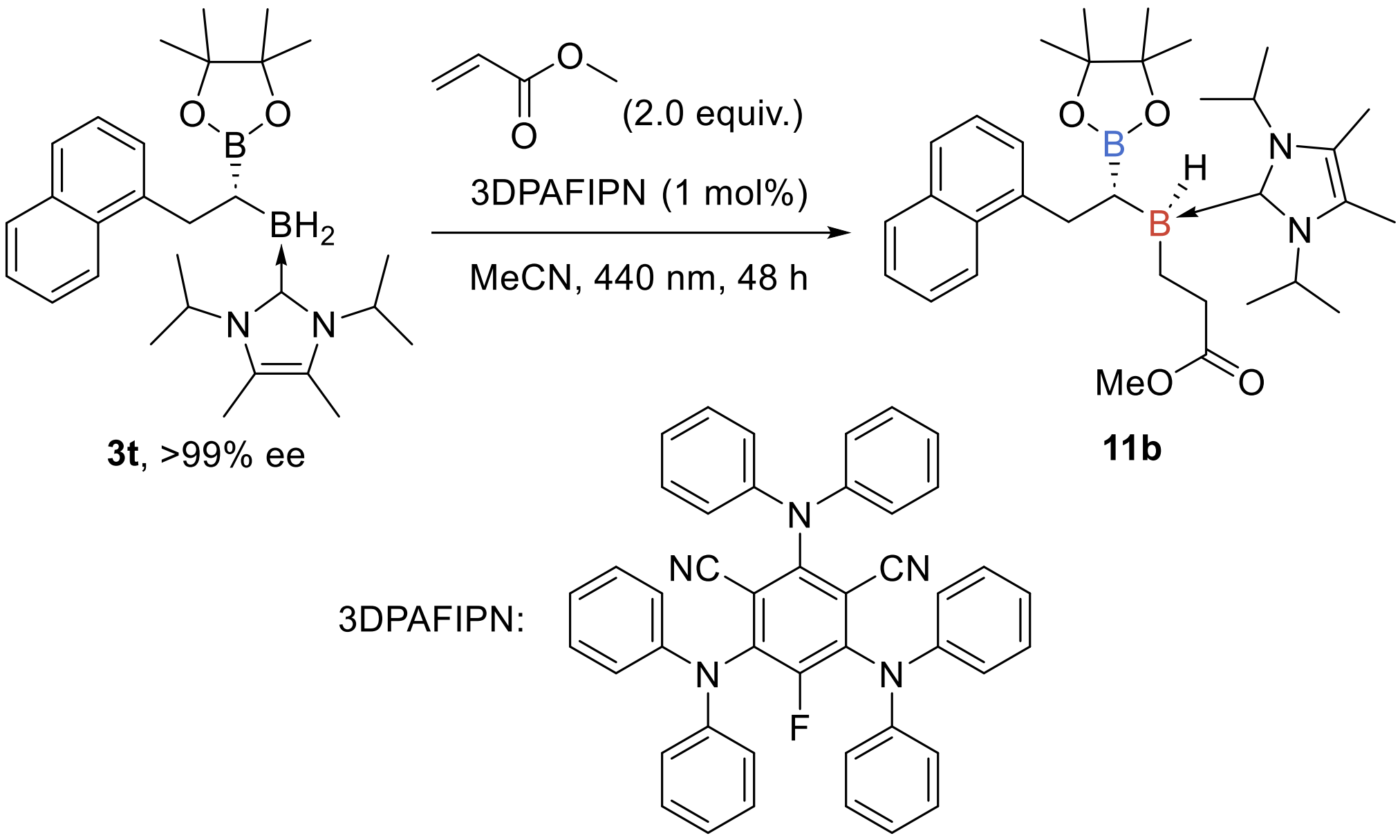

According to a reported literature,[17] under argon, to an oven dried 4 mL vial charged with magnetic stir bar was added **3t** (47.4 mg, 0.1 mmol, 1.0 equiv.), 3DPAFIPN (3.2 mg, 0.001 mmol, 1 mol%), methyl acrylate (17.2 mg, 0.2 mmol, 2.0 equiv.) and MeCN (2 mL). The reaction mixture was irradiated under 440 nm at room temperature for 48 h. Upon completion, the reaction mixture was then passed through a short pad of silica, washed with ethyl acetate and concentrated in vacuo. The resulting residue was purified by flash column chromatography to afford the desired product **11b** (51%, 28.6 mg).

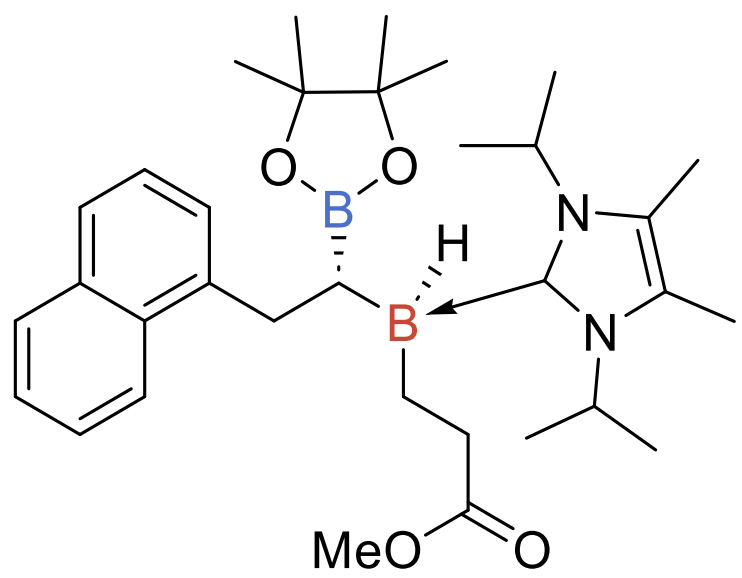

11b

11b: 14:1 d.r. from crude ^{1}H NMR, isolated yield: 28.6 mg, 51%, 99% ee, >20:1 d.r., white solid, R_f = 0.4 (*n*-hexane/ethyl acetate = 2/1).
^{1}H NMR (400 MHz, $CDCl_3$) δ 8.37 – 8.11 (m, 1H), 7.86 – 7.71 (m, 1H), 7.64 – 7.53 (m, 1H), 7.53 – 7.36 (m, 3H), 7.32 – 7.28 (m, 1H), 6.06 – 5.90 (m, 1H), 5.40 – 5.09 (m, 1H), 3.64 (s, 3H), 3.55 – 3.28 (m, 1H), 3.29 – 2.89 (m, 1H), 2.40 – 2.19 (m, 7H), 1.80 – 1.64 (m, 2H), 1.54 – 1.39 (m, 12H), 0.98 (s, 6H), 0.82 – 0.74 (m, 7H), 0.69 – 0.56 (m, 1H).
^{13}C NMR (101 MHz, $CDCl_3$) δ 178.2, 144.3, 133.9, 132.6, 128.4, 125.6, 125.5, 125.2, 125.0, 124.9, 124.5, 124.3, 81.2, 51.3, 49.8, 49.3, 36.8, 35.1, 25.4, 24.2, 22.2, 18.5, 10.6.
^{11}B NMR (128 MHz, $CDCl_3$) δ 35.6 (s, 1B), -17.45 (d, J = 84.8 Hz, 1B).
HRMS (ESI): Calcd for $C_{33}H_{50}B_2N_2O_4Na^+$ $[M+Na]^+$: 583.3849, found: 583.3854.
Chiral HPLC (ODH, ^{i}PrOH/*n*-hexane = 1/99, 1.0 mL/min) t_R 13.9 min (minor), 16.2 min (major).

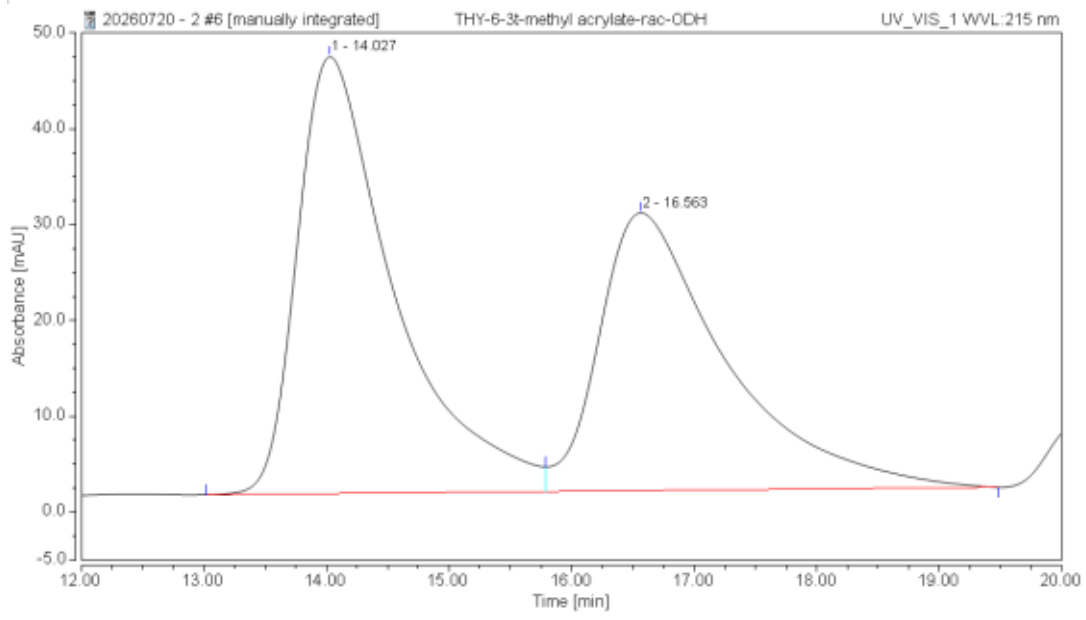

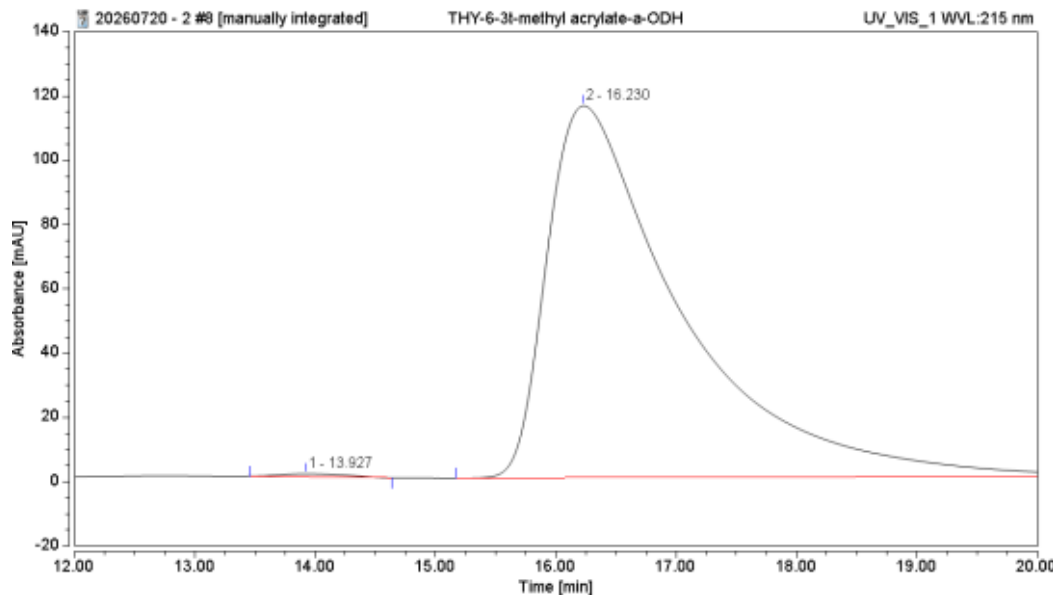

	Retention Time	Area	% Area
1	14.027	38.7341	52.32
2	16.563	35.3004	47.68

	Retention Time	Area	% Area
1	13.927	0.6005	0.40
2	16.230	149.8622	99.60

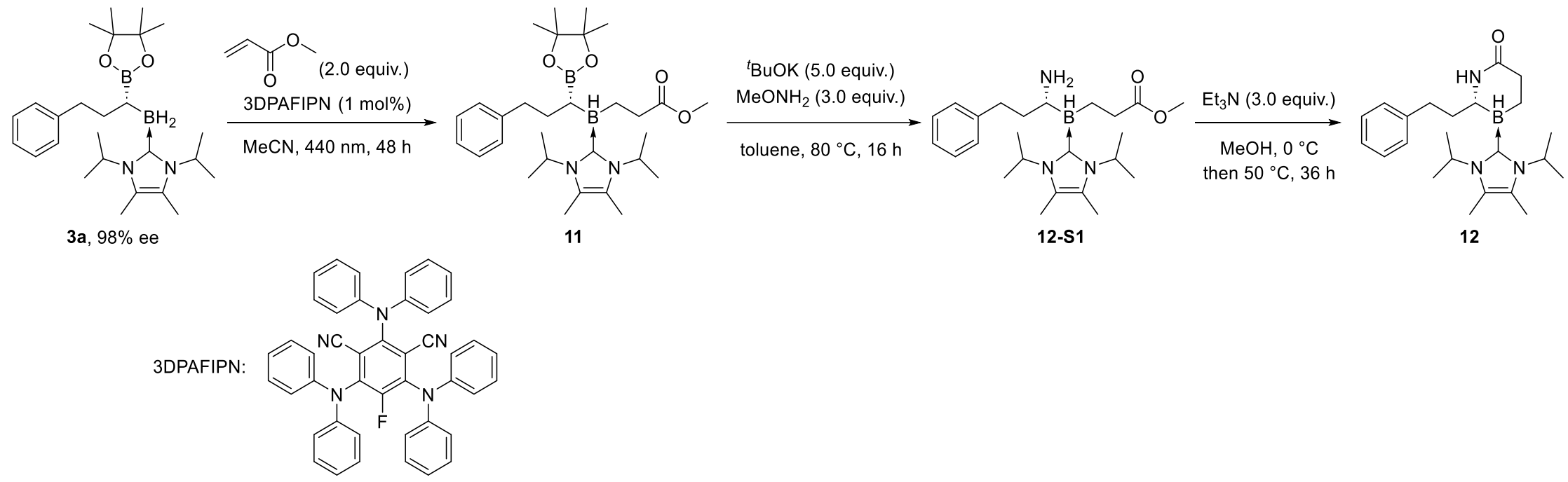

Under argon, to an oven dried 4 mL vial charged with magnetic stir bar was added **3a** (43.8 mg, 0.1 mmol, 1.0 equiv.), 3DPAFIPN (3.2 mg, 0.001 mmol, 1 mol%), methyl acrylate (17.2 mg, 0.2 mmol, 2.0 equiv.) and MeCN (2 mL). The reaction mixture was irradiated under 440 nm at room temperature for 48 h. Upon completion, the reaction mixture was then passed through a short pad of silica, washed with ethyl acetate and concentrated in vacuo. The resulting crude residue **11** was used for the next step without purification.

Under argon, to an oven dried 4 mL reaction tube equipped with magnetic stir bar was added crude residual **11** from the last step, $MeONH_2$ (1.5 M in THF, 200 µL, 0.3 mmol, 3.0 equiv.), ^{t}BuOK (56.1 mg, 0.5 mmol, 5.0 equiv.) and toluene (500 µL). The reaction mixture was then heated at 80 °C and stirred for 12 h. Upon completion, the reaction was quenched with H_2O and extracted with ethyl acetate (3 × 3 mL). The combined organic phases were dried over $MgSO_4$, and the solvent was removed in vacuo. The resulting crude residue **12-S1** was used for the next step without purification.

Under argon, to an oven dried 4 mL reaction tube charged with magnetic stir bar was added crude reaction mixture **12-S1** from the last step and MeOH (1 mL). The reaction was cooled to 0 °C and Et_3N (30.4 mg, 0.3 mmol, 3.0 equiv.) was added slowly. The reaction mixture was then heated at 50 °C and stirred for 36 h. Upon completion, the reaction was quenched with H_2O and extracted with ethyl acetate (3 × 3 mL). The combined organic phases were dried over $MgSO_4$, and the solvent was removed in vacuo. The crude sample was purified by flash column chromatography to afford the desired product **12** (18%, 6.8 mg).

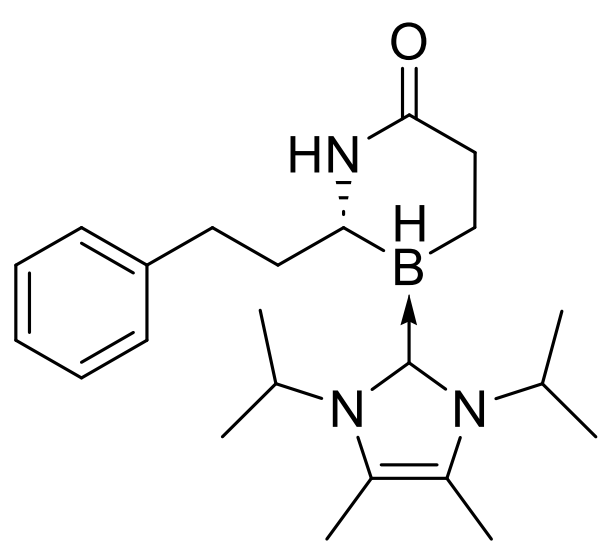

12

A mixture of diastereomers in ~ 1:1 ratio

12: Yield: 6.8 mg, 18%, 1:1 d.r., colorless oil, R_f = 0.4 (*n*-hexane/ethyl acetate = 1/3).

^{1}H NMR (400 MHz, $CDCl_3$) δ 7.23 – 7.14 (m, 2H), 7.15 – 7.04 (m, 3H), 6.17 (s, 0.5H), 5.97 (s, 0.5H), 5.57 – 5.20 (m, 2H), 2.85 – 2.75 (m, 0.5H), 2.72 – 2.63 (m, 1H), 2.61 – 2.50 (m, 1H), 2.49 – 2.39 (m, 1H), 2.39 – 2.26 (m, 1.5H), 2.26 – 2.19 (m, 6H), 1.83 – 1.65 (m, 2H), 1.47 – 1.36 (m, 6H), 1.37 – 1.30 (m, 6H), 0.85 – 0.76 (m, 1H), 0.69 – 0.54 (m, 1H).

^{13}C NMR (101 MHz, $CDCl_3$) δ 178.7, 178.0, 143.0, 142.9, 128.5, 128.4, 128.4, 128.3, 125.7, 125.6, 124.7, 49.1, 42.5, 38.0, 36.2, 34.9, 34.1, 33.6, 22.0, 21.4, 10.7.

^{11}B NMR (128 MHz, $CDCl_3$) δ -19.2 (d, *J* = 81.2 Hz, 1B).

HRMS (ESI): Calcd for $C_{23}H_{36}BN_3ONa^+$ $[M+Na]^+$: 404.2844, found: 404.2847.

IR (neat, cm^{-1}): ν 3285, 2922, 2852, 2358, 1672, 1622, 1495, 1390, 1217, 1195.

$[\alpha]_D^{25}$= -28.00 (*c* = 0.1, $CHCl_3$).

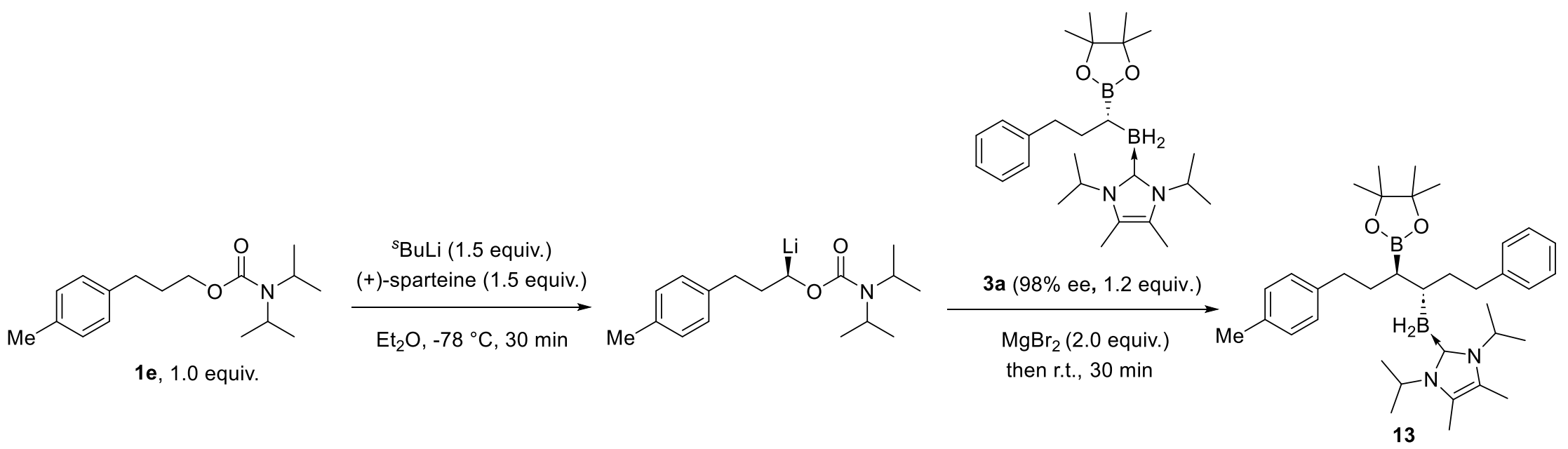

Under argon, to an oven dried 4 mL reaction tube equipped with a magnetic stir bar and a septum was charged with **1e** (27.7 mg, 0.1 mmol, 1.0 equiv.), (+)-sparteine (35.2 mg, 0.15 mmol, 1.5 equiv.) and Et_2O (500 μL). The reaction was cooled to -78 °C and stirred for 10 min. ^{s}BuLi (1.3 M in cyclohexane, 0.12 mL, 0.15 mmol, 1.5 equiv.) was then slowly added and the mixture was stirred vigorously (without splashing) at -78 °C for 30 min. After that, a solution of the **3a** (52.6 mg, 0.12 mmol, 1.2 equiv.) in Et_2O (1.2 mL) was added dropwise. The reaction mixture was stirred at this temperature for an additional 10 min. $MgBr_2$ (freshly made from Mg and 1,2-dibromoethane, 0.5 M in Et_2O, 0.4 mL, 0.2 mmol, 2.0 equiv.) was then added dropwise and the reaction mixture was stirred at -78 °C for 20 min. The reaction mixture was then warmed to room temperature and stirred for another 30 min. Upon completion, the reaction was quenched with aqueous NH_4Cl and extracted with Et_2O (3 × 3 mL). The combined organic phases were dried over $MgSO_4$, and the solvent was removed in vacuo. The resulting residue was purified by flash column chromatography to afford the desired product **13** (36%, 20.5 mg).

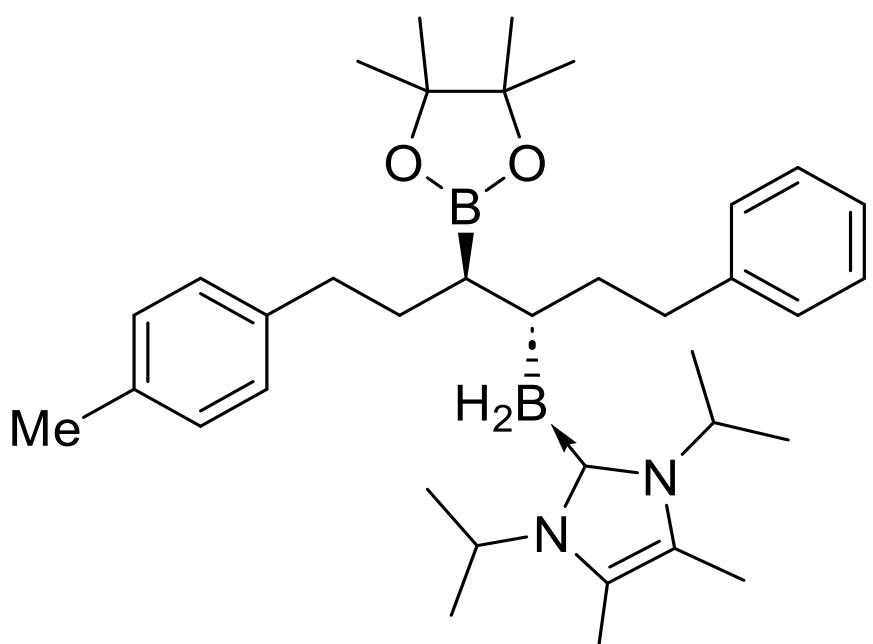

13

13: Yield: 20.5 mg, 36%, >99% ee, >20:1 d.r., colorless oil, R_f = 0.5 (*n*-hexane/ethyl acetate = 3/1).

^{1}H NMR (400 MHz, $CDCl_3$) δ 7.22 – 7.15 (m, 2H), 7.15 – 6.95 (m, 7H), 5.75 – 5.45 (m, 2H), 2.72 – 2.54 (m, 2H), 2.47 – 2.35 (m, 2H), 2.28 (s, 3H), 2.18 (s, 6H), 2.04 – 1.86 (m, 1H), 1.83 – 1.71 (m, 1H), 1.56 – 1.48 (m, 1H), 1.40 – 1.33 (m, 6H), 1.33 – 1.24 (m, 19H), 1.16 – 1.08 (m, 1H), 0.89 – 0.72 (m, 1H).

^{13}C NMR (101 MHz, $CDCl_3$) δ 145.4, 141.6, 134.4, 128.8, 128.7, 128.6, 127.9, 124.8, 123.6, 82.2, 49.4, 36.5, 36.4, 36.2, 30.9, 25.4, 25.2, 21.8, 21.5, 21.1, 10.6.

^{11}B NMR (128 MHz, $CDCl_3$) δ 34.8 (s, 1B), -25.2 (t, *J* = 85.5 Hz, 1B).

HRMS (ESI): Calcd for $C_{36}H_{56}B_2N_2O_2Na^+$ $[M+Na]^+$: 593.4420, found: 593.4416.

IR (neat, cm^{-1}): *ν* 2974, 2929, 2851, 2271, 1495, 1387, 1303, 1145, 1108.

Chiral HPLC (IG, ^{i}PrOH/*n*-hexane = 3/97, 1.0 mL/min) t_R 5.1 min (minor), 5.8 min (major), 6.5 min (minor), 7.4 min (minor).

$[\alpha]_D^{25}$= -8.00 (*c* = 0.4, $CHCl_3$).

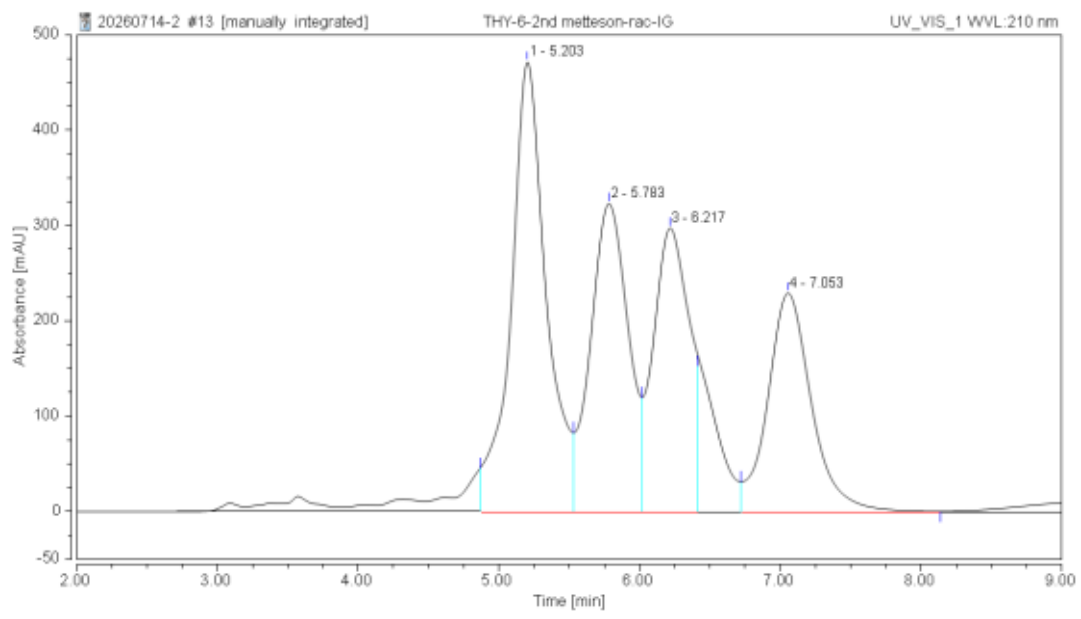

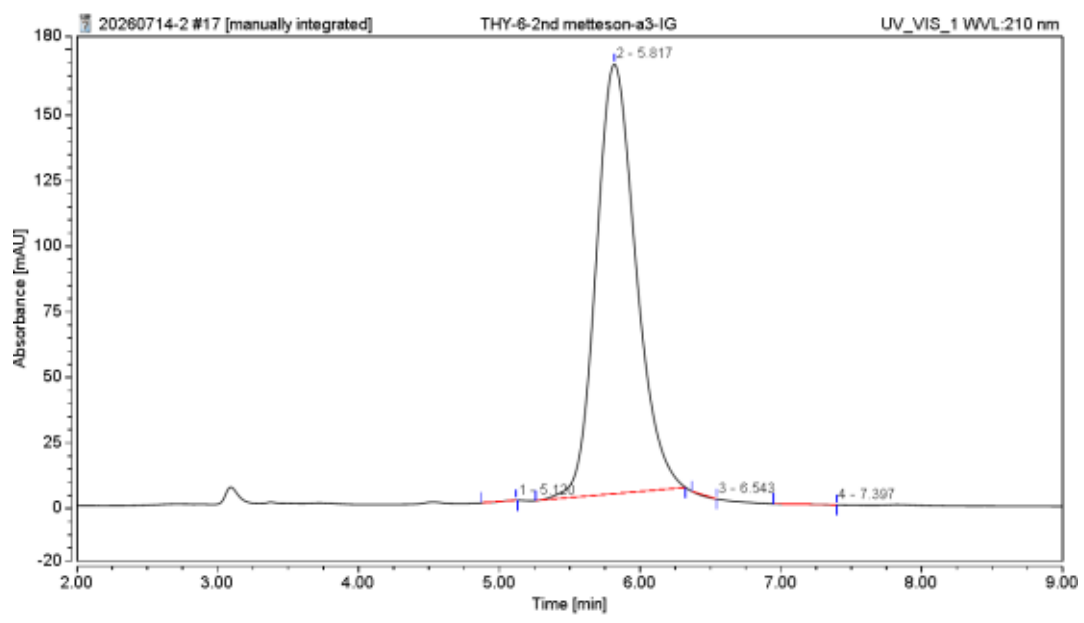

	Retention Time	% Area	Area
1	5.203	137.2315	33.62
2	5.783	100.6751	24.67
3	6.217	87.5154	21.44
4	7.053	82.7305	20.27

	Retention Time	% Area	Area
1	5.120	0.0047	0.01
2	5.817	53.5351	99.90
3	6.543	0.045	0.08
4	7.397	0.0033	0.01

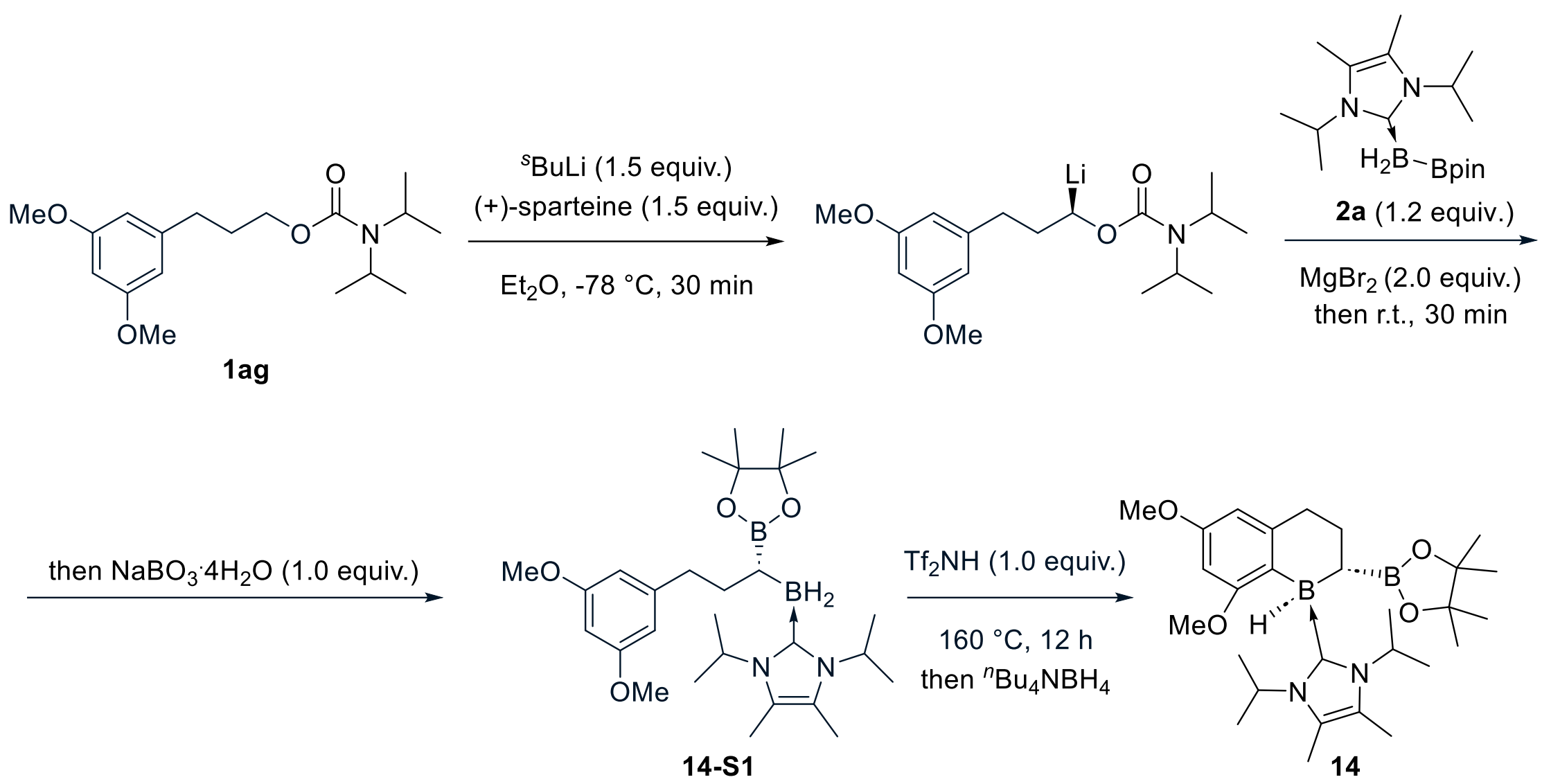

Under argon, to an oven dried 4 mL reaction tube equipped with a magnetic stir bar and a septum was charged with **1ag** (32.3 mg, 0.1 mmol, 1.0 equiv.), (+)-sparteine (35.2 mg, 0.15 mmol, 1.5 equiv.) and Et_2O (500 µL). The reaction was cooled to -78 °C and stirred for 10 min. ^{s}BuLi (1.3 M in cyclohexane, 0.12 mL, 0.15 mmol, 1.5 equiv.) was then slowly added and the mixture was stirred vigorously (without splashing) at -78 °C for 30 min. After that, a solution of the **2a** (38.4 mg, 0.12 mmol, 1.2 equiv.) in Et_2O (1.2 mL) was added dropwise. The reaction mixture was stirred at this temperature for an additional 10 min. $MgBr_2$ (freshly made from Mg and 1,2-dibromoethane, 0.5 M in Et_2O, 0.4 mL, 0.2 mmol, 2.0 equiv.) was then added dropwise and the reaction mixture was stirred at -78 °C for 20 min. The reaction mixture was then warmed to room temperature and stirred for another 30 min. Upon completion, the reaction was quenched with aqueous NH_4Cl and extracted with Et_2O (3 × 3 mL). The combined organic phases were dried over $MgSO_4$, and the solvent was removed in vacuo. The resulting crude residue was a mixture of **14-S1** and unreacted diboron **2a**, which was then treated with sodium perborate tetrahydrate (0.1 mmol, 1.0 equiv.) in THF/H_2O (1:1, 2 mL in total) until the TLC showed full consumption of **2a**. Upon completion, the reaction was quenched with H_2O and extracted with Et_2O (3 × 3 mL). The combined organic phases were dried over $MgSO_4$, and the solvent was removed in vacuo. The resulting residue was briefly purified by flash column chromatography to give crude product **14-S1** that was used for the next step.

According to a reported literature,[18] under argon, to an oven dried 4 mL reaction tube equipped with magnetic stir bar was added **14-S1** (0.1 mmol, 1.0 equiv.), Tf_2NH (28.1 mg, 0.1 mmol, 1.0 equiv.) and toluene (500 µL). The reaction mixture was then stirred at 160 °C for 12 h. Upon completion, the reaction was quenched with $^{n}Bu_4NBH_4$ (25.7 mg, 0.1 mmol, 1.0 equiv.). The reaction mixture was then passed through a short pad of silica, washed with ethyl acetate and concentrated in vacuo. The resulting residue was purified by flash column chromatography to afford the desired product **14** (26%, 12.9 mg).

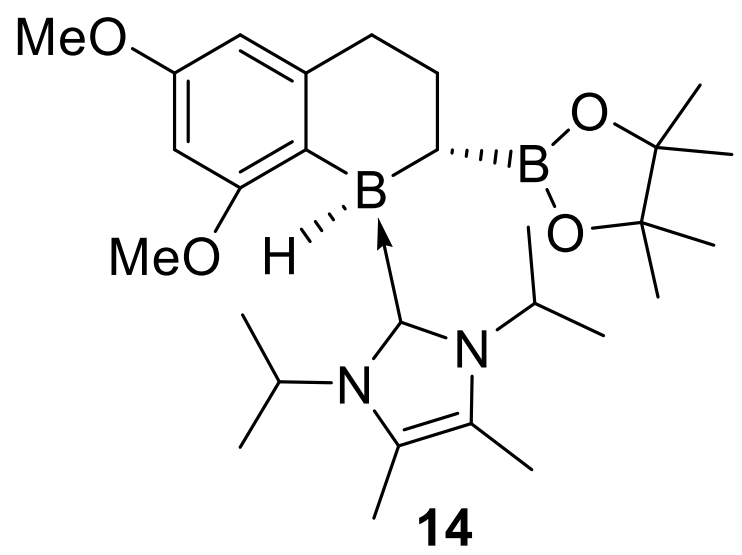

14

14: Yield: 12.9 mg, 26%, >20:1 d.r., colorless oil, R_f = 0.4 (*n*-hexane/ethyl acetate = 3/1).

^{1}H NMR (400 MHz, $CDCl_3$) δ 6.44 – 6.16 (m, 1H), 6.16 – 5.90 (m, 1H), 3.75 (s, 3H), 3.38 (s, 3H), 2.98 – 2.75 (m, 1H), 2.73 – 2.51 (m, 1H), 2.25 – 2.12 (m, 6H), 1.87 – 1.72 (m, 2H), 1.44 – 1.38 (m, 12H), 1.24 – 1.12 (m,12H), -0.01 – -0.04 (m, 1H).

^{13}C NMR (101 MHz, $CDCl_3$) δ 162.8, 157.4, 146.8, 123.3, 104.8, 95.1, 81.2, 55.3, 54.5, 49.1, 35.7, 26.5, 25.4, 24.4, 21.4, 10.7.

^{11}B NMR (128 MHz, $CDCl_3$) δ 36.1 (s, 1B), -23.8 (d, *J* = 82.7 Hz, 1B).

HRMS (ESI): Calcd for $C_{28}H_{46}B_2N_2O_4Na^+$ $[M+Na]^+$: 519.3536, found: 519.3546.

IR (neat, cm^{-1}): *ν* 3396, 2970, 2931, 2850, 1688, 1595, 1460, 1368, 1310, 1205, 1150, 1055.

$[\alpha]_D^{25}$= -6.86 (*c* = 0.2, $CHCl_3$).

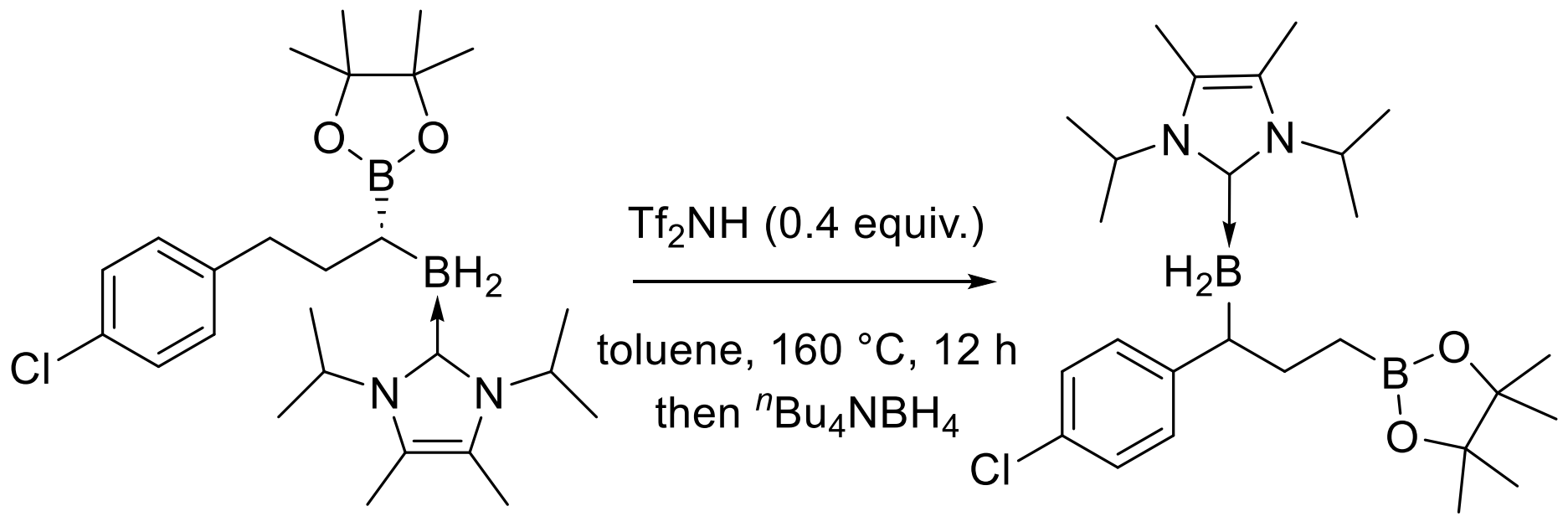

Under argon, to a 4 mL tube equipped with magnetic stir bar was added **3i** (47.2 mg, 0.1 mmol, 1.0 equiv.), Tf_2NH (11.2 mg, 0.04 mmol, 0.4 equiv.) and toluene (500 μL). The reaction mixture was then stirred at 160 °C for 12 h. Upon completion, the reaction was quenched with ^{n}Bu_4NBH_4. The reaction mixture was then passed through a short pad of silica, washed with ethyl acetate and concentrated in vacuo. The resulting residue was purified by flash column chromatography to afford the desired product **14-side** (43%, 20.3 mg).

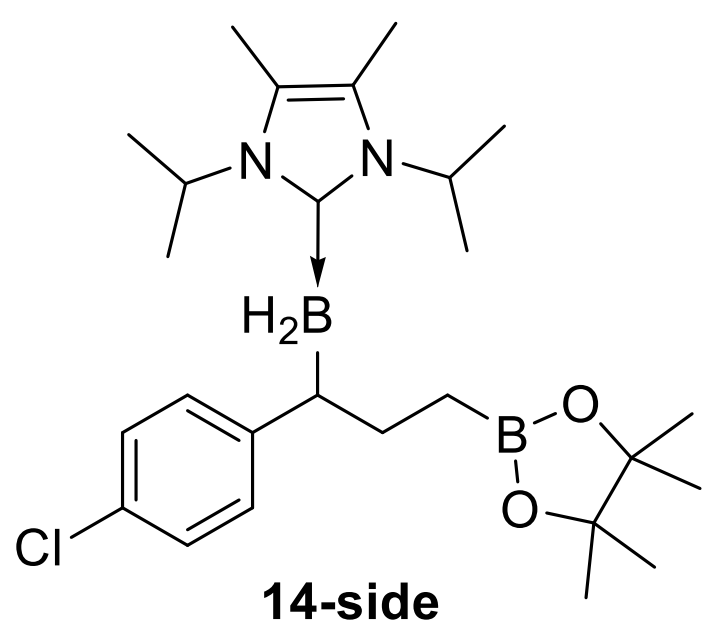

14-side

14-side: Yield: 20.3 mg, 43%, colorless oil, $R_f = 0.4$ (*n*-hexane/ethyl acetate = 3/1).

^{1}H NMR (500 MHz, $CDCl_3$) δ 7.02 (d, J = 8.1 Hz, 2H), 6.90 (d, J = 8.1 Hz, 2H), 5.32 – 5.08 (m, 2H), 2.15 (s, 6H), 1.87 – 1.77 (m, 1H), 1.38 (d, J = 7.1 Hz, 6H), 1.31 – 1.24 (m, 2H), 1.20 (s, 12H), 1.10 (d, J = 7.1 Hz, 6H), 0.87 – 0.78 (m, 1H), 0.76 – 0.61 (m, 1H).

^{13}C NMR (126 MHz, $CDCl_3$) δ 153.3, 128.8, 127.6, 127.6, 123.7, 82.6, 49.8, 31.8, 25.0, 21.7, 21.1, 10.5.

^{11}B NMR (128 MHz, $CDCl_3$) δ 34.8 (s, 1B), -22.4 (t, J = 84.8 Hz, 1B).

HRMS (ESI): Calcd for $C_{26}H_{43}B_2N_2O_2ClNa^+$ $[M+Na]^+$: 495.3091, found: 495.3101.

IR (neat, cm^{-1}): v 3394, 2974, 2927, 2292, 1690, 1486, 1400, 1370, 1220, 1144, 1090.

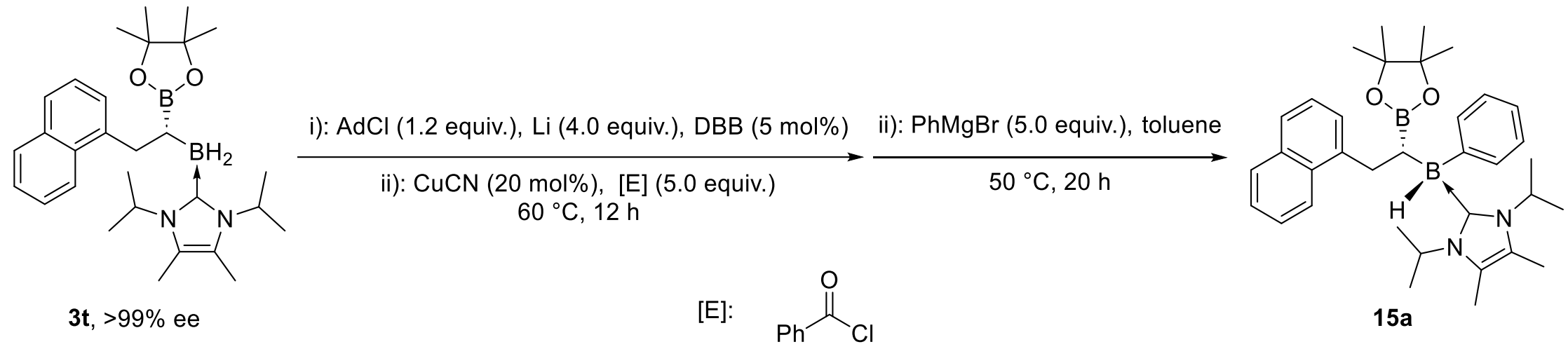

In an argon-charged glovebox, to an oven dried 4 mL vial equipped with magnetic stir bar was added **3t** (47.7 mg, 0.1 mmol, 1.0 equiv.), 1-chloroadamantane (18.9 mg, 0.11 mmol, 1.1 equiv.), 1-tert-butyl-4-(4-tert-butylphenyl)benzene (DBB) (1.4 mg, 0.005 mmol, 5 mol%), lithium (freshly cut, 3.5 mg, 5.0 equiv.) and anhydrous THF (150 μL). The vial was capped and stirred at room temperature for 1 h until the reaction mixture turned dark red, indicating the formation of the borate complex. The solution was transferred to another reaction tube equipped with a magnetic stir bar, and the residual lithium was washed with anhydrous THF (350 μL). To the reaction tube equipped with magnetic stir bar was added copper cyanide (1.8 mg, 0.02 mmol, 20 mol%) and benzoyl chloride (70.3 mg, 0.5 mmol, 5.0 equiv.). Then, the tube was capped, sealed with Teflon tape, brought out from glovebox, and heated at 60 °C for 12 h. Upon completion, the reaction mixture was passed through a short pad of silica, washed with ethyl acetate and concentrated in vacuo. The crude sample was dissolved in dry toluene (1 mL) and phenylmagnesium bromide (1 M in THF, 0.5 mL, 0.5 mmol, 5.0 equiv.) was then slowly added at room temperature. The reaction mixture was heated at 50 °C and stirred for 20 h. The reaction was quenched with H_2O and extracted with ethyl acetate (3 × 3 mL). The combined organic phases were dried over $MgSO_4$, and the solvent was removed in vacuo. The residue resulting was purified by deactivated silica gel column chromatography to give the desired product. The resulting residue was purified by flash column chromatography to afford the desired product **15a** (20%, 11.0 mg).

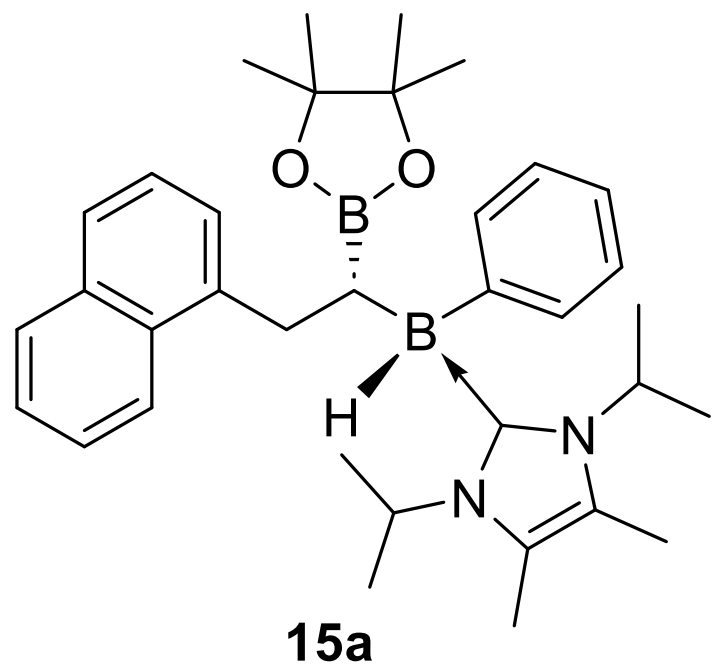

15a

15a: Yield: 11.0 mg, 20%, >20:1 d.r., white solid, m.p. = 169 – 175 °C, R_f = 0.5 (*n*-hexane/ethyl acetate = 5/1).

^{1}H NMR (400 MHz, $CDCl_3$) δ 8.44 – 8.16 (m, 1H), 7.88 – 7.69 (m, 2H), 7.69 – 7.58 (m, 1H), 7.50 – 7.28 (m, 5H), 7.19 – 7.09 (m, 2H), 7.09 – 6.97 (m, 1H), 5.66 (s, 2H), 3.66 – 3.26 (m, 2H), 2.21 (s, 6H), 1.62 (d, *J* = 7.0 Hz, 6H), 1.41 – 0.99 (m, 13H), 0.66 (s, 6H).

^{13}C NMR (101 MHz, $CDCl_3$) δ 143.9, 133.8, 133.6, 132.8, 128.2, 127.2, 125.5, 125.3, 125.1, 124.9, 124.8, 124.8, 124.3, 123.6, 81.3, 50.2, 35.6, 25.5, 23.9, 22.5, 21.8, 21.2, 14.2, 10.7.

^{11}B NMR (128 MHz, $CDCl_3$) δ 37.1 (s, 1B), -16.8 (d, *J* = 86.1 Hz, 1B).

HRMS (ESI): Calcd for $C_{35}H_{48}B_2N_2O_2Na^+$ $[M+Na]^+$: 573.3794, found: 573.3811.

IR (neat, cm^{-1}): ν 3045, 2976, 2934, 2330, 1466, 1429, 1284, 1142, 1025.

$[\alpha]_D^{25}$= 60.36 (*c* = 0.9, $CHCl_3$).

7. Synthesis of boron-stereogenic compounds using CDBA reagent.

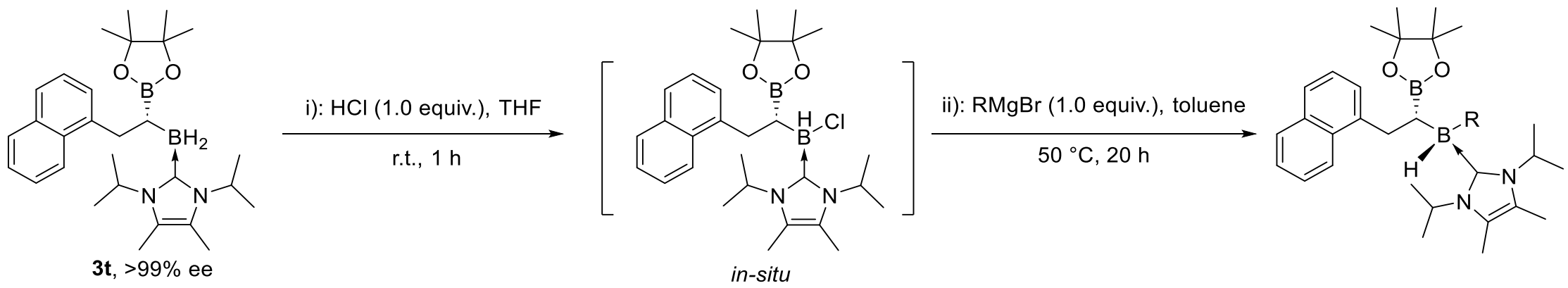

Under argon, to an oven dried 10 mL Schlenk tube equipped with magnetic stir bar and a septum was charged with **3t** (47.4 mg, 0.1 mmol, 1.0 equiv.) and THF (1 mL). HCl (1 M in Et_2O, 100 μL, 0.1 mmol, 1.0 equiv.) was slowly added and the reaction mixture was stirred at room temperature for 1 h. Upon completion, the solvent was removed in vacuo and the crude sample was dissolved in dry toluene (1 mL). Then, Grignard reagent (0.1 mmol, 1.0 equiv.) was slowly added at room temperature. The reaction mixture was heated at 50 °C and stirred for 20 h. The reaction was quenched with H_2O and extracted with ethyl acetate (3 × 3 mL). The combined organic phases were dried over $MgSO_4$, and the solvent was removed in vacuo. The residue resulting was purified by deactivated silica gel column chromatography to give the desired product.

Note: This stereoselective pathway was inspired by the pioneering work of Chuzel and coworkers.[19] In this work, they reported that the chlorination of a cyclic chiral NHC-borane followed by nucleophilic arylation with aryl Grignard reagent yields a boron-

stereogenic center with high diastereoselectivity. Based on their experimental observations and theoretical investigations, they proposed a probable SRN1 type mechanism initiated by a Single Electron Transfer (SET) from the Grignard reagent to the chlorinated NHC-borane. This generates a radical anion intermediate that undergoes fragmentation to afford a key boryl radical along with a chloride ion, which subsequently enters a classical SRN1 chain mechanism. Given the structural and reactivity similarities, we anticipate that our system operates through a related mechanism, where the adjacent chiral carbon effectively induces chirality at the sp^3-boron center (**Fig. S4**). It is also noteworthy that an ionic pathway via a borenium intermediate cannot be entirely ruled out.

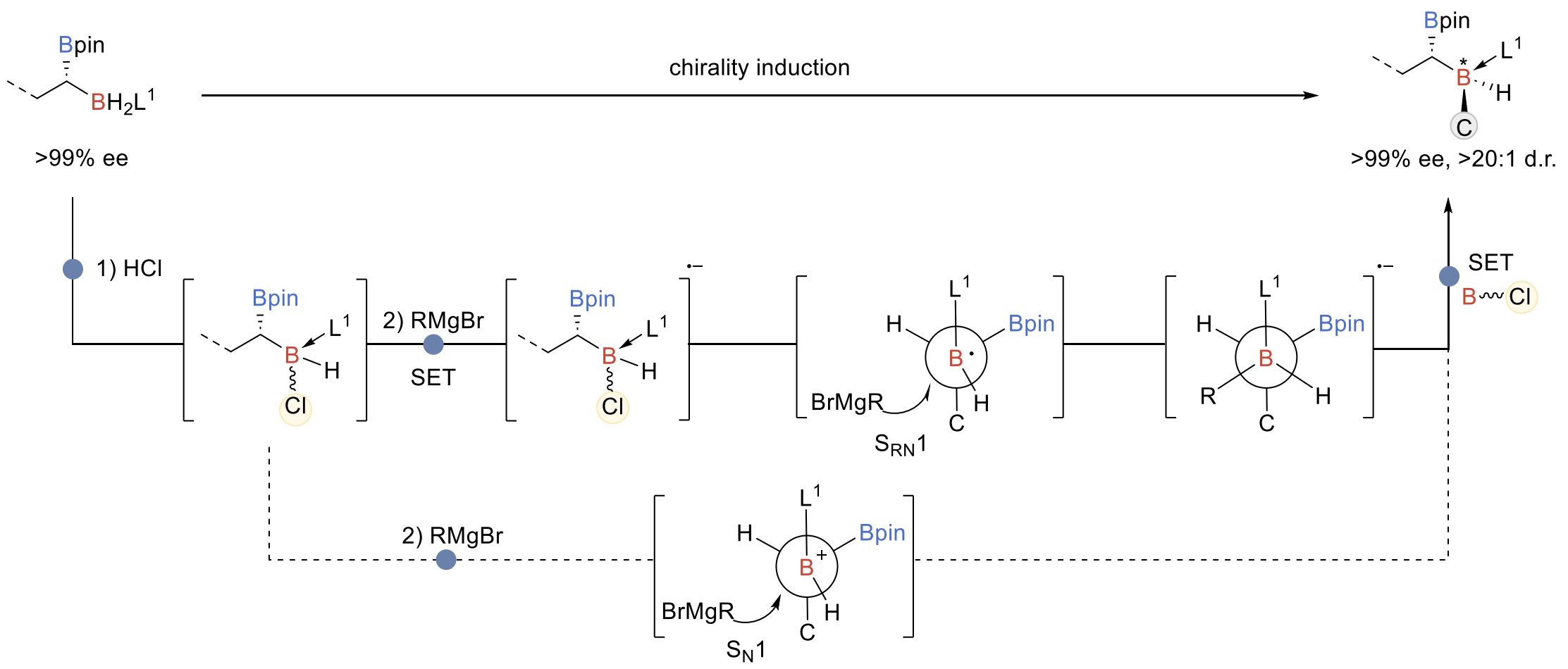

Fig. S4. A proposed reaction mechanism and stereochemical rationale based on Chuzel's work.

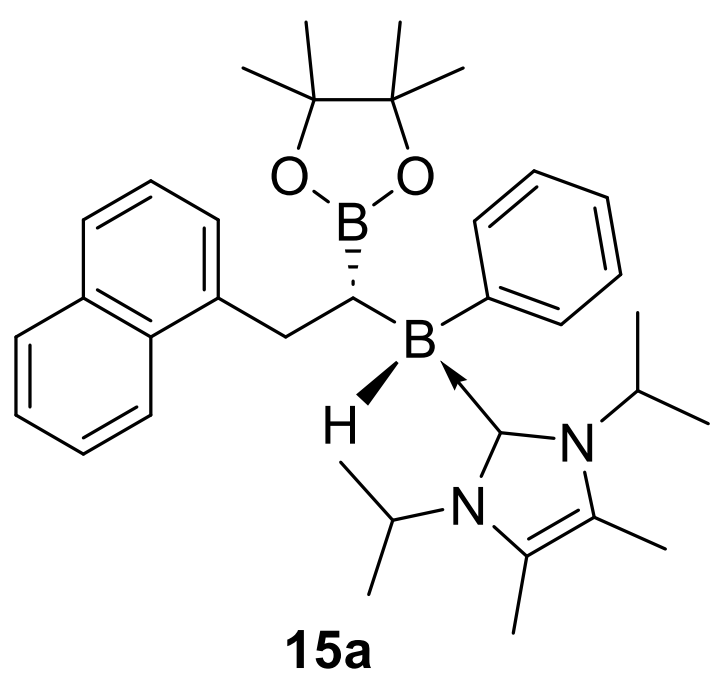

15a

15a: Yield: 39.1 mg, 71%, >99% ee, >20:1 d.r., white solid, m.p. = 169 – 175 °C, R_f = 0.5 (*n*-hexane/ethyl acetate = 5/1).

^{1}H NMR (400 MHz, $CDCl_3$) δ 8.44 – 8.16 (m, 1H), 7.88 – 7.69 (m, 2H), 7.69 – 7.58 (m, 1H), 7.50 – 7.28 (m, 5H), 7.19 – 7.09 (m, 2H), 7.09 – 6.97 (m, 1H), 5.66 (s, 2H), 3.66 – 3.26 (m, 2H), 2.21 (s, 6H), 1.62 (d, *J* = 7.0 Hz, 6H), 1.41 – 0.99 (m, 13H), 0.66 (s, 6H).

^{13}C NMR (101 MHz, $CDCl_3$) δ 143.9, 133.8, 133.6, 132.8, 128.2, 127.2, 125.5, 125.3, 125.1, 124.9, 124.8, 124.8, 124.3, 123.6, 81.3, 50.2, 35.6, 25.5, 23.9, 22.5, 21.8, 21.2, 14.2, 10.7.

^{11}B NMR (128 MHz, $CDCl_3$) δ 37.1 (s, 1B), -16.8 (d, *J* = 86.1 Hz, 1B).
HRMS (ESI): Calcd for $C_{35}H_{48}B_2N_2O_2Na^+$ $[M+Na]^+$: 573.3794, found: 573.3811.
IR (neat, cm^{-1}): *v* 3045, 2976, 2934, 2330, 1466, 1429, 1284, 1142, 1025.
Chiral HPLC (IA-3, ^{i}PrOH/*n*-hexane = 1/99, 0.5 mL/min) t_R 11.1 min (major), 12.1 min (minor).
$[\alpha]_D^{25}$ = 61.56 (*c* = 0.9, $CHCl_3$).

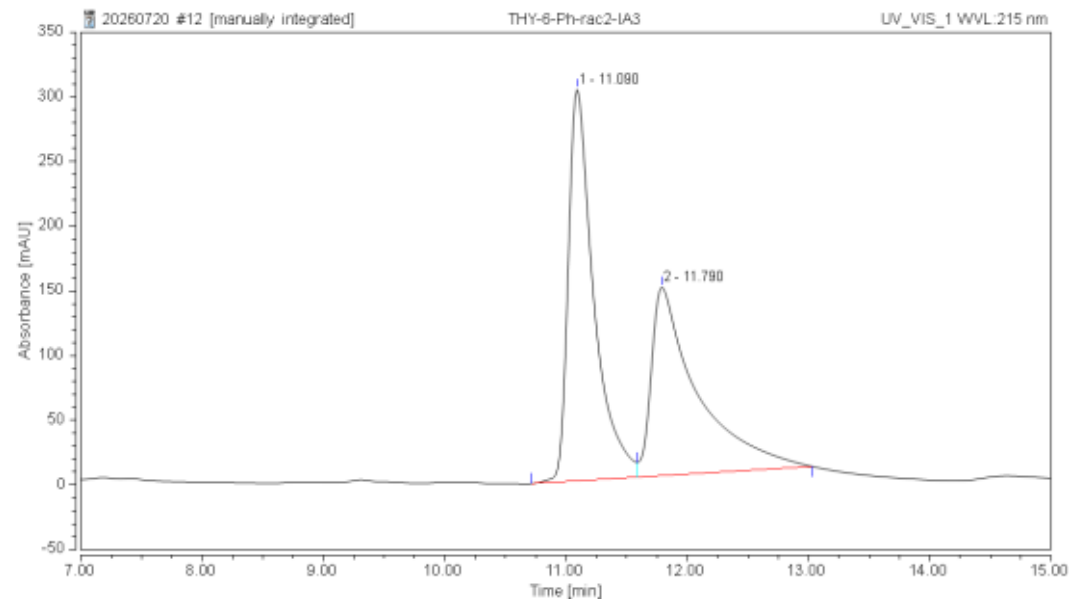

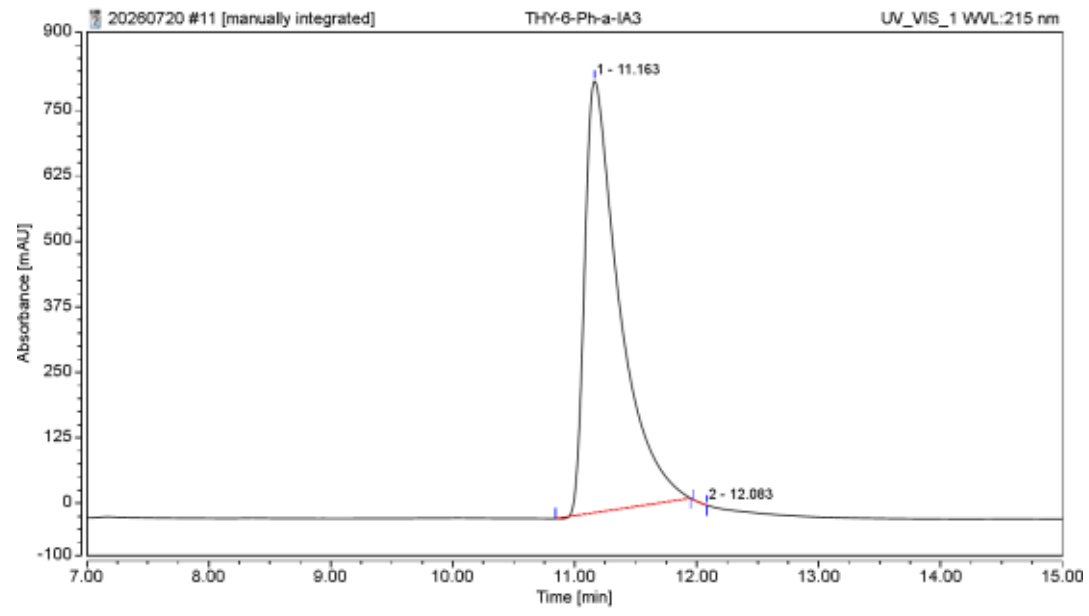

	Retention Time	% Area	Area
1	11.09	73.6178	53.36
2	11.79	64.3389	46.64

	Retention Time	% Area	Area
1	11.163	271.4558	99.98
2	12.083	0.0505	0.02

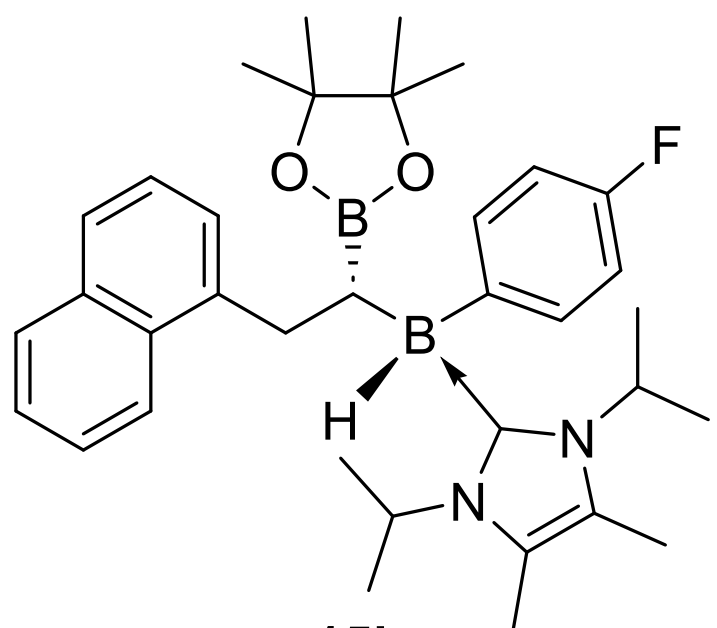

15b

15b: Yield: 51.7 mg, 91%, >99% ee, >20:1 d.r., white solid, m.p. = 188 – 191 °C, R_f = 0.5 (*n*-hexane/ethyl acetate = 5/1).
^{1}H NMR (500 MHz, $CDCl_3$) δ 8.33 – 8.23 (m, 1H), 7.96 – 7.77 (m, 1H), 7.77 – 7.70 (m, 1H), 7.70 – 7.61 (m, 1H), 7.48 – 7.37 (m, 3H), 7.33 – 7.19 (m, 2H), 6.91 – 6.78 (m, 2H), 5.93 – 5.45 (m, 2H), 3.38 (d, *J* = 7.3 Hz, 2H), 2.21 (s, 6H), 1.61 (d, *J* = 7.1 Hz, 6H), 1.34 – 0.99 (m, 13H), 0.67 (s, 6H).
^{13}C NMR (128 MHz, $CDCl_3$) δ 160.8 (d, $J_{C\text{-}F}$ = 239.5 Hz), 143.7, 134.7 (d, $J_{C\text{-}F}$ = 6.3 Hz), 133.6, 132.8, 128.3, 125.5, 125.2, 125.1, 125.0, 124.9, 124.8, 124.4, 113.7 (d, $J_{C\text{-}F}$ = 18.6 Hz), 81.4, 50.1, 35.5, 25.5, 23.9, 21.8, 21.3, 10.7.
^{19}F NMR (471 MHz, $CDCl_3$) δ -121.37 – -121.69 (m, 1F).
^{11}B NMR (160 MHz, $CDCl_3$) δ 37.0 (s, 1B), -17.2 (d, *J* = 85.9 Hz, 1B).
HRMS (ESI): Calcd for $C_{35}H_{47}B_2FN_2O_2Na^+$ $[M+Na]^+$: 591.3700, found: 591.3716.
IR (neat, cm^{-1}): *v* 2977, 2337, 1467, 1389, 1358, 1214, 1142, 1013.
Chiral HPLC (ODH, ^{i}PrOH/*n*-hexane = 1/99, 0.5 mL/min) t_R 14.5 min (minor), 15.7 min (major).
$[\alpha]_D^{25}$ = 63.90 (*c* = 1.0, $CHCl_3$).

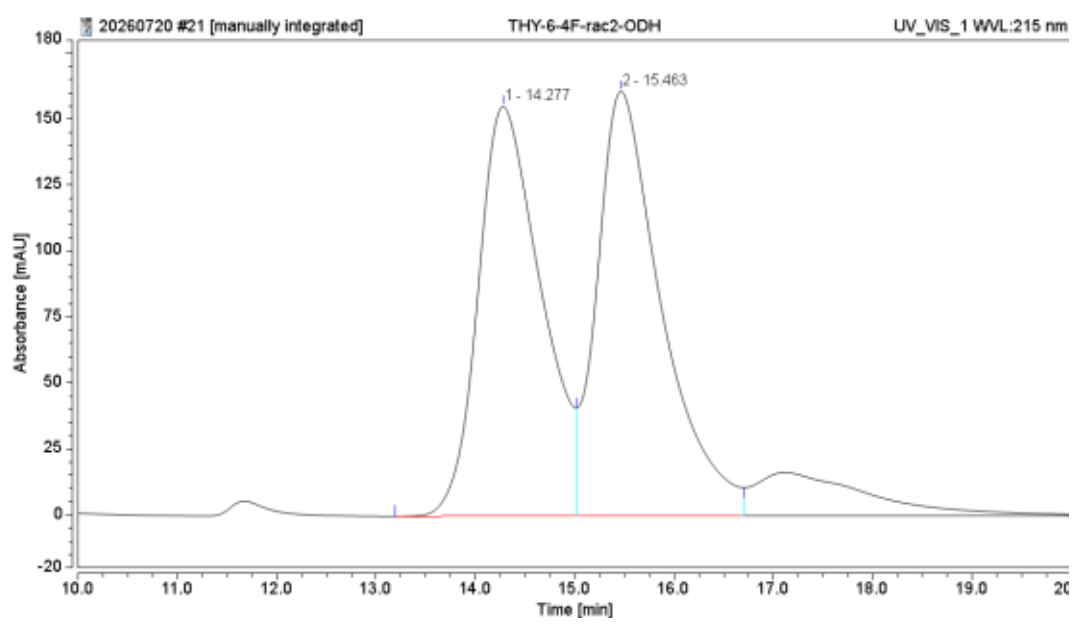

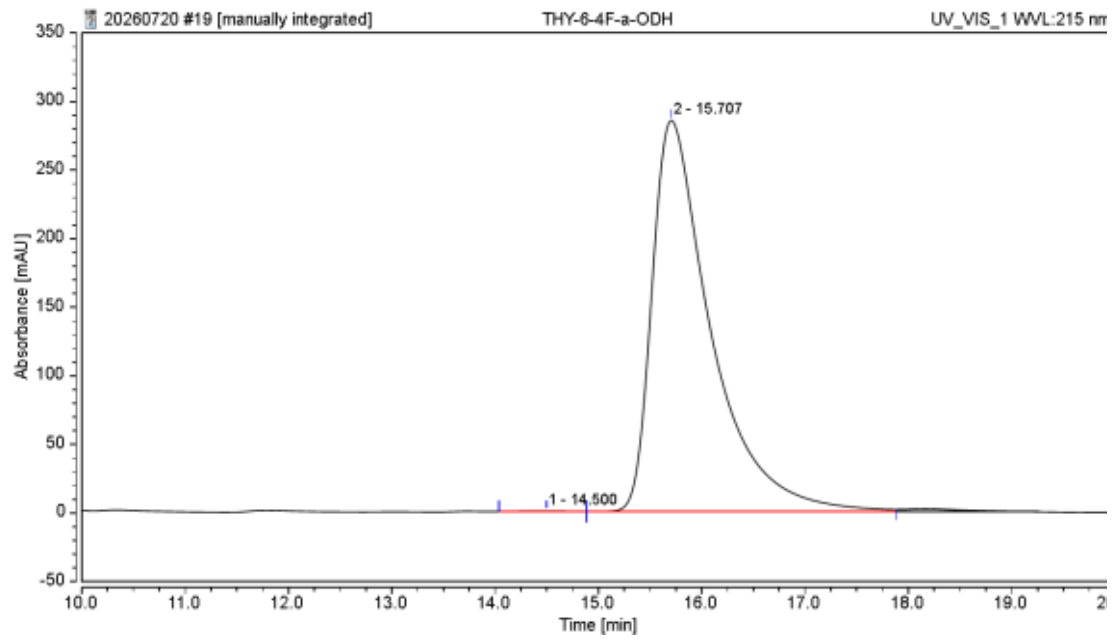

	Retention Time	% Area	Area
1	14.277	113.8264	48.33
2	15.463	121.7158	51.67

	Retention Time	% Area	Area
1	14.500	0.1361	0.07
2	15.707	188.1899	99.93

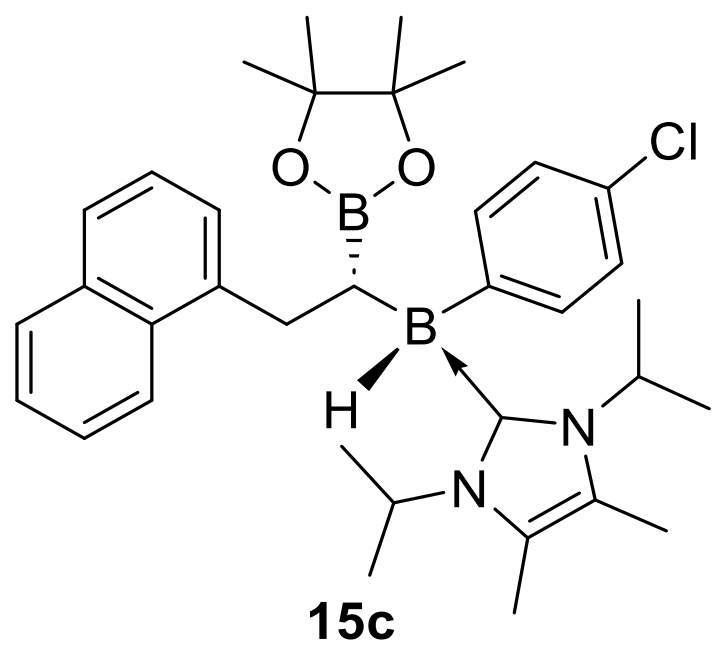

15c

15c: Yield: 55.5 mg, 95%, >99% ee, >20:1 d.r., white solid, m.p. = 187 – 191 °C, R_f = 0.5 (*n*-hexane/ethyl acetate = 5/1).

^{1}H NMR (400 MHz, $CDCl_3$) δ 8.36 – 8.13 (m, 1H), 7.86 – 7.77 (m, 1H), 7.76 – 7.70 (m, 1H), 7.67 – 7.62 (m, 1H), 7.52 – 7.39 (m, 3H), 7.36 – 7.27 (m, 2H), 7.22 – 7.07 (m, 2H), 5.75 – 5.41 (m, 2H), 3.37 (d, *J* = 7.3 Hz, 2H), 2.21 (s, 6H), 1.61 (d, *J* = 7.1 Hz, 6H), 1.37 – 0.99 (m, 13H), 0.67 (s, 6H).

^{13}C NMR (101 MHz, $CDCl_3$) δ 143.6, 135.1, 133.6, 132.7, 129.3, 128.3, 127.1, 125.5, 125.3, 125.1, 125.0, 124.9, 124.8, 124.5, 81.4, 50.1, 35.5, 25.5, 23.9, 21.8, 21.3, 14.2, 10.7.

^{11}B NMR (128 MHz, $CDCl_3$) δ 37.4 (s, 1B), -17.2 (d, *J* = 87.8 Hz, 1B).

HRMS (ESI): Calcd for $C_{35}H_{47}B_2ClN_2O_2Na^+$ $[M+Na]^+$: 607.3404, found: 607.3419.

IR (neat, cm^{-1}): *v* 3047, 2976, 2932, 2339, 1358, 1306, 1241, 1094, 1053.

Chiral HPLC (ODH, ^{i}PrOH/*n*-hexane = 1/99, 1.0 mL/min) t_R 6.6 min (minor), 7.3 min (major).

$[\alpha]_D^{25}$= 63.00 (*c* = 1.0, $CHCl_3$).

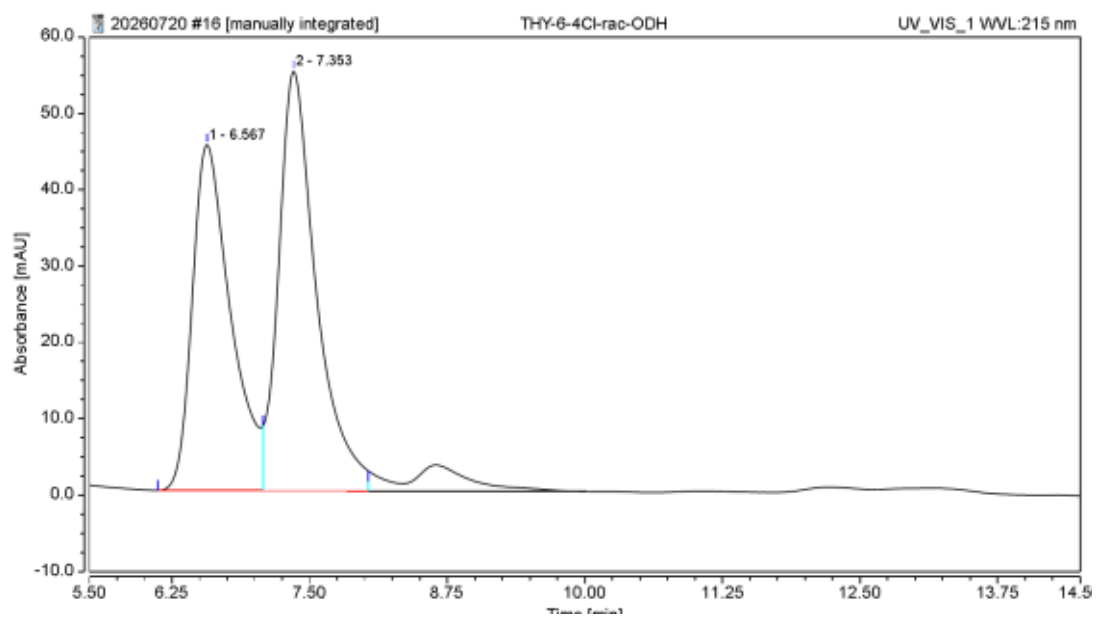

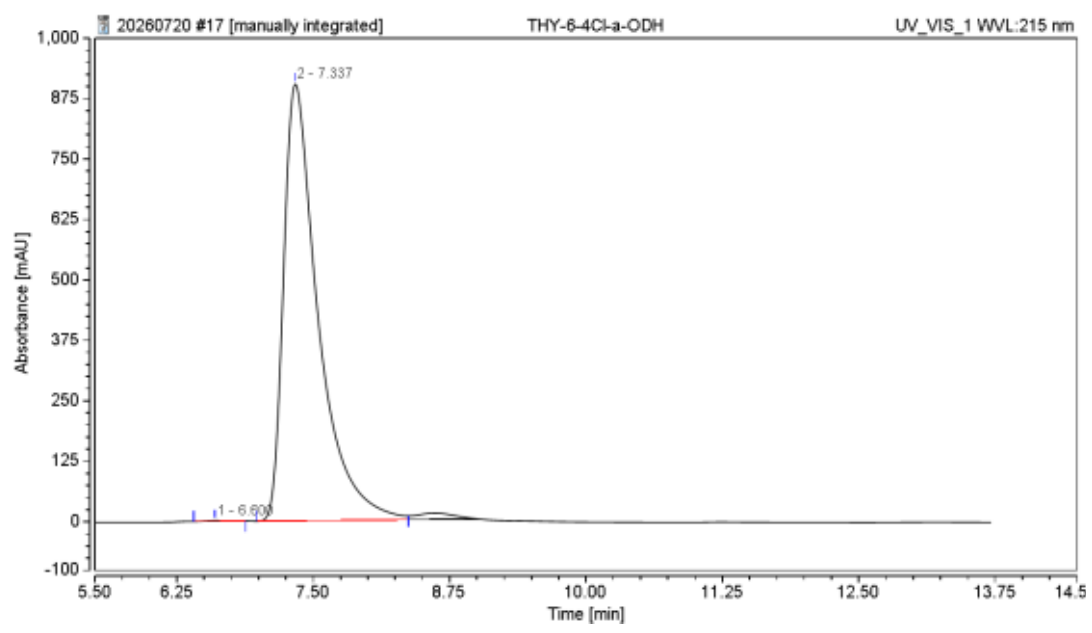

	Retention Time	% Area	Area
1	6.567	18.3763	45.81
2	7.353	21.7379	54.19

	Retention Time	% Area	Area
1	6.600	0.2961	0.09
2	7.337	330.5017	99.91

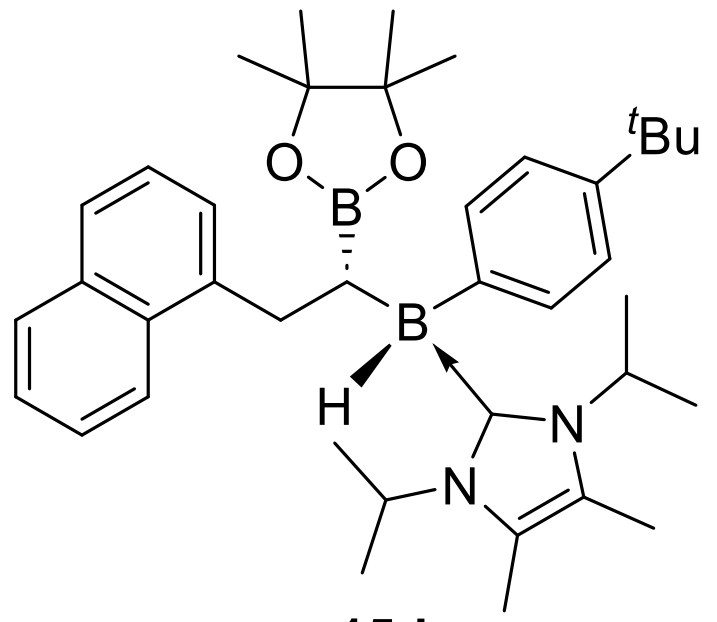

15d

15d: Yield: 43.6 mg, 72%, >99% ee, >20:1 d.r., white solid, decomposed at 210 °C, R_f = 0.5 (*n*-hexane/ethyl acetate = 5/1).

^{1}H NMR (400 MHz, $CDCl_3$) δ 8.48 – 7.98 (m, 1H), 7.93 – 7.67 (m, 2H), 7.62 (d, *J* = 8.1 Hz, 1H), 7.54 – 7.41 (m, 3H), 7.28 (s, 2H), 7.26 – 7.13 (m, 2H), 5.64 (s, 2H), 3.70 – 3.20 (m, 2H), 2.20 (s, 6H), 1.60 (d, *J* = 6.9 Hz, 6H), 1.37 – 1.22 (m, 16H), 1.01 (s, 6H), 0.64 (s, 6H).

^{13}C NMR (101 MHz, $CDCl_3$) δ 146.1, 144.1, 134.8, 133.6, 133.4, 132.9, 128.2, 125.6, 125.5, 125.1, 124.9, 124.8, 124.8, 124.8, 124.2, 124.1, 81.3, 50.1, 35.7, 34.2, 31.7, 25.5, 25.0, 23.9, 21.8, 21.1, 10.7.

^{11}B NMR (128 MHz, $CDCl_3$) δ 38.4 (s, 1B), -17.2 (d, *J* = 85.5 Hz, 1B).

HRMS (ESI): Calcd for $C_{39}H_{56}B_2N_2O_2Na^+$ $[M+Na]^+$: 629.4420, found: 629.4437.

IR (neat, cm^{-1}): *v* 3047, 2969, 2328, 1464, 1360, 1144, 1103.

Chiral HPLC (ODH, ^{i}PrOH/*n*-hexane = 1/99, 0.5 mL/min) t_R 9.9 min (minor), 10.5 min (major).

$[\alpha]_D^{25}$= 36.50 (*c* = 1.0, $CHCl_3$).

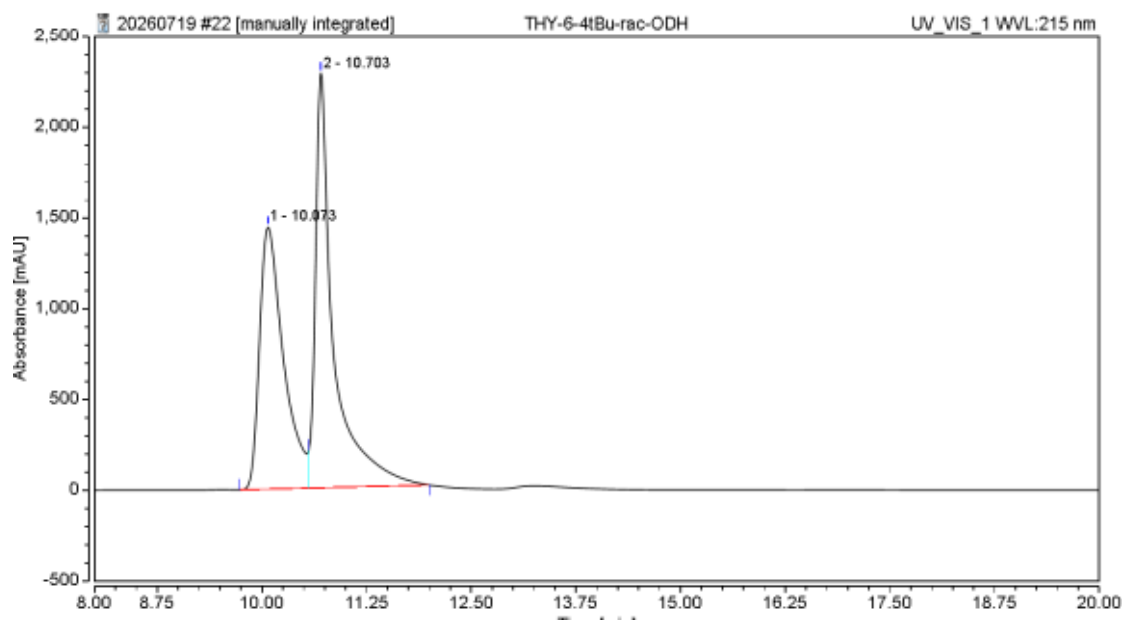

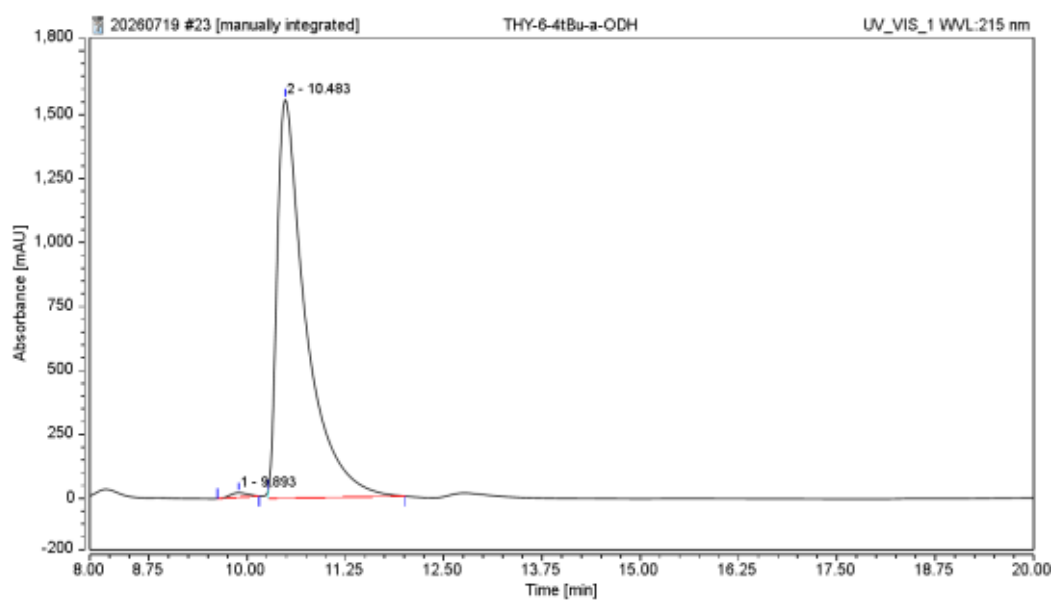

	Retention Time	% Area	Area
1	10.073	489.988	46.58
2	10.703	562.0411	53.42

	Retention Time	% Area	Area
1	9.893	2.3167	0.35
2	10.483	655.3558	99.65

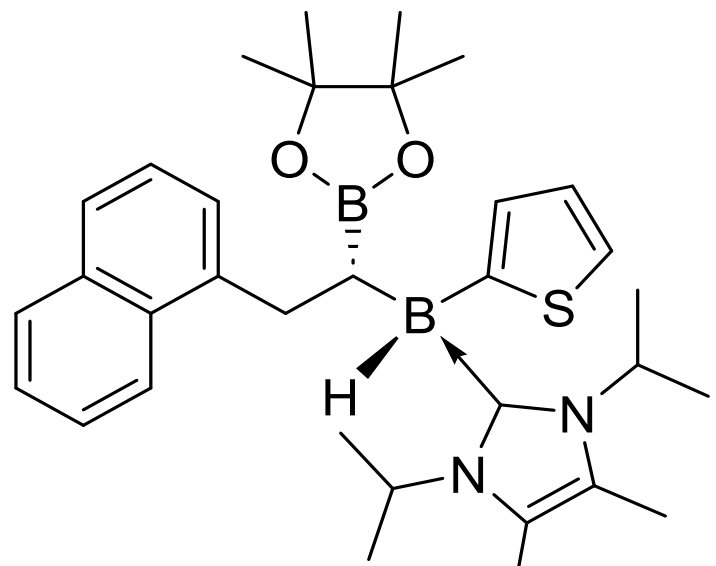

15e

15e: Yield: 48.4 mg, 87%, >99% ee, >20:1 d.r., yellow solid, decomposed at 205 °C, R_f = 0.5 (*n*-hexane/ethyl acetate = 5/1).

^{1}H NMR (400 MHz, $CDCl_3$) δ 8.43 – 8.29 (m, 1H), 7.97 – 7.72 (m, 1H), 7.71 – 7.60 (m, 2H), 7.52 – 7.33 (m, 3H), 7.26 – 7.19 (m, 1H), 7.12 – 6.89 (m, 2H), 6.05 – 5.51 (m, 2H), 3.96 – 3.44 (m, 1H), 3.46 – 3.08 (m, 1H), 2.21 (s, 6H), 1.61 (d, *J* = 7.0 Hz, 6H), 1.38 – 1.26 (m, 7H), 0.99 (s, 6H), 0.69 (s, 6H).

^{13}C NMR (101 MHz, $CDCl_3$) δ 144.0, 133.7, 132.7, 128.2, 127.3, 127.3, 125.5, 125.3, 125.2, 124.9, 124.8, 124.5, 124.2, 81.4, 50.3, 36.0, 25.4, 24.1, 21.8, 21.4, 10.7.

^{11}B NMR (128 MHz, $CDCl_3$) δ 36.7 (s, 1B), -18.8 (d, *J* = 85.8 Hz, 1B).

HRMS (ESI): Calcd for $C_{33}H_{46}B_2N_2O_2SNa^+$ $[M+Na]^+$: 579.3358, found: 579.3375.

IR (neat, cm^{-1}): *v* 2926, 2857, 2360, 1462, 1389, 1286, 1143, 1104.

Chiral HPLC (ODH, ^{i}PrOH/*n*-hexane = 1/99, 1.0 mL/min) t_R 6.9 min (minor), 7.3 min (major), 8.9 min (minor), 11.0 min (minor).

$[\alpha]_D^{25}$ = 28.30 (*c* = 1.0, $CHCl_3$).

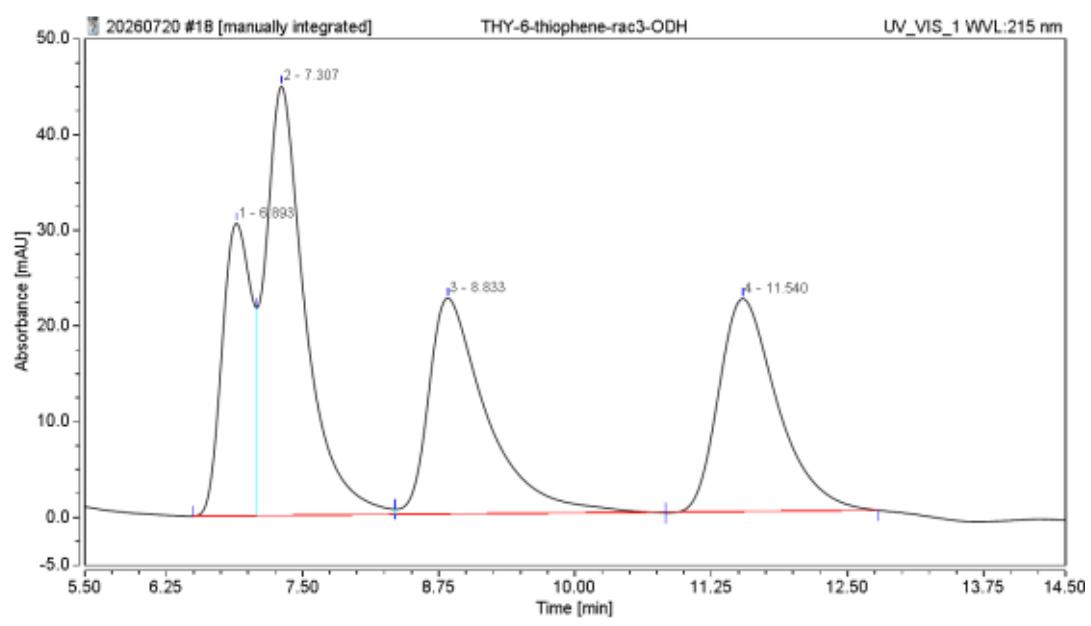

	Retention Time	% Area	Area
1	6.893	9.4914	16.86
2	7.307	19.0967	33.92
3	8.833	13.8781	24.65
4	11.54	13.8391	24.58

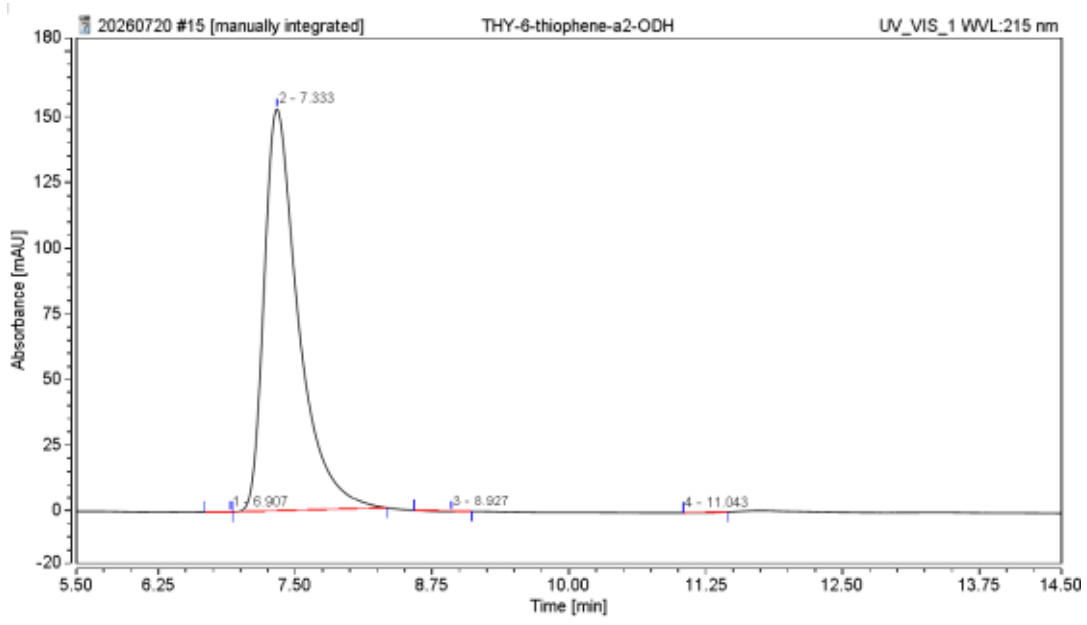

	Retention Time	% Area	Area
1	6.907	0.00	0.00
2	7.333	55.5384	99.83
3	8.927	0.06590	0.12
4	11.043	0.02990	0.05

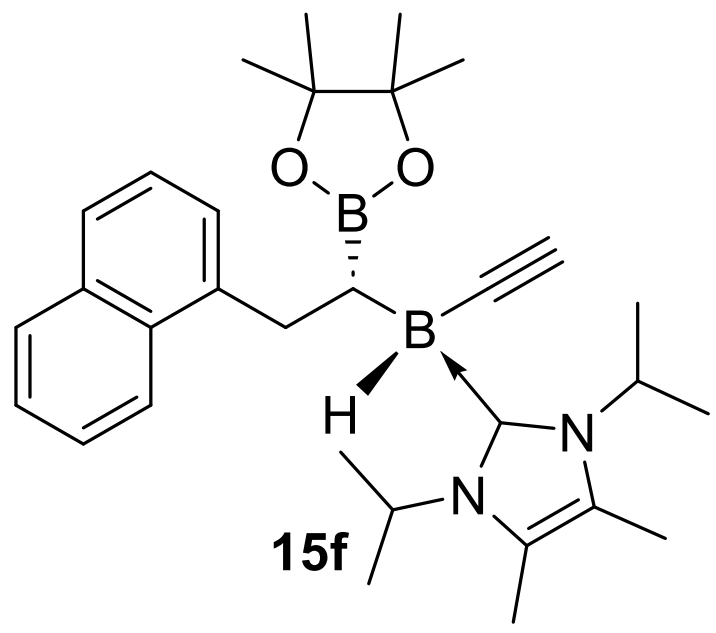

15f

15f: Yield: 22.9 mg, 46%, >99% ee, >20:1 d.r., white solid, decomposed at 195 °C, R_f = 0.5 (*n*-hexane/ethyl acetate = 5/1).

^{1}H NMR (500 MHz, $CDCl_3$) δ 8.28 – 8.17 (m, 1H), 7.88 – 7.70 (m, 1H), 7.66 – 7.55 (m, 1H), 7.55 – 7.46 (m, 1H), 7.44 – 7.36 (m, 2H), 7.35 – 7.28 (m, 1H), 6.03 – 5.35 (m, 2H), 3.43 – 3.40 (m, 1H), 3.29 – 3.11 (m, 1H), 2.24 (s, 6H), 2.20 – 2.12 (m, 1H), 1.58 – 1.47 (m, 13H), 1.05 (s, 6H), 0.89 (s, 6H).

^{13}C NMR (126 MHz, $CDCl_3$) δ 144.0, 133.9, 132.5, 128.4, 125.5, 125.5, 125.3, 125.3, 124.9, 124.9, 124.6, 82.7, 81.6, 50.2, 35.1, 25.5, 24.2, 21.9, 21.8, 10.7.

^{11}B NMR (160 MHz, $CDCl_3$) δ 36.1 (s, 1B), -25.2 (d, J = 86.9 Hz, 1B).

Note: After column chromatography, preparative thin-layer chromatography (PTLC) was employed to remove trace amounts of impurities to ensure the highest purity for the NMR spectra.

HRMS (ESI): Calcd for $C_{31}H_{44}B_2N_2O_2Na^+$ $[M+Na]^+$: 521.3495, found: 521.3481.

IR (neat, cm^{-1}): ν 3258, 3024, 2976, 2925, 2358, 1509, 1466, 1399, 1283, 1164, 1144.

Chiral HPLC (ODH, ^{i}PrOH/*n*-hexane = 1/99, 0.5 mL/min) t_R 22.9 min (major), 29.6 min (minor).

$[\alpha]_D^{25}$= 15.60 (c = 0.5, $CHCl_3$).

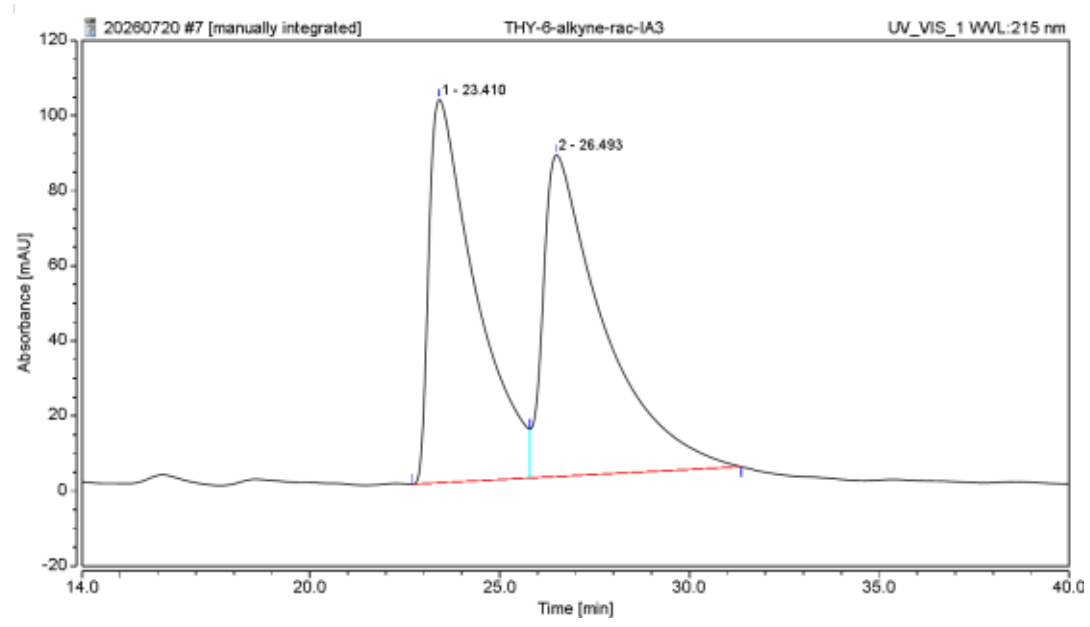

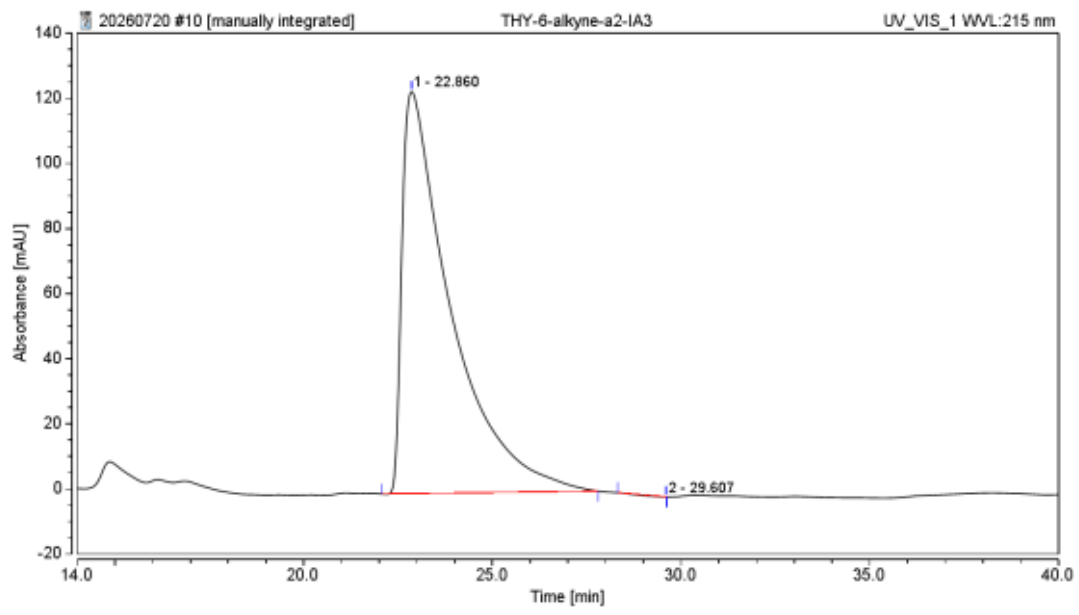

	Retention Time	Area	% Area
1	23.41	145.4979	47.86
2	26.493	158.5382	52.14

	Retention Time	Area	% Area
1	22.86	184.9015	99.92
2	29.607	0.1501	0.08

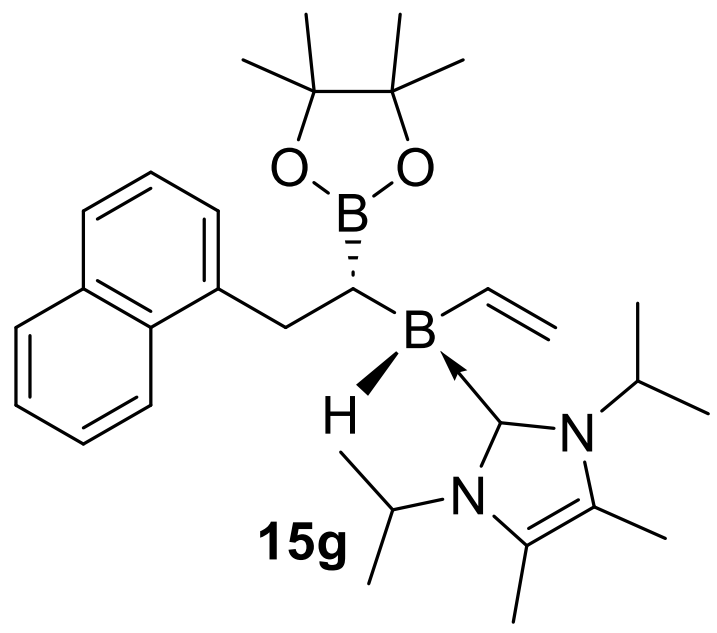

15g

15g: Yield: 18.5 mg, 37%, >99% ee for both diastereomers, 83:17 d.r., colorless oil, R_f = 0.5 (*n*-hexane/ethyl acetate = 5/1).

^{1}H NMR (500 MHz, $CDCl_3$) δ 8.30 – 8.17 (m, 0.78H), 8.13 – 8.03 (m, 0.22H), 7.97 – 7.90 (m, 0.19H), 7.85 – 7.71 (m, 1.22H), 7.67 – 7.63 (m, 0.18H), 7.60 – 7.51 (m, 1.86H), 7.49 – 7.30 (m, 3.61H), 6.69 – 6.58 (m, 0.83H), 6.56 – 6.49 (m, 0.17H), 5.89 – 5.44 (m, 1.83H), 5.43 – 5.24 (m, 0.89H), 5.28 – 5.18 (m, 0.23H), 5.12 – 5.00 (m, 0.83H), 4.94 – 4.82 (m, 0.18H), 3.56 – 3.33 (m, 1H), 3.32 – 3.08 (m, 1H), 2.22 (s, 6H), 1.55 – 1.42 (m, 9H), 1.34 – 1.20 (m, 3H), 1.21 – 1.17 (m, 3H), 1.13 – 1.05 (m, 2H), 0.99 (s, 4H), 0.76 (s, 4H).

^{13}C NMR (126 MHz, $CDCl_3$) δ 144.5, 140.5, 133.9, 132.6, 128.3, 125.5, 125.5, 125.3, 125.2, 124.9, 119.0, 83.3, 83.0, 81.8, 81.2, 50.0, 49.5, 35.4, 25.5, 25.1, 25.0, 25.0, 25.0, 24.7, 24.1, 21.9, 10.7.

^{11}B NMR (160 MHz, $CDCl_3$) δ 34.61 (s, 1B), -17.8 (d, *J* = 84.5 Hz, 1B).

HRMS (ESI): Calcd for $C_{31}H_{46}B_2N_2O_2Na^+$ $[M+Na]^+$: 523.3638, found: 523.3652.

IR (neat, cm^{-1}): *v* 2974, 2923, 2357, 1464, 1387, 1325, 1144.

Chiral HPLC (ODH, ^{i}PrOH/*n*-hexane = 1/99, 1.0 mL/min) t_R 6.2 min (major), 7.8 min (minor), 10.0 min (minor), 10.4 min (major).

$[\alpha]_D^{25}$= -22.75 (*c* = 0.4, $CHCl_3$).

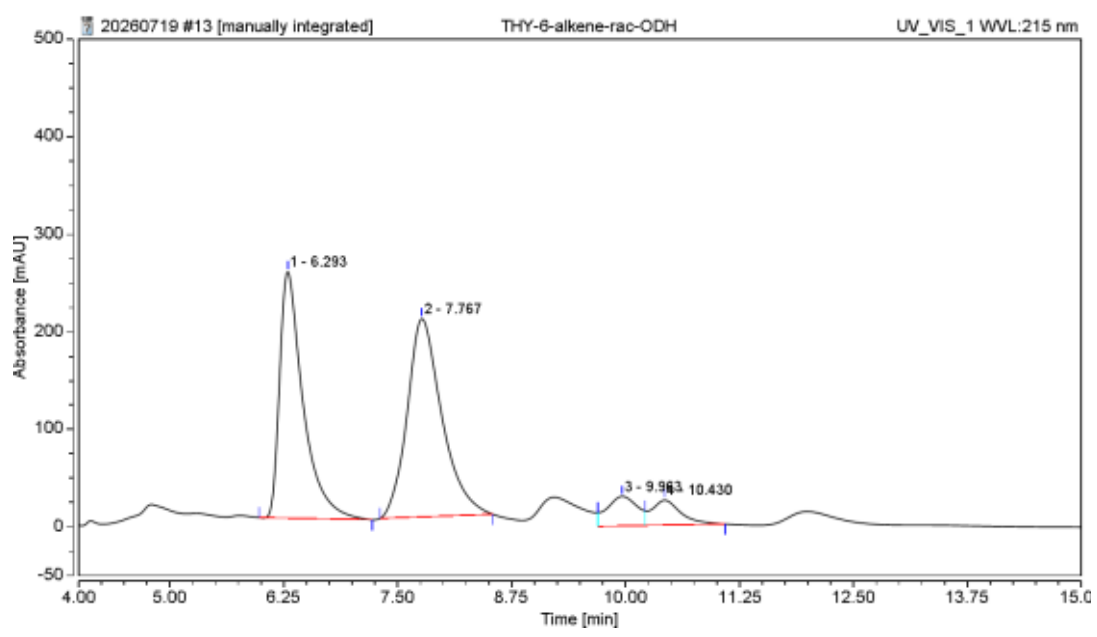

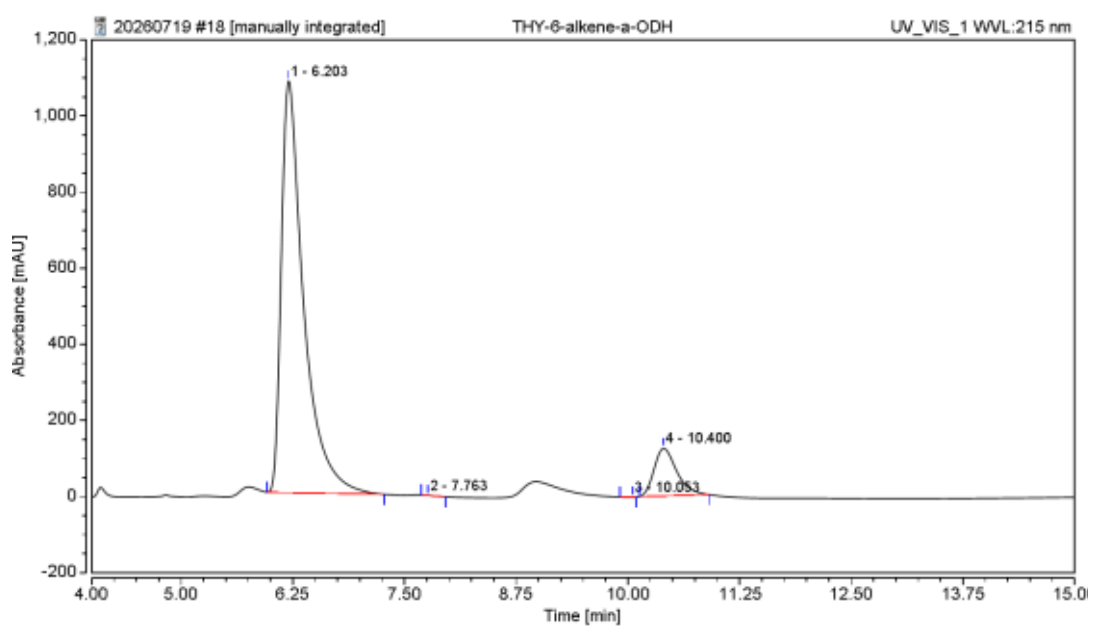

	Retention Time	% Area	Area
1	6.893	9.4914	16.86
2	7.307	19.0967	33.92
3	8.833	13.8781	24.65
4	11.54	13.8391	24.58

	Retention Time	% Area	Area
1	6.203	312.9988	89.44
2	7.763	0.0447	0.01
3	10.053	0.0527	0.02
4	10.400	36.8698	10.54

8. Synthesis of boron-containing drug molecules.

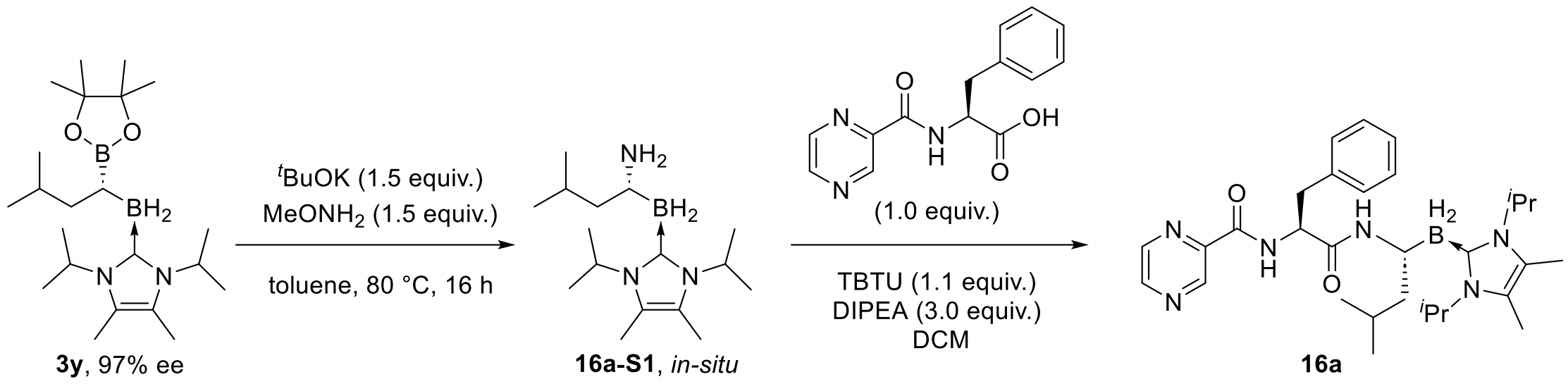

Under argon, to an oven dried 4 mL reaction tube equipped with magnetic stir bar was added **3y** (39.0 mg, 0.1 mmol, 1.0 equiv.), $MeONH_2$ (1.5 M in THF, 100 μL, 0.15 mmol, 1.5 equiv.), ^{t}BuOK (16.8 mg, 0.15 mmol, 1.5 equiv.) and toluene (500 μL). The reaction mixture was then heated at 80 °C and stirred for 16 h. Upon completion, the reaction was quenched with H_2O and extracted with ethyl acetate (3 × 3 mL). The combined organic phases were dried over $MgSO_4$, and the solvent was removed in vacuo. The resulting crude residue **16a-S1** was used for the next step without purification.

Under N_2, to an oven dried 20 mL vial equipped with magnetic stir bar was added crude reaction mixture **16a-S1** (*in-situ*, 0.1 mmol, 1.0 equiv.), 2-(1H-benzotriazole-1yl)-1,1,3,3-tetramethylaminium tetrafluoroborate (TBTU; 35.3 mg, 0.11 mmol, 1.1 equiv.), *N*-(pyrazinylcarbonyl)-*L*-phenylalanine (27.1 mg, 0.1 mmol, 1.0 equiv.) and dry DCM (1 mL). The reaction was cooled to 0 °C and ethyldiisopropylamine (DIPEA; 38.8 mg, 0.3 mmol, 3.0 equiv.) was added slowly. The reaction mixture was then stirred at room temperature for 12 h. Upon completion, the reaction was quenched with H_2O and extracted with DCM (3 × 3 mL). The combined organic phases were dried over $MgSO_4$, and the solvent was removed in vacuo. The resulting residue was purified by flash column chromatography to give product **16a** (58%, 30.6 mg).

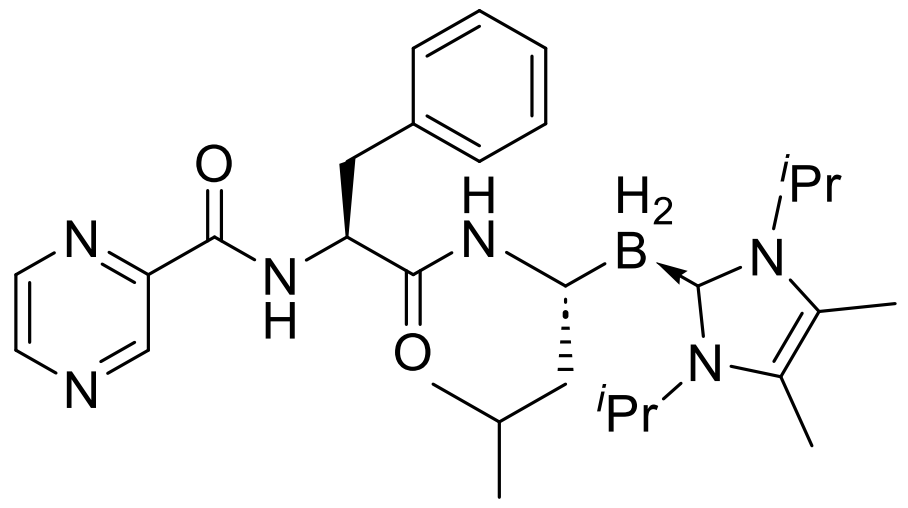

16a

16a: Yield: 30.6 mg, 58%, >20:1 d.r., white solid, decomposed at 210 °C, R_f = 0.5 (DCM/ethyl acetate = 1/3).

^{1}H NMR (400 MHz, $CDCl_3$) δ 9.32 (d, *J* = 1.5 Hz, 1H), 8.72 (d, *J* = 2.5 Hz, 1H), 8.57 – 8.47 (m, 1H), 8.30 (d, *J* = 8.8 Hz, 1H), 7.25 – 7.13 (m, 5H), 5.64 – 5.38 (m, 3H), 4.82 – 4.61 (m, 1H), 3.59 – 3.37 (m, 1H), 3.33 – 3.19 (m, 1H), 3.17 – 3.06 (m, 1H), 2.22 (s, 6H), 1.55 – 1.38 (m, 12H), 1.29 – 1.20 (m, 3H), 0.81 (d, *J* = 6.5 Hz, 3H), 0.72 (d, *J* = 6.6 Hz, 3H).

^{13}C NMR (101 MHz, $CDCl_3$) δ 168.5, 162.8, 147.3, 144.4, 142.9, 137.2, 129.7, 128.4, 126.7, 124.1, 54.9, 50.0, 48.7, 38.6, 25.8, 23.8, 22.5, 21.7, 21.5, 10.6.

^{11}B NMR (128 MHz, $CDCl_3$) δ -24.4 (t, *J* = 84.7 Hz, 1B).

HRMS (ESI): Calcd for $C_{30}H_{45}BN_6O_2Na^+$ $[M+Na]^+$: 555.3589, found: 555.3595.

IR (neat, cm^{-1}): v 3361, 2958, 2927, 2857, 2319, 1671, 1556, 1521, 1399, 1236.
$[\alpha]_D^{25}$= -4.24 (c = 0.4, $CHCl_3$).

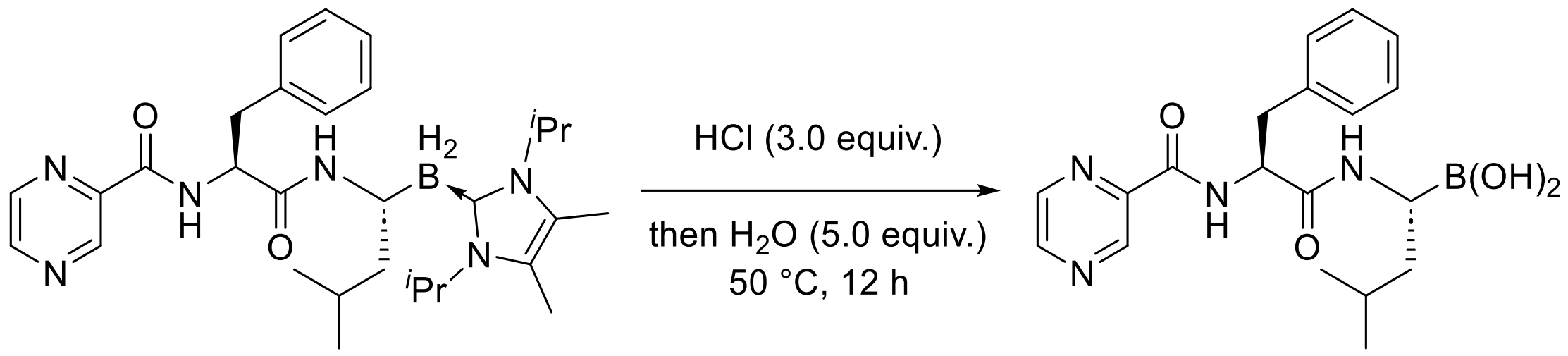

16a **16b**

Under argon, to an oven dried 4 mL reaction tube equipped with magnetic stir bar was added Masked-Bortezomib **16a** (26.6 mg, 0.05 mmol, 1.0 equiv.) and THF (500 μL). HCl (4 M in dioxane, 38 μL, 0.15 mmol, 3.0 equiv.) was then added slowly, followed by the addition of degassed H_2O (4.5 mg, 0.25 mmol, 5.0 equiv.). The reaction mixture was then stirred at 50 °C for 12 h. Upon completion, the solvent was removed in vacuo and the resulting residue was dissolved in 50% CH_3CN: 50% H_2O with 0.1% TFA. After filtration through a 0.2 μm filter, the solution was purified by reverse phase HPLC (3.0 mL/min, 10 – 60% CH_3CN/H_2O over 35 min, both CH_3CN and H_2O containing 0.1% TFA) and the desired fractions were concentrated to remove the CH_3CN and water to give product **16b** (56%, 10.7 mg).

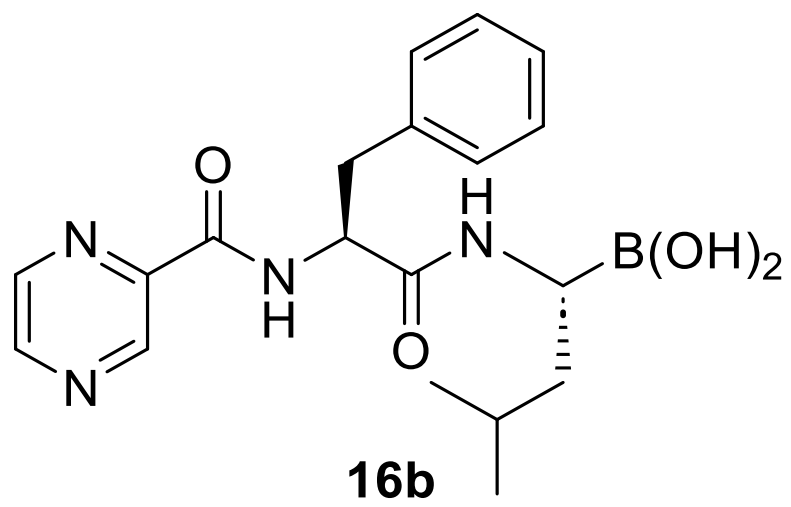

16b

16b: Yield: 10.7 mg, 56%, >20: 1 d.r., white solid.
^{1}H NMR (400 MHz, CD_3CN/D_2O 10:1) δ 9.15 – 9.10 (m, 1H), 8.77 – 8.72 (m, 1H), 8.63 – 8.57 (m, 1H), 7.29 – 7.14 (m, 6H), 4.82 – 4.74 (m, 1H), 3.20 – 3.17 (m, 1H), 3.15 – 3.03 (m, 1H), 3.03 – 2.91 (m, 1H), 1.51 – 1.32 (m, 2H), 1.31 – 1.20 (m, 1H), 0.99 – 0.63 (m, 6H).
^{13}C NMR (101 MHz, CD_3CN/D_2O 10:1) δ 172.1, 164.3, 148.7, 145.0, 144.5, 144.5, 137.8, 130.4, 129.4, 127.8, 118.7, 54.9, 40.2, 38.6, 25.9, 23.6, 22.0.
^{11}B NMR (128 MHz, CD_3CN/D_2O 10:1) δ 29.0 (s, 1B).
HRMS (ESI): Calcd for $C_{19}H_{25}BN_4O_4Na^+$ $[M+Na]^+$: 407.1861, found: 407.1865.
$[\alpha]_D^{25}$= -23.7 (c = 0.3, CD_3CN/D_2O 5:1).
The NMR data and optical rotation matched with those reported in literature.[20]

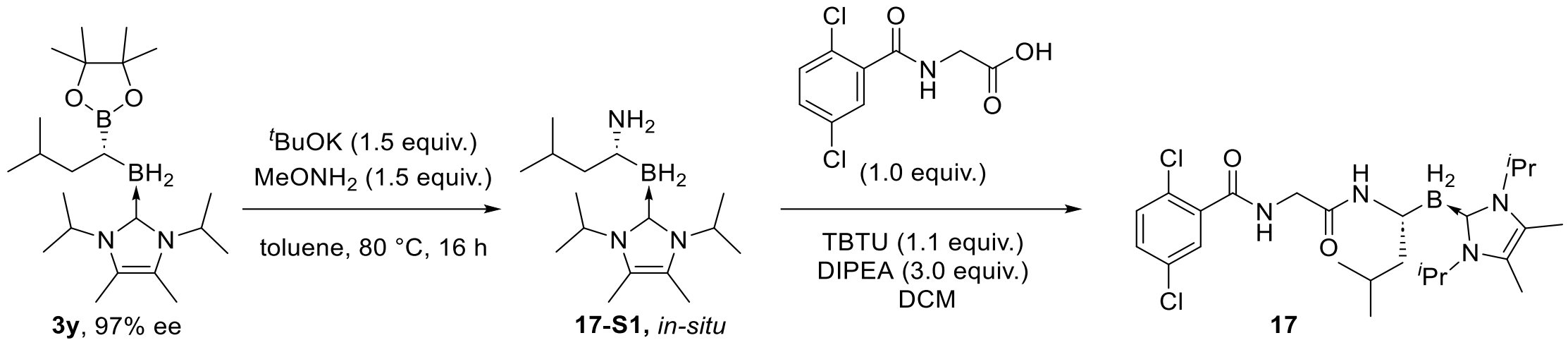

Under argon, to an oven dried 4 mL reaction tube equipped with magnetic stir bar was added **3y** (39.0 mg, 0.1 mmol, 1.0 equiv.), $MeONH_2$ (1.5 M in THF, 100 µL, 0.15 mmol, 1.5 equiv.), ^{t}BuOK (16.8 mg, 0.15 mmol, 1.5 equiv.) and toluene (500 µL). The reaction mixture was then heated at 80 °C and stirred for 16 h. Upon completion, the reaction was quenched with H_2O and extracted with ethyl acetate (3 × 3 mL). The combined organic phases were dried over $MgSO_4$, and the solvent was removed in vacuo. The resulting crude residue **17-S1** was used for the next step without purification.

Under N_2, to an oven dried 20 mL vial equipped with magnetic stir bar was added crude reaction mixture **17-S1** (*in-situ*, 0.1 mmol, 1.0 equiv.), 2-(1H-benzotriazole-1yl)-1,1,3,3,tetramethylaminium tetrafluoroborate (TBTU; 35.3 mg, 0.11 mmol, 1.1 equiv.), 2-(2,5-dichlorobenzamido)acetic acid (24.8 mg, 0.1 mmol, 1.0 equiv.) and dry DCM (1 mL). The reaction was cooled to 0 °C and ethyldiisopropylamine (DIPEA; 38.8 mg, 0.3 mmol, 3.0 equiv.) was added slowly. The reaction mixture was then stirred at room temperature for 12 h. Upon completion, the reaction was quenched with H_2O and extracted with DCM (3 × 3 mL). The combined organic phases were dried over $MgSO_4$, and the solvent was removed in vacuo. The resulting residue was purified by flash column chromatography to give product **17** (71%, 36.0 mg).

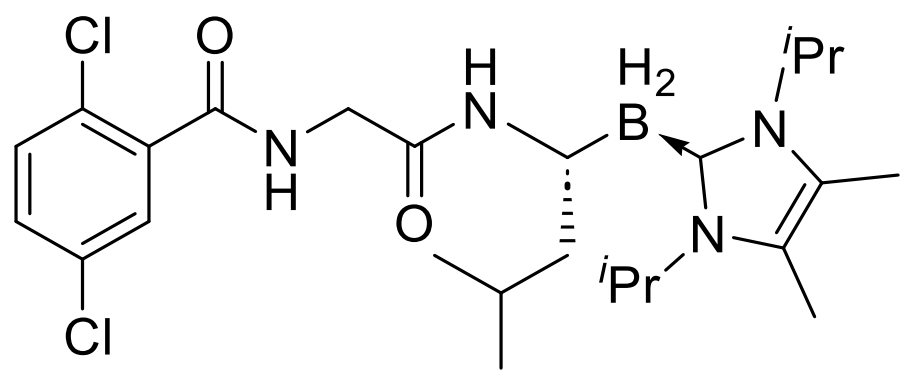

17

17: Yield: 36.0 mg, 71%, 91% ee, white solid, m.p. = 177 – 182 °C, R_f = 0.2 (*n*-hexane/ethyl acetate = 1/2).

^{1}H NMR (400 MHz, $CDCl_3$) δ 7.69 – 7.42 (m, 1H), 7.34 – 7.29 (m, 2H), 7.13 – 6.97 (m, 1H), 5.47 – 5.38 (m, 2H), 5.37 – 5.29 (m, 1H), 4.19 – 3.86 (m, 2H), 3.54 – 3.39 (m, 1H), 2.22 (s, 6H), 1.61 – 1.55 (m, 1H), 1.57 – 1.30 (m, 12H), 1.10 – 1.01 (m, 1H), 0.90 – 0.52 (m, 7H).

^{13}C NMR (101 MHz, $CDCl_3$) δ 165.9, 165.1, 136.3, 133.1, 131.5, 131.3, 130.0, 129.4, 124.2, 50.0, 48.8, 43.7, 25.9, 23.9, 22.4, 21.7, 21.5, 10.6.

^{11}B NMR (128 MHz, $CDCl_3$) δ -24.2 (t, *J* = 85.3 Hz, 1B).

HRMS (ESI): Calcd for $C_{25}H_{39}BCl_2N_4O_2Na^+$ $[M+Na]^+$: 531.2435, found: 531.2440.

IR (neat, cm^{-1}): *ν* 3291, 2972, 2930, 2294, 1638, 1511, 1460, 1401, 1220, 1098.

Chiral HPLC (AD-H, ^{i}PrOH/*n*-hexane = 20/80, 1.0 mL/min) t_R 5.7 min (minor), 7.4 min (major).

$[\alpha]_D^{25}$= 49.5 (*c* = 0.1, $CHCl_3$).

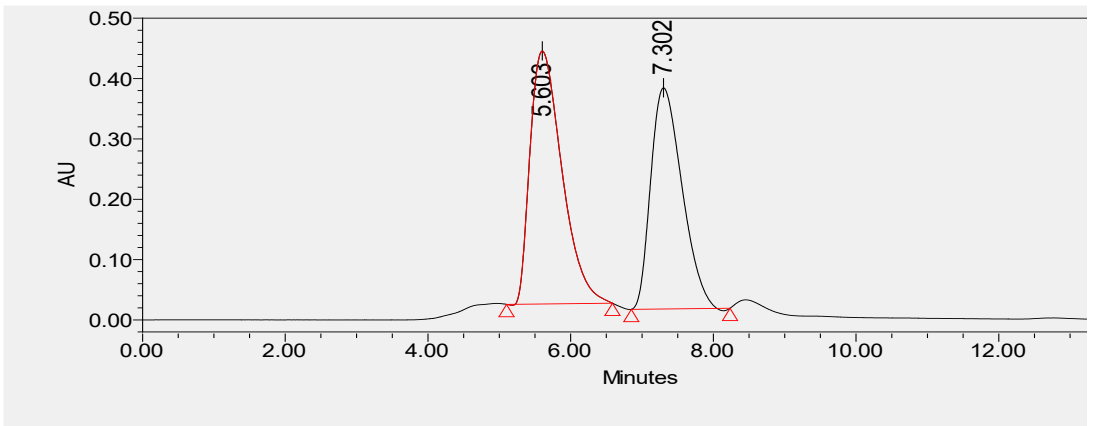

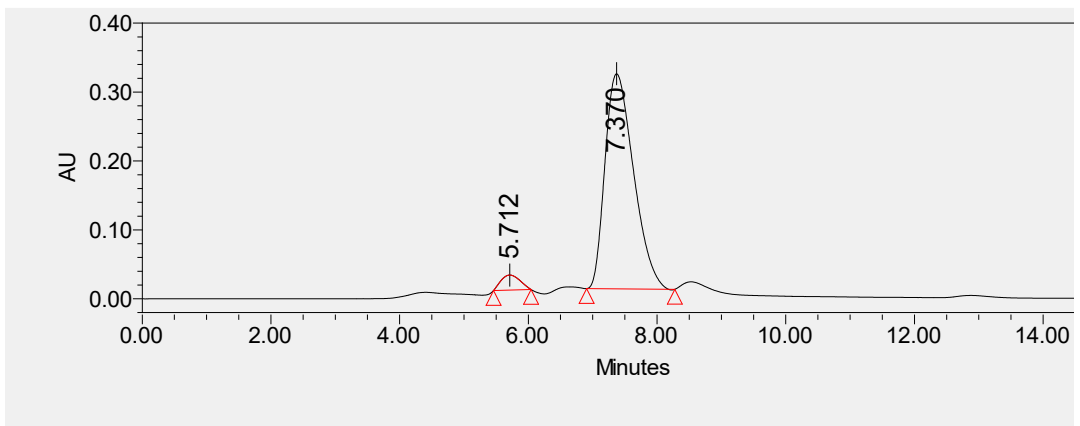

	Retention Time	Area	% Area
1	5.603	11555304	50.80
2	7.302	11192844	49.20

	Retention Time	Area	% Area
1	5.712	448510	4.45
2	7.370	9632042	95.55

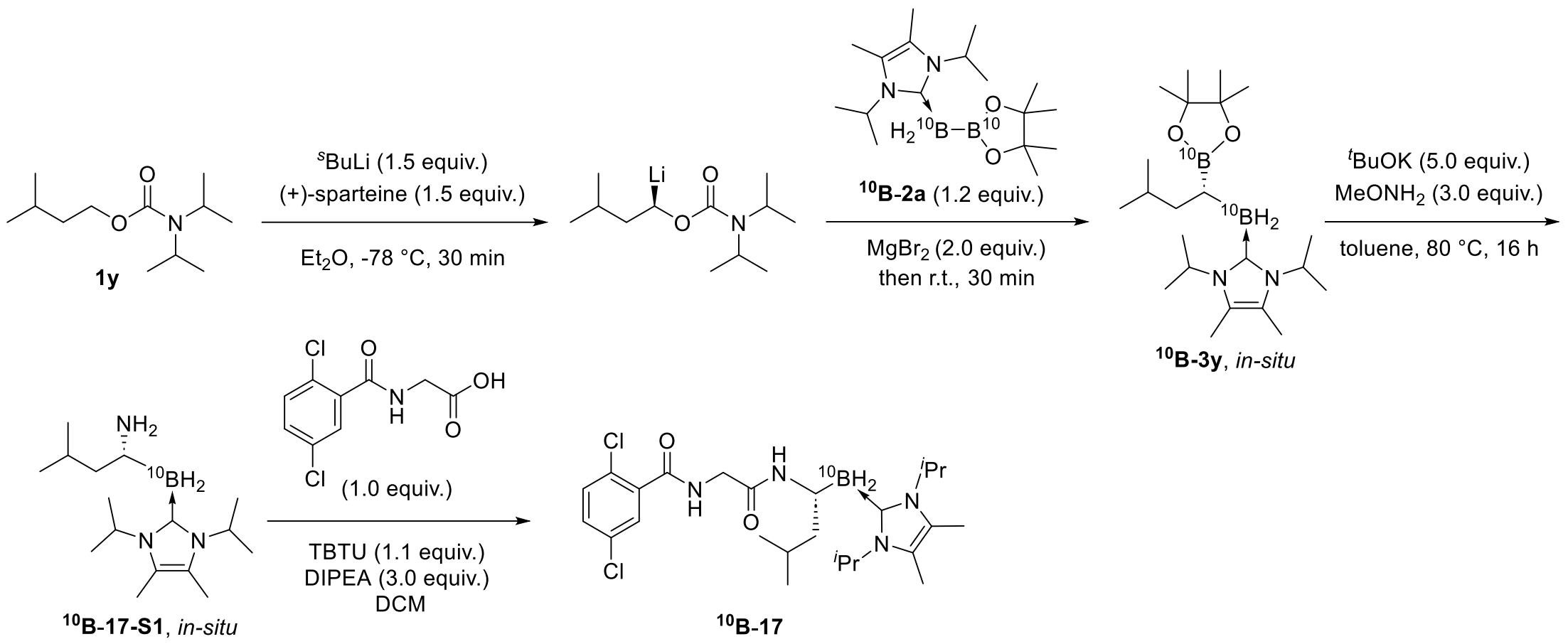

Under argon, to an oven dried 4 mL reaction tube equipped with a magnetic stir bar and a septum was charged with **1y** (21.5 mg, 0.1 mmol, 1.0 equiv.), (+)-sparteine (35.2 mg, 0.15 mmol, 1.5 equiv.) and Et_2O (500 μL). The reaction was cooled to -78 °C and stirred for 10 min. ^{s}BuLi (1.3 M in cyclohexane, 0.12 mL, 0.15 mmol, 1.5 equiv.) was then slowly added and the mixture was stirred vigorously (without splashing) at -78 °C for 30 min. After that, a solution of the **2a** (38.4 mg, 0.12 mmol, 1.2 equiv.) in Et_2O (1.2 mL) was added dropwise. The reaction mixture was stirred at this temperature for an additional 10 min. $MgBr_2$ (freshly made from Mg and 1,2-dibromoethane, 0.5 M in Et_2O, 0.4 mL, 0.2 mmol, 2.0 equiv.) was then added dropwise and the reaction mixture was stirred at -78 °C for 20 min. The reaction mixture was then warmed to room temperature and stirred for another 30 min. Upon completion, the reaction was quenched with aqueous NH_4Cl and extracted with Et_2O (3 × 3 mL). The combined organic phases were dried over $MgSO_4$, and the solvent was removed in vacuo. The resulting crude residue **^{10}B-3y** was used for the next step without purification.

Under argon, to an oven dried 4 mL reaction tube equipped with magnetic stir bar was added **^{10}B-3y** (*in-situ*, 0.1 mmol, 1.0 equiv.), $MeONH_2$ (1.5 M in THF, 200 μL, 0.3 mmol, 3.0 equiv.), ^{t}BuOK (56.0 mg, 0.5 mmol, 5.0 equiv.) and toluene (500 μL). The reaction mixture was then heated at 80 °C and stirred for 16 h. Upon completion, the reaction was quenched with H_2O and extracted with ethyl acetate (3 × 3 mL). The combined organic phases were dried over $MgSO_4$, and the solvent was removed in vacuo. The resulting crude residue **^{10}B-17-S1** was used for the next step without purification.

Under N_2, to an oven dried 20 mL vial equipped with magnetic stir bar was added crude reaction mixture **^{10}B-17-S1** (*in-situ*, 0.1 mmol, 1.0 equiv.), 2-(1H-benzotriazole-1yl)-1,1,3,3,tetramethylaminium tetrafluoroborate (TBTU; 35.3 mg, 0.11 mmol, 1.1 equiv.), 2-(2,5-dichlorobenzamido)acetic acid (24.8 mg, 0.1 mmol, 1.0 equiv.) and dry DCM (1 mL). The reaction was cooled to 0 °C and and ethyldiisopropylamine (DIPEA; 38.8 mg, 0.3 mmol, 3.0 equiv.) was added slowly. The reaction mixture was then stirred at room temperature for 12 h. Upon completion, the reaction was quenched with H_2O and extracted with DCM (3 × 3 mL). The combined organic phases were dried over $MgSO_4$, and the solvent was removed in vacuo. The resulting residue was purified by flash column chromatography to give product **^{10}B-17** (35%, 18.2 mg).

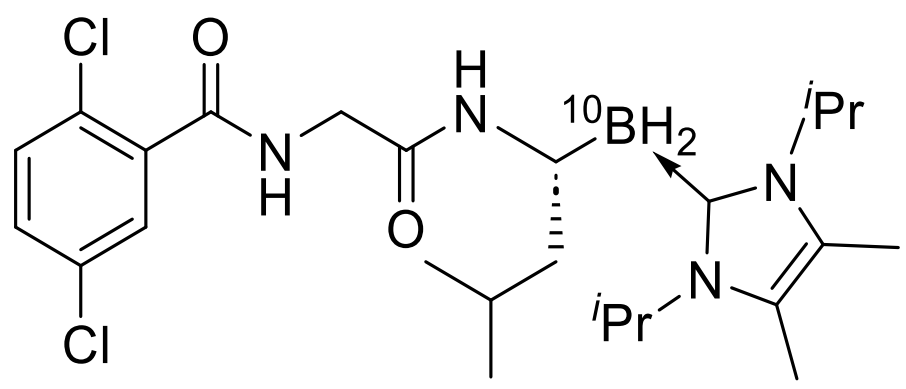

^{10}B-17

^{10}B-17: Three-step yield: 18.2 mg, 35%, 91% ee, white solid, m.p. = 176 – 180 °C, R_f = 0.2 (*n*-hexane/ethyl acetate = 1/2).

^{1}H NMR (400 MHz, $CDCl_3$) δ 7.63 – 7.54 (m, 1H), 7.33 – 7.29 (m, 2H), 7.11 – 7.02 (m, 1H), 5.69 – 4.95 (m, 3H), 4.11 – 3.74 (m, 2H), 3.58 – 3.20 (m, 1H), 2.22 (s, 6H), 1.60 – 1.57 (m, 1H), 1.74 – 1.32 (m, 12H), 1.08 – 1.01 (m, 1H), 0.96 – 0.62 (m, 7H).

^{13}C NMR (101 MHz, $CDCl_3$) δ 165.9, 165.0, 136.3, 133.1, 131.5, 131.3, 129.9, 129.4, 124.2, 50.0, 48.7, 43.7, 25.9, 23.9, 22.4, 21.7, 21.5, 10.6.

HRMS (ESI): Calcd for $C_{25}H_{39}{}^{10}BCl_2N_4O_2Na^+$ $[M+Na]^+$: 530.2472, found: 530.2467.

IR (neat, cm^{-1}): *ν* 3293, 2951, 2927, 2866, 2307, 1642, 1516, 1461, 1401, 1260, 1097, 1046, 1020.

Chiral HPLC (AD-H, ^{i}PrOH/*n*-hexane = 5/95, 1.0 mL/min) t_R 15.8 min (minor), 26.6 min (major).

$[\alpha]_D^{25}$= 54.4 (*c* = 0.1, $CHCl_3$).

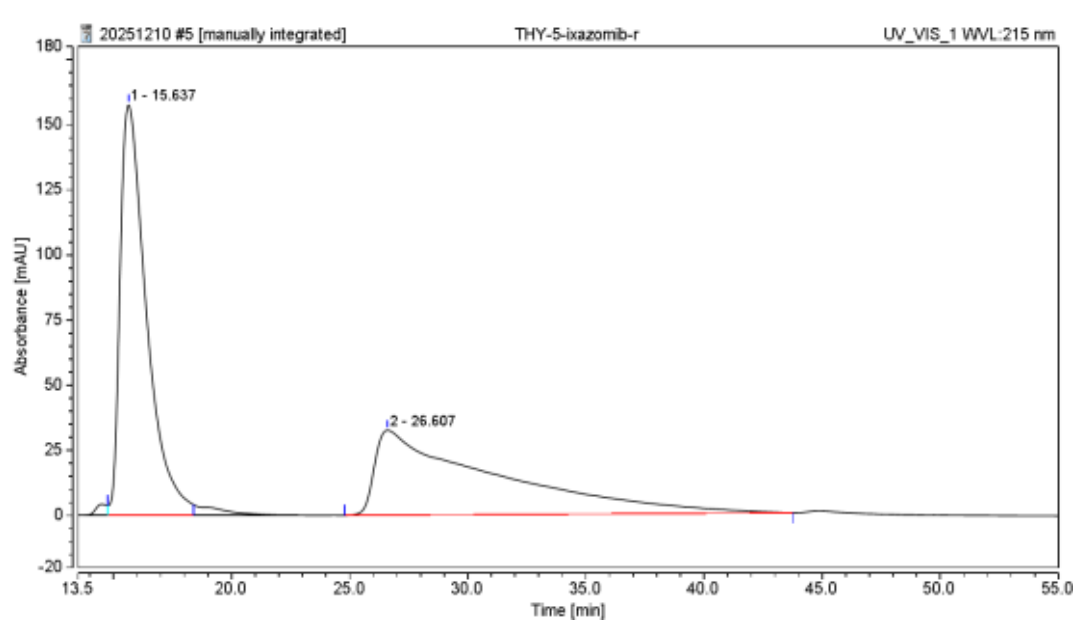

	Retention Time	Area	% Area
1	15.637	203.4222	51.73
2	26.607	189.8406	48.27

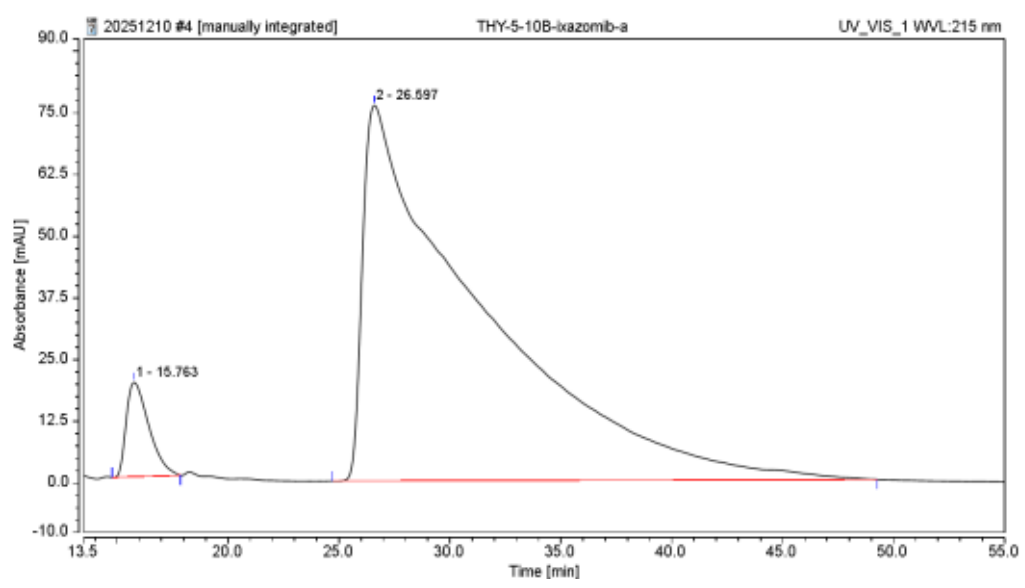

	Retention Time	Area	% Area
1	15.763	22.658	4.62
2	26.597	467.7731	95.38

9. Synthesis of fully substituted CDBAs and their applications.

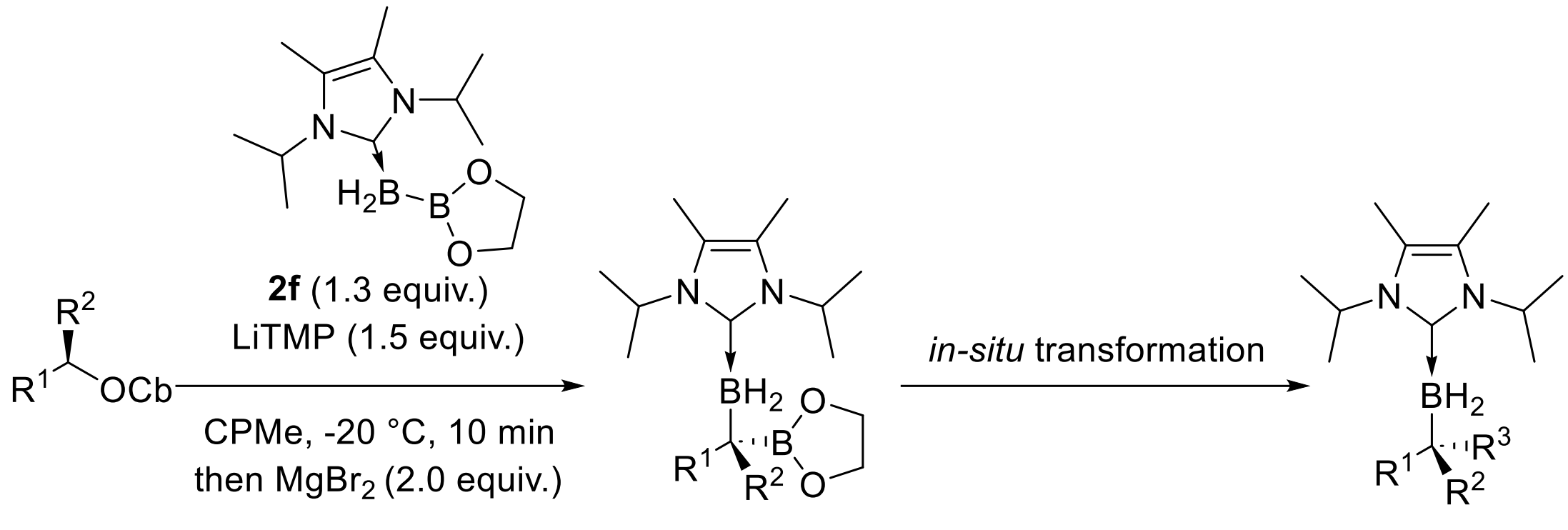

Preparation for LiTMP:

At 0 °C, to an oven dried 4 mL reaction tube charged with 2,2,6,6-tetramethylpiperidine (183.6 mg, 1.3 mmol, 1.0 equiv.) and dry cyclopentyl methyl ether (CPMe, 500 μL) was added ^{n}BuLi (1.7 M in *n*-hexane, 0.76 mL, 1.3 mmol, 1.0 equiv.) dropwise. The reaction was then warmed to room temperature and stirred for 30 min to give a clear colorless 1.0 M solution of LiTMP.

General procedure:

Under argon, to an oven dried 4 mL reaction tube equipped with a magnetic stir bar and a septum was charged with benzylcarbamate (0.1 mmol, 1.0 equiv.), diboron reagent **2f** (34.3 mg, 0.13 mmol, 1.3 equiv.) and CPMe (500 μL). The reaction was cooled to -20 °C and stirred for 10 min. Freshly prepared LiTMP (1.0 M in CPMe, 0.15 mL, 0.15 mmol, 1.5 equiv.) was slowly added and the mixture was stirred at -20 °C for 10 min. Then $MgBr_2$ (freshly made from Mg and 1,2-dibromoethane, 0.5 M in Et_2O, 0.4 mL, 0.2 mmol, 2.0 equiv.) was added dropwise. The reaction mixture was then warmed to room temperature and stirred for another 30 min. Upon completion, the reaction was quenched with aqueous NH_4Cl and extracted with Et_2O (3 × 3 mL). The combined organic phases were dried over $MgSO_4$, and the solvent was removed in vacuo. The resulting crude CDBA was used for the next step without purification. The racemic product was prepared using the same procedure, with diboron reagent **2a** substituted for diboron reagent **2f** and THF substituted for CPMe.

*Note: Generally, the newly prepared CDBA from diboron **2f** is not stable towards silica gel purification. Therefore, in-situ transformation was conducted.*

***In-situ* oxidation**:

Under argon, to an oven dried 4 mL reaction tube charged with a magnetic stir bar was added crude residue from the last step, sodium perborate tetrahydrate (77.0 mg, 0.5 mmol, 5.0 equiv.) and THF/H_2O (1:1, 2 mL in total). The reaction mixture was vigorously stirred at room temperature and monitored by the TLC. Upon completion, the reaction was quenched with H_2O and extracted with Et_2O (3 × 3 mL). The combined organic phases were dried over $MgSO_4$, and the solvent was removed in vacuo. The resulting residue was purified by flash column chromatography to afford the desired product.

Note: Generally, oxidation could be finished within 30 min.

***In-situ* protodeboration**:

Under argon, to an oven dried 4 mL reaction tube equipped with magnetic stir bar was added the crude CDBA from last step, ^{t}BuOK (56.1 mg, 0.5 mmol, 5.0 equiv.) and toluene (500 μL). The reaction mixture was then heated at 80 °C and stirred for 16 h. Upon completion, the reaction was quenched with H_2O and extracted with ethyl acetate (3 × 3 mL). The combined organic phases were dried over $MgSO_4$, and the solvent was removed in vacuo. The resulting crude residue was purified by flash column chromatography to afford the desired product.

***In-situ* deuterodeborylation**:

Under argon, to an oven dried 4 mL reaction tube equipped with magnetic stir bar was added crude residual from the last step, ^{t}BuOK (56.1 mg, 0.5 mmol, 5.0 equiv.), MeOD (16.5 mg, 0.5 mmol, 5.0 equiv.) and toluene (500 μL). The reaction mixture was then heated at 80 °C and stirred for 16 h. Upon completion, the reaction mixture was then passed through a short pad of silica gel, washed with ethyl acetate and concentrated in vacuo. The resulting crude residue was purified by flash column chromatography to afford the desired product.

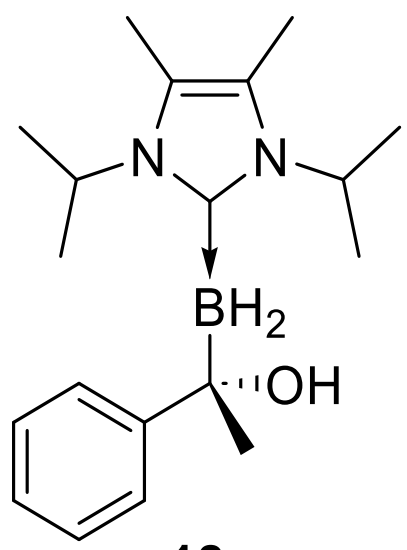

18a

18a: Yield: 14.3 mg, 47%, 99% ee, colorless oil, R_f = 0.4 (*n*-hexane/ethyl acetate = 3/1).

^{1}H NMR (400 MHz, $CDCl_3$) δ 7.32 – 7.27 (m, 2H), 7.22 – 7.09 (m, 2H), 7.01 – 6.90 (m, 1H), 5.40 – 5.18 (m, 2H), 2.16 (s, 6H), 1.32 (d, *J* = 7.1 Hz, 6H), 1.25 (s, 3H), 1.09 (d, *J* = 7.2 Hz, 6H).

^{13}C NMR (101 MHz, $CDCl_3$) δ 157.0, 127.7, 124.3, 124.0, 123.6, 50.4, 33.2, 21.4, 21.0, 10.7.

^{11}B NMR (128 MHz, $CDCl_3$) δ -20.5 (t, *J* = 86.3 Hz, 1B).

HRMS (ESI): Calcd for $C_{19}H_{31}BN_2ONa^+$ $[M+Na]^+$: 337.2422, found: 337.2425.

IR (neat, cm^{-1}): *v* 2919, 2850, 2359, 1402, 1264, 1093.

Chiral HPLC (OX-H, ^{i}PrOH/*n*-hexane = 5/95, 1.0 mL/min) t_R 25.4min (minor), 27.1 min (major).

$[\alpha]_D^{25}$ = -35.67 (*c* = 0.6, $CHCl_3$).

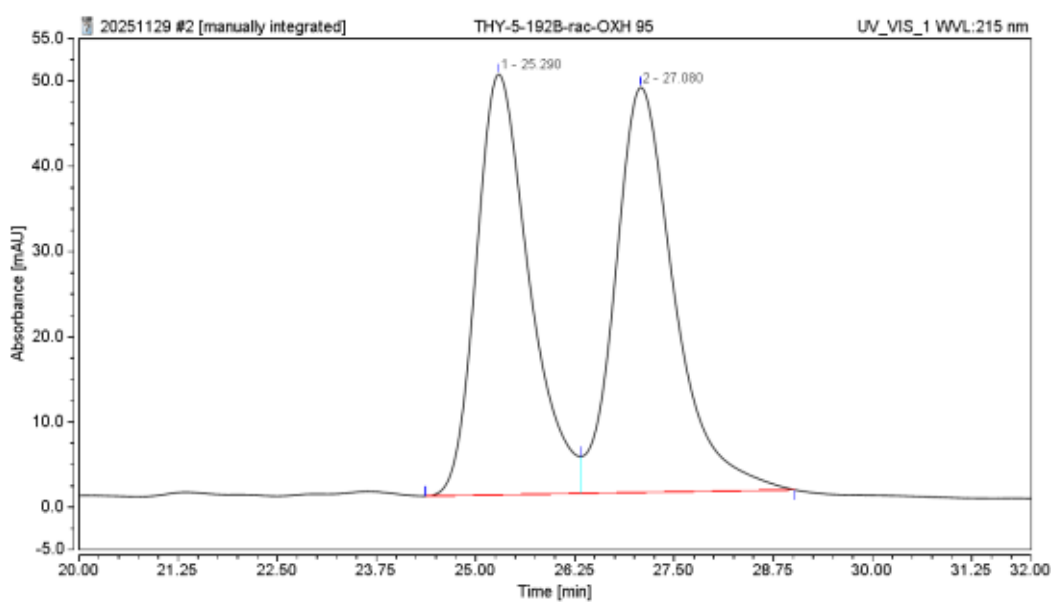

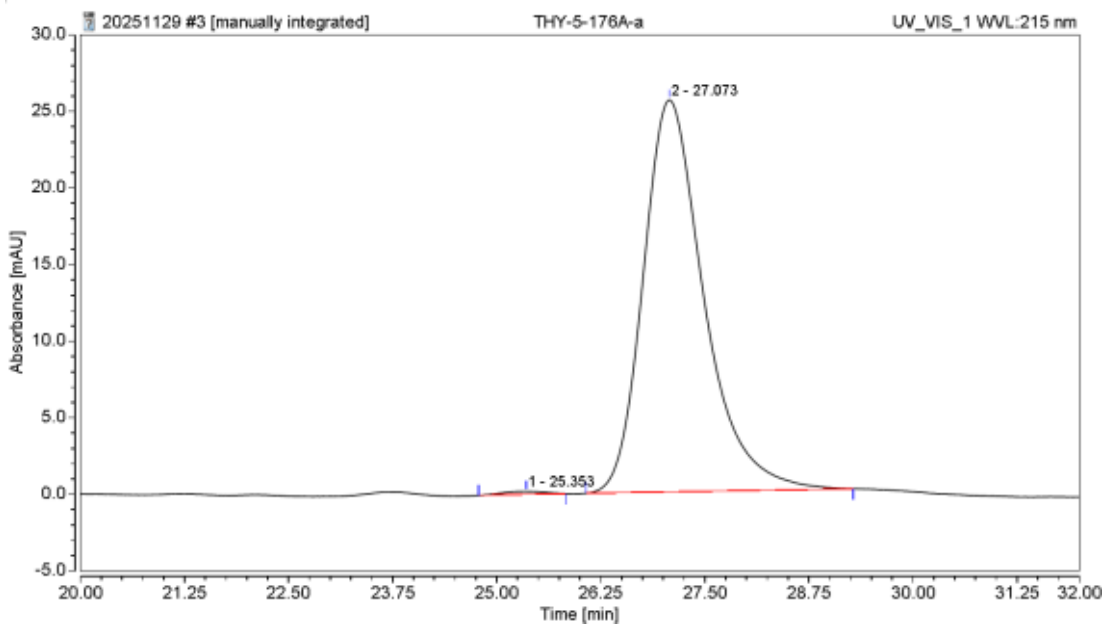

	Retention Time	Area	% Area
1	25.290	38.113	48.16
2	27.080	41.0298	51.84

	Retention Time	Area	% Area
1	25.353	0.1177	0.54
2	27.073	21.5937	99.46

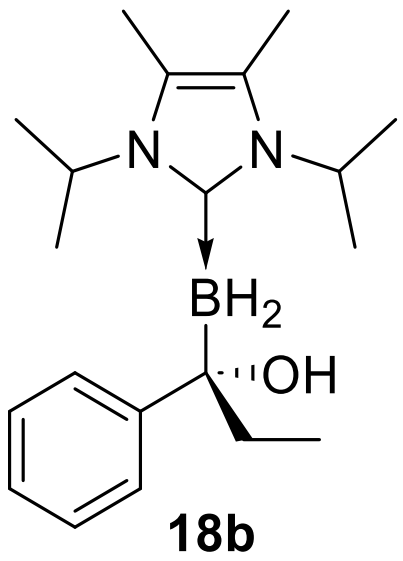

18b

18b: Yield: 20.0 mg, 61%, 96% ee, white solid, m.p. = 132 – 137 °C, R_f = 0.4 (*n*-hexane/ethyl acetate = 3/1).

^{1}H NMR (400 MHz, $CDCl_3$) δ 7.22 – 7.11 (m, 4H), 6.97 – 6.90 (m, 1H), 5.38 – 5.16 (m, 2H), 2.21 – 2.06 (m, 7H), 2.04 – 1.93 (m, 1H), 1.32 (d, *J* = 7.1 Hz, 6H), 1.04 (d, *J* = 7.1 Hz, 6H), 0.70 (t, *J* = 7.3 Hz, 3H).

^{13}C NMR (101 MHz, $CDCl_3$) δ 154.5, 127.6, 124.8, 123.9, 123.3, 50.3, 36.3, 21.5, 21.0, 10.7, 7.8.

^{11}B NMR (128 MHz, $CDCl_3$) δ -20.5 (t, *J* = 86.5 Hz, 1B).

HRMS (ESI): Calcd for $C_{20}H_{33}BN_2ONa^+$ [M+Na]$^+$: 351.2578, found: 351.2582.

IR (neat, cm^{-1}): *ν* 3391. 2972. 2330, 1439, 1401, 1261, 1112, 1070.

Chiral HPLC (OX-H, ^{i}PrOH/*n*-hexane = 5/95, 1.0 mL/min) t_R 11.4 min (minor), 13.9 min (major).

$[\alpha]_D^{25}$= -25.50 (*c* = 0.6, $CHCl_3$).

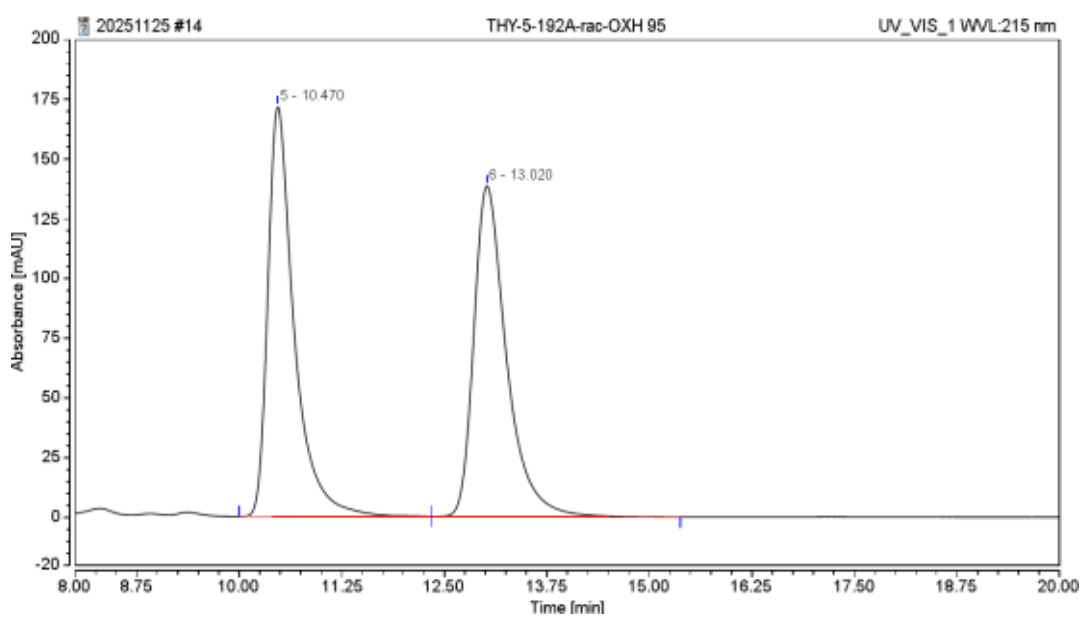

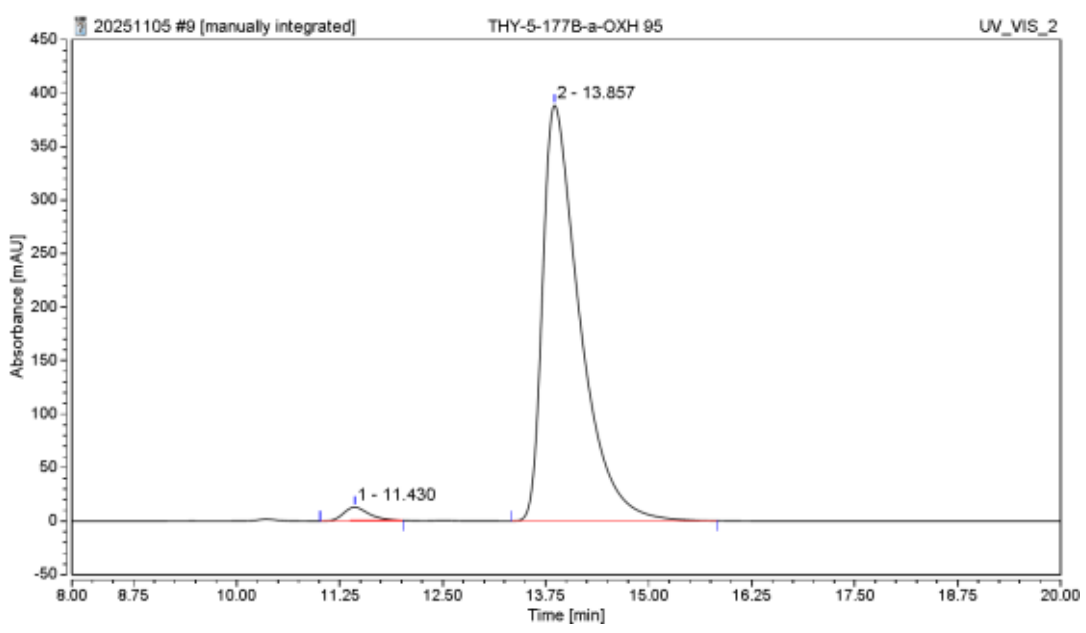

	Retention Time	Area	% Area
1	10.470	22.1623	49.64
2	13.020	22.4863	50.36

	Retention Time	Area	% Area
1	11.430	4.5397	2.19
2	13.857	202.5654	97.81

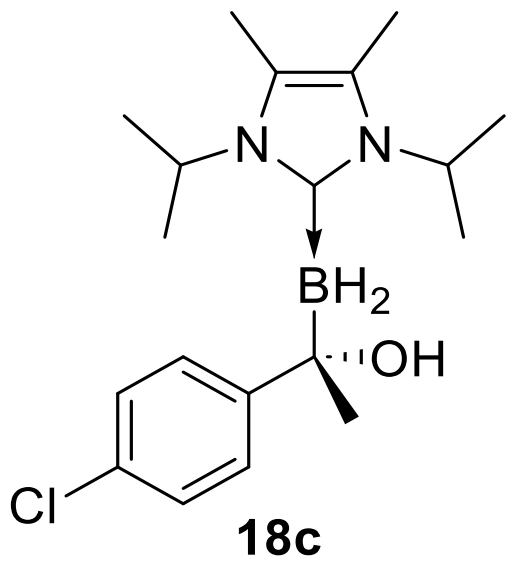

18c

18c: Yield: 21.9 mg, 63%, 91% ee, white solid, m.p. = 172 – 175 °C, R_f = 0.4 (*n*-hexane/ethyl acetate = 4/1).

^{1}H NMR (400 MHz, $CDCl_3$) δ 7.24 – 7.16 (m, 2H), 7.16 – 7.01 (m, 2H), 5.40 – 5.10 (m, 2H), 2.17 (s, 6H), 1.32 (d, *J* = 7.0 Hz, 6H), 1.13 (d, *J* = 7.1 Hz, 6H), 0.06 (s, 3H).

^{13}C NMR (126 MHz, $CDCl_3$) δ 155.5, 129.0, 127.5, 125.9, 124.1, 50.3, 33.0, 21.4, 21.0, 10.7, 1.1.

^{11}B NMR (128 MHz, $CDCl_3$) δ -20.4 (t, *J* = 86.4 Hz, 1B).

HRMS (ESI): Calcd for $C_{19}H_{30}BN_2OClNa^+$ $[M+Na]^+$: 371.2032, found: 371.2035.

IR (neat, cm^{-1}): *v* 2972, 2937, 2851, 2302, 1681, 0397, 1318, 1090, 1010.

Chiral HPLC (OX-H, ^{i}PrOH/*n*-hexane = 10/90, 1.0 mL/min) t_R 9.3 min (minor), 11.6 min (major).

$[\alpha]_D^{25}$= -39.67 (*c* = 0.6, $CHCl_3$).

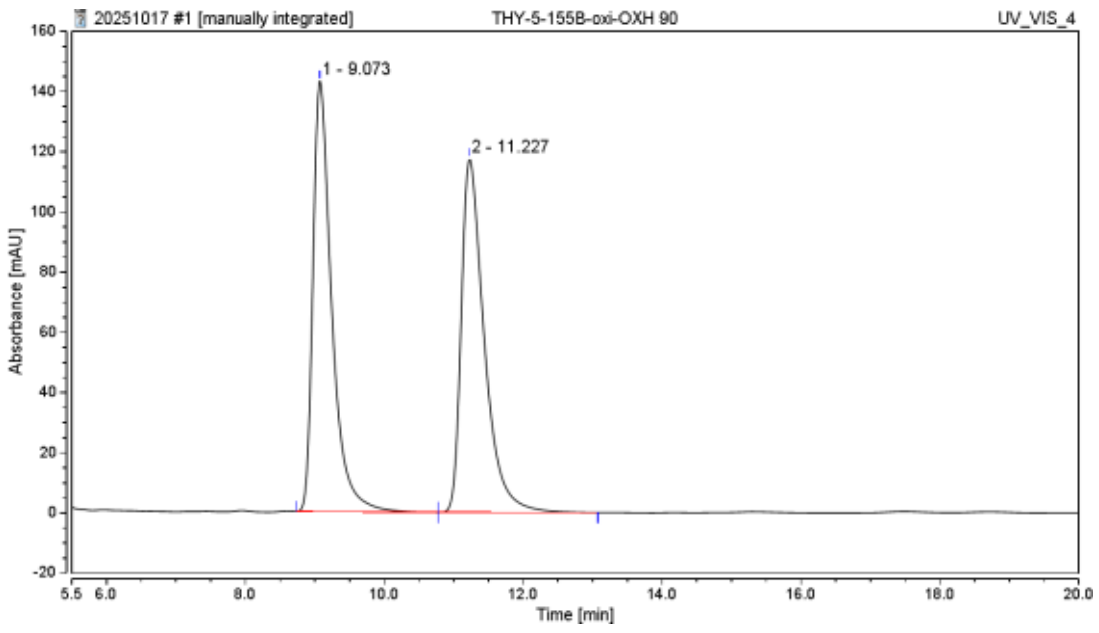

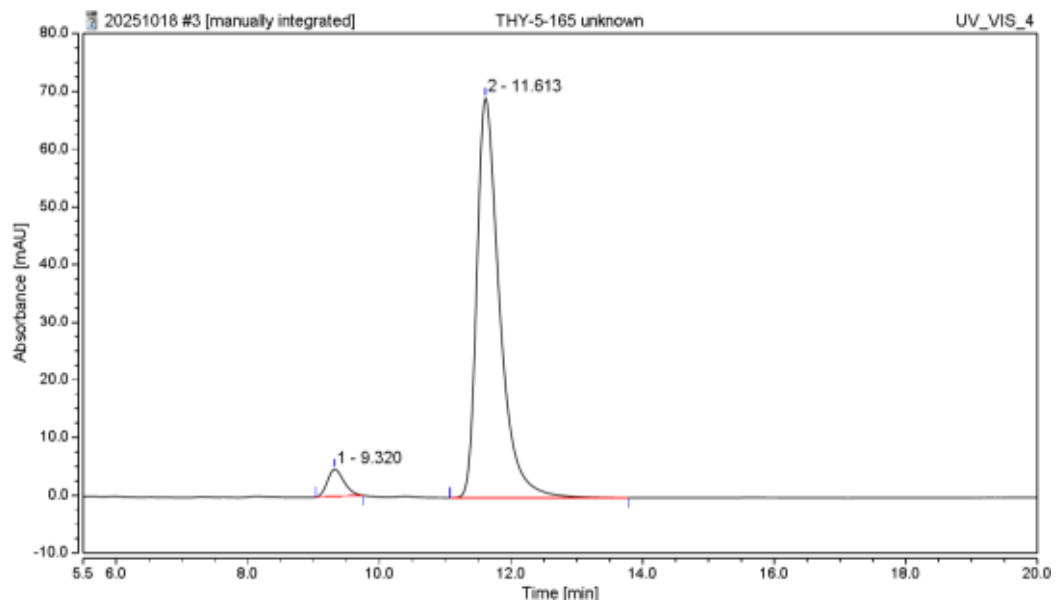

	Retention Time	Area	% Area
1	9.073	44.8349	49.89
2	11.227	45.0401	50.11

	Retention Time	Area	% Area
1	9.320	10.7989	4.56
2	11.613	225.8378	95.44

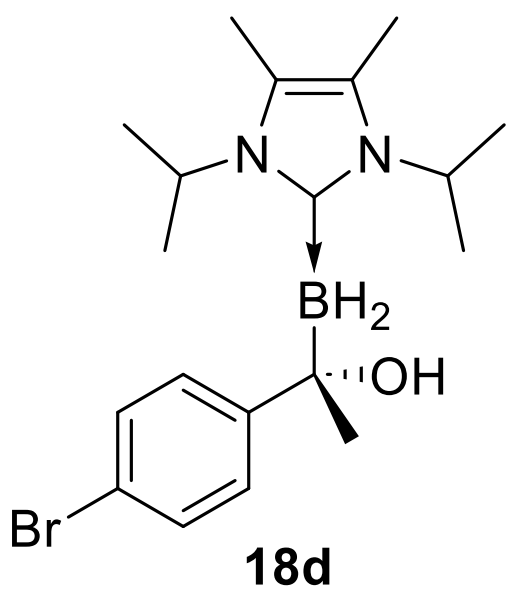

18d

18d: Yield: 29.8 mg, 76%, 84% ee, colorless oil, R_f = 0.4 (*n*-hexane/ethyl acetate = 3/1).

^{1}H NMR (400 MHz, $CDCl_3$) δ 7.31 – 7.25 (m, 2H), 7.20 – 7.08 (m, 2H), 5.72 – 4.55 (m, 2H), 2.16 (s, 6H), 1.32 (d, *J* = 7.1 Hz, 6H), 1.12 (s, 6H), 0.06 (s, 3H).

^{13}C NMR (126 MHz, $CDCl_3$) δ 156.1, 130.4, 126.3, 124.1, 117.1, 50.3, 32.9, 21.4, 21.0, 10.7, 1.1.
^{11}B NMR (128 MHz, $CDCl_3$) δ -20.6 (t, J = 86.5 Hz, 1B).
HRMS (ESI): Calcd for $C_{19}H_{30}BN_2OBrNa^+$ $[M+Na]^+$: 415.1527, found: 415.1530.
IR (neat, cm^{-1}): v 2970, 2928, 2851, 2306, 1627, 1396, 1260, 1074, 1036, 1006.
Chiral HPLC (OX-H, ^{i}PrOH/n-hexane = 10/90, 1.0 mL/min) t_R 9.8 min (major), 13.0 min (minor).
$[\alpha]_D^{25}$= -60.17 (c = 0.6, $CHCl_3$).

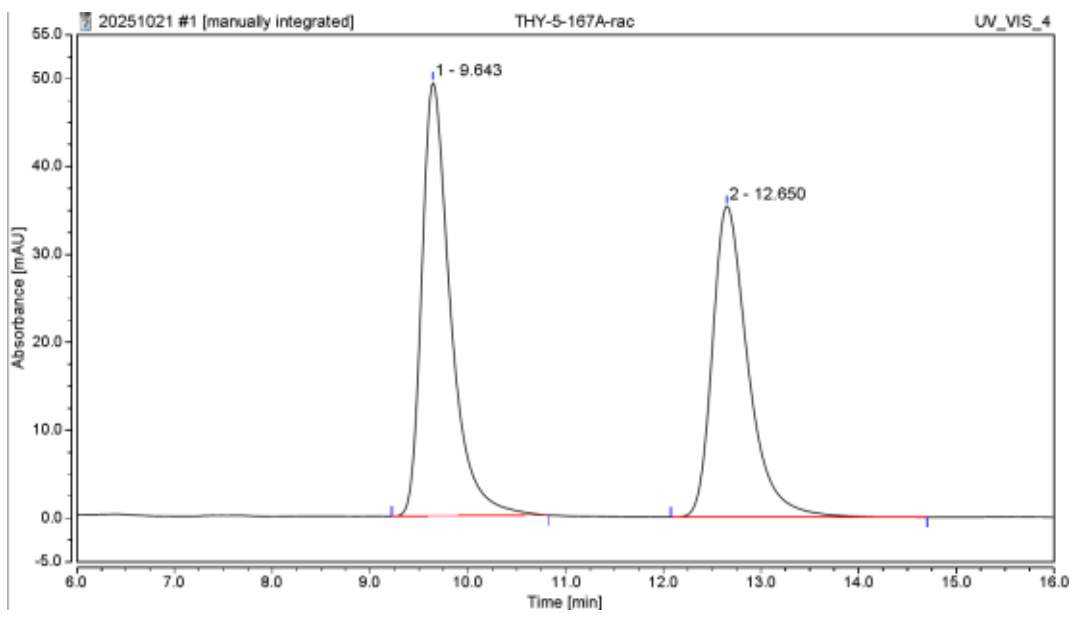

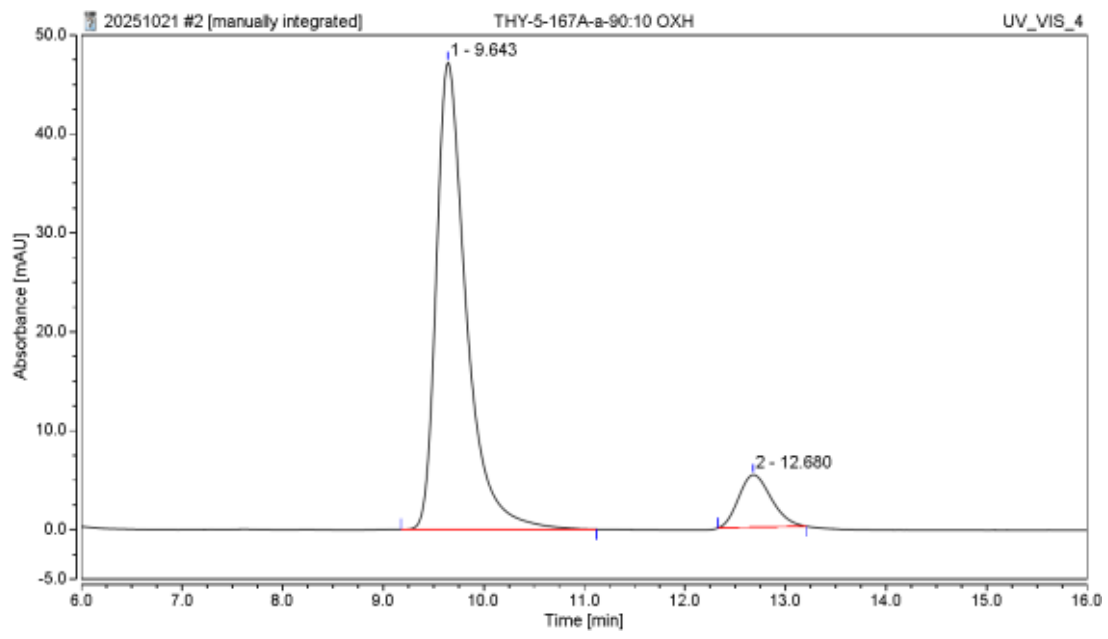

	Retention Time	Area	% Area
1	9.643	16.7565	52.84
2	12.650	14.9551	47.16

	Retention Time	Area	% Area
1	9.757	66.3826	91.81
2	12.960	5.9180	8.19

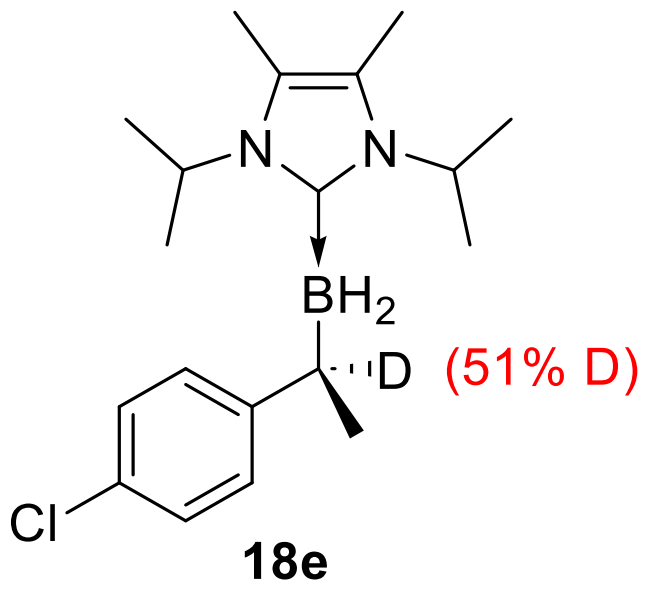

18e

18e: Yield: 23.6 mg, 71%, 87% ee, colorless oil, R_f = 0.4 (n-hexane/ethyl acetate = 3/1).
^{1}H NMR (400 MHz, $CDCl_3$) δ 7.15 – 6.97 (m, 2H), 6.97 – 6.80 (m, 2H), 5.52 – 4.99 (m, 2H), 2.17 (s, 6H), 2.05 – 1.91 (m, 0.49H), 1.40 (d, J = 7.1 Hz, 6H), 1.31 – 1.23 (m, 3H), 1.13 (d, J = 7.1 Hz, 6H).
^{1}H{^{11}B} NMR (400 MHz, $CDCl_3$) δ 7.13 – 7.02 (m, 2H), 7.00 – 6.82 (m, 2H), 5.52 – 4.99 (m, 2H), 2.17 (s, 6H), 2.01 – 1.90 (m, 0.49H), 1.57 (s, 2H) (BH_2), 1.49 – 1.37 (m, 6H), 1.27 (s, 3H), 1.19 – 1.05 (m, 6H).
^{2}H NMR (61 MHz, CDCl3) δ 2.05 – 1.91 (m, 0.51D).
^{13}C NMR (101 MHz, $CDCl_3$) δ 155.3, 127.8, 127.8, 127.6, 123.7, 49.8, 23.0, 23.0, 21.7, 21.1, 10.6.
^{11}B NMR (128 MHz, $CDCl_3$) δ -21.2 (t, J = 86.4 Hz, 1B).
HRMS (ESI): Calcd for $C_{19}H_{29}DBN_2ClNa^+$ $[M+Na]^+$: 356.2146, found: 356.2141.
IR (neat, cm^{-1}): v 2937, 2858, 2330, 1510, 1319, 1278.
Chiral HPLC (OJ-3, ^{i}PrOH/n-hexane = 5/95, 1.0 mL/min) t_R 10.3 min (major), 17.7 min (minor).
$[\alpha]_D^{25}$= 65.96 (c = 0.7, $CHCl_3$).

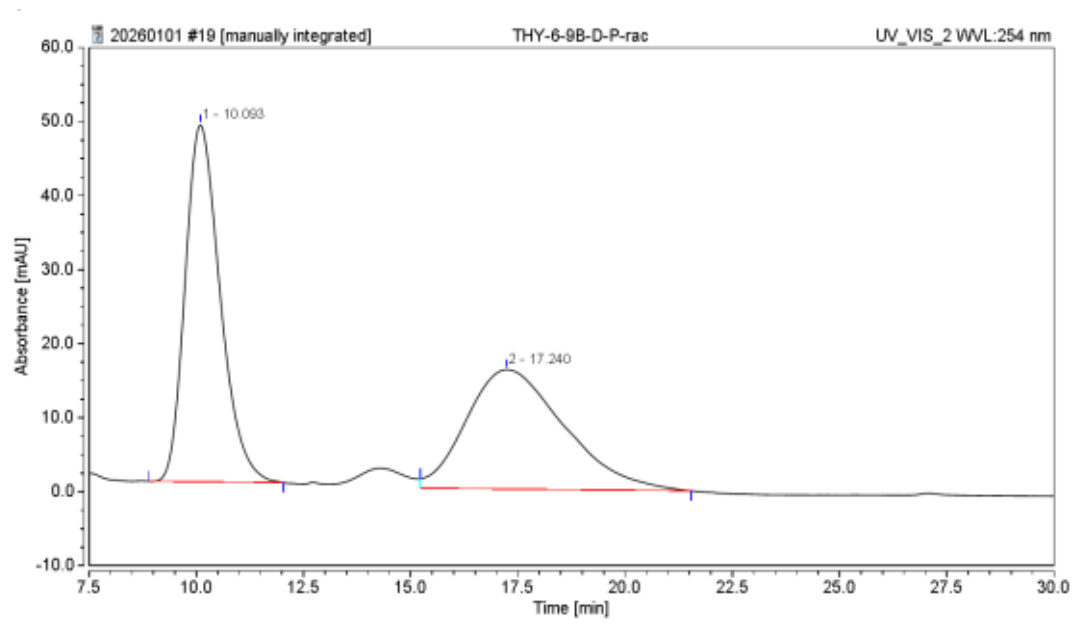

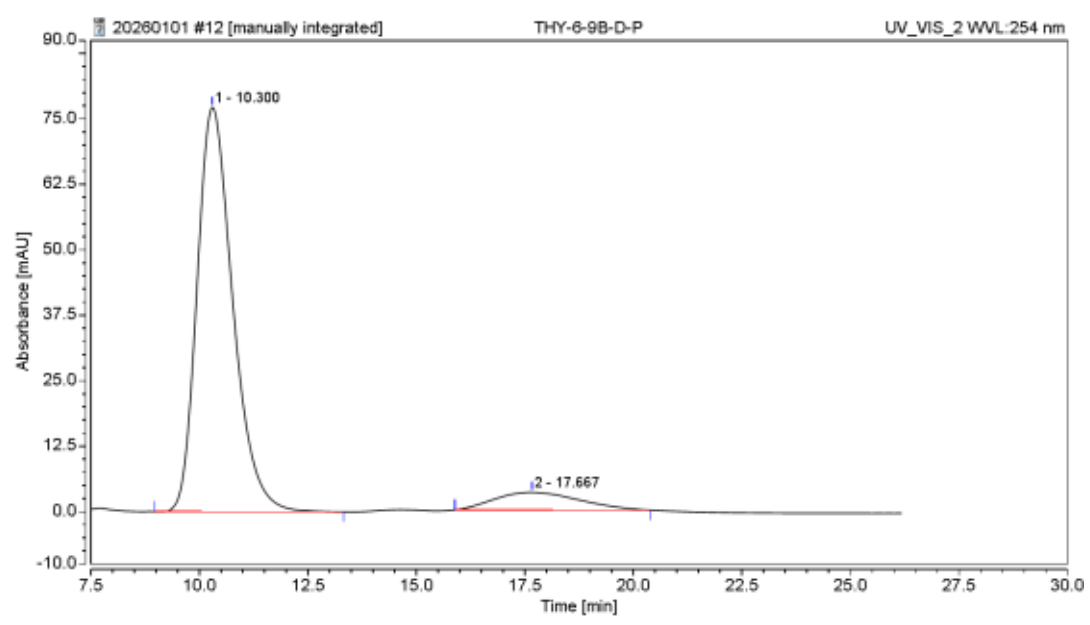

	Retention Time	Area	% Area
1	10.093	45.5559	51.65
2	17.240	42.6389	48.35

	Retention Time	Area	% Area
1	10.300	73.5323	93.51
2	17.667	5.1039	6.49

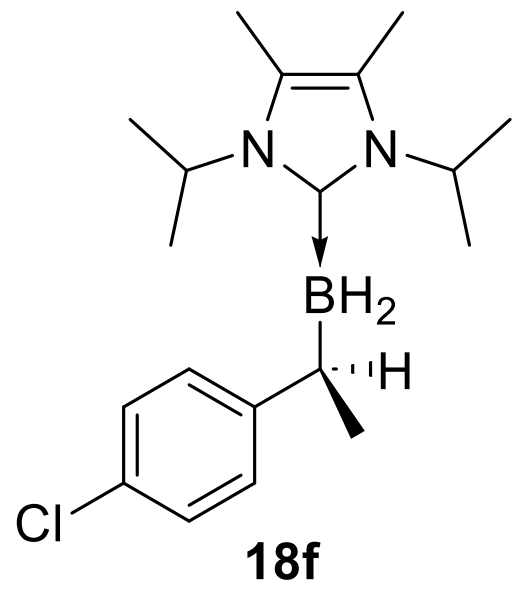

18f

18f: Yield: 22.6 mg, 68%, 92% ee, colorless oil, R_f = 0.4 (*n*-hexane/ethyl acetate = 3/1).
^{1}H NMR (400 MHz, $CDCl_3$) δ 7.11 – 7.02 (m, 2H), 7.01 – 6.82 (m, 2H), 5.40 – 5.10 (m, 2H), 2.17 (s, 6H), 2.02 – 1.91 (m, 1H), 1.39 (d, *J* = 7.1 Hz, 6H), 1.31 – 1.23 (m, 3H), 1.13 (d, *J* = 7.1 Hz, 6H).
^{13}C NMR (101 MHz, $CDCl_3$) δ 155.3, 127.8, 127.8, 127.6, 123.7, 49.8, 27.9, 23.0, 21.7, 21.1, 10.6.
^{11}B NMR (128 MHz, $CDCl_3$) δ -21.1 (t, *J* = 86.7 Hz, 1B).
HRMS (ESI): Calcd for $C_{19}H_{30}BN_2ClNa^+$ $[M+Na]^+$: 355.2083, found: 355.2086.
IR (neat, cm^{-1}): *ν* 2975, 2937, 2855, 2358, 2310, 1530, 1399, 1220.
Chiral HPLC (OJ-3, ^{i}PrOH/*n*-hexane = 5/95, 1.0 mL/min) t_R 8.3 min (major), 14.3 min (minor).
$[\alpha]_D^{25}$ = 67.70 (*c* = 0.7, $CHCl_3$).

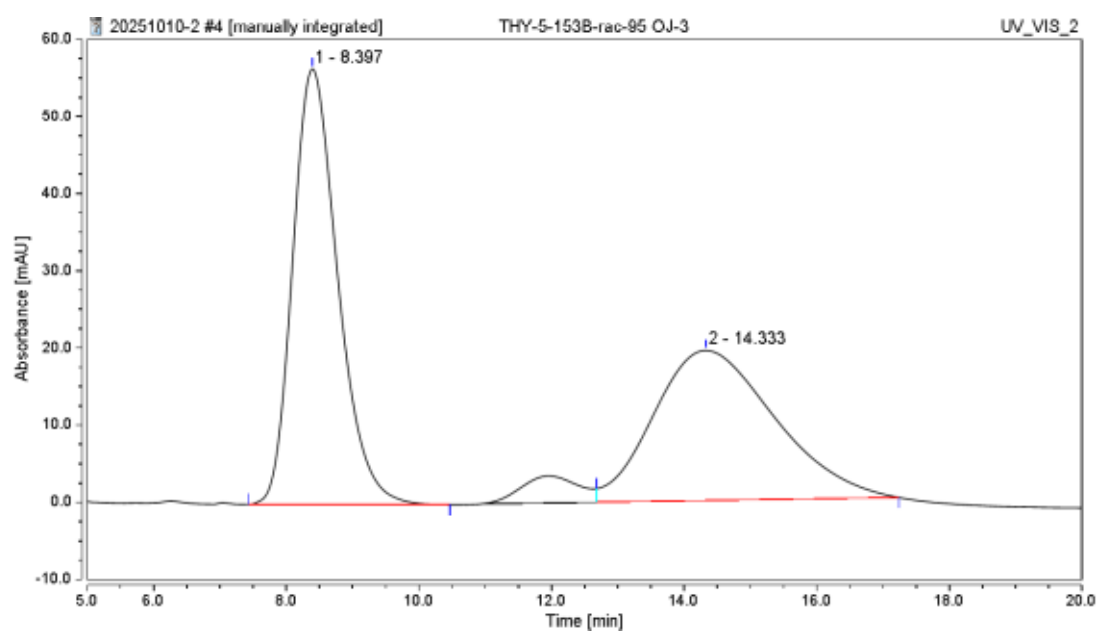

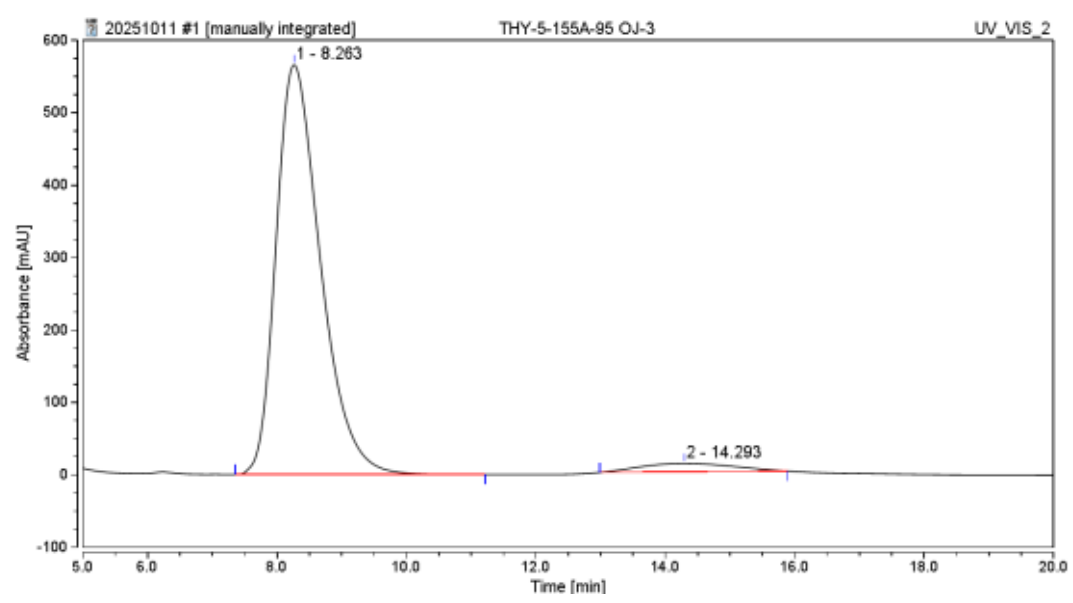

	Retention Time	Area	% Area
1	8.397	71.5070	49.09
2	14.323	74.1519	50.91

	Retention Time	Area	% Area
1	8.263	450.6385	95.89
2	14.293	19.2926	4.11

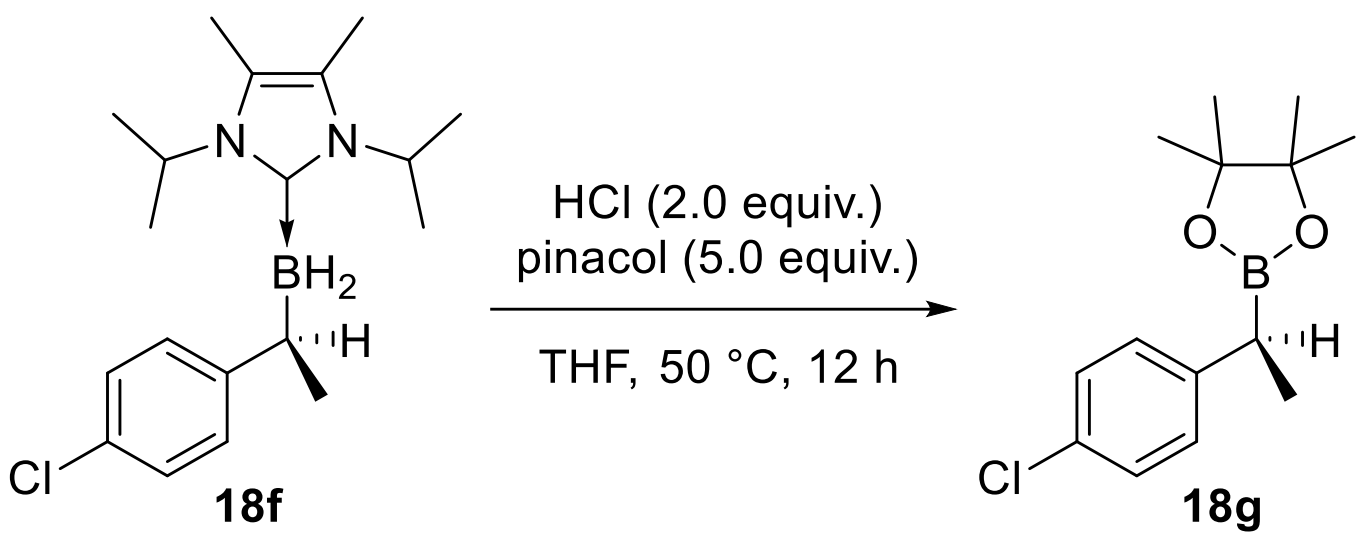

Under argon, to an oven dried 4 mL reaction tube equipped with a magnetic stir bar was added **18f** (33.2 mg, 0.1 mmol, 1.0 equiv.) and THF (1 mL). HCl (1 M in Et_2O, 200 µL, 0.2 mmol, 2.0 equiv.) was slowly added and the reaction mixture was stirred at room temperature for 1 h. After that, pinacol (59.1 mg, 0.5 mmol, 5.0 equiv.) was added and the reaction mixture was stirred at 50 °C for 12 h. Upon completion, the reaction was quenched with H_2O and extracted with ethyl acetate (3 × 3 mL). The combined organic phases were dried over $MgSO_4$, and the solvent was removed in vacuo. The residue resulting was purified by flash column chromatography to give the desired product **18g** (88%, 23.4 mg).

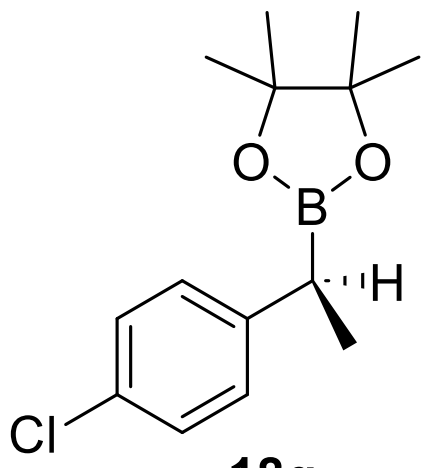

18g

18g: Yield: 23.4 mg, 88%, 85% ee, yellow oil, R_f = 0.2 (*n*-hexane).
Enantioselectivity was determined by the *in-situ* oxidized product.
^{1}H NMR (400 MHz, $CDCl_3$) δ 7.26 – 7.18 (m, 2H), 7.18 – 7.10 (m, 2H), 2.41 (q, *J* = 7.5 Hz, 1H), 1.31 (d, *J* = 7.5 Hz, 3H), 1.27 – 1.15 (m, 12H).
^{13}C NMR (126 MHz, $CDCl_3$) δ 143.6, 130.8, 129.2, 128.5, 83.6, 24.7, 24.7, 17.1.
^{11}B NMR (128 MHz, $CDCl_3$) δ 33.48 (s, 1B).
Chiral HPLC (OD-H, ^{i}PrOH/*n*-hexane = 5/95, 1.0 mL/min) t_R 8.9 min (minor), 9.8 min (major).
$[\alpha]_D^{25}$= -39.8 (*c* = 0.7, $CHCl_3$), Ref: $[\alpha]_D^{27}$= -40.1 (*c* = 0.75, $CHCl_3$).[21]
The NMR data and HPLC condition matched with those reported in literature.[21]

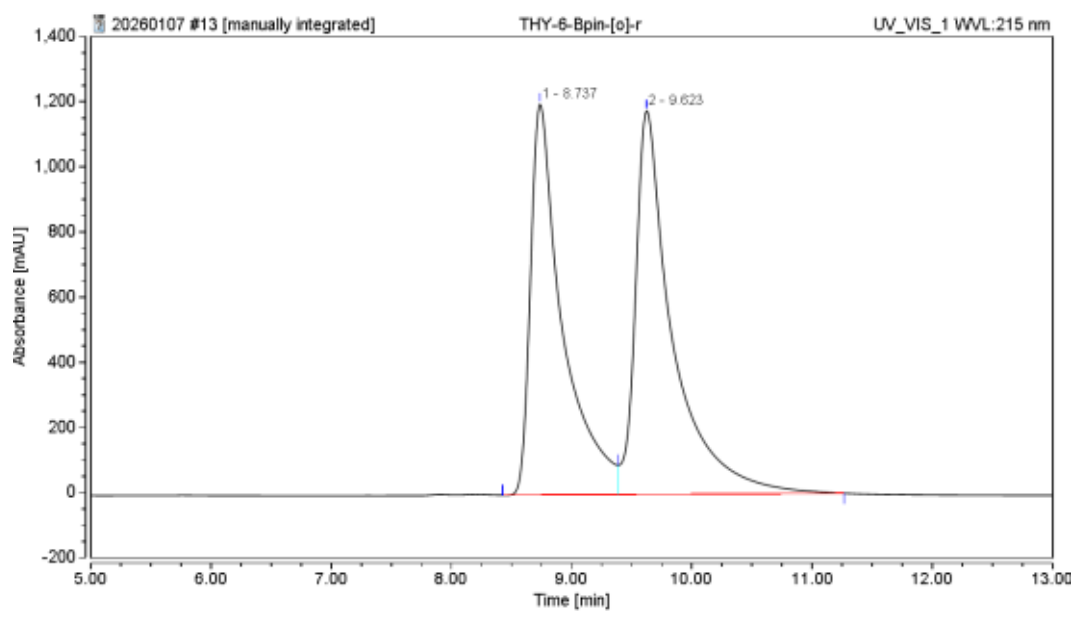

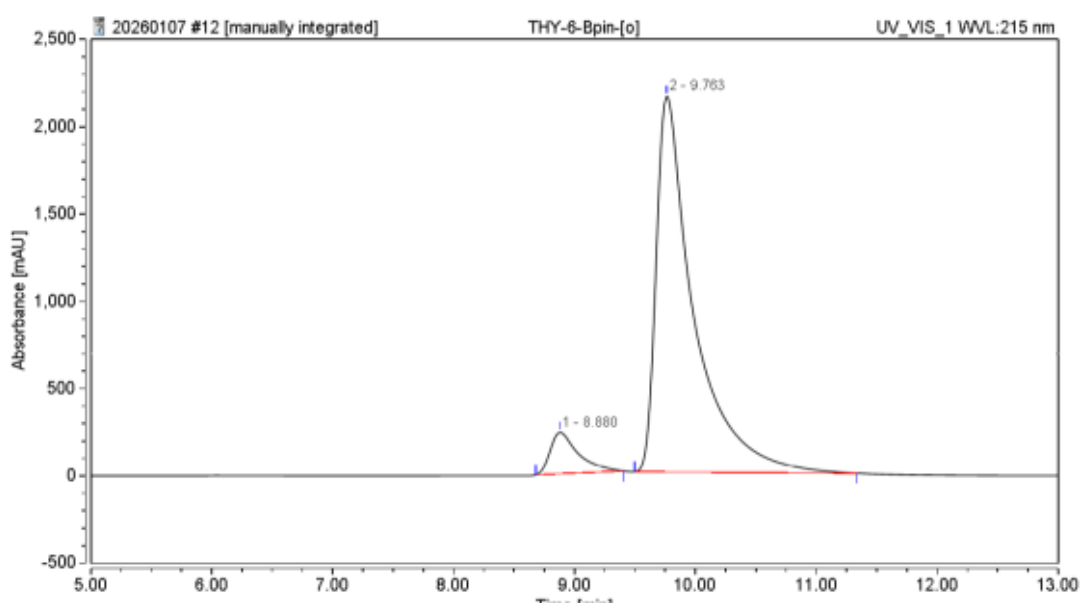

	Retention Time	Area	% Area
1	8.737	369.7911	46.83
2	9.623	419.9155	53.17

	Retention Time	Area	% Area
1	8.880	64.7586	7.69
2	9.763	776.8681	92.31

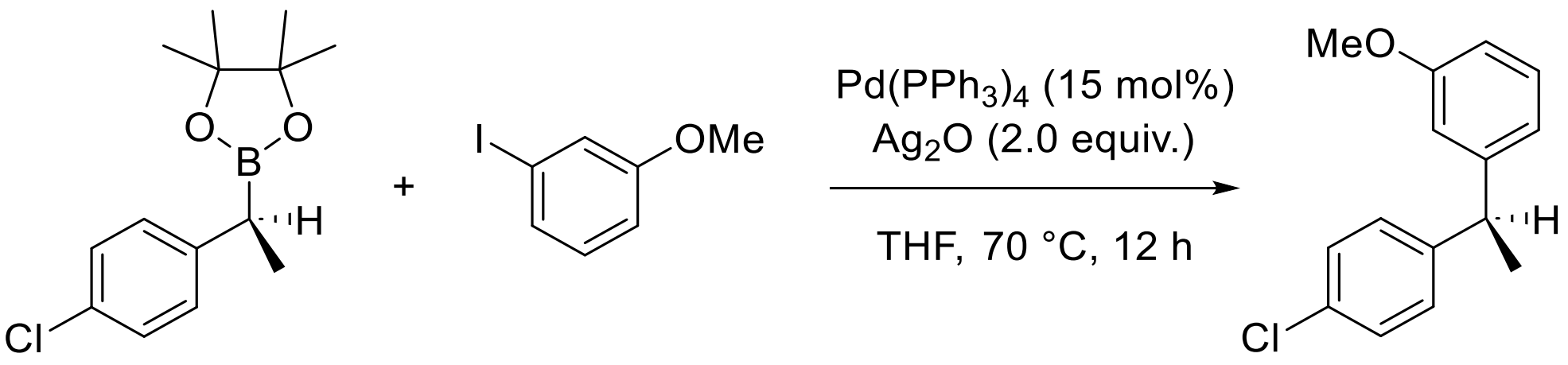

18g **18h**

According to a reported literature,[22] under argon, to an oven dried 4 mL reaction tube charged with a magnetic stir bar was added **18g** (39.9 mg, 0.15 mmol, 1.5 equiv.), 1-iodo-3-methoxybenzene (23.4 mg, 0.1 mmol, 1.0 equiv.), $Pd(PPh_3)_4$ (17.0 mg, 0.015 mmol, 15mol%), Ag_2O (46.3 mg, 0.2 mmol, 2.0 equiv.) and THF (1 mL). The reaction mixture stirred at 70 °C for 12 h. Upon completion, the reaction was quenched with H_2O and extracted with ethyl acetate (3 × 3 mL). The combined organic phases were dried over $MgSO_4$, and the solvent was removed in vacuo. The residue resulting was purified by flash column chromatography to give the desired product **18h** (78%, 17.5 mg).

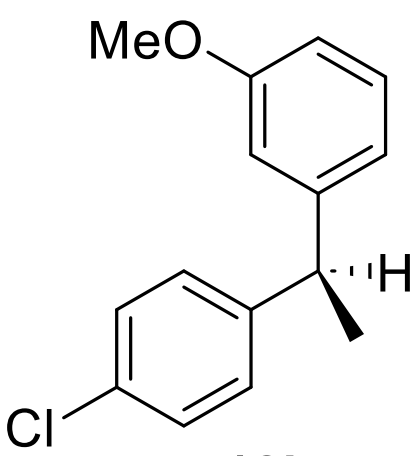

18h

18h: Yield: 17.5 mg, 78%, 72% ee, colorless oil, R_f = 0.5 (*n*-hexane).
^{1}H NMR (400 MHz, $CDCl_3$) δ 7.25 – 7.18 (m, 3H), 7.17 – 7.13 (m, 2H), 6.83 – 6.70 (m, 3H), 4.09 (q, *J* = 7.2 Hz, 1H), 3.77 (s, 3H), 1.60 (d, *J* = 7.2 Hz, 3H).
^{13}C NMR (126 MHz, $CDCl_3$) δ 159.8, 147.6, 144.8, 131.9, 129.6, 129.1, 128.6, 120.1, 113.9, 111.2, 553, 44.3, 21.9.
HRMS (ESI): Calcd for $C_{15}H_{16}ClO^+$ $[M+H]^+$: 247.0884, found: 247.0885.
IR (neat, cm^{-1}): *v* 2968, 2840, 1615, 1515, 1476, 1307, 1284, 1188, 1053.
Chiral HPLC (OJ-3, ^{i}PrOH/*n*-hexane = 2/98, 1.0 mL/min) t_R 10.7 min (major), 12.3 min (minor).
$[\alpha]_D^{25}$= 2.14 (*c* = 0.3, $CHCl_3$).

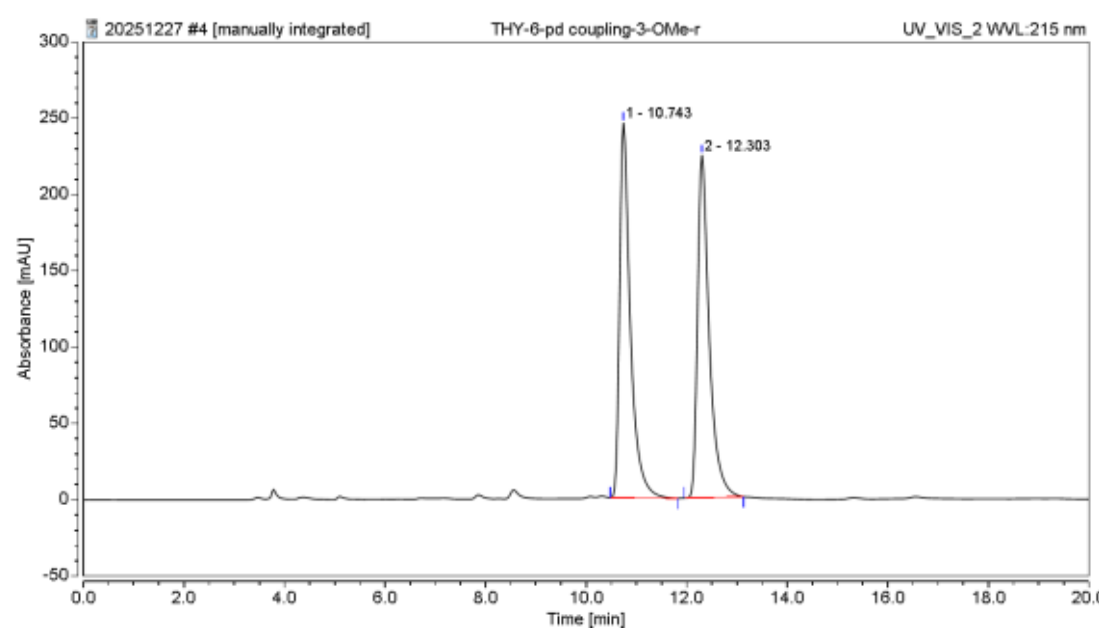

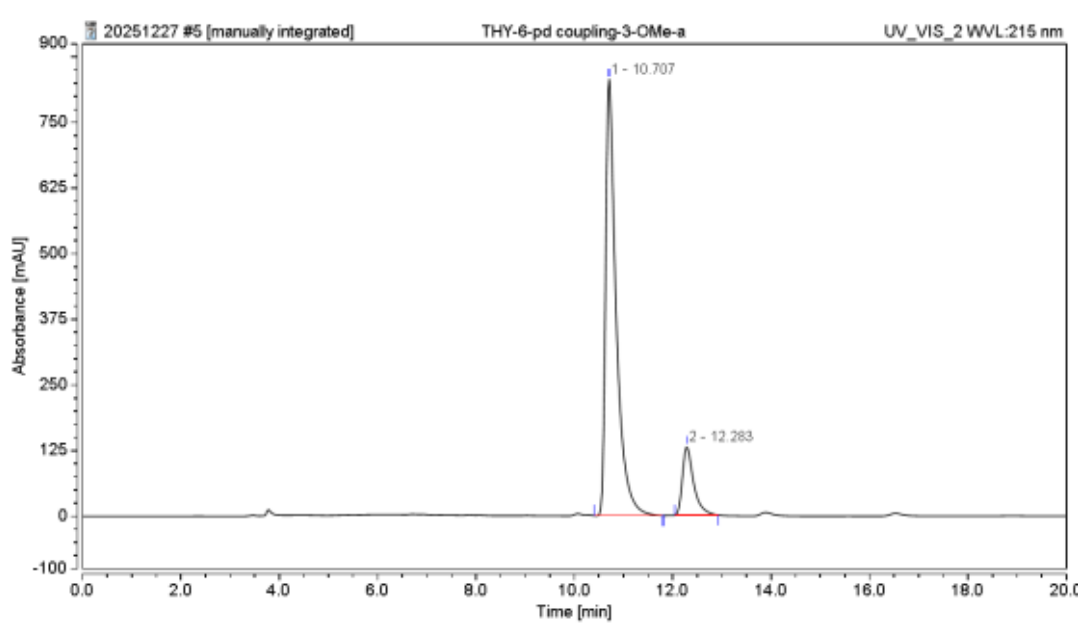

	Retention Time	Area	% Area
1	10.743	61.0341	50.30
2	12.303	60.3064	49.70

	Retention Time	Area	% Area
1	10.707	207.8277	85.77
2	12.283	34.4746	14.23

10. Synthesis and transformations of 1,2- and 1,3-diborylalkanes.

10.1. Synthesis of compound 19.

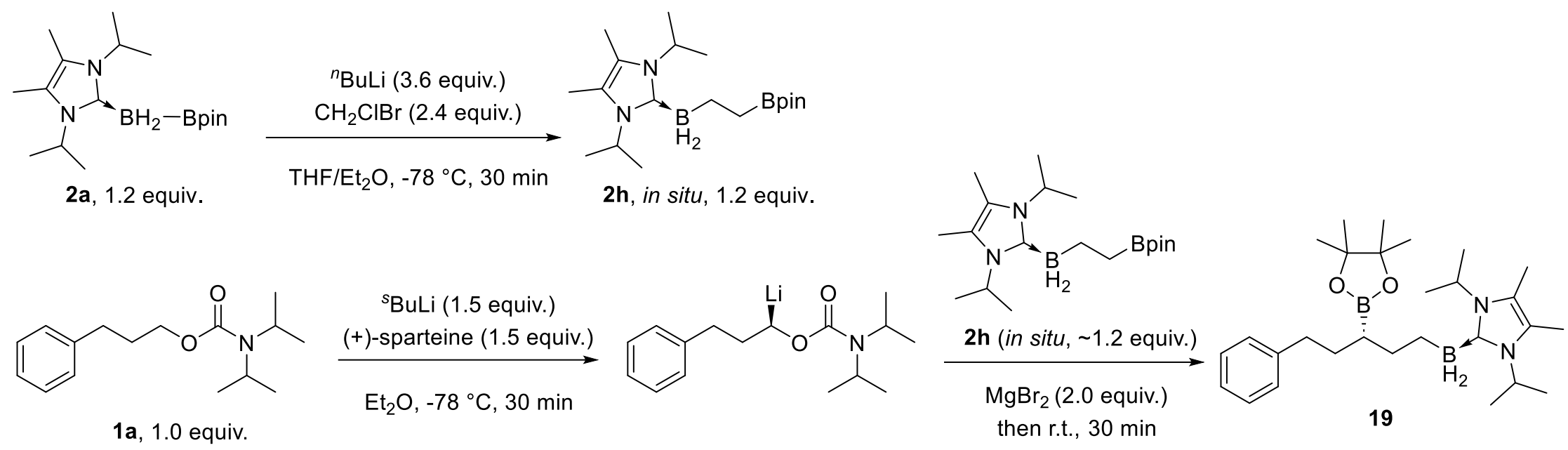

Under argon, to an oven-tried 4 mL reaction tube equipped with a magnetic stir bar and a septum was charged with **2a** (38.4 mg, 0.12 mmol, 1.2 equiv.) and chlorobromomethane (31.0 mg, 0.35 mmol, 2.4 equiv.) in THF (1 mL). The reaction was cooled to -78 °C and stirred for 10 min. ^{n}BuLi (1.6 M in *n*-hexane; 0.14 mL, 0.21 mmol, 3.6 equiv.) was then slowly added and the mixture was stirred vigorously (without splashing) at -78 °C for 30 min. The reaction mixture was then warmed to room temperature and stirred for another 30 min. Upon completion, the reaction mixture was filtered through a pad of silica gel, washed with ethyl acetate and concentrated to dryness in vacuo to give the crude **2h** that was used for next step without purification.

Under argon, to an oven dried 4 mL reaction tube equipped with a magnetic stir bar and a septum was charged with **1a** (26.4 mg, 0.1 mmol, 1.0 equiv.), (+)-sparteine (35.2 mg, 0.15 mmol, 1.5 equiv.) in Et_2O (500 µL). The reaction was cooled to -78 °C and stirred for 10 min. ^{s}BuLi (1.3 M in cyclohexane, 0.12 mL, 0.15 mmol, 1.5 equiv.) was then slowly added and the mixture was stirred vigorously (without splashing) at -78 °C for 30 min. After that, a solution of the **2h** (*in-situ*, 0.12 mmol, 1.2 equiv.) in Et_2O (1.2 mL) was added dropwise. The reaction mixture was stirred at this temperature for an additional 10 min. $MgBr_2$ (freshly made from Mg and 1,2-dibromoethane, 0.5 M in Et_2O, 0.4 mL, 0.2 mmol, 2.0 equiv.) was then added dropwise and the reaction mixture was stirred at -78 °C for 20 min. The reaction mixture was then warmed to room temperature and stirred for another 30 min. Upon completion, the reaction was quenched with aqueous NH_4Cl and extracted with Et_2O (3 × 3 mL). The combined organic phases were dried over $MgSO_4$, and the solvent was removed in vacuo. The resulting crude residue was purified by flash column chromatography to give the product **19** (30%, 14.0 mg).

*Note: Compound **2h** partially decomposes during silica gel column chromatography. Therefore, its use without further purification is recommended.*

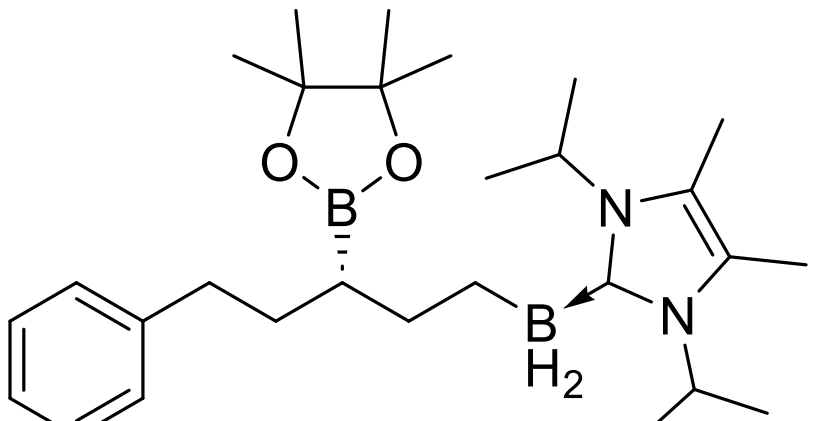

19

19: Yield: 14.0 mg, 30%, 99% ee, colorless oil, R_f = 0.4 (*n*-hexane/ethyl acetate = 4/1).
^{1}H NMR (500 MHz, $CDCl_3$) δ 7.26 – 7.03 (m, 5H), 5.57 – 5.38 (m, 2H), 2.57 (t, *J* = 8.3 Hz, 2H), 2.20 (s, 6H), 1.73 – 1.61 (m, 2H), 1.52 – 1.32 (m, 13H), 1.34 – 1.29 (m, 1H), 1.25 – 1.17 (m, 12H), 1.08 – 1.00 (m, 1H), 0.47 – 0.30 (m, 2H).
^{13}C NMR (126 MHz, $CDCl_3$) δ 144.0, 128.6, 128.5, 128.2, 125.4, 123.5, 82.6, 49.5, 36.2, 34.5, 34.1, 25.2, 25.0, 21.7, 21.7, 10.5.
^{11}B NMR (160 MHz, $CDCl_3$) δ 34.9 (s, 1B), -26.2 (t, *J* = 83.4 Hz, 1B).
Note: After column chromatography, preparative thin-layer chromatography (PTLC) was employed to remove trace amounts of impurities to ensure the highest purity for the NMR spectra.
HRMS (ESI): Calcd for $C_{28}H_{48}B_2N_2O_2Na^+$ $[M+Na]^+$: 489.3794, found: 489.3807.
IR (neat, cm^{-1}): *v* 2998, 2359, 1452, 1309, 1259, 1024.
Chiral HPLC (IC, ^{i}PrOH/*n*-hexane = 5/95, 1.0 mL/min) t_R 16.7 min (major), 20.3 min (minor).
$[\alpha]_D^{25}$ = -15.00 (*c* = 0.3, $CHCl_3$).

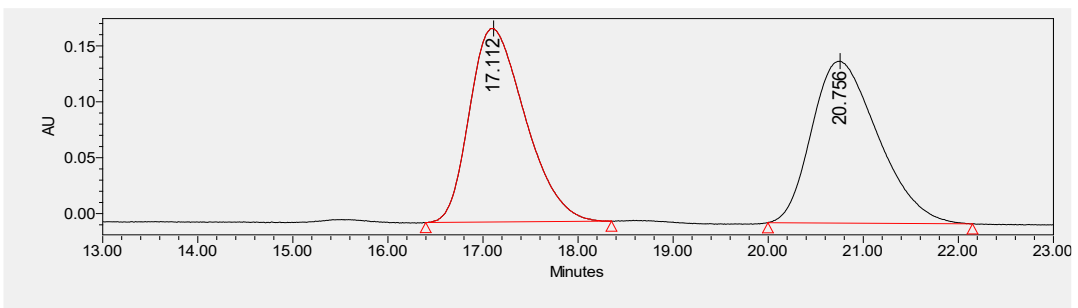

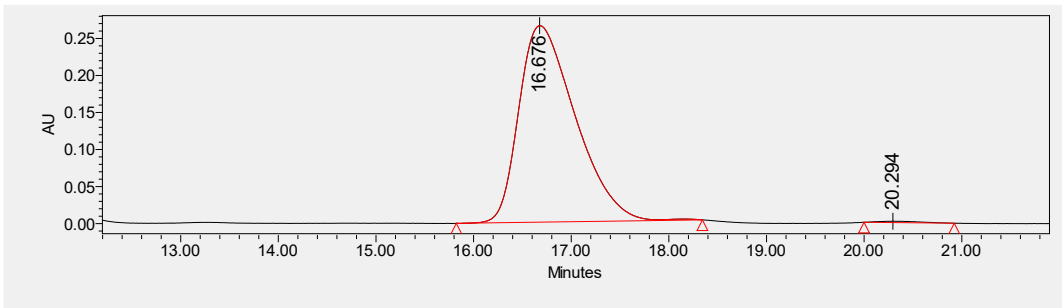

	Retention Time	Area	% Area
1	17.112	7034603	50.10
2	20.756	7005568	49.90

	Retention Time	Area	% Area
1	16.676	10660158	99.48
2	20.294	55374	0.52

10.2. Synthesis of compound 20.

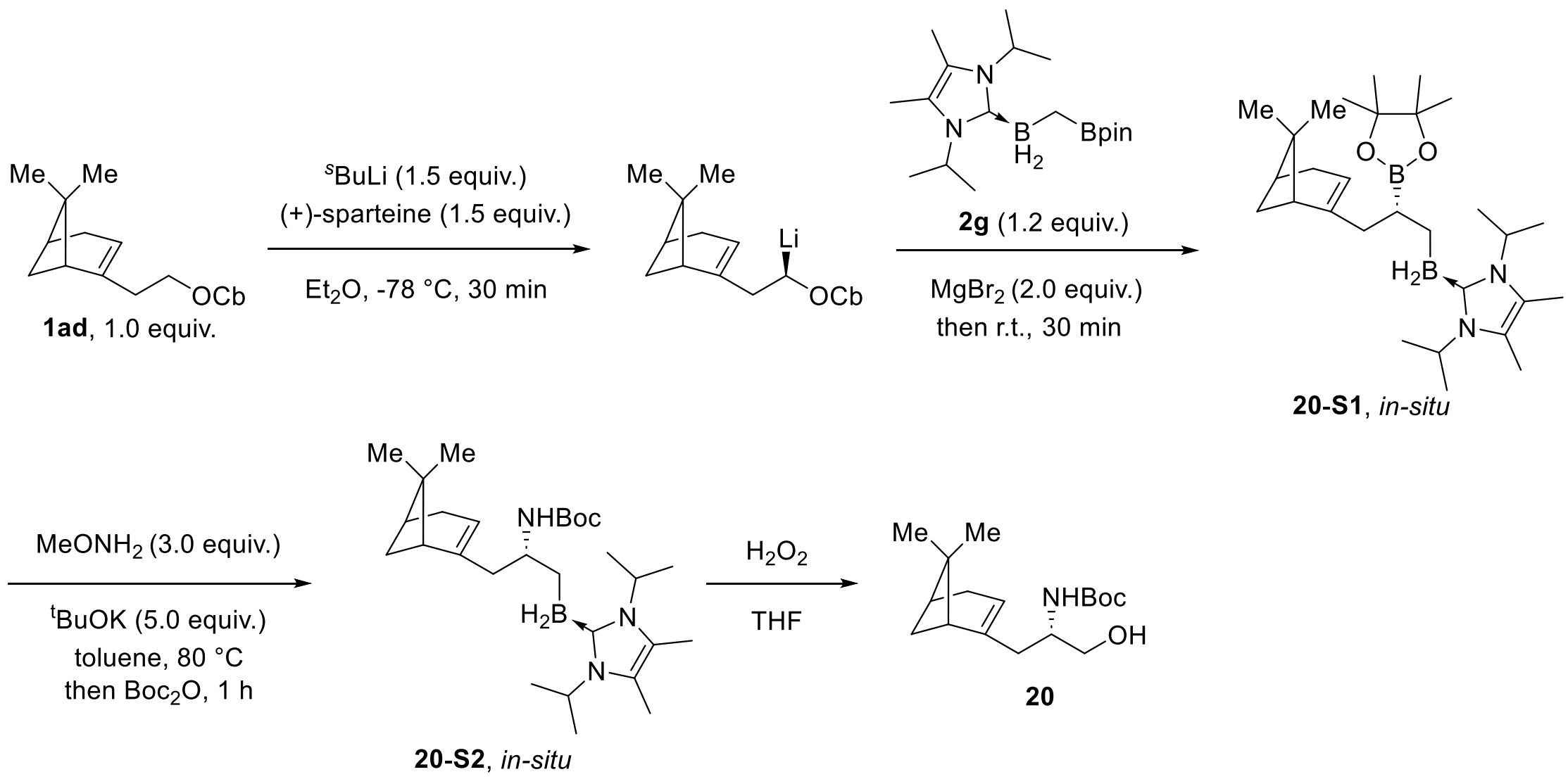

Under argon, to an oven dried 4 mL reaction tube equipped with a magnetic stir bar and a septum was charged with **1ad** (293 mg, 1.0 mmol, 1.0 equiv.), (+)-sparteine (352 mg, 0.15 mmol, 1.5 equiv.) and Et_2O (5 mL). The reaction was cooled to -78 °C and stirred for 10 min. ^{s}BuLi (1.3 M in cyclohexane, 1.2 mL, 1.5 mmol, 1.5 equiv.) was then slowly added and the mixture was stirred vigorously (without splashing) at -78 °C for 30 min. After that, a solution of the **2g** (384 mg, 1.2 mmol, 1.2 equiv.) in Et_2O (12 mL) was added dropwise. The reaction mixture was stirred at this temperature for an additional 10 min. $MgBr_2$ (freshly made from Mg and 1,2-dibromoethane, 0.5 M in Et_2O, 4 mL, 2.0 mmol, 2.0 equiv.) was then added dropwise and the reaction mixture was stirred at -78 °C for 20 min. The reaction mixture was then warmed to room temperature and stirred for another 30 min. Upon completion, the reaction was quenched with aqueous NH_4Cl and extracted with Et_2O (3 × 30 mL). The combined organic phases were dried over $MgSO_4$, and the solvent was removed in vacuo to give the crude **20-S1** that was used for next step without purification.
Under argon, to an oven dried 4 mL reaction tube equipped with magnetic stir bar was added **20-S1** (*in-situ*, 1.0 mmol, 1.0 equiv.), $MeONH_2$ (1.5 M in THF, 2 mL, 3.0 mmol, 3.0 equiv.), ^{t}BuOK (561 mg, 5.0 mmol, 5.0 equiv.) and toluene (5 mL). The reaction mixture was then heated at 80 °C and stirred for 12 h. Upon completion, Boc_2O (1 M in THF, 3 mL, 3.0 mmol, 1.5 equiv.) was added and the reaction mixture was stirred at room temperature for another 1 h. Upon completion, the reaction was quenched with aqueous NH_4Cl and extracted with ethyl acetate (3 × 10 mL). The combined organic phases were dried over $MgSO_4$, and the solvent was removed in vacuo. The resulting crude residue **20-S2** was used for the next step without purification.
Under air, to an oven dried 20 mL vial equipped with magnetic stir bar was added crude reaction mixture **20-S2** and THF (10 mL). A solution of 30 wt% H_2O_2/ ~15 wt% NaOH (1:2, 15 mL in total) was slowly added at 0 °C and the reaction mixture was then warmed to room temperature and stirred for another 6 h. Upon completion, the reaction was quenched with H_2O and extracted with ethyl acetate (3 × 10 mL). The combined organic phases were dried over $MgSO_4$, and the solvent was removed in vacuo. The resulting residue was purified by flash column chromatography to give product **20** (51%, 150.5 mg).

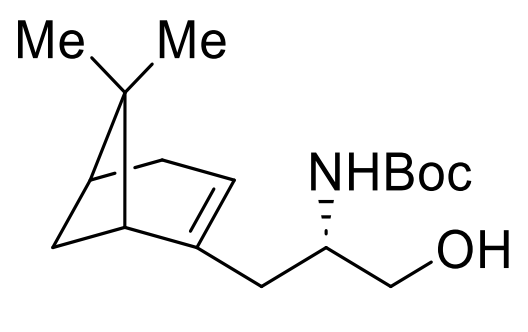

20

20: Three-step yield: 150.5 mg, 51%, >20: 1 d.r., colorless oil, R_f = 0.4 (*n*-hexane/ethyl acetate = 1/1).
^{1}H NMR (400 MHz, $CDCl_3$) δ 5.52 – 5.19 (m, 1H), 4.65 (s, 1H), 3.90 – 3.29 (m, 3H), 2.94 (s, 1H), 2.44 – 2.33 (m, 1H), 2.32 – 2.14 (m, 3H), 2.14 – 1.98 (m, 3H), 1.42 (s, 9H), 1.26 (s, 3H), 0.94 – 0.65 (m, 4H).
^{13}C NMR (101 MHz, $CDCl_3$) δ 156.9, 144.7, 120.1, 79.9, 66.7, 50.9, 45.5, 40.7, 38.8, 38.0, 31.9, 31.5, 28.5, 26.3, 21.2.
HRMS (ESI): Calcd for $C_{17}H_{29}NO_3Na^+$ $[M+Na]^+$: 318.2045, found: 318.2040.

IR (neat, cm^{-1}): v 3348, 2978, 2921, 1688, 1507, 1366, 1248, 1160, 1048.
$[\alpha]_D^{25}$= -13.10 (c = 1.0, $CHCl_3$)

10.3. Synthesis of compounds 21 and 24.

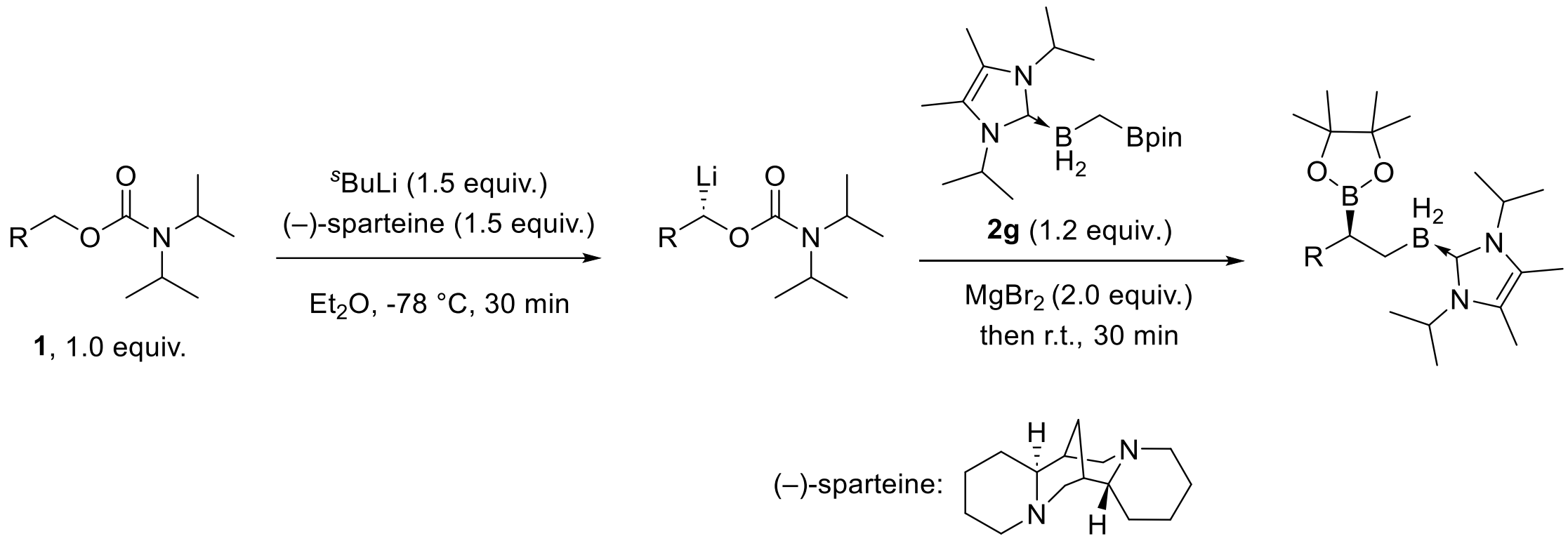

Under argon, to an oven dried 4 mL reaction tube equipped with a magnetic stir bar and a septum was charged with carbamate **1** (0.1 mmol, 1.0 equiv.), (–)-sparteine (35.2 mg, 0.15 mmol, 1.5 equiv.) and Et_2O (500 μL). The reaction was cooled to -78 °C and stirred for 10 min. ^{s}BuLi (1.3 M in cyclohexane, 0.12 mL, 0.15 mmol, 1.5 equiv.) was then slowly added and the mixture was stirred vigorously (without splashing) at -78 °C for 30 min. After that, a solution of **2g** (40.1 mg, 0.12 mmol, 1.2 equiv.) in Et_2O (1.2 mL) was added dropwise. The reaction mixture was stirred at this temperature for an additional 10 min. $MgBr_2$ (freshly made from Mg and 1,2-dibromoethane, 0.5 M in Et_2O, 0.4 mL, 0.2 mmol, 2.0 equiv.) was then added dropwise and the reaction mixture was kept stirring at -78 °C for 20 min. After that, the reaction mixture warmed to room temperature and stirred for another 30 min. Upon completion, the reaction was quenched with aqueous NH_4Cl and extracted with ethyl acetate (3 × 3 mL). The combined organic phase was dried over $MgSO_4$, and the solvent was removed in vacuo. The resulting residue was purified by flash column chromatography or preparative thin layer chromatography to give the products. The racemic product was prepared using the same procedure, with *N,N,N',N'*-tetramethylethylenediamine (TMEDA) substituted for (–)-sparteine.
Note: Flash column chromatography is suggested to be finished within 30 min.

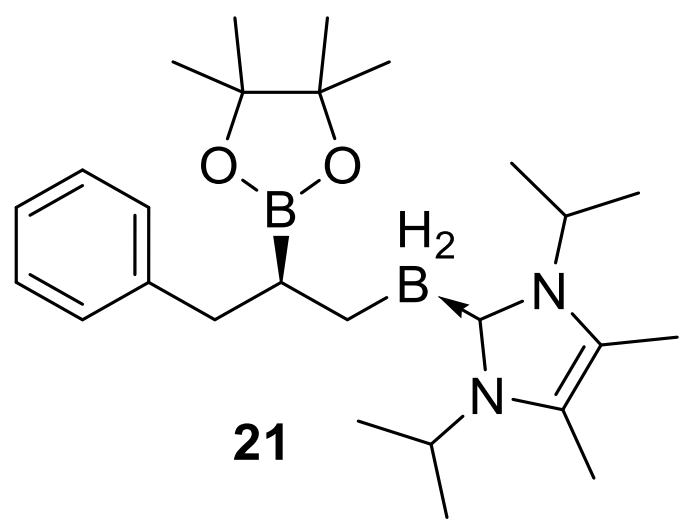

21

21: Yield: 29.3 mg, 67%, ~96% ee (deduced based on the ee of compound **22**), colorless oil, R_f = 0.4 (n-hexane/ethyl acetate = 3/1).

^{1}H NMR (400 MHz, $CDCl_3$) δ 7.25 – 7.14 (m, 4H), 7.12 – 7.03 (m, 1H), 5.55 – 5.24 (m, 2H), 2.88 – 2.57 (m, 2H), 2.18 (s, 6H), 1.57 – 1.30 (m, 13H), 1.31 – 0.98 (m, 12H), 0.63 – 0.12 (m, 2H).
^{13}C NMR (101 MHz, $CDCl_3$) δ 144.4, 129.2, 127.7, 124.9, 123.4, 82.2, 49.4, 40.7, 25.1, 25.0, 21.7, 21.7, 10.5.
^{11}B NMR (128 MHz, $CDCl_3$) δ 34.8 (s, 1B), -26.7 (t, J = 83.5 Hz, 1B).
Note: After column chromatography, preparative thin-layer chromatography (PTLC) was employed to remove trace amounts of impurities to ensure the highest purity for the NMR spectra.
HRMS (ESI): Calcd for $C_{26}H_{44}B_2N_2O_2Na^+$ $[M+Na]^+$: 461.3481, found: 461.3479.
IR (neat, cm^{-1}): v 2974, 2932, 2276, 1370, 1309, 1221, 1144, 1110.
$[\alpha]_D^{25}$= -10.33 (c = 0.6, $CHCl_3$).

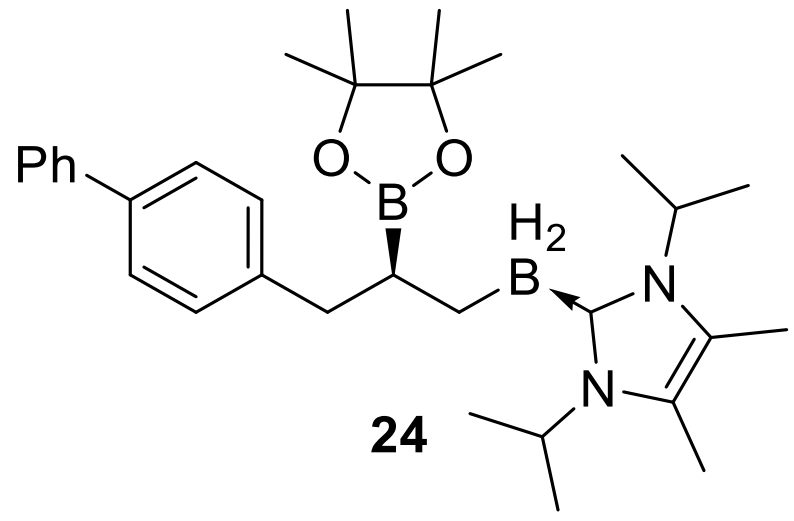

24

24: Yield: 26.2 mg, 51%, 96% ee (deduced based on the ee of compound **25**), white solid, m.p. = 178 – 182 °C, R_f = 0.4 (n-hexane/ethyl acetate = 3/1).
^{1}H NMR (400 MHz, $CDCl_3$) δ 7.91 – 7.28 (m, 9H), 5.74 – 5.35 (m, 2H), 2.94 – 2.57 (m, 2H), 2.18 (s, 6H), 1.52 – 1.28 (m, 13H), 1.28 – 0.73 (m, 12H), 0.64 – 0.27 (m, 2H).
^{13}C NMR (101 MHz, $CDCl_3$) δ 143.7, 141.9, 137.8, 129.6, 128.7, 127.1, 126.7, 126.5, 123.4, 82.3, 49.5, 40.2, 25.1, 25.1, 21.8, 21.7, 10.5.
^{11}B NMR (128 MHz, $CDCl_3$) δ 34.2 (s, 1B), -26.9 (t, J = 84.8 Hz, 1B).
HRMS (ESI): Calcd for $C_{32}H_{48}B_2N_2O_2Na^+$ $[M+Na]^+$: 537.3794, found: 537.3805.
IR (neat, cm^{-1}): v 2974, 2933, 2271, 1370, 1221, 1143, 1110.
$[\alpha]_D^{25}$= 4.00 (c = 0.7, $CHCl_3$).

10.4. Transformations of compounds 21 and 24.

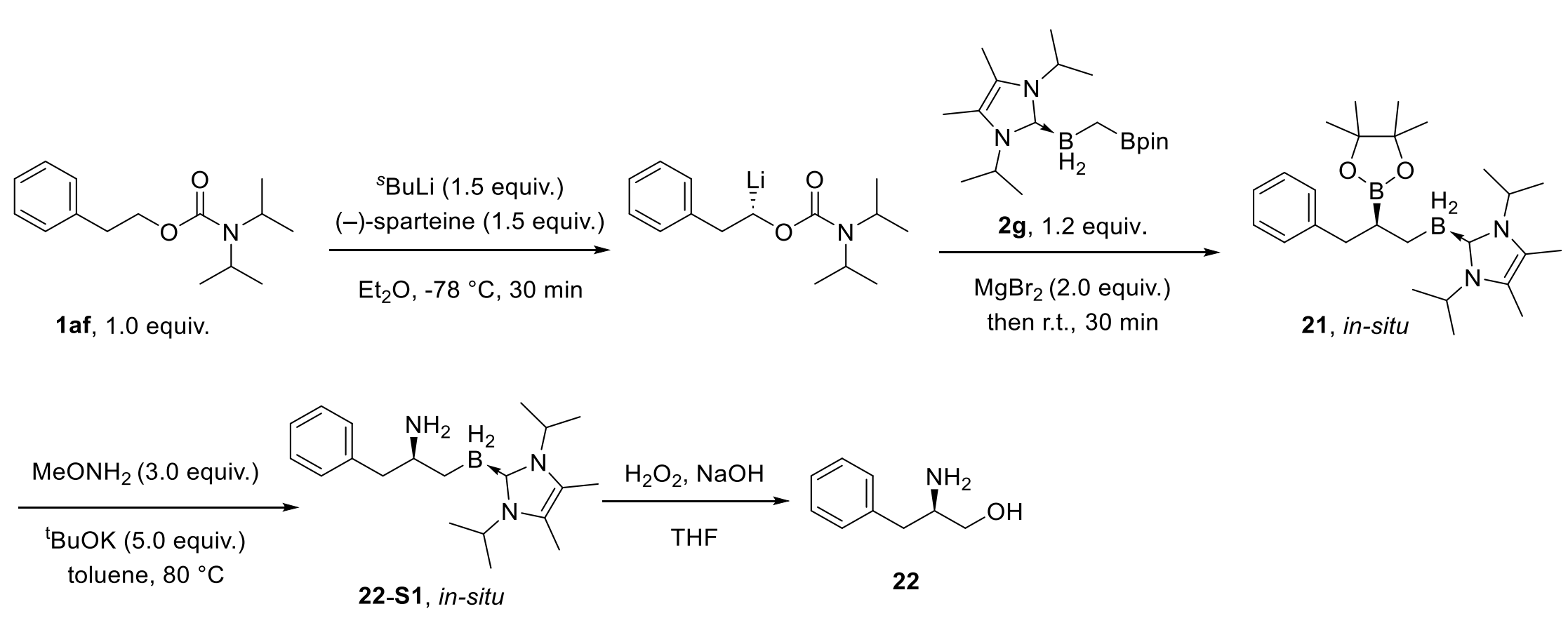

Under argon, to an oven dried 4 mL reaction tube equipped with a magnetic stir bar and a septum was charged with **1af** (24.9 mg, 0.10 mmol, 1.0 equiv.), (–)-sparteine (35.2

mg, 0.15 mmol, 1.5 equiv.) and Et_2O (500 μL). The reaction was cooled to -78 °C and stirred for 10 min. ^{s}BuLi (1.3 M in cyclohexane, 0.12 mL, 0.15 mmol, 1.5 equiv.) was then slowly added and the mixture was stirred vigorously (without splashing) at -78 °C for 30 min. After that, a solution of **2g** (40.1 mg, 0.12 mmol, 1.2 equiv.) in Et_2O (1.2 mL) was added dropwise. The reaction mixture was stirred at this temperature for an additional 10 min. $MgBr_2$ (freshly made from Mg and 1,2-dibromoethane, 0.5 M in Et_2O, 0.4 mL, 0.2 mmol, 2.0 equiv.) was then added dropwise and the reaction mixture was stirred at -78 °C for 20 min. The reaction mixture was then warmed to room temperature and stirred for another 30 min. Upon completion, the reaction was quenched with aqueous NH_4Cl and extracted with Et_2O (3 × 3 mL). The combined organic phases were dried over $MgSO_4$, and the solvent was removed in vacuo to give the crude **21** that was used for next step without purification.
Under argon, to an oven dried 4 mL reaction tube equipped with magnetic stir bar was added crude **21** (*in-situ*, 0.1 mmol, 1.0 equiv.), $MeONH_2$ (1.5 M in THF, 200 μL, 0.3 mmol, 3.0 equiv.), ^{t}BuOK (56.1 mg, 0.5 mmol, 5.0 equiv.) and toluene (500 μL). The reaction mixture was then heated at 80 °C and stirred for 12 h. Upon completion, the reaction was quenched with aqueous NH_4Cl and extracted with ethyl acetate (3 × 3 mL). The combined organic phases were dried over $MgSO_4$, and the solvent was removed in vacuo. The resulting crude residue **22-S1** was used for the next step without purification.
Under air, to an oven dried 20 mL vial equipped with magnetic stir bar was added crude **22-S1** and THF (1 mL). A solution of 30 wt% H_2O_2/ ~15 wt% NaOH (1:2, 1.5 mL in total) was slowly added at 0 °C and the reaction mixture was then warmed to room temperature and stirred for another 6 h. Upon completion, the reaction was quenched with H_2O and extracted with ethyl acetate (3 × 3 mL). The combined organic phases were dried over $MgSO_4$, and the solvent was removed in vacuo. The resulting residue was purified by flash column chromatography to give product **22** (66%, 10.0 mg).

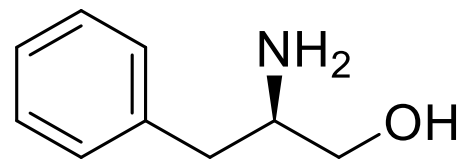

22

22: Three-step yield: 10.0 mg, 66%, 96% ee, white solid, R_f = 0.4 (MeOH/DCM = 1/10).
^{1}H NMR (400 MHz, $CDCl_3$) δ 7.35 – 7.27 (m, 2H), 7.25 – 7.16 (m, 3H), 3.82 – 3.48 (m, 1H), 3.47 – 3.30 (m, 1H), 3.17 – 2.99 (m, 1H), 2.83 – 2.69 (m, 1H), 2.65 – 2.35 (m, 1H), 2.17 – 1.74 (m, 3H).
^{13}C NMR (101 MHz, $CDCl_3$) δ 138.8, 129.3, 128.7, 126.5, 66.5, 54.3, 41.0.
HRMS (ESI): Calcd for $C_9H_{13}NONa^+$ $[M+Na]^+$: 174.0895, found: 174.0889.
Chiral HPLC (OD-H, ^{i}PrOH/*n*-hexane = 3/97, 0.8 mL/min) t_R 36.0 min (major), 43.2 min (minor).
$[\alpha]_D^{25}$= 24.90 (*c* = 1.0, $CHCl_3$), Ref: $[\alpha]_D^{25}$= 25.90 (*c* = 1.1, $CHCl_3$).[23]
The NMR data matched with those reported in literature.[23]

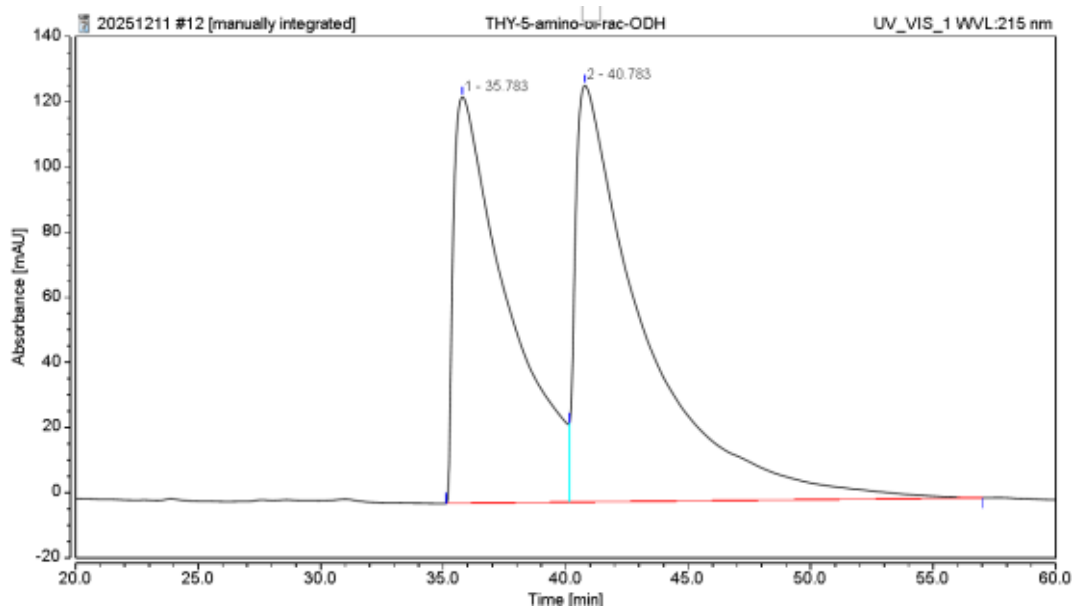

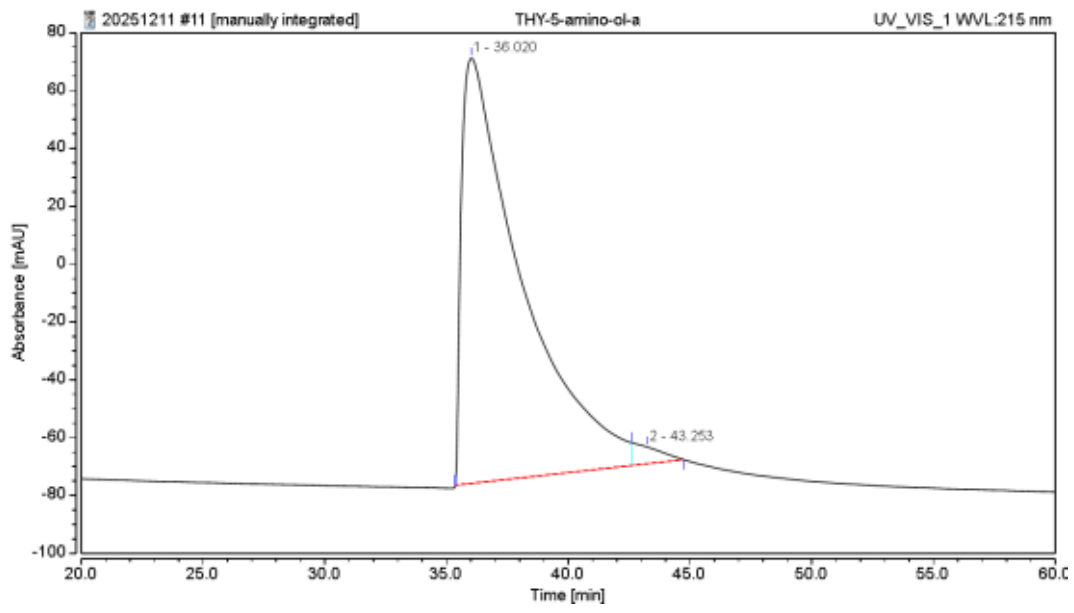

	Retention Time	Area	% Area
1	35.783	332.479	45.28
2	40.783	401.7863	54.72

	Retention Time	Area	% Area
1	36.020	418.6587	97.99
2	43.253	8.597	2.01

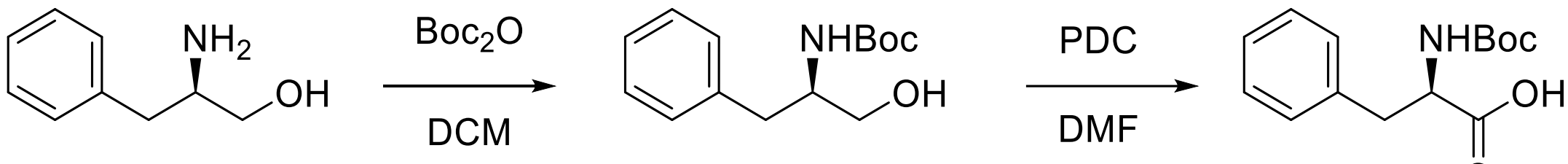

22, 96% ee **23-S1** **23**

Under air, to an oven dried 20 mL vial equipped with a magnetic stir bar and the mixture of **22** (166 mg, 0.2 mmol, 1.0 equiv.) and Boc_2O (43.7 mg, 0.2 mmol, 1.0 equiv.) in DCM (5 mL). The reaction mixture was stirred at room temperature for 12 h. Upon completion, the reaction was quenched with aqueous NH_4Cl and extracted with Et_2O (3 × 3 mL). The combined organic phases were dried over $MgSO_4$, and the solvent was removed in vacuo to give the crude **23-S1** that was used for next step without purification.

According to a reported literature,[24] under air, to an oven dried 20 mL vial equipped with a magnetic stir bar and the mixture of **23-S1** (*in-situ*, 0.2 mmol, 1.0 equiv.) from the last step and DMF (1 mL). Pyridinium dichromate (PDC; 374 mg, 1.0 mmol, 5.0 equiv.) was added and the reaction mixture was stirred at room temperature for 12 h. Upon completion, H_2O (10 mL) and Et_2O (10 mL) were added and the aqueous phase was extracted with Et_2O (3 × 7 mL). The combined organic phases were dried over $MgSO_4$ and the solvent was removed in vacuo. The crude product was dissolved in ethyl acetate and extracted with saturated $NaHCO_3$ solution. The aqueous layer was washed with ethyl acetate, acidified with 2N HCl, and extracted with ethyl acetate. The combined organic phases were dried over $MgSO_4$ and the solvent was removed in vacuo. The resulting residue was purified through recrystallization from *n*-hexane/ethyl acetate to afford the desired product **23** (98%, 52.0 mg).

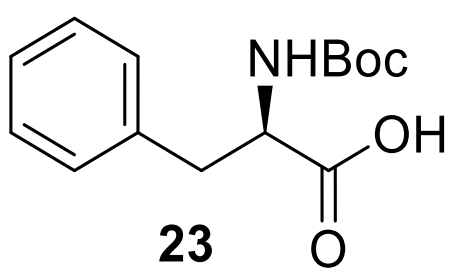

23

(a mixture of carbamate rotamers)

23: Yield: 52.0 mg, 98%, 96% ee, white solid, R_f = 0.4 (MeOH/DCM = 1/10).

^{1}H NMR (400 MHz, $CDCl_3$) δ 10.68 – 9.96 (m, 1H), 7.35 – 7.26 (m, 3H), 7.24 – 7.15 (m, 2H), 6.75 – 6.32 (m, 0.4H), 5.35 – 4.84 (m, 0.6H), 4.79 – 4.49 (m, 0.6H), 4.49 – 4.16 (m, 0.4H), 3.43 – 2.75 (m, 2H), 1.42 (s, 5.5H), 1.28 (s, 3.5H).

^{13}C NMR (101 MHz, $CDCl_3$) δ 176.7, 176.1, 156.7, 155.5, 136.7, 136.0, 129.7, 129.5, 128.7, 127.2, 127.0, 81.7, 80.4, 56.0, 54.4, 39.4, 37.9, 28.4, 28.1.
Chiral HPLC (AD-H, ^{i}PrOH/*n*-hexane = 15/85, 1.0 mL/min) t_R 8.6 min (major), 21.0 min (minor).
$[\alpha]_D^{25}$= 22.15 (*c* = 1.0, $CHCl_3$), Ref: $[\alpha]_D^{26}$= 22.60 (*c* = 1.0, $CHCl_3$).[25]
The NMR data[26] and HPLC condition[27] matched with those reported in literature.

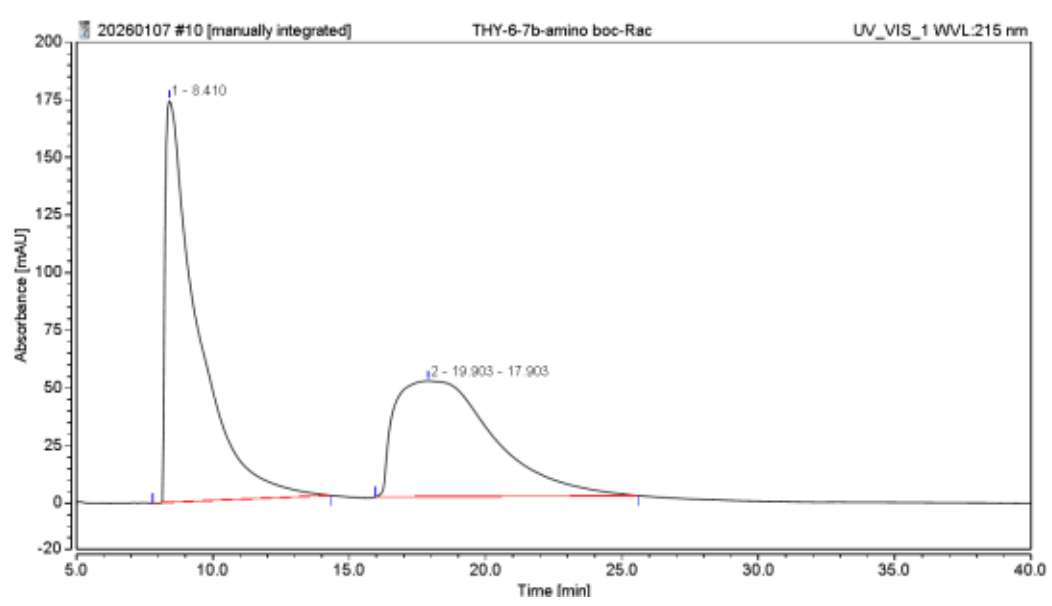

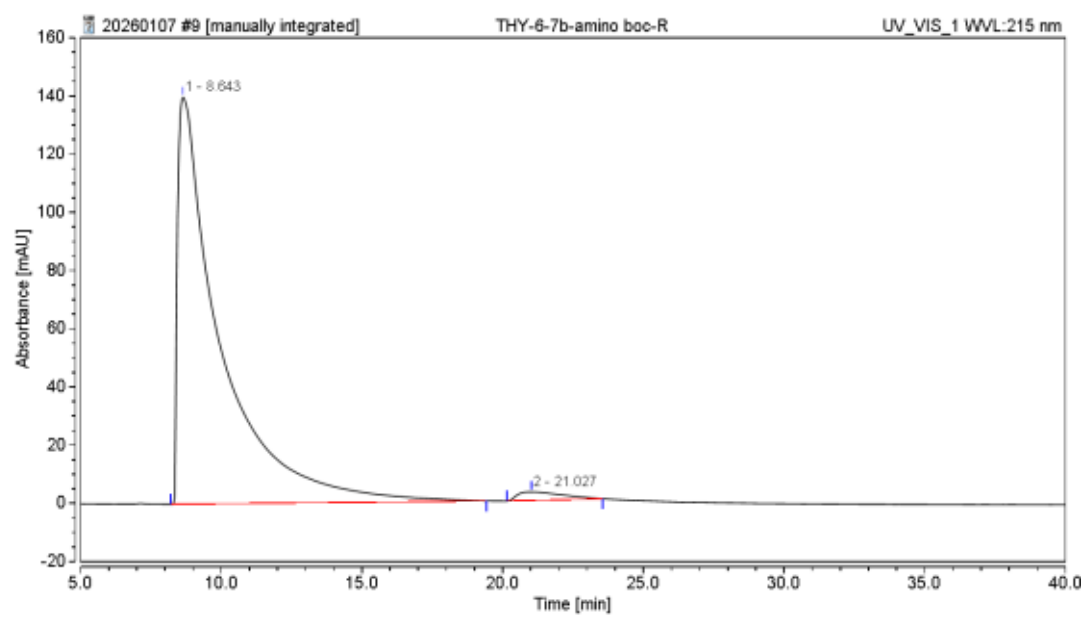

	Retention Time	Area	% Area
1	8.41	214.4752	50.47
2	17.903	210.4953	49.53

	Retention Time	Area	% Area
1	8.643	245.0586	97.85
2	21.027	5.3783	2.15

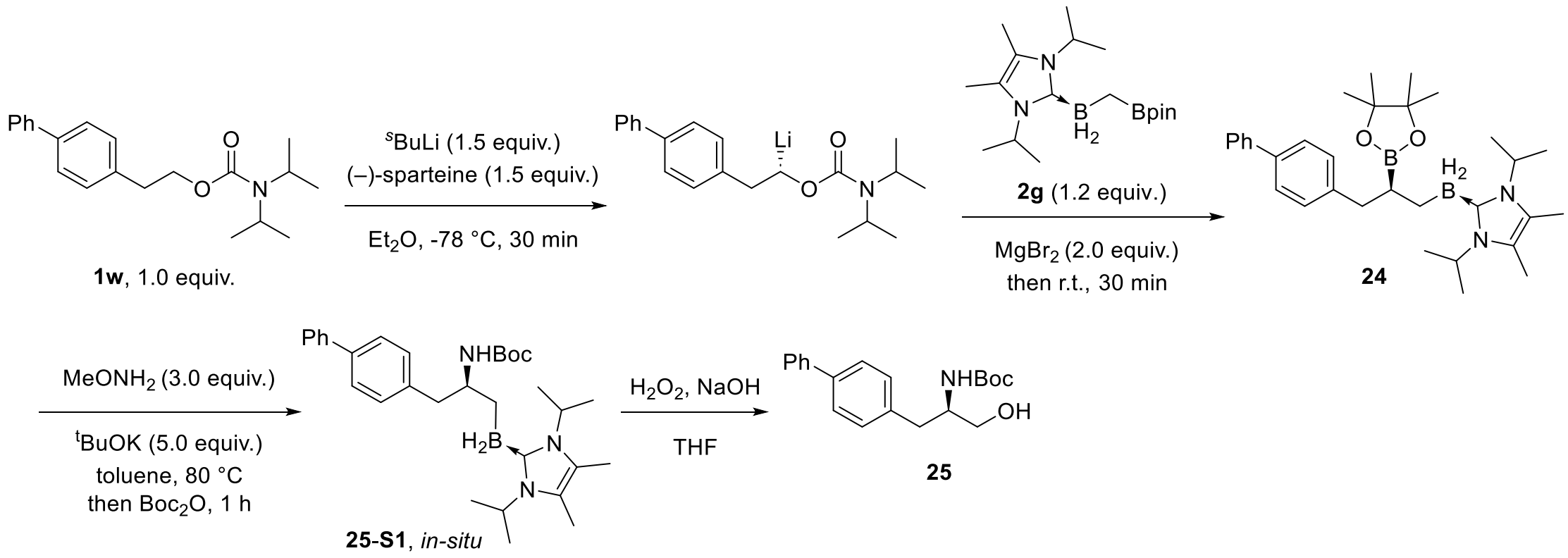

Under argon, to an oven dried 50 mL reaction tube equipped with a magnetic stir bar and a septum was charged with **1w** (325 mg, 1.0 mmol, 1.0 equiv.), (–)-sparteine (352 mg, 0.15 mmol, 1.5 equiv.) and Et_2O (5 mL). The reaction was cooled to -78 °C and stirred for 10 min. ^{s}BuLi (1.3 M in cyclohexane, 1.2 mL, 1.5 mmol, 1.5 equiv.) was then slowly added and the mixture was stirred vigorously (without splashing) at -78 °C for 30 min. After that, a solution of **2g** (400 mg, 1.2 mmol, 1.2 equiv.) in Et_2O (12 mL) was added dropwise. The reaction mixture was stirred at this temperature for an additional 10 min. $MgBr_2$ (freshly made from Mg and 1,2-dibromoethane, 0.5 M in Et_2O, 4 mL, 2.0 mmol, 2.0 equiv.) was then added dropwise and the reaction mixture was stirred at -78 °C for 20 min. The reaction mixture was then warmed to room temperature and stirred for another 30 min. Upon completion, the reaction was quenched with aqueous NH_4Cl and extracted with Et_2O (3 × 30 mL). The combined organic phases were dried over $MgSO_4$, and the solvent was removed in vacuo to give the crude **24** that was used for next step without purification.
Under argon, to an oven dried 20 mL reaction tube equipped with magnetic stir bar was added crude **24** (*in-situ*, 1.0 mmol, 1.0 equiv.), $MeONH_2$ (1.5 M in THF, 2 mL, 3.0

mmol, 3.0 equiv.), ^{t}BuOK (561 mg, 5.0 mmol, 5.0 equiv.) and toluene (5 mL). The reaction mixture was then heated at 80 °C and stirred for 12 h. Upon completion, Boc_2O (3.0 mL, 1 M in THF, 3.0 mmol, 1.5 equiv.) was added and the reaction mixture was stirred at room temperature for another 1 h. Upon completion, the reaction was quenched with aqueous NH_4Cl and extracted with ethyl acetate (3 × 10 mL). The combined organic phases were dried over $MgSO_4$, and the solvent was removed in vacuo. The resulting crude residue **24-S1** was used for the next step without purification. Under air, to a 20 mL vial equipped with magnetic stir bar was added crude **24-S1** and THF (10 mL). A solution of 30 wt% H_2O_2/ ~15 wt% NaOH (1:2, 15 mL in total) was slowly added at 0 °C and the reaction mixture was then warmed to room temperature and stirred for another 6 h. Upon completion, the reaction was quenched with H_2O and extracted with ethyl acetate (3 × 10 mL). The combined organic phases were dried over $MgSO_4$, and the solvent was removed in vacuo. The resulting residue was purified by flash column chromatography to give product **25** (88%, 300.8 mg).

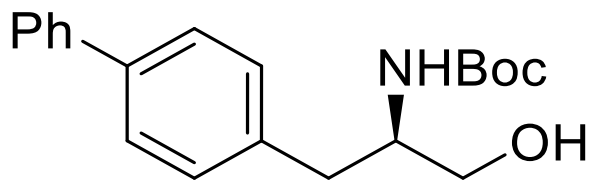

25

25: Three-step yield: 300.8 mg, 92%, 96% ee, white solid, R_f = 0.4 (*n*-hexane/ethyl acetate = 1/1).

^{1}H NMR (400 MHz, $CDCl_3$) δ 7.65 – 7.50 (m, 4H), 7.49 – 7.41 (m, 2H), 7.39 – 7.27 (m, 3H), 4.81 (d, *J* = 8.1 Hz, 1H), 3.91 (s, 1H), 3.78 – 3.50 (m, 2H), 2.89 (d, *J* = 7.2 Hz, 2H), 2.47 (s, 1H), 1.42 (s, 9H).

^{13}C NMR (101 MHz, $CDCl_3$) δ 156.3, 140.9, 139.5, 137.1, 129.9, 128.8, 127.3, 127.3, 127.1, 79.8, 64.2, 53.7, 37.1, 28.5.

HRMS (ESI): Calcd for $C_{20}H_{25}NO_3Na^+$ $[M+Na]^+$: 350.1732, found: 350.1722.

Chiral HPLC (AD-H, ^{i}PrOH/*n*-hexane = 3/97, 1.0 mL/min) t_R 61.2 min (minor), 63.3 min (major).

$[\alpha]_D^{25}$= 7.38 (*c* = 0.3, $CHCl_3$).

The NMR data and HPLC condition matched with those reported in literature.

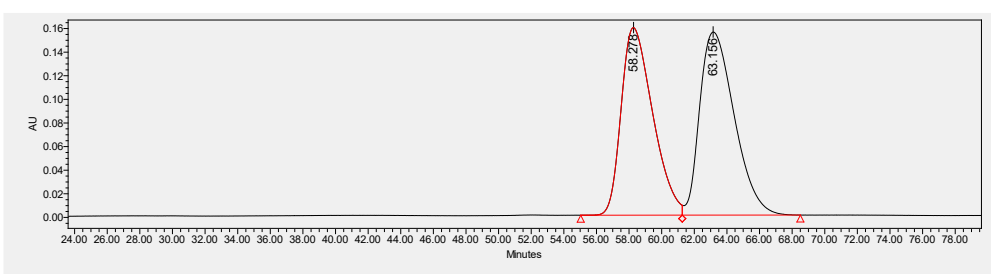

	Retention Time	Area	% Area
1	58.278	21789552	49.32
2	63.156	22393797	50.68

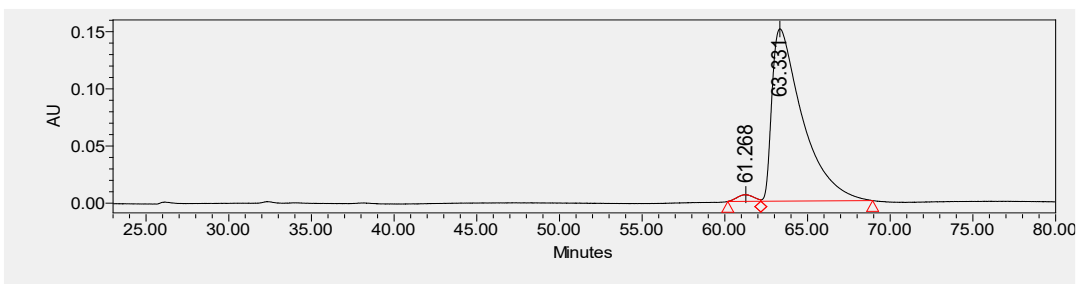

	Retention Time	Area	% Area
1	61.268	407941	2.06
2	63.331	19384025	97.94

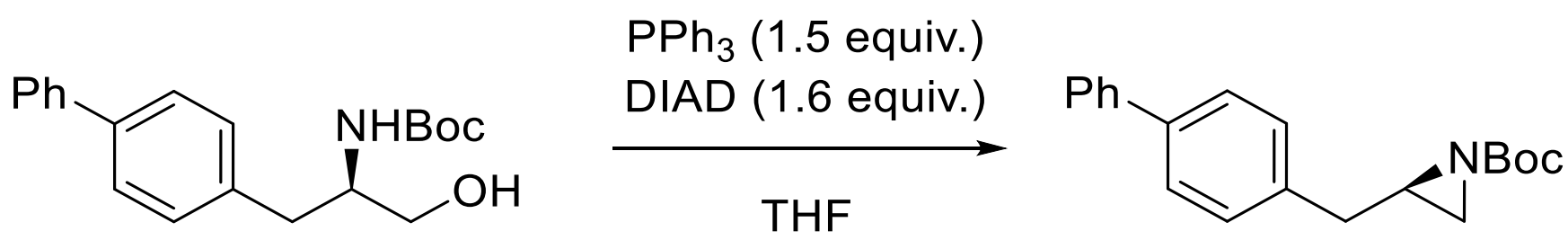

25, 96% ee **26**

Under N_2, to an oven dried 4 mL reaction tube equipped with magnetic stir bar was added **25** (32.7 mg, 0.1 mmol, 1.0 equiv.), triphenylphosphine (39.3 mg, 0.15 mmol, 1.5 equiv.) and anhydrous THF (1 mL). The reaction mixture was cooled to 0 °C and diethyl azodicarboxylate (DIAD; 32.3 mg, 0.16 mmol, 1.6 equiv.) was added dropwise. The reaction was then warmed to room temperature and stirred until completion (monitored by TLC). Upon completion, the solvent was removed in vacuo. The resulting residue was purified by flash column chromatography to give product **26** (95%, 29.4 mg).

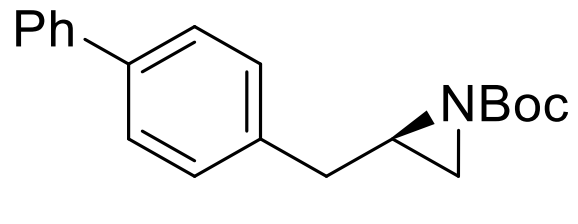

26

26: Yield: 29.4 mg, 95%, 96% ee, white solid, R_f = 0.4 (*n*-hexane/ethyl acetate = 2/1).
^{1}H NMR (400 MHz, $CDCl_3$) δ 7.68 – 7.52 (m, 4H), 7.49 – 7.32 (m, 5H), 3.17 – 2.94 (m, 1H), 2.86 – 2.56 (m, 2H), 2.35 (d, *J* = 5.9 Hz, 1H), 2.07 (d, *J* = 3.6 Hz, 1H), 1.46 (s, 9H).
^{13}C NMR (101 MHz, $CDCl_3$) δ 162.5, 141.1, 139.6, 137.2, 129.3, 128.9, 127.3, 127.3, 127.1, 81.3, 38.4, 38.2, 31.5, 28.0.
Chiral HPLC (AD-H, ^{i}PrOH/*n*-hexane = 1/99, 1.0 mL/min) t_R 13.6 min (major), 16.4 min (minor).
$[\alpha]_D^{25}$ = -47.38 (*c* = 0.8, $CHCl_3$).
The NMR data matched with those reported in literature.[28]

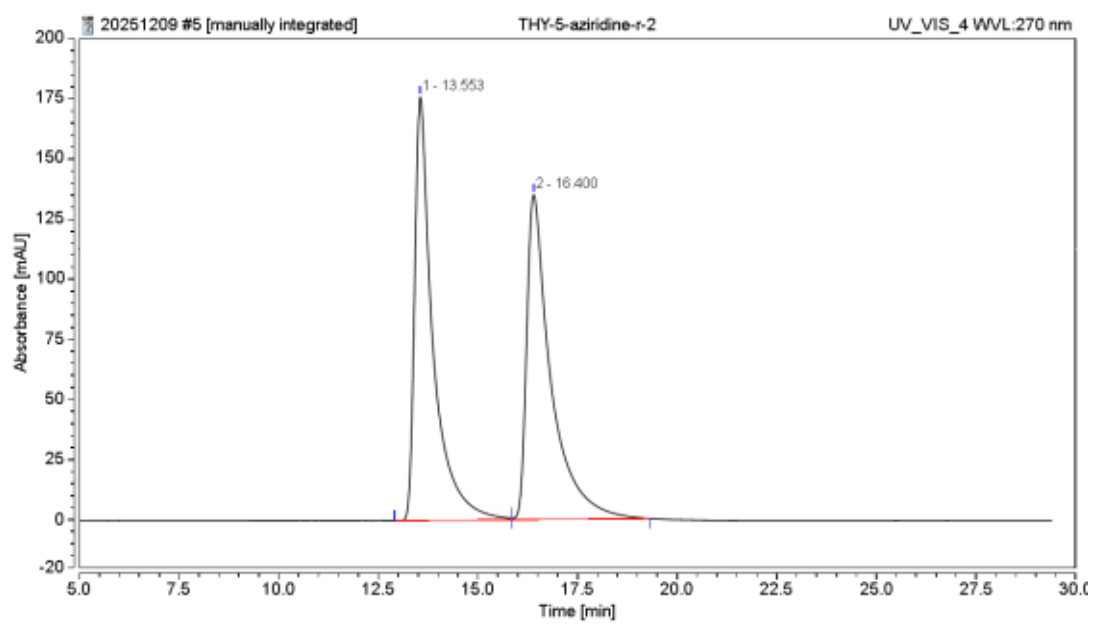

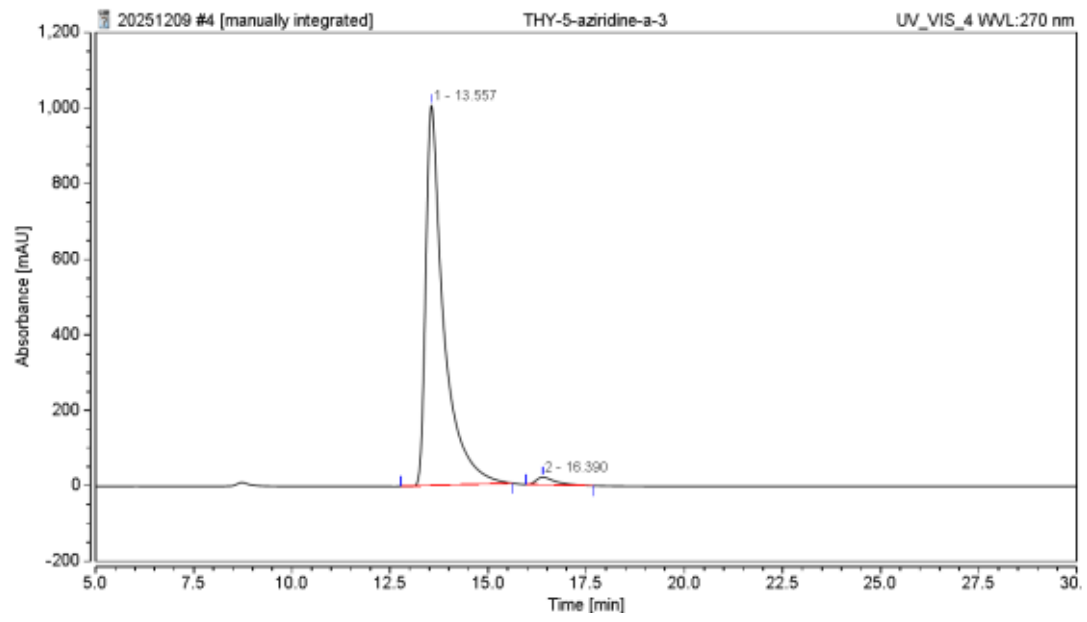

	Retention Time	Area	% Area
1	13.553	93.8466	49.74
2	16.400	94.8298	50.26

	Retention Time	Area	% Area
1	13.557	533.1944	97.90
2	16.390	11.459	2.10

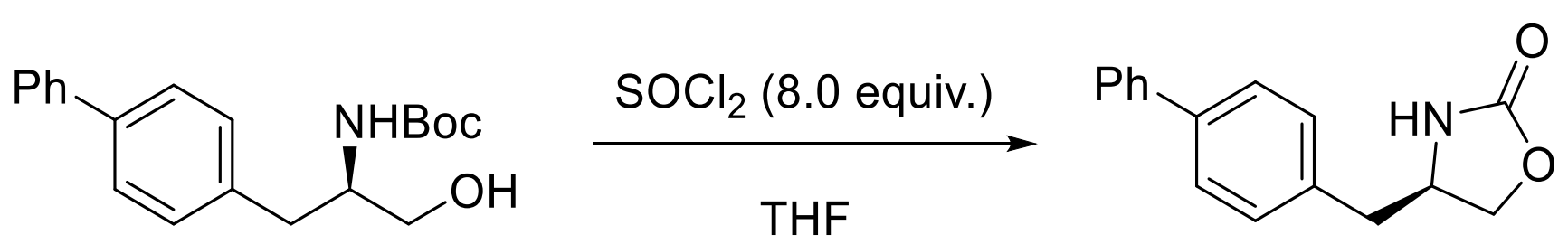

25, 96% ee **27**

Under N_2, to an oven dried 4 mL reaction tube equipped with magnetic stir bar was added **25** (32.7 mg, 0.1 mmol, 1.0 equiv.) and anhydrous THF (1 mL). The reaction mixture was cooled to 0 °C and $SOCl_2$ (190 mg, 0.8 mmol, 8.0 equiv.) was added dropwise. The reaction was then warmed to room temperature and stirred until

completion (monitored by TLC). Upon completion, the solvent was removed in vacuo. The resulting residue was purified by flash column chromatography to give product **27** (91%, 23.0 mg).

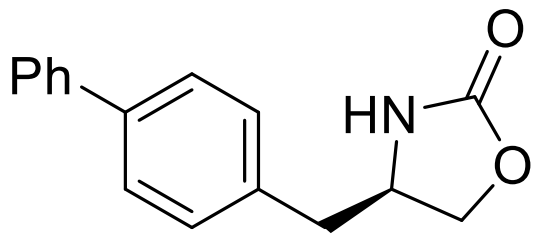

27

27: Yield: 23.0 mg, 91%, 96% ee, white solid, R_f = 0.4 (*n*-hexane/ethyl acetate = 1/2).
^{1}H NMR (400 MHz, $CDCl_3$) δ 7.84 – 7.51 (m, 4H), 7.49 – 7.40 (m, 2H), 7.39 – 7.29 (m, 1H), 7.28 – 7.05 (m, 2H), 5.95 (s, 1H), 4.46 (t, *J* = 8.2 Hz, 1H), 4.29 – 3.99 (m, 2H), 3.04 – 2.78 (m, 2H).
^{13}C NMR (101 MHz, $CDCl_3$) δ 159.6, 140.6, 140.3, 135.0, 129.6, 128.9, 127.8, 127.5, 127.1, 69.7, 53.9, 41.1.
Chiral HPLC (AD-H, ^{i}PrOH/*n*-hexane = 10/90, 1.0 mL/min) t_R 15.8 min (minor), 17.0 min (major).
$[\alpha]_D^{25}$ = 49.17 (*c* = 0.6, $CHCl_3$).
The NMR data matched with those reported in literature.[29]

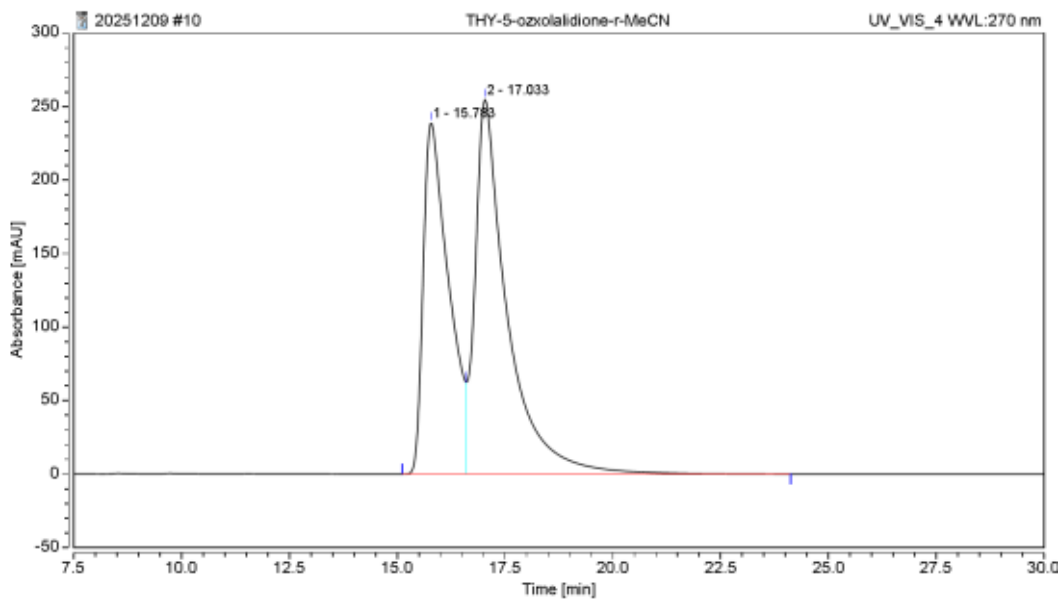

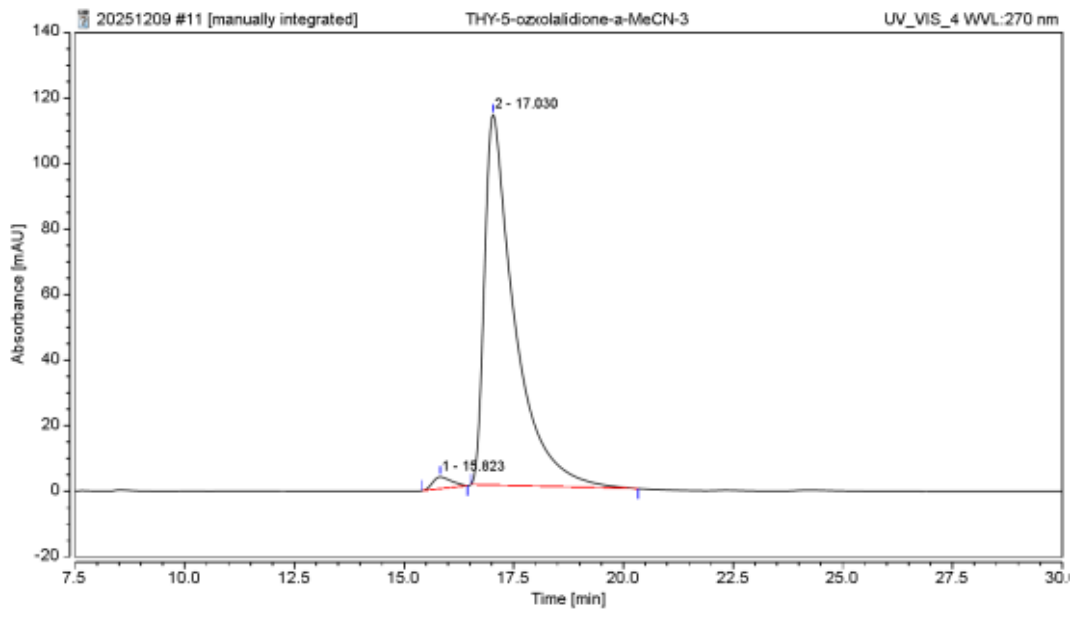

	Retention Time	Area	% Area
1	15.783	239.3274	42.51
2	17.033	323.6257	57.49

	Retention Time	Area	% Area
1	15.823	1.7831	1.95
2	17.030	89.4594	98.05

10.5. Synthesis of compounds 28 and 29.

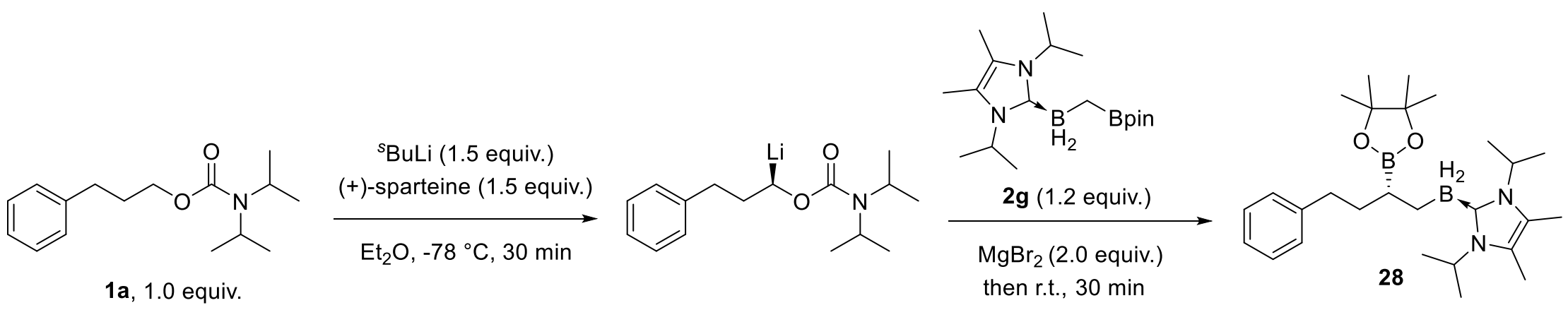

Under argon, to an oven dried 4 mL reaction tube equipped with a magnetic stir bar and a septum was charged with **1a** (26.4 mg, 0.1 mmol, 1.0 equiv.), (+)-sparteine (35.2 mg, 0.15 mmol, 1.5 equiv.) in Et_2O (500 µL). The reaction was cooled to -78 °C and stirred for 10 min. ^{s}BuLi (1.3 M in cyclohexane, 0.12 mL, 0.15 mmol, 1.5 equiv.) was then slowly added and the mixture was stirred vigorously (without splashing) at -78 °C for

30 min. After that, a solution of the **2g** (40.1 mg, 0.12 mmol, 1.2 equiv.) in Et_2O (1.2 mL) was added dropwise. The reaction mixture was stirred at this temperature for an additional 10 min. $MgBr_2$ (freshly made from Mg and 1,2-dibromoethane, 0.5 M in Et_2O, 0.4 mL, 0.2 mmol, 2.0 equiv.) was then added dropwise and the reaction mixture was stirred at -78 °C for 20 min. The reaction mixture was then warmed to room temperature and stirred for another 30 min. Upon completion, the reaction was quenched with aqueous NH_4Cl and extracted with Et_2O (3 × 3 mL). The combined organic phases were dried over $MgSO_4$, and the solvent was removed in vacuo. The resulting crude residue was purified by flash column chromatography to give the product **28** (74%, 33.4 mg).

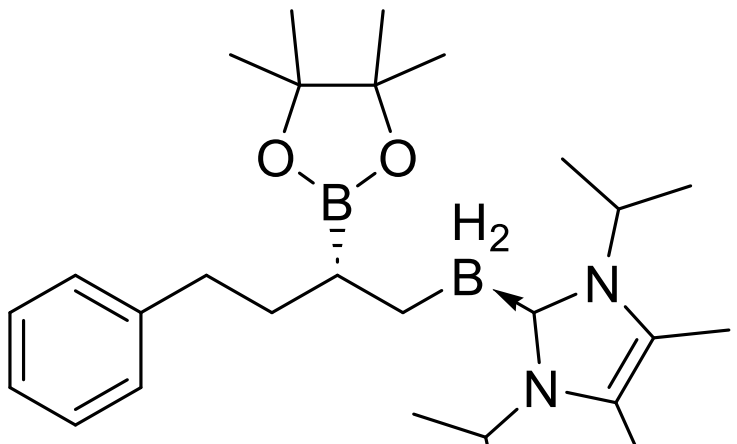

28

28: Yield: 33.4 mg, 74%, 97% ee, colorless oil, R_f = 0.4 (*n*-hexane/ethyl acetate = 3/1).
^{1}H NMR (400 MHz, $CDCl_3$) δ 7.25 – 7.07 (m, 5H), 5.66 – 5.30 (m, 2H), 2.70 – 2.51 (m, 2H), 2.19 (s, 6H), 1.83 – 1.66 (m, 2H), 1.53 – 1.39 (m, 12H), 1.28 (s, 12H), 1.07 – 0.97 (m, 1H), 0.57 – 0.35 (m, 2H).
^{13}C NMR (101 MHz, $CDCl_3$) δ 144.5, 128.6, 128.1, 125.2, 123.4, 82.2, 49.4, 37.5, 36.4, 25.3, 25.0, 21.8, 10.5.
^{11}B NMR (128 MHz, $CDCl_3$) δ 35.1 (s, 1B), -26.6 (t, J = 83.6 Hz, 1B).
HRMS (ESI): Calcd for $C_{27}H_{46}B_2N_2O_2Na^+$ $[M+Na]^+$: 475.3638, found: 475.3647.
IR (neat, cm^{-1}): ν 3022, 2923, 2584, 2367, 1495, 1453, 1260, 1141, 1022.
Chiral HPLC (OX-H, ^{i}PrOH/*n*-hexane = 2/98, 1.0 mL/min) t_R 16.4 min (minor), 21.5 min (major).
$[\alpha]_D^{25}$ = -15.00 (c = 0.7, $CHCl_3$).

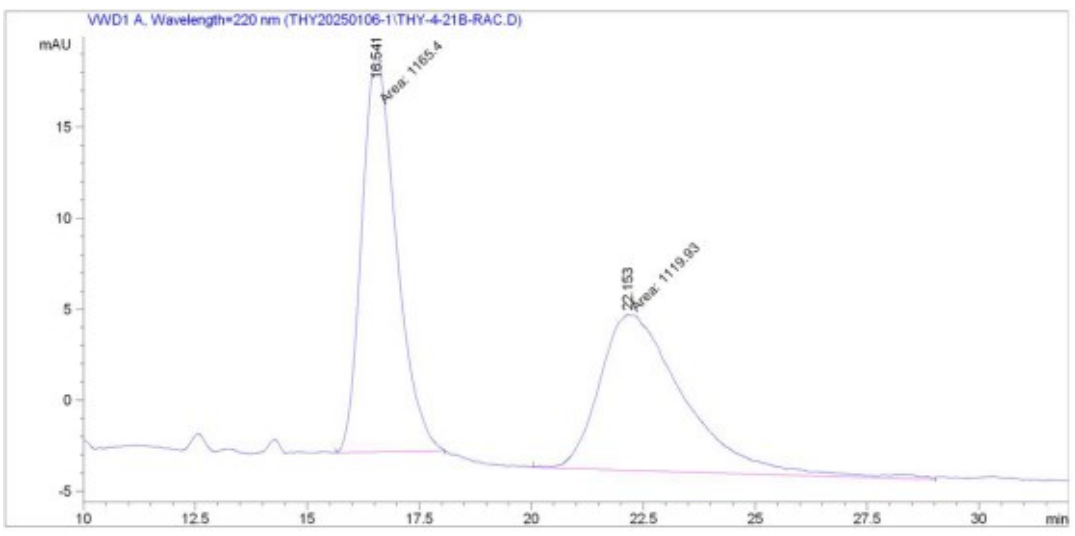

Peak #	RetTime [min]	Type	Width [min]	Area [mAU*s]	Height [mAU]	Area %
1	16.541	MM	0.8959	1165.40344	21.67994	50.9949
2	22.153	MM	2.1894	1119.92810	8.52547	49.0051

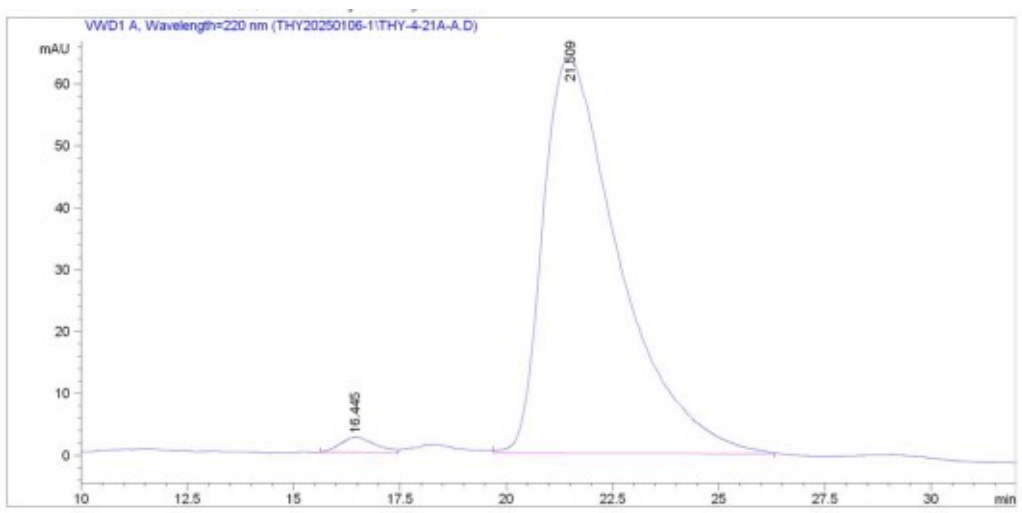

Peak #	RetTime [min]	Type	Width [min]	Area [mAU*s]	Height [mAU]	Area %
1	16.445	BV	0.7527	138.70741	2.45942	1.7046
2	21.509	VB	1.5630	7998.36426	63.36776	98.2954

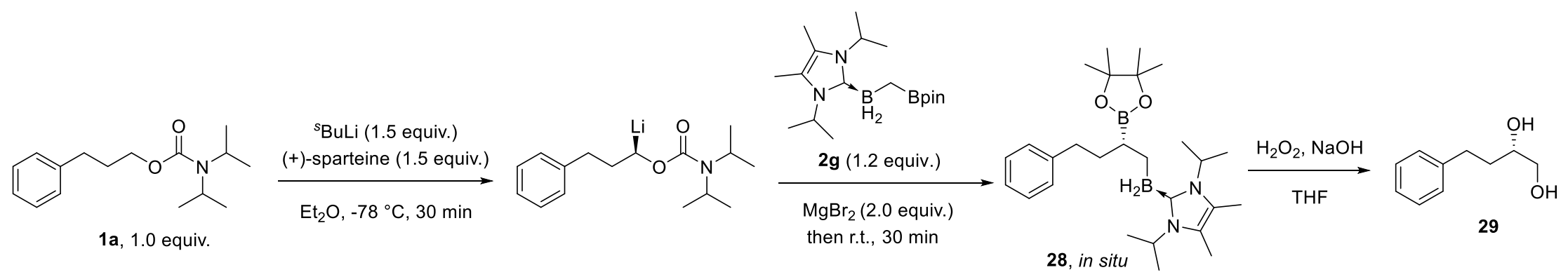

Under argon, to an oven dried 4 mL reaction tube equipped with a magnetic stir bar and a septum was charged with **1a** (26.4 mg, 0.1 mmol, 1.0 equiv.), (+)-sparteine (35.2 mg, 0.15 mmol, 1.5 equiv.) and Et_2O (500 µL). The reaction was cooled to -78 °C and stirred for 10 min. ^{s}BuLi (1.3 M in cyclohexane, 0.12 mL, 0.15 mmol, 1.5 equiv.) was then slowly added and the mixture was stirred vigorously (without splashing) at -78 °C for 30 min. After that, a solution of the **2g** (40.1 mg, 0.12 mmol, 1.2 equiv.) in Et_2O (1.2 mL) was added dropwise. The reaction mixture was stirred at this temperature for an additional 10 min, and then $MgBr_2$ (freshly made from Mg and 1,2-dibromoethane, 0.5 M in Et_2O, 0.4 mL, 0.2 mmol, 2.0 equiv.) was added dropwise. The reaction mixture was then warmed to room temperature and stirred for another 30 min. Upon completion, the reaction was quenched with aqueous NH_4Cl and extracted with Et_2O (3 × 3 mL). The combined organic phases were dried over $MgSO_4$, and the solvent was removed in vacuo. The resulting crude residue **28** was used for the next step without purification.
Under air, to a 20 mL vial equipped with magnetic stir bar was added crude **28** and THF (1 mL). A solution of 30 wt% H_2O_2/ ~15 wt% NaOH (1:2, 1.5 mL in total) was slowly added at 0 °C and the reaction mixture was then warmed to room temperature and stirred for 6 h. Upon completion, the reaction was quenched with H_2O and extracted with ethyl acetate (3 × 3 mL). The combined organic phases were dried over $MgSO_4$, and the solvent was removed in vacuo. The resulting residue was purified by flash column chromatography to give product **29** (74%, 12.3 mg).

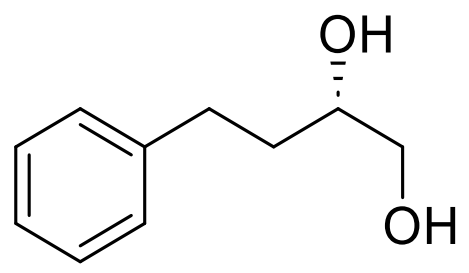

29

29: Two-step yield: 12.3 mg, 74%, 96% ee, colorless oil, R_f = 0.4 (*n*-hexane/ethyl acetate = 1/1).
^{1}H NMR (400 MHz, $CDCl_3$) δ 7.33 – 7.27 (m, 1H), 7.24 – 7.16 (m, 3H), 3.81 – 3.61 (m, 2H), 3.58 – 3.37 (m, 1H), 2.90 – 2.74 (m, 1H), 2.74 – 2.64 (m, 1H), 2.58 – 2.32 (m, 2H), 2.01 – 1.59 (m, 2H).
^{13}C NMR (101 MHz, $CDCl_3$) δ 141.8, 128.6, 128.5, 126.1, 71.7, 66.9, 34.8, 31.9.
HRMS (ESI): Calcd for $C_{10}H_{14}O_2Na^+$ $[M+Na]^+$: 189.0892, found: 189.0886.
Chiral HPLC (IC, ^{i}PrOH/*n*-hexane = 5/95, 1.0 mL/min) t_R 24.3 min (minor), 26.7 min (major).
$[\alpha]_D^{25}$= -13.00 (*c* = 0.1, $CHCl_3$), Ref: $[\alpha]_D^{20}$= -13.6 (*c* = 1.00, $CHCl_3$).[30]
The NMR data and HPLC condition matched with those reported in literature.[31]

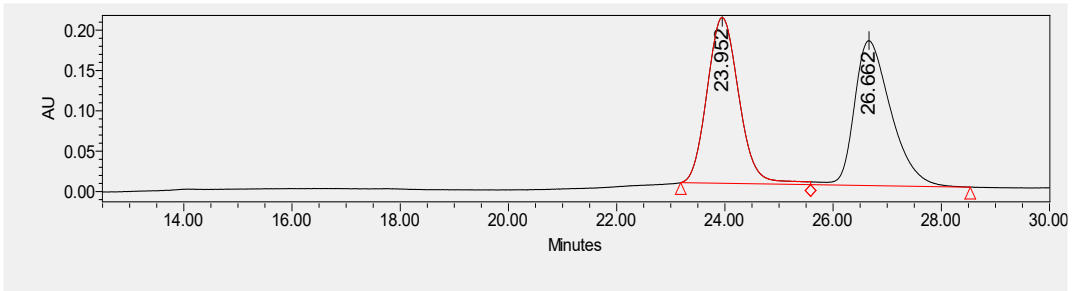

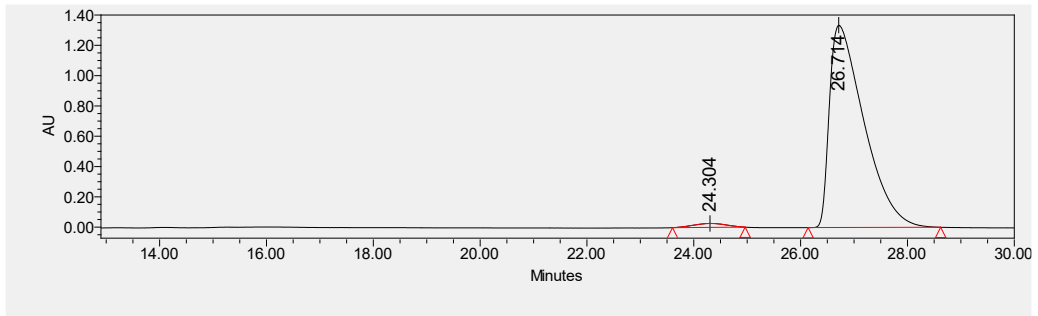

	Retention Time	Area	% Area
1	23.952	8408402	49.98
2	26.662	8415905	50.02

	Retention Time	Area	% Area
1	24.304	1111191	1.82
2	26.714	59819984	98.18

11. X-ray crystallographic data.

Single-crystal X-ray data of **3t**, **3ae**, **11b** and **15a** were collected at 150.0 K on a Bruker D8 Venture with PHOTO III diffractometer using Cu-Kα radiation, λ = 1.54178 Å. The structures were solved with the SHELXT structure solution program[32] using Intrinsic Phasing and refined with the SHELXL refinement package[33] using Least Squares minimization with Olex2 graphic interface.[34]

CCDC 2537415, CCDC 2565472, CCDC 2575777 and CCDC 2537417 contain the supplementary crystallographic data for this paper. These data can be obtained free of charge from the Cambridge Crystallographic Data Centre via www.ccdc.cam.ac.uk/data_request/cif.

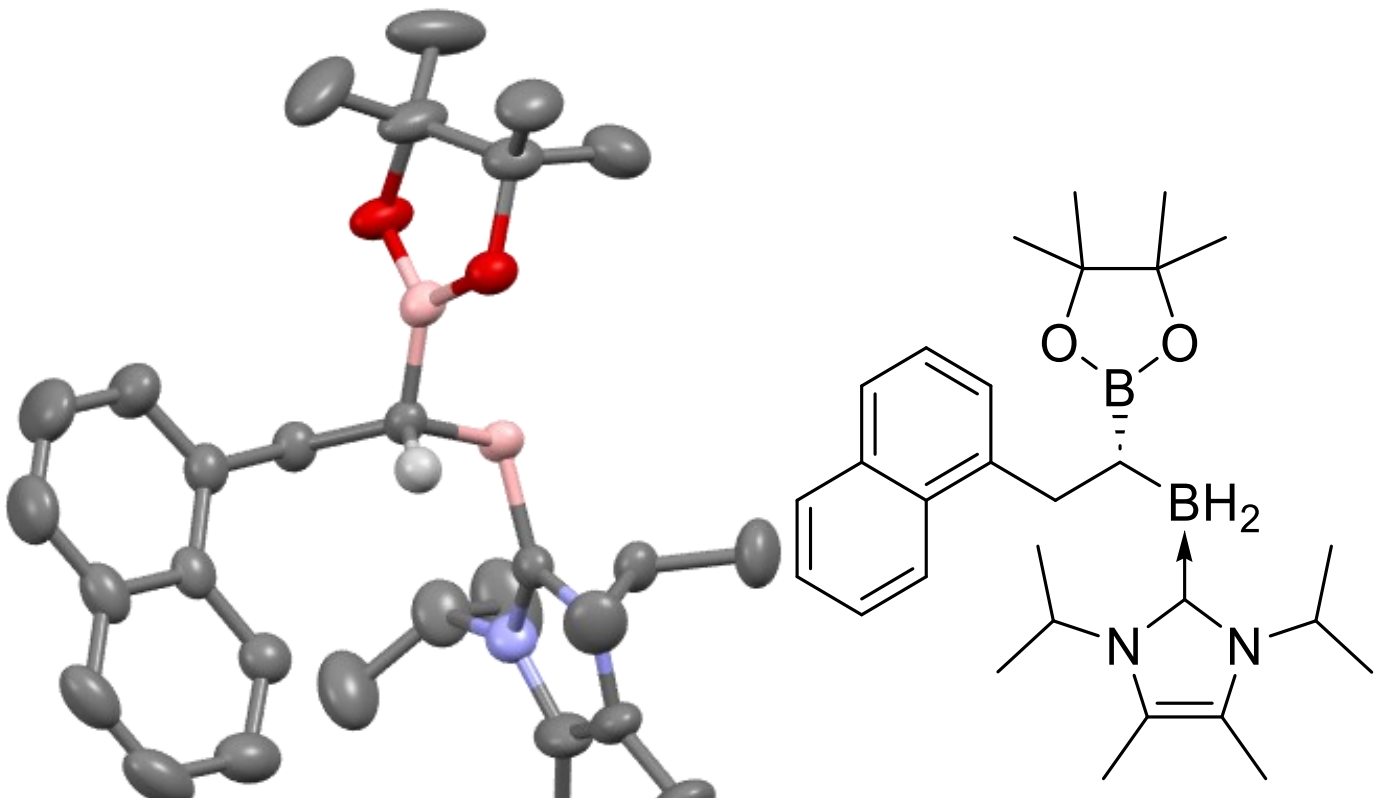

3t

X-ray crystallography of compound **3t**. the ellipsoid contour % probability level is 50%.

Fig. S5. Crystal structure of compound **3t** (CCDC 2537415)

Table S3. Crystal data and structure refinement for compound 3t

Empirical formula	$C_{29}H_{44}B_2N_2O_2$
Formula weight	474.28
Temperature/K	150.0
Crystal system	tetragonal
Space group	I_4
a/Å	24.2974(10)
b/Å	24.2974(10)
c/Å	10.4479(7)
α/°	90
β/°	90

γ/°	90
Volume/Å^3	6168.1(7)
Z	8
ρ_{calc}g/cm^3	1.021
μ/mm^{-1}	0.476
F(000)	2064.0
Crystal size/mm^3	0.15 × 0.12 × 0.03
Radiation	CuKα (λ = 1.54178)
2Θ range for data collection/°	5.144 to 136.136
Index ranges	-29 ≤ h ≤ 28, -29 ≤ k ≤ 29, -12 ≤ l ≤ 12
Reflections collected	53592
Independent reflections	5635 [R_{int} = 0.0297, R_{sigma} = 0.0157]
Data/restraints/parameters	5635/1/334
Goodness-of-fit on F^2	1.024
Final R indexes [I>=2σ (I)]	R_1 = 0.0309, wR_2 = 0.0820
Final R indexes [all data]	R_1 = 0.0311, wR_2 = 0.0822
Largest diff. peak/hole / e Å^{-3}	0.12/-0.17
Flack parameter	0.02(2)

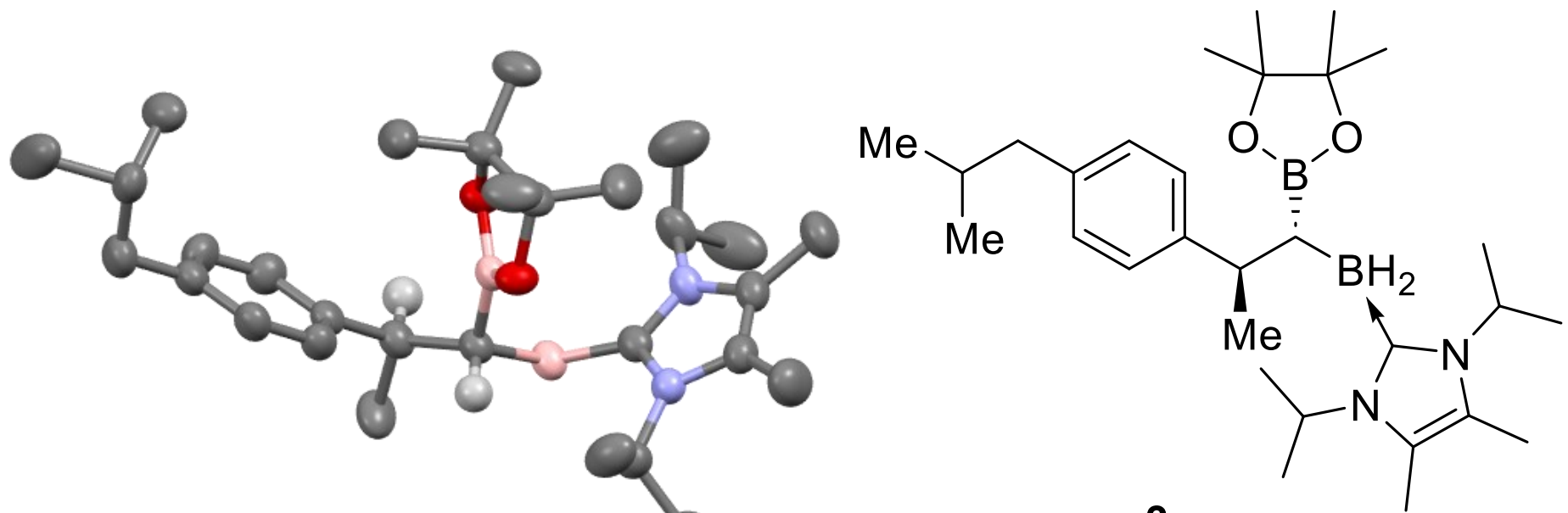

3ae

X-ray crystallography of compound **3ae**. the ellipsoid contour % probability level is 50%.

Fig. S6. Crystal structure of compound **3ae** (CCDC 2565472)

Table S4. Crystal data and structure refinement for compound 3ae

Empirical formula	$C_{30}H_{48.59}B_2N_2O_2$
Formula weight	490.92
Temperature/K	200.0
Crystal system	orthorhombic
Space group	$P2_12_12_1$
a/Å	11.2475(8)
b/Å	14.6524(10)
c/Å	18.9477(13)
α/°	90
β/°	90
γ/°	90

Volume/Å^3	3122.6(4)
Z	4
ρ_{calc}g/cm^3	1.044
μ/mm^{-1}	0.482
F(000)	1074.0
Crystal size/mm^3	0.154 × 0.105 × 0.082
Radiation	CuKα (λ = 1.54178)
2Θ range for data collection/°	7.626 to 136.364
Index ranges	-13 ≤ h ≤ 13, -17 ≤ k ≤ 15 , -22 ≤ l ≤ 22
Reflections collected	28756
Independent reflections	5509 [R_{int} = 0.0403, R_{sigma} = 0.0304]
Data/restraints/parameters	5509/84/367
Goodness-of-fit on F^2	1.057
Final R indexes [I>=2σ (I)]	R_1 = 0.0420, wR_2 = 0.1176
Final R indexes [all data]	R_1 = 0.0475, wR_2 = 0.1248
Largest diff. peak/hole / e Å^{-3}	0.47/-0.17
Flack parameter	-0.04(5)

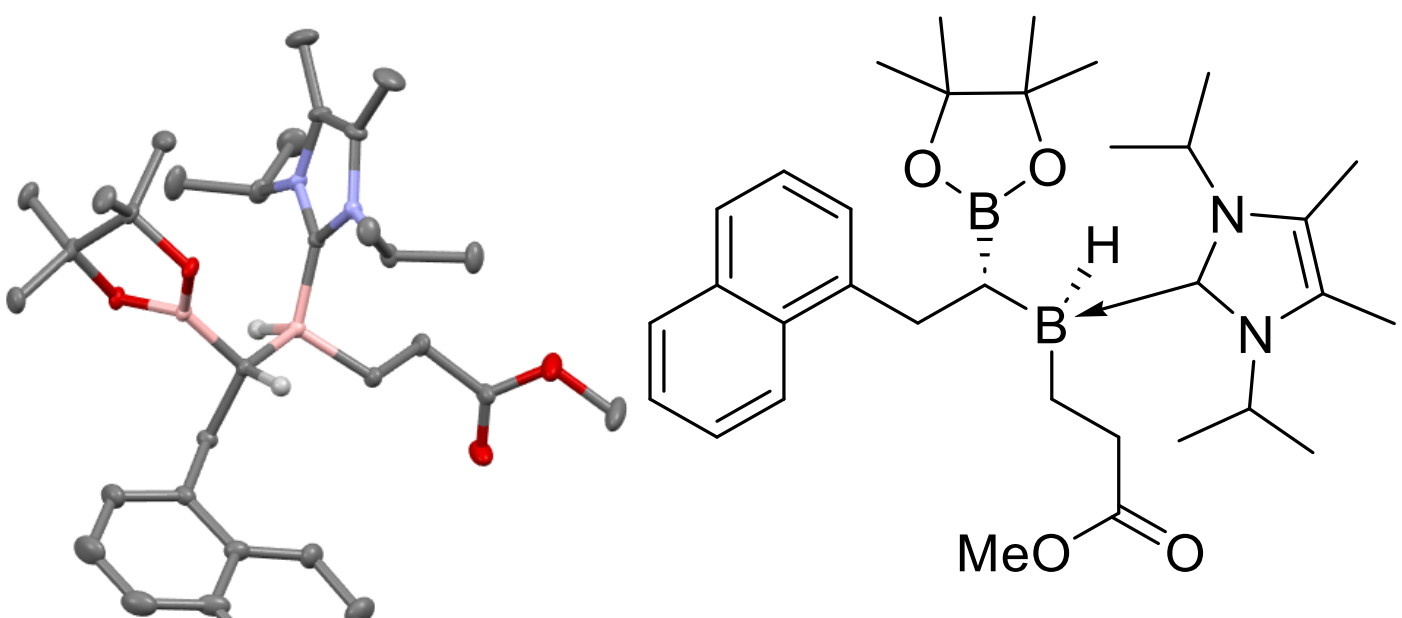

11b

X-ray crystallography of compound **11b**. the ellipsoid contour % probability level is 50%.

Fig. S7. Crystal structure of compound **11b** (CCDC 2575777)

Table S5. Crystal data and structure refinement for compound 15a

Identification code	zhiyi_50_Cu100K_auto
Empirical formula	$C_{33}H_{50}B_2N_2O_4$
Formula weight	560.424
Temperature/K	99.92(17)
Crystal system	orthorhombic
Space group	$P2_12_12_1$
a/Å	11.40017(15)
b/Å	15.35528(19)
c/Å	18.8468(2)
α/°	90

β/°	90
γ/°	90
Volume/Å^3	3299.19(7)
Z	4
ρ_{calc}g/cm^3	1.128
μ/mm^{-1}	0.564
F(000)	1219.7
Crystal size/mm^3	0.17 × 0.13 × 0.1
Radiation	CuKα (λ = 1.54184)
2Θ range for data collection/°	7.42 to 149.84
Index ranges	$-14 \leq h \leq 14$, $-19 \leq k \leq 18$, $-15 \leq l \leq 23$
Reflections collected	20272
Independent reflections	6657 [R_{int} = 0.0536, R_{sigma} = 0.0500]
Data/restraints/parameters	6657/39/499
Goodness-of-fit on F^2	1.002
Final R indexes [I>=2σ (I)]	R_1 = 0.0410, wR_2 = 0.1011
Final R indexes [all data]	R_1 = 0.0444, wR_2 = 0.1046
Flack parameter	-0.33(11)

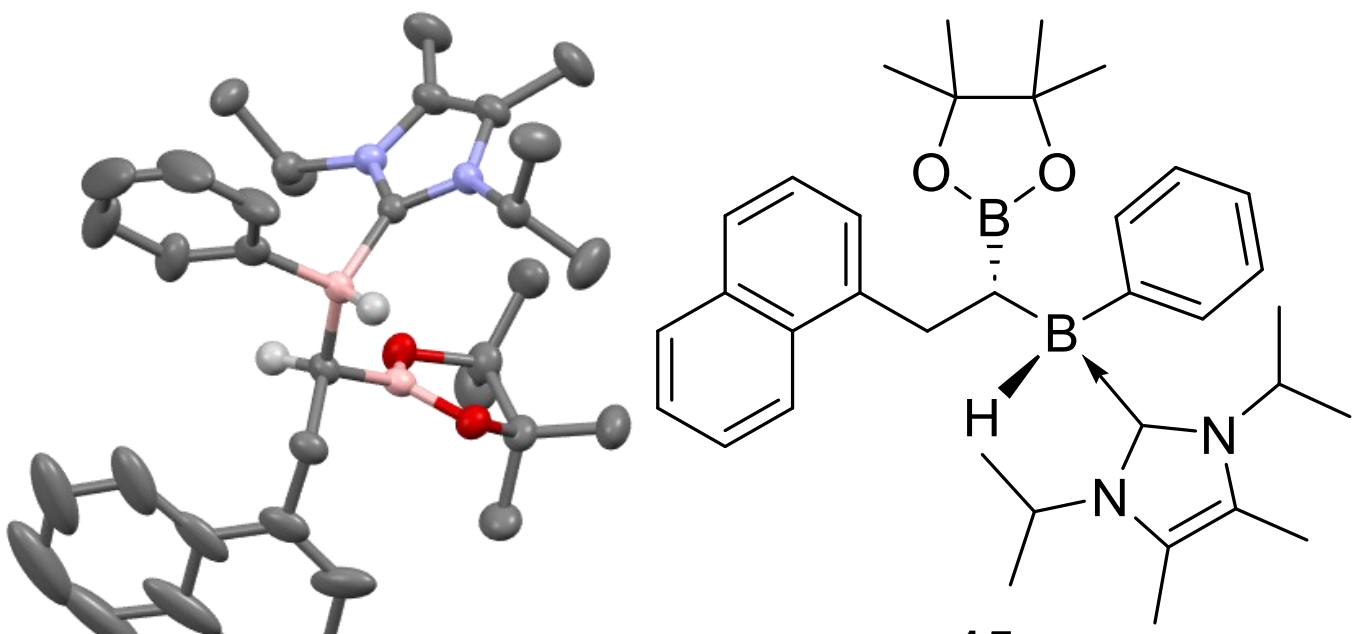

15a

X-ray crystallography of compound **15a**. the ellipsoid contour % probability level is 50%.

Fig. S8. Crystal structure of compound **15a** (CCDC 2537417)

Table S6. Crystal data and structure refinement for compound 15a

Empirical formula	$C_{35}H_{48}B_2N_2O_2$
Formula weight	550.37
Temperature/K	150.00
Crystal system	monoclinic
Space group	$P2_1$
a/Å	9.5929(3)
b/Å	17.7621(6)
c/Å	9.9511(3)
α/°	90
β/°	102.288(2)

γ/°	90
Volume/Å^3	1656.72(9)
Z	2
ρ_{calc}g/cm^3	1.103
μ/mm^{-1}	0.509
F(000)	596.0
Crystal size/mm^3	0.194 × 0.057 × 0.04
Radiation	CuKα (λ = 1.54178)
2Θ range for data collection/°	9.436 to 136.61
Index ranges	$-11 \leq h \leq 11$, $-21 \leq k \leq 20$, $-11 \leq l \leq 11$
Reflections collected	41251
Independent reflections	5984 [R_{int} = 0.0385, R_{sigma} = 0.0273]-
Data/restraints/parameters	5984/121/380
Goodness-of-fit on F^2	1.032
Final R indexes [I>=2σ (I)]	R_1 = 0.0406, wR_2 = 0.1054
Final R indexes [all data]	R_1 = 0.0411, wR_2 = 0.10598
Largest diff. peak/hole / e Å^{-3}	0.26/-0.33
Flack parameter	0.02(4)

12. References.

1. Jang, W. J.; Yun, J. Catalytic Asymmetric Conjugate Addition of a Borylalkyl Copper Complex for Chiral Organoboronate Synthesis. *Angew. Chem. Int. Ed.* **2019,** *58*, 18131-18135.

2. Ye, Z.; Kwok, C. Y.; Lam, S. L.; Wu, L.; Lyu, H. Copper-Catalyzed C–B(sp^3) Bond Formation through the Intermediacy of Cu–B(sp^3) Complex. *J. Am. Chem. Soc.* **2025,** *147*, 14915-14923.

3. You, T.; Luu, Q. H.; Li, J. A C-to-B Atom Swap on Coumarins and Dibenzolactones. *Angew. Chem. Int. Ed.* **2025,** *64*, e202509674.

4. Stymiest, J. L.; Dutheuil, G.; Mahmood, A.; Aggarwal, V. K. Lithiated Carbamates: Chiral Carbenoids for Iterative Homologation of Boranes and Boronic Esters. *Angew. Chem. Int. Ed.* **2007,** *46*, 7491-7494.

5. Gerleve, C.; Kischkewitz, M.; Studer, A. Synthesis of α-Chiral Ketones and Chiral Alkanes Using Radical Polar Crossover Reactions of Vinyl Boron Ate Complexes. *Angew. Chem. Int. Ed.* **2018,** *57*, 2441-2444.

6. Li, H.-Z.; Ren, G.-A.; Chen, X.; Jiao, R.-Q.; Ding, T.; Liu, X.-Y. Nickel-Catalyzed C(sp^3)–C(sp^3) Cross-Coupling of Carboxamides and Unactivated Alkyl Iodides. *J. Org. Chem.* **2025,** *90*, 7345-7350.

7. Hoang, G. L.; Takacs, J. M. Enantioselective γ-borylation of unsaturated amides and stereoretentive Suzuki-Miyaura cross-coupling. *Chem. Sci.* **2017,** *8*, 4511-4516.

8. Wang, Z.; Zhou, J.; Lin, L.; Hu, M.-H. Discovery of a far-red carbazole-benzoindolium fluorescent ligand that selectively targets mitochondrial DNA and suppresses breast cancer growth. *Eur. J. Med. Chem.* **2024,** *264*, 116046.

9. Guo, X.; Price, N. G.; Zhu, Q. Electrochemical Cyanation of Alcohols Enabled by an Iodide-Mediated Phosphine P(V/III) Redox Couple. *Org. Lett.* **2024,** *26*, 7347-7351.

10. Roesner, S.; Blair, D. J.; Aggarwal, V. K. Enantioselective installation of adjacent tertiary benzylic stereocentres using lithiation–borylation–protodeboronation methodology. Application to the synthesis of bifluranol and fluorohexestrol. *Chem. Sci.* **2015,** *6*, 3718-3723.

11. Lunic, D.; Vystavkin, N.; Qin, J.; Teskey, C. J. Dual-Catalytic Structural Isomerisation as a Route to α-Arylated Ketones. *Angew. Chem. Int. Ed.* **2024,** *63*, e202409388.

12. Blair, D. J.; Zhong, S.; Hesse, M. J.; Zabaleta, N.; Myers, E. L.; Aggarwal, V. K. Full chirality transfer in the synthesis of hindered tertiary boronic esters under in situ lithiation–borylation conditions. *Chem. Comm.* **2016,** *52*, 5289-5292.

13. Burns, M.; Essafi, S.; Bame, J. R.; Bull, S. P.; Webster, M. P.; Balieu, S.; Dale, J. W.; Butts, C. P.; Harvey, J. N.; Aggarwal, V. K. Assembly-line synthesis of organic molecules with tailored shapes. *Nature* **2014,** *513*, 183-188.

14. (a) Bailey, W. F.; Patricia, J. J. The mechanism of the lithium - halogen Interchange reaction : a review of the literature. *J. Organomet. Chem.* **1988,** *352*, 1-46; (b) Fandrick, K. R.; Patel, N. D.; Mulder, J. A.; Gao, J.; Konrad, M.; Archer, E.; Buono, F. G.; Duran, A.; Schmid, R.; Daeubler, J.; Fandrick, D. R.; Ma, S.; Grinberg, N.; Lee, H.; Busacca,

C. A.; Song, J. J.; Yee, N. K.; Senanayake, C. H. Addressing the Configuration Stability of Lithiated Secondary Benzylic Carbamates for the Development of a Noncryogenic Stereospecific Boronate Rearrangement. *Org. Lett.* **2014,** *16*, 4360-4363.
15. Liang, H.; Berwanger, M. R.; Morken, J. P. Stereospecific Phosphination and Thioetherification of Organoboronic Esters. *J. Am. Chem. Soc.* **2024,** *146*, 18873-18878.
16. Edelstein, E. K.; Grote, A. C.; Palkowitz, M. D.; Morken, J. P. A protocol for direct stereospecific amination of primary, secondary, and tertiary alkylboronic esters. *Synlett* **2018,** *29*, 1749-1752.
17. Li, F.-X.; Liu, Y.; Zhang, K.; Wang, C.; Yu, J.-X.; He, X.-J.; Liang, G.; Hou, H.-Y.; Tung, C.-H.; Wu, L.-Z. Multifunctionalization of N-Heterocyclic Carbene Boranes by a Single Metal-free Photocatalyst. *Adv. Synth. Catal.* **2025,** *367*, e202401440.
18. Prokofjevs, A.; Jermaks, J.; Borovika, A.; Kampf, J. W.; Vedejs, E. Electrophilic C–H Borylation and Related Reactions of B–H Boron Cations. *Organomet.* **2013,** *32*, 6701-6711.
19. Aupic, C.; Abdou Mohamed, A.; Figliola, C.; Nava, P.; Tuccio, B.; Chouraqui, G.; Parrain, J.-L.; Chuzel, O. Highly diastereoselective preparation of chiral NHC-boranes stereogenic at the boron atom. *Chem. Sci.* **2019,** *10*, 6524-6530.
20. Gao, D.-W.; Gao, Y.; Shao, H.; Qiao, T.-Z.; Wang, X.; Sanchez, B. B.; Chen, J. S.; Liu, P.; Engle, K. M. Cascade CuH-catalysed conversion of alkynes into enantioenriched 1,1-disubstituted products. *Nat. Catal.* **2020,** *3*, 23-29.
21. Noh, D.; Chea, H.; Ju, J.; Yun, J. Highly Regio- and Enantioselective Copper-Catalyzed Hydroboration of Styrenes. *Angew. Chem. Int. Ed.* **2009,** *48*, 6062-6064.
22. Zheng, P.; Zhou, P.; Wang, D.; Xu, W.; Wang, H.; Xu, T. Dual Ni/photoredox-catalyzed asymmetric cross-coupling to access chiral benzylic boronic esters. *Nat. Commun.* **2021,** *12*, 1646.
23. Erb, W.; Grassot, J.-M.; Linder, D.; Neuville, L.; Zhu, J. Enantioselective Synthesis of Putative Lipiarmycin Aglycon Related to Fidaxomicin/Tiacumicin B. *Angew. Chem. Int. Ed.* **2015,** *54*, 1929-1932.
24. Medina, E.; Moyano, A.; Pericàs, M. A.; Riera, A. Synthesis of N-Boc-β-Aryl Alanines and of N-Boc-β-Methyl-β-aryl Alanines by Regioselective Ring-Opening of Enantiomerically Pure N-Boc-Aziridines. *J. Org. Chem.* **1998,** *63*, 8574-8578.
25. Vilaivan, T. A rate enhancement of tert-butoxycarbonylation of aromatic amines with Boc2O in alcoholic solvents. *Tetrahedron Lett.* **2006,** *47*, 6739-6742.
26. Morales-Ortega, G.; Merino, E.; Carreras, J. Visible-light-initiated metal-free Csp^3–Csp^3 to Csp^3–N conversion in homobenzylic sulfonamides with N-iodoimides. *Chem. Sci.* **2025,** *16*, 11413-11418.
27. Pan, T.; Jiang, X.; Huang, M.; Zhang, L.; Luo, S. Visible Light-Promoted Deracemization of α-Amino Aldehyde by Synergistic Chiral Primary Amine and Hypervalent Iodine Catalysis. *J. Am. Chem. Soc.* **2025,** *147*, 6280-6287.
28. Xue, Y.; Wu, S.; 徐强; 黄双; 李维思; 杨健 Method for preparing sacubitril intermediate. CN107382779B, 2020-04-17.
29. Lang, Q.; Zhao, J.; Liu, C.; Zhang, Z.; Ding, X. Sabobiqu intermediate, preparation method and application thereof. CN116987012A, 2023-11-03.

30. Martynow, J. G.; Jóźwik, J.; Szelejewski, W.; Achmatowicz, O.; Kutner, A.; Wiśniewski, K.; Winiarski, J.; Zegrocka-Stendel, O.; Gołębiewski, P. A New Synthetic Approach to High-Purity (15R)-Latanoprost. *Eur. J. Org. Chem.* **2007,** *2007*, 689-703.
31. Gao, Q.; Li, Y.; Chen, L.; Xie, L.-J.; Shao, X.; Ke, Z.; Xu, S. Enantioselective α-C(sp^3)–H Borylation of Masked Primary Alcohols Enabled by Iridium Catalysis. *J. Am. Chem. Soc.* **2025,** *147*, 88-95.
32. Sheldrick, G. M. SHELXT–Integrated space-group and crystal-structure determination. *Found. Crystallogr.* **2015,** *71*, 3-8.
33. Sheldrick, G. M. Crystal structure refinement with SHELXL. *C: Cryst. Struct. Commun.* **2015,** *71*, 3-8.
34. Dolomanov, O. V.; Bourhis, L. J.; Gildea, R. J.; Howard, J. A.; Puschmann, H. OLEX2: a complete structure solution, refinement and analysis program. *J. Appl. Crystallogr.* **2009,** *42*, 339-341.

13. NMR spectra.

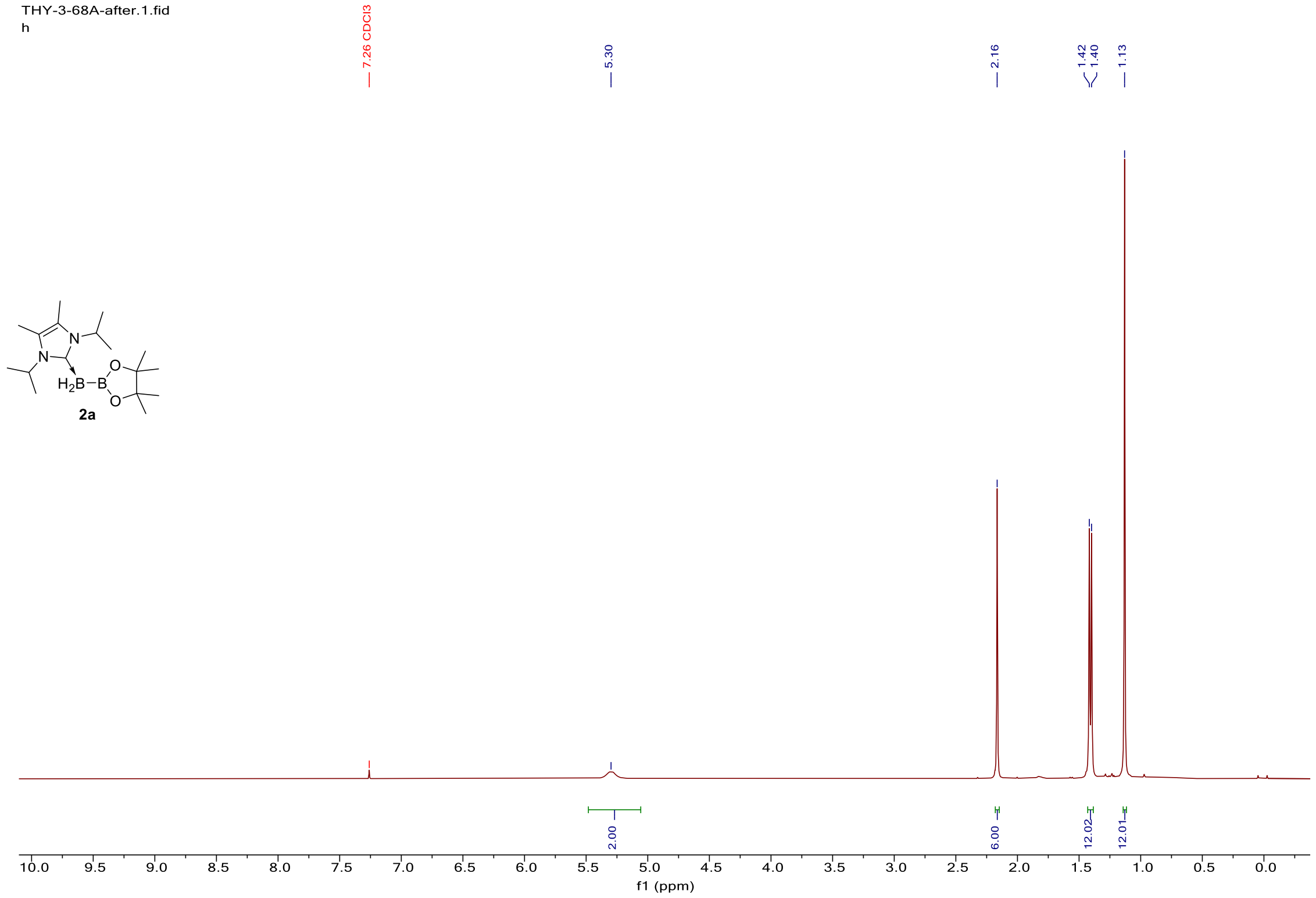

^{1}H NMR (400 MHz, $CDCl_3$)

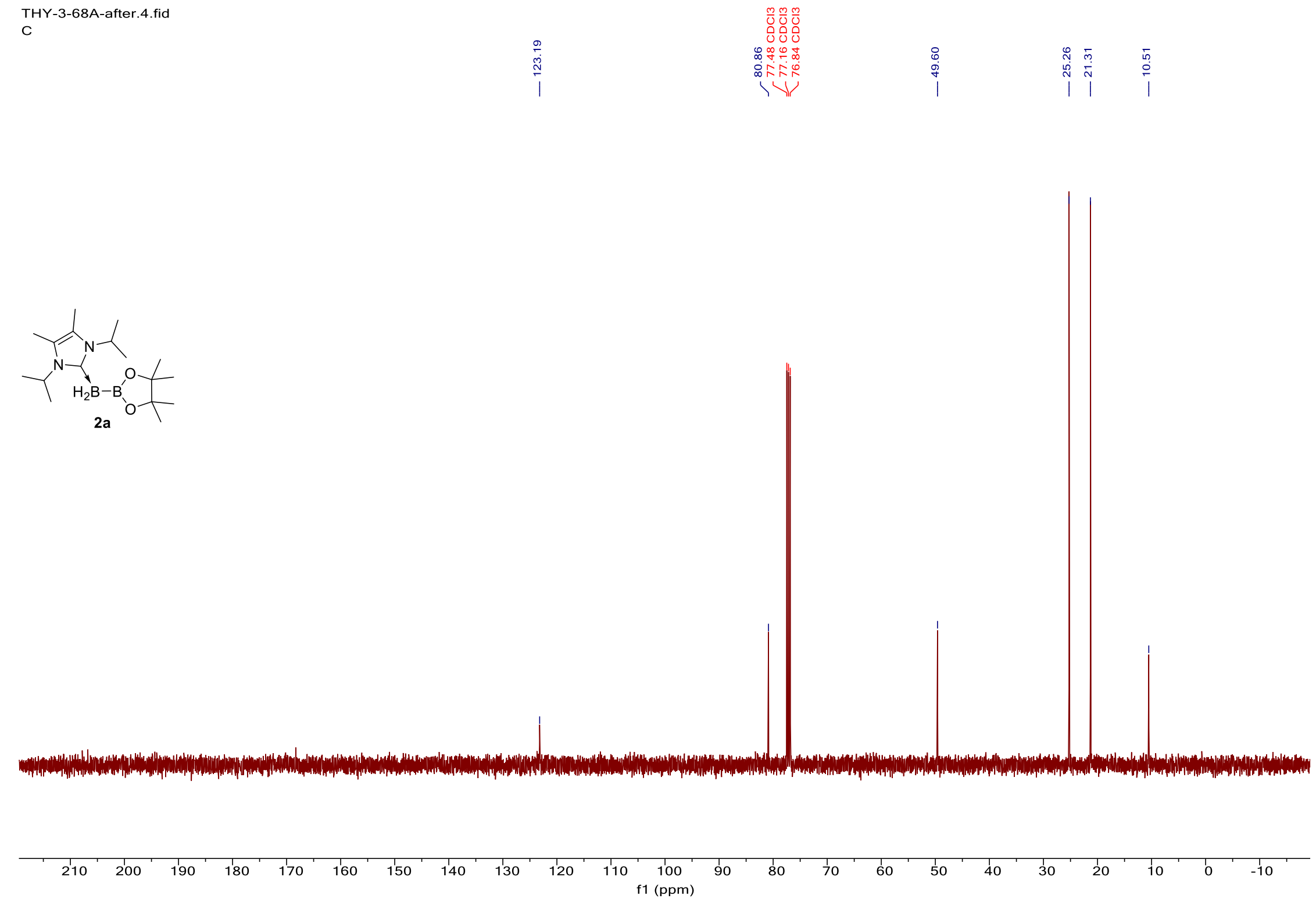

^{13}C NMR (101 MHz, $CDCl_3$)

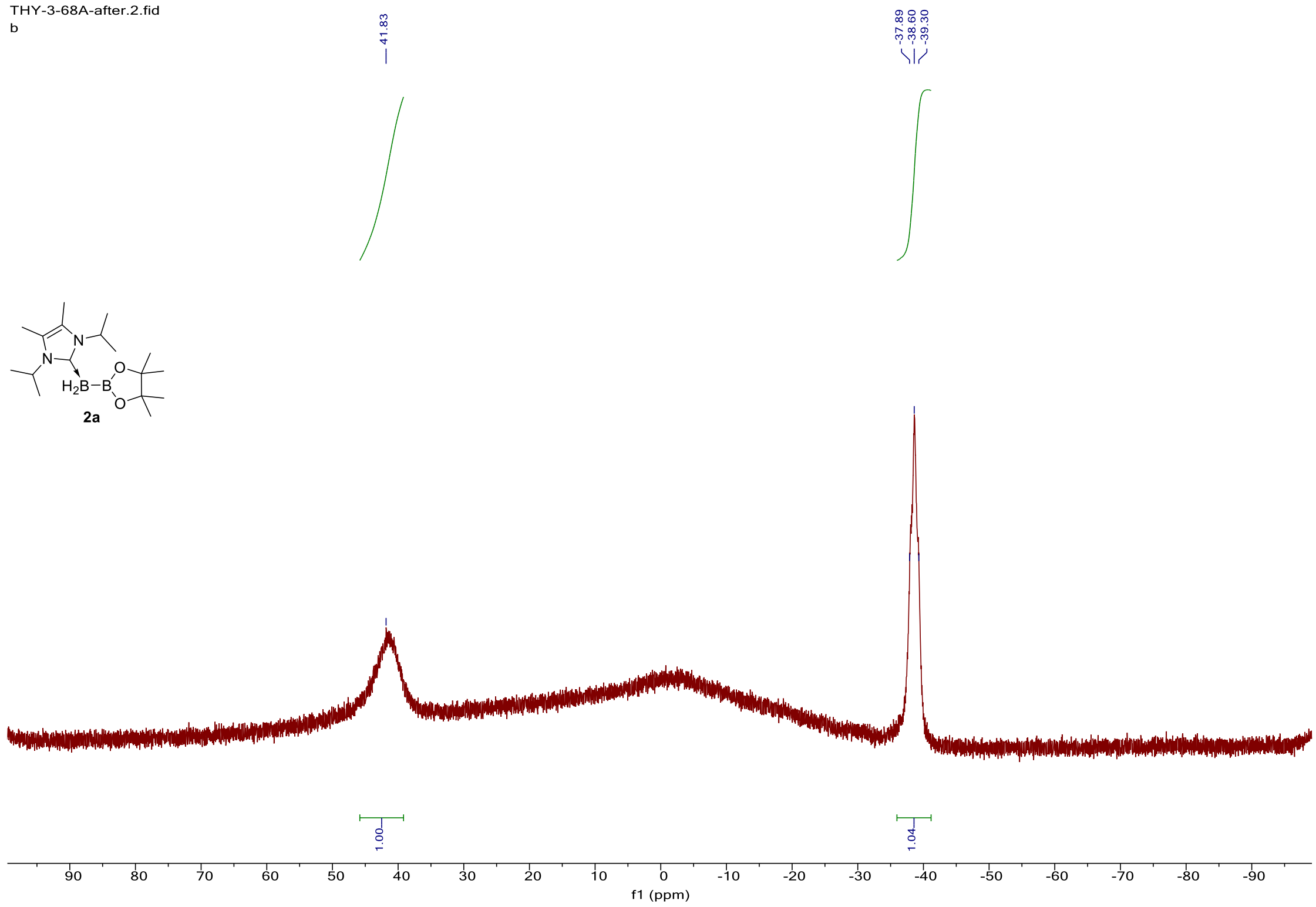

^{11}B NMR (128 MHz, $CDCl_3$)

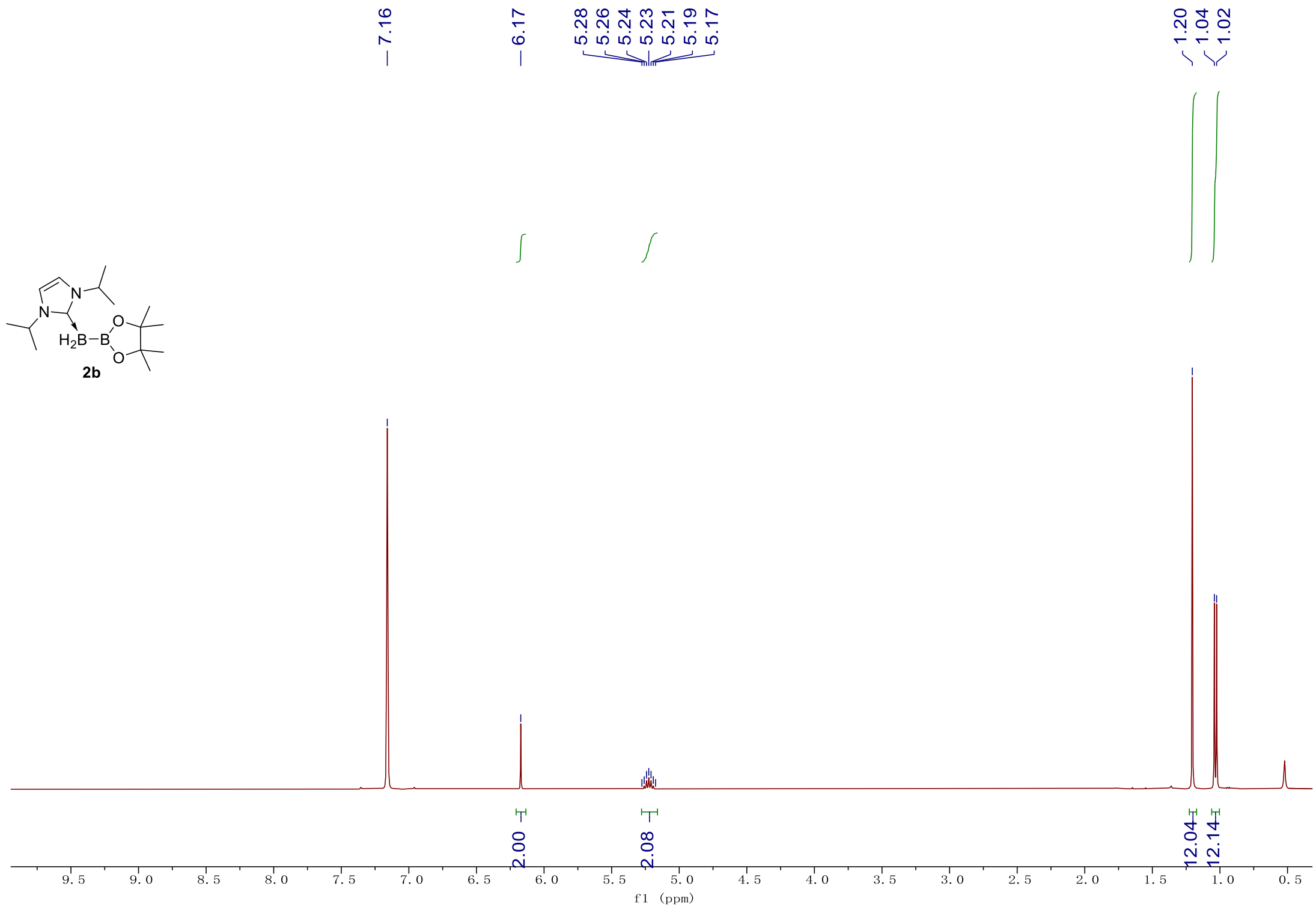

^{1}H NMR (400 MHz, C_6D_6)

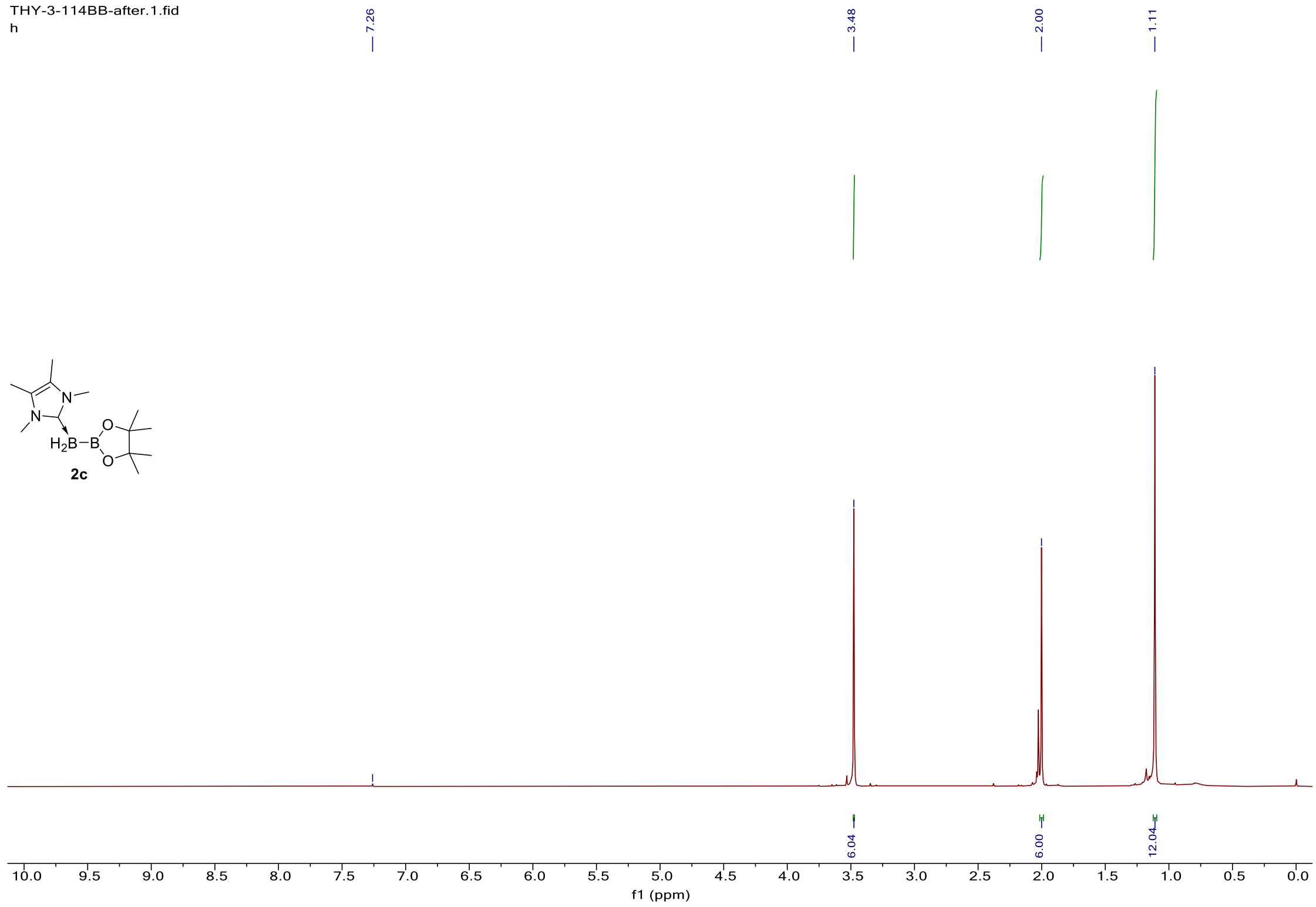

^{1}H NMR (400 MHz, $CDCl_3$)

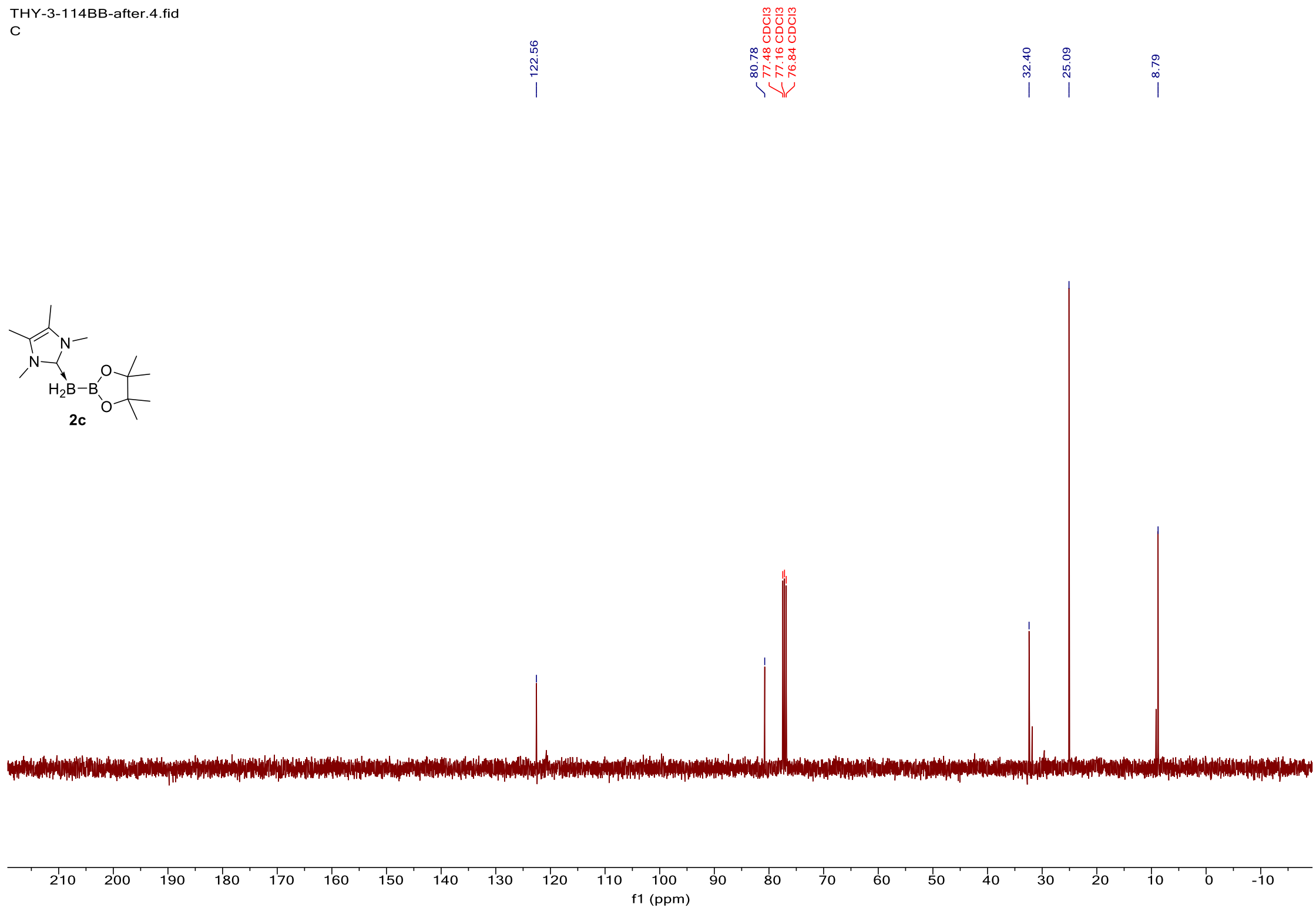

^{13}C NMR (101 MHz, $CDCl_3$)

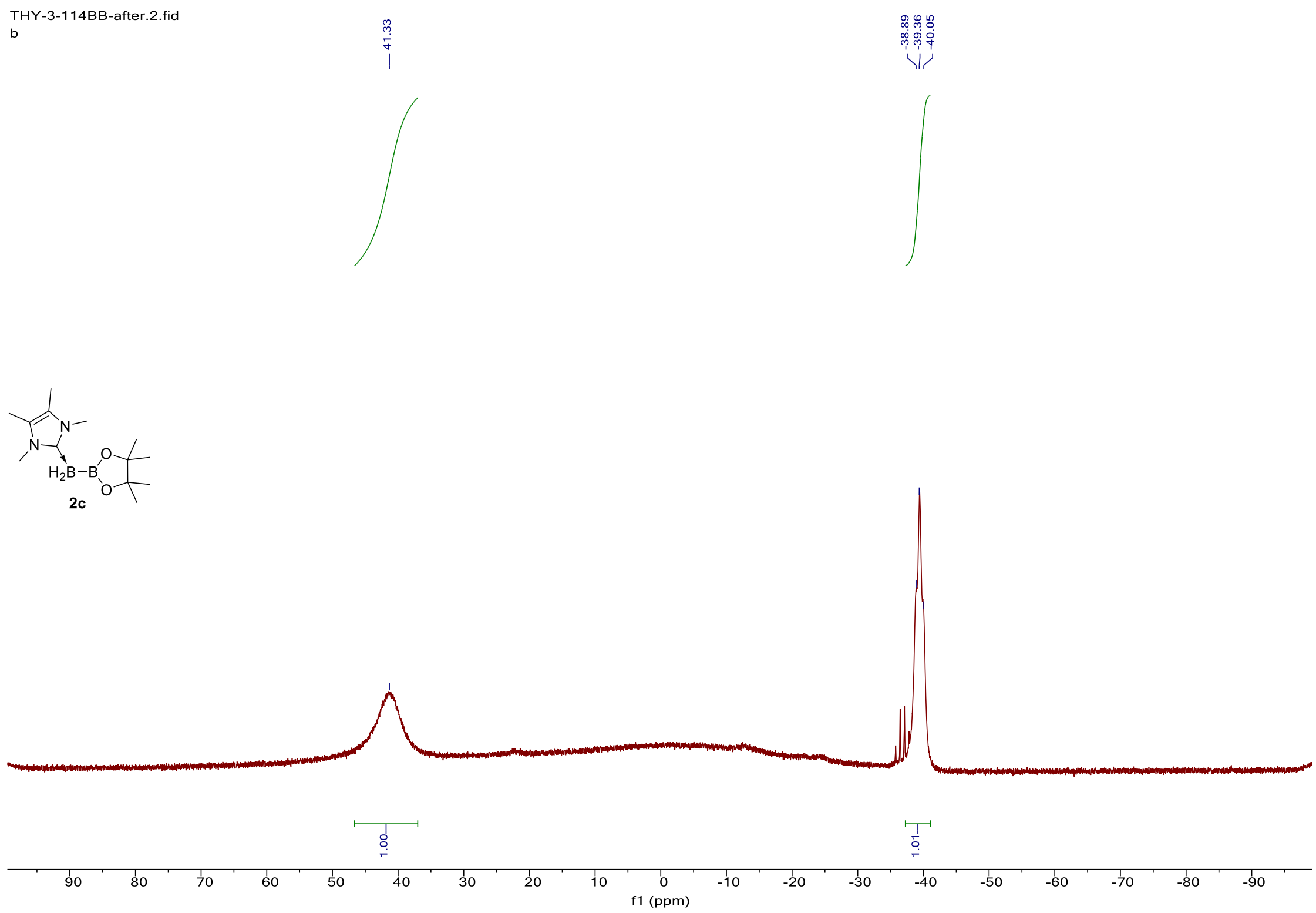

^{11}B NMR (128 MHz, $CDCl_3$)

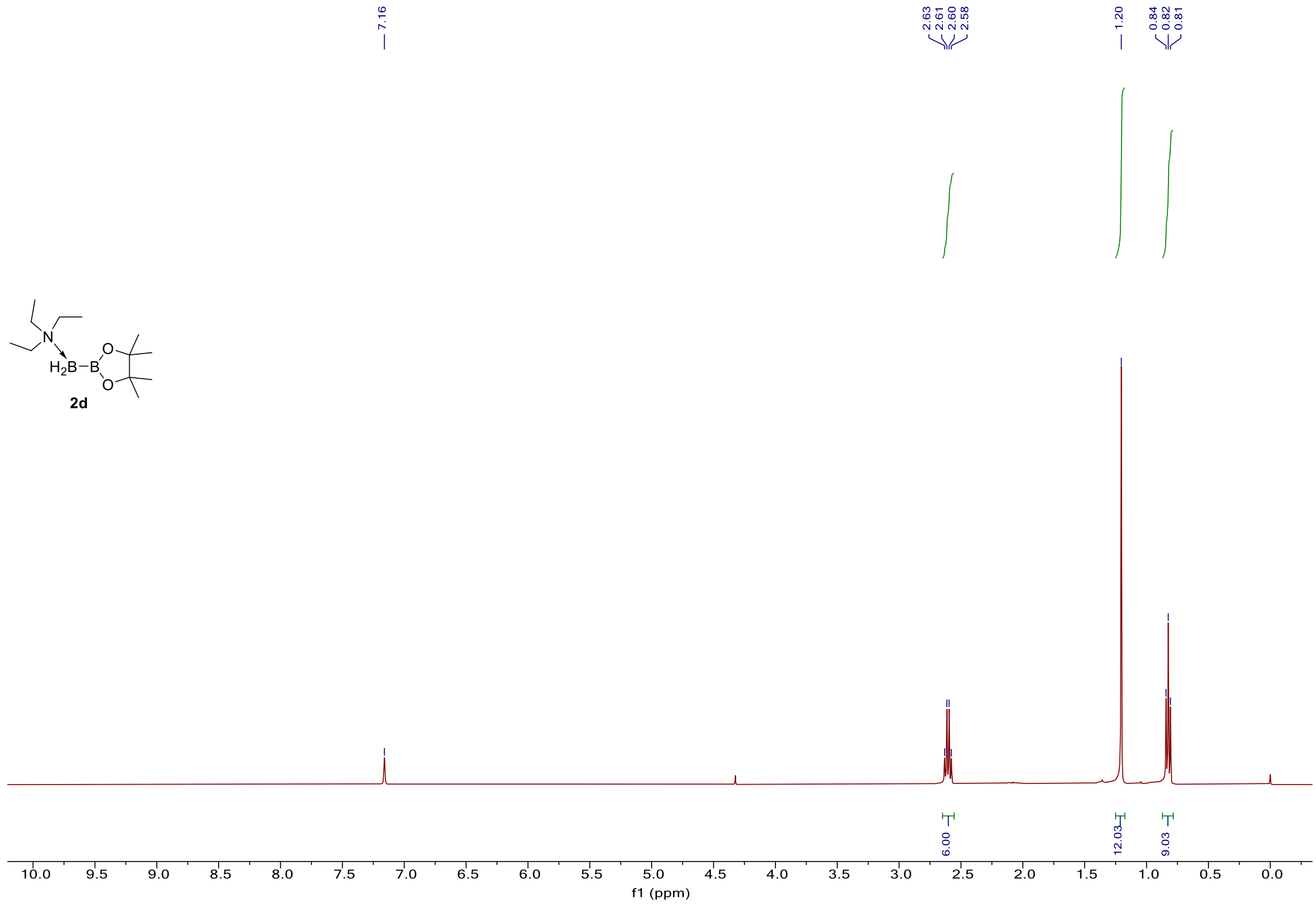

^{1}H NMR (400 MHz, C_6D_6)

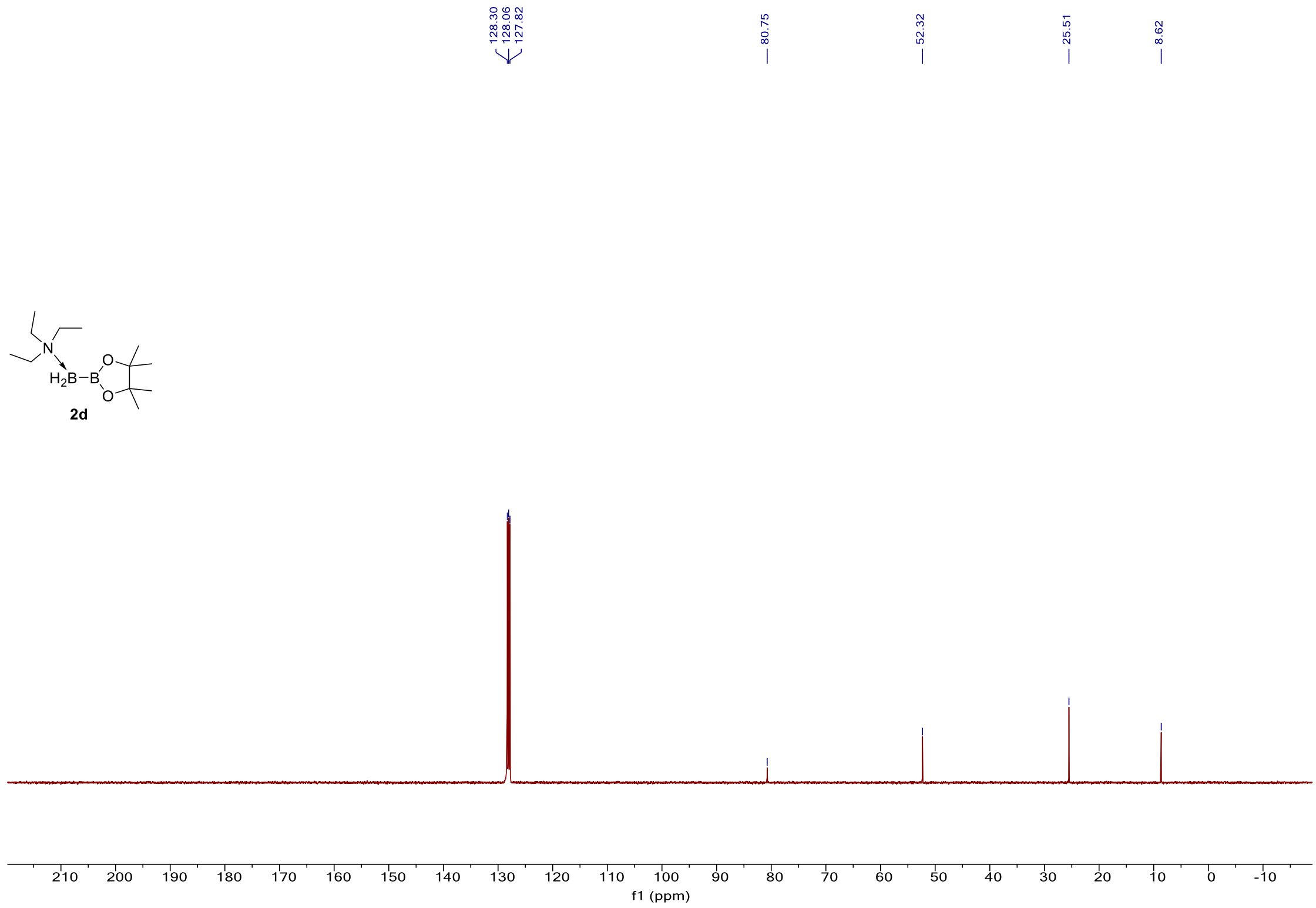

^{13}C NMR (101 MHz, C_6D_6)

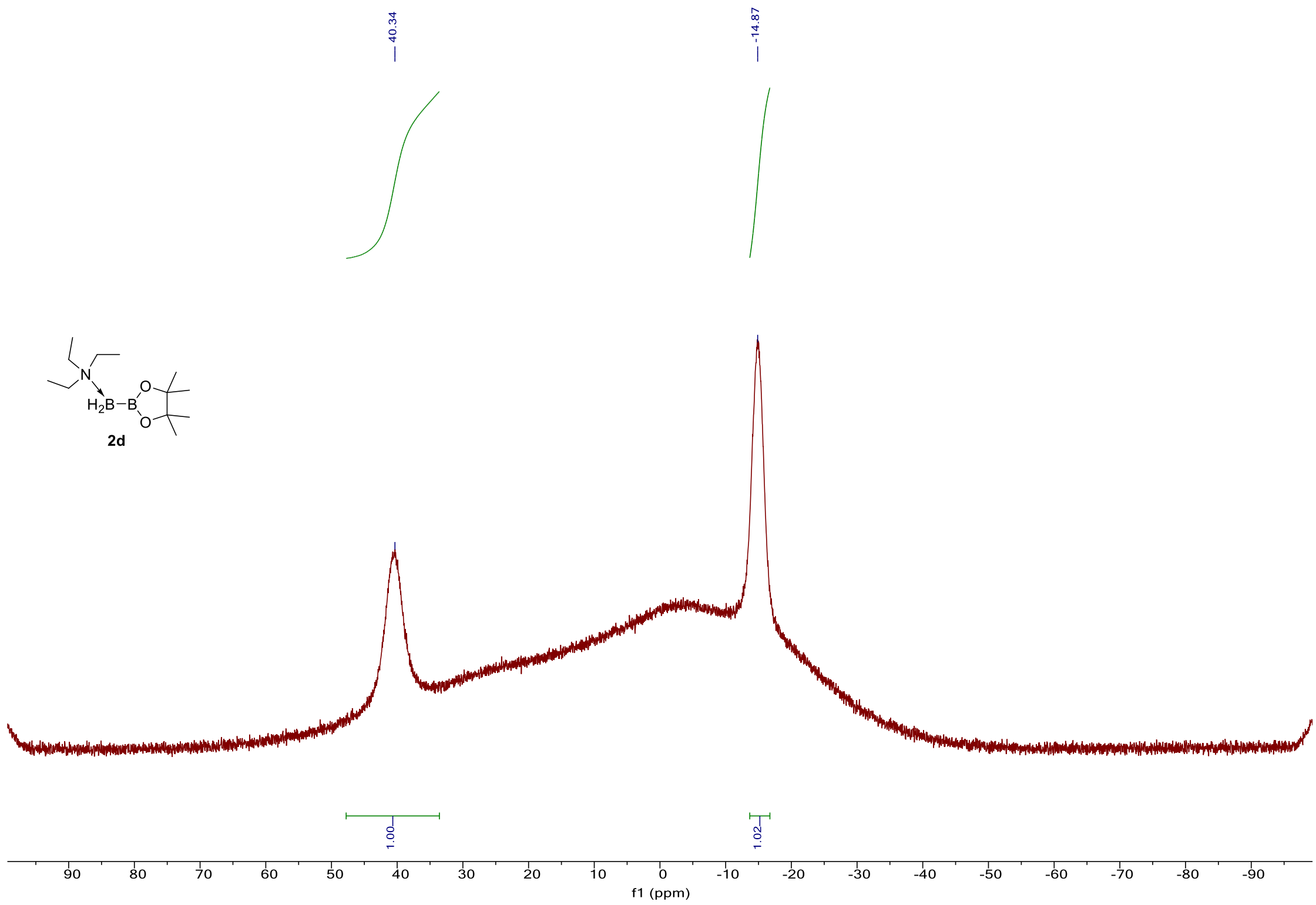

^{11}B NMR (128 MHz, C_6D_6)

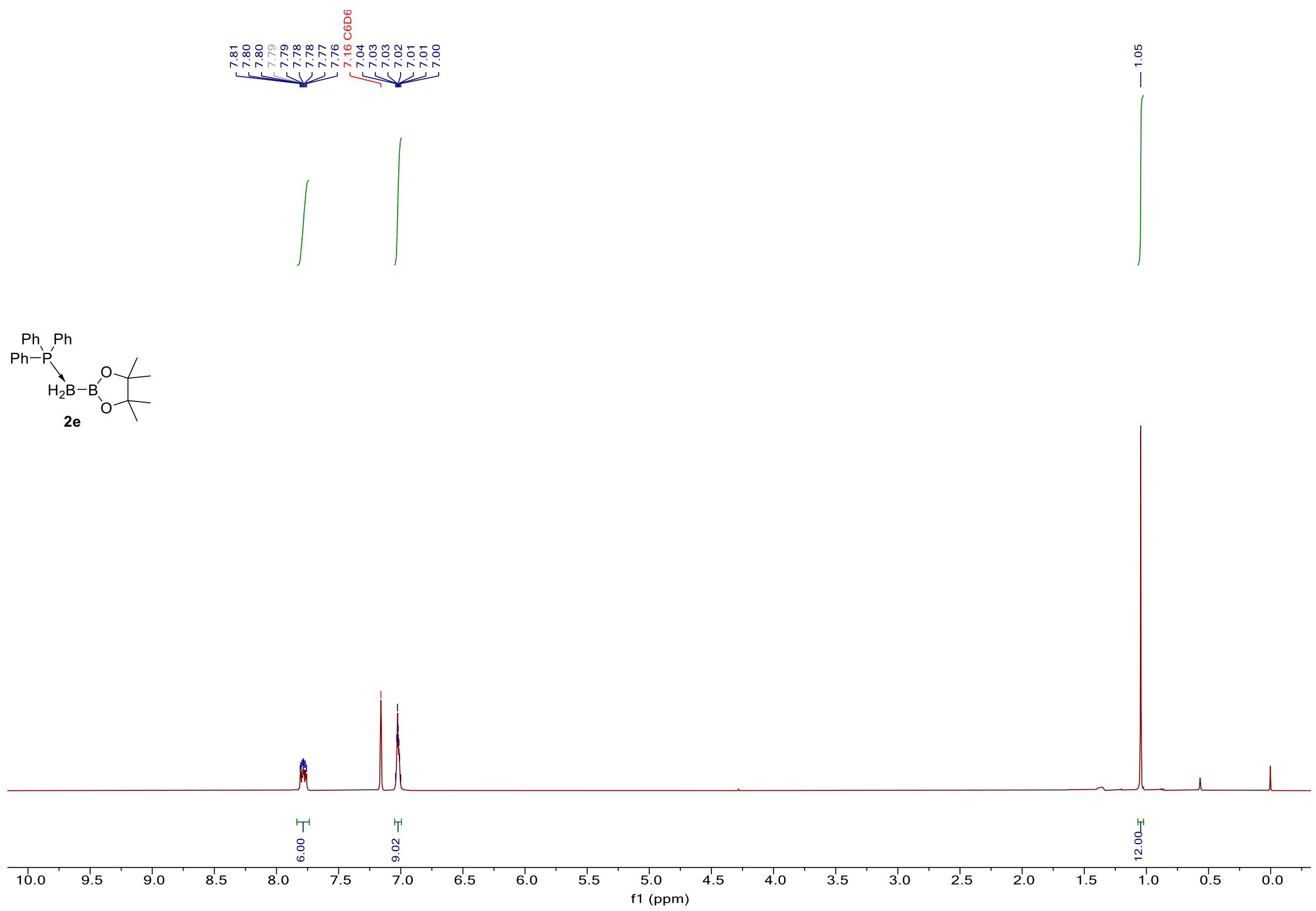

^{1}H NMR (400 MHz, C_6D_6)

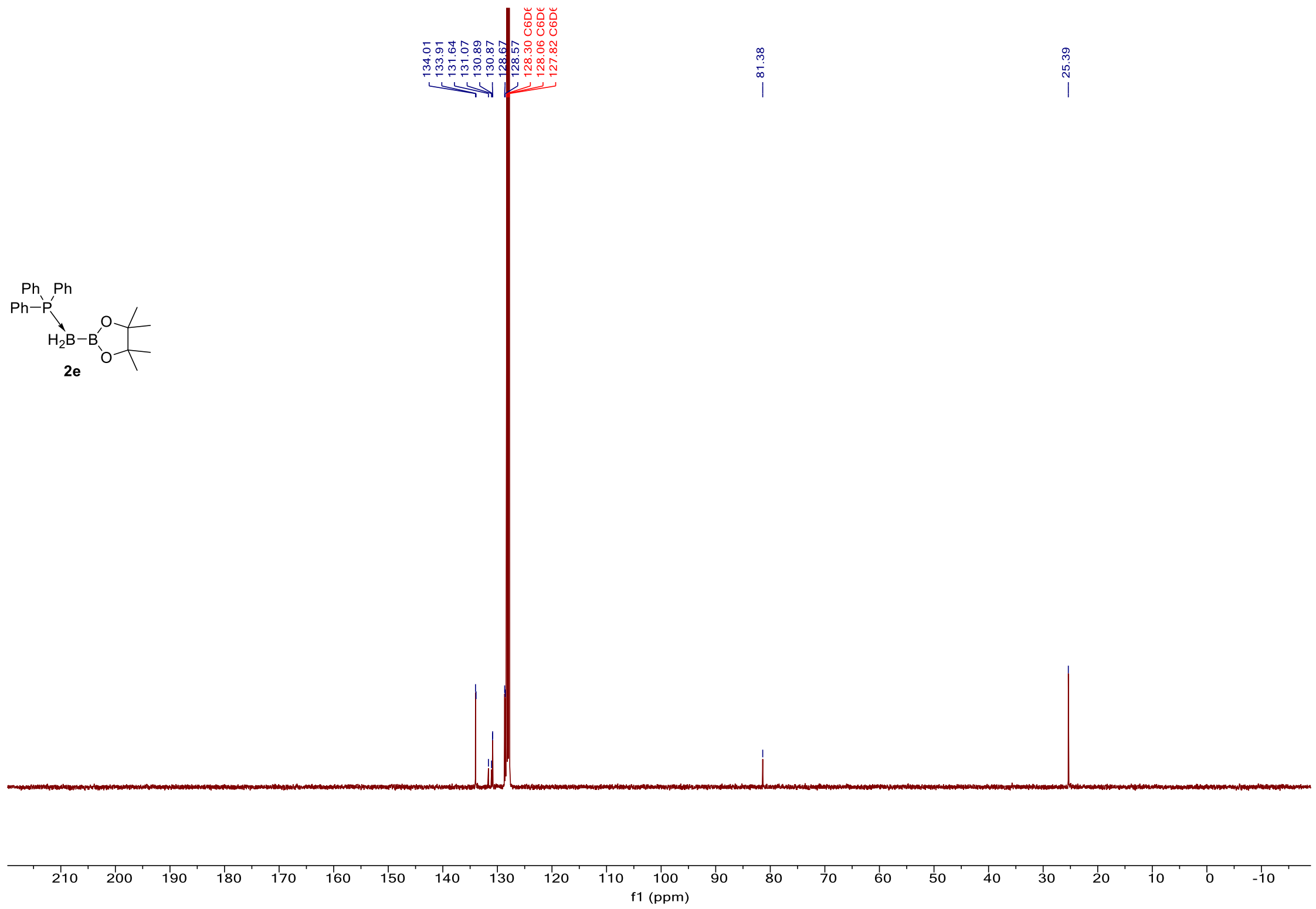

^{13}C NMR (101 MHz, C_6D_6)

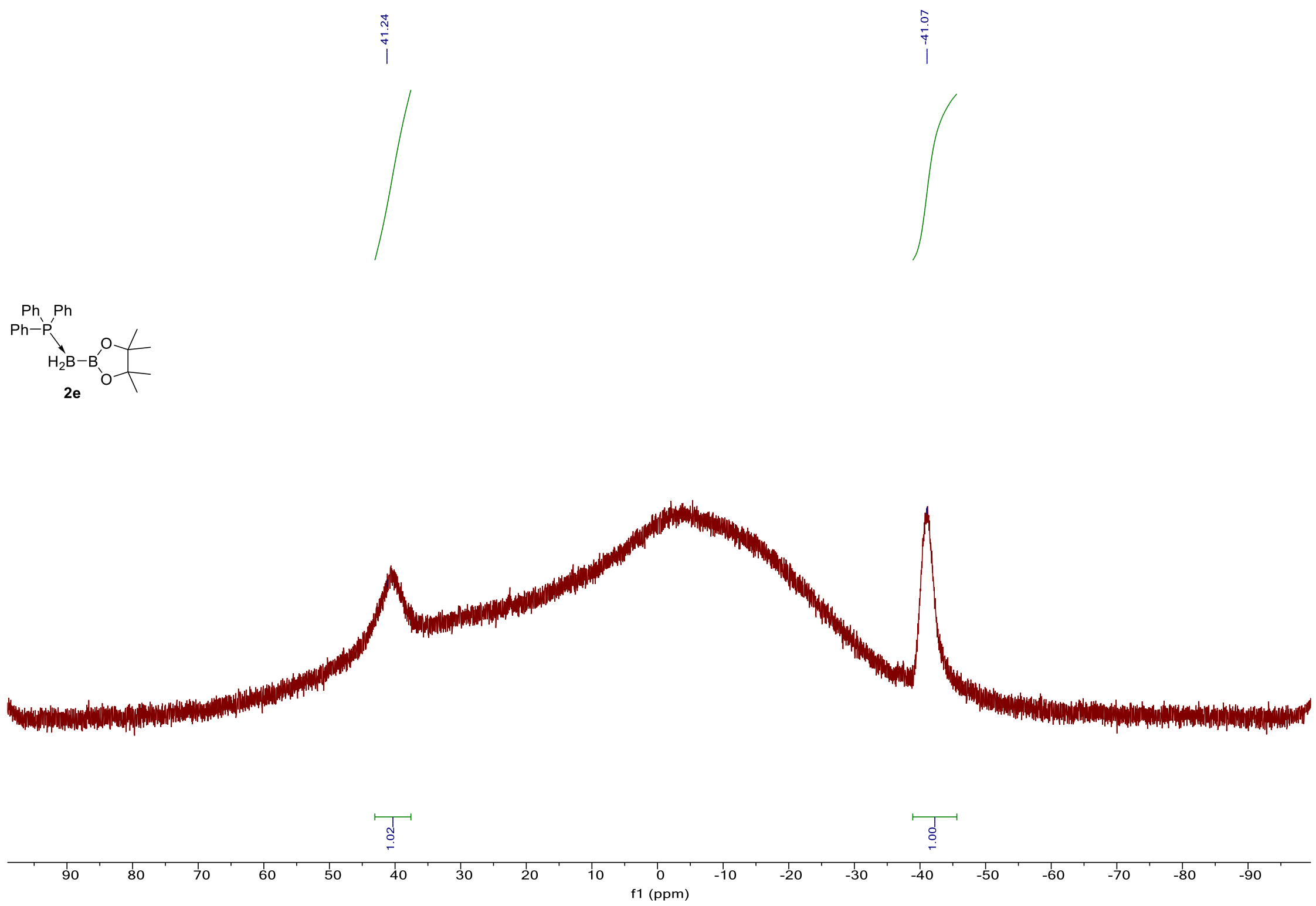

^{11}B NMR (128 MHz, C_6D_6)

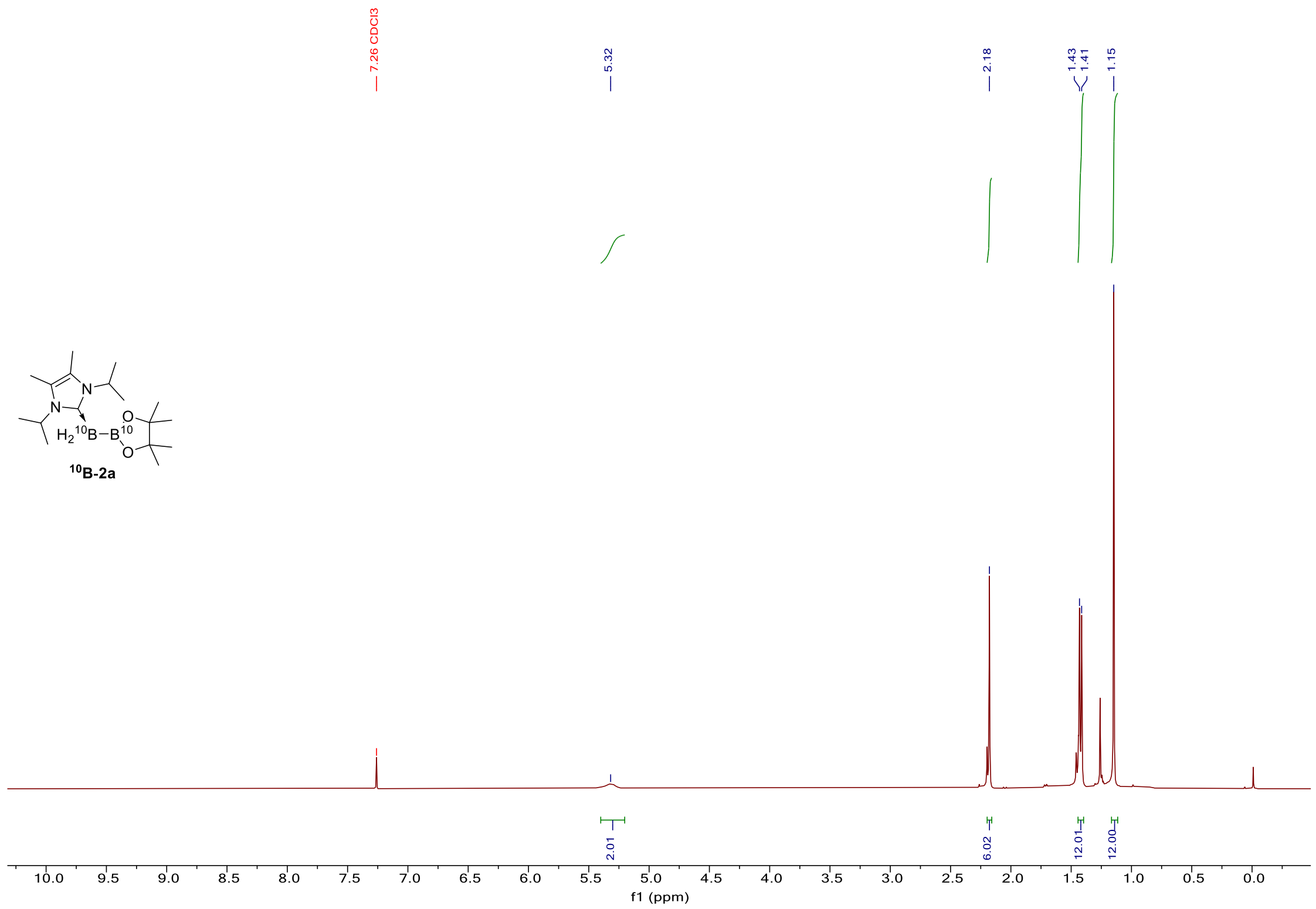

^{1}H NMR (400 MHz, $CDCl_3$)

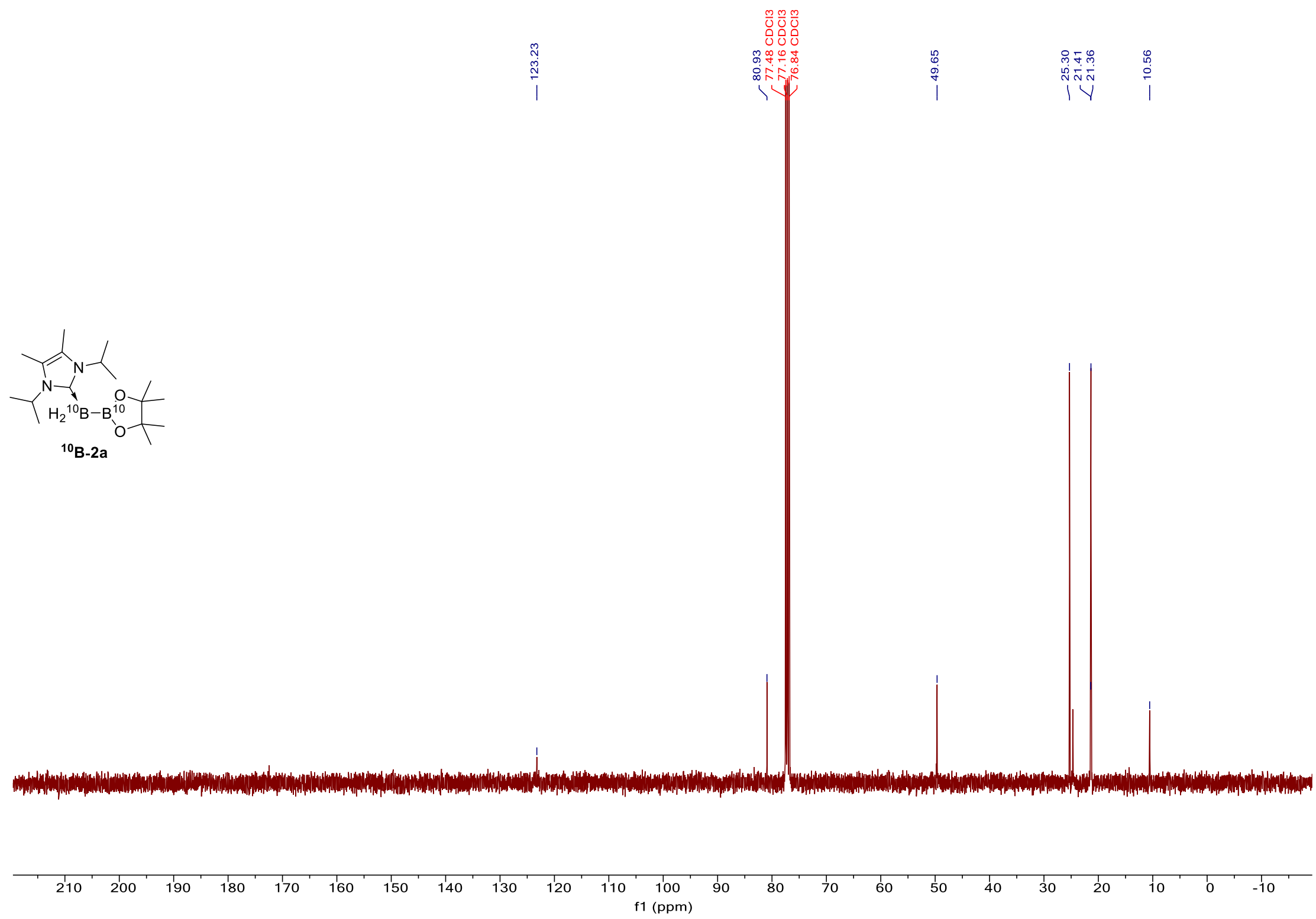

^{13}C NMR (101 MHz, $CDCl_3$)

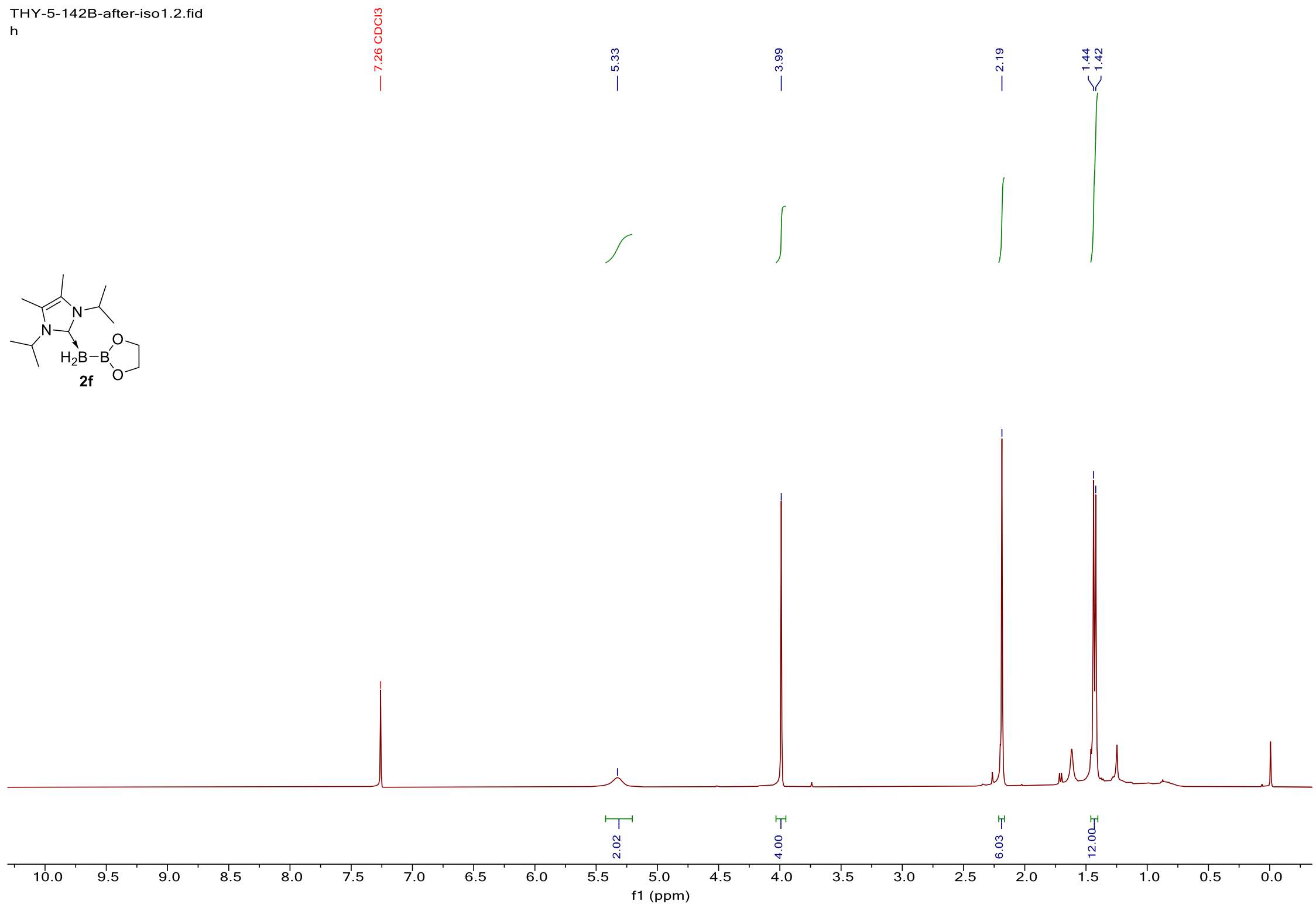

^{1}H NMR (500 MHz, $CDCl_3$)

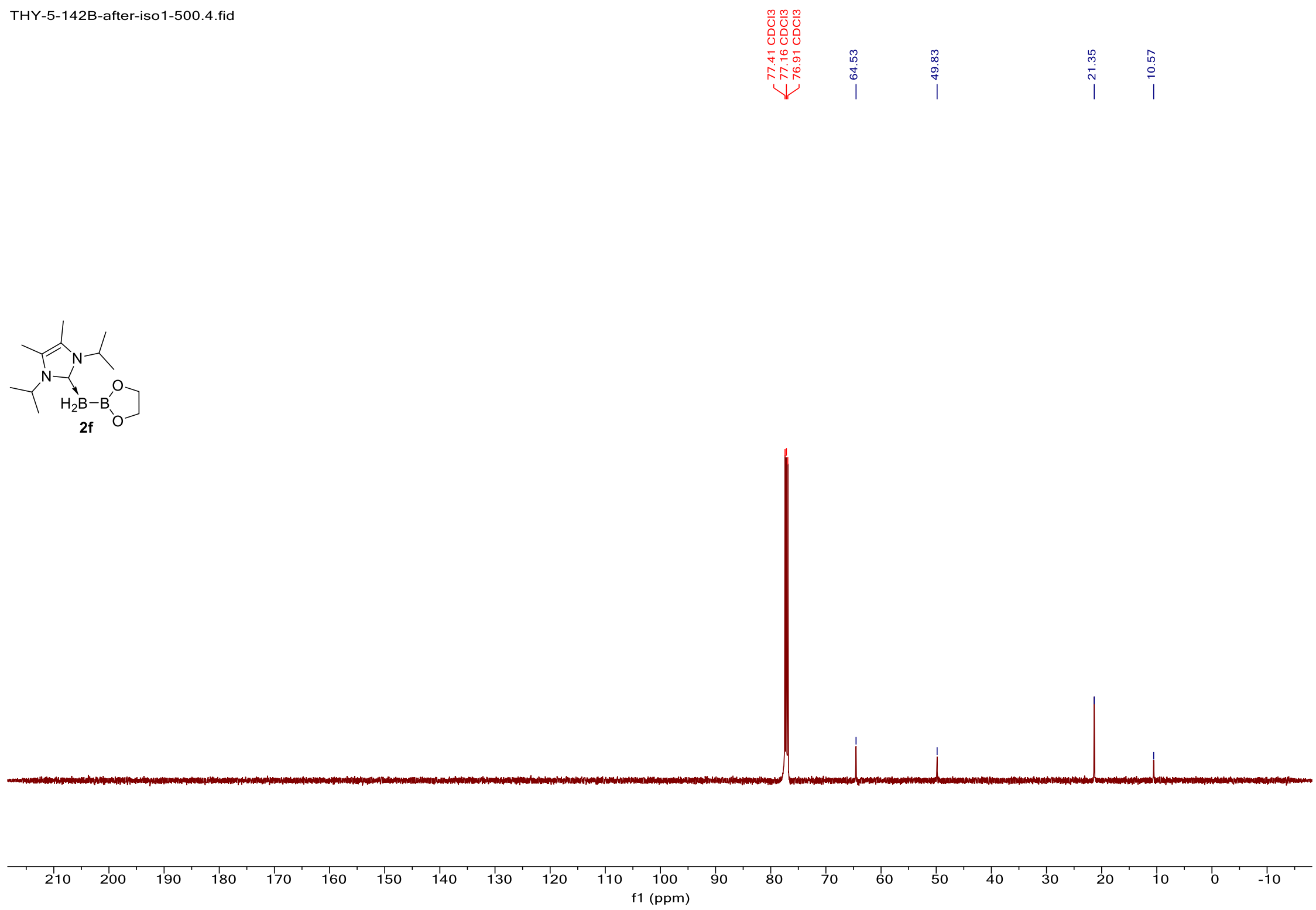

^{13}C NMR (126 MHz, CDCl$_3$)

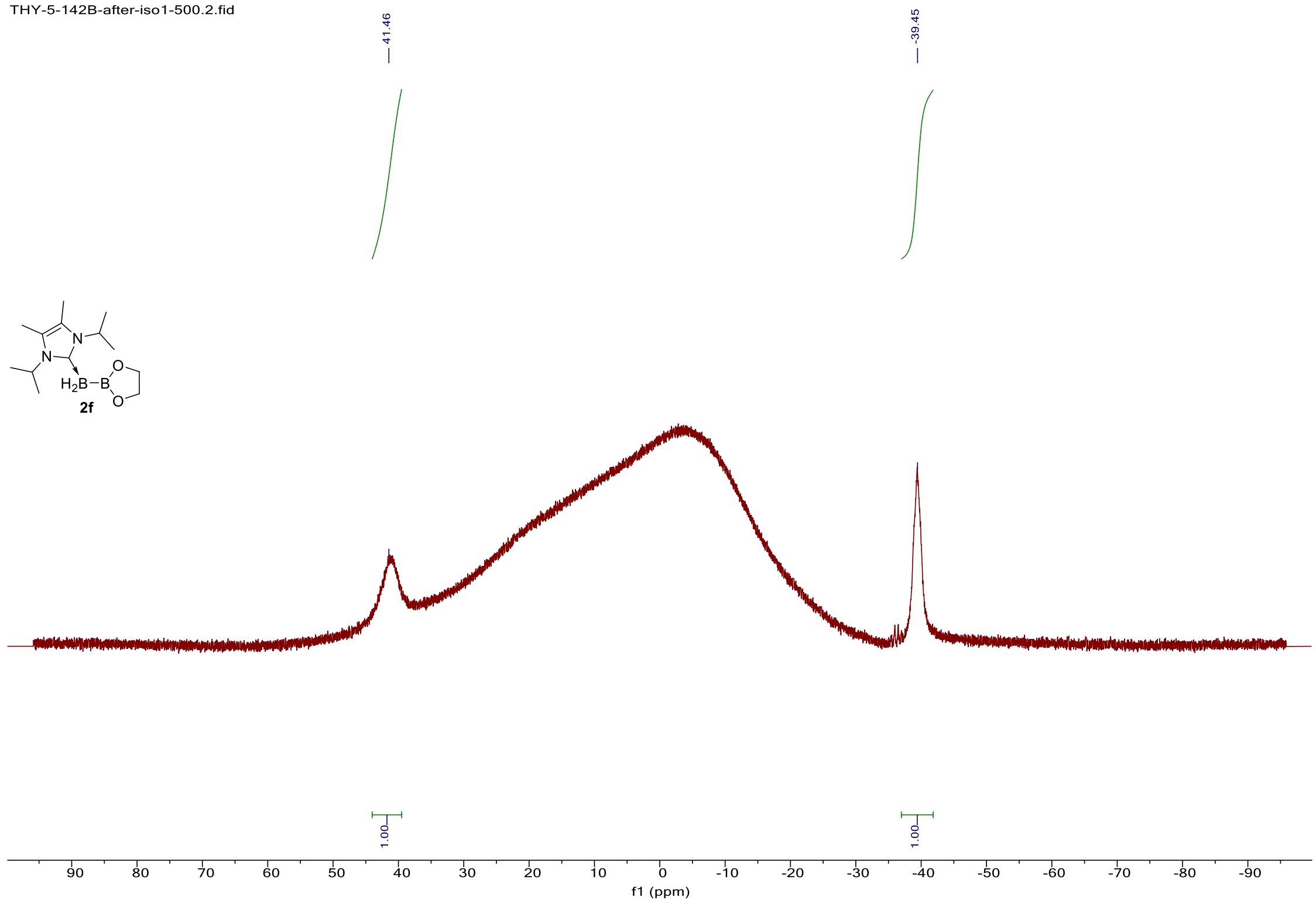

^{11}B NMR (160 MHz, CDCl$_3$)

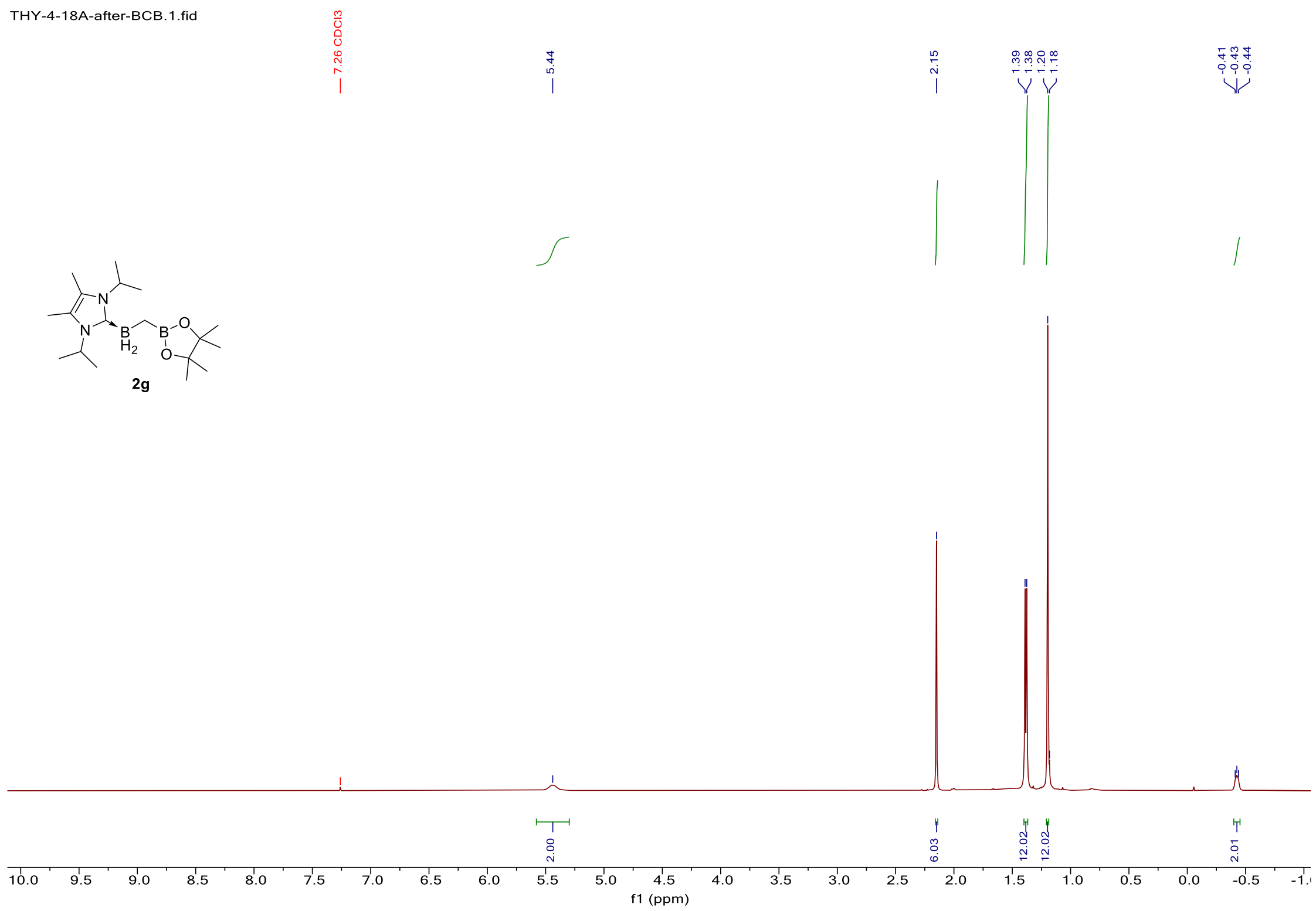

^{1}H NMR (500 MHz, $CDCl_3$)

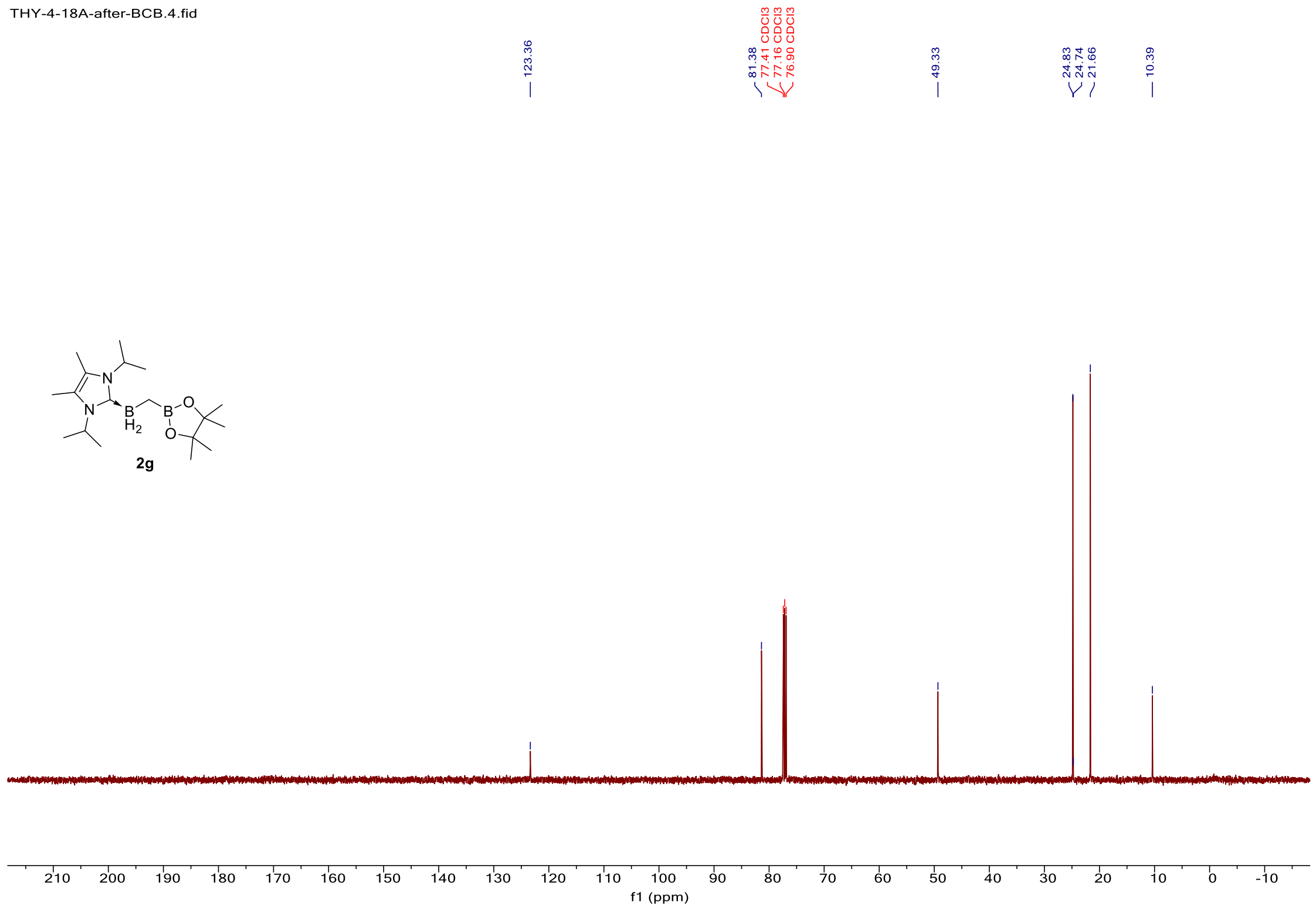

^{13}C NMR (126 MHz, $CDCl_3$)

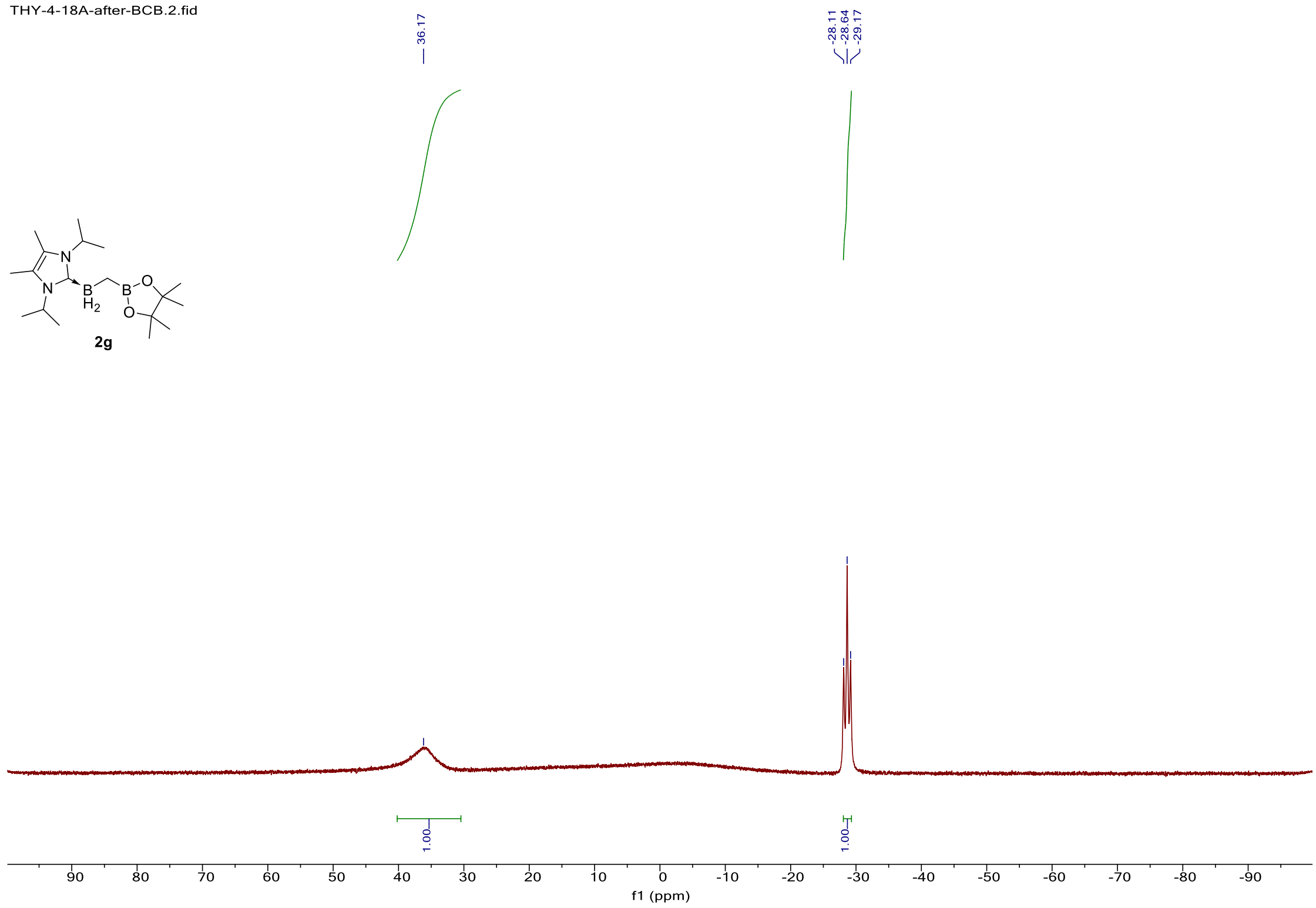

2g

^{11}B NMR (160 MHz, CDCl3)

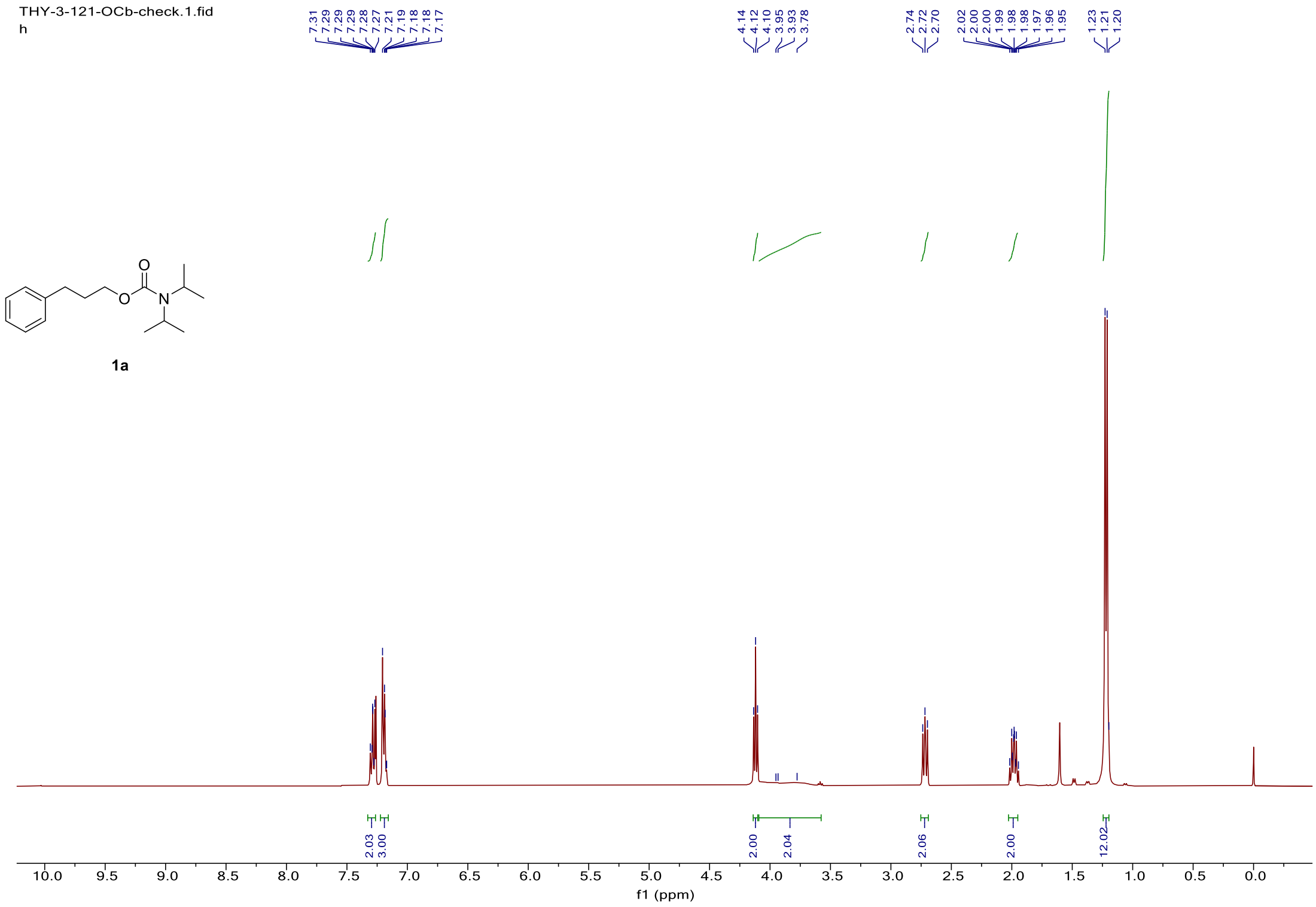

1a

^{1}H NMR (400 MHz, CDCl3)

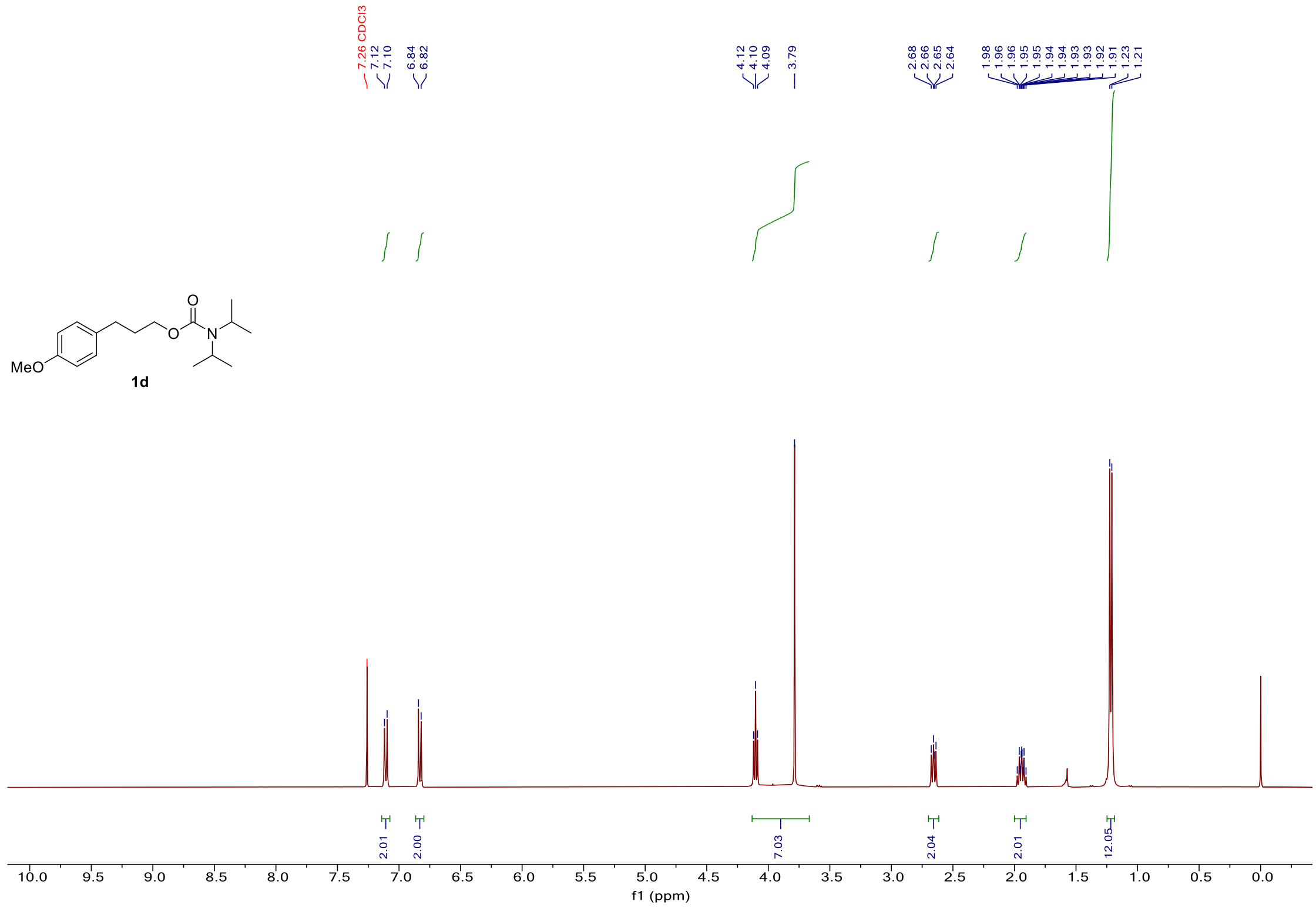

^{1}H NMR (400 MHz, $CDCl_3$)

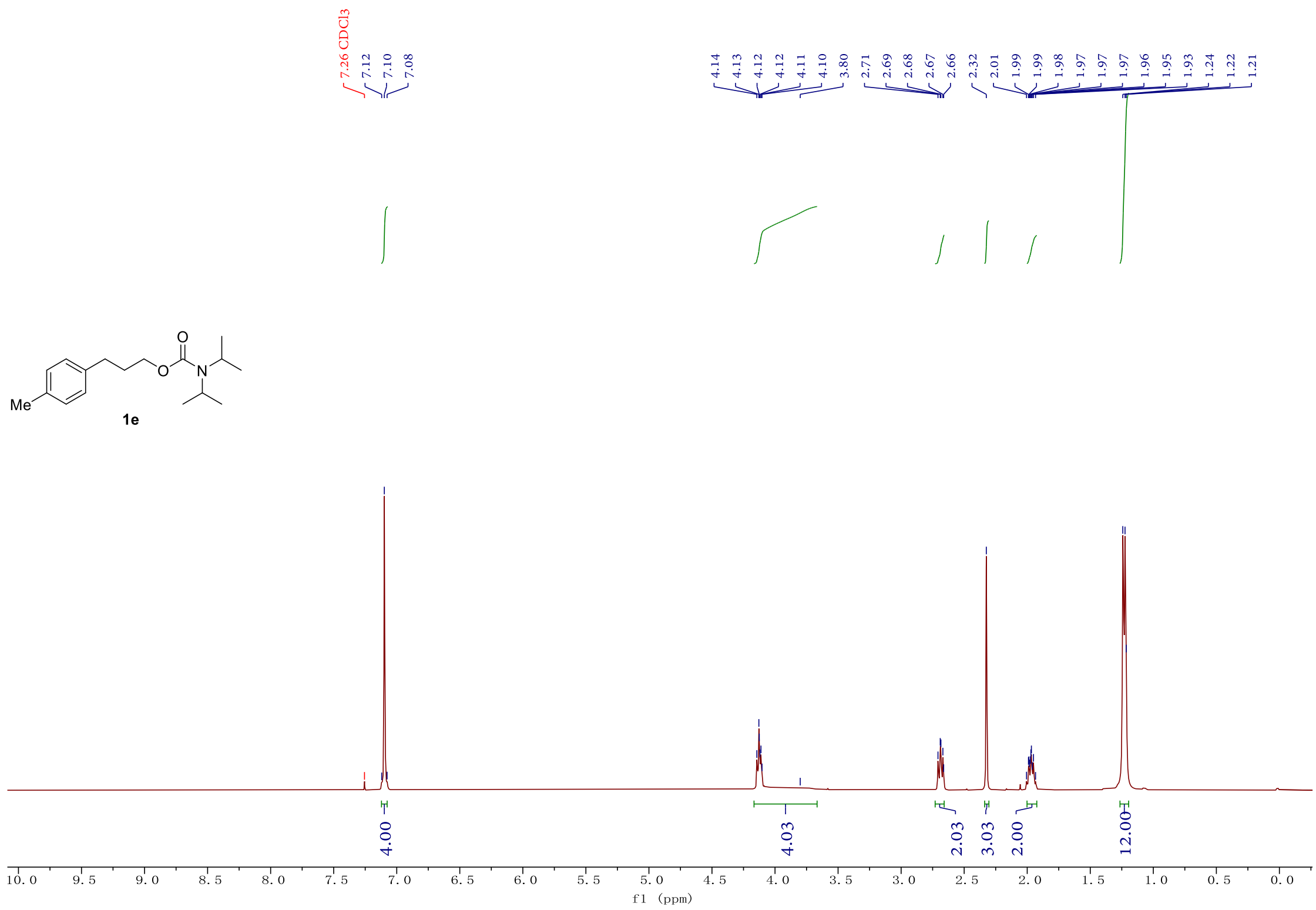

^{1}H NMR (400 MHz, $CDCl_3$)

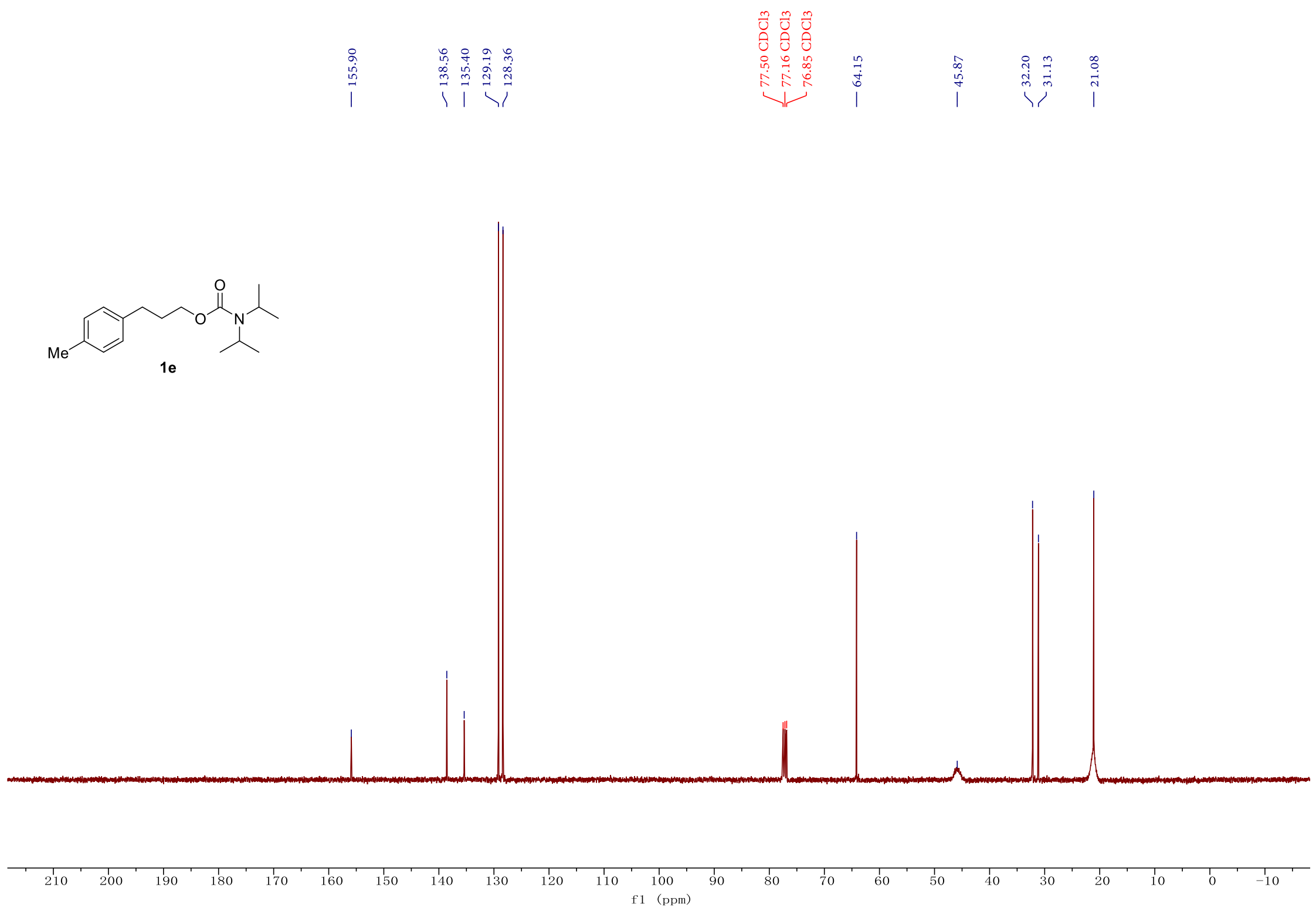

^{13}C NMR (101 MHz, $CDCl_3$)

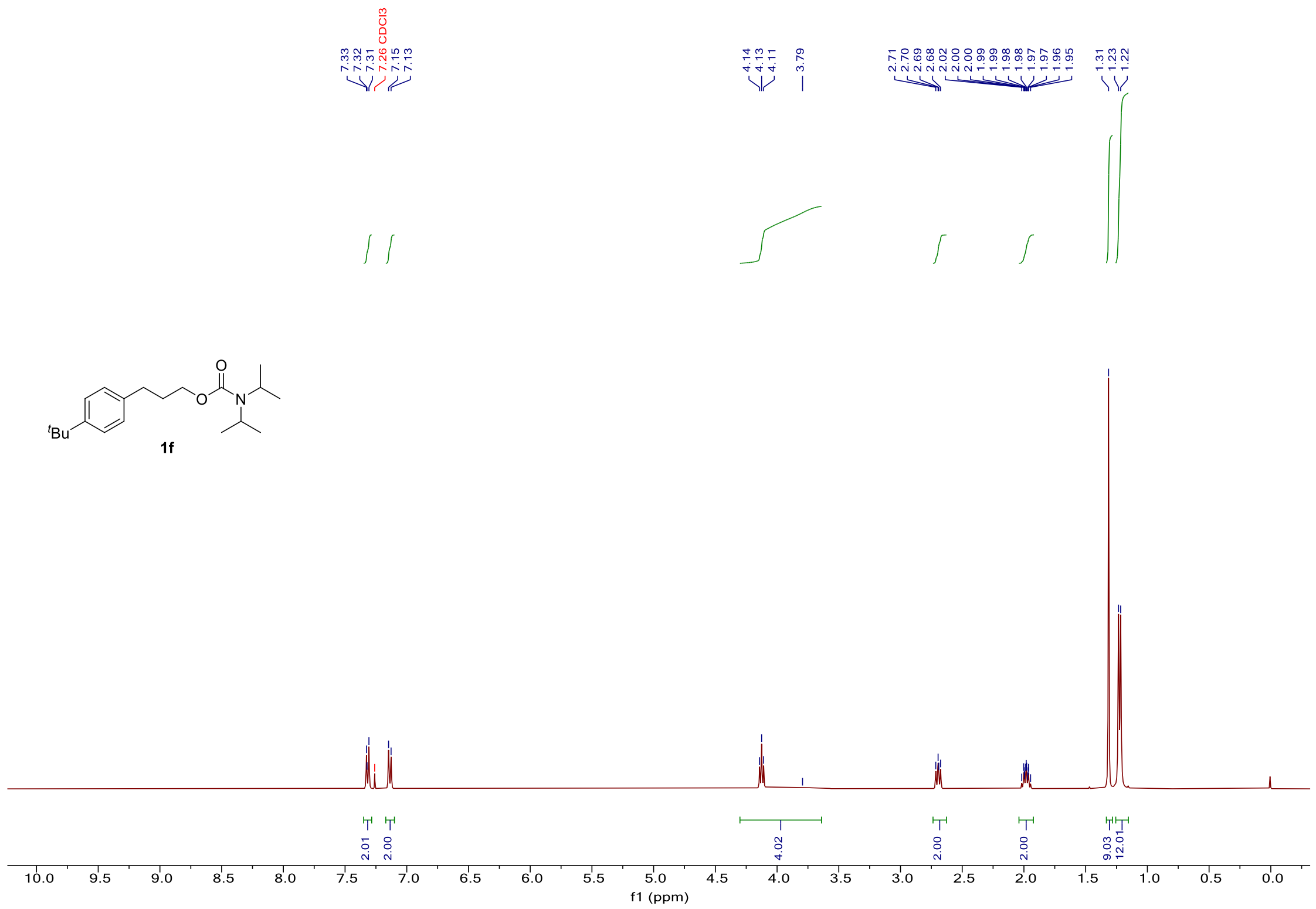

^{1}H NMR (400 MHz, $CDCl_3$)

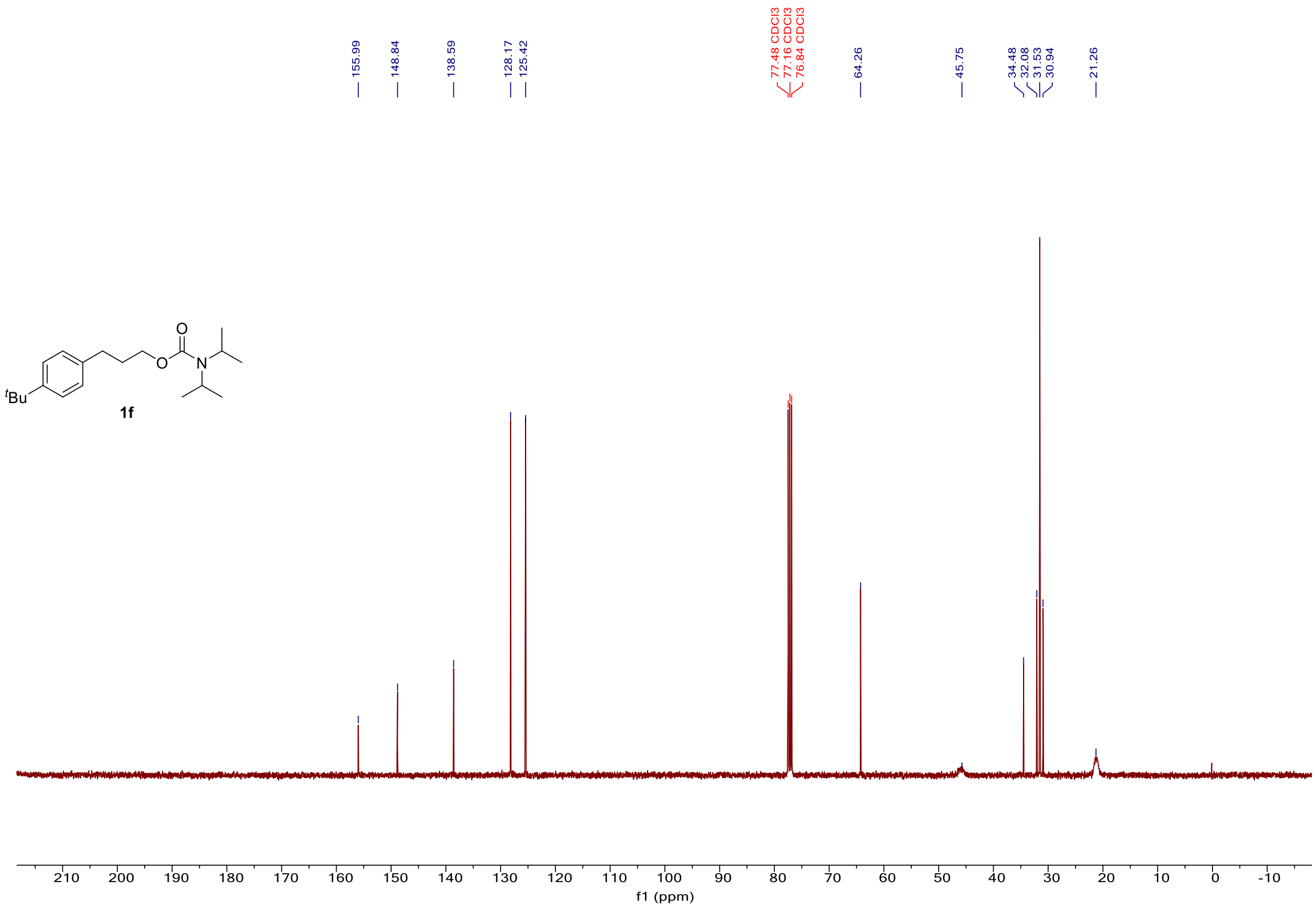

^{13}C NMR (101 MHz, $CDCl_3$)

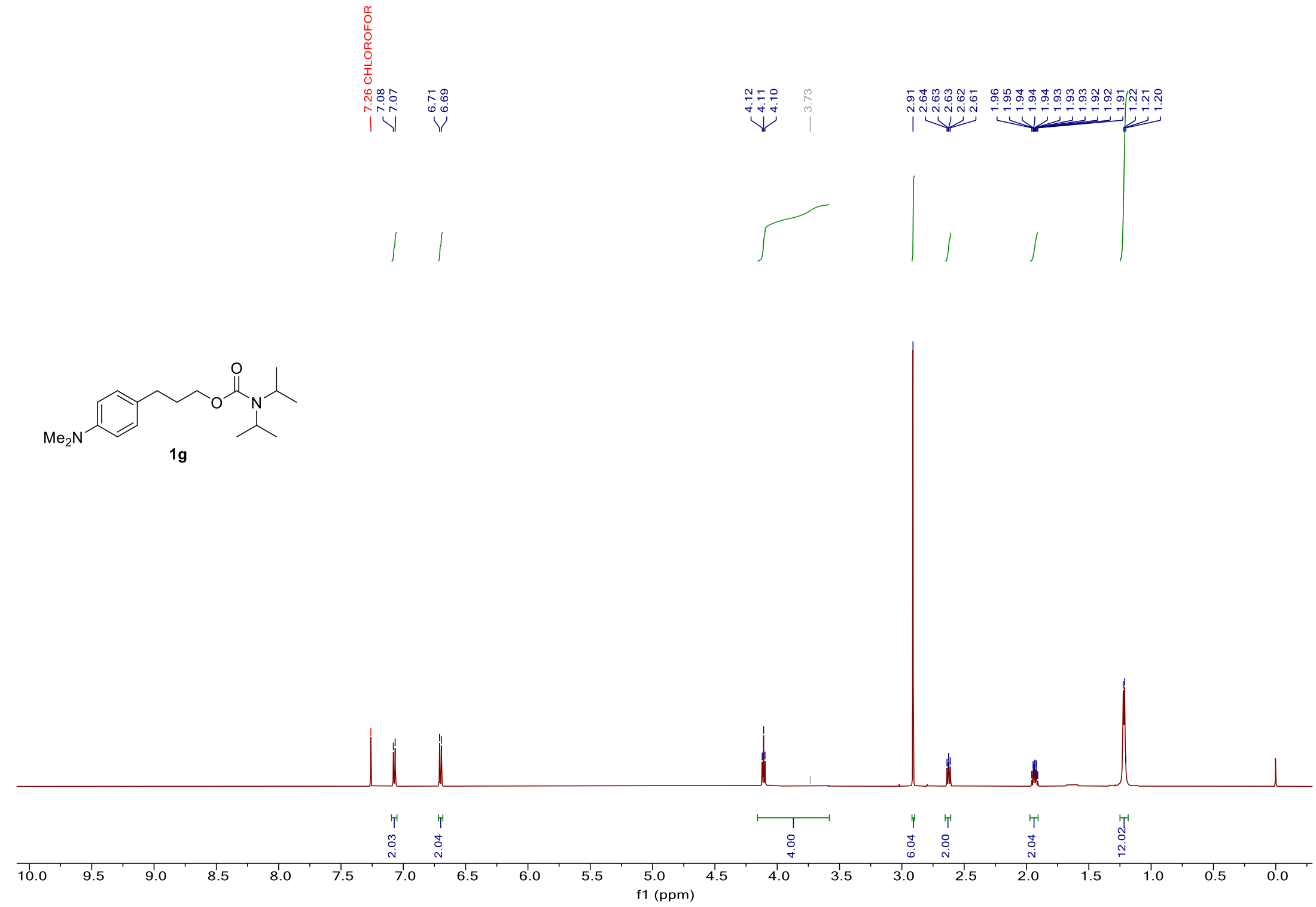

^{1}H NMR (600 MHz, $CDCl_3$)

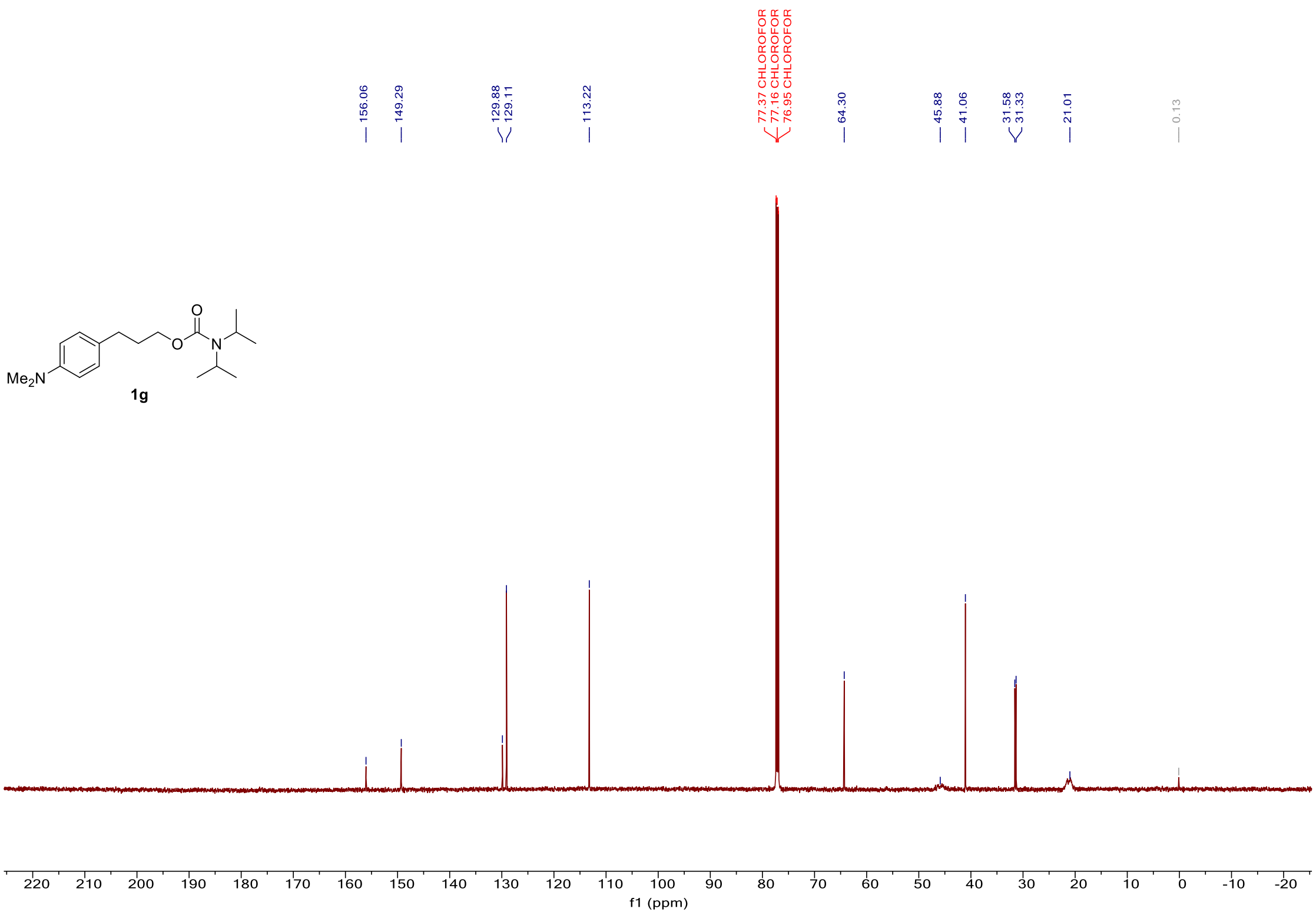

^{13}C NMR (151 MHz, CDCl$_3$)

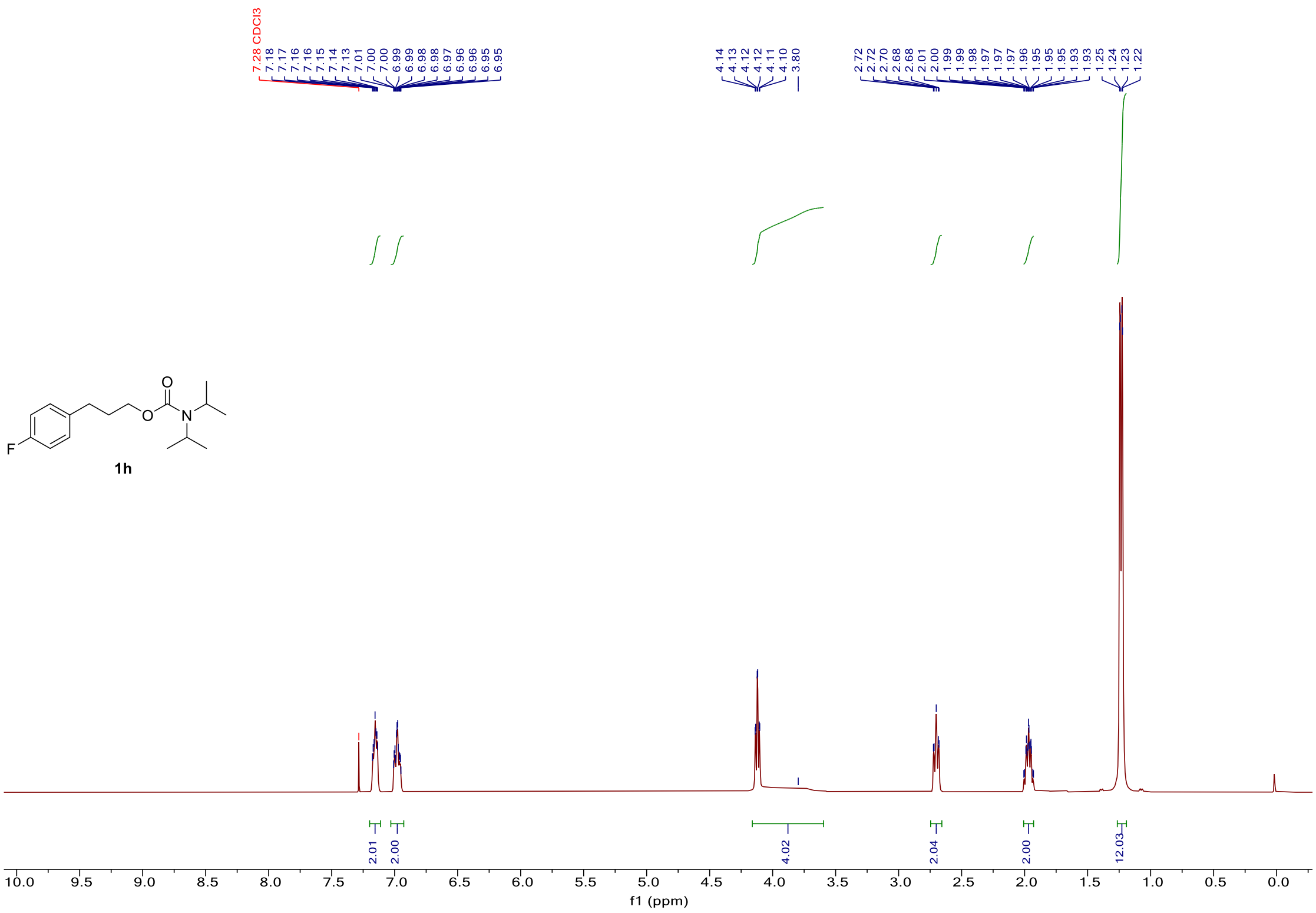

^{1}H NMR (400 MHz, CDCl$_3$)

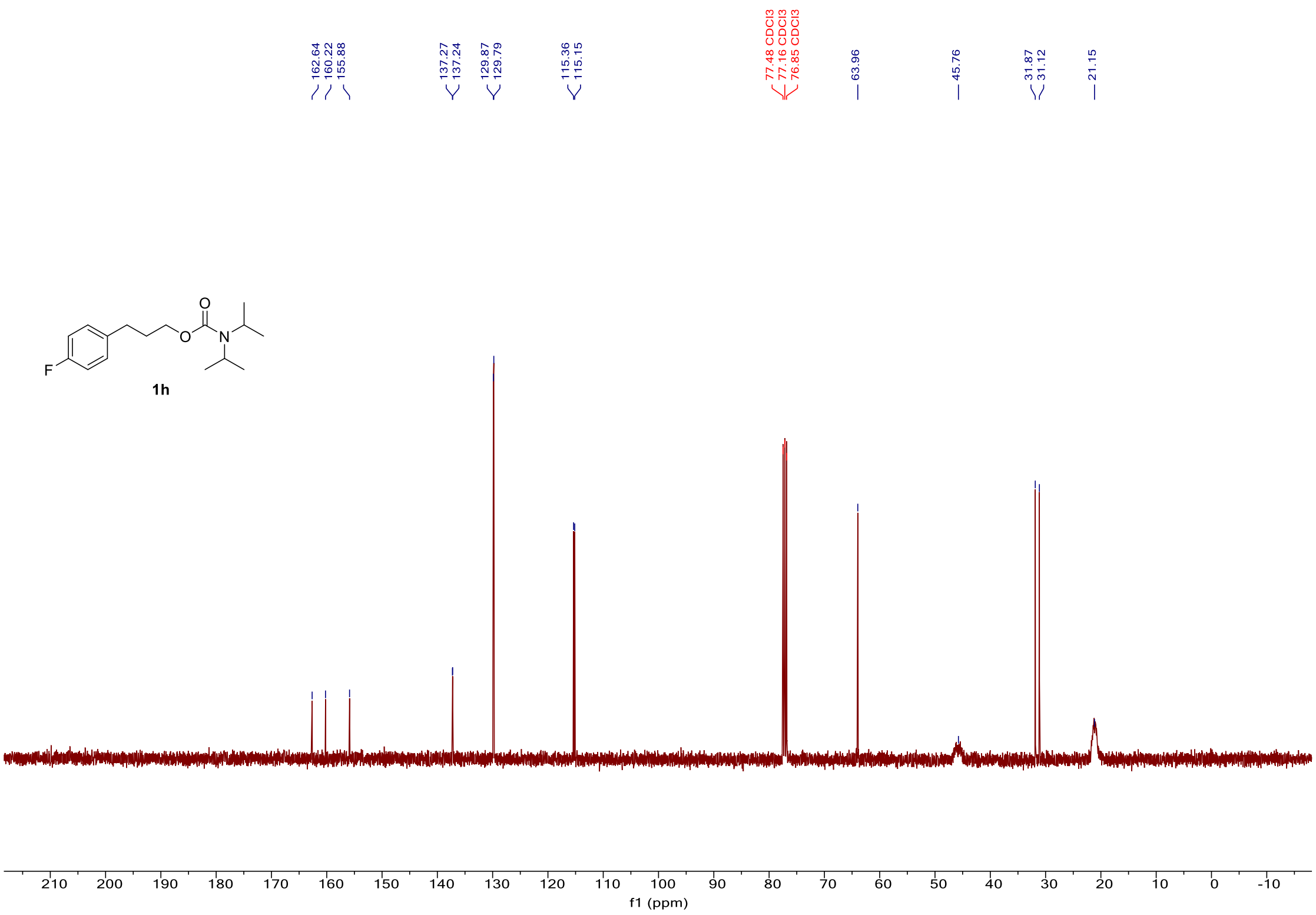

^{13}C NMR (101 MHz, $CDCl_3$)

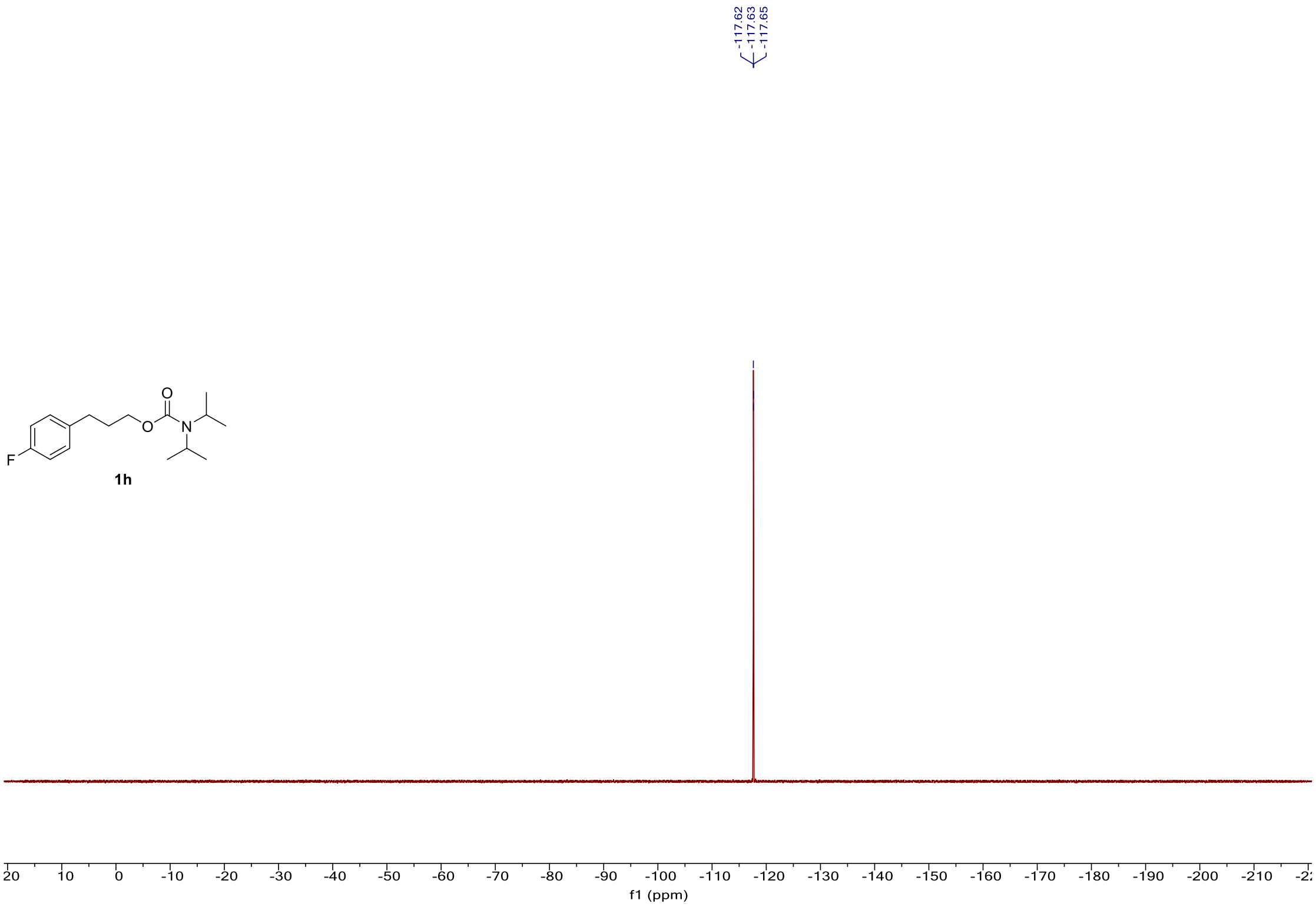

^{19}F NMR (377 MHz, $CDCl_3$)

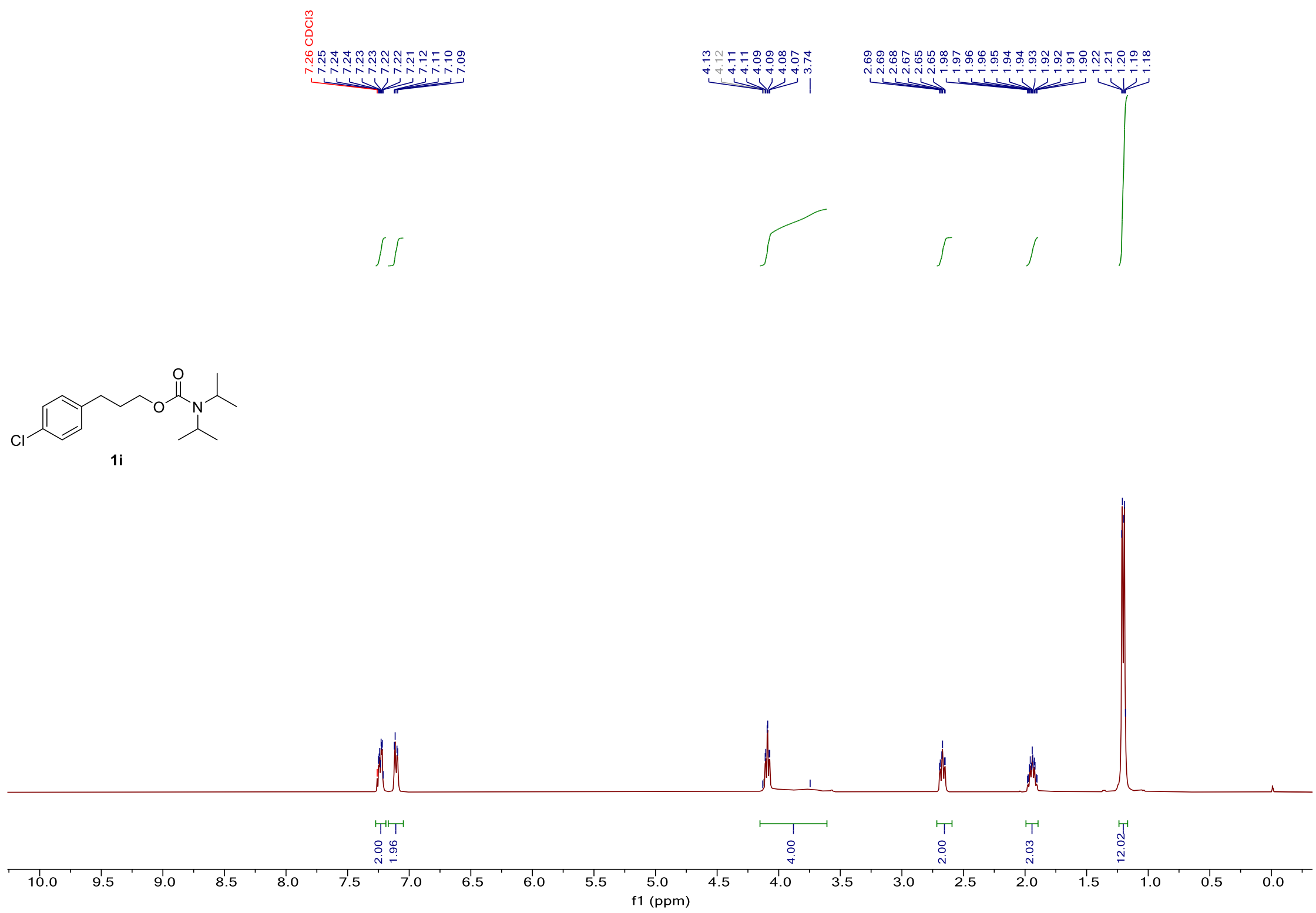

^{1}H NMR (400 MHz, $CDCl_3$)

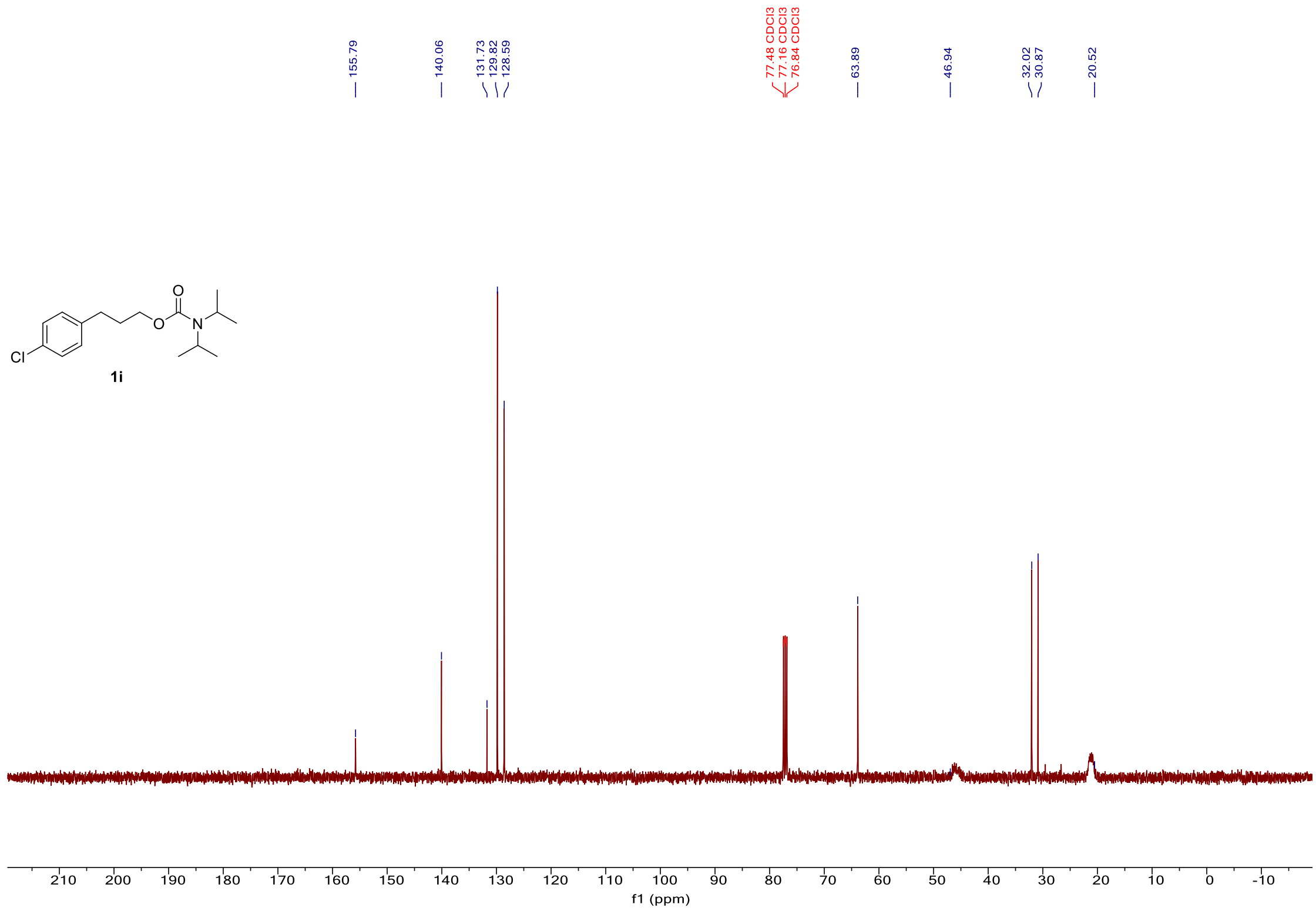

^{13}C NMR (101 MHz, $CDCl_3$)

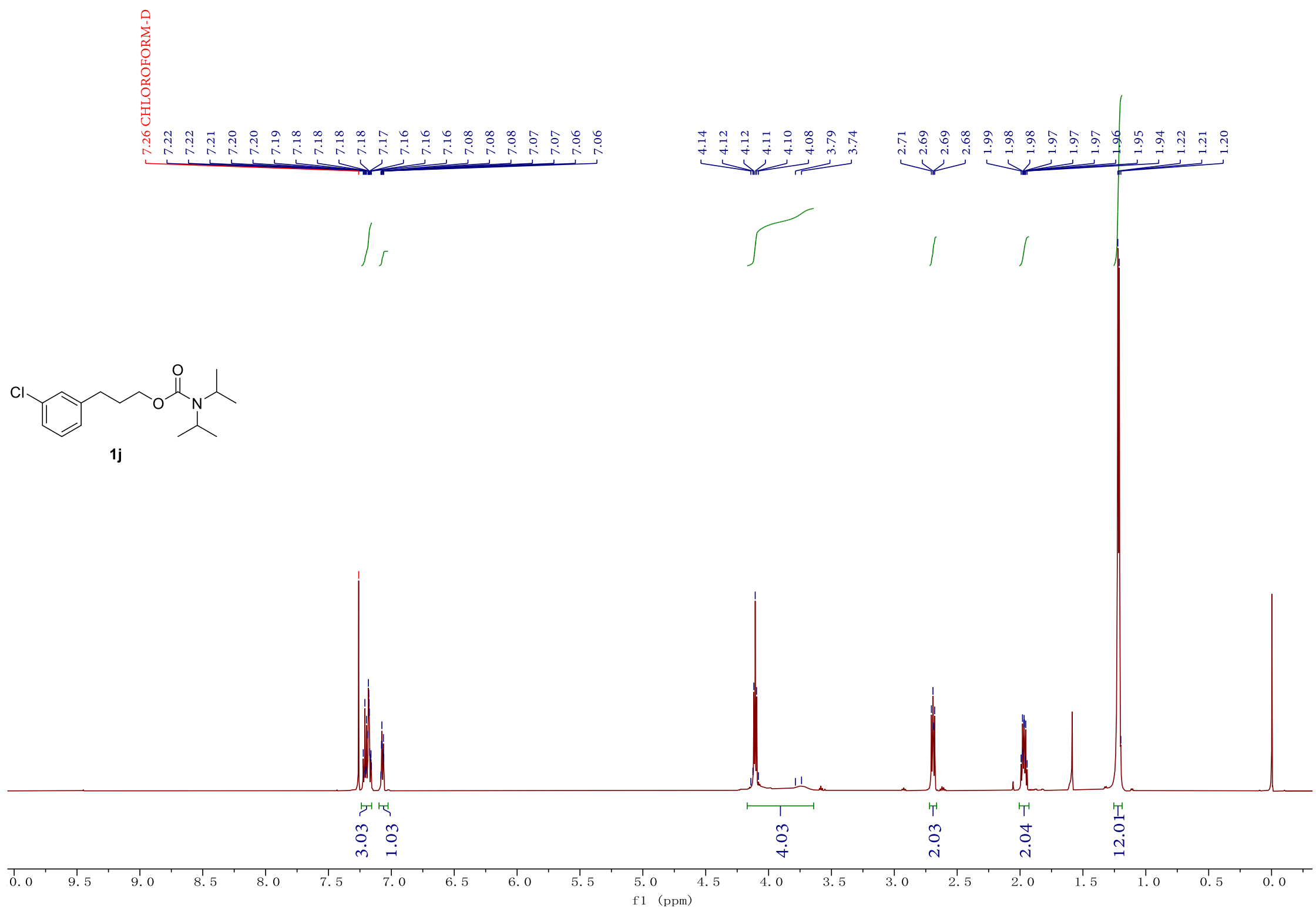

^{1}H NMR (400 MHz, $CDCl_3$)

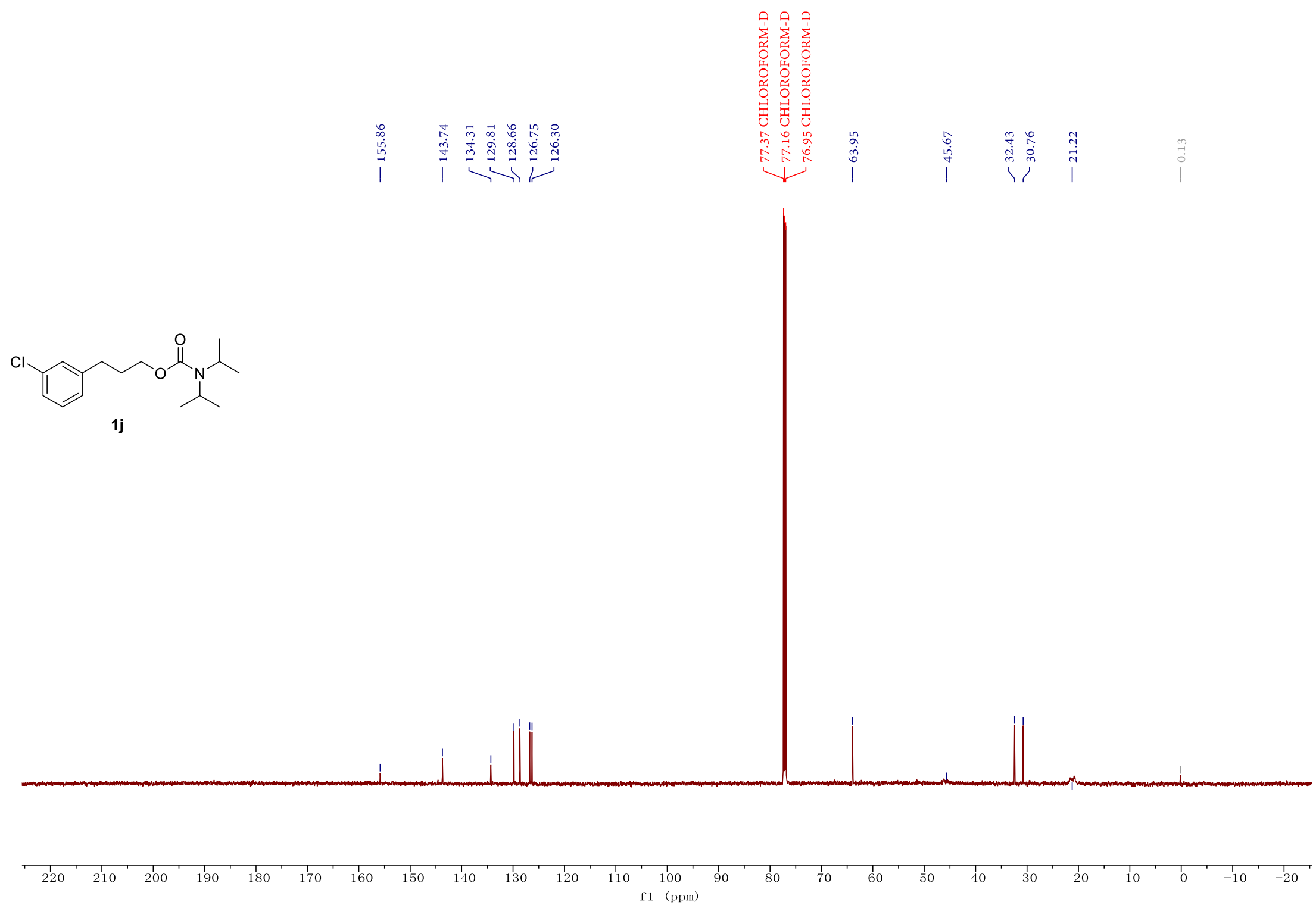

^{13}C NMR (101 MHz, $CDCl_3$)

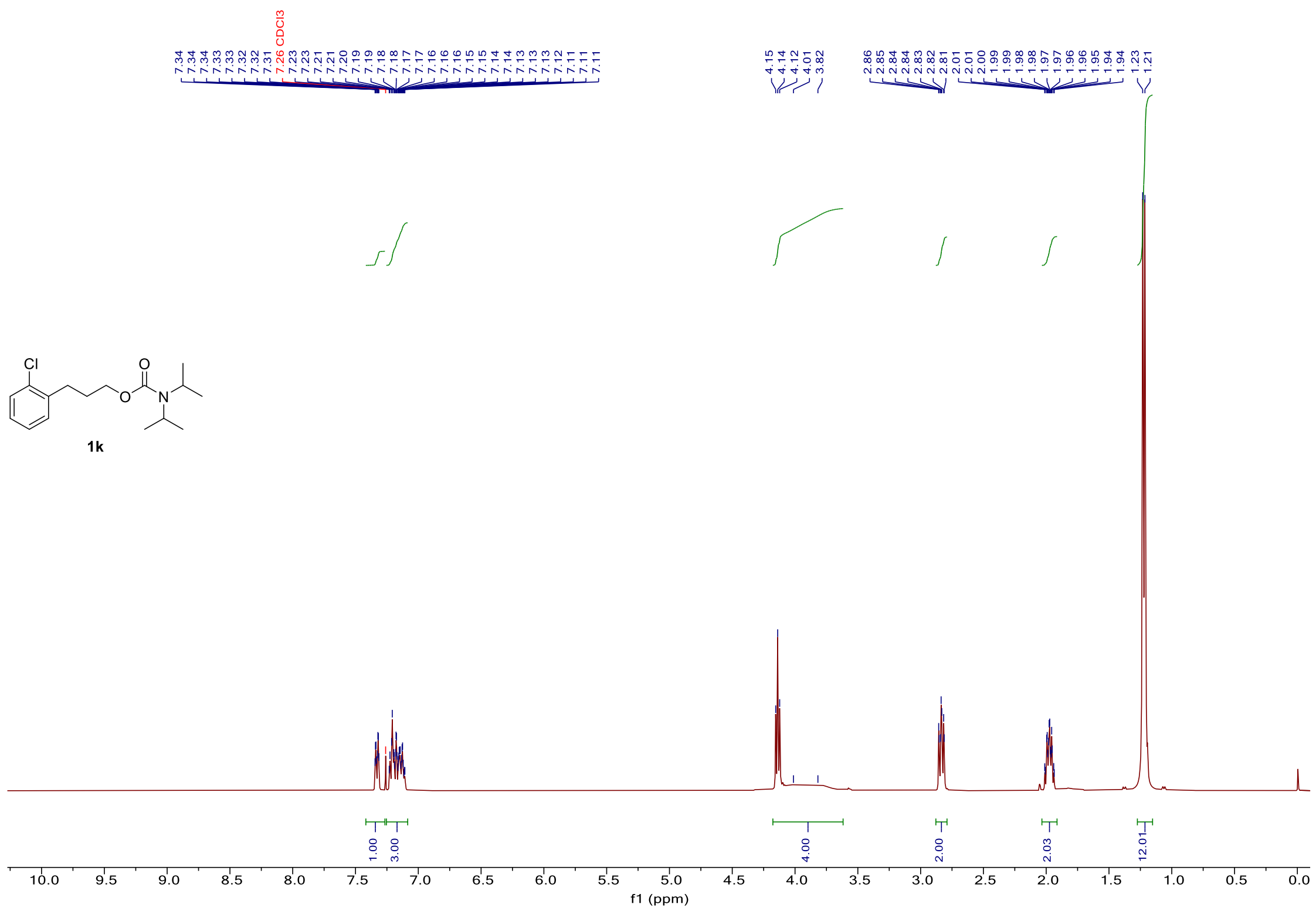

^1H NMR (400 MHz, $CDCl_3$)

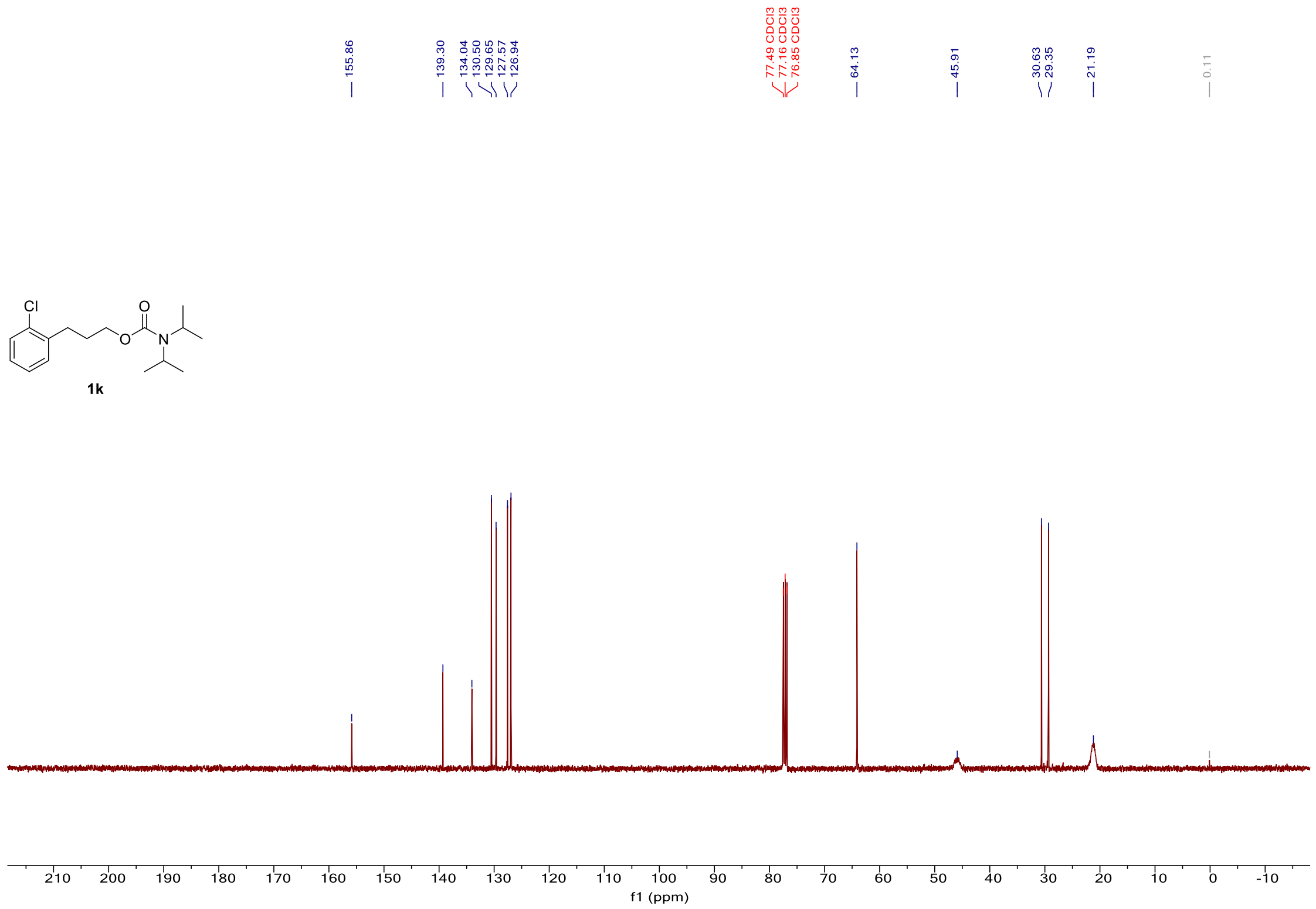

^{13}C NMR (101 MHz, $CDCl_3$)

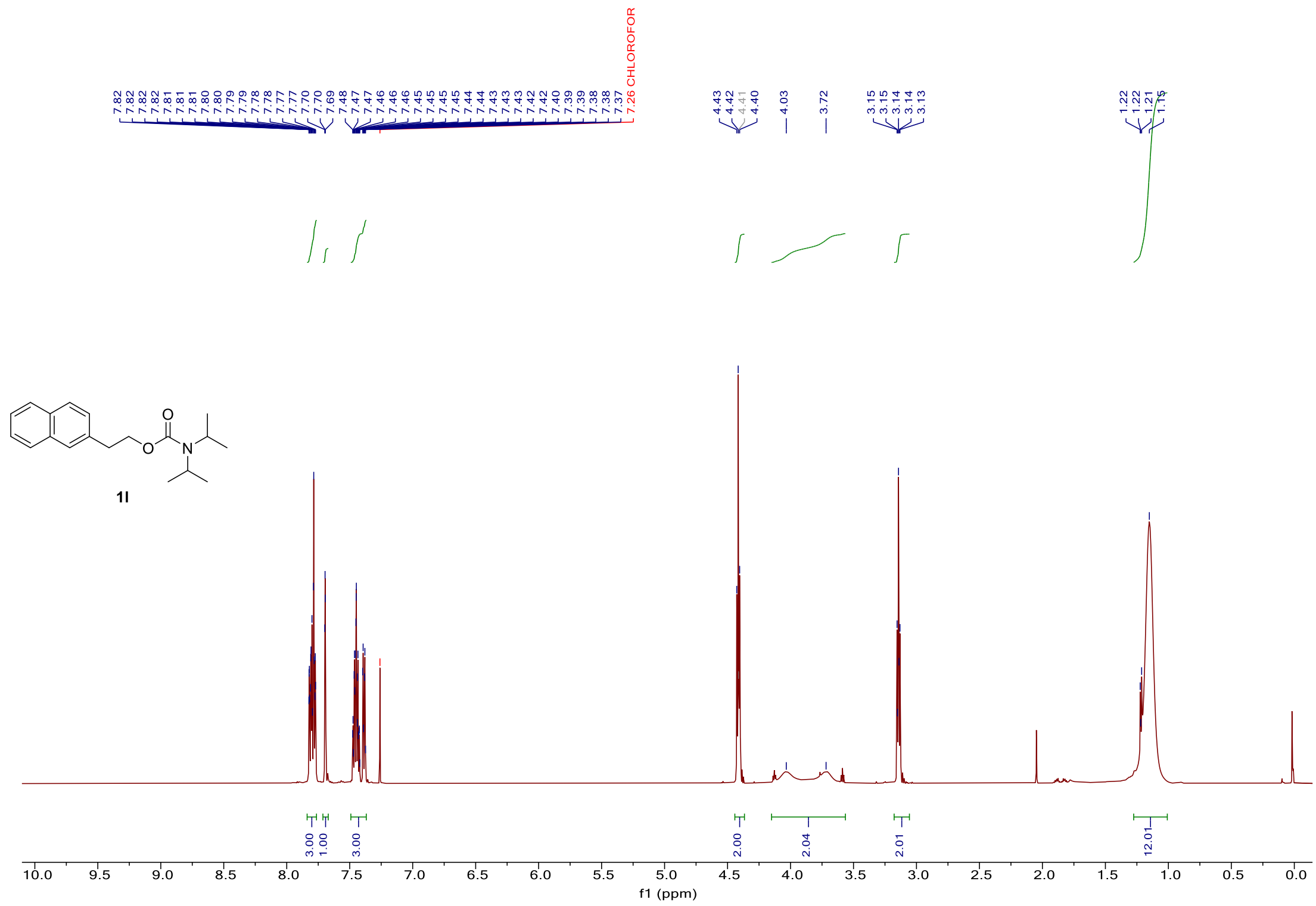

^{1}H NMR (600 MHz, $CDCl_3$)

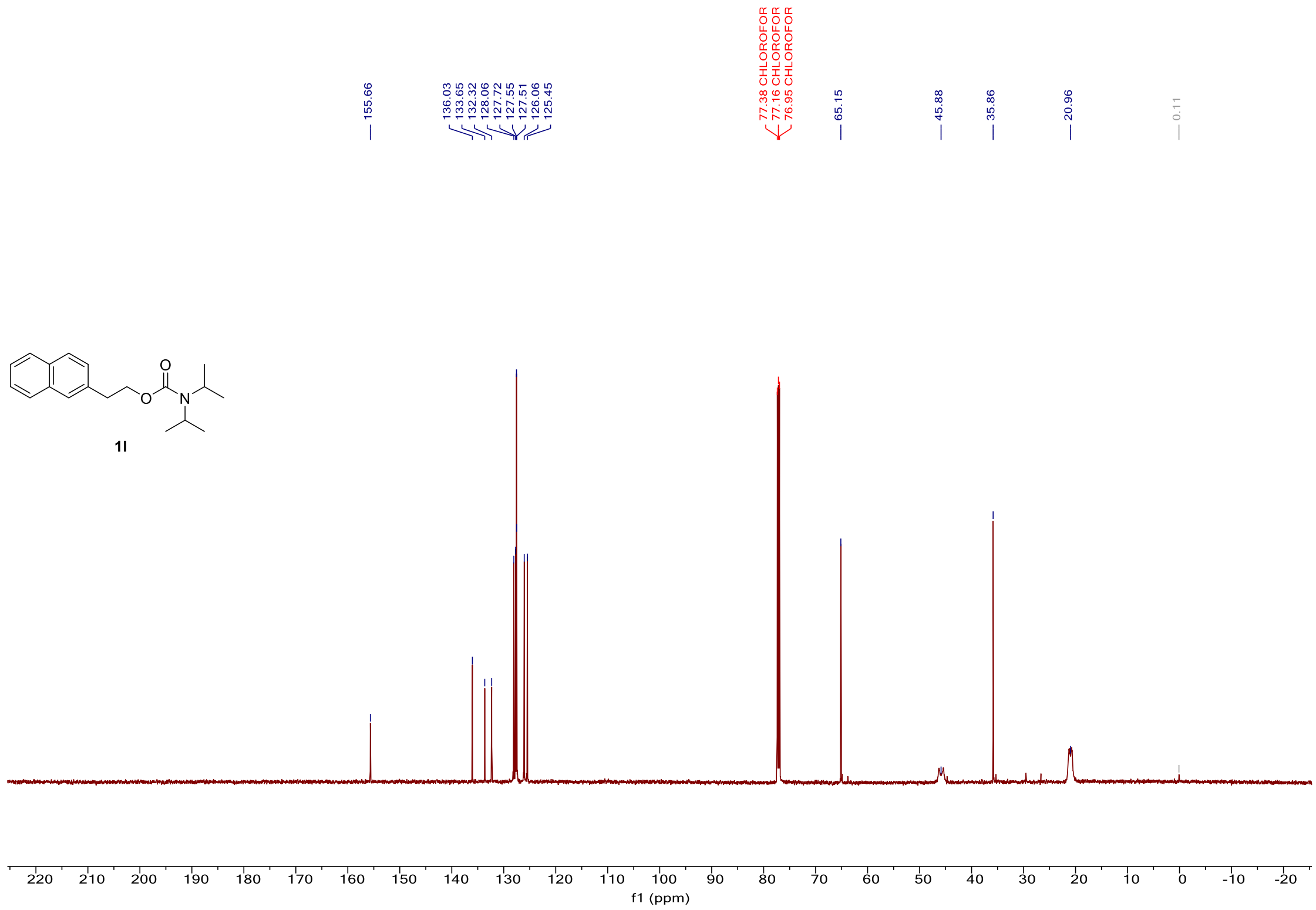

^{13}C NMR (151 MHz, $CDCl_3$)

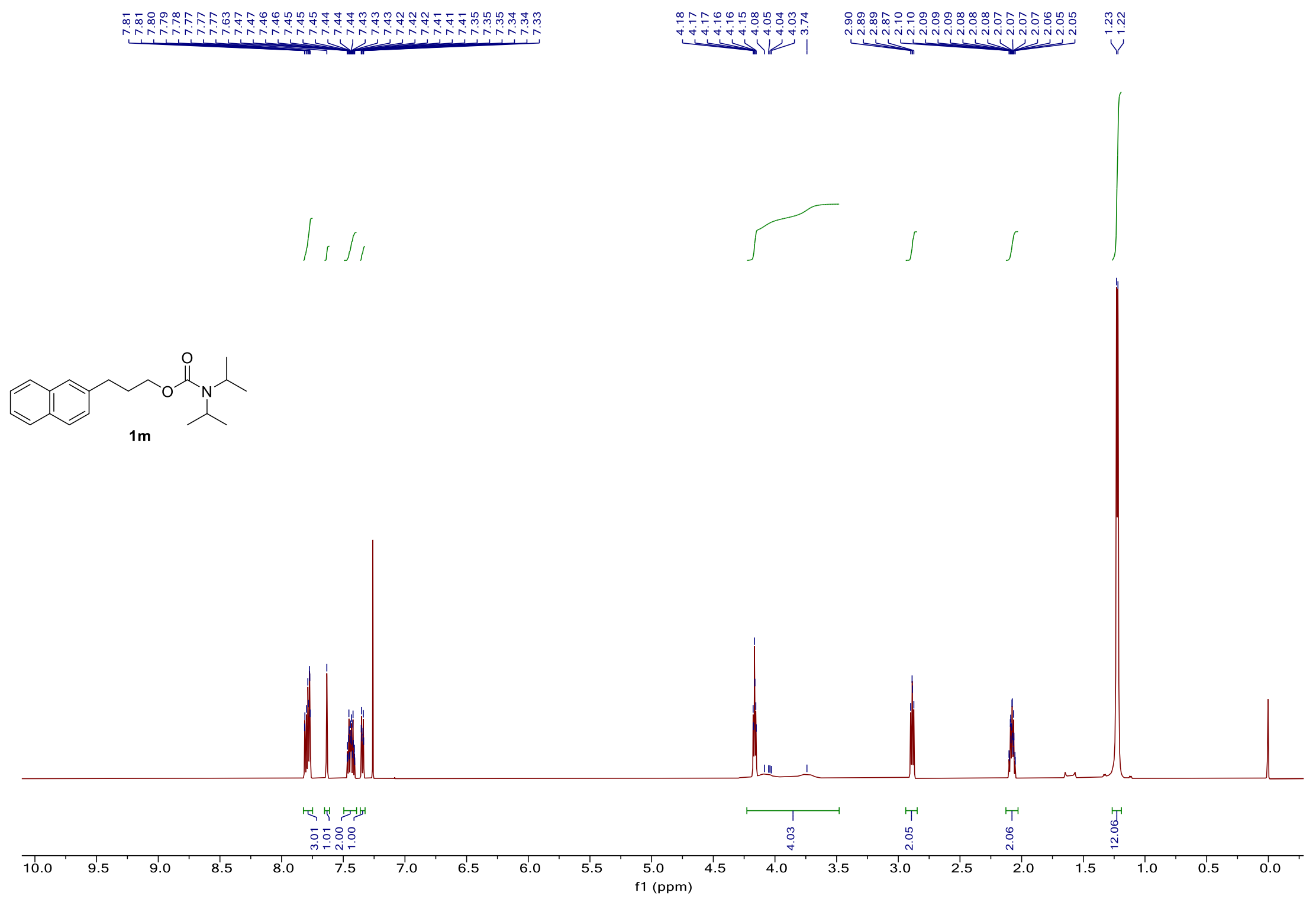

^{1}H NMR (600 MHz, $CDCl_3$)

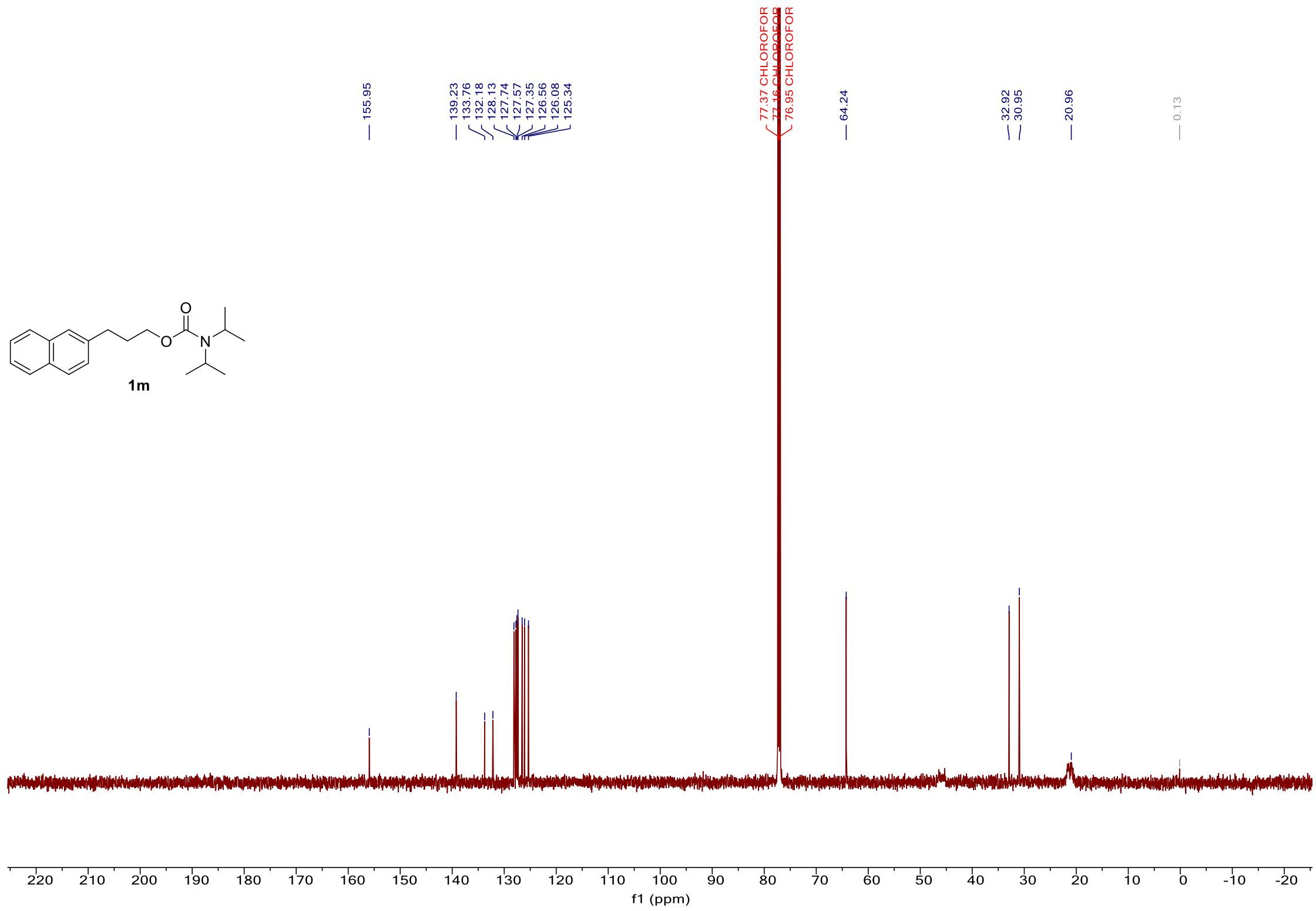

^{13}C NMR (151 MHz, $CDCl_3$)

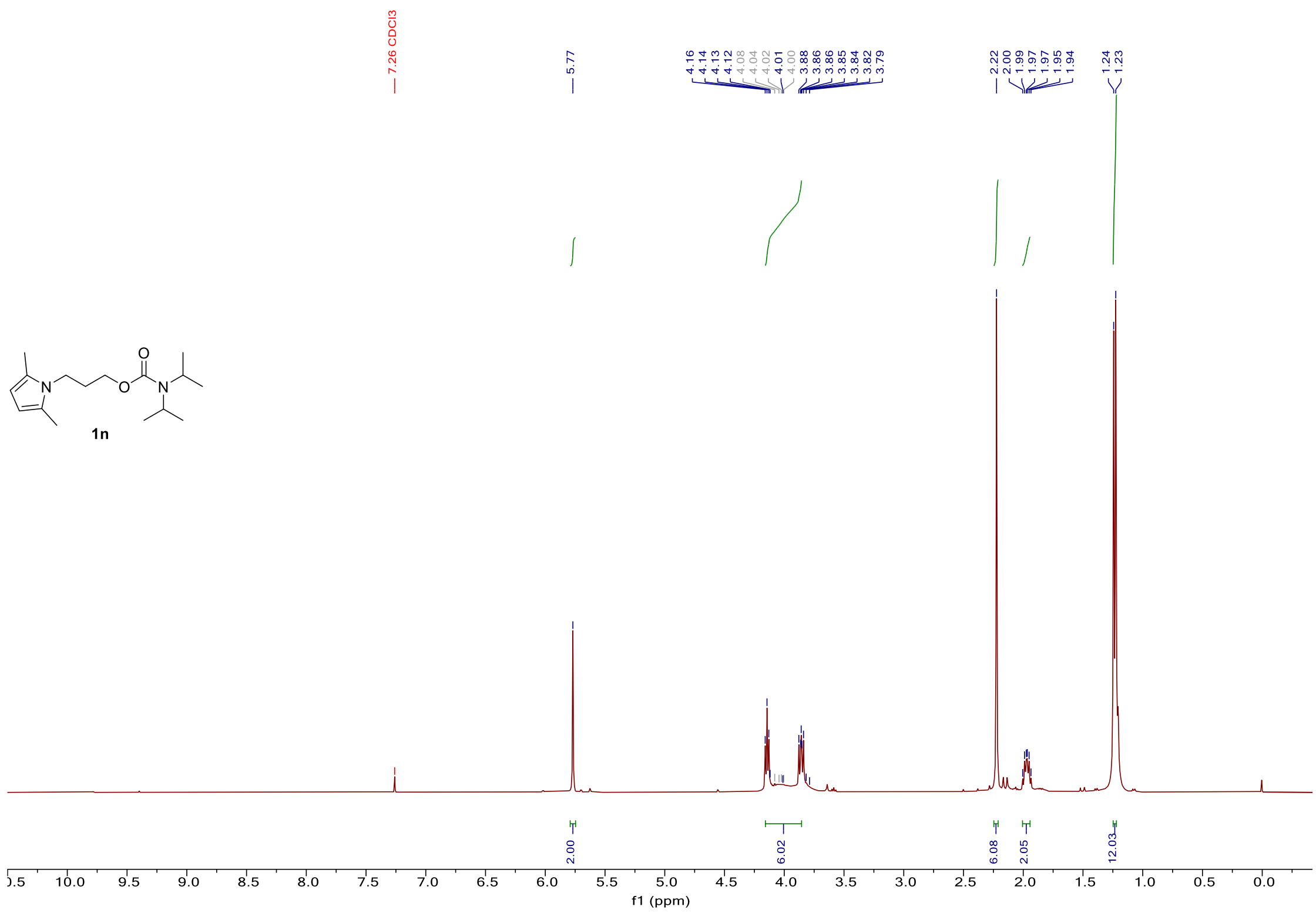

^{1}H NMR (400 MHz, $CDCl_3$)

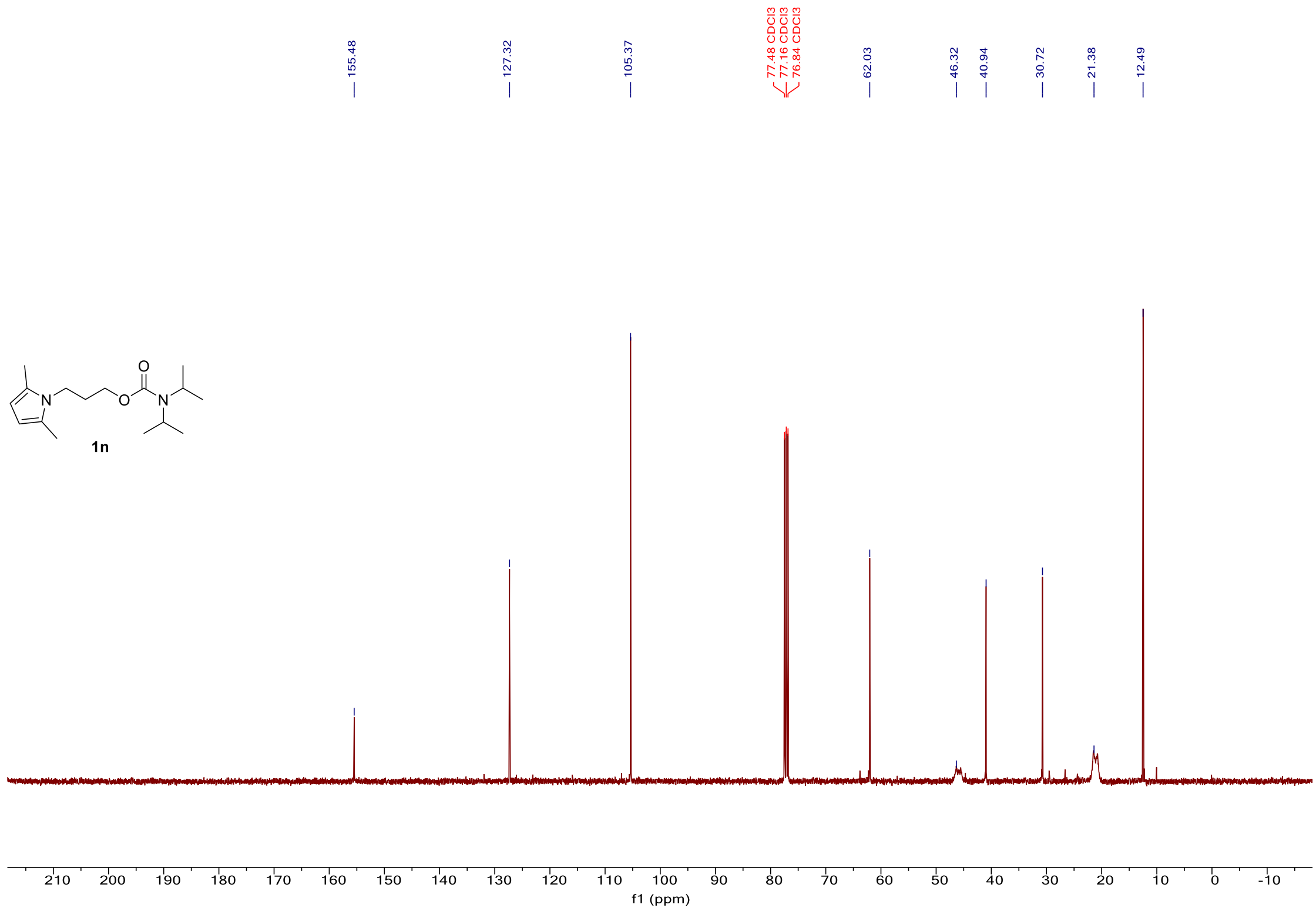

^{13}C NMR (101 MHz, $CDCl_3$)

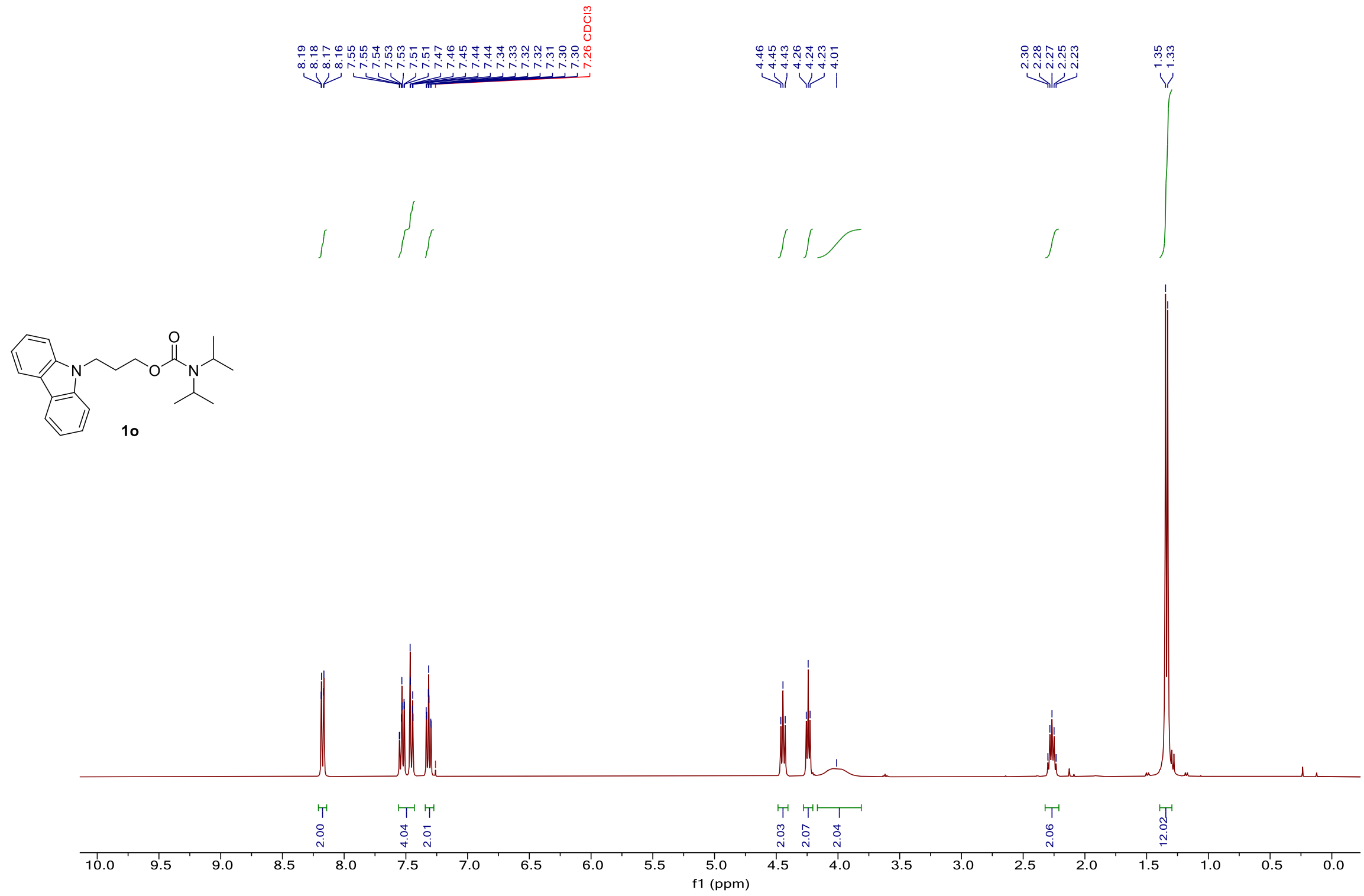

1o

^{1}H NMR (400 MHz, $CDCl_3$)

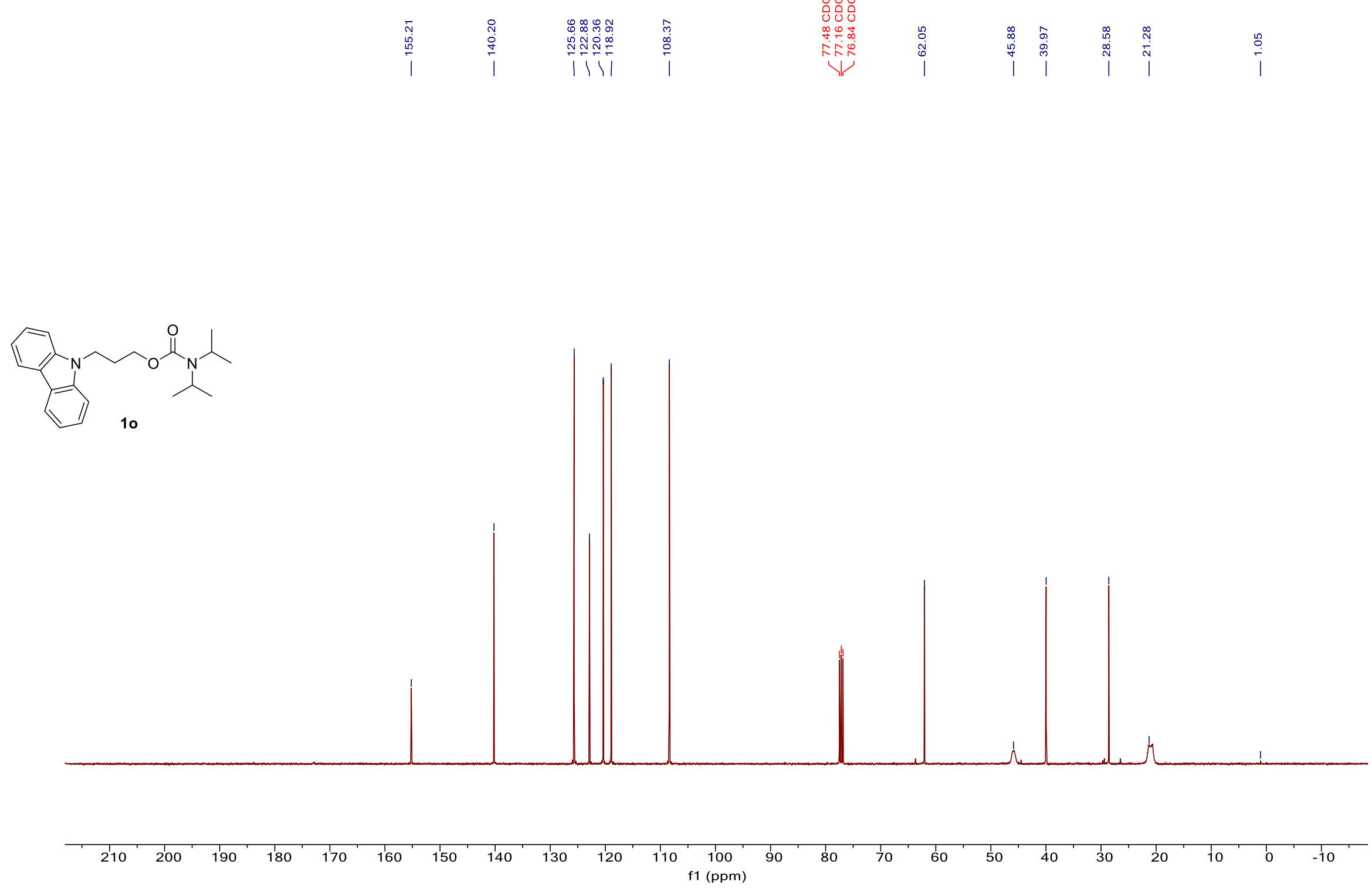

1o

^{13}C NMR (101 MHz, $CDCl_3$)

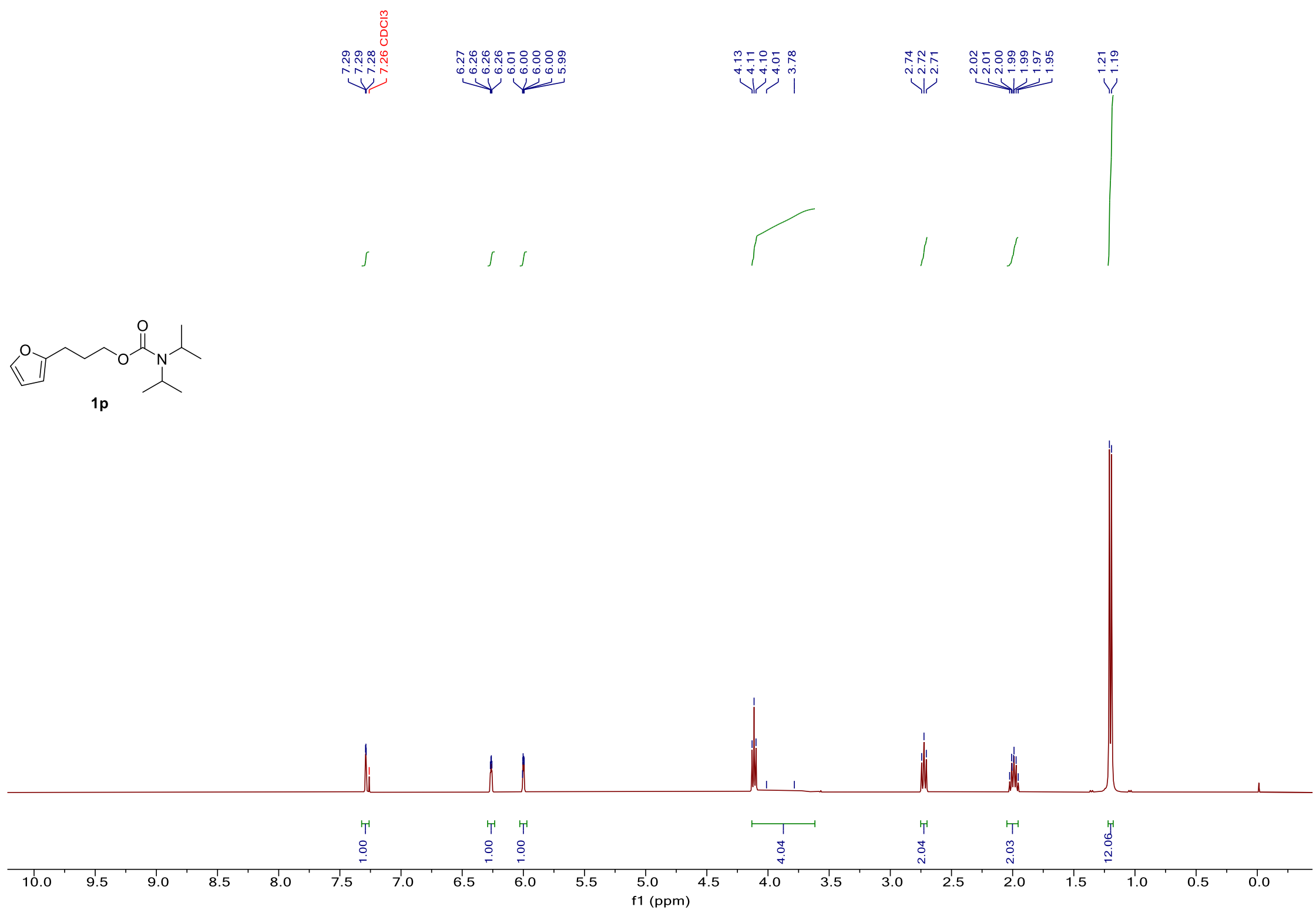

^1H NMR (400 MHz, $CDCl_3$)

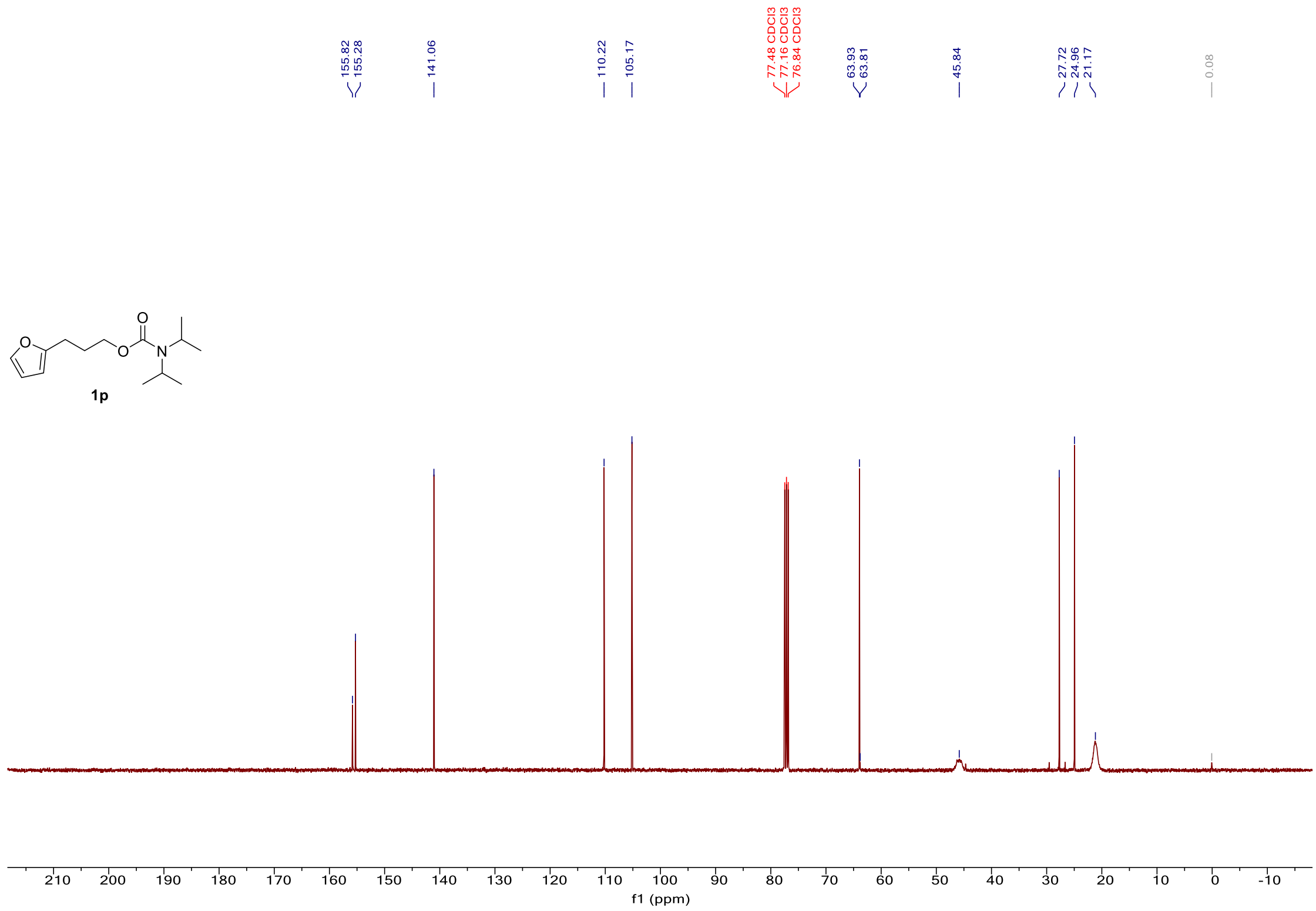

^{13}C NMR (101 MHz, $CDCl_3$)

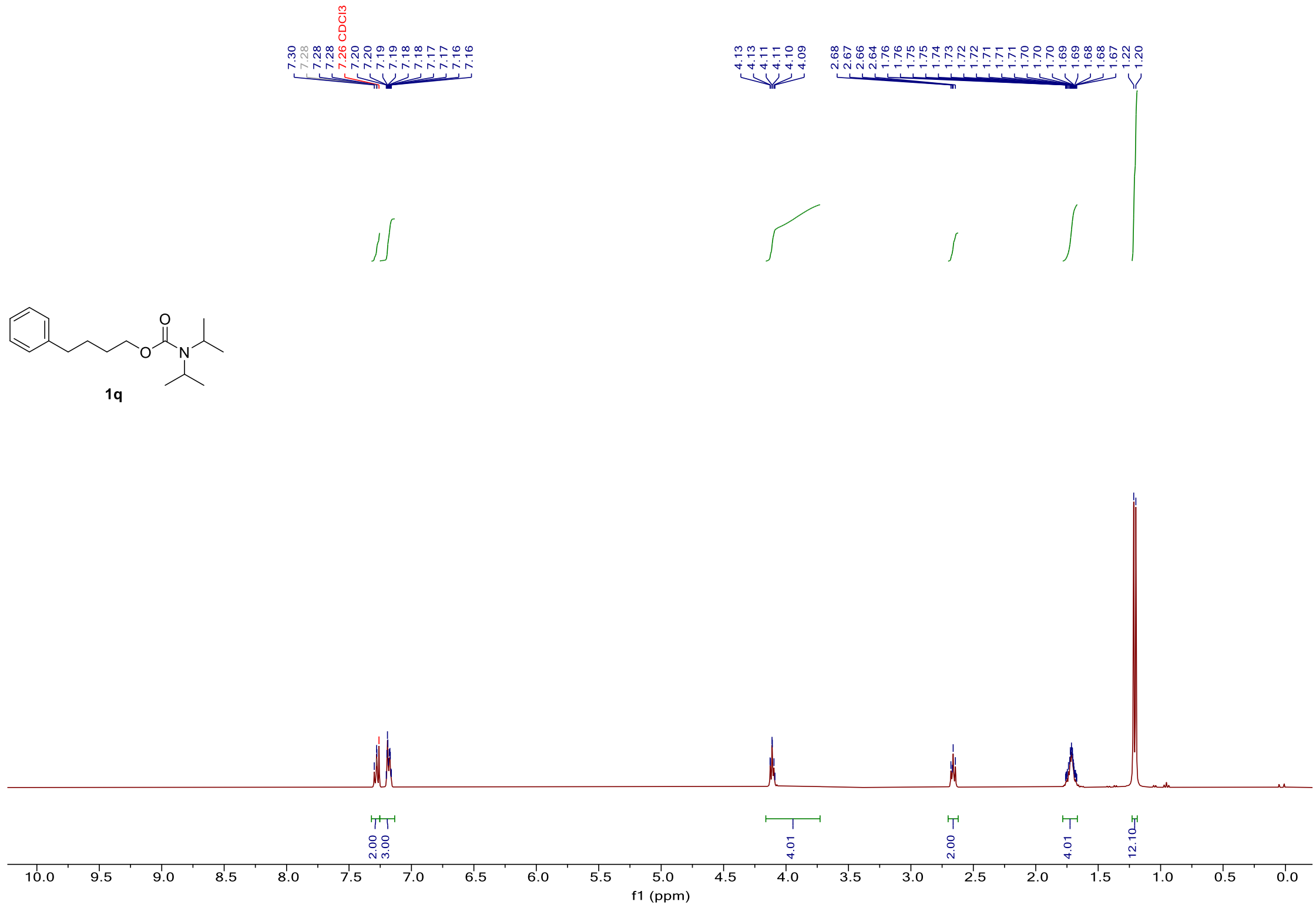

^{1}H NMR (400 MHz, $CDCl_3$)

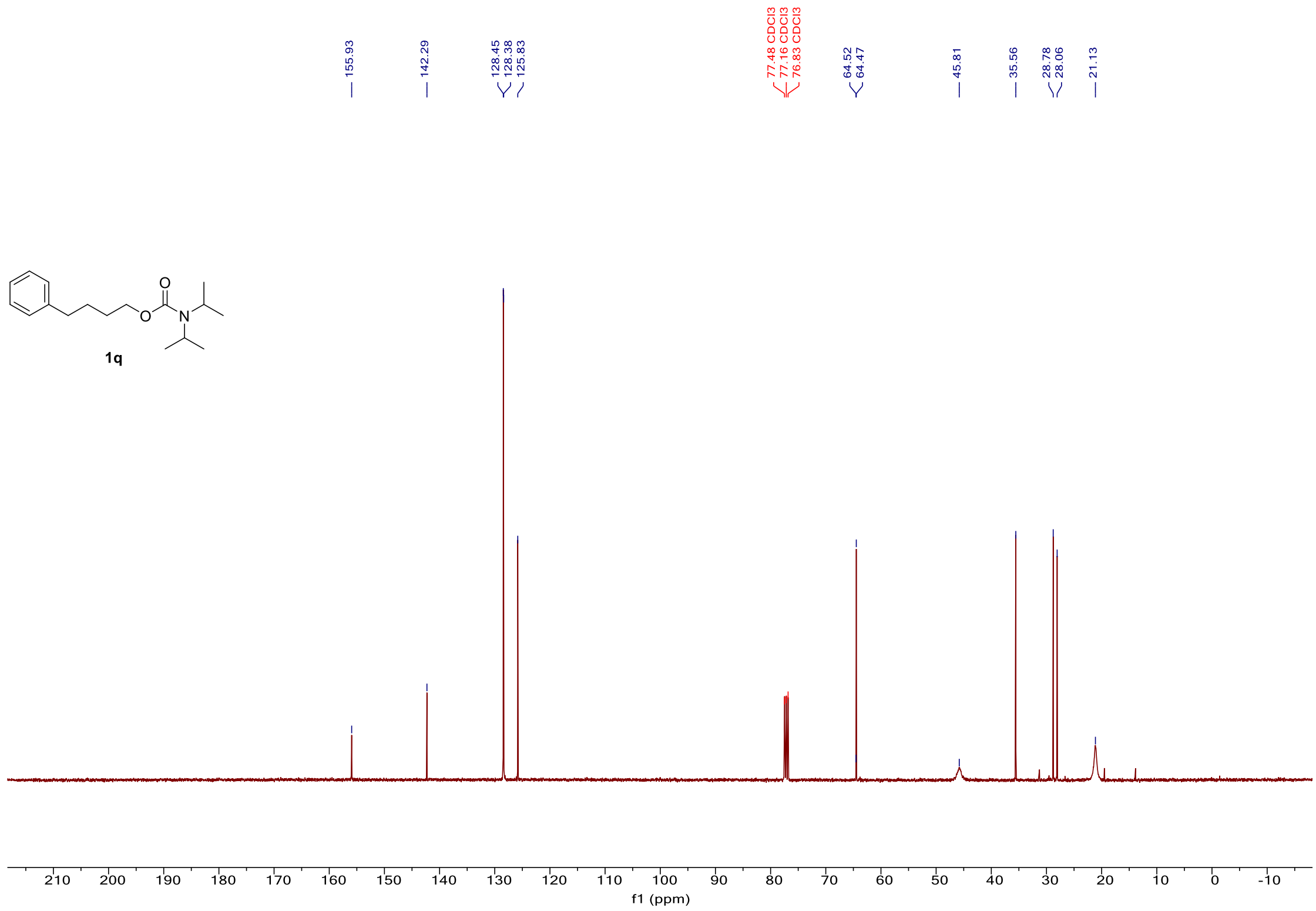

^{13}C NMR (101 MHz, $CDCl_3$)

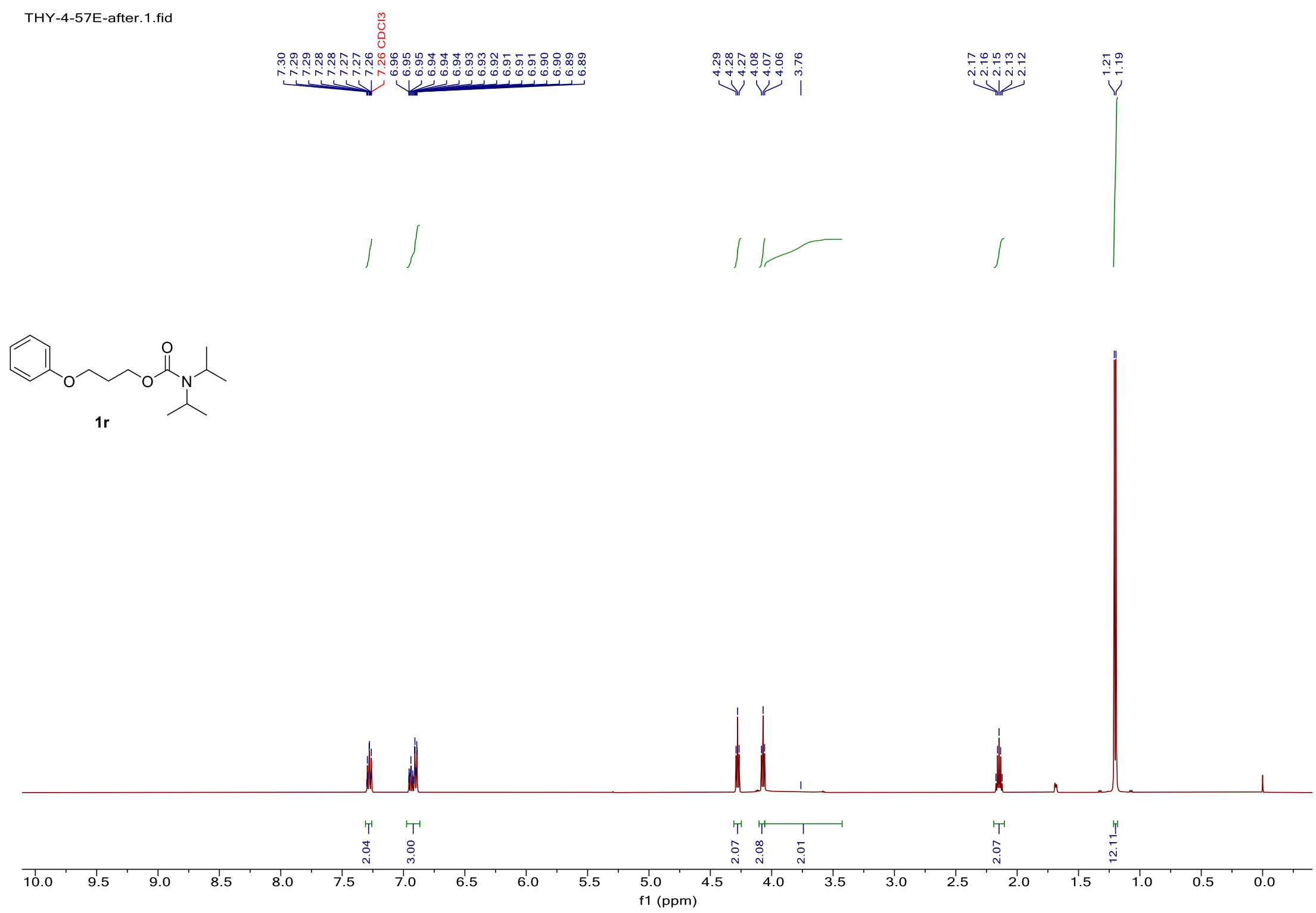

^{1}H NMR (400 MHz, $CDCl_3$)

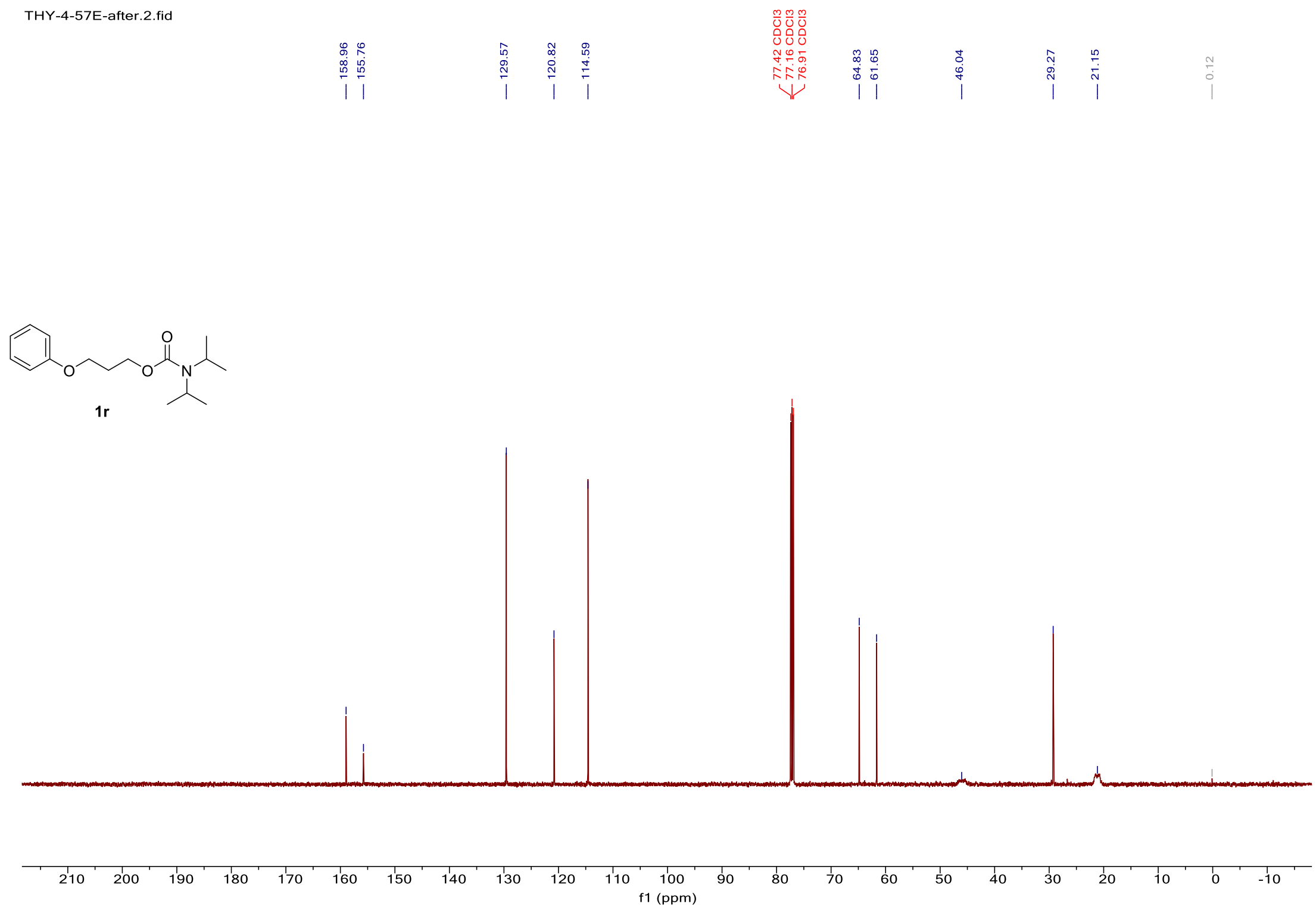

^{13}C NMR (101 MHz, $CDCl_3$)

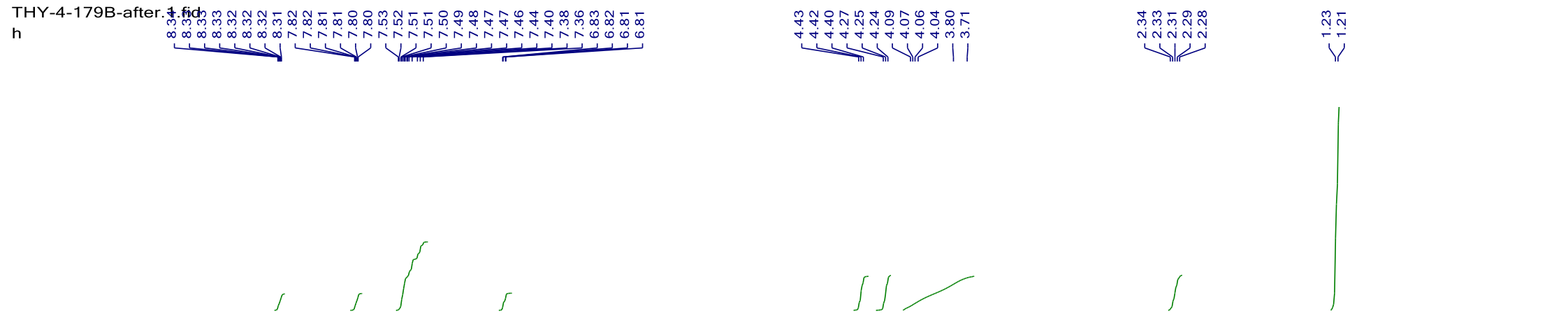

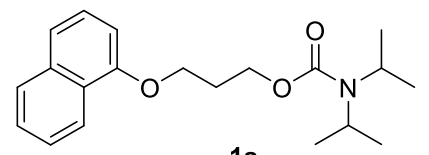

1s

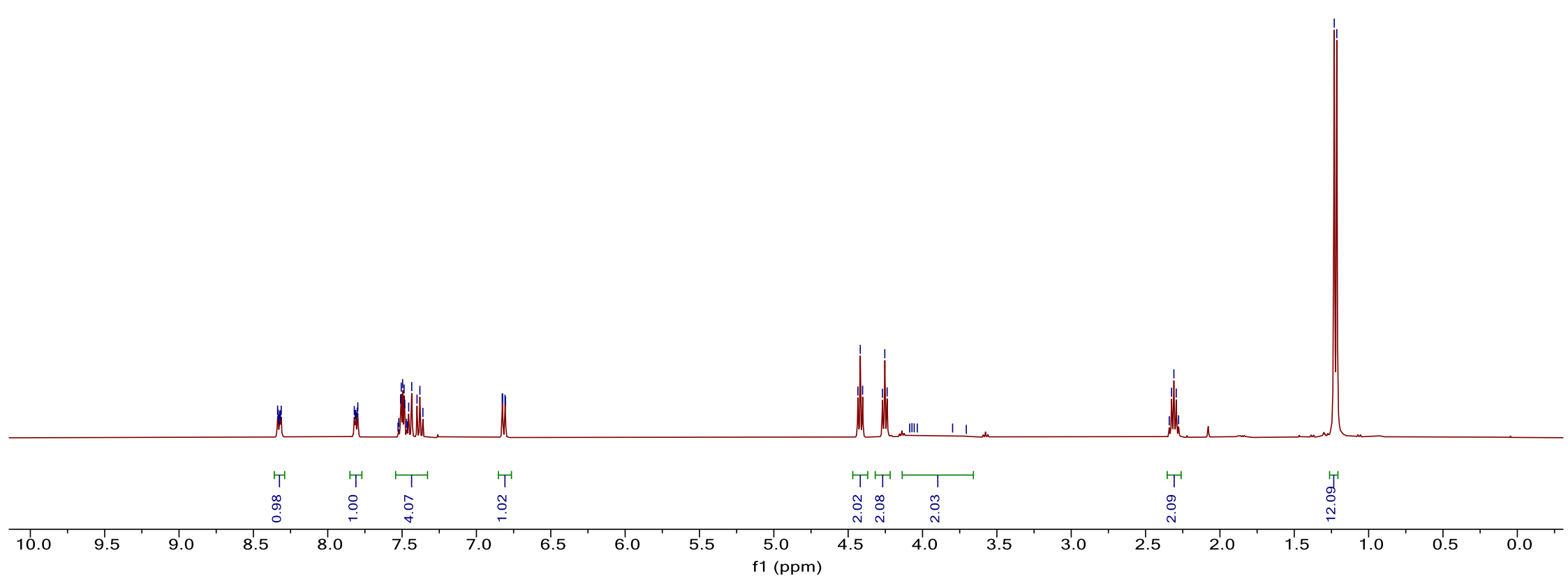

^{1}H NMR (400 MHz, $CDCl_3$)

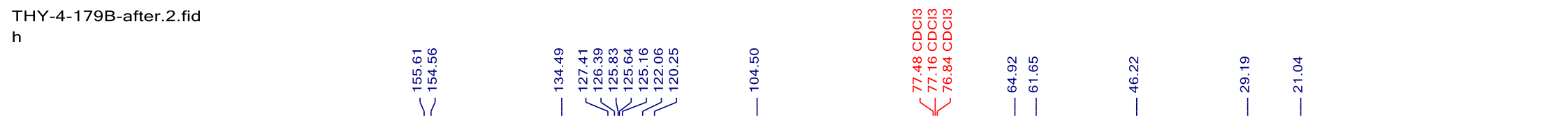

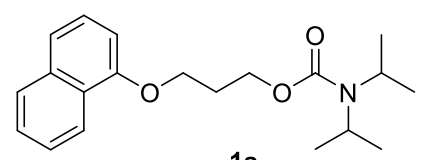

1s

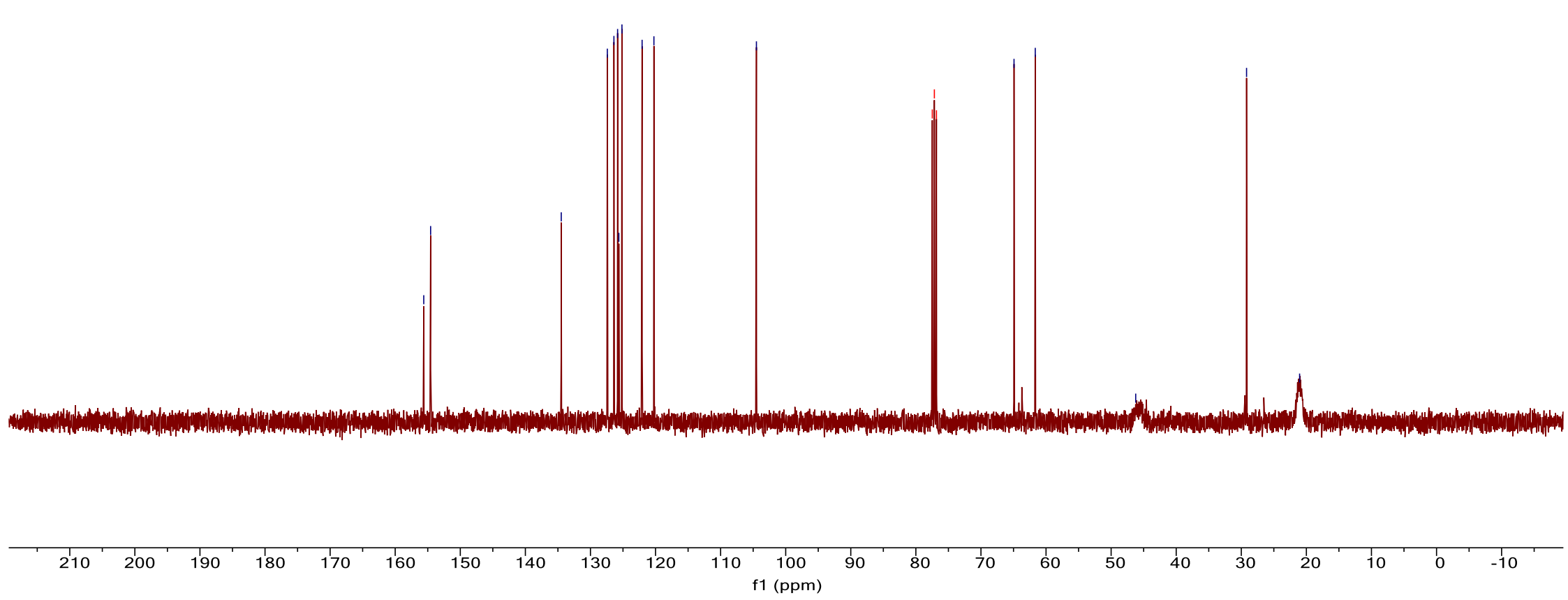

^{13}C NMR (101 MHz, $CDCl_3$)

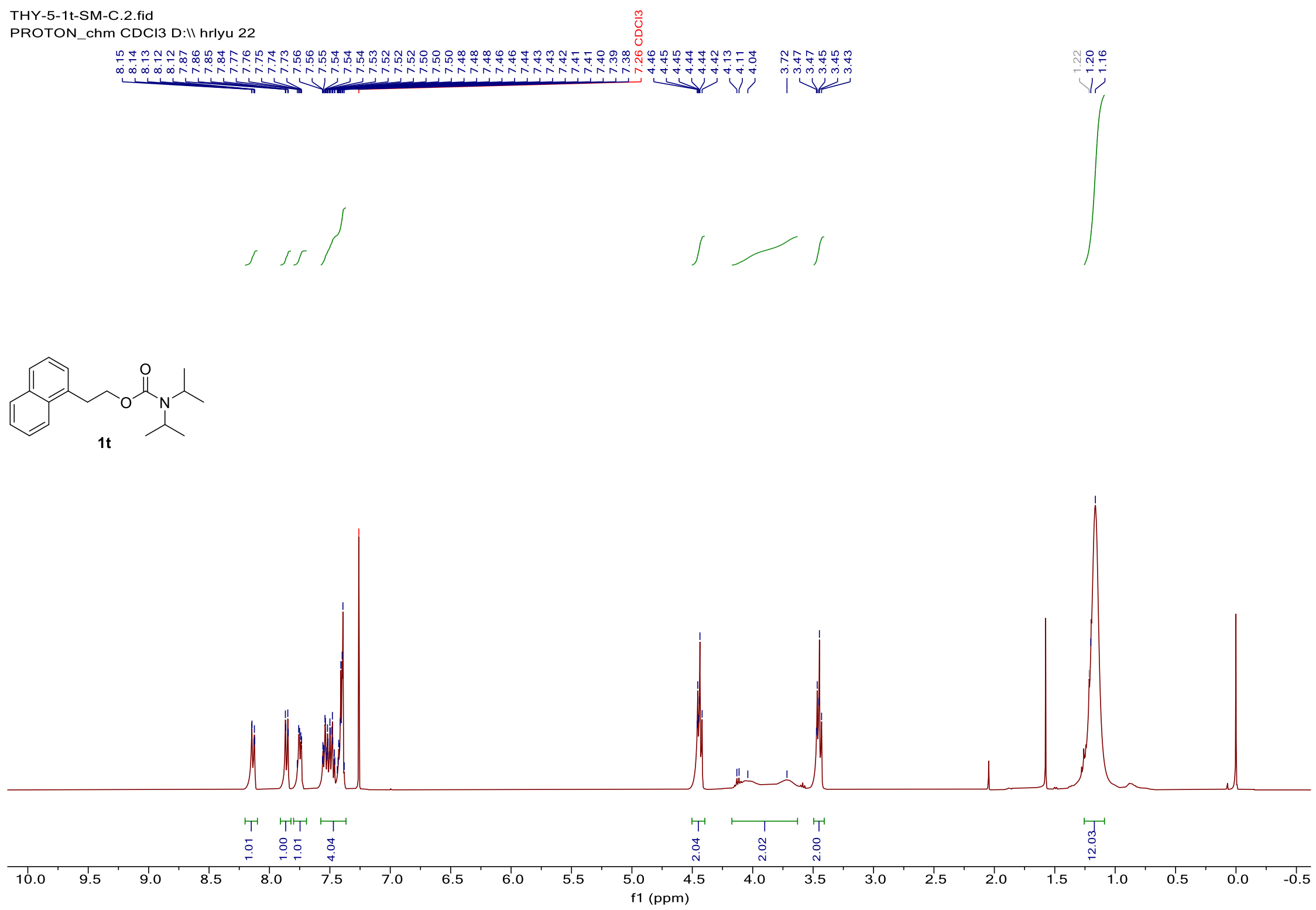

^{1}H NMR (400 MHz, CDCl3)

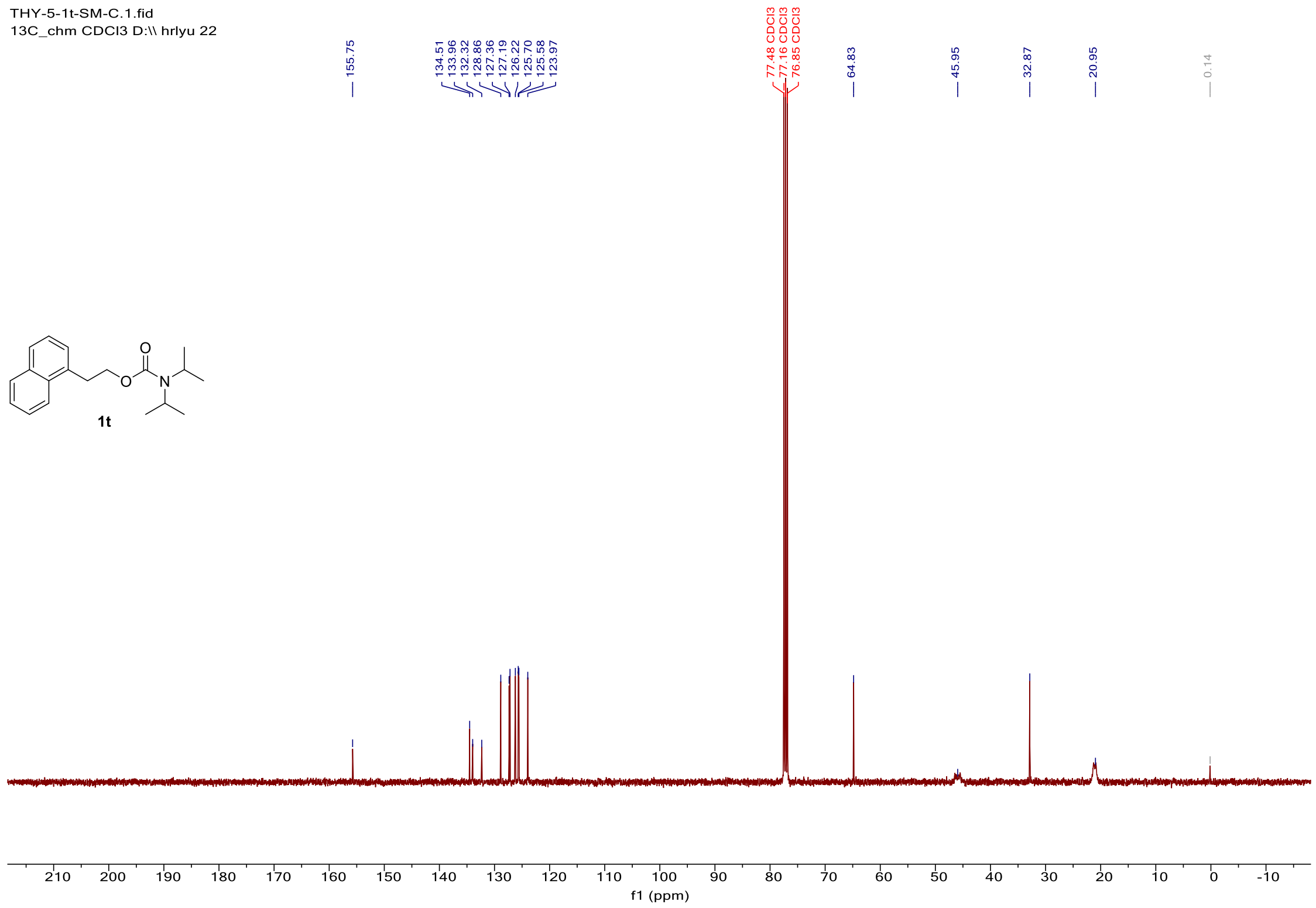

^{13}C NMR (101 MHz, CDCl3)

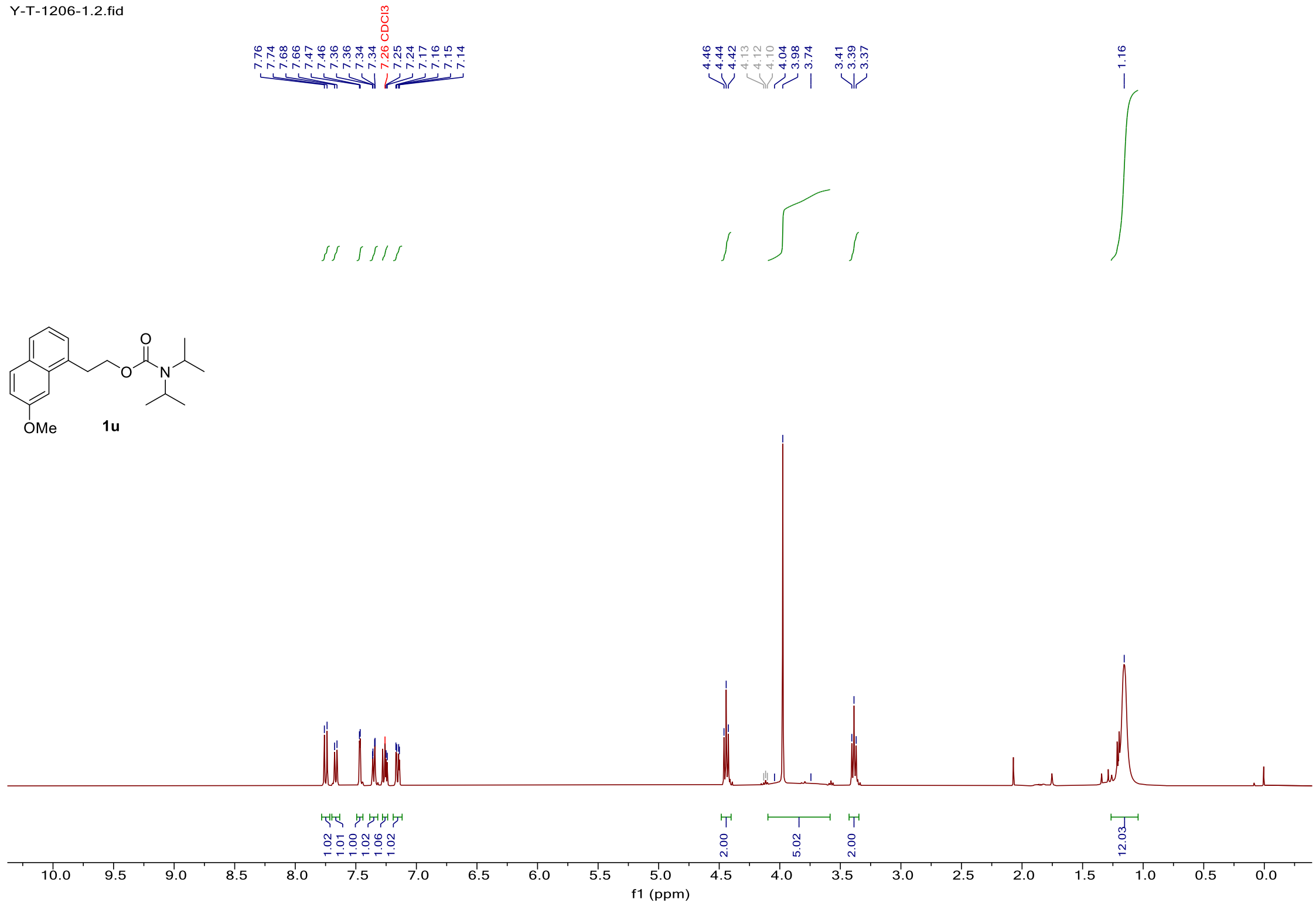

^{1}H NMR (400 MHz, $CDCl_3$)

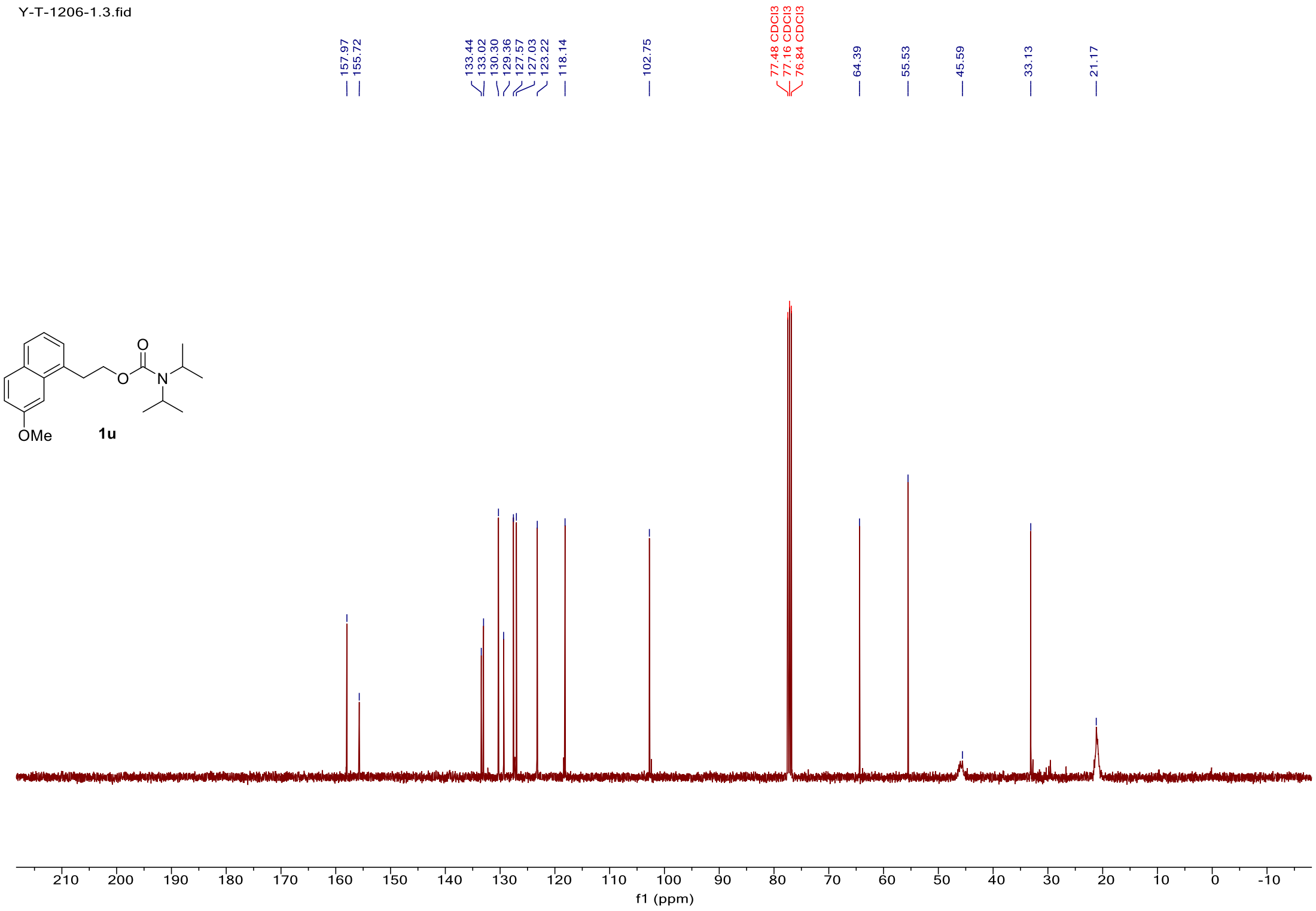

^{13}C NMR (101 MHz, $CDCl_3$)

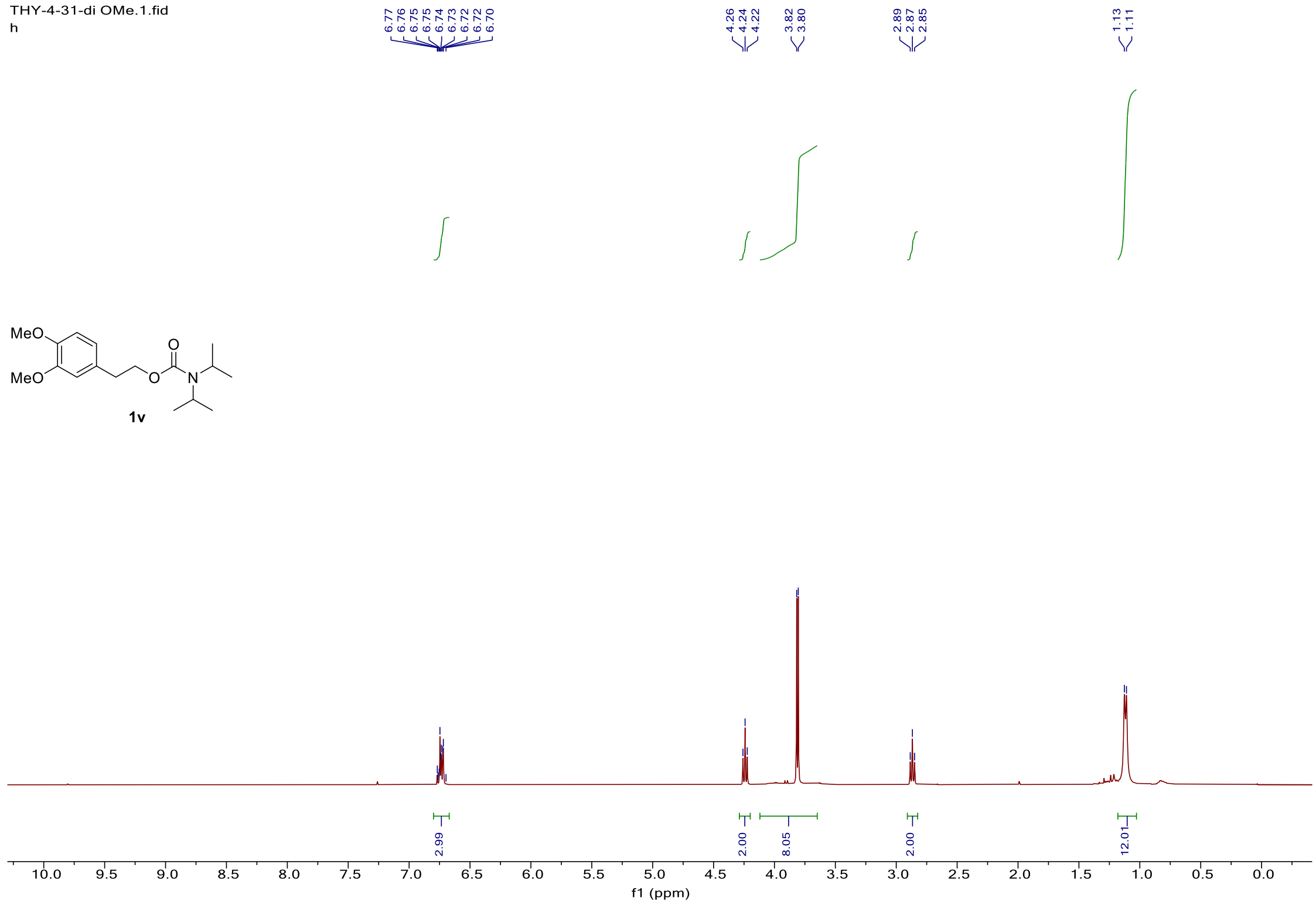

^{1}H NMR (400 MHz, $CDCl_3$)

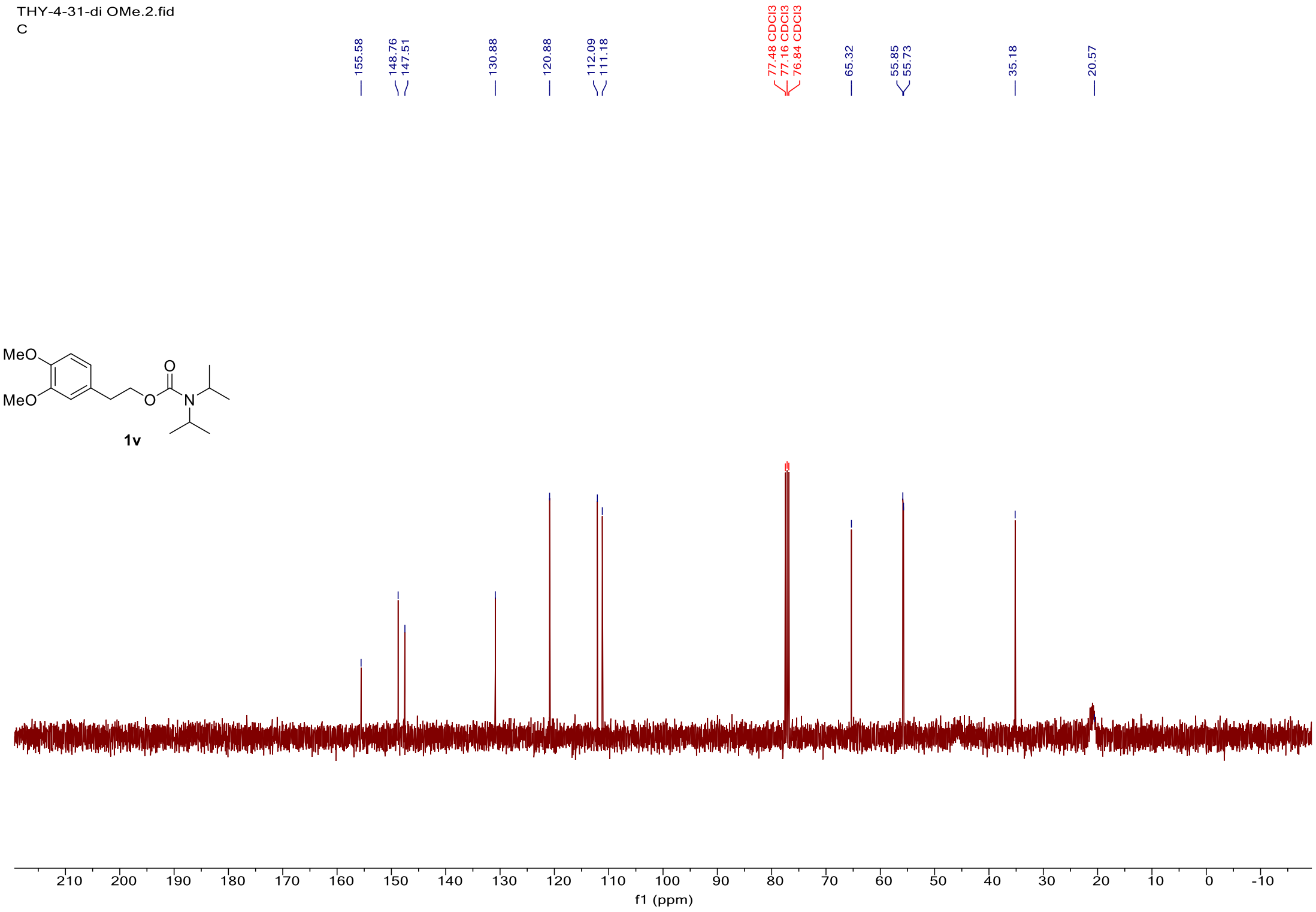

^{13}C NMR (101 MHz, $CDCl_3$)

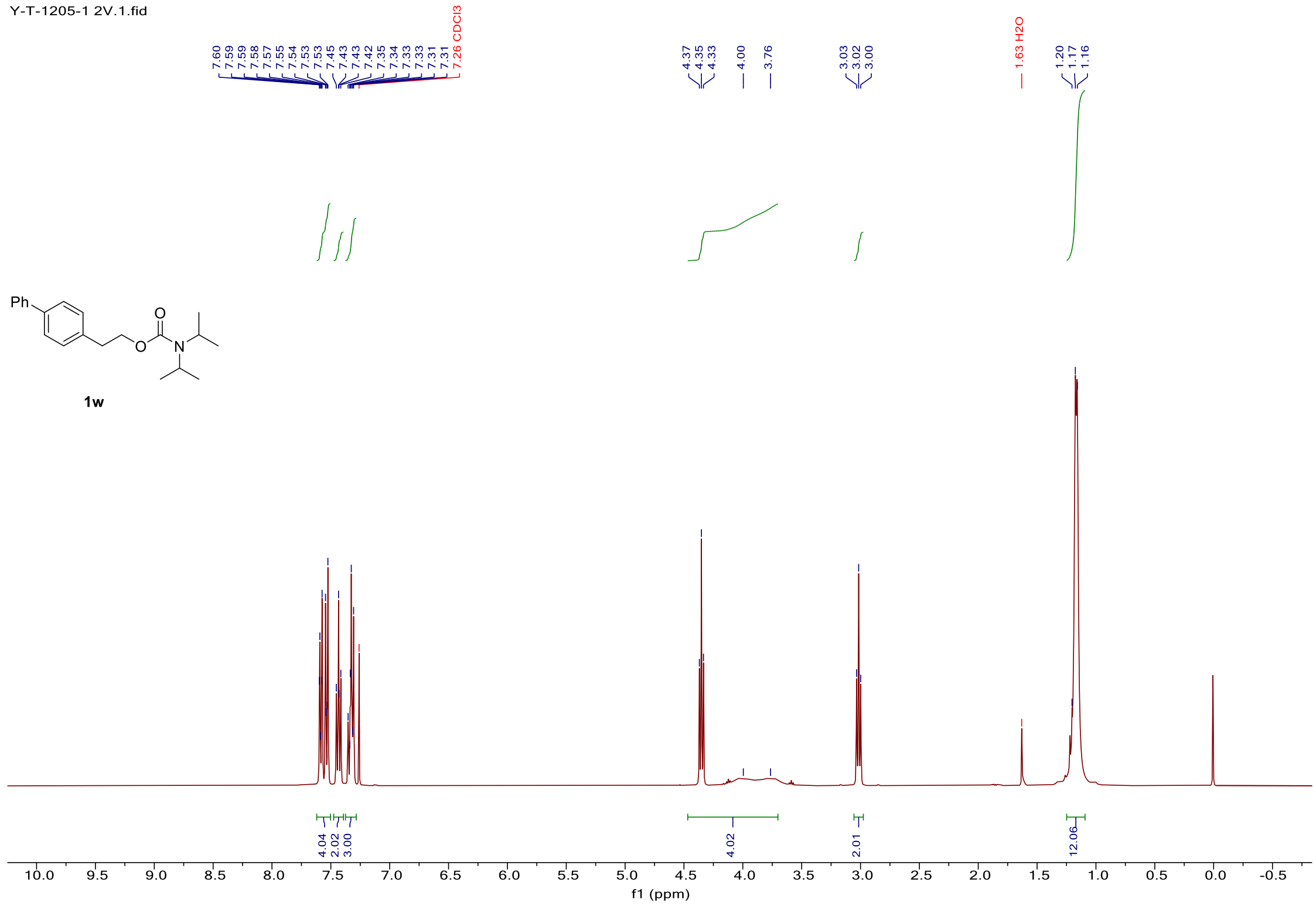

^{1}H NMR (400 MHz, CDCl$_3$)

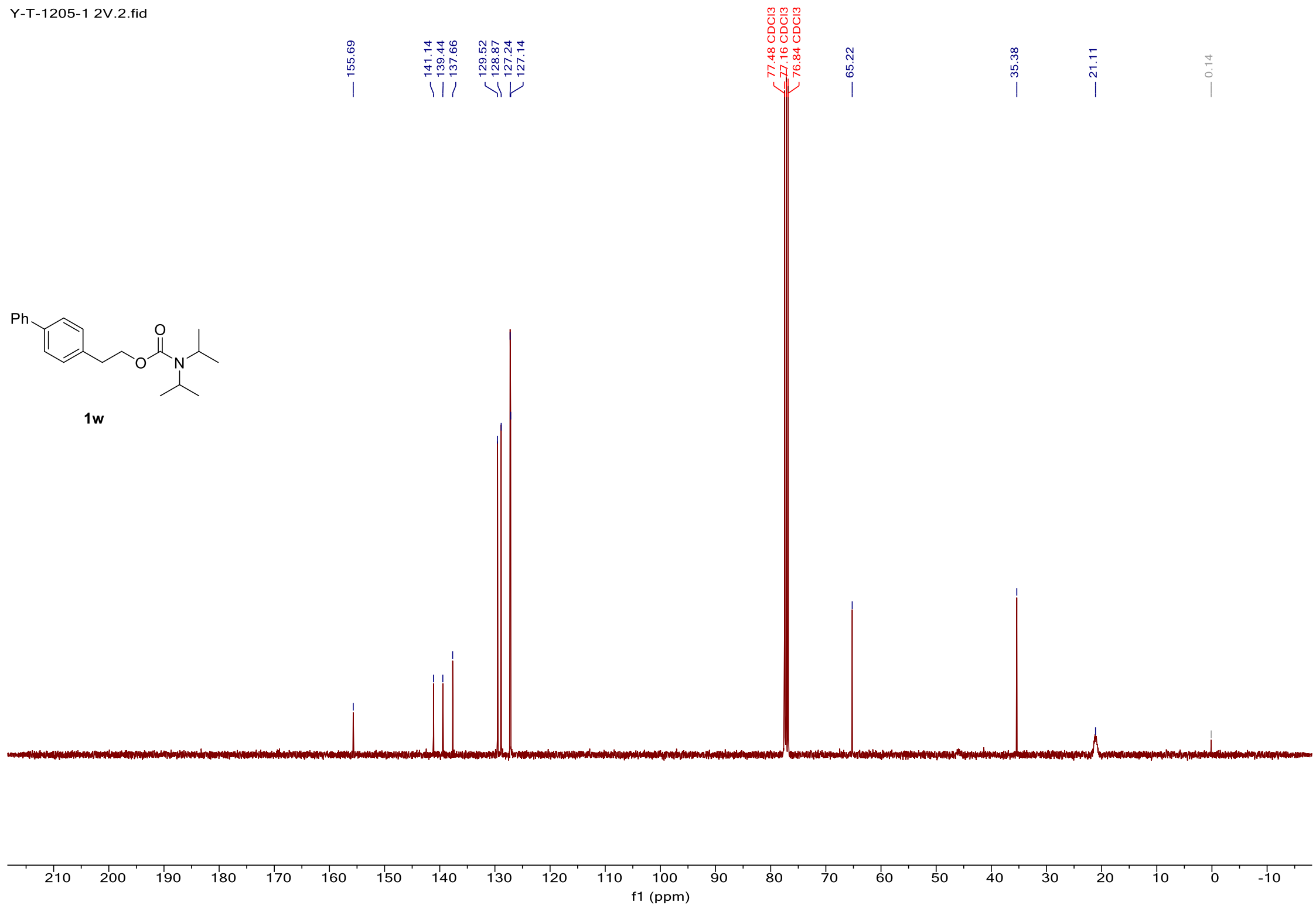

^{13}C NMR (101 MHz, CDCl$_3$)

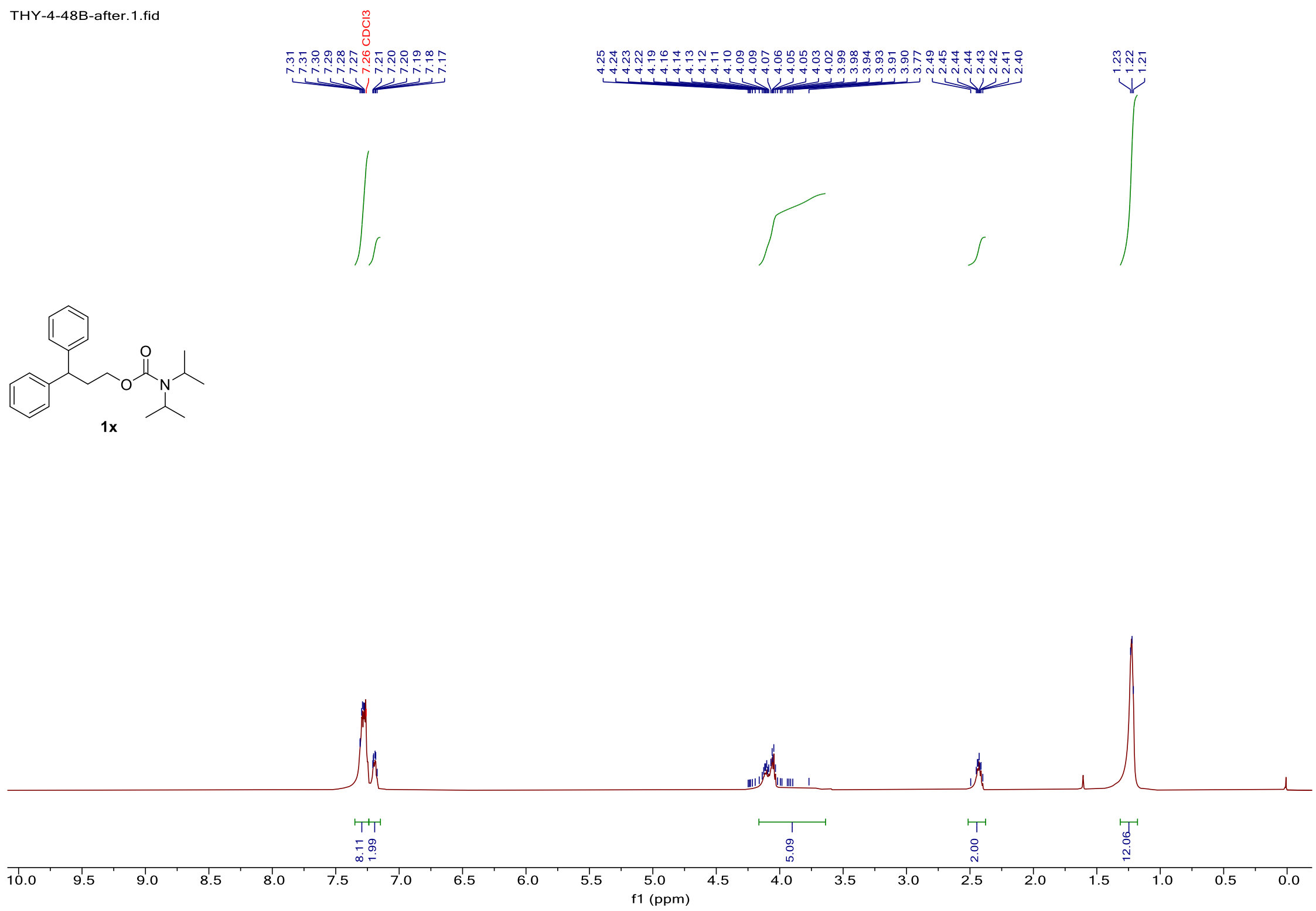

^{1}H NMR (400 MHz, $CDCl_3$)

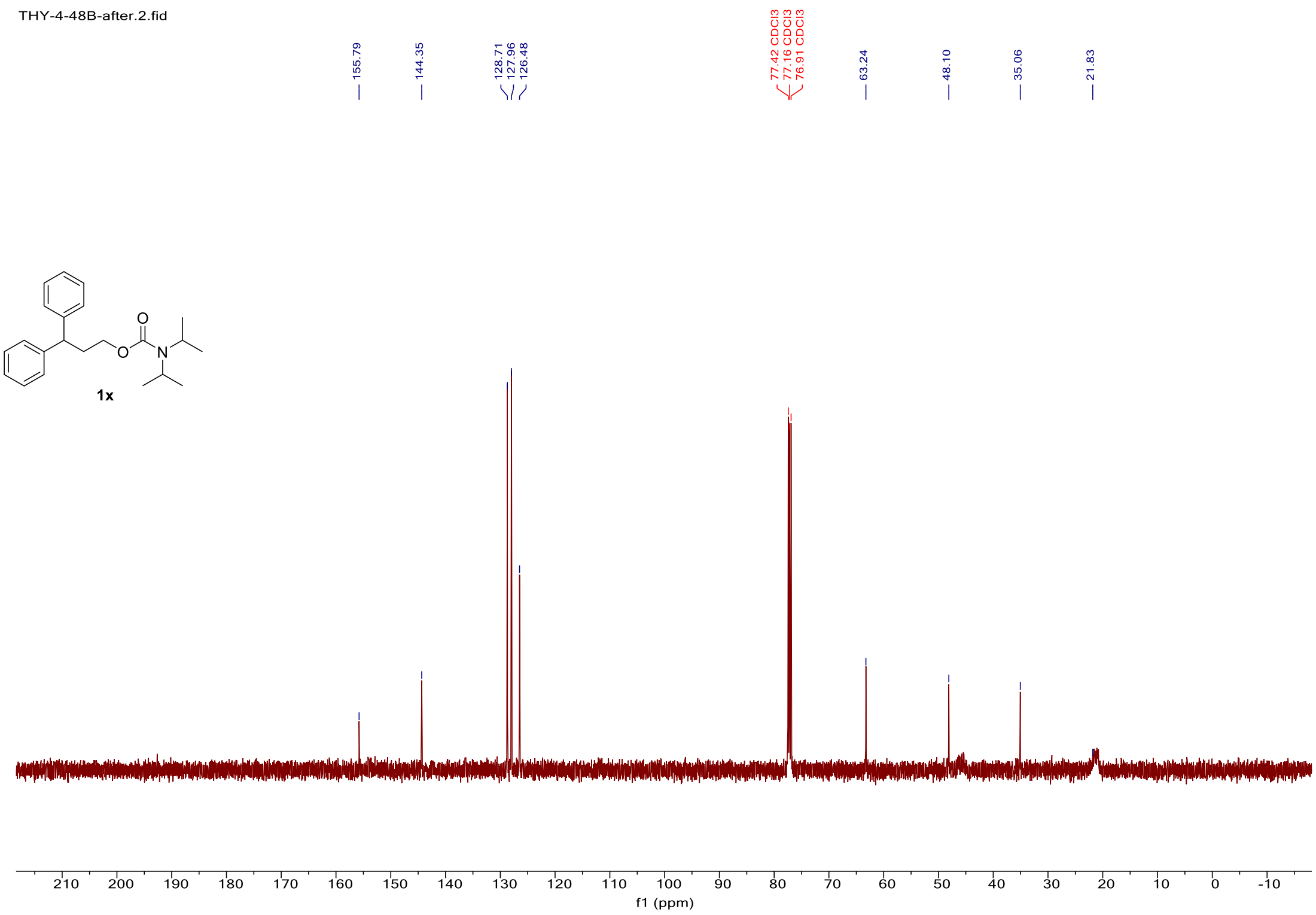

^{13}C NMR (101 MHz, $CDCl_3$)

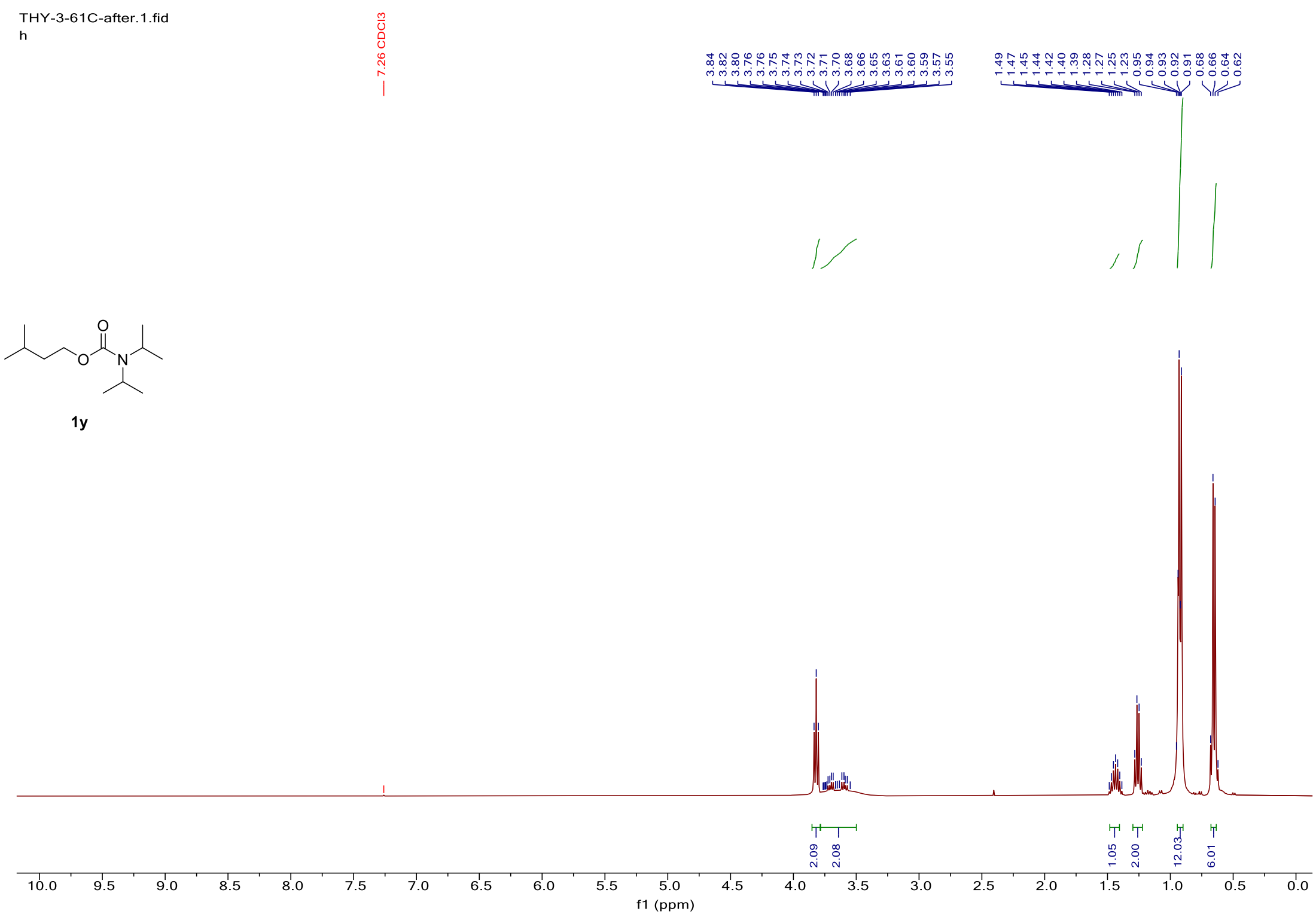

^{1}H NMR (400 MHz, $CDCl_3$)

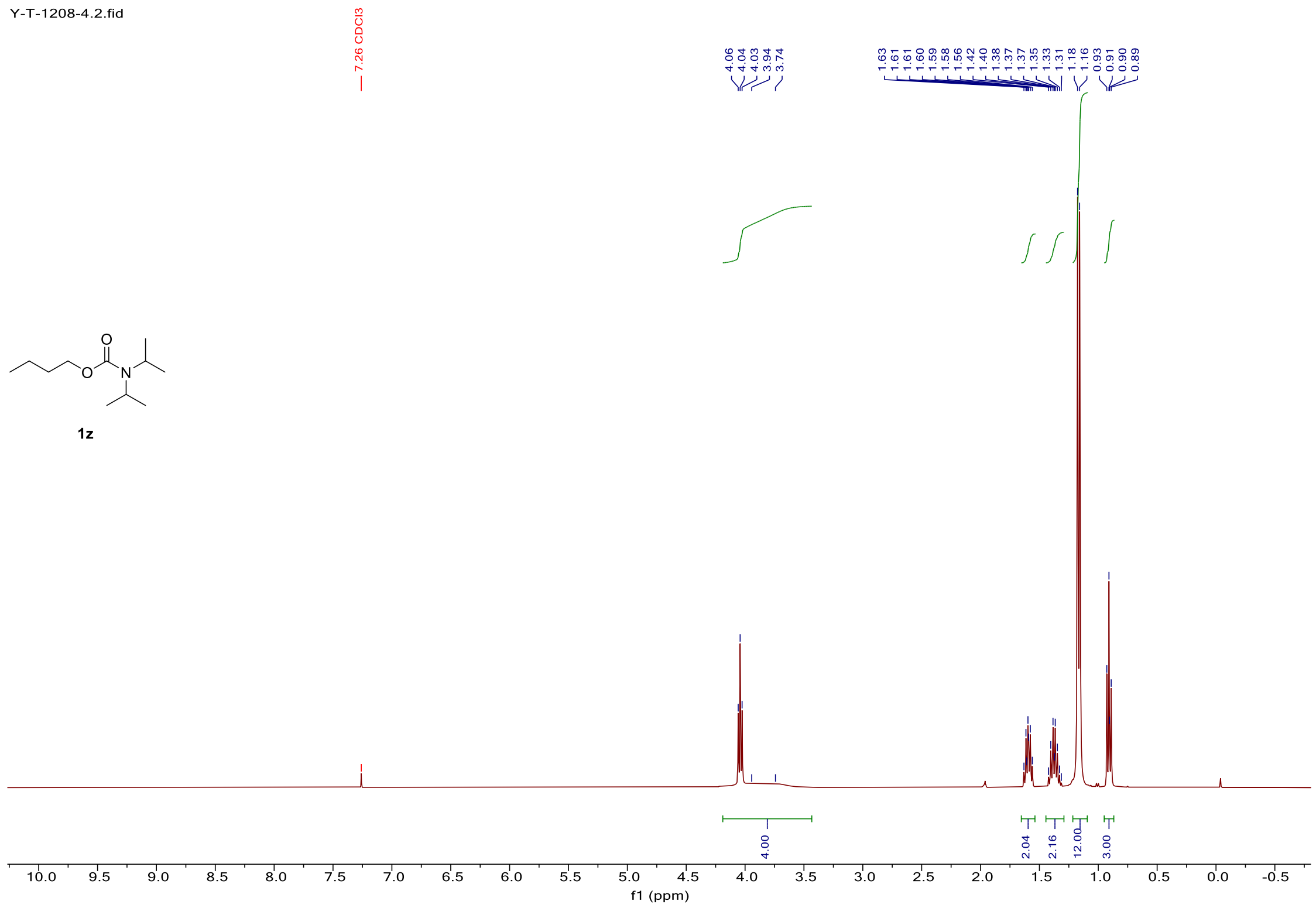

^{1}H NMR (400 MHz, $CDCl_3$)

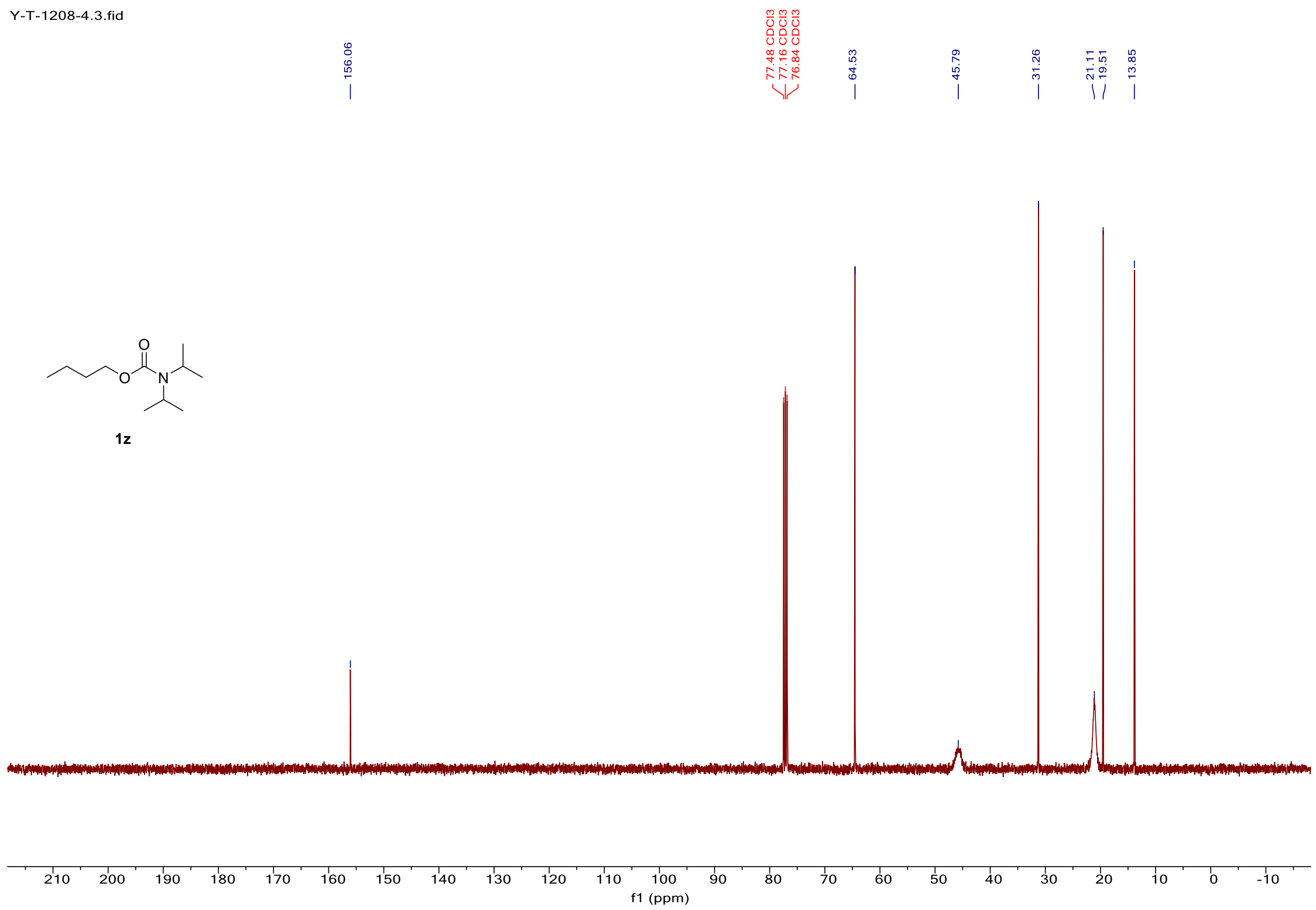

^{13}C NMR (101 MHz, $CDCl_3$)

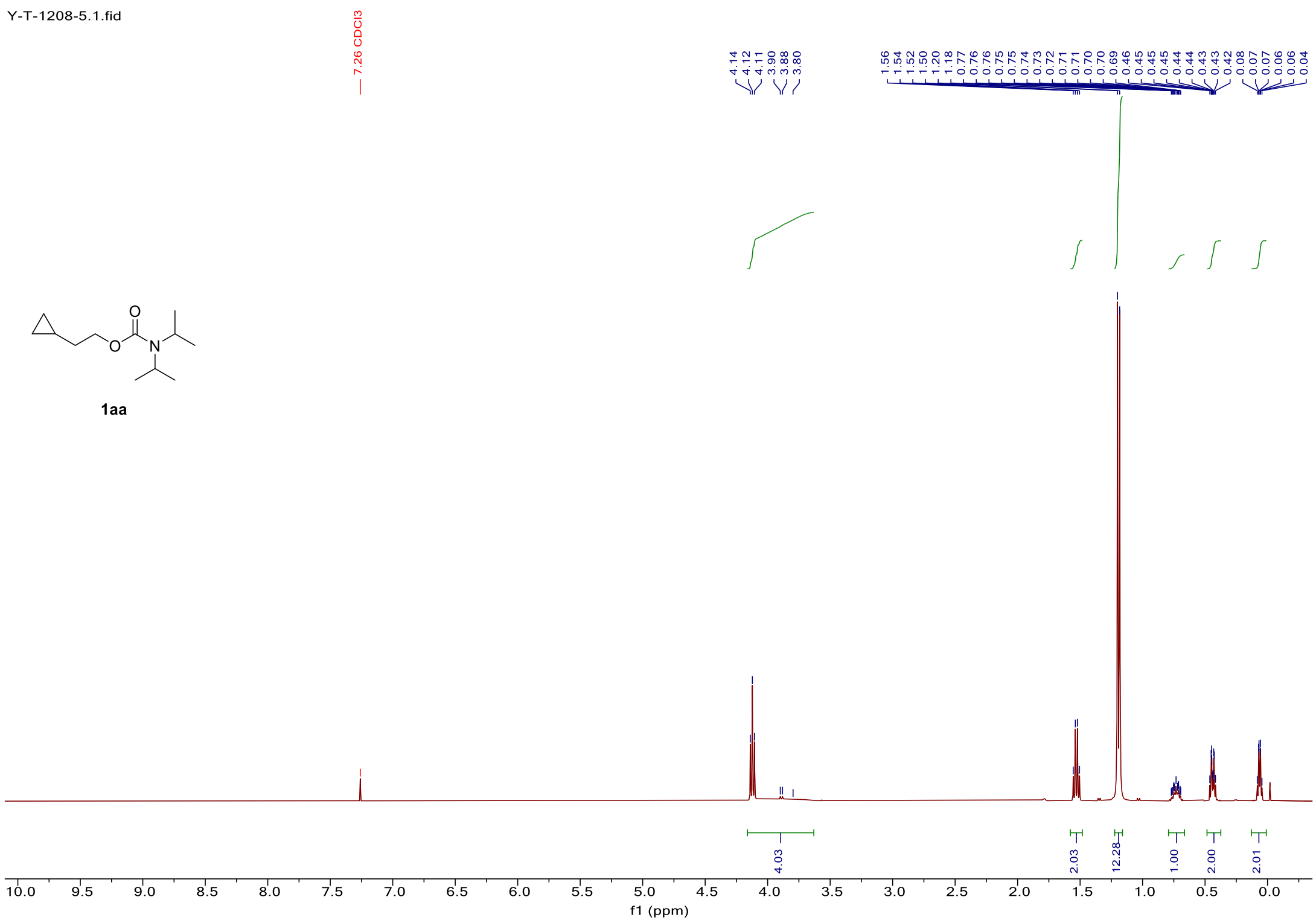

^{1}H NMR (400 MHz, $CDCl_3$)

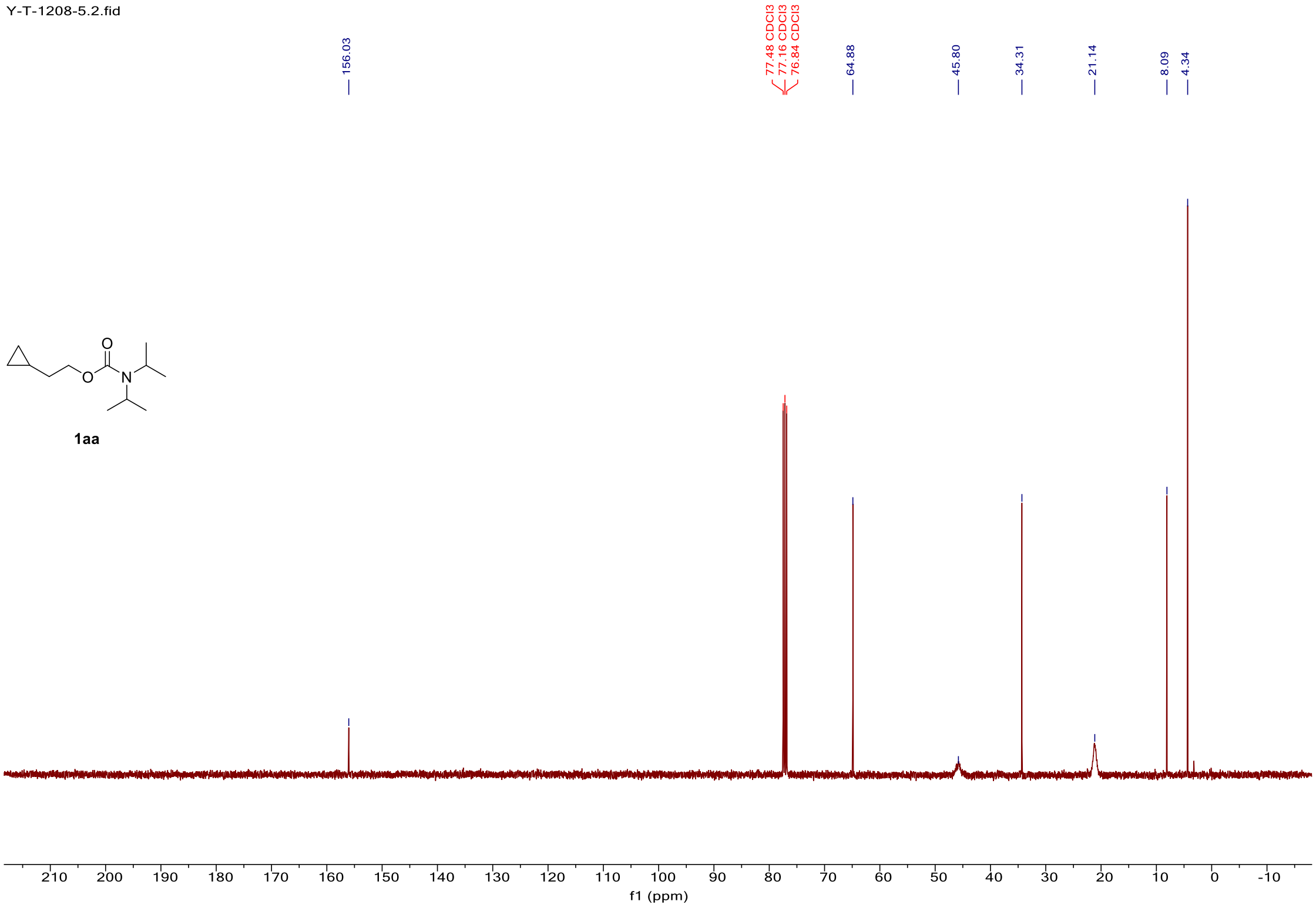

1aa

^{13}C NMR (101 MHz, $CDCl_3$)

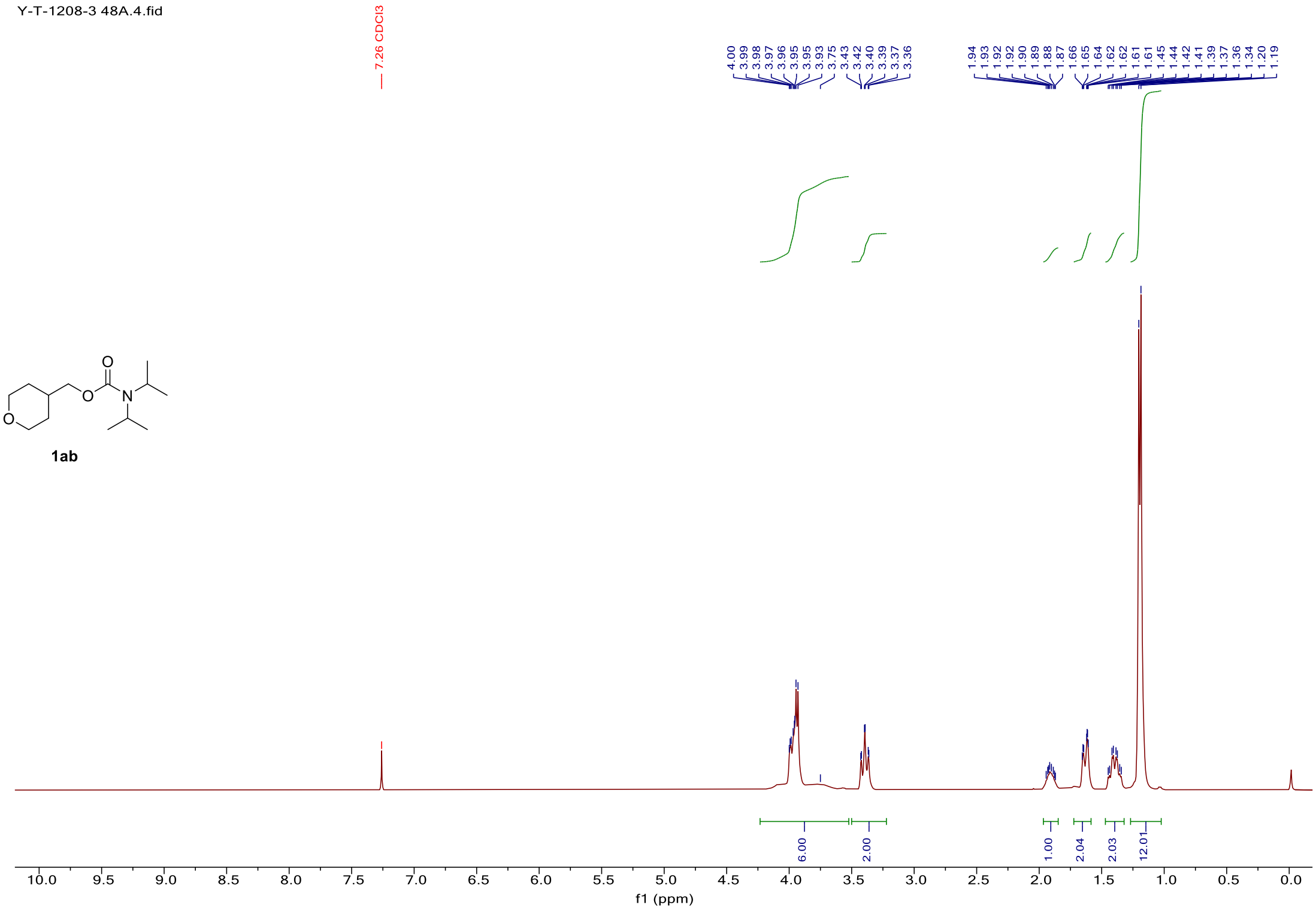

1ab

^{1}H NMR (400 MHz, $CDCl_3$)

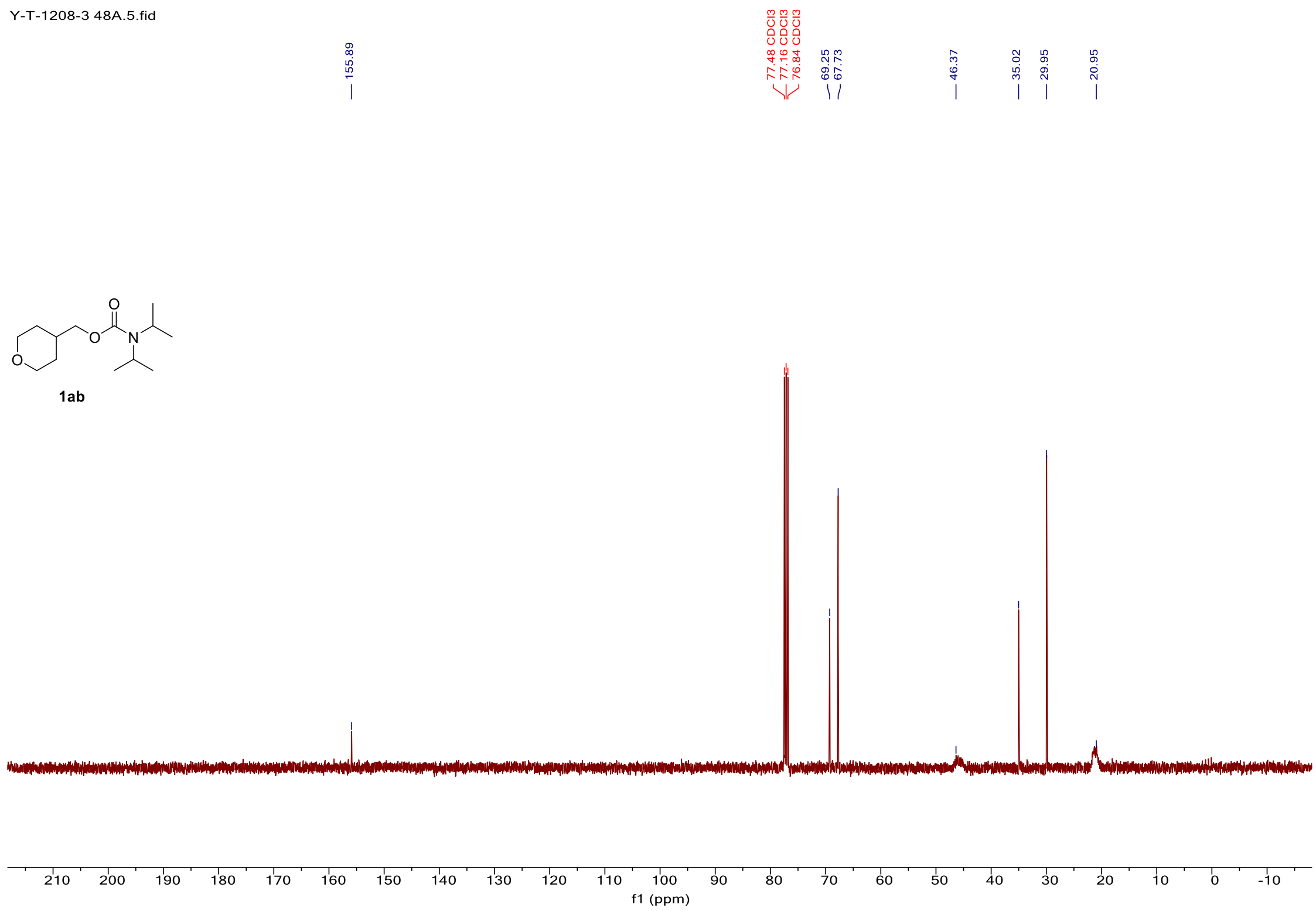

^{13}C NMR (101 MHz, $CDCl_3$)

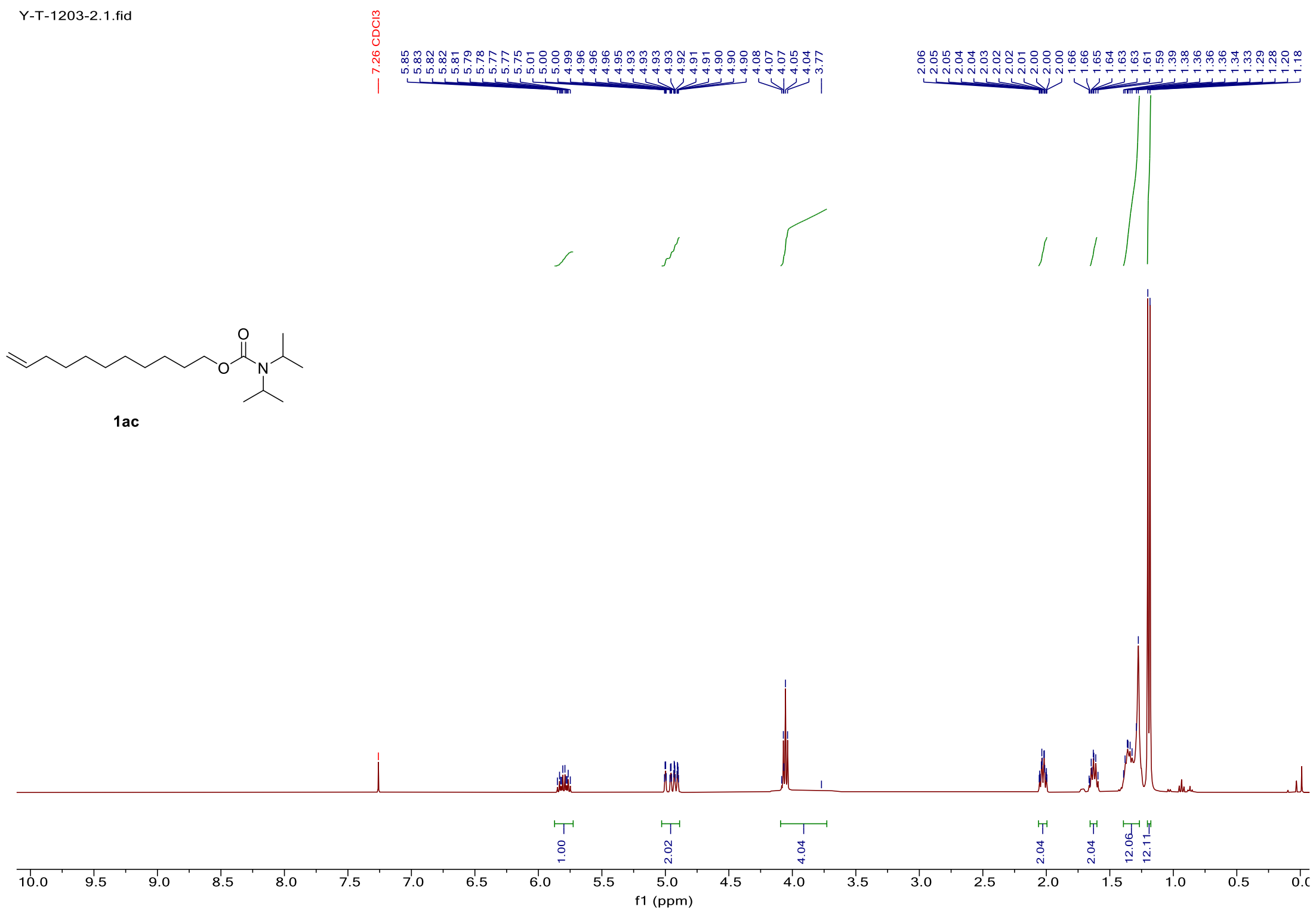

^{1}H NMR (400 MHz, $CDCl_3$)

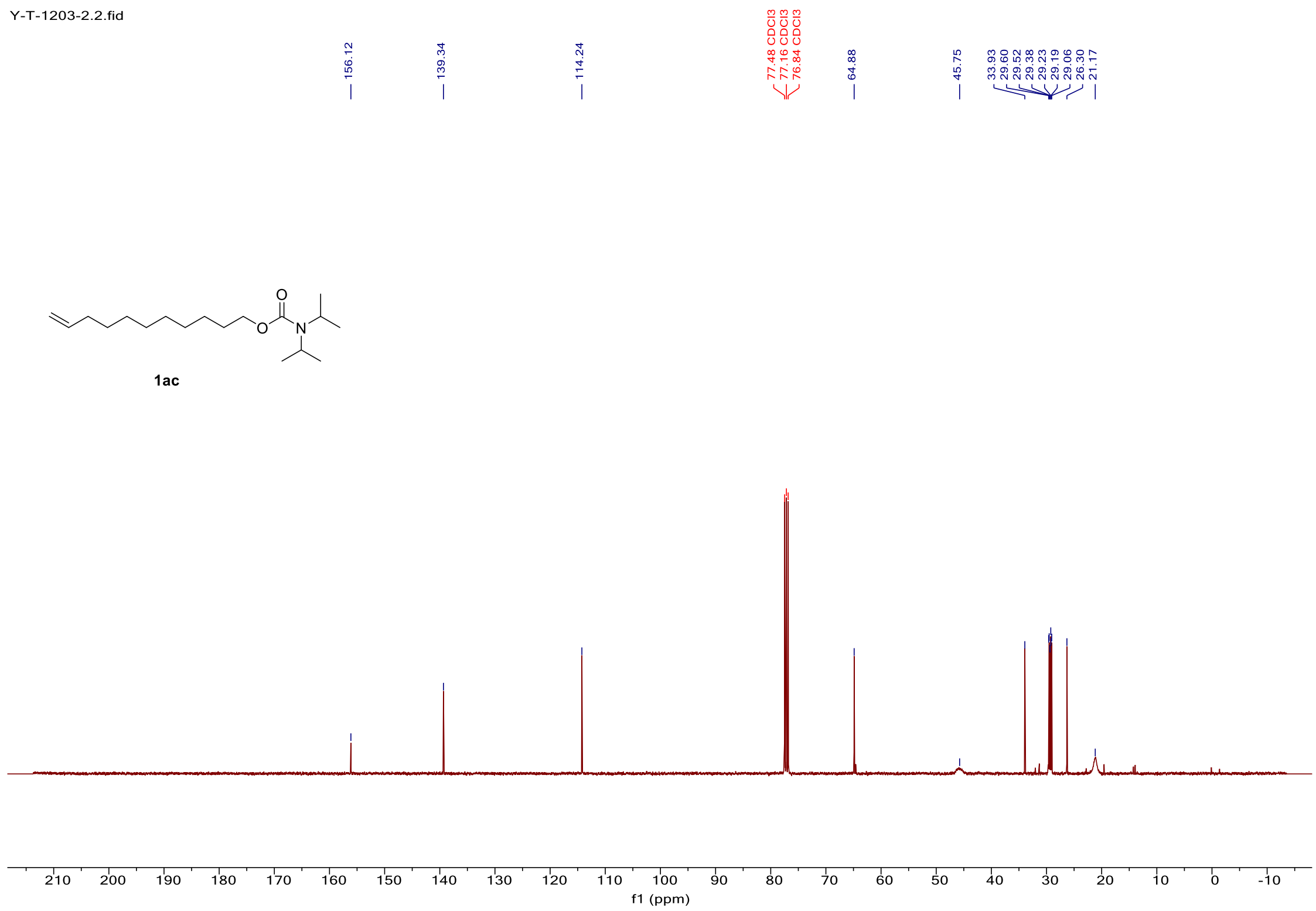

^{13}C NMR (101 MHz, $CDCl_3$)

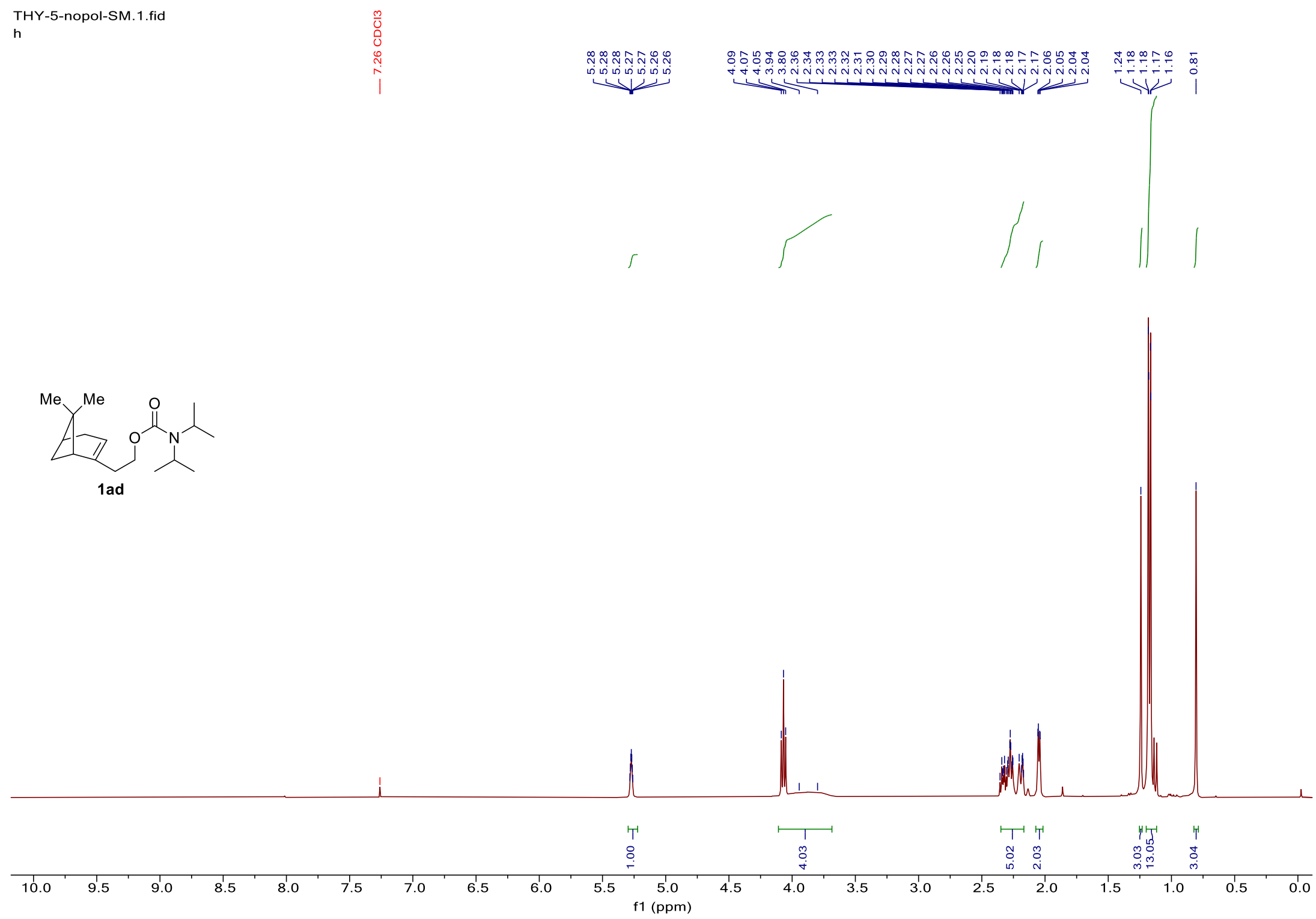

^{1}H NMR (400 MHz, $CDCl_3$)

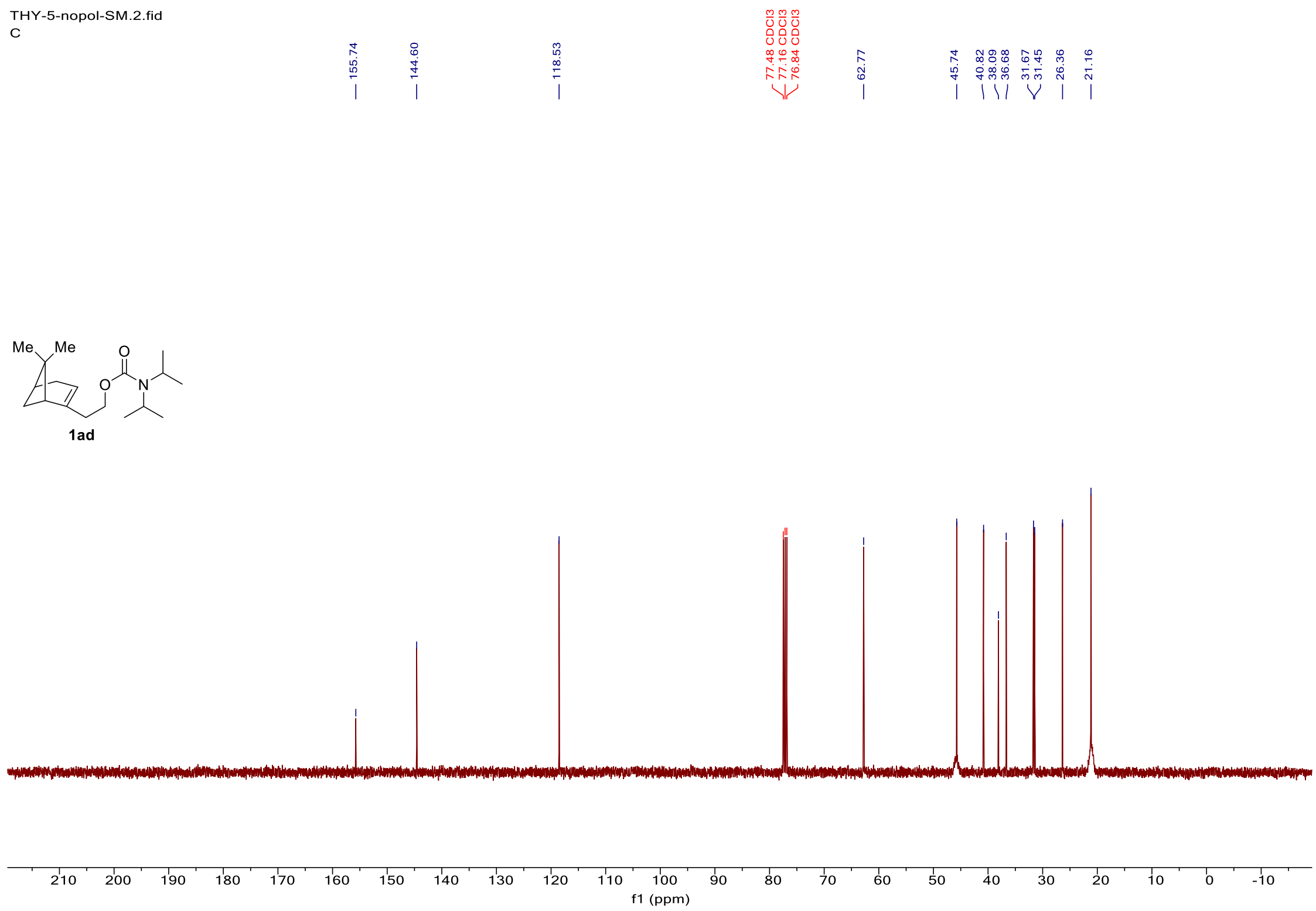

^{13}C NMR (101 MHz, CDCl3)

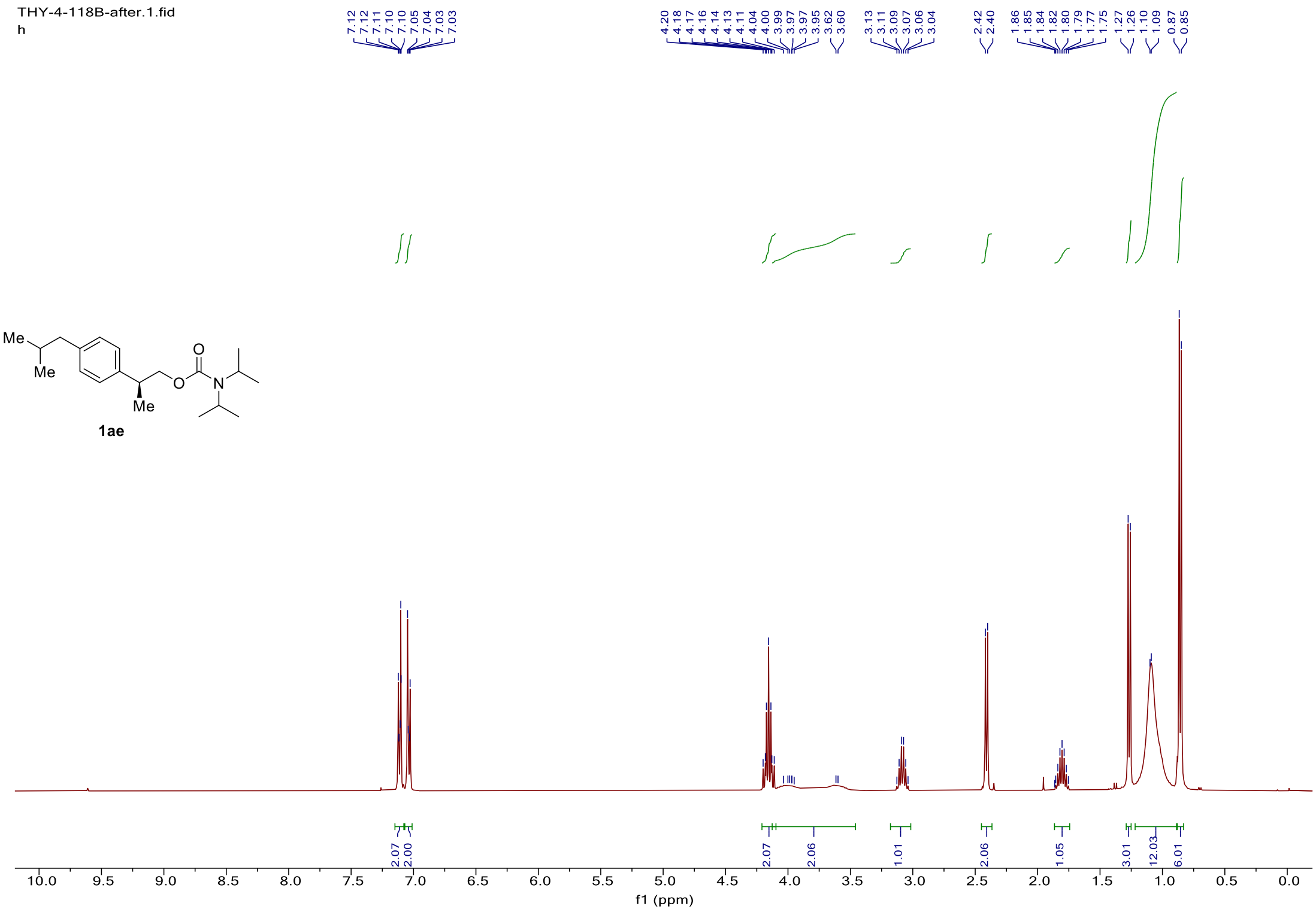

^{1}H NMR (400 MHz, CDCl3)

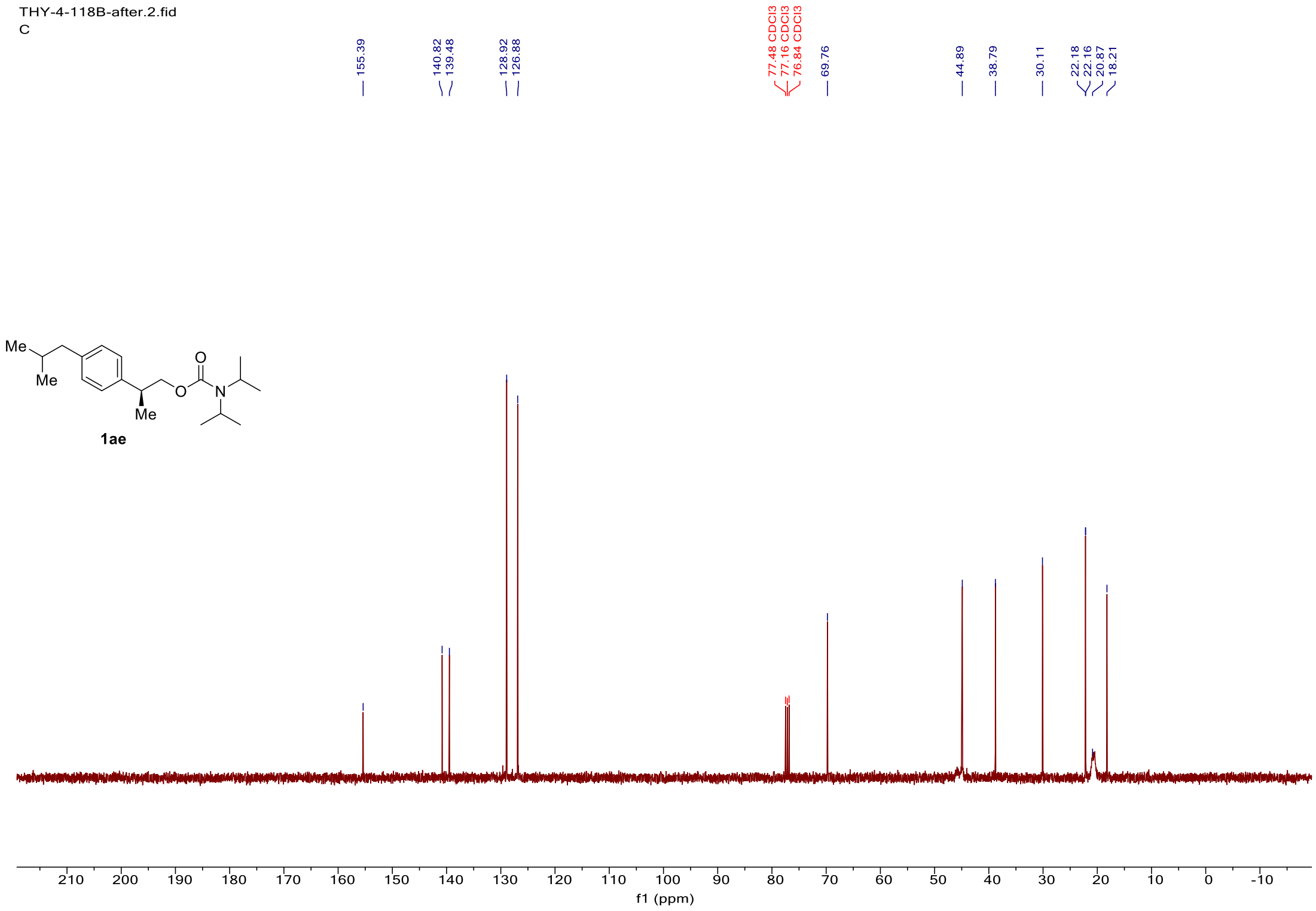

^{13}C NMR (101 MHz, $CDCl_3$)

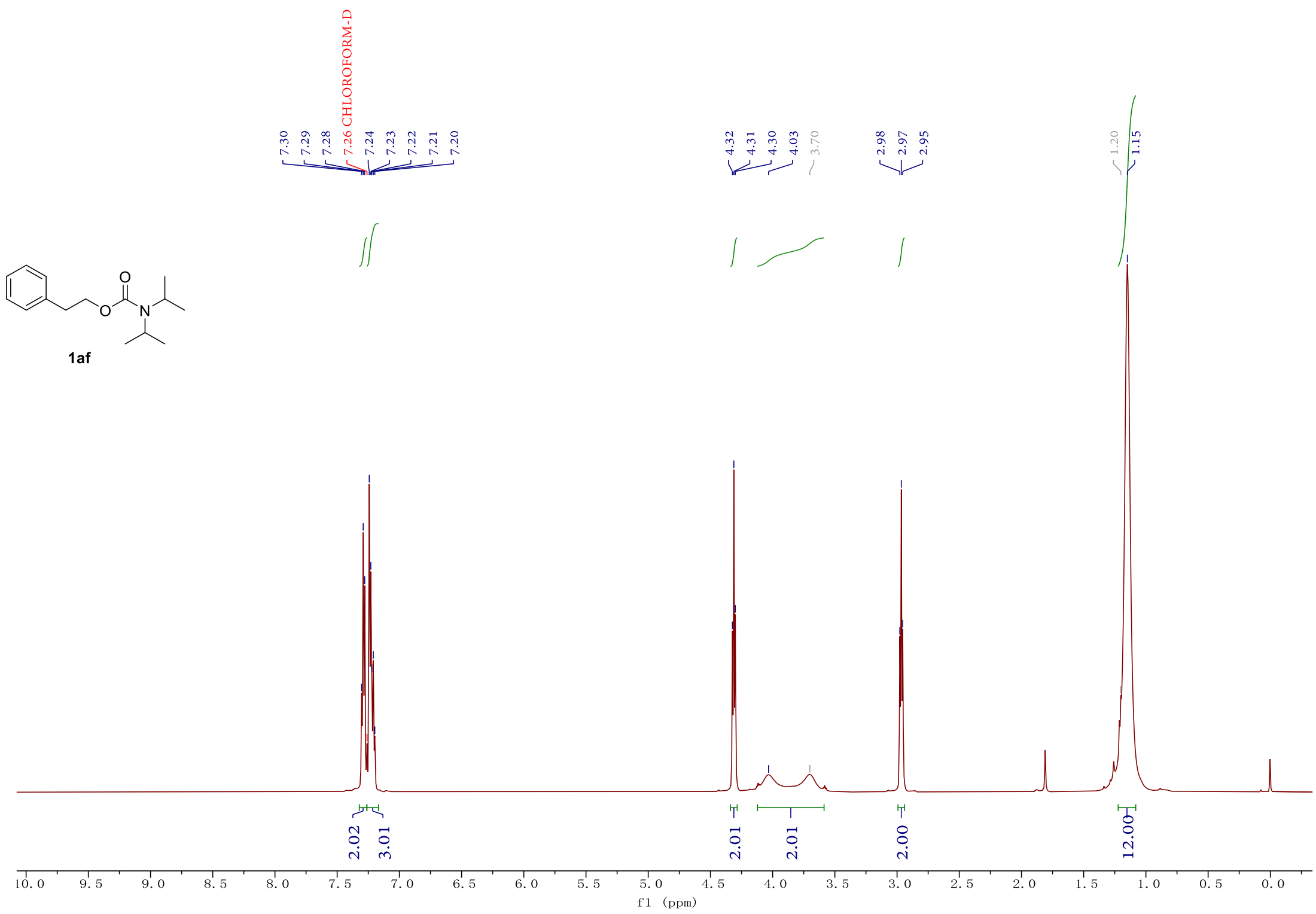

^{1}H NMR (600 MHz, $CDCl_3$)

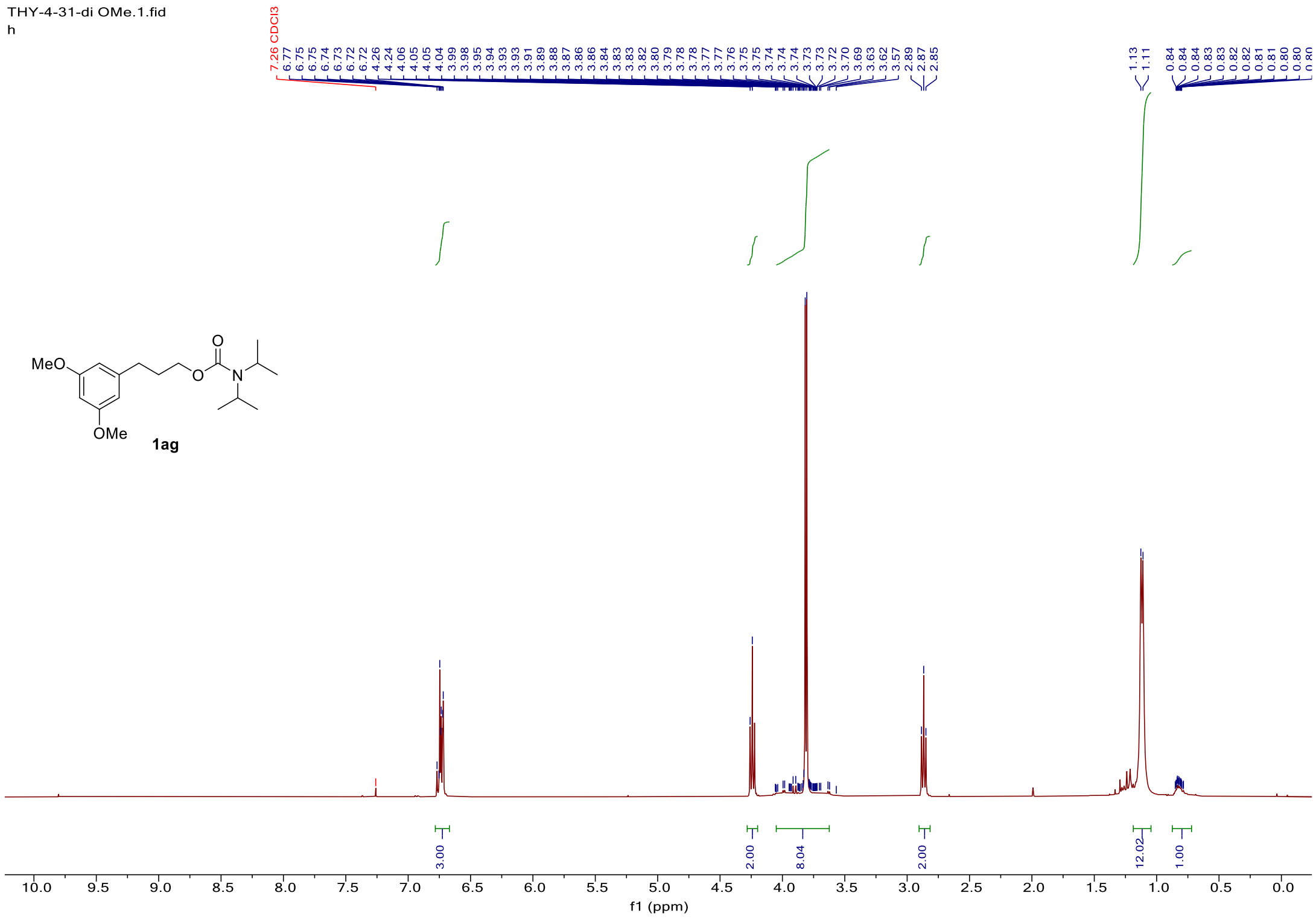

^{1}H NMR (400 MHz, $CDCl_3$)

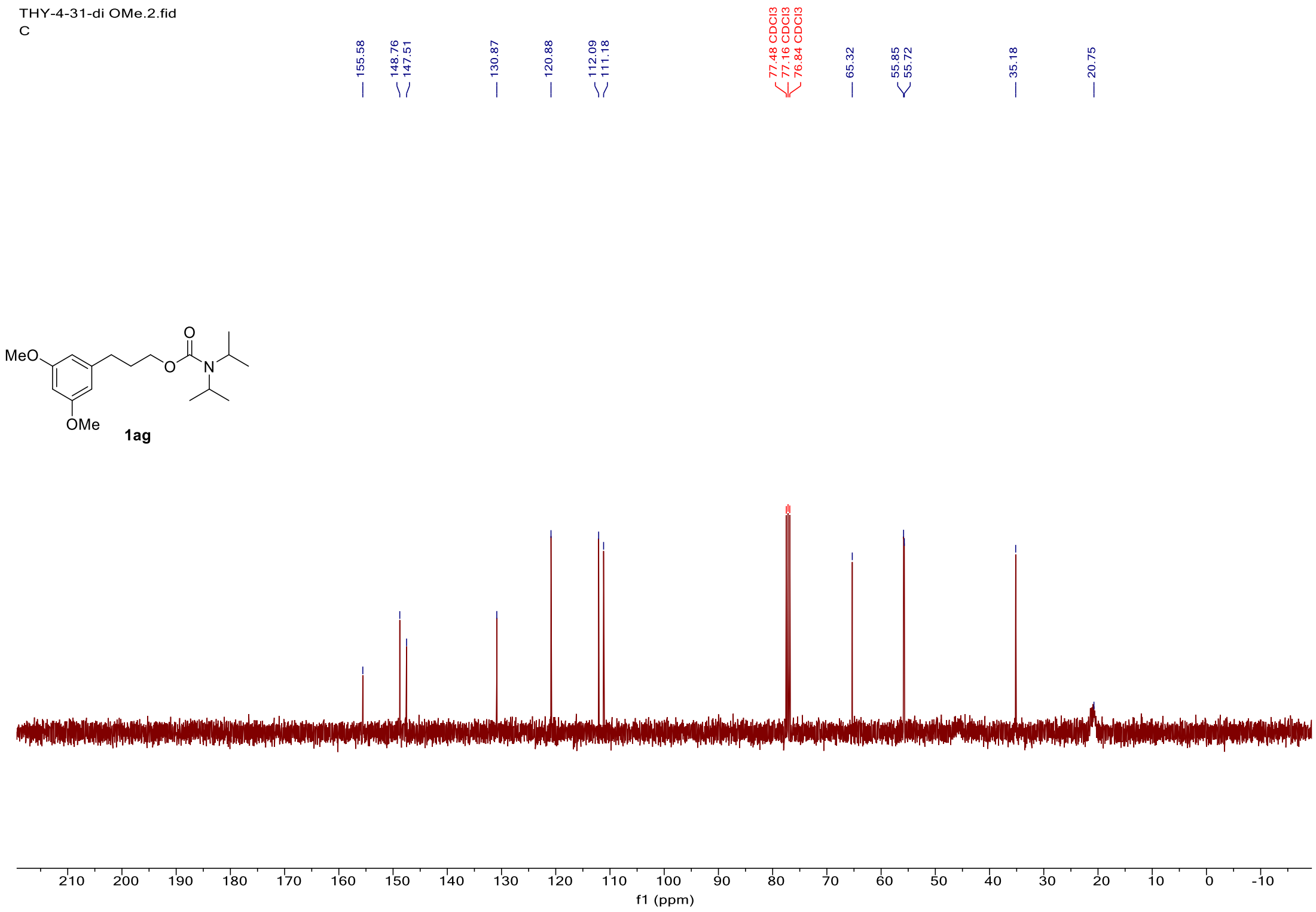

^{13}C NMR (101 MHz, $CDCl_3$)

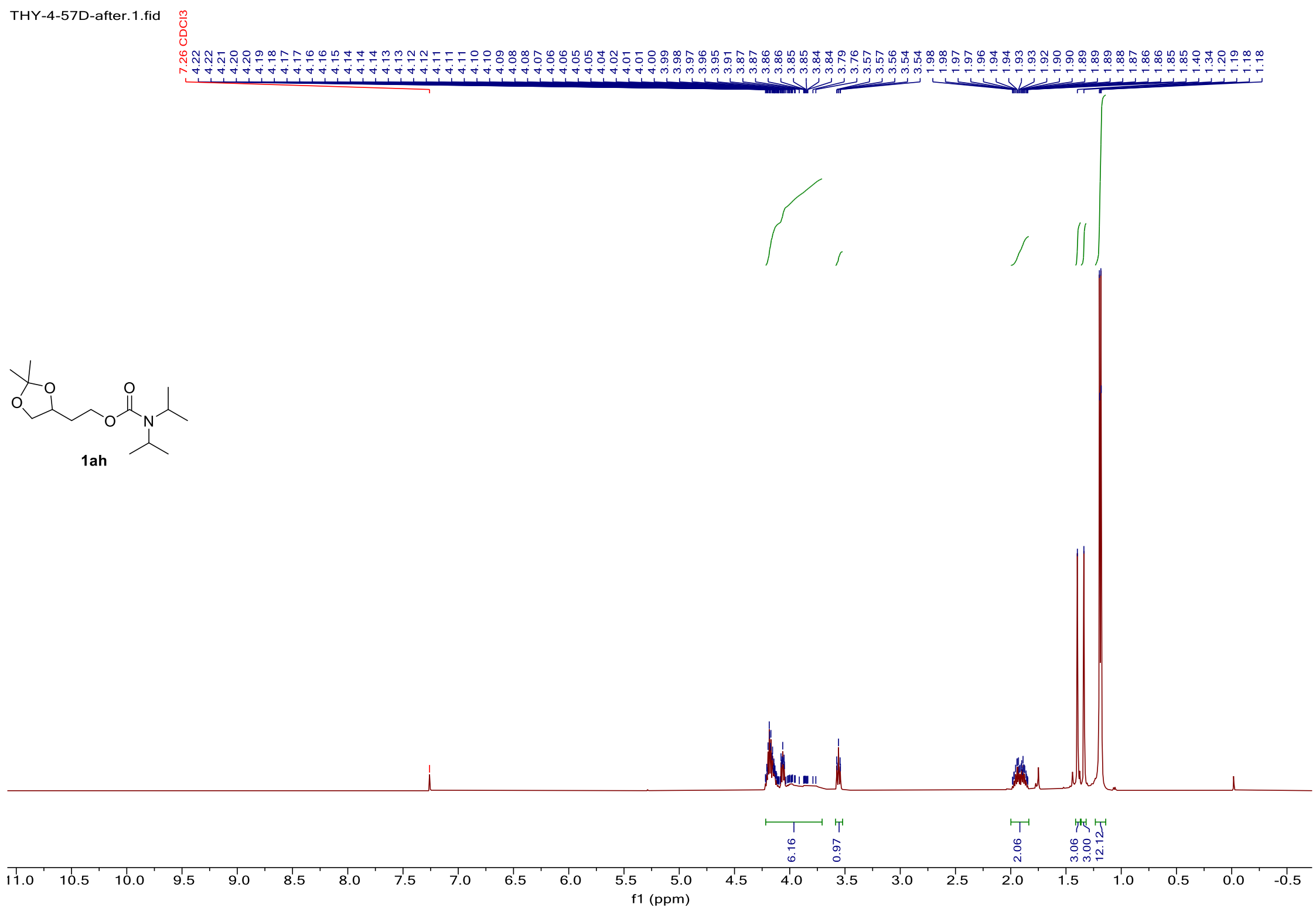

^{1}H NMR (400 MHz, $CDCl_3$)

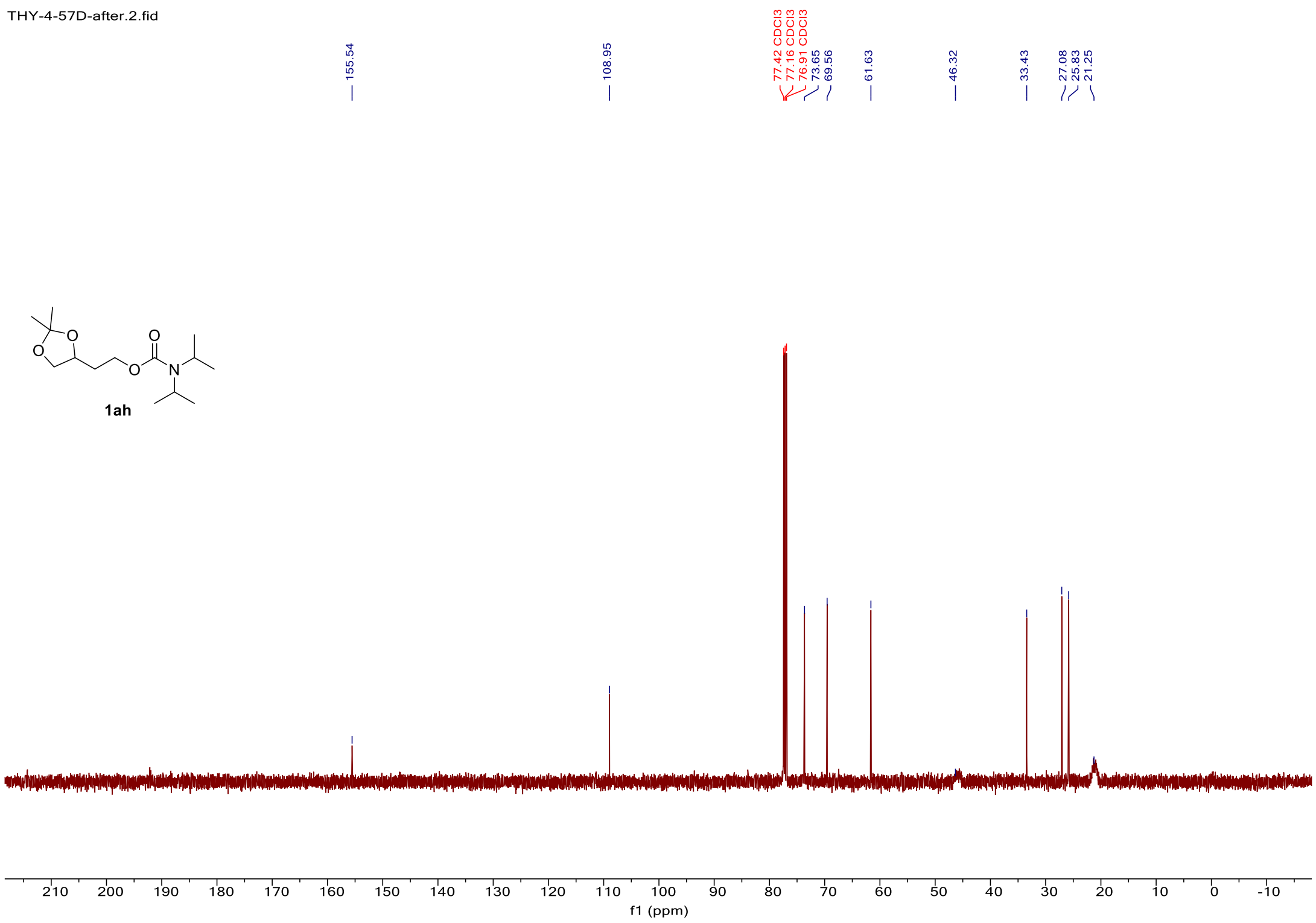

^{13}C NMR (101 MHz, $CDCl_3$)

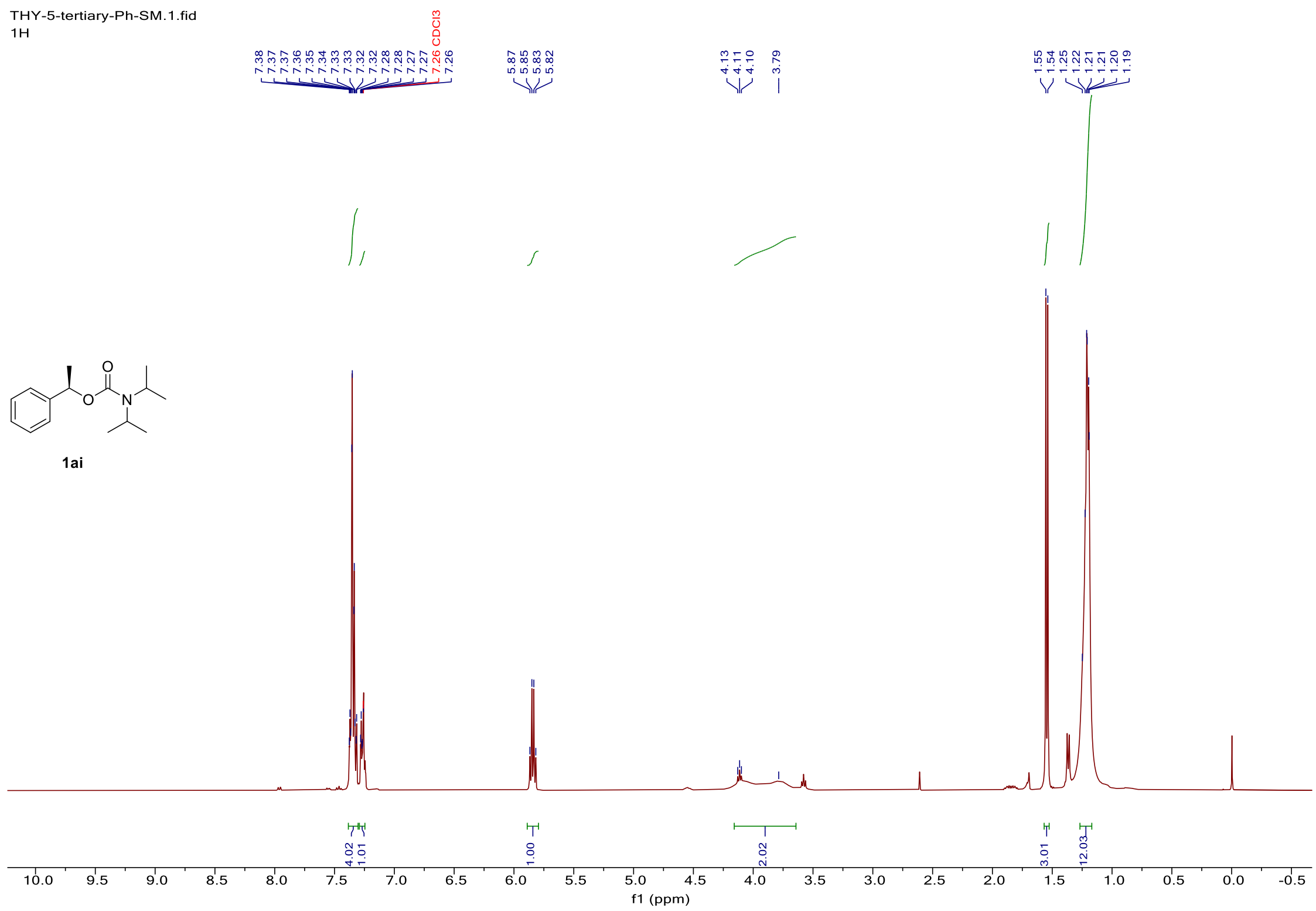

^{1}H NMR (400 MHz, $CDCl_3$)

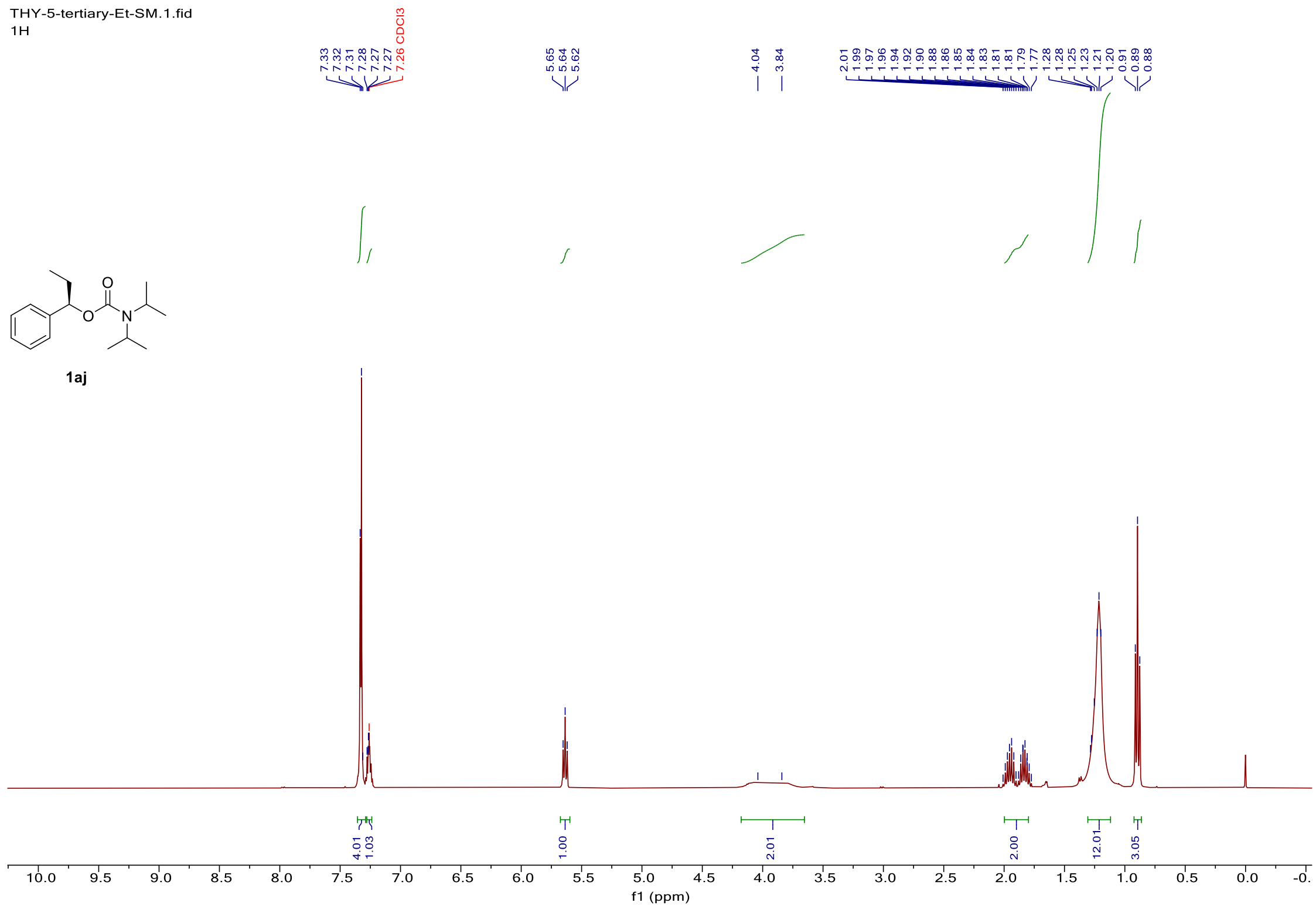

^{1}H NMR (400 MHz, $CDCl_3$)

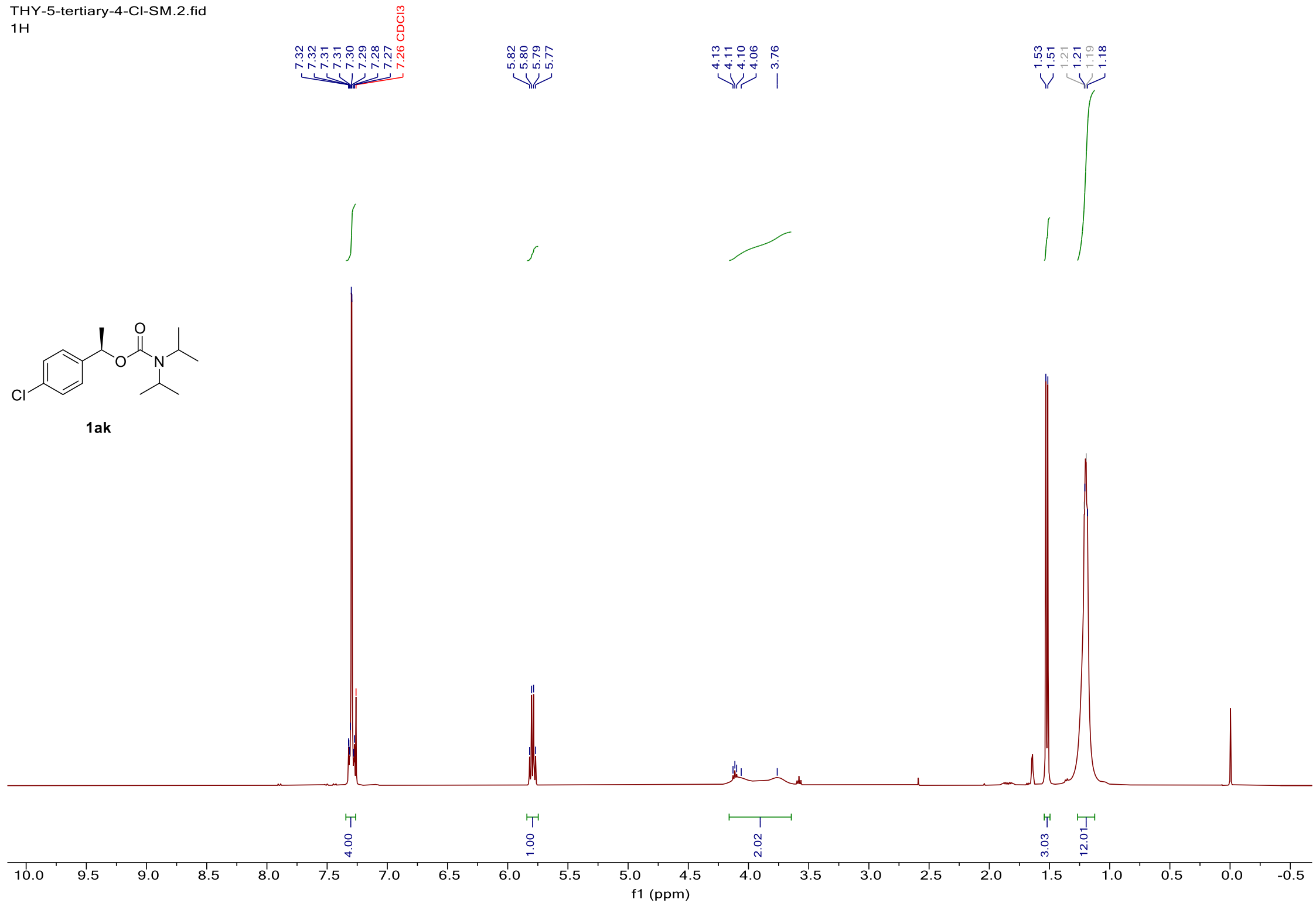

^{1}H NMR (400 MHz, CDCl$_3$)

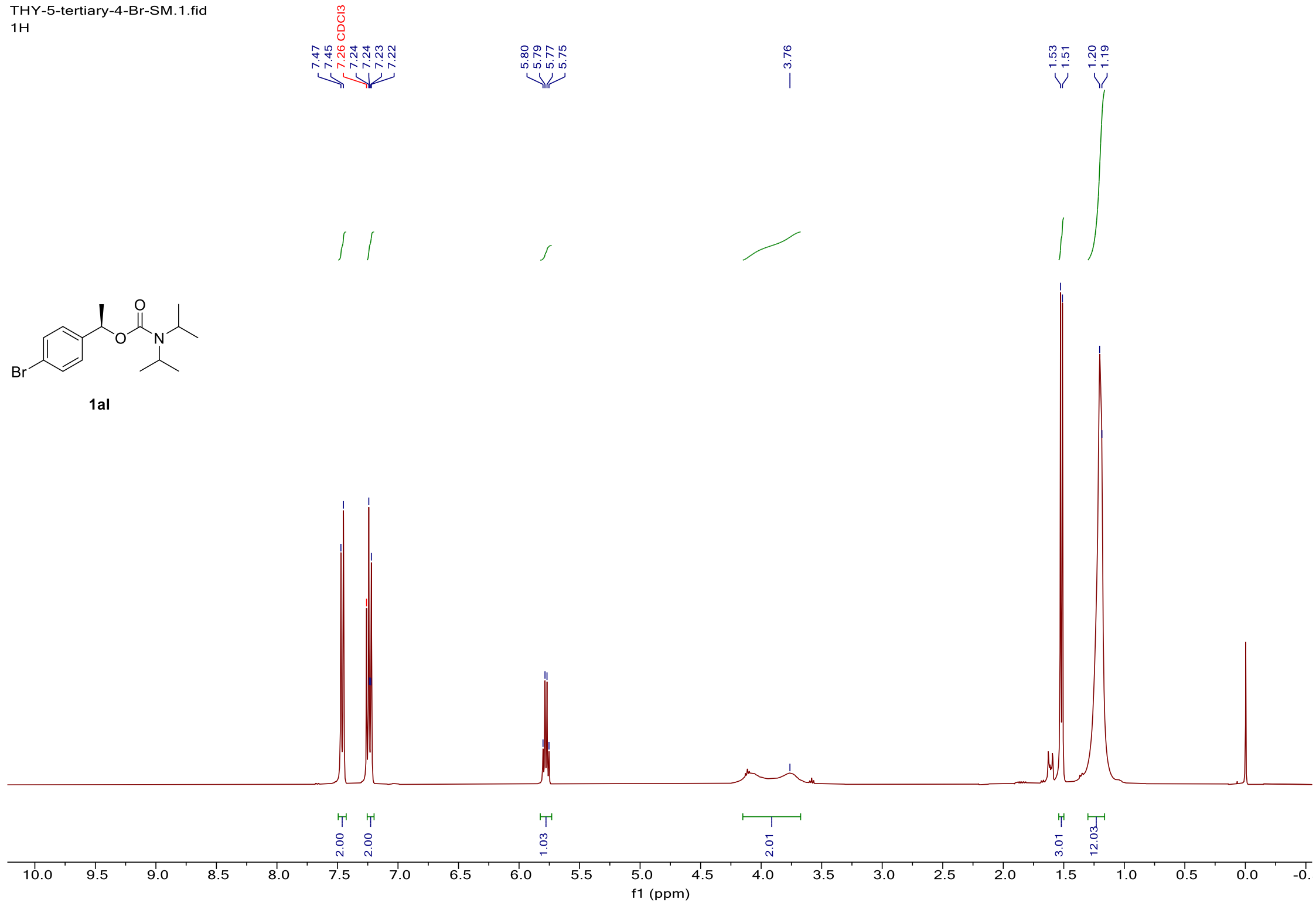

^{1}H NMR (400 MHz, CDCl$_3$)

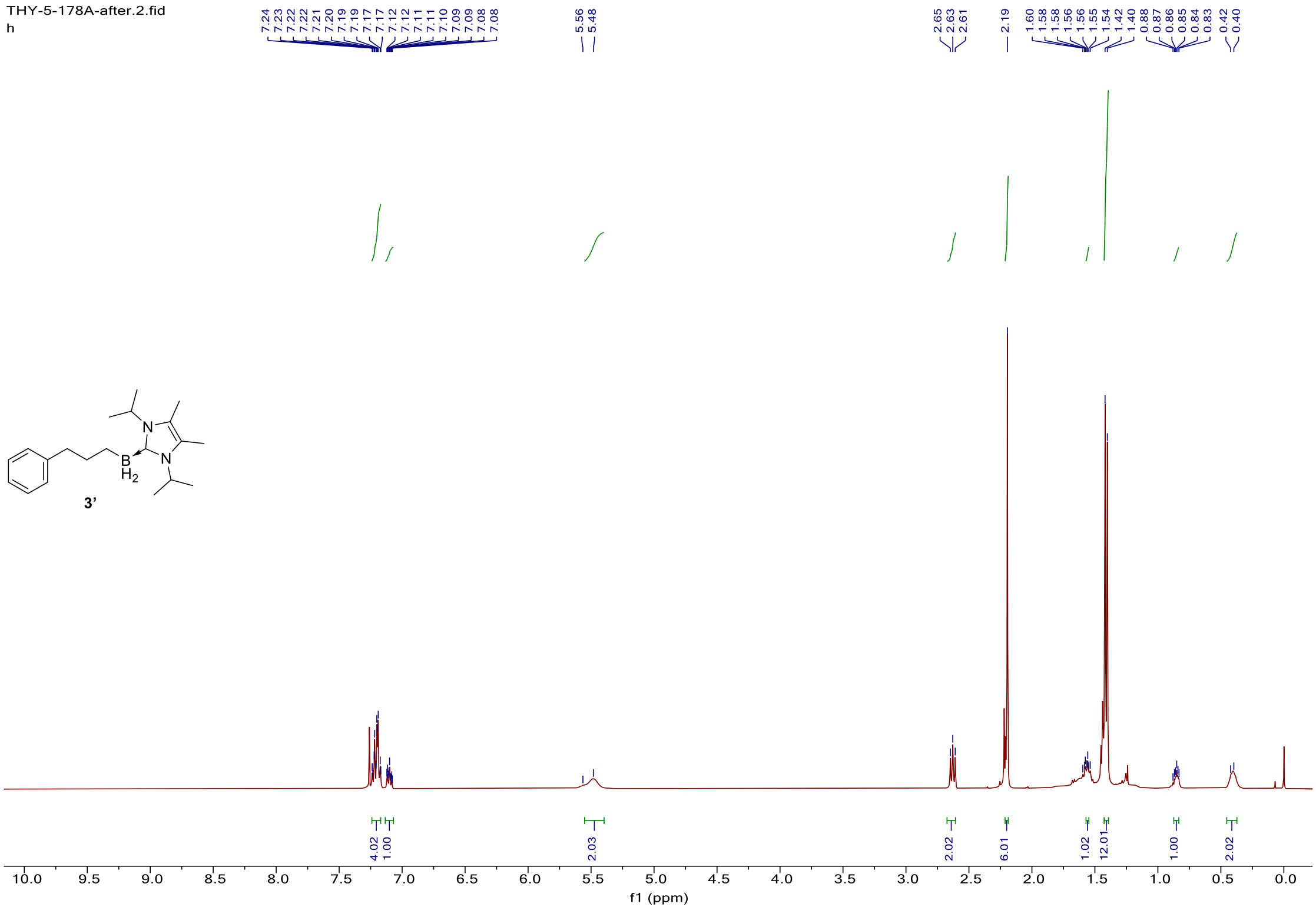

^{1}H NMR (400 MHz, $CDCl_3$)

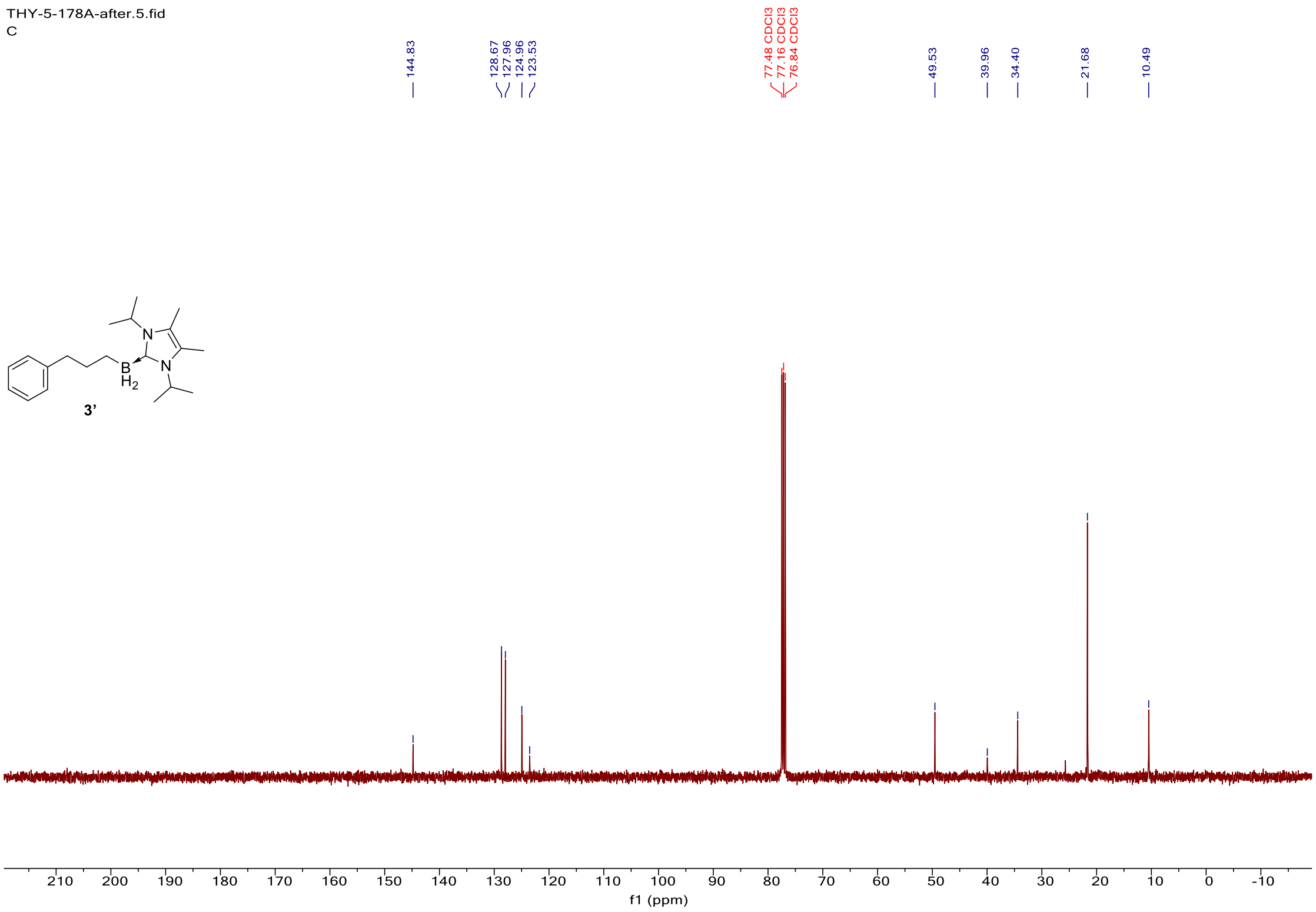

^{13}C NMR (101 MHz, $CDCl_3$)

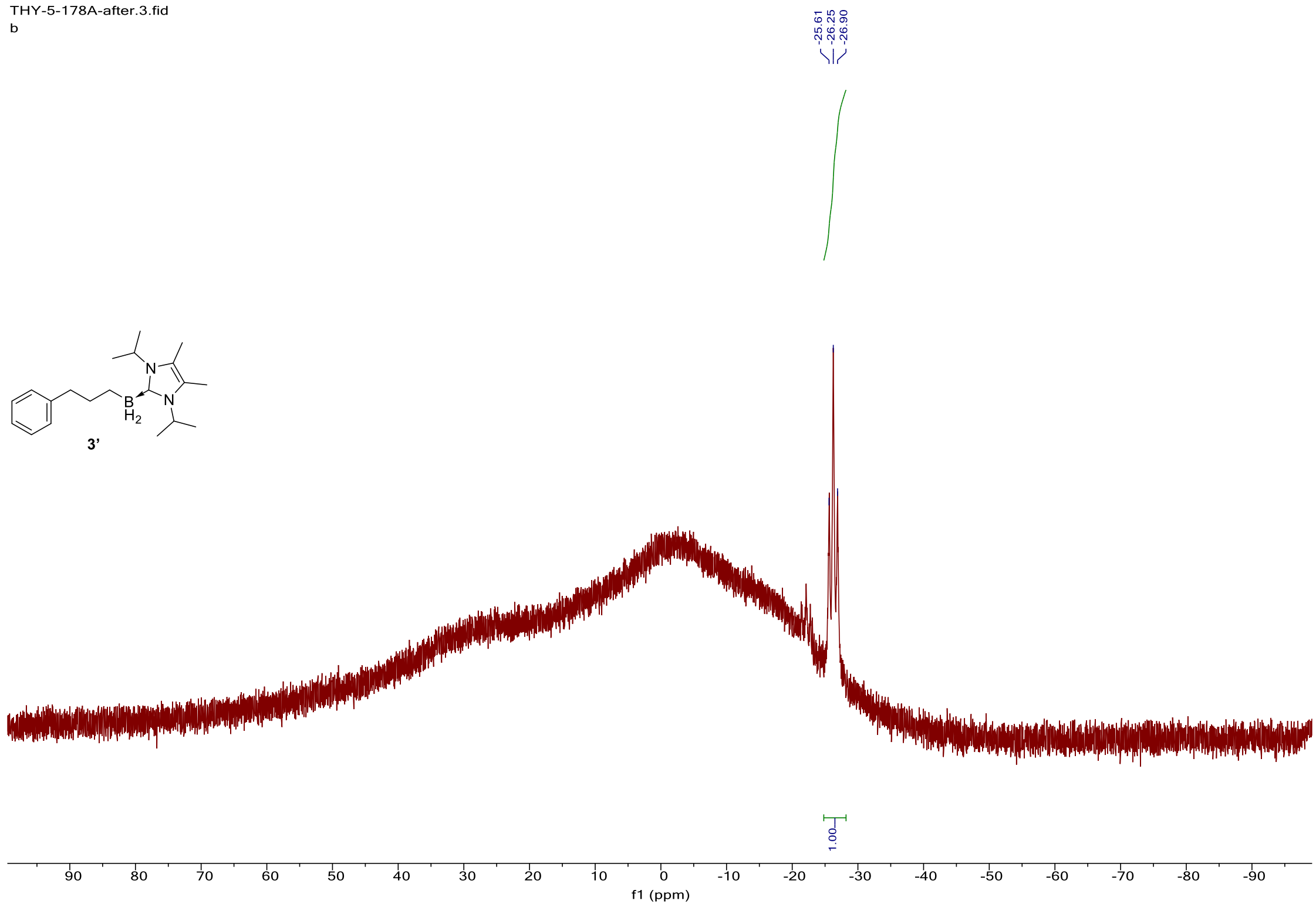

^{11}B NMR (128 MHz, $CDCl_3$)

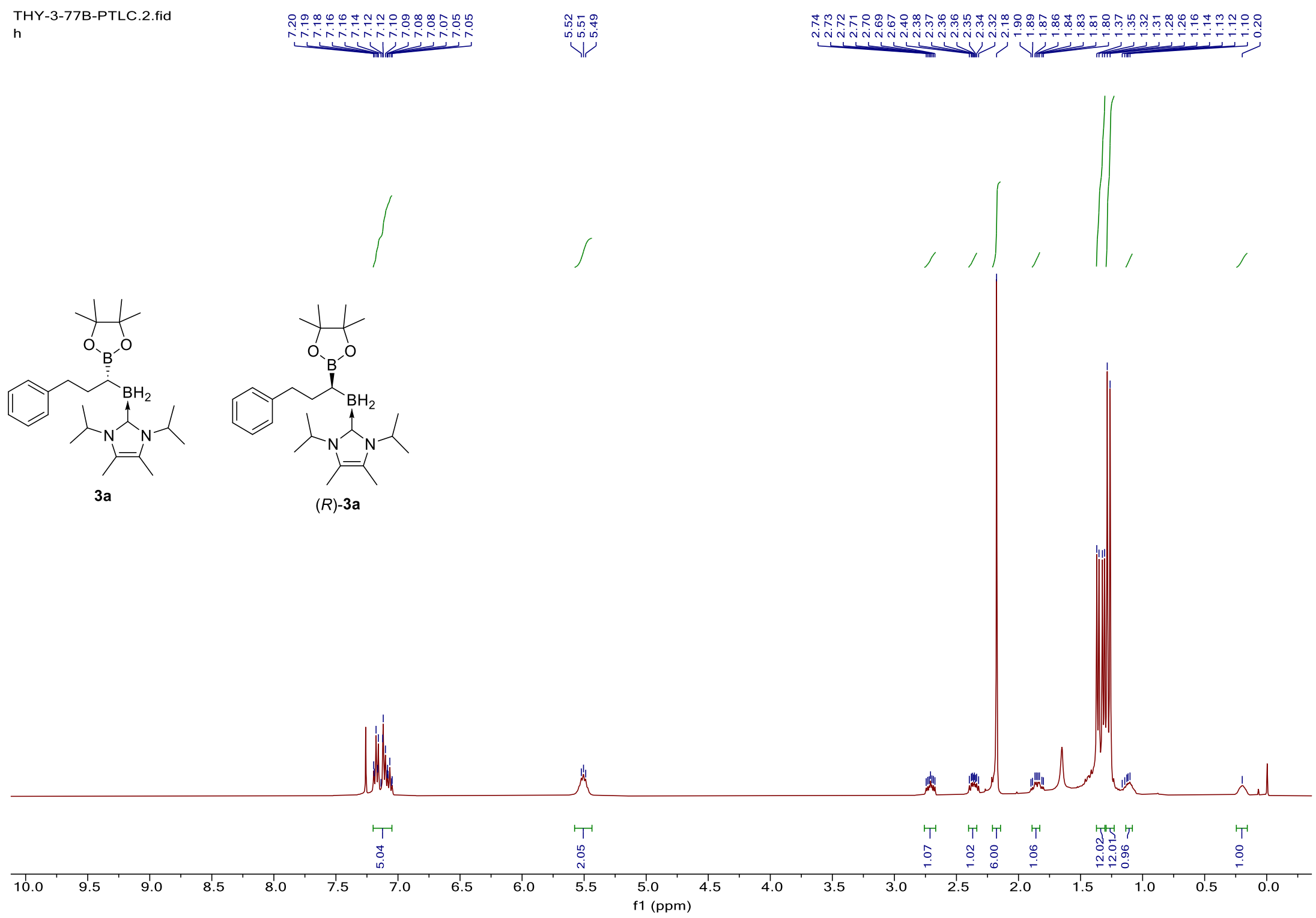

^{1}H NMR (400 MHz, $CDCl_3$)

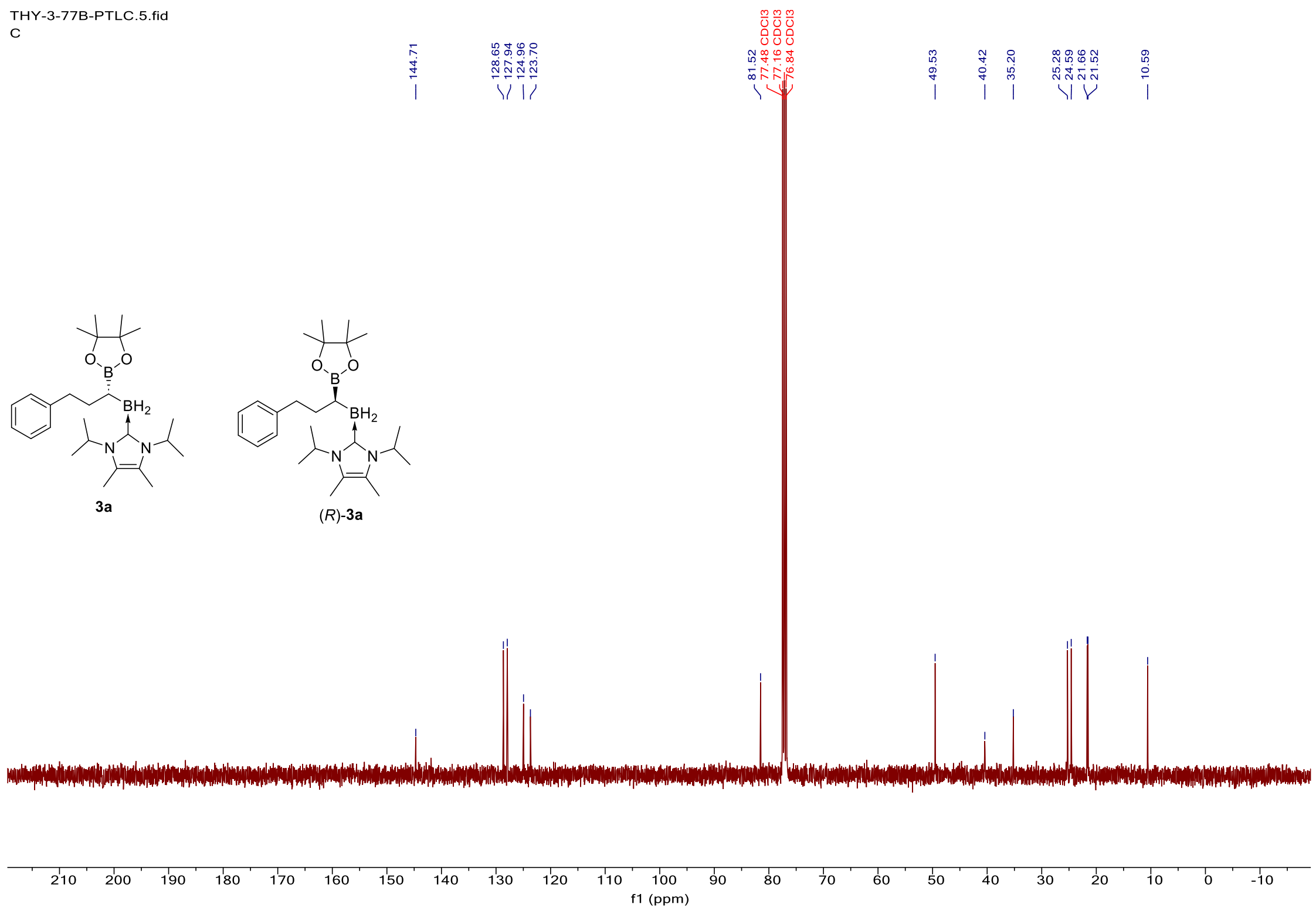

^{13}C NMR (101 MHz, $CDCl_3$)

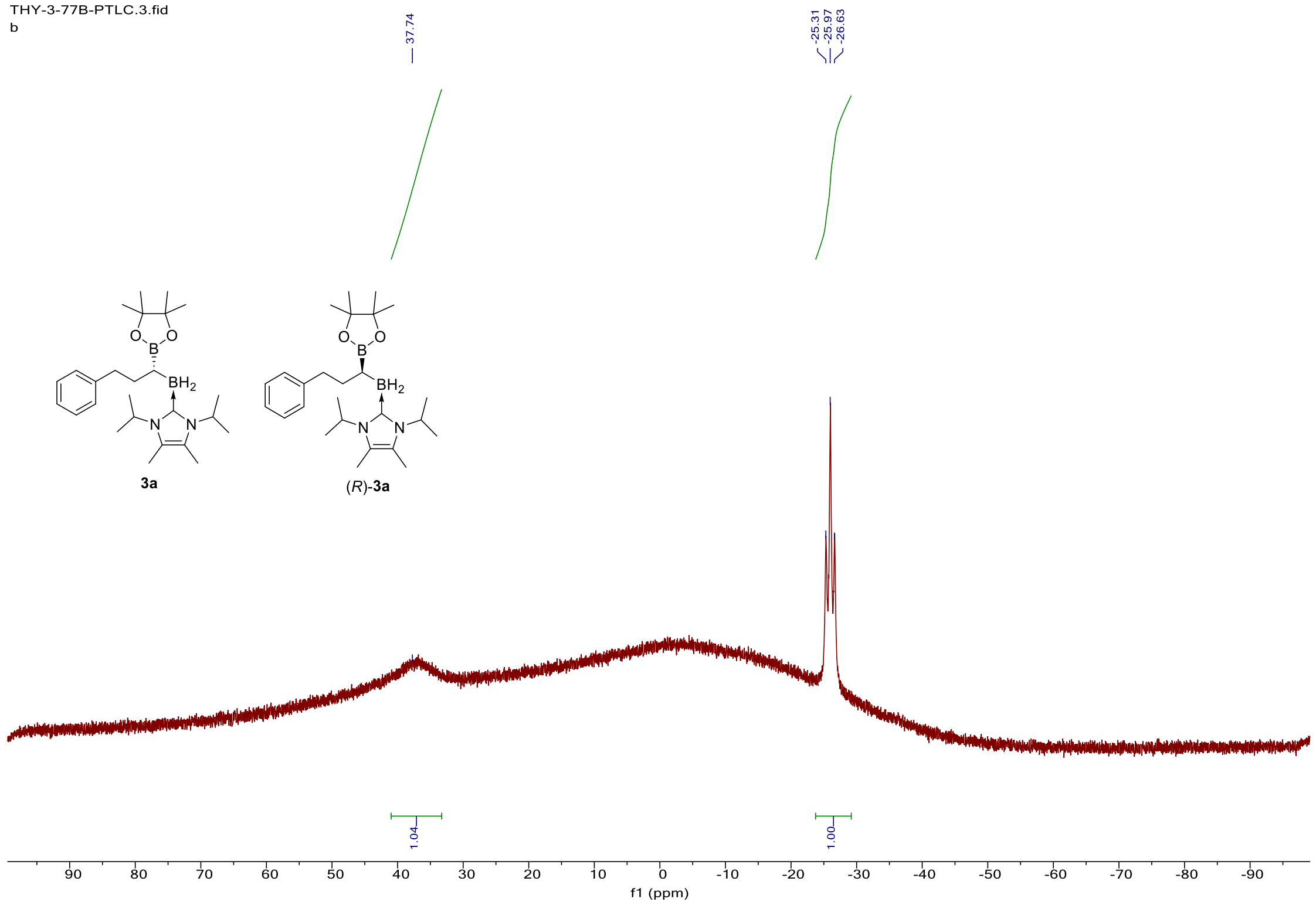

^{11}B NMR (128 MHz, $CDCl_3$)

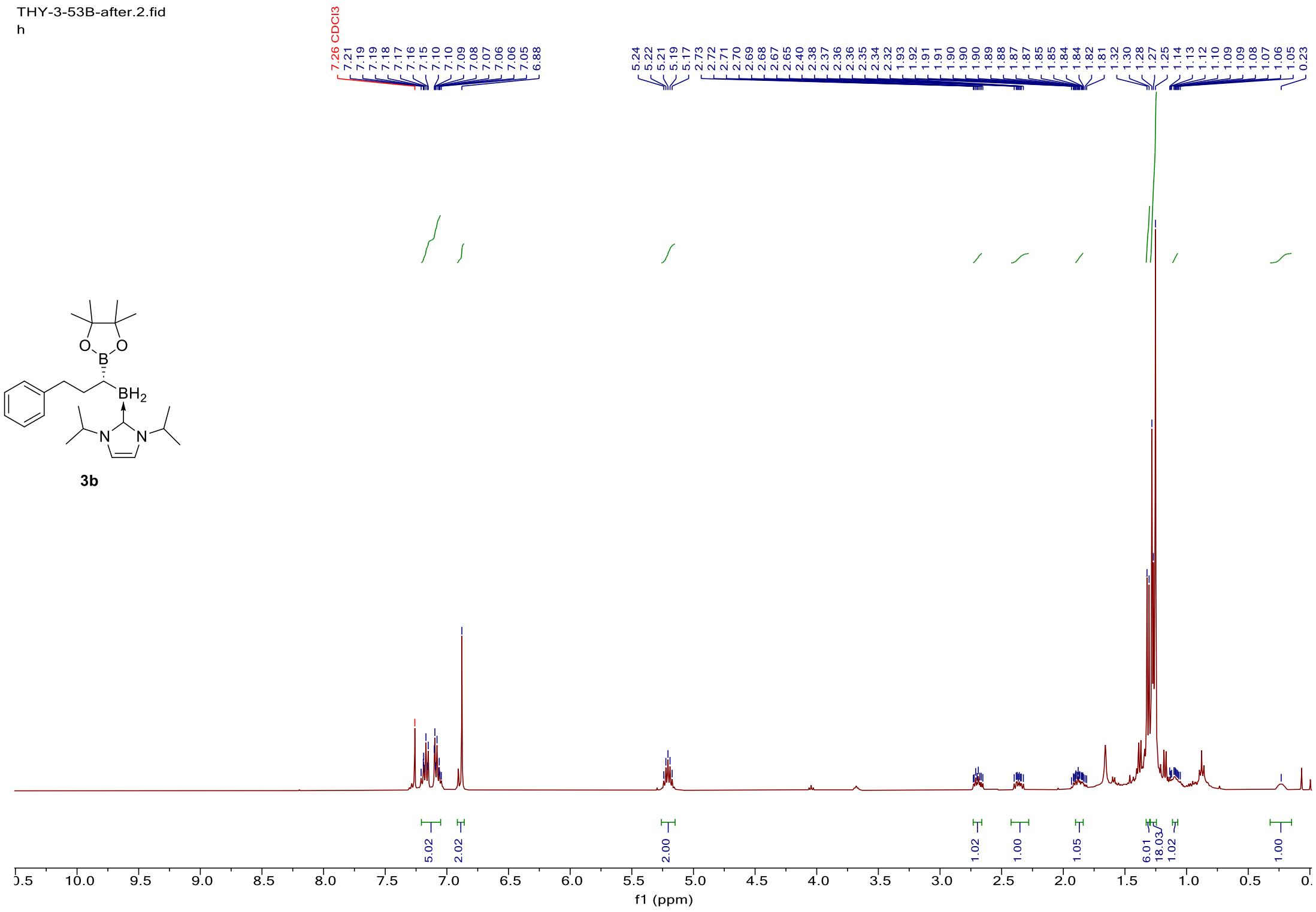

^{1}H NMR (400 MHz, $CDCl_3$)

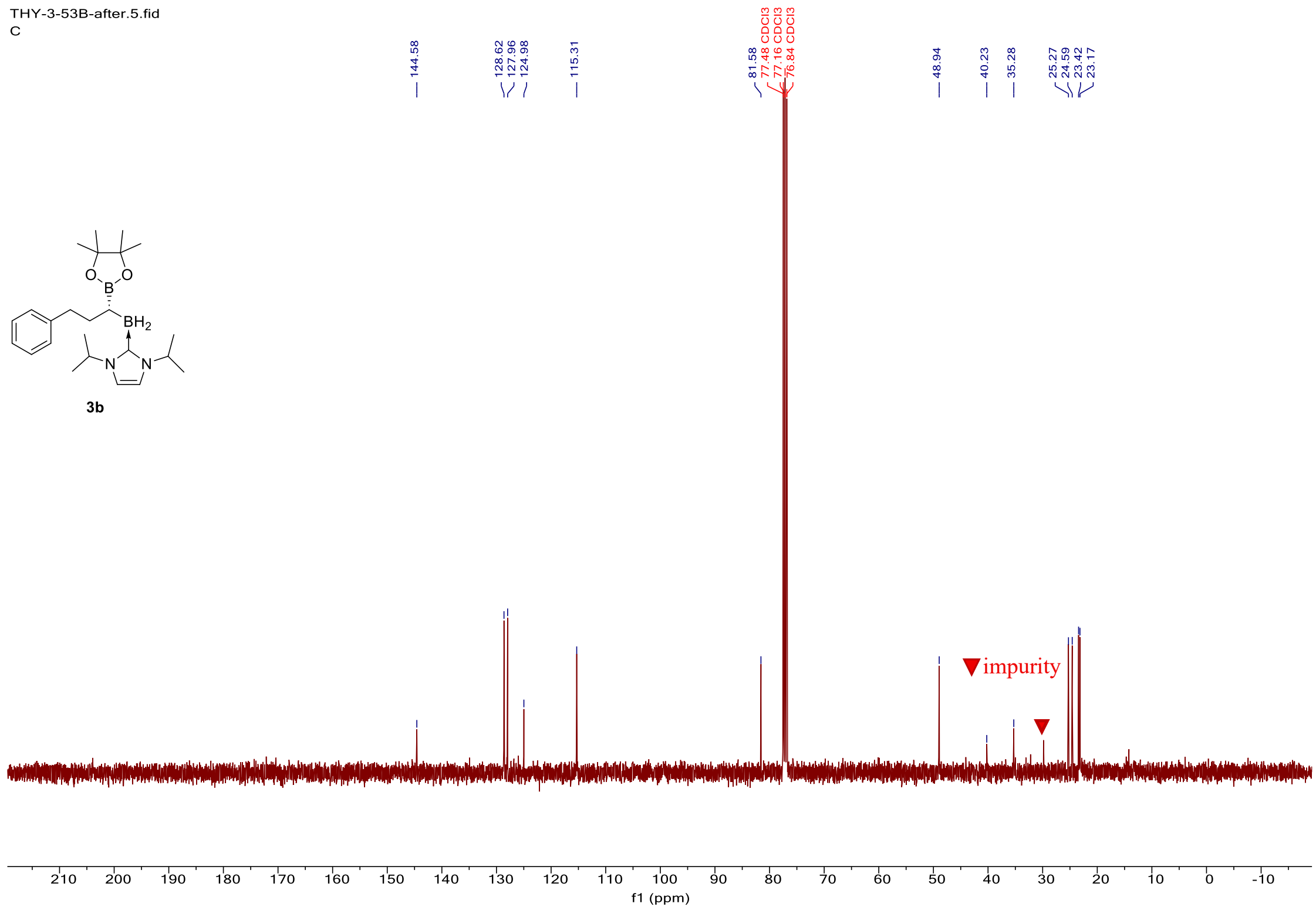

^{13}C NMR (101 MHz, $CDCl_3$)

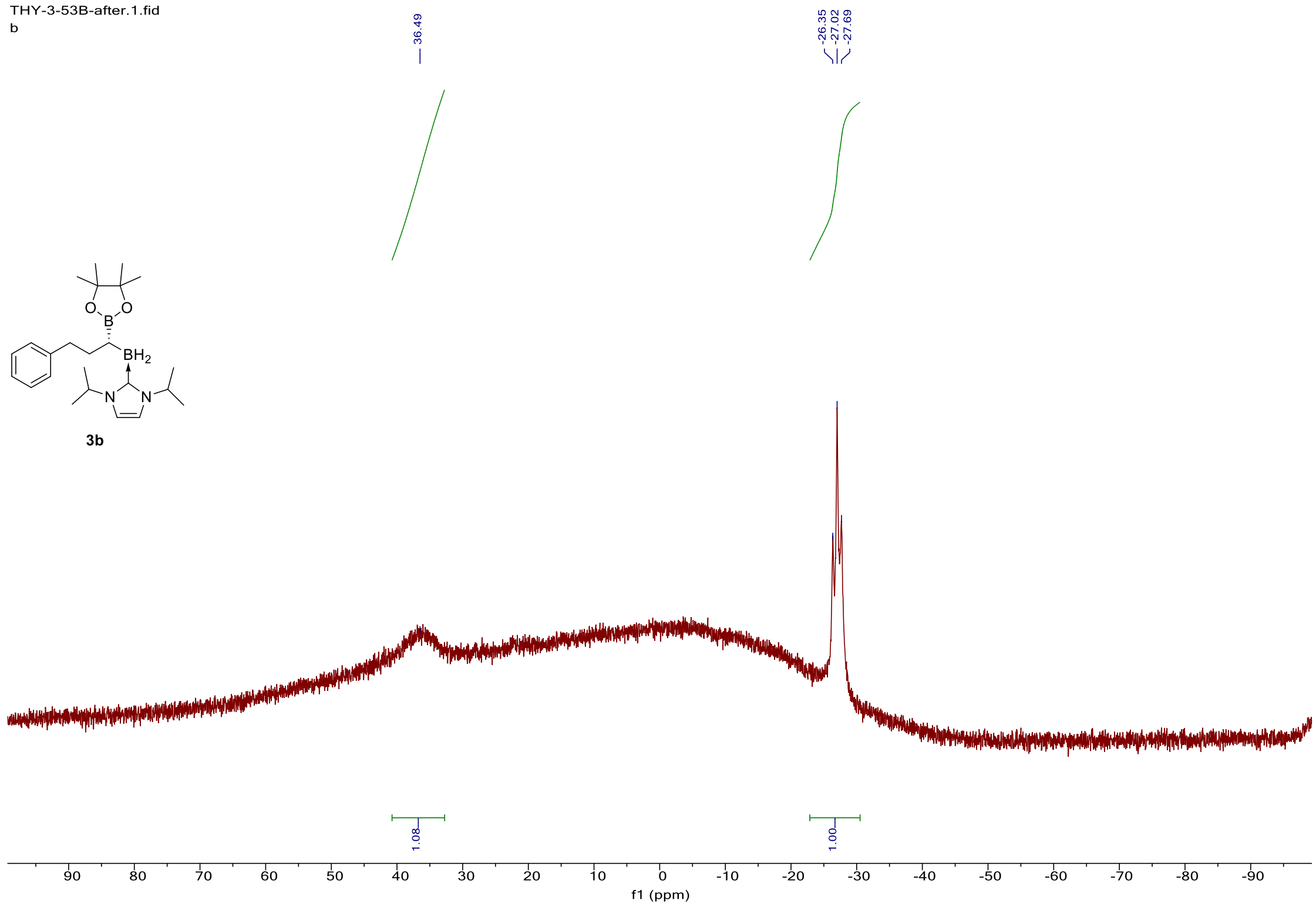

^{11}B NMR (128 MHz, CDCl$_3$)

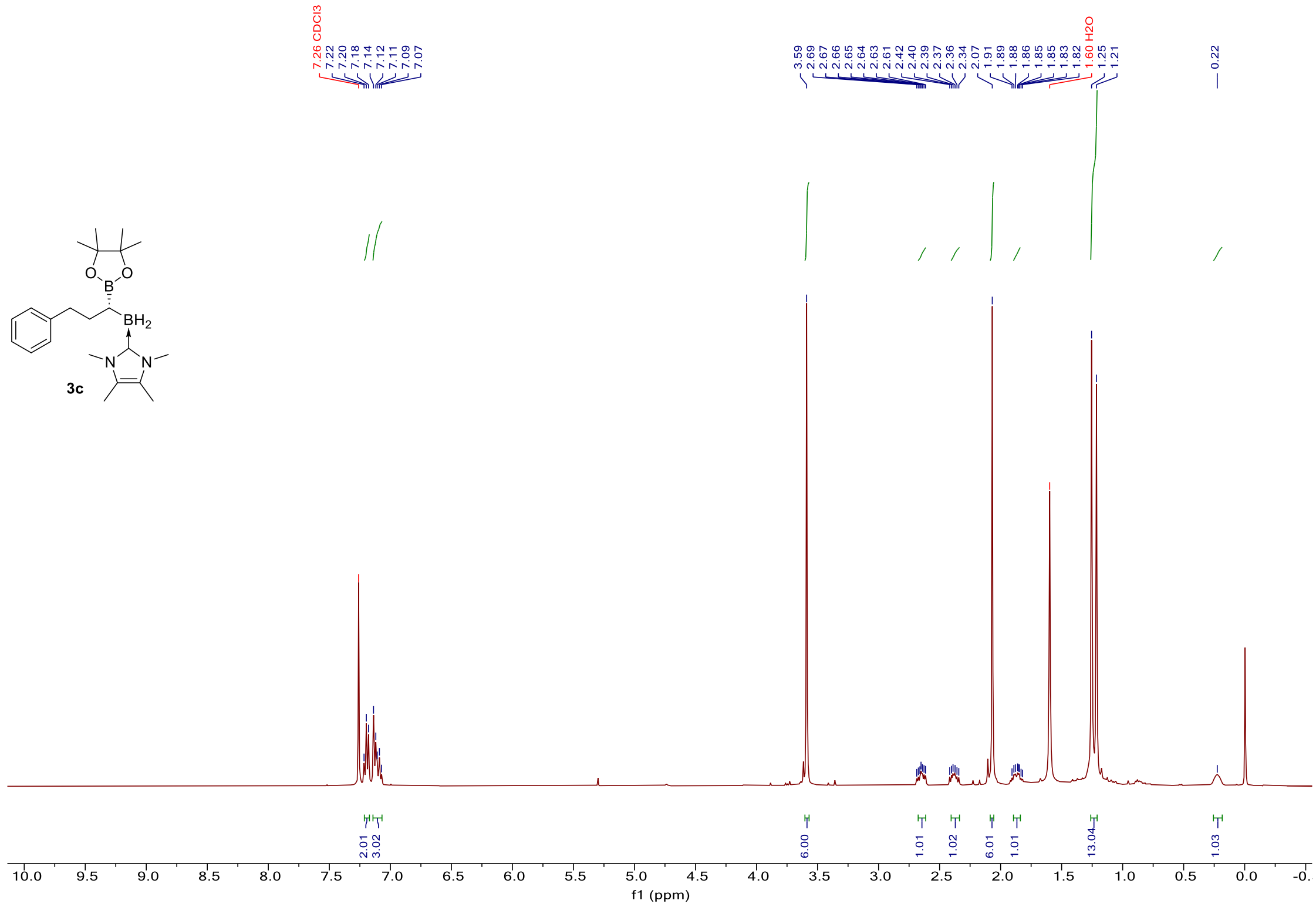

^{1}H NMR (400 MHz, CDCl$_3$)

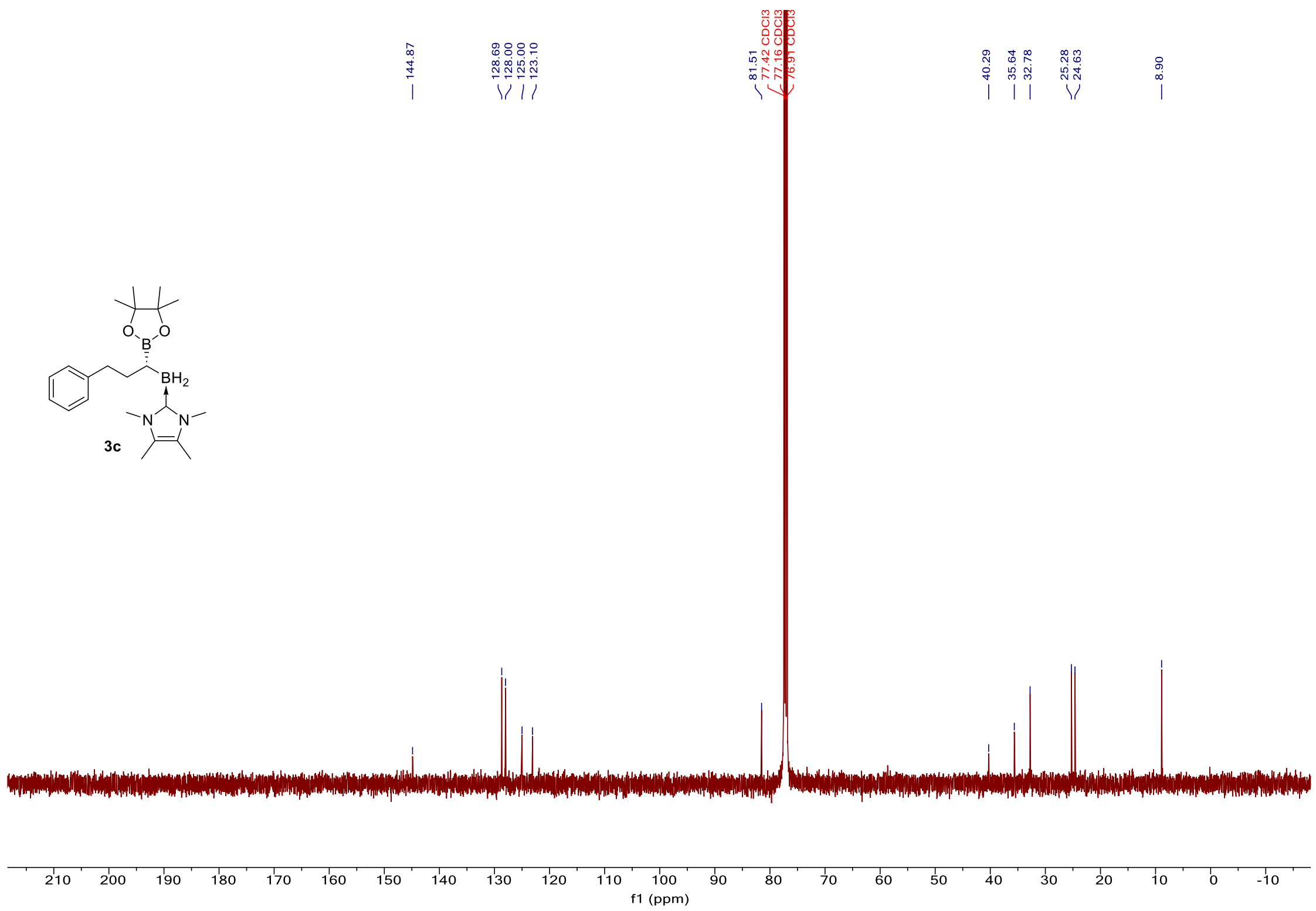

^{13}C NMR (126 MHz, $CDCl_3$)

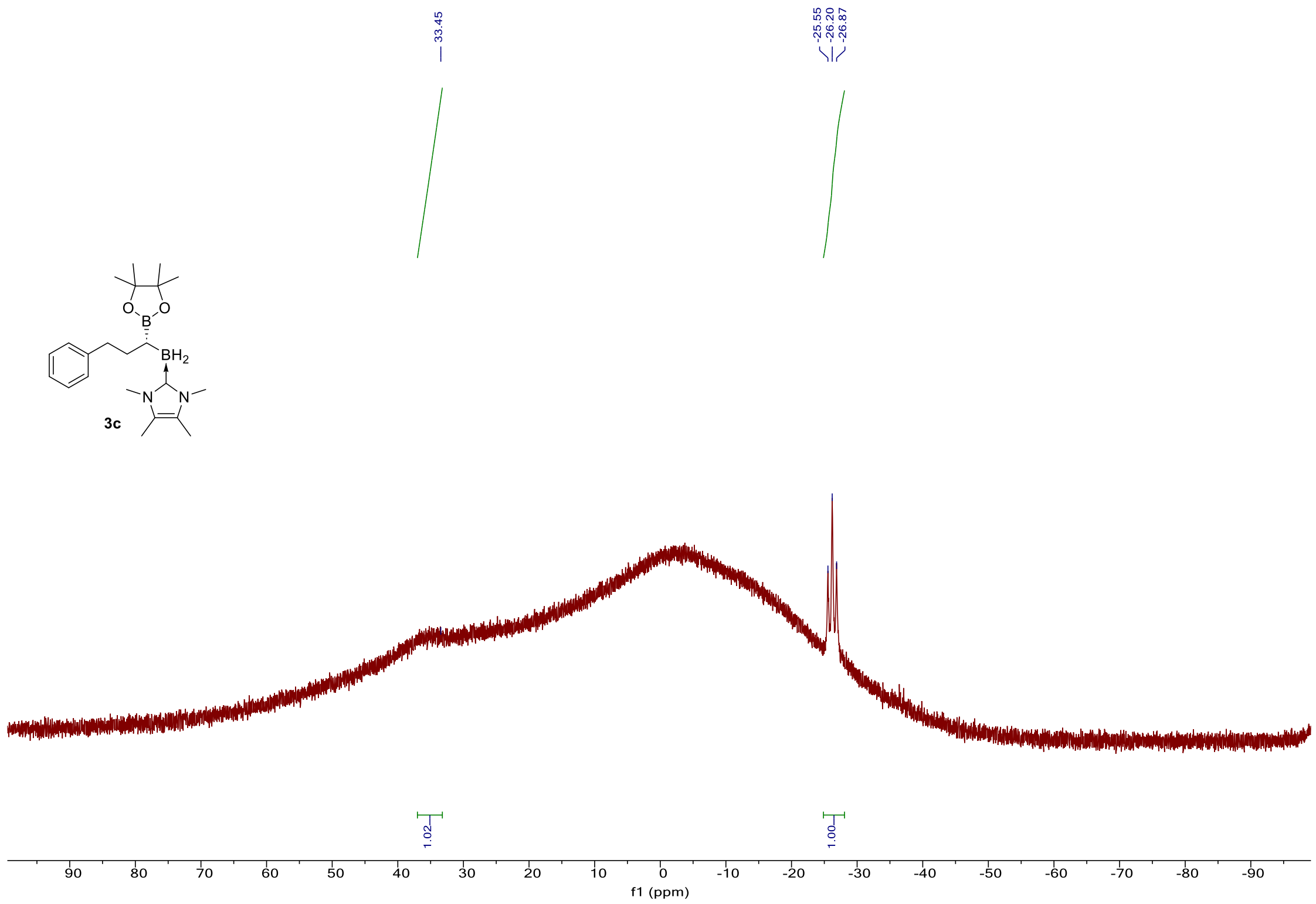

^{11}B NMR (128 MHz, $CDCl_3$)

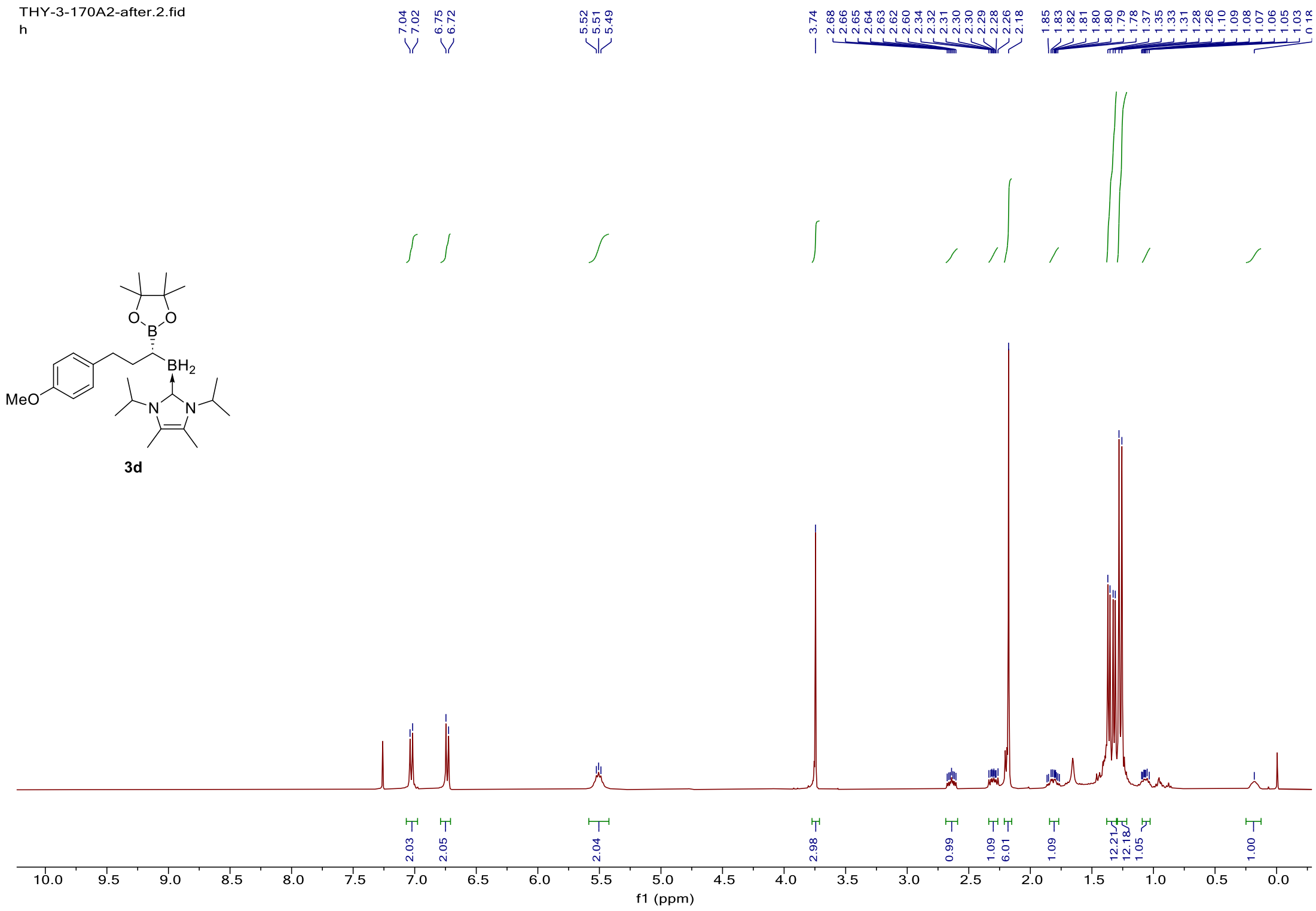

^{1}H NMR (400 MHz, $CDCl_3$)

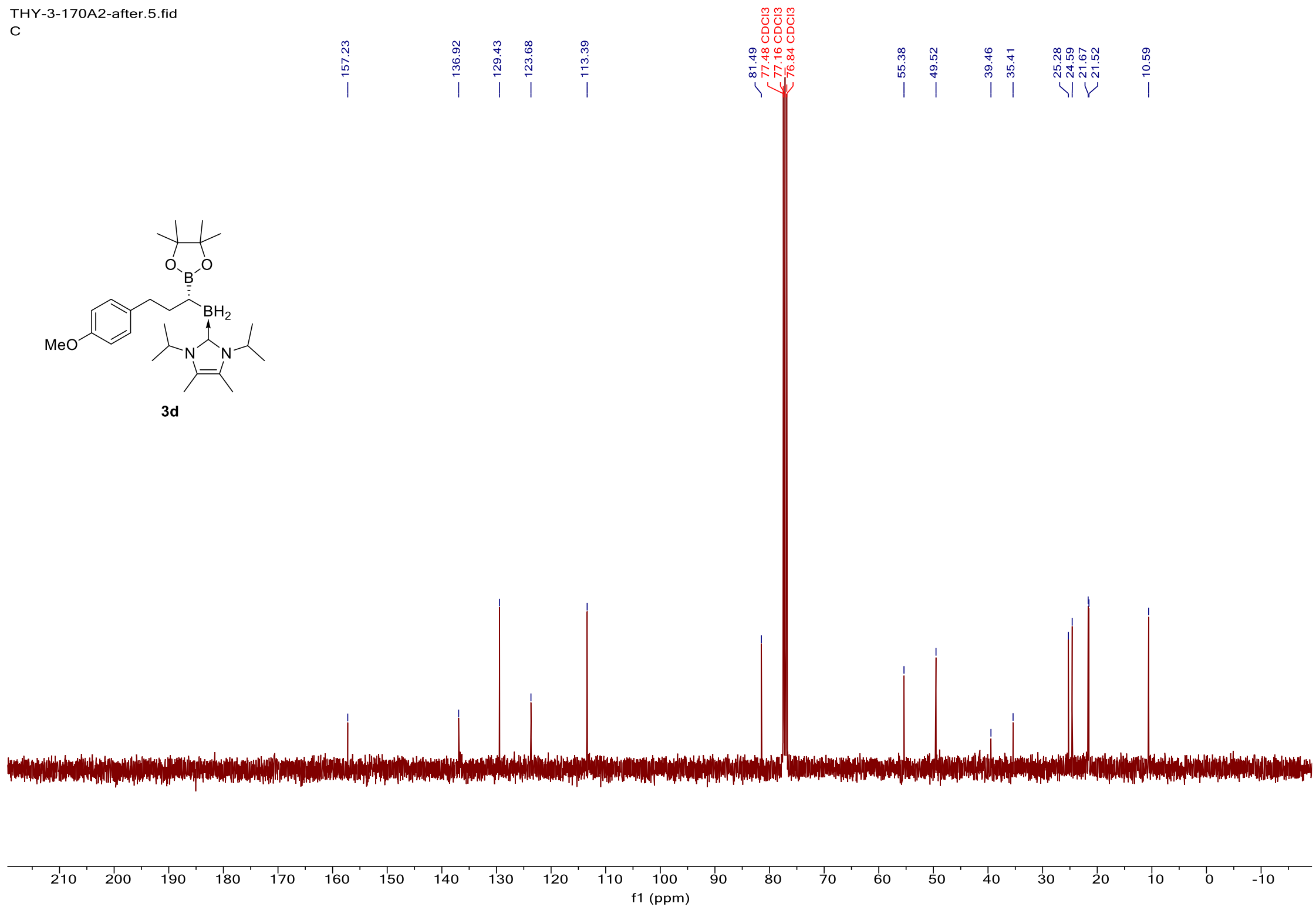

^{13}C NMR (101 MHz, $CDCl_3$)

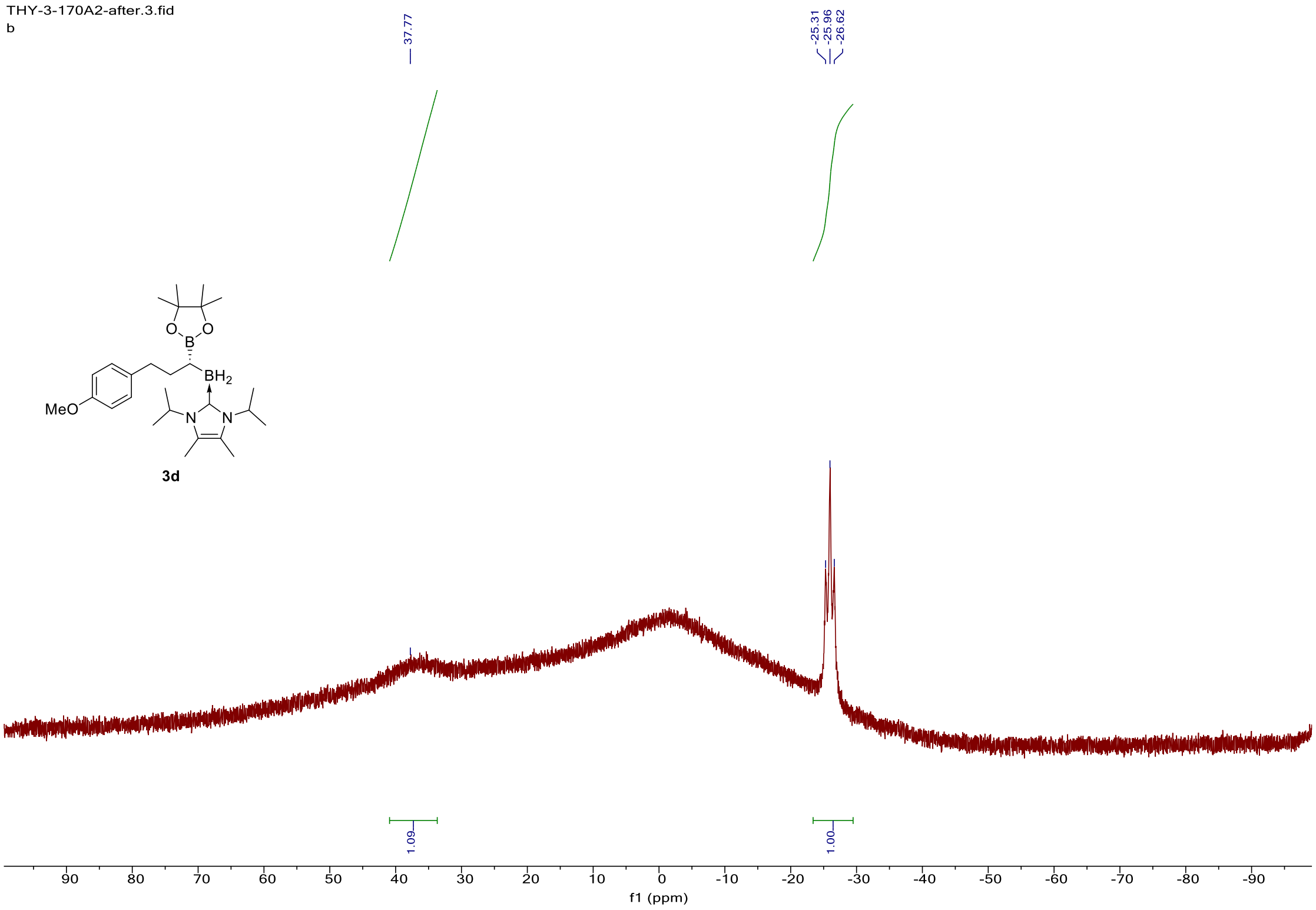

^{11}B NMR (128 MHz, $CDCl_3$)

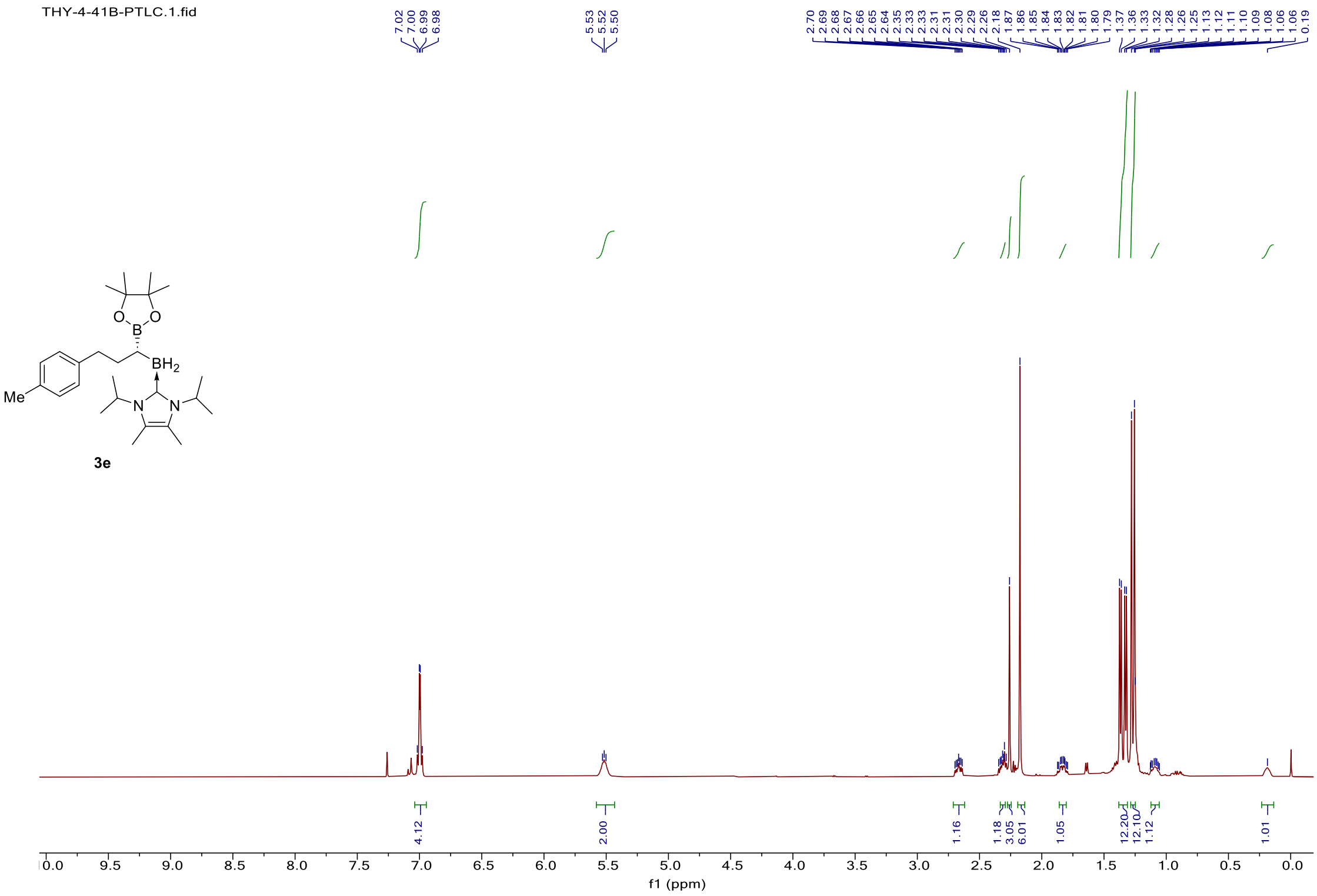

^{1}H NMR (500 MHz, $CDCl_3$)

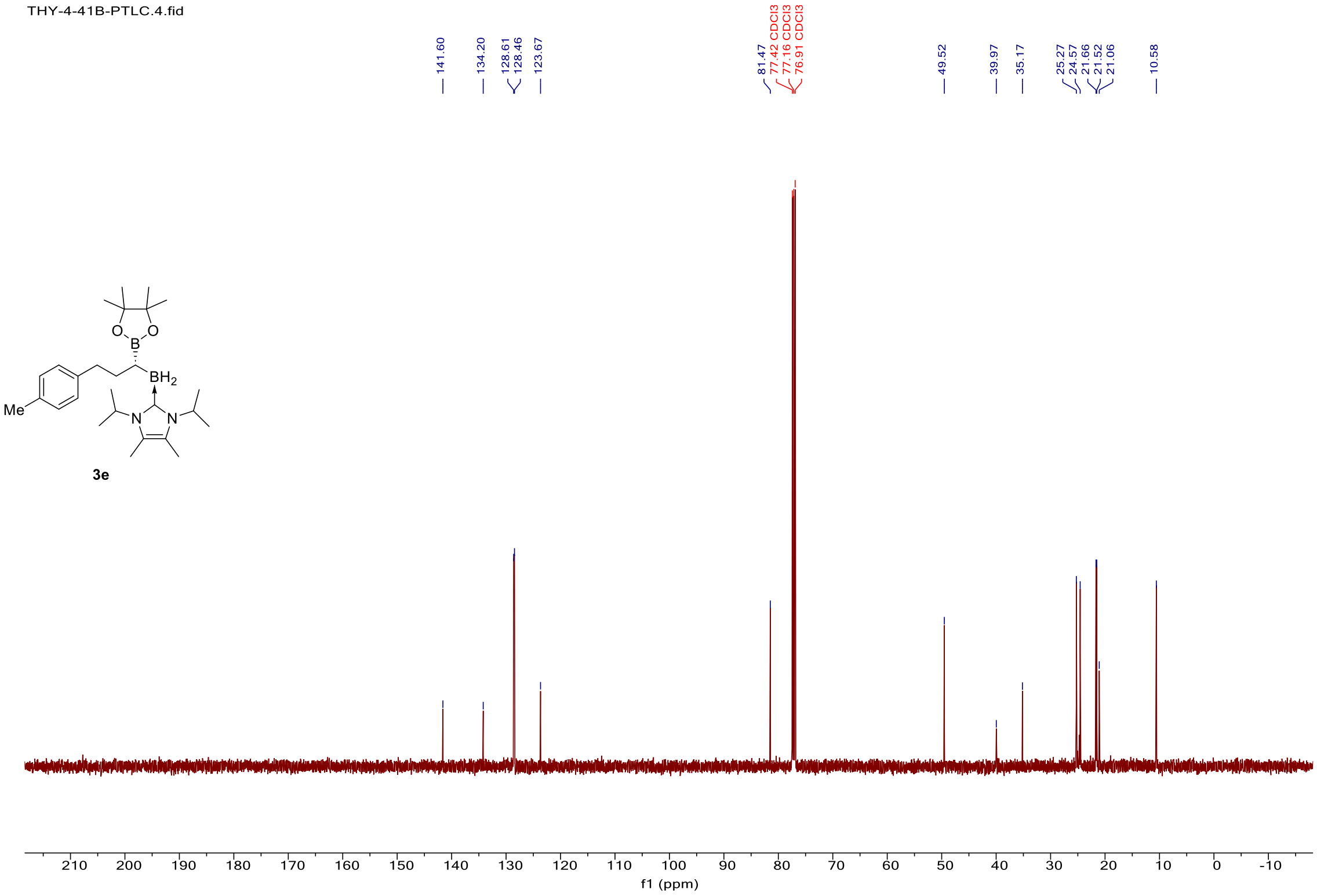

^{13}C NMR (126 MHz, CDCl$_3$)

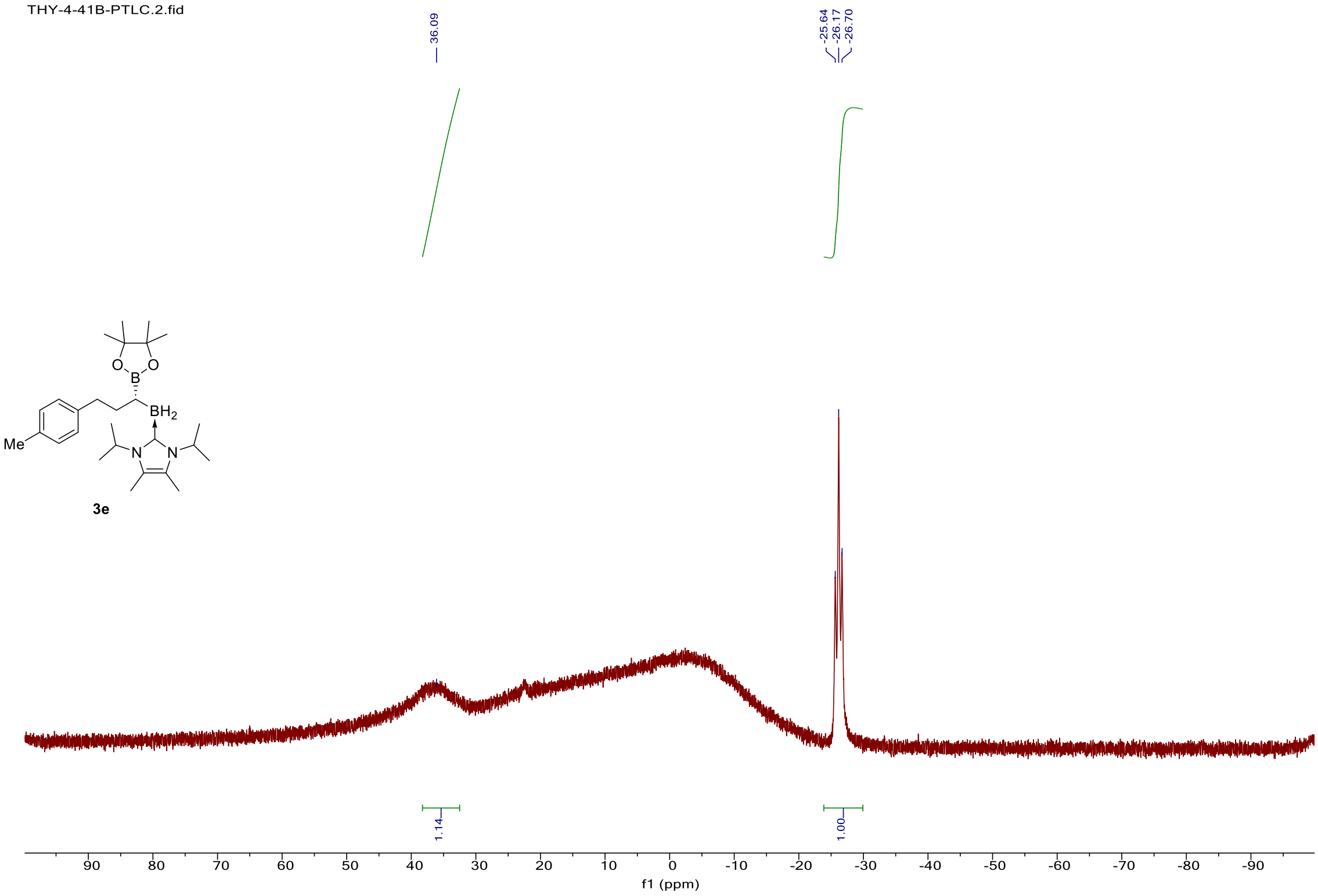

^{11}B NMR (160 MHz, CDCl$_3$)

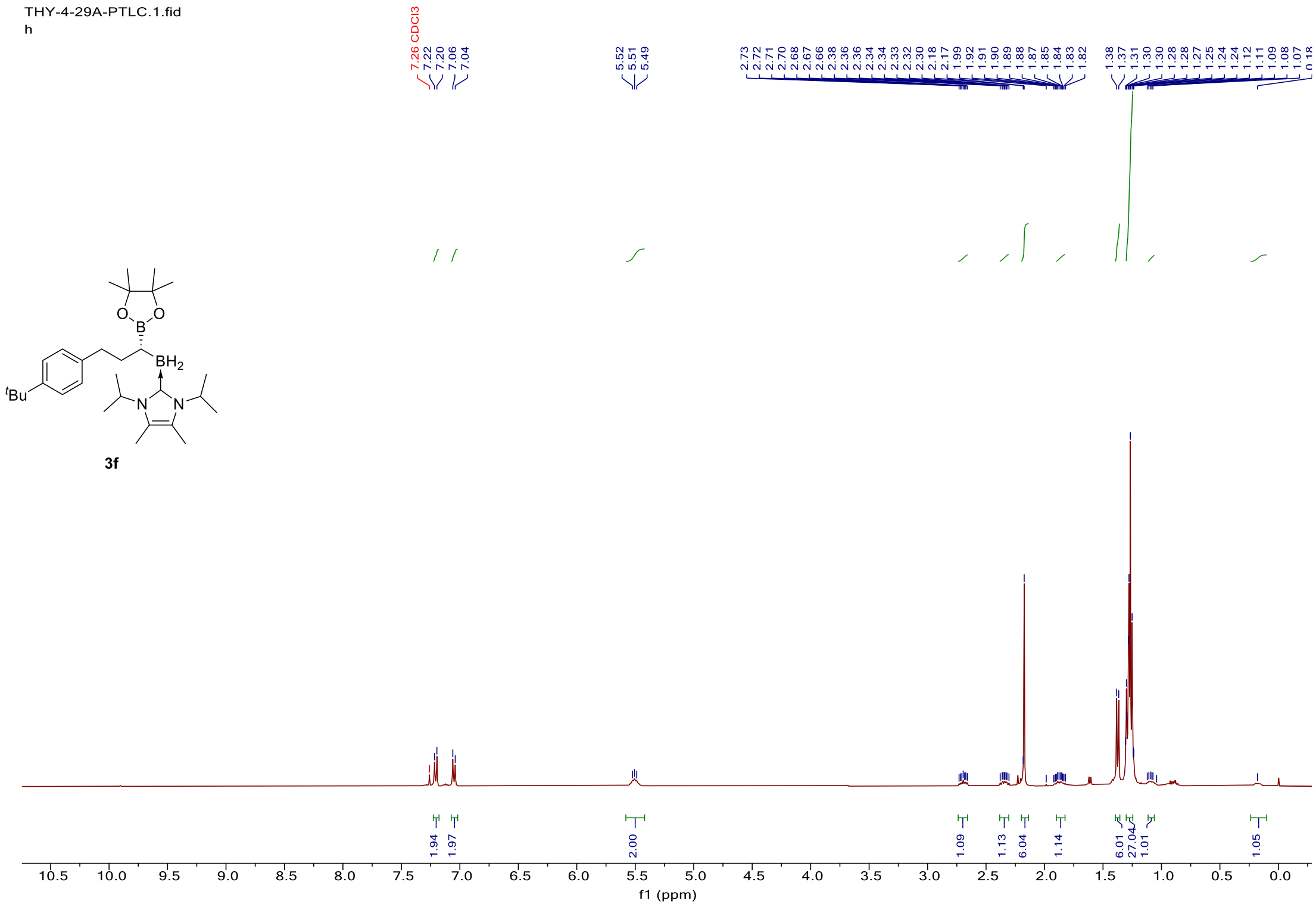

^{1}H NMR (400 MHz, CDCl$_3$)

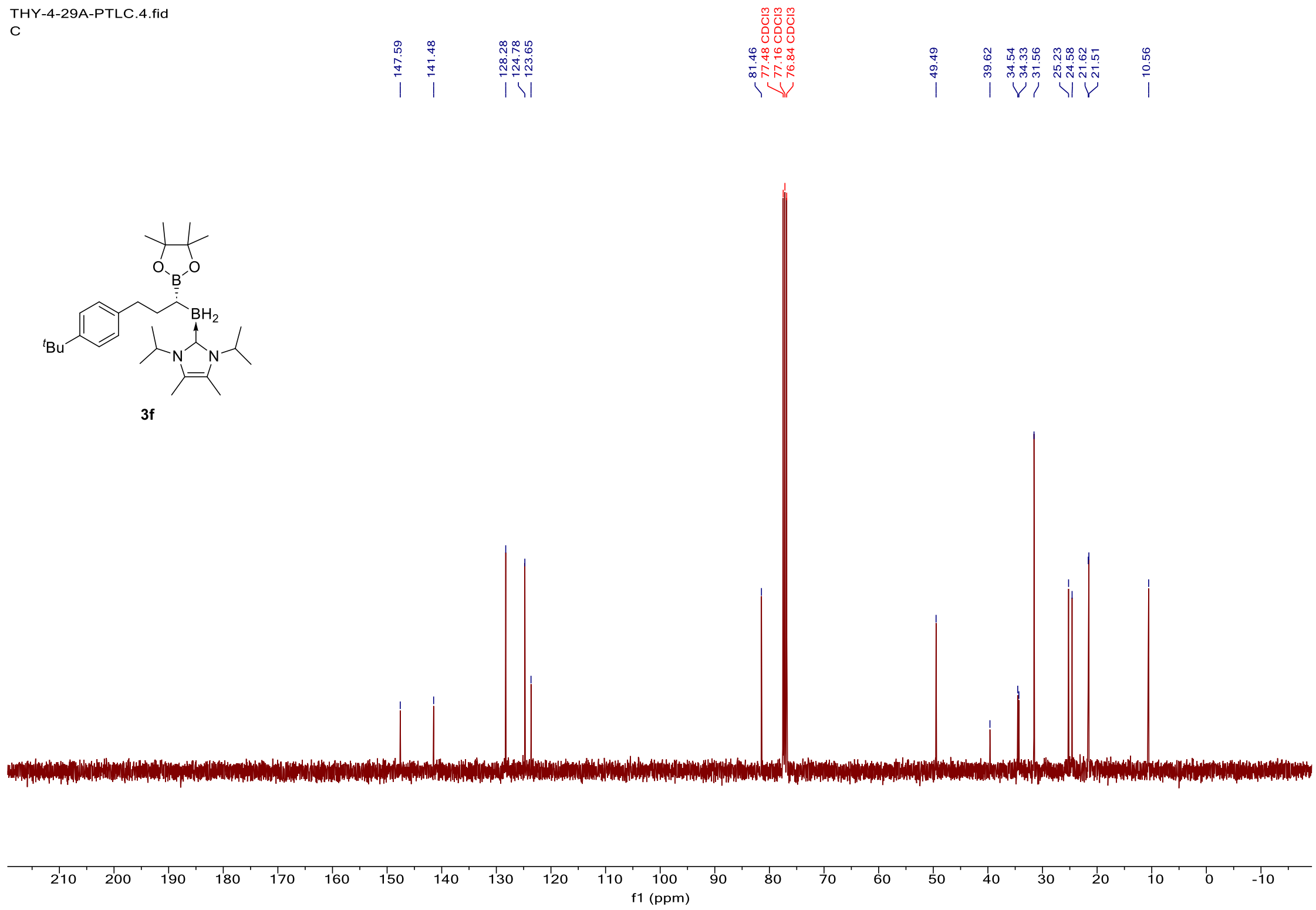

^{13}C NMR (101 MHz, CDCl$_3$)

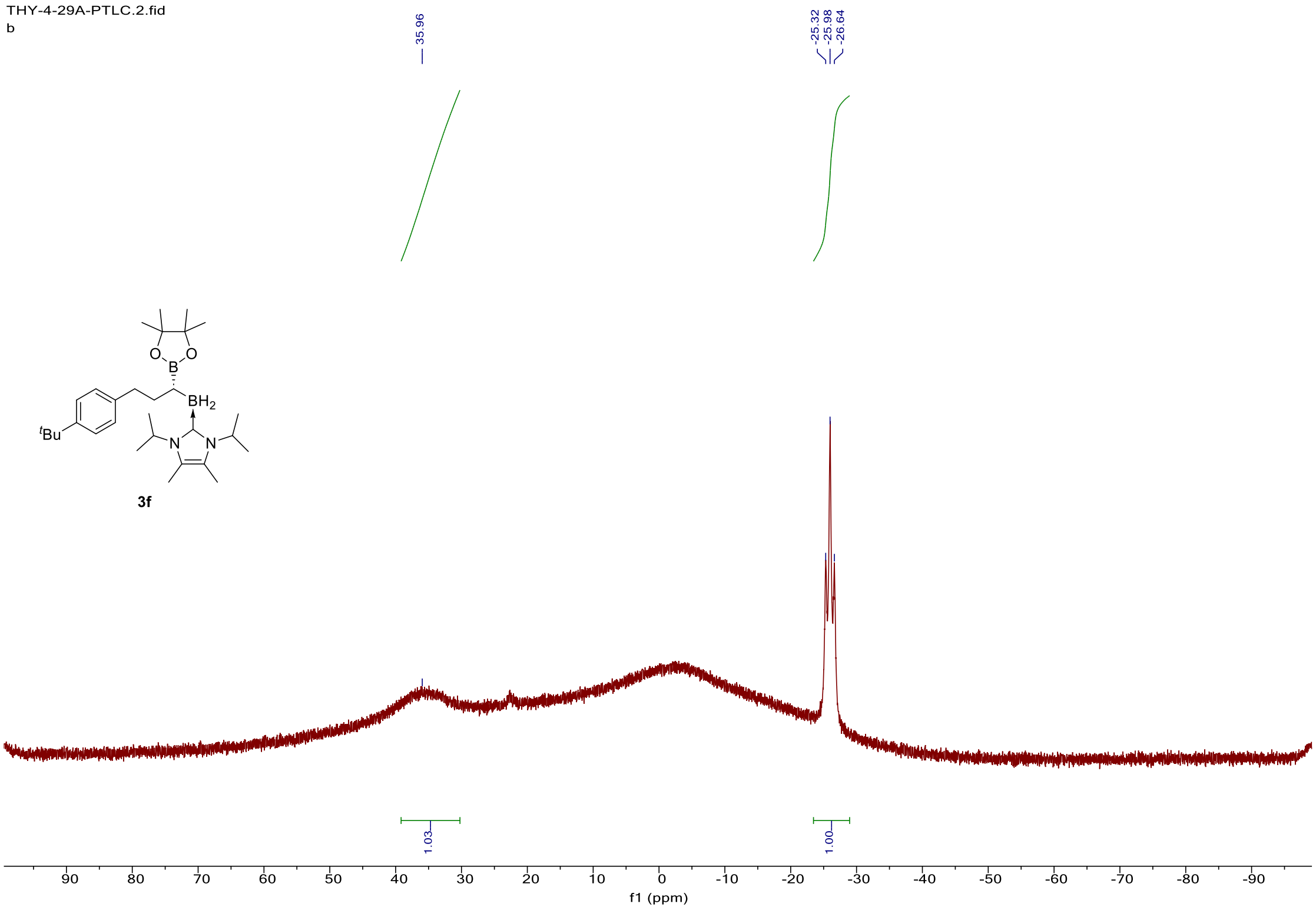

^{11}B NMR (128 MHz, $CDCl_3$)

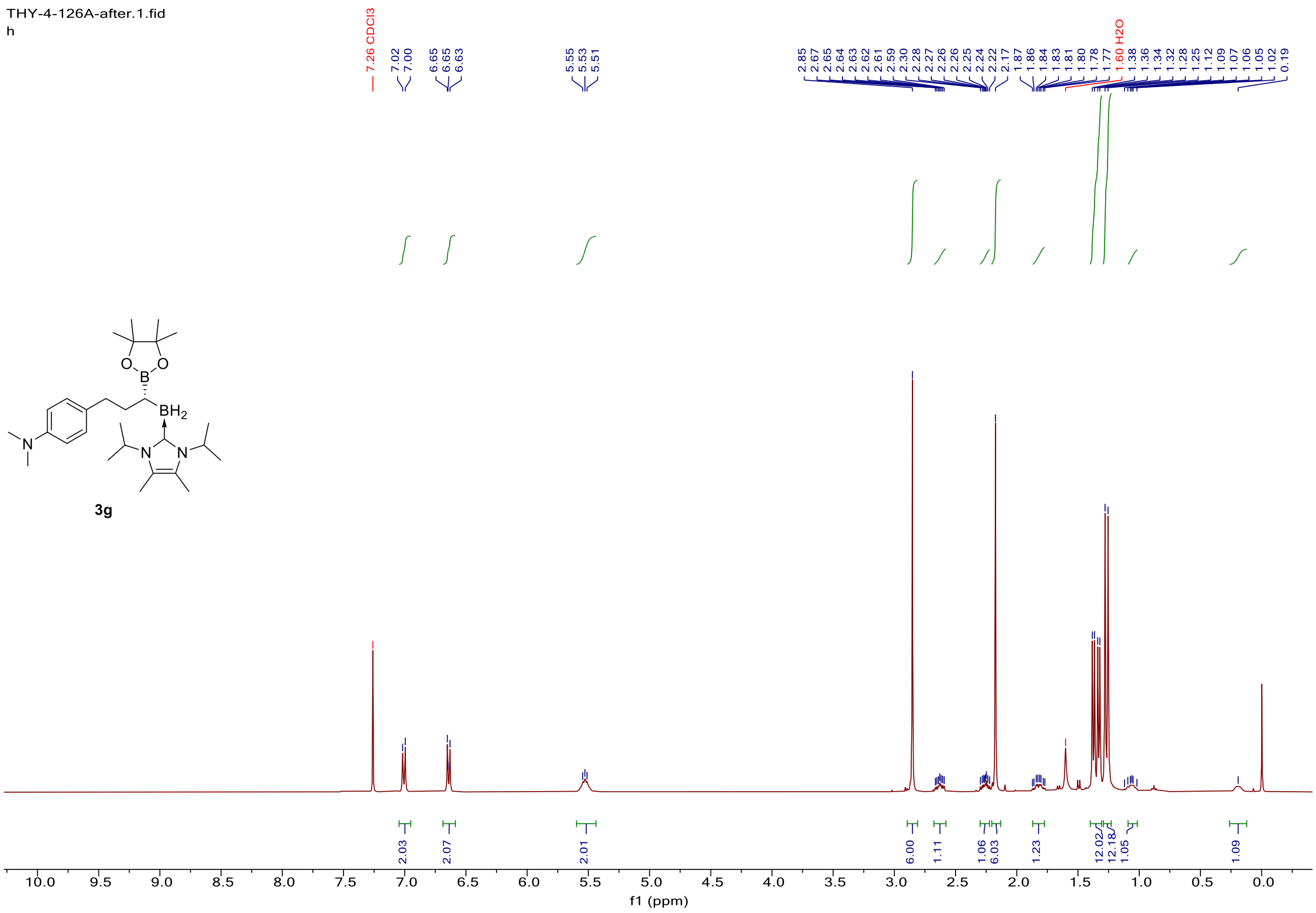

^{1}H NMR (400 MHz, $CDCl_3$)

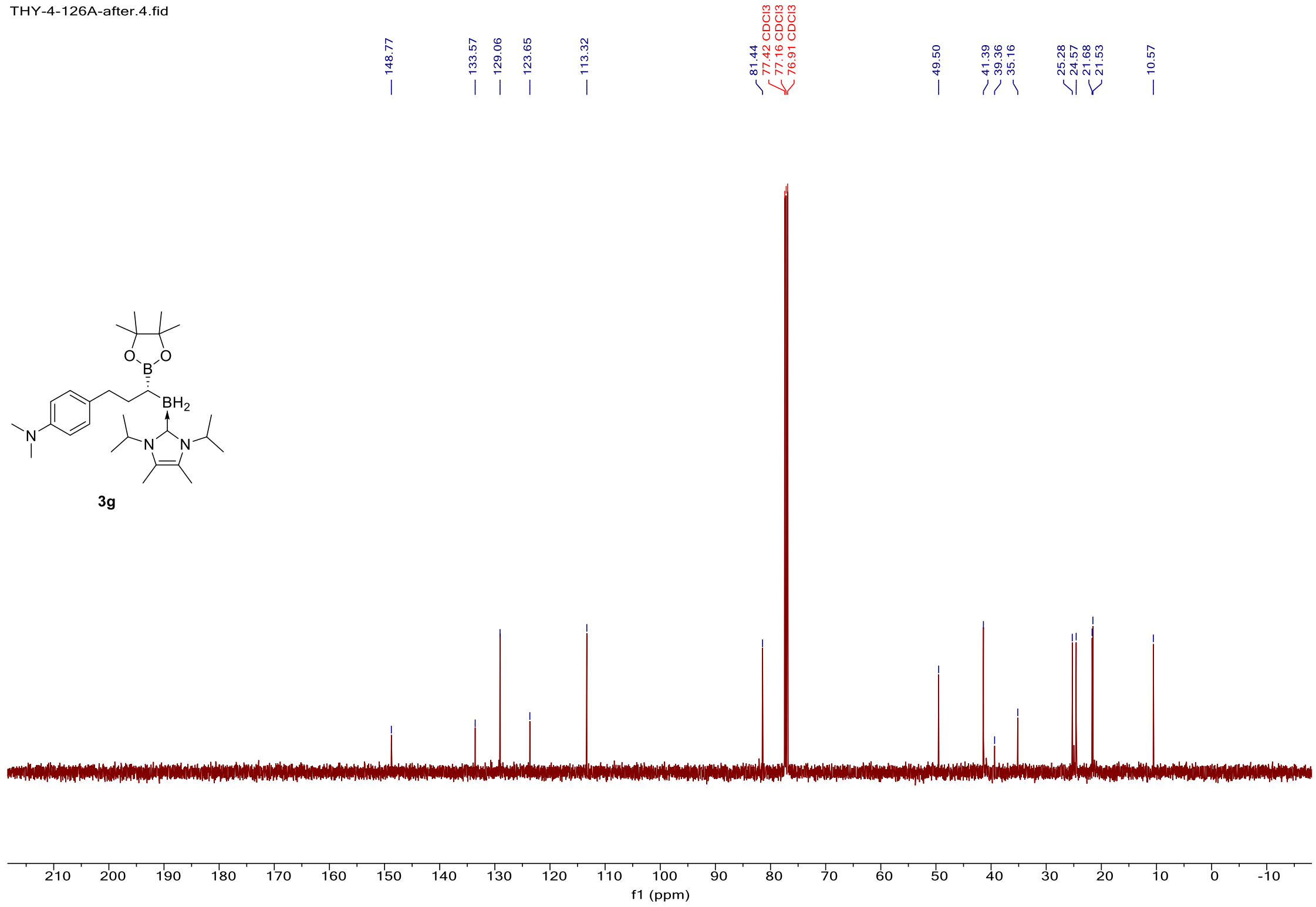

^{13}C NMR (101 MHz, $CDCl_3$)

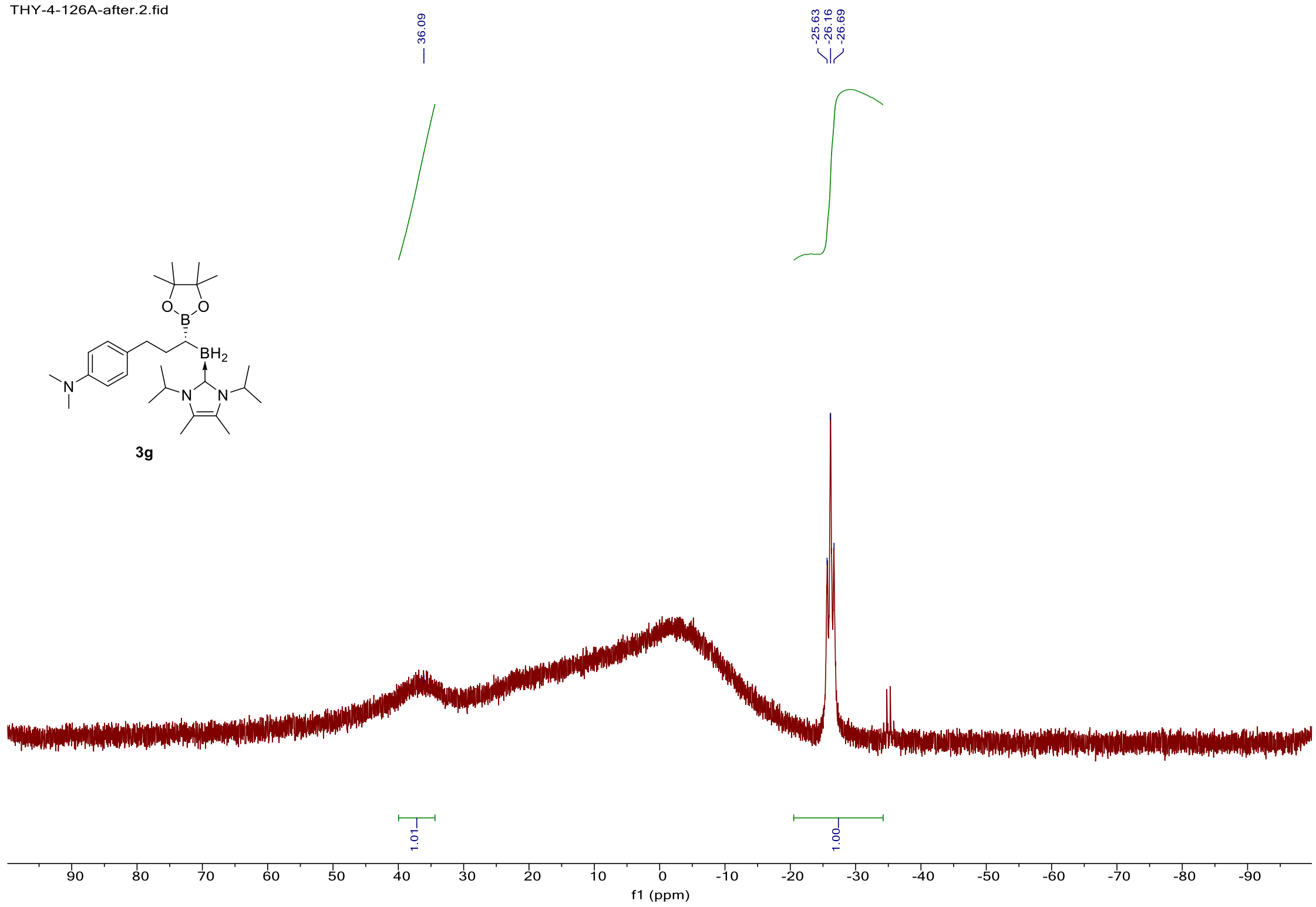

^{11}B NMR (128 MHz, $CDCl_3$)

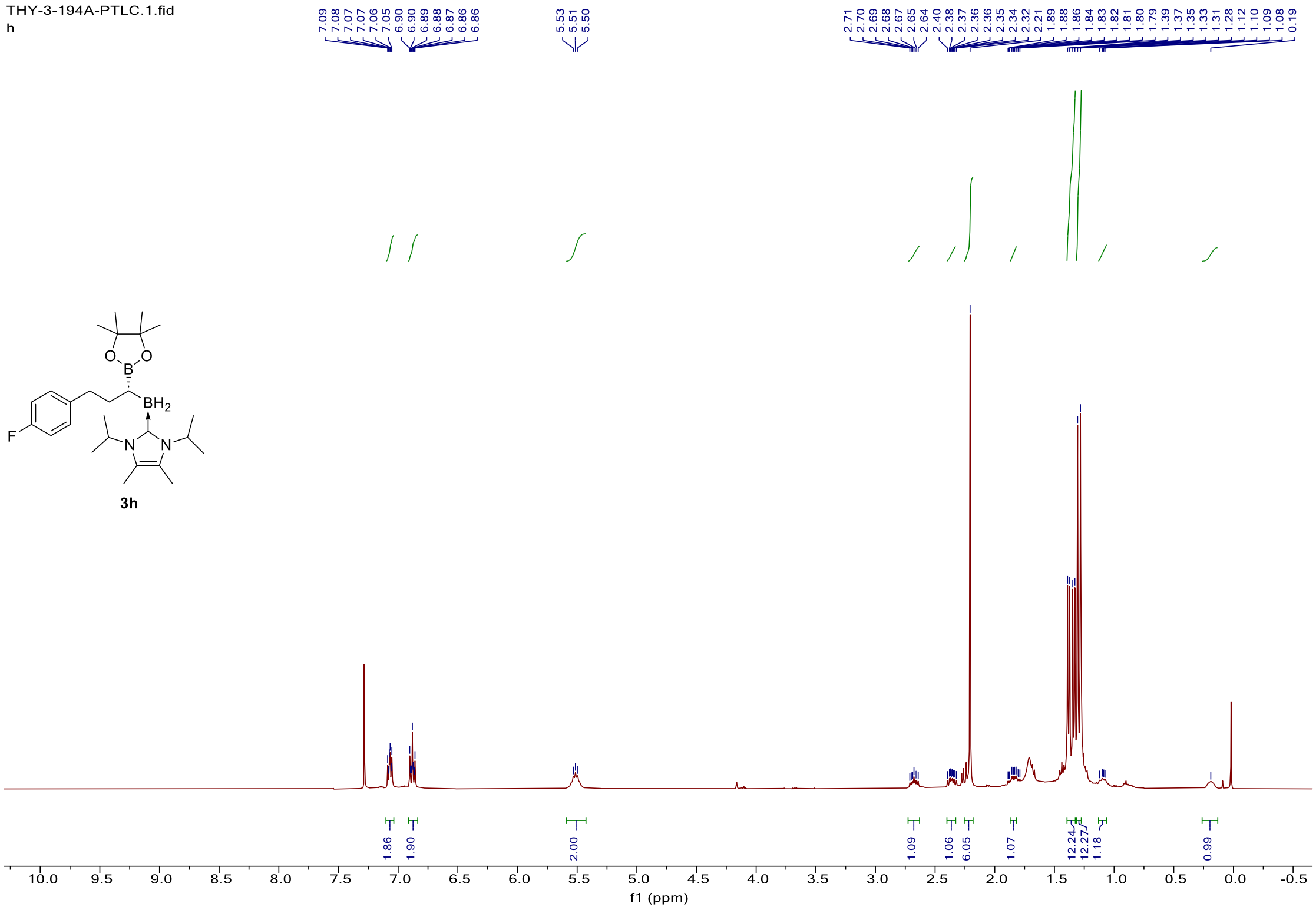

^{1}H NMR (400 MHz, $CDCl_3$)

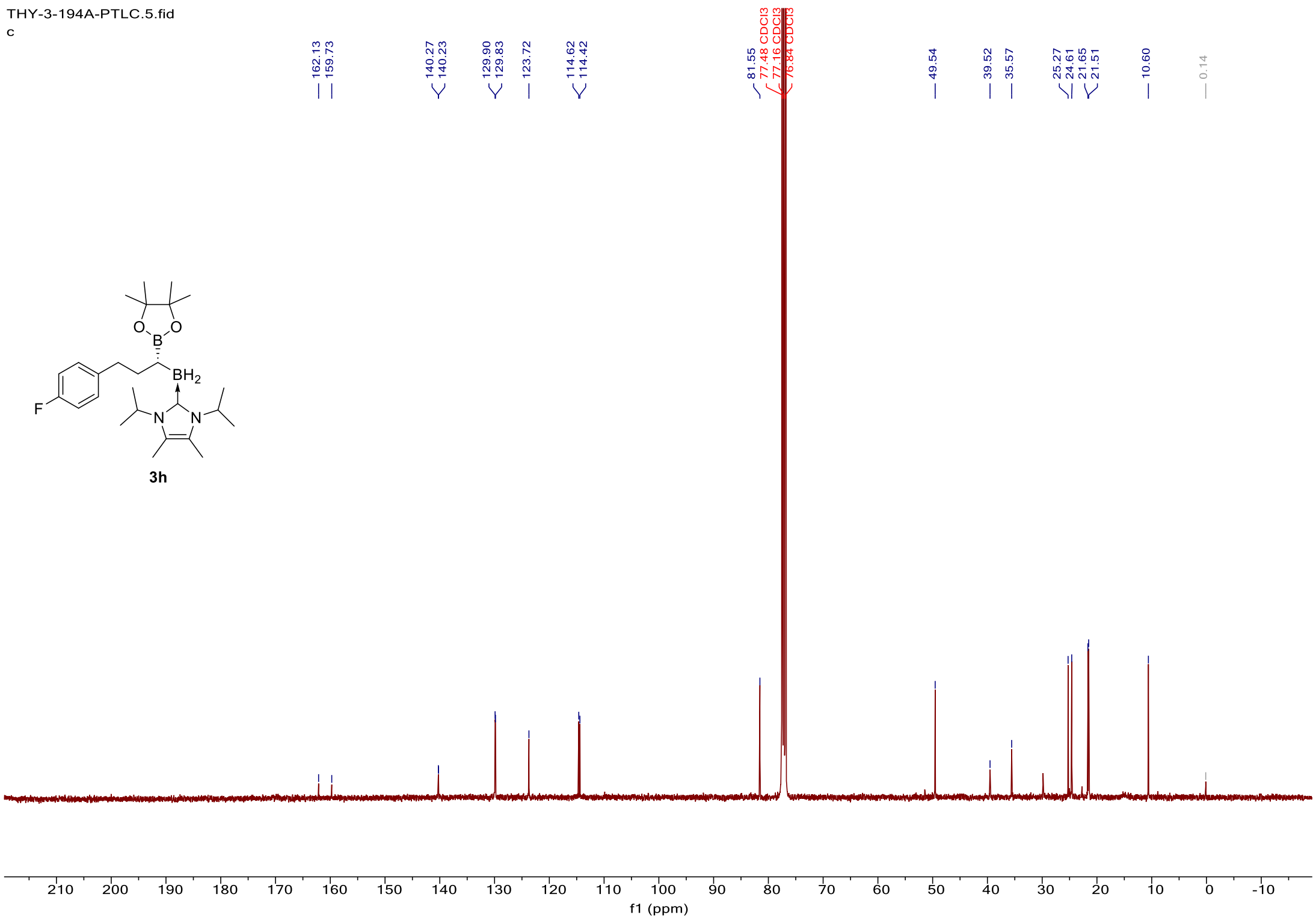

^{13}C NMR (101 MHz, $CDCl_3$)

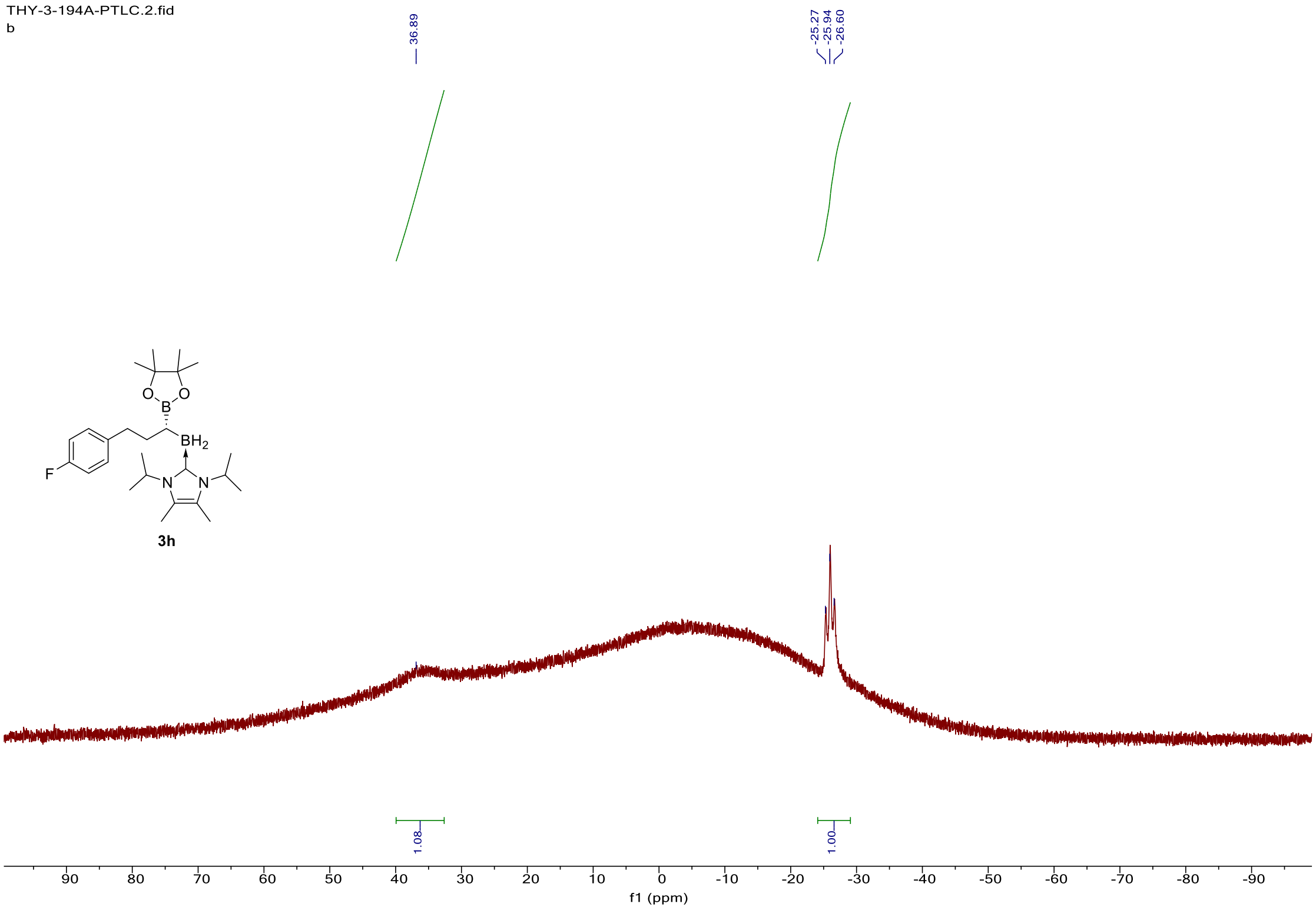

^{11}B NMR (128 MHz, $CDCl_3$)

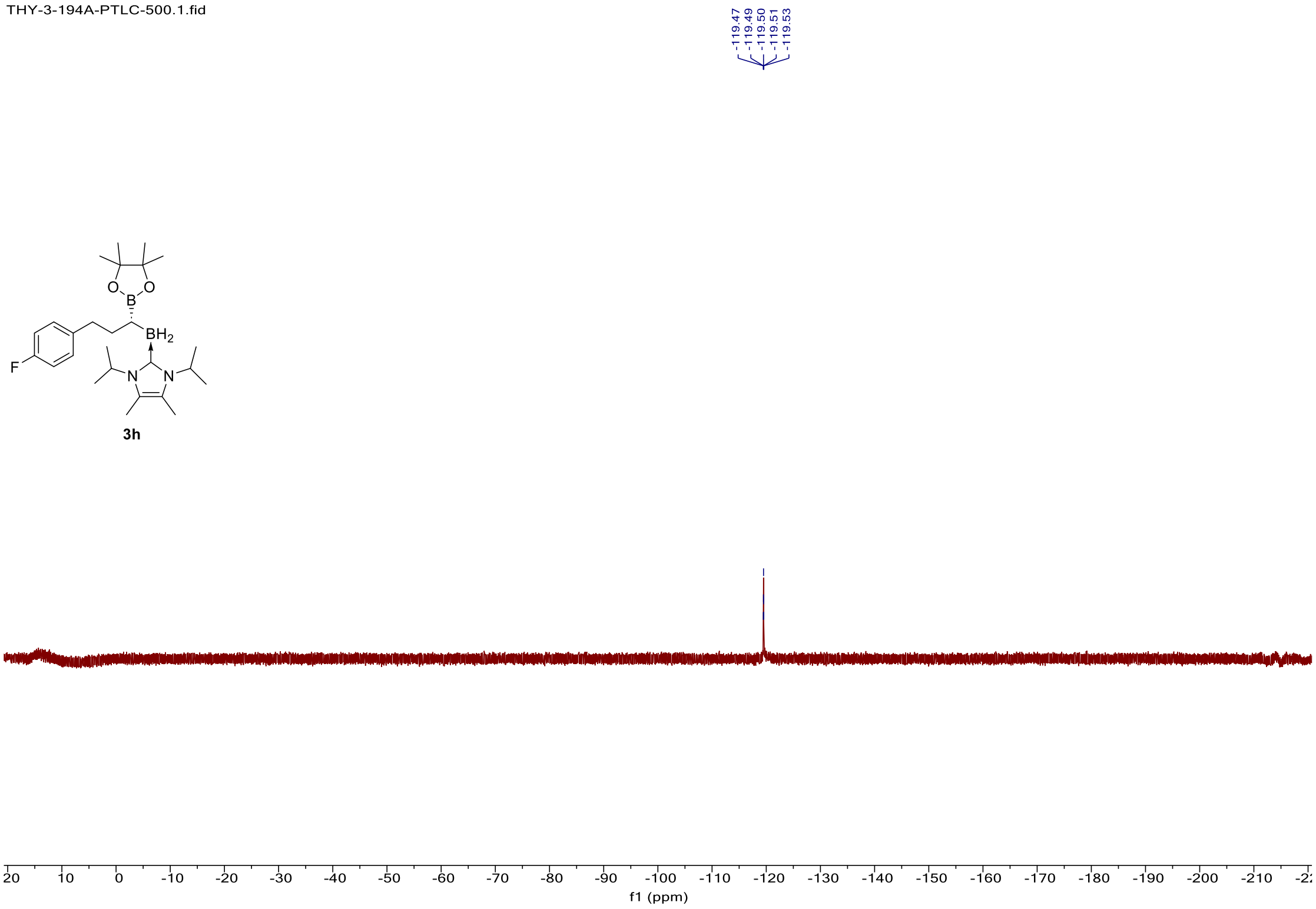

^{19}F NMR (471 MHz, $CDCl_3$)

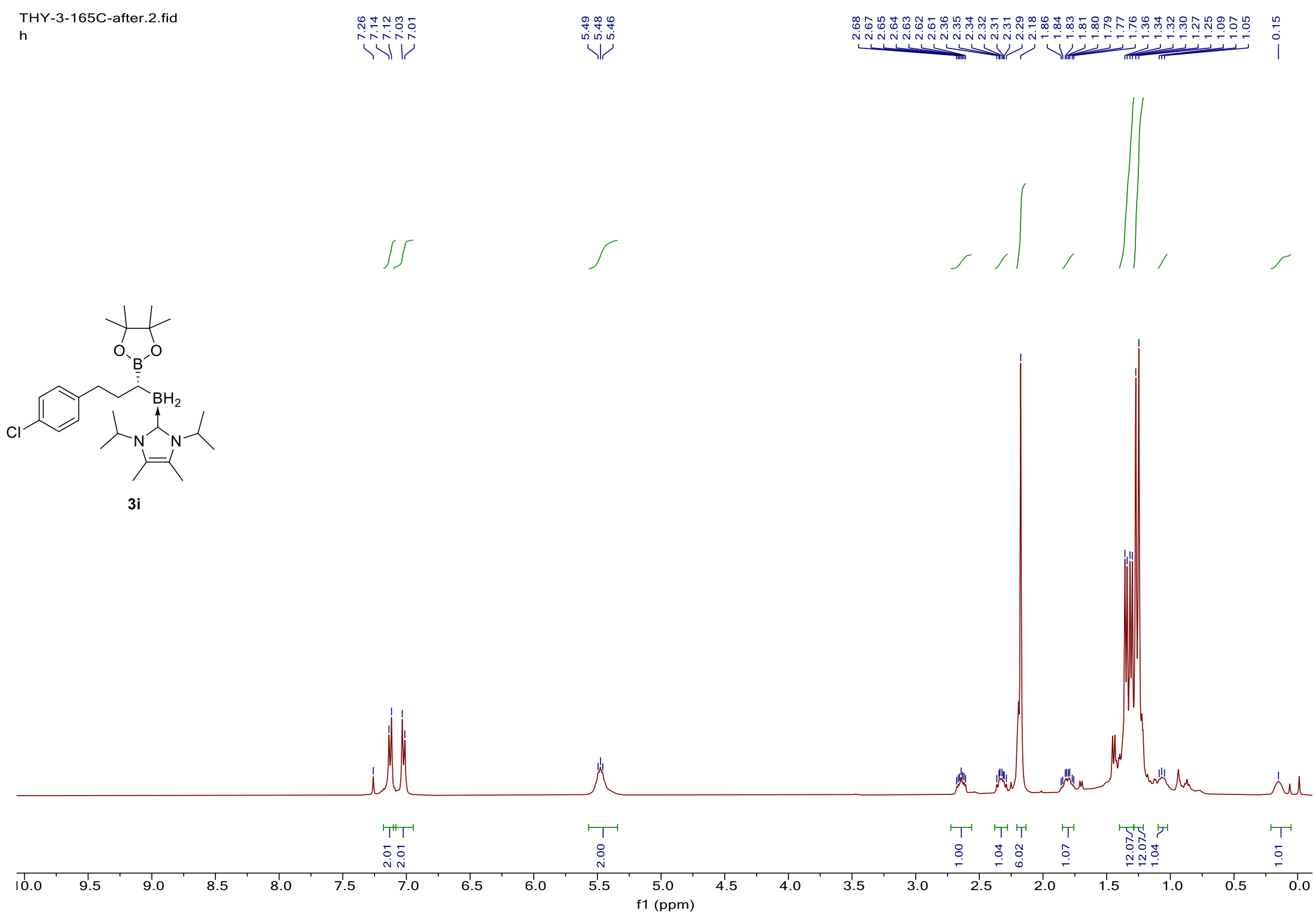

^{1}H NMR (400 MHz, $CDCl_3$)

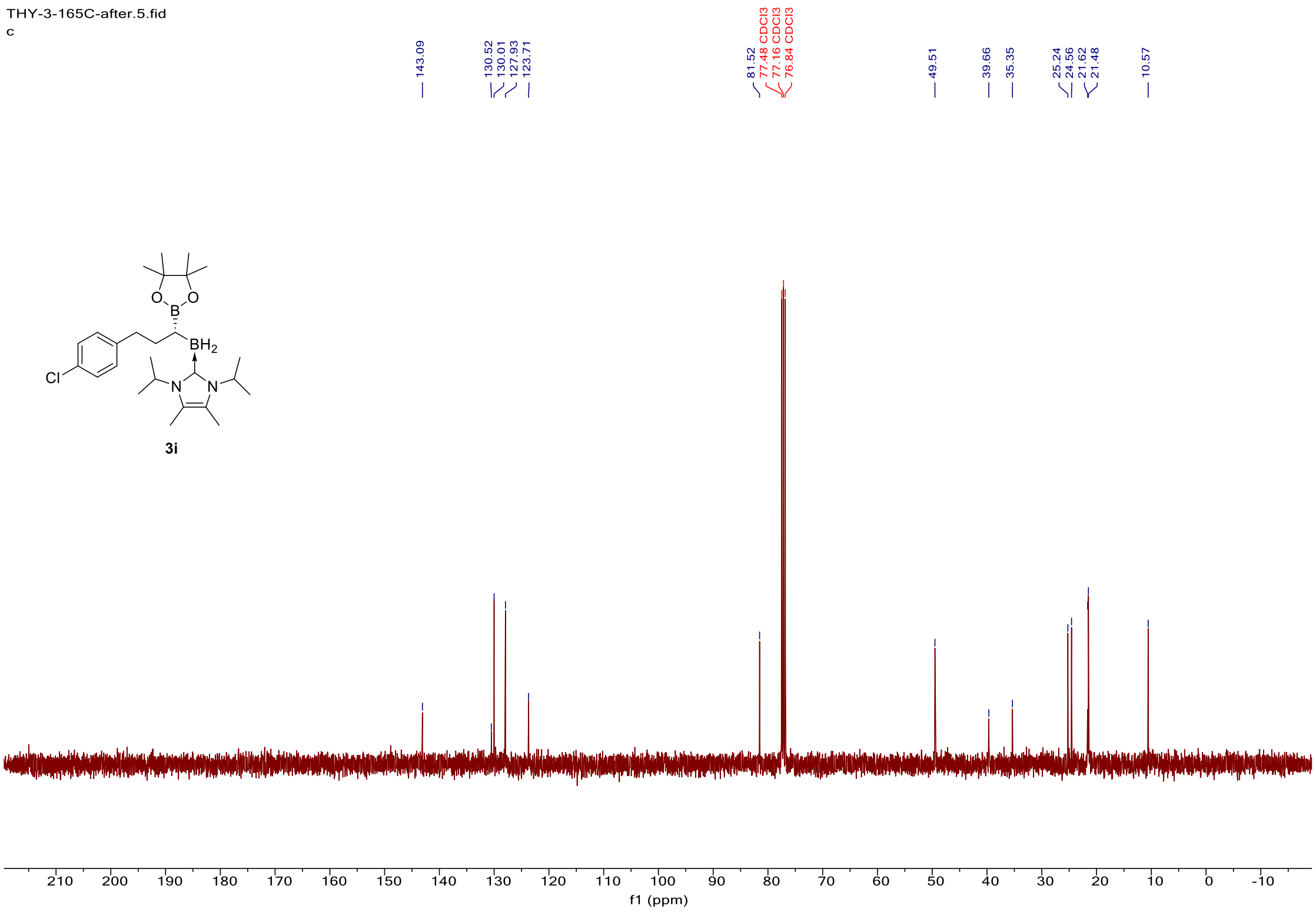

^{13}C NMR (101 MHz, $CDCl_3$)

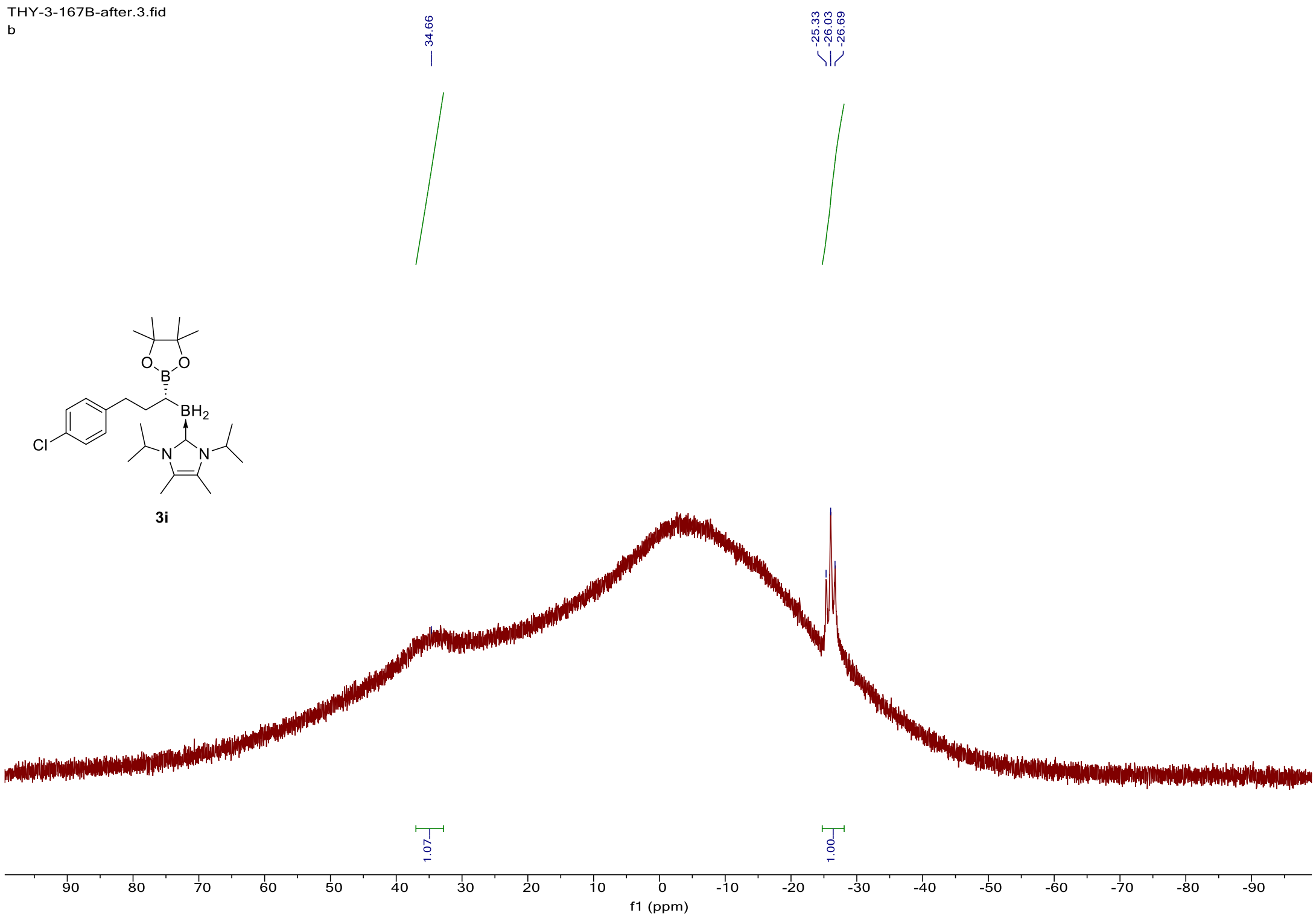

^{11}B NMR (128 MHz, $CDCl_3$)

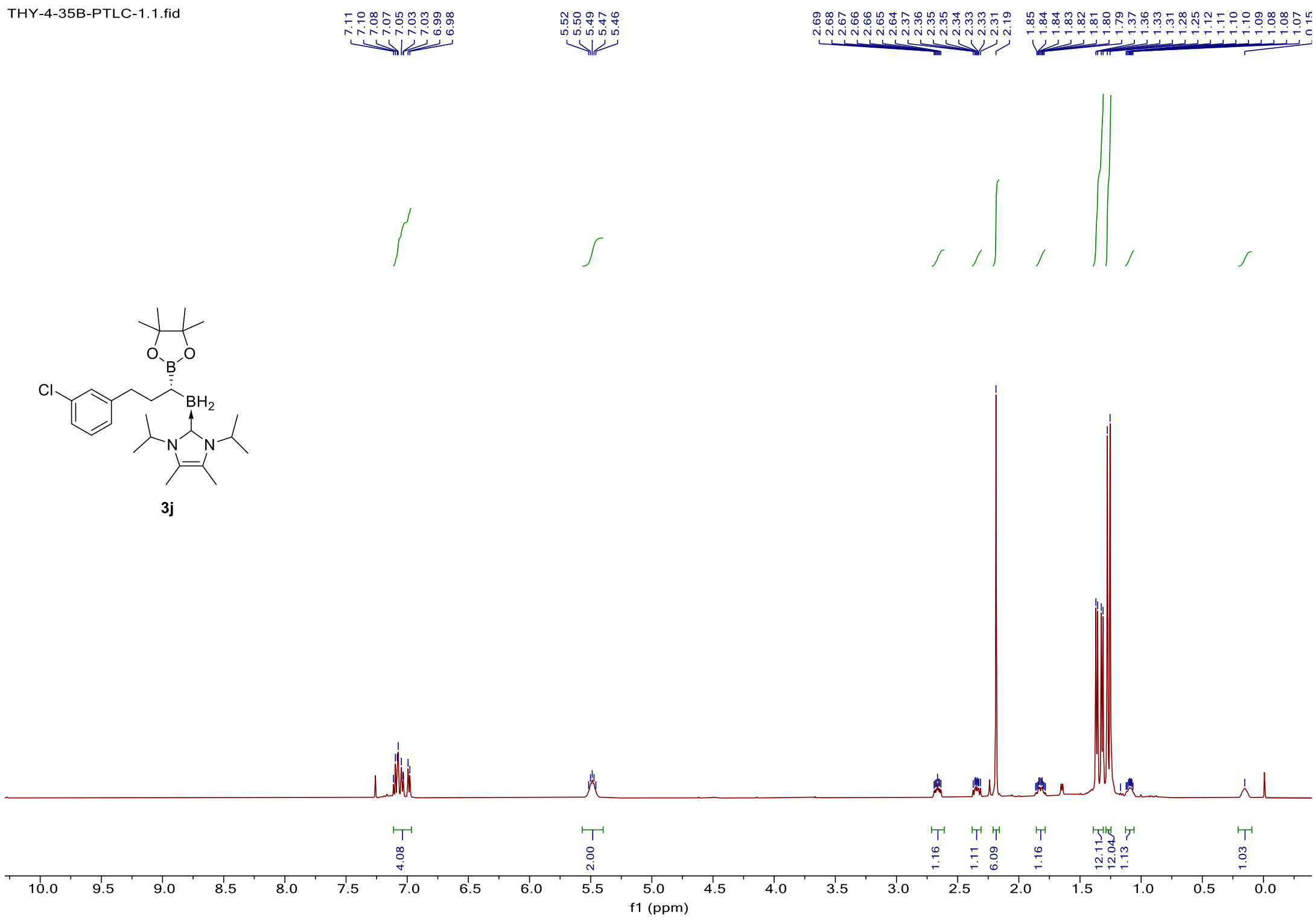

^{1}H NMR (500 MHz, $CDCl_3$)

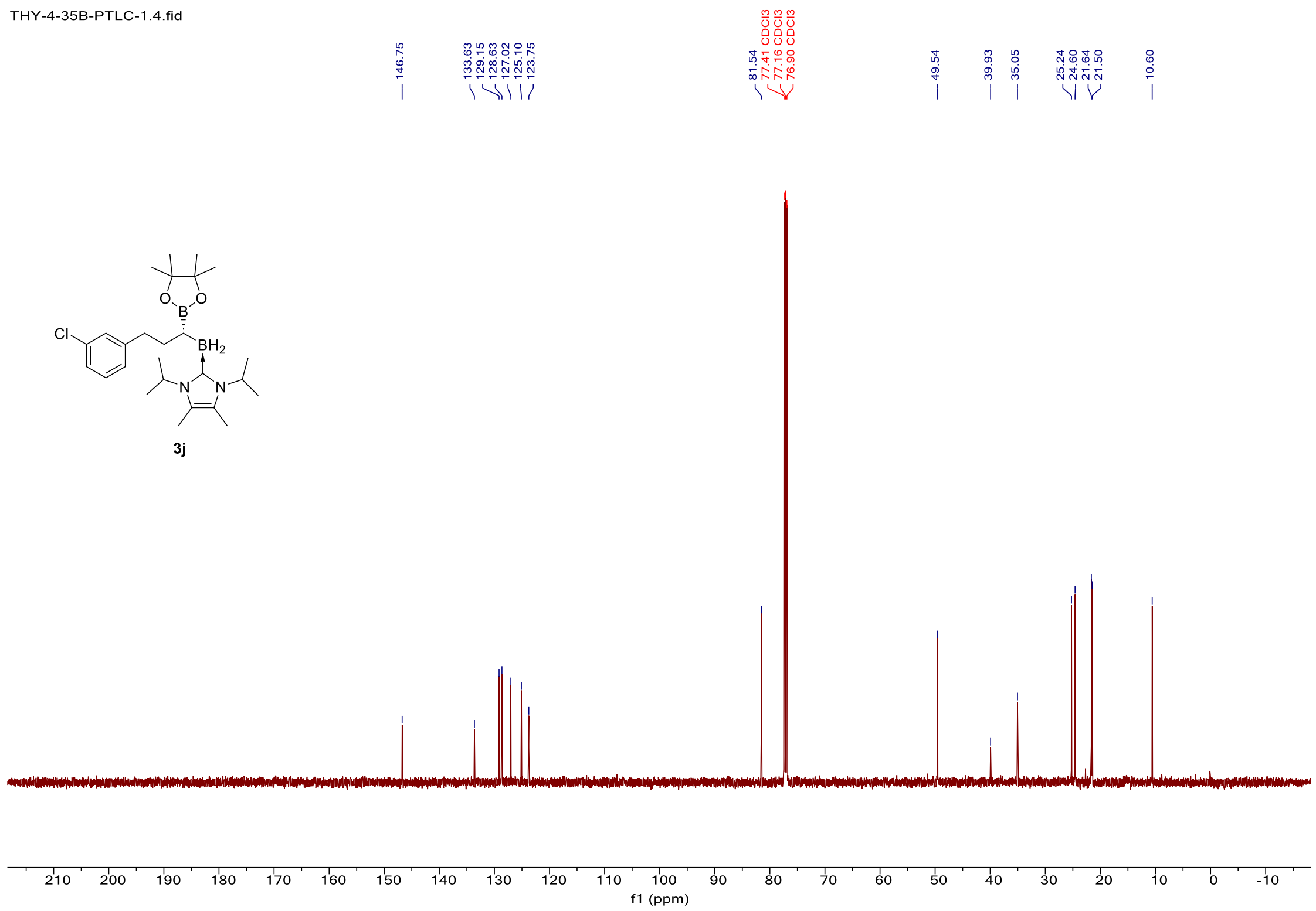

^{13}C NMR (126 MHz, $CDCl_3$)

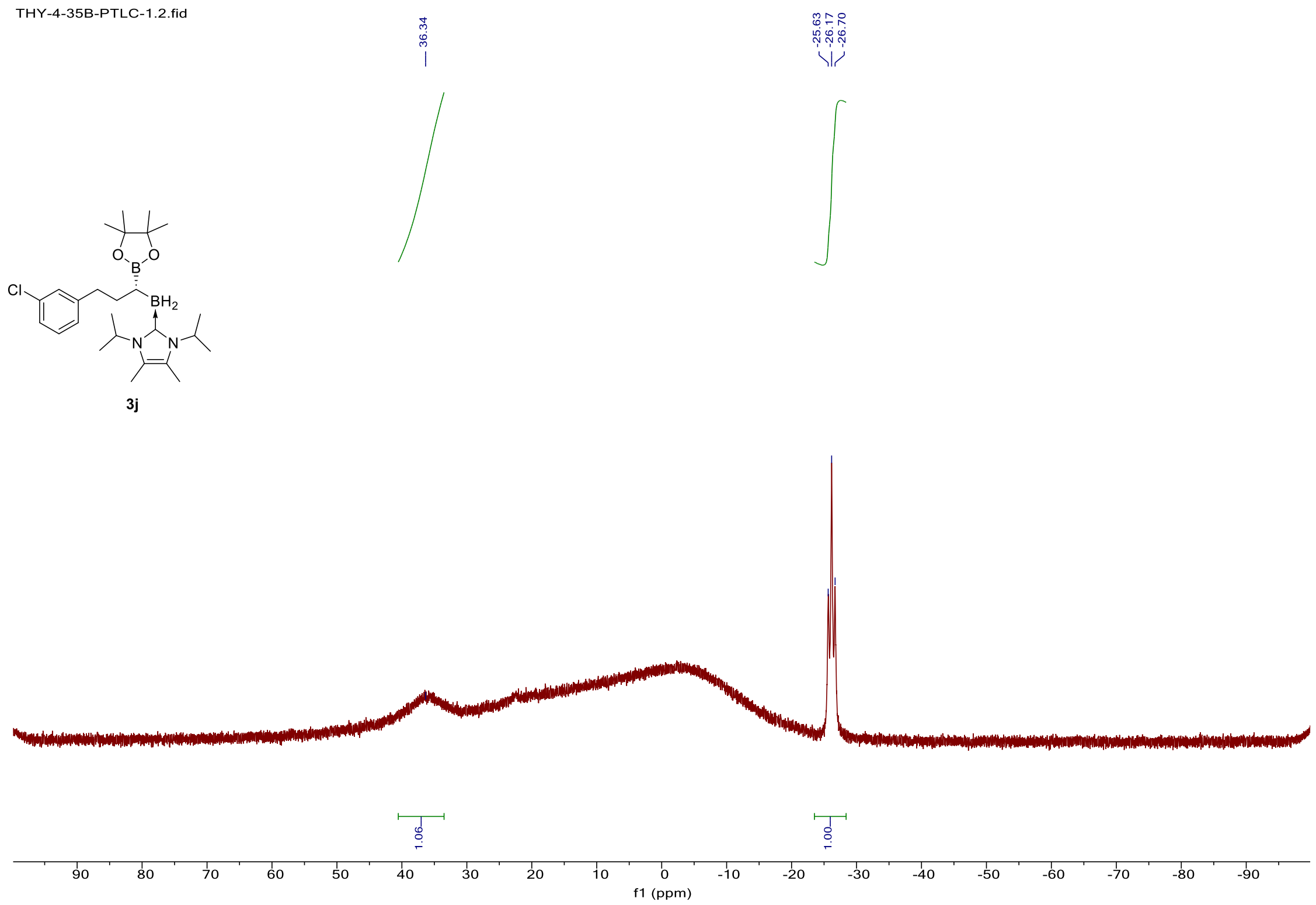

^{11}B NMR (160 MHz, $CDCl_3$)

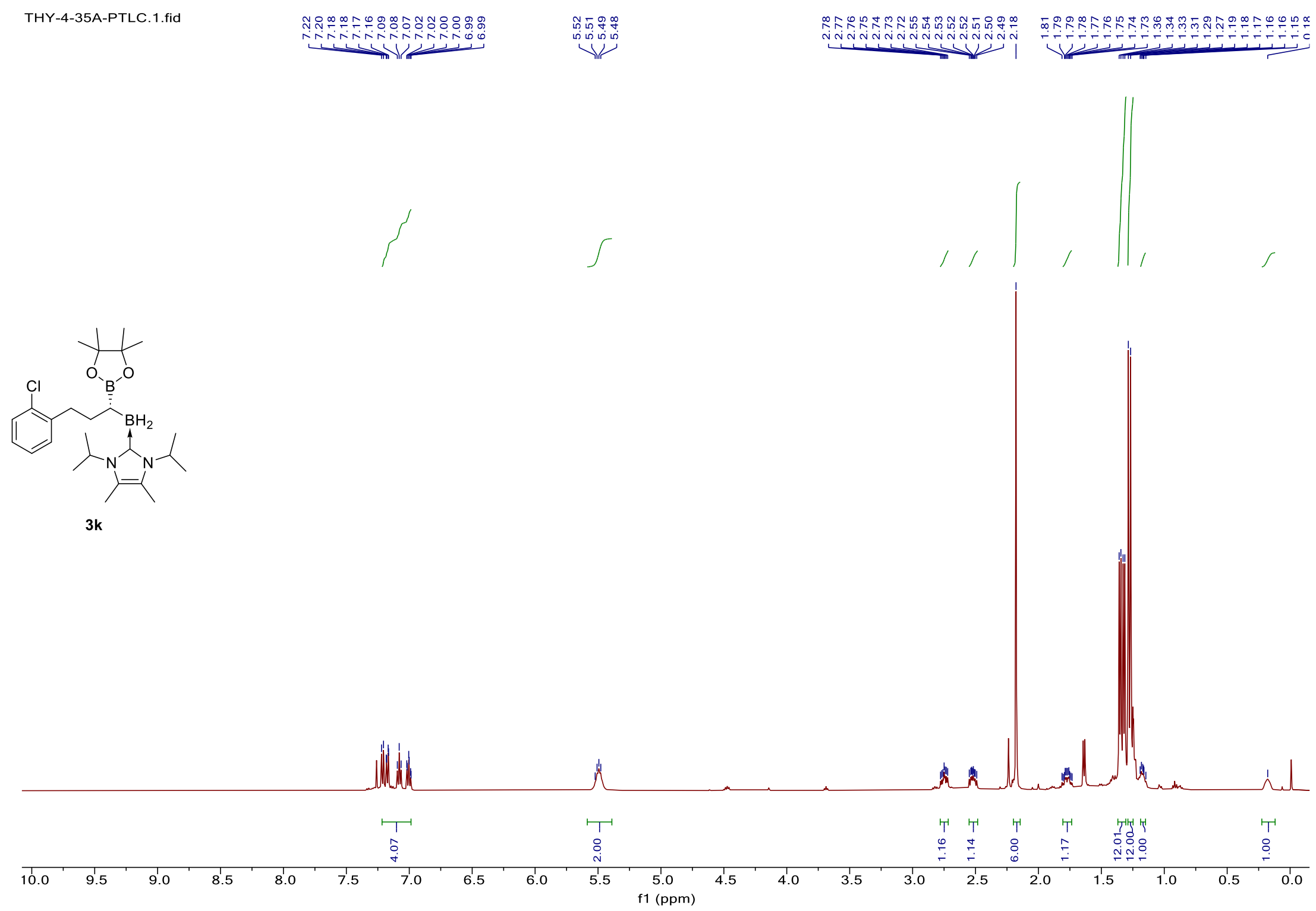

^{1}H NMR (500 MHz, $CDCl_3$)

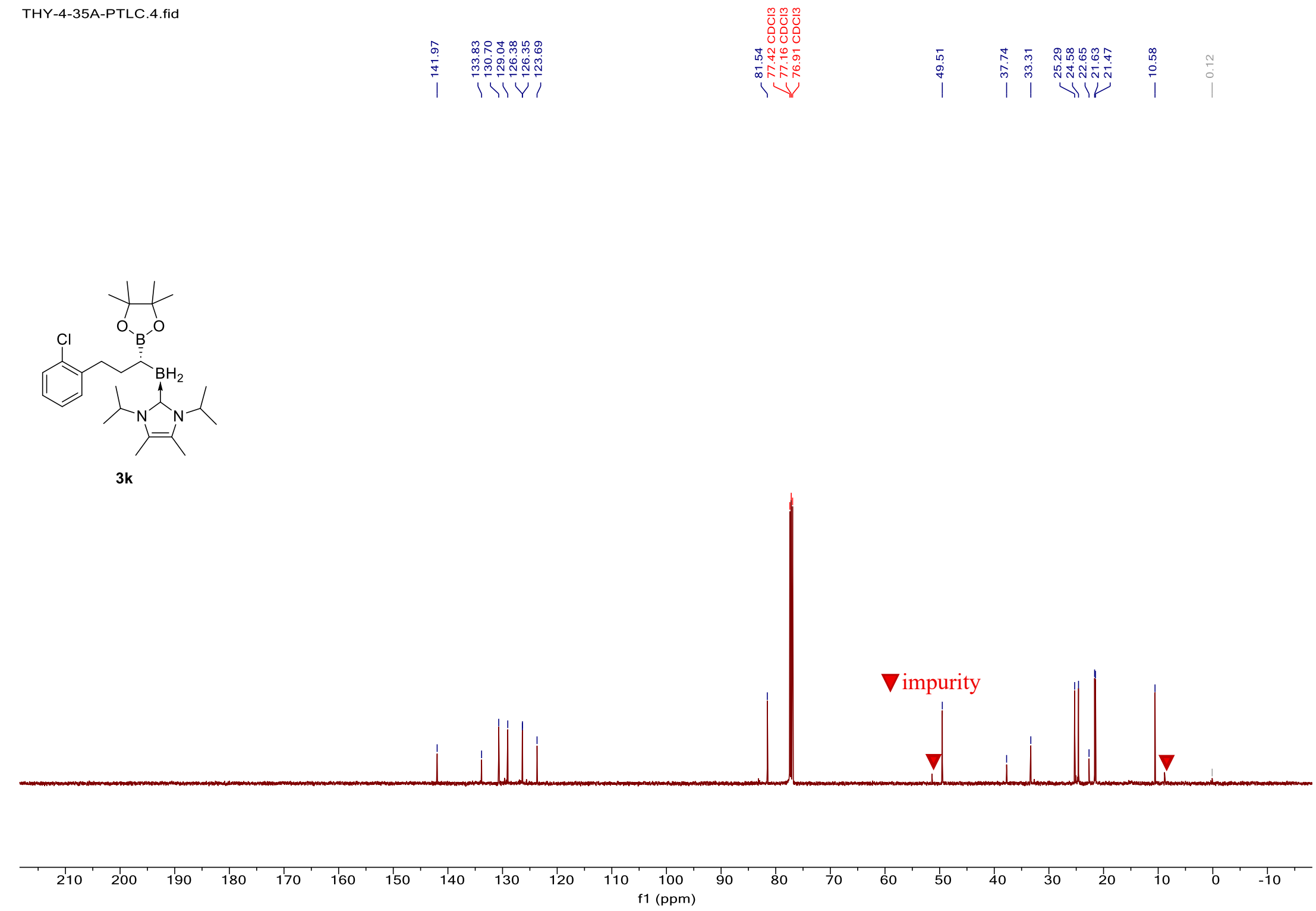

^{13}C NMR (128 MHz, $CDCl_3$)

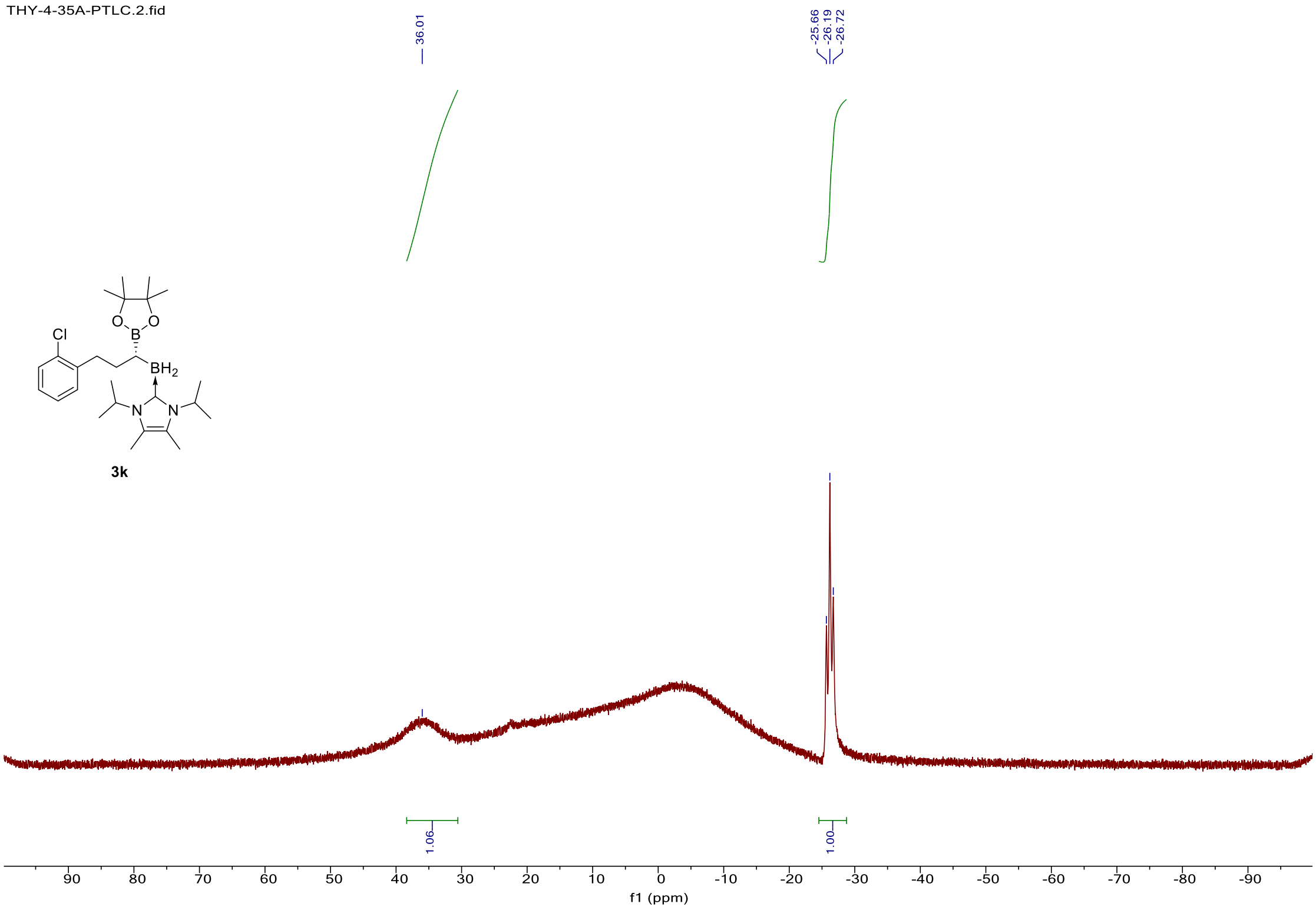

^{11}B NMR (160 MHz, $CDCl_3$)

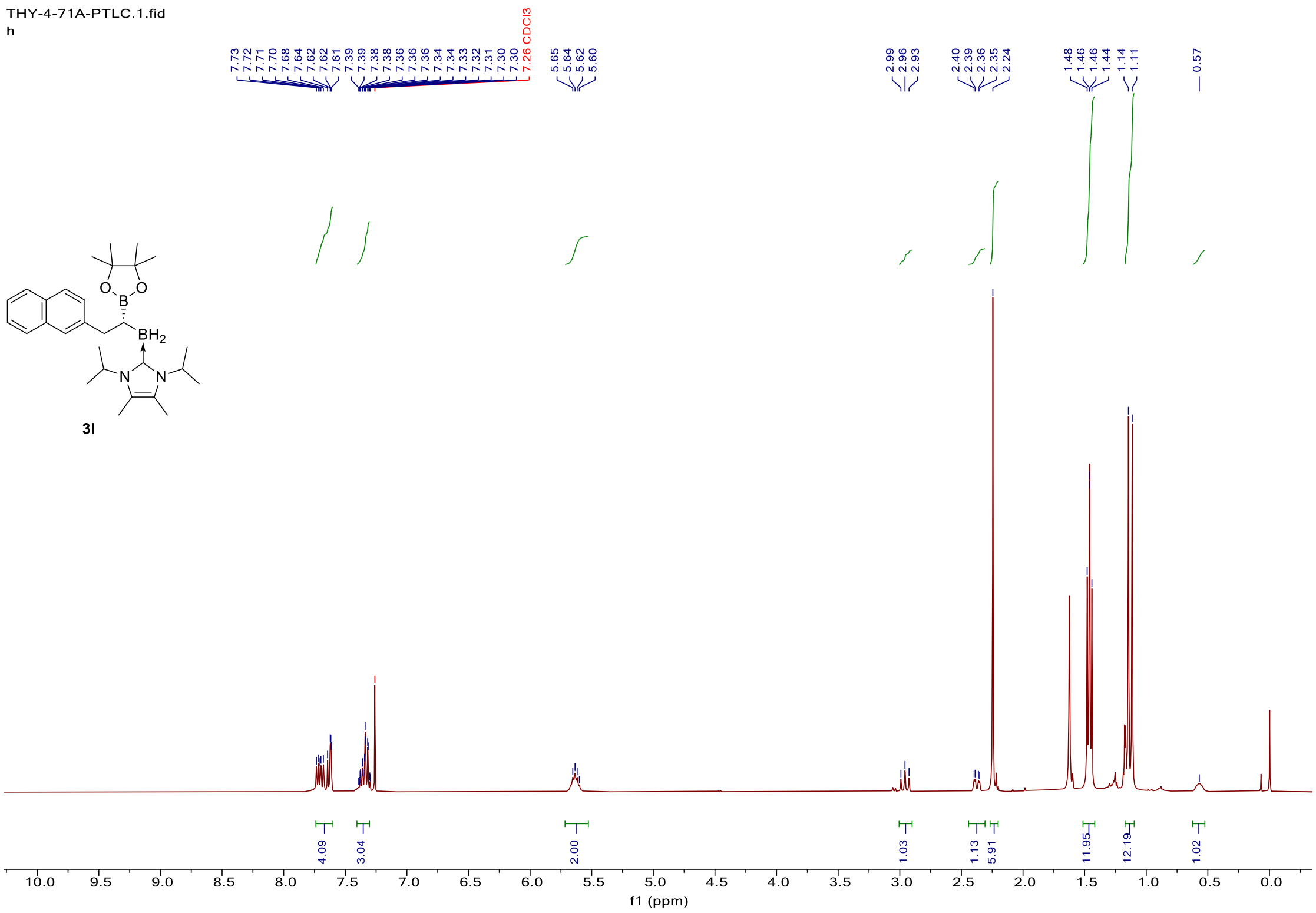

^{1}H NMR (400 MHz, $CDCl_3$)

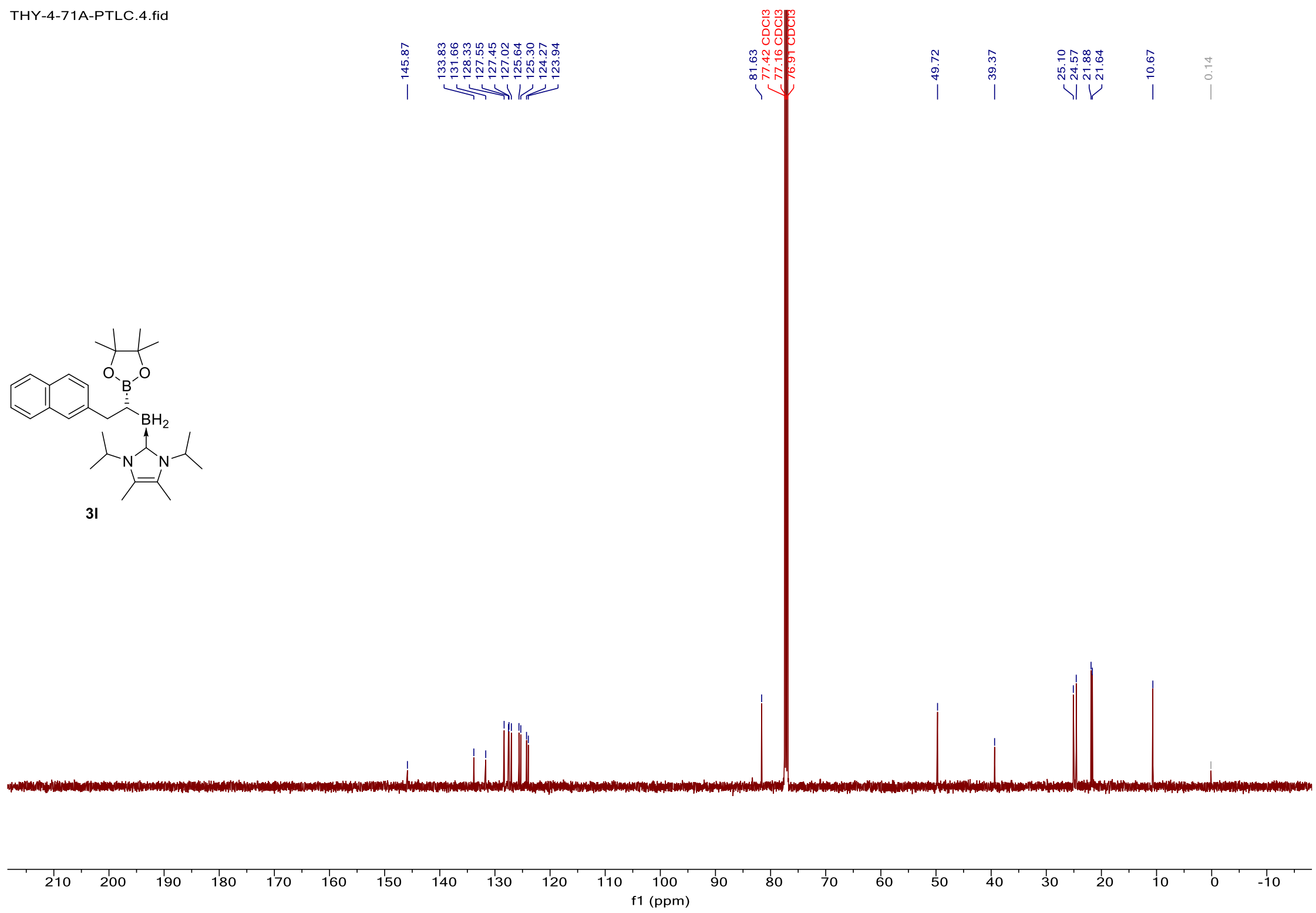

^{13}C NMR (126 MHz, $CDCl_3$)

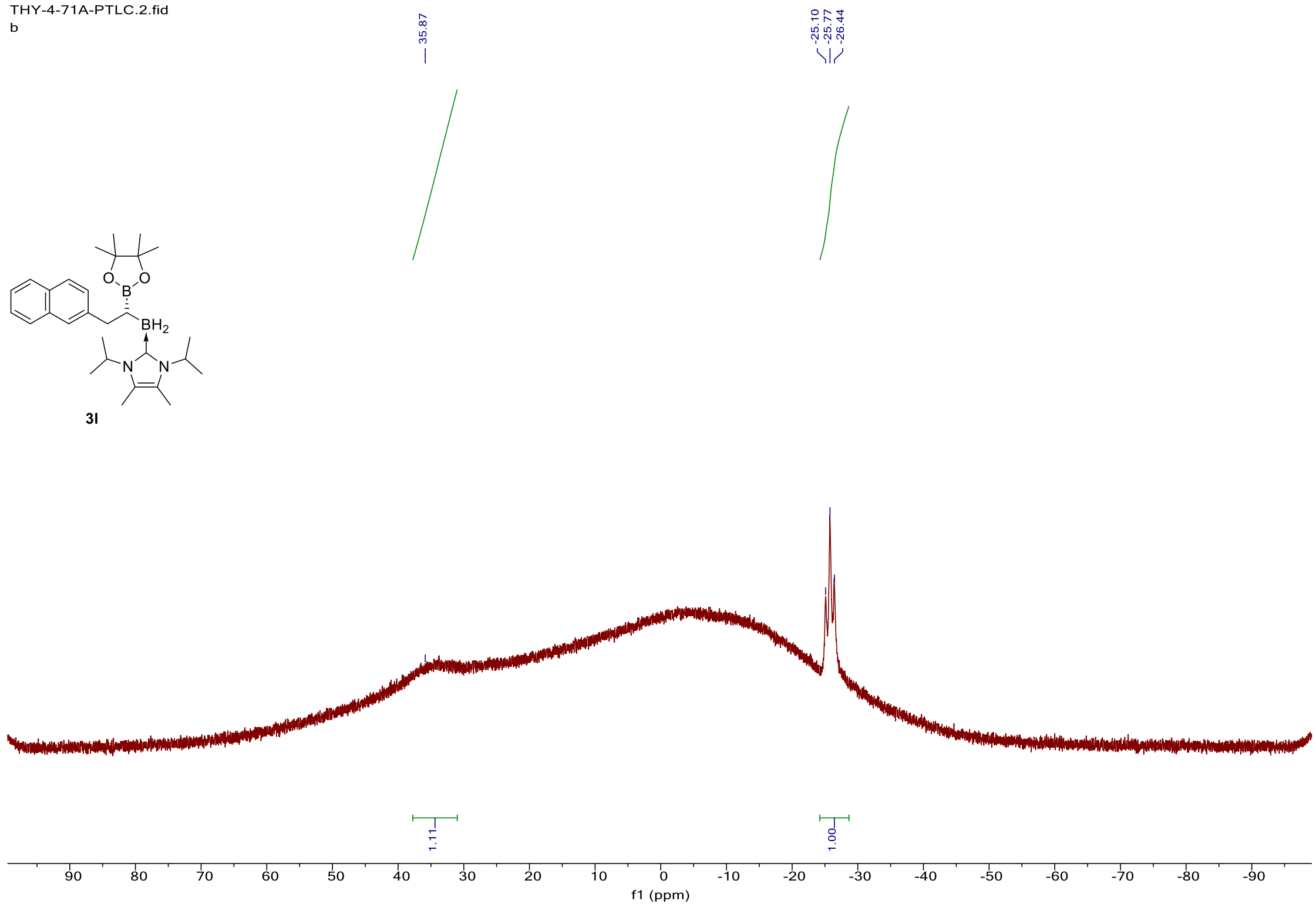

^{11}B NMR (128 MHz, $CDCl_3$)

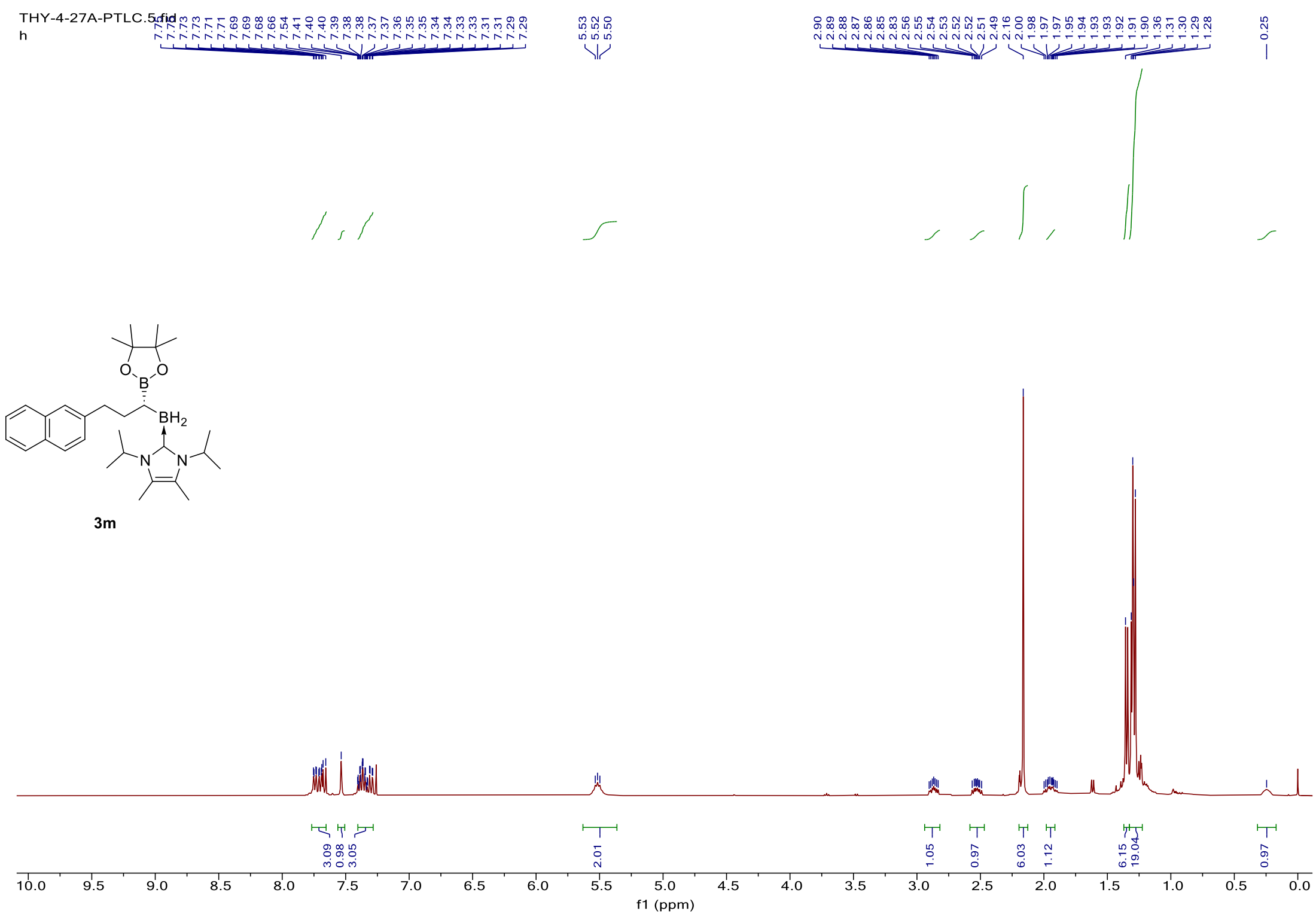

^{1}H NMR (400 MHz, $CDCl_3$)

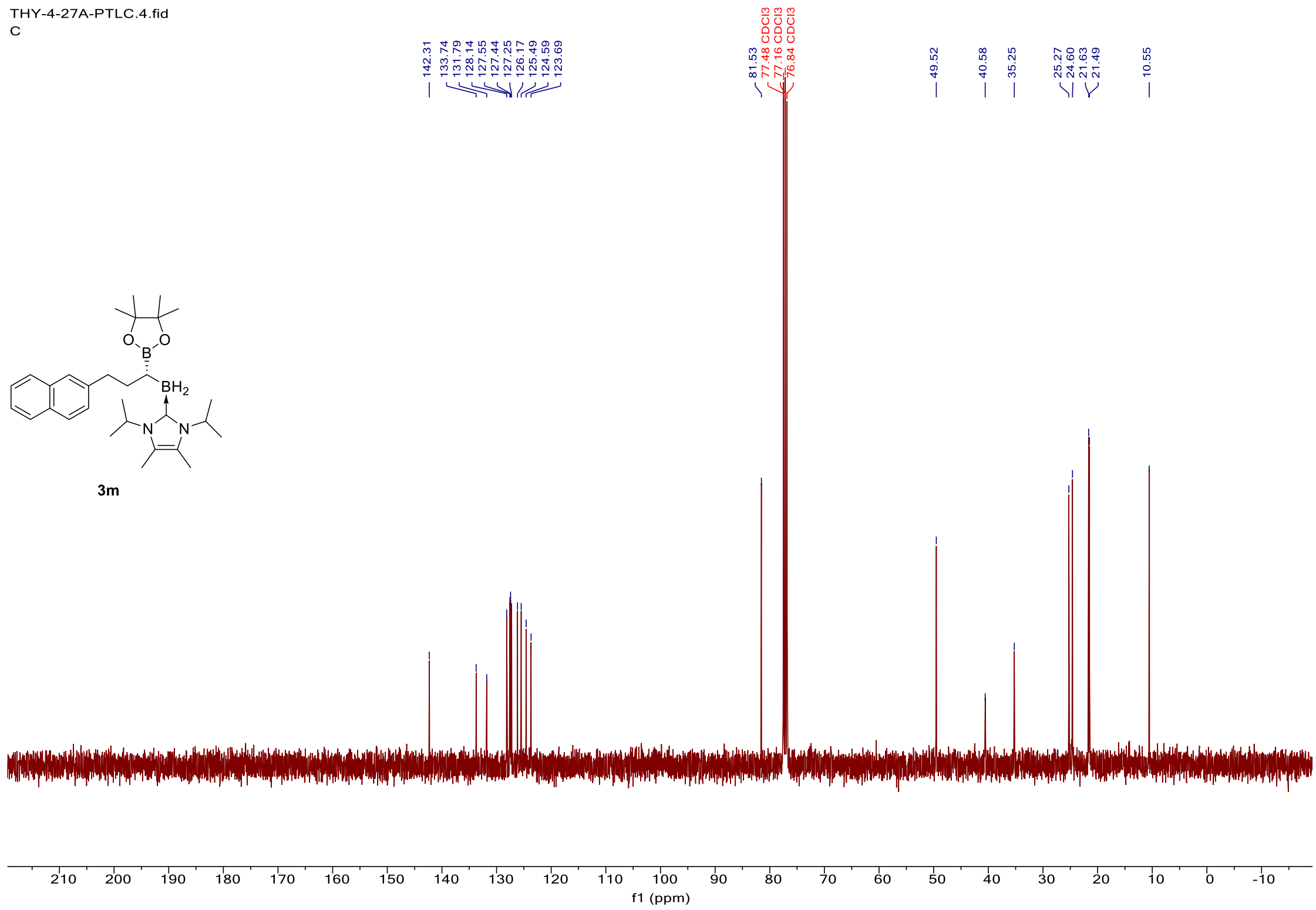

^{13}C NMR (101 MHz, $CDCl_3$)

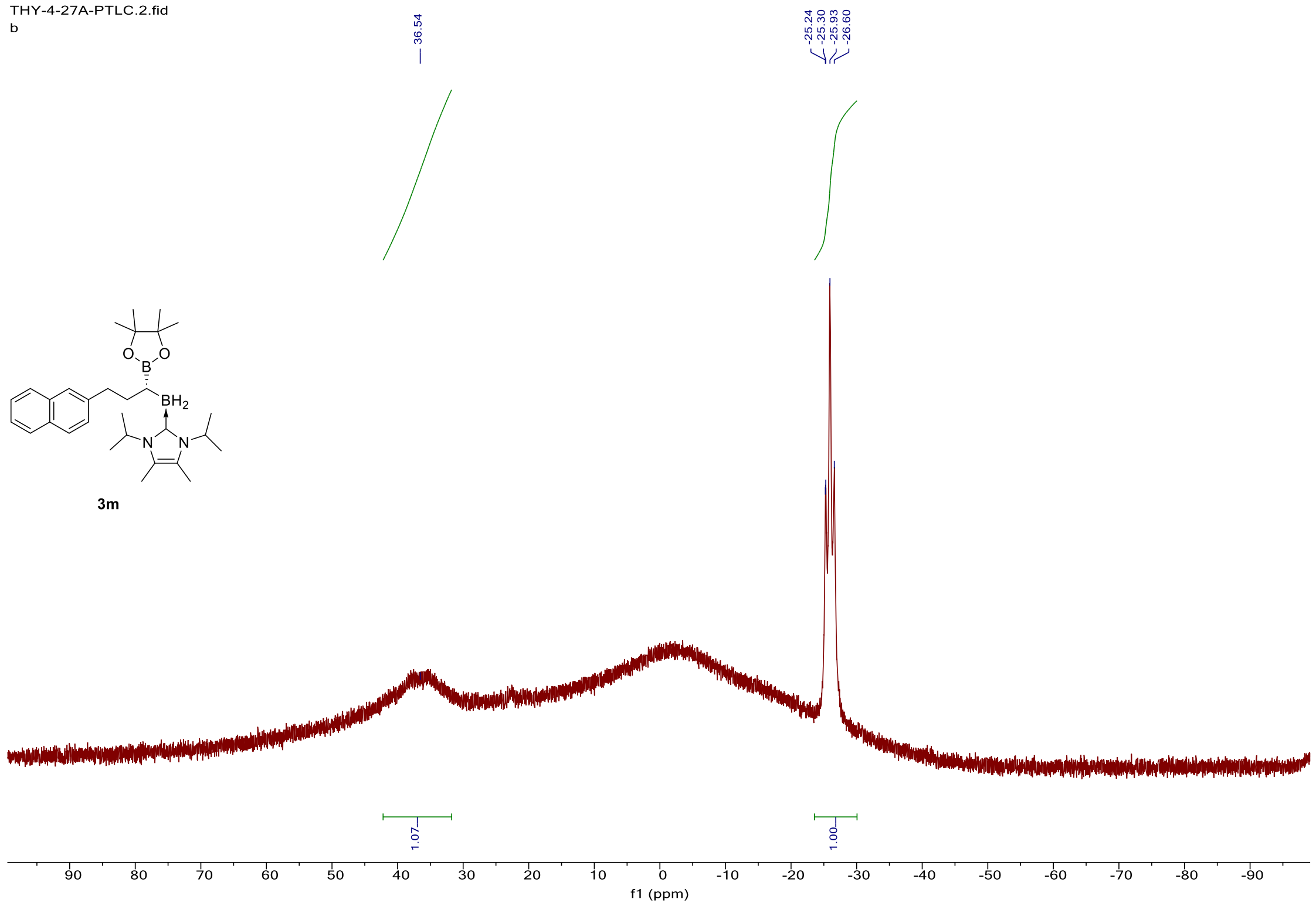

^{11}B NMR (128 MHz, $CDCl_3$)

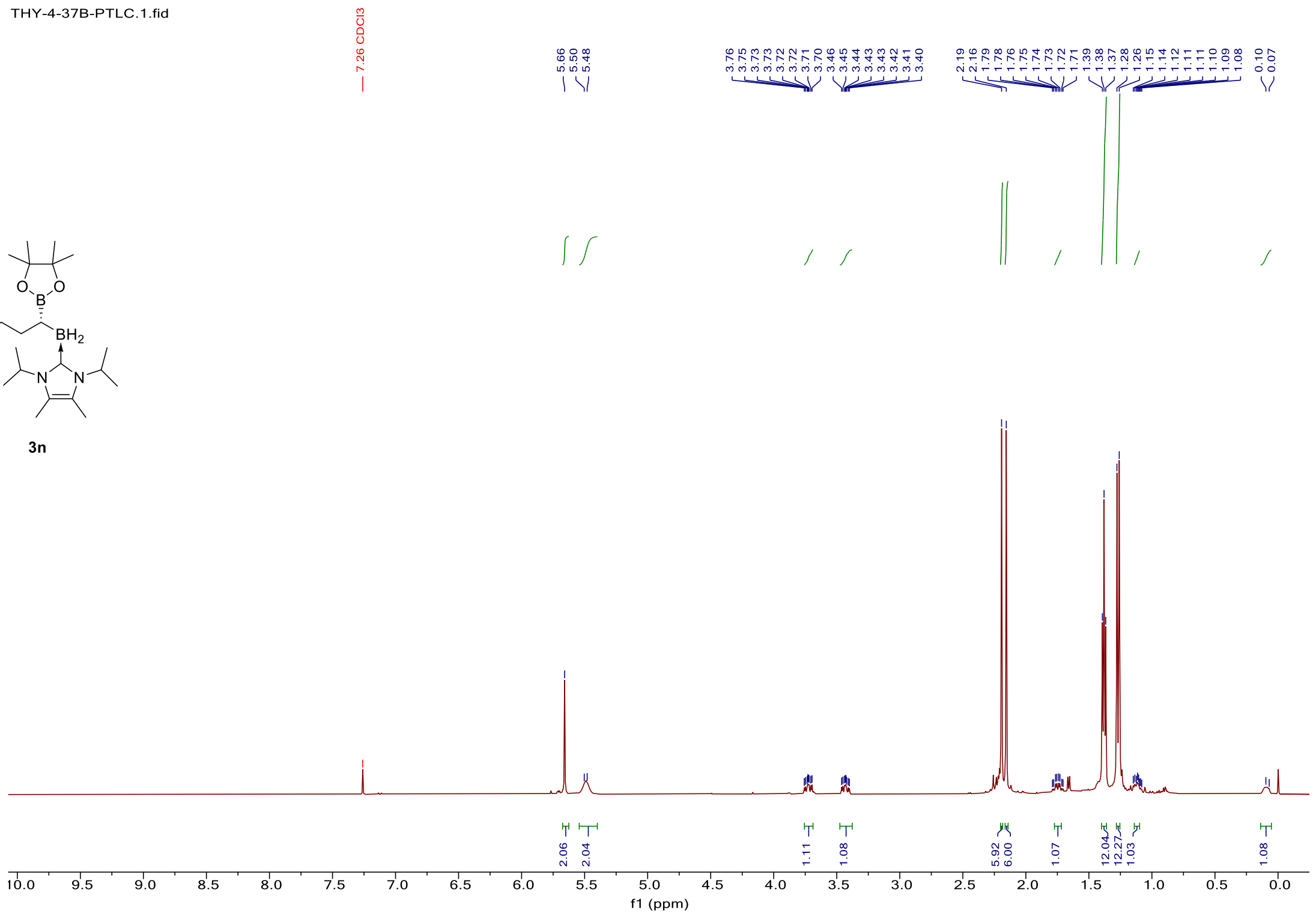

^{1}H NMR (500 MHz, $CDCl_3$)

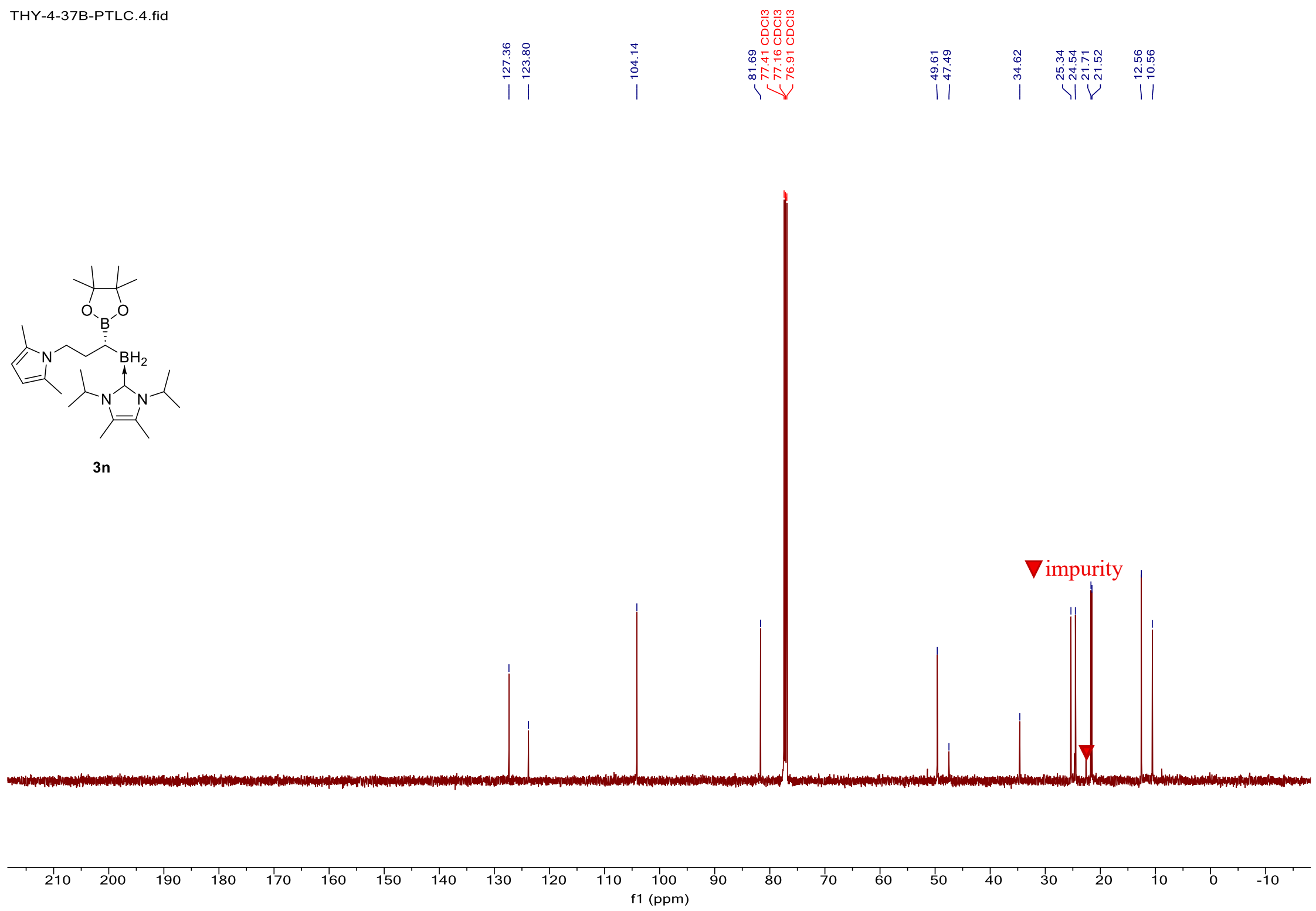

^{13}C NMR (126 MHz, $CDCl_3$)

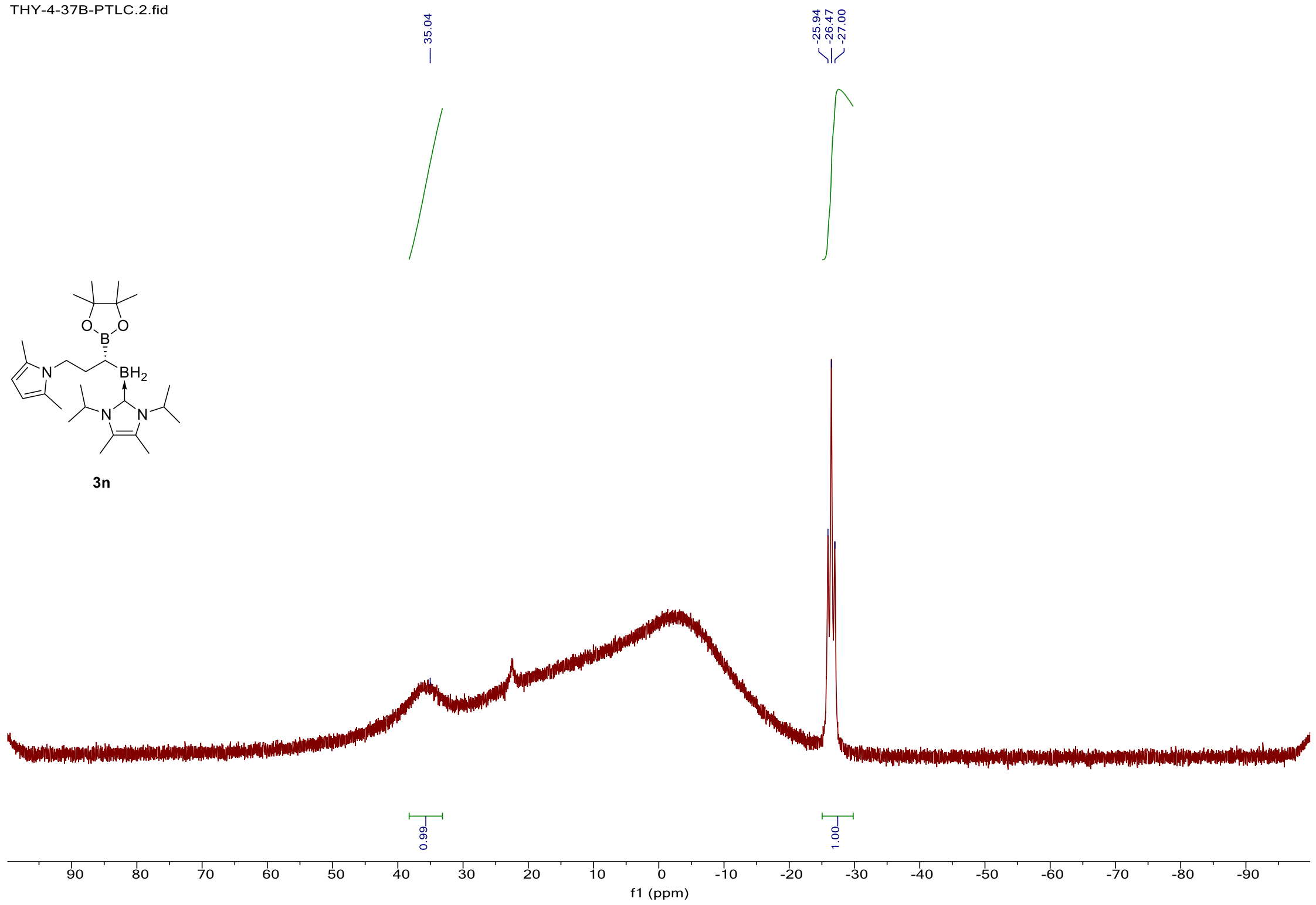

^{11}B NMR (160 MHz, $CDCl_3$)

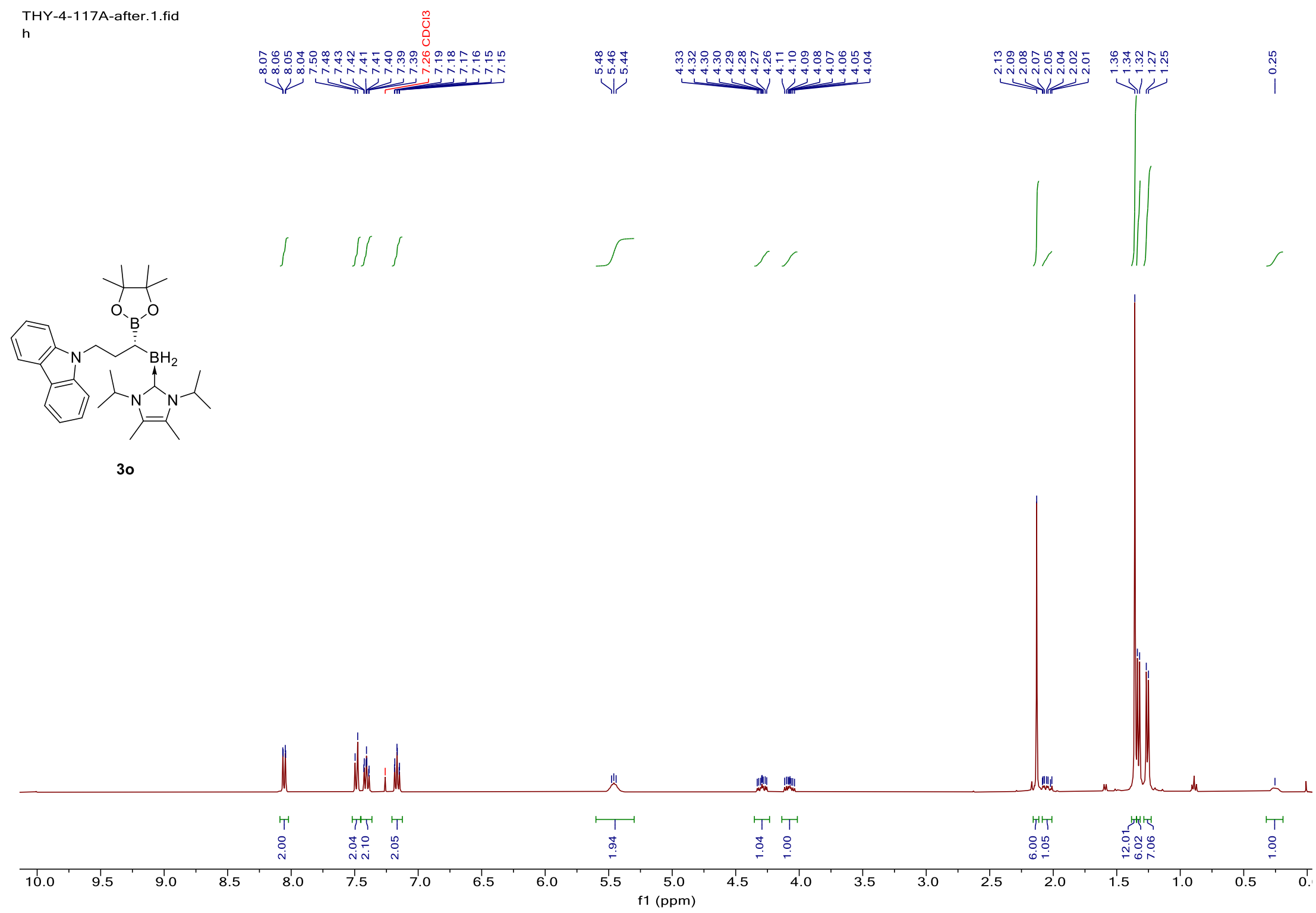

^1H NMR (400 MHz, $CDCl_3$)

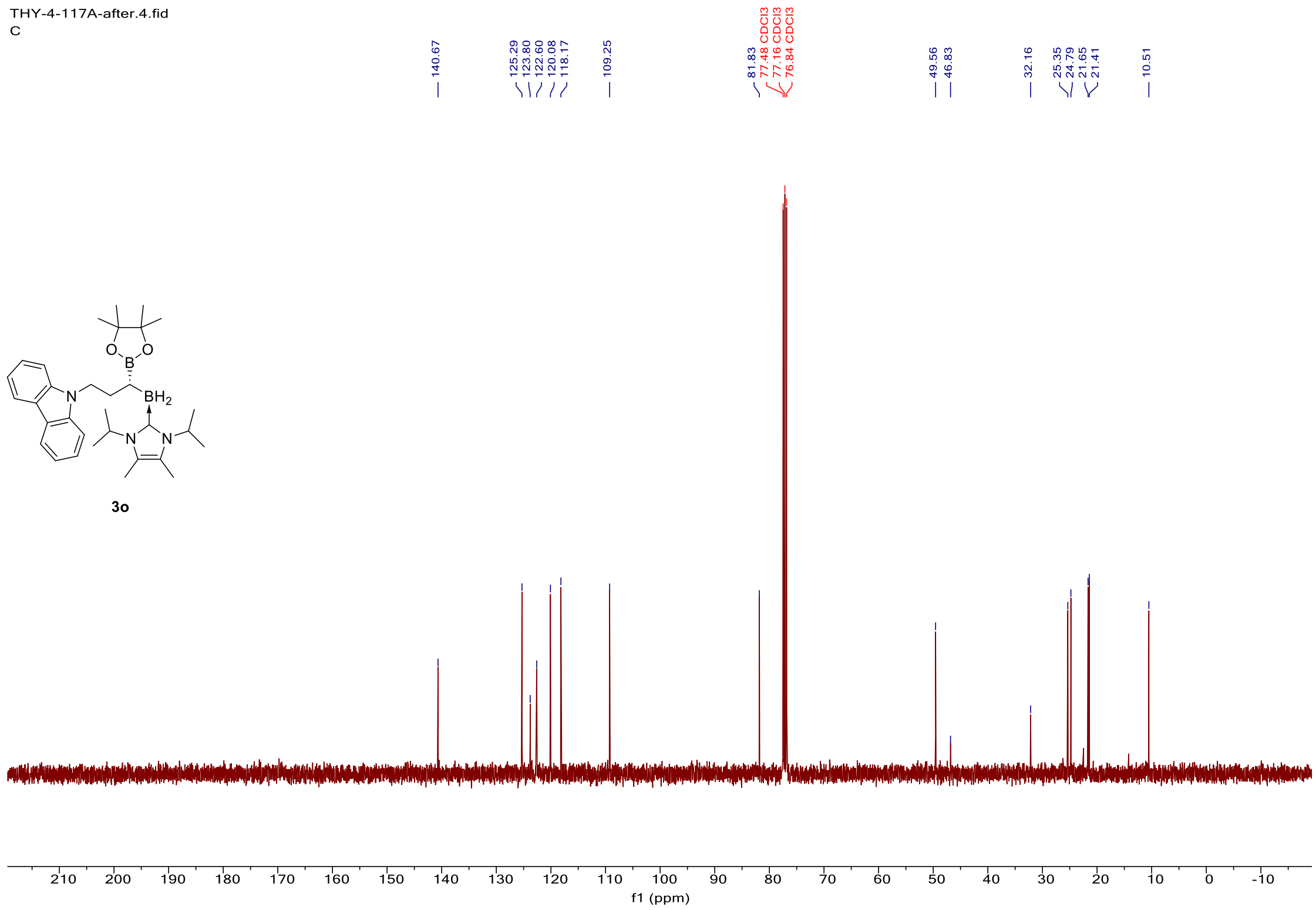

^{13}C NMR (101 MHz, $CDCl_3$)

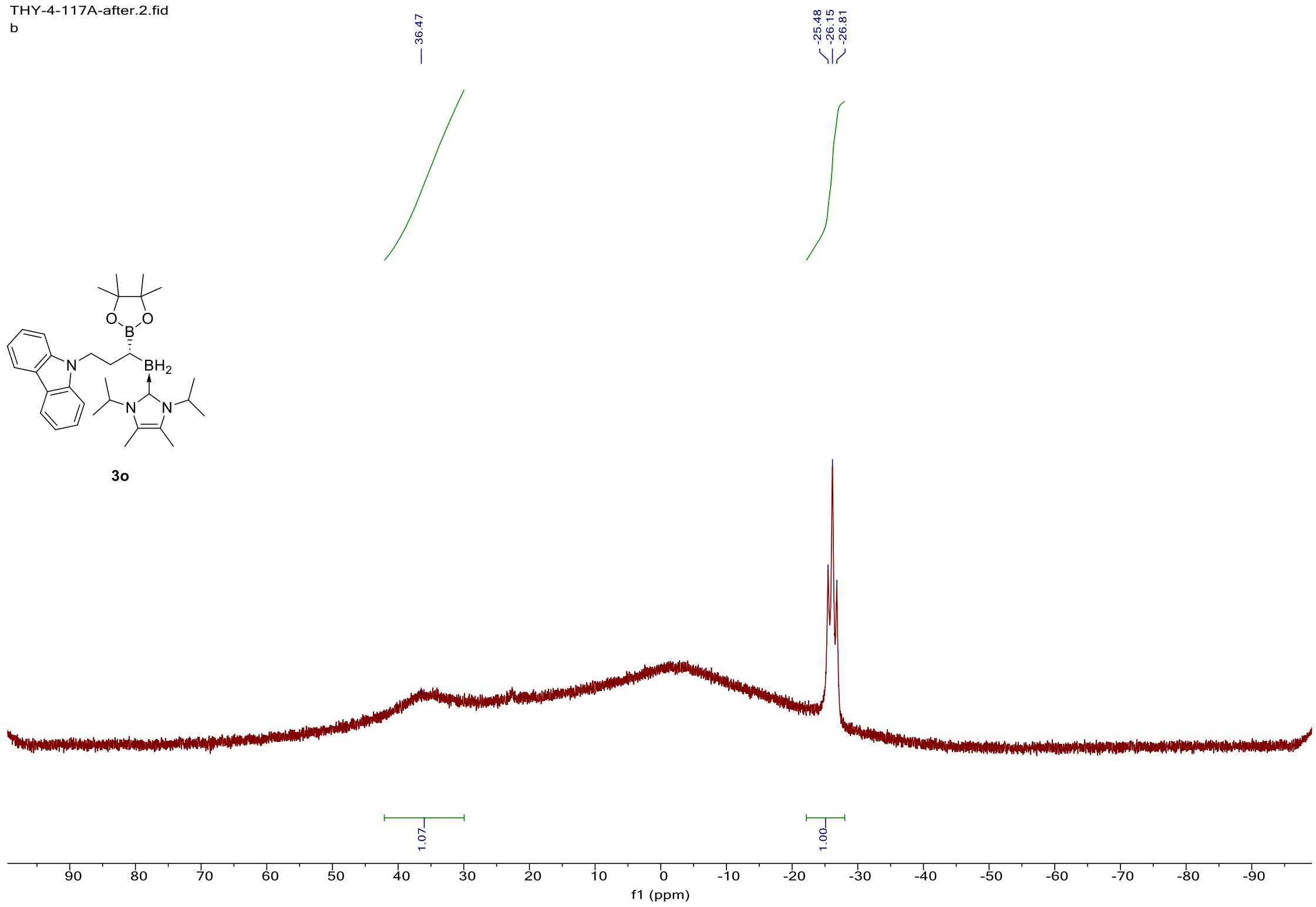

^{11}B NMR (128 MHz, $CDCl_3$)

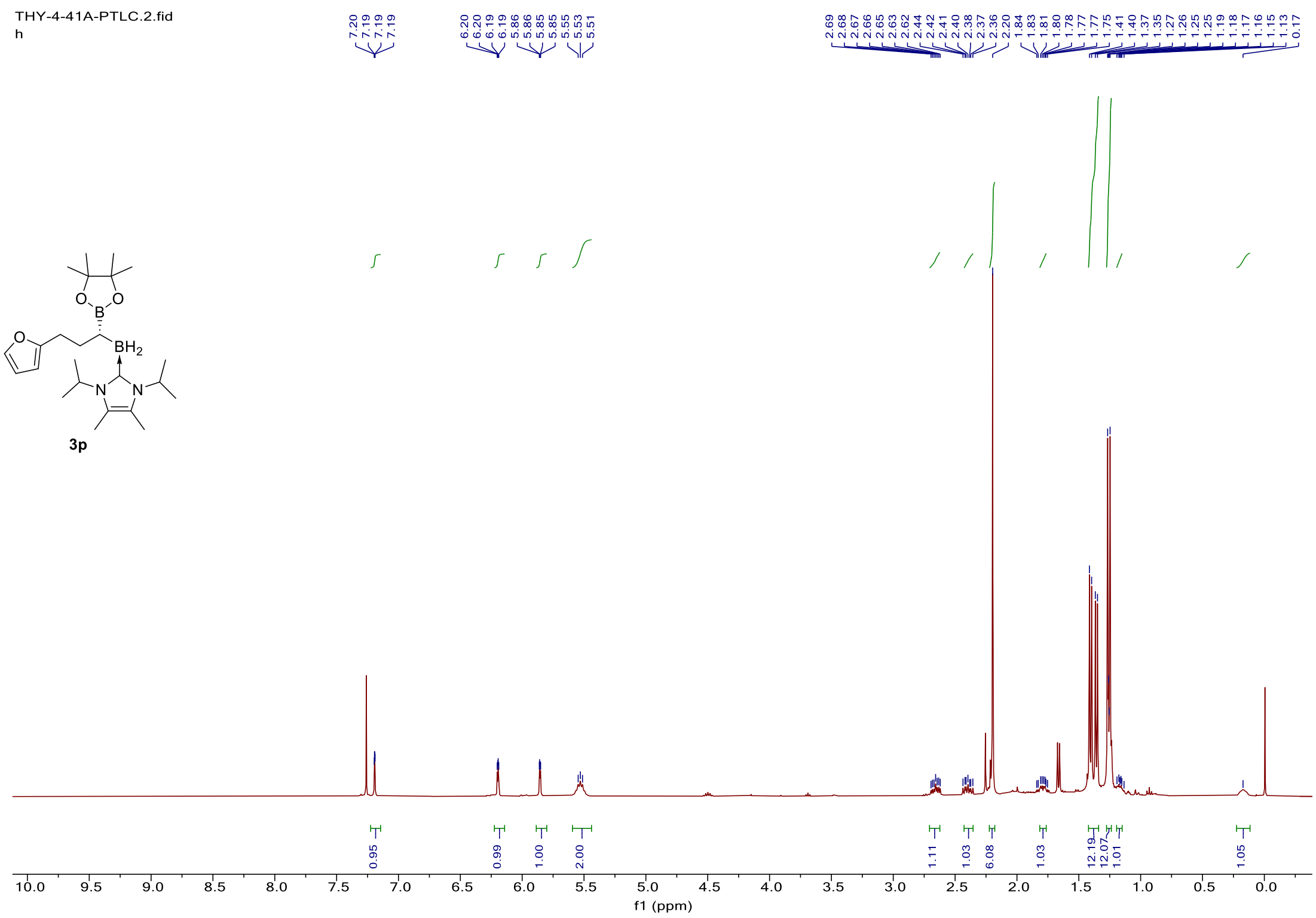

^{1}H NMR (400 MHz, $CDCl_3$)

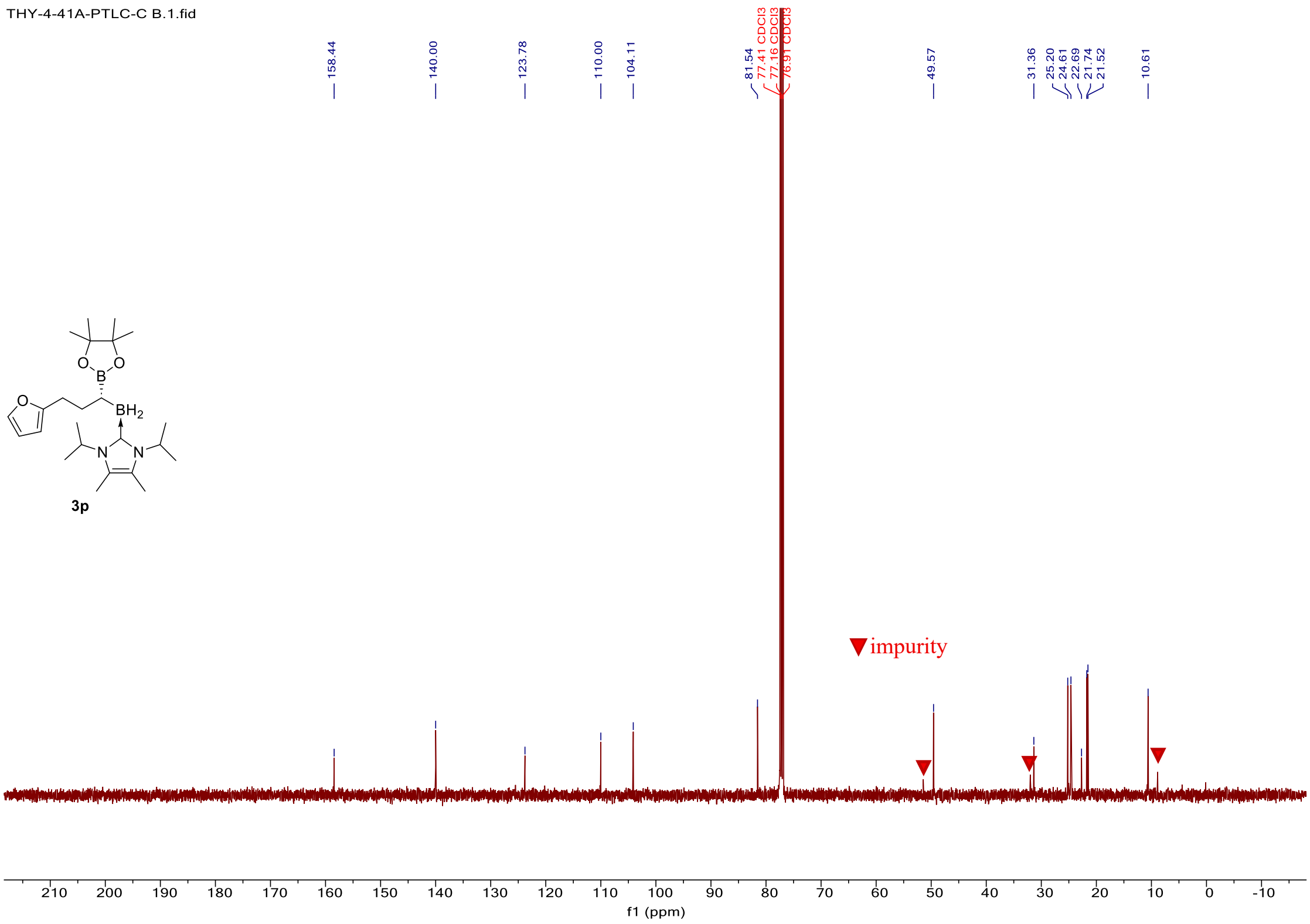

^{13}C NMR (126 MHz, $CDCl_3$)

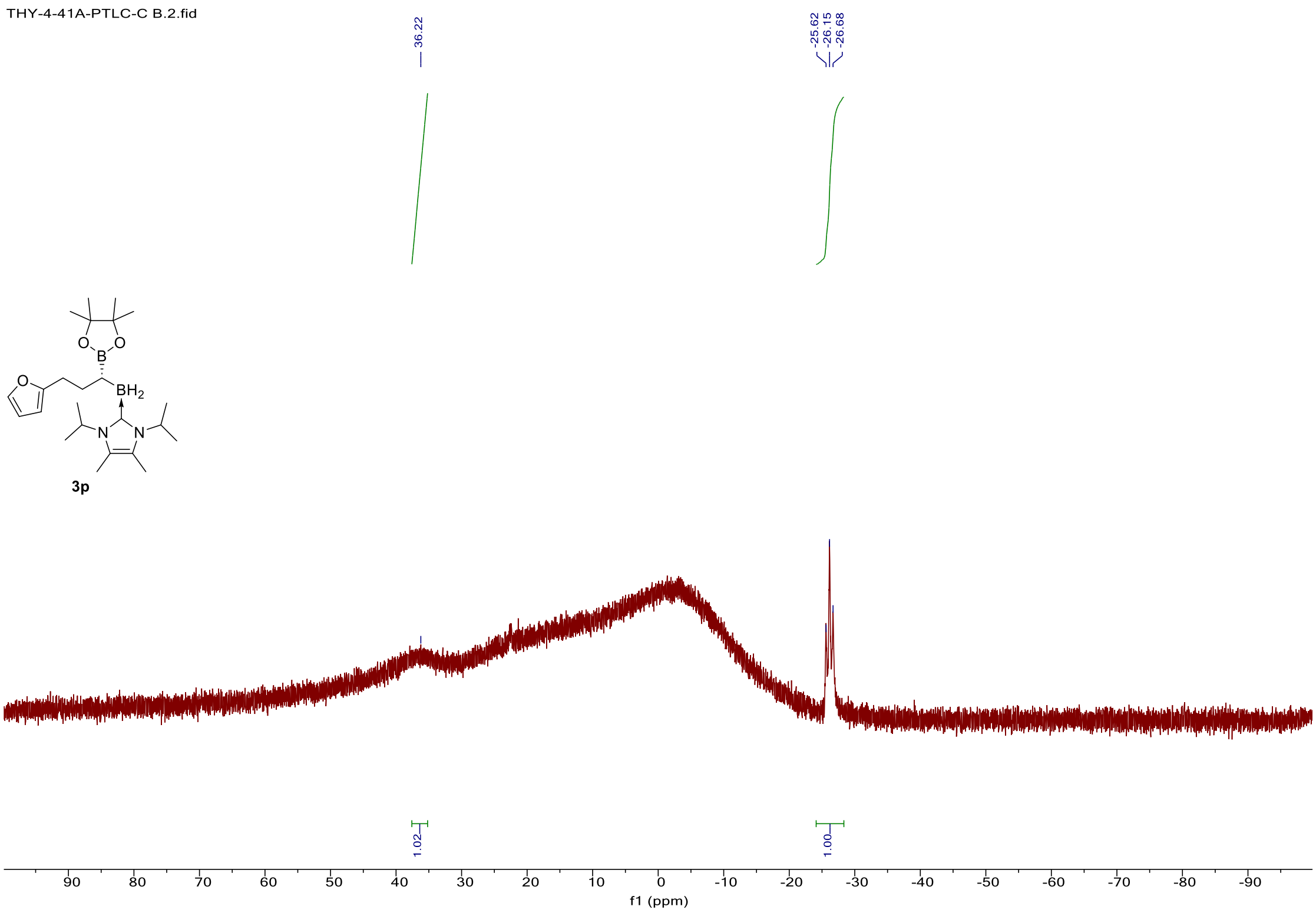

^{11}B NMR (160 MHz, $CDCl_3$)

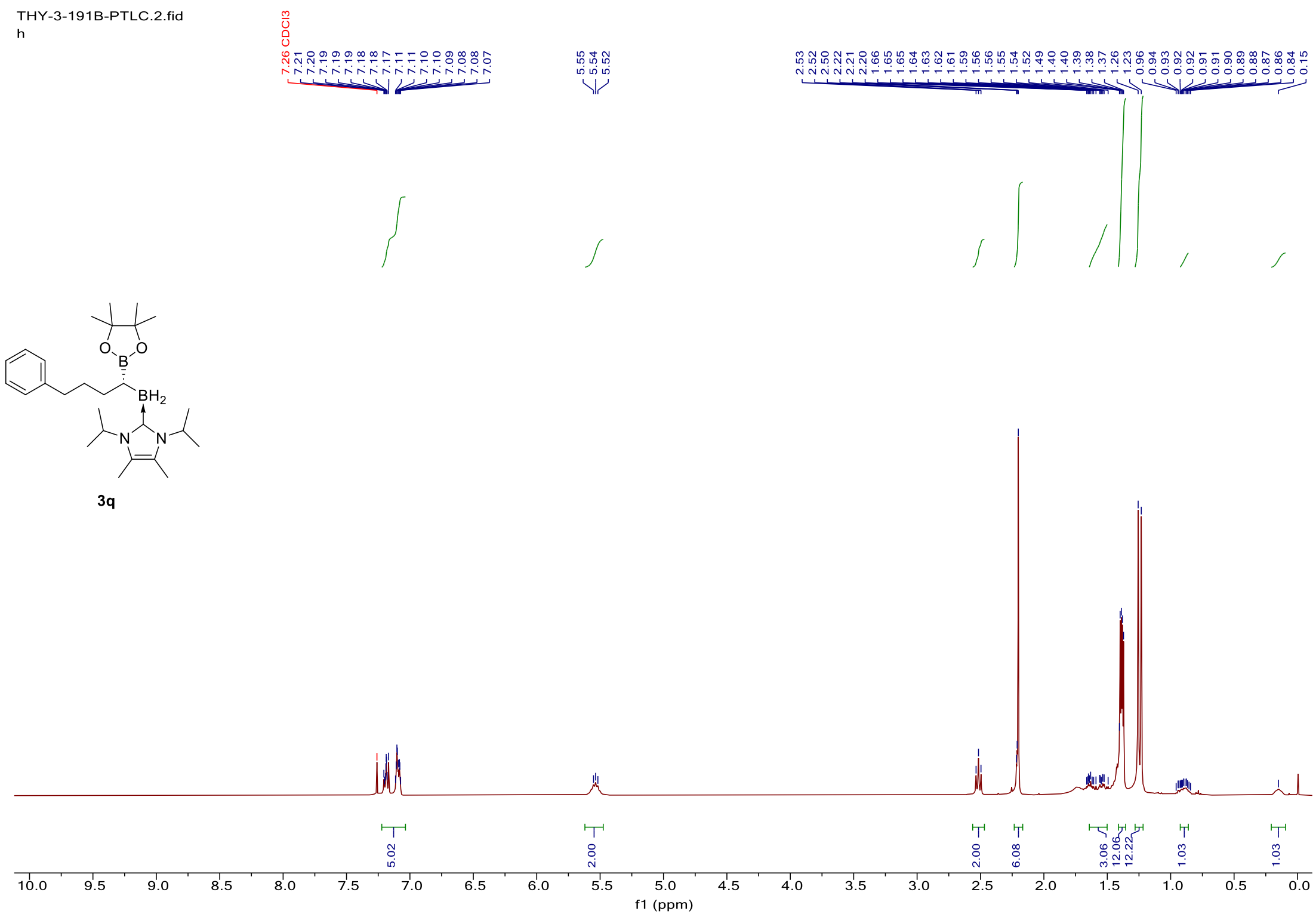

^1H NMR (400 MHz, $CDCl_3$)

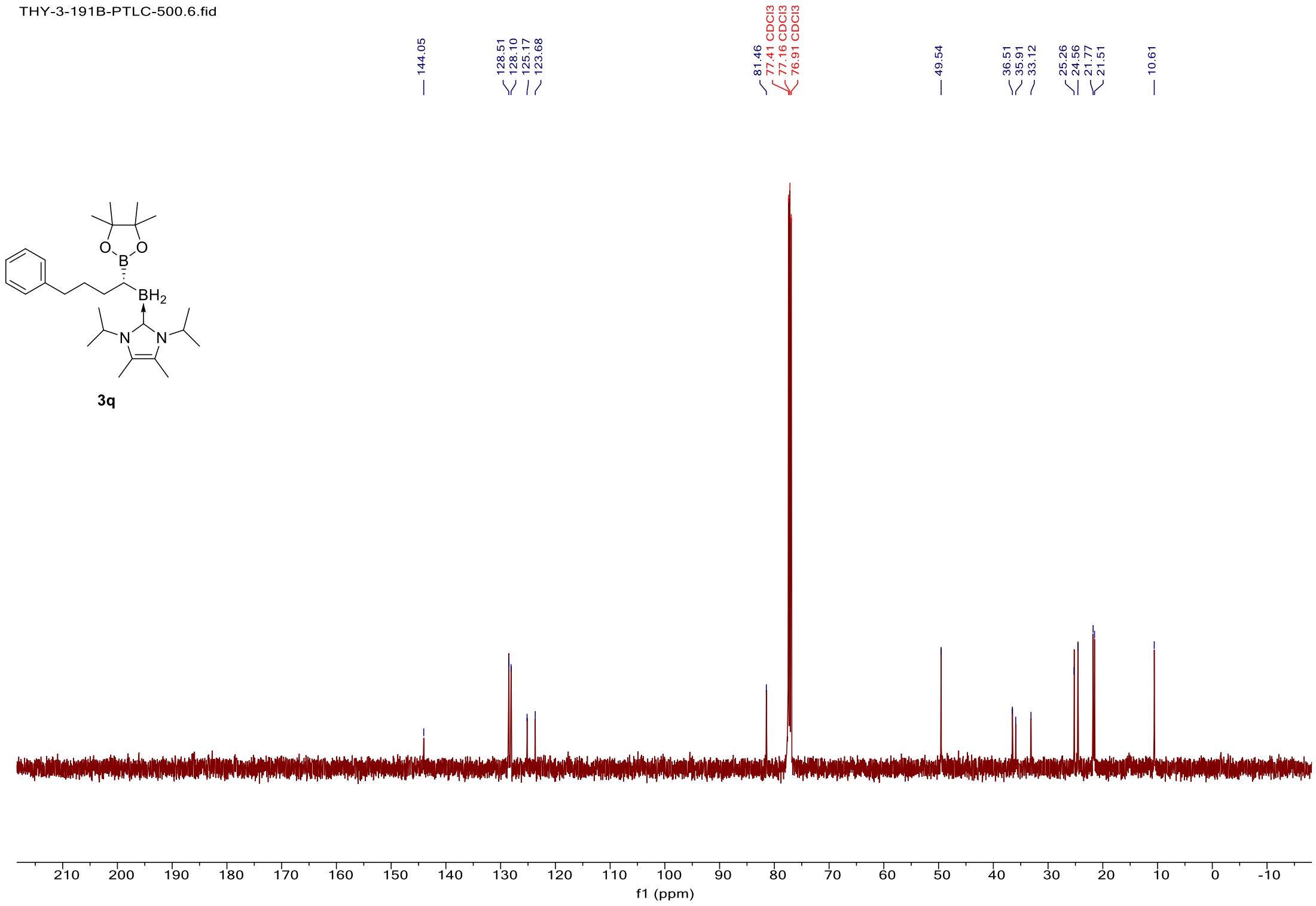

^{13}C NMR (126 MHz, $CDCl_3$)

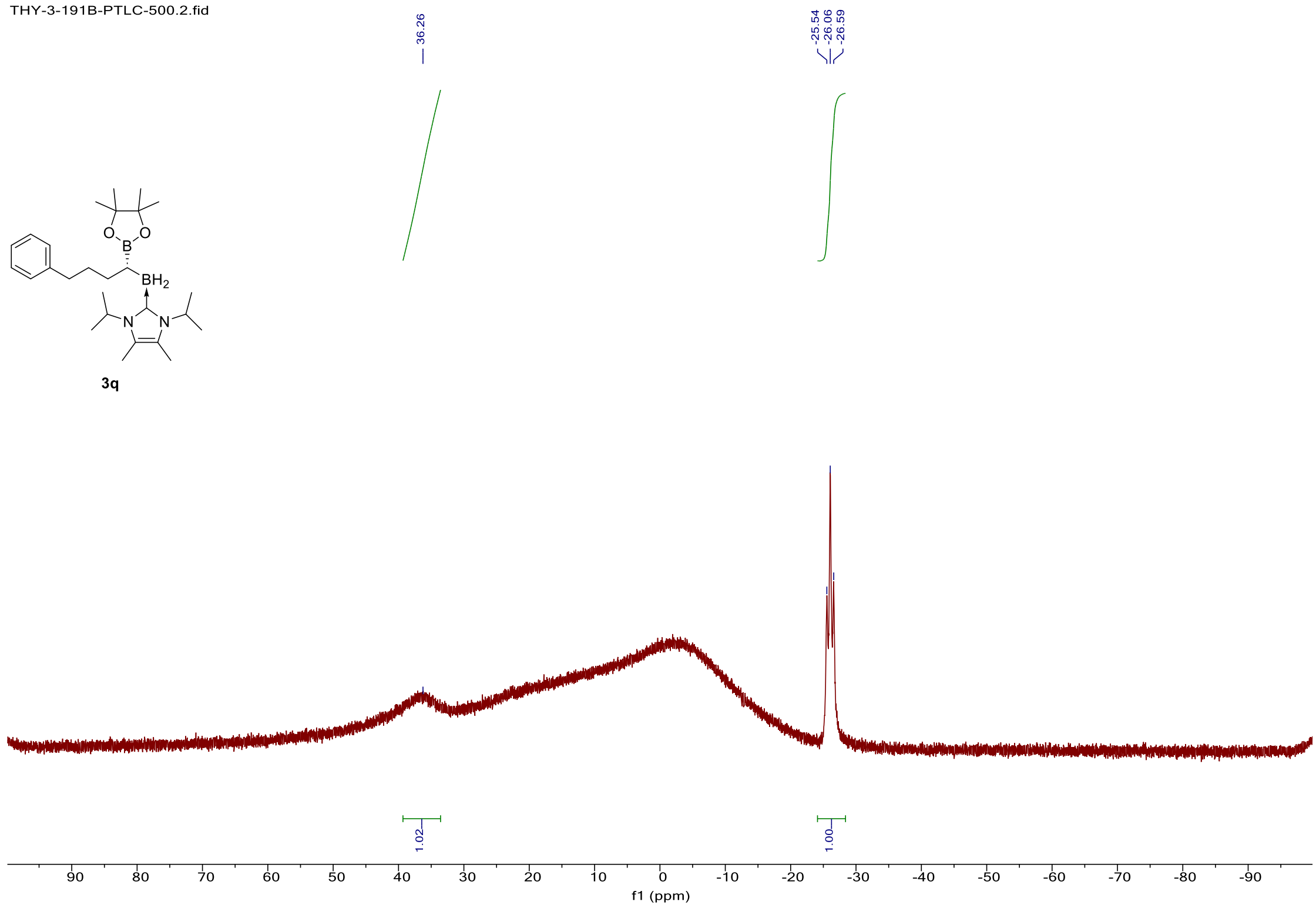

^{11}B NMR (160 MHz, $CDCl_3$)

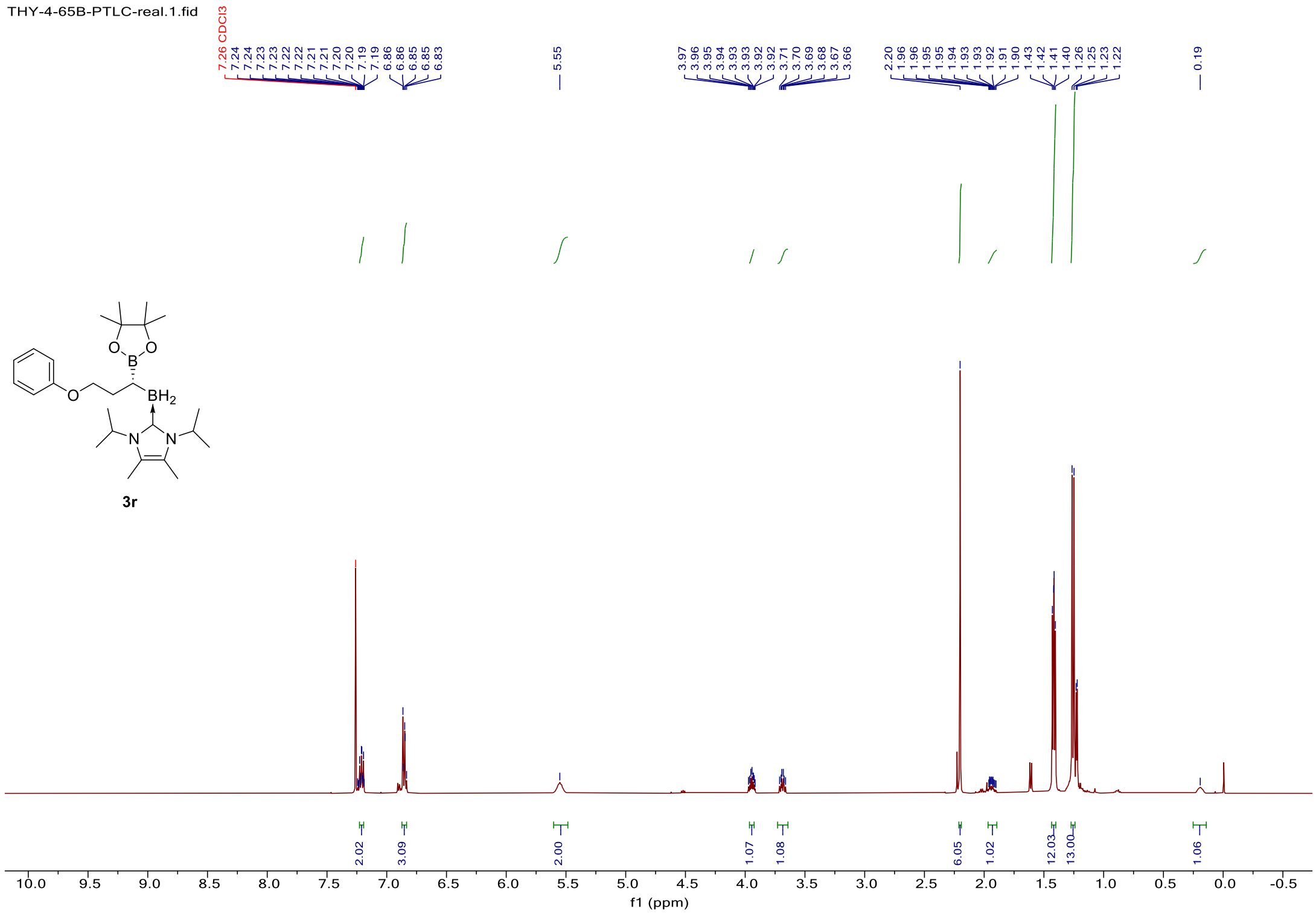

^{1}H NMR (500 MHz, $CDCl_3$)

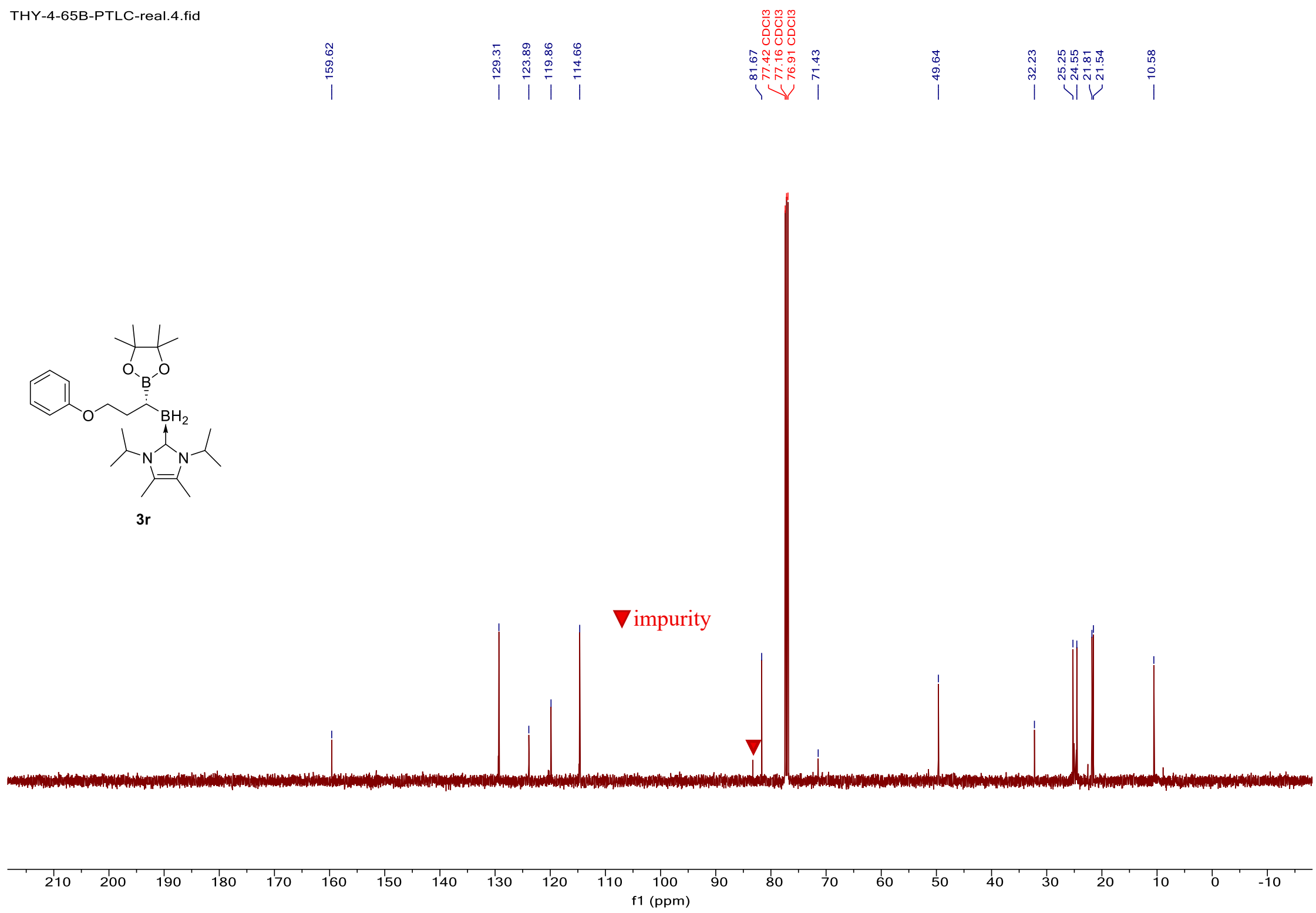

^{13}C NMR (126 MHz, CDCl$_3$)

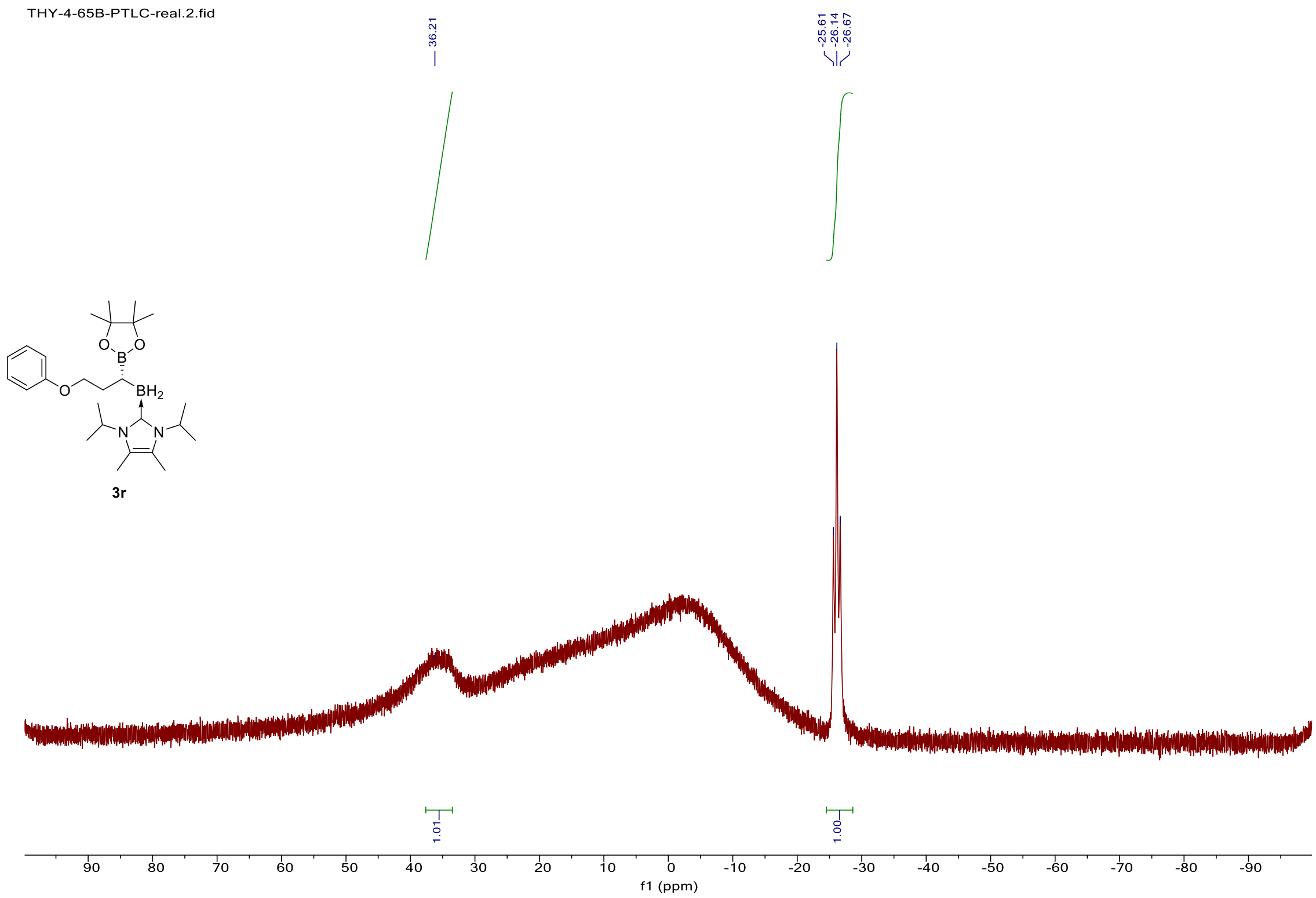

^{11}B NMR (160 MHz, CDCl$_3$)

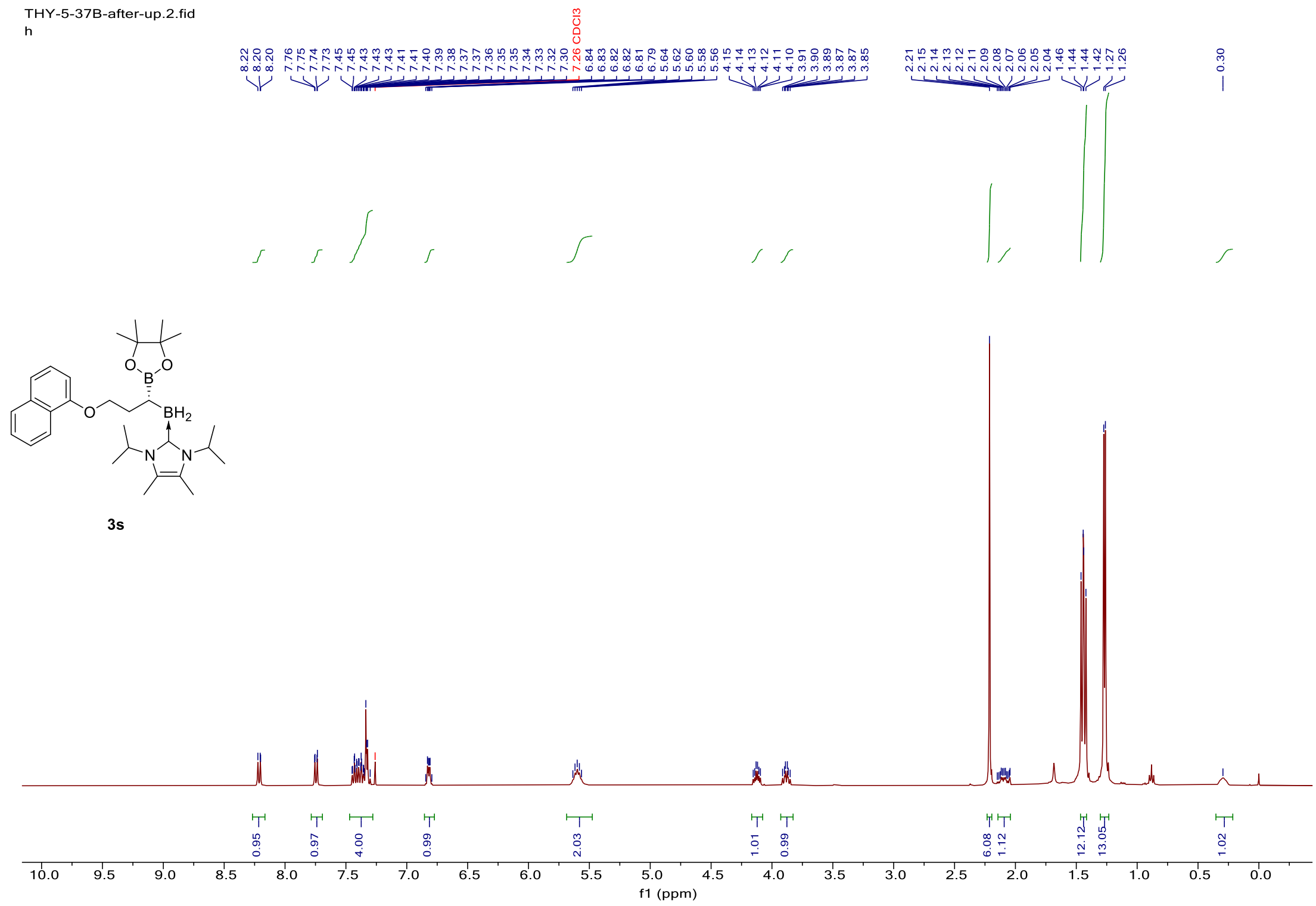

^{1}H NMR (400 MHz, $CDCl_3$)

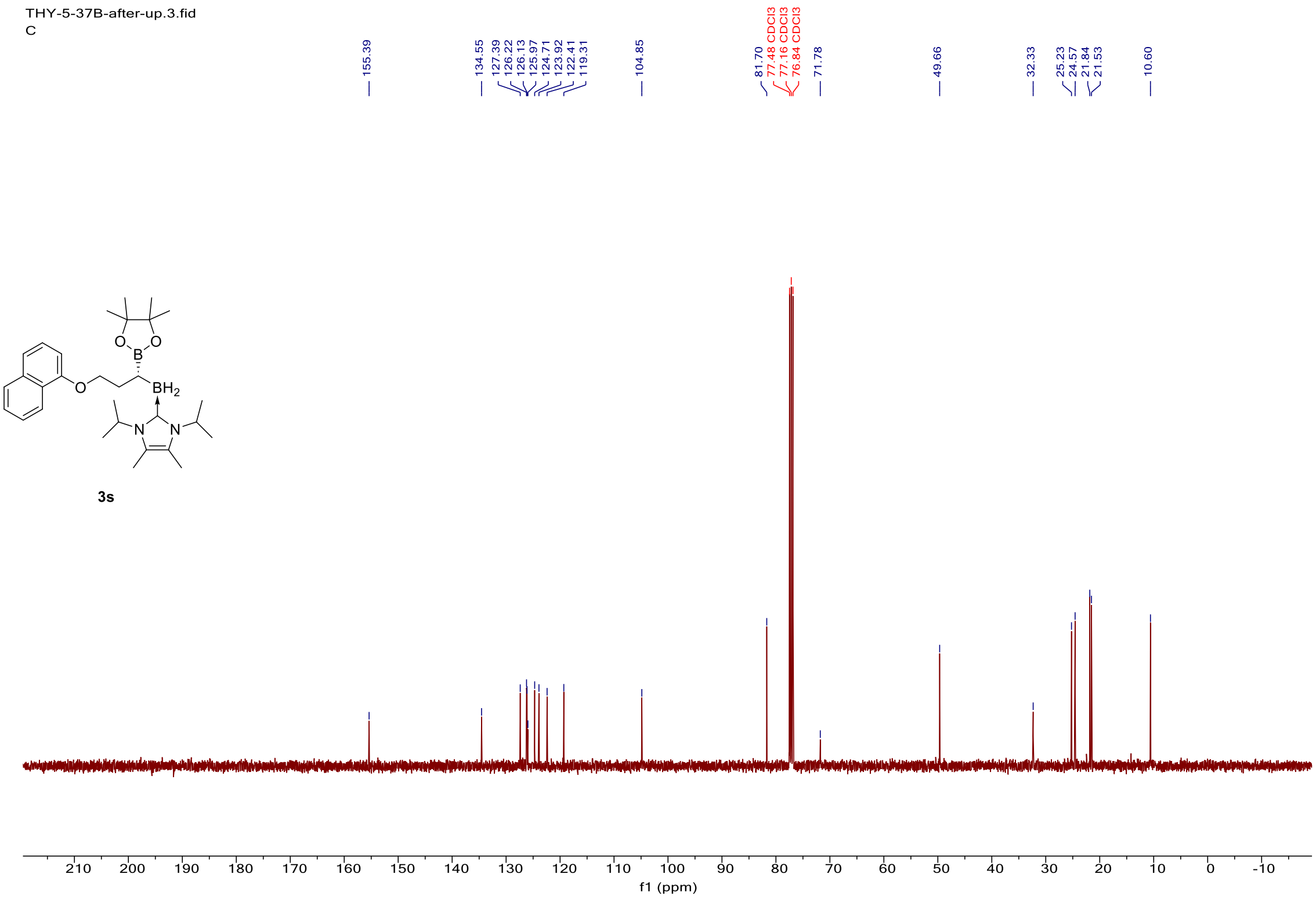

^{13}C NMR (101 MHz, $CDCl_3$)

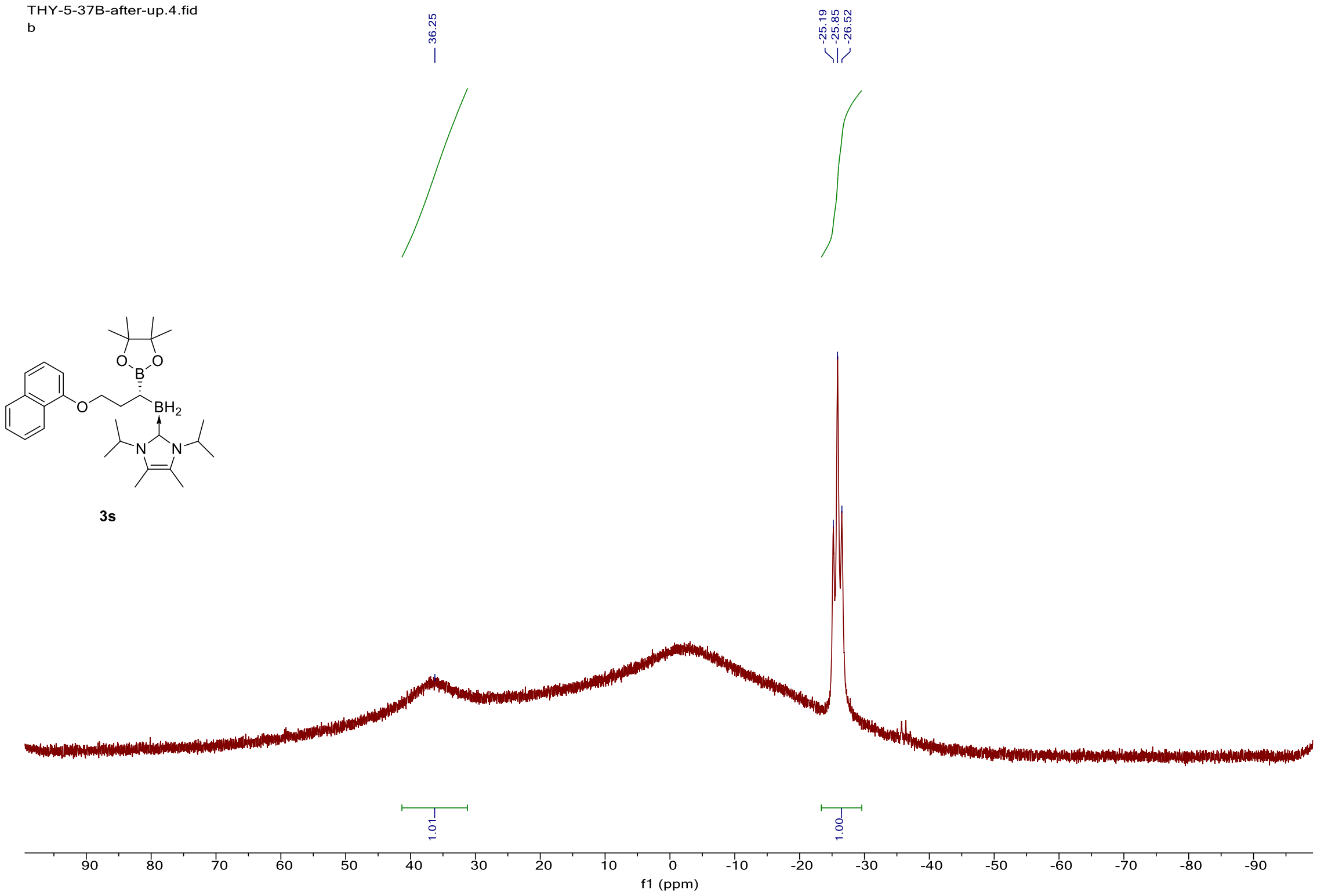

^{11}B NMR (128 MHz, $CDCl_3$)

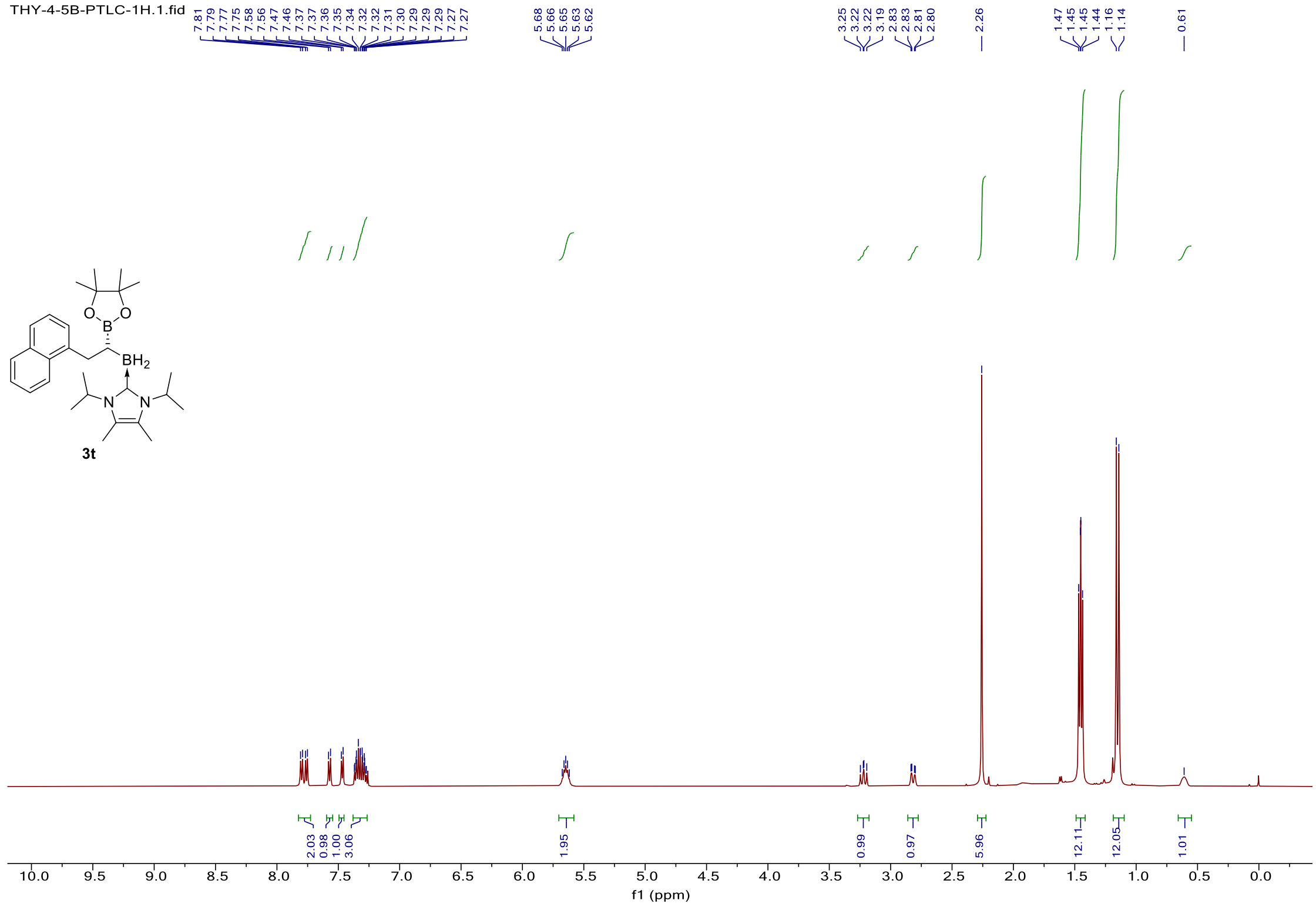

^{1}H NMR (500 MHz, $CDCl_3$)

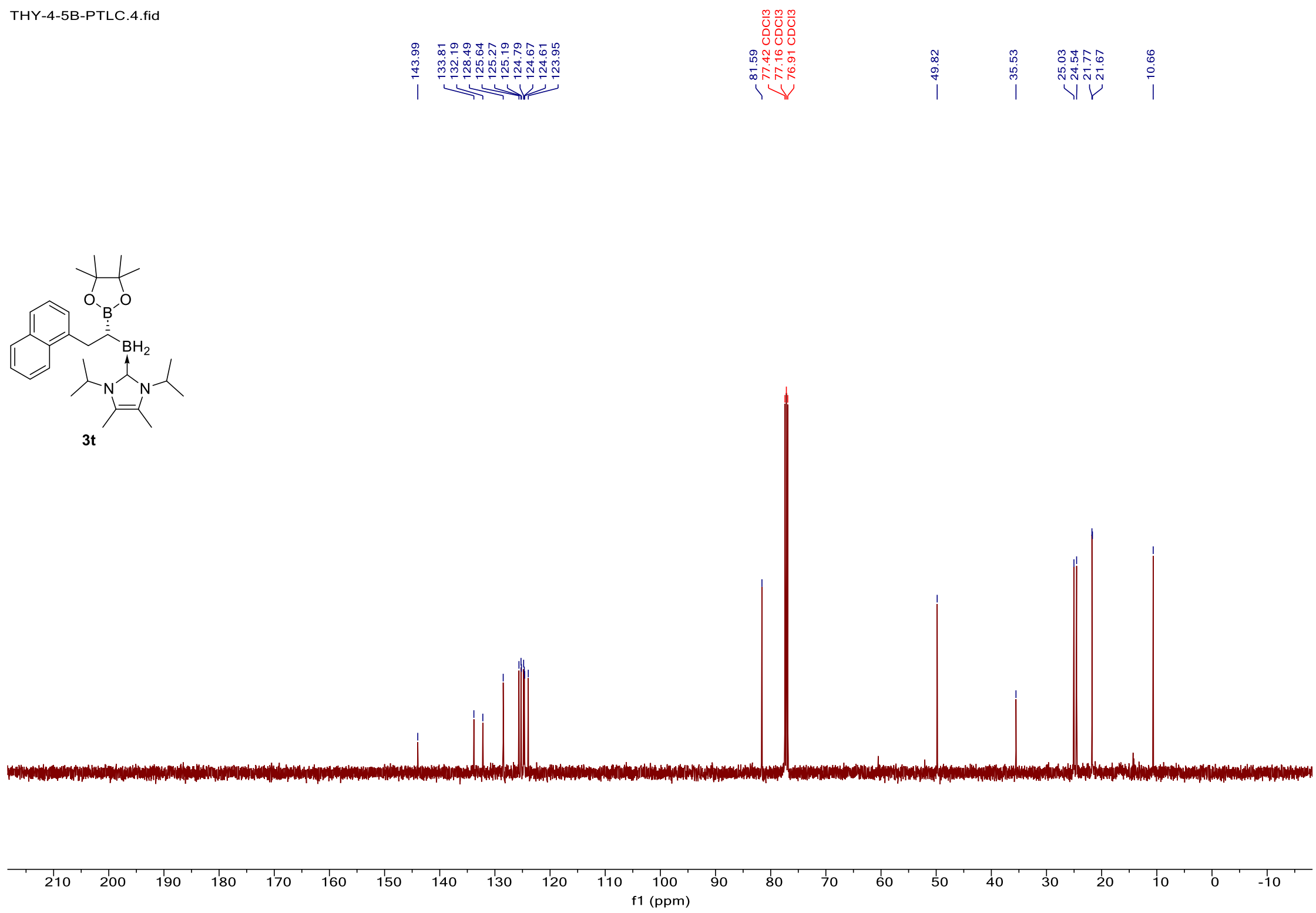

^{13}C NMR (126 MHz, $CDCl_3$)

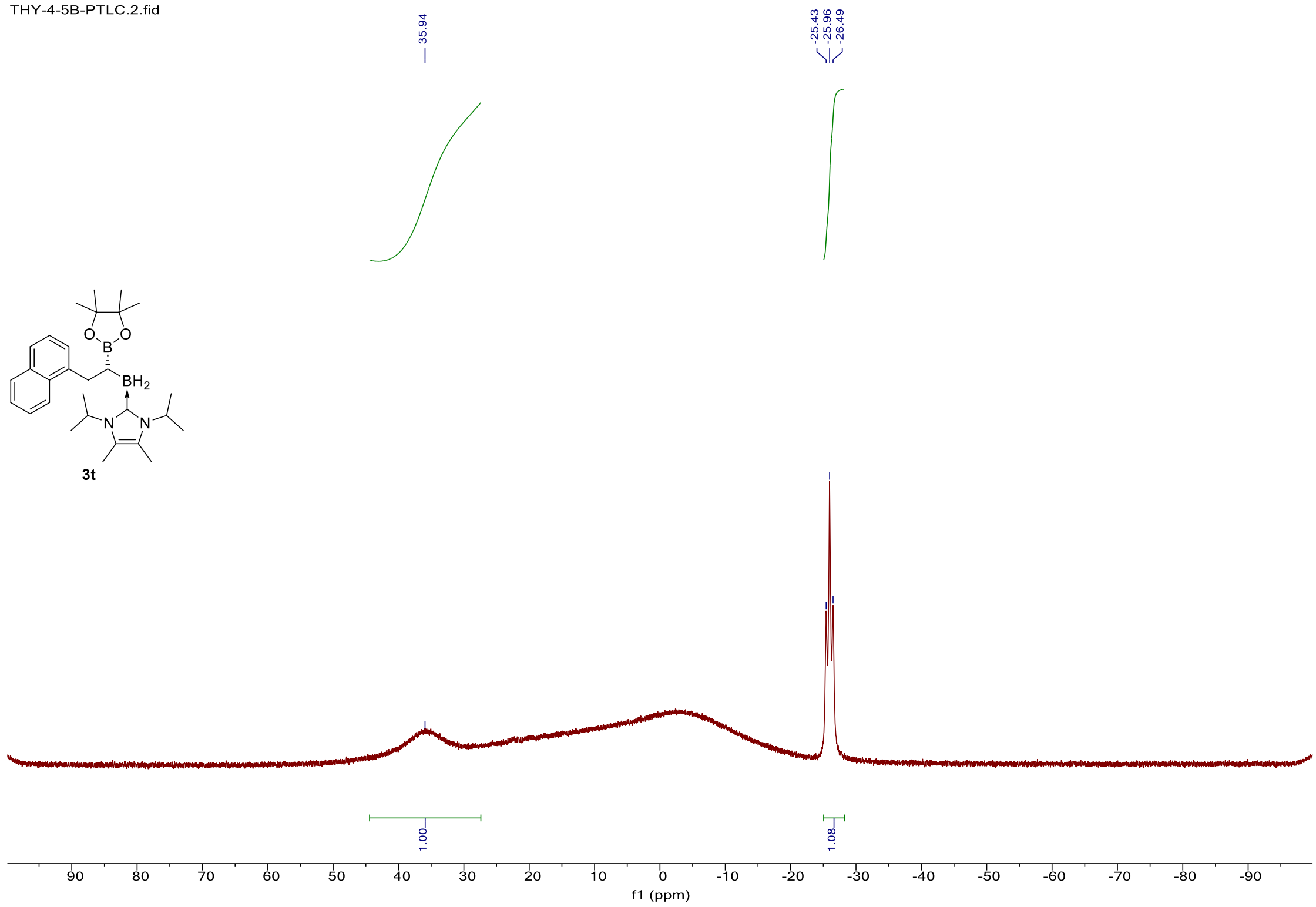

^{11}B NMR (160 MHz, $CDCl_3$)

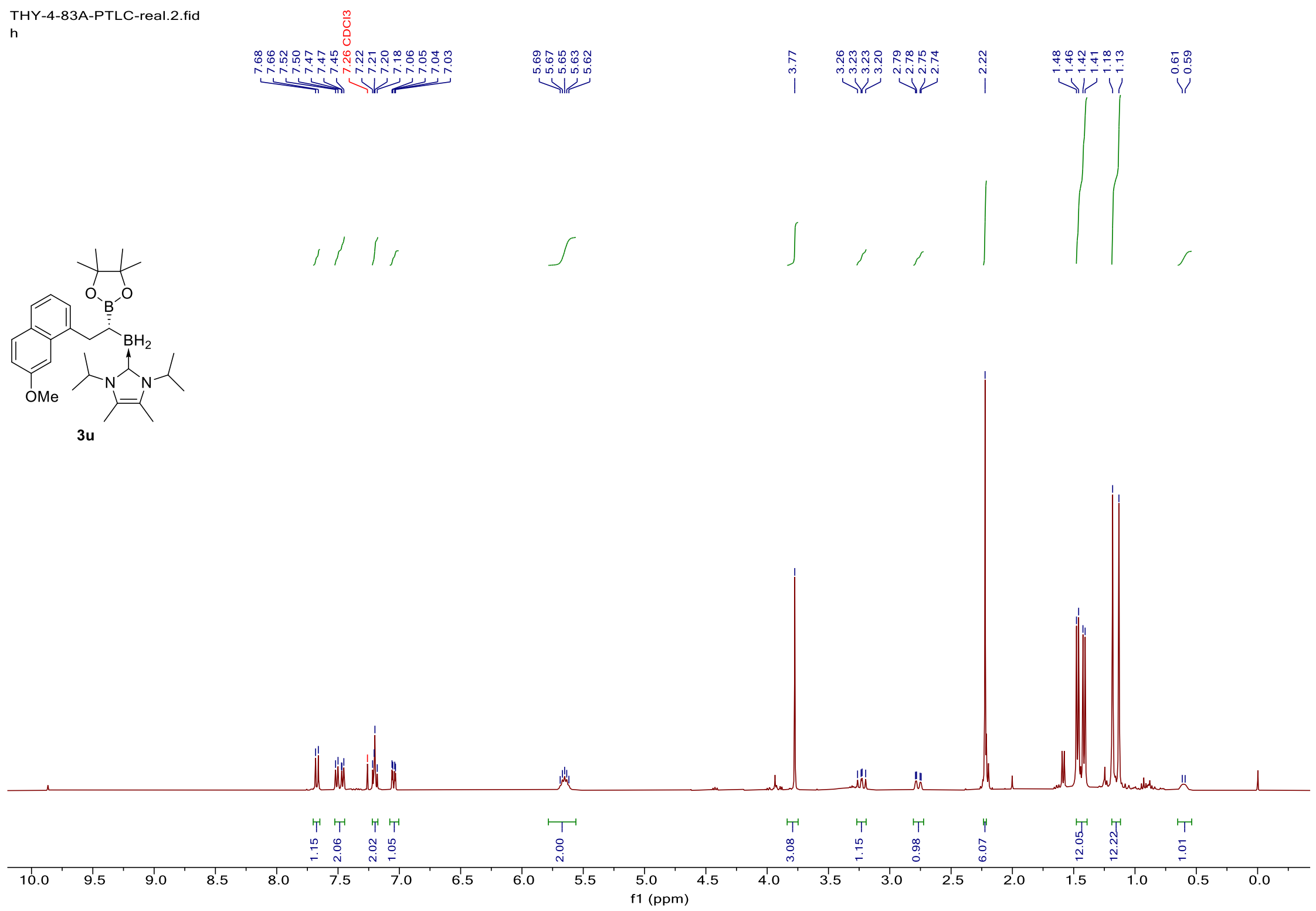

^{1}H NMR (400 MHz, $CDCl_3$)

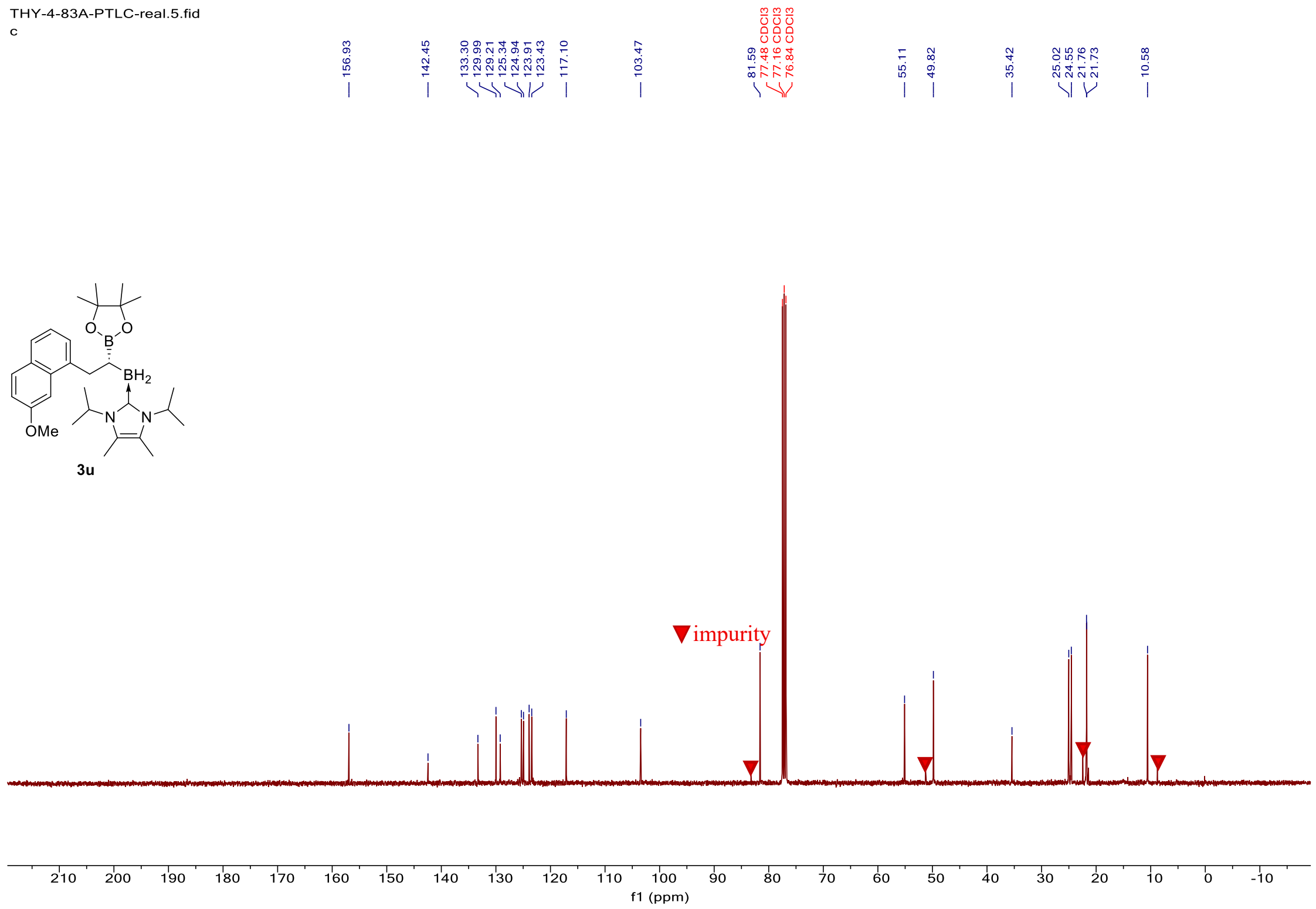

^{13}C NMR (101 MHz, $CDCl_3$)

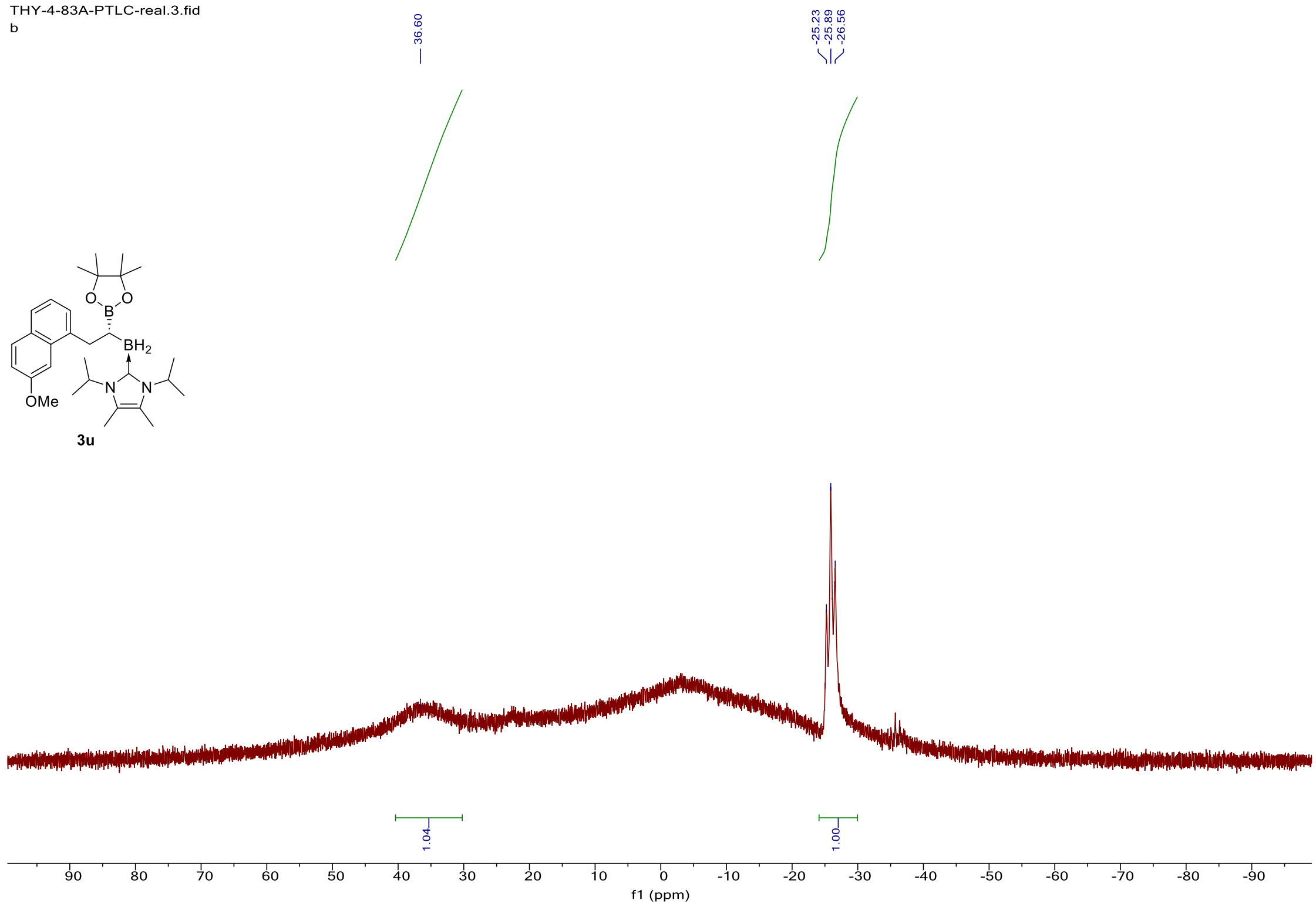

^{11}B NMR (128 MHz, $CDCl_3$)

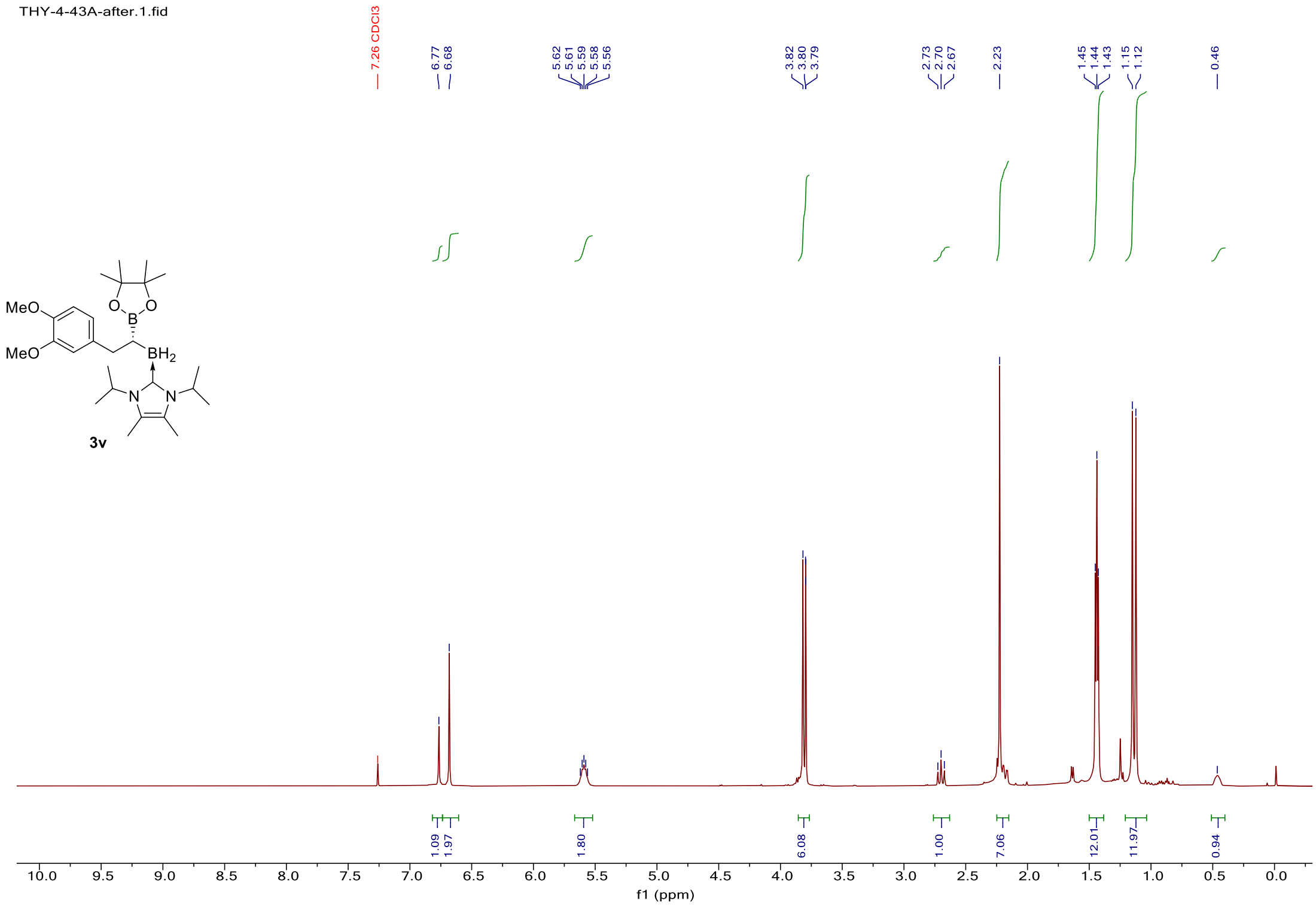

^{1}H NMR (500 MHz, $CDCl_3$)

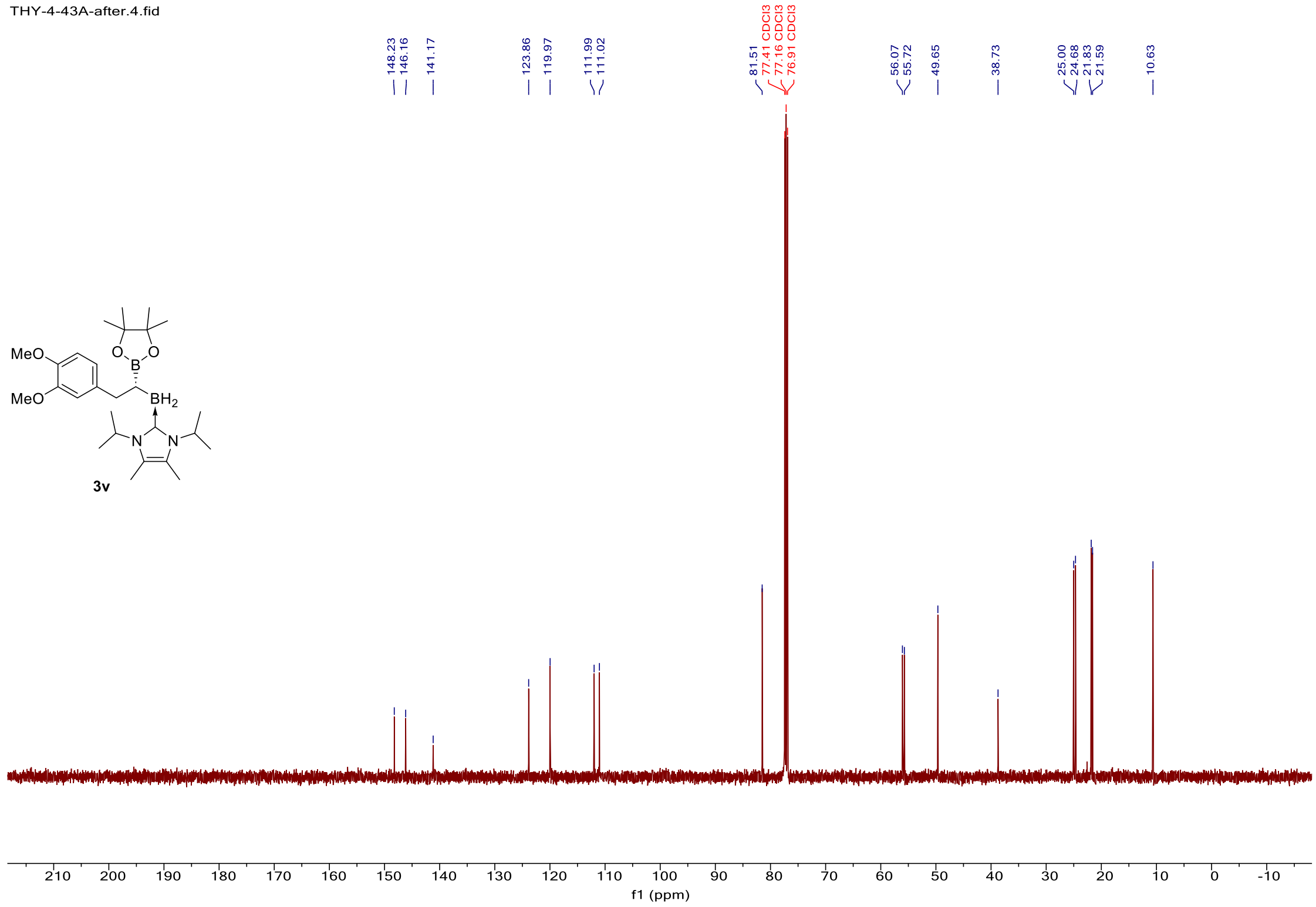

^{13}C NMR (126 MHz, $CDCl_3$)

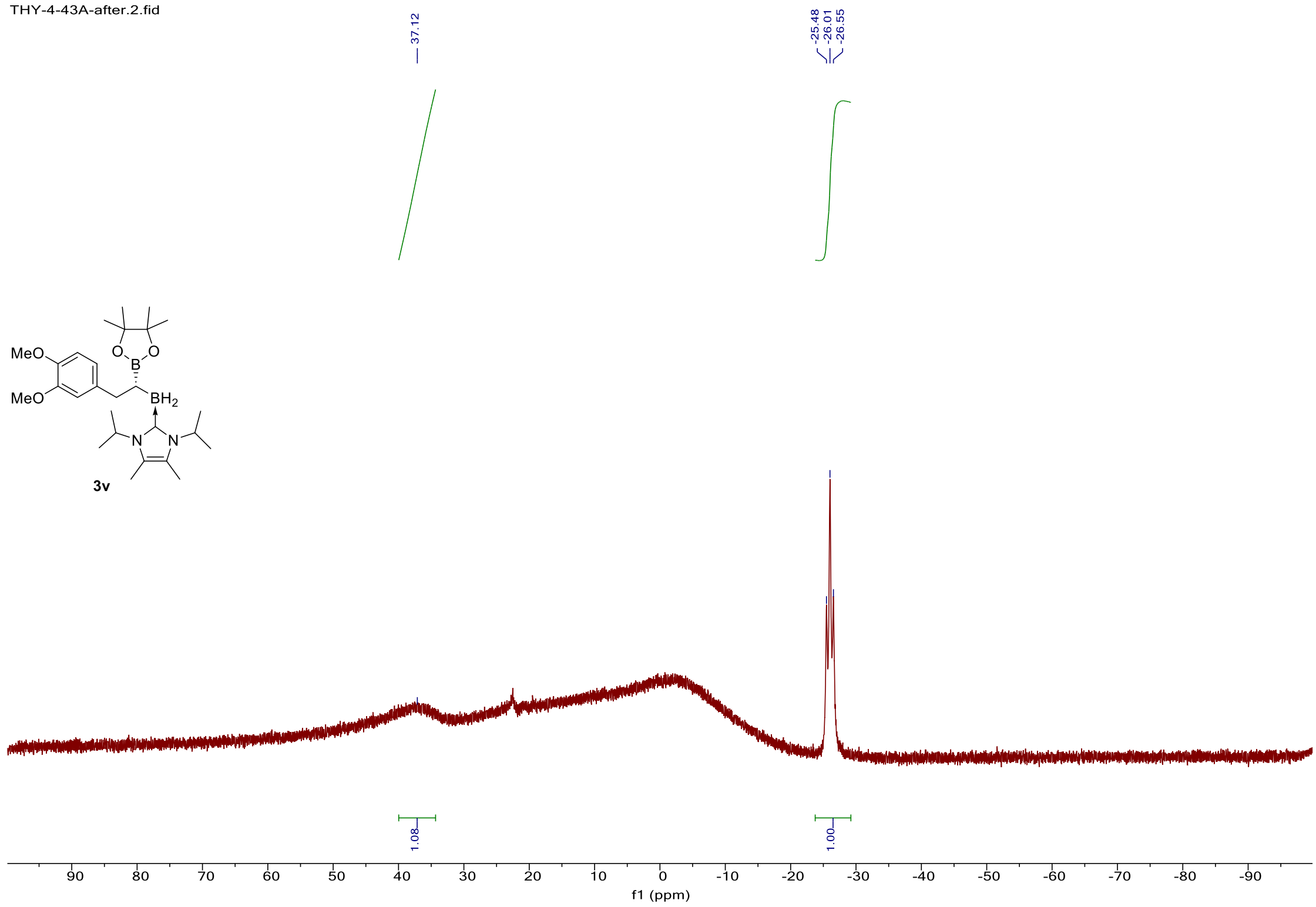

^{11}B NMR (160 MHz, $CDCl_3$)

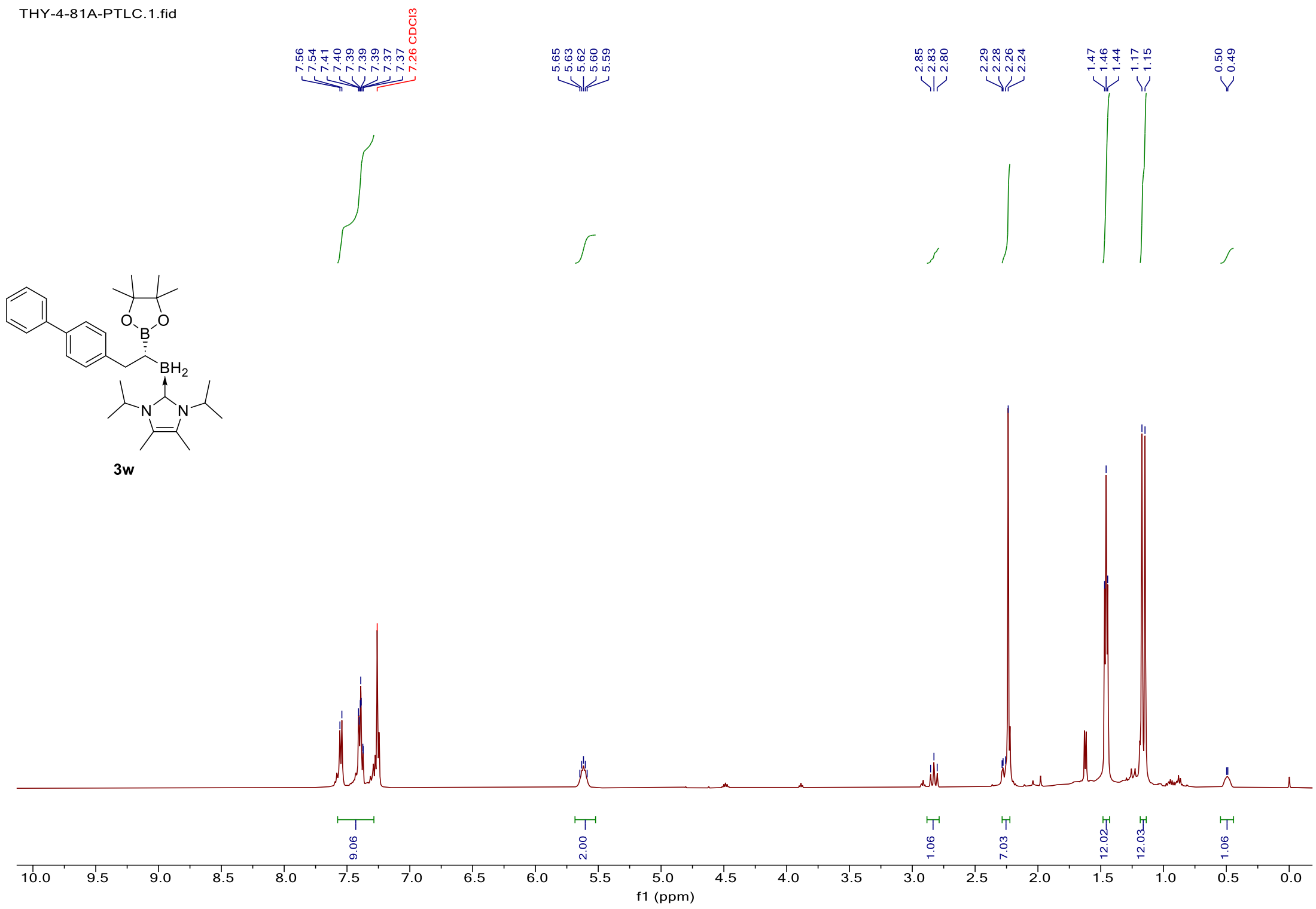

^{1}H NMR (500 MHz, CDCl$_3$)

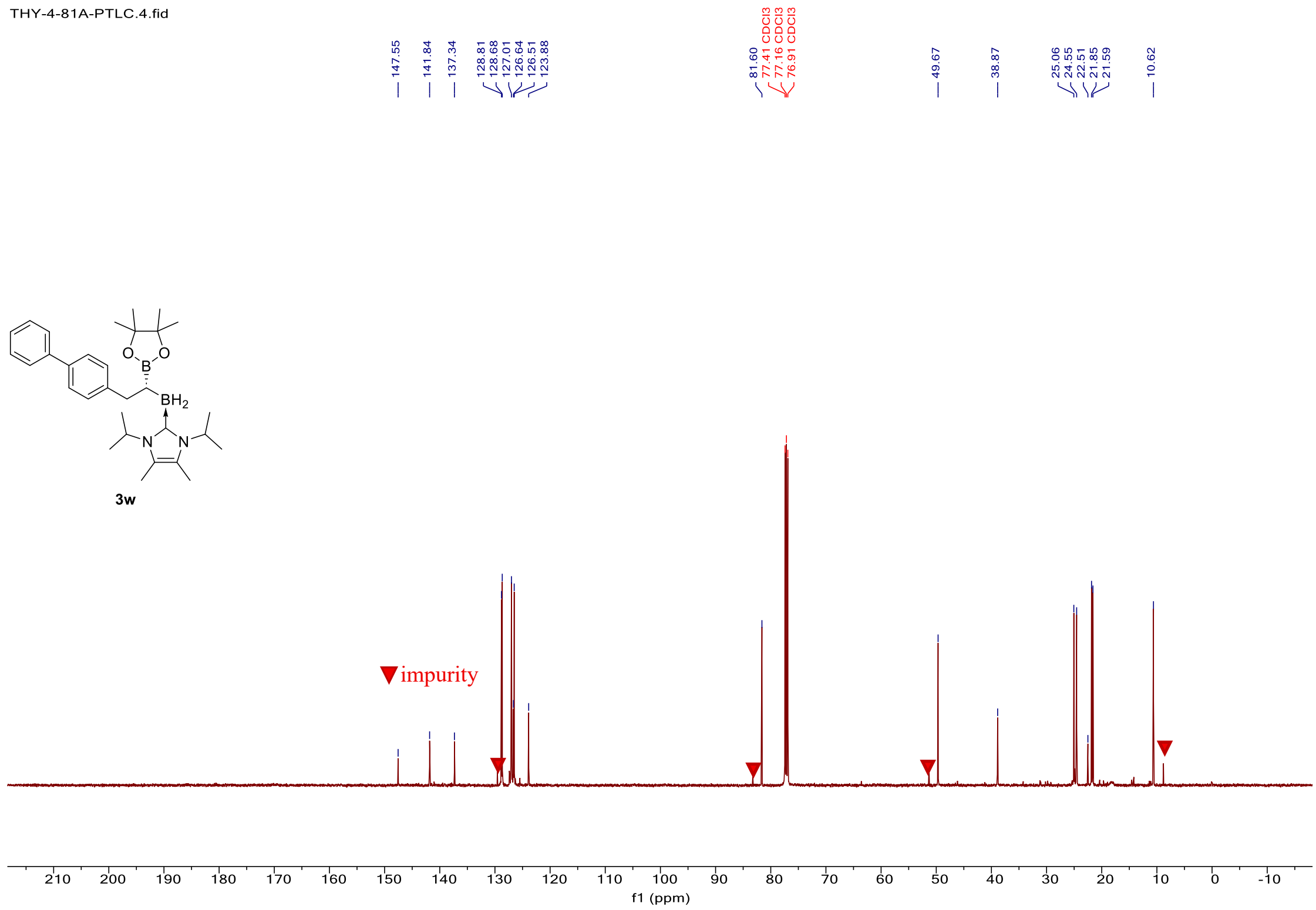

^{13}C NMR (126 MHz, CDCl$_3$)

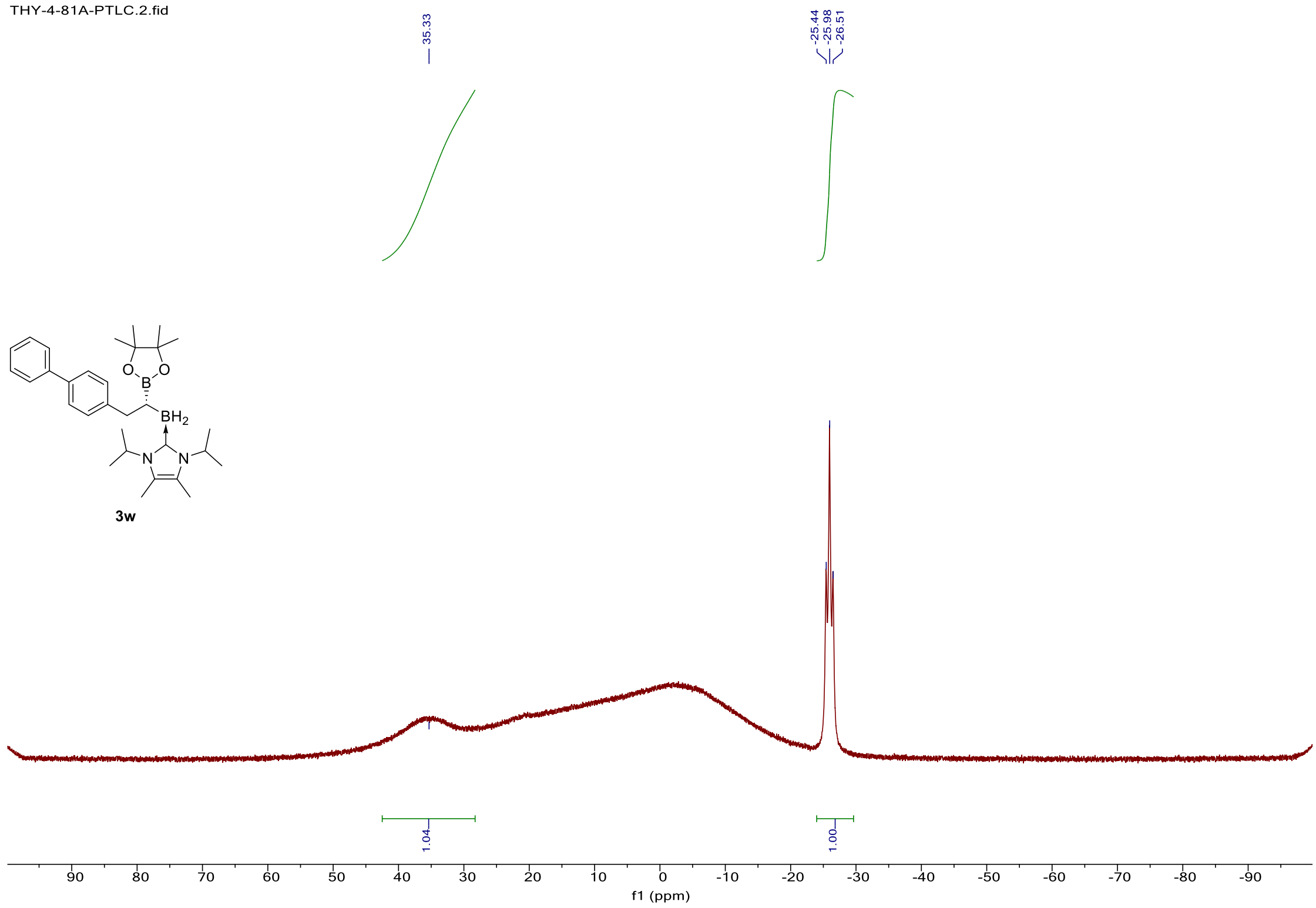

^{11}B NMR (160 MHz, $CDCl_3$)

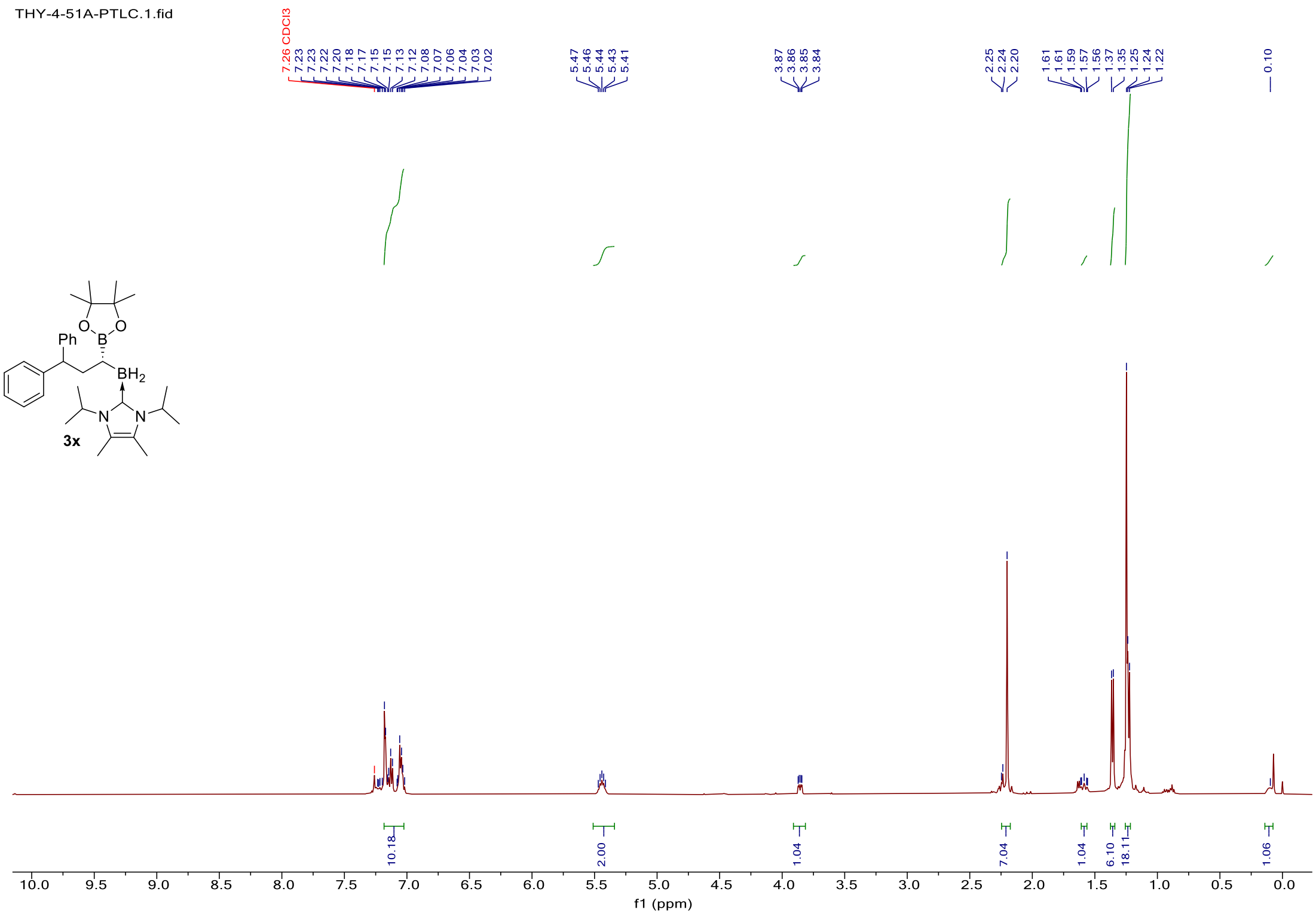

^{1}H NMR (500 MHz, $CDCl_3$)

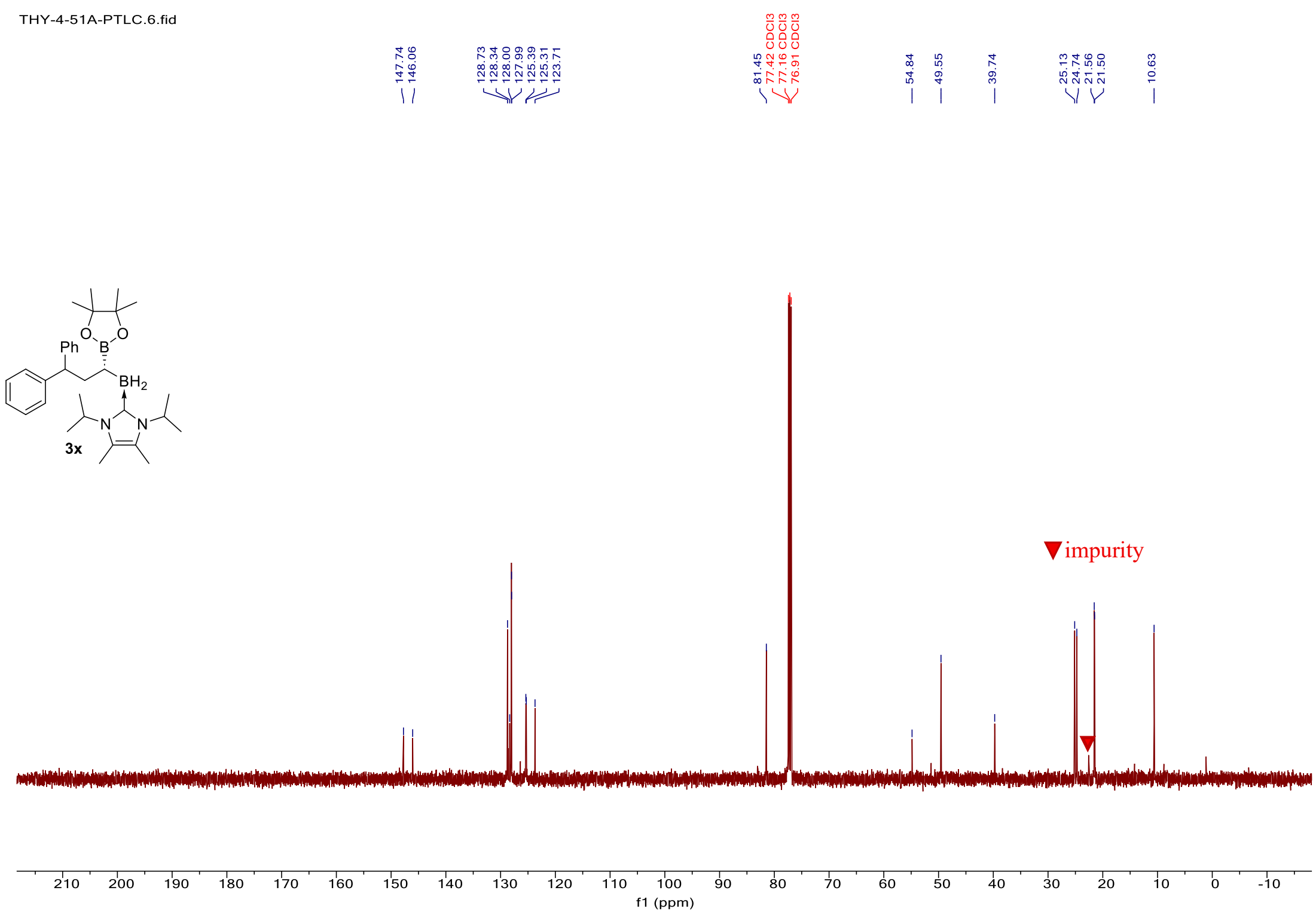

^{13}C NMR (126 MHz, $CDCl_3$)

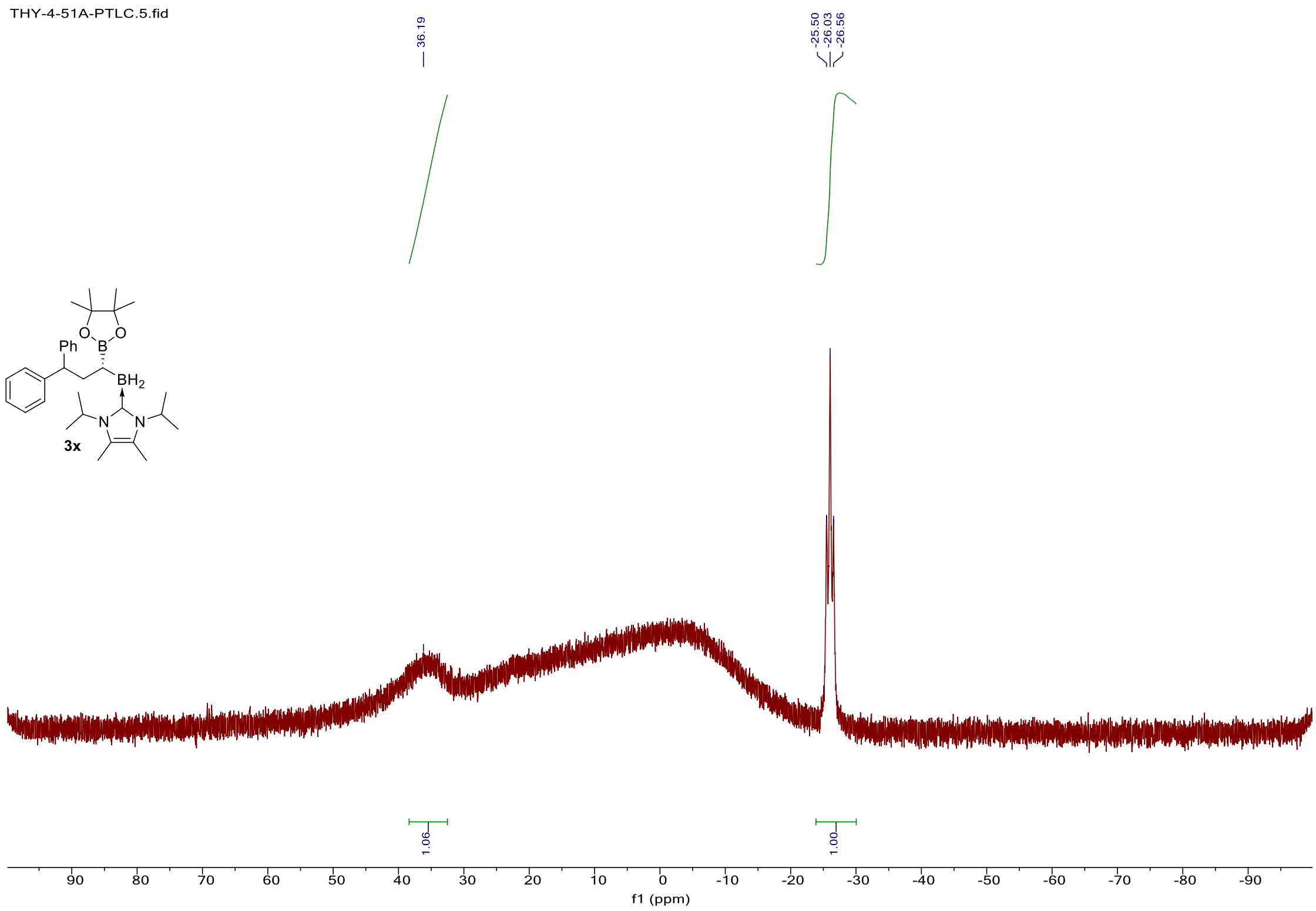

^{11}B NMR (160 MHz, $CDCl_3$)

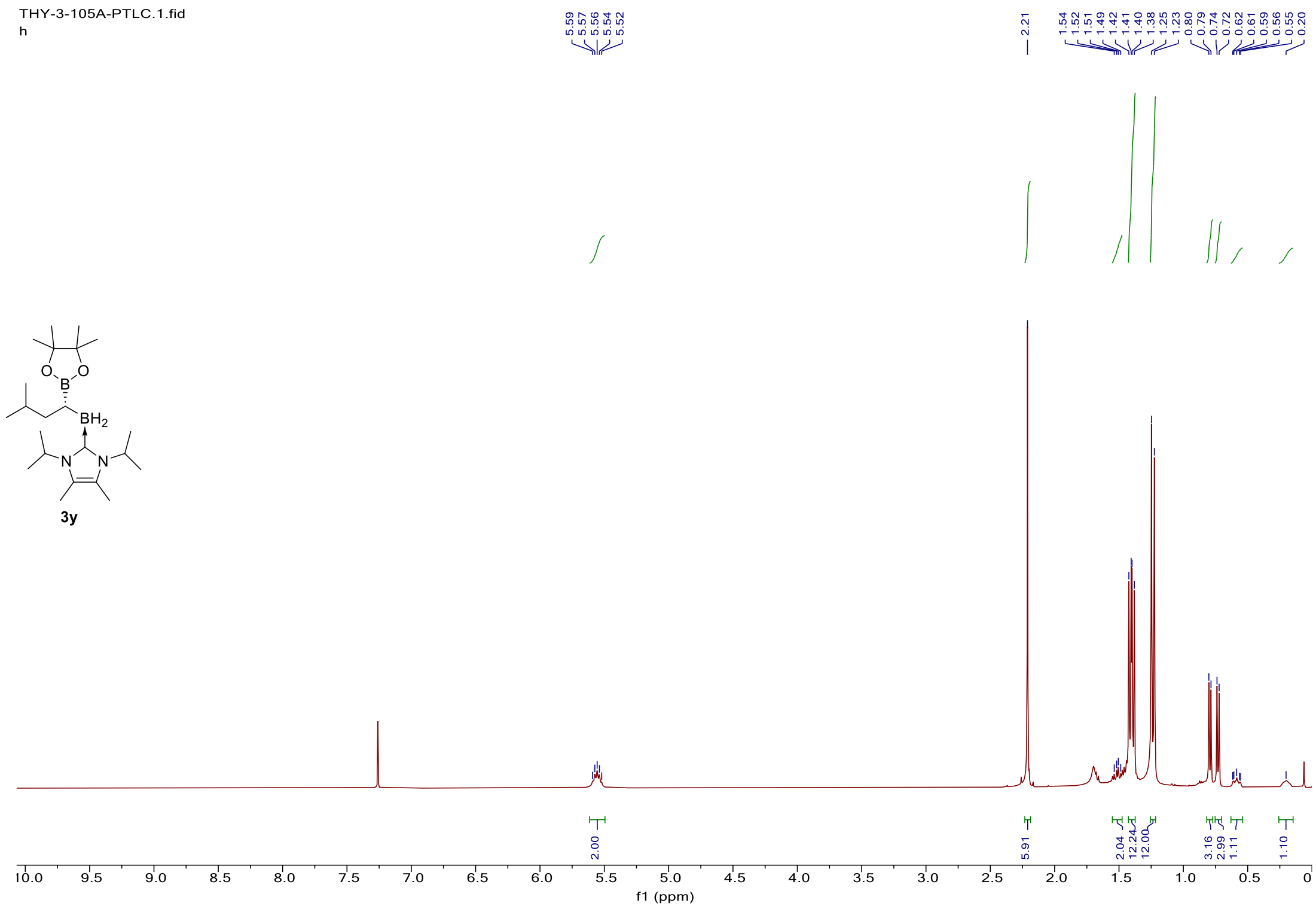

^1H NMR (400 MHz, $CDCl_3$)

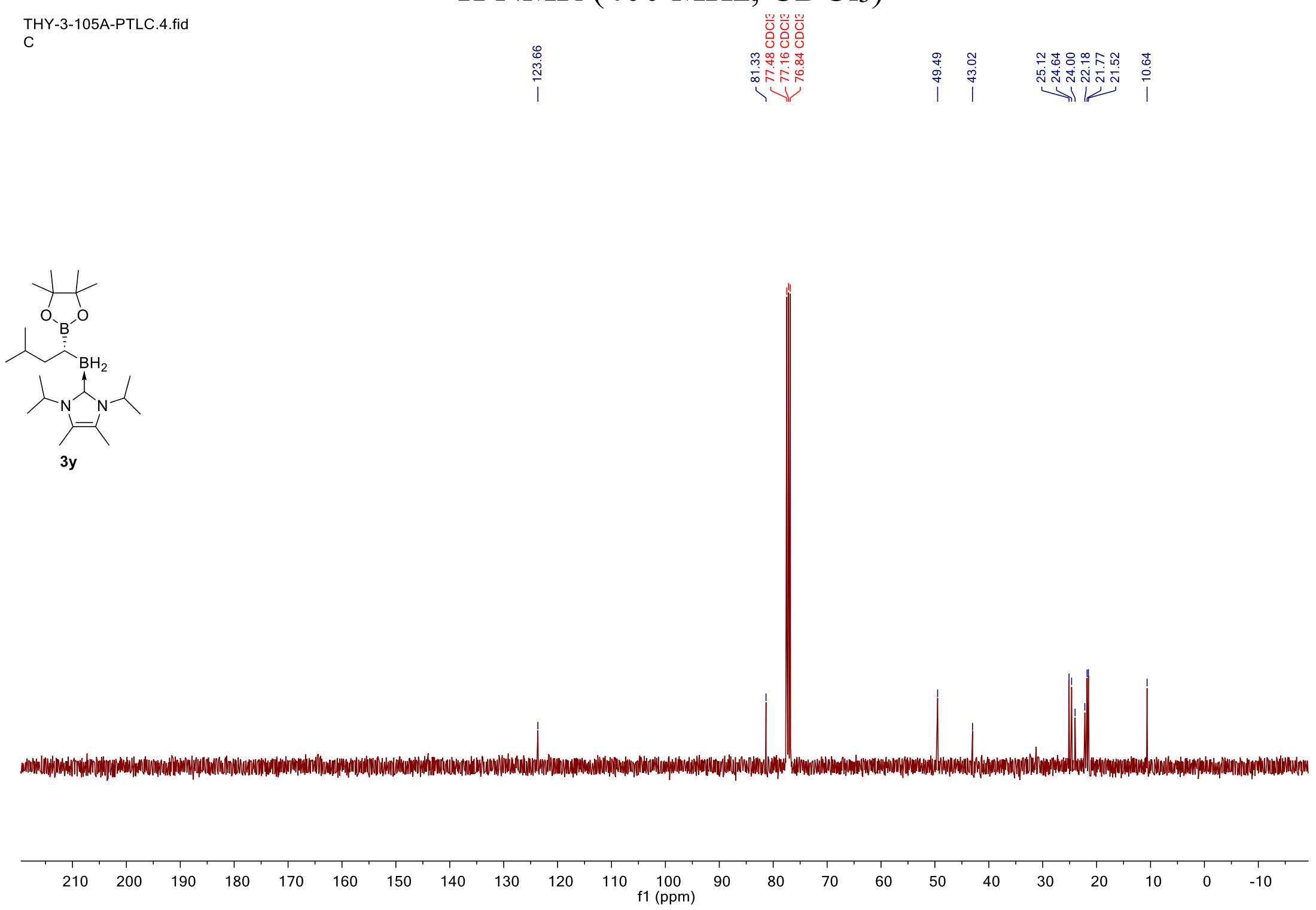

^{13}C NMR (101 MHz, $CDCl_3$)

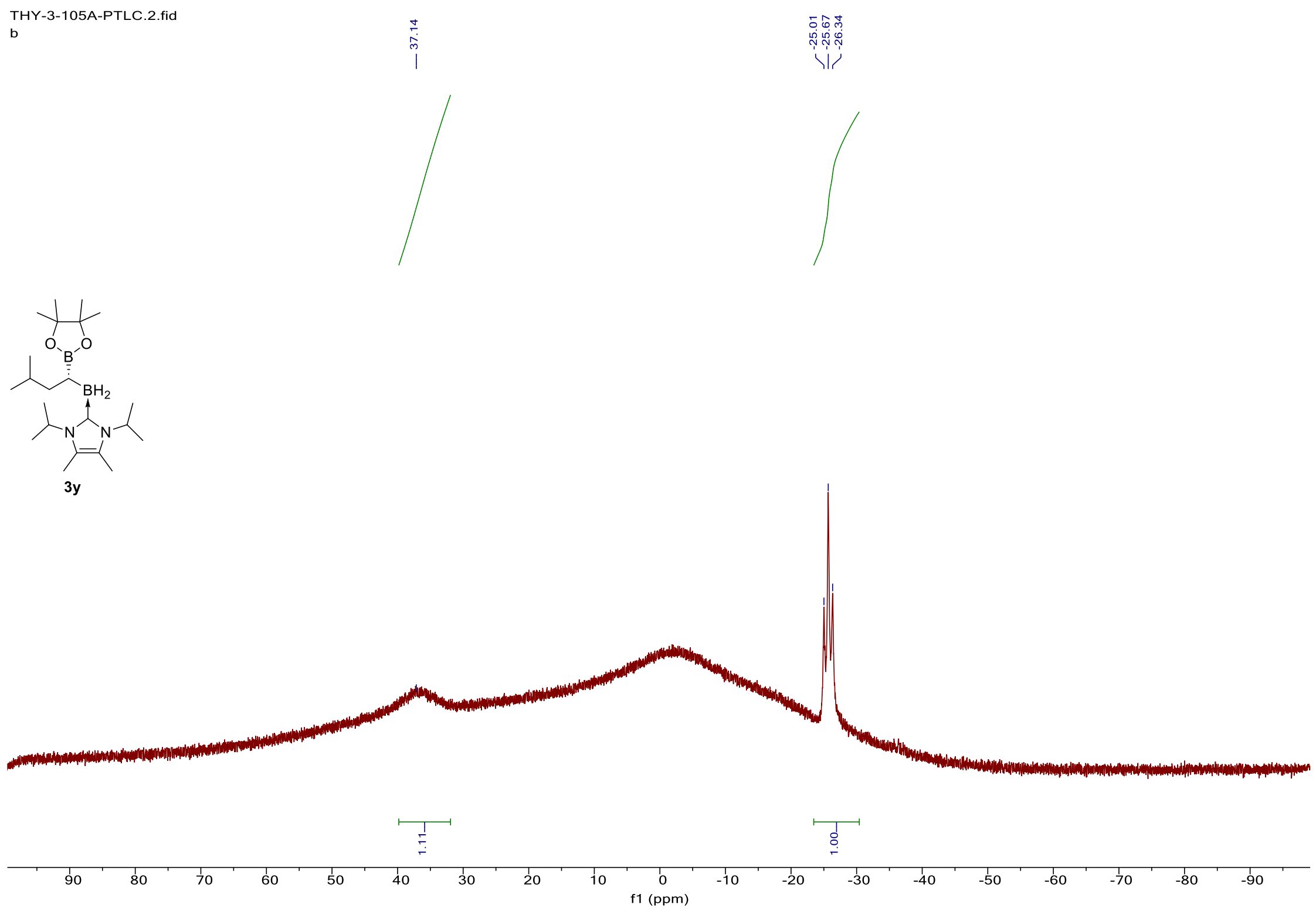

^{11}B NMR (128 MHz, $CDCl_3$)

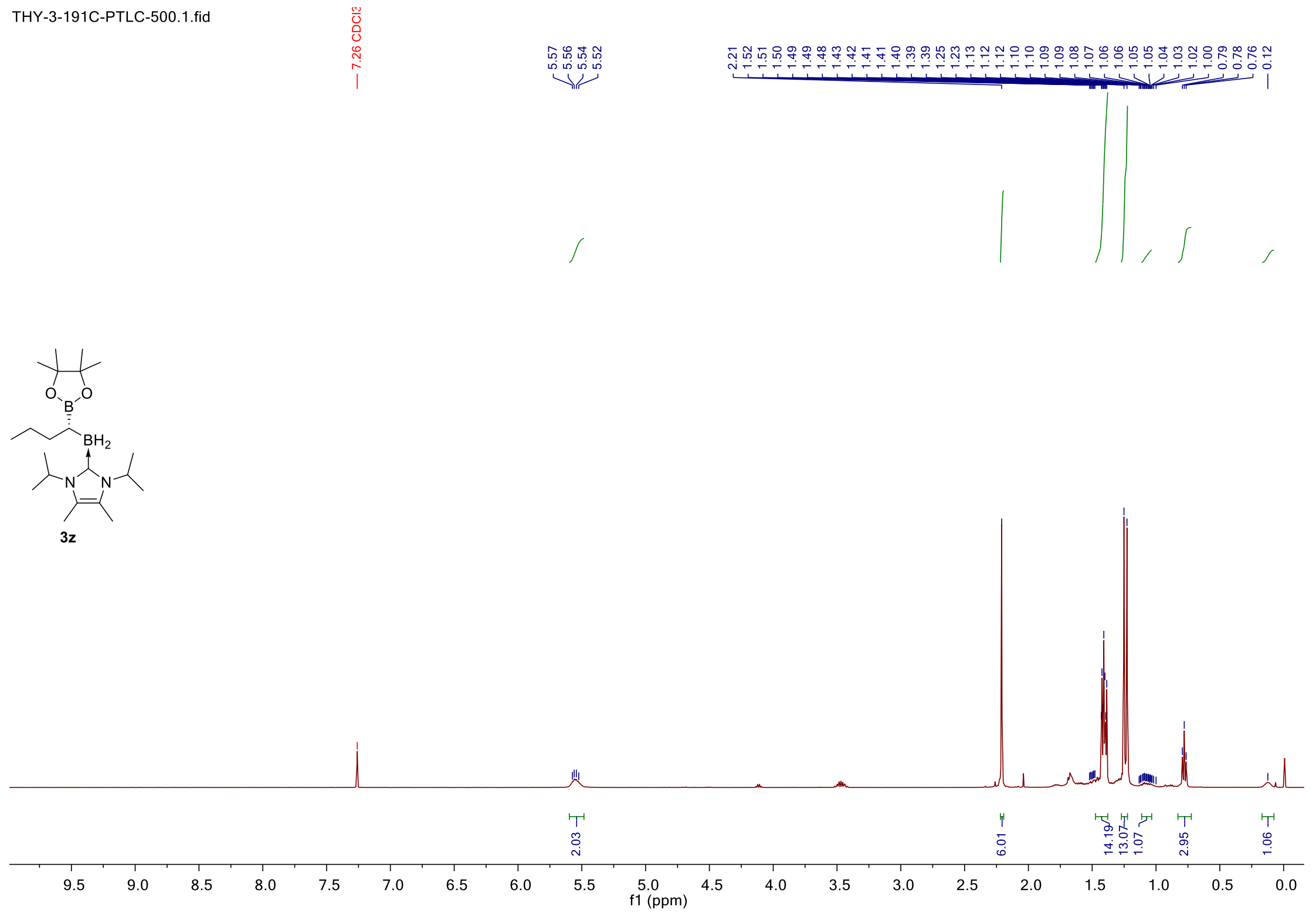

^{1}H NMR (500 MHz, $CDCl_3$)

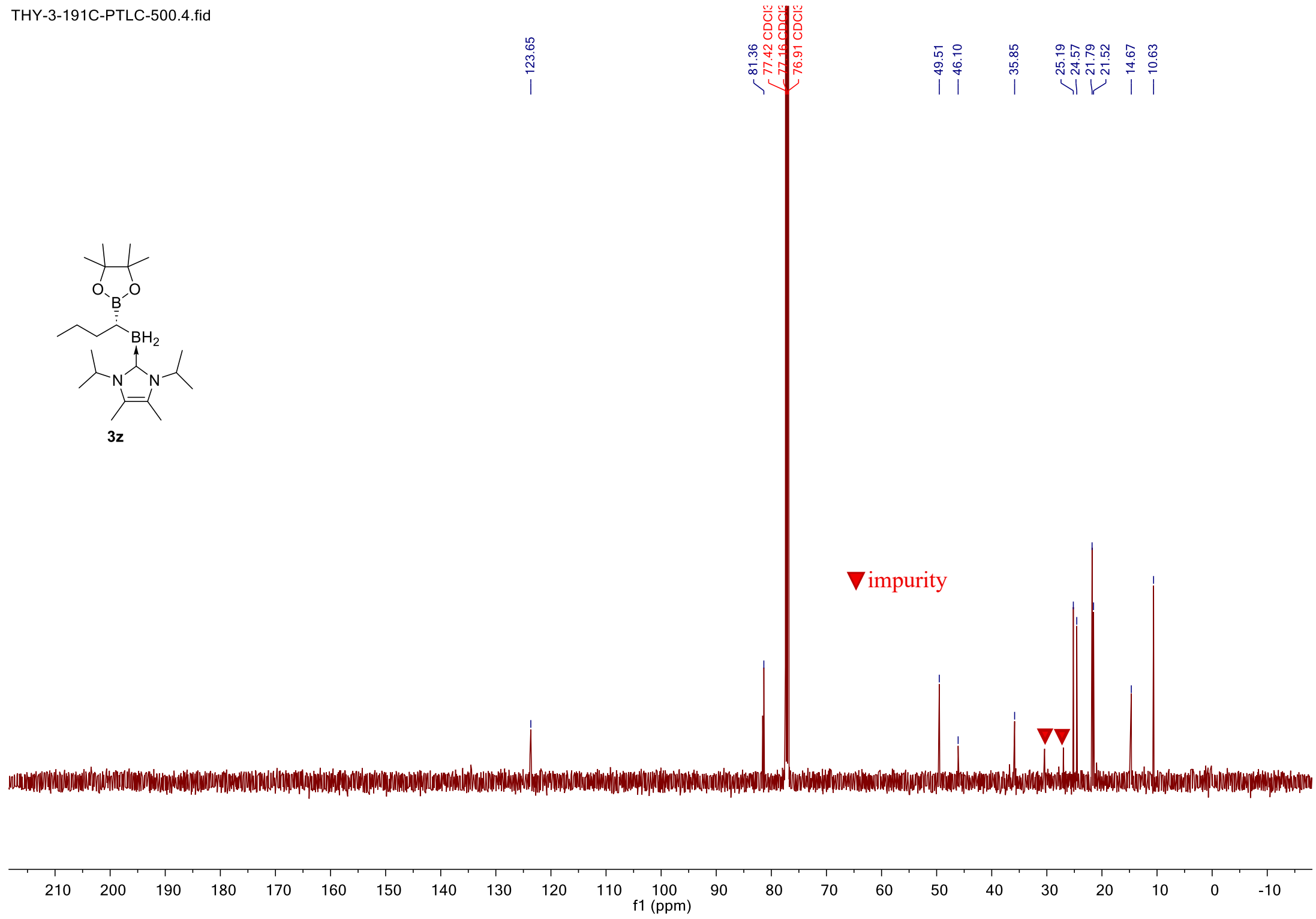

^{13}C NMR (126 MHz, $CDCl_3$)

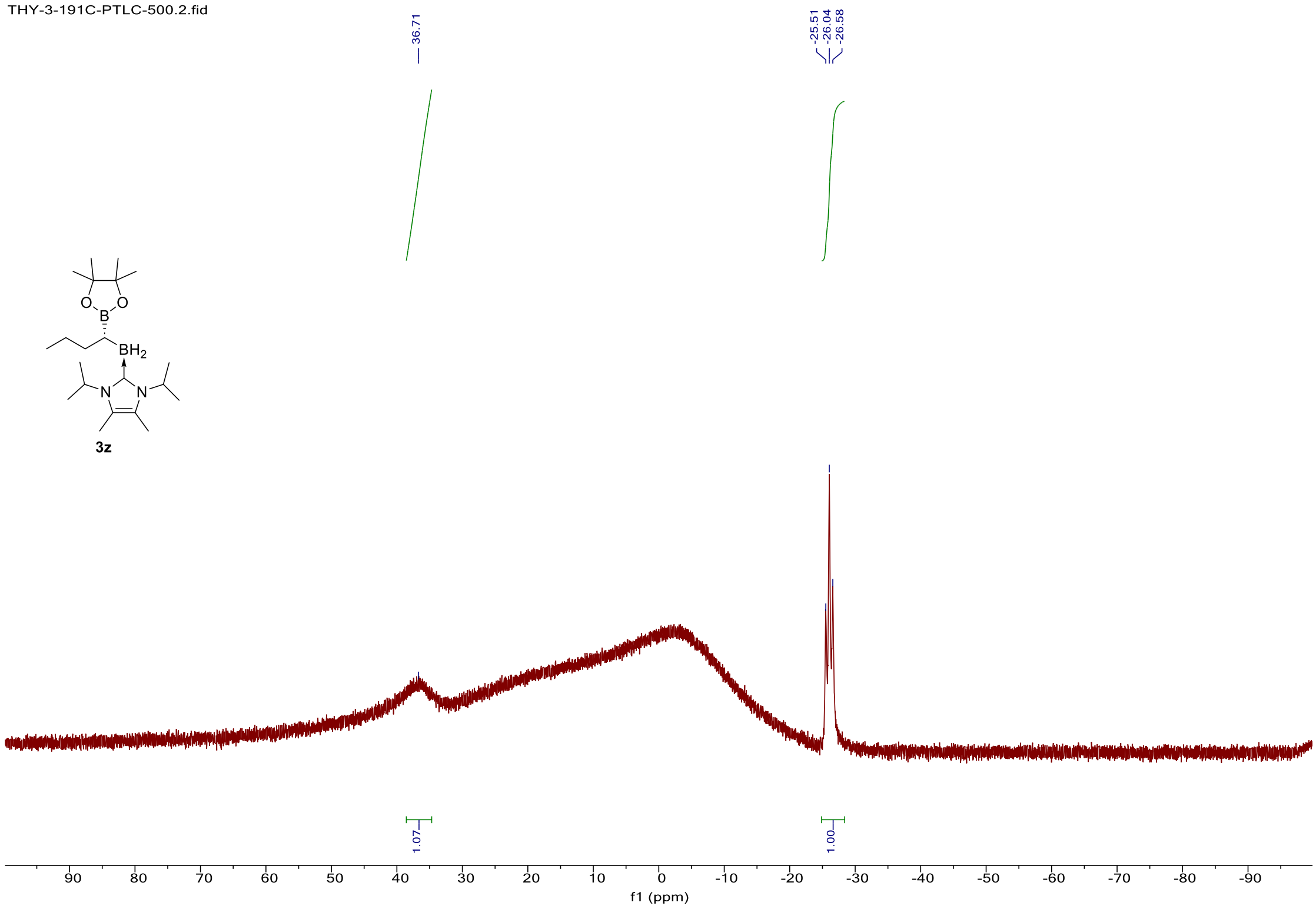

^{11}B NMR (160 MHz, $CDCl_3$)

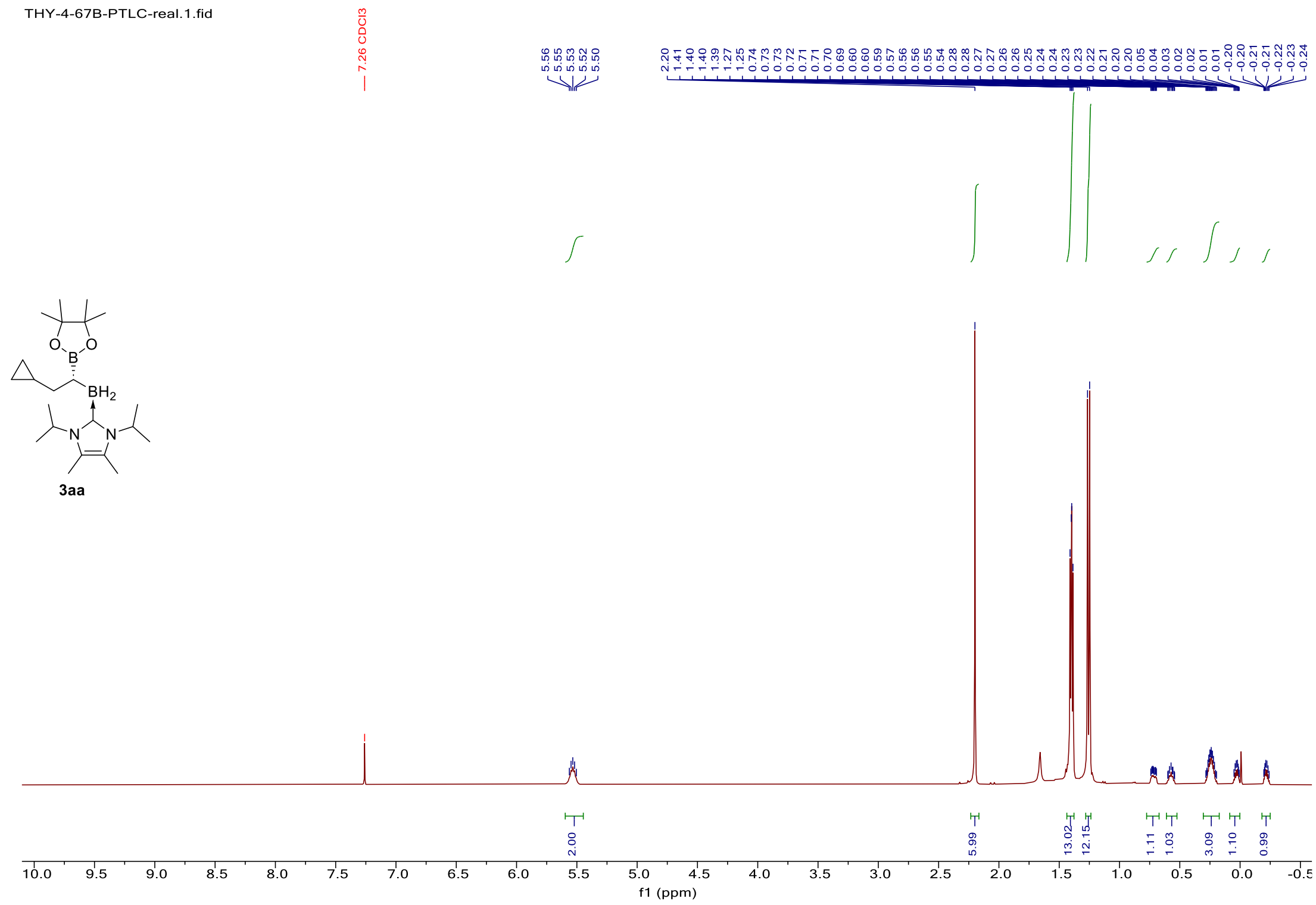

^{1}H NMR (500 MHz, CDCl3)

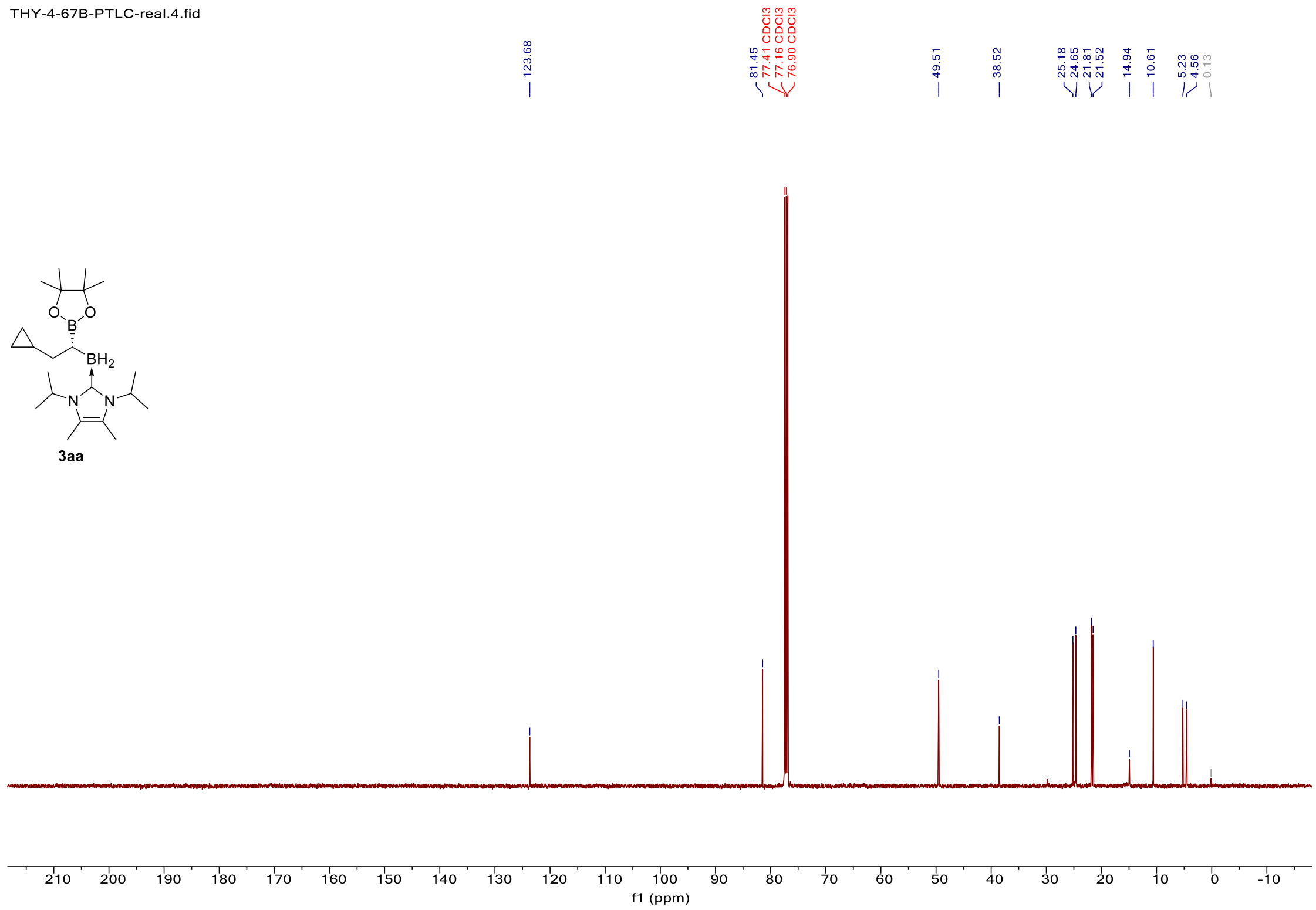

^{13}C NMR (126 MHz, CDCl3)

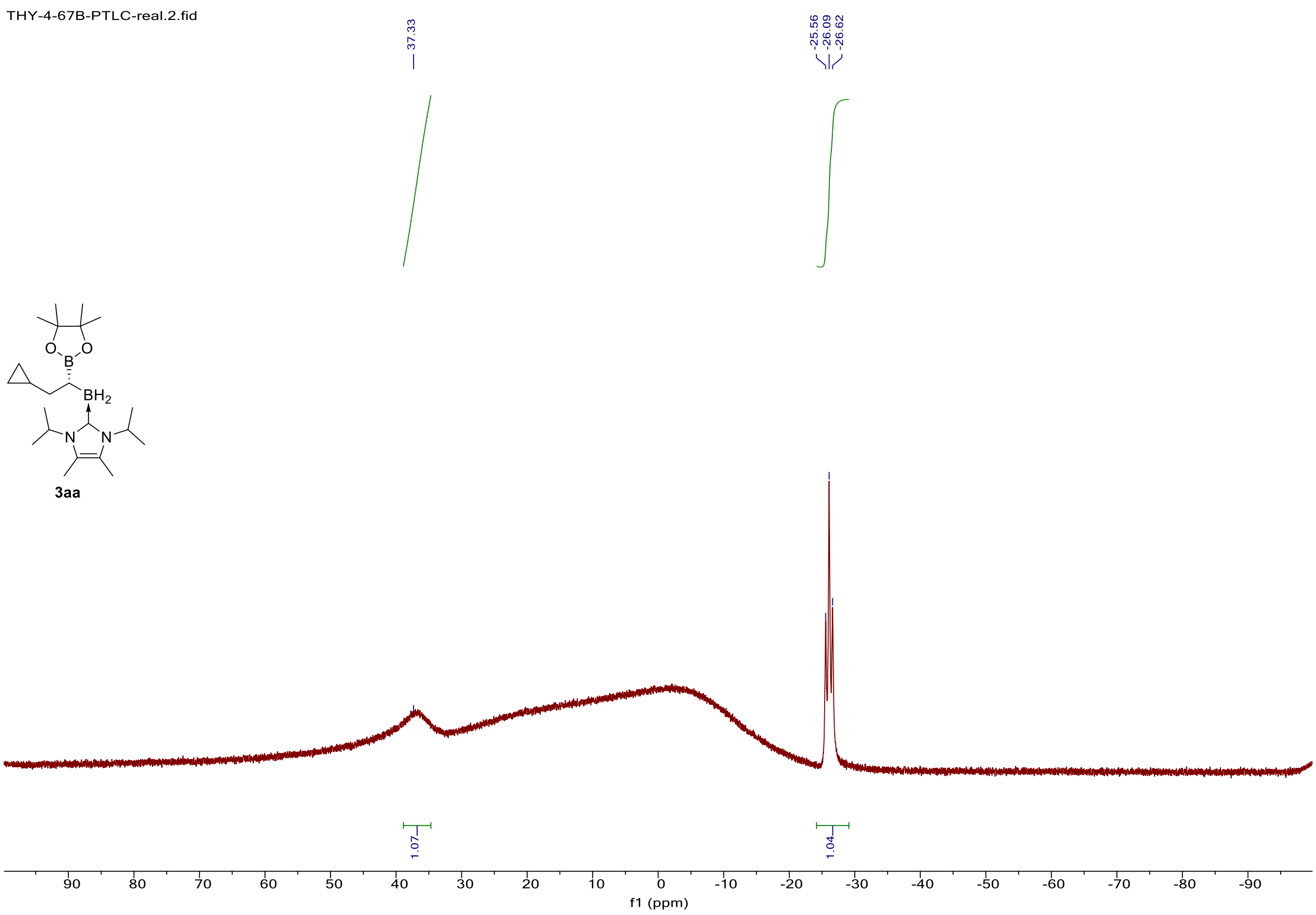

^{11}B NMR (160 MHz, $CDCl_3$)

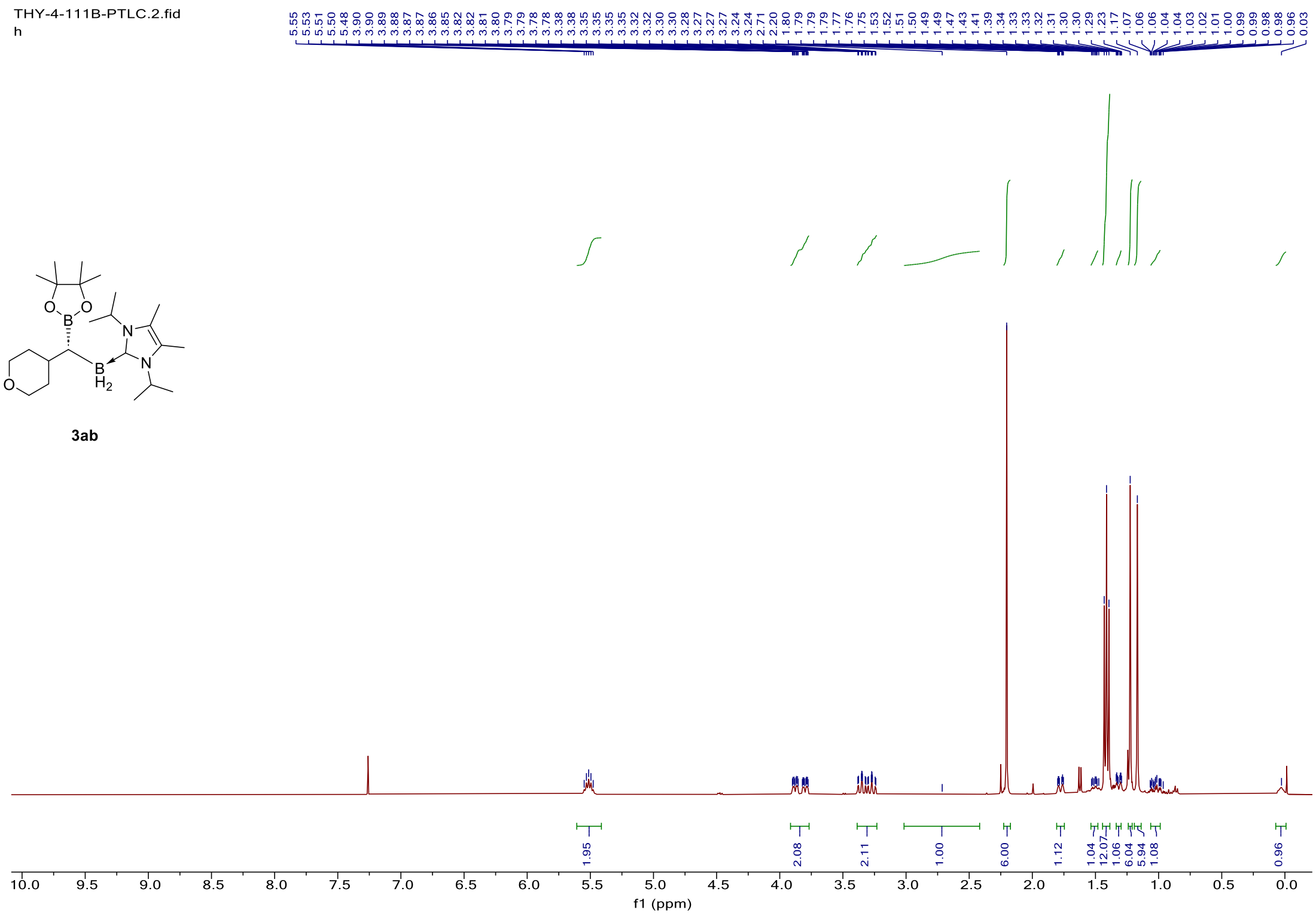

^{1}H NMR (400 MHz, $CDCl_3$)

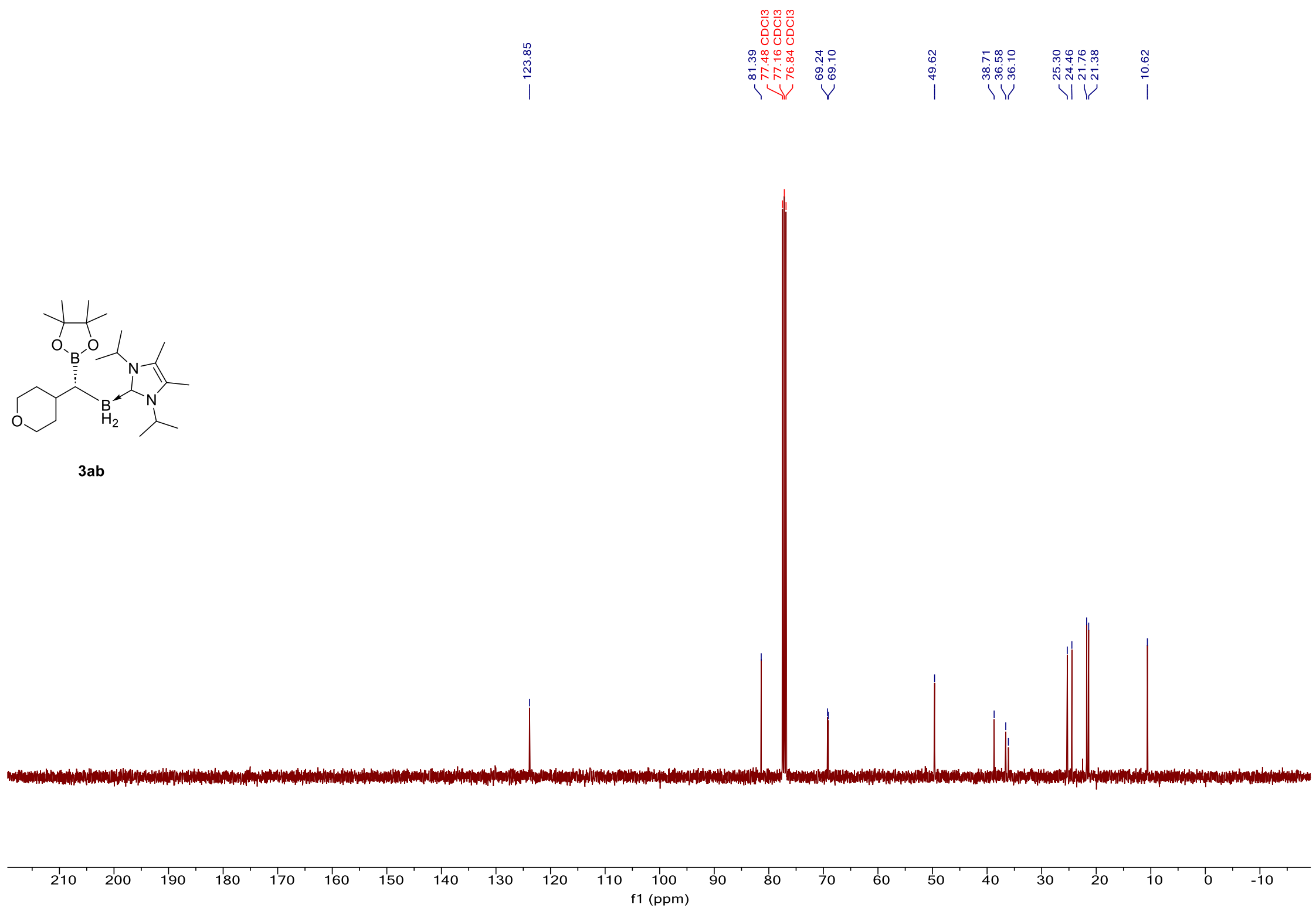

^{13}C NMR (101 MHz, $CDCl_3$)

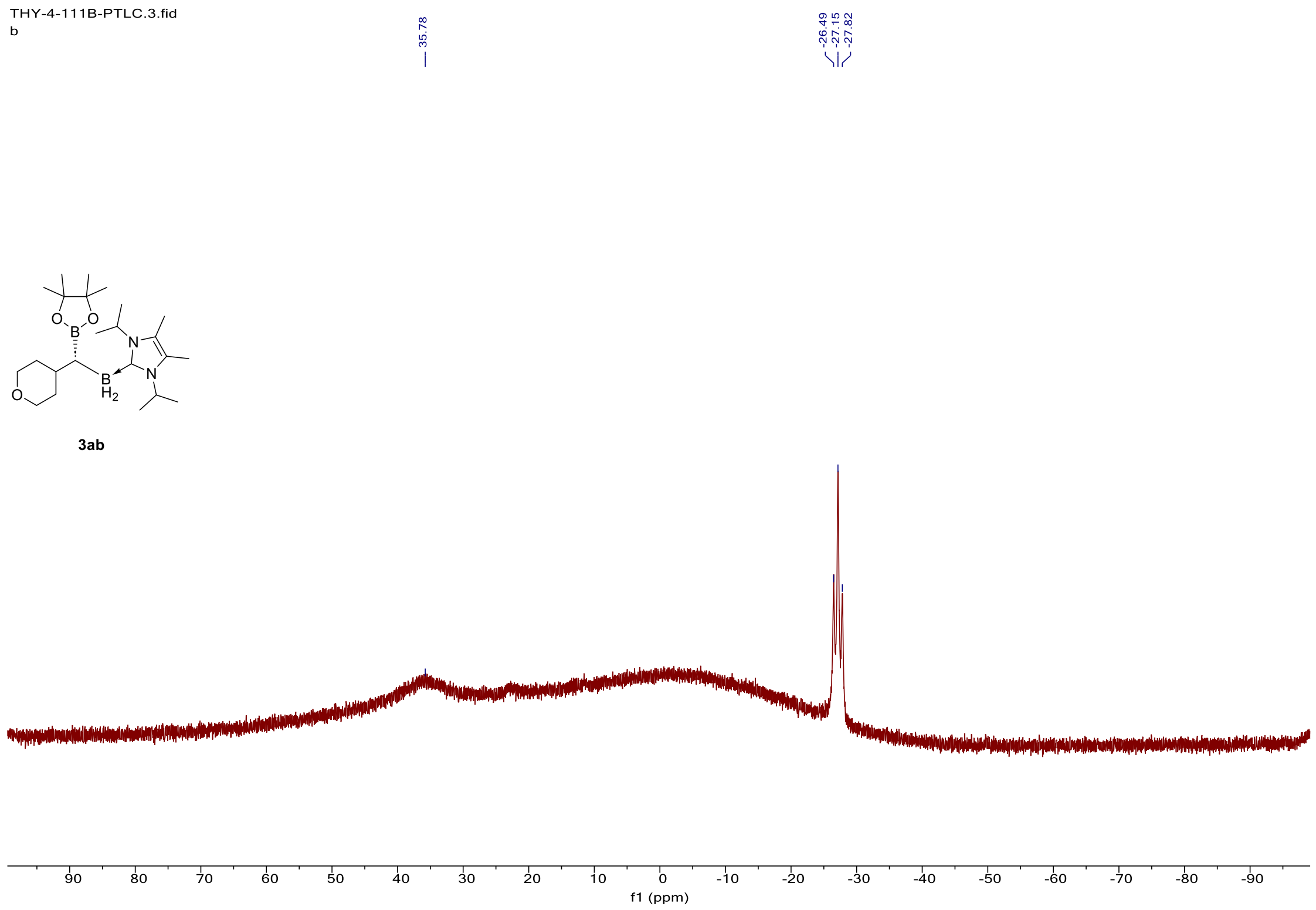

^{11}B NMR (128 MHz, $CDCl_3$)

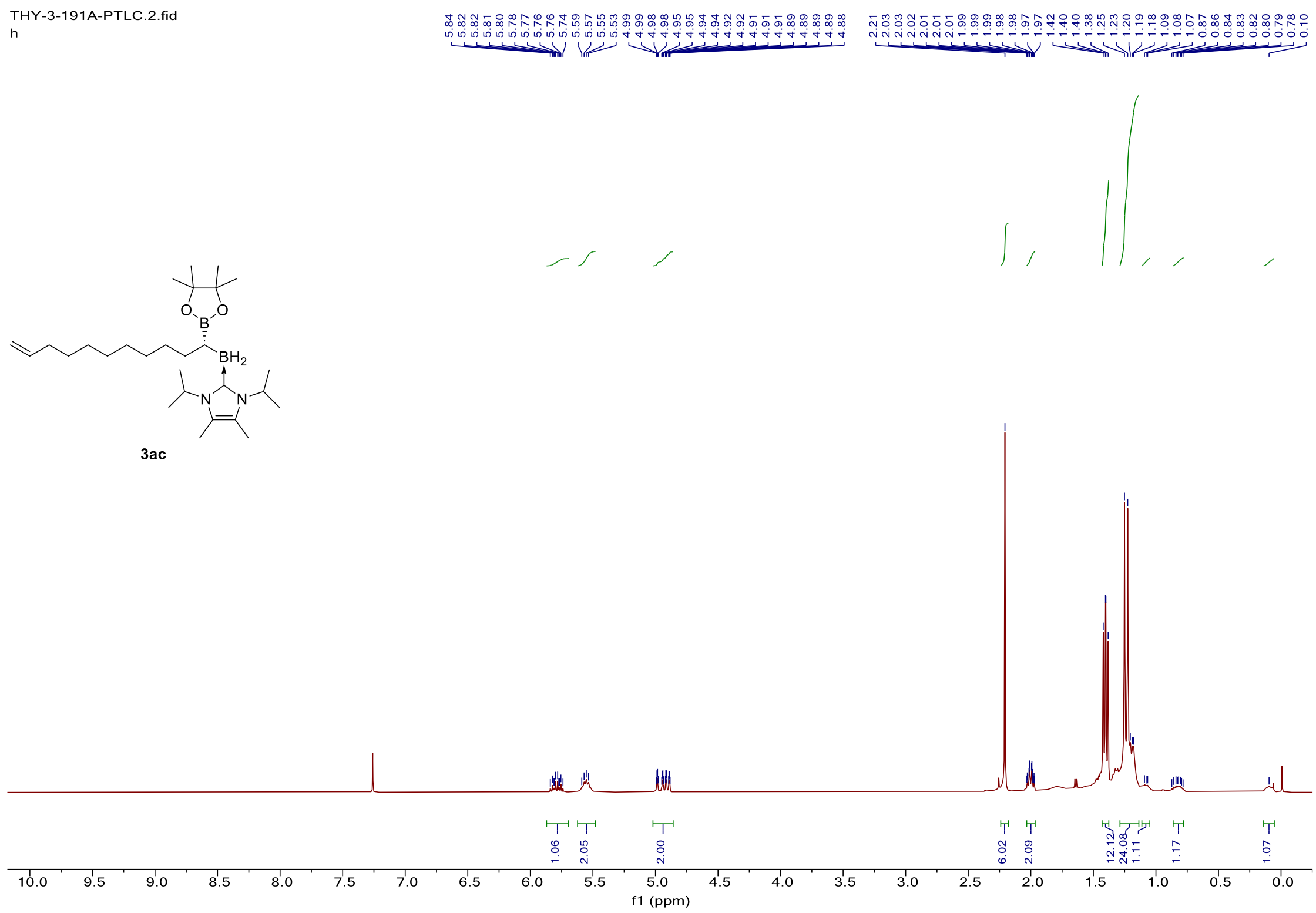

¹H NMR (400 MHz, $CDCl_3$)

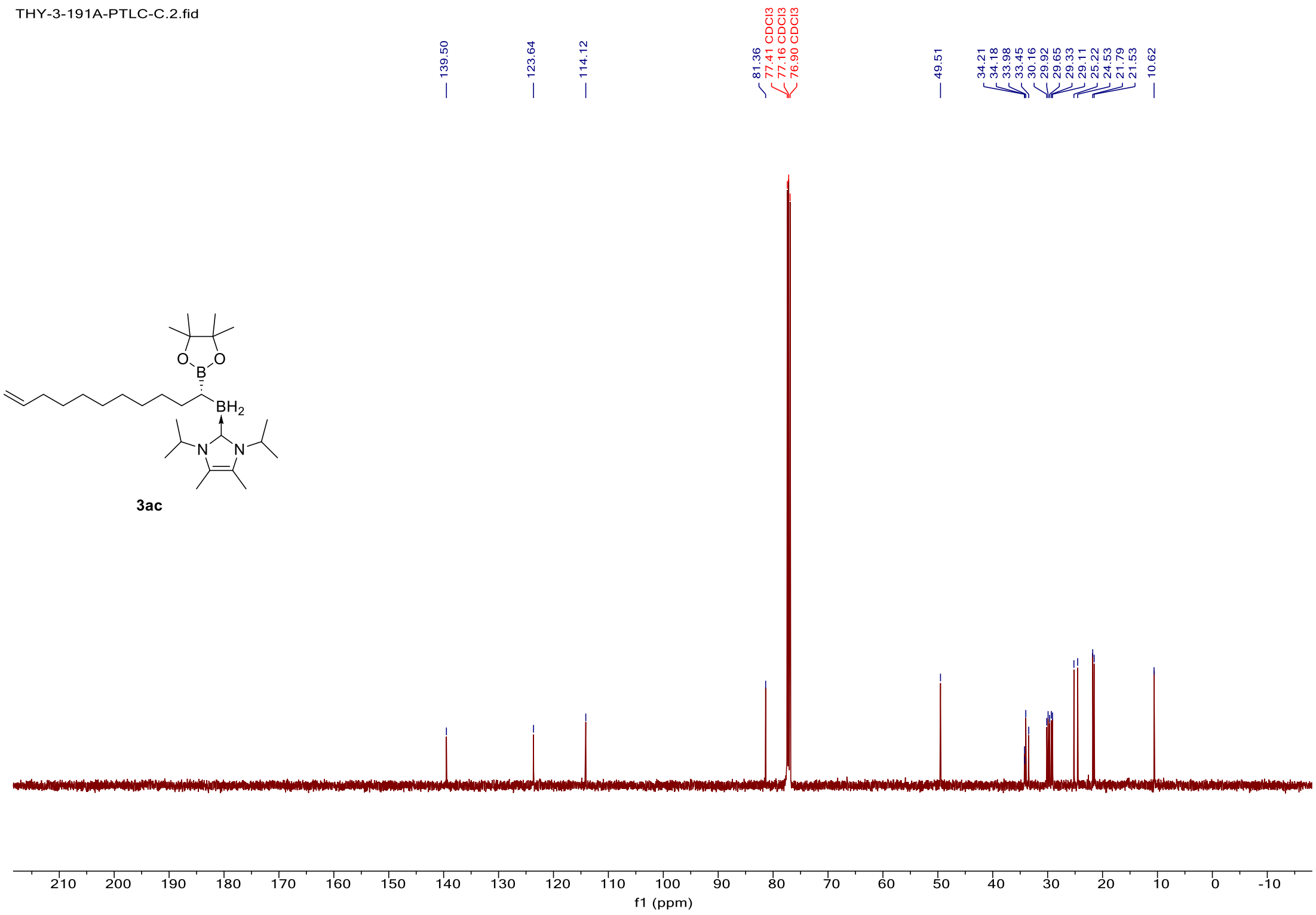

¹³C NMR (126 MHz, $CDCl_3$)

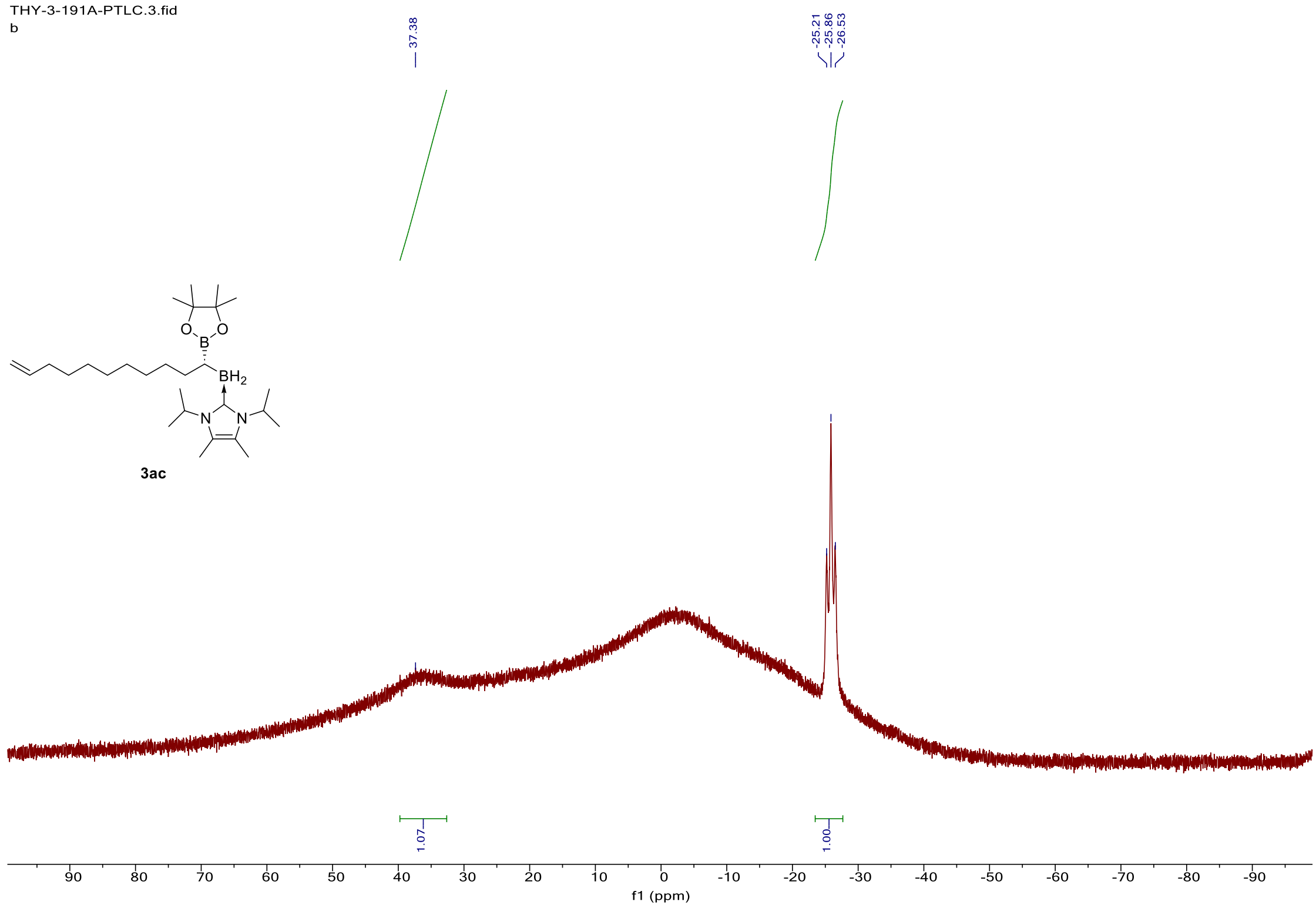

^{11}B NMR (128 MHz, $CDCl_3$)

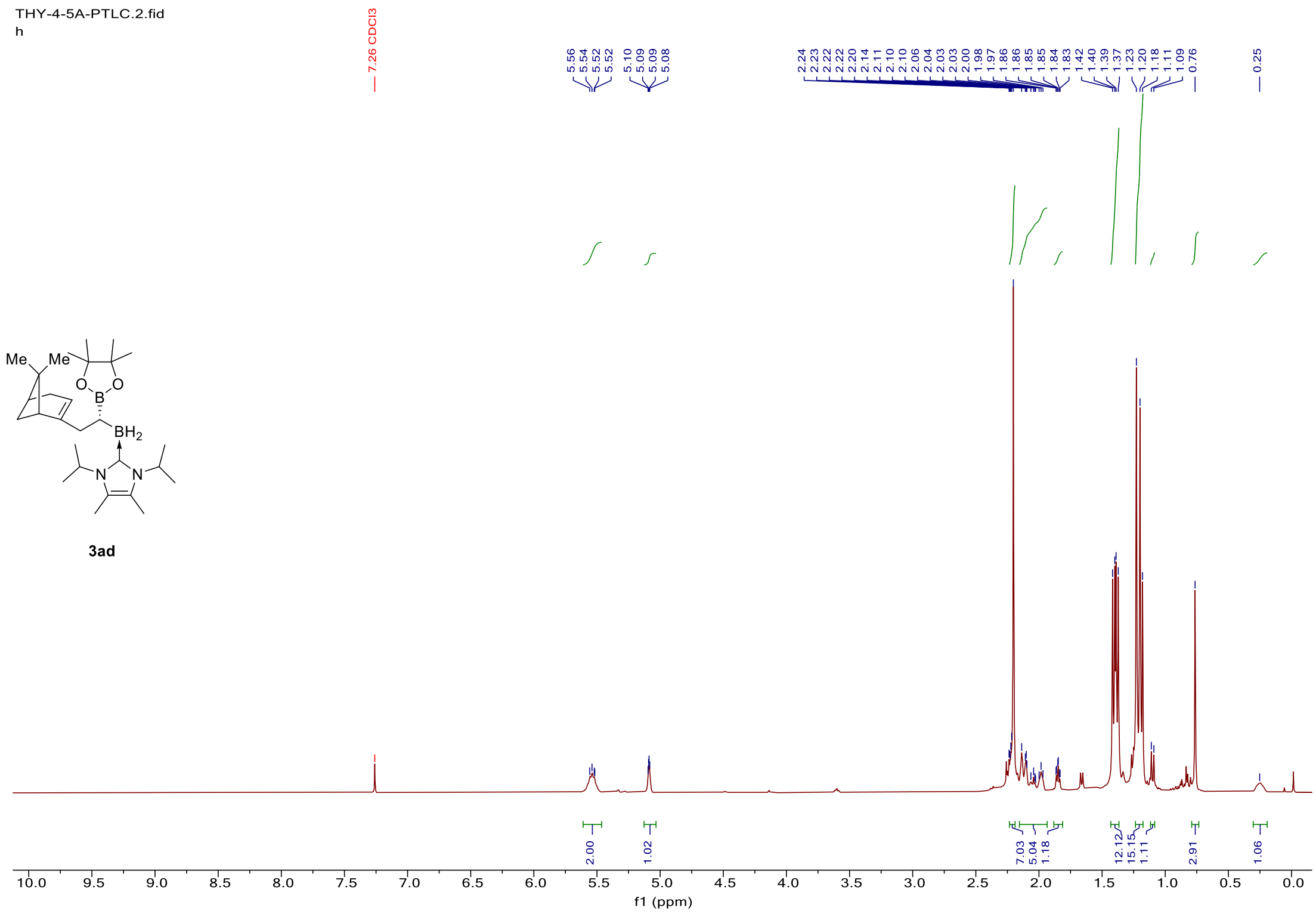

^{1}H NMR (400 MHz, $CDCl_3$)

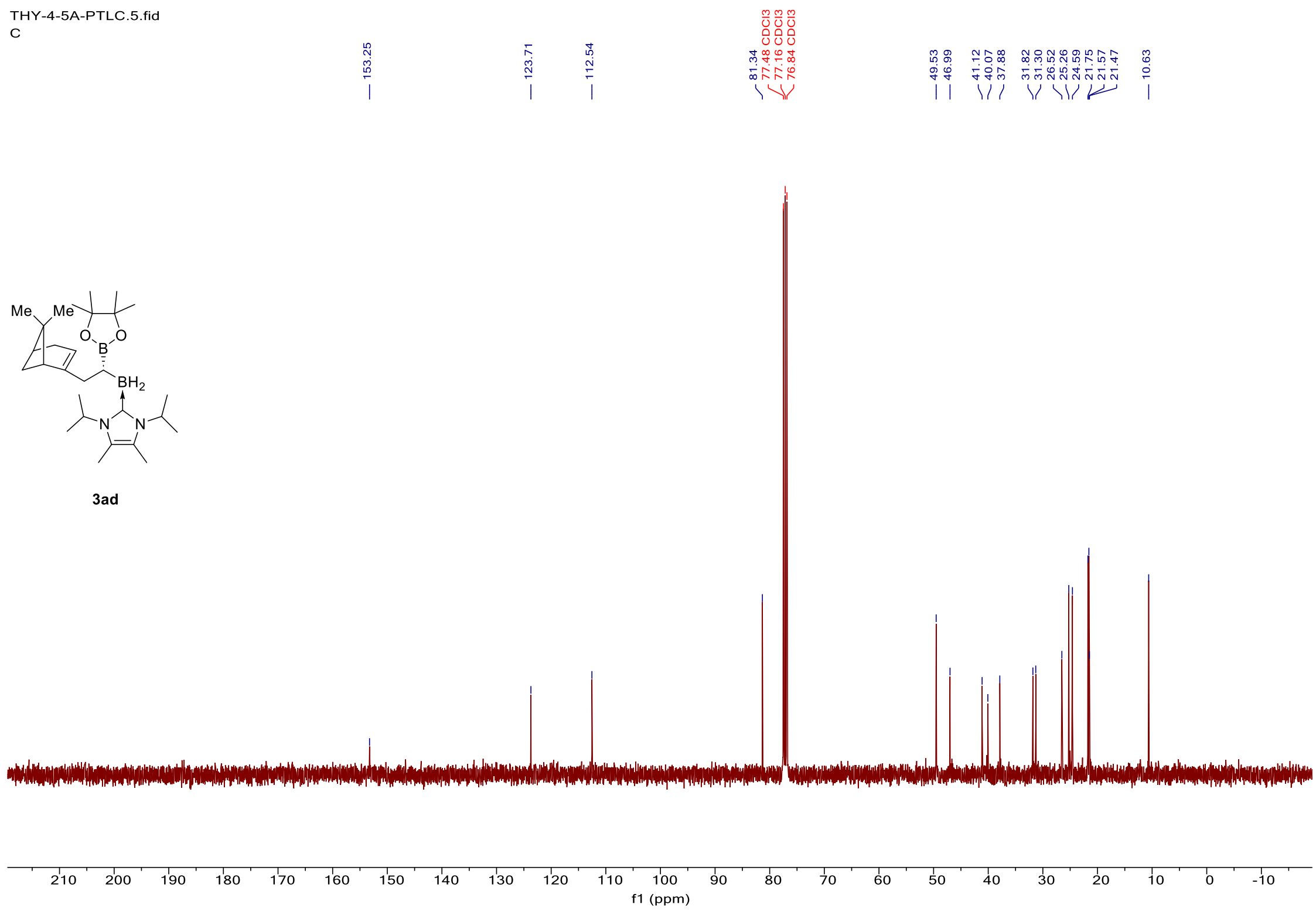

^{13}C NMR (101 MHz, $CDCl_3$)

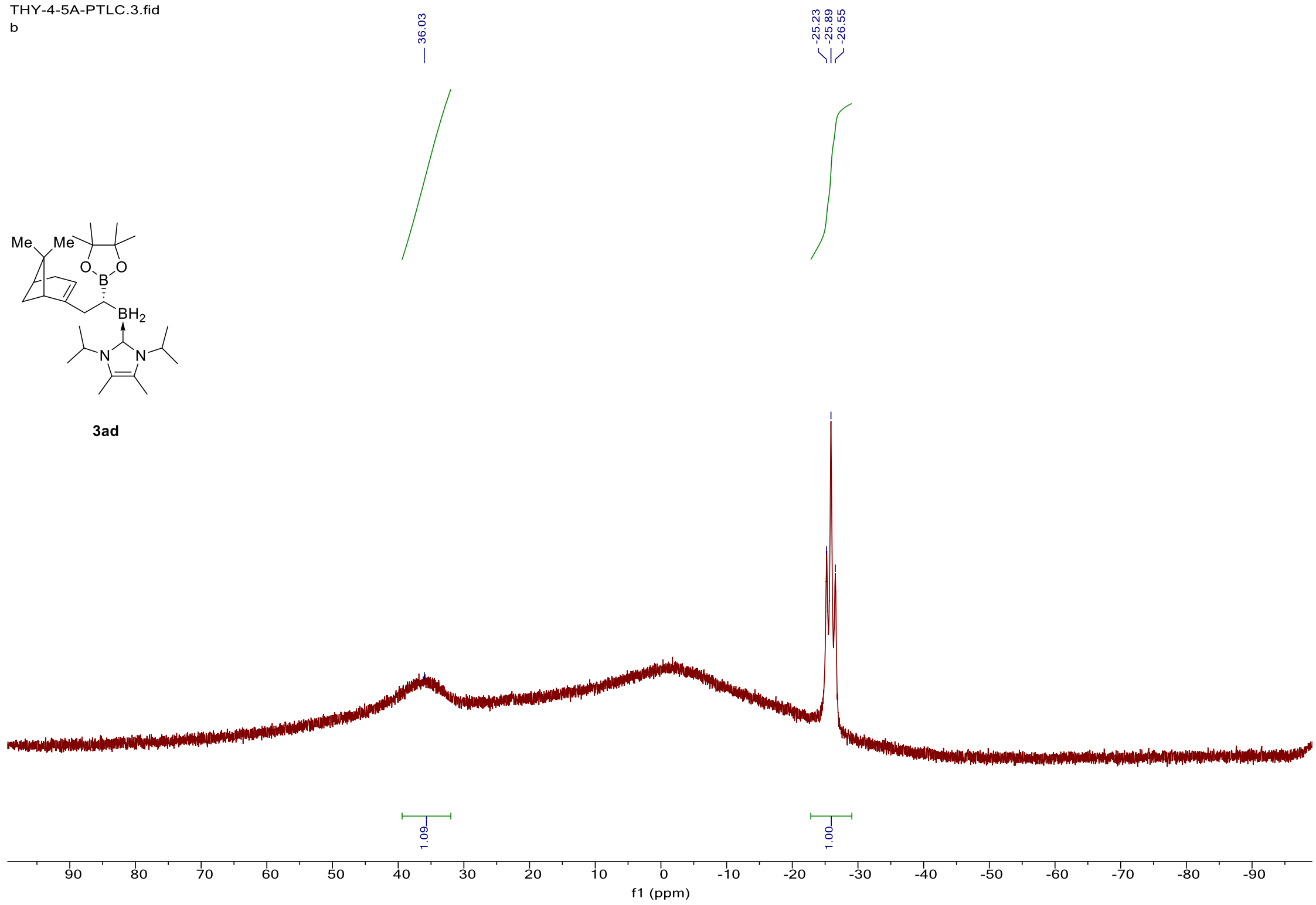

^{11}B NMR (128 MHz, $CDCl_3$)

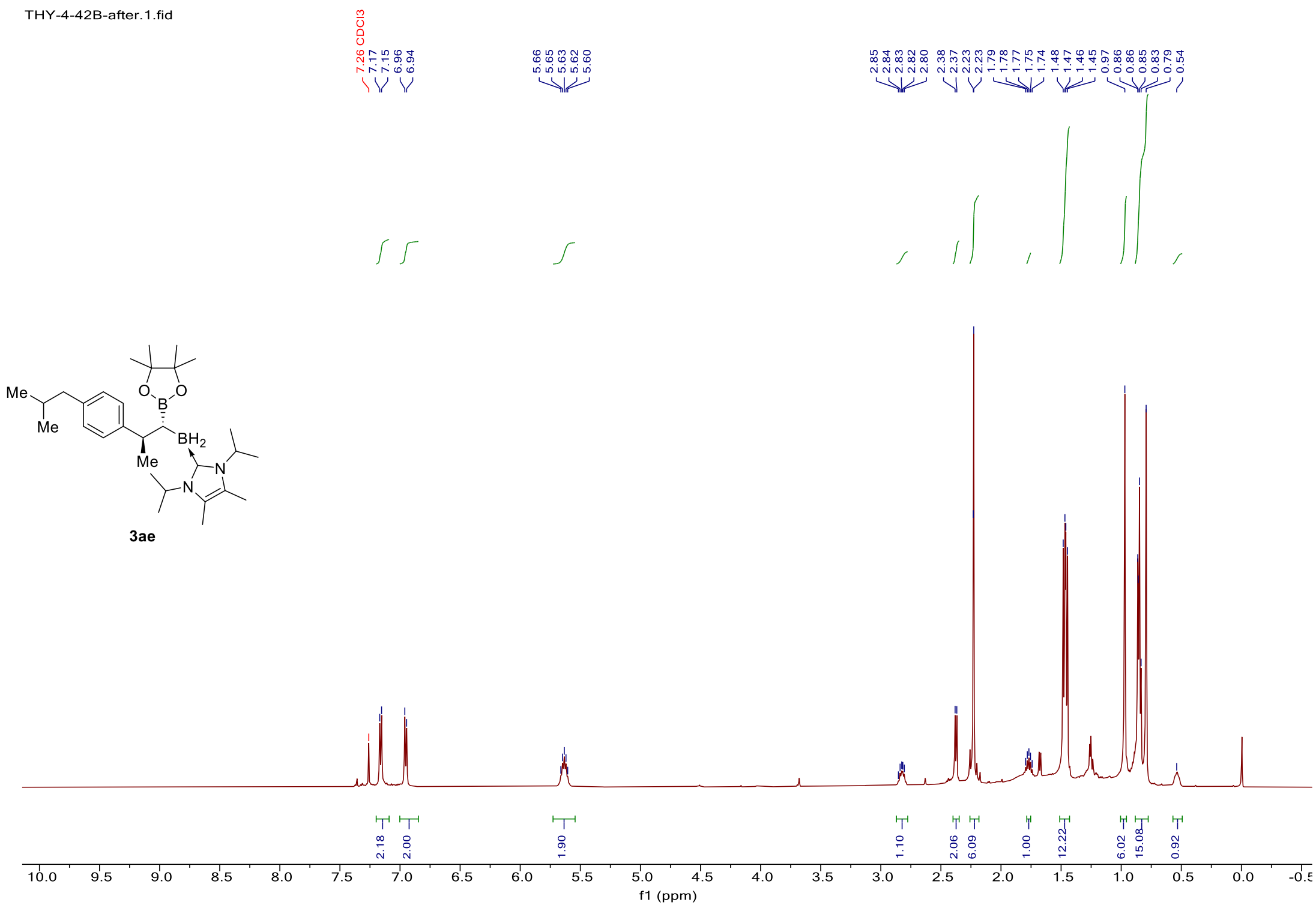

^{1}H NMR (500 MHz, $CDCl_3$)

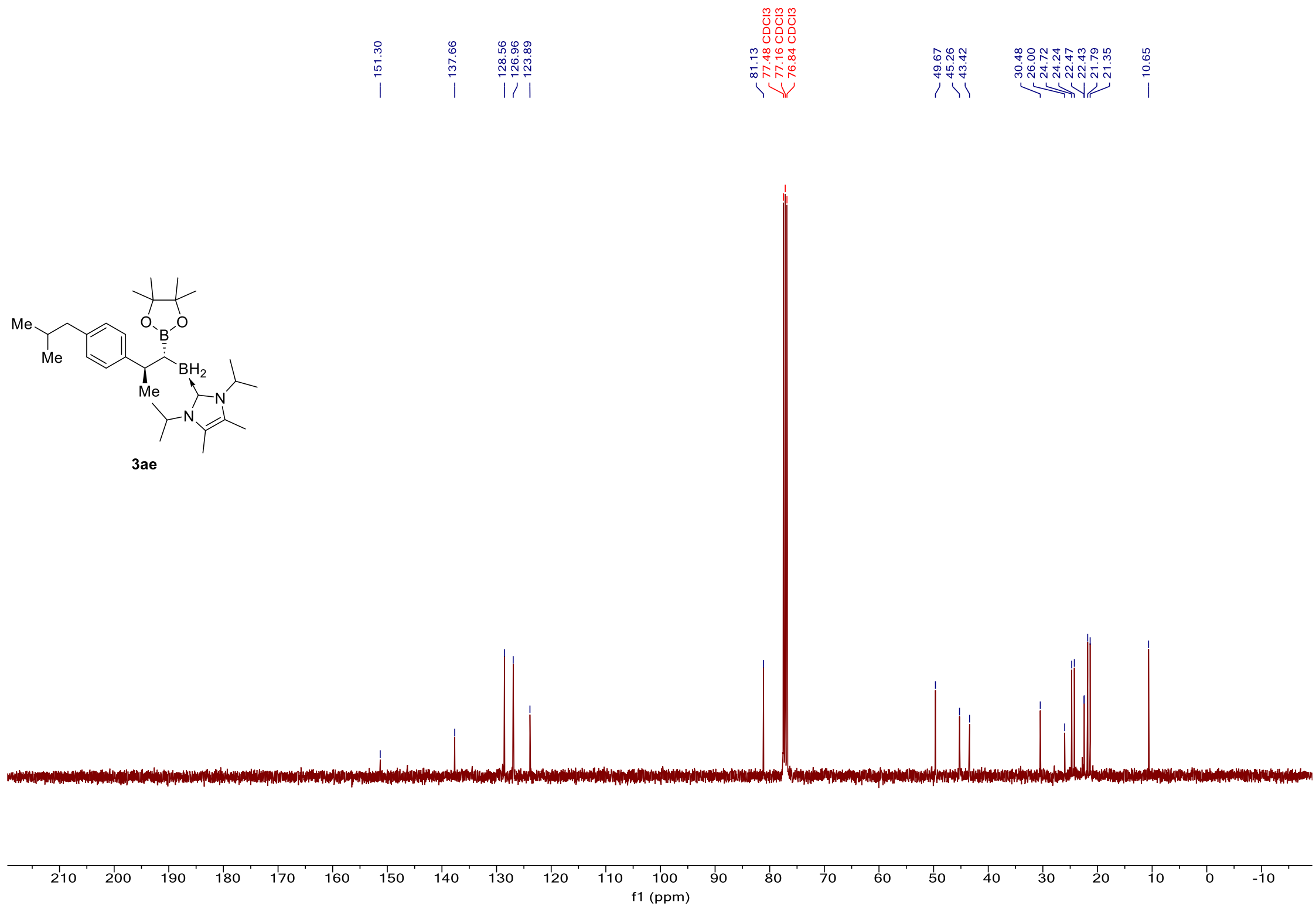

^{13}C NMR (101 MHz, $CDCl_3$)

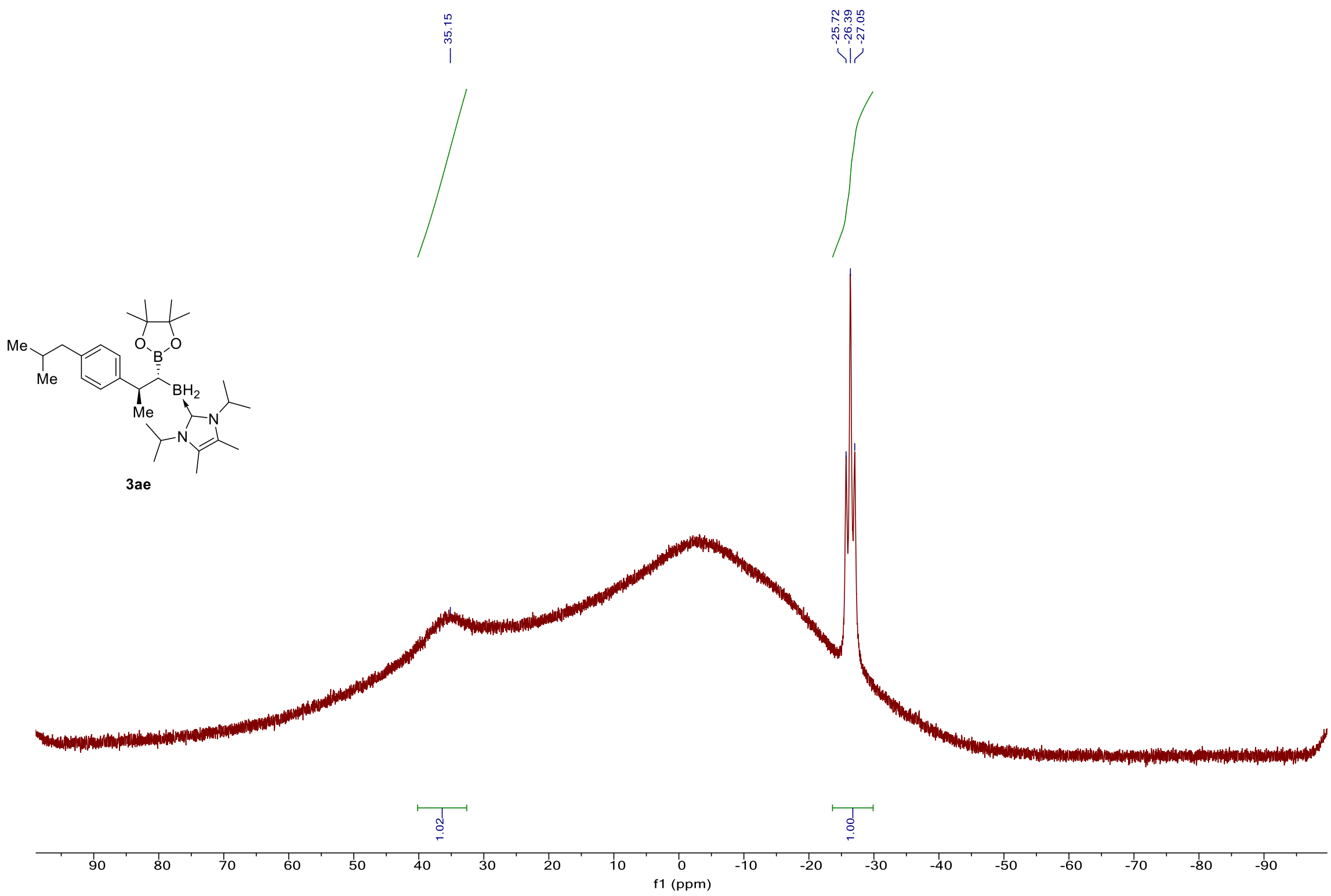

^{11}B NMR (160 MHz, $CDCl_3$)

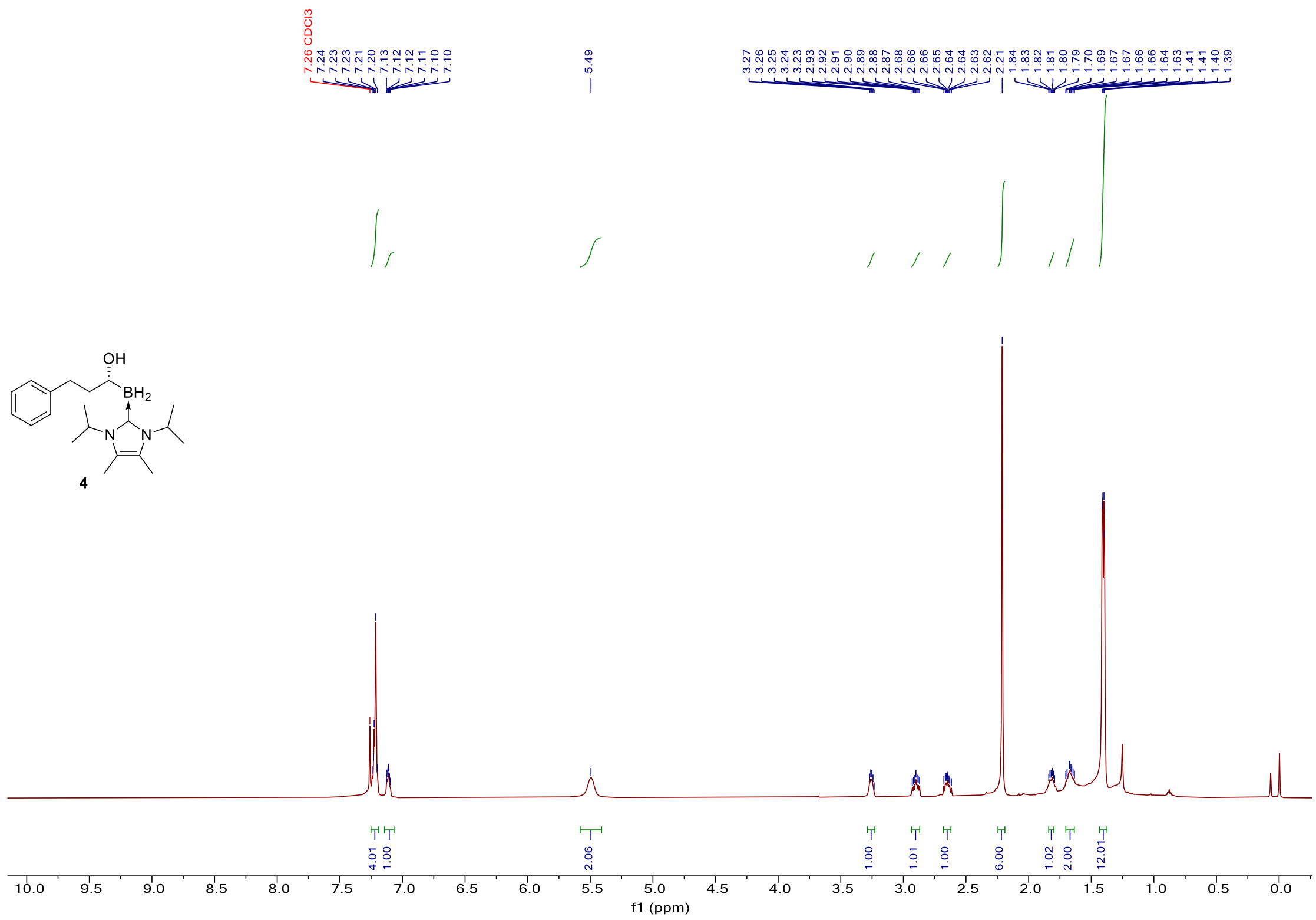

^{1}H NMR (500 MHz, $CDCl_3$)

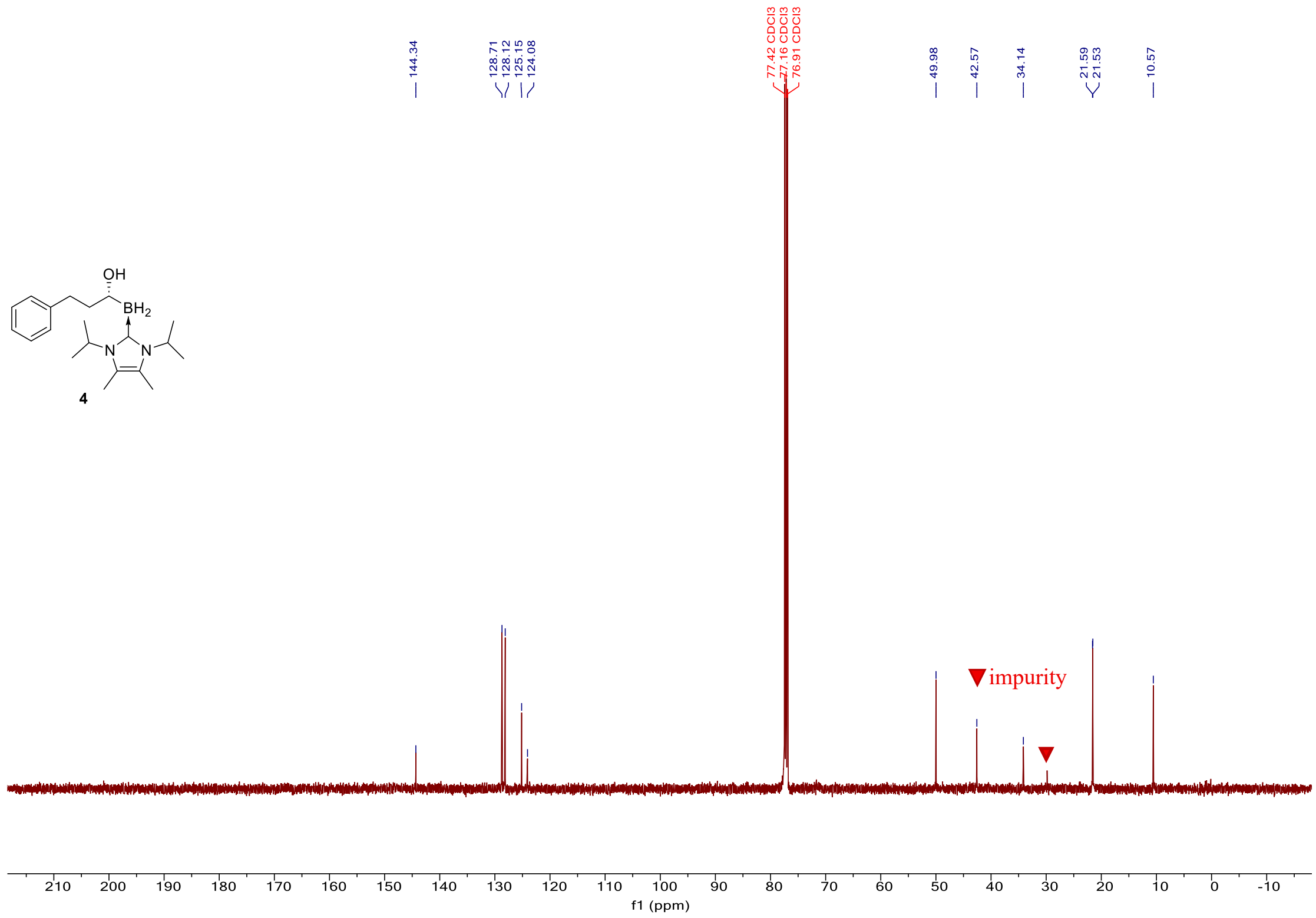

^{13}C NMR (126 MHz, $CDCl_3$)

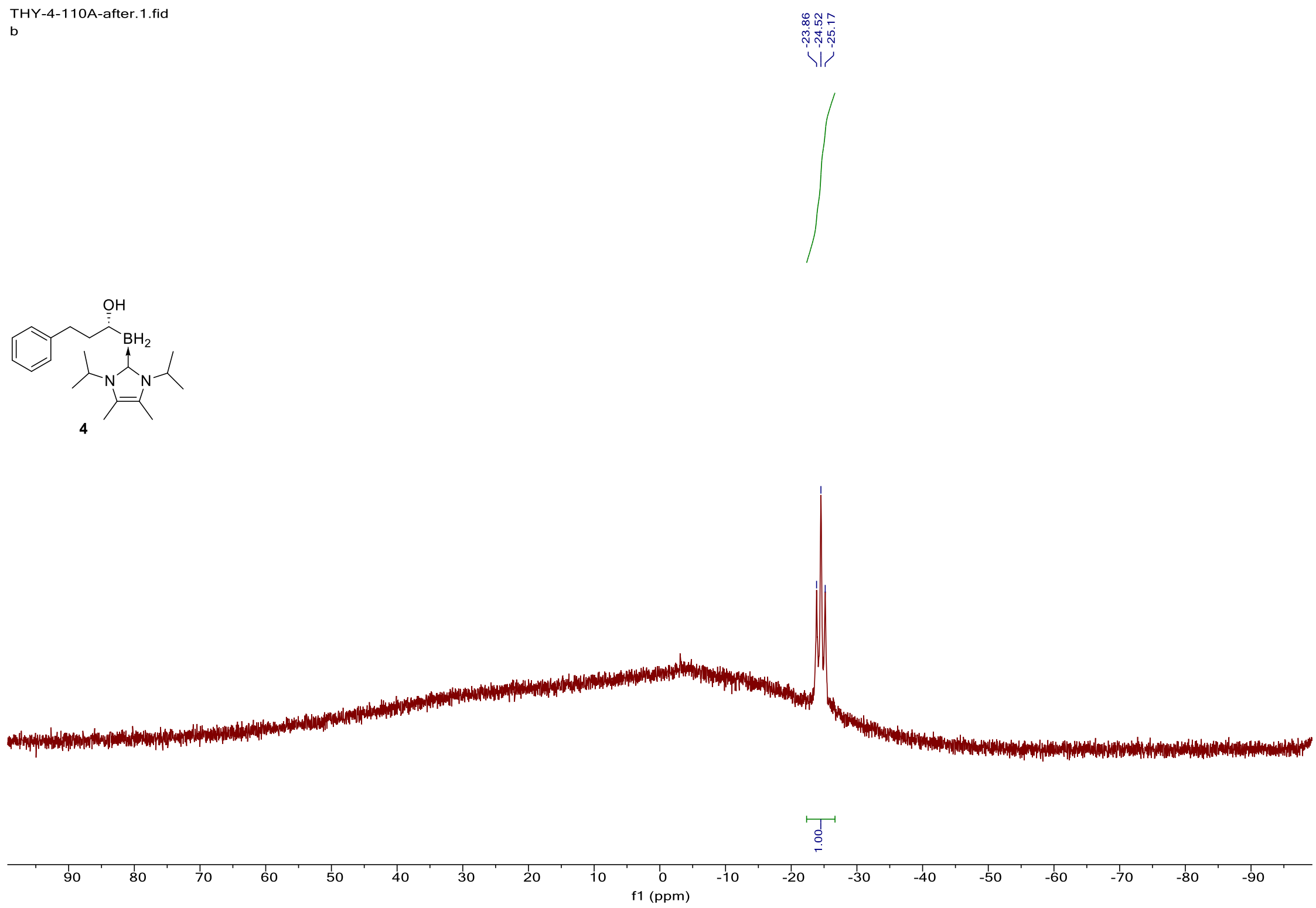

^{11}B NMR (128 MHz, $CDCl_3$)

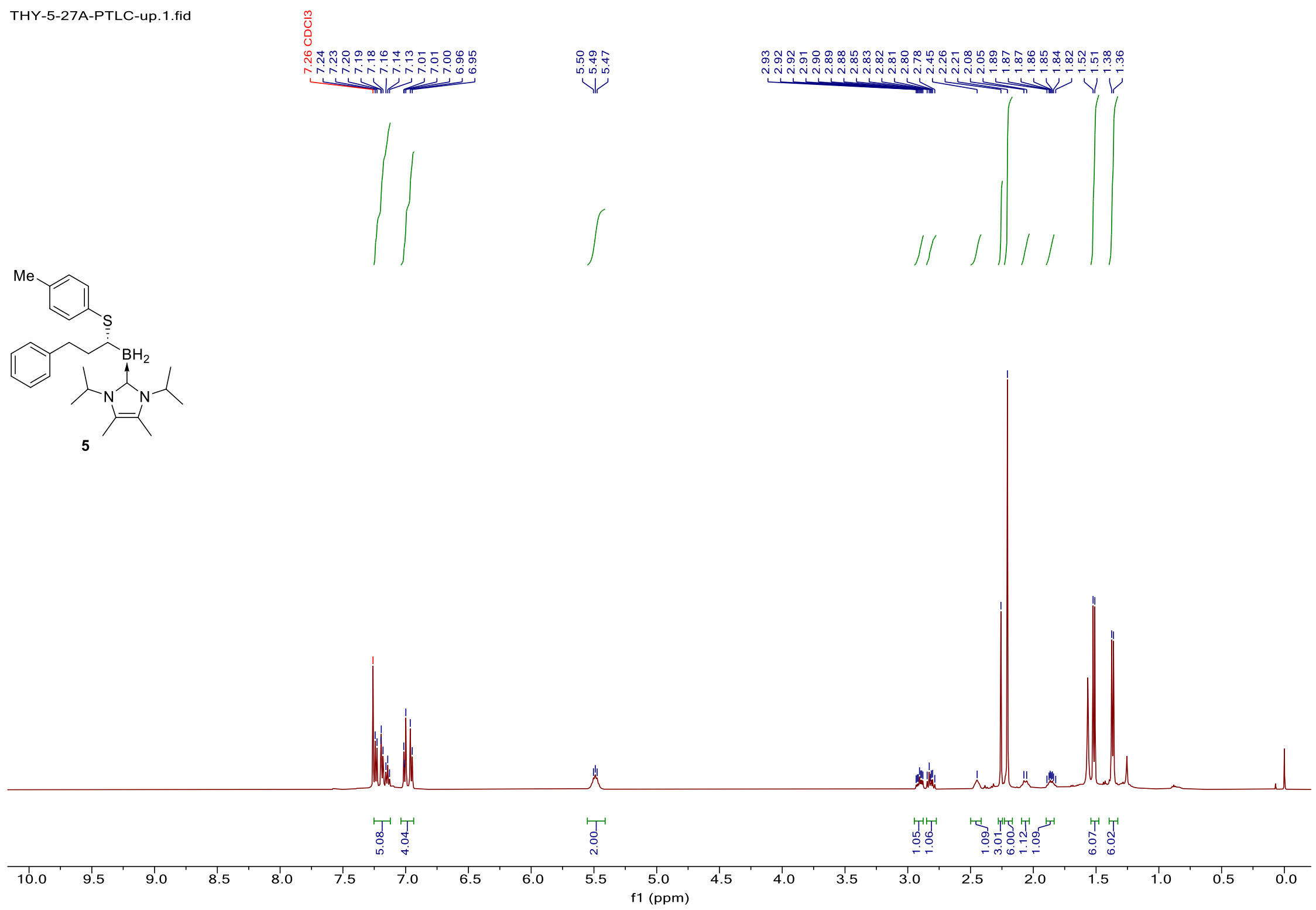

^{1}H NMR (500 MHz, $CDCl_3$)

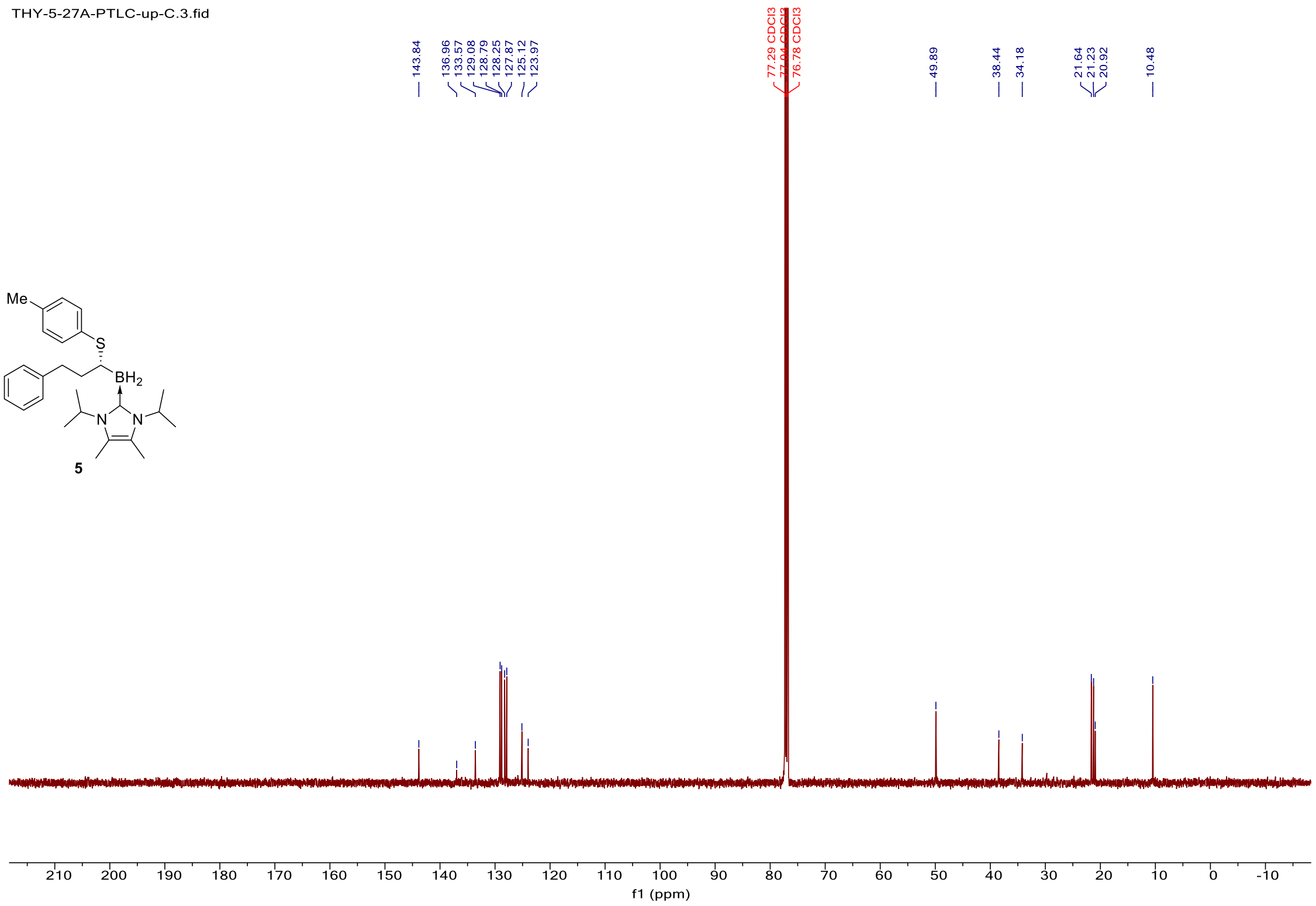

^{13}C NMR (126 MHz, $CDCl_3$)

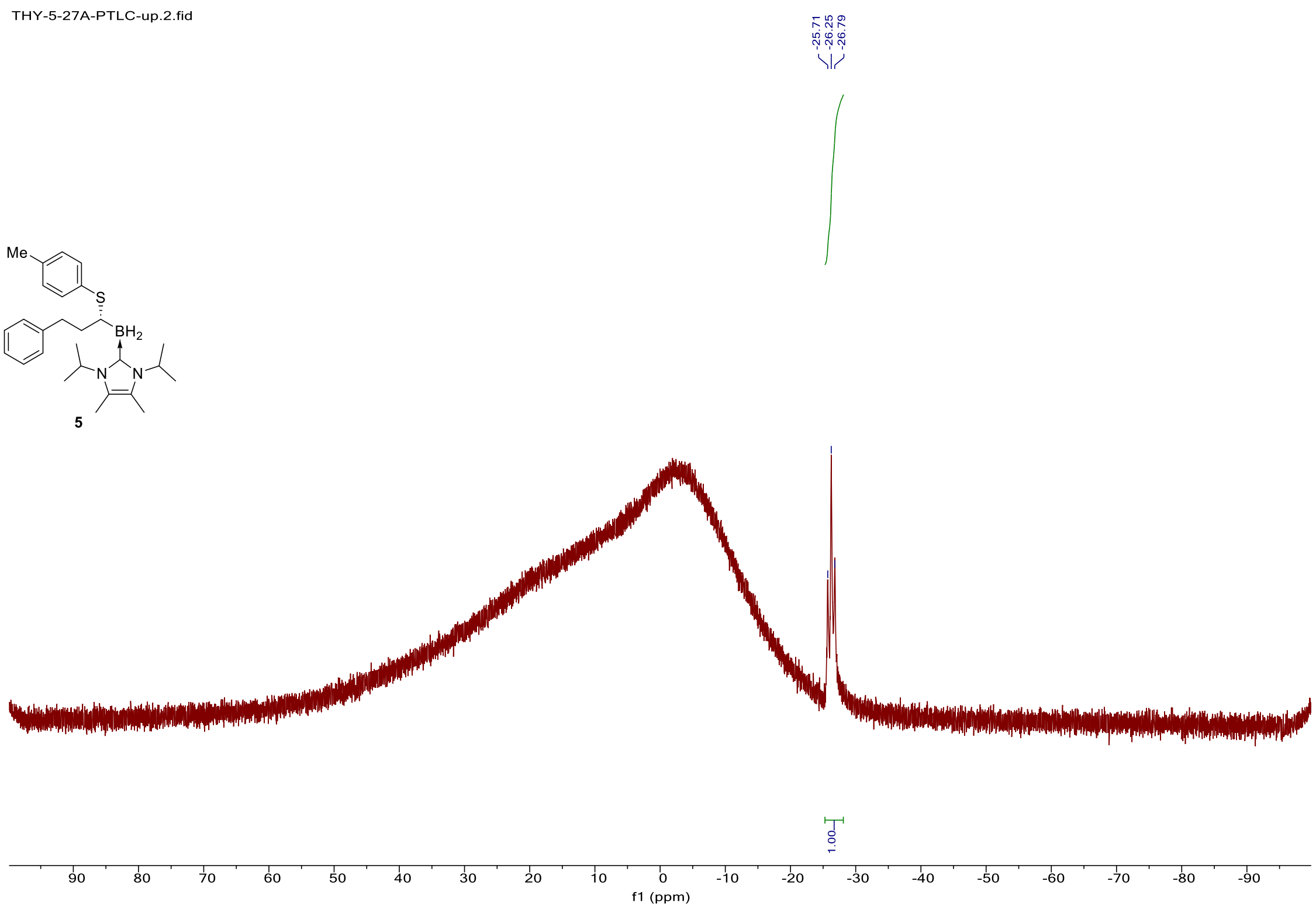

^{11}B NMR (160 MHz, $CDCl_3$)

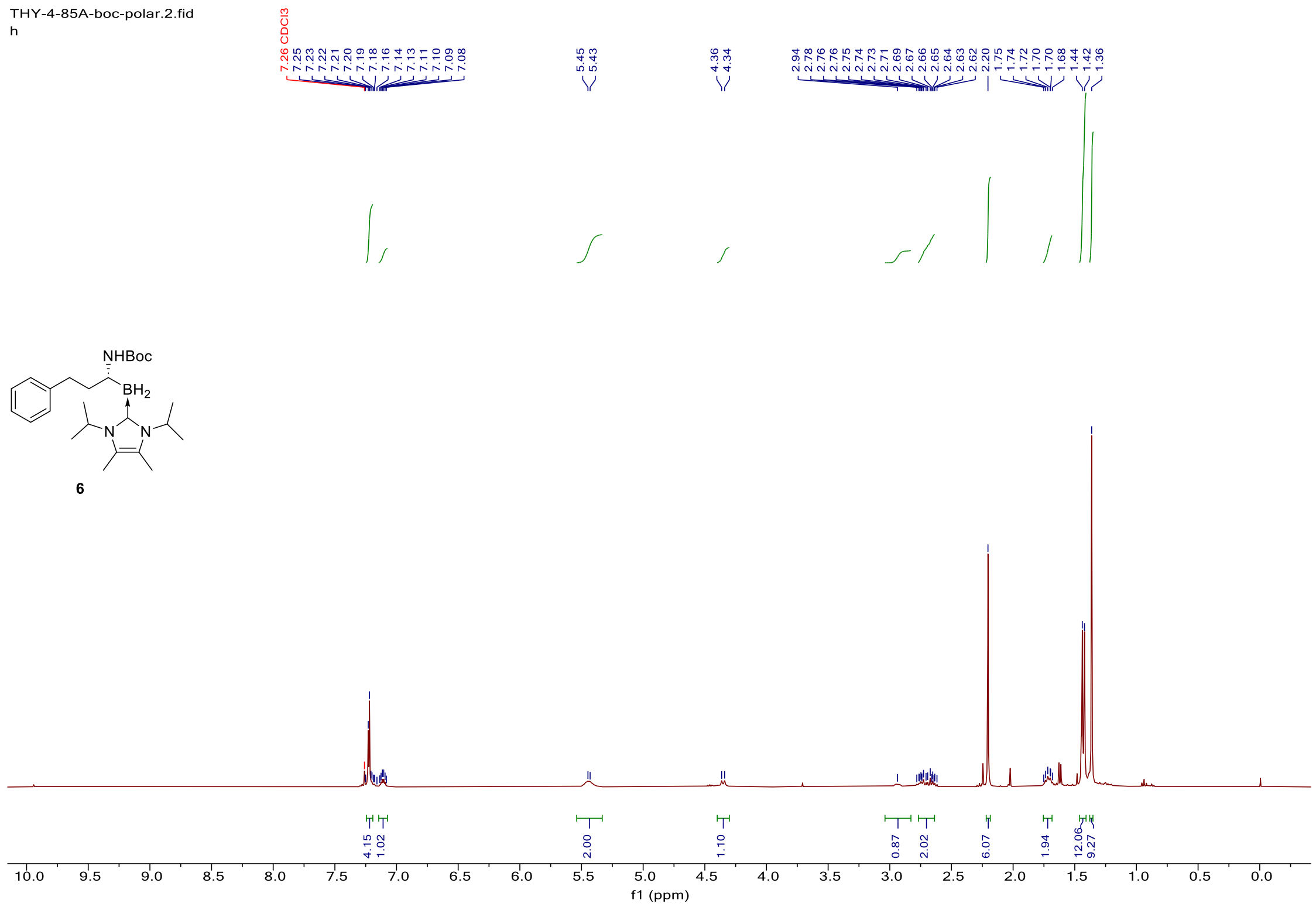

^{1}H NMR (400 MHz, $CDCl_3$)

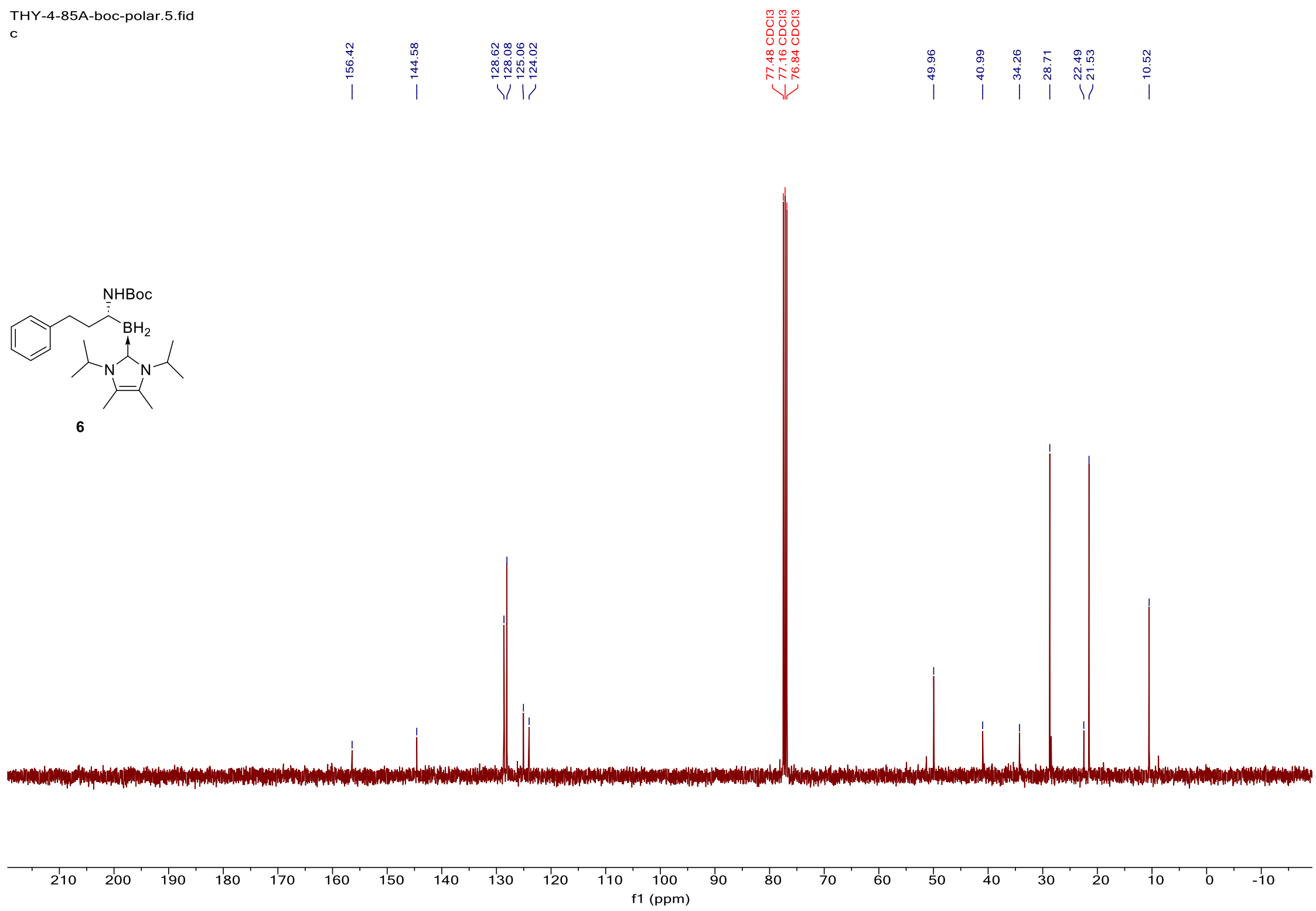

^{13}C NMR (101 MHz, $CDCl_3$)

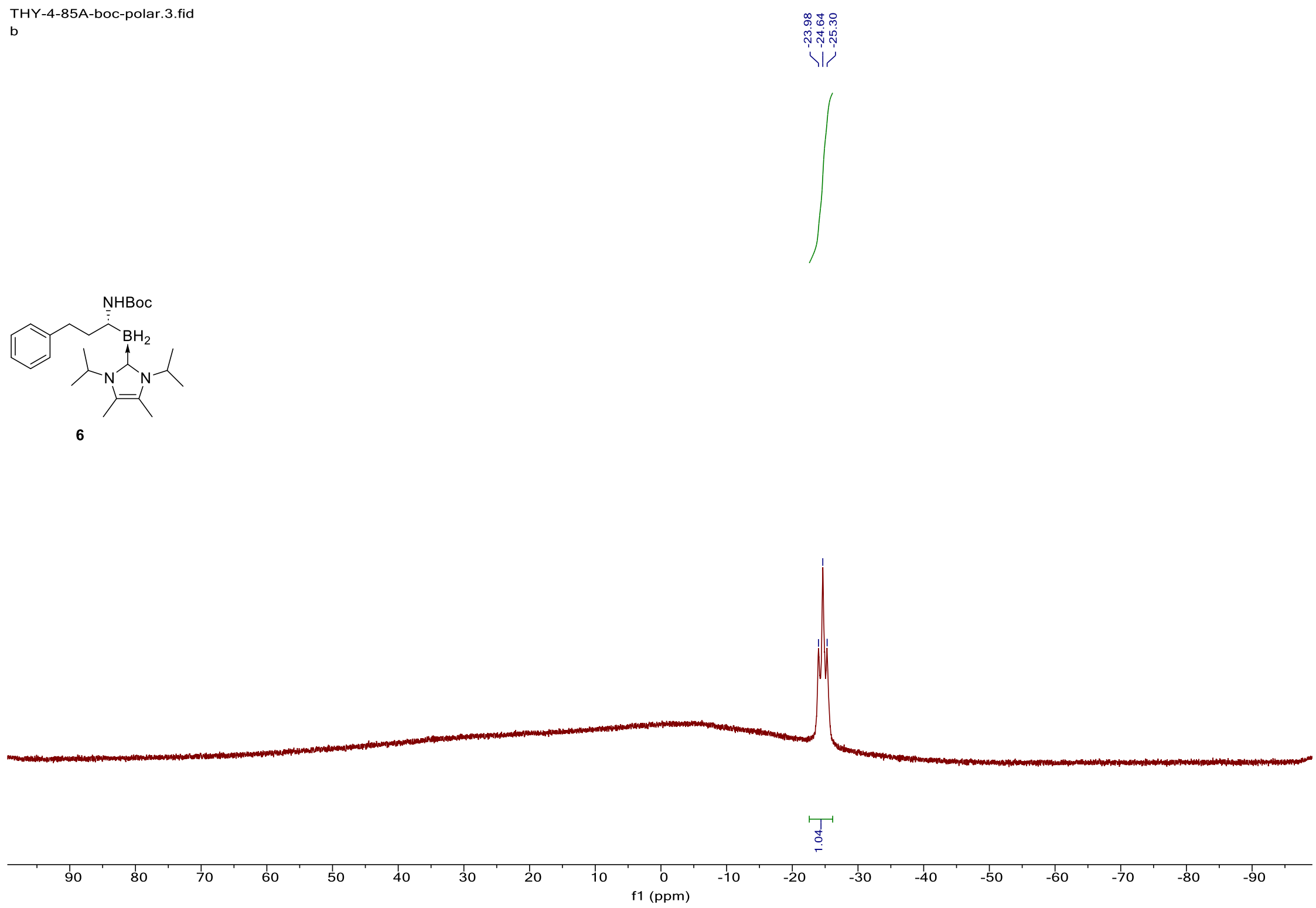

^{11}B NMR (128 MHz, $CDCl_3$)

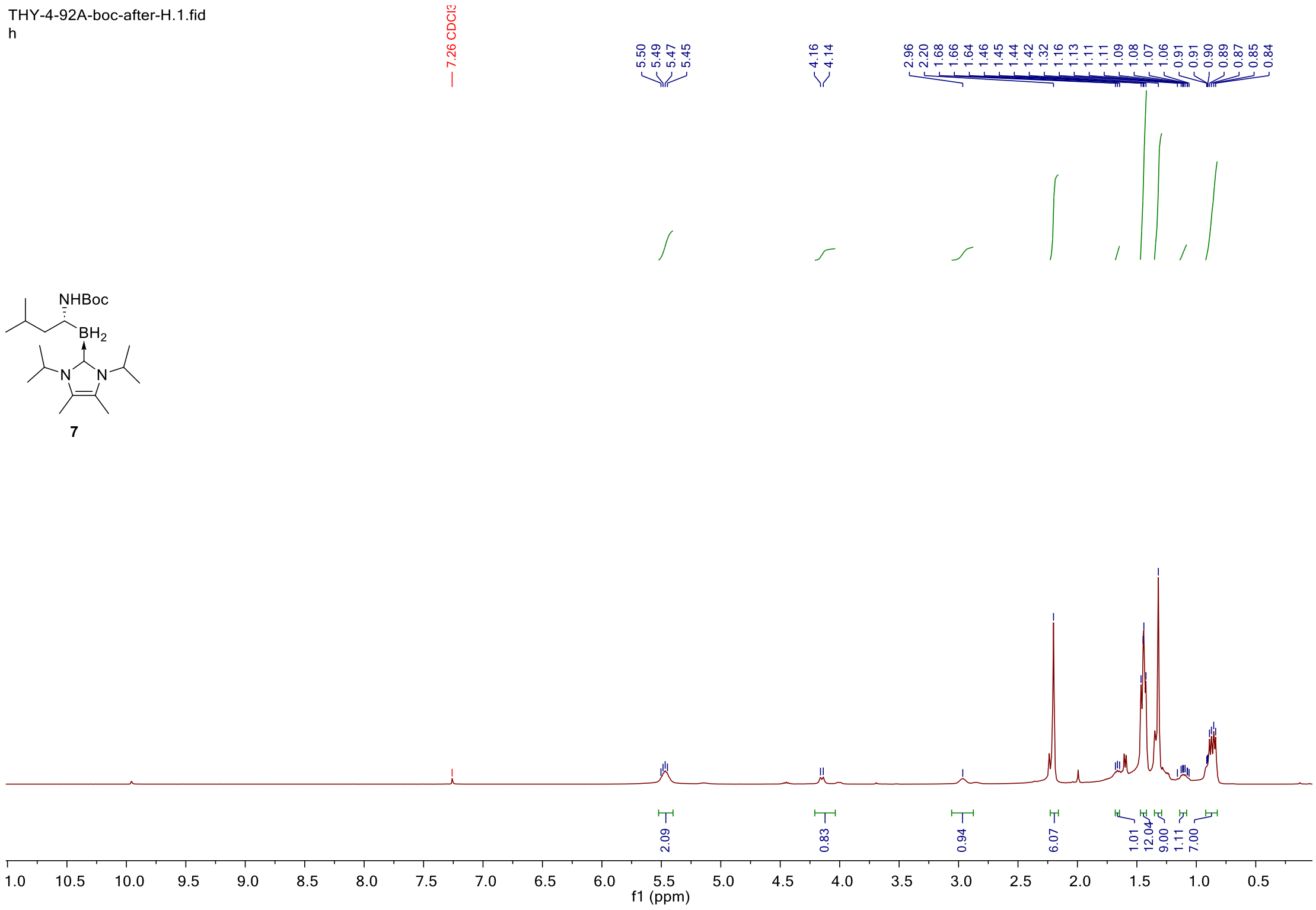

^{1}H NMR (500 MHz, $CDCl_3$)

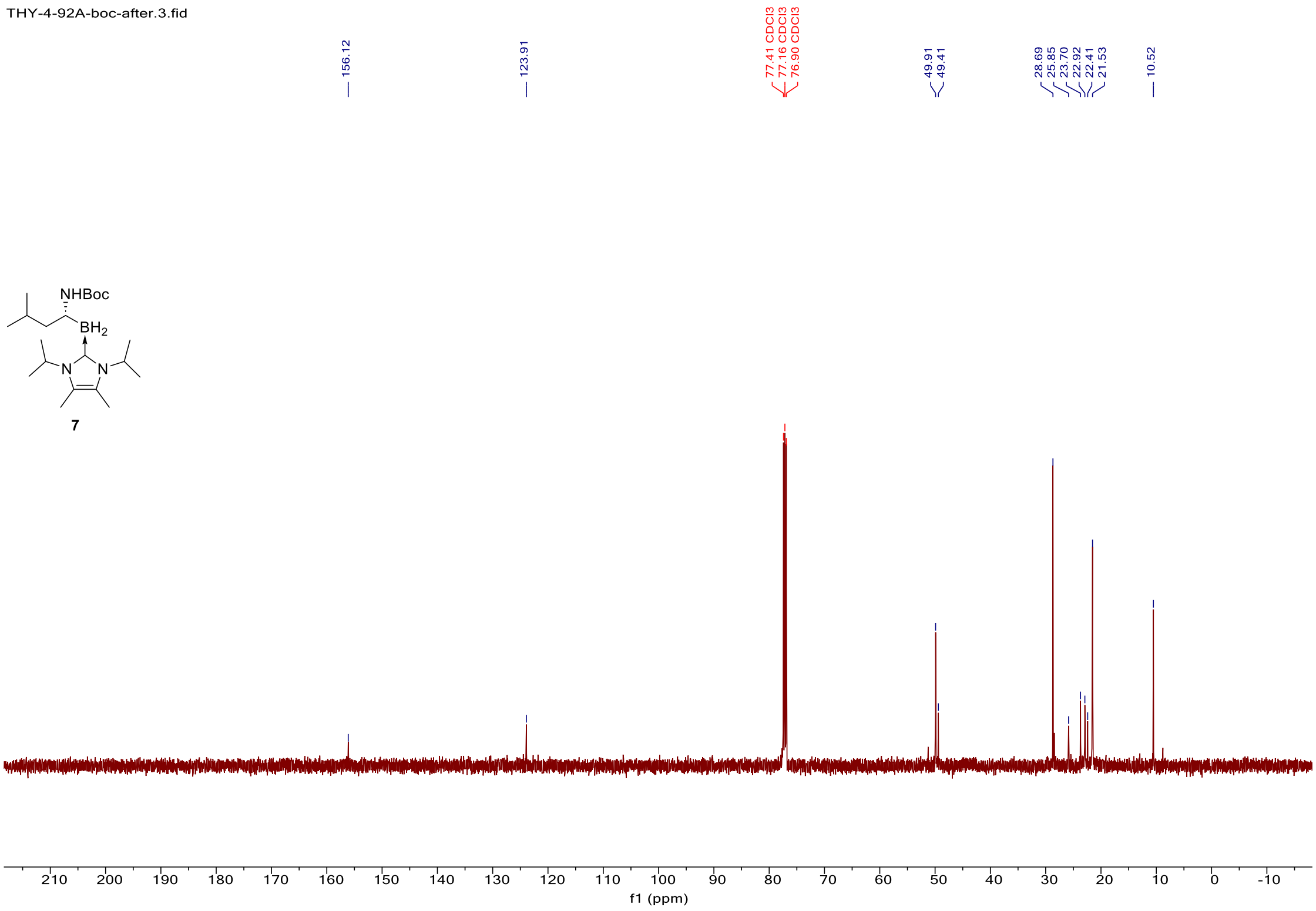

^{13}C NMR (126 MHz, $CDCl_3$)

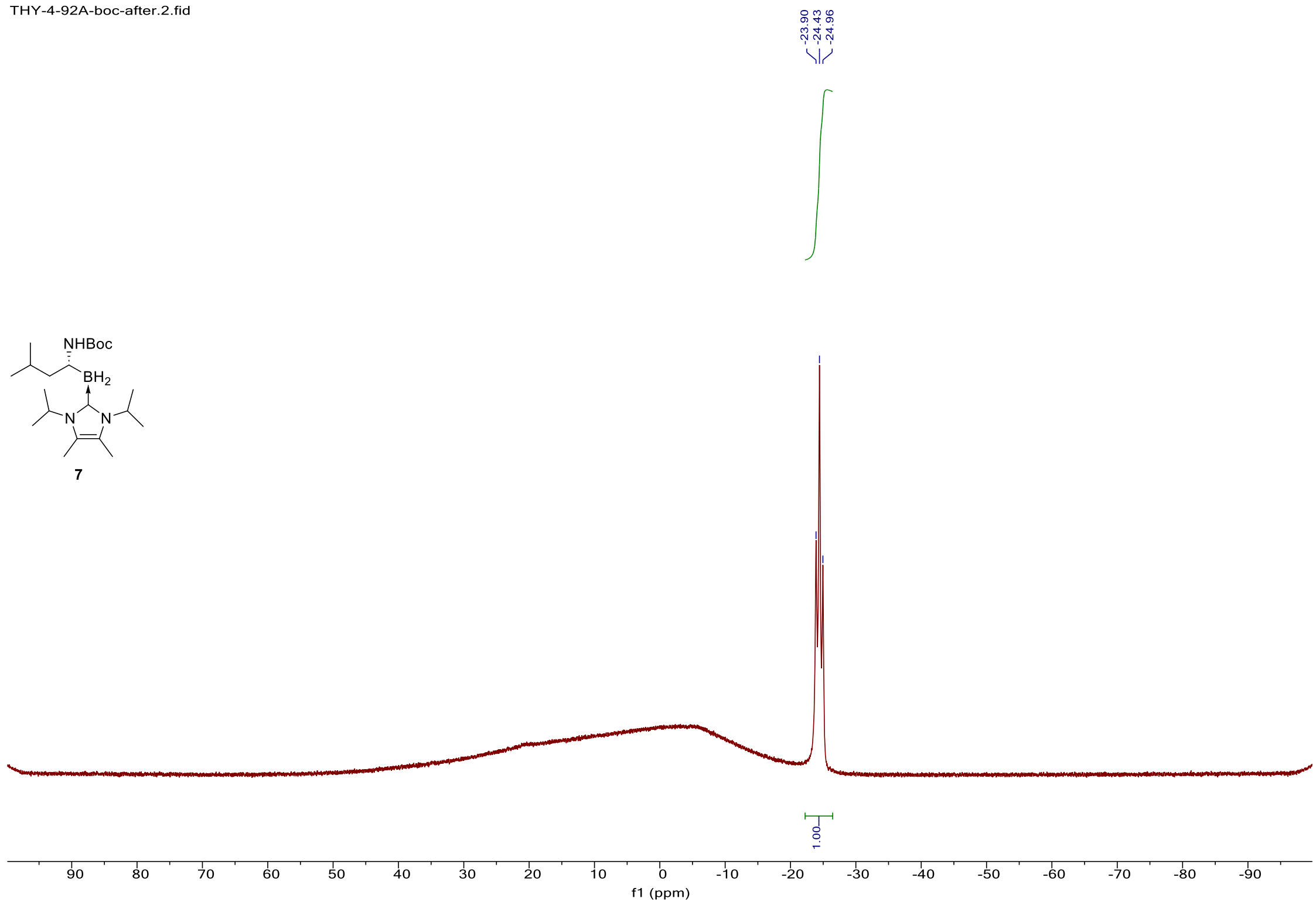

^{11}B NMR (160 MHz, $CDCl_3$)

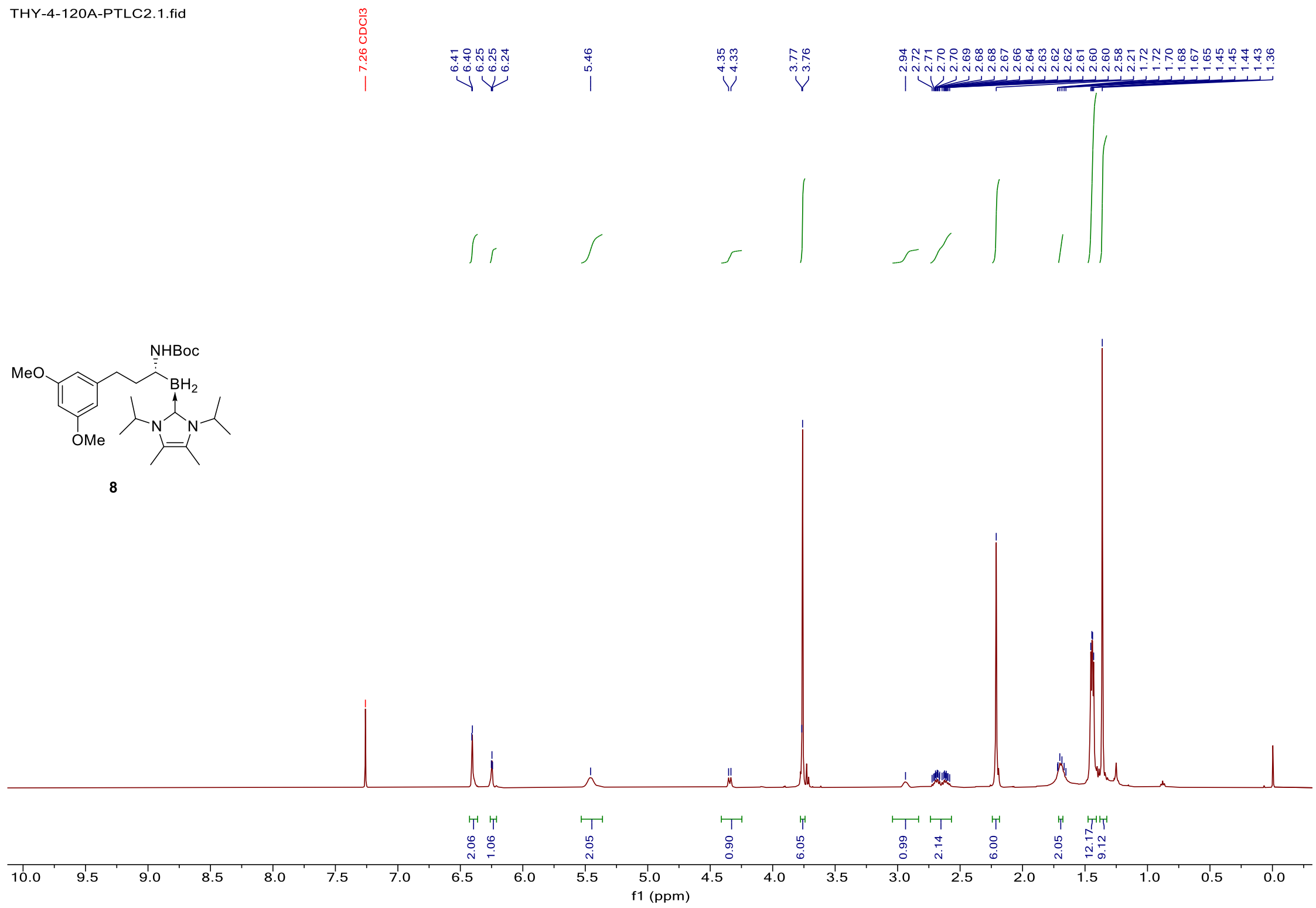

^{1}H NMR (500 MHz, $CDCl_3$)

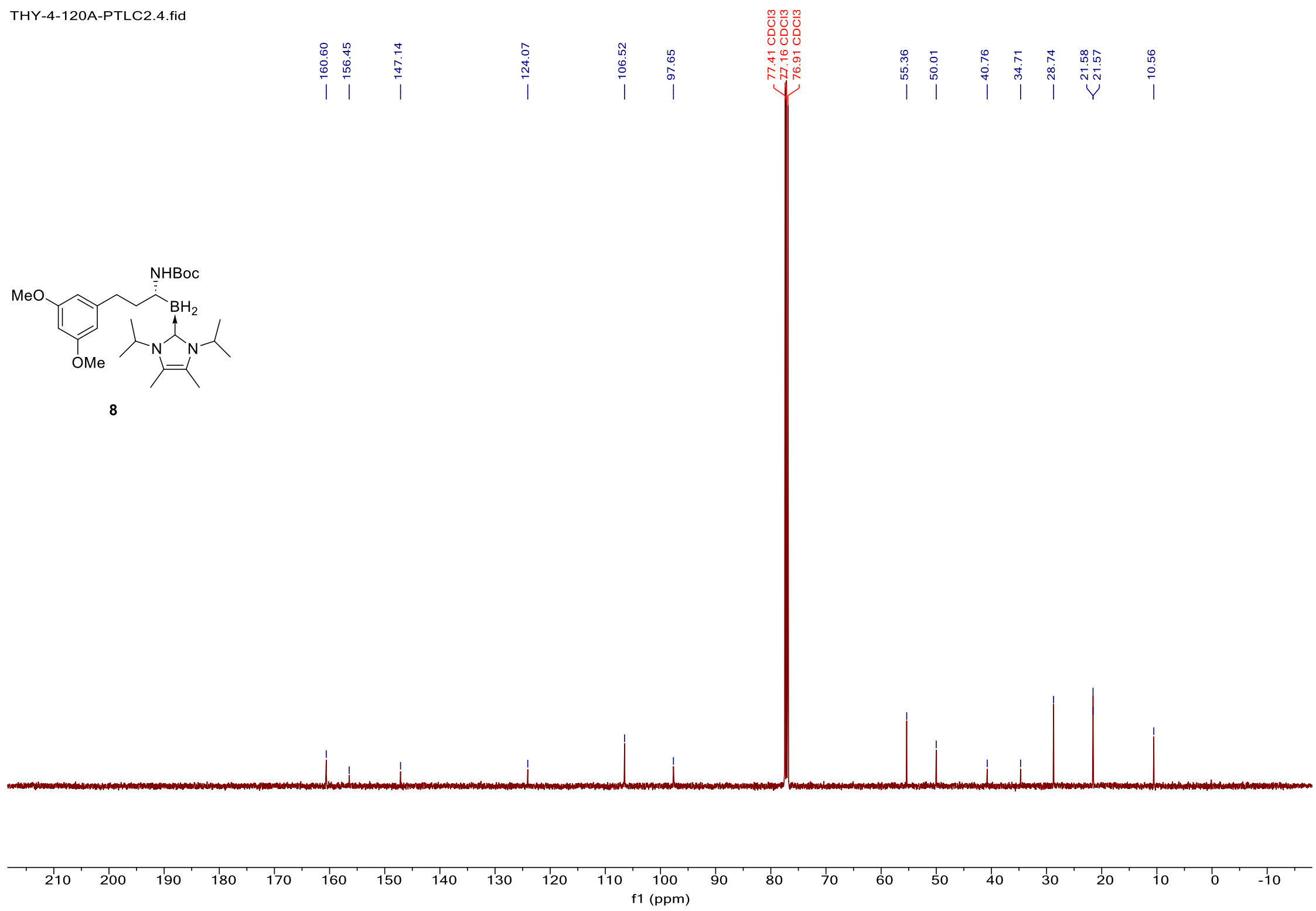

^{13}C NMR (126 MHz, $CDCl_3$)

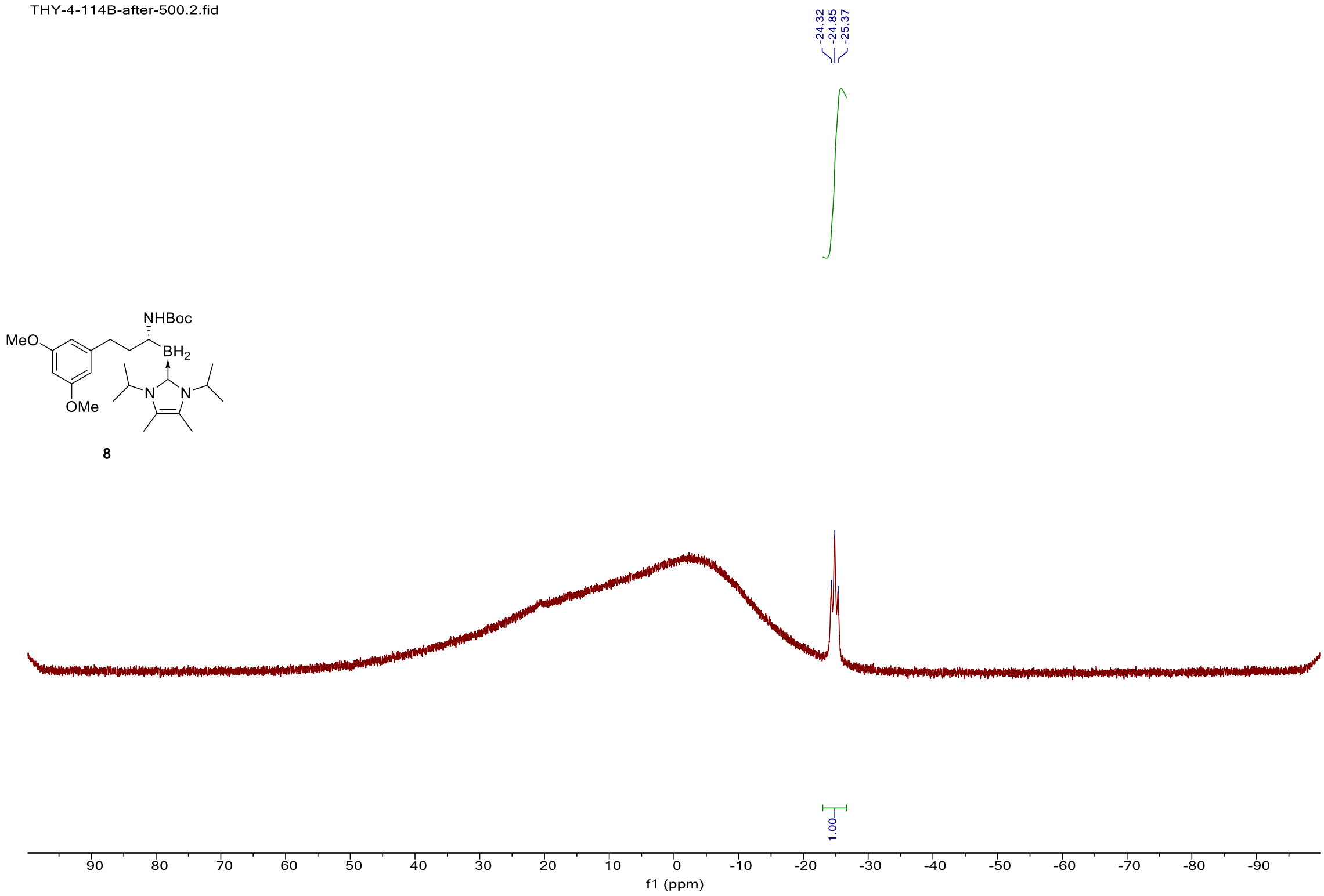

^{11}B NMR (160 MHz, $CDCl_3$)

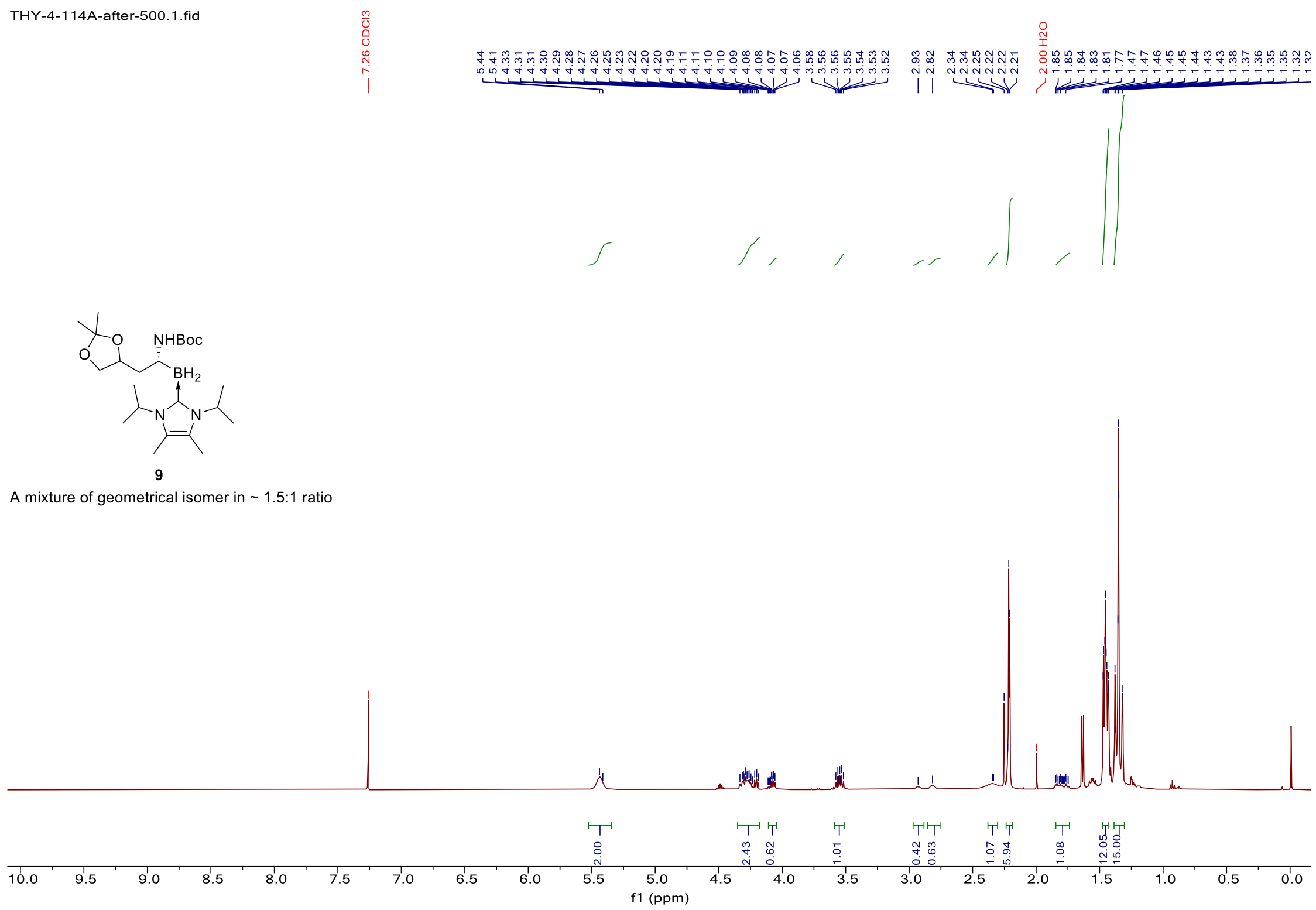

^{1}H NMR (500 MHz, $CDCl_3$)

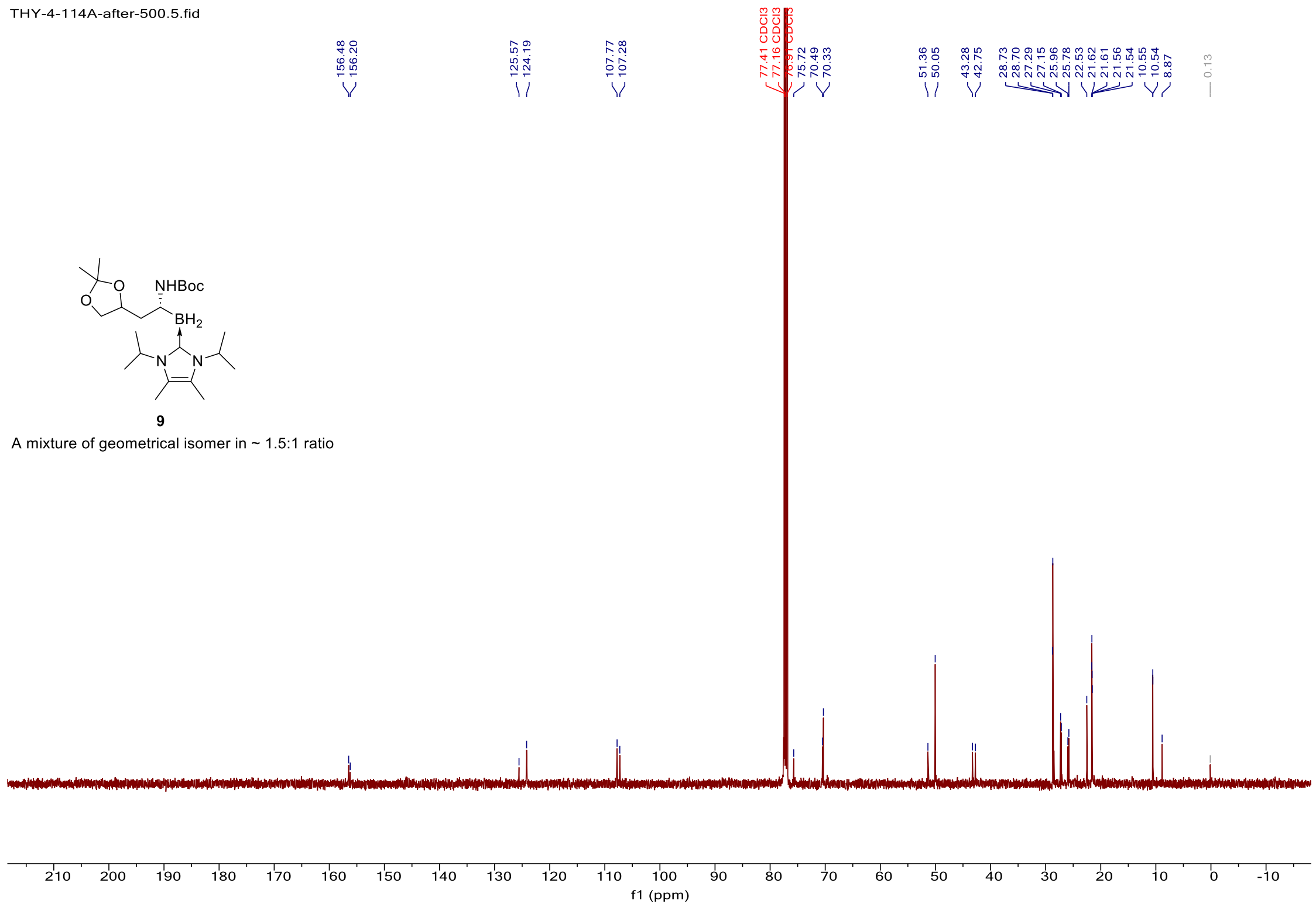

^{13}C NMR (126 MHz, $CDCl_3$)

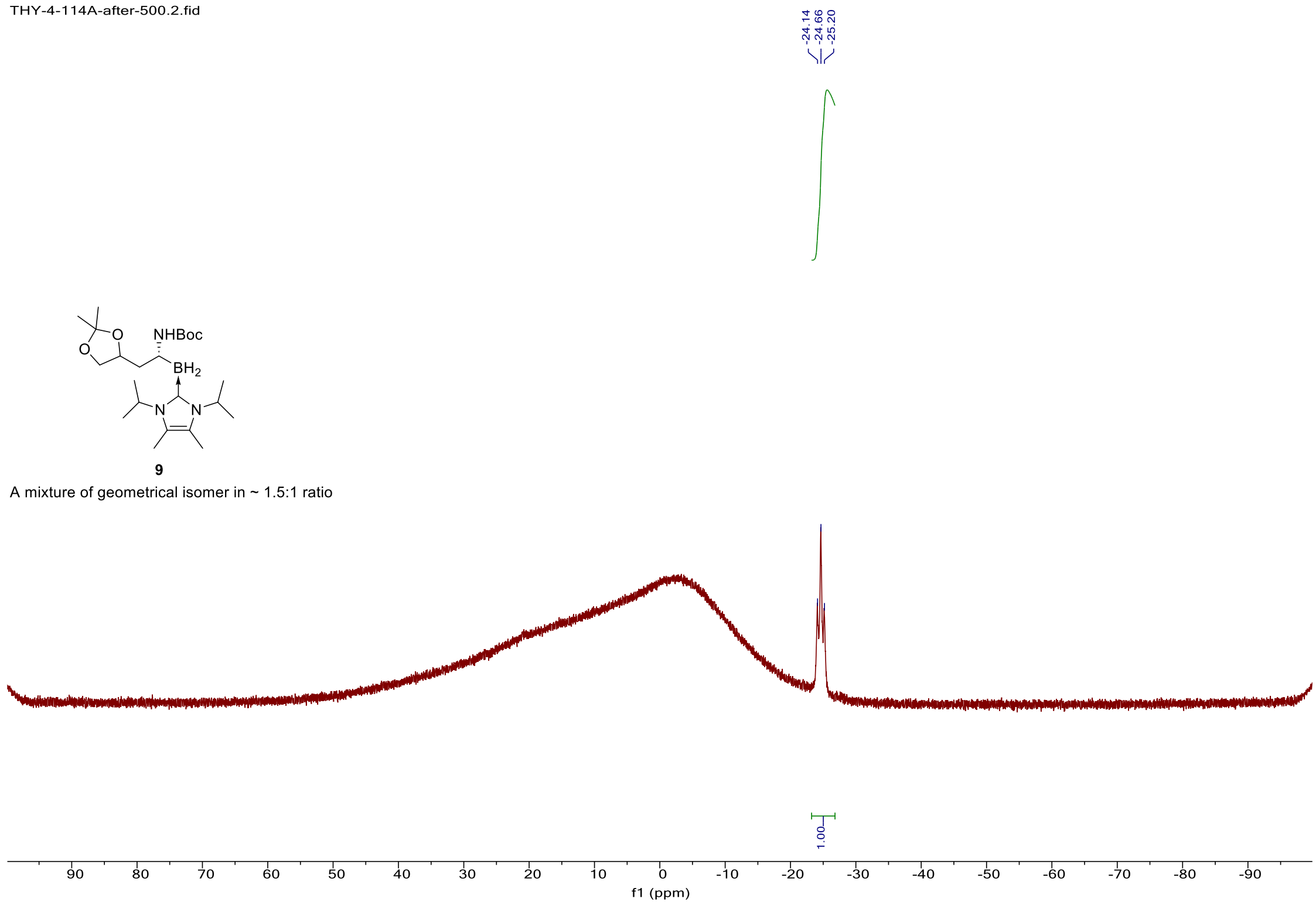

9

A mixture of geometrical isomer in ~ 1.5:1 ratio

^{11}B NMR (160 MHz, $CDCl_3$)

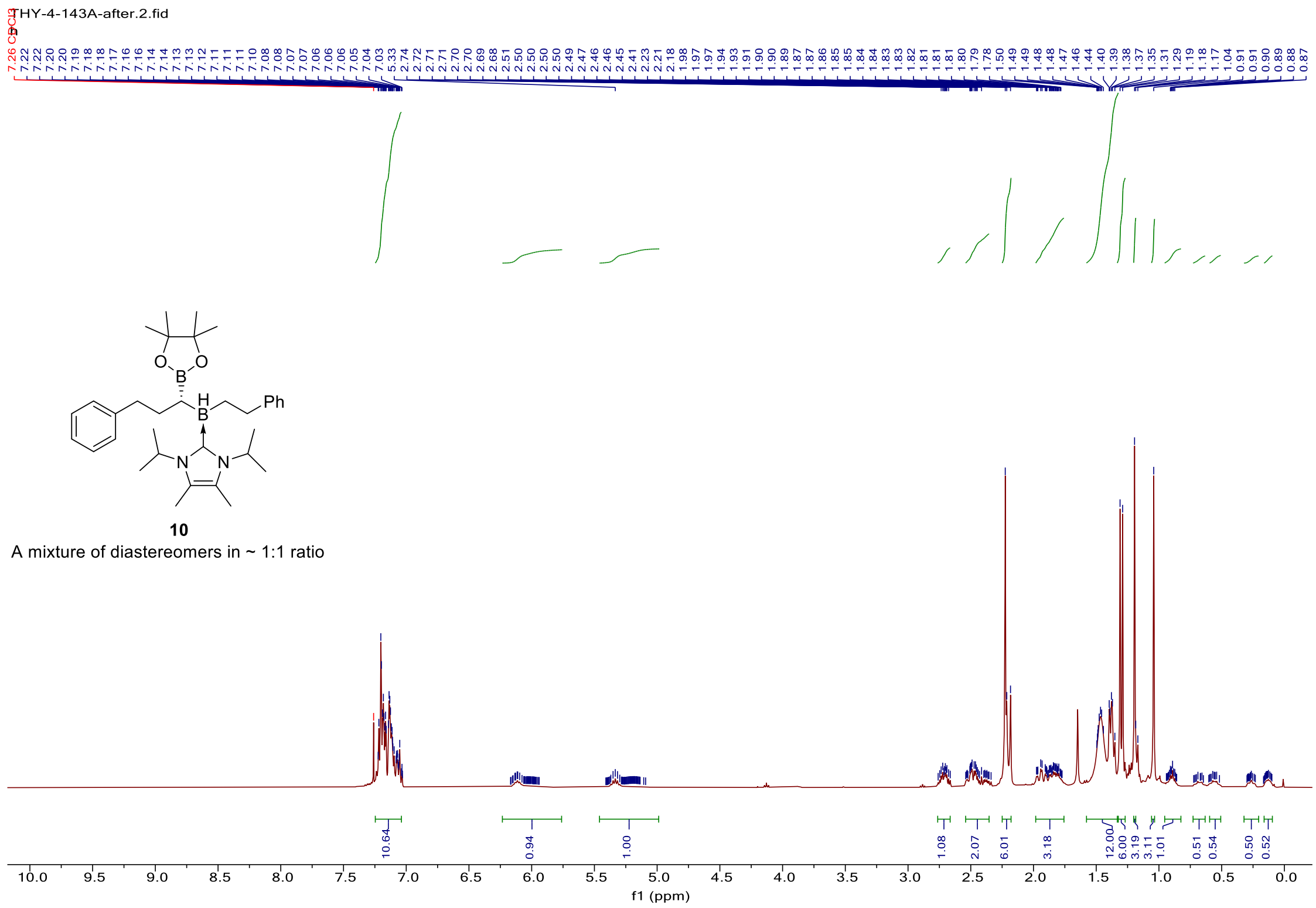

10

A mixture of diastereomers in ~ 1:1 ratio

^{1}H NMR (400 MHz, $CDCl_3$)

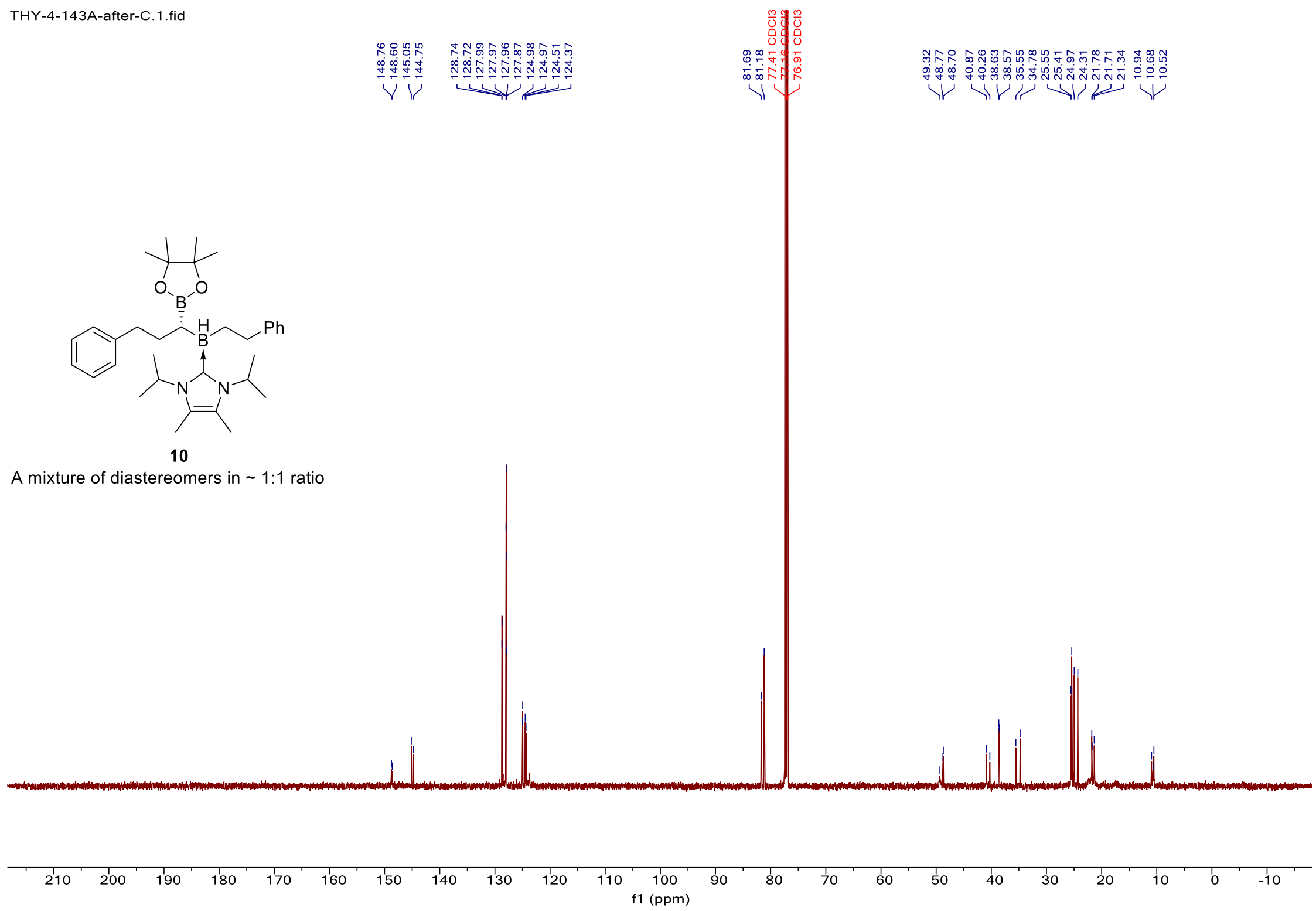

^{13}C NMR (101 MHz, $CDCl_3$)

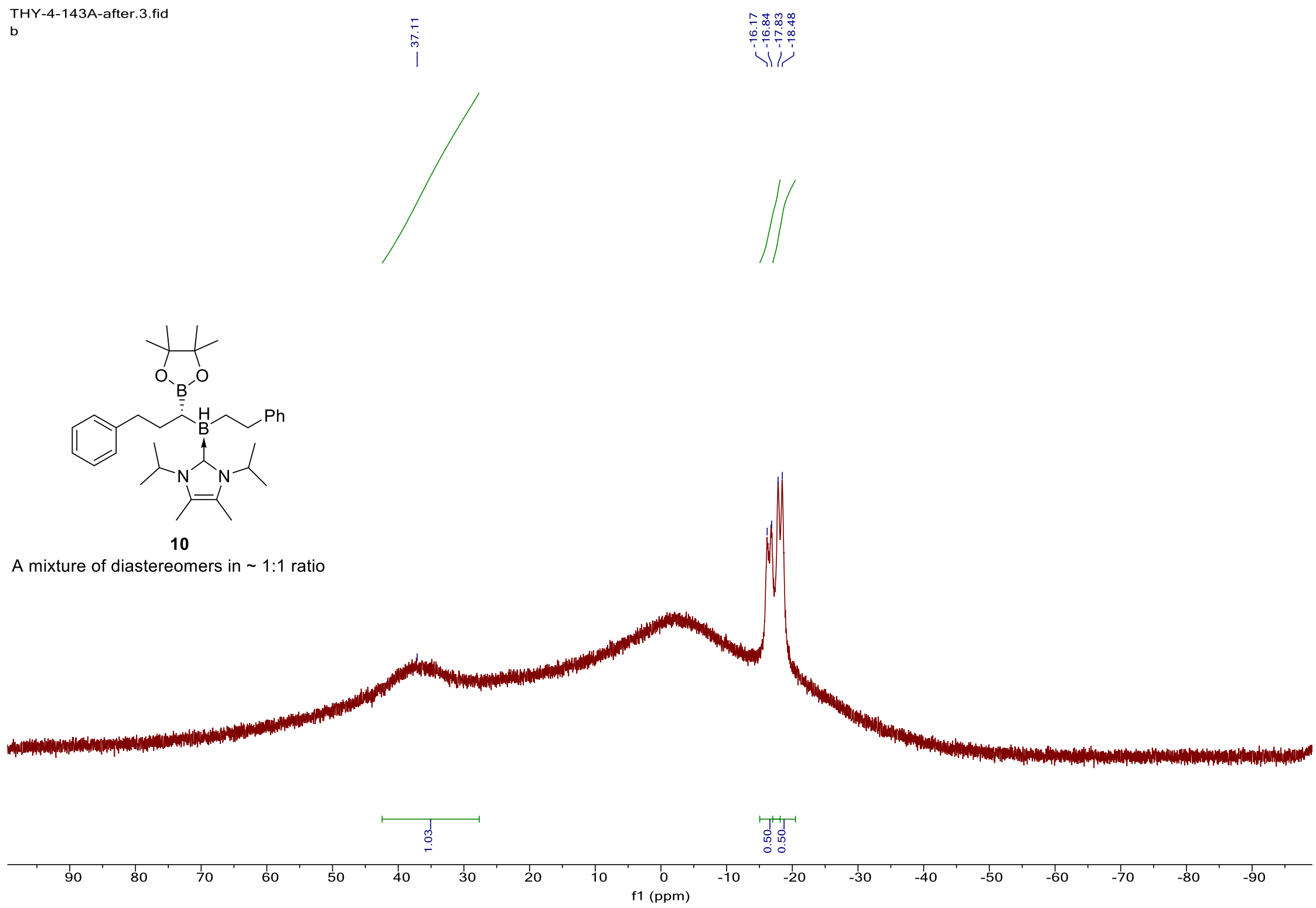

^{11}B NMR (128 MHz, $CDCl_3$)

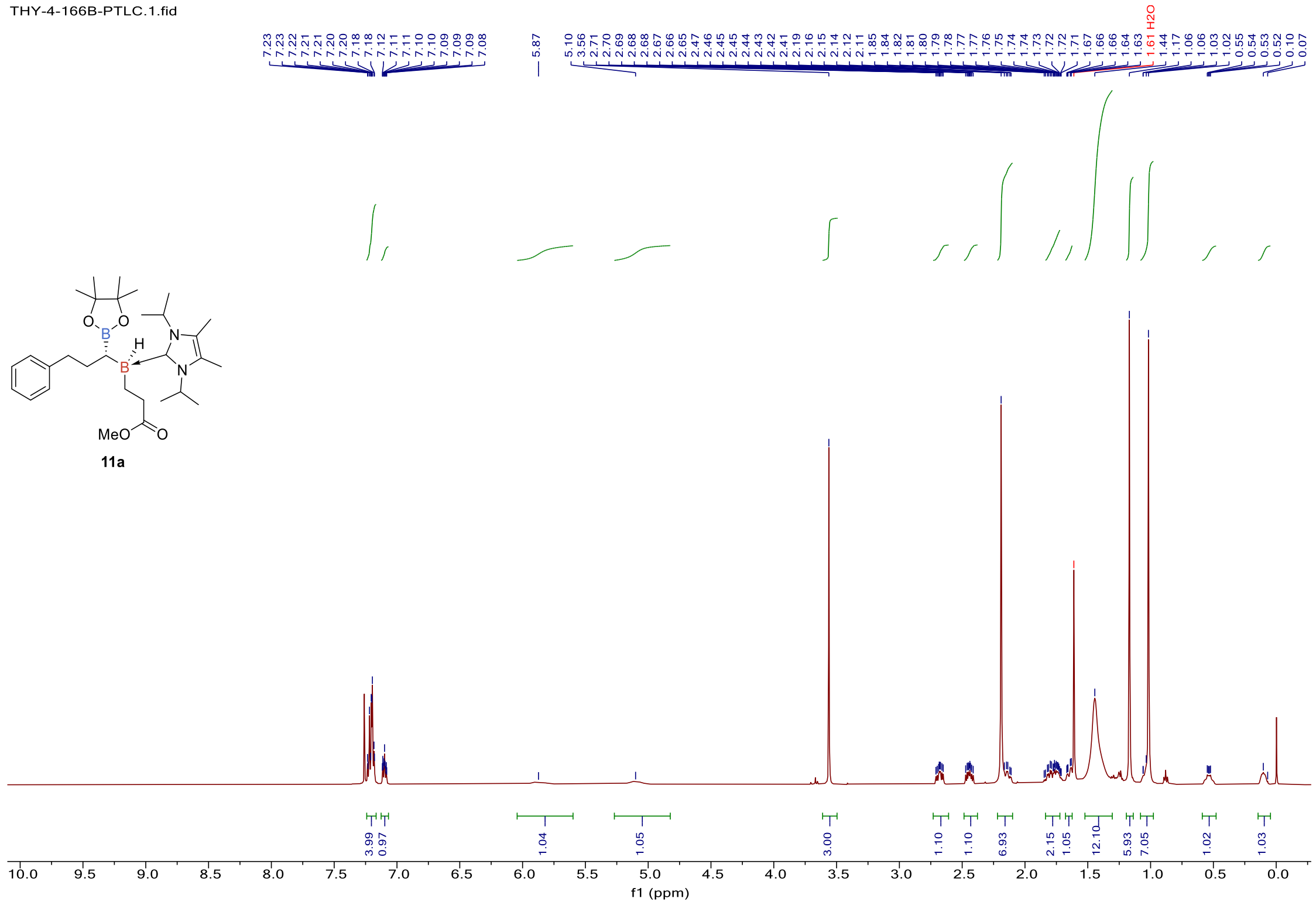

^{1}H NMR (400 MHz, $CDCl_3$)

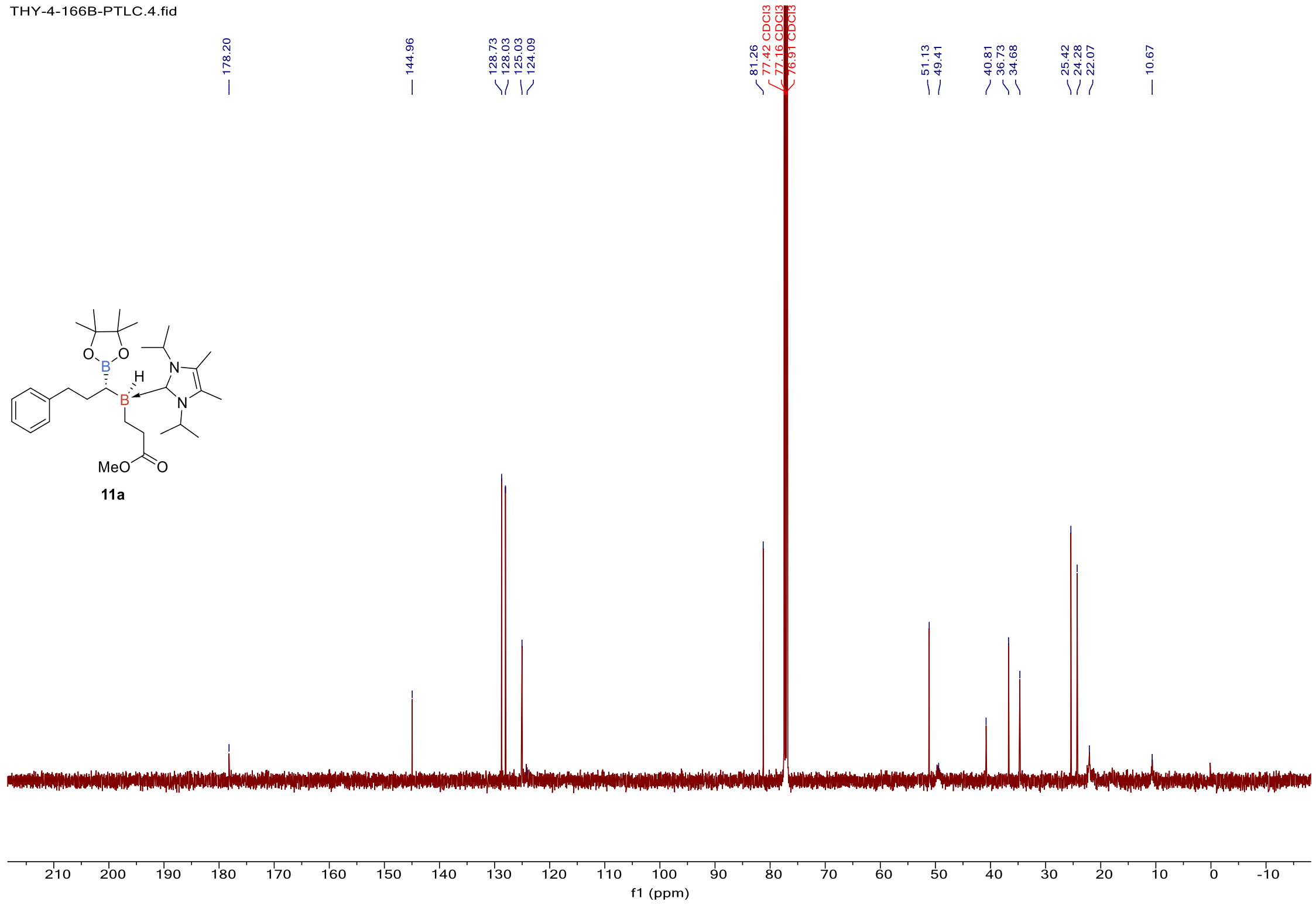

^{13}C NMR (101 MHz, $CDCl_3$)

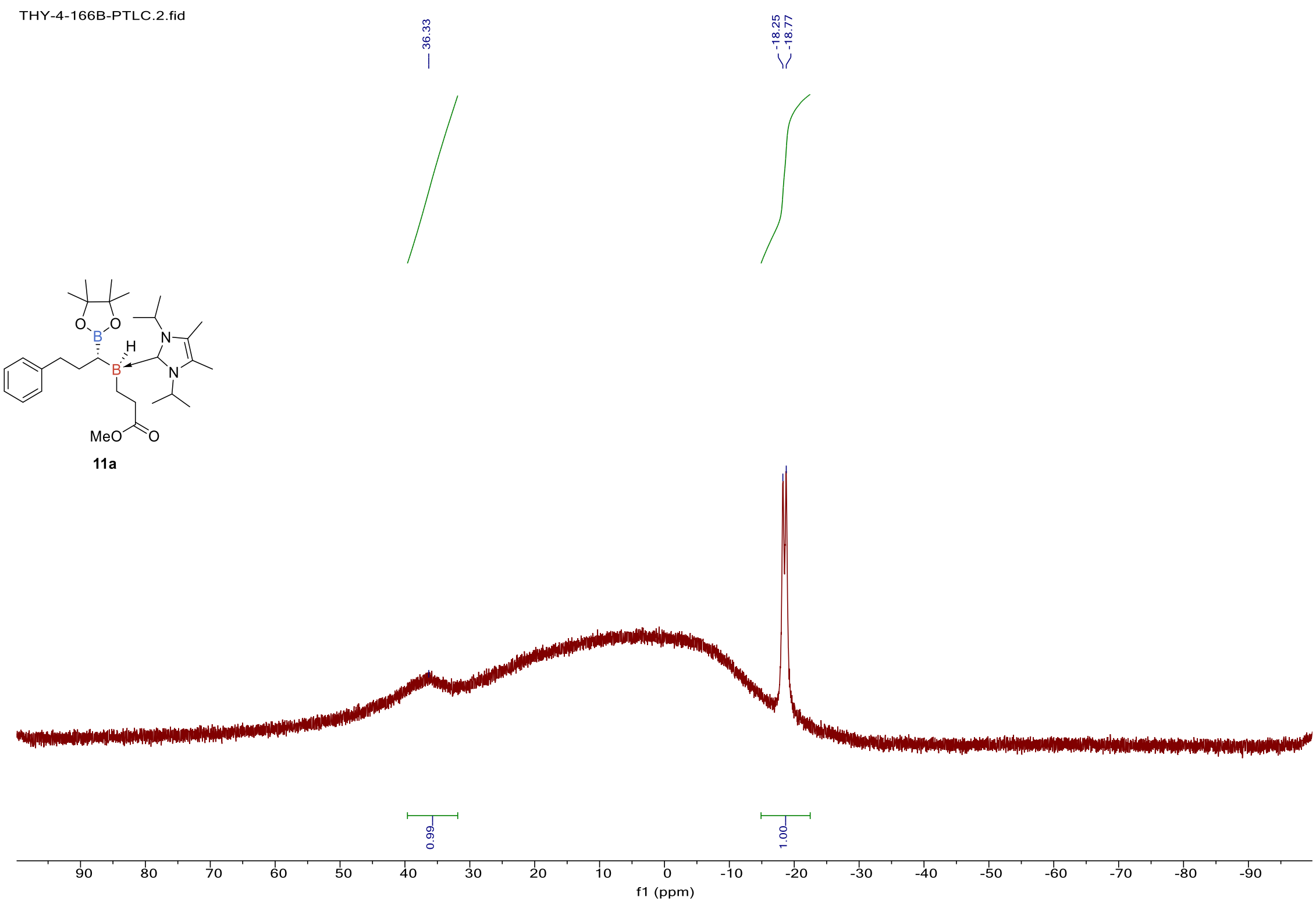

^{11}B NMR (128 MHz, $CDCl_3$)

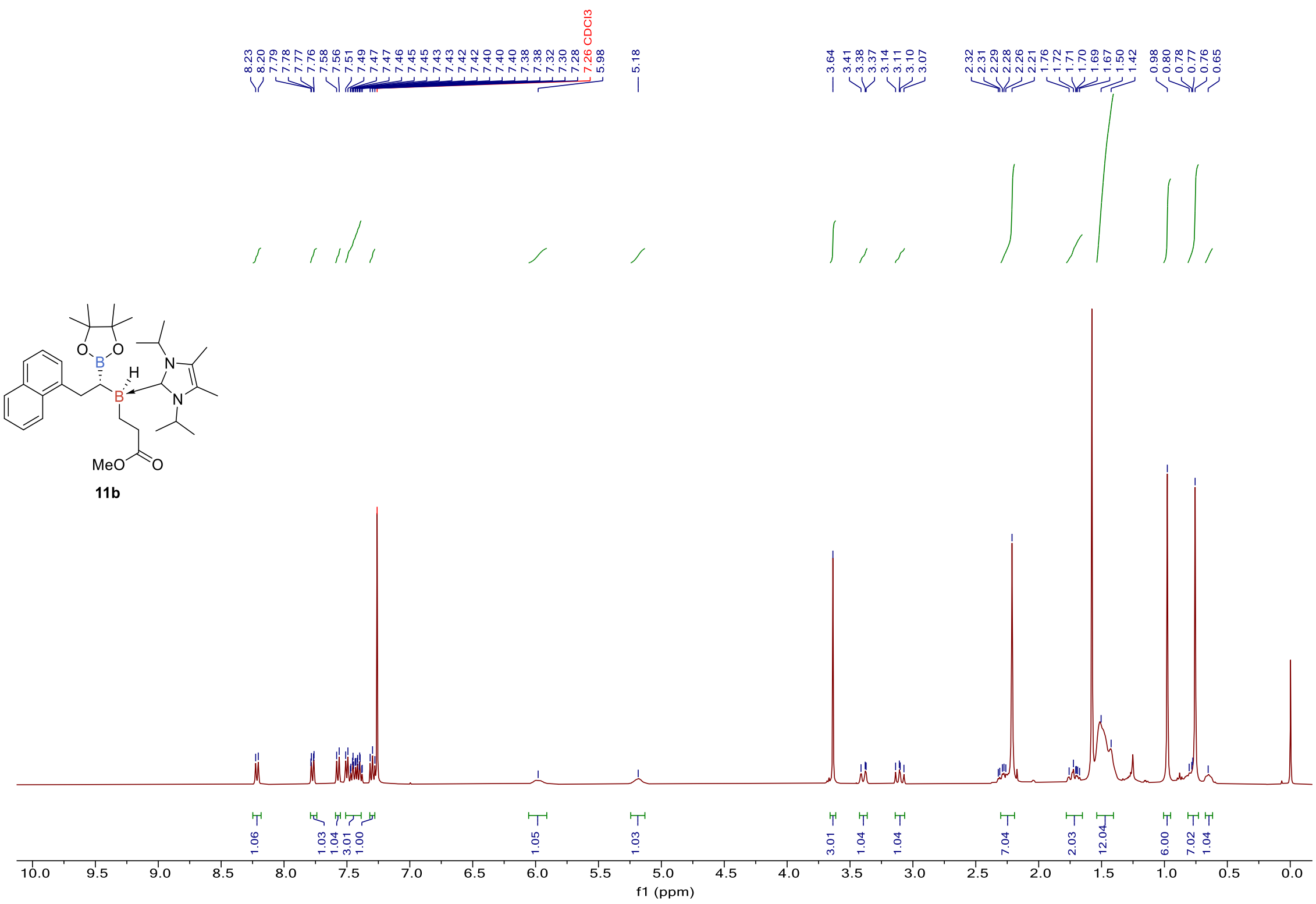

^{1}H NMR (400 MHz, $CDCl_3$)

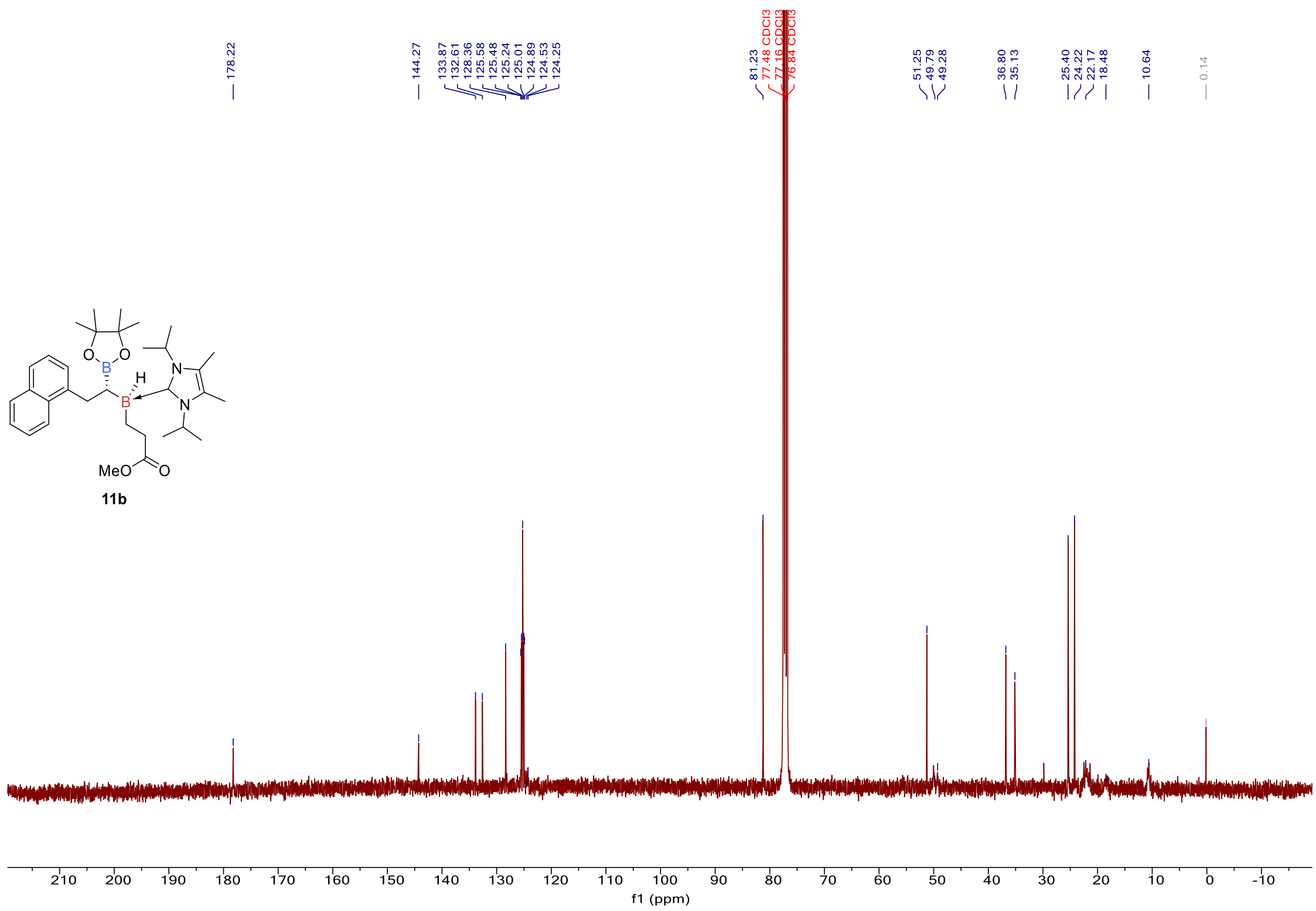

^{13}C NMR (101 MHz, $CDCl_3$)

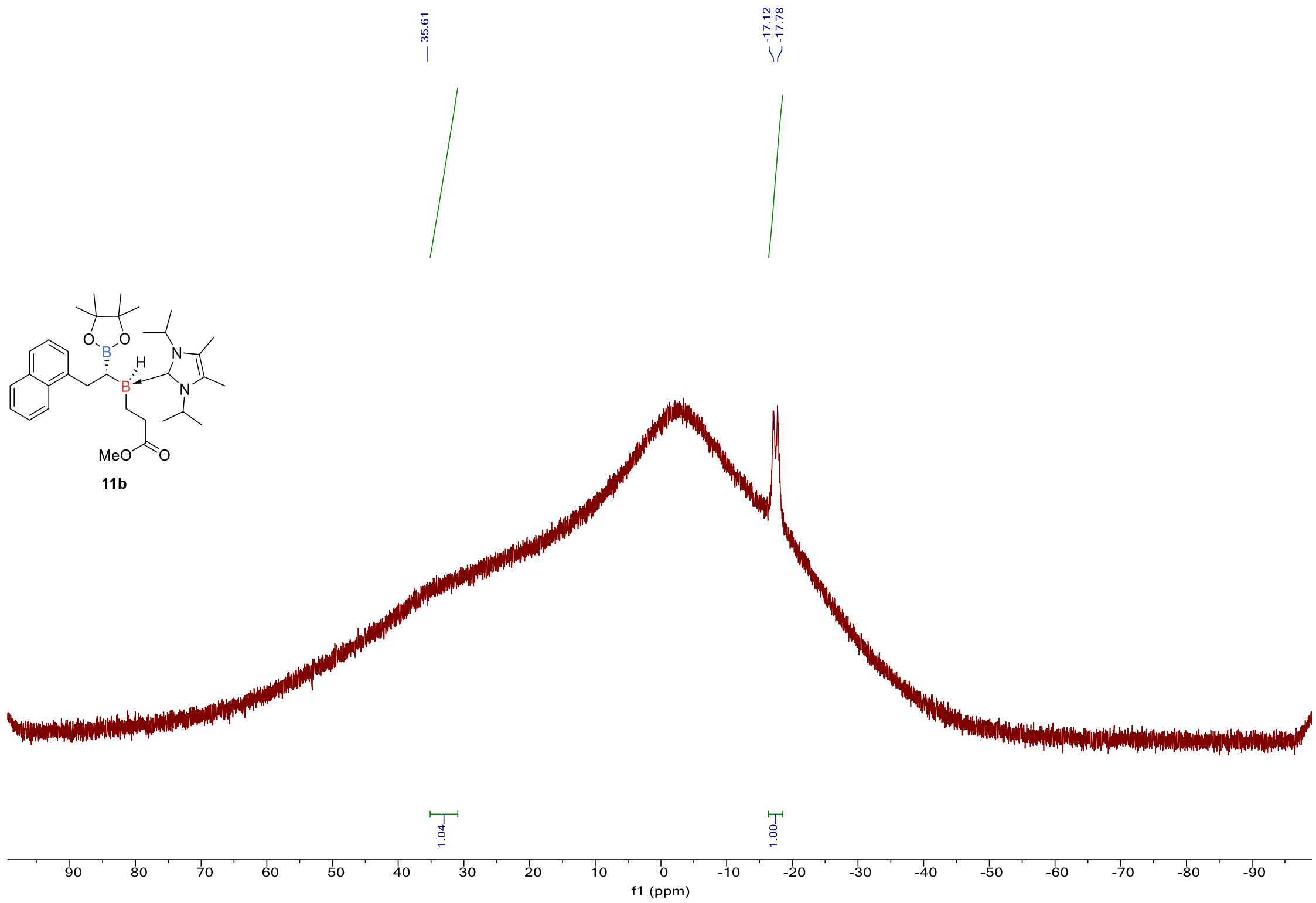

^{11}B NMR (128 MHz, $CDCl_3$)

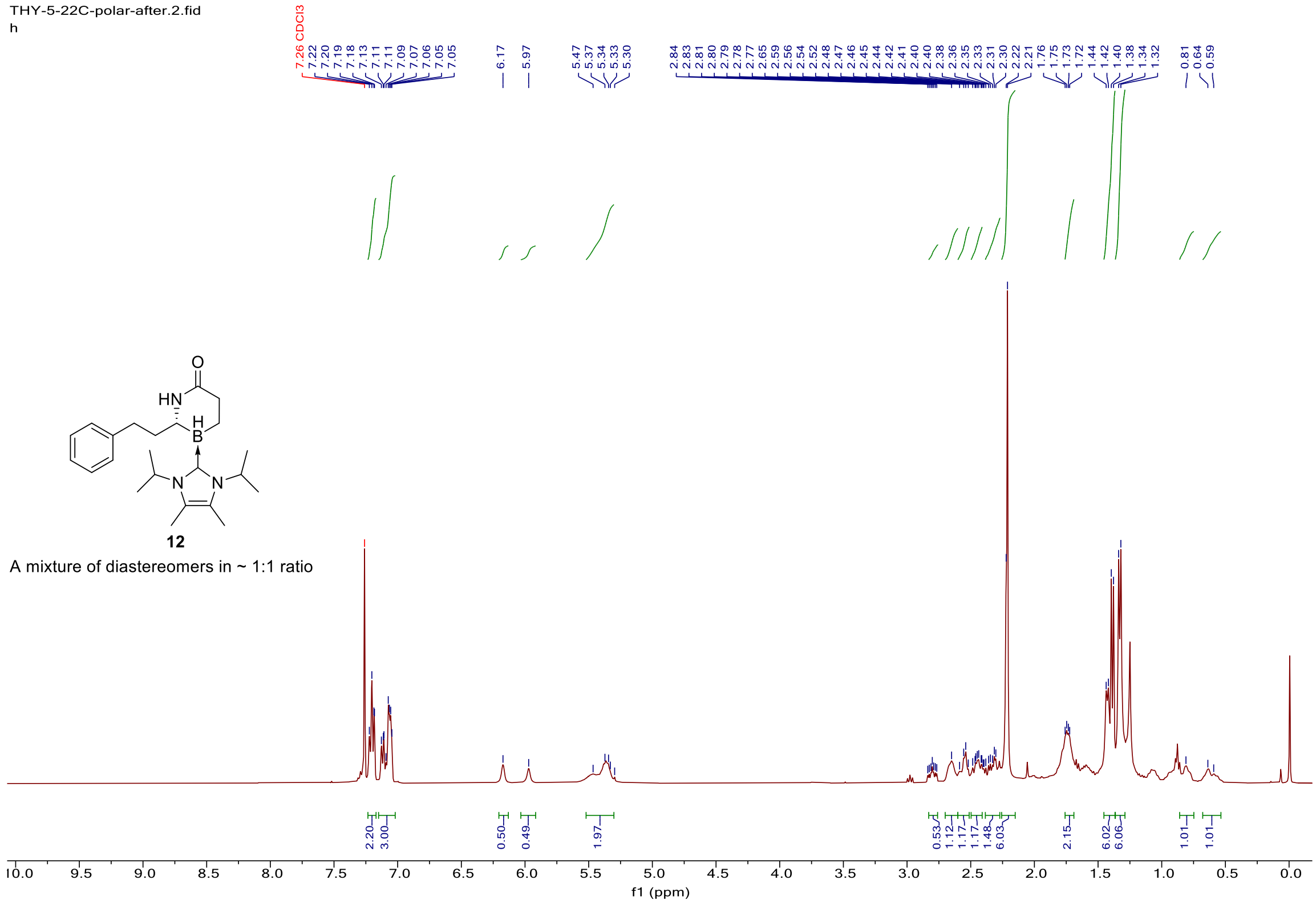

^{1}H NMR (400 MHz, $CDCl_3$)

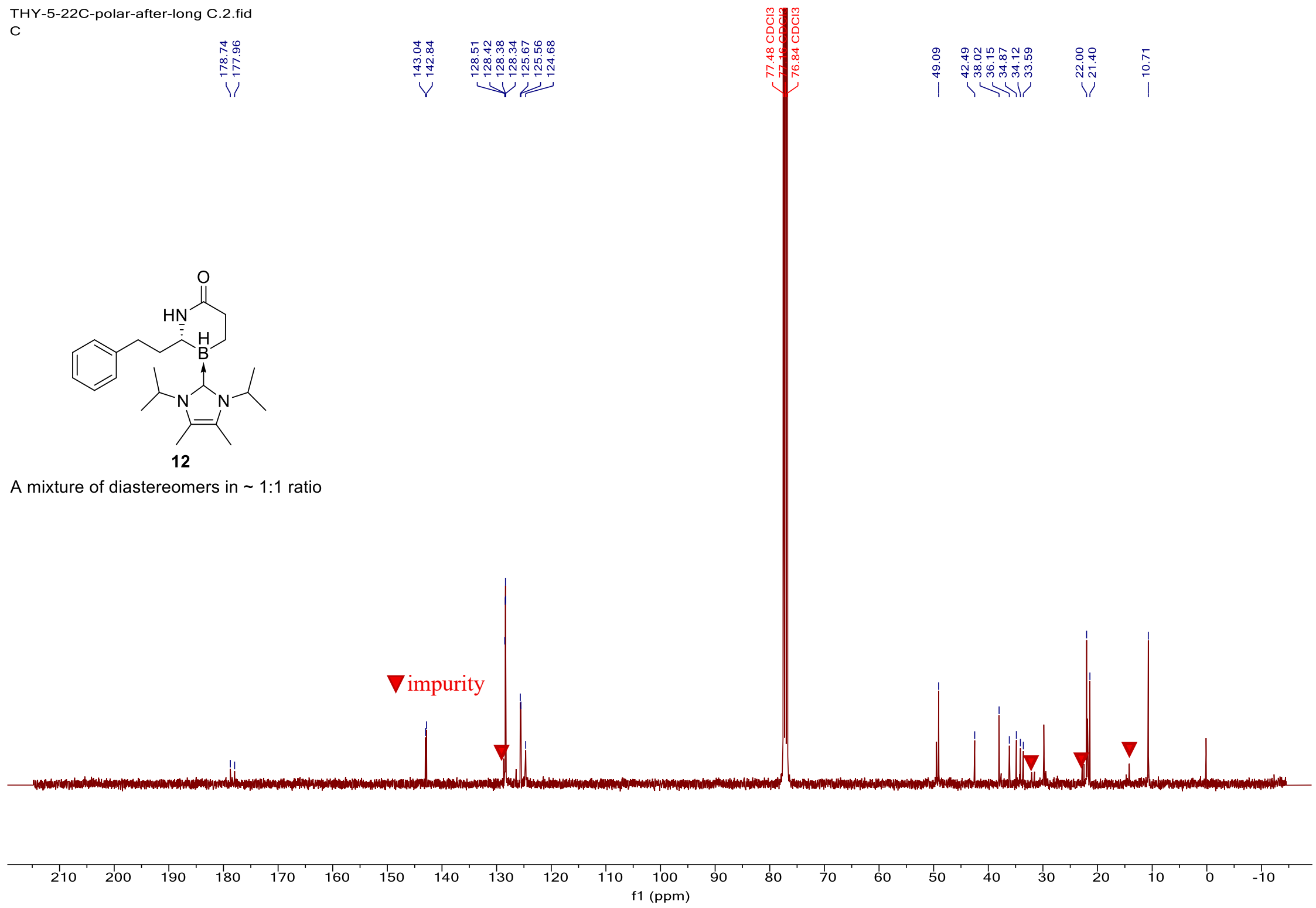

^{13}C NMR (101 MHz, $CDCl_3$)

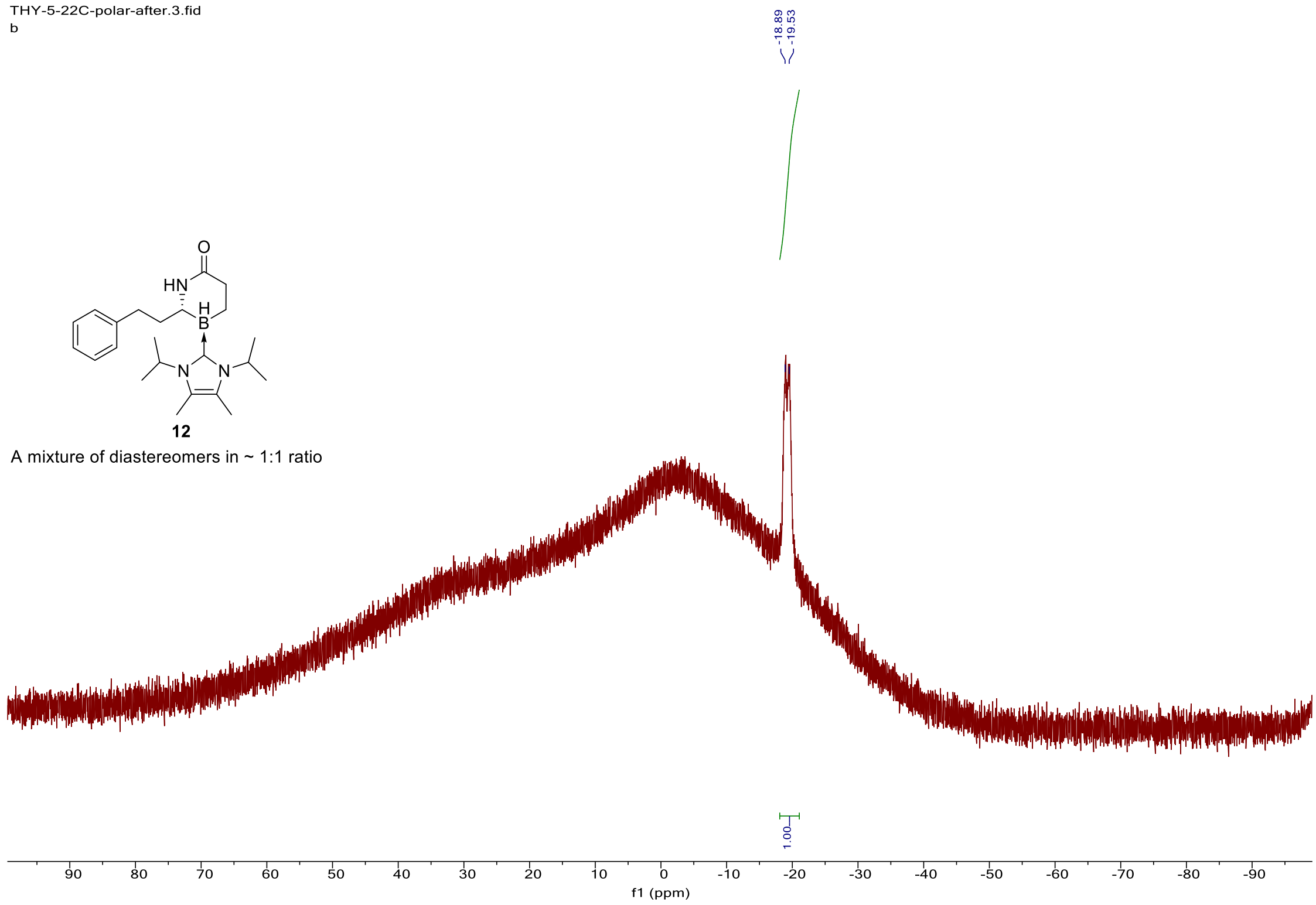

^{11}B NMR (128 MHz, $CDCl_3$)

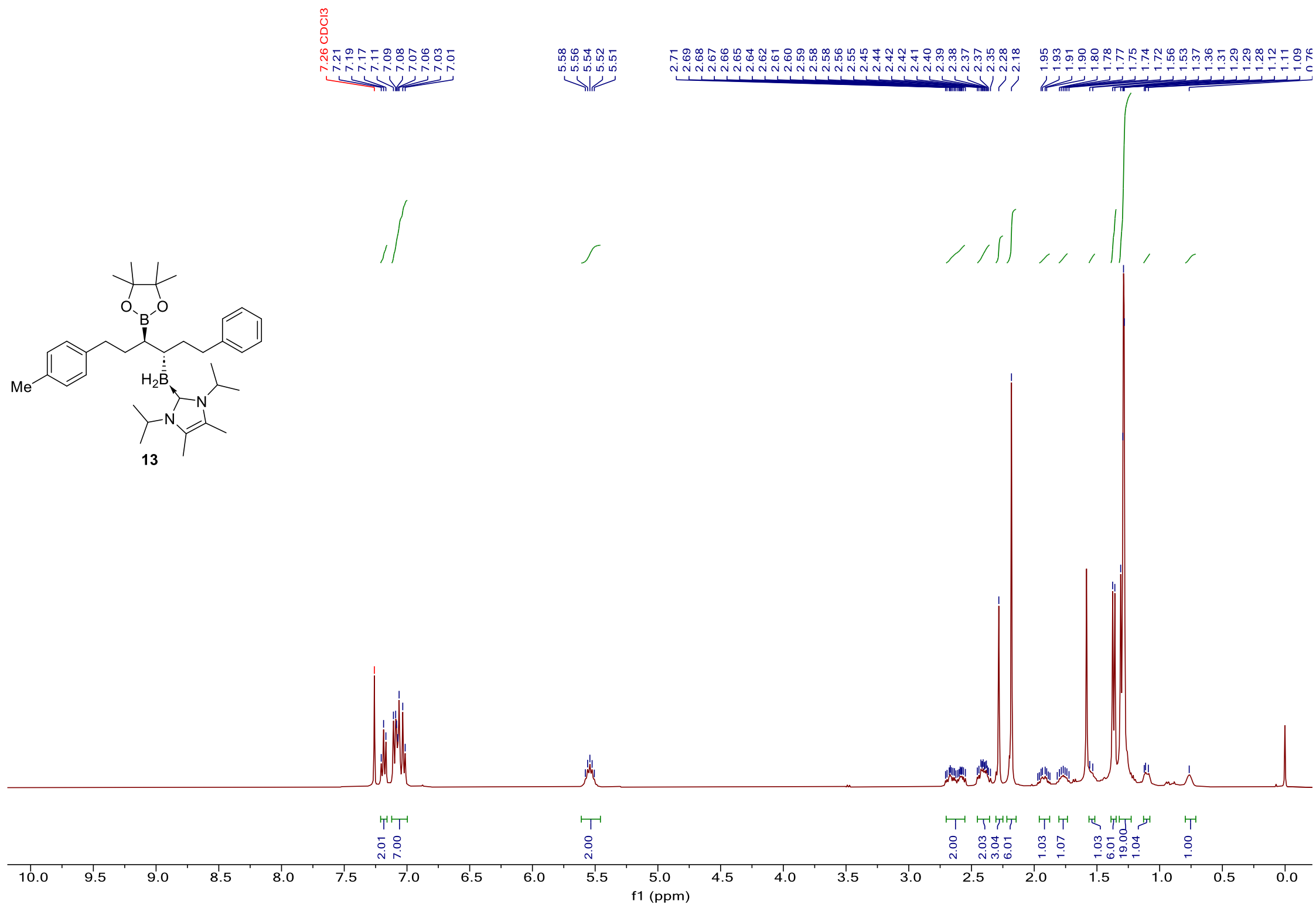

^{1}H NMR (400 MHz, $CDCl_3$)

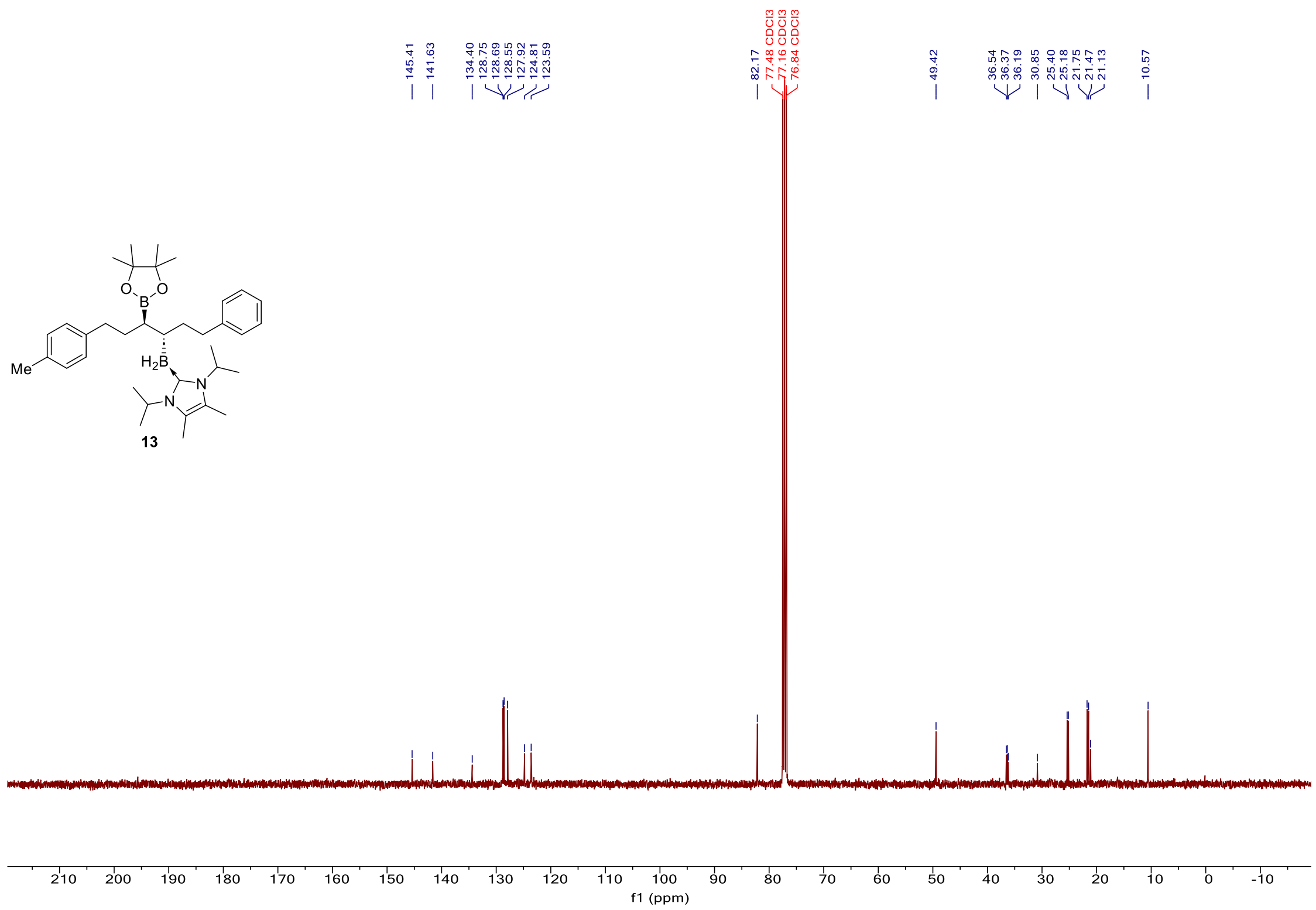

^{13}C NMR (101 MHz, $CDCl_3$)

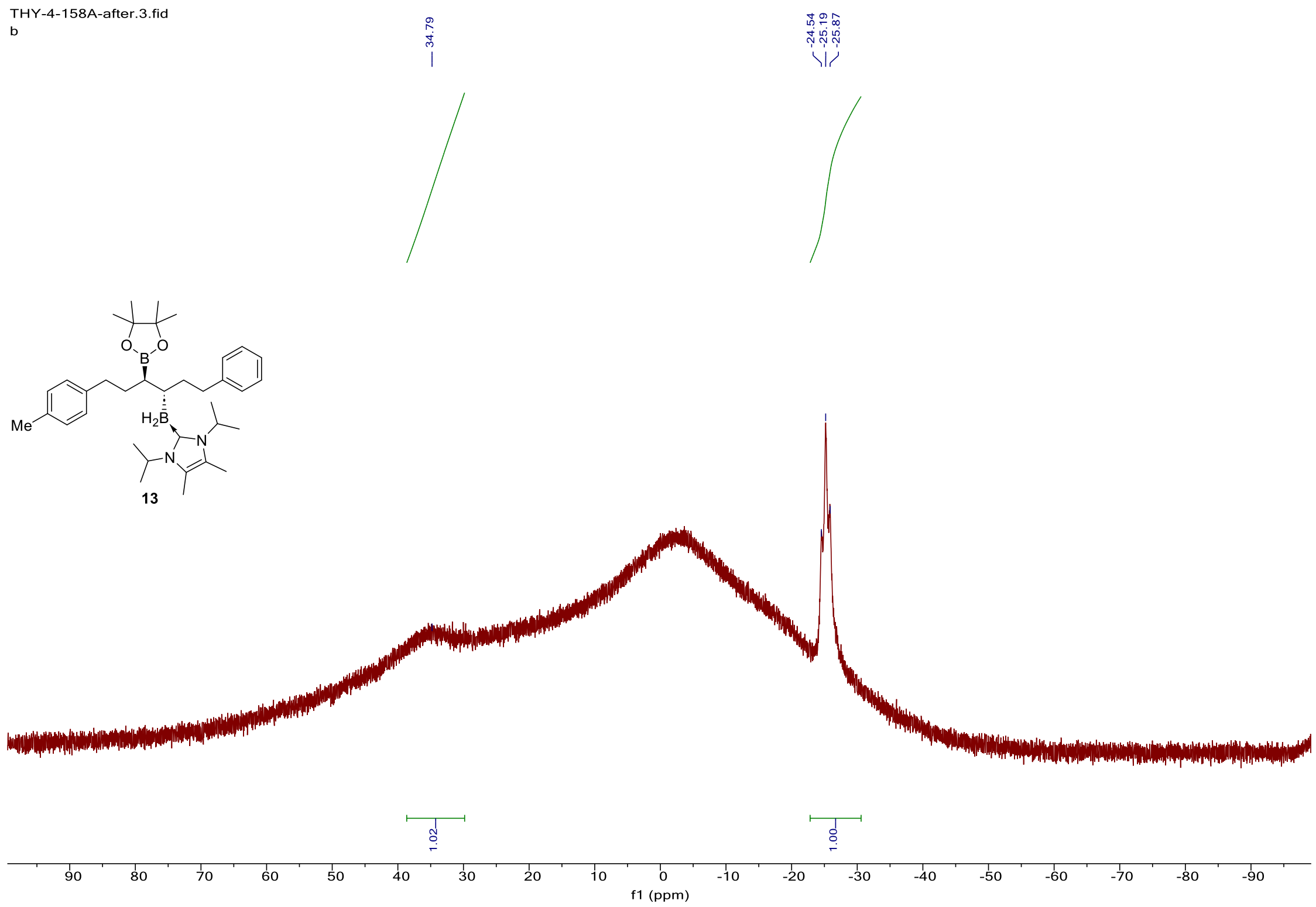

^{11}B NMR (128 MHz, $CDCl_3$)

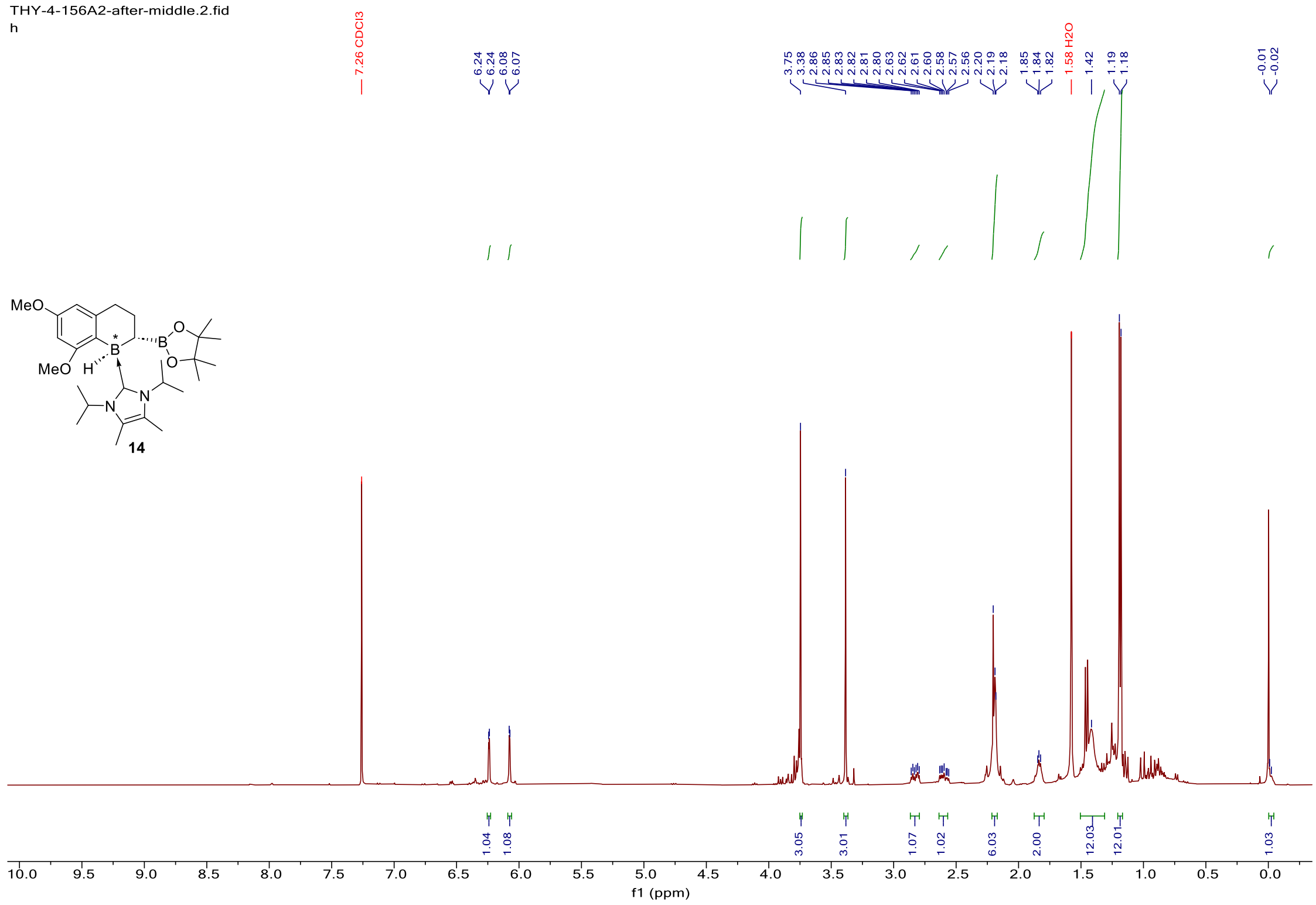

^{1}H NMR (400 MHz, $CDCl_3$)

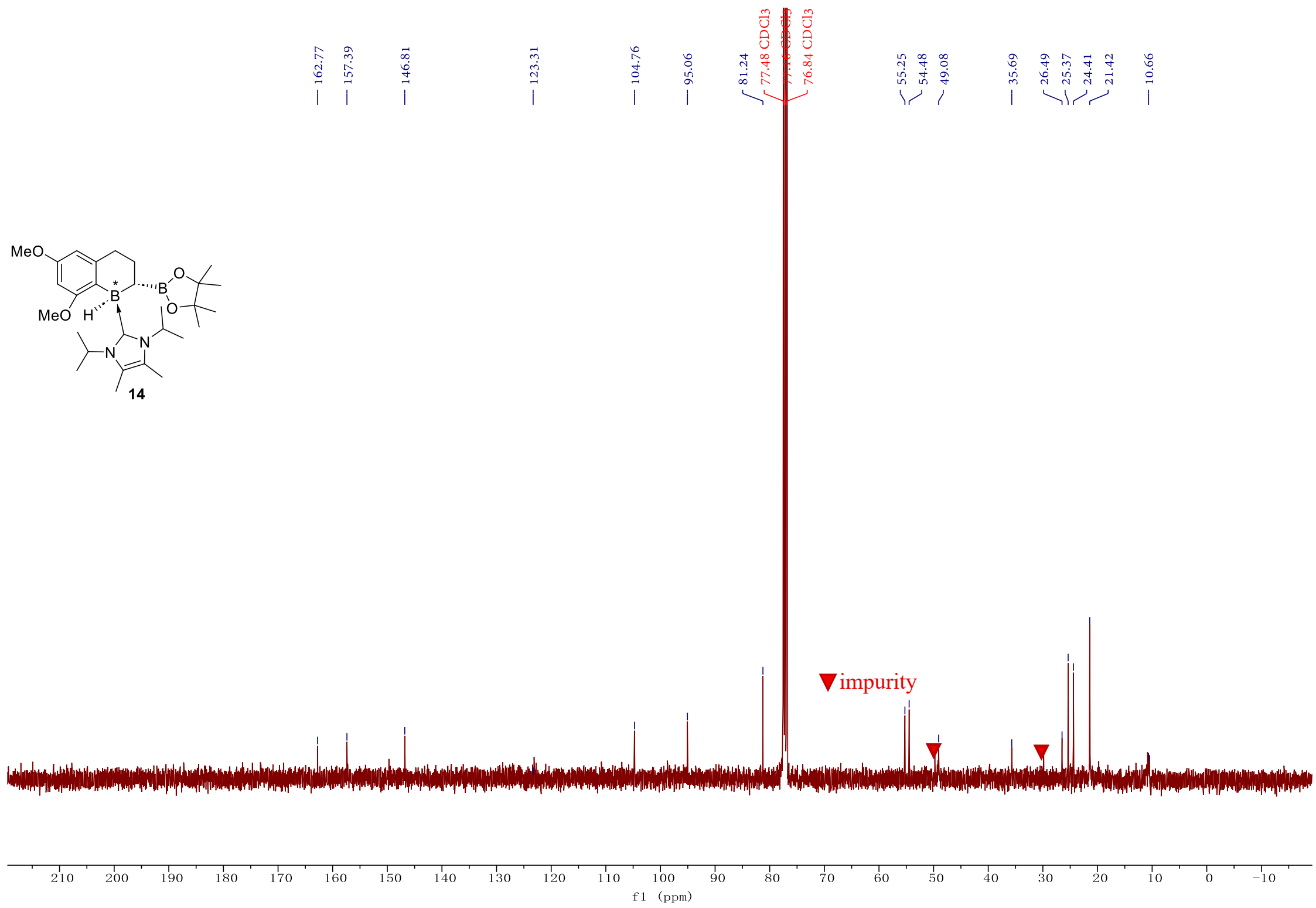

^{13}C NMR (101 MHz, $CDCl_3$)

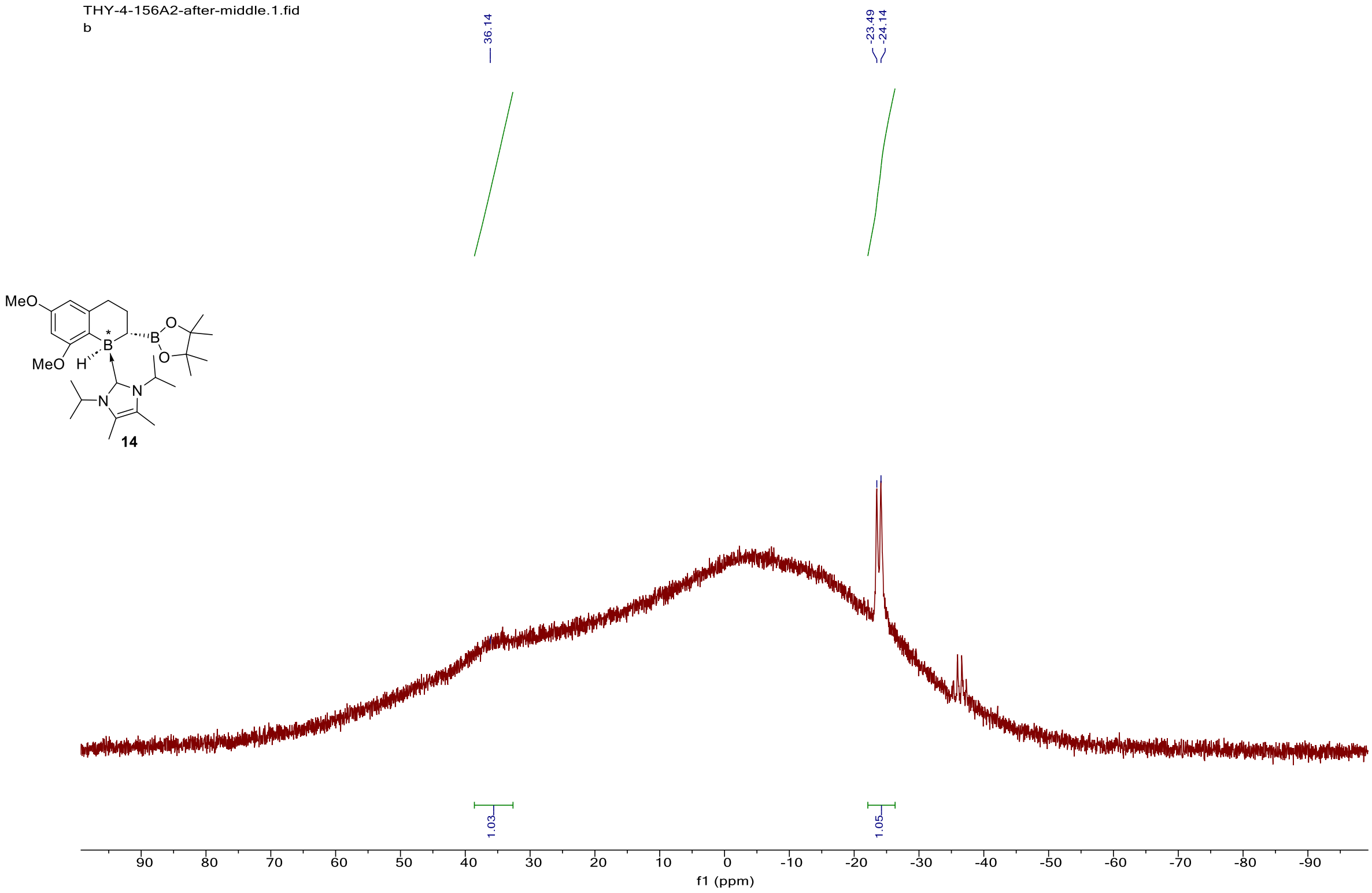

^{11}B NMR (128 MHz, $CDCl_3$)

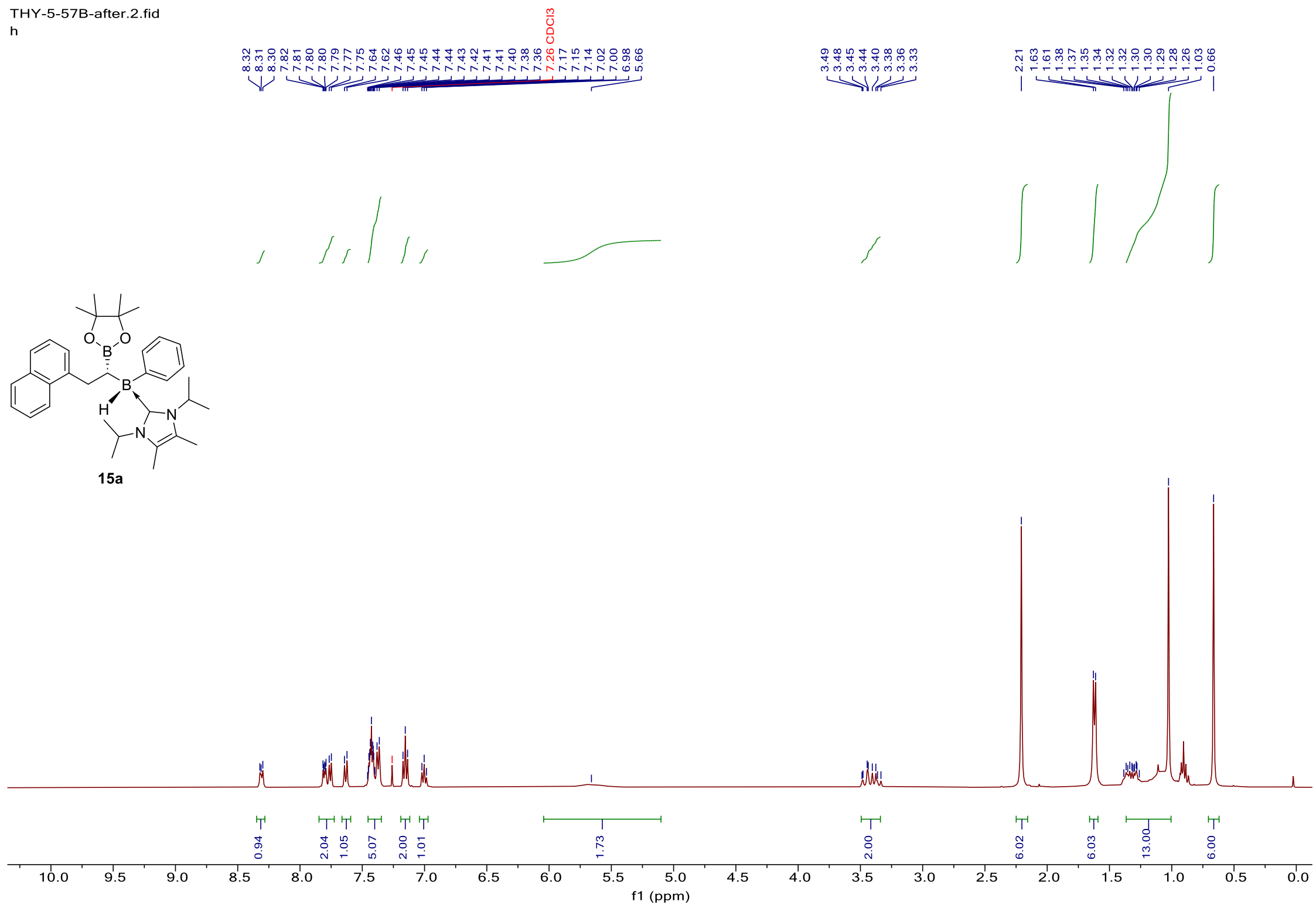

^{1}H NMR (400 MHz, $CDCl_3$)

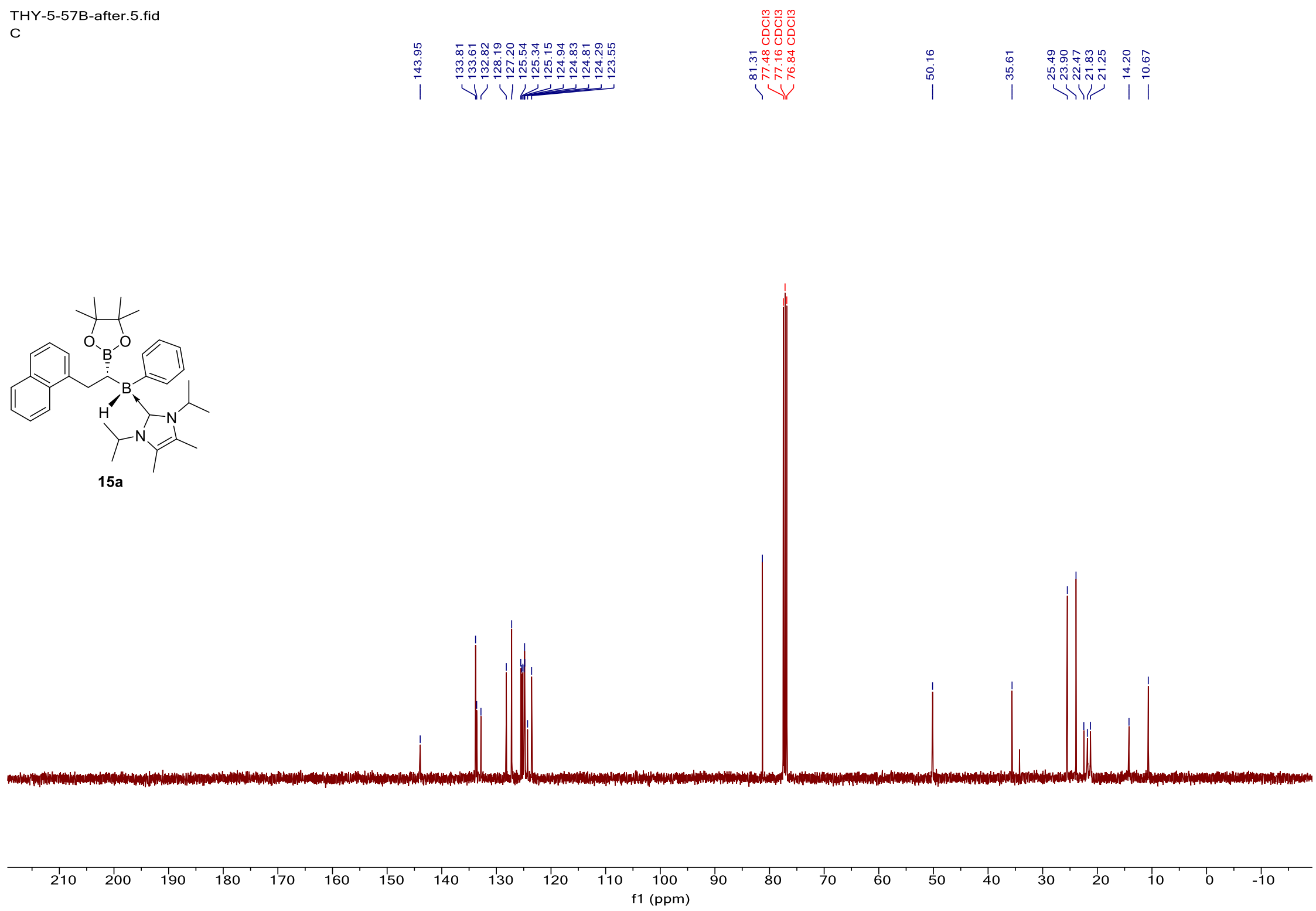

^{13}C NMR (101 MHz, $CDCl_3$)

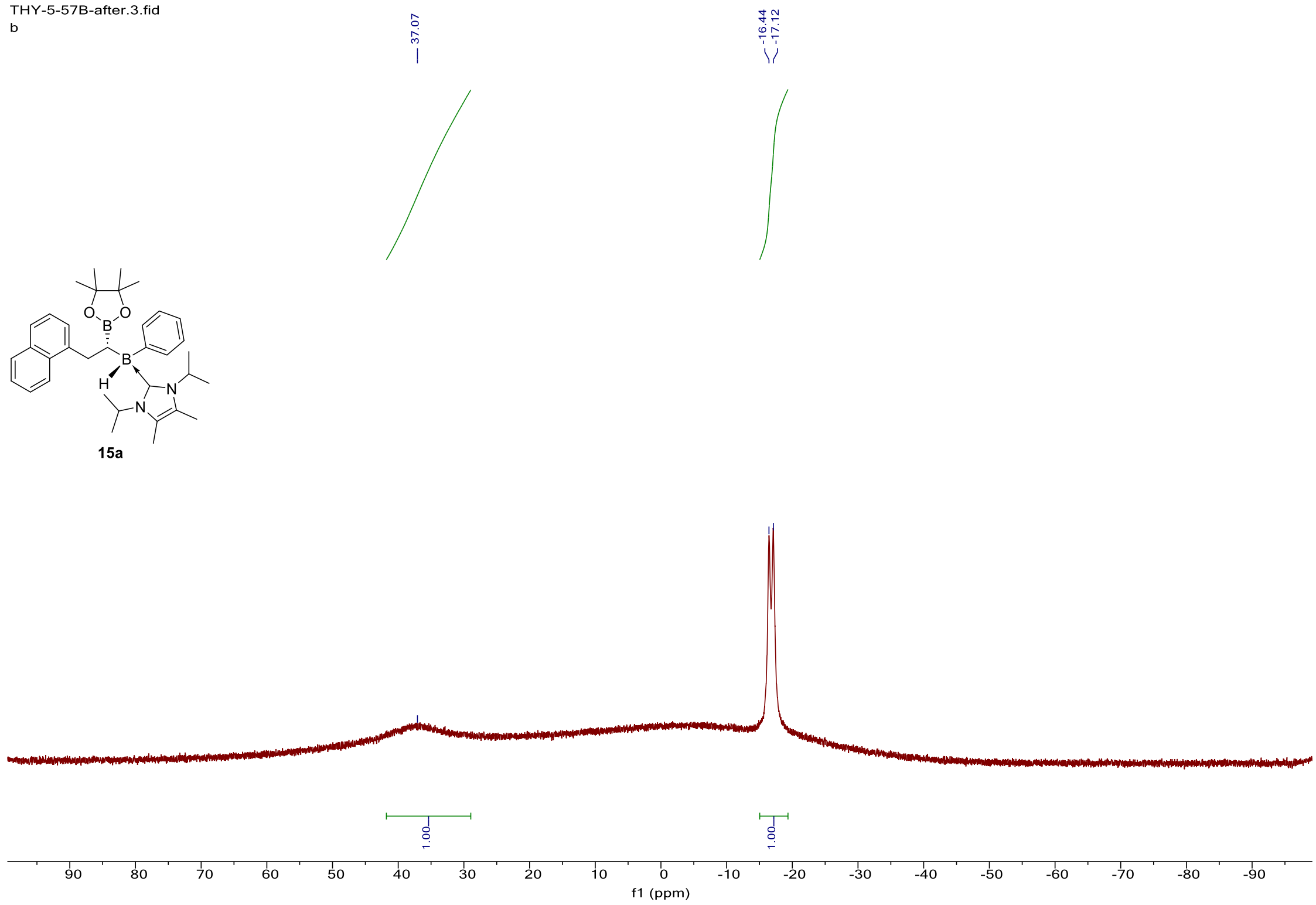

^{11}B NMR (128 MHz, $CDCl_3$)

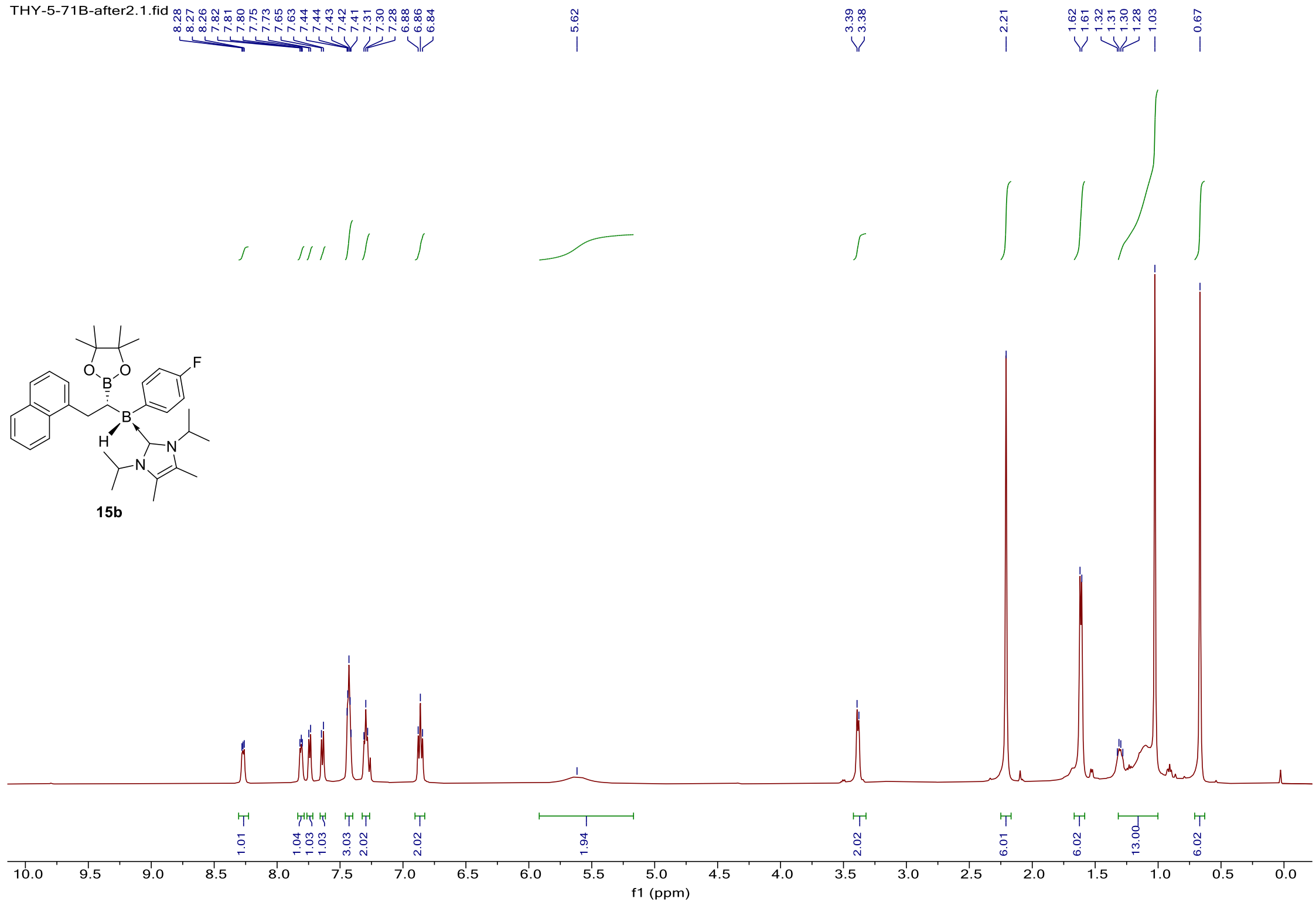

^{1}H NMR (500 MHz, $CDCl_3$)

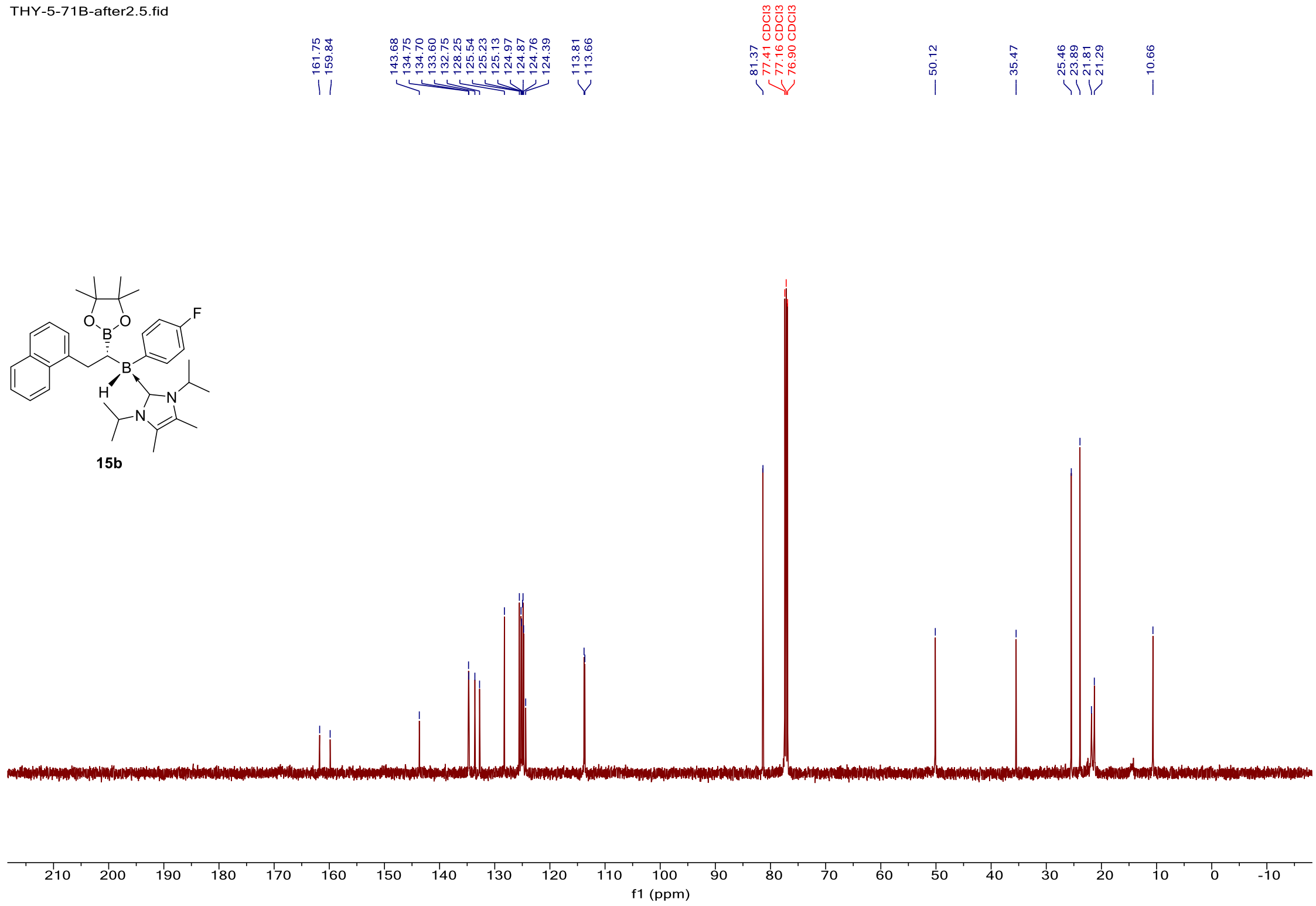

^{13}C NMR (128 MHz, $CDCl_3$)

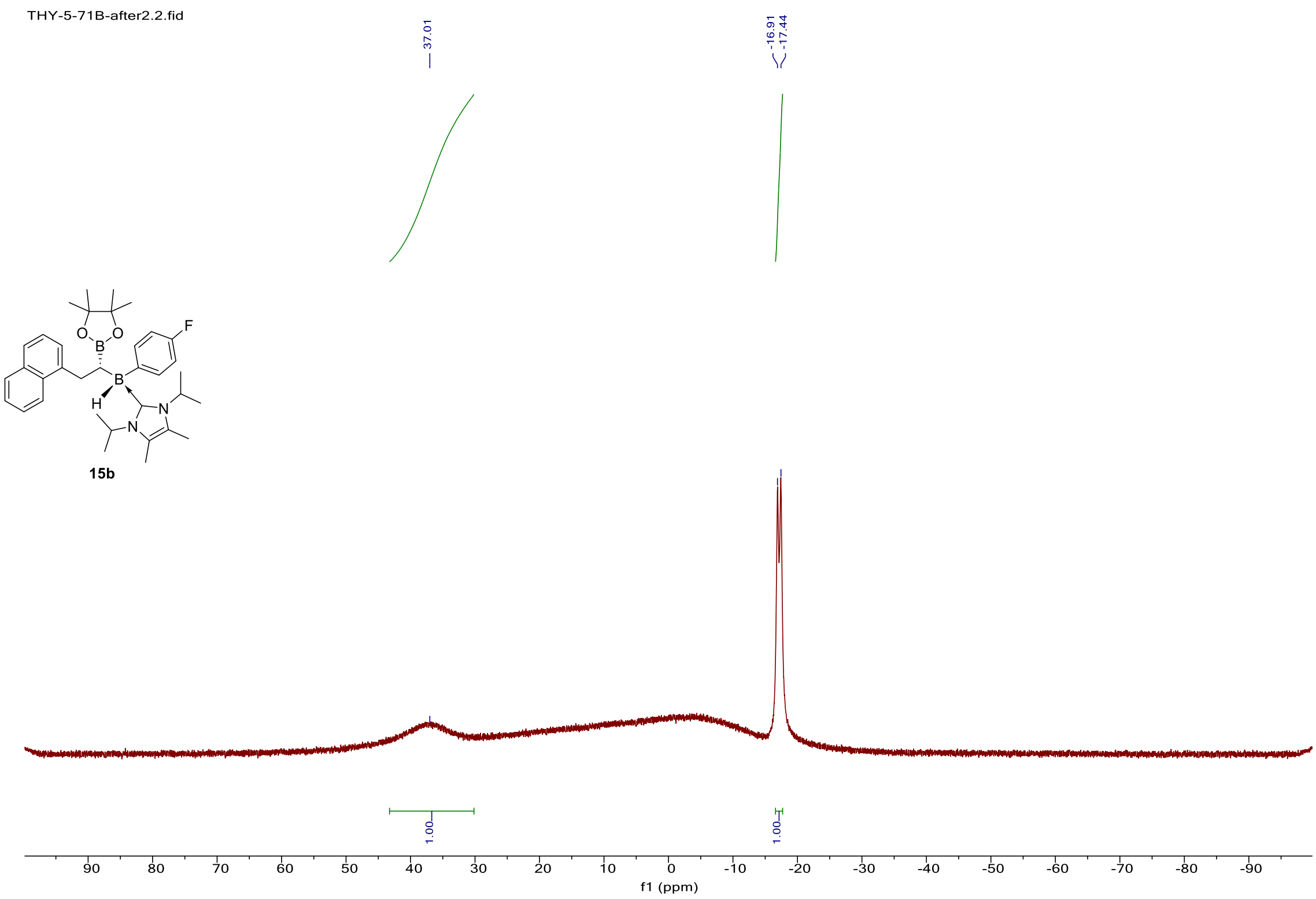

^{11}B NMR (160 MHz, $CDCl_3$)

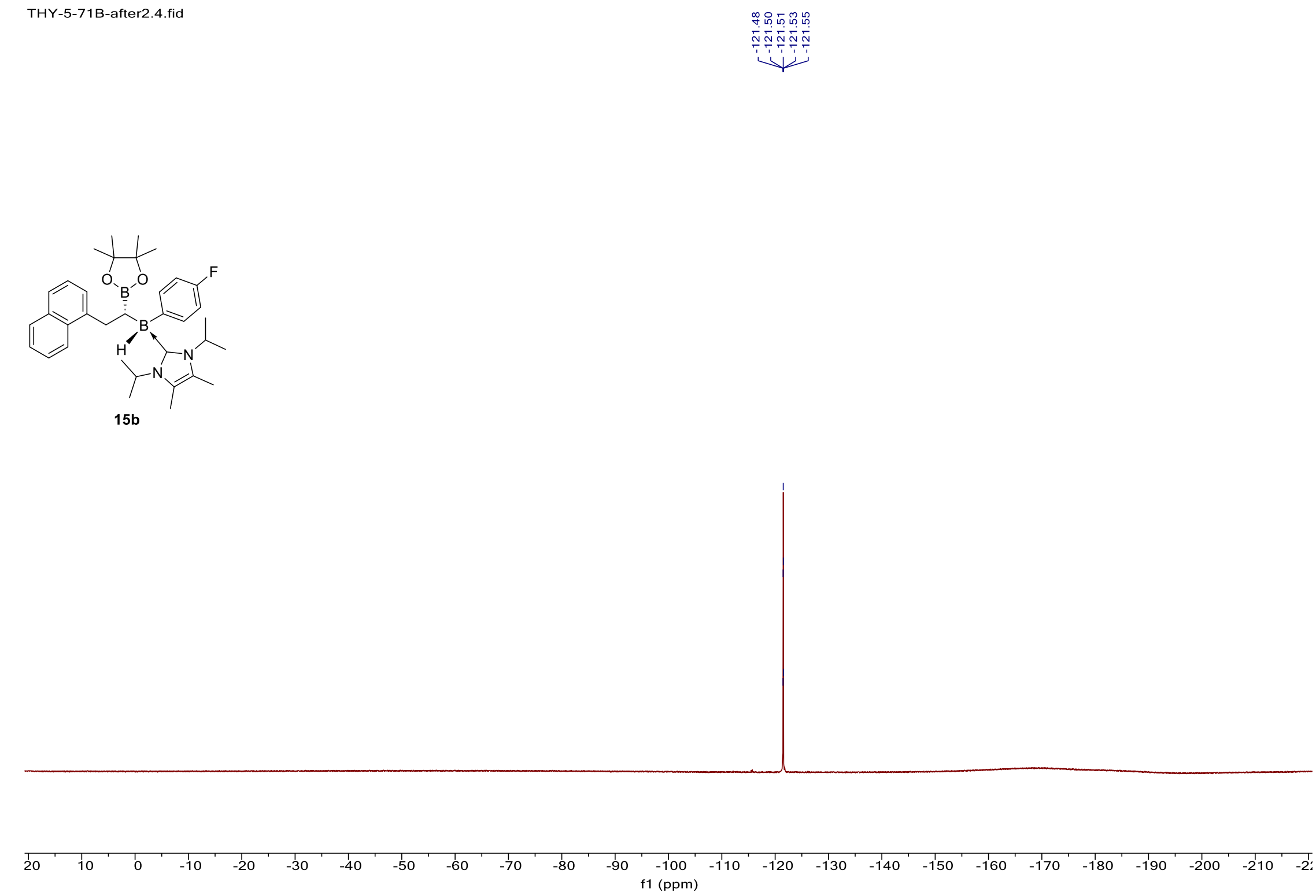

^{19}F NMR (471 MHz, $CDCl_3$)

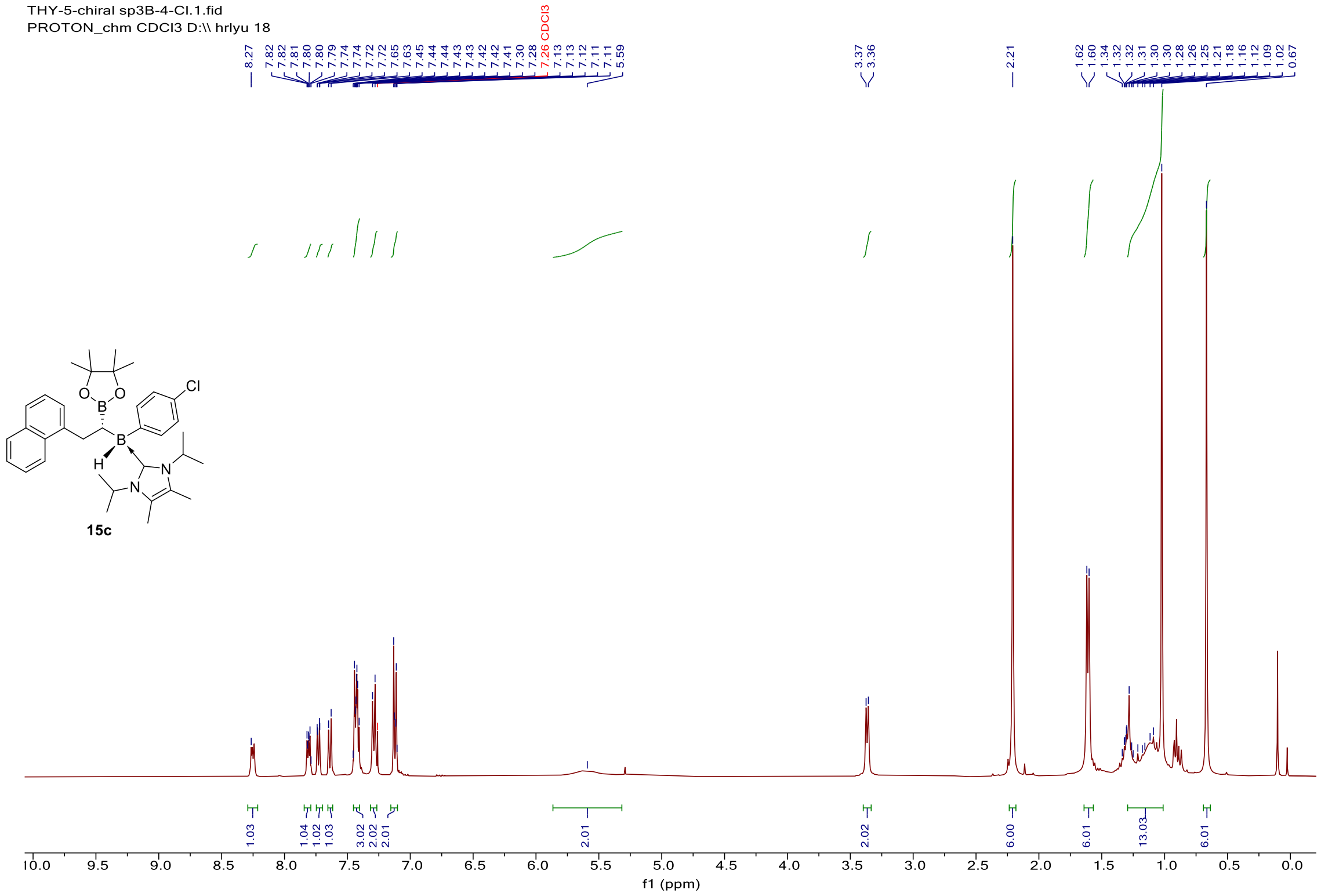

^{1}H NMR (400 MHz, $CDCl_3$)

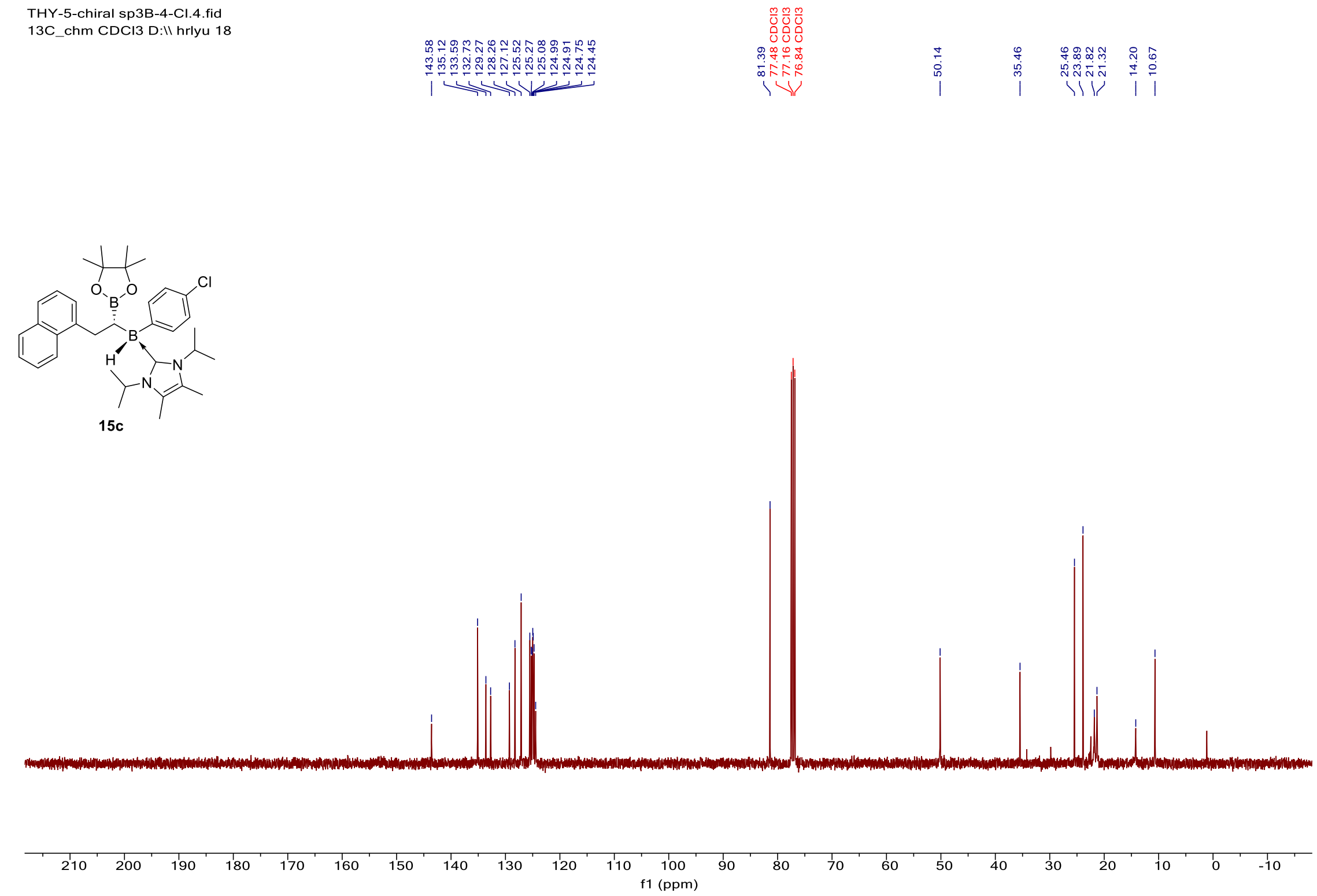

^{13}C NMR (101 MHz, $CDCl_3$)

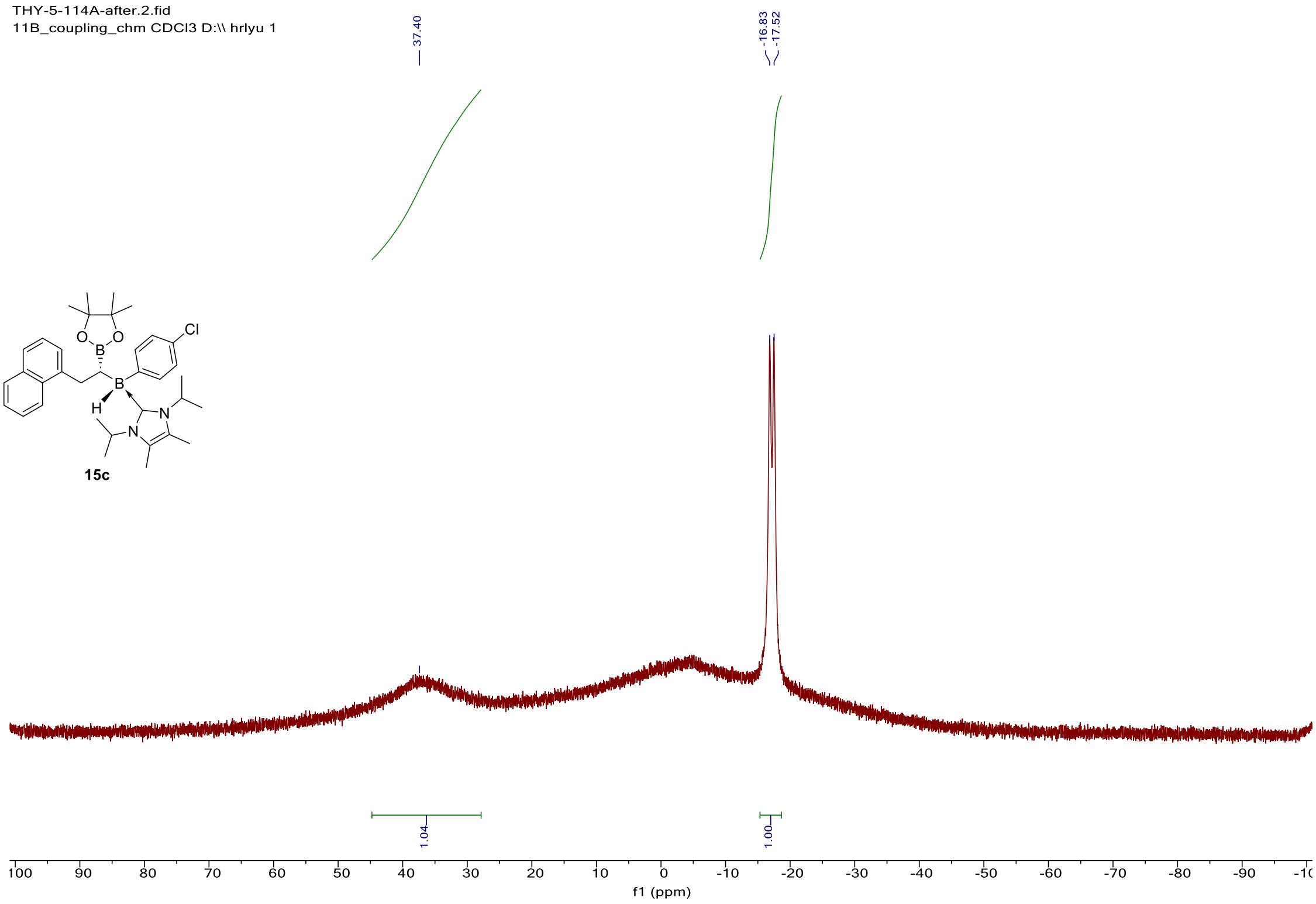

^{11}B NMR (128 MHz, $CDCl_3$)

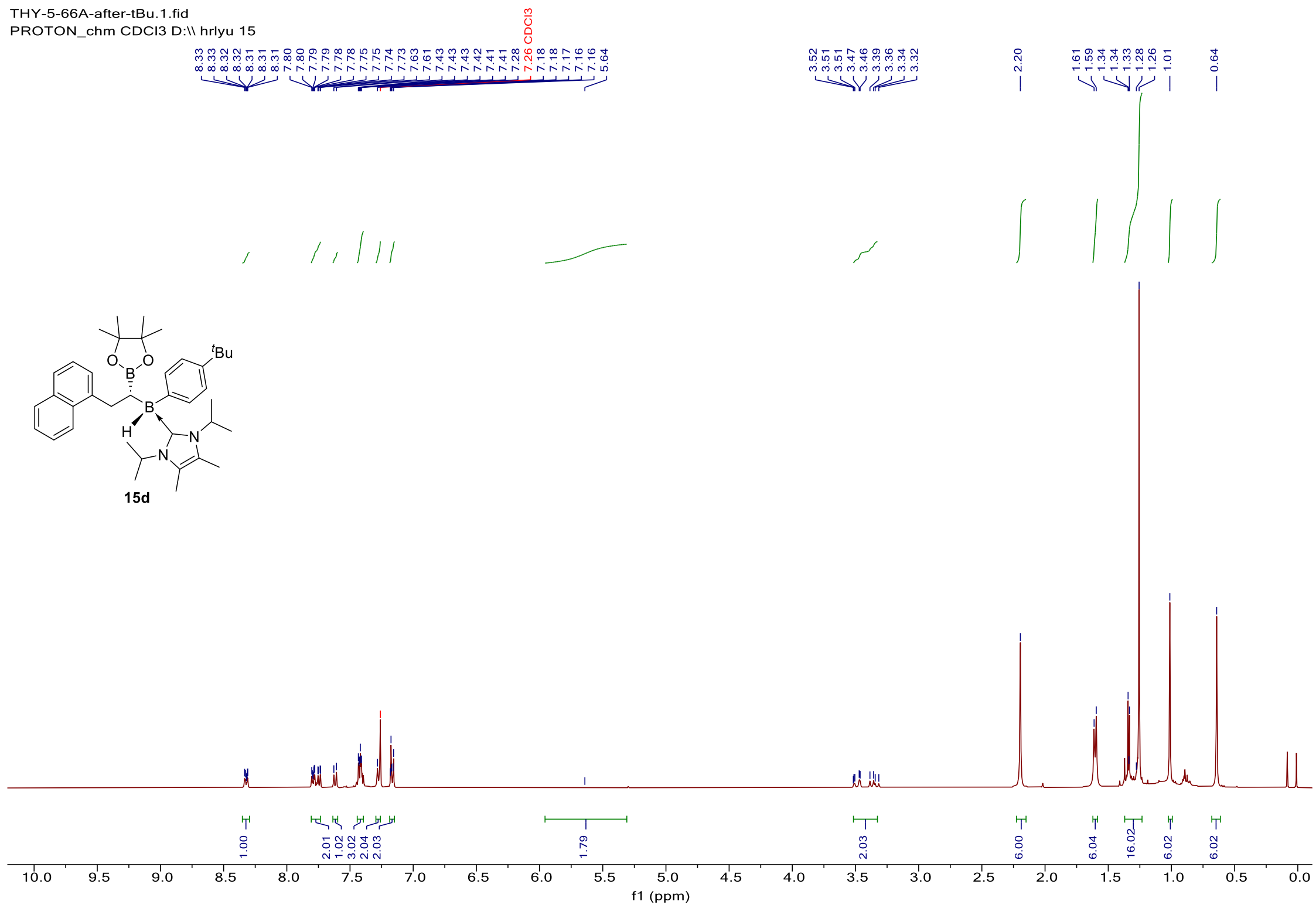

^{1}H NMR (400 MHz, $CDCl_3$)

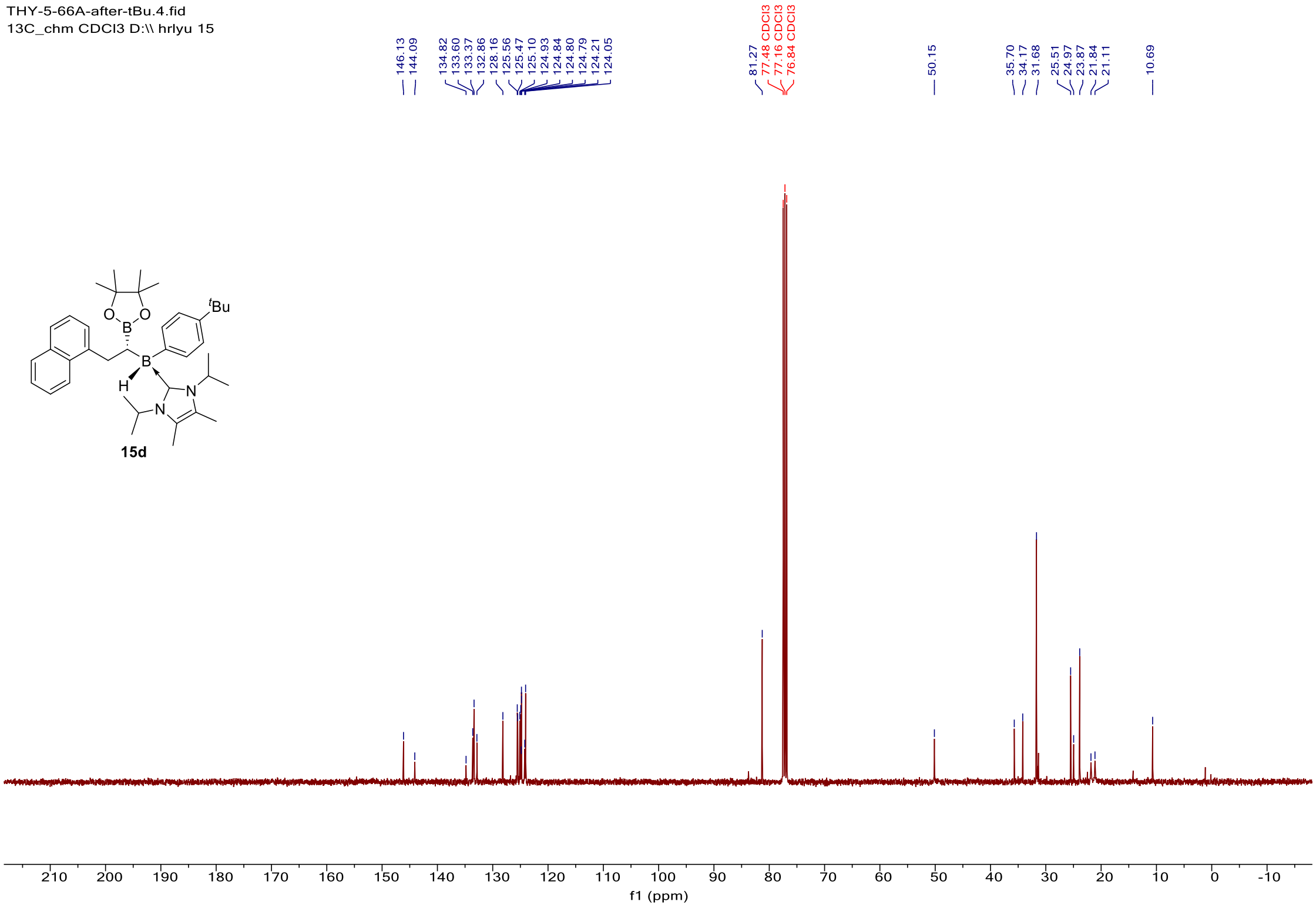

^{13}C NMR (101 MHz, $CDCl_3$)

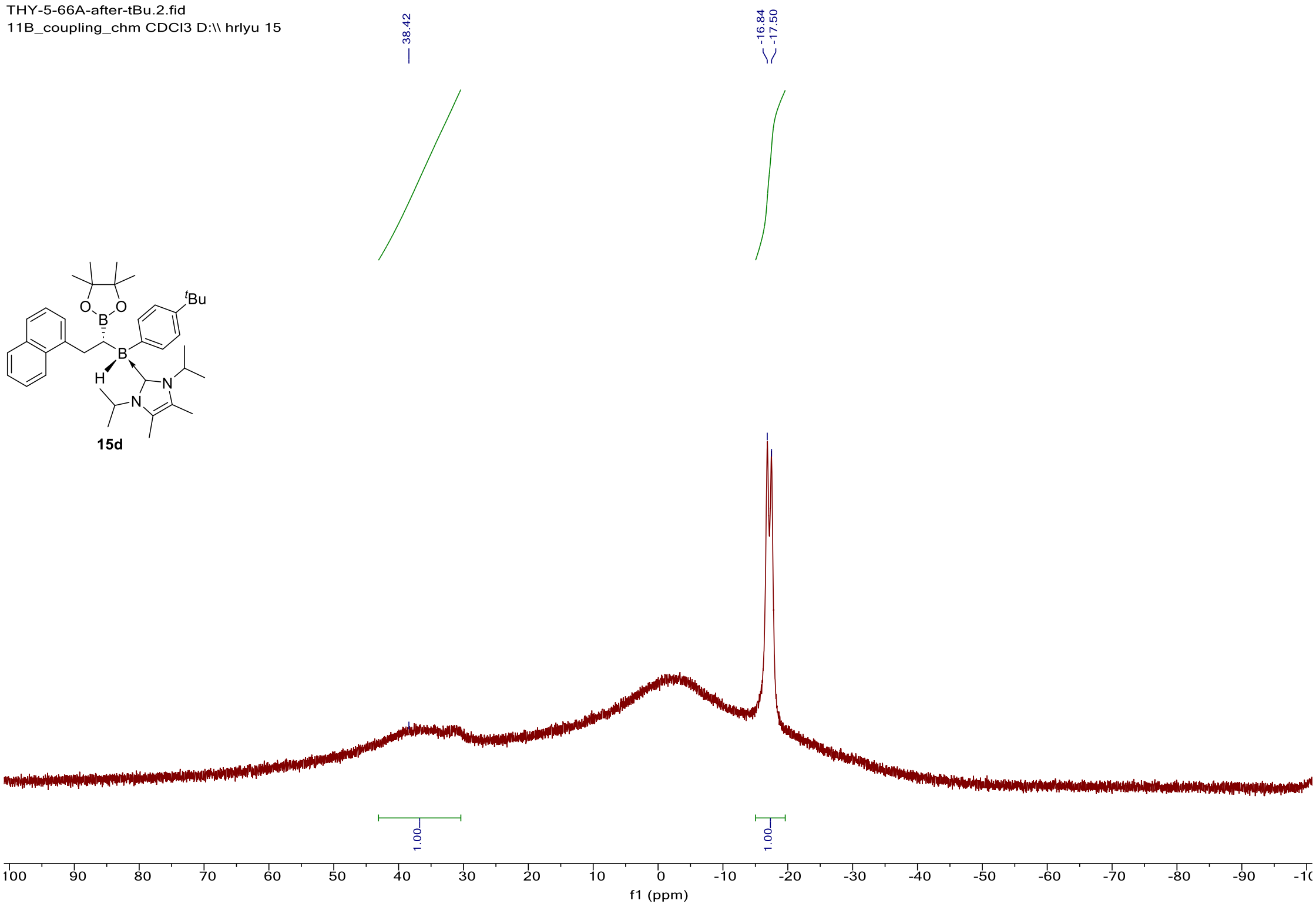

^{11}B NMR (128 MHz, $CDCl_3$)

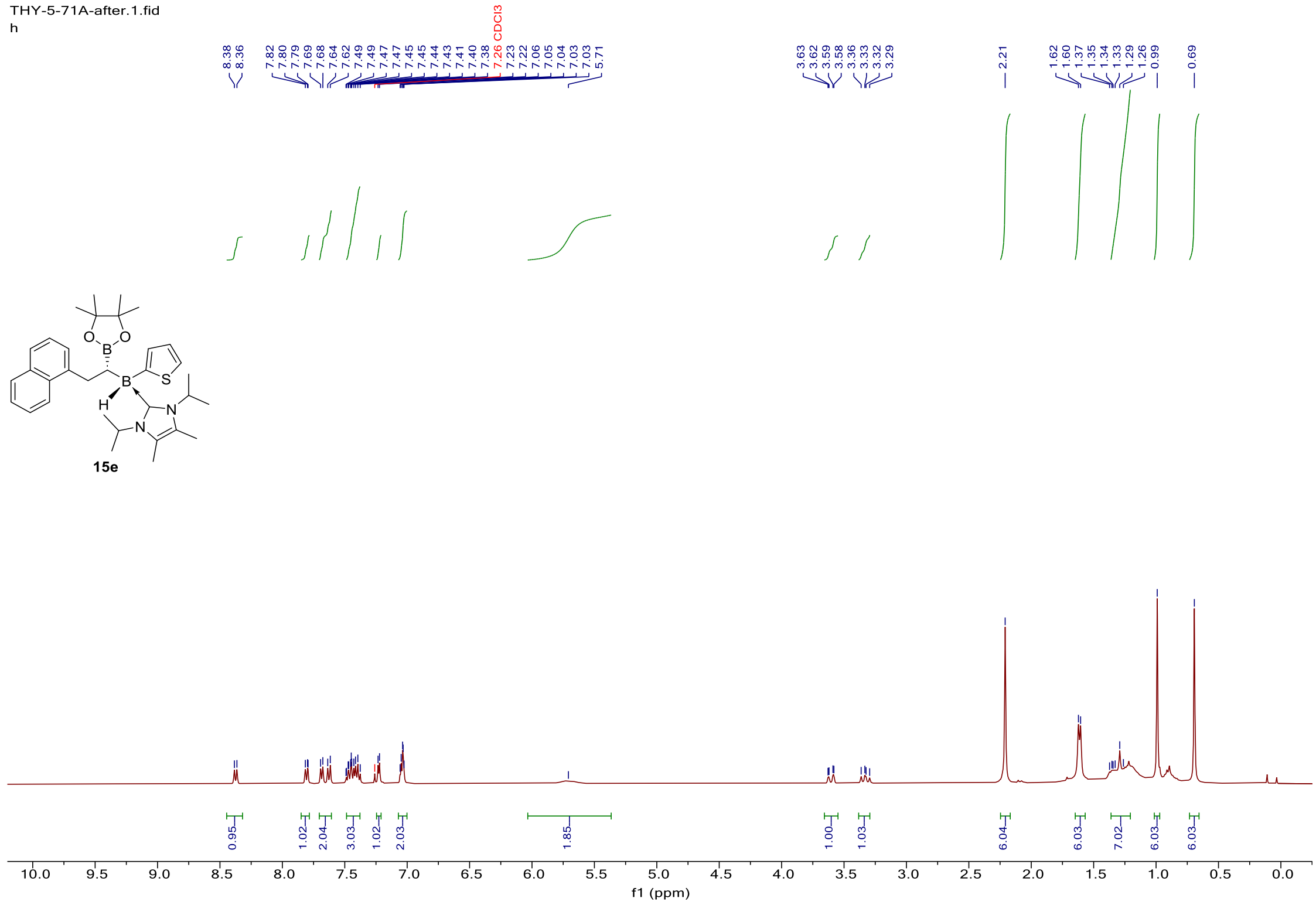

^{1}H NMR (400 MHz, $CDCl_3$)

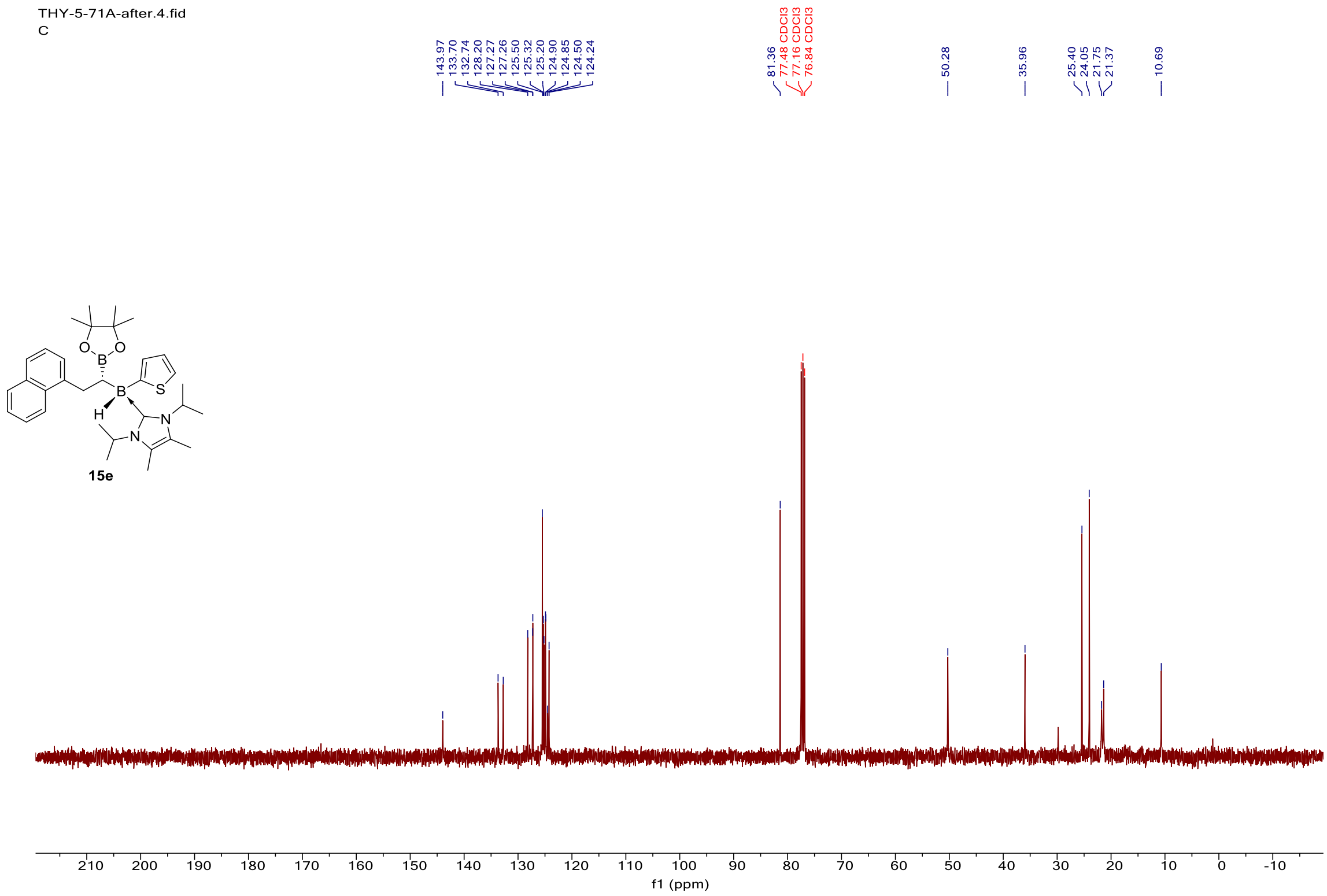

^{13}C NMR (101 MHz, $CDCl_3$)

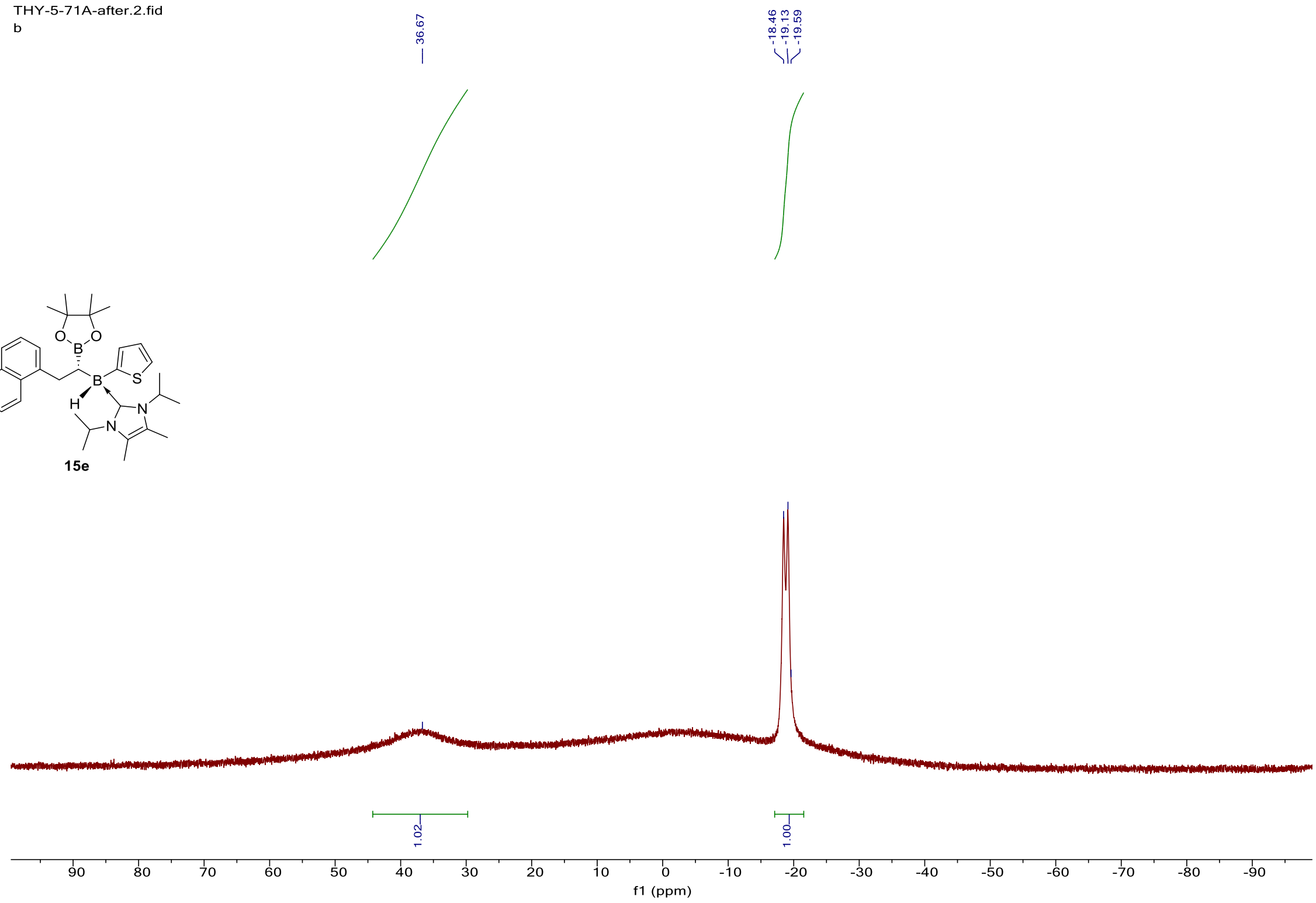

^{11}B NMR (128 MHz, $CDCl_3$)

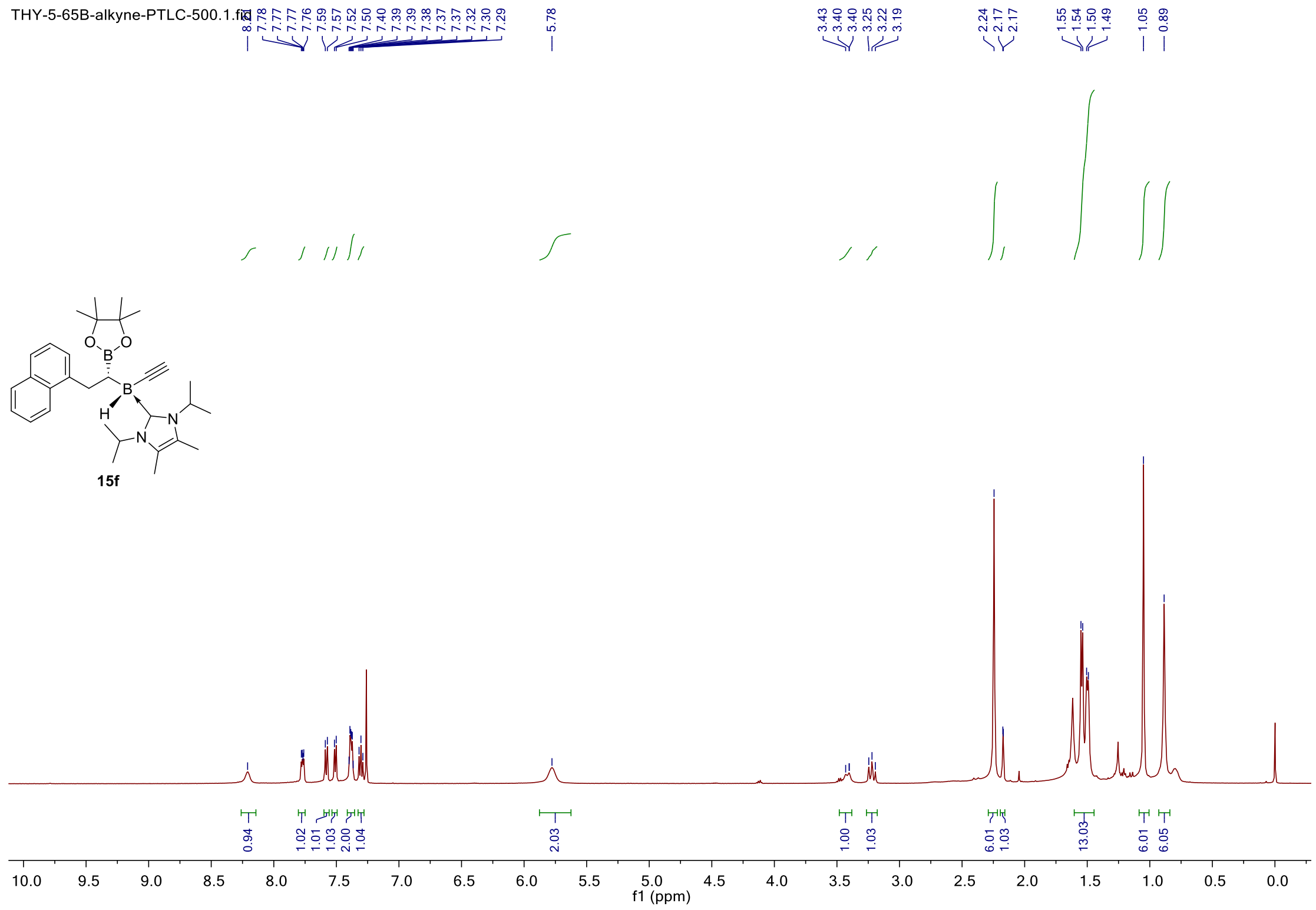

^{1}H NMR (500 MHz, $CDCl_3$)

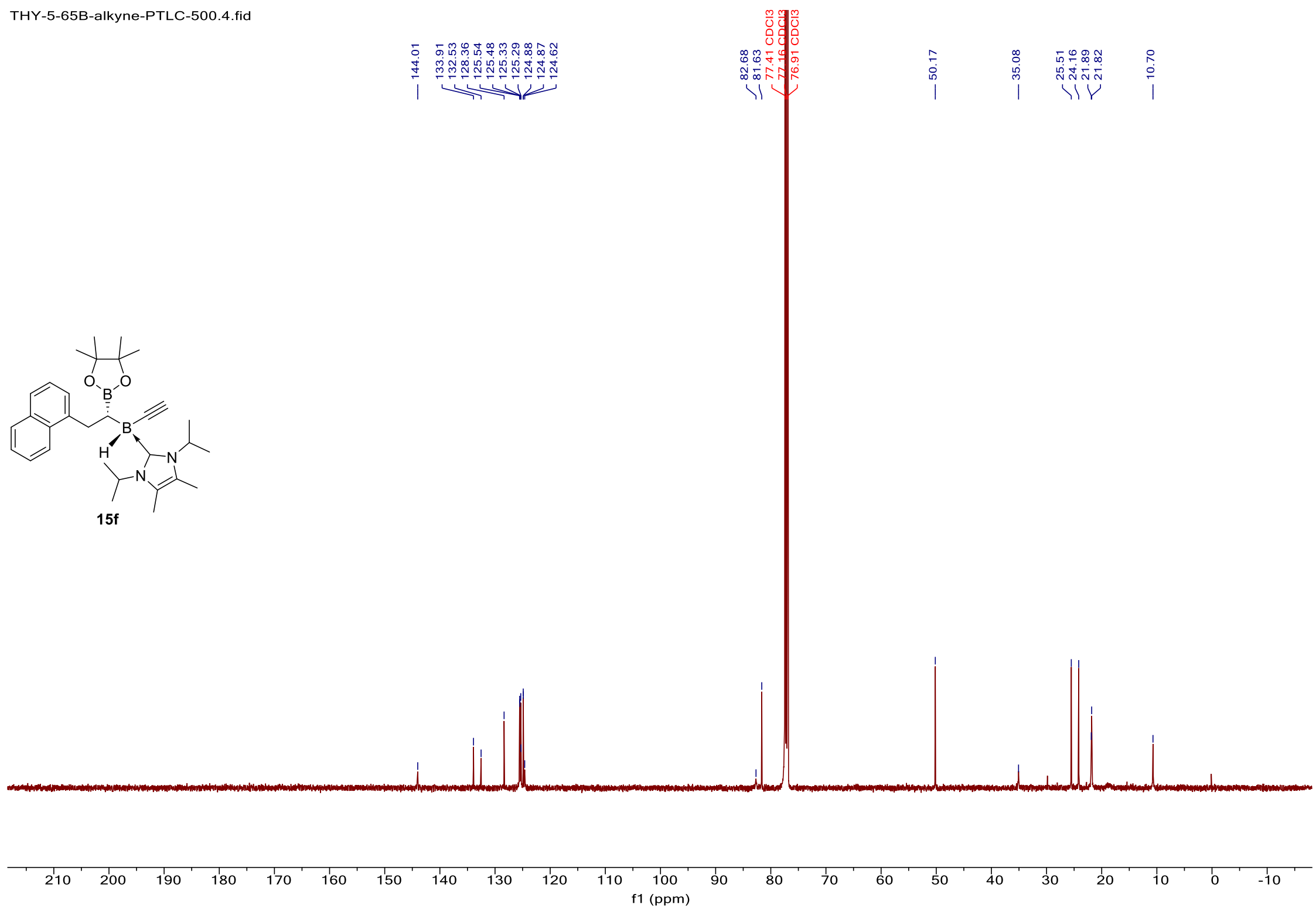

^{13}C NMR (126 MHz, $CDCl_3$)

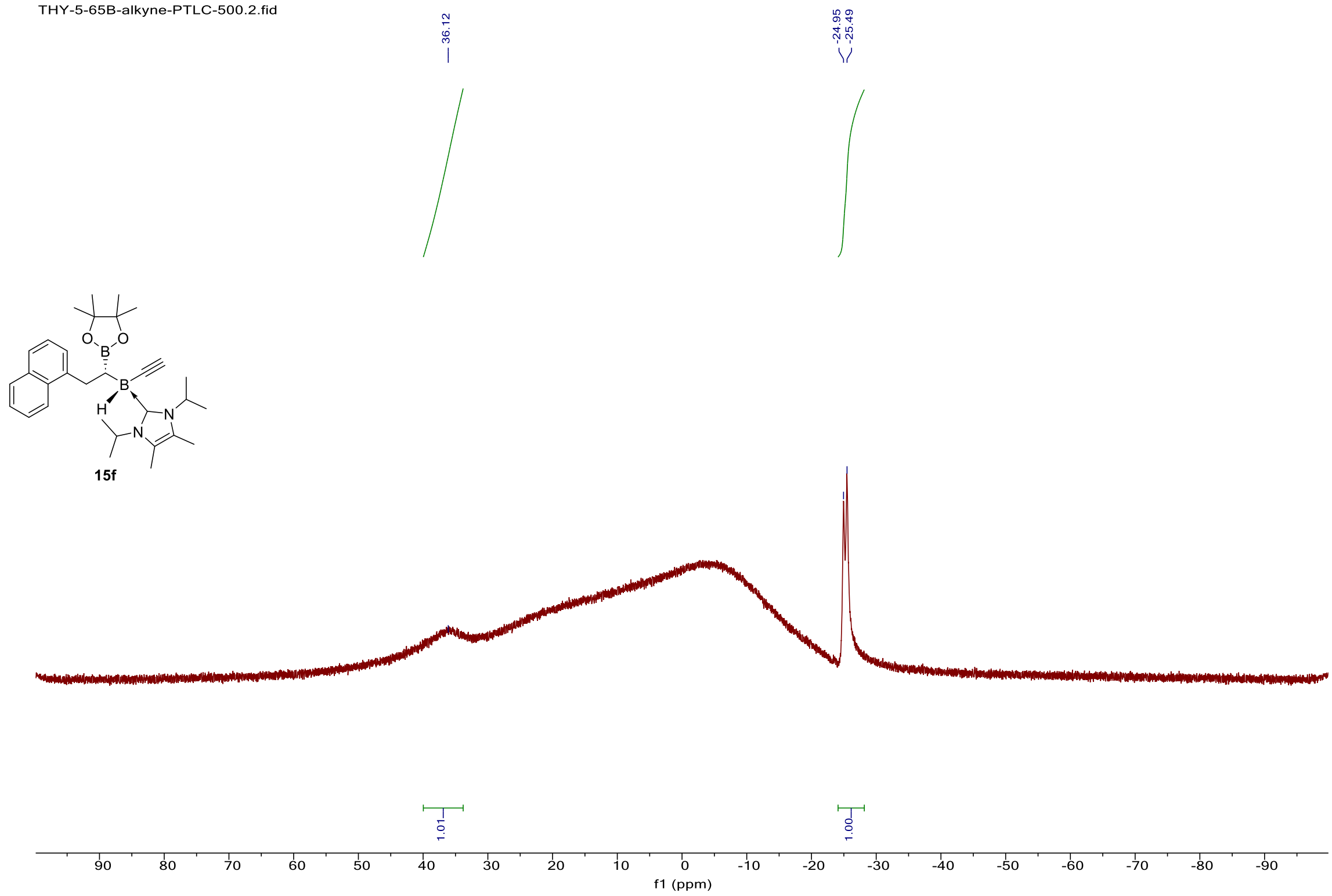

^{11}B NMR (160 MHz, $CDCl_3$)

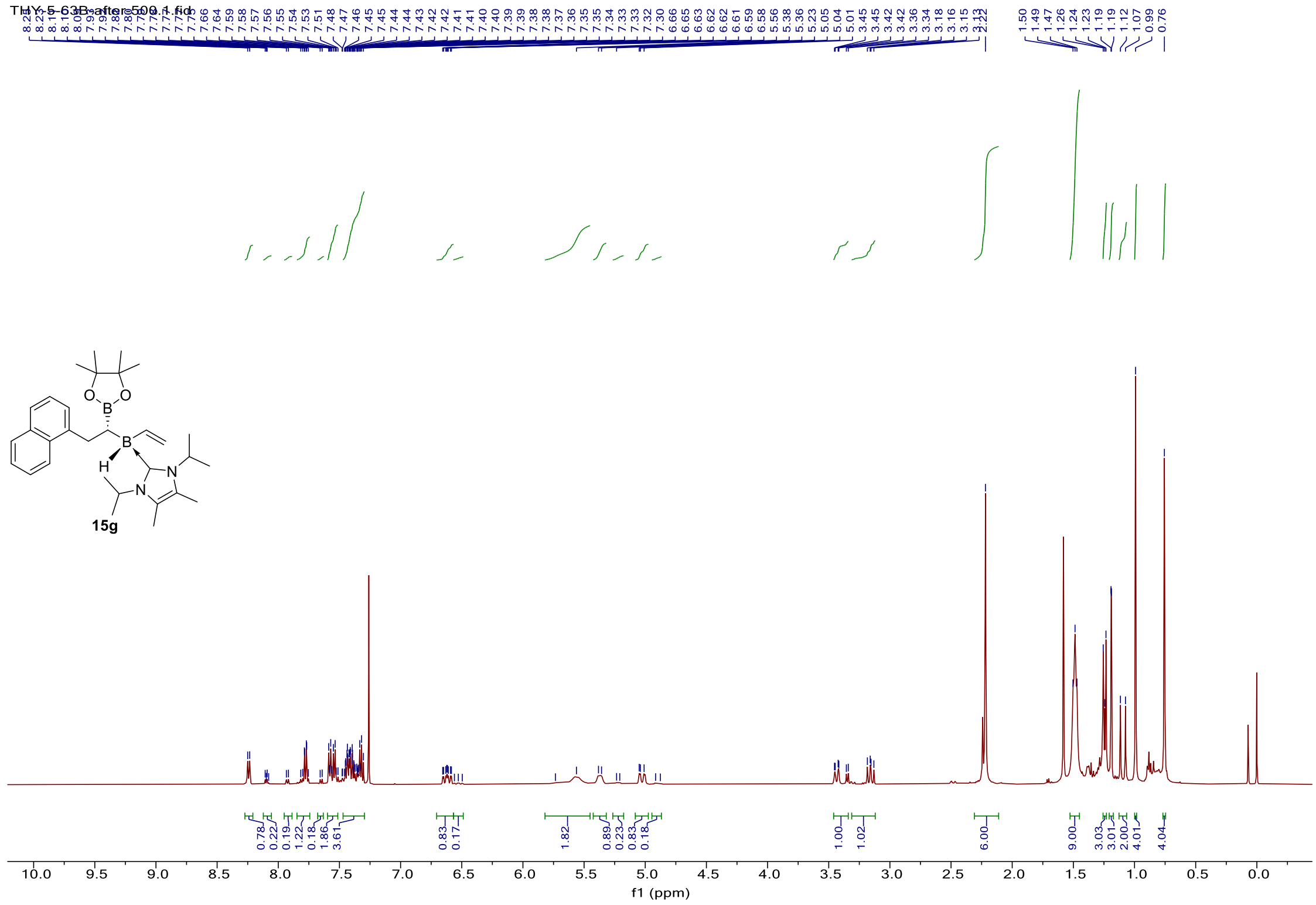

^{1}H NMR (500 MHz, $CDCl_3$)

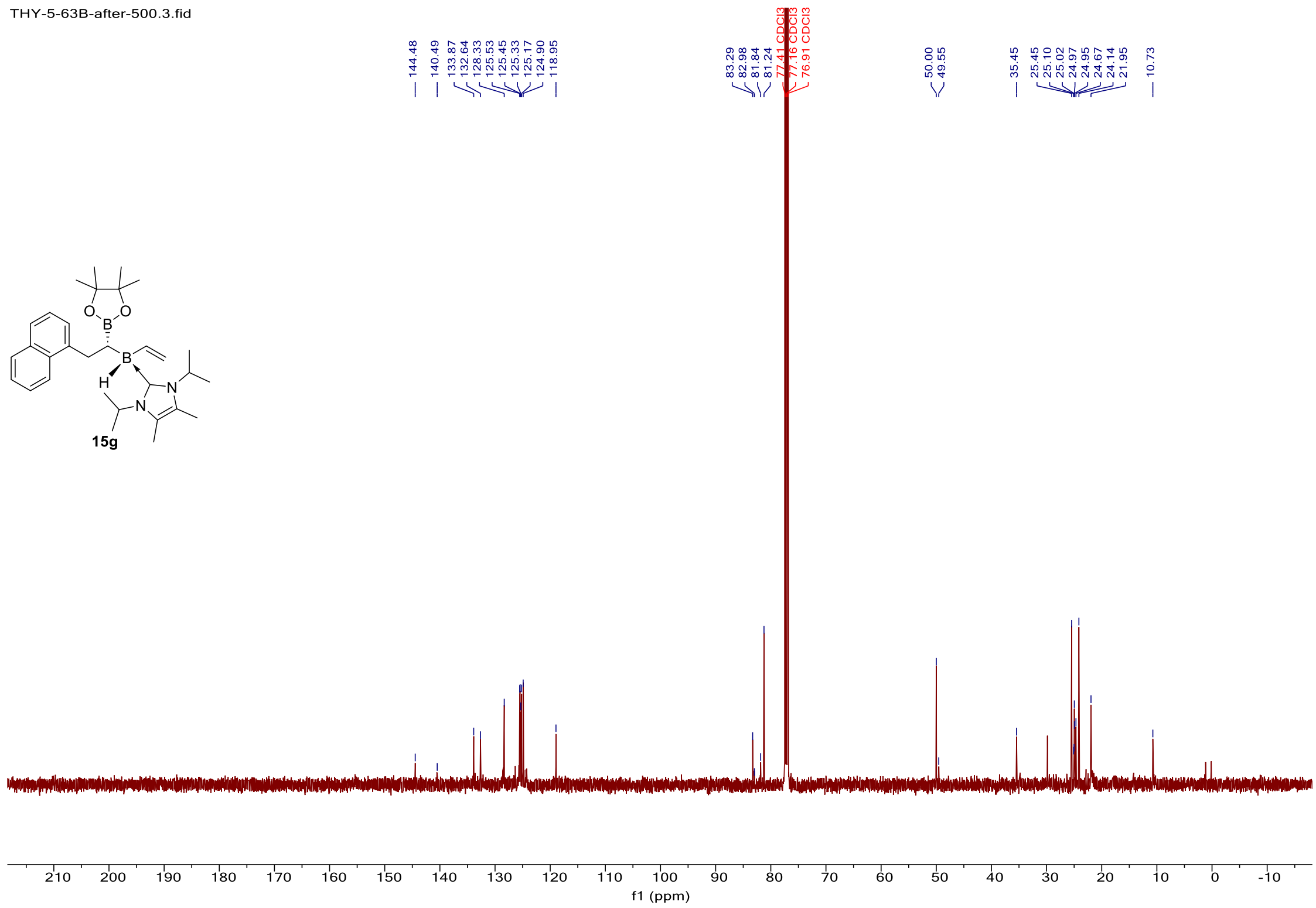

^{13}C NMR (126 MHz, $CDCl_3$)

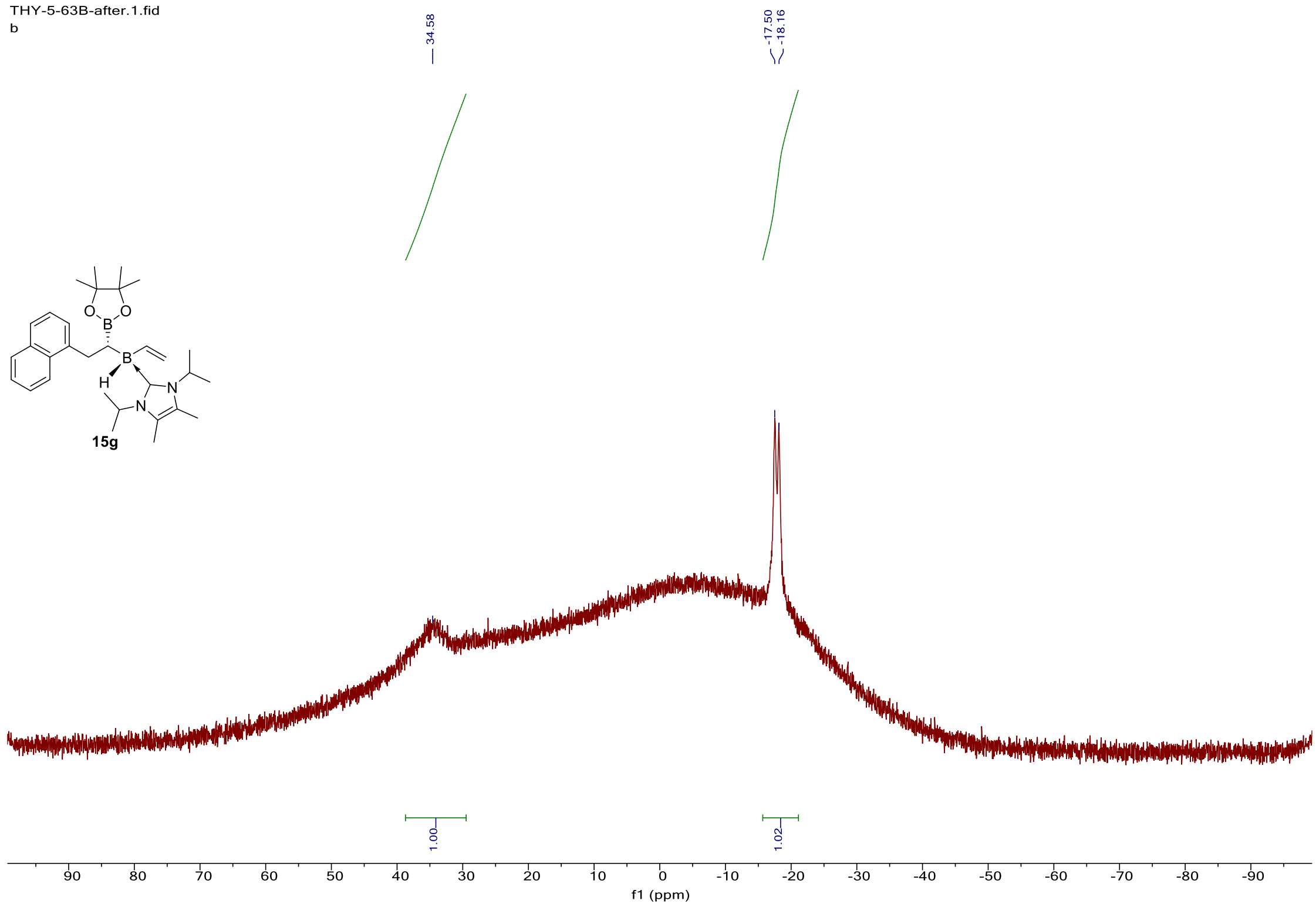

^{11}B NMR (160 MHz, $CDCl_3$)

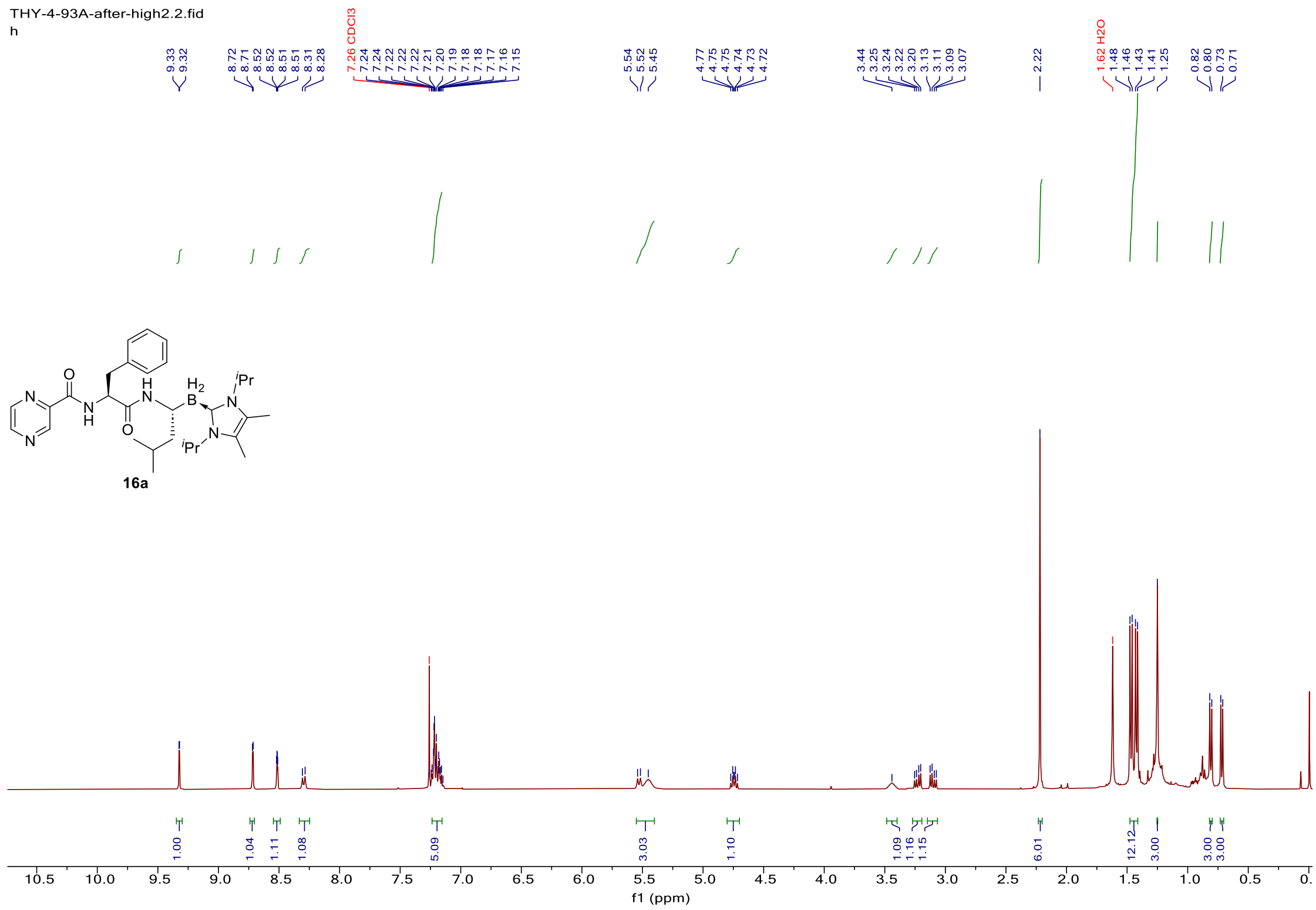

^{1}H NMR (400 MHz, $CDCl_3$)

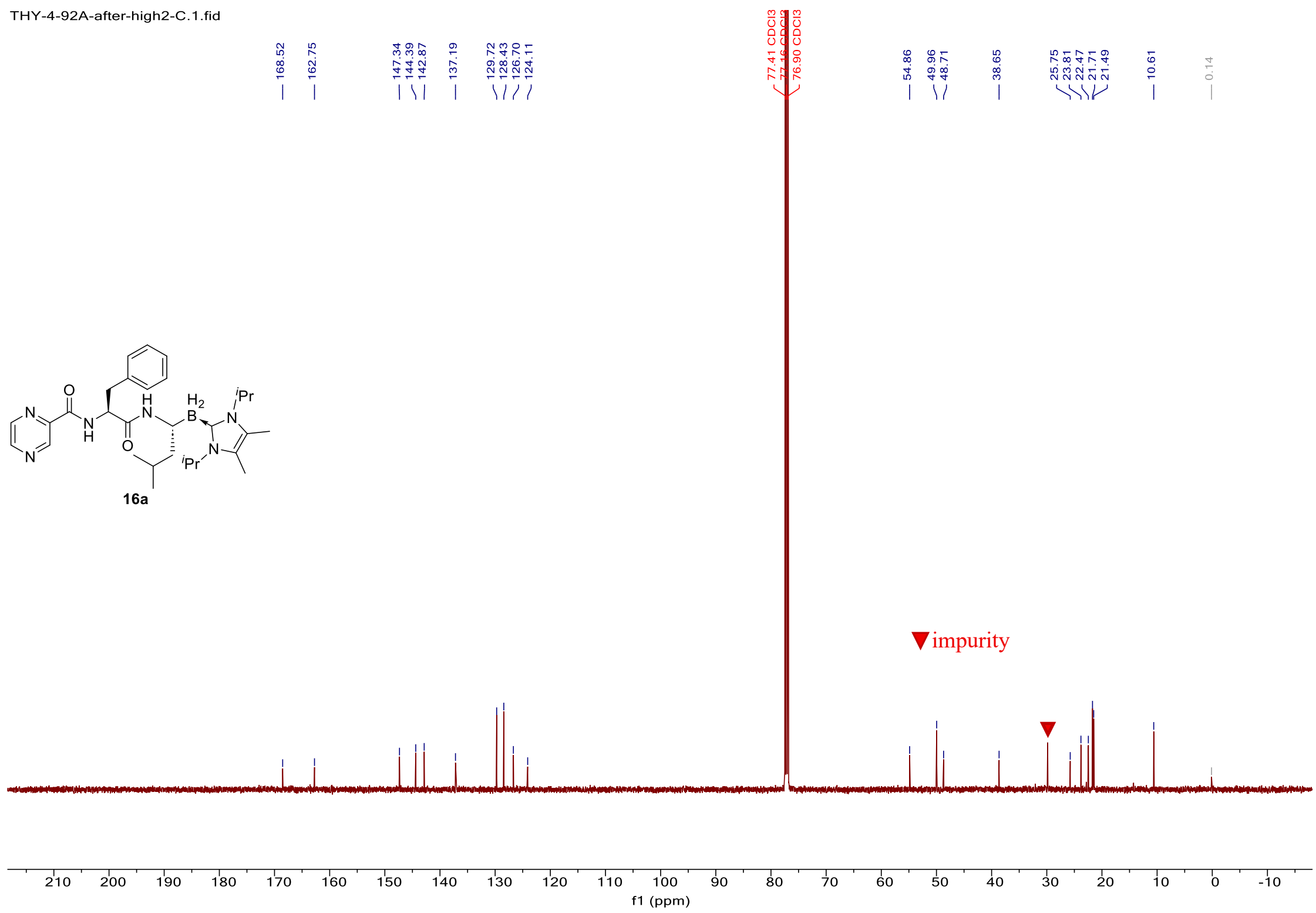

^{13}C NMR (101 MHz, $CDCl_3$)

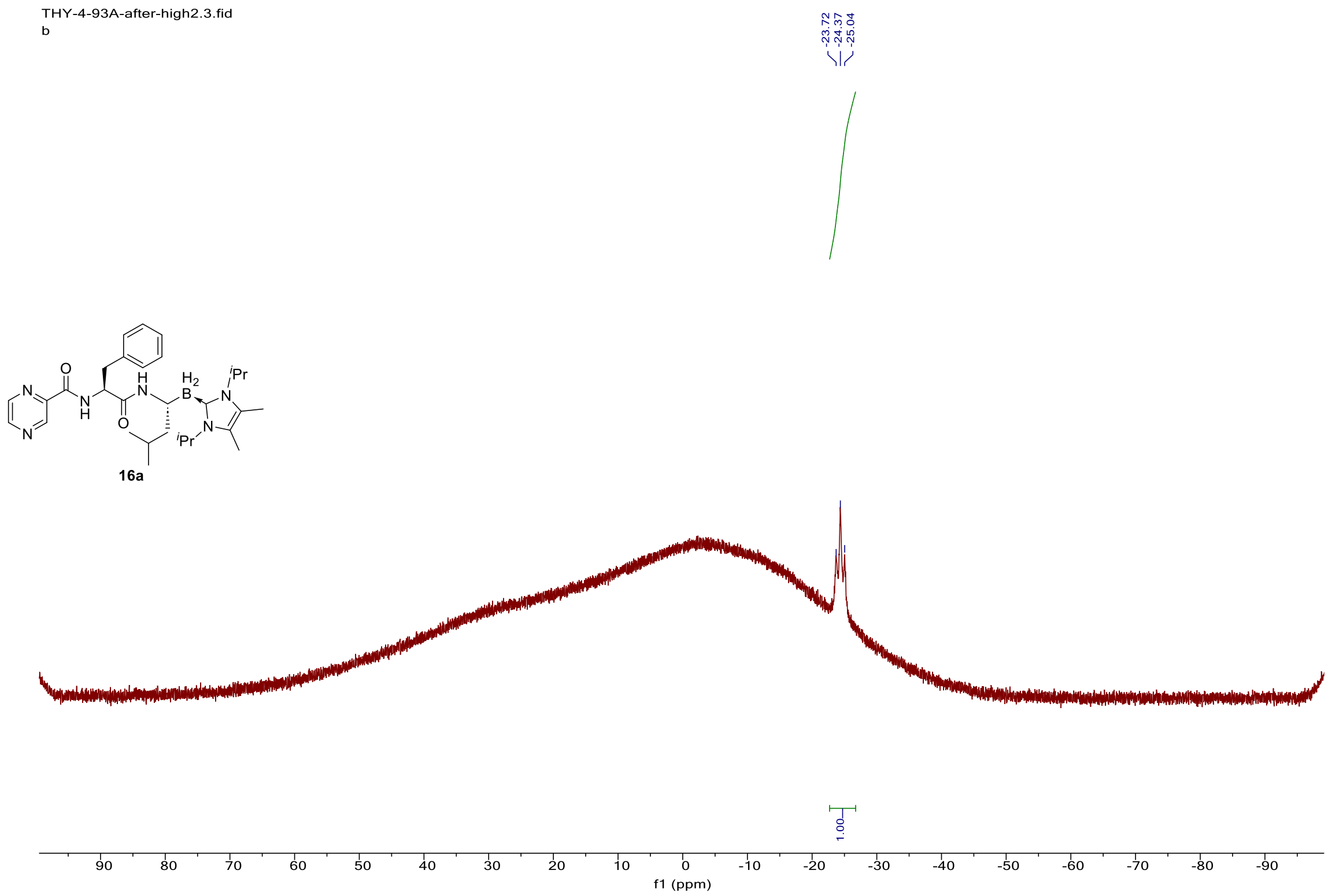

^{11}B NMR (128 MHz, $CDCl_3$)

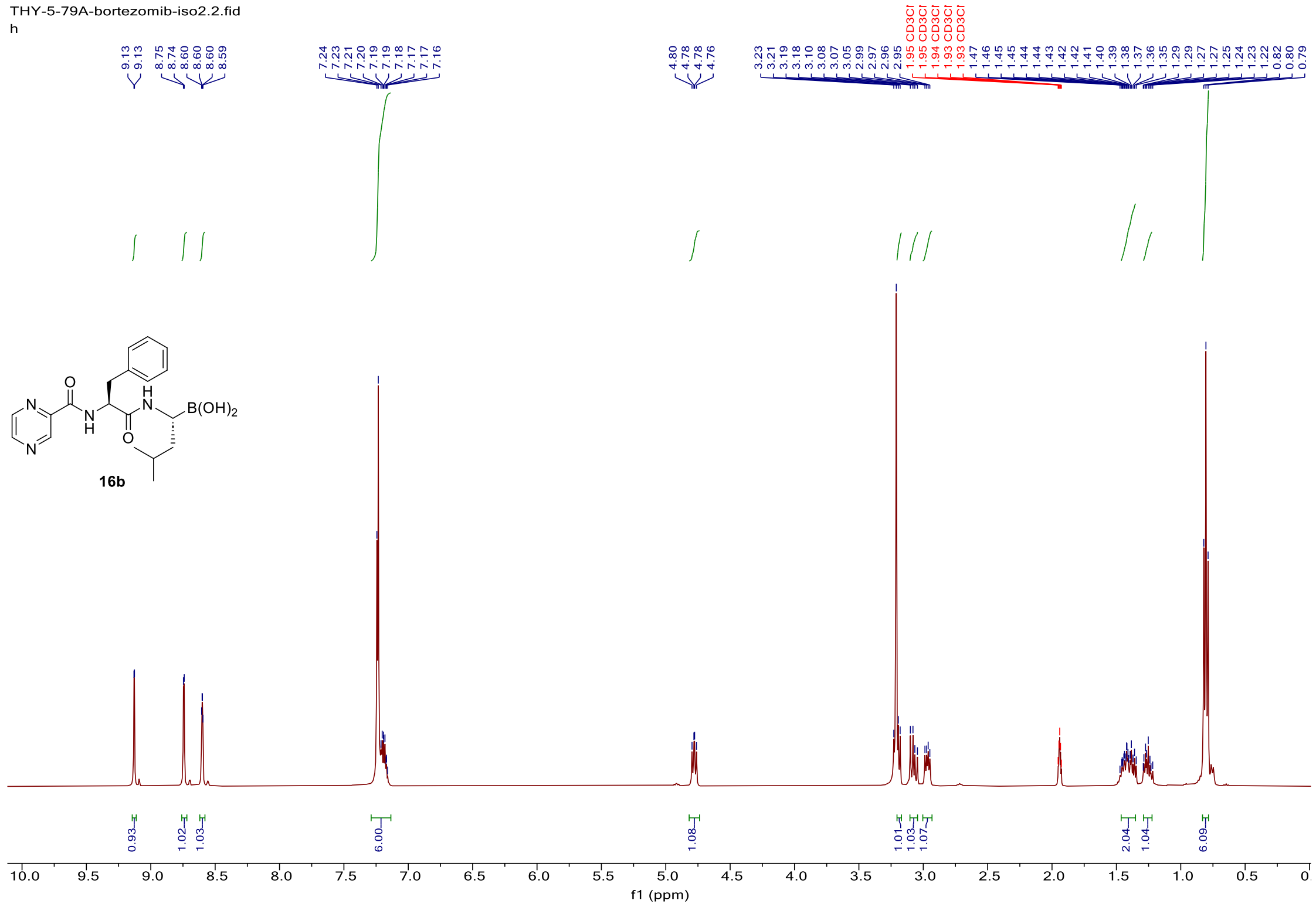

^{1}H NMR (400 MHz, CD_3CN/D_2O 10: 1)

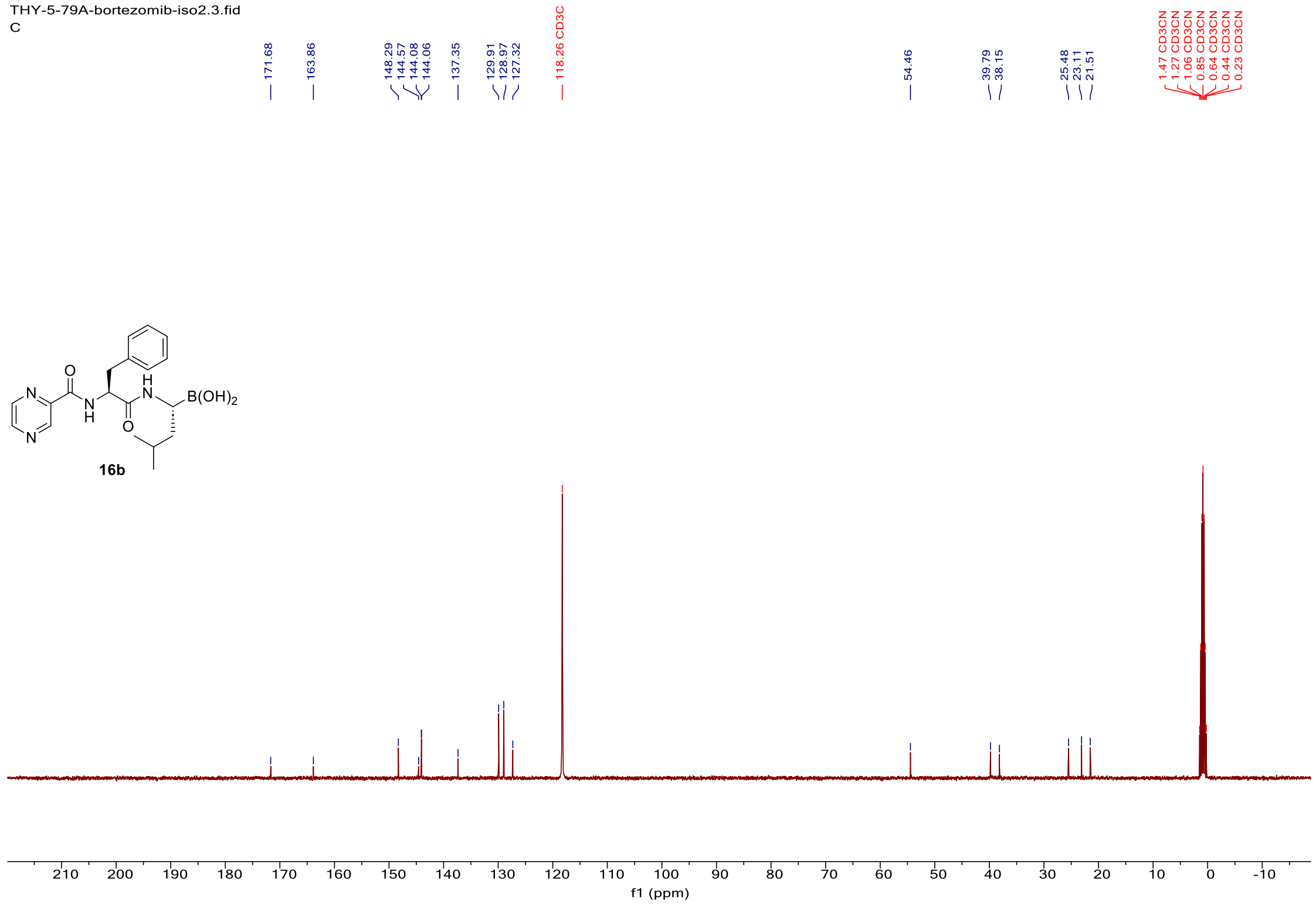

^{13}C NMR (101 MHz, CD_3CN/D_2O 10: 1)

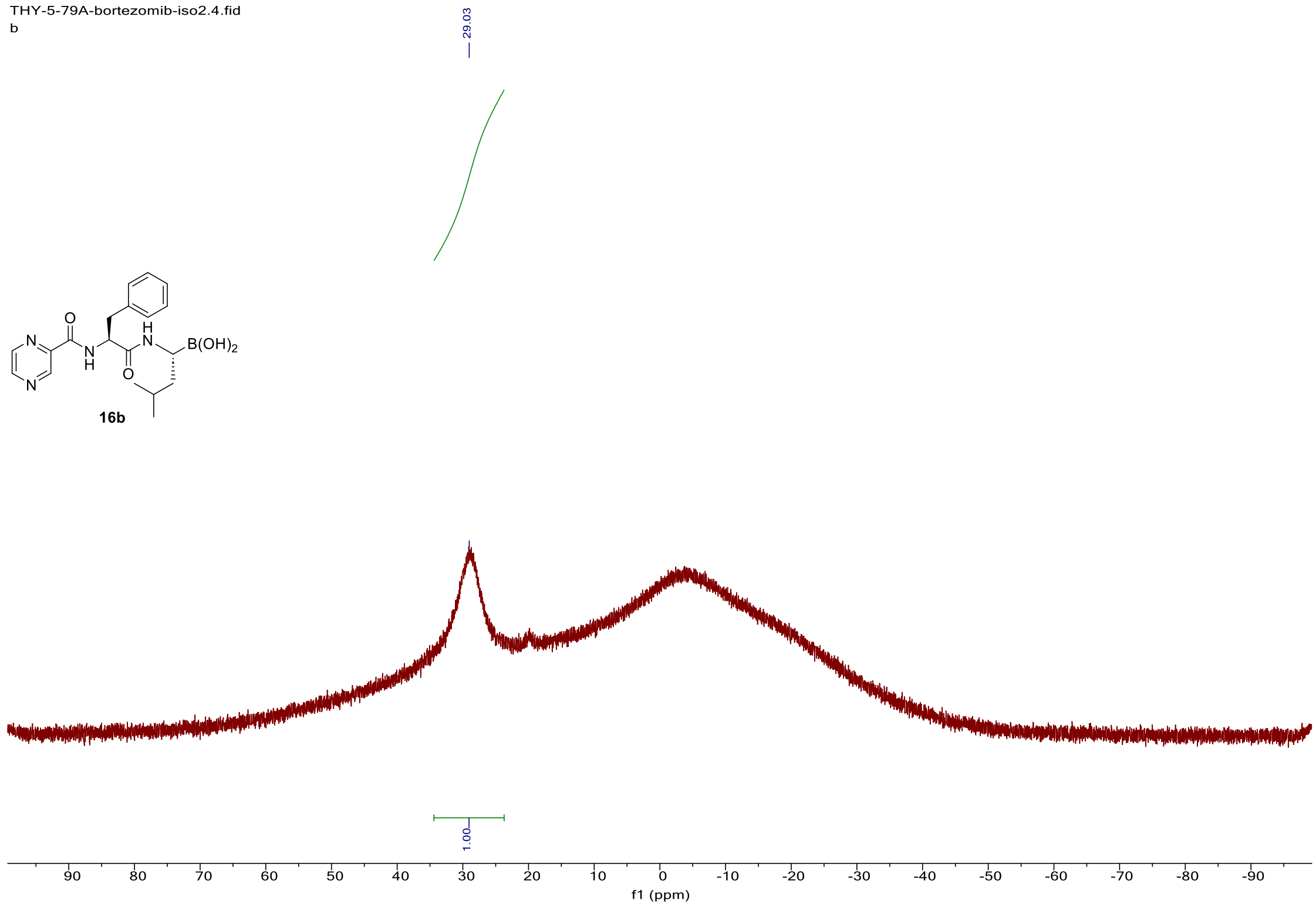

^{11}B NMR (128 MHz, CD_3CN/D_2O 10: 1)

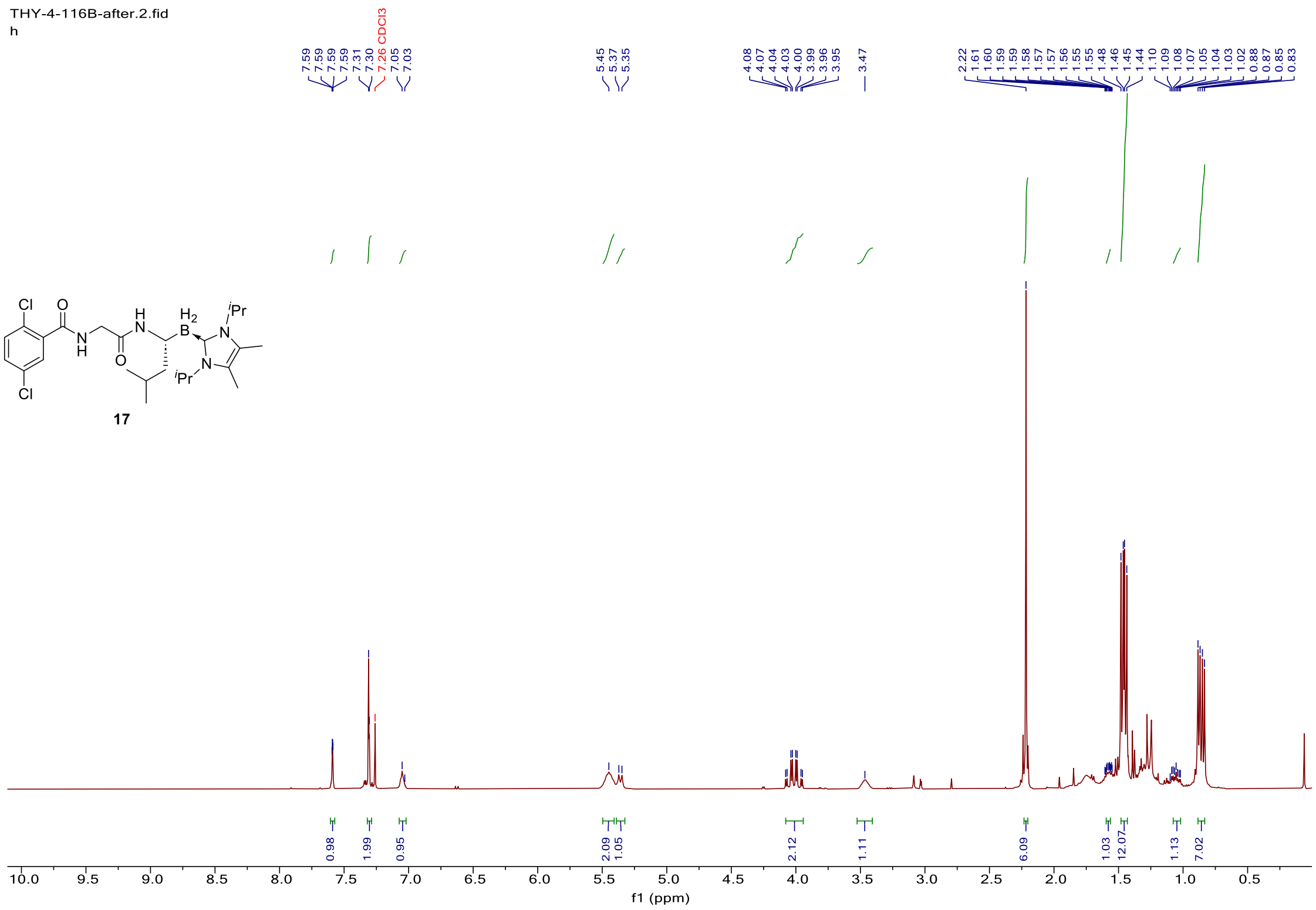

^{1}H NMR (400 MHz, $CDCl_3$)

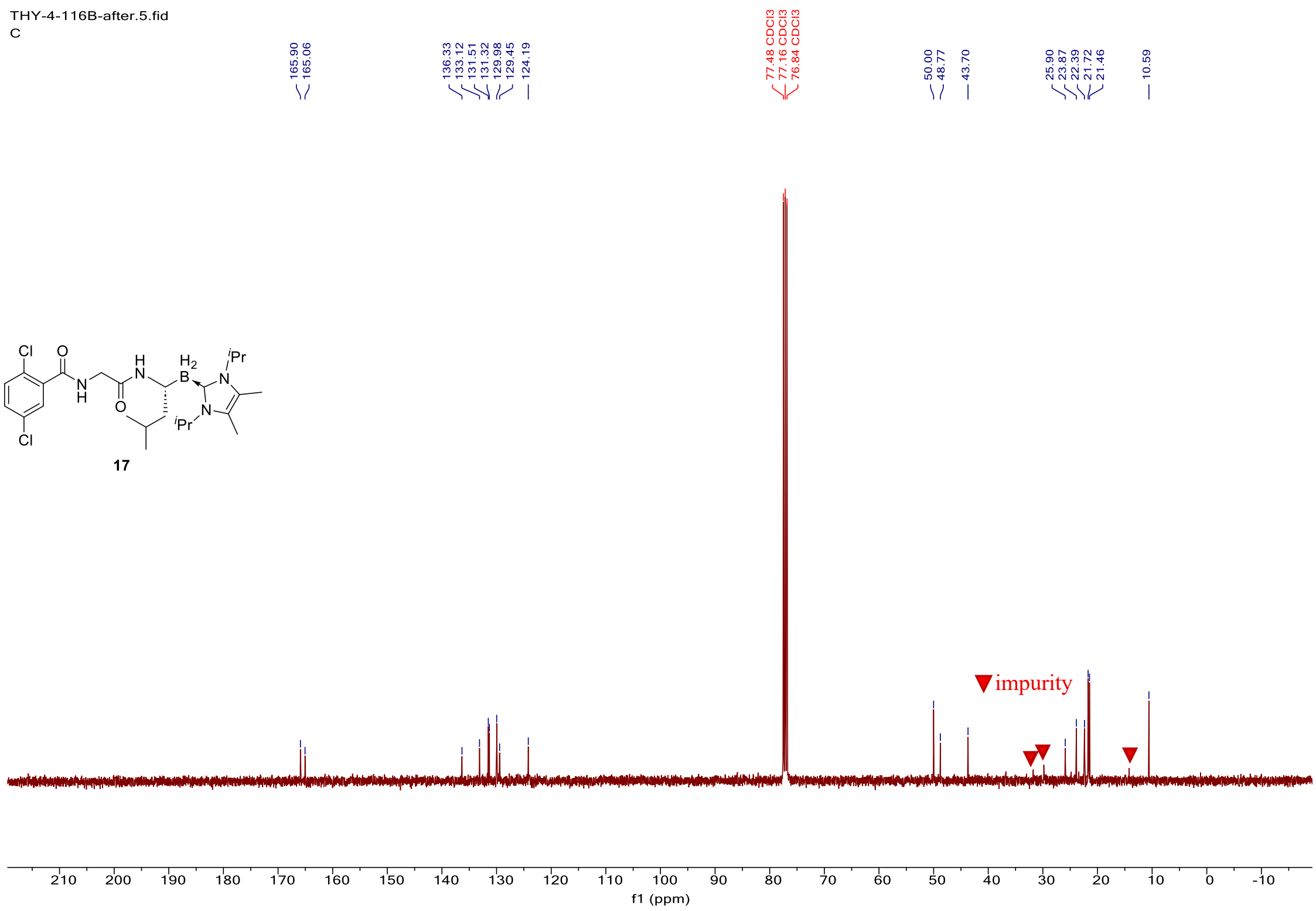

^{13}C NMR (101 MHz, $CDCl_3$)

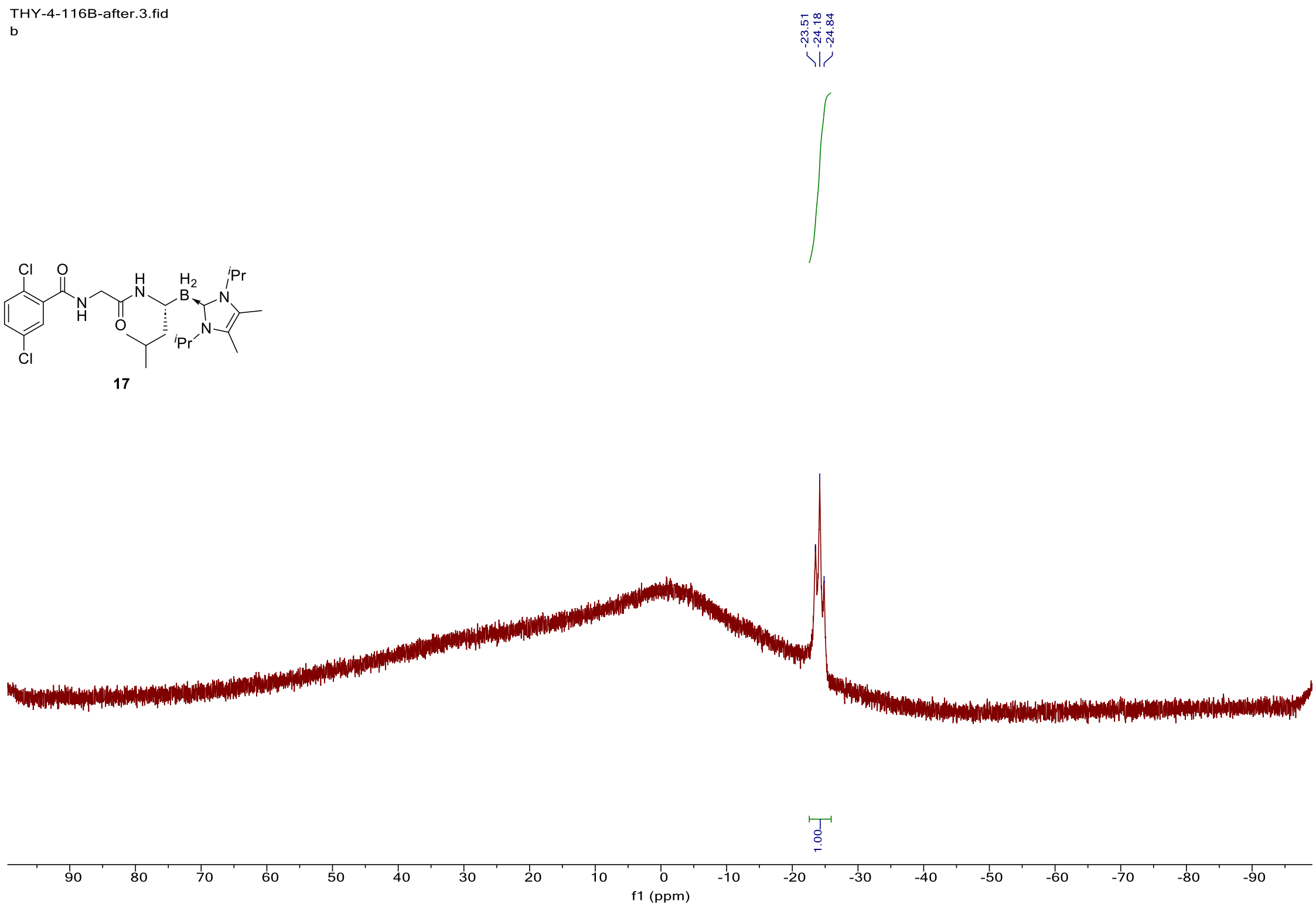

^{11}B NMR (128 MHz, $CDCl_3$)

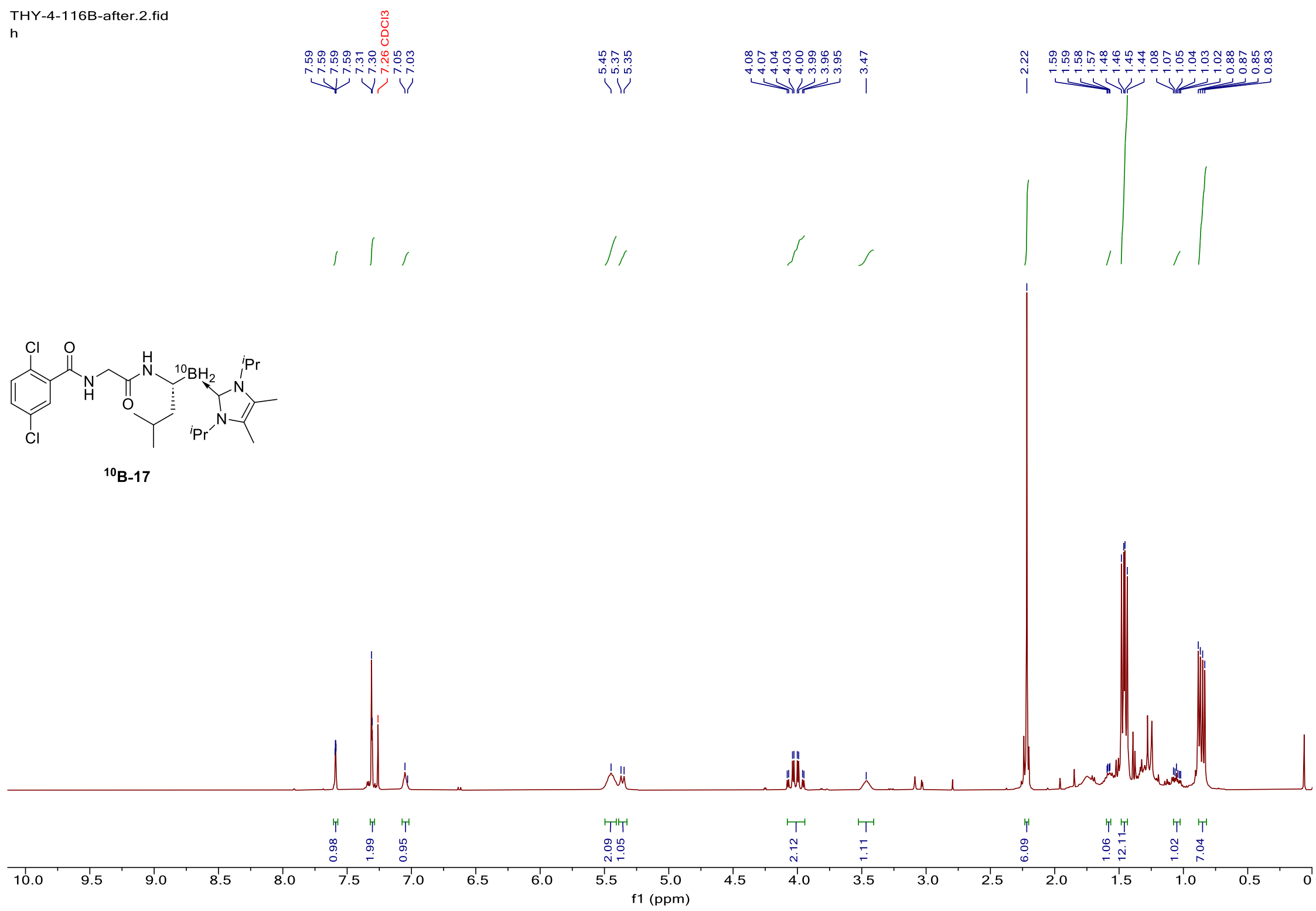

^{1}H NMR (400 MHz, $CDCl_3$)

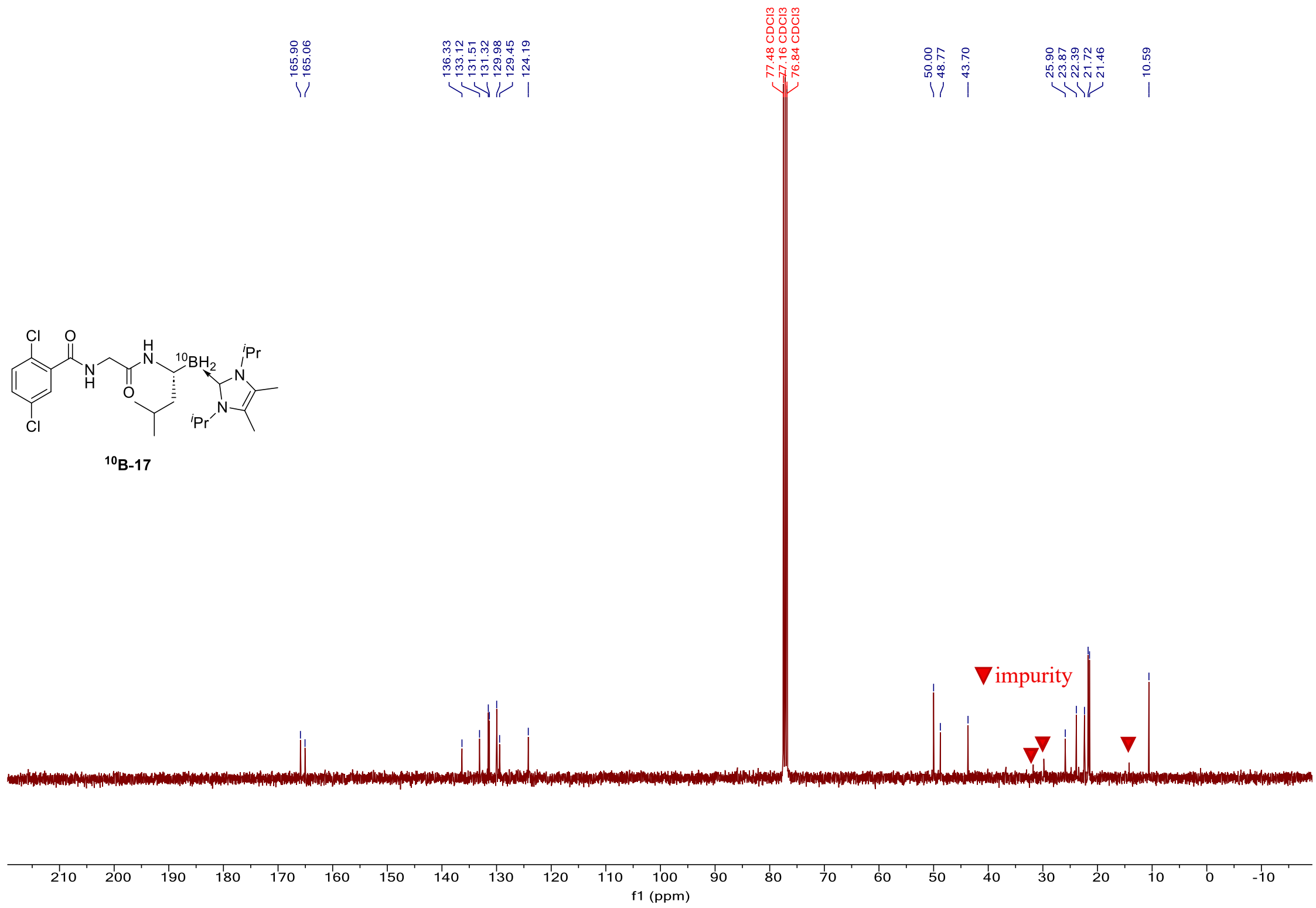

^{13}C NMR (101 MHz, $CDCl_3$)

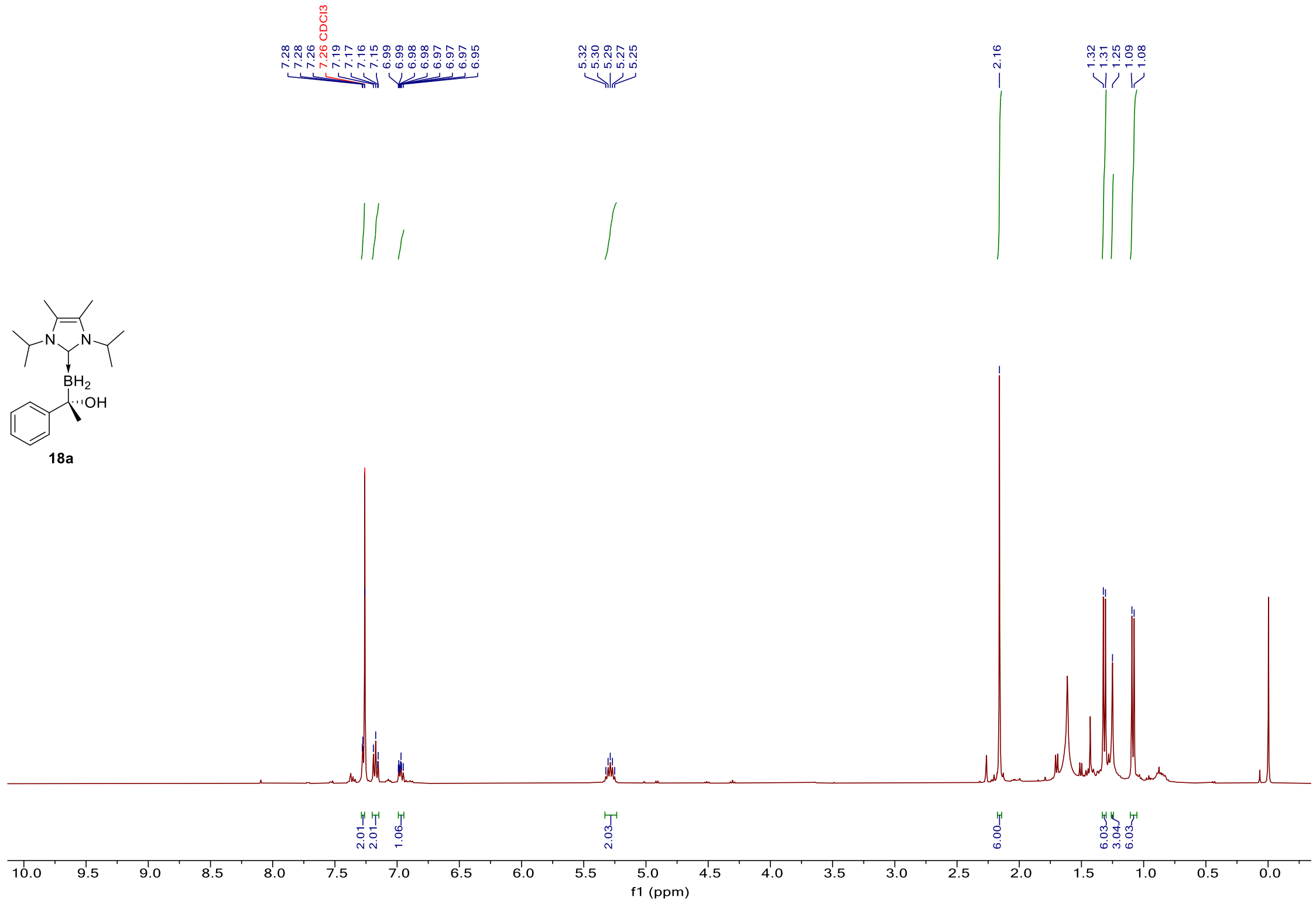

^{1}H NMR (400 MHz, $CDCl_3$)

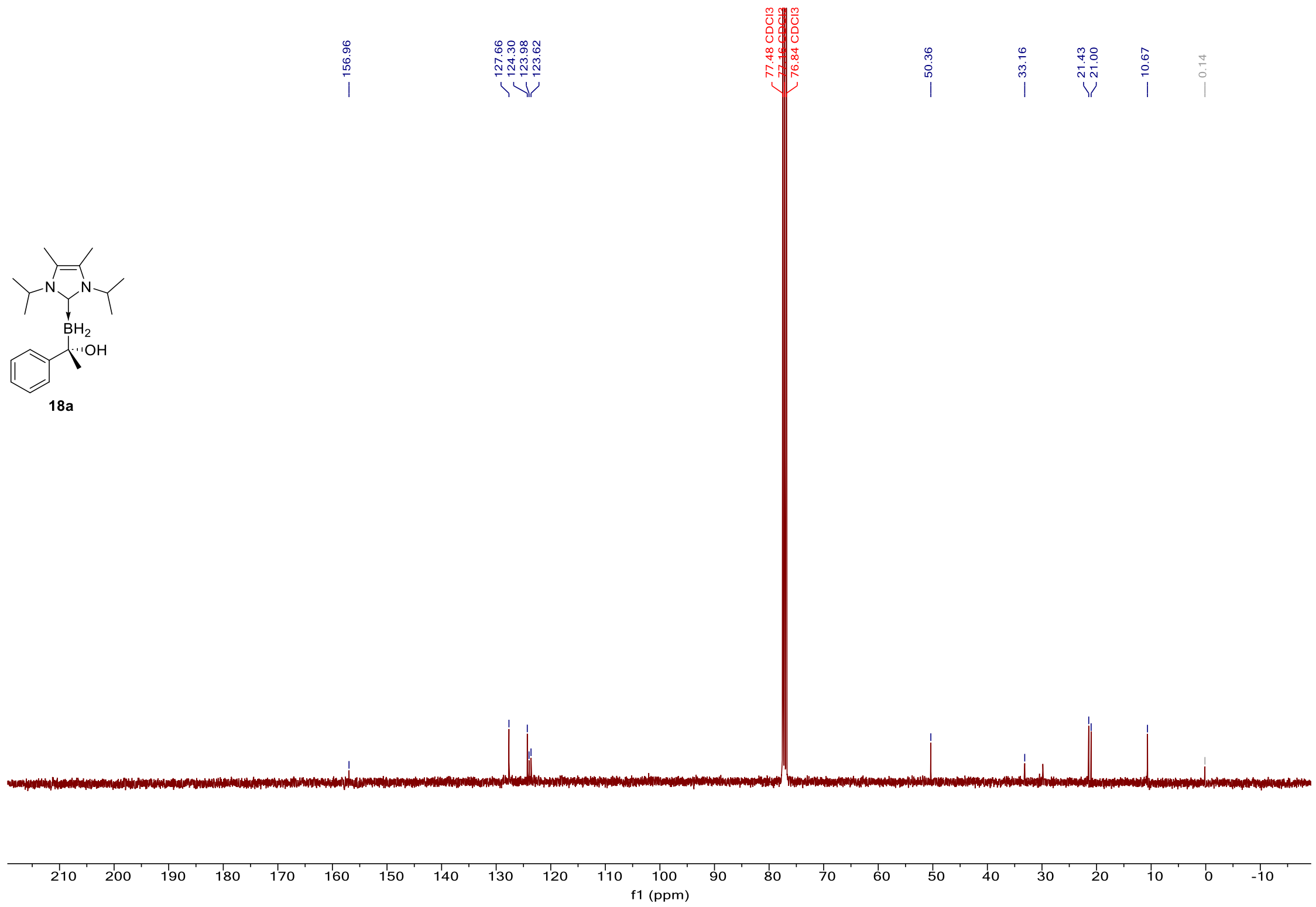

^{13}C NMR (101 MHz, $CDCl_3$)

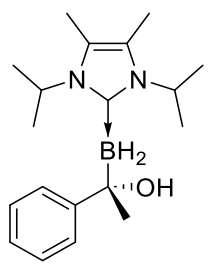

18a

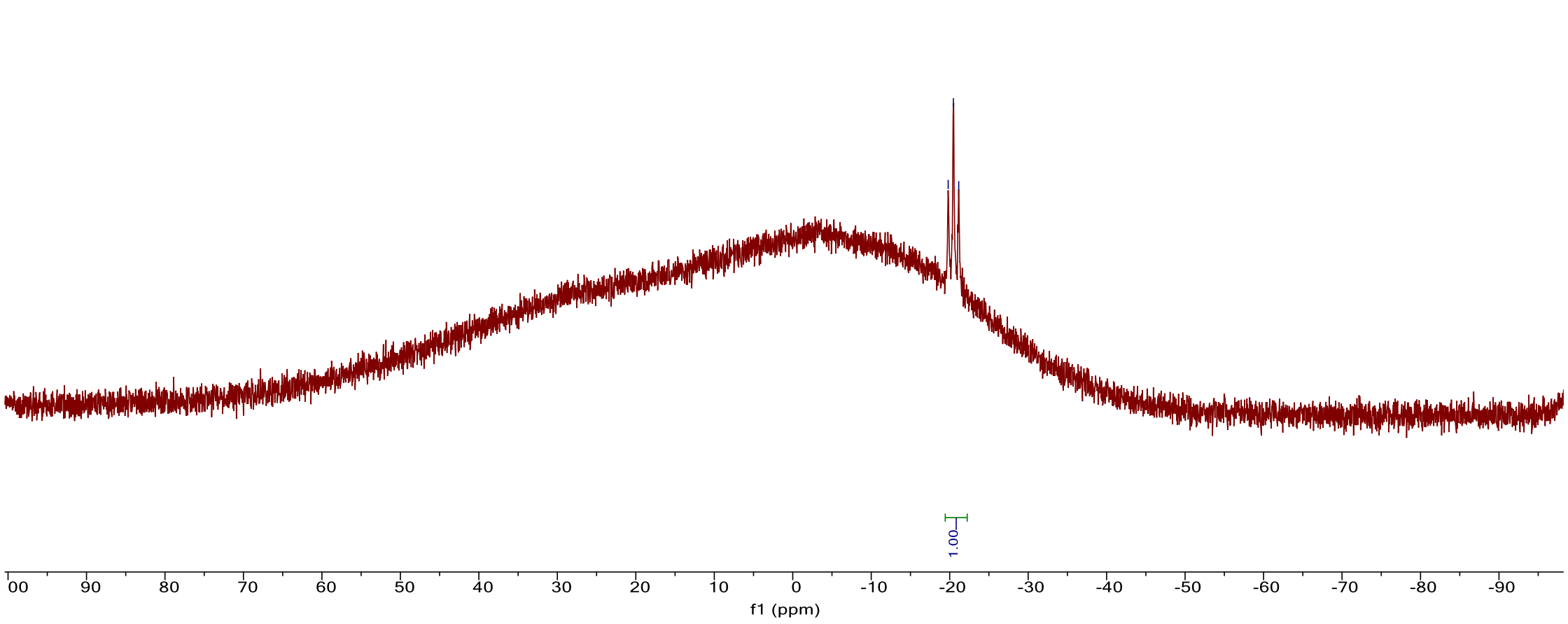

^{11}B NMR (128 MHz, $CDCl_3$)

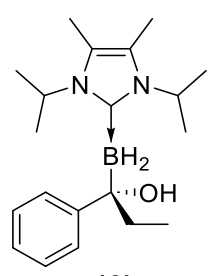

18b

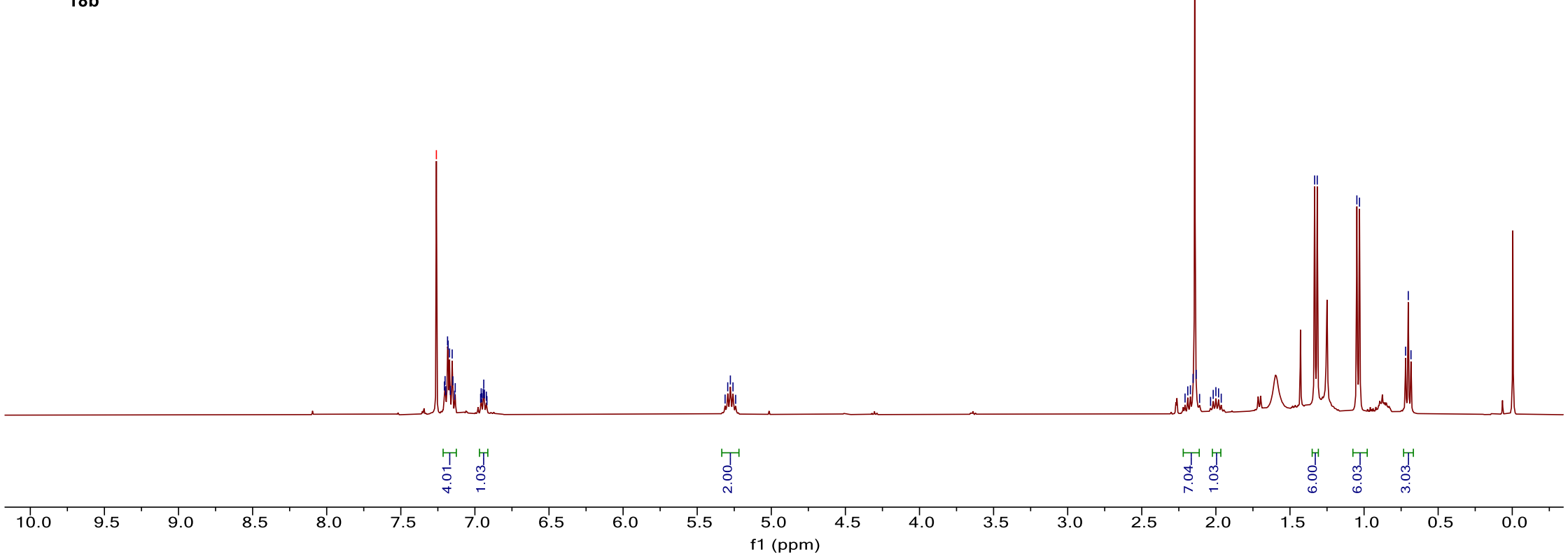

^{1}H NMR (400 MHz, $CDCl_3$)

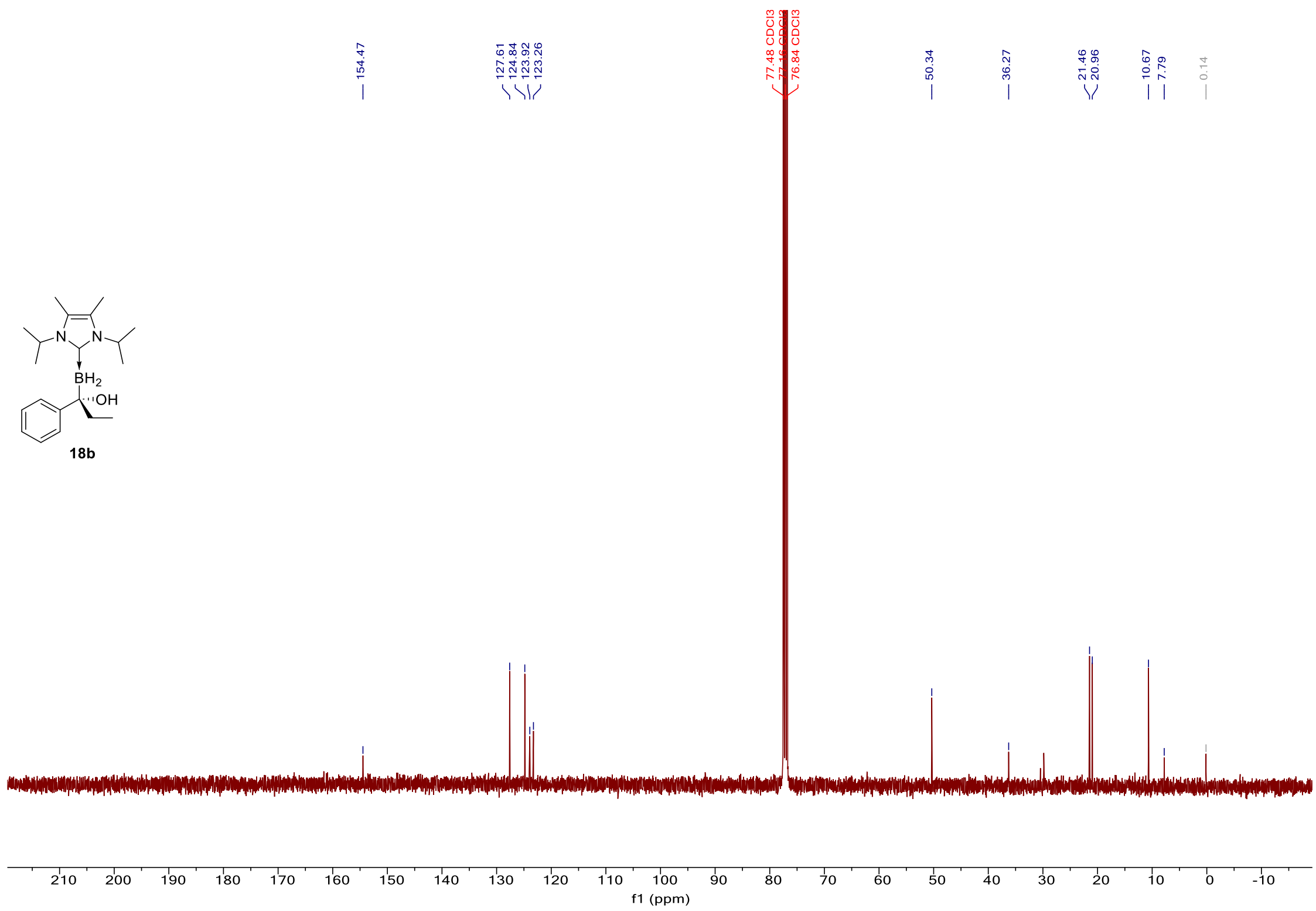

^{13}C NMR (101 MHz, $CDCl_3$)

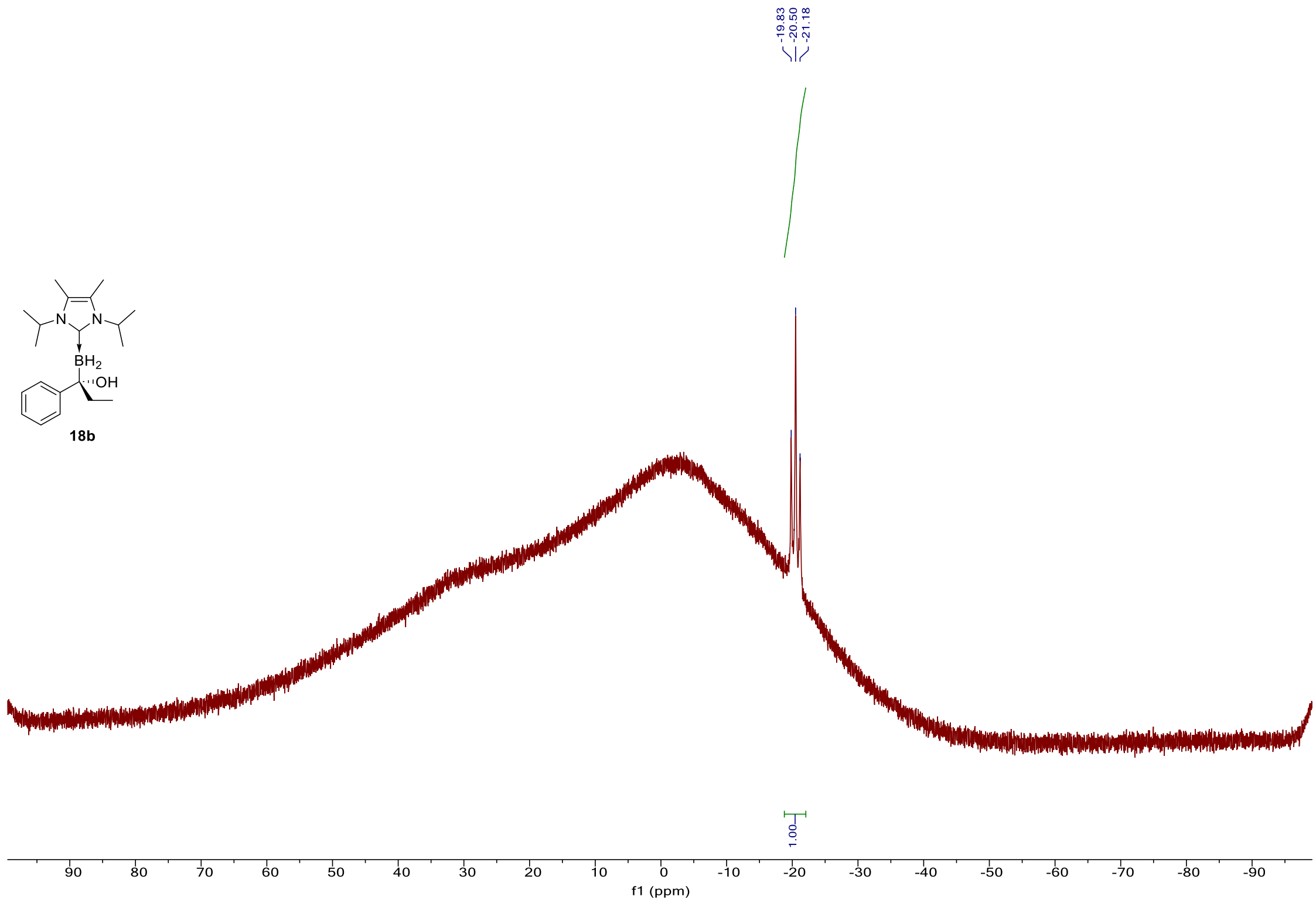

^{11}B NMR (128 MHz, $CDCl_3$)

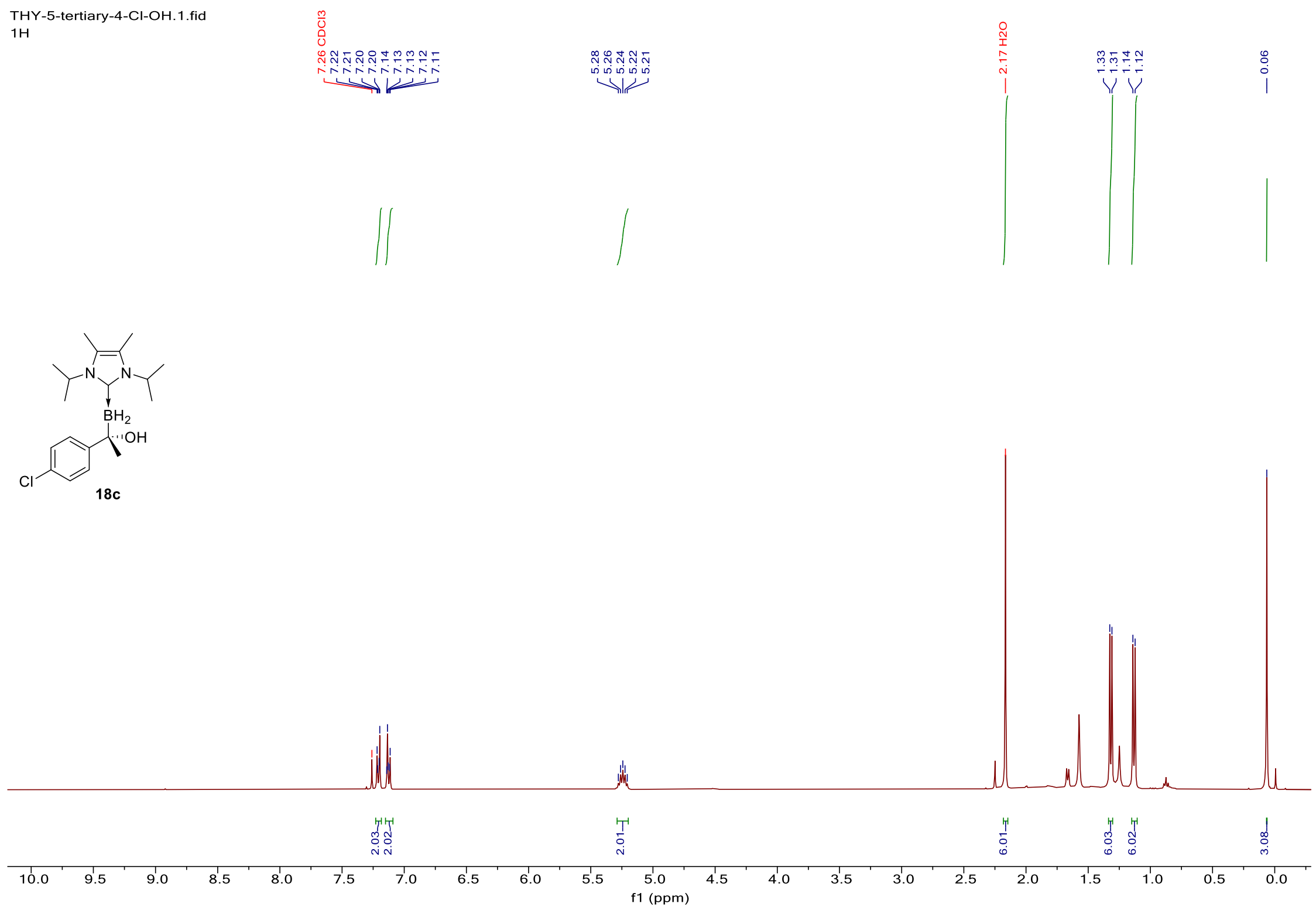

^{1}H NMR (400 MHz, $CDCl_3$)

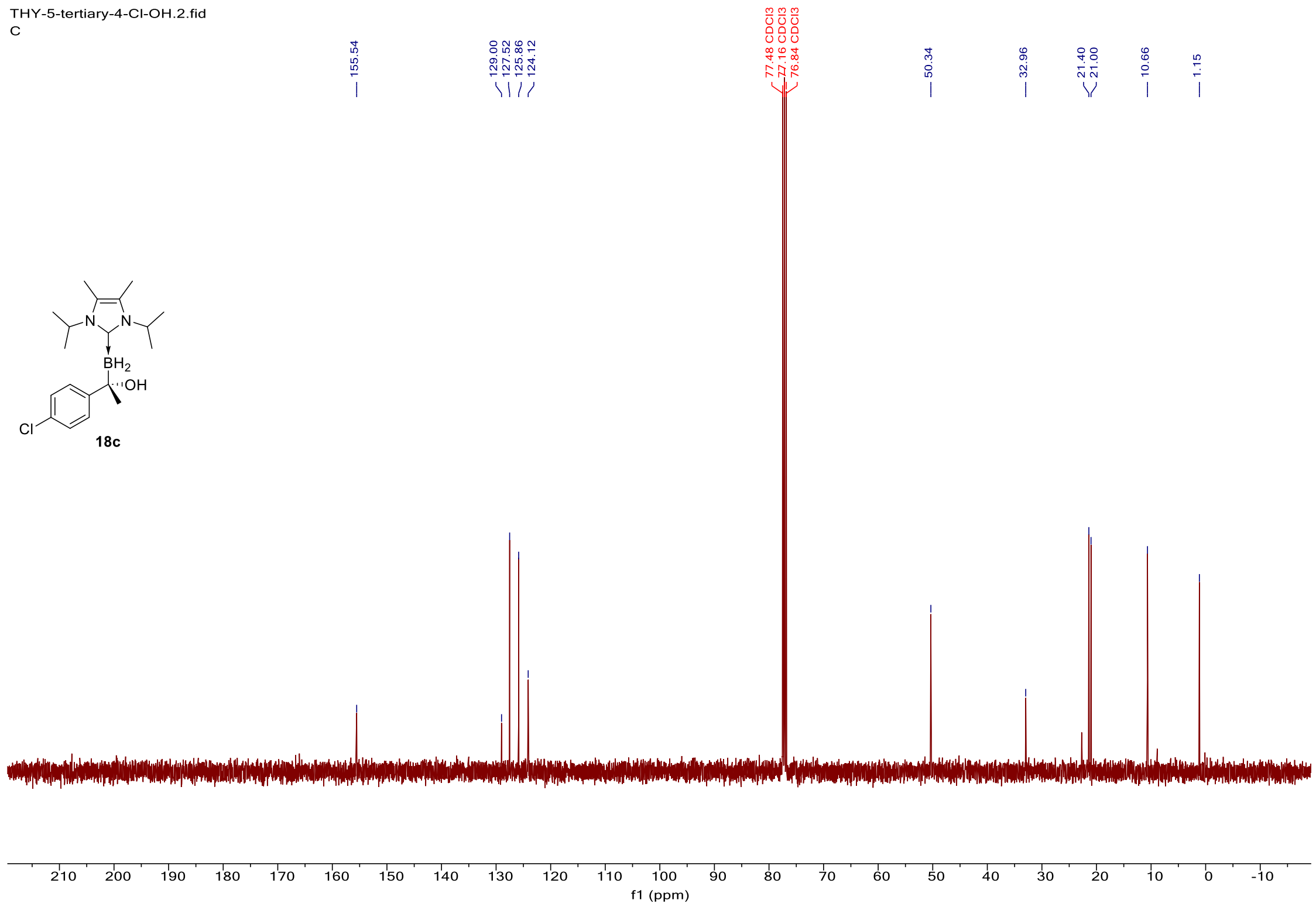

^{13}C NMR (101 MHz, $CDCl_3$)

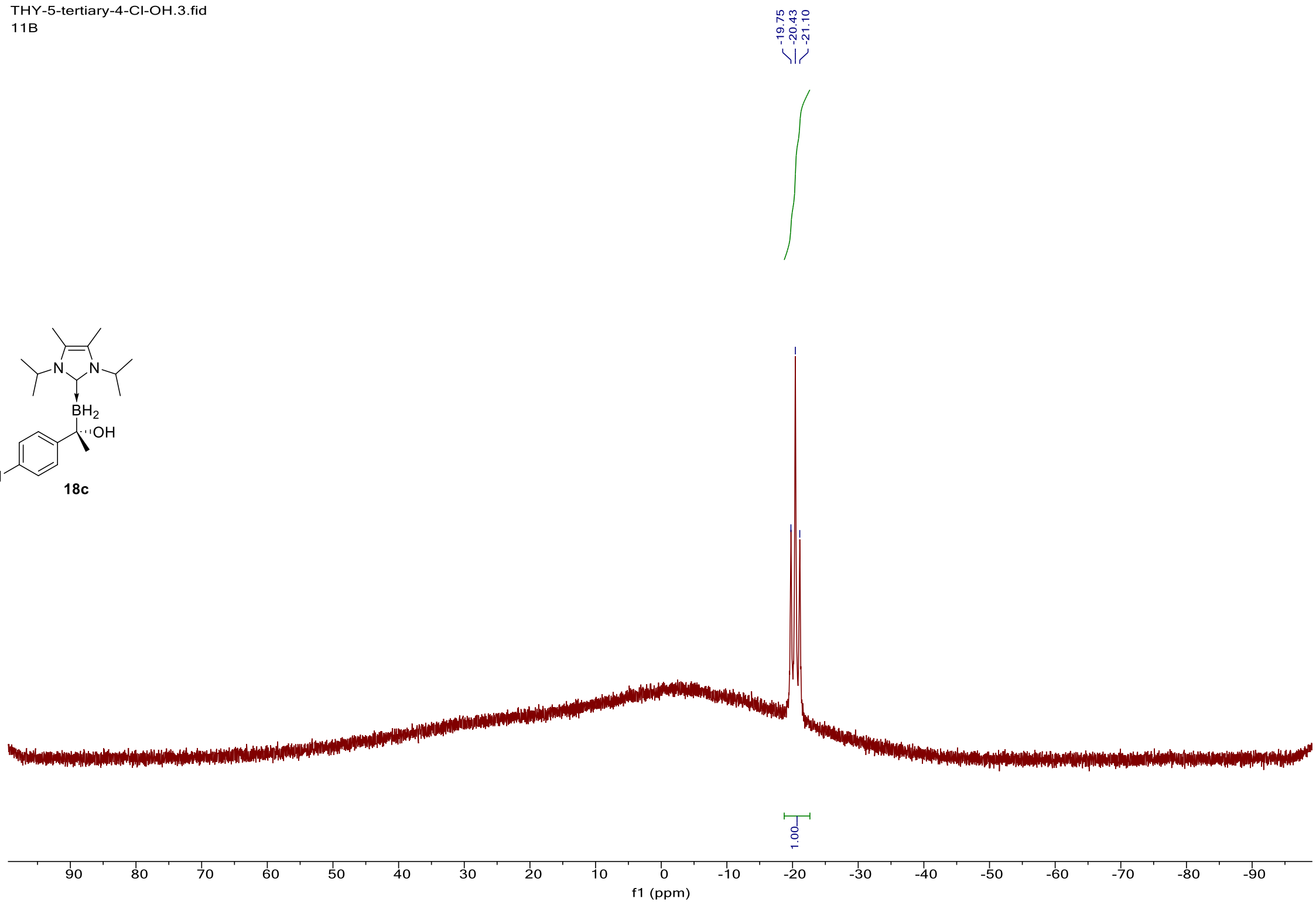

^{11}B NMR (128 MHz, $CDCl_3$)

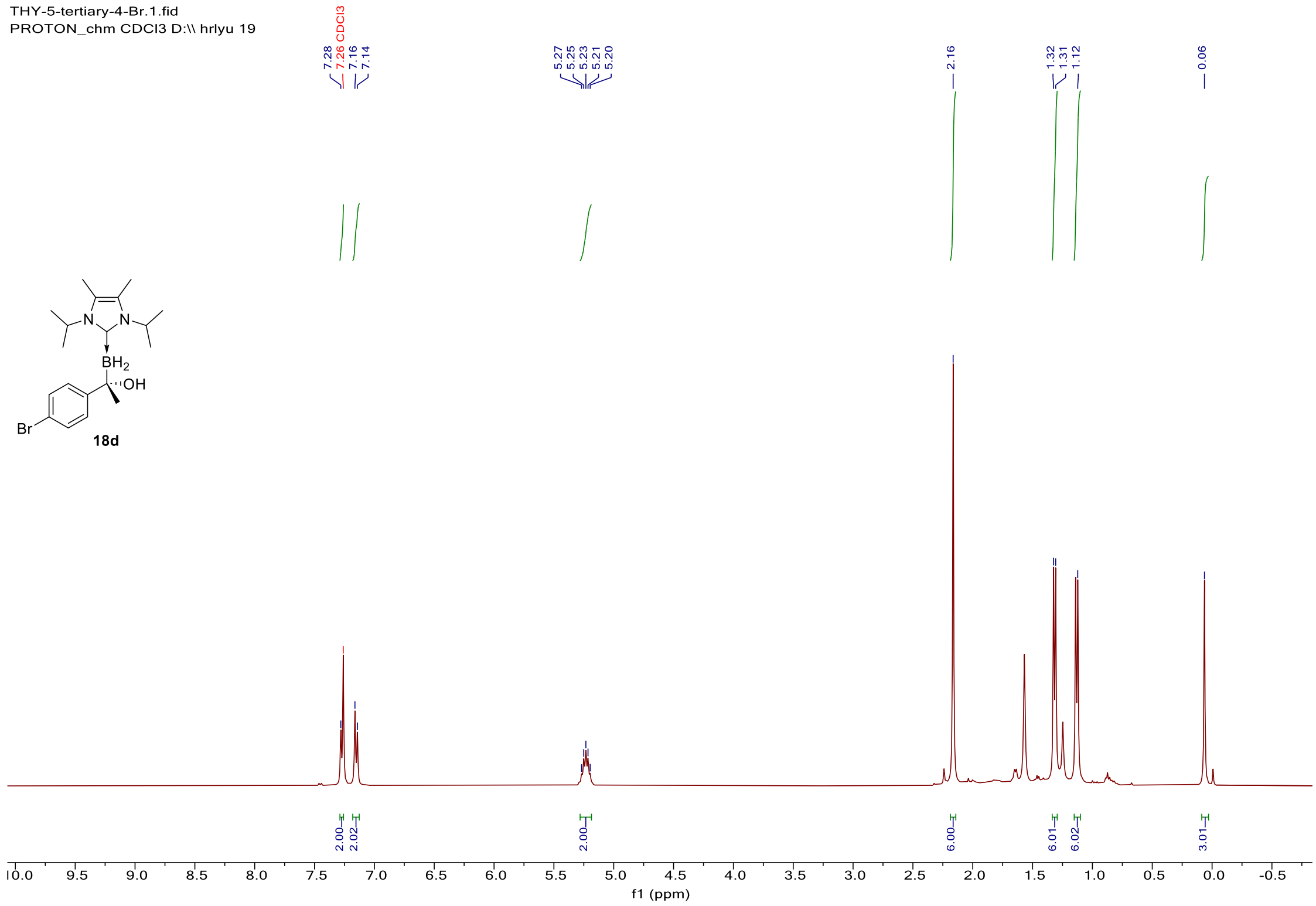

^{1}H NMR (400 MHz, $CDCl_3$)

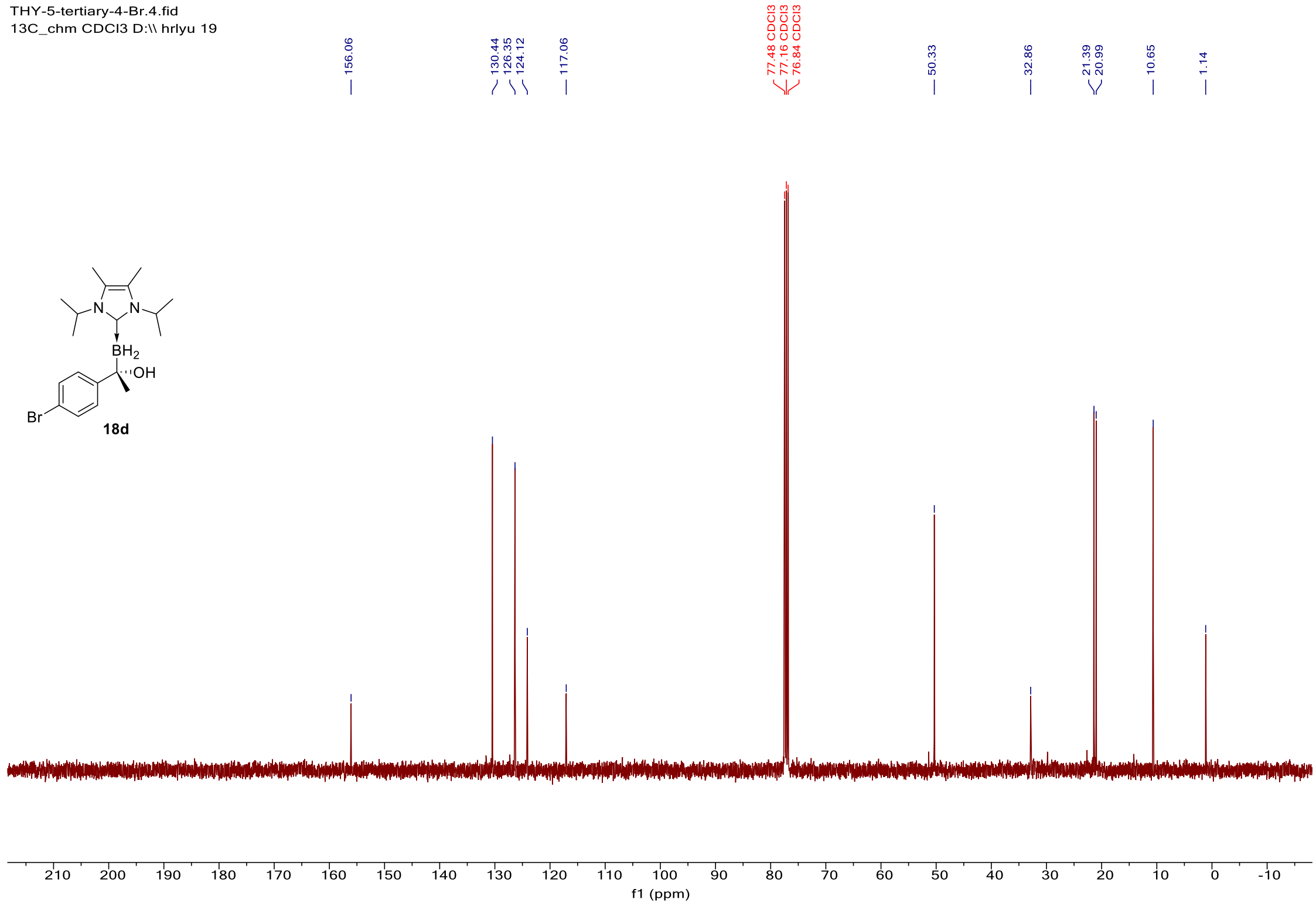

^{13}C NMR (101 MHz, CDCl$_3$)

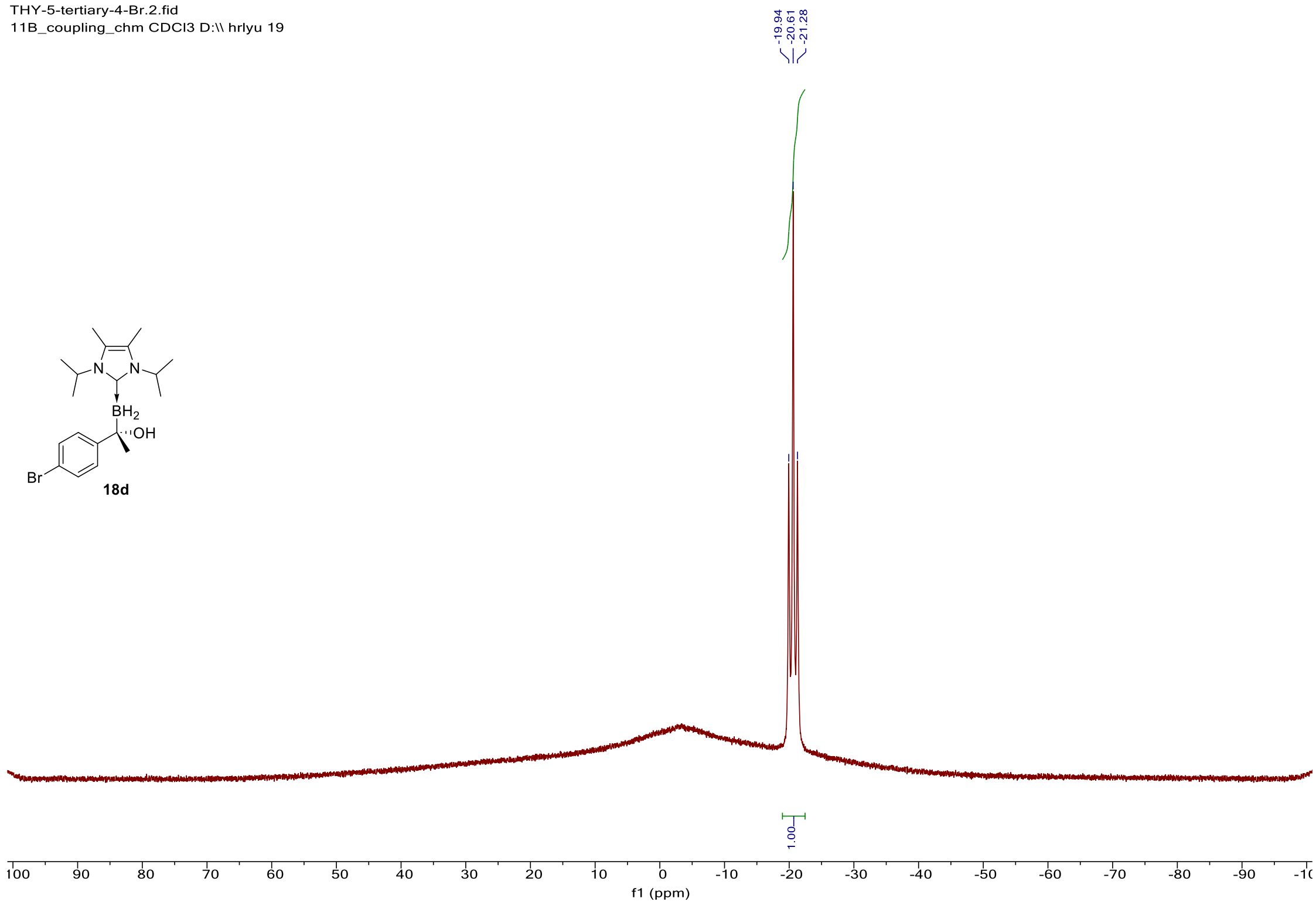

^{11}B NMR (128 MHz, CDCl$_3$)

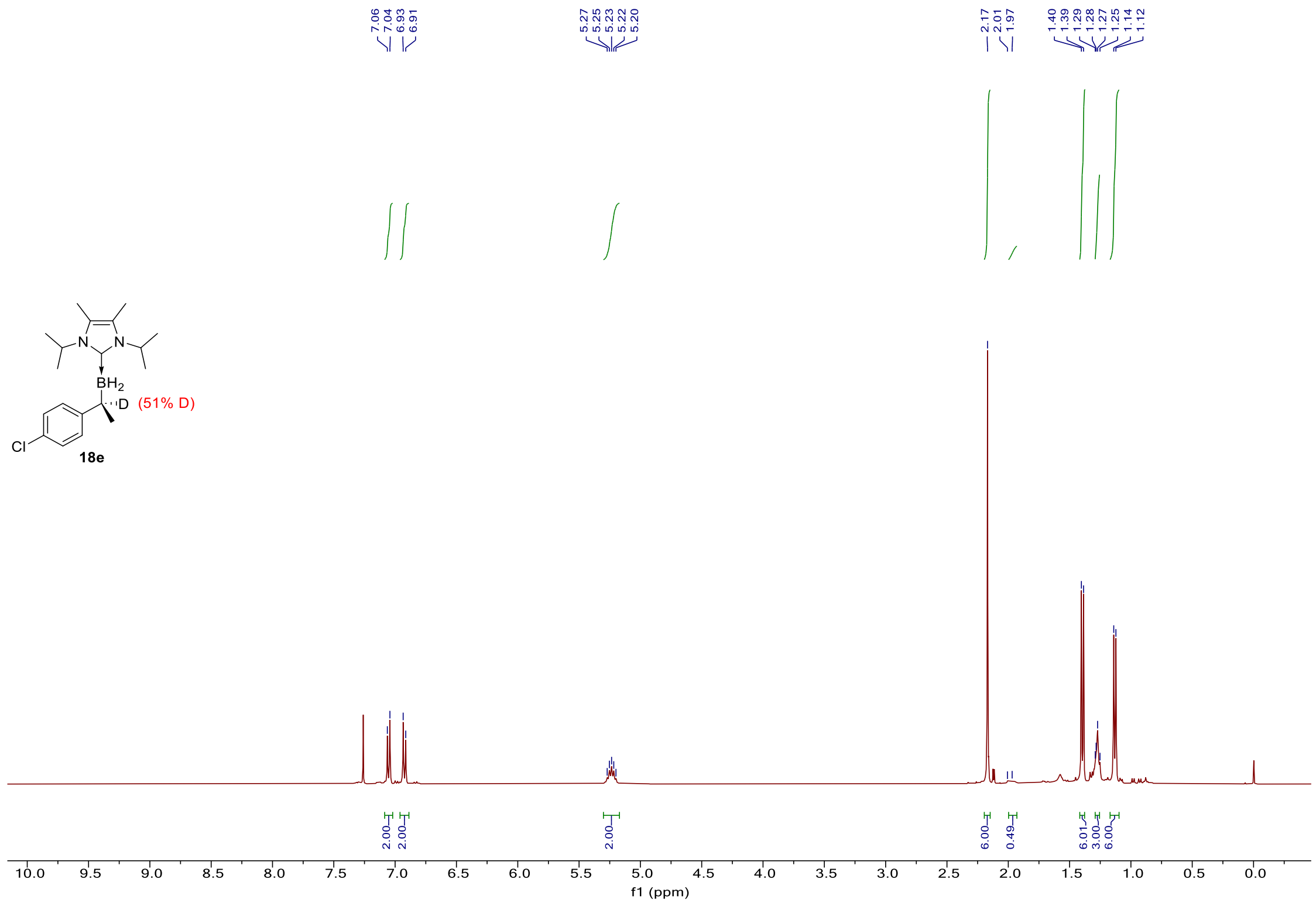

^1H NMR (400 MHz, $CDCl_3$)

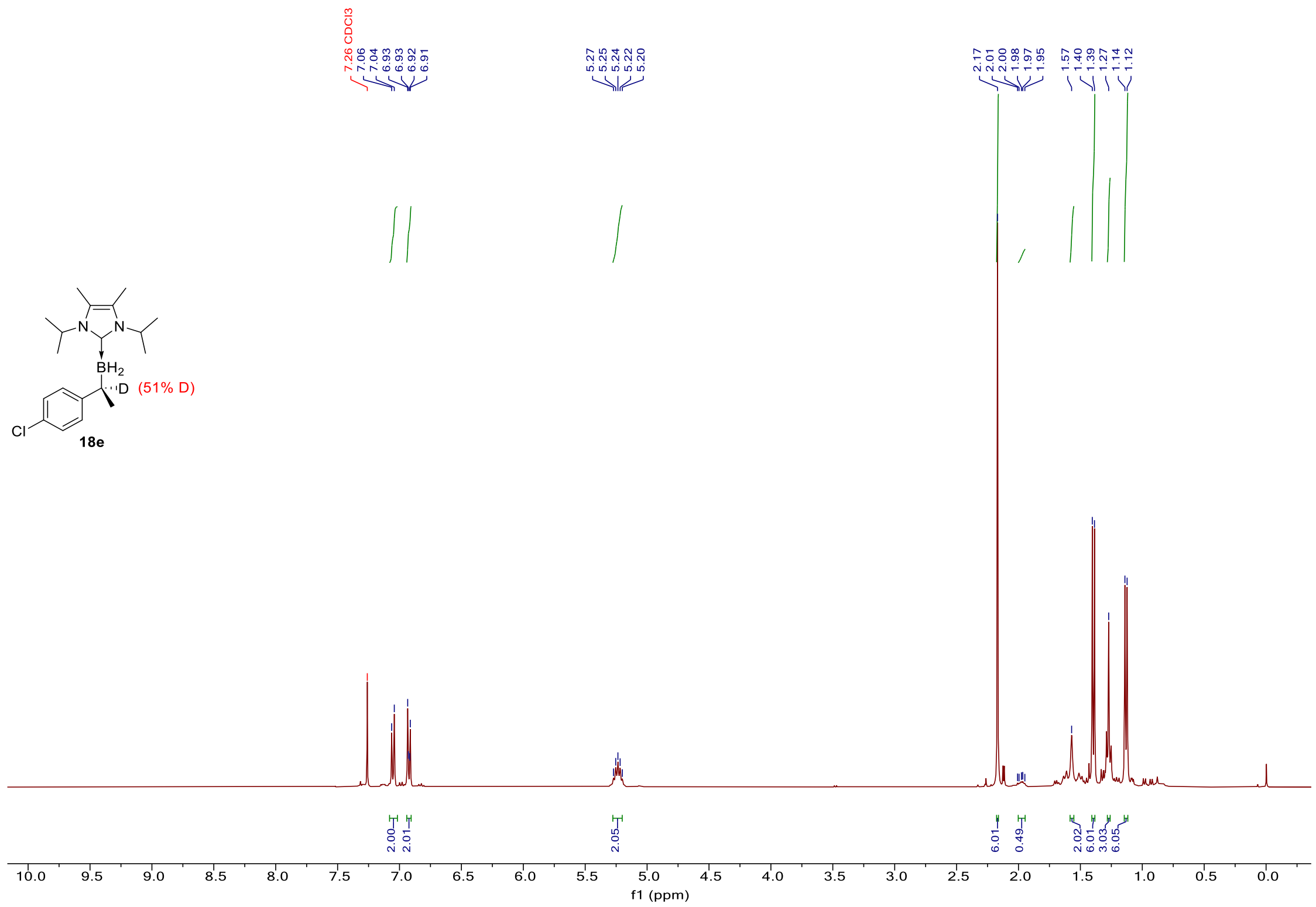

$^1H\{^{11}B\}$ NMR (400 MHz, $CDCl_3$)

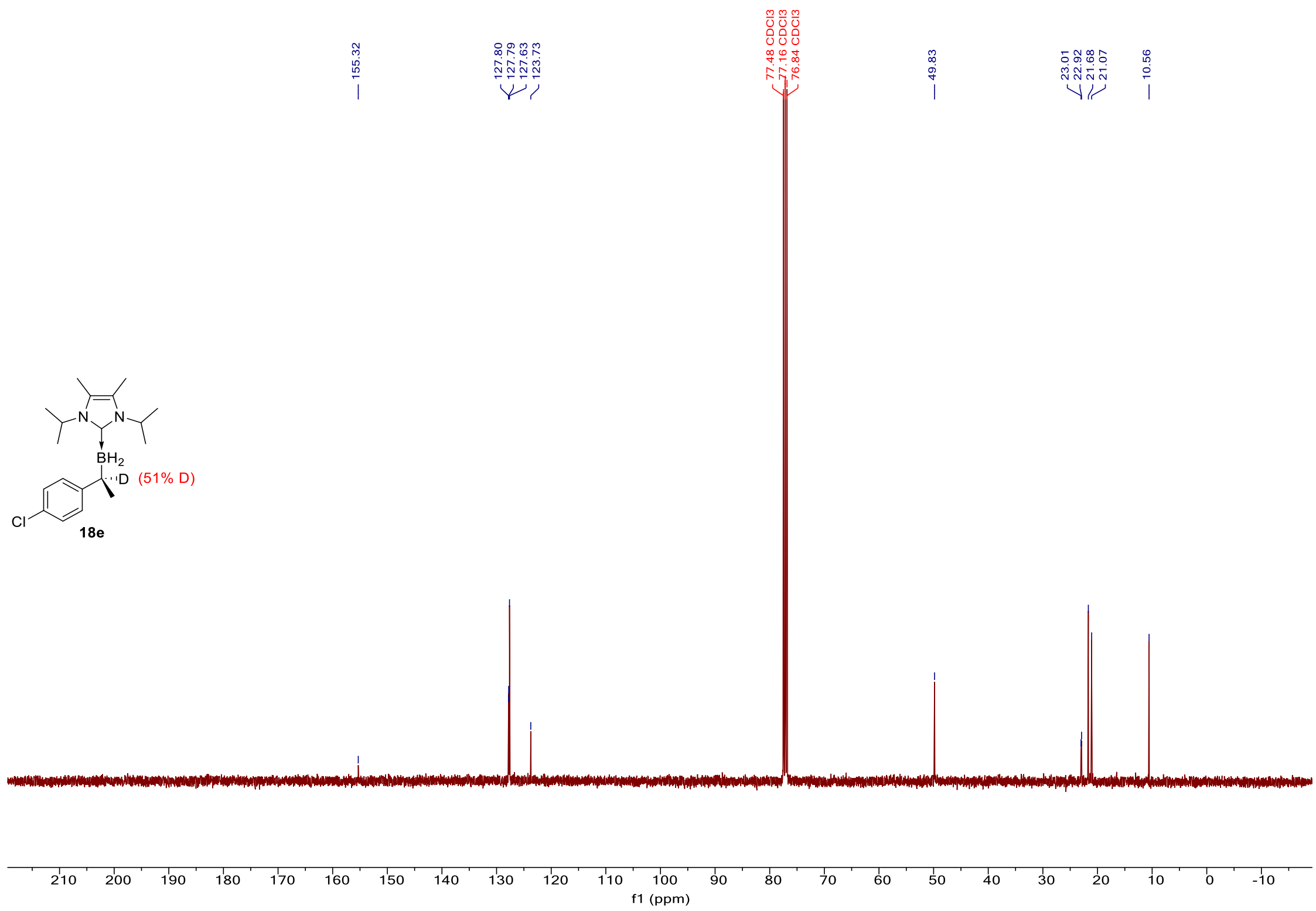

^{13}C NMR (101 MHz, $CDCl_3$)

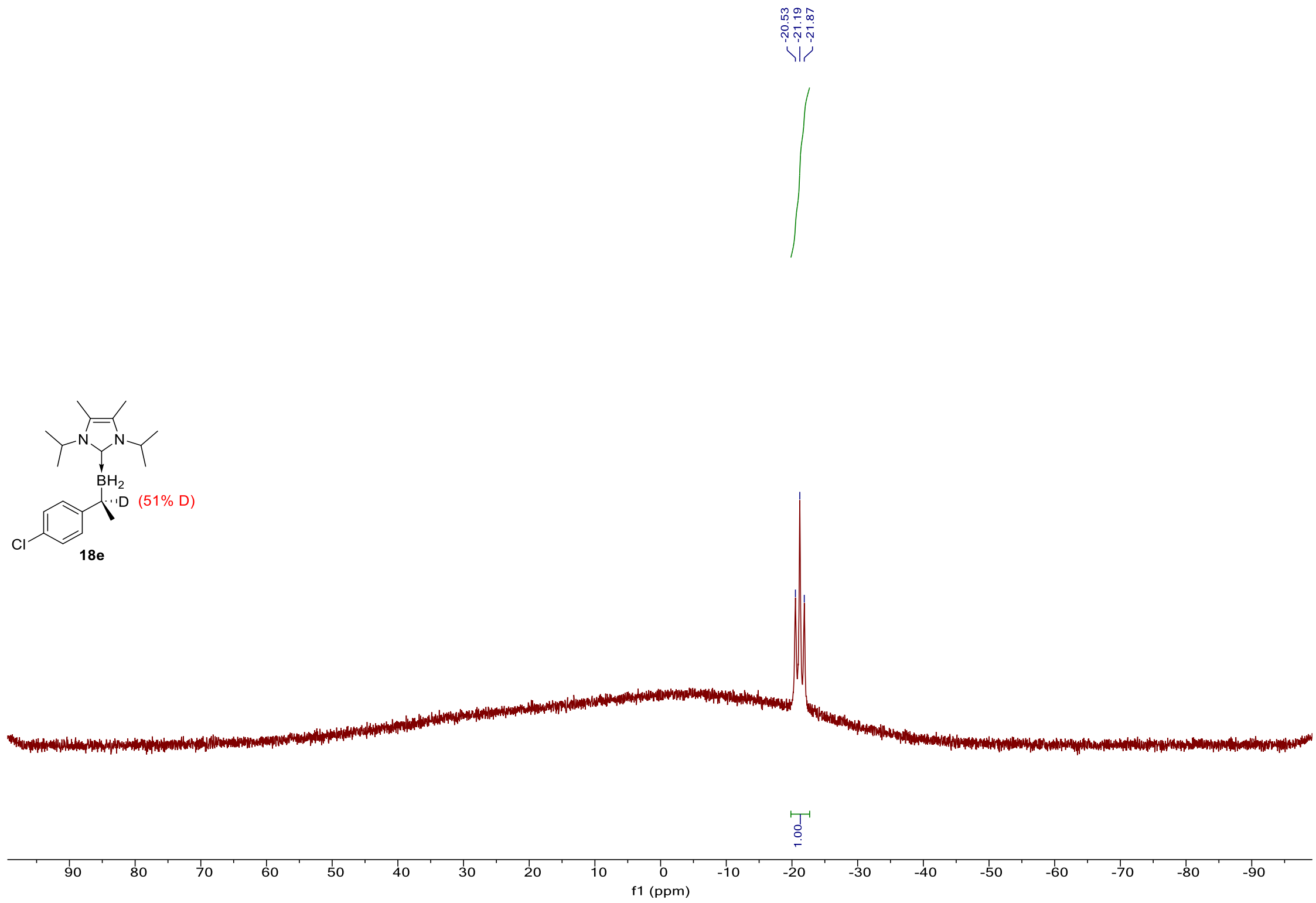

^{11}B NMR (128 MHz, $CDCl_3$)

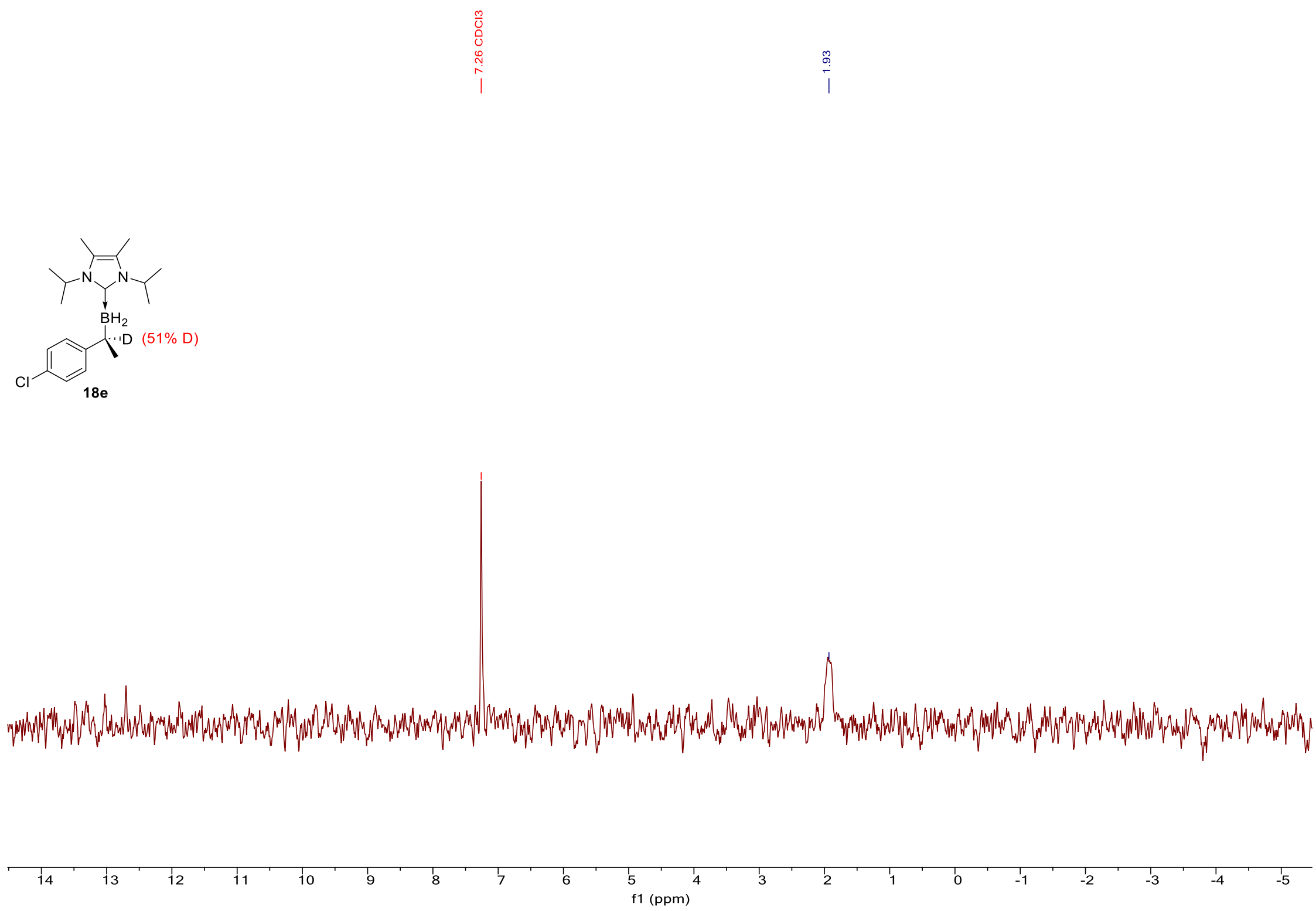

^{2}H NMR (61 MHz, $CDCl_3$)

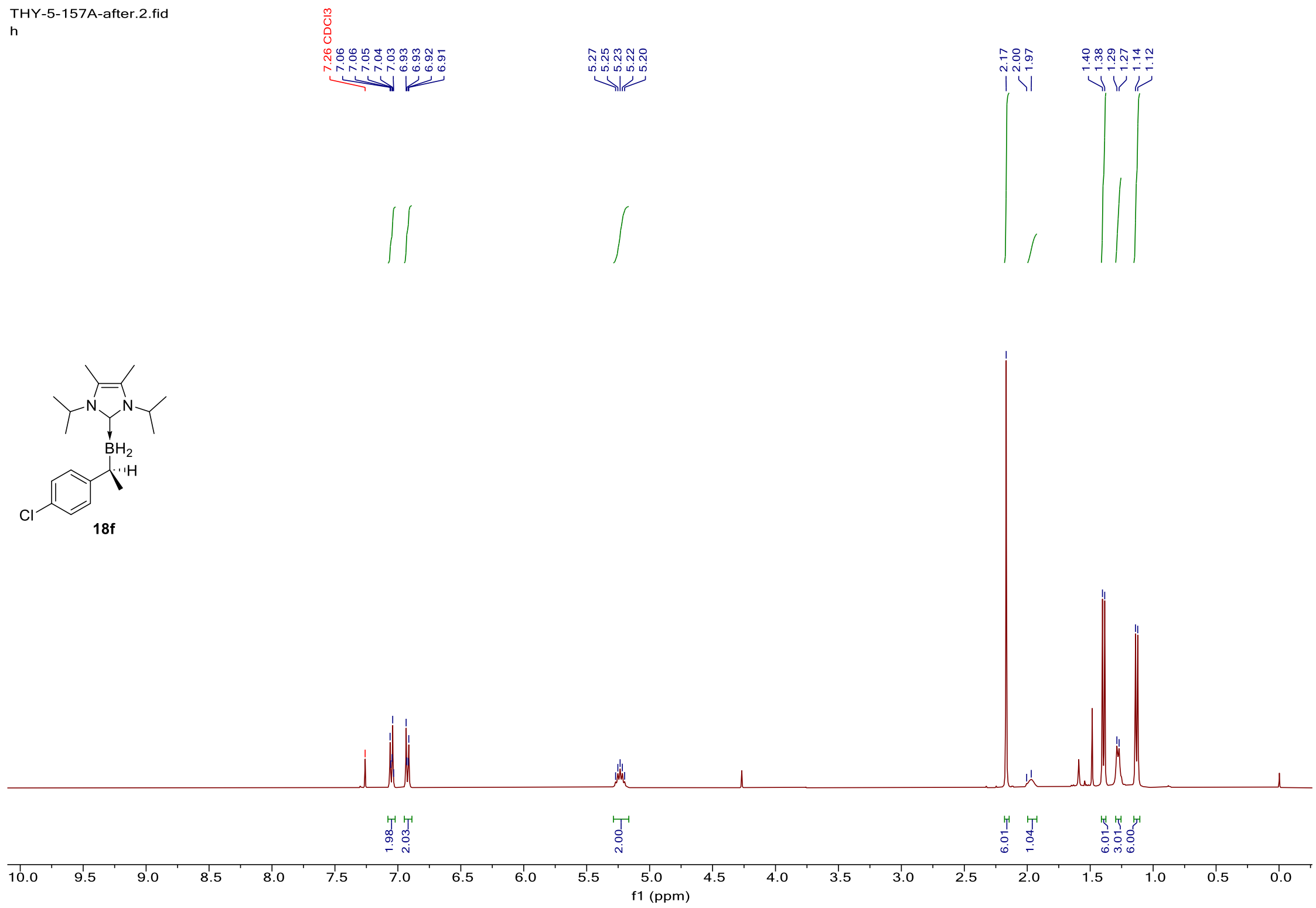

^{1}H NMR (400 MHz, $CDCl_3$)

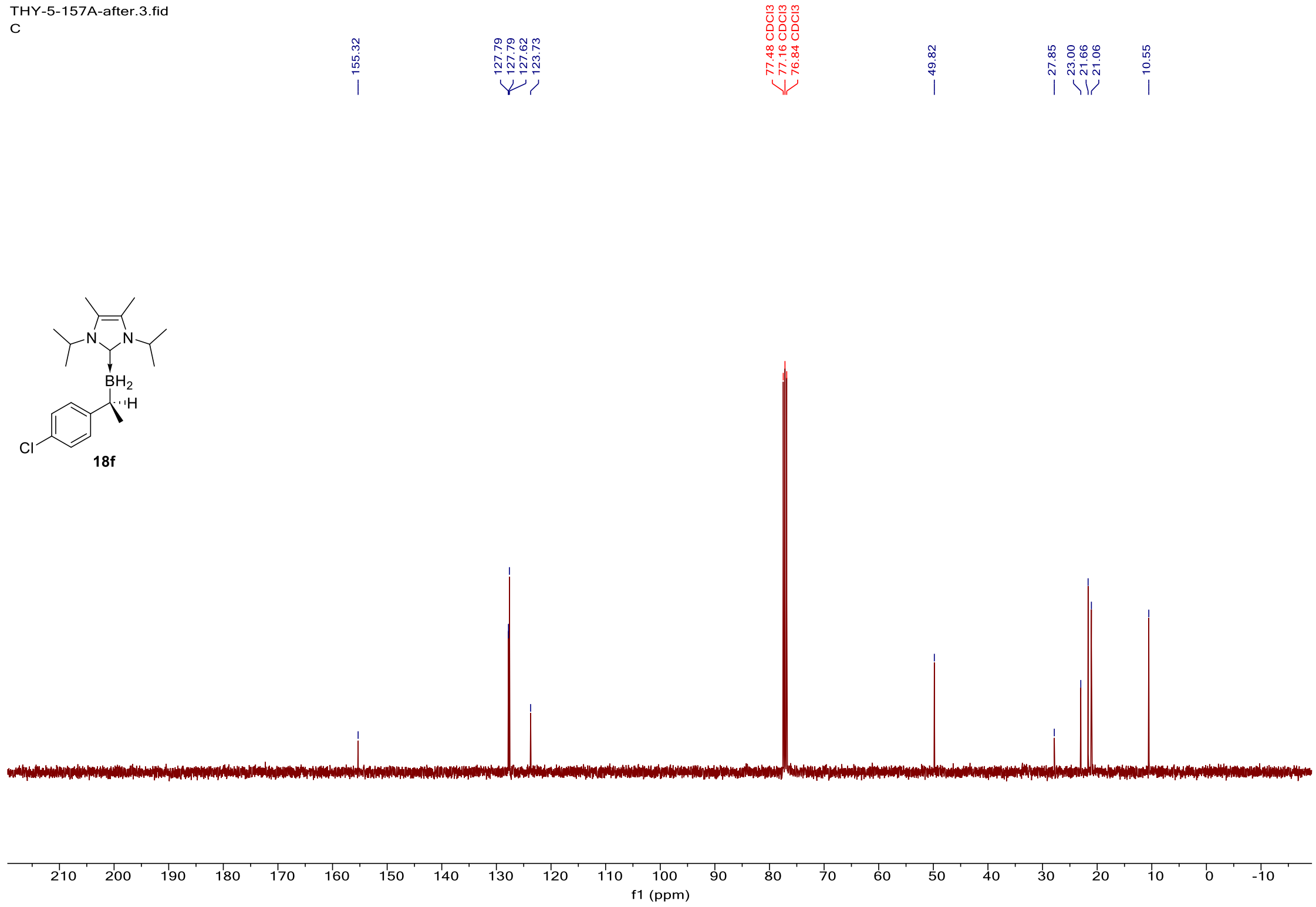

^{13}C NMR (101 MHz, $CDCl_3$)

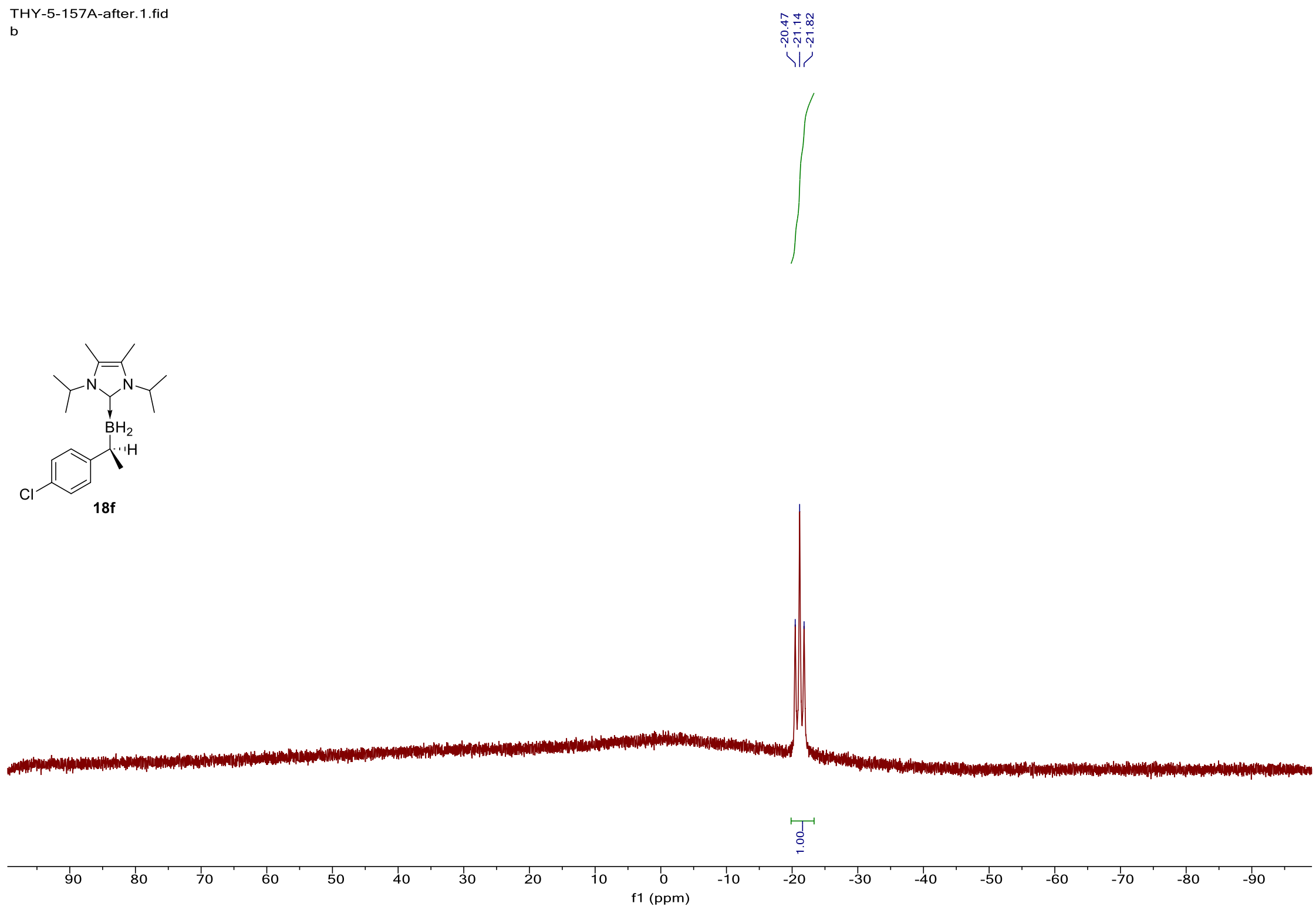

^{11}B NMR (128 MHz, $CDCl_3$)

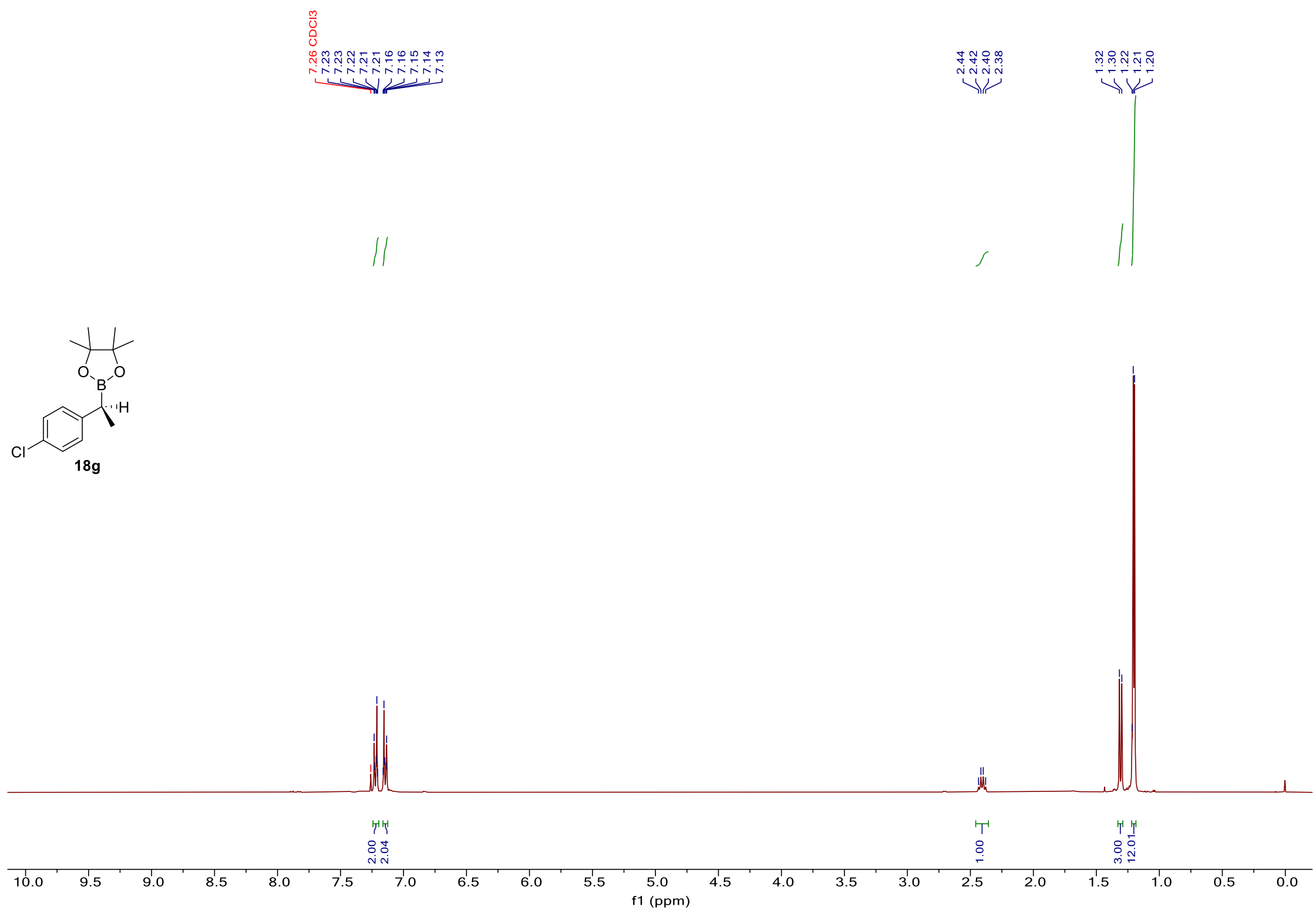

^{1}H NMR (400 MHz, $CDCl_3$)

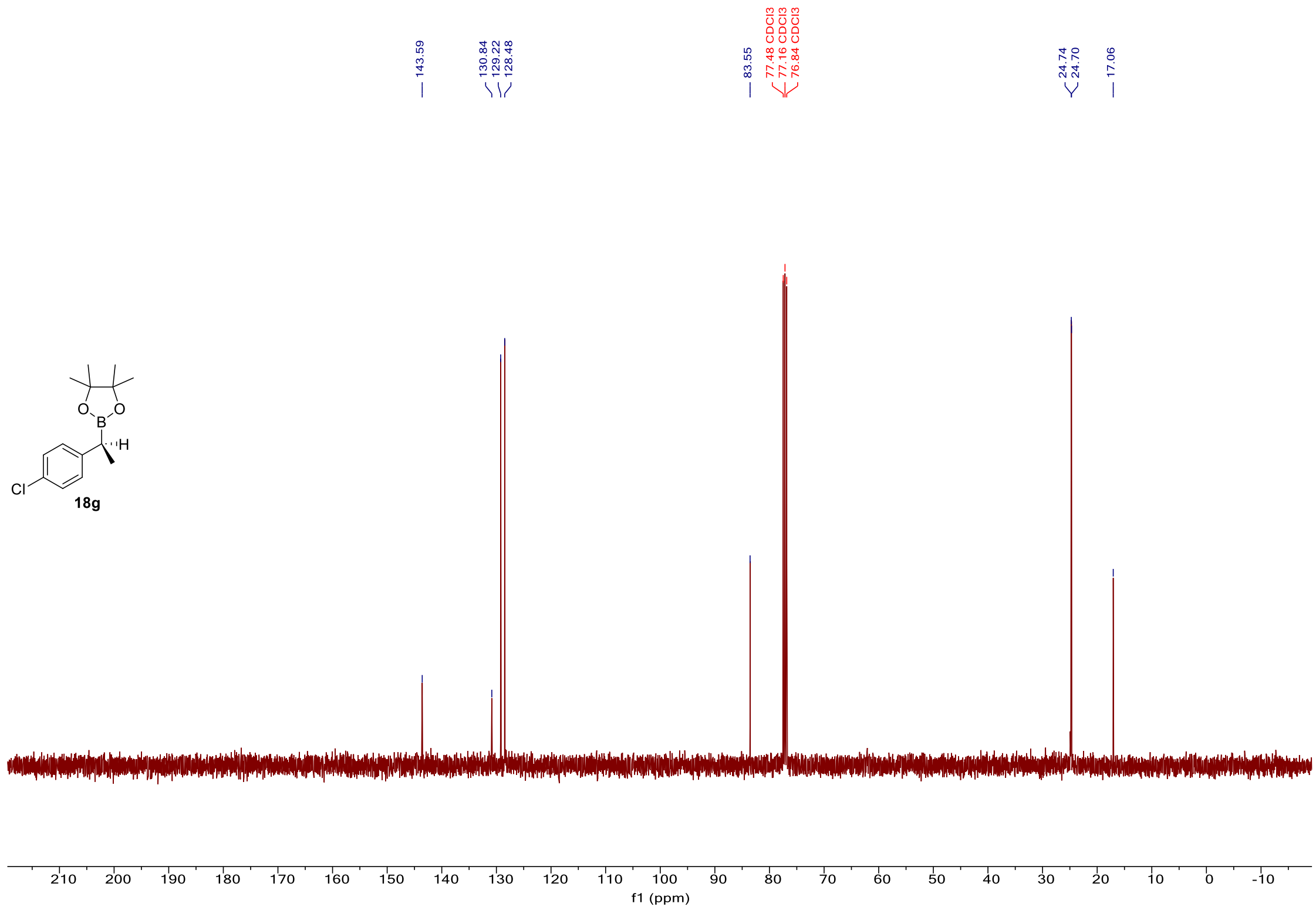

^{13}C NMR (101 MHz, $CDCl_3$)

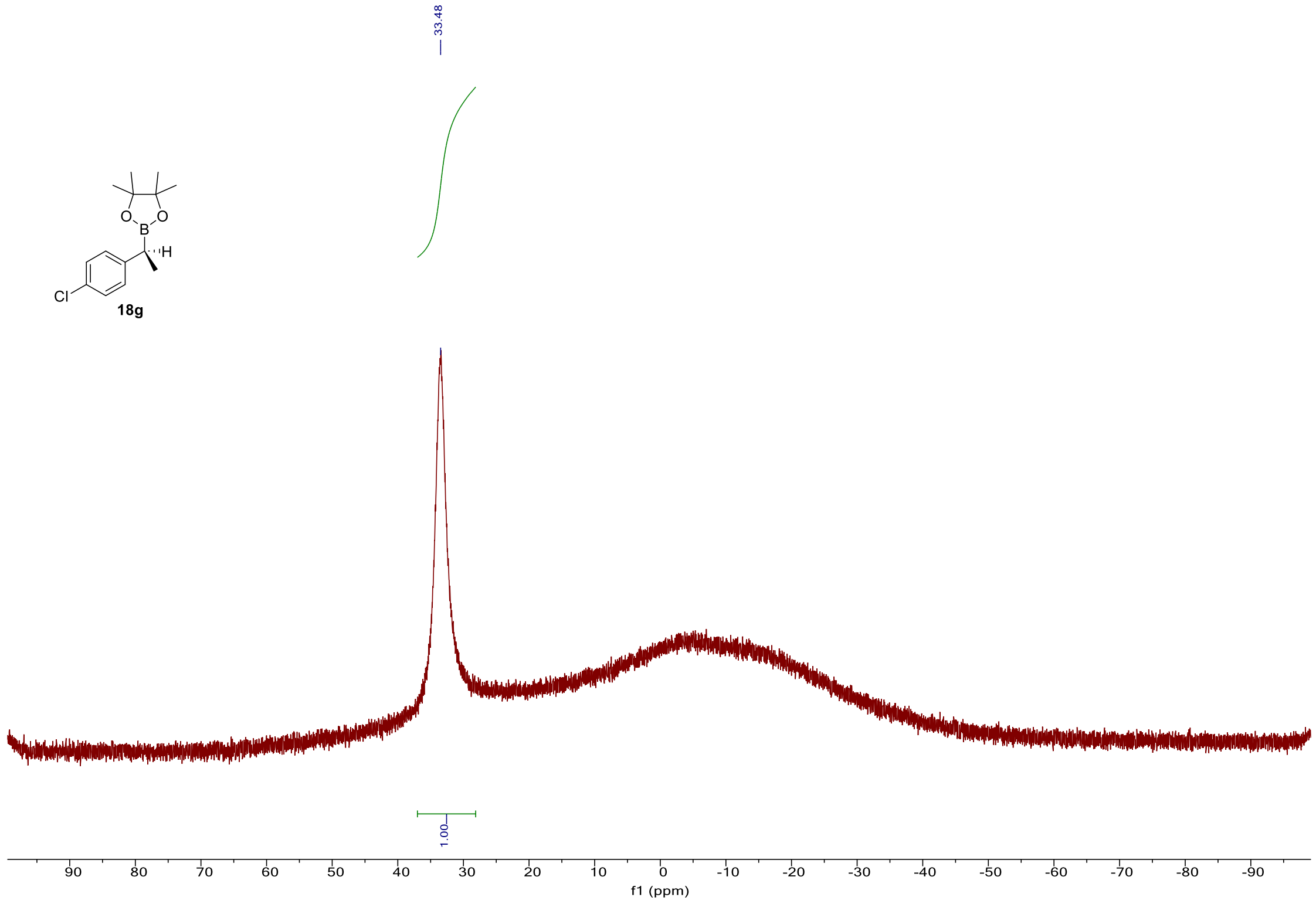

^{11}B NMR (128 MHz, $CDCl_3$)

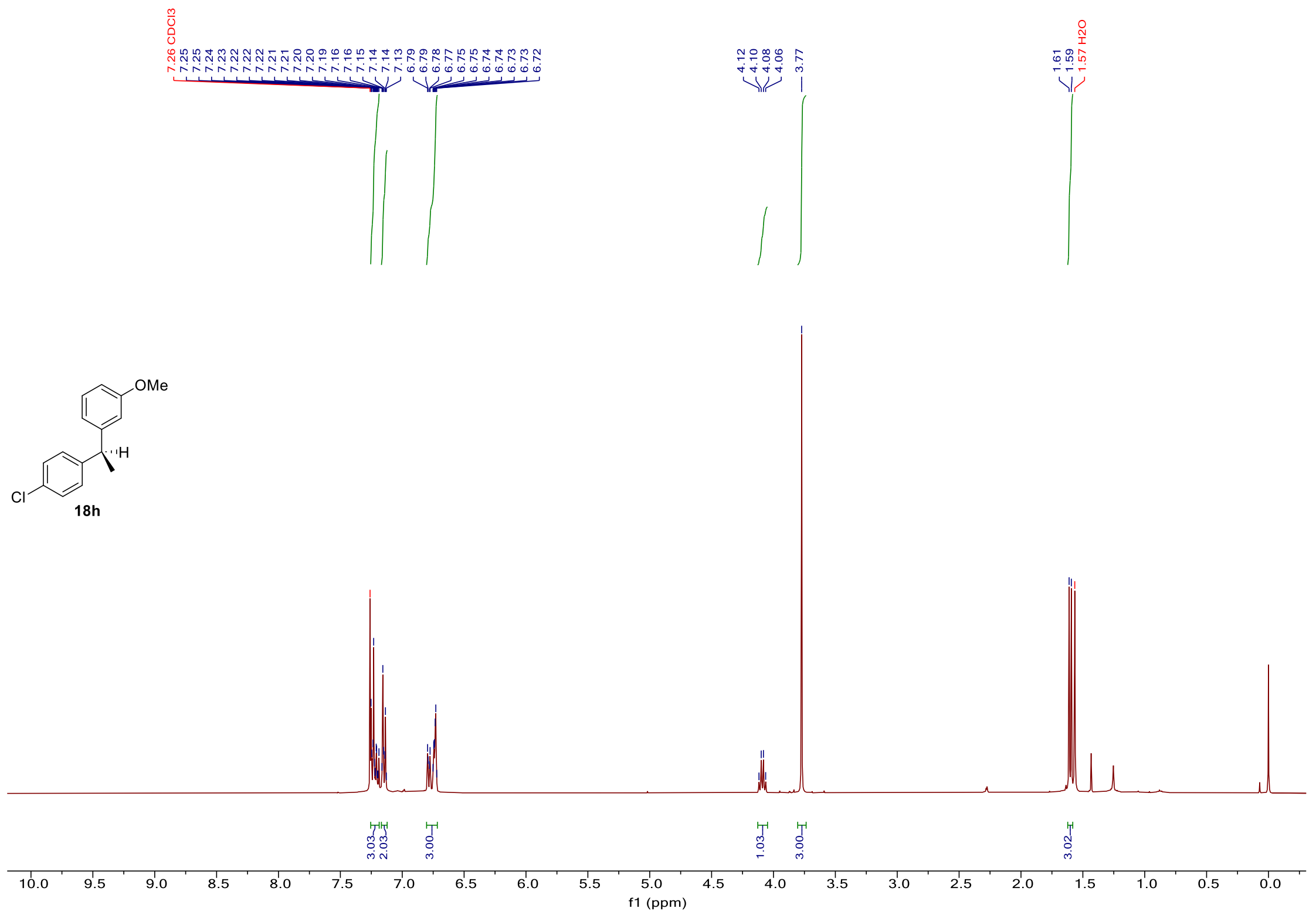

^{1}H NMR (400 MHz, $CDCl_3$)

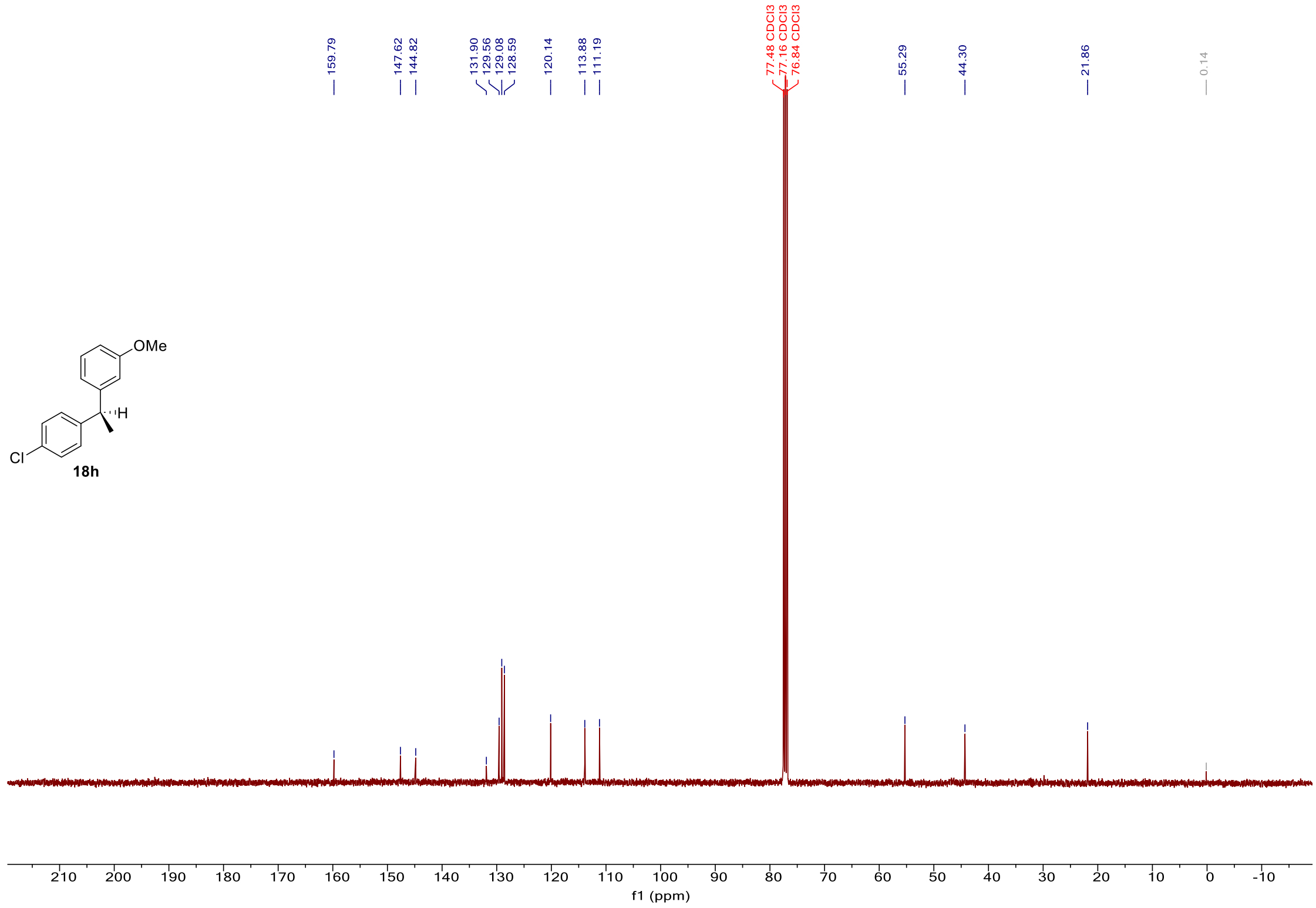

^{13}C NMR (101 MHz, $CDCl_3$)

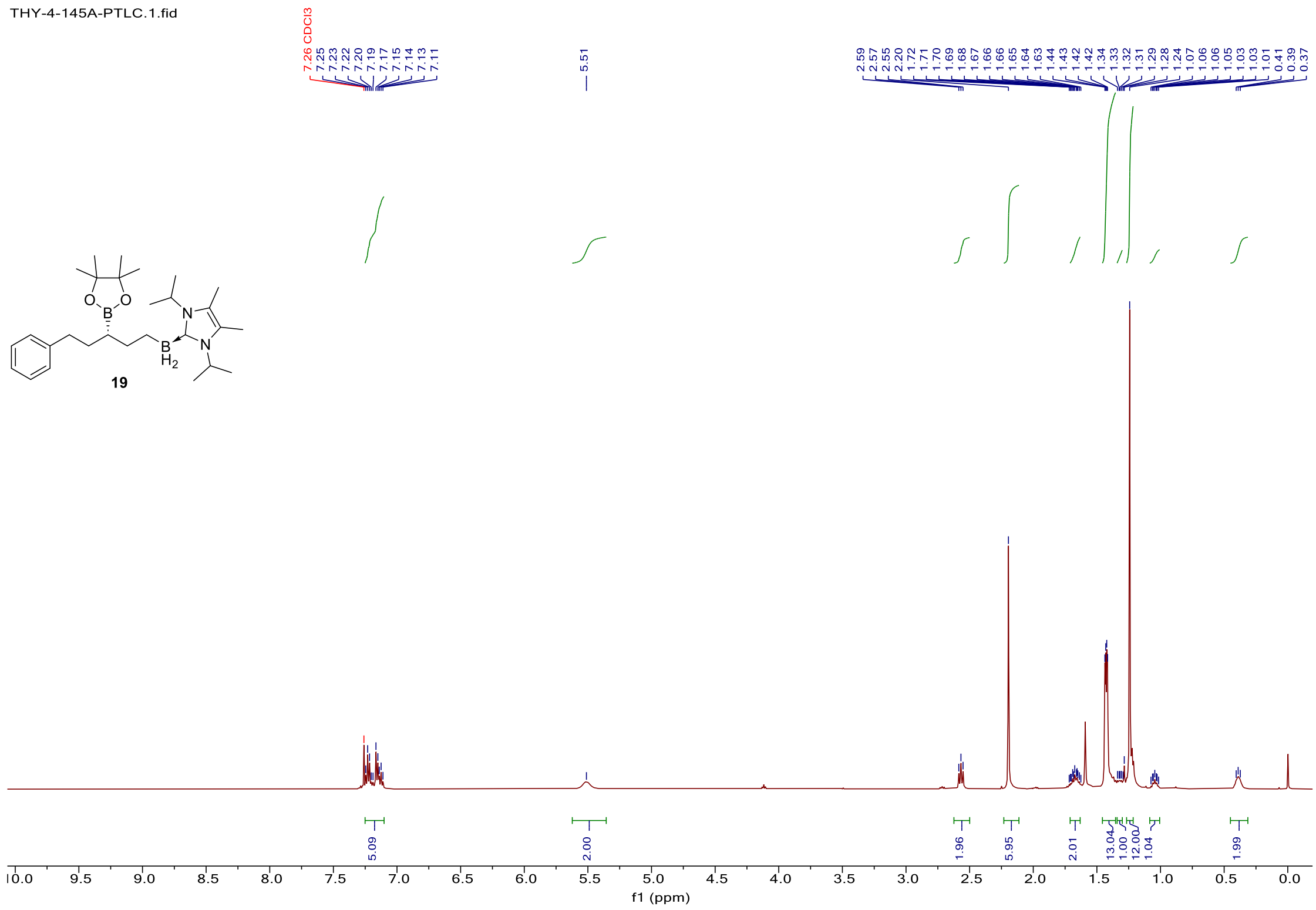

^{1}H NMR (500 MHz, $CDCl_3$)

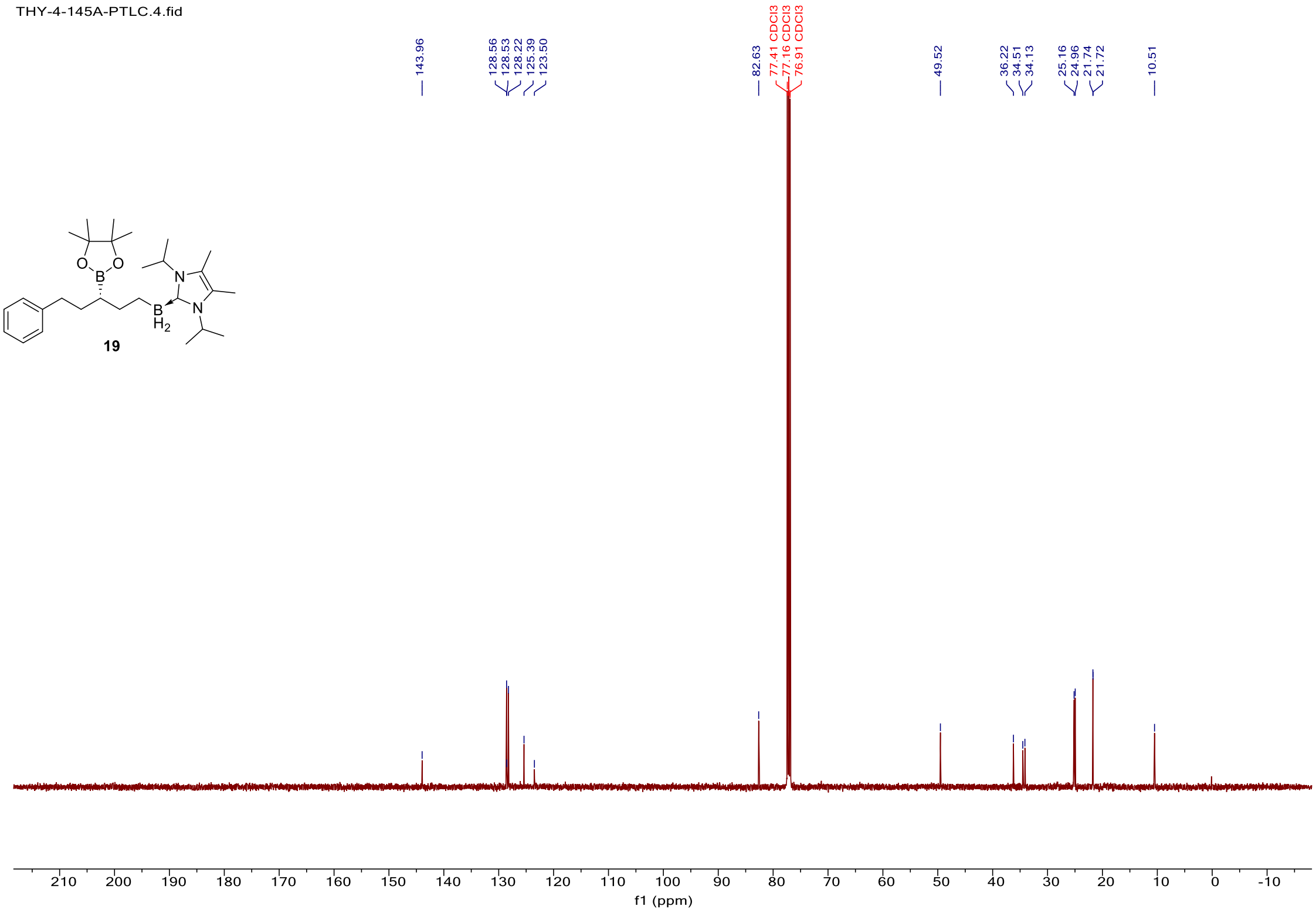

^{13}C NMR (126 MHz, $CDCl_3$)

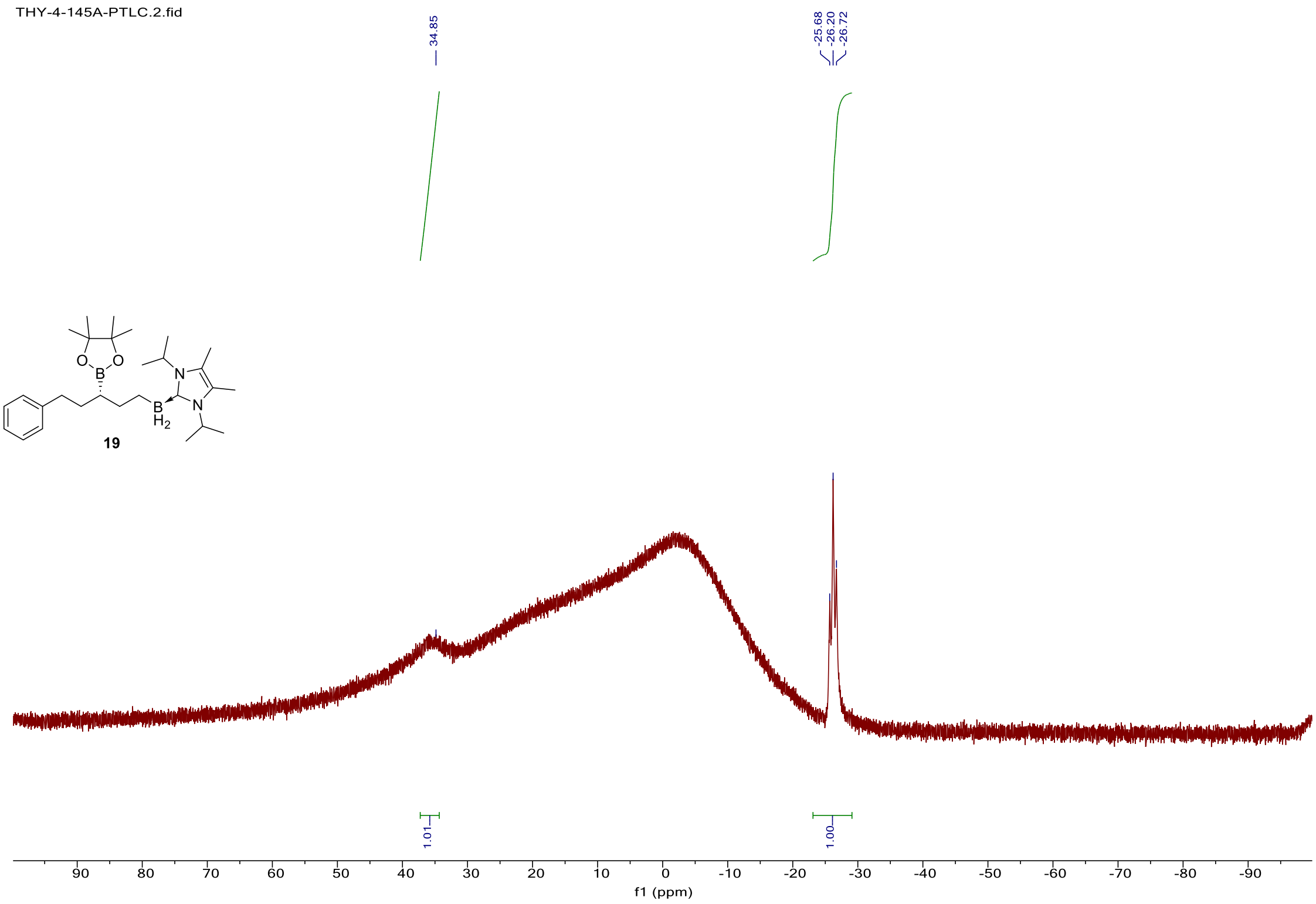

^{11}B NMR (160 MHz, $CDCl_3$)

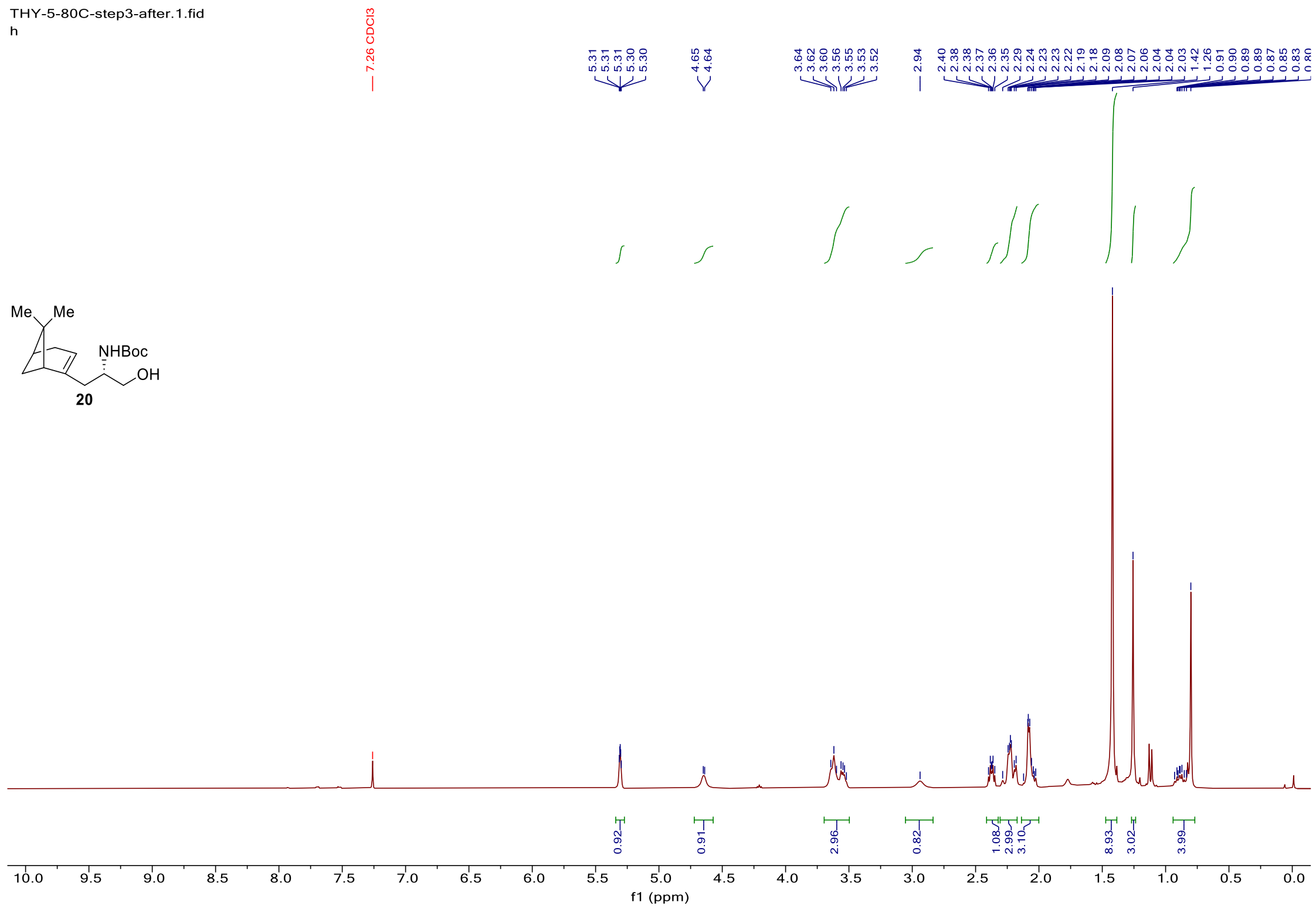

^{1}H NMR (400 MHz, $CDCl_3$)

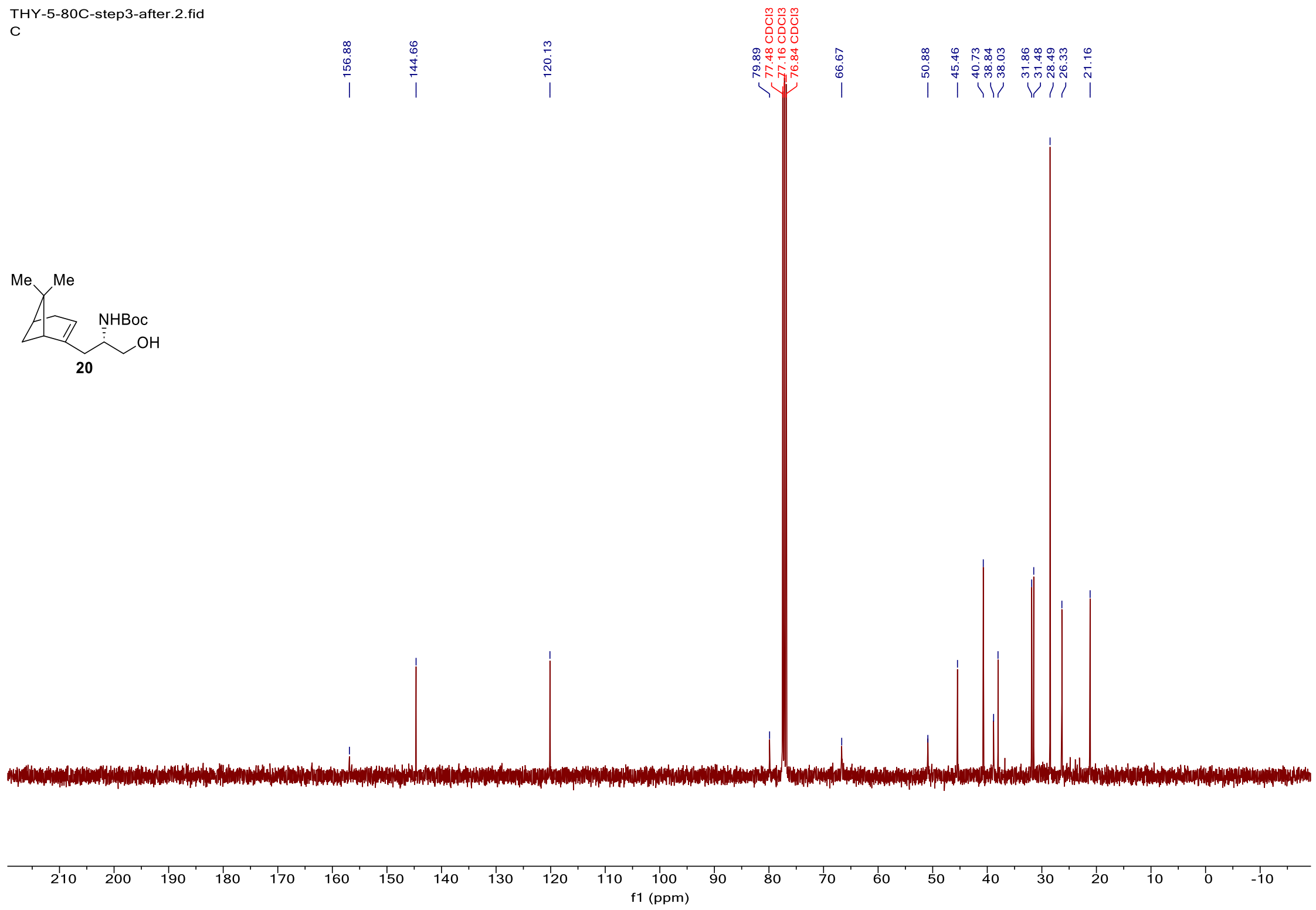

^{13}C NMR (101 MHz, $CDCl_3$)

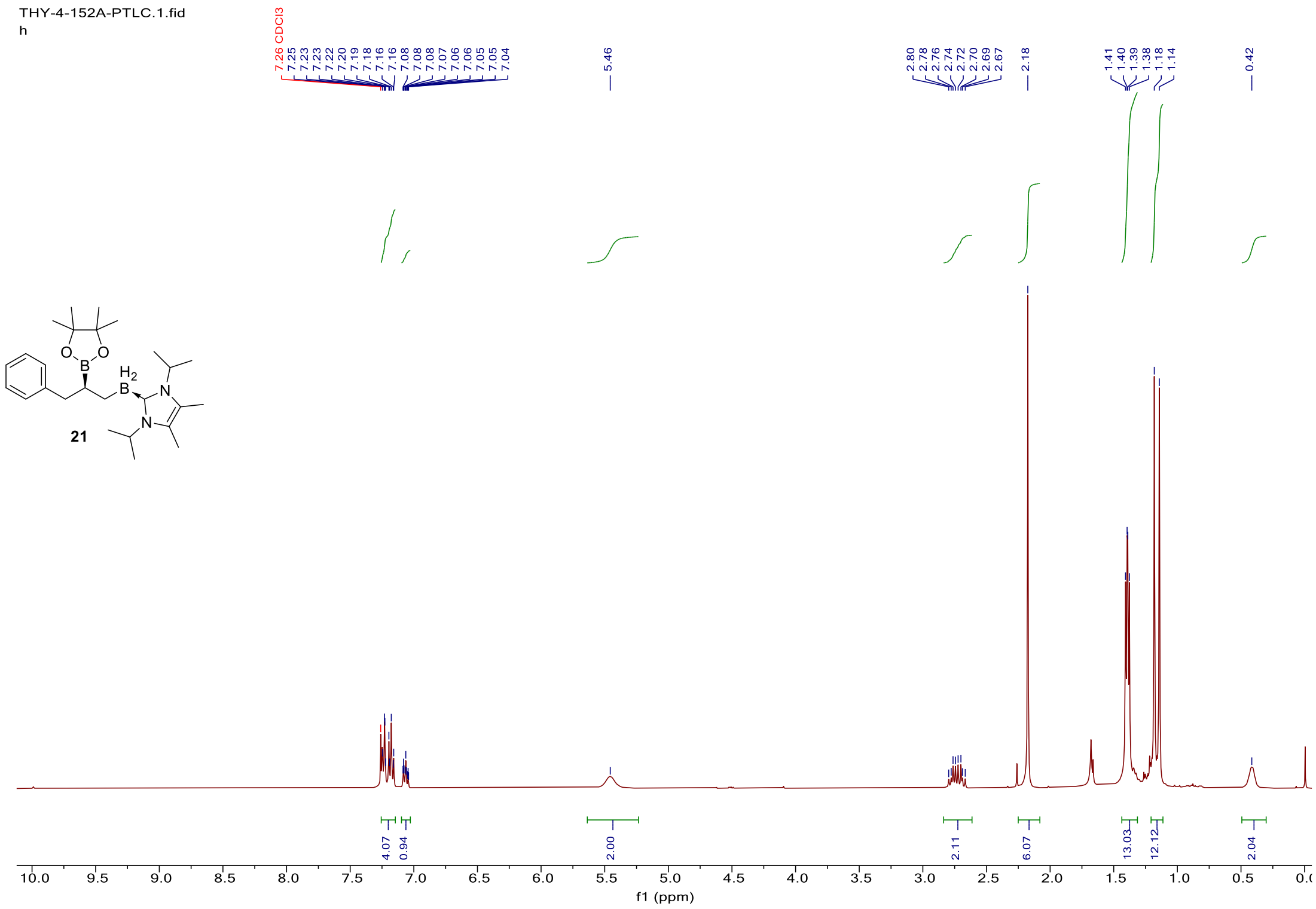

^{1}H NMR (400 MHz, CDCl3)

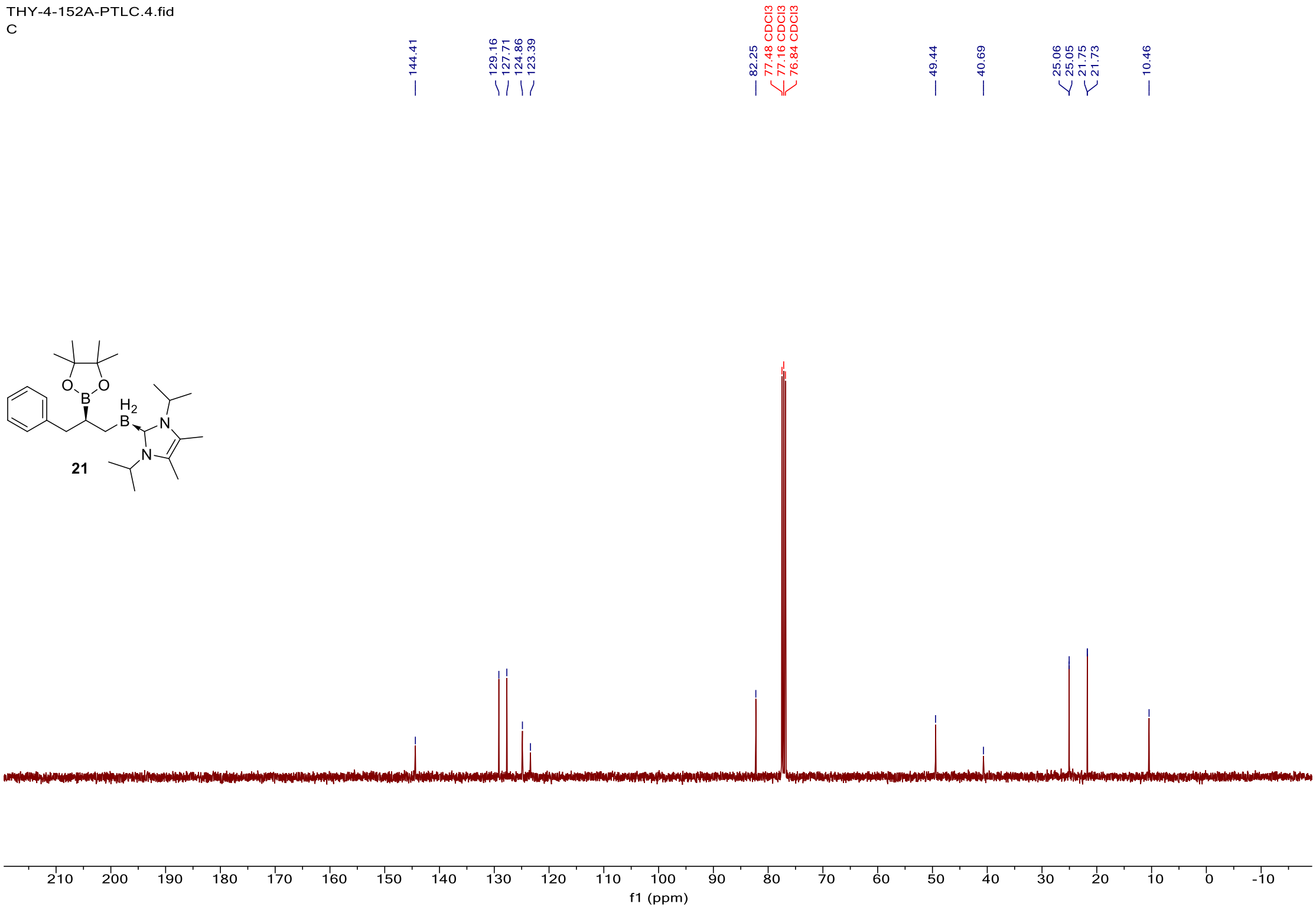

^{13}C NMR (101 MHz, CDCl3)

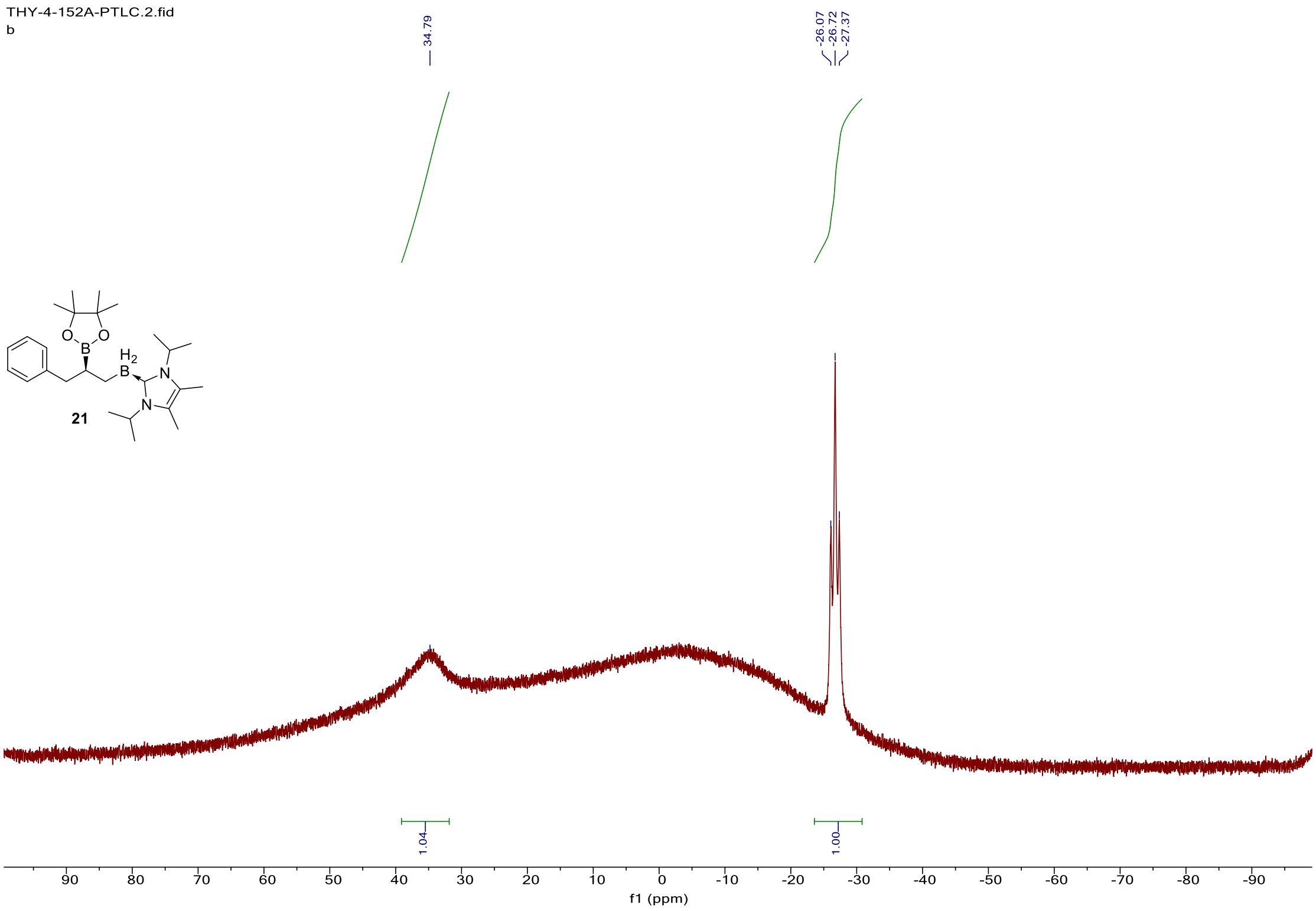

^{11}B NMR (128 MHz, $CDCl_3$)

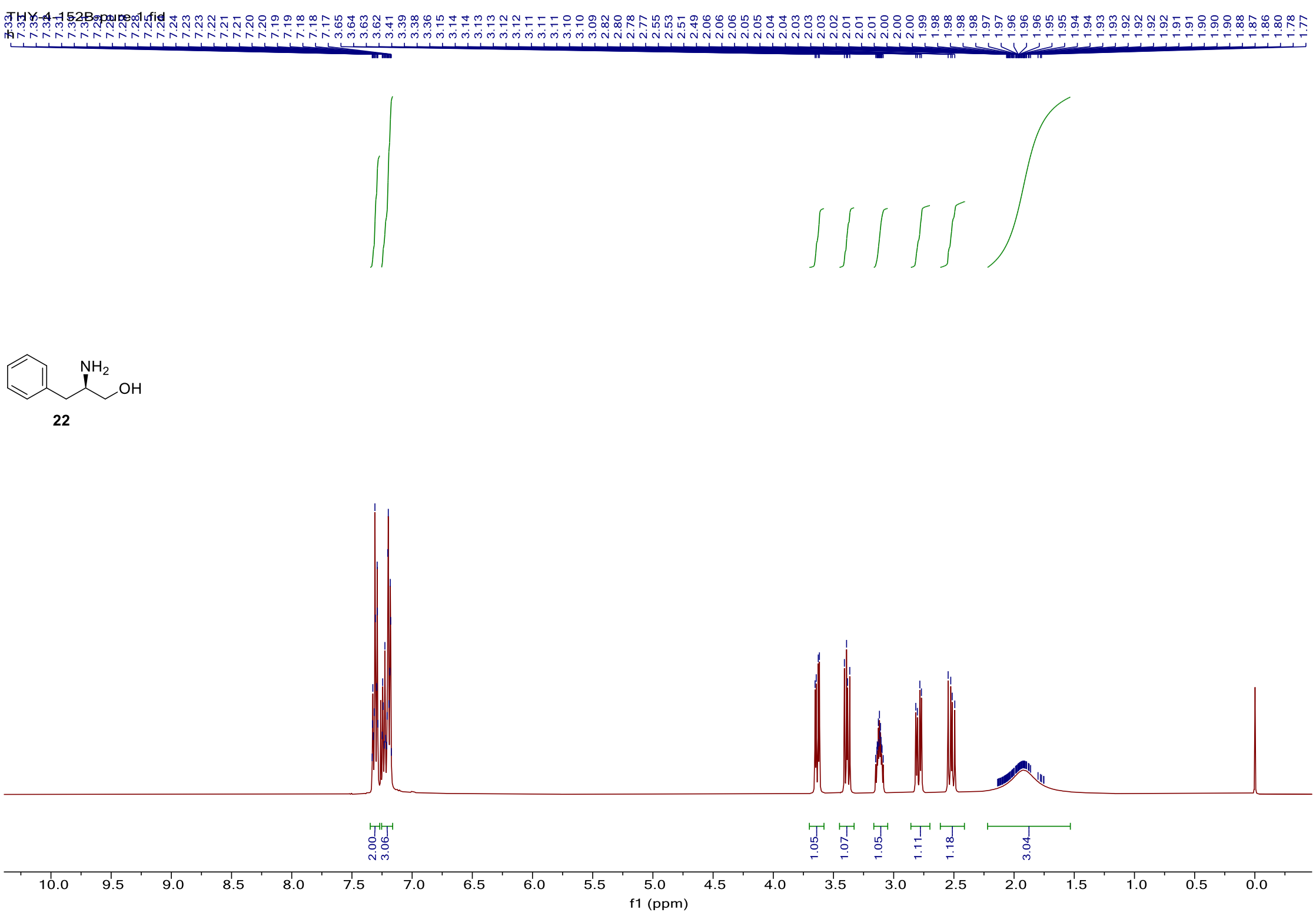

^{1}H NMR (400 MHz, $CDCl_3$)

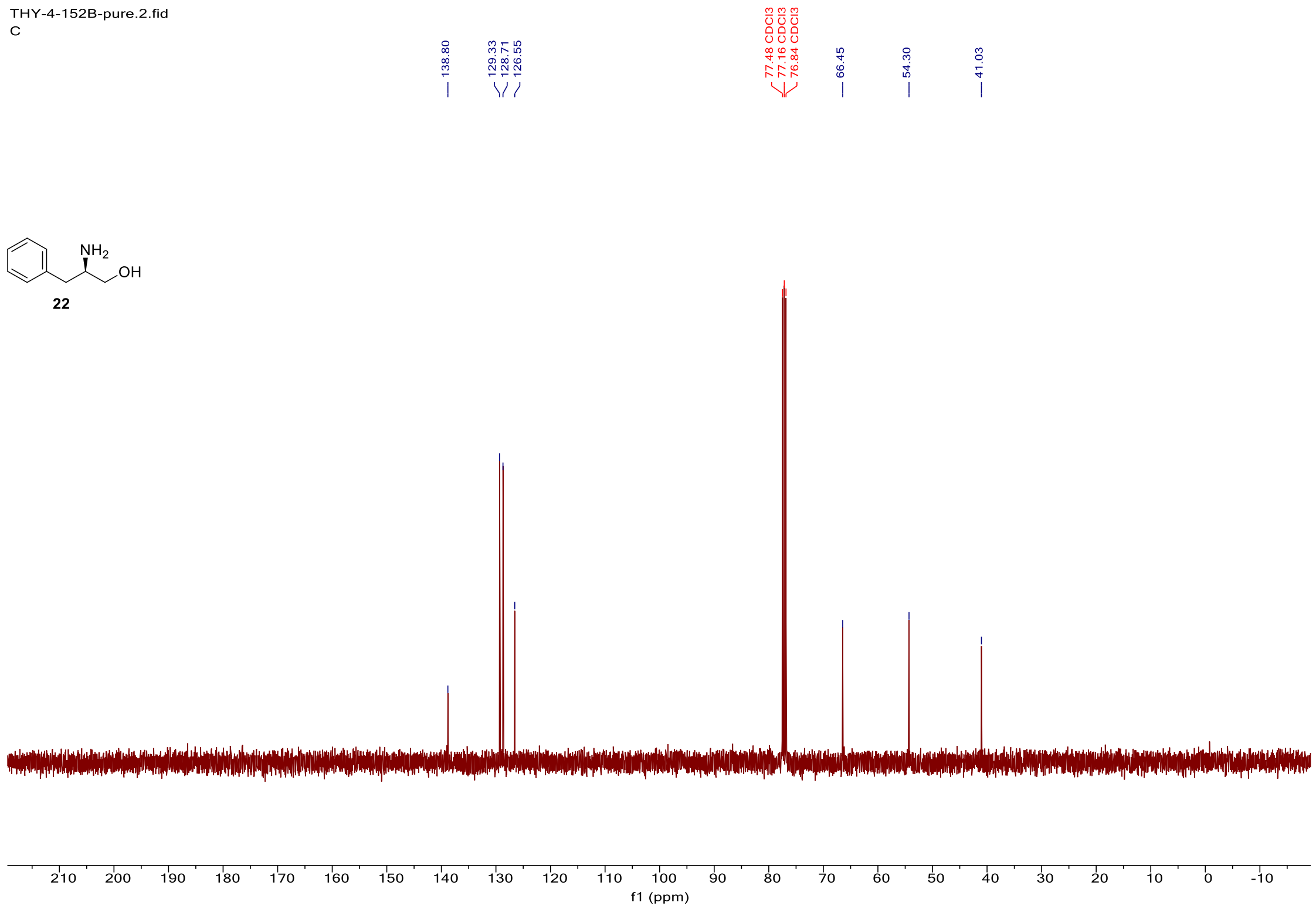

^{13}C NMR (101 MHz, $CDCl_3$)

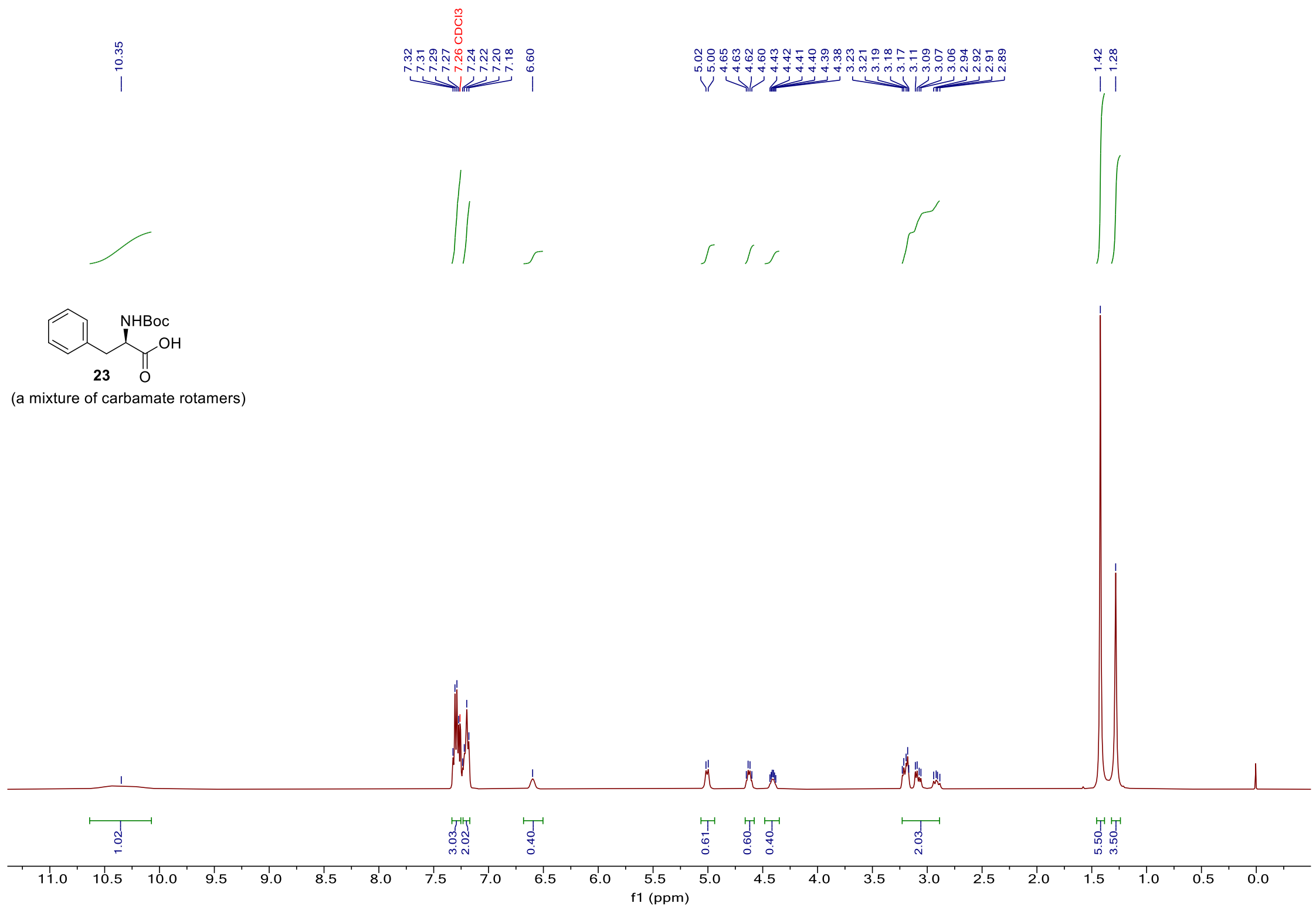

^{1}H NMR (400 MHz, $CDCl_3$)

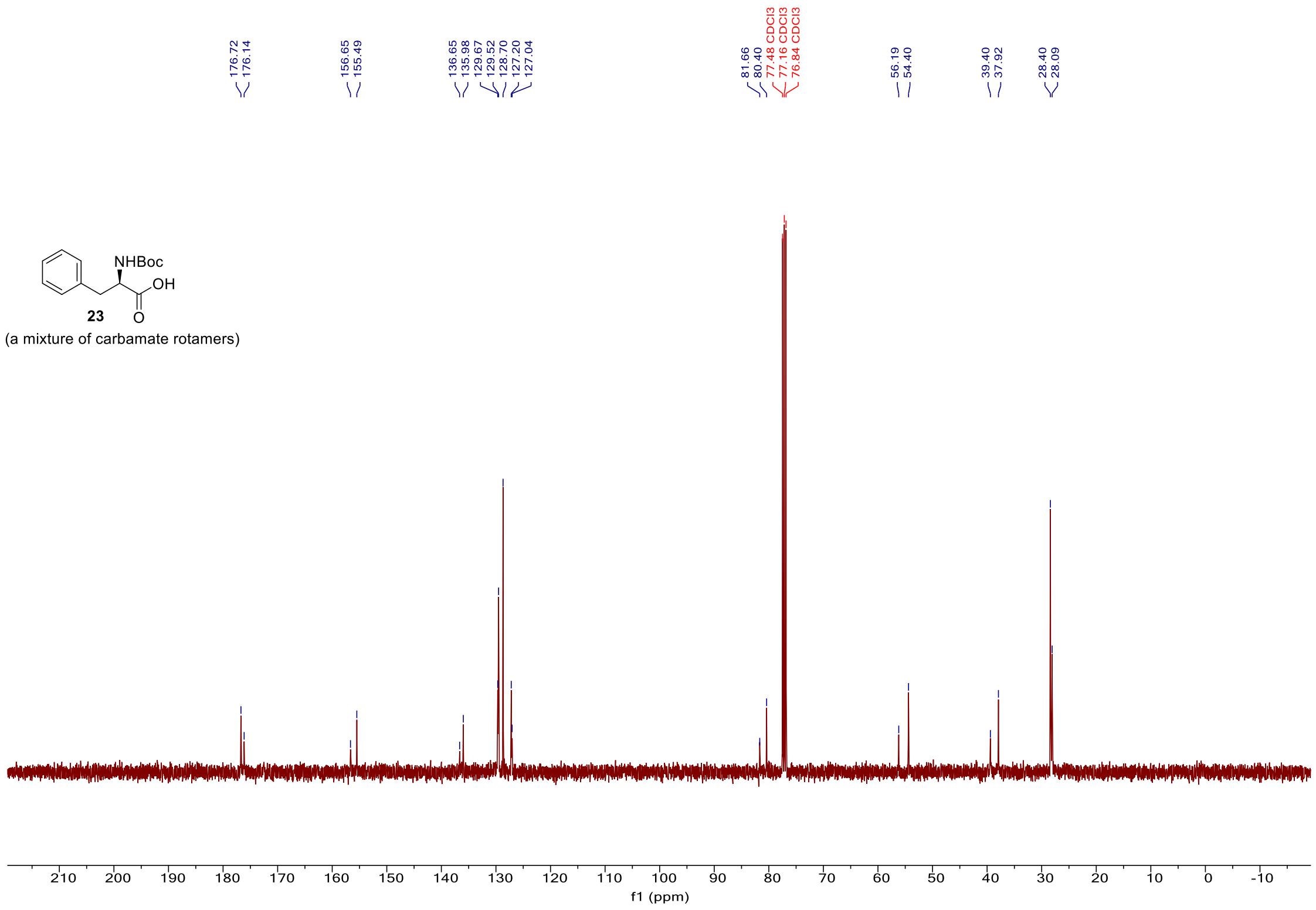

^{13}C NMR (101 MHz, $CDCl_3$)

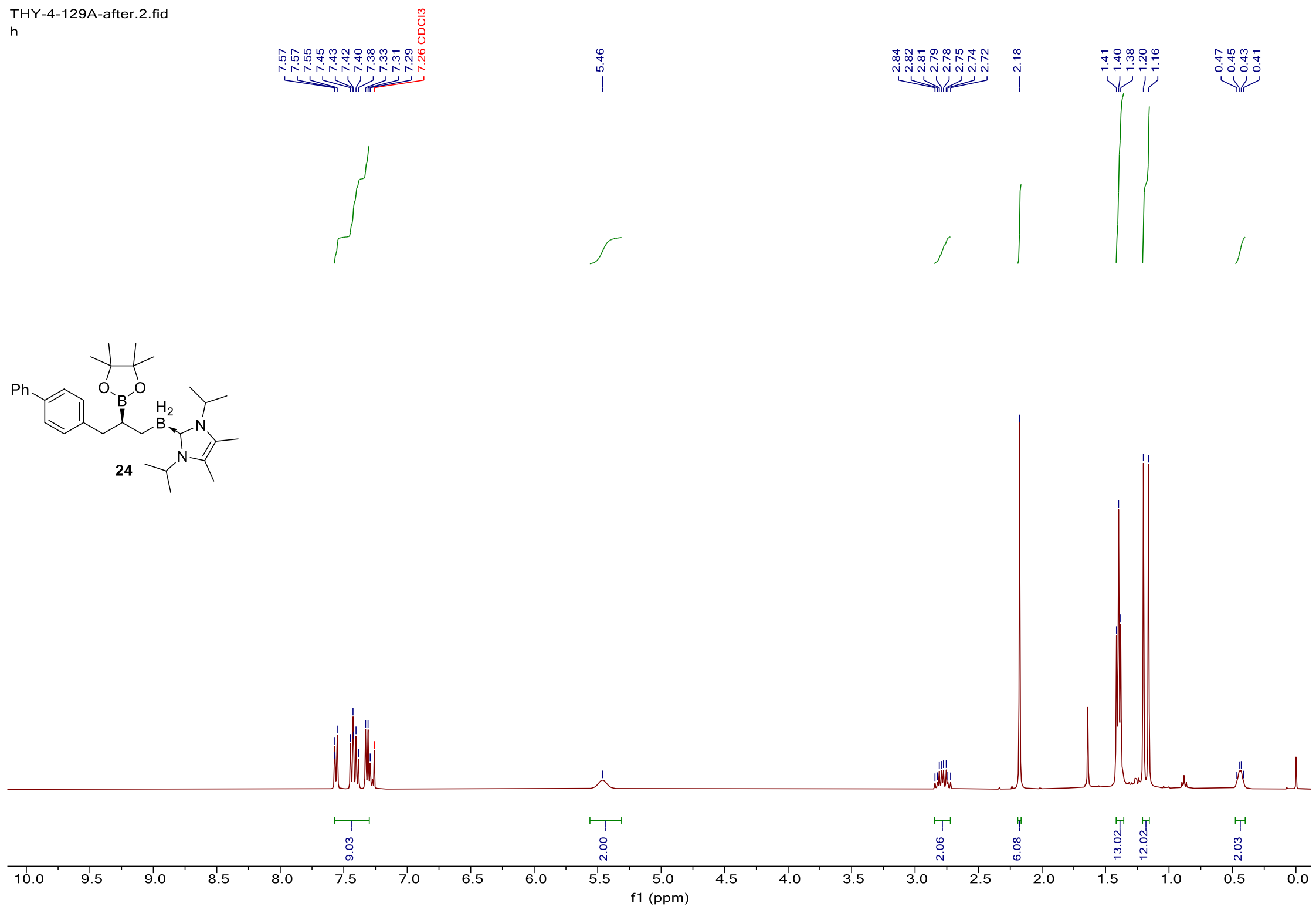

^{1}H NMR (400 MHz, $CDCl_3$)

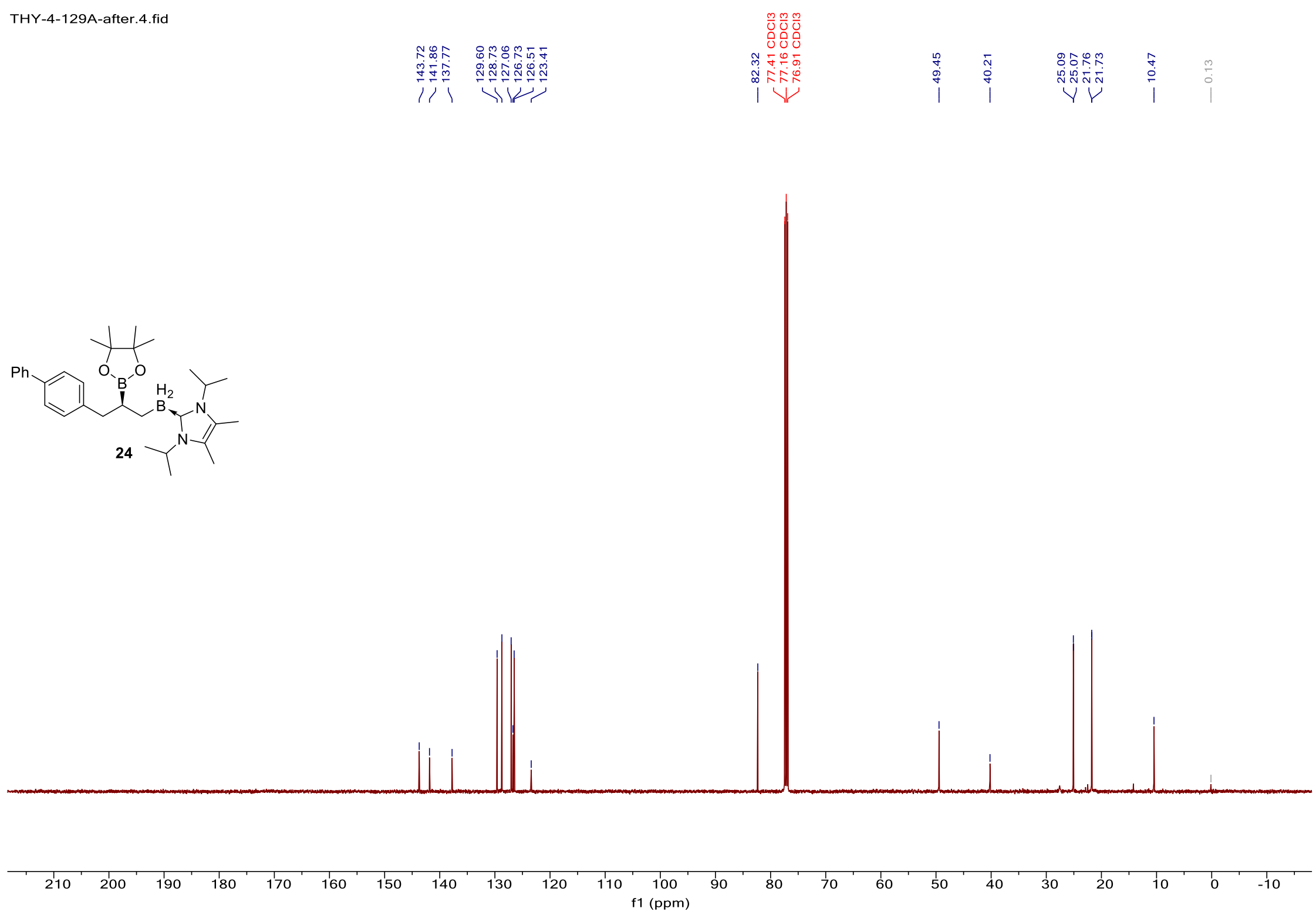

^{13}C NMR (101 MHz, $CDCl_3$)

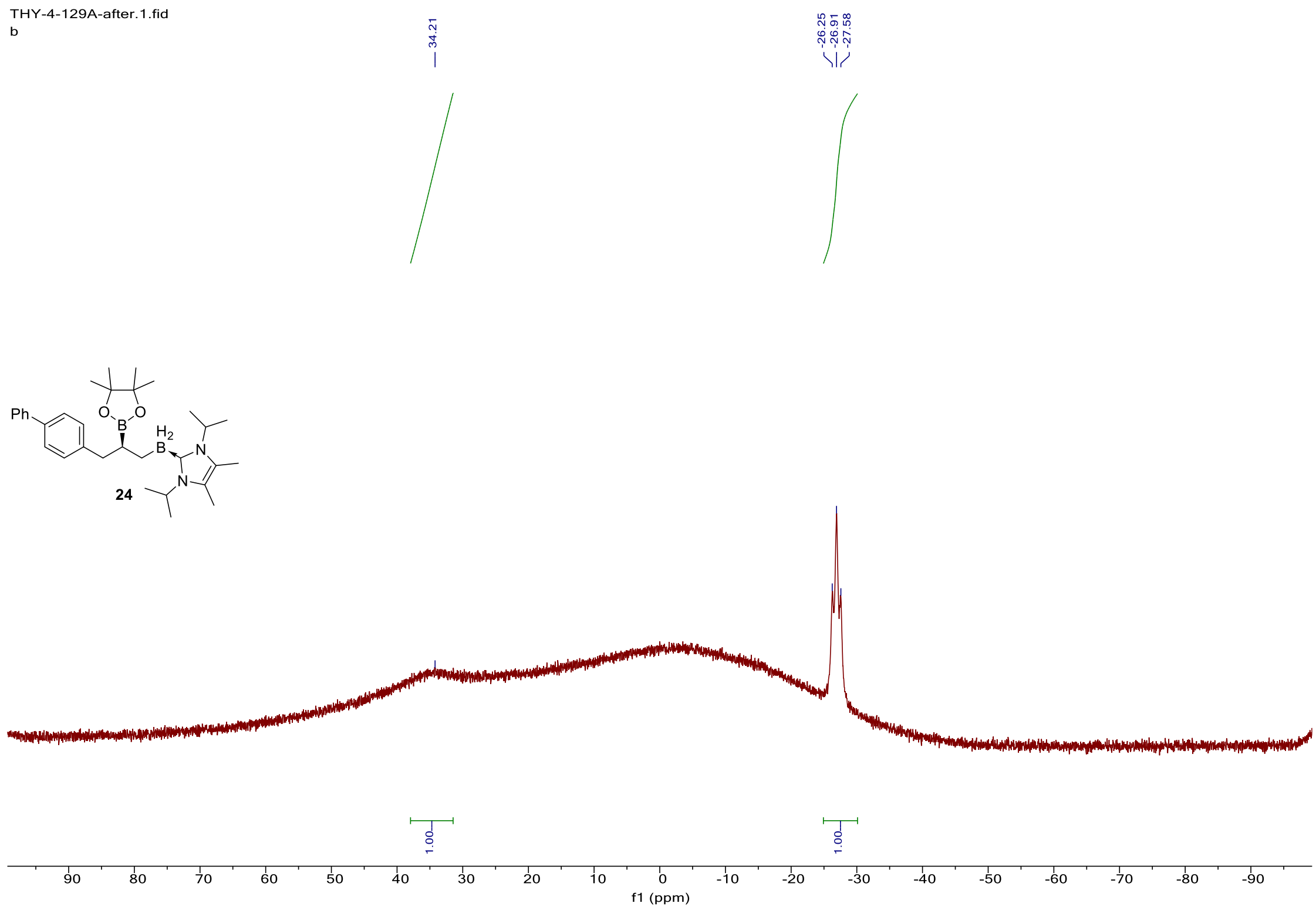

^{11}B NMR (128 MHz, $CDCl_3$)

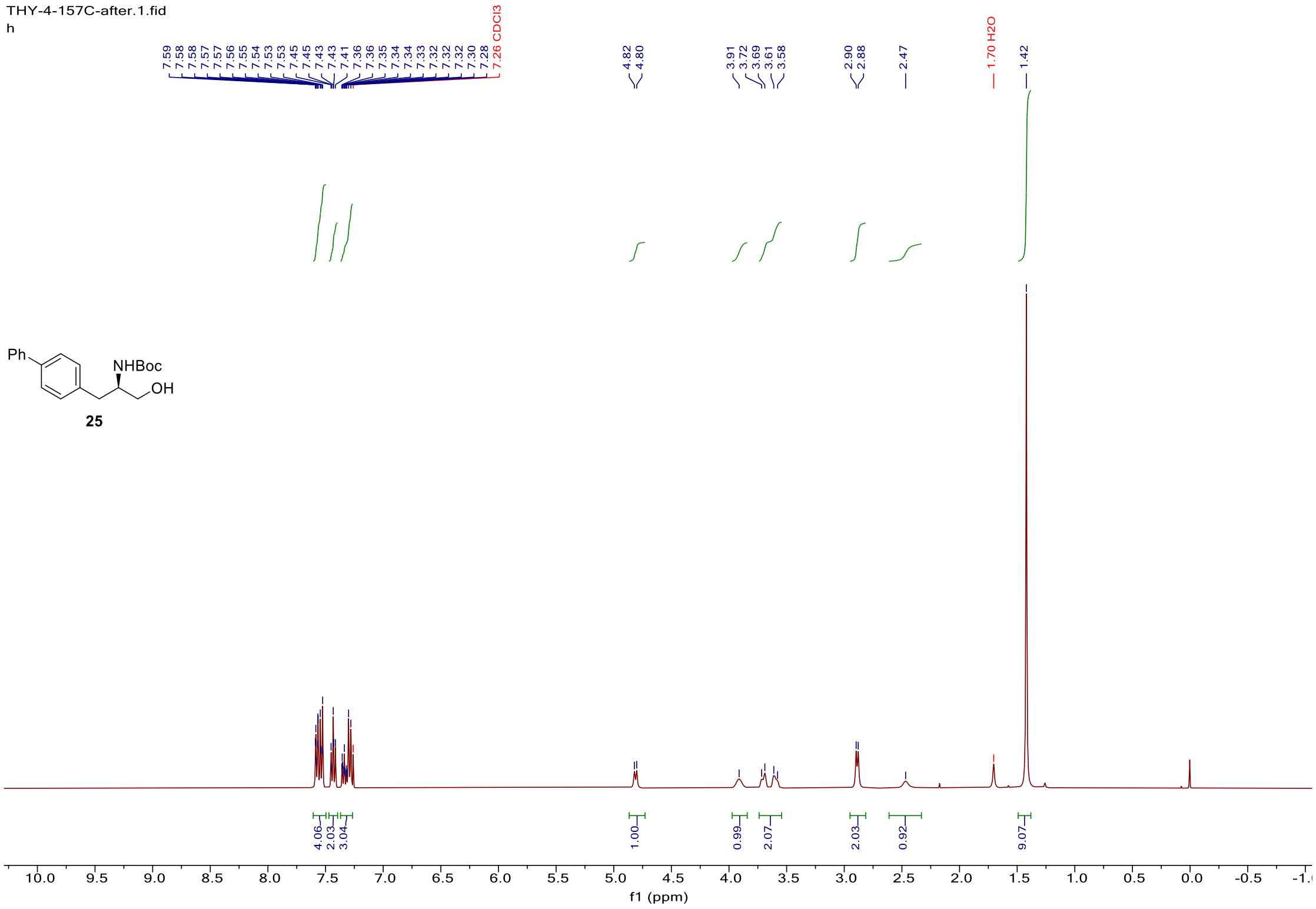

^{1}H NMR (400 MHz, $CDCl_3$)

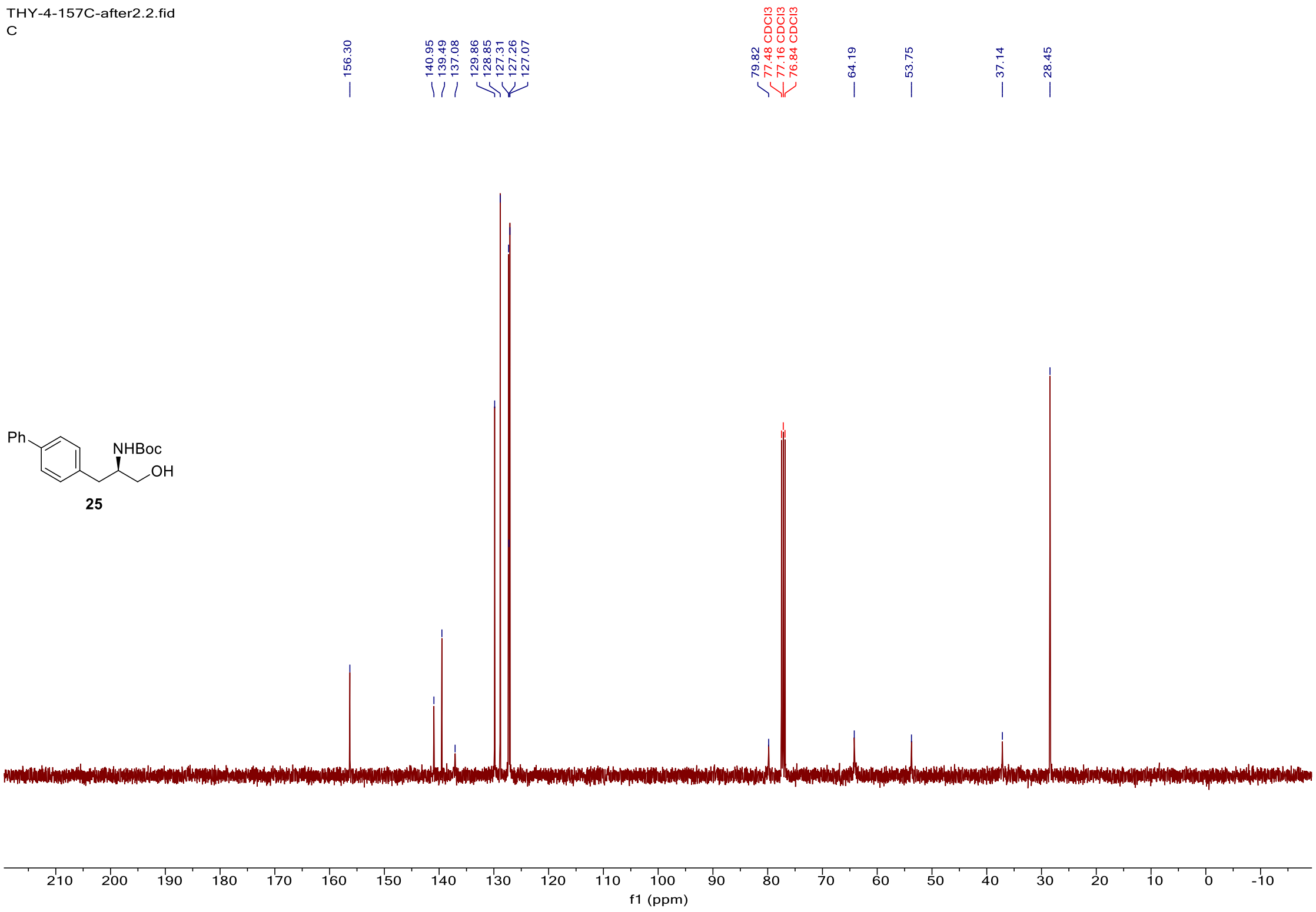

^{13}C NMR (101 MHz, $CDCl_3$)

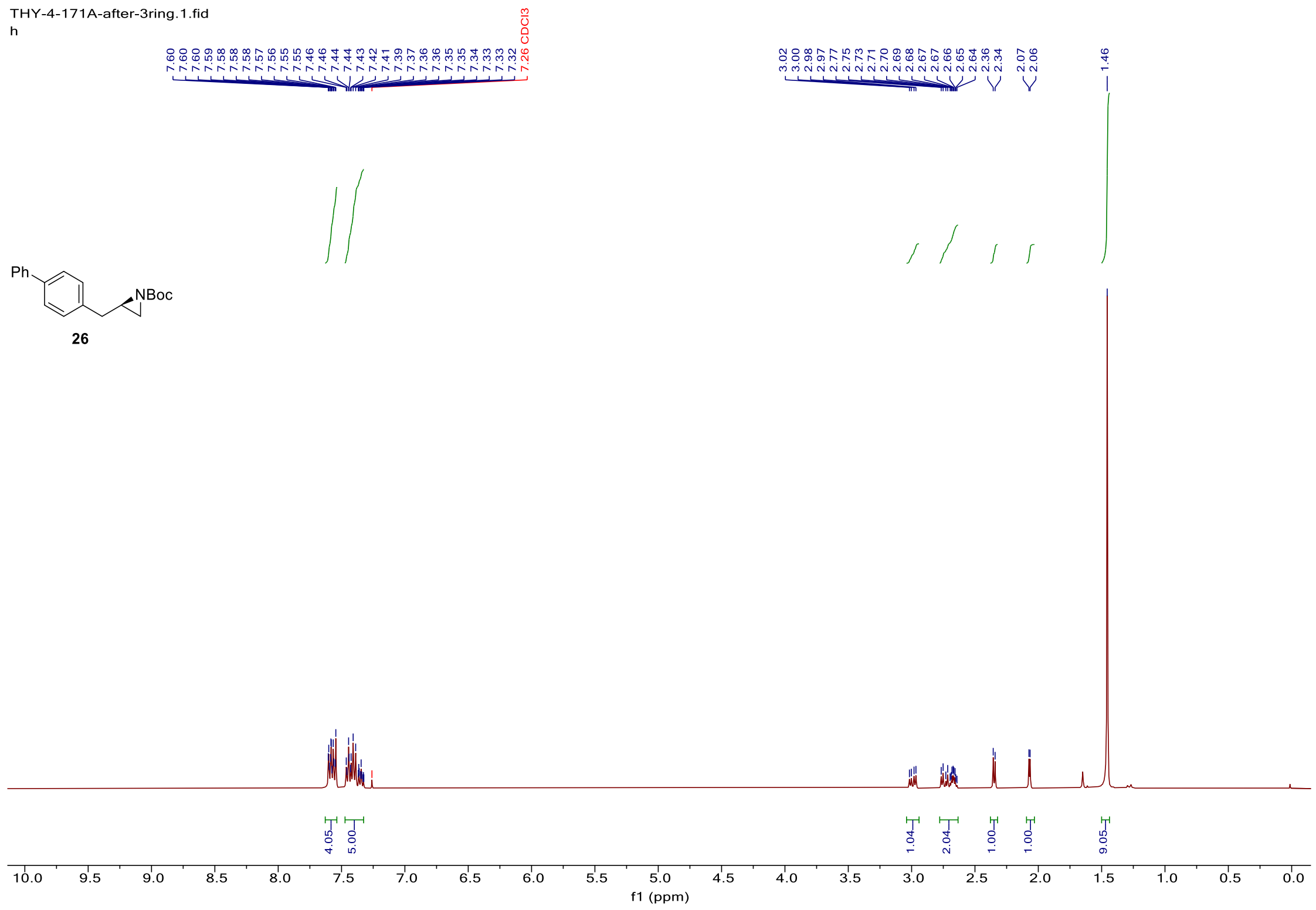

^{1}H NMR (400 MHz, $CDCl_3$)

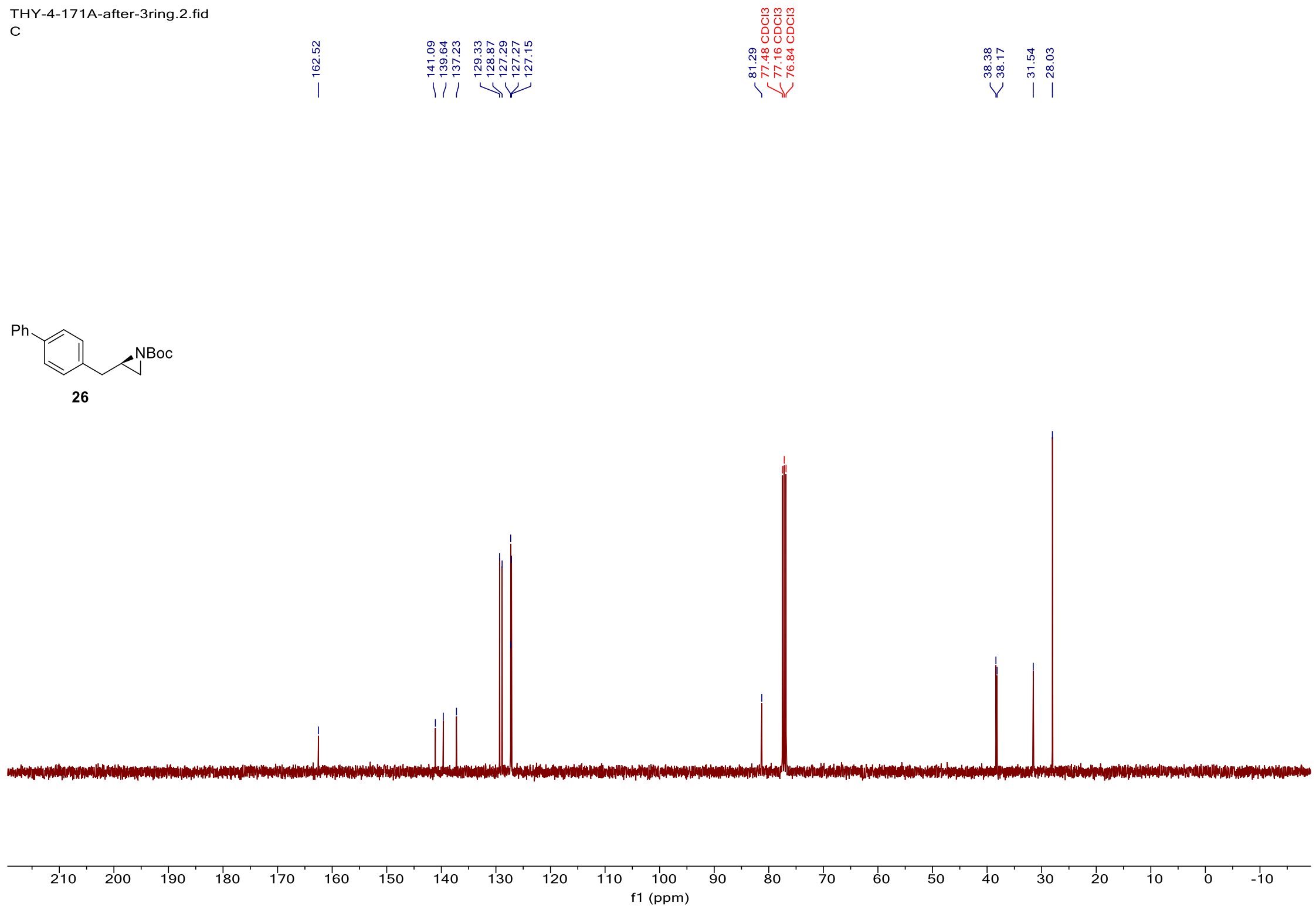

^{13}C NMR (101 MHz, $CDCl_3$)

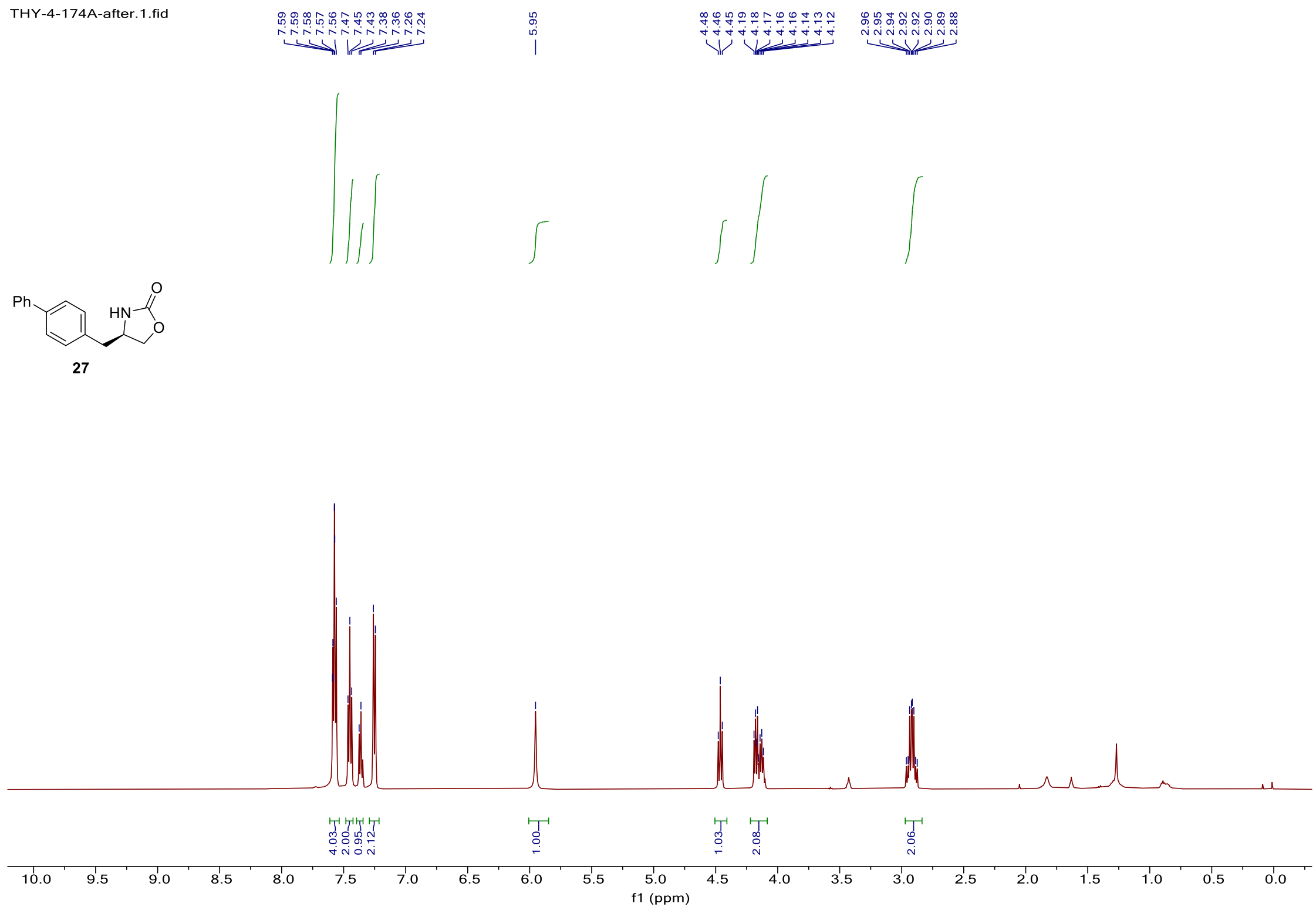

^{1}H NMR (400 MHz, $CDCl_3$)

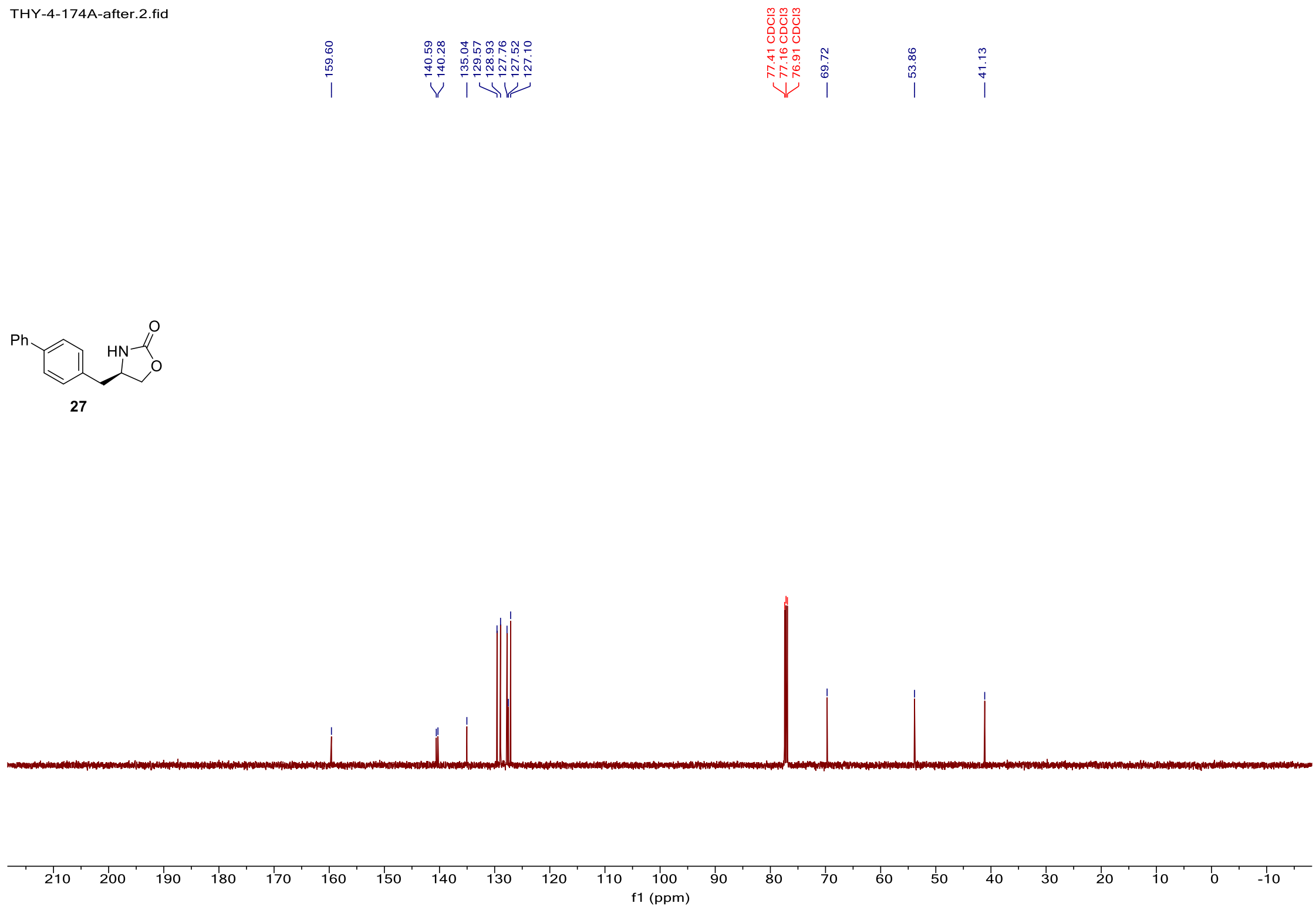

^{13}C NMR (101 MHz, $CDCl_3$)

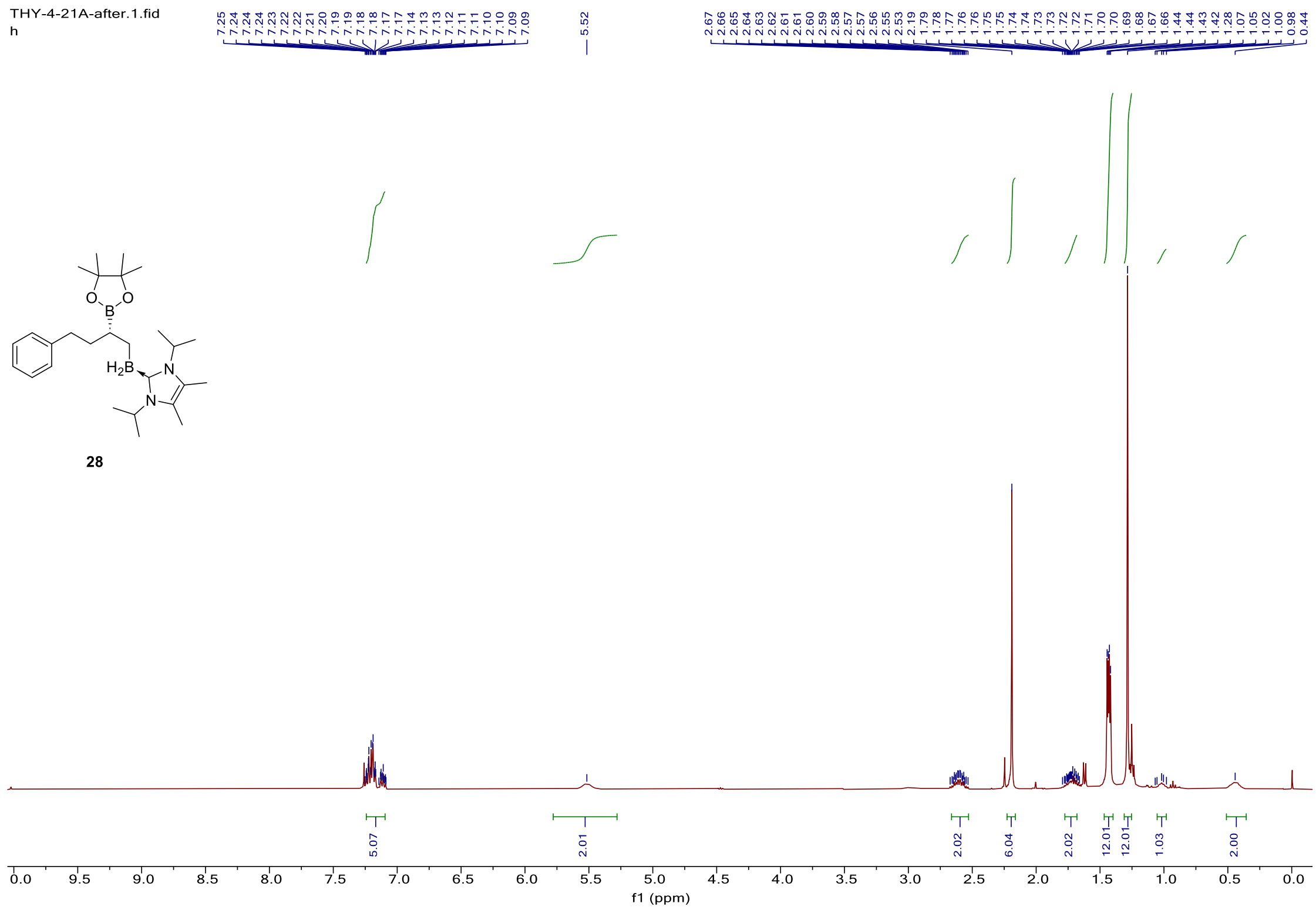

^{1}H NMR (400 MHz, $CDCl_3$)

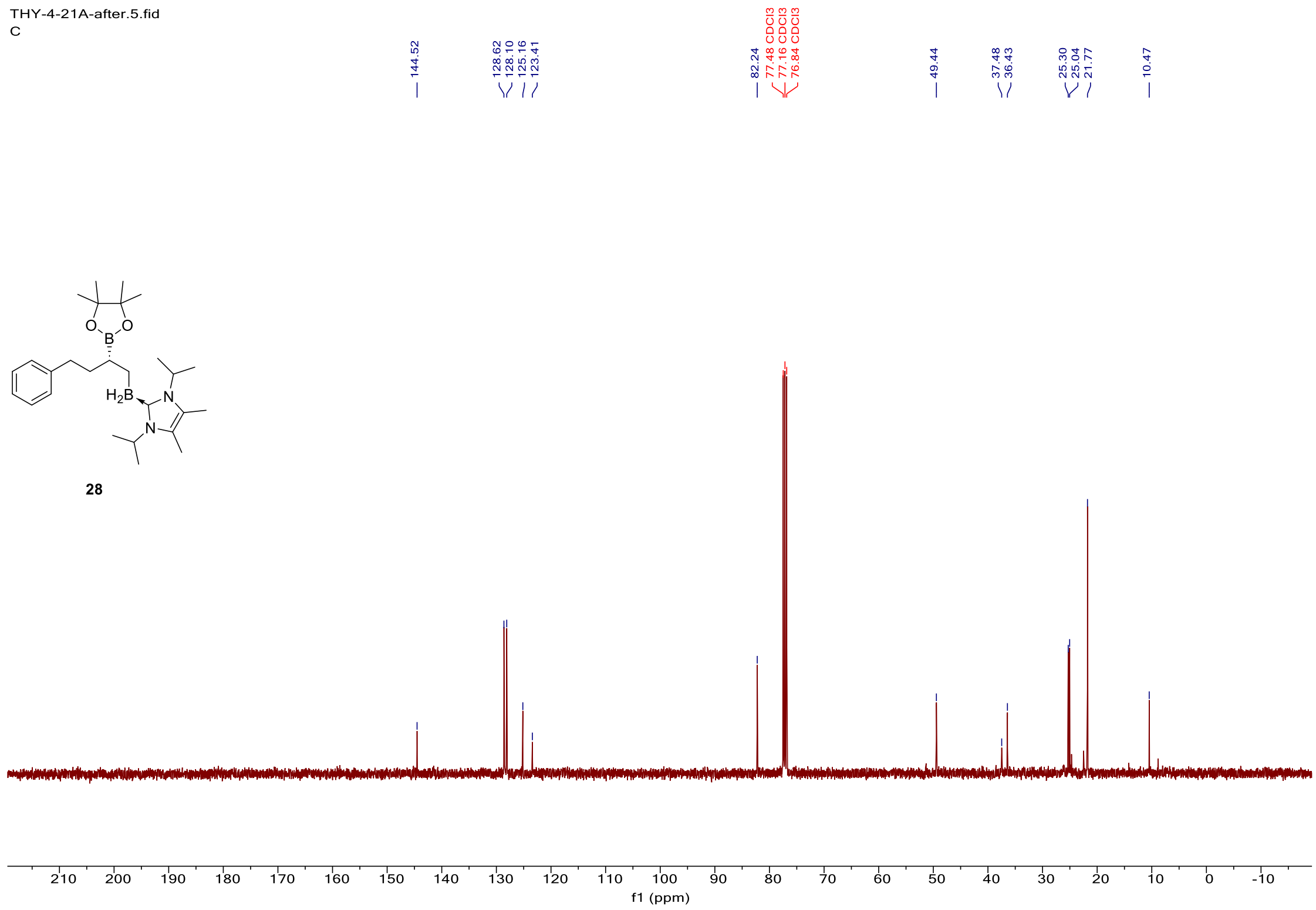

^{13}C NMR (101 MHz, $CDCl_3$)

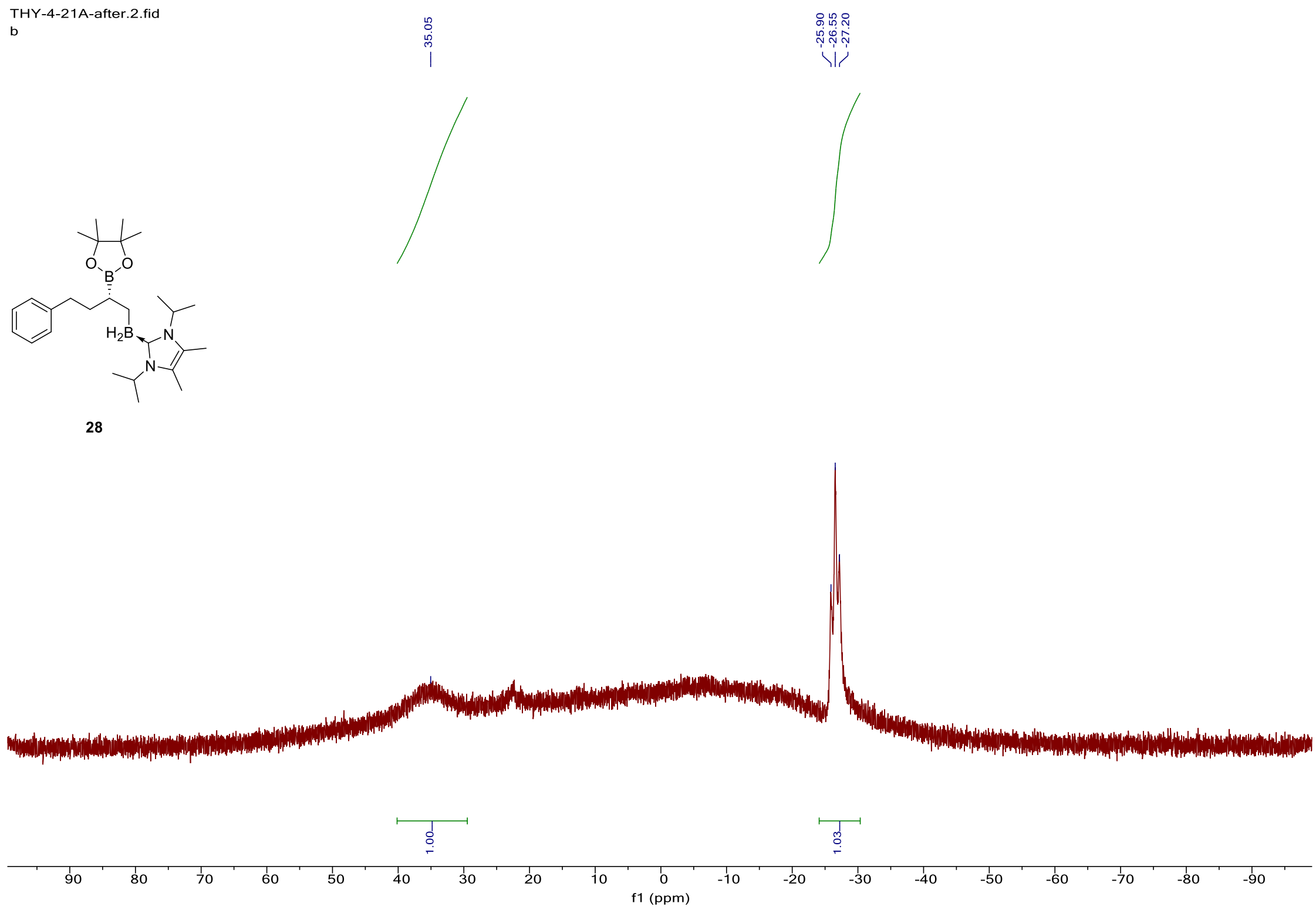

^{11}B NMR (128 MHz, $CDCl_3$)

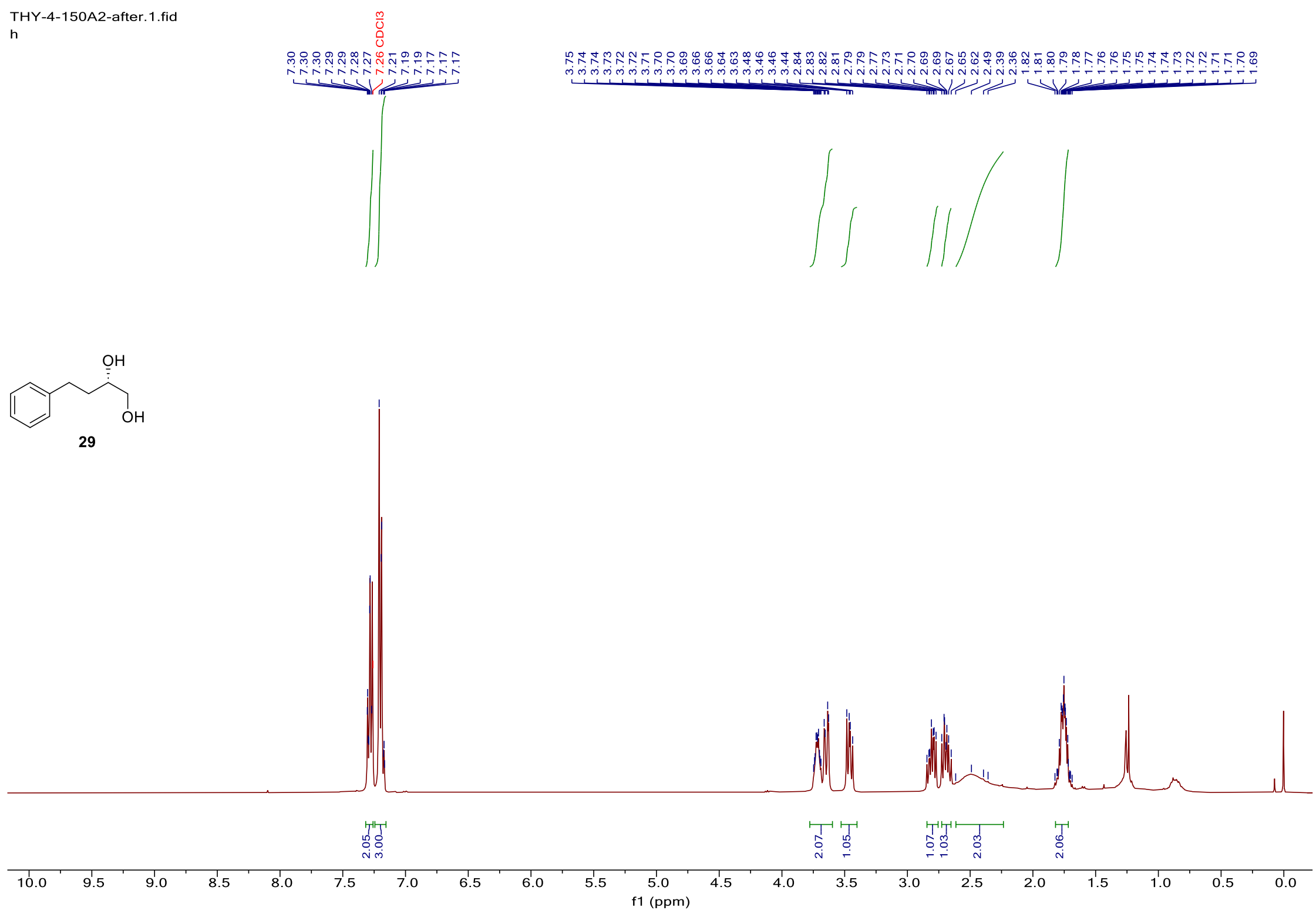

^{1}H NMR (400 MHz, $CDCl_3$)

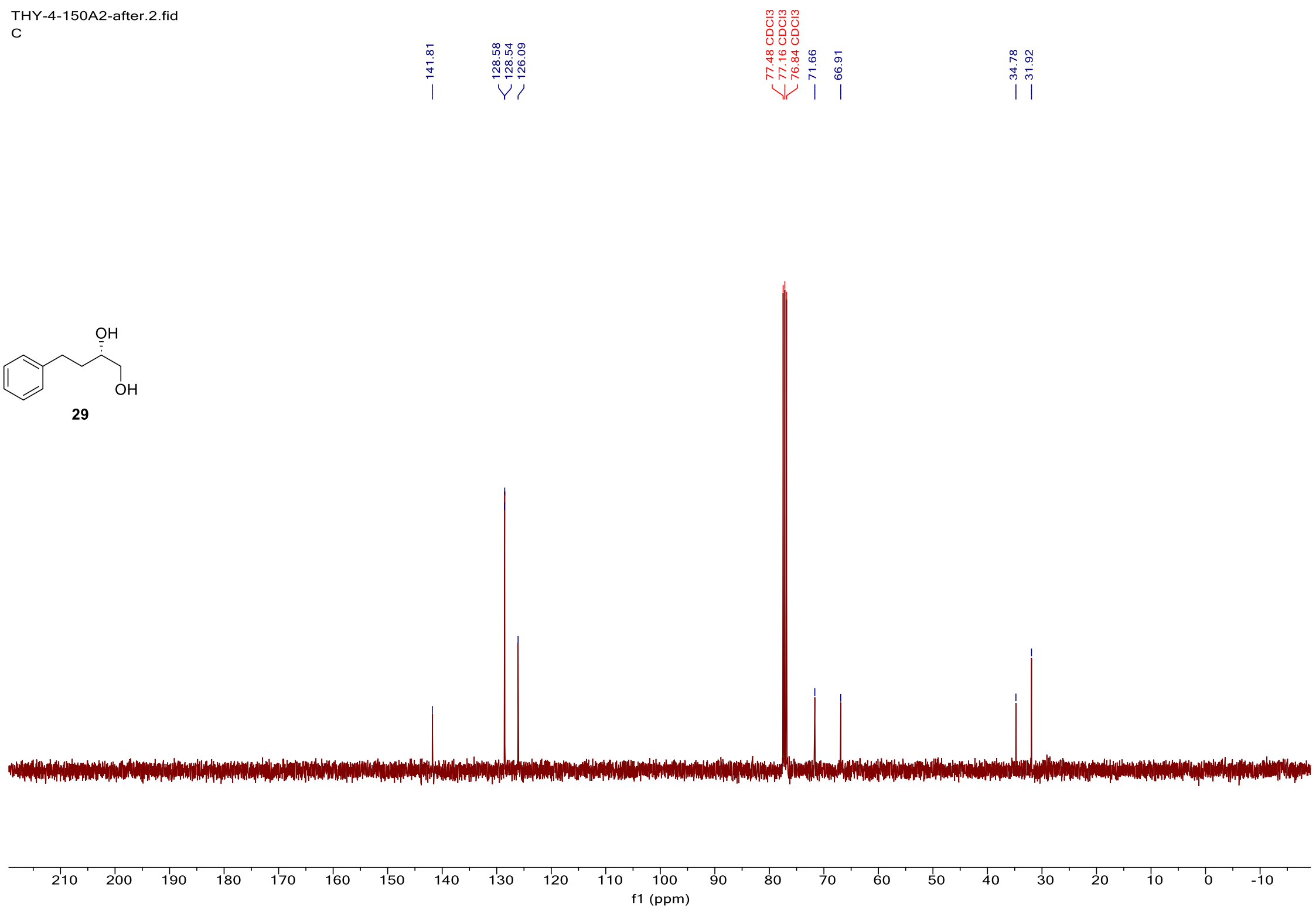

29

^{13}C NMR (101 MHz, $CDCl_3$)

THY-4-144A-major.3.fid

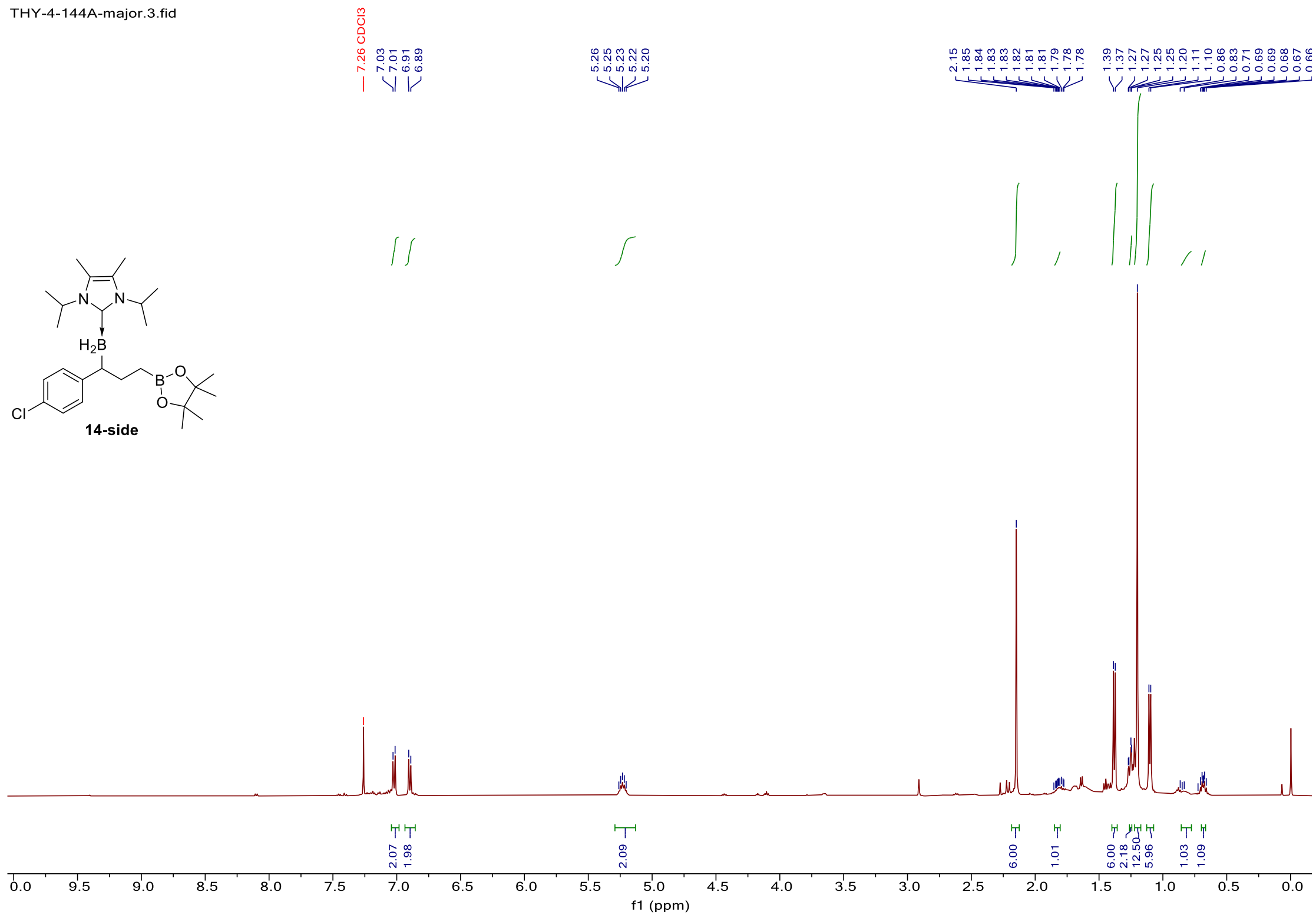

^{1}H NMR (500 MHz, $CDCl_3$)

THY-4-144A-major.6.fid

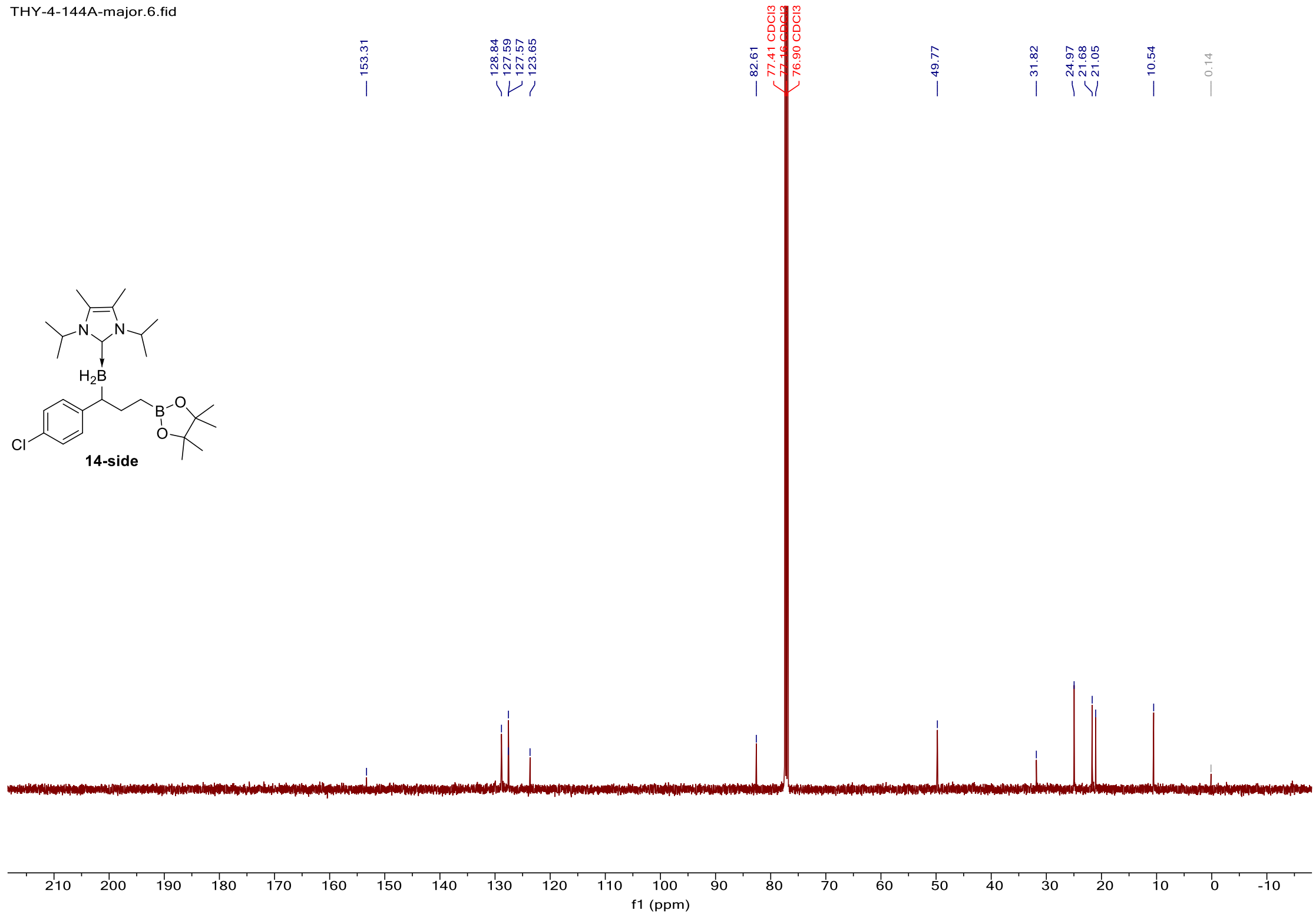

^{13}C NMR (126 MHz, $CDCl_3$)

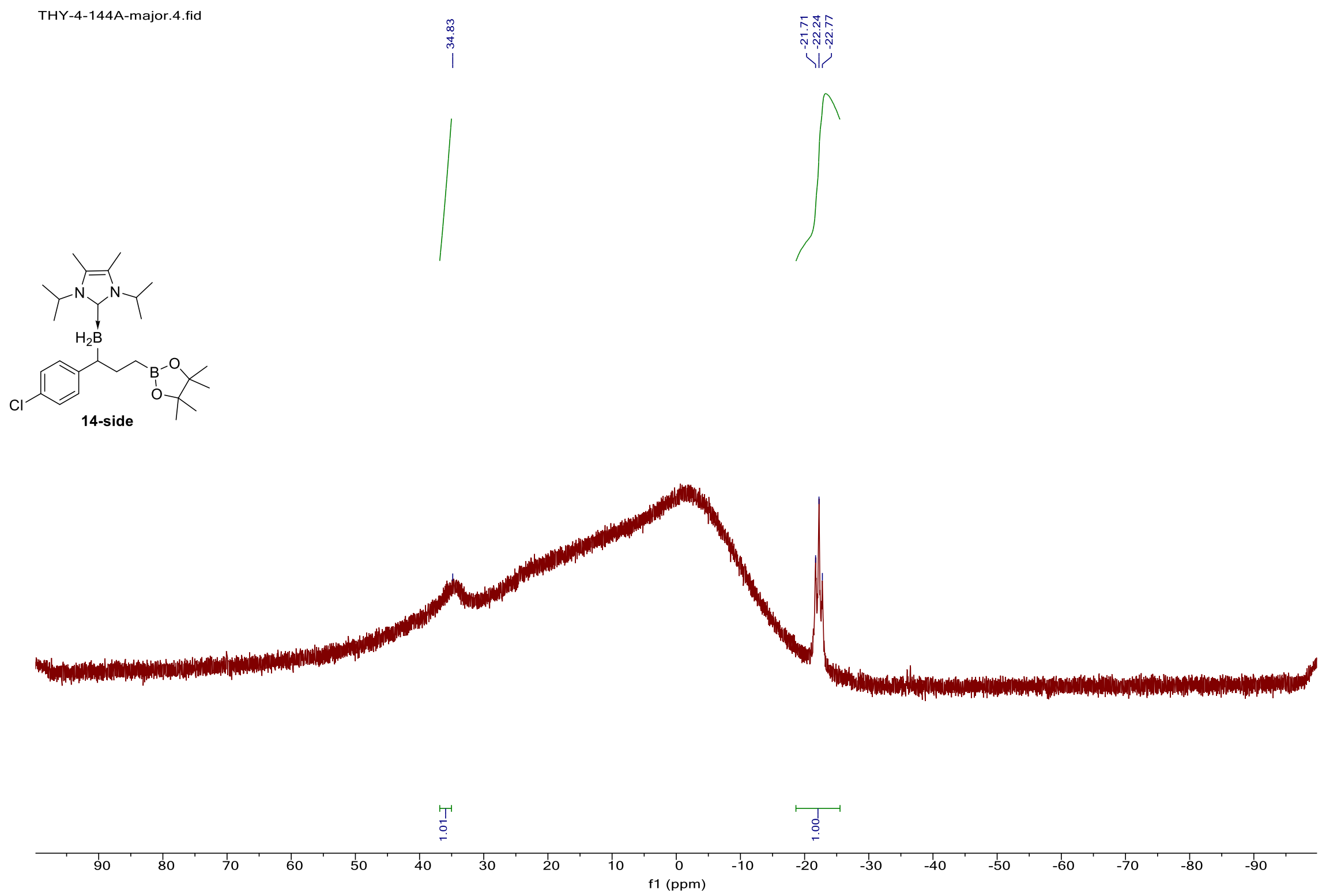

^{11}B NMR (160 MHz, $CDCl_3$)
